# Genomic Aberrations Generate Fusion Gene FOXK2::TP63 and Activate NFKB1 in Cutaneous T-Cell Lymphoma

**DOI:** 10.3390/biomedicines10082038

**Published:** 2022-08-21

**Authors:** Stefan Nagel, Claudia Pommerenke, Hilmar Quentmeier, Corinna Meyer, Maren Kaufmann, Roderick A. F. MacLeod

**Affiliations:** Department of Human and Animal Cell Lines, Leibniz-Institute DSMZ, 38124 Braunschweig, Germany

**Keywords:** T-ALL, TPL1XR1::TP63, MOTN-1

## Abstract

Cutaneous T-cell lymphoma (CTCL) is a severe lymphoid malignancy with a worse prognosis lacking curative treatment regimens. Several gene mutations and deregulated pathways, including NFkB signaling, have been implicated in its pathogenesis. Accordingly, CTCL cell line HUT-78 reportedly contains mutated NFKB2, which is constitutively activated via partial gene deletion, also demonstrating that genomic rearrangements cause driving mutations in this malignancy. Here, along with HUT-78, we analyzed CTCL cell line HH to identify additional aberrations underlying gene deregulation. Karyotyping and genomic profiling of HH showed several rearrangements worthy of detailed investigation. Corresponding to the established karyotype, RNA-seq data and PCR analysis confirmed the presence of t(3;17)(q28;q25), generating a novel fusion gene, FOXK2::TP63. Furthermore, chromosomal rearrangement t(1;4)(p32;q25) was connected to amplification at 4q24–26, affecting aberrant NFKB1 overexpression thereat. Transcription factor binding-site analysis and knockdown experiments demonstrated that IRF4 contributed to NFKB1 expression. Within the same amplicon, we identified amplification and overexpression of NFkB signaling activator CAMK2D (4q26) and p53-inhibitor UBE2D3 (4q24). Genomic profiling data for HUT-78 detailed a deletion at 10q25 underlying reported NFKB2 activation. Moreover, amplifications of ID1 (20q11) and IKZF2 (2q34) in this cell line drove overexpression of these NK cell differentiation factors and possibly thus formed corresponding lineage characteristics. Target gene analysis for NFKB1 via siRNA-mediated knockdown in HH revealed activation of TP63, MIR155, and NOTCH pathway component RBPJ. Finally, treatment of HH with NFkB inhibitor demonstrated a role for NFkB in supporting proliferation, while usage of inhibitor DAPT showed significant survival effects via the NOTCH pathway. Collectively, our data suggest that NFkB and/or NOTCH inhibitors may represent reasonable treatment options for subsets of CTCL patients.

## 1. Introduction

Cutaneous T-cell lymphoma (CTCL) covers a heterogeneous group of lymphomas presenting in the skin. This malignancy becomes manifest in subtypes with distinct histopathologies and clinical features and includes mycosis fungoides, Sézary syndrome, lymphomatoid papulosis, and primary cutaneous anaplastic large cell lymphoma [1]. CTCL is a fatal disease with a worse prognosis that lacks curative treatment options, demanding the identification of novel diagnostic markers and therapeutic targets. Cytogenetic and sequencing studies have revealed several pathogenic alterations in CTCL patients, including chromosomal rearrangements, deregulation of the cell cycle, and aberrant activities of signaling pathways [1,2].

Analyses of chromosomal and genomic aberrations in CTCL patients and cell lines have shown the presence of translocations, fusion genes, and copy number alterations [3,4,5,6,7,8]. Generally, fusion genes play important diagnostic and pathological roles in hematopoietic malignancies [9]. In CTCL, various fusion genes have been described, including PCM1::JAK2, TBL1XR1::TP63, and ATXN1::TP63 [2,10,11,12]. Rearrangements of TP63, including TP63 fusions, are frequently described in several types of T-cell lymphoma, where they operate oncogenically via the generation of a truncated factor [13,14,15].

Deregulated signaling pathways in CTCL patients include the JAK-STAT pathway, MAP kinase signaling, p53 pathway, and NFkB signaling [1]. The NFkB-signaling pathway is normally activated by various extracellular stimuli, resulting in the translocation of cytoplasmic NFkB transcription factors (TFs) into the nucleus. Humans possess five NFkB factors, comprising REL, RELA, RELB, NFKB1, and NFKB2. These factors operate as dimers and share the Rel homology domain, enabling DNA binding [16]. NFKB1 (p50) and NFKB2 (p52) are generated as larger precursor proteins (p105 and p100, respectively) and activated upon stimulation by proteolytic cleavage. In CTCL, NFKB2 is oncogenically altered by generating a C-terminally deleted and thus constitutively activated factor. CTCL cell line HUT-78 has served as a model to identify and analyze this mutated oncogene that has also been detected in CTCL patients [17,18].

Chromosomal aberrations and copy number alterations underlying activation of oncogenes or inhibition of tumor suppressor genes have been frequently described in CTCL [3,4,5,6,7]. Here, we used CTCL cell line HH as a model to reveal novel genomic aberrations and their targeted genes, contributing to the pathogenesis of this malignancy.

## 2. Materials and Methods

### 2.1. Cell Lines and Treatments

Cell lines HH, JURKAT, NK-92, KHYG-1, and DERL-2 were obtained from the DSMZ (German Collection of Microorganisms and Cell Lines, Braunschweig, Germany), HUT-78 from the ATCC (American Type Culture Collection, Manassas, VA, USA), and the cell lines MAC-1 and MAC-2A kindly provided by Marshall E. Kadin (Boston University School of Medicine, Providence, RI, USA). Cell culture conditions and backgrounds of each cell line are described elsewhere [19]. Cell lines were authenticated and tested free of mycoplasma and viral contamination.

Gene-specific siRNA oligonucleotides and AllStars negative control siRNA (siCTR) were purchased from Qiagen (Hilden, Germany). SiRNAs (80 pmol) were transfected into 1 × 10^6^ cells by electroporation using the EPI-2500 impulse generator (Fischer, Heidelberg, Germany) at 350 V for 10 ms. Transfected cells were harvested after 20 h cultivation. Transfection experiments were performed twice, generating similar results.

Additional cell treatments were performed with 14 µM NFKB inhibitor (Sigma, Taufkirchen, Germany) or with 10 µM DAPT (N-[N-(3,5-Difluorphenacetyl)-L-alanyl]-S-phenylglycin-tert-butylester; Sigma), both dissolved in dimethyl sulfoxide (DMSO). Treated cells were functionally analyzed with the IncuCyte S3 Live-Cell Analysis System (Essen Bioscience, Hertfordshire, UK). This system allows continuous documentation of living cells within the incubator. Subsequent analysis of the microscopical images using the associated software allows adapted examination of the data, graphical presentation, and statistical calculations. For detection of apoptotic cells, we additionally used the IncuCyte Caspase-3/7 Green Apoptosis Assay diluted at 1:2000 (Essen Bioscience, Ann Arbor, MI, USA). This assay stains apoptotic cells detectable by integrated fluorescence microscopy. Thus, proliferating and apoptotic cells are detectable at once. Live-cell imaging experiments were performed twice with four-fold parallel tests.

### 2.2. Chromosomal and Genomic Analyses

Karyotyping, spectral karyotyping (SKY), and fluorescence in situ hybridization (FISH) were performed as described previously [20]. BAC clone RP11-348F2 was obtained from BacPac Resources, Children’s Hospital Oakland Research Institute (Oakland, CA, USA) to analyze NFKB1 at 4q24. Insert DNA was harvested using the Big BAC DNA Kit (Princeton Separations, Adelphia, NJ, USA) and directly labeled by nick translation with dUTP-fluors (Dyomics, Jena, Germany). Fluorescent images were captured and analyzed with an Axio-Imager microscope (Zeiss, Göttingen, Germany) configured to a dual Spectral Imaging FISH system (Applied Spectral Imaging, Vätö, Sweden).

For genomic profiling, the genomic DNA of CTCL cell lines was prepared by the Qiagen Gentra Puregene Kit (Qiagen). Labeling, hybridizatin, and scanning of HD Cytoscan arrays were performed at the Genome Analytics Facility of the Helmholtz Centre for Infection Research (Braunschweig, Germany), using HD arrays according to the manufacturer´s protocols (Affymetrix, High Wycombe, UK). Data were visualized and interpreted using the Chromosome Analysis Suite software version 2.0.1.2 (Affymetrix).

### 2.3. Transcriptome Analysis Based on RNA-Seq and Expression Profiling Data

Transcriptome data from 100 leukemia/lymphoma cell lines (LL-100) were obtained from the European Nucleotide Archive (ENA; www.ebi.ac.uk/ena; accessed on 1 March 2022) using data set E-MTAB-7721 [21]. Bioinformatic identification of fusion genes was performed using FusionCatcher on hg38 with ≥ 1 min-split-read + ≥ 3 min-span-pairs (version 0.99.6a beta) [22]. Graphical presentation of LL-100 data was performed using shinyNGS (https://github.com/pinin4fjords/shinyngs; accessed on 1 March 2022) for bar plots and Heatmapper for heatmaps [23]. RNA-seq data for primary cells were obtained from The Human Protein Atlas (www.proteinatlas.org; accessed on 1 March 2022) [24].

Expression profiling data sets from cell lines were generated by Dr. Robert Geffers (Genome Analytics Facility) using HG U133 Plus 2.0 gene chips (Affymetrix, High Wycombe, UK) and obtained from GEO (https://www.ncbi.nlm.nih.gov/gds/; accessed on 1 March 2022) as reported previously [25,26]. After RMA-background correction and quantile normalization of the spot intensities, profiling data were expressed as ratios of the sample mean and subsequently log2 transformed. Data processing, including hierarchical clustering (Ward’s method) and its visualization as a dendrogram, was performed via R/Bioconductor using limma and affy packages.

### 2.4. Polymerase Chain Reaction (PCR) Analyses

Total RNA was extracted from cell line samples using TRIzol reagent (Invitrogen, Darmstadt, Germany). cDNA was synthesized by random priming from 5 µg RNA using Superscript II (Invitrogen).

To analyze fusion genes in cell lines, we used the following oligonucleotides: FOXK2-for 5′-GGTCTTCAGGGTACAAGGTGG-3′, FOXK2-rev 5′-GCCCTTGTCCGCAGTCCTGTAG-3′, TP63-for1 5′-TGAACCATCAGAAGATGGTGCG-3′, TP63-rev1 5′-GCATCGAAGGTGGAGCTGGGC-3′, TBL1XR1-for 5′-GAAAGCCATATCAGTCAGTCC-3′, TBL1XR1-rev 5′-TCTATCAGGGACAGAGACTCT-3′, TP63-for2 5′-GTTCAGCCCATTGACTTGAAC-3′, TP63-rev2 5′-CTGCTGGTCCATGCTGTTC-3′. To analyze a described mutation in NFKB1, we amplified the according gene fragment using cDNA as a template and oligonucleotides NFKB1-for 5′-CTTCAGAATGGCAGAAGATGATCC-3′ and NFKB1-rev 5′-CAGGTAGTCCACCATGGGATG-3′. PCR products were generated using taqpol (Qiagen) and thermocycler TGradient (Biometra, Göttingen, Germany), analyzed by gel electrophoresis and documented with the Azure c200 Gel Imaging System (Azure Biosystems, Dublin, CA, USA). Oligonucleotides were obtained from Eurofins MWG (Ebersberg, Germany). PCR products were cloned into pCR4-TOPO (Invitrogene) and sequenced at Eurofins MWG. The sequences were analyzed using nucleotide BLAST (https://blast.ncbi.nlm.nih.gov/Blast.cgi; accessed on 2 March 2022).

Real-time quantitative (RQ) PCR analysis was performed with the 7500 Real-time System, using commercial buffer and primer sets (Thermo Fisher Scientific, Darmstadt, Germany). For normalization of expression levels, we analyzed the transcript of TATA box binding protein (TBP). We used the ddCT method, and the obtained values are indicated as fold expressions in relation to one selected sample that was set to 1. For quantification of genomic copy numbers for ID1, we used the following oligonucleotides: ID1-1 5′-CTACGACATGAACGGCTGTTACTC-3′ and ID1-2 5′-TTCGGATTCCGAGTTCAGCTCC-3′. The MEF2C locus was used as a control: MEF2C-1 5′-AGAAGGCTTATGAGCTGAGC-3′ and MEF2C-2 5′-AGACTGGCATCTCGAAGTTG-3′. Genomic DNA was prepared as described above.

Quantitative PCR analyses were performed in triplicate. Standard deviations are calculated for each experiment and presented in the figures as error bars. Statistical significance was assessed by *t*-test and derived *p*-values indicated by asterisks (* *p* < 0.05, ** *p* < 0.01, *** *p* < 0.001, n.s. not significant).

### 2.5. Protein Analyses

Western blots were generated by the semi-dry method, and protein lysates from cell lines were prepared using SIGMAFast protease inhibitor cocktail (Sigma). Proteins were transferred onto nitrocellulose membranes (Bio-Rad, München, Germany) and blocked with 5% dry milk powder dissolved in phosphate-buffered saline buffer (PBS). The following antibodies were used: alpha-Tubulin (Sigma), NFKB1 (R&D Systems, Wiesbaden, Germany), and NFKB2 (Abcam, Cambridge, UK). The used antibodies were diluted as follows: alpha-Tubulin (TUBA) 1:1000, NFKB1 1:1000, NFKB2 1:500. For loading controls, blots were reversibly stained with Poinceau (Sigma), and detection of TUBA was performed thereafter. Secondary antibodies were linked to peroxidase for detection by Western-Lightning-ECL (Perkin Elmer, Waltham, MA, USA). Western blot analyses were performed twice. Documentation was performed using the digital system ChemoStar Imager (INTAS, Göttingen, Germany).

## 3. Results

### 3.1. Karyotyping, Genomic Profiling, and RNA-Seq Analysis of CTCL Cell Line HH

**Figure 1:**
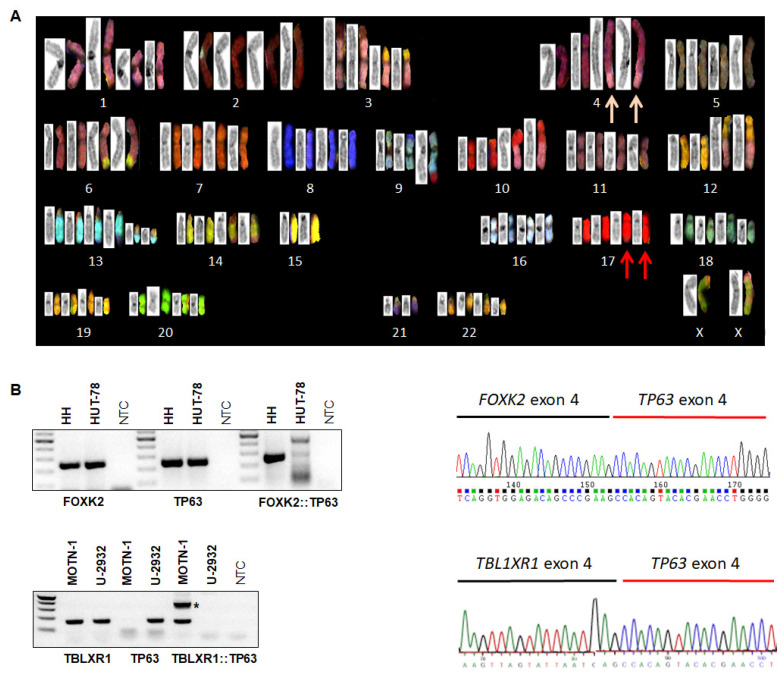
Karyotyping and fusion gene analysis. (**A**) Reverse DAPI G-banding (left images) and SKY (right) showing multiple rearrangements in HH, notably der(4)—pink arrows and semi-cryptic der(17)t(3;17)(q28;q25)—red arrows. (**B**) RT-PCR (left) and sequence analyses (right) of HH (above) and MOTN-1 (below) showed the presence of fusion genes FOXK2::TP63 and TBL1R1::TP63, respectively. In MOTN-1, one PCR product corresponded to an out-of-frame fusion and is labeled by an asterisk. * *p* < 0.05.

Chromosomal and genomic aberrations may result in the deregulation of specific genes contributing to the pathogenesis of hematopoietic malignancies. To identify novel oncogenes that may potentially serve as therapeutic targets in CTCL, we used cell line HH as a model and searched for chromosomal aberrations by performing classical karyotyping, SKY, and FISH analyses (Figure 1A). The consensus karyotype of HH was: 86-104 < 4n > XX,-Y,-Y, der(1)del(1)(p11p31)del(1)(q32)x2, der(3)t(2;3)(p15;p25)x2, der(3)t(3;12)(q11;p11)x2, der(4)dup(4)(q24q27)amp(4)(q2?)t(1;4)(p32;q25)x2, t(6;14)(q25;q24)x2, der(9)t(9;10)(q34;q23)x2, der(10)t(1;10)(q22;q23)x2, del(12)p12p13), +13, +13, del(13)(q11q31.2)x2, der(13)t(13;21)(q33;q22)x2, −15, −15, der(17)t(3;17)(q28;q25), −21, −21. Thus, significant genetic heterogeneity was detected.

Fusion genes form a special class of oncogenes with proven pathological and diagnostic potential. To check if cytogenetic aberrations in cell line HH generated fusion genes, we analyzed our published LL-100 RNA-seq data set bioinformatically. This procedure revealed a potential fusion between FOXK2 at 17q25 and TP63 at 3q28, highlighted by the cytogenetic data showing explicit der(17) partners of the semi-cryptic t(3;17)(q28;q25). According to the RNA-seq data and verified by RT-PCR and Sanger sequencing analyses, we observed the expression of a FOXK2::TP63 fusion transcript in HH, joining FOXK2 exon 4 and TP63 exon 4 (Figure 1B). In addition, RNA-seq data showed raised TP63 RNA levels in HH attributable to this fusion transcript and driven by the ubiquitously expressed FOXK2 gene (Figure S1).

Interestingly, T-cell large granular lymphocyte leukemia (T-LGL) cell line MOTN-1 also showed elevated TP63 levels (Figure S1). Accordingly, bioinformatic analysis of MOTN-1 RNA-seq data indicated fusion of TBL1XR1 exon 4 and TP63 exon 4, again confirmed by RT-PCR and Sanger sequencing analyses (Figure 1B). Similarly, the 5′-fusion-partner, TBL1XR1, showed high expression levels in the complete LL-100 cell line panel (Figure S1). Thus, we identified two different TP63-fusion genes: FOXK2::TP63 generated by t(3;17)(q28;q25) in CTCL cell line HH, and TBL1XR1::TP63 in T-LGL cell line MOTN-1. The fusion gene FOXK2::TP63 represents a novel finding, while TPL1XR1::TP63 has been detected in both diffuse large B-cell lymphoma and peripheral T-cell lymphoma patients, generated by inv(3)(q26q28) [13,14,15,27].

**Figure 2:**
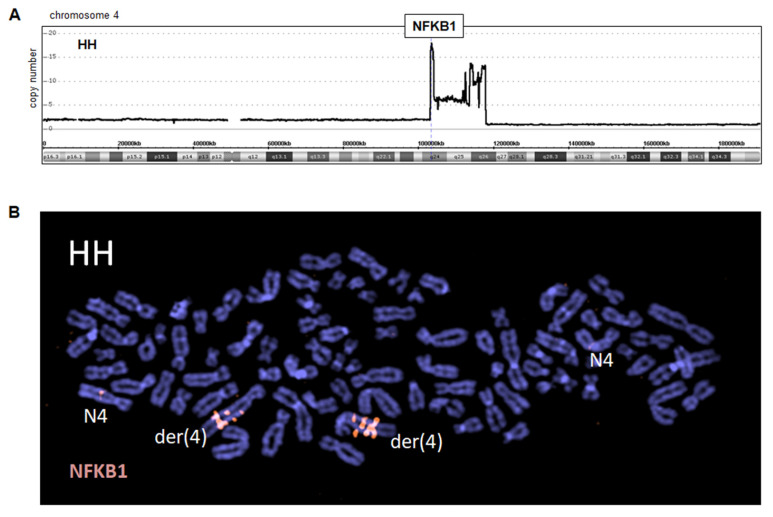
Amplification at 4q24–26 in cell line HH. (**A**) Genomic profiling data for chromosome 4 indicates an amplicon at 4q24–26 and telomeric deletion. (**B**) FISH analysis using a red-labeled probe (348F2), which covers the locus for NFKB1 at 4q24, demonstrating strong amplification/triplication on der(4) while normal chromosomes 4 (N4) show single copies.

We also performed genomic profiling of CTCL cell lines HH and HUT-78, revealing copy number alterations that may underlie specific gene deregulations. The results for all chromosomes are shown in Figures S2 and S3, respectively. According to these data, we detected in HH a conspicuous amplicon at band 4q24–26 accompanied telomerically by a genomic deletion, coinciding with the translocation breakpoint at 4q26 inside der(4) (Figure 2A). The peak amplification (15–20x) included NFKB1, which encodes NFkB-TF p105/p50. FISH analysis using BAC probe 348F2 covering the NFKB1 gene confirmed this amplification and its peak localization to 4q24 together with additional iterative signals due to a chromosomal duplication (Figure 2B). Thus, we identified in CTCL cell line HH a genomic amplification at 4q24–26, which might be etiologically related to the chromosomal rearrangement t(1;4)(p32;q25). In the following, we focused on this conspicuous amplification, which may activate novel oncogenic players in CTCL.

### 3.2. Gene Analyses for Genomic Amplification at 4q24–26 in HH

#### 3.2.1. Aberrant Activation of NFKB1

**Figure 3:**
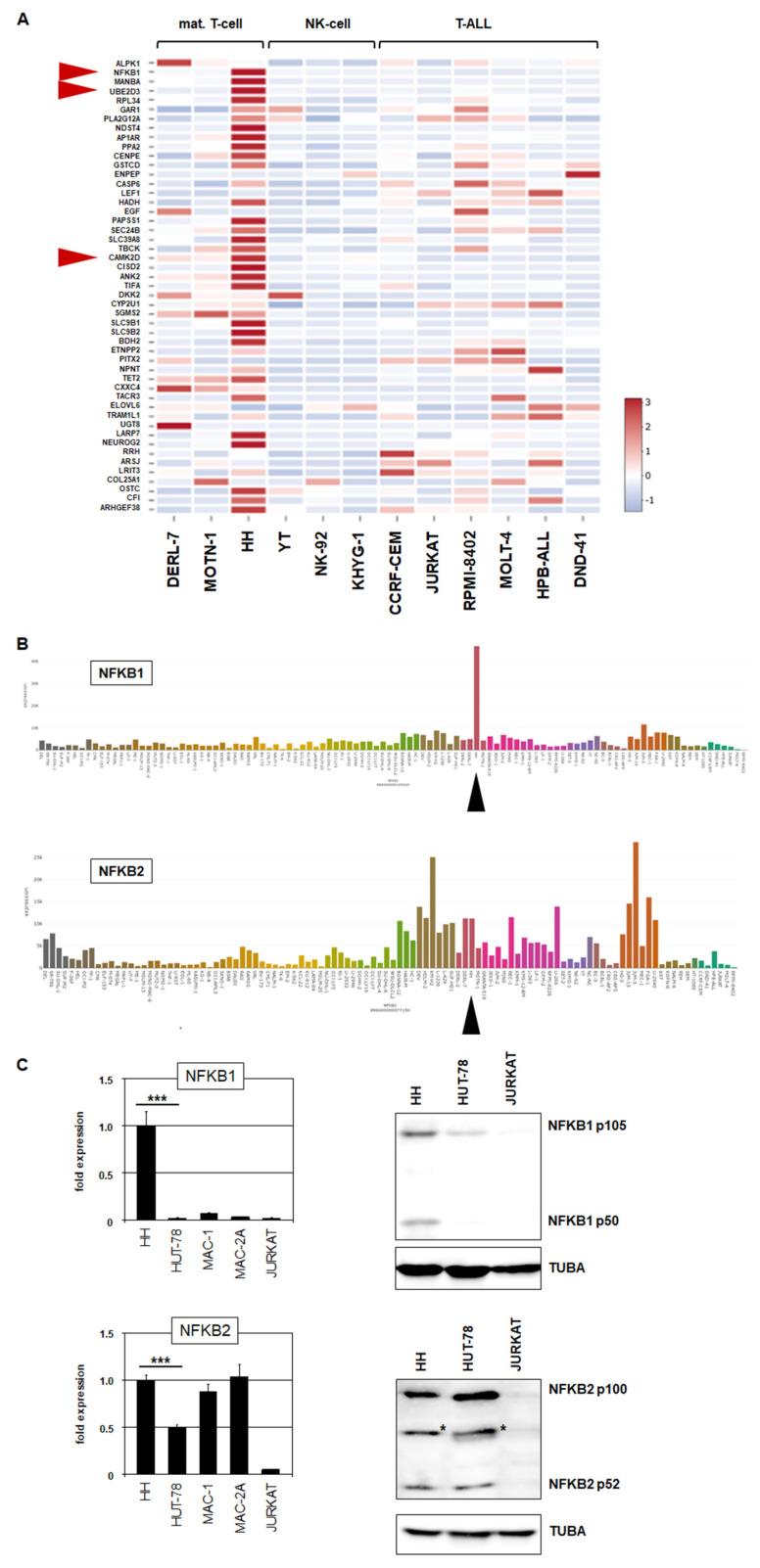
Amplification at 4q24–26 in HH activates NFKB1. (**A**) Heatmap showing RNA-seq-based expression levels for 49 genes amplified at 4q24–26 in HH. In comparison to CTCL cell line HH, two additional mature T-cell lines, three NK-cell lines, and six T-ALL cell lines were analyzed. Genes NFKB1, UBE2D3, and CAMK2D are indicated by red arrowheads. (**B**) LL-100 RNA-seq gene expression data for NFKB1 (above) and NFKB2 (below) are shown as bar plots. CTCL cell line HH is indicated by a black arrowhead. (**C**) RQ-PCR (left) and Western blot analysis (right) of NFKB1 (above) and NFKB2 (below) in CTCL cell lines and control T-cell line JURKAT. Artificial bands are labeled by asterisks. * *p* < 0.05, *** *p* < 0.001.

To investigate transcriptional consequences of the genomic amplification at 4q24–26, we analyzed LL-100 RNA-seq data of HH in comparison to 11 leukemia/lymphoma control cell lines comprising DERL-7, MOTN-1 (T-cell lymphoma), CCRF-CEM, JURKAT, RPMI-8402, MOLT-4, HPB-ALL, DND-41 (T-cell leukemia), and YT, NK-92, KHYG-1 (NK-cell leukemia/lymphoma). We assigned 49 amplified genes and illustrated their activities in a heatmap (Figure 3A). For HH, the data revealed overexpression of NFKB1 in addition to several other genes, including CAMK2D and UBE2D3, involved in NFkB signaling and the p53 pathway, respectively [28,29].

Expression data for NFKB1 using the complete LL-100 RNA-seq data set and RQ-PCR analysis of NFKB1 in CTCL and T-ALL cell lines showed high transcript levels exclusive to HH (Figure 3B,C). Recently, the closely related gene NFKB2 has been reported to be rearranged in HUT-78, generating a C-terminally deleted and thus constitutively activated protein [17]. Our genomic array data for HUT-78 demonstrated a corresponding microdeletion at 10q25 (Figure S4A). In addition, the data indicated that HUT-78 also bears a genomic deletion at NFKB1 (Figure S4B), suggesting similar consequences for this related TF, although it was not significantly expressed in this cell line. RQ-PCR analysis of NFKB2 showed elevated levels in CTCL cell lines while T-ALL control cell line JURKAT expressed low levels (Figure 3C).

Finally, Western blot analysis of NFKB1 and NFKB2 in CTCL and control cell lines showed results corresponding to the RNA data. Accordingly, NFKB1 was highly expressed in HH, while NFKB2 was elevated in all analyzed CTCL cell lines, including HH, HUT-78, MAC-1, and MAC-2A (Figure 3C). Taken together, in the CTCL cell line, HUT-78 NFKB2 is aberrantly activated via a genomic microdeletion at 10q25, while HH contains a genomic amplification at 4q24–26, which underlies aberrant overexpression of an otherwise normally configured NFKB1. Thus, NFKB1 may represent a novel oncogenic player in CTCL.

#### 3.2.2. Regulation and Sequence Analysis of NFKB1 in HH

**Figure 4:**
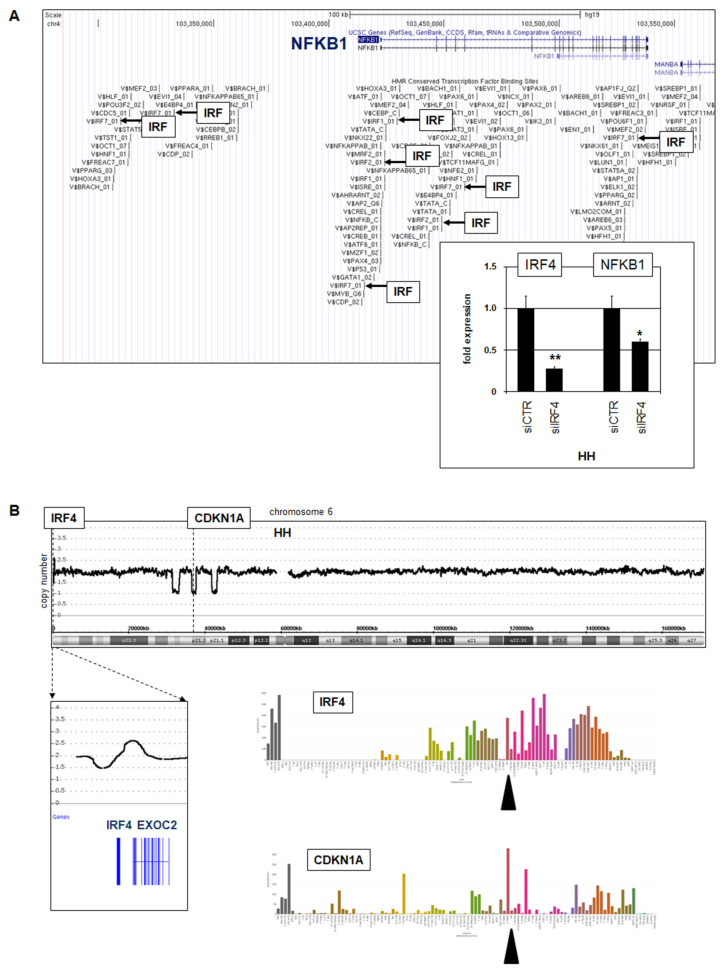
IRF4 activates NFKB1. (**A**) TF binding-site analysis using UCSC genome browser data indicates several potential IRF sites at NFKB1. RQ-PCR analysis of HH treated for siRNA-mediated knockdown of IRF4 demonstrated concomitant downregulation of IRF4 and NFKB1 (insert). Statistical significance was assessed by *t*-test and derived *p*-values indicated by asterisks (* *p* < 0.05, ** *p* < 0.01). (**B**) Genomic profiling data for chromosome 6 of CTCL cell line HH showing a genomic gain at 6p25 and three microdeletions at 6p21, targeting IRF4 and CDKN1A, respectively (above). LL-100 RNA-seq data showing expression levels of IRF4 and CDKN1A (below). The cell line HH is indicated by a black arrowhead.

To identify regulators driving transcription of the amplified and overexpressed gene NFKB1, we looked for potential TF binding sites using the UCSC genome browser (www.genome.cse.ucsc.edu/index.html; accessed on 1 March 2022). This exercise revealed several IRF sites (Figure 4A), suggesting that particular IRF family members may activate NFKB1. RNA-seq data showed prominent expression of IRF4 in HH (Figure 4B), representing an IRF-member involved in T-cell differentiation [30]. Subsequent siRNA-mediated knockdown of IRF4 resulted in concomitant downregulation of NFKB1 (Figure 4A), demonstrating that IRF4 activated NFKB1 transcription in this cell line.

Moreover, genomic array data indicated a gain at 6p25, which targets regulatory regions of the IRF4 gene and may plausibly contribute to elevated IRF4 expression in HH. In addition, chromosome 6 showed three microdeletions on its short arm, one of which targeted CDKN1A. Accordingly, HH expressed low transcript levels of this cell cycle regulator and tumor suppressor (Figure 4B). Taken together, in CTCL cell line HH we detected amplified and overexpressed NFKB1 subject to activation by IRF4, whose locus was, in turn, augmented by a genomic gain.

Recently, Park and colleagues described several mutations in CTCL patients, including H67Y in NFKB1 [31]. To analyze if this particular mutation is present in HH and/or HUT-78, we sequenced 10 cloned PCR products covering the mutated region from each cell line. However, our data indicated wild-type configurations for all sequences, discounting mutation NFKB1 (H67Y) therein.

#### 3.2.3. Target Gene Analysis of NFKB1 in HH

**Figure 5:**
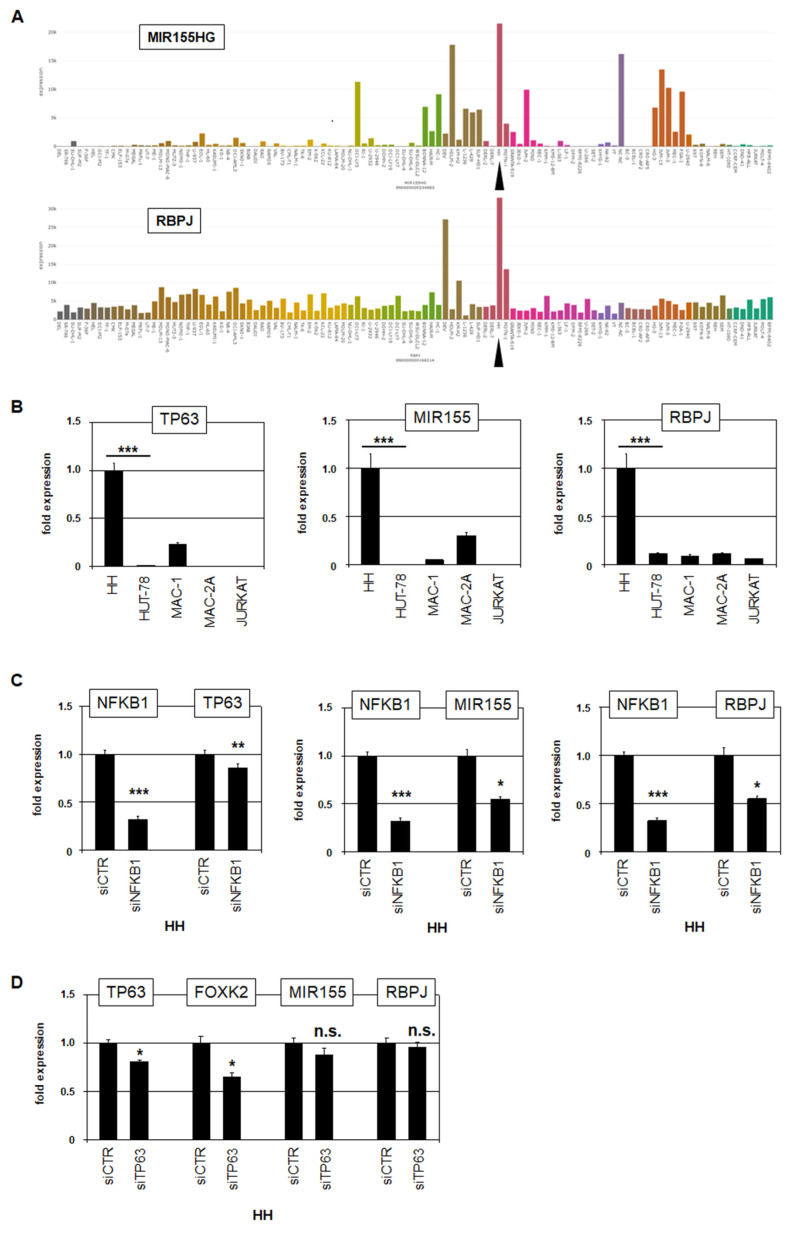
NFKB1 activates TP63, MIR155, and RBPJ in HH. (**A**) LL-100 RNA-seq data showing expression levels of MIR155 (above) and RBPJ (below). The cell line HH is indicated by a black arrowhead. (**B**) RQ-PCR analysis of TP63 (left), MIR155 (middle), and RBPJ (right) in CTCL and control cell lines showing high expression levels in HH. (**C**) RQ-PCR mediated target gene analysis of HH treated for siRNA-mediated knockdown of NFKB1. (**D**) RQ-PCR analysis of HH treated for siRNA-mediated knockdown of TP63. Statistical significance was assessed by *t*-test and derived *p*-values indicated by asterisks (* *p* < 0.05, ** *p* < 0.01, *** *p* < 0.001, n.s. not significant).

Enhanced NFKB1 expression was restricted to HH, while NFKB2 was highly expressed in all CTCL cell lines analyzed. To identify candidate target genes of NFKB1, we compared expression profiling data from HH and HUT-78. Among the top-1000 overexpressed genes in HH, we identified NFKB1, CAMK2D, UBE2D3, MIR155, RBPJ, and TP63 (Table S1). NFKB1, CAMK2D, and UBE2D3 were amplified at 4q24–26, TP63 was part of the novel fusion gene, while the loci of MIR155 (21q21) and RBPJ (4p15) were not genomically rearranged. RNA-seq data and RQ-PCR analysis confirmed elevated expression of TP63, MIR155, and RBPJ in HH (Figure S1 and Figure 5). SiRNA-mediated knockdown of NFKB1 resulted in concomitant downregulation of TP63, MIR155 and RBPJ (Figure 5C), demonstrating that NFKB1 activated these genes in HH. TP63 was fused to FOXK2 and, thereby, N-terminally deleted, forming a constellation reported to operate oncogenically [32]. MIR155 is a negative gene regulator aberrantly overexpressed in CTCL patients [33,34]. RBPJ encodes a TF that interacts with NOTCH, indicating aberrant activation of NOTCH signaling via RBPJ overexpression. The NOTCH pathway is aberrantly activated in several T-cell malignancies, including CTCL [35,36,37]. Thus, we identified three oncogenes, namely FOXK2::TP63, MIR155, and RBPJ, which were activated by NFKB1 in HH.

Furthermore, NFkB factors reportedly cooperate with TP63 [38]. To analyze if the identified fusion gene FOXK2::TP63 cooperates with NFKB1 in HH, we performed siRNA-mediated knockdown of the fusion transcript by targeting the TP63 moiety. Subsequent RQ-PCR analysis confirmed the knockdown of the fusion partners TP63 and FOXK2. However, the expression levels of MIR155 and RBPJ remained unperturbed (Figure 5D), discounting cooperation between NFKB1 and FOXK2::TP63 in the regulation of these genes. Collectively, our data show that NFKB1 activates well-known oncogenic processes in CTCL, supporting the pathological role of aberrantly expressed NFkB factors in this malignancy.

### 3.3. Analyses of Selected Copy Number Alterations in HH and HUT-78

#### 3.3.1. Deregulation of CAMK2D, UBE2D3, and TP53 in HH

**Figure 6:**
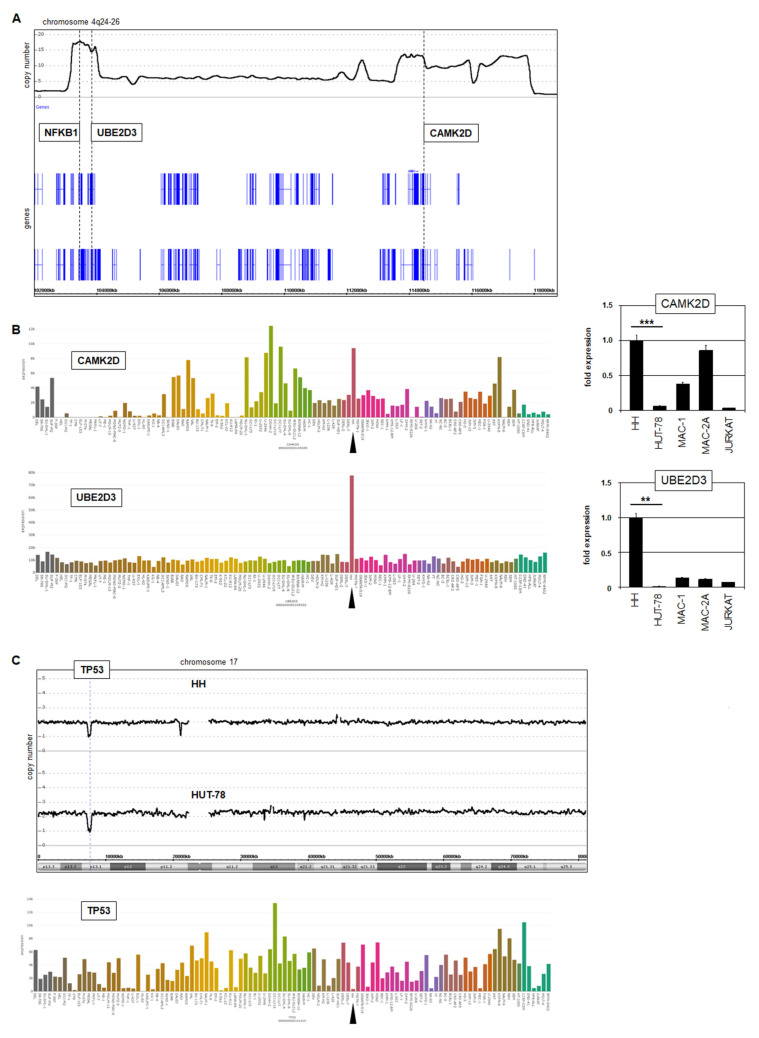
Additional copy number alterations in HH and HUT-78. (**A**) Genomic profiling data for HH showing the amplified region at 4q24–26, which hosts the genes NFKB1, UBE2D3 and CAMK2D. (**B**) LL-100 RNA-seq data (left) and RQ-PCR analysis (right) of CAMK2D and UB2D3 showing elevated expression levels in HH (indicated by an arrowhead). Statistical significance was assessed by *t*-test and derived *p*-values indicated by asterisks (** *p* < 0.01, *** *p* < 0.001). (**C**) Genomic profiling data for chromosome 17 of HH and HUT-78 showing microdeletions at 17p12 covering TP53 (above). LL-100 RNA-seq data of TP53 (below) showing reduced expression levels in HH (arrowhead).

Our genomic profiling data for HH showed that CAMK2D and UBE2D3 were co-amplified with NFKB1 at 4q24–26 (Figure 6A). LL-100 RNA-seq data and RQ-PCR analysis demonstrated their enhanced expression in this cell line (Figure 6B). CAMK2D encodes a kinase that reportedly interacts with IKKB to drive NFkB signaling [28]. Elevated expression levels of CAMK2D were observed in cell lines derived from other entities as well, indicating a general impact on hematopoietic malignancies. In contrast, UBE2D3 overexpression was restricted to cell line HH, demonstrating a more specific relationship. UBE2D3 encodes a ubiquitin-conjugating enzyme mediating the degradation of tumor suppressor p53 [29]. Moreover, one allele of TP53 was genomically deleted in both HH and HUT-78 (Figure 6B). Thus, our data for CTCL cell line HH revealed different modes of TP53-deregulation, forming an important step in the pathogenesis of this malignancy [1,31,39].

#### 3.3.2. Amplification of ID1 and IKZF2 in HUT-78

**Figure 7:**
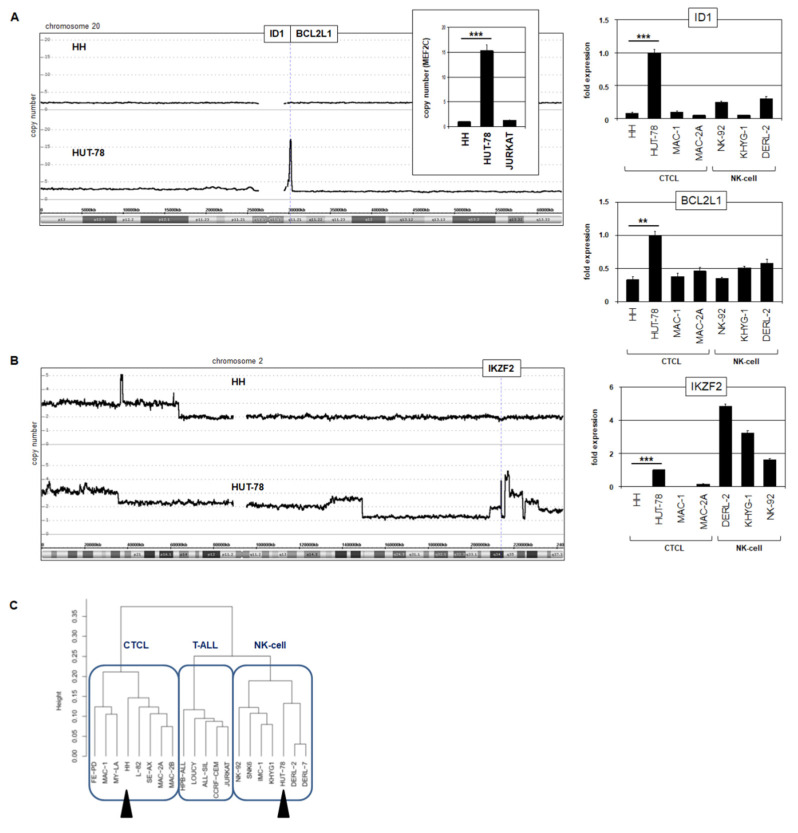
Amplification of NK-cell factors in HUT-78. (**A**) Genomic profiling data of chromosome 20 for HH and HUT-78 showing an amplification at 20q11 in HUT-78 that contains the genes ID1 and BCL2L1 (left). Copy number analysis of ID1 in comparison to MEF2C by RQ-PCR for three cell lines. The values for HH were set to unity (insert). RQ-PCR analysis of ID1 and BCL2L1 showing elevated expression levels in HUT-78 (right). NK-cell lines served as additional controls. (**B**) Genomic profiling data of chromosome 2 for HH and HUT-78 showing amplification of IKZF2 at 2q34 (left). RQ-PCR analysis of IKZF2 (left) showing elevated expression levels in HUT-78 and selected NK-cell lines (right). Statistical significance was assessed by *t*-test and derived *p*-values indicated by asterisks (** *p* < 0.01, *** *p* < 0.001). (**C**) Dendrogram illustrating the result of a cluster analysis for expression profiling data from CTCL, T-ALL, and NK-cell cell lines. The cell lines HH and HUT-78 are segregated and indicated by black arrowheads.

Analysis of our genomic profiling data for HUT-78 revealed two interesting copy number alterations. First, a compact focal amplification at 20q11 comprised the genes ID1 and BCL2L1 (Figure 7A). Genomic RQ-PCR analysis confirmed amplification of ID1, and expression analysis via RQ-PCR demonstrated enhanced transcript levels of both ID1 and BCL2L1 in HUT-78 (Figure 7A). ID1 encodes a repressive TF of the helix-loop-helix family and BCL2L1 a survival factor of the BCL2 family, both potential oncogenes.

Second, a complex amplification at 2q34 contained IKZF2, which showed enhanced expression levels in HUT-78 (Figure 7B). IKZF2 encodes a TF physiologically involved in NK-cell differentiation, while ID1 may aberrantly substitute the role of ID2 and ID3 in the same developmental lineage [40,41]. Both ID1 and IKZF2 were elevated in HUT-78 and NK-cell lines but not in HH. Interestingly, cluster analysis of expression profiling data for selected cell lines derived from CTCL, T-ALL, and NK-cell leukemia/lymphoma assigned HH and HUT-78 to separate entities (Figure 7B). HH clustered within the CTCL group while HUT-78 fell within the NK-cell group, reflecting deregulation of the driving NK-cell lineage TFs ID1 and IKZF2.

### 3.4. Functional Analyses of NFkB and NOTCH Pathways in CTCL Cell Lines

**Figure 8:**
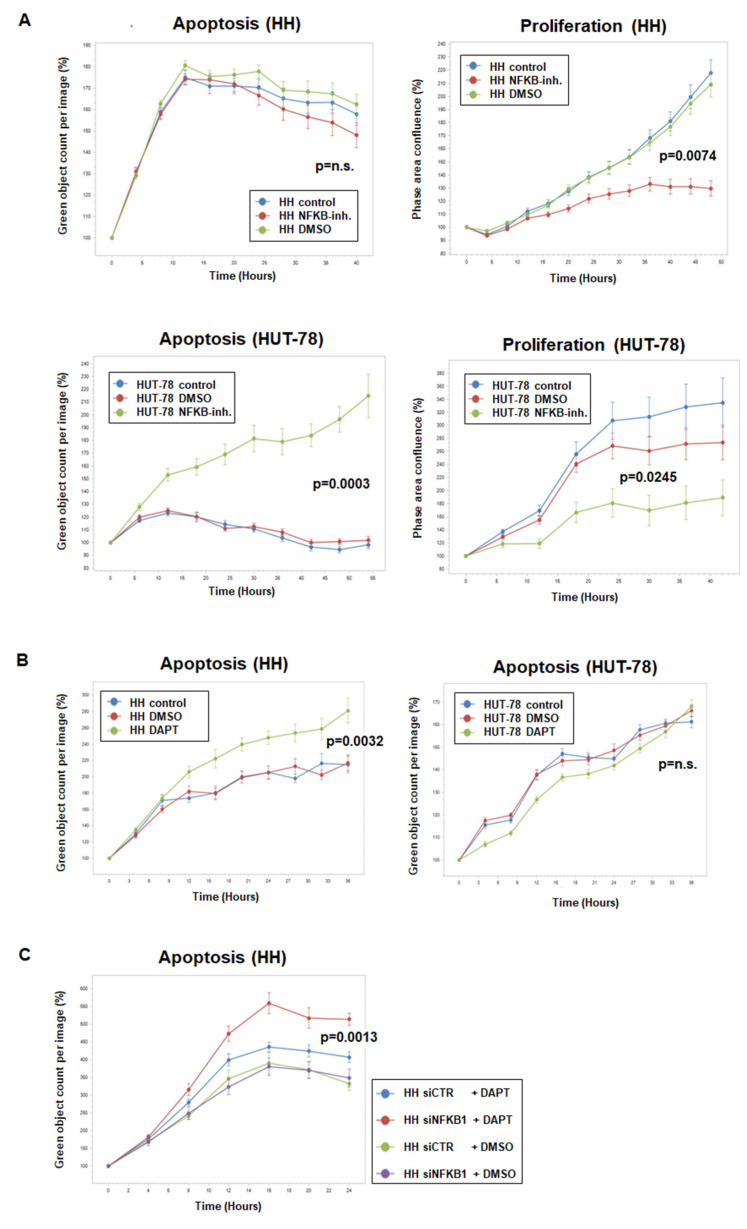
Live-cell imaging analysis of CTCL cell lines HH and HUT-78. (**A**) Analysis of apoptosis (left) and proliferation (right) of HH (above) and HUT-78 (below) after treatment with NFkB inhibitor. (**B**) Analysis of apoptosis in HH (left) and HUT-78 (right) after treatment with NOTCH-inhibitor DAPT. (**C**) Analysis of apoptosis in HH after siRNA-mediated knockdown of NFKB1 and simultaneous treatment with NOTCH-inhibitor DAPT. Indicated *p*-values refer to terminal time points of treated versus control cells.

Using cell line HH as a model and resource to reveal novel genomic rearrangements driving gene deregulation in CTCL, we identified overexpressed NFKB1 and RBPJ. These data indicated that NFkB and NOTCH signaling play oncogenic roles in HH. For functional studies, we treated HH and HUT-78 with NFkB inhibitor for two days and performed live-cell-imaging analyses. Representative images are shown in Figure S5. The results demonstrated that NFkB signaling supported proliferation in both cell lines, while the additional impact on apoptosis was detected solely in HUT-78 (Figure 8A). Treatment of these cell lines with NOTCH-inhibitor DAPT indicated that NOTCH signaling supported survival in HH, unlike HUT-78 (Figure 8B). Finally, siRNA-mediated knockdown of NFKB1 enhanced the pro-apoptotic effect of NOTCH-inhibitor DAPT in HH (Figure 8C), demonstrating the indirect contribution of NFKB1 to survival.

Taken together, these experiments revealed differences in the sensitivities and responses to pharmacological inhibition of NFkB and NOTCH pathways, indicating that targeted gene deregulations in CTCL subsets carry significant therapeutic potential.

## 4. Discussion

**Figure 9:**
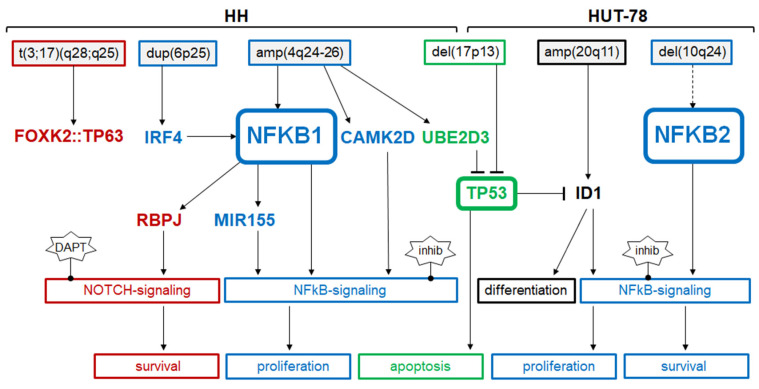
Summary of the results from this study showing upstream and downstream factors of NFKB factors in CTCL cell lines HH and HUT-78. Chromosomal aberrations are indicated above. Factors and functions are shown for NFkB signaling (indicated in blue), NOTCH signaling (red), p53 pathway (green), and NK-cell differentiation (black).

Hematopoietic tumor cell lines bear chromosomal and genomic aberrations, corresponding to the derived patient and tumor entity. Thus, they represent suitable model systems and may serve as resources to identify novel oncogenes [21,42]. CTCL cell line HUT-78 harbors a genomic aberration that targets the gene NFKB2, generating a truncated and thus activated TF [17]. This oncogenic rearrangement has been subsequently detected in CTLC patients, confirming the clinical relevance of this finding and deregulated NFkB signaling in this malignancy [17,18]. In our study, we used CTCL cell line HH as a model for this disease and performed karyotyping and copy number analyses. The resulting data revealed novel rearrangements underlying deregulation of clinically relevant oncogenes and tumor suppressor genes. Knockdown experiments and functional live-cell imaging analyses were employed to evaluate the identified genes and to generate an aberrant gene network shown in Figure 9.

We detected in HH a novel fusion gene, FOXK2::TP63, generated by t(3;17)(q28;q25). Fusion genes containing TP63 have been reported in various T-cell malignancies, including CTCL [2,10,12]. These rearrangements convert TP63 into an oncogene. The encoded truncated part of TP63 shows structural similarity to an isoform lacking the N-terminus. N-terminal deleted p63 differs functionally from the full-length isoform in the regulation of apoptosis and inhibition of family member p53 [32]. FOXK2 encodes a TF of the forkhead-box family [43]. It shows ubiquitous activity in hematopoietic cells, pointing to a role as a universal driver of truncated TP63. In breast cancer, lung cancer, and glioma, FOXK2 performs tumor suppressor activity that may correspond to its mutation in the context of this fusion gene [44,45,46]. However, in colorectal cancer, FOXK2 operates as an oncogene and is activated by NFkB signaling [47]. The latter effect may be of relevance in CTCL as well. Thus, the role of FOXK2 in CTCL remains to be resolved while our findings highlight rearranged TP63 as an important oncogene in CTCL.

Additionally, we detected the fusion gene TBLXR1::TP63 in cell line MOTN-1. Both FOXK2 and TBLXR1 are ubiquitously expressed in hematopoietic cell lines. In contrast, TP63 is expressed in selected cell lines at various levels. Thus, wild-type-configured TP63 is expressed in HH but not in MOTN-1. However, in both cell lines, the level of TP63 transcript was enhanced, possibly due to the fusion to FOXK2 or TBLXR1, respectively.

Chromosomal rearrangement t(1;4)(q25;q26) in HH was intimately connected with the complex amplification at 4q24–26, targeting the NFKB1 locus. NFKB1 and NFKB2 are functionally related and encode TFs, which execute gene regulation via the NFkB signaling pathway. This pathway plays an oncogenic role in several hematopoietic malignancies, including CTCL [48,49]. Our data highlight the role of the NFkB pathway in the proliferation and survival of the affected tumor cells and endorse the cell lines HH and HUT-78 as tools for investigating the therapeutic targeting of NFkB factors. Thus, two different chromosomal aberrations mediate oncogenic activation of NFkB-TFs in CTCL—genomic deletion at 10q24 activated NFKB2 via truncation in HUT-78, and amplification at 4q24 resulted in overexpression of NFKB1 in HH.

Target gene analysis of NFKB1 revealed transcriptional activation of MIR155 and RBPJ. MIR155 encodes the micro-RNA miR155, which affects the suppression of specific genes and operates as an oncogene in several hematopoietic malignancies, including CTCL [33,34]. The expression of miR155, together with other micro-RNAs, has diagnostic relevance, permitting differentiation of CTCL from benign skin diseases and mycosis fungoides from Sézary syndrome [50,51]. Finally, MIR155 is involved in NFkB signaling and has been described as an NFkB target in bronchial epithelial cells and as an NFkB activator via repression of IKKE in immune cells [52,53]. Thus, MIR155 operates as an oncogene in CTCL and is part of the NFkB pathway.

RBPJ encodes a TF that interacts with the activated intracellular NOTCH receptor to regulate NOTCH target genes. The aberrantly activated NOTCH pathway plays an oncogenic role in T-cell malignancies, including CTCL [35,36,37]. Our functional analyses indicated that NFKB1-mediated NOTCH activation supported the survival of the tumor cells. Furthermore, NFKB1 may cooperate with FOXK2::TP63 in gene regulation [38]. However, our data discounted the coregulation of MIR155 and RBPJ.

The amplicon at 4q24–26 also contained the genes CAMK2D and UBE2D3, which are involved in NFkB signaling and p53 degradation, respectively. CAMK2D encodes a protein kinase that binds and inhibits the repressor-protein IKKB, resulting in activation of NFkB signaling [28]. Downregulation of p53 plays an important oncogenic role in CTCL [1,31,39]. In addition to deletion of the TP53 locus at 17p13, our data for HH showed aberrant overexpression of the ubiquitin-conjugating enzyme UBE2D3, previously shown to mediate p53-ubiquitination and degradation [29]. Therefore, together with the aforementioned activity of TP63 fusion proteins, these results revealed three independent mechanisms of p53 inhibition in CTCL.

Finally, we identified enhanced expression of ID1 and IKZF2 in CTCL cell line HUT-78. These TFs are involved in NK-cell differentiation and may, thus, shift the phenotype of HUT-78 toward NK-cells as indicated by cluster analysis [40,41]. CTCL cells from patients are heterogeneous and reportedly carry NK-cell markers, supporting our findings and interpretations [54]. ID1 is negatively regulated by p53 [55]. Suppressed TP53 may, thus, support ID1 expression in CTCL. Moreover, ID1 activates and interacts with NFkB [56,57]. Thus, amplification and overexpression of ID1 have two pathological effects: alteration of cell identity and activation of oncogenic NFkB signaling.

## 5. Conclusions

We have identified widespread genomic alterations in CTCL cell line HH, separately generating the fusion gene FOXK2::TP63, overexpression of NFKB1, and three different modes of p53 suppression. Our data highlight the pathogenic impact of NFkB and NOTCH signaling in CTCL, recommending therapeutic targeting of these pathways. CTCL cell lines HH and HUT-78 differ in mode and type of aberrantly activated NFkB factors and may, thus, serve to develop adjusted treatment protocols for affected patient subsets. However, additional analyses are required to support our findings in patients.

## Data Availability

Data are contained within the article or Supplementary Materials.

## Supplementary Materials

The following supporting information can be downloaded at: https://www.mdpi.com/article/10.3390/biomedicines10082038/s1, Figure S1: Gene expression via RNA-seq data; Figure S2: Copy number analysis of HH; Figure S3: Copy number analysis of HUT-78; Figure S4: Copy number state of NFKB2 and NFKB1 in HUT-78; Figure S5: live-cell imaging data; Table S1: Comparative gene expression profiling of HH and HUT-78.
