# Supplementary material for: Genomic Aberrations Generate Fusion Gene FOXK2::TP63 and Activate NFKB1 in Cutaneous T-Cell Lymphoma
Source: Biomedicines. 2022 Aug 21;10(8):2038. doi: 10.3390/biomedicines10082038 (PMC9406051; doi:10.3390/biomedicines10082038)
Supplement: Supplementary file 1 [file biomedicines-10-02038-s001.zip › biomedicines-1835044-supplementary.pdf]

Figure S1

A

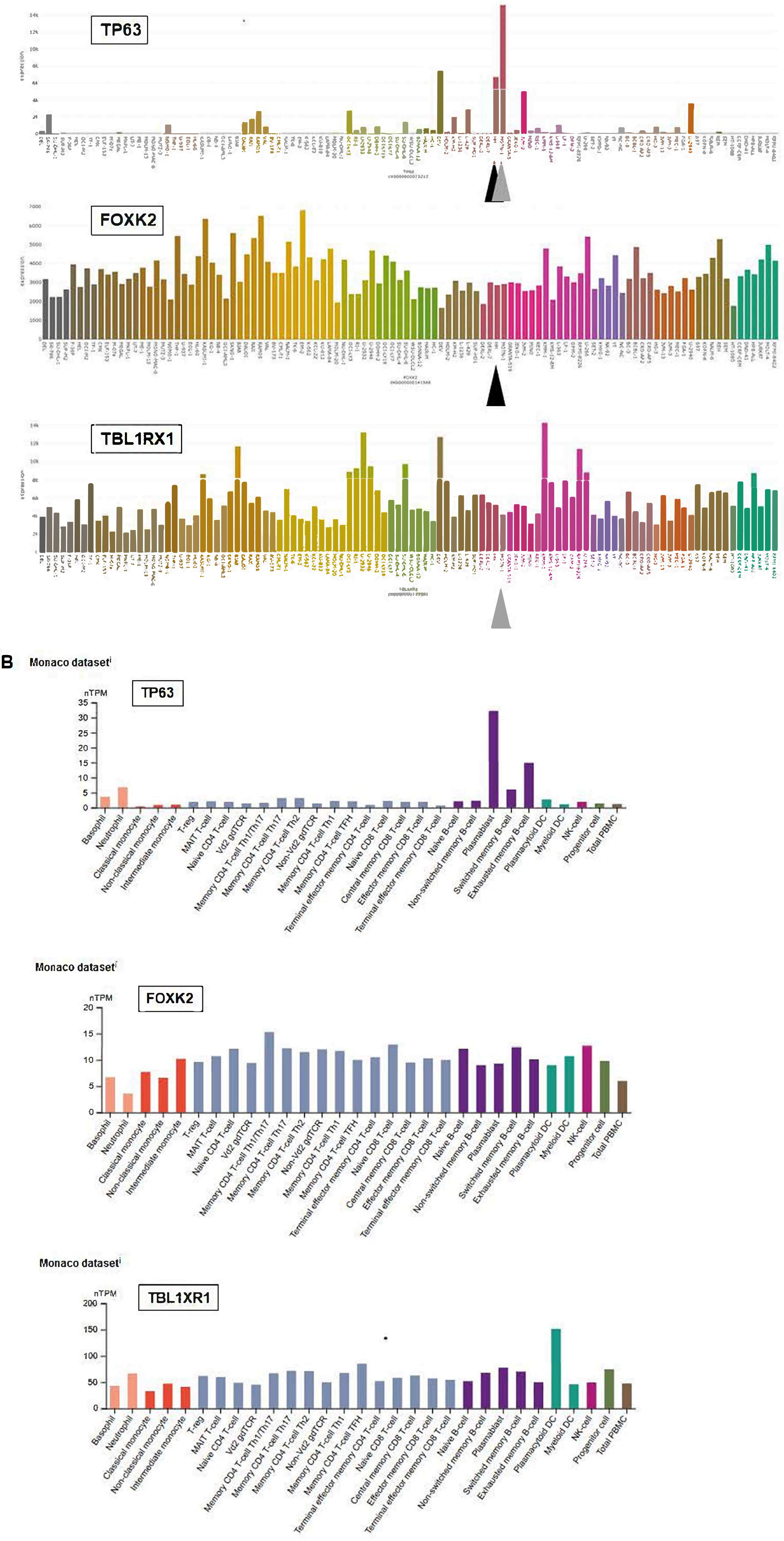

Figure S1. RNA-seq gene expression levels for TP63, FOXK2 and TBL1RX1 are shown for 100 leukemia/Lymphoma cell lines (E-MTAB-7721). The colors of the bars refer to different disease entities. CTCL cell line HH is indicated by a black and T-LGL cell line MOTN-1 by a grey arrowhead. (B) RNA-seq gene expression levels for TP63, FOXK2 and TBL1RX1 were obtained from The Human Protein Atlas (www.proteinatlas.org).

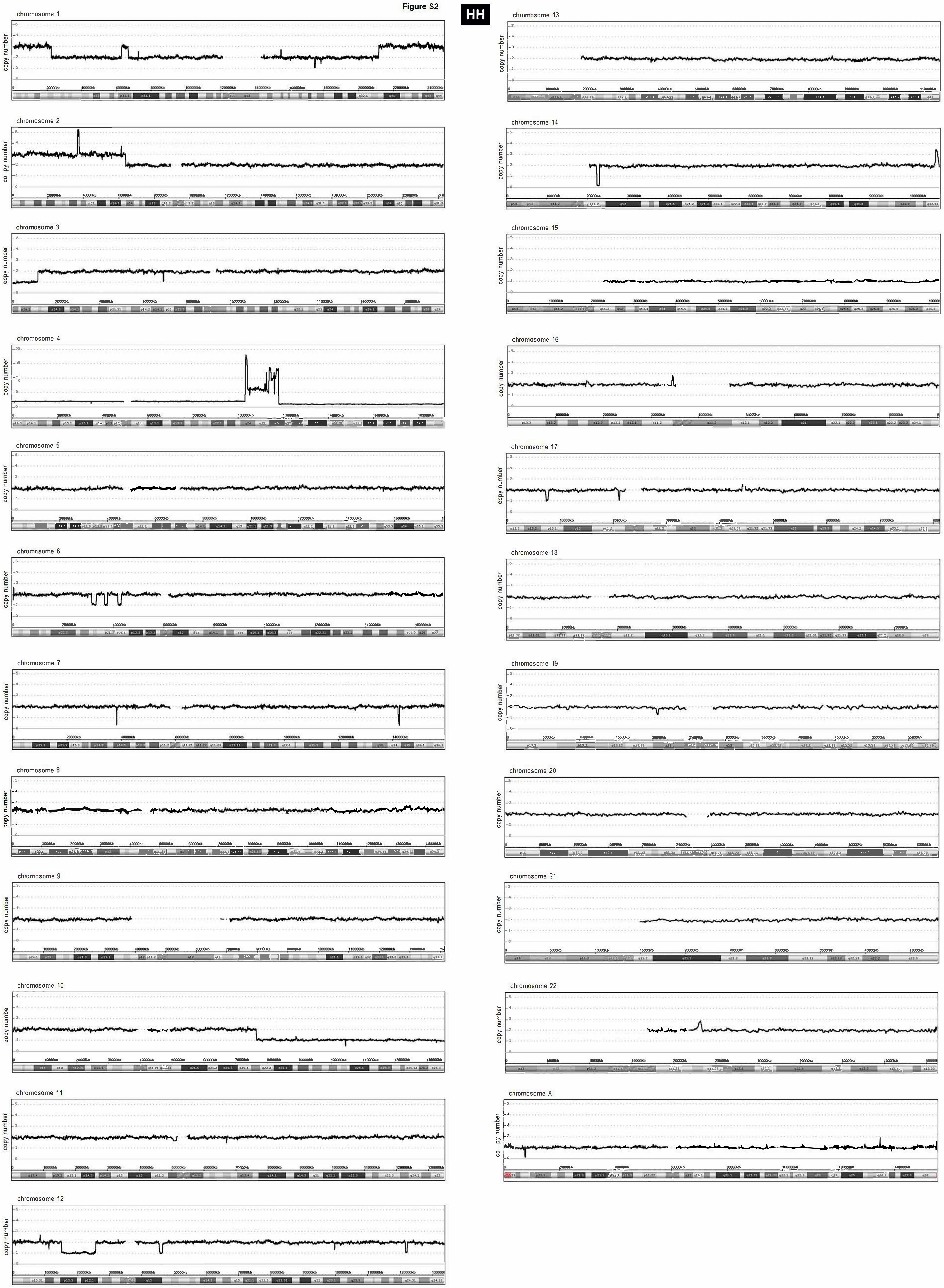

Figure S2. Copy number data for all chromosomes from CTCL cell line HH generated by genomic profiling.

Figure S3

HUT-78

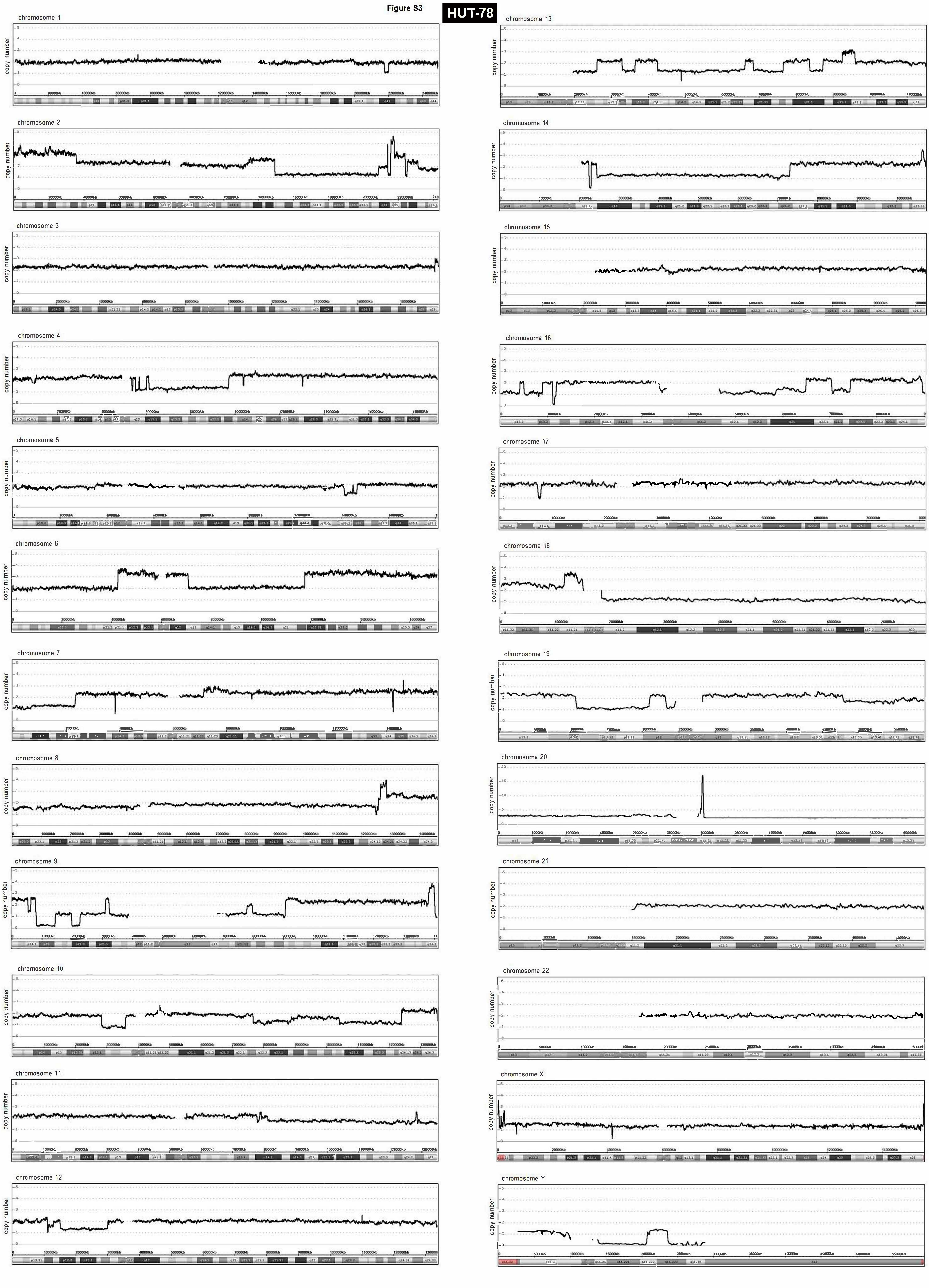

Figure S3. Copy number data for all chromosomes from CTCL cell line HUT-78 generated by genomic profiling.

Figure S4

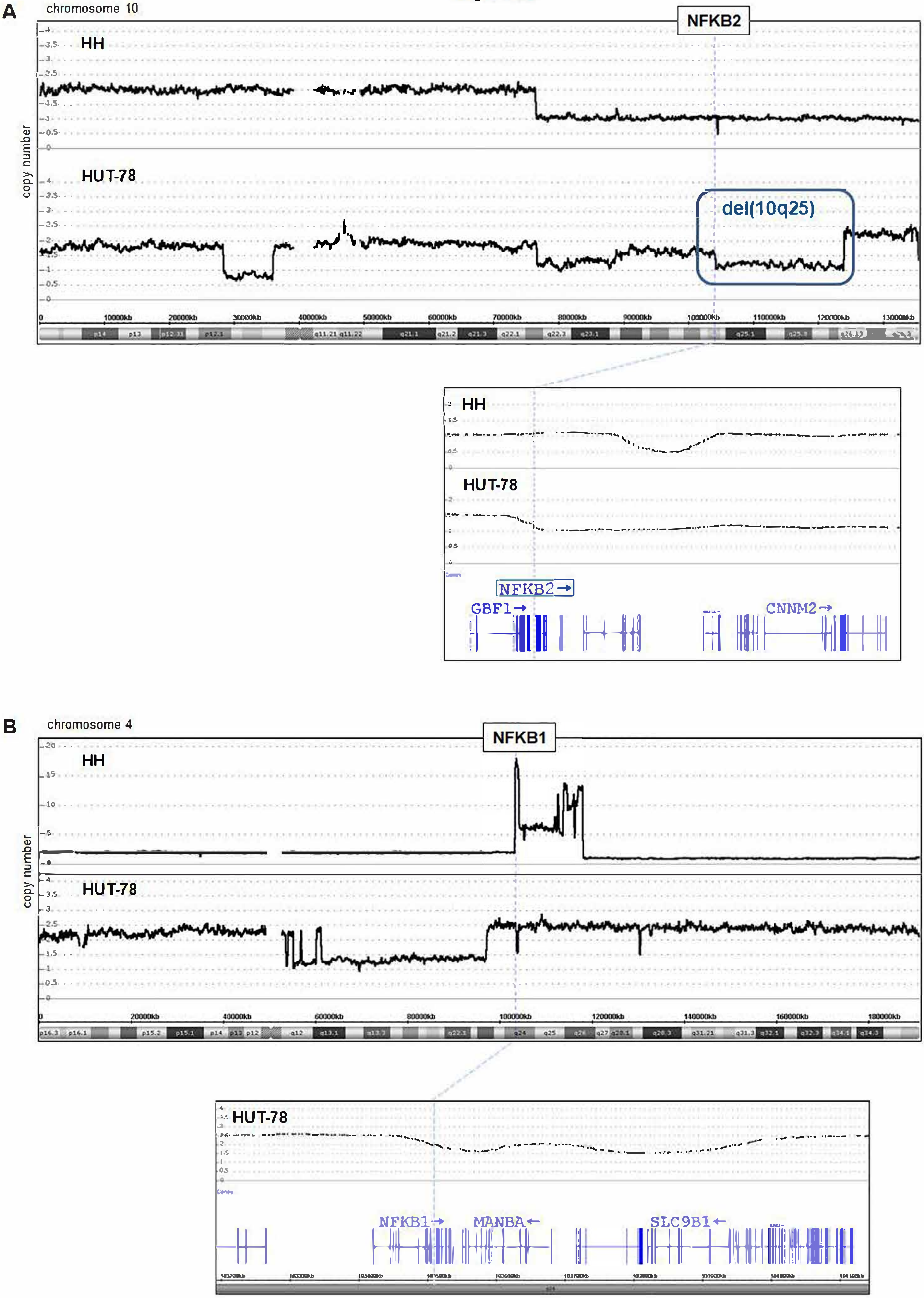

Figure S4. Copy number data for chromosome 10 (A) and chromosome 4 (B) from CTCL cell lines HH and HUT-78. Enlarged regions and corresponding genes are shown below.

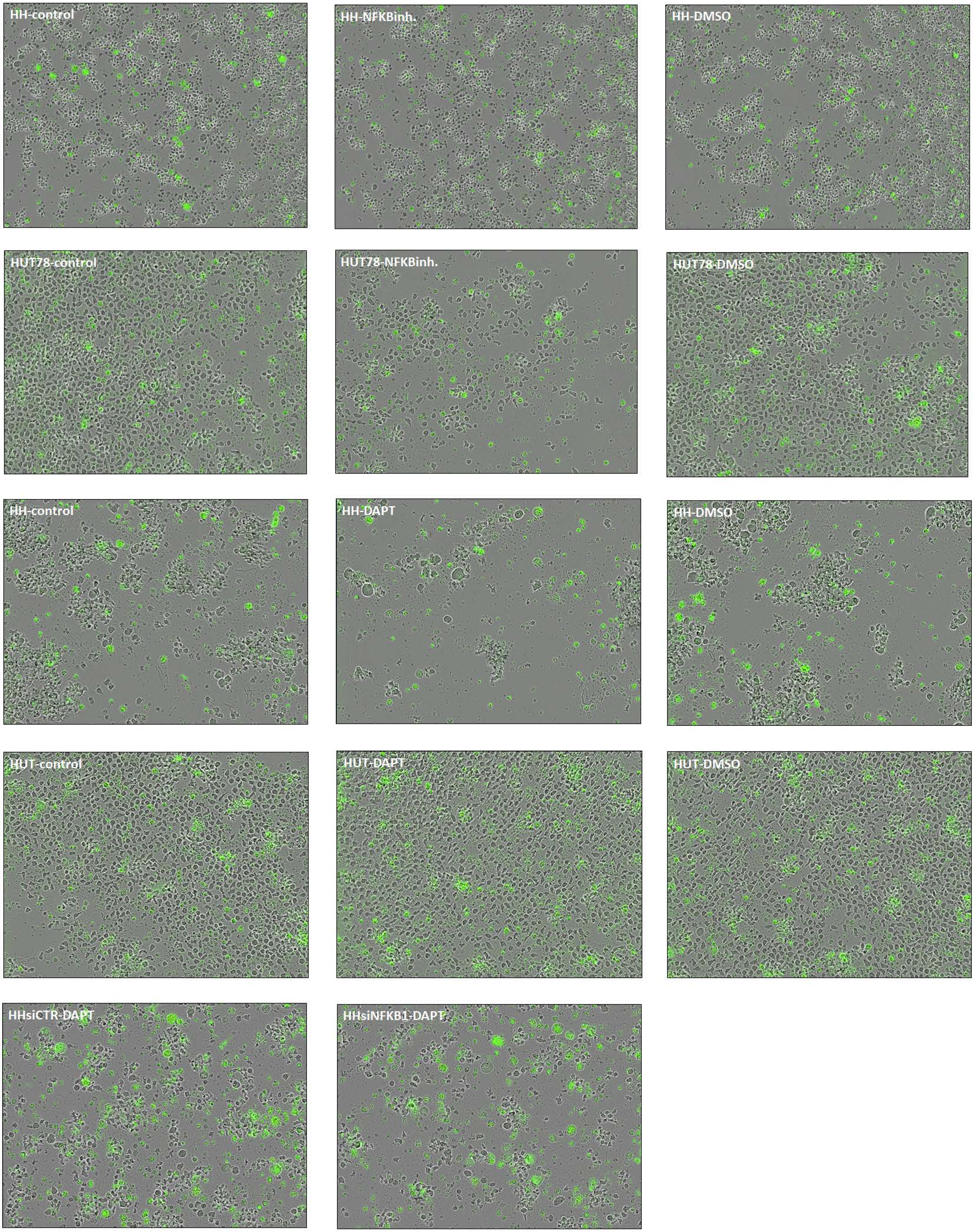

Figure S5

Representative live-cell-imaging data showing microscopical pictures of living cells (HH and HUT-78) taken at the last day of analysis (see also Figure 8). Green cells indicate apoptosis.

Table S1: Comparative gene expression profiling of HH and HUT-78.

Probe set ID	gene	HH	HUT-78	HH-HUT78
212698_s_at	40422	6,755103438	-2,4284334	9,183536838
222974_at	IL22	6,317272412	-2,4284334	8,745705812
213831_at	HLA-DQA1 /// LOC	6,327960064	-2,142286822	8,470246886
223062_s_at	PSAT1	5,903781497	-2,312054429	8,215835926
226517_at	BCAT1	5,986343899	-2,186086603	8,172430502
209301_at	CA2	5,821635598	-2,142286822	7,96392242
238605_at	NOL4	5,115580177	-2,536339282	7,651919458
200824_at	GSTP1	6,989659199	-0,661178575	7,650837774
224499_s_at	AICDA	5,145580394	-2,501359113	7,646939507
206045_s_at	NOL4	5,923116008	-1,665791776	7,588907784
225285_at	BCAT1	6,312054481	-1,234835326	7,546889807
244050_at	PTPLAD2	5,014397839	-2,466349414	7,480747254
201438_at	COL6A3	5,795699422	-1,665791776	7,461491198
228438_at	LOC100132891	4,996753949	-2,4284334	7,425187349
233052_at	DNAH8	5,792565538	-1,616924751	7,409490289
227034_at	SOWAHC	5,685228499	-1,715576125	7,400804624
216620_s_at	ARHGEF10	6,328409443	-1,057301851	7,385711294
243175_at	UTS2D	5,335944076	-2,049747196	7,385691272
209480_at	HLA-DQB1	5,184742504	-2,186086603	7,370829107
202363_at	SPOCK1	5,413285247	-1,956681069	7,369966317
1559646_a_at	LINC00184	5,514518512	-1,763664074	7,278182586
207245_at	UGT2B17	4,653725229	-2,536339282	7,190064511
212386_at	TCF4	5,412242272	-1,763664074	7,175906346
238870_at	KCNK9	5,332289905	-1,763664074	7,095953979
203642_s_at	COBLL1	4,799977847	-2,27001641	7,069994257
219496_at	SOWAHC	4,650069766	-2,350444759	7,000514525
1559645_at	LINC00184	4,540832283	-2,142286822	6,683119105
229764_at	LOC100505861 ///	6,38982439	-0,231045907	6,620870297
205638_at	BAI3	4,144849571	-2,466349414	6,611198985
226676_at	ZNF521	3,795218988	-2,8064286	6,601647587
205573_s_at	SNX7	4,718186291	-1,862269442	6,580455733
236203_at	HLA-DQA1 /// LOC	3,903941986	-2,665156778	6,569098764
203998_s_at	SYT1	3,754428453	-2,8064286	6,560857053
226677_at	ZNF521	3,513985141	-3,035090645	6,549075786
213415_at	CLIC2	4,543207821	-2,005029581	6,548237402
224412_s_at	TRPM6	4,191105499	-2,350444759	6,541550258
212387_at	TCF4	4,326621119	-2,142286822	6,46890794
203355_s_at	PSD3	4,038663603	-2,4284334	6,467097003
203753_at	TCF4	4,142807036	-2,312054429	6,454861465
219841_at	AICDA	4,834334905	-1,616924751	6,451259656
224894_at	YAP1	5,018947904	-1,422257026	6,44120493
228253_at	LOXL3	5,328536884	-1,100943374	6,429480258
220138_at	HAND1	4,711723394	-1,715576125	6,427299519
209032_s_at	CADM1	5,049246526	-1,372734086	6,421980612
223709_s_at	WNT10A	5,078657307	-1,32740143	6,406058737
231303_at	LINC00158	3,978219531	-2,391863529	6,370083059
202437_s_at	CYP1B1	3,529682801	-2,832036647	6,361719448
208121_s_at	PTPRO	3,931608669	-2,4284334	6,360042069

235278_at	MACROD2	4,628242806	-1,715576125	6,343818931
206065_s_at	DPYS	4,335356502	-2,005029581	6,340386083
219049_at	CSGALNACT1	3,726612406	-2,603471267	6,330083673
210999_s_at	GRB10	5,182433607	-1,145057014	6,327490621
206134_at	ADAMDEC1	5,766181318	-0,554696666	6,320877984
218097_s_at	CUEDC2	4,043821727	-2,27001641	6,313838137
201212_at	LGMN	5,578223842	-0,734355396	6,312579238
201427_s_at	SEPP1	4,402175336	-1,862269442	6,264444778
213010_at	PRKCDBP	4,05770789	-2,186086603	6,243794493
232968_at	FANK1	4,230697503	-2,005029581	6,235727084
203999_at	SYT1	4,945274128	-1,282520722	6,22779485
231969_at	STOX2	3,513985141	-2,695640852	6,209625993
209099_x_at	JAG1	5,961924469	-0,172194225	6,134118693
201798_s_at	MYOF	4,265403137	-1,862269442	6,127672579
209894_at	LEPR	3,704211994	-2,391863529	6,096075523
219355_at	CXorf57	4,116324812	-1,956681069	6,073005881
207480_s_at	MEIS2	4,335356502	-1,715576125	6,050932627
200632_s_at	NDRG1	7,031085546	0,996453274	6,034632273
210432_s_at	SCN3A	3,636292808	-2,391863529	6,028156337
236787_at	LOC100507286	4,209562243	-1,763664074	5,973226317
214720_x_at	40422	4,087244545	-1,862269442	5,949513987
208334_at	NDST4	3,991087145	-1,956681069	5,947768214
210473_s_at	GPR125	3,322661262	-2,603471267	5,926132528
226333_at	IL6R	4,306090192	-1,616924751	5,923014943
226545_at	CD109	4,441588953	-1,471833228	5,913422181
213891_s_at	TCF4	4,799977847	-1,100943374	5,900921221
202241_at	TRIB1	5,343407632	-0,554696666	5,898104298
221185_s_at	IQCG	6,481731789	0,600385088	5,881346701
226771_at	ATP8B2	4,586396217	-1,282520722	5,868916939
221045_s_at	PER3	3,161901883	-2,695640852	5,857542735
210527_x_at	TUBA3C /// TUBA3	5,944981929	0,109458907	5,835523022
222943_at	GBA3	4,022766247	-1,763664074	5,786430321
218613_at	PSD3	3,816747242	-1,956681069	5,773428311
229437_at	MIR155 /// MIR15	6,779494612	1,008210238	5,771284374
203132_at	RB1	3,978219531	-1,763664074	5,741883605
204917_s_at	MLLT3	3,816747242	-1,911206569	5,727953811
204301_at	KBTBD11	3,223556135	-2,466349414	5,689905549
209928_s_at	MSC	3,8018113	-1,862269442	5,664080742
1554600_s_at	LMNA	5,355255298	-0,290694975	5,645950273
225673_at	MYADM	4,600469559	-1,015398016	5,615867575
212086_x_at	LMNA	5,645320963	0,03784737	5,607473593
220132_s_at	CLEC2D	5,490289402	-0,089635713	5,579925114
1563209_a_at	MACROD2	3,252865595	-2,312054429	5,564920024
1553678_a_at	ITGB1	5,590492943	0,03784737	5,552645572
225447_at	GPD2	3,873276566	-1,665791776	5,539068342
207315_at	CD226	3,559161816	-1,956681069	5,515842886
204797_s_at	EML1	3,450420588	-2,049747196	5,500167784
231501_at	LOC100507162	3,296548503	-2,186086603	5,482635106
225123_at	SESN3	3,928402242	-1,520022123	5,448424365
203411_s_at	LMNA	6,077520912	0,634301128	5,443219784

230978_at	LOC100505659	3,906843271	-1,520022123	5,426865394
202085_at	TJP2	4,906308159	-0,518232988	5,424541147
203641_s_at	COBLL1	3,278243793	-2,142286822	5,420530614
228067_at	C2orf55	3,185443259	-2,229233437	5,414676697
224895_at	YAP1	4,328738561	-1,057301851	5,386040411
227828_s_at	FAM176A	3,559161816	-1,811780055	5,370941871
211864_s_at	MYOF	2,99543179	-2,350444759	5,345876549
202436_s_at	CYP1B1	3,6787904	-1,665791776	5,344582176
220377_at	KIAA0125	3,660947664	-1,665791776	5,32673944
233500_x_at	CLEC2D	5,731510642	0,405752839	5,325757804
213306_at	MPDZ	3,171328694	-2,142286822	5,313615516
219631_at	LRP12	2,843031657	-2,466349414	5,309381071
225081_s_at	CDCA7L	3,418470674	-1,862269442	5,280740116
219983_at	HRASLS	3,218770562	-2,049747196	5,268517758
224994_at	CAMK2D	4,779371898	-0,484558493	5,263930392
219511_s_at	SNCAIP	4,233002276	-1,015398016	5,248400292
232151_at	MACC1	2,855122714	-2,391863529	5,246986243
213435_at	SATB2	3,628556015	-1,616924751	5,245480766
202435_s_at	CYP1B1	2,972517974	-2,27001641	5,242534384
208365_s_at	GRK4	3,813758784	-1,422257026	5,23601581
221165_s_at	IL22	5,130891823	-0,089635713	5,220527536
227623_at	CACNA2D1	2,575362572	-2,635549085	5,210911657
221802_s_at	KIAA1598	2,560606634	-2,635549085	5,196155719
230710_at	MIR210HG	3,331722254	-1,862269442	5,193991696
231335_at	MS4A6E	3,03433073	-2,142286822	5,176617551
228367_at	ALPK2	3,792209911	-1,372734086	5,164943997
220892_s_at	PSAT1	3,742498599	-1,422257026	5,164755625
216268_s_at	JAG1	5,917900402	0,757638486	5,160261915
1555141_a_at	SLC9B1	3,19484646	-1,956681069	5,151527529
238058_at	LOC150381	3,533714224	-1,616924751	5,150638976
205463_s_at	PDGFA	2,605420261	-2,536339282	5,141759542
1556182_x_at	ANKRD65	3,603313992	-1,520022123	5,123336116
204753_s_at	HLF	3,547953548	-1,568385657	5,116339205
224797_at	ARRDC3	4,418104951	-0,697182596	5,115287548
211026_s_at	MGLL	3,252865595	-1,862269442	5,115135037
1552578_a_at	MYO3B	2,560606634	-2,536339282	5,096945916
228555_at	CAMK2D	4,92247179	-0,172194225	5,094666015
225442_at	DDR2	3,671843723	-1,422257026	5,094100749
220253_s_at	LRP12	2,285848435	-2,8064286	5,092277035
1555122_at	GPR125	2,552836808	-2,536339282	5,08917609
238846_at	TNFRSF11A	4,826403949	-0,201789721	5,02819367
203397_s_at	GALNT3	3,096793374	-1,911206569	5,007999943
221796_at	NTRK2	3,533714224	-1,471833228	5,005547452
234317_s_at	STOX2	2,692288672	-2,312054429	5,004343101
222651_s_at	TRPS1	2,771643417	-2,229233437	5,000876855
203510_at	MET	5,39380421	0,405752839	4,988051371
223595_at	TMEM133	2,348178434	-2,635549085	4,983727519
219954_s_at	GBA3	3,012642182	-1,956681069	4,969323251
220358_at	BATF3	6,197469989	1,2298509	4,967619089
202364_at	MXI1	5,660441385	0,711934445	4,94850694

231250_at	HOXB-AS4	3,122148189	-1,811780055	4,933928244
209410_s_at	GRB10	4,377039749	-0,554696666	4,931736415
238804_at	-	3,685074281	-1,234835326	4,919909607
212070_at	GPR56	2,908690075	-2,005029581	4,913719656
205831_at	CD2	5,757392622	0,85655369	4,900838932
240058_at	-	2,582572508	-2,312054429	4,894626937
207720_at	LOR	2,621163856	-2,27001641	4,891180266
214452_at	BCAT1	2,444186596	-2,4284334	4,872619996
203037_s_at	MTSS1	6,067409644	1,199296812	4,868112832
232594_at	HSBP1L1	2,50022283	-2,350444759	4,85066759
203946_s_at	ARG2	4,515915598	-0,322340048	4,838255646
204796_at	EML1	2,33082239	-2,501359113	4,832181503
215241_at	ANO3	2,365121417	-2,466349414	4,831470831
203473_at	SLCO2B1	4,091994743	-0,734355396	4,826350139
240070_at	TIGIT	3,300874993	-1,520022123	4,820897116
238900_at	HLA-DRB1 /// HLA-	2,276736537	-2,536339282	4,813075818
240687_at	PASD1	3,340803206	-1,471833228	4,812636434
203661_s_at	TMOD1	4,448374788	-0,354781582	4,803156369
212730_at	SYNM	4,586396217	-0,201789721	4,788185938
218502_s_at	TRPS1	2,149438457	-2,635549085	4,784987542
212094_at	PEG10	2,908690075	-1,862269442	4,770959517
218503_at	FOCAD	2,365121417	-2,391863529	4,756984946
215723_s_at	PLD1	2,705643918	-2,049747196	4,755391114
204041_at	MAOB	3,822956033	-0,931539147	4,75449518
201564_s_at	FSCN1 /// LOC100(	4,330688003	-0,418248858	4,748936861
205803_s_at	TRPC1	2,436165655	-2,312054429	4,748220084
213992_at	COL4A6	3,081633533	-1,665791776	4,747425309
204777_s_at	MAL	5,427716821	0,681874787	4,745842034
222146_s_at	TCF4	2,878891456	-1,862269442	4,741160897
1556181_at	ANKRD65	3,621852128	-1,100943374	4,722795502
241118_at	LINC00462	2,745538988	-1,956681069	4,702220057
229972_at	LOC100505500	3,765107151	-0,931539147	4,696646298
236026_at	GPATCH2	3,214269085	-1,471833228	4,686102313
238183_at	-	2,671492308	-2,005029581	4,676521889
222716_s_at	SNX24	3,660947664	-1,015398016	4,67634568
205445_at	PRL	3,649735397	-1,015398016	4,665133413
236480_at	MIR210HG	2,127965541	-2,536339282	4,664304823
226973_at	VSTM2L	2,99543179	-1,665791776	4,661223566
212382_at	TCF4	3,03433073	-1,616924751	4,651255481
212999_x_at	HLA-DQB1 /// LOC	3,161901883	-1,471833228	4,633735111
1565809_x_at	-	3,509819135	-1,100943374	4,61076251
227955_s_at	EFNA5	2,890618076	-1,715576125	4,606194201
228547_at	NRXN1	2,732422196	-1,862269442	4,594691638
209012_at	TRIO	3,610731271	-0,972893339	4,58362461
204749_at	NAP1L3	2,914752575	-1,665791776	4,580544352
1553829_at	CYP1B1-AS1	2,096248267	-2,466349414	4,562597681
206022_at	NDP	2,41945542	-2,142286822	4,561742241
210869_s_at	MCAM	3,57383031	-0,972893339	4,546723648
204918_s_at	MLLT3	2,56794744	-1,956681069	4,524628509
201525_at	APOD	4,125707946	-0,386896102	4,512604048

226433_at	RNF157	4,183891989	-0,322340048	4,506232037
212989_at	SGMS1	4,359963018	-0,144765583	4,504728601
227561_at	DDR2	2,926639205	-1,568385657	4,495024862
39248_at	AQP3	3,610367622	-0,869117945	4,479485567
236180_at	-	3,414594082	-1,057301851	4,471895932
206772_at	PTH2R	3,322661262	-1,145057014	4,467718276
202672_s_at	ATF3	3,945594088	-0,518232988	4,463827077
202498_s_at	SLC2A3	4,975438323	0,515457602	4,459980721
201313_at	ENO2	4,36640141	-0,089635713	4,456037122
209098_s_at	JAG1	2,312738346	-2,142286822	4,455025168
212151_at	PBX1	3,642593015	-0,811769547	4,454362562
202022_at	ALDOC	5,766181318	1,315897482	4,450283836
202894_at	EPHB4	3,257413106	-1,19085694	4,448270046
223497_at	FAM135A	2,392346173	-2,049747196	4,442093369
219948_x_at	UGT2A3	1,936380191	-2,501359113	4,437739304
205945_at	IL6R	1,936380191	-2,501359113	4,437739304
232176_at	SLITRK6	2,914752575	-1,520022123	4,434774699
209409_at	GRB10	2,468978138	-1,956681069	4,425659208
235147_at	-	2,804738618	-1,616924751	4,421663369
231793_s_at	CAMK2D	3,6787904	-0,734355396	4,413145796
218888_s_at	NETO2	2,791258167	-1,616924751	4,408182918
236453_at	-	1,92317941	-2,466349414	4,389528824
226713_at	CCDC50	5,325305807	0,936232181	4,389073625
203896_s_at	PLCB4	2,159837409	-2,229233437	4,389070846
225102_at	MGLL	3,152091004	-1,234835326	4,38692633
1553633_s_at	SLC9B1	2,99543179	-1,372734086	4,368165875
1564479_a_at	-	1,857989965	-2,501359113	4,359349079
209031_at	CADM1	5,618002504	1,259081178	4,358921326
212385_at	TCF4	3,366614157	-0,972893339	4,339507496
209863_s_at	TP63	5,048031556	0,711934445	4,336097111
231042_s_at	-	3,446505209	-0,889222211	4,33572742
241074_at	-	3,402275492	-0,931539147	4,333814639
230249_at	KHDRBS3	2,190140919	-2,142286822	4,332427741
219368_at	NAP1L2	1,97249974	-2,350444759	4,322944499
210506_at	FUT7	2,365121417	-1,956681069	4,321802487
211945_s_at	ITGB1	7,167396728	2,848029002	4,319367726
228499_at	PFKFB4	3,466076444	-0,84928999	4,315366434
212148_at	PBX1	2,356619952	-1,956681069	4,313301021
222329_x_at	-	1,528073958	-2,780225148	4,308299106
204730_at	RIMS3	4,191105499	-0,116767475	4,307872974
223614_at	MMP16	2,031158928	-2,27001641	4,301175338
225019_at	CAMK2D	6,062793906	1,76396664	4,298827266
205888_s_at	JAKMIP2	2,38349585	-1,911206569	4,29470242
244485_at	HLA-DPB1	2,530390387	-1,763664074	4,294054461
206307_s_at	FOXD1	2,149438457	-2,142286822	4,291725279
1563933_a_at	PLD5	1,788922638	-2,501359113	4,290281751
229604_at	CMAHP	4,805309979	0,515457602	4,289852377
227856_at	C4orf32	6,446755911	2,167793428	4,278962483
201136_at	PLP2	5,990909768	1,715863686	4,275046082
200832_s_at	SCD	6,219321649	1,9592579	4,260063749

211282_x_at	TNFRSF25	2,642918178	-1,616924751	4,25984293
219984_s_at	HRASLS	2,107136847	-2,142286822	4,249423668
232481_s_at	SLITRK6	3,513985141	-0,734355396	4,248340537
210042_s_at	CTSZ	3,828857041	-0,418248858	4,247105899
209781_s_at	KHDRBS3	6,044722928	1,797682937	4,247039991
203895_at	PLCB4	2,41945542	-1,811780055	4,231235474
210652_s_at	TTC39A	2,41945542	-1,811780055	4,231235474
206482_at	PTK6	2,365121417	-1,862269442	4,227390859
205138_s_at	UST	1,995527416	-2,229233437	4,224760854
242002_at	NKAIN2	1,686984721	-2,536339282	4,223324002
235976_at	SLITRK6	1,857989965	-2,350444759	4,208434725
231984_at	MTAP	1,671397663	-2,536339282	4,207736944
207021_at	ZPBP	2,590269697	-1,616924751	4,207194448
225700_at	GLCCI1	2,590269697	-1,616924751	4,207194448
223360_at	C21orf56	2,484824532	-1,715576125	4,200400657
205139_s_at	UST	1,92317941	-2,27001641	4,19319582
244472_at	TRABD2B	2,179708195	-2,005029581	4,184737776
237301_at	-	3,291641956	-0,889222211	4,180864167
204642_at	S1PR1	3,122148189	-1,057301851	4,17945004
203662_s_at	TMOD1	4,601971043	0,424693306	4,177277738
226636_at	PLD1	3,584942056	-0,589001171	4,173943227
213117_at	KLHL9	1,671397663	-2,501359113	4,172756776
211470_s_at	SULT1C2	1,476175815	-2,695640852	4,171816667
212089_at	LMNA	3,152091004	-1,015398016	4,16748902
200795_at	SPARCL1	2,053550345	-2,09678811	4,150338455
242517_at	KISS1R	2,955426542	-1,19085694	4,146283481
207392_x_at	UGT2B15	3,045068376	-1,100943374	4,14601175
1555370_a_at	CAMTA1	2,621163856	-1,520022123	4,141185979
218705_s_at	SNX24	3,291641956	-0,84928999	4,140931946
1554748_at	CLCNKB	2,51558601	-1,616924751	4,132510761
213233_s_at	KLHL9	3,200057698	-0,931539147	4,131596844
1555420_a_at	KLF7	2,937809196	-1,19085694	4,128666136
213083_at	SLC35D2	3,071179046	-1,057301851	4,128480897
227599_at	MB21D2	4,05770789	-0,063377083	4,121084973
207139_at	ATP4A	2,304041045	-1,811780055	4,1158211
209914_s_at	NRXN1	1,760255626	-2,350444759	4,110700385
226908_at	LRIG3	1,246401134	-2,856570137	4,10297127
211654_x_at	HLA-DQB1	6,256083501	2,167793428	4,088290074
209829_at	FAM65B	3,185443259	-0,889222211	4,07466547
1553299_at	DUSP5P	2,649978302	-1,422257026	4,072235329
216323_x_at	TUBA3C /// TUBA3	5,613597248	1,547376668	4,066220581
219423_x_at	TNFRSF25	3,007119335	-1,057301851	4,064421186
204268_at	S100A2	3,007119335	-1,057301851	4,064421186
219338_s_at	LRRC49	4,45671768	0,405752839	4,050964842
225615_at	IFFO2	3,353217129	-0,697182596	4,050399725
229900_at	CD109	2,621163856	-1,422257026	4,043420882
226452_at	PDK1	4,531363785	0,497424714	4,033939071
205222_at	EHHADH	1,936380191	-2,09678811	4,033168301
225912_at	TP53INP1	4,09423887	0,061773582	4,032465287
215393_s_at	COBLL1	1,802733148	-2,229233437	4,031966585

226939_at	CPEB2	3,209439373	-0,811769547	4,02120892
209546_s_at	APOL1	4,374910757	0,366031214	4,008879544
222088_s_at	SLC2A14 /// SLC2A	5,511503557	1,508107415	4,003396141
223457_at	COPG2	3,442765386	-0,554696666	3,997462052
203778_at	MANBA	5,164559092	1,167919222	3,996639871
218424_s_at	STEAP3	2,75889404	-1,234835326	3,993729366
206404_at	FGF9	2,276736537	-1,715576125	3,992312662
1553530_a_at	ITGB1	5,137769258	1,146010634	3,991758624
204800_s_at	DHRS12	2,374261973	-1,616924751	3,991186725
238429_at	TMEM71	5,27108412	1,28853674	3,98254738
224480_s_at	AGPAT9	2,460890032	-1,520022123	3,980912155
216867_s_at	PDGFA	2,507817181	-1,471833228	3,979650409
1558212_at	FLJ35024	3,6787904	-0,290694975	3,969485375
239777_at	C14orf182	1,421391637	-2,536339282	3,957730919
1554628_at	ZNF57	1,528073958	-2,4284334	3,956507359
205208_at	ALDH1L1	2,932161212	-1,015398016	3,947559228
229598_at	COBLL1	1,307201325	-2,635549085	3,94275041
234473_at	-	1,246401134	-2,695640852	3,942041985
239914_at	-	1,671397663	-2,27001641	3,941414073
238520_at	TRERF1	2,878891456	-1,057301851	3,936193307
212445_s_at	NEDD4L	4,155757614	0,222432814	3,9333248
214186_s_at	HCG26	2,019346222	-1,911206569	3,930552791
227542_at	SOCS6	2,063971513	-1,862269442	3,926240955
1566766_a_at	MACC1	2,597647262	-1,32740143	3,925048692
236398_s_at	-	3,300874993	-0,623254098	3,924129091
219424_at	EBI3	2,732422196	-1,19085694	3,923279136
237833_s_at	SNCAIP	2,492739983	-1,422257026	3,914997009
228109_at	RASGRF2	3,023747815	-0,889222211	3,912970026
50221_at	TFEB	3,137060171	-0,772543284	3,909603455
213469_at	PGAP1	2,096248267	-1,811780055	3,908028322
229442_at	C18orf54	3,7799838	-0,116767475	3,896751275
206028_s_at	MERTK	2,705643918	-1,19085694	3,896500858
204755_x_at	HLF	3,007119335	-0,889222211	3,896341546
235155_at	BDH2	1,544940605	-2,350444759	3,895385364
208303_s_at	CRLF2	2,229377375	-1,665791776	3,895169151
228262_at	MAP7D2	1,88491011	-2,005029581	3,889939691
205158_at	RNASE4	1,701542258	-2,186086603	3,887628861
230272_at	LINC00461 /// MIR	3,559161816	-0,322340048	3,881501864
220118_at	ZBTB32	2,401508182	-1,471833228	3,873341409
229830_at	-	1,686984721	-2,186086603	3,873071324
209977_at	PLG	2,053550345	-1,811780055	3,8653304
243687_at	LOC100498859	2,804738618	-1,057301851	3,862040469
207039_at	CDKN2A	1,716003175	-2,142286822	3,858289996
230428_at	-	2,33082239	-1,520022123	3,850844513
228042_at	ADPRH	2,085817135	-1,763664074	3,849481209
227994_x_at	PPDPF	3,852052012	0,013190398	3,838861614
207840_at	CD160	1,830468611	-2,005029581	3,835498192
200878_at	EPAS1 /// LOC100(	6,275925903	2,440833194	3,83509271
204447_at	PROSAPIP1	2,312738346	-1,520022123	3,832760469
211071_s_at	MLLT11	5,523034165	1,694547353	3,828486812

213355_at	ST3GAL6	1,160996507	-2,665156778	3,826153285
200904_at	HLA-E	5,514518512	1,694547353	3,819971159
212599_at	AUTS2	2,096248267	-1,715576125	3,811824392
219687_at	HHAT	2,836219929	-0,972893339	3,809113268
234985_at	LDLRAD3	2,138605583	-1,665791776	3,80439736
212912_at	RPS6KA2	1,65554524	-2,142286822	3,797832061
229355_at	UBE2D3	4,827919576	1,032267596	3,795651979
209374_s_at	IGHM	2,321828846	-1,471833228	3,793662074
209087_x_at	MCAM	3,166767593	-0,623254098	3,790021692
206210_s_at	CETP	1,476175815	-2,312054429	3,788230244
212859_x_at	LOC100505584 ///	5,303412216	1,516067292	3,787344925
1557733_a_at	CHRM3-AS2	2,855122714	-0,931539147	3,786661861
204604_at	CDK14	4,504407021	0,727123109	3,777283912
226382_at	CAMK1D /// LOC2{	4,533526699	0,757638486	3,775888212
227868_at	LOC154761	3,931608669	0,155747281	3,775861389
232090_at	DNM3OS	1,20396005	-2,570522741	3,774482791
233233_at	RASSF3	1,816938997	-1,956681069	3,773620067
203673_at	TG	2,582572508	-1,19085694	3,773429448
226864_at	PKIA	3,858118781	0,085534992	3,772583789
209034_at	PNRC1	5,365205123	1,593605508	3,771599614
237515_at	TMEM56	2,199941239	-1,568385657	3,768326895
214624_at	UPK1A	2,248155141	-1,520022123	3,768177264
228159_at	-	3,649735397	-0,116767475	3,766502872
205691_at	SYNGR3	3,204847647	-0,554696666	3,759544313
213342_at	YAP1	2,476788561	-1,282520722	3,759309283
223362_s_at	37865	2,902957057	-0,84928999	3,752247047
226689_at	CISD2	6,516898371	2,767331883	3,749566488
240681_at	-	1,246401134	-2,501359113	3,747760247
202201_at	BLVRB	5,537973435	1,790914523	3,747058912
231899_at	ZC3H12C	1,510970097	-2,229233437	3,740203535
213397_x_at	RNASE4	1,730929079	-2,005029581	3,73595866
224218_s_at	TRPS1	1,307201325	-2,4284334	3,735634725
238778_at	MPP7	1,421391637	-2,312054429	3,733446066
225971_at	DDHD1	4,610466181	0,883374248	3,727091932
212998_x_at	HLA-DQB1 /// LOC	6,277990016	2,55501164	3,722978376
213713_s_at	GLB1L2	2,149438457	-1,568385657	3,717824114
244324_at	C18orf54	2,855122714	-0,84928999	3,704412704
1558827_a_at	ZNF831	2,552836808	-1,145057014	3,697893823
219213_at	JAM2	2,765070895	-0,931539147	3,696610042
213241_at	PLXNC1	4,875022718	1,189078962	3,685943755
225080_at	MYO1C	3,166767593	-0,518232988	3,685000581
231032_at	LOC286071	2,159837409	-1,520022123	3,679859532
210942_s_at	ST3GAL6	1,493647653	-2,186086603	3,679734256
204686_at	IRS1	1,760255626	-1,911206569	3,671462195
243582_at	SH3RF2	2,436165655	-1,234835326	3,671000981
212786_at	CLEC16A	1,440265662	-2,229233437	3,6694991
240162_at	-	2,295108393	-1,372734086	3,667842479
210102_at	VWA5A	2,476788561	-1,19085694	3,6676455
230489_at	CD5	4,435374233	0,772793615	3,662580618
229491_at	SLC9B2	4,650069766	0,996453274	3,653616492

1552789_at	SEC62	1,5944803	-2,049747196	3,644227496
219532_at	ELOVL4	2,074841895	-1,568385657	3,643227552
1553133_at	C9orf72	2,752615529	-0,889222211	3,641837739
203603_s_at	ZEB2	1,544940605	-2,09678811	3,641728715
209917_s_at	TP53TG1	3,466076444	-0,172194225	3,638270669
209846_s_at	BTN3A2	4,774699135	1,146010634	3,628688502
215536_at	HLA-DQB2	1,160996507	-2,466349414	3,627345921
209013_x_at	TRIO	1,716003175	-1,911206569	3,627209744
226122_at	PLEKHG1	1,910557155	-1,715576125	3,62613328
1552511_a_at	CPA6	1,267146969	-2,350444759	3,617591729
225604_s_at	GLIPR2 /// LOC100	2,804738618	-0,811769547	3,616508165
200699_at	KDELR2	3,525566827	-0,089635713	3,61520254
203035_s_at	PIAS3	2,285848435	-1,32740143	3,613249865
218211_s_at	MLPH	3,789279973	0,178191865	3,611088108
205079_s_at	MPDZ	2,138605583	-1,471833228	3,610438811
206486_at	LAG3	4,64066268	1,032267596	3,608395084
209644_x_at	CDKN2A	2,276736537	-1,32740143	3,604137967
206478_at	KIAA0125	2,365121417	-1,234835326	3,599956743
177_at	PLD1	2,648954853	-0,949550196	3,598505048
222239_s_at	INTS6	3,723597116	0,133104519	3,590492597
209197_at	SYT11	3,498020785	-0,089635713	3,587656497
214169_at	SUN1	1,871521385	-1,715576125	3,58709751
223660_at	ADORA3	1,578719607	-2,005029581	3,583749188
203354_s_at	PSD3	2,107136847	-1,471833228	3,578970074
204688_at	SGCE	4,12127125	0,550017041	3,57125421
225919_s_at	C9orf72	2,75889404	-0,811769547	3,570663586
221979_at	LOC100129250	4,590648292	1,032267596	3,558380696
212397_at	RDX	5,765450537	2,207440269	3,558010269
217894_at	KCTD3	3,001575397	-0,554696666	3,556272064
226810_at	OGFRL1	4,10471458	0,550017041	3,55469754
210514_x_at	HLA-G	5,608763656	2,055739171	3,553024485
228796_at	CPNE4	1,5944803	-1,956681069	3,551161369
225803_at	FBXO32	1,88491011	-1,665791776	3,550701886
204562_at	IRF4	6,153179494	2,613615641	3,539563853
202499_s_at	SLC2A3	6,020489043	2,482273566	3,538215478
238750_at	CCL28	2,908690075	-0,623254098	3,531944174
207555_s_at	TBXA2R	1,716003175	-1,811780055	3,527783229
212613_at	BTN3A2	3,603313992	0,085534992	3,517779
214724_at	DIXDC1	3,029186714	-0,484558493	3,513745208
235863_at	JSRP1	1,745502609	-1,763664074	3,509166683
239715_at	-	2,179708195	-1,32740143	3,507109625
1561961_at	PHF2P1	1,000459215	-2,501359113	3,501818328
236831_at	CCDC50	3,657457797	0,155747281	3,501710516
227970_at	GPR157	4,636598653	1,135235995	3,501362658
201137_s_at	HLA-DPB1	7,192038583	3,692642024	3,499396559
220254_at	LRP12	1,182925501	-2,312054429	3,49497993
214469_at	HIST1H2AB /// HIS	1,97249974	-1,520022123	3,492521863
202973_x_at	FAM13A	2,51558601	-0,972893339	3,488479348
219051_x_at	METRN	1,871521385	-1,616924751	3,488446136
1564233_at	FLJ33534	2,752615529	-0,734355396	3,486970925

235164_at	ZNF25	1,960819106	-1,520022123	3,48084123
1558105_a_at	SLC9A7	1,716003175	-1,763664074	3,479667249
210847_x_at	TNFRSF25	2,50022283	-0,972893339	3,473116169
224973_at	FAM46A	1,948628481	-1,520022123	3,468650604
230047_at	ARHGAP42	1,510970097	-1,956681069	3,467651167
201642_at	IFNGR2	4,57799322	1,112548615	3,465444604
213268_at	CAMTA1	1,458255986	-2,005029581	3,463285567
233675_s_at	TPTE2P6	0,735999505	-2,723114724	3,459114229
244600_at	-	4,087244545	0,634301128	3,452943417
225585_at	RAP2A	5,060079822	1,60918327	3,450896552
208949_s_at	LGALS3	7,222718116	3,774776136	3,44794198
242100_at	CHSY3	0,97663406	-2,466349414	3,442983474
214390_s_at	BCAT1	2,705643918	-0,734355396	3,439999314
214539_at	SERPINB10	1,476175815	-1,956681069	3,432856885
1565549_at	-	2,149438457	-1,282520722	3,431959179
201403_s_at	LOC100505828 ///	7,166916198	3,738875087	3,428041112
229603_at	BBS12	3,77412549	0,346964736	3,427160754
212096_s_at	MTUS1	2,613382867	-0,811769547	3,425152414
204334_at	KLF7	3,461795772	0,03784737	3,423948402
1553757_at	IQCG	1,510970097	-1,911206569	3,422176667
230925_at	APBB1IP	4,346015214	0,936232181	3,409783033
222505_at	LMBR1	3,147261986	-0,260526297	3,407788282
211913_s_at	MERTK	1,544940605	-1,862269442	3,407210047
224649_x_at	CCNY	1,88491011	-1,520022123	3,404932234
226666_at	DAAM1	4,865309532	1,46719264	3,398116893
235456_at	-	3,505636113	0,109458907	3,396177207
222107_x_at	LZTS1	1,345611839	-2,049747196	3,395359035
231690_at	-	1,000459215	-2,391863529	3,392322743
223342_at	RRM2B	3,657457797	0,265475485	3,391982312
220403_s_at	TP53AIP1	1,20396005	-2,186086603	3,390046653
232271_at	HNF4G	2,149438457	-1,234835326	3,384273783
207785_s_at	RBPJ	7,185004455	3,802659695	3,38234476
227526_at	CDON	2,56794744	-0,811769547	3,379716986
206255_at	BLK	4,193391347	0,815200271	3,378191077
239178_at	FGF9	0,677745787	-2,695640852	3,373386639
209239_at	NFKB1	6,277147611	2,906008589	3,371139022
205983_at	DPEP1	1,364555912	-2,005029581	3,369585493
226959_at	CAMK1D /// LOC2	3,501655014	0,133104519	3,368550494
238763_at	RBM20	1,225123479	-2,142286822	3,367410301
206518_s_at	RGS9	1,225123479	-2,142286822	3,367410301
202679_at	NPC1	4,563443322	1,199296812	3,36414651
210007_s_at	GPD2	1,094118704	-2,27001641	3,364135114
206399_x_at	CACNA1A	2,16969527	-1,19085694	3,36055221
222774_s_at	NETO2	2,468978138	-0,889222211	3,358200349
1568666_at	PLIN5	1,544940605	-1,811780055	3,35672066
209011_at	TRIO	2,38349585	-0,972893339	3,356389189
230840_at	SMIM1	1,640193838	-1,715576125	3,355769963
209105_at	NCOA1	3,816747242	0,461385738	3,355361504
237127_at	-	0,926325262	-2,4284334	3,354758662
203131_at	PDGFRA	1,000459215	-2,350444759	3,350903974

229127_at	JAM2	1,578719607	-1,763664074	3,342383681
219501_at	ENOX1	2,492739983	-0,84928999	3,342029973
208047_s_at	NAB1	0,764143511	-2,570522741	3,334666252
242293_at	ING3	3,370643409	0,03784737	3,332796039
216526_x_at	HLA-C	8,24932811	4,926000868	3,323327242
1558738_at	NOL3	1,510970097	-1,811780055	3,322750152
239069_s_at	-	4,364327647	1,043469112	3,320858534
214203_s_at	PRODH	1,898032047	-1,422257026	3,320289073
200667_at	UBE2D3	7,622417666	4,303088912	3,319328754
212739_s_at	NME4	2,38349585	-0,931539147	3,315034997
232088_x_at	LOC100271722	1,160996507	-2,142286822	3,303283329
206300_s_at	PTHLH	0,95222556	-2,350444759	3,30267032
204806_x_at	HLA-F	6,9137372	3,617435945	3,296301255
217142_at	-	2,321828846	-0,972893339	3,294722185
1569620_s_at	LOC100128079	1,716003175	-1,568385657	3,284388831
228831_s_at	GNG7	1,326463531	-1,956681069	3,2831446
230780_at	LOC730091	2,267141818	-1,015398016	3,282539835
227233_at	TSPAN2	2,179708195	-1,100943374	3,280651569
205997_at	ADAM28	2,507817181	-0,771340337	3,279157518
205500_at	C5	1,610353504	-1,665791776	3,27614528
222723_at	VWA1	1,267146969	-2,005029581	3,27217655
226556_at	MAP3K13	3,618399139	0,346964736	3,271434403
232035_at	HIST1H4A /// HIST	1,898032047	-1,372734086	3,270766133
217783_s_at	YPEL5	4,859875458	1,593605508	3,26626995
225974_at	TMEM64	4,554653912	1,28853674	3,266117172
212747_at	ANKS1A	5,35957938	2,093850742	3,265728638
211529_x_at	HLA-G	7,263500014	4,001778634	3,26172138
224314_s_at	EGLN1	2,843031657	-0,418248858	3,261280515
222859_s_at	DAPP1	4,07586392	0,815200271	3,260663649
203935_at	ACVR1	2,705643918	-0,554696666	3,260340584
211530_x_at	HLA-G	5,833556882	2,578283758	3,255273124
211557_x_at	SLCO2B1	1,97249974	-1,282520722	3,255020462
218938_at	FBXL15	1,024284941	-2,229233437	3,253518378
206385_s_at	ANK3	2,107136847	-1,145057014	3,252193861
238972_at	-	1,440265662	-1,811780055	3,252045717
209030_s_at	CADM1	5,581288285	2,332940758	3,248347527
1561402_at	LOC339894	2,692288672	-0,554696666	3,246985338
213572_s_at	SERPINB1	4,721089228	1,475630127	3,2454591
202731_at	MIR4680 /// PDCD	4,792135367	1,547376668	3,2447587
232269_x_at	METRN	2,053550345	-1,19085694	3,244407285
238482_at	KLF7	1,871521385	-1,372734086	3,244255471
229016_s_at	TRERF1	2,096248267	-1,145057014	3,241305282
212567_s_at	MAP4	3,300874993	0,061773582	3,239101411
211799_x_at	HLA-C	6,59896285	3,363771078	3,235191772
209822_s_at	VLDLR	2,943764123	-0,290694975	3,234459099
211340_s_at	MCAM	3,243218364	0,013190398	3,230027966
221428_s_at	TBL1XR1	3,828857041	0,600385088	3,228471952
230263_s_at	DOCK5	3,742498599	0,515457602	3,227040997
203665_at	HMOX1	2,031158928	-1,19085694	3,222015867
227020_at	YPEL2	3,257413106	0,03784737	3,219565736

221207_s_at	NBEA	1,307201325	-1,911206569	3,218407894
206781_at	DNAJC4	1,402495885	-1,811780055	3,214275939
212975_at	DENND3	3,595895594	0,385582632	3,210312962
218276_s_at	SAV1	2,949541692	-0,260526297	3,210067989
216873_s_at	ATP8B2	2,063971513	-1,145057014	3,209028527
202920_at	ANK2	2,545254728	-0,661178575	3,206433302
201151_s_at	MBNL1	5,764543524	2,558652146	3,205891378
238383_at	C6orf58	1,92317941	-1,282520722	3,205700132
205141_at	ANG	1,440265662	-1,763664074	3,203929736
216594_x_at	AKR1C1	2,229377375	-0,972893339	3,202270714
1569996_at	ANKRD26P3	2,053550345	-1,145057014	3,198607359
222408_s_at	YPEL5	4,496331177	1,298349073	3,197982104
201627_s_at	INSIG1	5,691817438	2,498884538	3,192932901
213135_at	TIAM1	5,034873434	1,842852096	3,192021338
240432_x_at	KLF7	2,492739983	-0,697182596	3,189922579
211841_s_at	TNFRSF25	2,25755351	-0,931539147	3,189092657
227088_at	PDE5A	1,139057614	-2,049747196	3,18880481
215436_at	HSDL2	1,716003175	-1,471833228	3,187836402
241390_at	-	0,648195588	-2,536339282	3,18453487
223233_s_at	CGN	2,732422196	-0,45121326	3,183635456
210875_s_at	ZEB1	1,948628481	-1,234835326	3,183463807
231416_at	DHDH	0,95222556	-2,229233437	3,181458998
230435_at	FAM228B	2,920736393	-0,260526297	3,18126269
205203_at	PLD1	3,190097062	0,013190398	3,176906664
213082_s_at	SLC35D2	1,364555912	-1,811780055	3,176335967
216589_at	-	1,458255986	-1,715576125	3,173832111
1552381_at	SRSF12	0,39105295	-2,780225148	3,171278098
209398_at	HIST1H1C	4,987088092	1,817403386	3,169684706
212914_at	CBX7	4,151573909	0,98381571	3,1677582
210387_at	HIST1H2BC /// HIS	1,160996507	-2,005029581	3,166026088
238469_at	OGFRL1	1,544940605	-1,616924751	3,161865356
211355_x_at	LEPR	0,58788177	-2,570522741	3,158404512
204567_s_at	ABCG1	1,92317941	-1,234835326	3,158014736
232629_at	PROK2	4,060218357	0,910483921	3,149734436
209839_at	DNM3	1,578719607	-1,568385657	3,147105263
215305_at	PDGFRA	1,047341799	-2,09678811	3,144129908
205352_at	SERPINI1	3,18076822	0,03784737	3,14292085
214770_at	MSR1	1,000459215	-2,142286822	3,142746036
203741_s_at	ADCY7	5,167863921	2,026957145	3,140906776
223588_at	THAP2	1,857989965	-1,282520722	3,140510688
218854_at	DSE	4,590648292	1,451507543	3,139140749
235725_at	SMAD4	2,685337919	-0,45121326	3,136551178
218202_x_at	MRPL44	2,285848435	-0,84928999	3,135138425
216835_s_at	DOK1	3,685074281	0,550017041	3,13505724
202910_s_at	CD97	5,04544017	1,911502437	3,133937733
204071_s_at	TOPORS	3,065977723	-0,063377083	3,129354805
1557098_s_at	HAR1A	3,533714224	0,405752839	3,127961386
228548_at	RAP1A	3,533714224	0,405752839	3,127961386
225065_x_at	C17orf76-AS1 /// S	6,487203205	3,363771078	3,123432126
219274_at	TSPAN12	0,425036312	-2,695640852	3,120677164

234088_at	-	2,229377375	-0,889222211	3,118599586
205207_at	IL6	1,745502609	-1,372734086	3,118236694
228734_at	-	1,402495885	-1,715576125	3,11807201
200669_s_at	UBE2D3	7,652353637	4,534332507	3,11802113
226214_at	GDE1	6,334339038	3,220021599	3,114317439
211682_x_at	UGT2B28	1,686984721	-1,422257026	3,109241747
202308_at	SREBF1	3,533714224	0,424693306	3,109020919
217728_at	S100A6	7,942442846	4,835344025	3,10709882
217047_s_at	FAM13A	1,775035811	-1,32740143	3,102437241
209198_s_at	SYT11	3,185443259	0,085534992	3,099908267
235299_at	SLC41A2	2,401508182	-0,697182596	3,098690778
209106_at	NCOA1	4,92247179	1,823960141	3,098511649
1552467_at	DSCR10	1,716003175	-1,372734086	3,08873726
219147_s_at	NMRK1	4,060218357	0,971607464	3,088610892
221478_at	BNIP3L	5,842208674	2,753767314	3,08844136
202880_s_at	CYTH1	3,969866565	0,883374248	3,086492316
229679_at	C12orf76	2,966903803	-0,116767475	3,083671278
225272_at	SAT2	2,348178434	-0,734355396	3,08253383
212074_at	SUN1	5,318066414	2,235857731	3,082208683
235283_at	INTS6	1,610353504	-1,471833228	3,082186731
203845_at	KAT2B	4,422748524	1,343200523	3,079548001
1558626_at	-	1,267146969	-1,811780055	3,078927024
210716_s_at	CLIP1	1,843966654	-1,234835326	3,07880198
213442_x_at	SPDEF	1,071336699	-2,005029581	3,07636628
211261_at	NUP214	2,304041045	-0,771340337	3,075381382
215071_s_at	HIST1H2AC	4,165622052	1,090640407	3,074981645
238516_at	BMPR2	0,97663406	-2,09678811	3,07342217
222896_at	TMEM38A	1,116462765	-1,956681069	3,073143834
205802_at	TRPC1	2,138605583	-0,931539147	3,07014473
206103_at	RAC3	2,295108393	-0,771340337	3,06644873
226074_at	PPM1M	2,860630824	-0,201789721	3,062420545
203120_at	TP53BP2	4,928564101	1,868130962	3,060433139
223232_s_at	CGN	2,127965541	-0,931539147	3,059504688
225537_at	TRAPPC6B	3,691400566	0,634301128	3,057099438
227442_at	COX18	5,123850804	2,071691486	3,052159319
211528_x_at	HLA-G	7,2810893	4,230731855	3,050357444
209289_at	NFIB	0,735999505	-2,312054429	3,048053934
223398_at	C9orf89	4,473867663	1,426280426	3,047587237
204141_at	TUBB2A	4,64066268	1,593605508	3,047057172
213434_at	STX2	4,37922506	1,333668715	3,045556345
203735_x_at	PPFIBP1	3,03433073	-0,011001236	3,045331966
235683_at	SESN3	1,182925501	-1,862269442	3,045194943
235061_at	PPM1K	4,303924985	1,259081178	3,044843807
235857_at	KCTD11	2,523100136	-0,518232988	3,041333125
202887_s_at	DDIT4	6,147928279	3,109293915	3,038634364
205198_s_at	ATP7A	1,936380191	-1,100943374	3,037323565
207346_at	STX2	2,019346222	-1,015398016	3,034744238
210205_at	B3GALT4	2,582572508	-0,45121326	3,033785768
1552296_at	BEST4	1,610353504	-1,422257026	3,03261053
225368_at	HIPK2	5,431138688	2,398910172	3,032228516

242224_at	GPATCH2	2,21987062	-0,811769547	3,031640167
50965_at	RAB26	1,070919826	-1,959469537	3,030389362
225481_at	FRMD6	1,364555912	-1,665791776	3,030347688
231183_s_at	JAG1	2,966903803	-0,063377083	3,030280886
228980_at	RAD51L3-RFFL ///	5,63842425	2,609615156	3,028809094
238209_at	-	1,116462765	-1,911206569	3,027669334
226521_s_at	FAM175A	3,470020347	0,443143223	3,026877124
227525_at	GLCCI1	5,188683224	2,162541631	3,026141593
236990_at	LOC100507360	1,92317941	-1,100943374	3,024122784
239719_at	CD109	0,926325262	-2,09678811	3,023113372
221609_s_at	WNT6	0,791908897	-2,229233437	3,021142335
229819_at	A1BG	3,147261986	0,133104519	3,014157466
243514_at	-	1,730929079	-1,282520722	3,013449801
235457_at	MAML2	3,581038515	0,568155442	3,012883072
227966_s_at	CCDC74A /// CCDC	4,16769828	1,156599976	3,011098303
219542_at	NEK11	1,995527416	-1,015398016	3,010925432
227179_at	STAU2	2,348178434	-0,661178575	3,009357009
244334_at	TRAM1L1	0,820160788	-2,186086603	3,006247391
204754_at	HLF	0,820160788	-2,186086603	3,006247391
216952_s_at	LMNB2	3,792209911	0,786391549	3,005818362
222861_x_at	FBXO44	1,094118704	-1,911206569	3,005325273
224831_at	CPEB4	2,031158928	-0,972893339	3,004052266
239288_at	TNIK	1,287793882	-1,715576125	3,003370007
235971_at	-	3,039689025	0,03784737	3,001841654
1558826_at	ZNF831	1,898032047	-1,100943374	2,998975421
227791_at	SLC9A9	3,305120269	0,306454867	2,998665402
218693_at	TSPAN15	1,625482993	-1,372734086	2,998217079
222026_at	RBM3	2,878891456	-0,116767475	2,99565893
218802_at	CCDC109B	6,078826967	3,086398747	2,99242822
238858_at	TIFA	3,029186714	0,03784737	2,991339344
213195_at	C17orf108	1,421391637	-1,568385657	2,989777294
212448_at	NEDD4L	2,365121417	-0,623254098	2,988375516
206729_at	TNFRSF8	4,873110637	1,887158627	2,98595201
227188_at	FAM176C	0,97663406	-2,005029581	2,981663641
209272_at	NAB1	3,599444854	0,61779983	2,981645023
233543_s_at	FAM175A	2,836219929	-0,144765583	2,980985512
208763_s_at	TSC22D3	6,014601324	3,035366122	2,979235202
219806_s_at	C11orf75	5,924341728	2,945366812	2,978974915
1559263_s_at	ZC3H12D	4,288236521	1,315897482	2,972339038
225842_at	PHLDA1	3,171328694	0,20062106	2,970707635
208089_s_at	TDRD3	3,336134047	0,366031214	2,970102834
209643_s_at	PLD2	1,910557155	-1,057301851	2,967859006
214293_at	40787	1,640193838	-1,32740143	2,967595268
217203_at	-	1,5944803	-1,372734086	2,967214386
204969_s_at	RDX	1,5944803	-1,372734086	2,967214386
235850_at	FAM162A	1,775035811	-1,19085694	2,965892751
217672_x_at	EIF1	2,410823487	-0,554696666	2,965520153
227867_at	TRABD2A	3,389999385	0,424693306	2,965306079
210075_at	02.03.15	2,545254728	-0,418248858	2,963503586
230439_at	LOC389458 /// RB/	0,820160788	-2,142286822	2,962447609

1554547_at	FAM13C	0,648195588	-2,312054429	2,960250017
235046_at	INPP4B	2,896717728	-0,063377083	2,960094811
204282_s_at	FARS2	4,716606434	1,757155322	2,959451113
223641_at	-	1,000459215	-1,956681069	2,957140284
221136_at	GDF2	1,094118704	-1,862269442	2,956388146
205226_at	PDGFRL	2,33082239	-0,623254098	2,954076488
216380_x_at	-	5,660441385	2,707013102	2,953428283
243718_at	-	1,578719607	-1,372734086	2,951453692
244422_at	-	1,578719607	-1,372734086	2,951453692
206560_s_at	MIA	1,760255626	-1,19085694	2,951112566
1556165_at	-	1,139057614	-1,811780055	2,950837669
213110_s_at	COL4A5	1,528073958	-1,422257026	2,950330985
234647_at	KCNIP3	1,528073958	-1,422257026	2,950330985
212868_x_at	MAPKAPK5-AS1	0,764143511	-2,186086603	2,950230114
1564467_at	FAM161A	0,900065578	-2,049747196	2,949812774
226117_at	TIFA	7,275338584	4,325963114	2,949375471
210355_at	PTHLH	0,556428218	-2,391863529	2,948291747
209311_at	BCL2L2	1,182925501	-1,763664074	2,946589575
1553743_at	LOC100506290 ///	3,122148189	0,178191865	2,943956324
209200_at	MEF2C	4,496331177	1,555165465	2,941165712
203386_at	TBC1D4	3,555373205	0,61779983	2,937573375
226559_at	IER5L	0,707657549	-2,229233437	2,936890986
1563719_a_at	-	1,510970097	-1,422257026	2,933227124
78383_at	LOC100129250	3,833407258	0,901386218	2,932021039
219799_s_at	DHRS9	0,926325262	-2,005029581	2,931354843
231873_at	BMPR2	2,575362572	-0,354781582	2,930144154
220937_s_at	ST6GALNAC4	2,575362572	-0,354781582	2,930144154
221582_at	HIST3H2A	5,292200339	2,36899666	2,923203678
205660_at	OASL	2,657308324	-0,260526297	2,917834621
200920_s_at	BTG1	5,467373717	2,55501164	2,912362077
214620_x_at	PAM	2,460890032	-0,45121326	2,912103292
1557321_a_at	CAPN14	0,244681185	-2,665156778	2,909837963
203706_s_at	FZD7	0,95222556	-1,956681069	2,90890663
217497_at	TYMP	0,677745787	-2,229233437	2,906979224
218804_at	ANO1	0,556428218	-2,350444759	2,906872977
221009_s_at	ANGPTL4	0,764143511	-2,142286822	2,906430333
215543_s_at	LARGE	1,094118704	-1,811780055	2,905898759
205841_at	JAK2	1,287793882	-1,616924751	2,904718633
210229_s_at	CSF2	2,732422196	-0,172194225	2,904616421
219665_at	NUDT18	2,75889404	-0,144765583	2,903659623
231390_at	-	0,20740896	-2,695640852	2,903049812
205277_at	PRDM2	4,025386557	1,123905343	2,901481215
221035_s_at	TEX14	1,843966654	-1,057301851	2,901268505
235626_at	CAMK1D	4,022766247	1,123905343	2,898860905
226576_at	ARHGAP26	1,92317941	-0,972893339	2,896072749
1560738_at	-	0,707657549	-2,186086603	2,893744152
238144_s_at	-	0,707657549	-2,186086603	2,893744152
227379_at	MBOAT1	4,424844786	1,531877749	2,892967037
219023_at	AP1AR	5,296107648	2,403191094	2,892916554
210797_s_at	OASL	2,537731447	-0,354781582	2,892513029

227368_at	-	3,876052392	0,98381571	2,892236682
227177_at	CORO2A	3,603313992	0,711934445	2,891379547
202800_at	SLC1A3	2,267141818	-0,623254098	2,890395917
204151_x_at	AKR1C1	3,045068376	0,155747281	2,889321096
234117_at	NPSR1-AS1	0,791908897	-2,09678811	2,888697007
221760_at	MAN1A1	5,569095514	2,682180306	2,886915208
221908_at	RNFT2	2,074841895	-0,811769547	2,886611442
216382_s_at	MLL2	1,024284941	-1,862269442	2,886554383
238265_x_at	-	0,926325262	-1,956681069	2,883006332
228773_at	LOC100506100	4,498569607	1,616354037	2,88221557
239846_at	LOC100506245	0,244681185	-2,635549085	2,88023027
220923_s_at	PNMA3	0,873789652	-2,005029581	2,878819233
213273_at	ODZ4	0,735999505	-2,142286822	2,878286327
238174_at	-	0,648195588	-2,229233437	2,877429025
1558123_at	FLJ35390 /// LOC1	1,402495885	-1,471833228	2,874329112
205683_x_at	TPSAB1	0,20740896	-2,665156778	2,872565738
221584_s_at	KCNMA1	4,786884621	1,917743773	2,869140848
243704_at	GPATCH2	0,556428218	-2,312054429	2,868482647
218280_x_at	HIST2H2AA3 /// HI	4,597104084	1,72935563	2,867748454
209295_at	TNFRSF10B	3,786189171	0,923203038	2,862986133
222376_at	-	4,030423661	1,167919222	2,86250444
202980_s_at	SIAH1	1,760255626	-1,100943374	2,861199
207176_s_at	CD80	1,094118704	-1,763664074	2,857782778
227539_at	GNA13	4,191105499	1,333668715	2,857436784
1554703_at	ARHGEF10	0,39105295	-2,466349414	2,857402364
211653_x_at	AKR1C2 /// LOC10	2,159837409	-0,697182596	2,857020005
236987_at	-	1,287793882	-1,568385657	2,856179539
229718_at	N4BP2L1	1,287793882	-1,568385657	2,856179539
220545_s_at	TSKS	0,319287178	-2,536339282	2,855626459
235522_at	CLEC2D	2,50022283	-0,354781582	2,855004412
1555399_a_at	DUSP16	1,139057614	-1,715576125	2,854633739
221875_x_at	HLA-F	6,957594739	4,103620228	2,853974511
229043_at	PAPD5	3,813758784	0,960042218	2,853716566
203030_s_at	PTPRN2	4,823280506	1,970106792	2,853173714
215054_at	EPOR	2,118167848	-0,734355396	2,852523244
203387_s_at	TBC1D4	3,8107521	0,960042218	2,850709881
208178_x_at	TRIO	1,476175815	-1,372734086	2,848909901
238604_at	-	4,632969749	1,784182261	2,848787488
204499_at	AGTPBP1	1,701542258	-1,145057014	2,846599272
230619_at	ARNT	3,269673646	0,424693306	2,84498034
1568817_at	-	0,791908897	-2,049747196	2,841656093
205454_at	HPCA	0,791908897	-2,049747196	2,841656093
238695_s_at	RAB39B	2,804738618	-0,036543687	2,841282305
223199_at	MKNK2	4,991134457	2,151985997	2,839148461
207156_at	HIST1H2AG /// HIS	3,185443259	0,346964736	2,838478524
221904_at	FAM131A	1,510970097	-1,32740143	2,838371527
218507_at	HILPDA	5,678854172	2,841176366	2,837677806
238029_s_at	SLC16A14	0,926325262	-1,911206569	2,837531832
228101_at	APBA1	0,926325262	-1,911206569	2,837531832
228726_at	SERPINB1	4,125707946	1,28853674	2,837171206

239860_at	LOC100130232	0,648195588	-2,186086603	2,834282191
214893_x_at	HCN2	1,116462765	-1,715576125	2,83203889
238513_at	PRRG4	2,476788561	-0,354781582	2,831570142
210260_s_at	TNFAIP8	6,350408867	3,523783625	2,826625242
211685_s_at	NCALD	4,466417603	1,644429915	2,821987688
201626_at	INSIG1	5,912431518	3,091548197	2,820883321
228837_at	TCF4	0,677745787	-2,142286822	2,820032609
222662_at	PPP1R3B	2,007220798	-0,811769547	2,818990345
219563_at	LINC00341	4,949910672	2,132145748	2,817764924
1552790_a_at	SEC62	3,559161816	0,742671819	2,816489997
228058_at	ZG16B	4,233002276	1,417157926	2,81584435
241370_at	LOC286052	2,118167848	-0,697182596	2,815350445
635_s_at	PPP2R5B	1,482792051	-1,332203171	2,814995222
240223_at	SLC2A9	0,95222556	-1,862269442	2,814495002
222448_s_at	CMPK1	5,890619329	3,0766152	2,814004129
235051_at	CCDC50	4,043821727	1,2298509	2,813970827
211660_at	POU2F2	1,440265662	-1,372734086	2,812999748
200698_at	KDELR2	4,237642543	1,426280426	2,811362117
212561_at	DENND5A	5,350868606	2,539809146	2,81105946
223633_s_at	-	1,047341799	-1,763664074	2,811005873
209290_s_at	NFIB	0,20740896	-2,603471267	2,810880227
225939_at	EIF4E3	1,528073958	-1,282520722	2,810594681
209360_s_at	LOC100506403 ///	3,768156719	0,960042218	2,808114501
205168_at	DDR2	1,139057614	-1,665791776	2,80484939
217291_at	CEACAM5	1,139057614	-1,665791776	2,80484939
235775_at	TMTC2	0,618277321	-2,186086603	2,804363924
216641_s_at	LAD1	0,618277321	-2,186086603	2,804363924
200704_at	LITAF	4,744801376	1,941115499	2,803685877
238461_at	EIF4E3	2,685337919	-0,116767475	2,802105393
218669_at	RAP2C	5,291057394	2,490640503	2,800416891
217202_s_at	GLUL	5,273387846	2,47417686	2,799210987
217887_s_at	EPS15	6,4475571	3,650351707	2,797205393
225259_at	RAB6B	0,791908897	-2,005029581	2,796938478
205215_at	RNF2	0,524545436	-2,27001641	2,794561846
210887_s_at	EVC	1,421391637	-1,372734086	2,794125723
227865_at	IDNK	3,473999051	0,681874787	2,792124264
227713_at	KATNAL1	3,012642182	0,222432814	2,790209368
244689_at	PPARA	0,926325262	-1,862269442	2,788594704
1564746_at	SLC9B2	2,943764123	0,155747281	2,788016843
1555168_a_at	CALN1	1,071336699	-1,715576125	2,786912824
217999_s_at	PHLDA1	0,319287178	-2,466349414	2,785636592
217058_at	GNAS	0,873789652	-1,911206569	2,784996221
227669_at	BRP44	2,798156197	0,013190398	2,784965799
221551_x_at	ST6GALNAC4	2,229377375	-0,554696666	2,784074041
227041_at	SESTD1	2,33082239	-0,45121326	2,782035649
207536_s_at	TNFRSF9	1,544940605	-1,234835326	2,779775931
227624_at	TET2	4,06801876	1,28853674	2,77948202
205486_at	TESK2	3,257413106	0,479089184	2,778323922
202096_s_at	TSPO	5,557142874	2,780838243	2,776304631
230171_at	-	2,41945542	-0,354781582	2,774237001

244841_at	SEC24A	1,88491011	-0,889222211	2,774132321
211194_s_at	TP63	2,107136847	-0,661178575	2,768315421
221586_s_at	E2F5	3,265594257	0,497424714	2,768169543
218731_s_at	VWA1	1,345611839	-1,422257026	2,767868865
236471_at	NFE2L3	2,989672611	0,222432814	2,767239797
207701_at	C22orf24	0,127732831	-2,635549085	2,763281915
236335_at	GUCY1A2	-0,043925712	-2,8064286	2,762502888
217778_at	SLC39A1	2,560606634	-0,201789721	2,762396355
225809_at	PARM1	0,900065578	-1,862269442	2,76233502
234858_at	-	0,900065578	-1,862269442	2,76233502
234491_s_at	SAV1	3,473999051	0,711934445	2,762064606
209255_at	KLHDC10	2,138605583	-0,623254098	2,761859682
233132_at	LOC96610	1,948628481	-0,811769547	2,760398027
219290_x_at	DAPP1	3,980708208	1,220470658	2,76023755
230203_at	FLJ46875	1,094118704	-1,665791776	2,75991048
213029_at	NFIB	0,847529938	-1,911206569	2,758736507
241801_at	PGAP1	0,847529938	-1,911206569	2,758736507
226390_at	STARD4	5,593744595	2,835336991	2,758407604
1562848_at	-	0,707657549	-2,049747196	2,757404745
219488_at	A4GALT	0,707657549	-2,049747196	2,757404745
204858_s_at	TYMP	1,983901861	-0,771340337	2,755242198
235520_at	ZNF280C	2,791258167	0,03784737	2,753410796
219048_at	PIGN	2,460890032	-0,290694975	2,751585007
227679_at	HDAC11	1,182925501	-1,568385657	2,751311158
215734_at	IZUMO4	2,427650693	-0,322340048	2,749990741
210886_x_at	TP53TG1	3,64600241	0,896834102	2,749168309
214064_at	TF	0,355751005	-2,391863529	2,747614534
224957_at	C18orf32 /// RPL1[illegible]	4,797158735	2,050286491	2,746872244
223159_s_at	NEK6	3,442765386	0,697221375	2,745544011
218486_at	KLF11	3,8107521	1,067365565	2,743386535
210926_at	POTEKP	2,452674045	-0,290694975	2,74336902
202777_at	SHOC2	4,514001815	1,771157334	2,742844481
218995_s_at	EDN1	0,556428218	-2,186086603	2,742514821
200706_s_at	LITAF	4,422748524	1,680963921	2,741784603
1554179_s_at	LYNX1	0,39105295	-2,350444759	2,74149771
200921_s_at	BTG1	6,293538771	3,552051596	2,741487175
224983_at	SCARB2	3,960533133	1,220470658	2,740062475
205248_at	DOPEY2	1,024284941	-1,715576125	2,739861066
209770_at	BTN3A1	4,027733188	1,28853674	2,739196448
208180_s_at	HIST1H4A /// HIST	1,364555912	-1,372734086	2,737289998
213327_s_at	USP12	4,64066268	1,905294823	2,735367857
218352_at	RCBTB1	3,19484646	0,461385738	2,733460722
215001_s_at	GLUL	7,600768671	4,868242744	2,732525927
212923_s_at	PXDC1	1,716003175	-1,015398016	2,731401191
222769_at	MTHFSD	0,820160788	-1,911206569	2,731367357
224937_at	PTGFRN	0,820160788	-1,911206569	2,731367357
202497_x_at	SLC2A3	4,68949381	1,9592579	2,730235911
216611_s_at	SLC6A2	0,458905032	-2,27001641	2,728921442
1555306_a_at	ECE2	0,458905032	-2,27001641	2,728921442
238739_at	IPMK	1,493647653	-1,234835326	2,728482979

223299_at	SEC11C	6,468340697	3,742201737	2,72613896
223674_s_at	CDC42SE1	2,063971513	-0,661178575	2,725150088
235081_x_at	TRIM65	2,902957057	0,178191865	2,724765192
241808_at	ZC2HC1A	3,273854609	0,550017041	2,723837569
205891_at	ADORA2B /// LOC1	3,822956033	1,101780212	2,721175822
208209_s_at	C4BPB	1,983901861	-0,734355396	2,718257257
208000_at	GML	1,246401134	-1,471833228	2,718234361
218528_s_at	RNF38	3,701002444	0,98381571	2,717186735
201929_s_at	PKP4	3,517785666	0,80114751	2,716638157
204199_at	RALGPS1	2,019346222	-0,697182596	2,716528818
230747_s_at	TTC39C	3,265594257	0,550017041	2,715577216
210241_s_at	TP53TG1	3,018305638	0,306454867	2,711850771
219256_s_at	SH3TC1	1,476175815	-1,234835326	2,711011141
235301_at	KIAA1324L	1,476175815	-1,234835326	2,711011141
214786_at	MAP3K1	1,383446753	-1,32740143	2,710848183
233946_at	SMU1	1,287793882	-1,422257026	2,710050908
225464_at	FRMD6	1,287793882	-1,422257026	2,710050908
221663_x_at	HRH3	1,775035811	-0,931539147	2,706574958
225116_at	HIPK2	3,505636113	0,80114751	2,704488603
228561_at	CDC37L1	2,007220798	-0,697182596	2,704403394
214036_at	EFNA5	2,348178434	-0,354781582	2,702960016
229837_s_at	KCTD5	0,556428218	-2,142286822	2,69871504
232230_at	LINC00263	0,648195588	-2,049747196	2,697942784
222665_at	FAM82B	2,75889404	0,061773582	2,697120458
209166_s_at	MAN2B1	2,896717728	0,20062106	2,696096668
230706_s_at	CAMK2N2	1,995527416	-0,697182596	2,692710013
211113_s_at	ABCG1	0,97663406	-1,715576125	2,692210185
204096_s_at	ELL	0,97663406	-1,715576125	2,692210185
209912_s_at	AP5Z1 /// MIR465(	1,364555912	-1,32740143	2,691957342
203672_x_at	TPMT	3,588695979	0,896834102	2,691861877
239586_at	FAM83A	1,544940605	-1,145057014	2,689997619
206374_at	DUSP8	1,071336699	-1,616924751	2,68826145
1557411_s_at	SLC25A43	3,353217129	0,666486137	2,686730992
228509_at	SPHKAP	0,873789652	-1,811780055	2,685569707
210933_s_at	FSCN1 /// LOC100(	1,402495885	-1,282520722	2,685016607
225602_at	GLIPR2 /// LOC10C	2,817838865	0,133104519	2,684734345
203604_at	ZNF516	0,58788177	-2,09678811	2,68466988
221566_s_at	NOL3	1,948628481	-0,734355396	2,682983877
205048_s_at	PSPH	1,948628481	-0,734355396	2,682983877
221102_s_at	TRPM6	0,820160788	-1,862269442	2,682430229
204343_at	ABCA3	2,41945542	-0,260526297	2,679981716
207839_s_at	TMEM8B	2,159837409	-0,518232988	2,678070397
218411_s_at	MBIP	2,920736393	0,244646697	2,676089696
204135_at	FILIP1L	4,102051194	1,426280426	2,675770767
222506_at	LMBR1	1,440265662	-1,234835326	2,675100988
220974_x_at	SFXN3	1,440265662	-1,234835326	2,675100988
212827_at	IGHM	2,085817135	-0,589001171	2,674818306
234293_x_at	-	0,282358733	-2,391863529	2,674222262
224964_s_at	GNG2	6,259605337	3,586015261	2,673590076
227730_at	-	1,345611839	-1,32740143	2,673013269

232017_at	TJP2	1,936380191	-0,734355396	2,670735587
219134_at	ELTD1	0,319287178	-2,350444759	2,669731937
201860_s_at	PLAT	0,167727503	-2,501359113	2,669086617
225246_at	STIM2	3,792209911	1,123905343	2,668304569
215271_at	TNN	0,618277321	-2,049747196	2,668024517
243883_at	MMP15	0,618277321	-2,049747196	2,668024517
242418_at	C2orf27A	0,355751005	-2,312054429	2,667805434
235631_at	DDR2	0,355751005	-2,312054429	2,667805434
225277_at	SLC39A13	2,312738346	-0,354781582	2,667519928
212792_at	DPY19L1	3,577387089	0,910483921	2,666903169
228564_at	LOC375295	1,000459215	-1,665791776	2,666250991
235369_at	C14orf28	2,042564225	-0,623254098	2,665818323
211756_at	PTHLH	1,730929079	-0,931539147	2,662468226
209107_x_at	NCOA1	4,898999808	2,24081821	2,658181598
225927_at	MAP3K1	5,453781975	2,796775008	2,657006967
228372_at	C10orf128	3,915780568	1,259081178	2,65669939
202847_at	PCK2	3,485638812	0,829309537	2,656329275
224722_at	MIB1	1,421391637	-1,234835326	2,656226963
224832_at	DUSP16	1,421391637	-1,234835326	2,656226963
237855_at	ZNF777	0,425036312	-2,229233437	2,65426975
221443_x_at	PRLH	0,791908897	-1,862269442	2,654178339
234884_x_at	CKAP2 /// IGLC1	0,648195588	-2,005029581	2,653225169
234380_x_at	-	0,556428218	-2,09678811	2,653216328
239067_s_at	PANX2	1,225123479	-1,422257026	2,647380505
201818_at	LPCAT1	4,341822193	1,694547353	2,647274839
215779_s_at	HIST1H2BC /// HIS	0,735999505	-1,911206569	2,647206074
213106_at	ATP8A1	5,17773944	2,531843788	2,645895653
1558699_a_at	HERPUD2	2,25755351	-0,386896102	2,644449612
218285_s_at	BDH2	5,838335106	3,195836661	2,642498445
205414_s_at	ARHGAP44	0,97663406	-1,665791776	2,642425836
225175_s_at	SLC44A2	1,024284941	-1,616924751	2,641209692
230576_at	BLOC1S3	1,024284941	-1,616924751	2,641209692
207634_at	PDCD1	1,024284941	-1,616924751	2,641209692
32541_at	PPP3CC	3,791256606	1,150127122	2,641129484
219562_at	RAB26	1,493647653	-1,145057014	2,638704667
225001_at	RAB3D	1,788922638	-0,84928999	2,638212628
201262_s_at	BGN	0,58788177	-2,049747196	2,637628966
223798_at	SLC41A2	1,402495885	-1,234835326	2,637331211
221510_s_at	GLS	5,35957938	2,723341187	2,636238193
226585_at	NEIL2	1,578719607	-1,057301851	2,636021457
38241_at	BTN3A3	3,402713533	0,768559813	2,63415372
204072_s_at	FRY	1,936380191	-0,697182596	2,633562788
238683_at	ZNF524	1,160996507	-1,471833228	2,632829735
230948_at	-	0,820160788	-1,811780055	2,631940843
204085_s_at	CLN5	2,56794744	-0,063377083	2,631324522
225579_at	PQLC3	5,649466975	3,019022994	2,630443981
204546_at	KIAA0513	1,345611839	-1,282520722	2,628132561
221658_s_at	IL21R	3,353217129	0,727123109	2,62609402
216383_at	-	4,226167036	1,601076277	2,625090759
208703_s_at	APLP2	2,613382867	-0,011001236	2,624384103

219114_at	C3orf18	1,775035811	-0,84928999	2,624325801
243631_at	-	0,618277321	-2,005029581	2,623306902
224893_at	ATL3	4,616350364	1,993309863	2,623040502
226614_s_at	FAM167A	2,949541692	0,326777209	2,622764483
207543_s_at	P4HA1	5,744560023	3,124666253	2,619893771
216321_s_at	NR3C1	5,601579484	2,982339418	2,619240066
207582_at	PIN1P1	1,246401134	-1,372734086	2,619135219
204906_at	RPS6KA2	1,246401134	-1,372734086	2,619135219
239933_x_at	C14orf45	1,000459215	-1,616924751	2,617383966
206082_at	HCP5	3,694479488	1,078619386	2,615860102
1569369_at	ZFYVE28	1,287793882	-1,32740143	2,615195312
243539_at	KIAA1841	3,327128828	0,711934445	2,615194383
1553286_at	ZNF555	1,094118704	-1,520022123	2,614140827
220371_s_at	SLC12A9	2,321828846	-0,290694975	2,612523821
219326_s_at	B3GNT2	3,398333742	0,786391549	2,611942193
214391_x_at	PTGER1	1,510970097	-1,100943374	2,611913471
1558890_at	LOC100507054	0,847529938	-1,763664074	2,611194012
224828_at	CPEB4	1,139057614	-1,471833228	2,610890842
226461_at	HOXB9	2,855122714	0,244646697	2,610476016
241363_at	EXD3	1,326463531	-1,282520722	2,608984253
221543_s_at	ERLIN2	3,858118781	1,249194639	2,608924142
1558971_at	THEMIS	4,267962641	1,659532492	2,608430149
236451_at	-	-0,088974936	-2,695640852	2,606665916
239280_at	-	1,182925501	-1,422257026	2,605182527
1569303_s_at	RGS20	1,182925501	-1,422257026	2,605182527
214523_at	CEBPE	0,648195588	-1,956681069	2,604876658
237460_x_at	C14orf182	1,458255986	-1,145057014	2,603313001
229014_at	FLJ42709	1,671397663	-0,931539147	2,60293681
1560007_at	LOC645984	0,458905032	-2,142286822	2,601191853
231201_at	PTGER1	0,458905032	-2,142286822	2,601191853
232101_s_at	PIGN	2,042564225	-0,554696666	2,597260891
225785_at	REEP3	4,713199262	2,116147513	2,597051749
220562_at	CYP2W1	0,282358733	-2,312054429	2,594413162
217462_at	C11orf9	0,282358733	-2,312054429	2,594413162
227115_at	LOC100506870	0,127732831	-2,466349414	2,594082245
241227_at	-	0,127732831	-2,466349414	2,594082245
225941_at	EIF4E3	3,909506676	1,315897482	2,593609193
211332_x_at	HFE	0,97663406	-1,616924751	2,593558811
240376_s_at	-	0,58788177	-2,005029581	2,592911351
209086_x_at	MCAM	2,605420261	0,013190398	2,592229863
231944_at	ERO1LB	3,273854609	0,681874787	2,591979822
243046_at	-	2,552836808	-0,036543687	2,589380495
205806_at	ROM1	0,873789652	-1,715576125	2,589365777
236598_at	-	0,677745787	-1,911206569	2,588952356
232341_x_at	HABP4	3,269673646	0,681874787	2,587798859
218975_at	COL5A3	0,355751005	-2,229233437	2,584984442
204509_at	CA12	0,355751005	-2,229233437	2,584984442
209785_s_at	PLA2G4C	0,820160788	-1,763664074	2,583824862
1561347_a_at	-	1,345611839	-1,234835326	2,580447165
205195_at	AP1S1	3,261582786	0,681874787	2,579708

210113_s_at	NLRP1	3,161901883	0,583607503	2,57829438
202351_at	ITGAV	3,632502157	1,055317091	2,577185065
232792_at	TRIM69	1,20396005	-1,372734086	2,576694136
203910_at	ARHGAP29	4,06801876	1,491644074	2,576374687
217824_at	UBE2J1	4,31266827	1,736450458	2,576217812
202365_at	UNC119B	5,036276071	2,460974054	2,575302017
214864_s_at	GRHPR	5,580308277	3,005076416	2,575231862
224925_at	PREX1	4,204964809	1,630734622	2,574230188
222067_x_at	HIST1H2BD	4,478885385	1,905294823	2,573590562
209199_s_at	MEF2C	5,142163546	2,570802332	2,571361215
239479_x_at	-	0,707657549	-1,862269442	2,56992699
204621_s_at	NR4A2	0,95222556	-1,616924751	2,569150311
227989_at	LTBP4	0,95222556	-1,616924751	2,569150311
207855_s_at	CLCC1	2,745538988	0,178191865	2,567347122
208523_x_at	HIST1H2BC /// HIS	2,365121417	-0,201789721	2,566911138
242303_at	NRG2	1,094118704	-1,471833228	2,565951932
225144_at	BMPR2	3,434684504	0,869824768	2,564859736
229756_at	-	1,139057614	-1,422257026	2,56131464
208242_at	RAX	1,139057614	-1,422257026	2,56131464
1554516_at	LINC00537	1,326463531	-1,234835326	2,561298857
203508_at	TNFRSF1B	3,716897585	1,156599976	2,560297608
217886_at	EPS15	4,741350888	2,182711118	2,55863977
1567256_at	OR1J2	0,20740896	-2,350444759	2,557853719
239446_x_at	DCBLD2	0,244681185	-2,312054429	2,556735614
227217_at	WNK2	0,791908897	-1,763664074	2,555572971
200648_s_at	GLUL	6,016484326	3,463284238	2,553200088
220812_s_at	HHLA2	0,282358733	-2,27001641	2,552375143
227049_at	ZADH2	3,761702872	1,209973561	2,551729311
206879_s_at	NRG2	1,493647653	-1,057301851	2,550949504
209815_at	PTCH1	1,701542258	-0,84928999	2,550832248
41397_at	ZNF821	0,80886896	-1,73941975	2,54828871
238045_at	TMEM65	0,735999505	-1,811780055	2,54777956
216060_s_at	DAAM1	4,506449869	1,9592579	2,547191969
228693_at	CCDC50	4,022766247	1,475630127	2,54713612
219742_at	PRR7	2,159837409	-0,386896102	2,546733511
218935_at	EHD3	1,92317941	-0,623254098	2,546433509
217678_at	SLC7A11	2,285848435	-0,260526297	2,546374732
229974_at	EVC2	1,88491011	-0,661178575	2,546088685
229438_at	-	0,58788177	-1,956681069	2,54456284
203979_at	CYP27A1	0,58788177	-1,956681069	2,54456284
207046_at	HIST1H4A /// HIST	1,024284941	-1,520022123	2,544307064
218773_s_at	MSRB2	4,996753949	2,45323331	2,543520639
241038_at	-	0,926325262	-1,616924751	2,543250013
205663_at	PCBP3	1,071336699	-1,471833228	2,543169926
217190_x_at	ESR1	0,355751005	-2,186086603	2,541837608
210888_s_at	ITIH1	0,355751005	-2,186086603	2,541837608
223981_at	NIN	0,355751005	-2,186086603	2,541837608
222898_s_at	DLL3	0,491906512	-2,049747196	2,541653708
220662_s_at	HEYL	0,677745787	-1,862269442	2,540015229
217127_at	CTH	4,882191987	2,34250725	2,539684736

229156_s_at	PRKAG2-AS1	2,671492308	0,133104519	2,538387789
200668_s_at	UBE2D3	8,025436985	5,488318513	2,537118472
226656_at	CRTAP	2,392346173	-0,144765583	2,537111756
228426_at	CLEC2D	1,160996507	-1,372734086	2,533730593
202730_s_at	MIR4680 /// PDCD	4,548572168	2,016139502	2,532432666
203169_at	RGP1	2,042564225	-0,484558493	2,527122718
208296_x_at	TNFAIP8	6,115910987	3,589866014	2,526044973
208127_s_at	SOCS5	2,436165655	-0,089635713	2,525801368
211911_x_at	HLA-B	7,822634058	5,297134253	2,525499805
209818_s_at	HABP4	2,107136847	-0,418248858	2,525385705
218407_x_at	NENF	5,560054503	3,035366122	2,524688381
235165_at	PARD6B	1,287793882	-1,234835326	2,522629208
219708_at	NT5M	1,287793882	-1,234835326	2,522629208
239959_x_at	-	1,421391637	-1,100943374	2,522335011
223875_s_at	EPC1	1,671397663	-0,84928999	2,520687653
1559272_at	EXOC3L1	0,707657549	-1,811780055	2,519437603
237189_at	-	1,047341799	-1,471833228	2,519175026
217456_x_at	HLA-E	6,680344236	4,162193397	2,518150838
226686_at	CISD2	5,218027369	2,700070732	2,517956637
202662_s_at	ITPR2	0,900065578	-1,616924751	2,516990329
217545_at	MYH14	1,094118704	-1,422257026	2,51637573
204283_at	FARS2	5,711540841	3,195836661	2,515704179
222919_at	TRDN	0,08619576	-2,4284334	2,51462916
223046_at	EGLN1	5,008796129	2,49476365	2,51403248
216065_at	-	0,556428218	-1,956681069	2,513109287
1558289_at	RFT1	0,556428218	-1,956681069	2,513109287
203950_s_at	CLCN6	2,597647262	0,085534992	2,51211227
212366_at	ZNF292	3,327128828	0,815200271	2,511928557
220313_at	GPR88	0,045517965	-2,466349414	2,511867379
224552_s_at	C11orf20 /// KCNK	0,282358733	-2,229233437	2,51159217
220778_x_at	SEMA6B	1,182925501	-1,32740143	2,510326931
226782_at	SLC25A30	3,127551022	0,61779983	2,509751192
1558147_a_at	FLJ90757	1,775035811	-0,734355396	2,509391207
1568853_at	-	2,248155141	-0,260526297	2,508681438
204637_at	CGA	0,791908897	-1,715576125	2,507485022
232616_at	LOC100129935	0,791908897	-1,715576125	2,507485022
224662_at	KIF5B	5,143322491	2,635864822	2,507457669
208376_at	CCR4	2,56794744	0,061773582	2,506173858
234344_at	LOC100288675	2,118167848	-0,386896102	2,50506395
213474_at	KCTD7 /// RABGEF	2,492739983	-0,011001236	2,503741219
201464_x_at	JUN	4,346015214	1,842852096	2,503163118
237559_at	GPR55	2,356619952	-0,144765583	2,501385535
210275_s_at	ZFAND5	5,892935274	3,392128215	2,500807059
229324_x_at	ISYNA1	0,58788177	-1,911206569	2,49908834
214412_at	H2AFB1 /// H2AFB	0,58788177	-1,911206569	2,49908834
207000_s_at	PPP3CC	3,786189171	1,28853674	2,497652431
230920_at	TRABD2B	0,491906512	-2,005029581	2,496936093
229771_at	MAP3K13	0,491906512	-2,005029581	2,496936093
222091_at	HPCAL4	0,491906512	-2,005029581	2,496936093
236318_x_at	FBLL1	0,491906512	-2,005029581	2,496936093

233571_x_at	PPDPF	3,895112363	1,399283506	2,495828857
215181_at	CDH22	0,926325262	-1,568385657	2,494710919
215726_s_at	CYB5A	5,628888391	3,134198147	2,494690244
213279_at	DHRS1	2,937809196	0,443143223	2,494665973
218221_at	ARNT	2,739067176	0,244646697	2,494420478
217368_at	-	2,042564225	-0,45121326	2,493777484
223920_s_at	TP53AIP1	1,071336699	-1,422257026	2,493593725
240272_at	-	1,071336699	-1,422257026	2,493593725
235980_at	PIK3CA	1,071336699	-1,422257026	2,493593725
219693_at	AGPAT4	1,561816652	-0,931539147	2,493355799
201402_at	ADRBK1	2,074841895	-0,418248858	2,493090753
202236_s_at	SLC16A1	5,386609612	2,894109605	2,492500007
231200_at	LSM14B	1,476175815	-1,015398016	2,491573831
240210_at	ATAD3C	0,873789652	-1,616924751	2,490714403
218629_at	SMO	0,873789652	-1,616924751	2,490714403
230731_x_at	ZDHHC8	0,873789652	-1,616924751	2,490714403
212904_at	LRRC47	6,686106961	4,196990526	2,489116435
224391_s_at	SIAE	2,25755351	-0,231045907	2,488599417
227248_at	PLEKHH3	1,20396005	-1,282520722	2,486480772
1560788_at	MYO3B	0,820160788	-1,665791776	2,485952564
227677_at	JAK3	4,364327647	1,881145546	2,4831821
231949_at	-	1,857989965	-0,623254098	2,481244064
206020_at	SOCS6	0,524545436	-1,956681069	2,481226506
214517_at	KRTAP5-9	0,524545436	-1,956681069	2,481226506
211133_x_at	LILRA6 /// LILRB3	1,745502609	-0,734355396	2,479858005
1562681_at	LOC338651	0,167727503	-2,312054429	2,479781932
202997_s_at	LOXL2	0,167727503	-2,312054429	2,479781932
239137_x_at	C6orf223	1,960819106	-0,518232988	2,479052095
235252_at	KSR1	4,049824219	1,571112806	2,478711414
215333_x_at	GSTM1	1,421391637	-1,057301851	2,478693488
210001_s_at	SOCS1	0,127732831	-2,350444759	2,47817759
231133_at	CCDC164	0,08619576	-2,391863529	2,478059289
236362_at	-	0,20740896	-2,27001641	2,47742537
224576_at	ERGIC1	4,218749628	1,743236669	2,475512958
206641_at	TNFRSF17	2,410823487	-0,063377083	2,474200569
205199_at	CA9	0,244681185	-2,229233437	2,473914623
227691_at	AKAP8L	1,000459215	-1,471833228	2,472292442
219125_s_at	SLC50A1	3,889146356	1,417157926	2,471988429
1557921_s_at	LOC100506498	-0,280273599	-2,752097204	2,471823605
204454_at	LDOC1	2,053550345	-0,418248858	2,471799203
220544_at	TSKS	0,707657549	-1,763664074	2,471321623
244285_at	-	0,707657549	-1,763664074	2,471321623
239725_at	PGAP1	0,707657549	-1,763664074	2,471321623
243595_at	SHB	0,282358733	-2,186086603	2,468445336
230677_at	EXOC3L4	0,282358733	-2,186086603	2,468445336
242071_x_at	ITGA8	0,282358733	-2,186086603	2,468445336
227192_at	PRRT2	0,556428218	-1,911206569	2,467634787
211502_s_at	CDK14	1,948628481	-0,518232988	2,466861469
214606_at	TSPAN2	1,493647653	-0,972893339	2,466540992
239426_at	SLC2A8	1,730929079	-0,734355396	2,465284475

202100_at	RALB	5,261388038	2,796775008	2,464613029
220549_at	FSBP /// RAD54B	1,802733148	-0,661178575	2,463911722
212566_at	MAP4	4,900499467	2,436714502	2,463784965
214028_x_at	TDRD3	2,705643918	0,244646697	2,46099722
223285_s_at	ST6GALNAC4	2,138605583	-0,322340048	2,460945631
219005_at	TMEM59L	1,402495885	-1,057301851	2,459797735
214027_x_at	DES /// FAM48A	1,402495885	-1,057301851	2,459797735
226038_at	LONRF1	4,49239618	2,03313004	2,45926614
209767_s_at	GP1BB /// SEPT5 /,	0,791908897	-1,665791776	2,457700673
225774_at	RSPRY1	2,843031657	0,385582632	2,457449026
207545_s_at	NUMB	1,97249974	-0,484558493	2,457058233
227837_at	LOC729570	2,339581693	-0,116767475	2,456349168
221214_s_at	NELF	2,41945542	-0,036543687	2,455999107
227029_at	FAM177A1	2,51558601	0,061773582	2,453812428
209833_at	CRADD	3,309267982	0,85655369	2,452714292
221920_s_at	SLC25A37	0,735999505	-1,715576125	2,45157563
218180_s_at	EPS8L2	2,190140919	-0,260526297	2,450667216
209691_s_at	DOK4	2,62869474	0,178191865	2,450502875
239669_at	-	0,58788177	-1,862269442	2,450151212
221866_at	TFEB	2,248155141	-0,201789721	2,449944862
221083_at	KCNQ4	0,491906512	-1,956681069	2,448587581
225312_at	COMMD6	6,467771477	4,020911969	2,446859508
212288_at	FNBP1	6,988392975	4,542978929	2,445414046
232184_at	ALS2	1,116462765	-1,32740143	2,443864195
230714_s_at	-	1,510970097	-0,931539147	2,442509244
203765_at	GCA	3,509819135	1,067365565	2,44245357
233919_s_at	HABP4	2,866549889	0,424693306	2,441856583
244512_at	HOXB-AS3	0,677745787	-1,763664074	2,441409861
217198_x_at	IGH@ /// IGHA2 //	0,677745787	-1,763664074	2,441409861
208498_s_at	ACTG1P4 /// AMY:	0,39105295	-2,049747196	2,440800146
1553493_a_at	TDH	0,39105295	-2,049747196	2,440800146
214472_at	HIST1H2AD /// HIS	1,383446753	-1,057301851	2,440748603
202336_s_at	PAM	4,381466431	1,941115499	2,440350932
213613_s_at	NADK	0,127732831	-2,312054429	2,43978726
200701_at	NPC2	5,388064835	2,948635566	2,439429269
241045_at	KDM8	0,167727503	-2,27001641	2,437743913
238776_x_at	OBSL1	0,20740896	-2,229233437	2,436642398
232674_at	UCN2	0,08619576	-2,350444759	2,436640519
220798_x_at	LPPR3	0,08619576	-2,350444759	2,436640519
228584_at	SGCB	1,775035811	-0,661178575	2,436214386
228463_at	FOXA3	0,524545436	-1,911206569	2,435752006
230976_at	AK8	0,524545436	-1,911206569	2,435752006
204551_s_at	AHSG	-0,135065865	-2,570522741	2,435456876
1553114_a_at	PTK6	1,983901861	-0,45121326	2,43511512
229157_at	PRKAG2-AS1	2,590269697	0,155747281	2,434522417
204981_at	SLC22A18	3,03433073	0,600385088	2,433945642
225929_s_at	RNF213	4,248669549	1,817403386	2,431266163
235006_at	CDKN2AIPNL	2,836219929	0,405752839	2,43046709
203467_at	PMM1	3,127551022	0,697221375	2,430329647
226679_at	SLC26A11	2,51558601	0,085534992	2,430051017

241618_at	-	0,764143511	-1,665791776	2,429935287
243417_at	ZADH2	0,764143511	-1,665791776	2,429935287
220151_at	C19orf73	0,764143511	-1,665791776	2,429935287
205342_s_at	SULT1C2	0,001114523	-2,4284334	2,429547923
230336_at	-	0,001114523	-2,4284334	2,429547923
220027_s_at	RASIP1	0,001114523	-2,4284334	2,429547923
219971_at	IL21R	2,138605583	-0,290694975	2,429300559
218668_s_at	RAP2C	4,711723394	2,283169614	2,42855378
208110_x_at	MED25	1,65554524	-0,771340337	2,426885576
211499_s_at	MAPK11	2,582572508	0,155747281	2,426825227
201012_at	ANXA1	6,982171914	4,555968307	2,426203607
224415_s_at	HINT2	5,365205123	2,939187671	2,426017452
234724_x_at	PCDHB18	0,282358733	-2,142286822	2,424645555
224501_at	C1orf170	0,95222556	-1,471833228	2,424058788
216220_s_at	ADORA1	0,95222556	-1,471833228	2,424058788
218021_at	DHRS4 /// DHRS4L	4,478885385	2,055739171	2,423146215
218645_at	ZNF277	5,466491247	3,043944324	2,422546923
222595_s_at	DIDO1	1,364555912	-1,057301851	2,421857763
232922_s_at	SLC17A9	1,364555912	-1,057301851	2,421857763
243252_at	-	2,989672611	0,568155442	2,421517169
229287_at	PCNX	2,304041045	-0,116767475	2,42080852
221892_at	H6PD	2,159837409	-0,260526297	2,420363706
210234_at	GRM4	0,900065578	-1,520022123	2,420087701
224125_at	PLEKHN1	0,900065578	-1,520022123	2,420087701
202783_at	NNT	4,135744086	1,715863686	2,419880401
221567_at	NOL3	1,830468611	-0,589001171	2,419469783
1556151_at	ITFG1	4,383685466	1,964670566	2,419014901
220158_at	LGALS14	0,556428218	-1,862269442	2,41869766
224920_x_at	MYADM	2,031158928	-0,386896102	2,41805503
202254_at	SIPA1L1	1,182925501	-1,234835326	2,417760827
207201_s_at	SLC22A1	1,528073958	-0,889222211	2,417296169
239428_at	-	0,319287178	-2,09678811	2,416075288
202068_s_at	LDLR	4,809922892	2,394276989	2,415645903
228811_at	-	0,458905032	-1,956681069	2,415586101
204990_s_at	ITGB4	0,458905032	-1,956681069	2,415586101
211354_s_at	LEPR	0,458905032	-1,956681069	2,415586101
225635_s_at	LOC100506710	3,269673646	0,85655369	2,413119956
214522_x_at	HIST1H2AD /// HIS	1,857989965	-0,554696666	2,412686632
239416_at	FBXL6	-0,088974936	-2,501359113	2,412384178
209802_at	PHLDA2	-0,088974936	-2,501359113	2,412384178
1552665_at	LOC84989	1,267146969	-1,145057014	2,412203984
228742_at	-	1,788922638	-0,623254098	2,412176736
235510_at	USHBP1	0,648195588	-1,763664074	2,411859662
202817_s_at	SS18	3,107338728	0,697221375	2,410117354
222726_s_at	EXOC5	3,107338728	0,697221375	2,410117354
214290_s_at	HIST2H2AA3 /// HI	5,31516215	2,906008589	2,409153561
232373_at	NOXA1	1,307201325	-1,100943374	2,408144699
221583_s_at	KCNMA1	2,71254166	0,306454867	2,406086793
244275_at	CISD2	0,355751005	-2,049747196	2,405498201
236127_at	-	0,491906512	-1,911206569	2,403113081

235548_at	APCDD1L	0,491906512	-1,911206569	2,403113081
236050_at	C11orf35	0,491906512	-1,911206569	2,403113081
244616_x_at	MDM2	1,983901861	-0,418248858	2,402150719
236054_at	-	0,735999505	-1,665791776	2,401791281
242385_at	RORB	1,510970097	-0,889222211	2,400192308
33579_i_at	GALR3	1,510970097	-0,889222211	2,400192308
220705_s_at	ADAMTS7 /// LOC:	0,58788177	-1,811780055	2,399661825
225954_s_at	MIDN	2,484824532	0,085534992	2,399289539
228276_at	SAMD1	0,97663406	-1,422257026	2,398891086
227564_at	HGSNAT	1,024284941	-1,372734086	2,397019027
219707_at	CPNE7	1,024284941	-1,372734086	2,397019027
203038_at	PTPRK	3,813758784	1,417157926	2,396600858
216405_at	-	0,045517965	-2,350444759	2,395962724
202770_s_at	CCNG2	3,473999051	1,078619386	2,395379665
235846_at	FSBP /// RAD54B	2,33082239	-0,063377083	2,394199473
231541_s_at	SPAG5-AS1	0,20740896	-2,186086603	2,393495563
209458_x_at	HBA1 /// HBA2	0,20740896	-2,186086603	2,393495563
210400_at	GRIN2C	0,20740896	-2,186086603	2,393495563
207927_at	HTR7	0,677745787	-1,715576125	2,393321912
210680_s_at	MASP1	0,001114523	-2,391863529	2,392978052
236615_at	-	1,802733148	-0,589001171	2,391734319
232470_at	SIK1	1,246401134	-1,145057014	2,391458148
214459_x_at	HLA-C	7,714209104	5,32370369	2,390505414
226112_at	SGCB	3,618399139	1,2298509	2,388548239
1555142_at	SLC9B1	0,820160788	-1,568385657	2,388546444
236845_at	TRIM62	0,820160788	-1,568385657	2,388546444
240630_at	-	1,936380191	-0,45121326	2,387593451
212223_at	IDS	4,116324812	1,72935563	2,386969182
234400_at	-	0,244681185	-2,142286822	2,386968007
207862_at	UPK2	0,524545436	-1,862269442	2,386814878
241405_at	LOC400604	0,524545436	-1,862269442	2,386814878
203029_s_at	PTPRN2	4,603614396	2,2171284	2,386485996
206723_s_at	LPAR2	2,063971513	-0,322340048	2,386311561
206670_s_at	GAD1	2,295108393	-0,089635713	2,384744106
205719_s_at	PAH	-0,043925712	-2,4284334	2,384507688
225940_at	EIF4E3	4,317231729	1,935144844	2,382086885
223858_at	ESRRB	0,618277321	-1,763664074	2,381941395
227563_at	FAM27E3	0,425036312	-1,956681069	2,381717382
244676_s_at	MTUS2	0,425036312	-1,956681069	2,381717382
205068_s_at	ARHGAP26	2,843031657	0,461385738	2,38164592
202506_at	SSFA2	3,649735397	1,268569037	2,38116636
230044_at	PCYT2	0,764143511	-1,616924751	2,381068262
201882_x_at	B4GALT1	0,764143511	-1,616924751	2,381068262
228789_at	MTMR6	1,364555912	-1,015398016	2,379953928
202828_s_at	MMP14	1,364555912	-1,015398016	2,379953928
235929_s_at	-	0,282358733	-2,09678811	2,379146843
231191_at	-	0,282358733	-2,09678811	2,379146843
227808_at	DNAJC15	3,060716358	0,681874787	2,378841572
214727_at	BRCA2	1,716003175	-0,661178575	2,377181749
227271_at	FGF11	1,857989965	-0,518232988	2,376222954

206080_at	PLCH2	1,047341799	-1,32740143	2,374743229
231710_at	CAPS	1,047341799	-1,32740143	2,374743229
219690_at	IGFLR1	3,509819135	1,135235995	2,37458314
238992_at	POLI	1,640193838	-0,734355396	2,374549234
208268_at	ADAM28	0,95222556	-1,422257026	2,374482587
211693_at	IGHA1	0,95222556	-1,422257026	2,374482587
206584_at	LY96	3,858118781	1,483698568	2,374420213
221068_at	KANK2	1,139057614	-1,234835326	2,37389294
229227_at	DICER1-AS1	1,182925501	-1,19085694	2,373782441
203382_s_at	APOE	0,707657549	-1,665791776	2,373449325
240389_at	TRPM6	1,000459215	-1,372734086	2,3731933
214165_s_at	HS6ST1	-0,230432956	-2,603471267	2,373038311
217436_x_at	HLA-J	6,262526809	3,89019679	2,372330018
235186_at	LOC388692	0,900065578	-1,471833228	2,371898806
224182_x_at	SEMA6B	1,440265662	-0,931539147	2,371804809
233660_at	EHD4	0,458905032	-1,911206569	2,370111601
230073_at	LOC100131564	0,458905032	-1,911206569	2,370111601
203686_at	MPG	3,481948861	1,112548615	2,369400245
227618_at	-	0,319287178	-2,049747196	2,369034374
231270_at	CA13 /// LOC1005(	0,319287178	-2,049747196	2,369034374
204776_at	THBS4	0,319287178	-2,049747196	2,369034374
205278_at	GAD1	1,745502609	-0,623254098	2,368756707
228869_at	SNX20	4,235548209	1,868130962	2,367417247
214934_at	ATP9B	2,771643417	0,405752839	2,365890579
218999_at	TMEM140	1,5944803	-0,771340337	2,365820637
239467_at	-	0,648195588	-1,715576125	2,363771713
244424_at	LOC439938	0,648195588	-1,715576125	2,363771713
1552912_a_at	IL23R	-0,49284695	-2,856570137	2,363723187
219582_at	OGFRL1	2,190140919	-0,172194225	2,362335144
232849_at	LOC100128988	1,345611839	-1,015398016	2,361009855
221978_at	HLA-F	1,345611839	-1,015398016	2,361009855
230657_at	-	0,355751005	-2,005029581	2,360780586
240792_at	-	0,355751005	-2,005029581	2,360780586
209759_s_at	ECI1	3,581038515	1,220470658	2,360567856
230104_s_at	TPPP	0,791908897	-1,568385657	2,360294554
228064_at	FAM211B	0,791908897	-1,568385657	2,360294554
212593_s_at	MIR4680 /// PDCD	6,241036142	3,881634568	2,359401574
212640_at	PTPLB	4,584332287	2,226451905	2,357880382
244030_at	STYX	0,127732831	-2,229233437	2,356966268
222080_s_at	SIRT5	0,167727503	-2,186086603	2,353814106
239768_x_at	GPATCH2	0,735999505	-1,616924751	2,352924256
225997_at	MOB1B	3,592169709	1,239268196	2,352901513
227001_at	NIPAL2	3,996262843	1,644429915	2,351832929
204416_x_at	APOC1	0,58788177	-1,763664074	2,351545844
220240_s_at	TMCO3	1,995527416	-0,354781582	2,350308998
209712_at	SLC35D1	3,107338728	0,757638486	2,349700242
223485_at	HAGHL	2,118167848	-0,231045907	2,349213755
227046_at	SLC39A11	3,559161816	1,209973561	2,349188255
1560767_at	HCG22	1,20396005	-1,145057014	2,349017064
213107_at	TNIK	1,686984721	-0,661178575	2,348163295

226708_at	-	-0,043925712	-2,391863529	2,347937817
208729_x_at	HLA-B	7,670856855	5,324257366	2,346599489
238327_at	ODF3B	0,873789652	-1,471833228	2,345622879
214992_s_at	DNASE2	2,824160383	0,479089184	2,345071199
203278_s_at	PHF21A	3,707586205	1,362900801	2,344685404
1557118_a_at	-	0,677745787	-1,665791776	2,343537563
1553257_at	GAL3ST3	0,244681185	-2,09678811	2,341469295
204934_s_at	HPN	0,244681185	-2,09678811	2,341469295
222156_x_at	CCPG1 /// DYX1C1	0,820160788	-1,520022123	2,340182911
243569_at	TMEM174	0,820160788	-1,520022123	2,340182911
241054_at	-	0,820160788	-1,520022123	2,340182911
241276_at	-	-0,088974936	-2,4284334	2,339458464
215021_s_at	NRXN3	-0,088974936	-2,4284334	2,339458464
217197_x_at	N4BP2L1	1,716003175	-0,623254098	2,339257273
227893_at	LINC00476	2,685337919	0,346964736	2,338373183
238850_at	LINC00461 /// MIR	2,972517974	0,634301128	2,338216846
227490_at	WDFY2	2,972517974	0,634301128	2,338216846
213056_at	FRMD4B	2,248155141	-0,089635713	2,337790854
221823_at	C5orf30	4,554653912	2,2171284	2,337525511
237108_x_at	FLJ42875	1,364555912	-0,972893339	2,337449251
227112_at	TMCC1	3,152091004	0,815200271	2,336890733
228005_at	ZXDB	2,21987062	-0,116767475	2,336638095
240550_at	-	0,524545436	-1,811780055	2,336325491
217936_at	ARHGAP5	2,621163856	0,286279868	2,334883988
1562747_at	-	-0,332405896	-2,665156778	2,332750882
207388_s_at	PTGES	0,764143511	-1,568385657	2,332529168
213666_at	38961	3,551632175	1,220470658	2,331161517
218174_s_at	C10orf57	2,267141818	-0,063377083	2,330518901
236737_at	C17orf56	1,139057614	-1,19085694	2,329914554
214578_s_at	ROCK1	2,791258167	0,461385738	2,329872429
227548_at	ORMDL1	2,636121902	0,306454867	2,329667035
1558459_s_at	LOC401320	1,440265662	-0,889222211	2,329487873
1556900_at	LOC149773	1,225123479	-1,100943374	2,326066853
244555_at	-	0,95222556	-1,372734086	2,324959646
208511_at	PTTG3P	3,768156719	1,44320939	2,324947329
234554_at	KCNK16	0,707657549	-1,616924751	2,3245823
203472_s_at	SLCO2B1	0,319287178	-2,005029581	2,324316759
209681_at	SLC19A2	3,544559557	1,220470658	2,324088899
214213_x_at	LMNA	1,936380191	-0,386896102	2,323276293
205257_s_at	AMPH	1,510970097	-0,811769547	2,322739644
201621_at	NBL1	3,77412549	1,451507543	2,322617947
229822_at	-	3,137734155	0,815200271	2,322533884
233757_x_at	-	2,890618076	0,568155442	2,322462633
213283_s_at	SALL2	2,62869474	0,306454867	2,322239873
1563370_at	-	0,458905032	-1,862269442	2,321174473
215461_at	ZNRF4	0,458905032	-1,862269442	2,321174473
211837_s_at	PTCRA	0,458905032	-1,862269442	2,321174473
237450_at	LOC389332	-0,180990326	-2,501359113	2,320368787
229583_at	-	0,556428218	-1,763664074	2,320092292
238904_at	NOVA2	0,556428218	-1,763664074	2,320092292

216845_x_at	MLL2	0,847529938	-1,471833228	2,319363165
218301_at	RNPEPL1	2,427650693	0,109458907	2,318191787
217379_at	-	6,243580567	3,925716571	2,317863996
229047_at	PLEKHB1	0,045517965	-2,27001641	2,315534375
216391_s_at	KLHL1	0,08619576	-2,229233437	2,315429197
221757_at	PIK3IP1	1,830468611	-0,484558493	2,315027105
1554068_s_at	C12orf66	2,16969527	-0,144765583	2,314460853
209915_s_at	NRXN1	0,648195588	-1,665791776	2,313987364
215819_s_at	RHCE /// RHD	0,648195588	-1,665791776	2,313987364
229326_at	TNFSF13	0,127732831	-2,186086603	2,313819434
223702_x_at	FTCD	0,127732831	-2,186086603	2,313819434
56256_at	SIDT2	3,987269395	1,673975776	2,313293618
214157_at	GNAS	3,533714224	1,220470658	2,313243566
237730_at	LOC100130700	0,001114523	-2,312054429	2,313168952
236604_at	BAHCC1	0,001114523	-2,312054429	2,313168952
1560744_at	LOC100505906	0,001114523	-2,312054429	2,313168952
225097_at	HIPK2	3,7799838	1,46719264	2,312791161
222222_s_at	HOMER3	0,355751005	-1,956681069	2,312432075
226705_at	FGFR1	4,253156553	1,941115499	2,312041054
235354_s_at	RSRC1	0,791908897	-1,520022123	2,311931021
1558906_a_at	LOC100505783	0,791908897	-1,520022123	2,311931021
214916_x_at	IGHA1 /// IGHA2 /,	0,791908897	-1,520022123	2,311931021
206098_at	ZBTB6	0,791908897	-1,520022123	2,311931021
228702_at	FLJ43663	3,912706782	1,601076277	2,311630505
208145_at	-	-0,384193355	-2,695640852	2,311447497
221014_s_at	RAB33B	2,597647262	0,286279868	2,311367394
204398_s_at	EML2	3,434684504	1,123905343	2,310779162
215796_at	-	1,421391637	-0,889222211	2,310613848
206287_s_at	ITIH4 /// MUSTN1	1,421391637	-0,889222211	2,310613848
216680_s_at	EPHB4	1,686984721	-0,623254098	2,310238819
242338_at	TMEM64	2,019346222	-0,290694975	2,310041197
221988_at	C19orf42	2,926639205	0,61779983	2,308839375
1569522_at	-	3,742498599	1,434743608	2,307754991
207623_at	ABCF2	1,024284941	-1,282520722	2,306805663
212221_x_at	IDS	5,949992459	3,643334026	2,306658433
221542_s_at	ERLIN2	2,484824532	0,178191865	2,306632667
205746_s_at	ADAM17	1,983901861	-0,322340048	2,306241909
238647_at	C14orf28	1,071336699	-1,234835326	2,306172025
1562577_at	-	1,20396005	-1,100943374	2,304903424
241809_at	FAM212B	0,735999505	-1,568385657	2,304385162
227251_at	DCAF5	0,735999505	-1,568385657	2,304385162
204248_at	GNA11	1,246401134	-1,057301851	2,303702984
229588_at	DNAJC10	1,246401134	-1,057301851	2,303702984
235047_x_at	NACC1	1,246401134	-1,057301851	2,303702984
207047_s_at	CLCNKA /// CLCNK	0,491906512	-1,811780055	2,303686566
242034_at	FBXL17	-0,088974936	-2,391863529	2,302888593
227203_at	FBXL17	2,745538988	0,443143223	2,302395765
241475_at	BREA2	0,39105295	-1,911206569	2,30225952
209699_x_at	AKR1C2 /// LOC10	2,209870035	-0,089635713	2,299505748
230938_x_at	ATF5	1,528073958	-0,771340337	2,299414295

230174_at	LYPLAL1	2,849132494	0,550017041	2,299115454
225965_at	DDHD1	2,99543179	0,697221375	2,298210415
1557002_x_at	ESPNL	0,873789652	-1,422257026	2,296046678
235136_at	ORMDL3	2,063971513	-0,231045907	2,29501742
227287_at	CITED2	0,244681185	-2,049747196	2,294428381
231828_at	LOC253039	4,070902947	1,777135134	2,293767813
223443_s_at	AMZ2P1	4,05770789	1,76396664	2,29374125
206085_s_at	CTH	3,618399139	1,324949741	2,293449398
220514_at	LOC100505870	-0,135065865	-2,4284334	2,293367535
205027_s_at	MAP3K8	2,492739983	0,20062106	2,292118923
217625_x_at	LOC100506190	0,820160788	-1,471833228	2,291994015
228087_at	CCDC126	3,742498599	1,451507543	2,290991056
227769_at	GPR27	4,68949381	2,398910172	2,290583638
205192_at	MAP3K14	1,701542258	-0,589001171	2,290543429
222223_s_at	IL36RN	-0,280273599	-2,570522741	2,290249142
226003_at	KIF21A	4,125707946	1,836262316	2,28944563
206687_s_at	PTPN6	2,955426542	0,666486137	2,288940404
226712_at	SSR1	3,389999385	1,101780212	2,288219173
221611_s_at	PHF7	0,524545436	-1,763664074	2,288209511
217468_at	CYP2D6	0,524545436	-1,763664074	2,288209511
204292_x_at	STK11	1,476175815	-0,811769547	2,287945362
235703_at	PLB1	0,282358733	-2,005029581	2,287388314
241707_at	TRABD2B	0,282358733	-2,005029581	2,287388314
210401_at	P2RX1	0,425036312	-1,862269442	2,287305754
225043_at	SLC15A4	3,761702872	1,475630127	2,286072745
208812_x_at	HLA-C	8,04240714	5,756809829	2,285597311
230011_at	MEI1	3,233229368	0,947673886	2,285555482
207861_at	CCL22	1,094118704	-1,19085694	2,284975644
203711_s_at	HIBCH	3,473999051	1,189078962	2,284920089
230902_at	-	0,764143511	-1,520022123	2,284165634
218784_s_at	SAYSD1	2,745538988	0,461385738	2,28415325
217180_at	-	0,618277321	-1,665791776	2,284069097
207037_at	TNFRSF11A	2,321828846	0,03784737	2,283981476
224525_s_at	OLA1	1,510970097	-0,771340337	2,282310434
213191_at	TICAM1	2,295108393	0,013190398	2,281917995
225205_at	KIF3B	3,624986574	1,343200523	2,28178605
214764_at	RRP15	1,830468611	-0,45121326	2,281681871
242961_x_at	DDX58	1,830468611	-0,45121326	2,281681871
227344_at	IKZF1	4,239531398	1,9592579	2,280273498
213081_at	ZBTB22	0,95222556	-1,32740143	2,27962699
1554208_at	MEI1	3,45766549	1,178100917	2,279564573
221660_at	MYL10	1,345611839	-0,931539147	2,277150986
236278_at	HIST1H3E	0,319287178	-1,956681069	2,275968247
210265_x_at	POU5F1P3	0,319287178	-1,956681069	2,275968247
1556911_at	-	1,578719607	-0,697182596	2,275902203
235429_at	EIF3E	2,312738346	0,03784737	2,274890976
1569089_a_at	FLJ35390 /// LOC1	0,045517965	-2,229233437	2,274751402
218232_at	C1QA	0,045517965	-2,229233437	2,274751402
211602_s_at	TRPC1	0,045517965	-2,229233437	2,274751402
219516_at	TRPV4	0,045517965	-2,229233437	2,274751402

216139_s_at	MAPK8IP3	0,045517965	-2,229233437	2,274751402
219523_s_at	ODZ3	0,045517965	-2,229233437	2,274751402
234980_at	TMEM56	4,155757614	1,881145546	2,274612068
1554237_at	SDCCAG8	1,983901861	-0,290694975	2,274596836
208248_x_at	APLP2	3,918896157	1,644429915	2,274466242
202027_at	TMEM184B	2,621163856	0,346964736	2,27419912
237105_at	LOC100506831	2,285848435	0,013190398	2,272658037
229667_s_at	HOXB8	0,556428218	-1,715576125	2,272004343
226516_at	MFSD12	1,610353504	-0,661178575	2,271532078
239697_x_at	C3orf67	0,001114523	-2,27001641	2,271130933
227340_s_at	RGMB	0,001114523	-2,27001641	2,271130933
204702_s_at	NFE2L3	4,281531373	2,010406245	2,271125128
216421_at	-	2,732422196	0,461385738	2,271036459
219136_s_at	LMF1	0,458905032	-1,811780055	2,270685086
243760_at	MIPEPP3	0,127732831	-2,142286822	2,270019652
243135_x_at	SNX8	0,127732831	-2,142286822	2,270019652
237753_at	IL21R	3,313347235	1,043469112	2,269878122
227471_at	HACE1	4,698307213	2,428719338	2,269587875
216096_s_at	NRXN1	-0,043925712	-2,312054429	2,268128717
241483_at	CLNK	-0,043925712	-2,312054429	2,268128717
205629_s_at	CRH	-0,043925712	-2,312054429	2,268128717
219317_at	POLI	3,685074281	1,417157926	2,267916355
226500_at	ZBTB47	0,355751005	-1,911206569	2,266957574
207196_s_at	TNIP1	5,095686705	2,828849722	2,266836983
37005_at	C1orf151-NBL1 ///	4,030989739	1,764173315	2,266816425
209043_at	PAPSS1	6,732322765	4,466269902	2,266052863
219078_at	GPATCH2	2,063971513	-0,201789721	2,265761234
220936_s_at	H2AFJ	0,648195588	-1,616924751	2,265120339
232530_at	PLD1	0,648195588	-1,616924751	2,265120339
220681_at	C22orf26	0,648195588	-1,616924751	2,265120339
239184_at	-	0,167727503	-2,09678811	2,264515613
231387_at	-	0,167727503	-2,09678811	2,264515613
209636_at	NFKB2	0,167727503	-2,09678811	2,264515613
237627_at	-	0,791908897	-1,471833228	2,263742125
1559102_at	-	1,745502609	-0,518232988	2,263735597
217996_at	PHLDA1	3,185443259	0,923203038	2,262240221
236226_at	BTLA	1,071336699	-1,19085694	2,262193638
241486_at	-	1,160996507	-1,100943374	2,261939881
213776_at	LOC157562	1,246401134	-1,015398016	2,26179915
205225_at	ESR1	-0,088974936	-2,350444759	2,261469824
243712_at	XIST	-0,088974936	-2,350444759	2,261469824
236553_at	LOC100507520	-0,088974936	-2,350444759	2,261469824
220195_at	MBD5	2,926639205	0,666486137	2,260153068
233701_at	-	0,97663406	-1,282520722	2,259154782
224974_at	SUDS3	3,621852128	1,362900801	2,258951327
217925_s_at	C6orf106	3,19484646	0,936232181	2,258614278
222870_s_at	B3GNT2	4,749149445	2,490640503	2,258508942
227877_at	ANXA2R	1,871521385	-0,386896102	2,258417487
226974_at	NEDD4L	2,085817135	-0,172194225	2,258011359
243463_s_at	RIT1	2,791258167	0,533258442	2,257999725

210083_at	SEMA7A	0,20740896	-2,049747196	2,257156156
203637_s_at	MID1	-0,135065865	-2,391863529	2,256797664
233320_at	TCAM1P	-0,135065865	-2,391863529	2,256797664
235487_at	-	0,735999505	-1,520022123	2,256021628
1569090_x_at	FLJ35390	0,491906512	-1,763664074	2,255570586
243263_at	LINC00482	0,491906512	-1,763664074	2,255570586
210645_s_at	TTC3 /// TTC3P1	5,41009057	3,155995136	2,254095433
232155_at	RNF213	2,267141818	0,013190398	2,25395142
219316_s_at	FLVCR2	1,802733148	-0,45121326	2,253946407
224297_s_at	SPTBN4	0,926325262	-1,32740143	2,253726692
222818_at	OSBPL10	0,58788177	-1,665791776	2,253673546
210249_s_at	NCOA1	4,885515979	2,63203137	2,253484608
1554234_at	KATNAL2	0,39105295	-1,862269442	2,253322392
215724_at	PLD1	-0,384193355	-2,635549085	2,25135573
207193_at	AGRP	0,244681185	-2,005029581	2,249710767
240393_at	-	1,730929079	-0,518232988	2,249162067
200797_s_at	MCL1	6,02615695	3,77783183	2,24832512
208644_at	PARP1	5,794713976	3,546728932	2,247985044
228658_at	MIAT	2,209870035	-0,036543687	2,246413722
218085_at	CHMP5	4,928564101	2,682180306	2,246383795
209506_s_at	NR2F1	0,677745787	-1,568385657	2,246131444
213375_s_at	N4BP2L1	3,807915835	1,563256447	2,244659388
228574_at	TMTC2	1,983901861	-0,260526297	2,244428158
239662_x_at	TMCC1	1,225123479	-1,015398016	2,240521495
228319_at	-	0,524545436	-1,715576125	2,240121561
209530_at	CACNB3	1,267146969	-0,972893339	2,240040308
211455_at	-	1,139057614	-1,100943374	2,240000988
230210_at	SUN1	1,307201325	-0,931539147	2,238740472
1554517_x_at	LINC00537	1,307201325	-0,931539147	2,238740472
242790_at	-	2,348178434	0,109458907	2,238719528
208480_s_at	ABCC6	-0,332405896	-2,570522741	2,238116846
204073_s_at	C11orf9	2,698955954	0,461385738	2,237570217
215510_at	ETV2	0,425036312	-1,811780055	2,236816367
234688_x_at	CNTROB	0,764143511	-1,471833228	2,235976739
1554961_at	FGFR4	-0,230432956	-2,466349414	2,235916458
230740_at	EHD3	0,95222556	-1,282520722	2,234746282
237322_at	MIAT	1,383446753	-0,84928999	2,232736743
229920_at	-	1,383446753	-0,84928999	2,232736743
209394_at	ASMTL	2,765070895	0,533258442	2,231812454
201347_x_at	GRHPR	5,540167563	3,308395318	2,231772245
229126_at	TMEM19	4,06801876	1,836262316	2,231756444
215313_x_at	HLA-A	8,245105013	6,01473878	2,230366233
213467_at	RND2	0,001114523	-2,229233437	2,23034796
227710_s_at	TPT1-AS1	0,001114523	-2,229233437	2,23034796
219939_s_at	CSDE1	6,985189249	4,755797163	2,229392087
214696_at	MIR22 /// MIR22H	1,640193838	-0,589001171	2,229195009
33850_at	MAP4	3,11945846	0,89035865	2,22909981
208683_at	CAPN2	6,529956571	4,300870341	2,22908623
222154_s_at	SPATS2L	4,965350893	2,736663738	2,228687155
207581_s_at	MAGEB4	0,08619576	-2,142286822	2,228482581

228619_x_at	TIPRL	4,817982599	2,589579418	2,228403181
232765_x_at	SLC22A31	1,493647653	-0,734355396	2,228003049
210161_at	-	0,707657549	-1,520022123	2,227679672
228383_at	PNPLA7	0,900065578	-1,32740143	2,227467008
224548_at	HES7	0,900065578	-1,32740143	2,227467008
235450_at	-	0,900065578	-1,32740143	2,227467008
222851_at	ZNF654	1,936380191	-0,290694975	2,227075166
228905_at	PCM1	1,528073958	-0,697182596	2,225256555
226737_at	SLC25A42	1,528073958	-0,697182596	2,225256555
242783_at	-	0,127732831	-2,09678811	2,22452094
219249_s_at	FKBP10	0,127732831	-2,09678811	2,22452094
32811_at	MYO1C	3,485470903	1,261012426	2,224458477
212947_at	SLC9A8	2,107136847	-0,116767475	2,223904321
213143_at	C2orf72	0,458905032	-1,763664074	2,222569106
203892_at	WFDC2	0,458905032	-1,763664074	2,222569106
231774_at	KCNIP3	0,458905032	-1,763664074	2,222569106
214689_at	PAPPA2	0,556428218	-1,665791776	2,222219994
222219_s_at	TLE6	0,556428218	-1,665791776	2,222219994
219209_at	IFIH1	4,096676757	1,874839058	2,221837699
214906_x_at	N4BP2L1	2,019346222	-0,201789721	2,221135943
239254_at	CCDC40	-0,280273599	-2,501359113	2,221085514
225972_at	TMEM64	1,898032047	-0,322340048	2,220372095
243441_at	HOXB-AS4	1,898032047	-0,322340048	2,220372095
204506_at	PPP3R1	3,751261994	1,531877749	2,219384245
202879_s_at	CYTH1	1,287793882	-0,931539147	2,219333029
1562587_at	CLNK	-0,384193355	-2,603471267	2,219277912
212331_at	RBL2	4,945274128	2,726850891	2,218423237
212848_s_at	C9orf3 /// LOC100	2,836219929	0,61779983	2,218420099
205104_at	SNPH	1,160996507	-1,057301851	2,218298358
218609_s_at	NUDT2	3,261582786	1,043469112	2,218113674
219834_at	ALS2CR8	0,355751005	-1,862269442	2,218020447
233939_at	REXO1	0,355751005	-1,862269442	2,218020447
33304_at	ISG20	3,767353444	1,549522217	2,217831227
207933_at	ZP2	0,167727503	-2,049747196	2,217474699
1553590_at	FAM27E1 /// FAM	0,167727503	-2,049747196	2,217474699
231861_at	LRP10	1,116462765	-1,100943374	2,217406139
201749_at	ECE1	1,830468611	-0,386896102	2,217364713
214482_at	ZBTB25	2,943764123	0,727123109	2,216641015
237401_at	ACTN1	1,326463531	-0,889222211	2,215685742
200831_s_at	LOC100652751 ///	3,414594082	1,199296812	2,215297269
202778_s_at	ZMYM2	3,473999051	1,259081178	2,214917873
218581_at	ABHD4	3,137734155	0,923203038	2,214531116
219943_s_at	USP46	1,402495885	-0,811769547	2,214265431
210782_x_at	GRIN1	1,402495885	-0,811769547	2,214265431
213376_at	ZBTB1	4,696534086	2,482273566	2,21426052
235614_at	TMEM151A	0,100803275	-2,112780549	2,213583825
225750_at	ERO1L	5,605561887	3,392128215	2,213433672
206083_at	BAI1	0,20740896	-2,005029581	2,212438541
213927_at	MAP3K9	0,97663406	-1,234835326	2,211469386
228381_at	ATF7IP2 /// LOC10	0,97663406	-1,234835326	2,211469386

233268_s_at	CHURC1	5,218963893	3,007560601	2,211403292
224897_at	WDR26	3,544559557	1,333668715	2,210890843
231084_at	WDR96	-0,180990326	-2,391863529	2,210873203
207612_at	WNT8B	-0,180990326	-2,391863529	2,210873203
1561500_at	CTU2	-0,180990326	-2,391863529	2,210873203
223498_at	SPECC1	1,476175815	-0,734355396	2,210531211
240963_x_at	PLXDC1	0,926325262	-1,282520722	2,208845984
238543_x_at	MDGA1	0,735999505	-1,471833228	2,207832733
1557008_at	LOC340107	0,735999505	-1,471833228	2,207832733
240147_at	C7orf50	0,491906512	-1,715576125	2,207482637
241667_x_at	-	0,491906512	-1,715576125	2,207482637
212984_at	ATF2	3,881435528	1,674071917	2,207363611
204820_s_at	BTN3A2 /// BTN3A	5,602635435	3,395519718	2,207115716
204540_at	EEF1A2	3,362258367	1,156599976	2,205658391
212594_at	MIR4680 /// PDCD	3,152091004	0,947673886	2,204417118
227708_at	EEF1A1 /// LOC10(	4,454387658	2,250228409	2,204159249
219397_at	COQ10B	3,789279973	1,586121802	2,203158172
202771_at	PIEZO1	4,258822873	2,055739171	2,203083702
228270_at	ZNF853	0,39105295	-1,811780055	2,202833005
226635_at	LOC100506710	4,642573329	2,440833194	2,201740135
226799_at	FGD6	0,244681185	-1,956681069	2,201362255
244199_at	TWF1	0,244681185	-1,956681069	2,201362255
220148_at	ALDH8A1	0,244681185	-1,956681069	2,201362255
1556724_at	LOC100288490	0,244681185	-1,956681069	2,201362255
223074_s_at	AIF1L	0,244681185	-1,956681069	2,201362255
238827_at	-	0,873789652	-1,32740143	2,201191082
213353_at	ABCA5	2,190140919	-0,011001236	2,201142155
217730_at	TMBIM1	4,624674869	2,424202743	2,200472125
213359_at	HNRNPD	4,26103332	2,06133574	2,19969758
221754_s_at	CORO1B	1,182925501	-1,015398016	2,198323517
1557113_at	LOC283588	-0,230432956	-2,4284334	2,198000444
233968_at	CST11	-0,230432956	-2,4284334	2,198000444
202643_s_at	TNFAIP3	5,126622398	2,930238085	2,196384313
237144_at	LTBP3	1,139057614	-1,057301851	2,196359465
36554_at	ASMTL	2,470825314	0,275647699	2,195177616
216366_x_at	-	1,640193838	-0,554696666	2,194890504
219172_at	UBTD1	0,282358733	-1,911206569	2,193565302
1553890_s_at	NTN5	0,282358733	-1,911206569	2,193565302
226844_at	MOB3B	0,820160788	-1,372734086	2,192894873
230931_at	PLG	1,047341799	-1,145057014	2,192398813
235628_x_at	FLJ33630	2,127965541	-0,063377083	2,191342624
244790_at	MTCP1	1,000459215	-1,19085694	2,191316155
202593_s_at	GDE1	6,058877233	3,868513789	2,190363444
203993_x_at	C21orf2	0,524545436	-1,665791776	2,190337212
235455_at	FAM131C	0,524545436	-1,665791776	2,190337212
226550_at	SLC9A7	2,51558601	0,326777209	2,1888088
227879_at	ALKBH7	0,425036312	-1,763664074	2,188700387
204698_at	ISG20	3,903941986	1,715863686	2,188078301
231447_at	DZANK1-AS1	0,045517965	-2,142286822	2,187804786
219379_x_at	ZNF358	0,045517965	-2,142286822	2,187804786

204242_s_at	ACOX3	2,042564225	-0,144765583	2,187329808
241430_at	C2orf51	0,001114523	-2,186086603	2,187201126
216734_s_at	CXCR5	0,001114523	-2,186086603	2,187201126
213717_at	LDB3	0,95222556	-1,234835326	2,187060886
209241_x_at	MINK1	0,618277321	-1,568385657	2,186662978
217226_s_at	SFXN3	0,618277321	-1,568385657	2,186662978
213988_s_at	SAT1	3,529682801	1,343200523	2,186482278
233883_at	SNX24	-0,384193355	-2,570522741	2,186329387
221752_at	SSH1	2,719381633	0,533258442	2,186123191
240434_at	-	3,406503548	1,220470658	2,18603289
1557339_at	-	-0,043925712	-2,229233437	2,185307726
1564598_a_at	VWA3B	-0,043925712	-2,229233437	2,185307726
206891_at	ACTN3	-0,043925712	-2,229233437	2,185307726
209234_at	KIF1B	4,558198545	2,373249966	2,184948579
204662_at	CCP110	5,417310128	3,234381926	2,182928202
204856_at	B3GNT3	0,900065578	-1,282520722	2,1825863
225583_at	UXS1	5,161840645	2,979585083	2,182255561
206599_at	SLC16A5	0,319287178	-1,862269442	2,181556619
228731_at	GUCY1A2	-0,088974936	-2,27001641	2,181041474
209140_x_at	HLA-B	8,060706665	5,879895133	2,180811532
226536_at	NSMCE2	5,766181318	3,586015261	2,180166057
239146_at	CLDND1	0,707657549	-1,471833228	2,179490776
1554980_a_at	ATF3	1,760255626	-0,418248858	2,178504484
205062_x_at	ARID4A	2,2388565	0,061773582	2,177082918
1560694_at	SFT2D1	1,287793882	-0,889222211	2,177016093
210931_at	RNF6	-0,135065865	-2,312054429	2,176988564
223571_at	C1QTNF6	-0,135065865	-2,312054429	2,176988564
213627_at	MAGED2	3,67542249	1,500059916	2,175362573
231008_at	UNC5CL	0,847529938	-1,32740143	2,174931368
214133_at	MUC6	0,458905032	-1,715576125	2,174481157
226021_at	RDH10	1,476175815	-0,697182596	2,173358411
224138_at	CBX2	0,556428218	-1,616924751	2,173352969
202972_s_at	FAM13A	0,556428218	-1,616924751	2,173352969
210794_s_at	MEG3	0,556428218	-1,616924751	2,173352969
1561579_at	LOC728445	0,167727503	-2,005029581	2,172757084
236258_at	C20orf151	0,167727503	-2,005029581	2,172757084
208590_x_at	GJA3	0,167727503	-2,005029581	2,172757084
235460_at	SNX22	0,167727503	-2,005029581	2,172757084
210937_s_at	PDX1	-0,49284695	-2,665156778	2,172309828
217312_s_at	COL7A1	1,071336699	-1,100943374	2,172280073
220262_s_at	DLK2	1,816938997	-0,354781582	2,171720579
204056_s_at	MVK	2,159837409	-0,011001236	2,170838645
228254_at	STAM2	2,613382867	0,443143223	2,170239644
201149_s_at	TIMP3	-0,180990326	-2,350444759	2,169454434
1553101_a_at	ALKBH5 /// LOC10	3,636292808	1,46719264	2,169100169
209505_at	NR2F1	-0,332405896	-2,501359113	2,168953217
204656_at	SHB	0,648195588	-1,520022123	2,168217711
202793_at	LPCAT3	1,995527416	-0,172194225	2,167721641
224203_at	SUFU	0,355751005	-1,811780055	2,16753106
217006_x_at	FASN	0,355751005	-1,811780055	2,16753106

1559675_at	-	0,97663406	-1,19085694	2,167491
239000_at	BRD4	0,97663406	-1,19085694	2,167491
223694_at	TRIM7	0,97663406	-1,19085694	2,167491
228185_at	ZNF25	2,276736537	0,109458907	2,16727763
214311_at	ZFPL1	1,610353504	-0,554696666	2,16505017
203945_at	ARG2	2,365121417	0,20062106	2,164500358
225769_at	COG6	4,310459066	2,147194726	2,16326434
222209_s_at	TMEM135	5,092485786	2,930238085	2,162247701
1569348_at	MIR4500HG /// TP	-0,230432956	-2,391863529	2,161430573
227806_at	C16orf74	0,926325262	-1,234835326	2,161160588
223640_at	HCST	4,85214636	2,693044596	2,159101764
214237_x_at	PAWR	0,735999505	-1,422257026	2,158256531
203722_at	ALDH4A1	0,491906512	-1,665791776	2,157698288
215700_x_at	CPNE6	0,491906512	-1,665791776	2,157698288
211001_at	TRIM29	0,491906512	-1,665791776	2,157698288
231151_at	DLGAP3	0,491906512	-1,665791776	2,157698288
228667_at	AGPAT4	0,491906512	-1,665791776	2,157698288
241333_x_at	-	0,491906512	-1,665791776	2,157698288
209328_x_at	HIGD2A	2,16969527	0,013190398	2,156504872
1555935_s_at	HUNK	1,307201325	-0,84928999	2,156491315
219587_at	TTC12	0,873789652	-1,282520722	2,156310374
220627_at	CST8	0,58788177	-1,568385657	2,156267427
207669_at	KRT83	0,244681185	-1,911206569	2,155887755
223460_at	CAMKK1	1,421391637	-0,734355396	2,155747033
206461_x_at	MT1H	4,958099314	2,802594885	2,155504429
232408_at	ZFYVE28	0,39105295	-1,763664074	2,154717025
213177_at	MAPK8IP3	0,39105295	-1,763664074	2,154717025
1552893_at	CAMK2N2	1,139057614	-1,015398016	2,15445563
241921_x_at	-	1,139057614	-1,015398016	2,15445563
210475_at	POU3F1	-0,384193355	-2,536339282	2,152145927
204306_s_at	CD151	2,685337919	0,533258442	2,152079477
219801_at	ZNF34	1,528073958	-0,623254098	2,151328057
202876_s_at	PBX2	2,62869474	0,479089184	2,149605556
233063_s_at	-	1,730929079	-0,418248858	2,149177937
224953_at	YIPF5	3,18076822	1,032267596	2,148500623
230039_at	-	0,820160788	-1,32740143	2,147562218
210784_x_at	LILRA6 /// LILRB3	0,820160788	-1,32740143	2,147562218
228261_at	MIB2	1,000459215	-1,145057014	2,145516229
210128_s_at	LTB4R	1,000459215	-1,145057014	2,145516229
236942_at	-	1,000459215	-1,145057014	2,145516229
242900_at	ALG10B	1,88491011	-0,260526297	2,145436407
235148_at	KRTCAP3	0,282358733	-1,862269442	2,144628175
1553183_at	UMODL1	-0,551284523	-2,695640852	2,144356329
203571_s_at	C10orf116	0,001114523	-2,142286822	2,143401344
212268_at	SERPINB1	4,030423661	1,887158627	2,143265034
224355_s_at	MS4A8B	0,045517965	-2,09678811	2,142306075
238943_at	FIBCD1	0,045517965	-2,09678811	2,142306075
204657_s_at	SHB	-0,043925712	-2,186086603	2,142160891
225020_at	DAB2IP	1,910557155	-0,231045907	2,141603062
216256_at	GRM8	0,524545436	-1,616924751	2,141470188

219726_at	NLGN3	0,524545436	-1,616924751	2,141470188
202926_at	NBAS	4,582263053	2,440833194	2,141429859
227063_at	C17orf61	4,57799322	2,436714502	2,141278717
206150_at	CD27	0,425036312	-1,715576125	2,140612437
206582_s_at	GPR56	0,425036312	-1,715576125	2,140612437
223116_at	BCAR1	0,425036312	-1,715576125	2,140612437
228670_at	TEP1	2,582572508	0,443143223	2,139429285
243840_at	CLSPN	1,816938997	-0,322340048	2,139279045
200805_at	LMAN2	4,613033266	2,47417686	2,138856407
223158_s_at	NEK6	4,221194173	2,082919929	2,138274244
214296_x_at	IZUMO4	1,476175815	-0,661178575	2,13735439
209304_x_at	GADD45B	3,336134047	1,199296812	2,136837235
231209_at	EXOC3L4	0,08619576	-2,049747196	2,135942956
202411_at	IFI27	0,08619576	-2,049747196	2,135942956
201865_x_at	NR3C1	5,887939753	3,7524971	2,135442653
244080_at	-	-0,135065865	-2,27001641	2,134950545
237143_at	-	-0,135065865	-2,27001641	2,134950545
225325_at	MFSD6	4,359963018	2,226451905	2,133511112
202249_s_at	DCAF8	2,096248267	-0,036543687	2,132791954
211650_x_at	IGH@ /// IGHA1 //	0,127732831	-2,005029581	2,132762412
229780_at	-	0,127732831	-2,005029581	2,132762412
207370_at	IBSP	0,127732831	-2,005029581	2,132762412
207784_at	ARSD	0,127732831	-2,005029581	2,132762412
210557_x_at	CSF1	0,319287178	-1,811780055	2,131067233
237027_at	LSAMP-AS1	-0,180990326	-2,312054429	2,131064103
218531_at	TMEM134	3,928402242	1,797682937	2,130719305
1553399_a_at	C17orf69	0,847529938	-1,282520722	2,13005066
214754_at	TET3	0,707657549	-1,422257026	2,129914575
210144_at	TBC1D22A	1,983901861	-0,144765583	2,128667444
219718_at	FGGY	1,071336699	-1,057301851	2,128638549
206993_at	ATP5S	1,610353504	-0,518232988	2,128586492
59625_at	NOL3	1,709189633	-0,418696293	2,127885926
217714_x_at	MIR3917 /// STMN	2,392346173	0,265475485	2,126870688
205538_at	CORO2A	3,228532861	1,101780212	2,12675265
219369_s_at	OTUB2	1,92317941	-0,201789721	2,124969131
229537_at	LMO4	0,458905032	-1,665791776	2,124696808
217153_at	ARHGAP1	0,458905032	-1,665791776	2,124696808
222092_at	PTPN21	0,458905032	-1,665791776	2,124696808
223784_at	TMEM27	0,167727503	-1,956681069	2,124408573
234302_s_at	ALKBH5 /// LOC10	4,350356009	2,226451905	2,123904104
225629_s_at	ZBTB4	2,507817181	0,385582632	2,122234549
201152_s_at	MBNL1	7,265836338	5,14488611	2,120950227
225003_at	TMEM205	3,925077605	1,804519951	2,120557654
239843_at	RIT1	2,365121417	0,244646697	2,12047472
203081_at	CTNNBIP1	3,418470674	1,298349073	2,120121601
230625_s_at	TSPAN12	0,648195588	-1,471833228	2,120028816
228876_at	BAIAP2L2	0,648195588	-1,471833228	2,120028816
228333_at	ZEB2	0,648195588	-1,471833228	2,120028816
242479_s_at	MCM4	0,648195588	-1,471833228	2,120028816
212884_x_at	APOE	0,648195588	-1,471833228	2,120028816

205795_at	NRXN3	-0,230432956	-2,350444759	2,120011803
206268_at	LEFTY1	-0,230432956	-2,350444759	2,120011803
237940_s_at	-	-0,230432956	-2,350444759	2,120011803
231158_x_at	PTBP1	-0,230432956	-2,350444759	2,120011803
204612_at	PKIA	4,876721708	2,757131856	2,119589852
217001_x_at	HLA-DOA	0,355751005	-1,763664074	2,119415079
239368_at	-	0,355751005	-1,763664074	2,119415079
237620_at	ODF3L2	0,791908897	-1,32740143	2,119310327
217413_s_at	TNXB	0,791908897	-1,32740143	2,119310327
232972_at	C17orf72	0,791908897	-1,32740143	2,119310327
203045_at	NINJ1	3,349017424	1,2298509	2,119166524
226023_at	MAP2K7	1,307201325	-0,811769547	2,118970872
221111_at	IL26	1,307201325	-0,811769547	2,118970872
1562428_at	LOC654780	0,20740896	-1,911206569	2,11861553
202527_s_at	SMAD4	3,577387089	1,459545271	2,117841819
234140_s_at	STIM2	3,470020347	1,352622437	2,117397909
212403_at	UBE3B	3,273854609	1,156599976	2,117254633
222835_at	THSD4	0,926325262	-1,19085694	2,117182202
227809_at	ZC3H6	1,88491011	-0,231045907	2,115956017
220344_at	C11orf16	1,225123479	-0,889222211	2,11434569
243148_at	-	-0,551284523	-2,665156778	2,113872255
209566_at	INSIG2	4,163636979	2,050286491	2,113350488
1557341_x_at	-	-0,280273599	-2,391863529	2,11158993
202769_at	CCNG2	4,482930296	2,373249966	2,10968033
237345_at	-	0,491906512	-1,616924751	2,108831263
230327_at	LOC730098	0,491906512	-1,616924751	2,108831263
240686_x_at	TFRC	0,491906512	-1,616924751	2,108831263
236030_at	RCOR2	0,735999505	-1,372734086	2,108733591
220303_at	PDZD3	0,735999505	-1,372734086	2,108733591
223284_at	NAT14	2,908690075	0,80114751	2,107542565
204500_s_at	AGTPBP1	4,29492784	2,187622928	2,107304912
218815_s_at	TMEM51	0,244681185	-1,862269442	2,106950627
233517_s_at	HIF3A	0,244681185	-1,862269442	2,106950627
207144_s_at	CITED1	0,39105295	-1,715576125	2,106629075
1568859_a_at	SLC8A3	0,39105295	-1,715576125	2,106629075
227078_at	TMEM110	2,392346173	0,286279868	2,106066306
206233_at	B4GALT6	2,530390387	0,424693306	2,105697081
204512_at	HIVEP1	4,005144537	1,899778623	2,105365914
227978_s_at	ZADH2	1,716003175	-0,386896102	2,102899277
207133_x_at	ALPK1	0,820160788	-1,282520722	2,10268151
226160_at	H6PD	2,25755351	0,155747281	2,10180623
228088_at	SESTD1	1,000459215	-1,100943374	2,101402589
217870_s_at	CMPK1	6,577314721	4,4761909	2,101123821
40562_at	GNA11	0,900069767	-1,20053272	2,100602487
218537_at	HCFC1R1	2,063971513	-0,036543687	2,1005152
210029_at	IDO1	0,677745787	-1,422257026	2,100002813
208091_s_at	VOPP1	6,122457274	4,022483924	2,09997335
216671_x_at	MUC8	2,159837409	0,061773582	2,098063827
230683_at	-	0,001114523	-2,09678811	2,097902633
209646_x_at	ALDH1B1	1,775035811	-0,322340048	2,097375859

230089_s_at	C19orf6	-0,088974936	-2,186086603	2,097111667
230936_at	DNAJB13	-0,088974936	-2,186086603	2,097111667
224695_at	C2orf29	4,747567561	2,65087728	2,096690281
221236_s_at	STMN4	-0,332405896	-2,4284334	2,096027504
232378_at	SLC5A9	0,045517965	-2,049747196	2,095265161
240305_at	-	0,045517965	-2,049747196	2,095265161
229122_x_at	NPRL3	0,045517965	-2,049747196	2,095265161
1559361_at	MACC1	-0,135065865	-2,229233437	2,094167572
220686_s_at	PIWIL2	-0,135065865	-2,229233437	2,094167572
208516_at	MTNR1B	-0,135065865	-2,229233437	2,094167572
228015_s_at	TRIM8	0,282358733	-1,811780055	2,094138788
1564573_at	IFITM10	0,282358733	-1,811780055	2,094138788
211656_x_at	HLA-DQB1 /// LOC	5,277968348	3,18412401	2,093844338
218409_s_at	DNAJC1	3,694479488	1,601076277	2,093403211
206480_at	LTC4S	0,524545436	-1,568385657	2,092931093
214819_at	IQSEC2	0,524545436	-1,568385657	2,092931093
212093_s_at	MTUS1	0,524545436	-1,568385657	2,092931093
223153_x_at	TMUB1	2,41945542	0,326777209	2,09267821
227204_at	PARD6G	1,160996507	-0,931539147	2,092535654
210515_at	HNF1A	1,160996507	-0,931539147	2,092535654
219475_at	OSGIN1	0,764143511	-1,32740143	2,091544941
202390_s_at	HTT	2,356619952	0,265475485	2,091144467
228213_at	H2AFJ	0,425036312	-1,665791776	2,090828088
219522_at	FJX1	0,425036312	-1,665791776	2,090828088
207914_x_at	EVX1	0,425036312	-1,665791776	2,090828088
241450_at	RSPO1	0,425036312	-1,665791776	2,090828088
217087_at	C1orf68	0,618277321	-1,471833228	2,090110549
1554571_at	APBB1IP	-0,180990326	-2,27001641	2,089026084
235672_at	MAP6	-0,180990326	-2,27001641	2,089026084
223215_s_at	JKAMP	4,407522442	2,31904917	2,088473273
211744_s_at	CD58	5,073400307	2,98507484	2,088325466
208704_x_at	APLP2	3,562907192	1,475630127	2,087277065
230314_at	-	1,071336699	-1,015398016	2,086734715
204121_at	GADD45G	1,071336699	-1,015398016	2,086734715
235201_at	FOXP2	2,452674045	0,366031214	2,086642832
210323_at	TEKT2	-0,609040214	-2,695640852	2,086600638
224036_s_at	LMBR1	1,730929079	-0,354781582	2,08571066
214855_s_at	RALGAPA1	2,719381633	0,634301128	2,085080505
226475_at	FAM118A	3,152091004	1,067365565	2,084725439
223800_s_at	LIMS3 /// LIMS3L	0,127732831	-1,956681069	2,0844139
221417_x_at	S1PR5	0,127732831	-1,956681069	2,0844139
210316_at	FLT4	0,127732831	-1,956681069	2,0844139
221250_s_at	MXD3	2,966903803	0,883374248	2,083529555
238051_x_at	PWWP2B	0,319287178	-1,763664074	2,082951252
232222_at	C18orf49	0,319287178	-1,763664074	2,082951252
205403_at	IL1R2	0,319287178	-1,763664074	2,082951252
201646_at	SCARB2	1,528073958	-0,554696666	2,082770625
221290_s_at	MUM1	1,760255626	-0,322340048	2,082595673
224872_at	DIP2B	3,691400566	1,60918327	2,082217296
1552327_at	ARMCX4	-0,384193355	-2,466349414	2,082156059

229151_at	SLC14A1	-0,384193355	-2,466349414	2,082156059
219663_s_at	TMEM121	-0,384193355	-2,466349414	2,082156059
228404_at	IRX2	-0,230432956	-2,312054429	2,081621473
230124_at	PRKCH	-0,230432956	-2,312054429	2,081621473
207004_at	BCL2	1,024284941	-1,057301851	2,081586792
222391_at	TMEM30A	4,82490062	2,743433212	2,081467407
207485_x_at	BTN3A1	1,383446753	-0,697182596	2,080629349
231436_at	-	0,707657549	-1,372734086	2,080391634
206669_at	GAD1	2,25755351	0,178191865	2,079361645
228855_at	NUDT7	3,18076822	1,101780212	2,078988008
211867_s_at	PCDHA10	0,167727503	-1,911206569	2,078934073
231168_at	-	0,167727503	-1,911206569	2,078934073
206758_at	EDN2	0,167727503	-1,911206569	2,078934073
229361_at	SLC25A27	0,167727503	-1,911206569	2,078934073
241213_x_at	-	0,167727503	-1,911206569	2,078934073
211123_at	SLC5A5	1,267146969	-0,811769547	2,078916516
204357_s_at	LIMK1	1,307201325	-0,771340337	2,078541662
38521_at	CD22	0,850179375	-1,228269847	2,078449222
236119_s_at	SPRR2G	0,97663406	-1,100943374	2,077577434
244157_x_at	-	0,97663406	-1,100943374	2,077577434
211356_x_at	LEPR	0,97663406	-1,100943374	2,077577434
215343_at	CCDC88C	3,861345795	1,784182261	2,077163535
221064_s_at	UNKL	2,209870035	0,133104519	2,076765516
1556627_at	DRP2	0,458905032	-1,616924751	2,075829783
207298_at	SLC17A3	0,458905032	-1,616924751	2,075829783
216247_at	RPS20 /// SNORD5	0,458905032	-1,616924751	2,075829783
1555628_a_at	HAVCR2	0,458905032	-1,616924751	2,075829783
216911_s_at	HIC2	0,458905032	-1,616924751	2,075829783
202180_s_at	MVP	3,418470674	1,343200523	2,075270151
227898_s_at	ZFP41	1,843966654	-0,231045907	2,075012561
228081_at	CCNG2	1,686984721	-0,386896102	2,073880823
226493_at	KCTD18	3,789279973	1,715863686	2,073416288
209649_at	STAM2	2,41945542	0,346964736	2,072490684
206883_x_at	GP9	1,182925501	-0,889222211	2,072147712
237853_x_at	KRTAP10-12	0,355751005	-1,715576125	2,07132713
230907_at	GPRC5C	0,355751005	-1,715576125	2,07132713
217390_x_at	-	0,355751005	-1,715576125	2,07132713
212819_at	ASB1	0,648195588	-1,422257026	2,070452614
235845_at	SP5	0,648195588	-1,422257026	2,070452614
1556155_at	-	0,648195588	-1,422257026	2,070452614
216596_at	DKFZP434L187	-0,280273599	-2,350444759	2,07017116
202391_at	BASP1	-0,280273599	-2,350444759	2,07017116
213181_s_at	MOCS1	0,20740896	-1,862269442	2,069678402
206782_s_at	DNAJC4	2,107136847	0,03784737	2,069289476
202203_s_at	AMFR	2,765070895	0,697221375	2,067849521
208490_x_at	HIST1H2BC /// HIS	3,614400403	1,547376668	2,067023735
221061_at	PKD2L1	1,094118704	-0,972893339	2,067012043
241342_at	TMEM65	2,199941239	0,133104519	2,06683672
202422_s_at	ACSL4	3,132805465	1,067365565	2,0654399
227878_s_at	ALKBH7	3,975619308	1,911502437	2,064116872

221608_at	WNT6	-0,437449947	-2,501359113	2,063909166
223482_at	TMEM120A	3,273854609	1,209973561	2,063881048
228386_s_at	DDX59	2,410823487	0,346964736	2,063858751
241999_at	SFXN5	0,735999505	-1,32740143	2,063400935
202484_s_at	MBD2	6,242440031	4,179683441	2,06275659
229382_at	FAM212B	1,047341799	-1,015398016	2,062739815
203271_s_at	UNC119	3,470020347	1,408220608	2,061799739
1553952_at	ZDHHC19	0,491906512	-1,568385657	2,060292168
217742_s_at	WAC	4,550683059	2,490640503	2,060042556
1570242_x_at	SPATA21	0,58788177	-1,471833228	2,059714998
243585_at	ATP13A5	-0,332405896	-2,391863529	2,059457633
240392_at	-	-0,332405896	-2,391863529	2,059457633
243575_at	MAST4	-0,332405896	-2,391863529	2,059457633
207451_at	NKX2-8	-0,332405896	-2,391863529	2,059457633
211671_s_at	NR3C1	5,56221414	3,502959177	2,059254962
212236_x_at	JUP /// KRT17	1,287793882	-0,771340337	2,059134219
213836_s_at	WIPI1	3,137734155	1,078619386	2,059114769
218498_s_at	ERO1L	3,768156719	1,709047448	2,059109271
212516_at	ARAP1	1,640193838	-0,418248858	2,058442696
207367_at	ATP12A	2,096248267	0,03784737	2,058400897
208762_at	SUMO1	1,671397663	-0,386896102	2,058293765
218599_at	REC8	1,000459215	-1,057301851	2,057761065
203827_at	WIPI1	2,739067176	0,681874787	2,057192389
214434_at	HSPA12A	0,39105295	-1,665791776	2,056844726
204866_at	PHF16	3,642593015	1,586121802	2,056471213
236482_at	-	0,244681185	-1,811780055	2,05646124
234271_at	OTOP2	0,244681185	-1,811780055	2,05646124
205056_s_at	GPR162	0,244681185	-1,811780055	2,05646124
243574_at	-	-0,609040214	-2,665156778	2,056116564
209112_at	CDKN1B	5,246614709	3,191011385	2,055603324
226954_at	UBE2R2	3,362258367	1,306903969	2,055354398
231772_x_at	CENPH	4,324702656	2,269637658	2,055064998
241372_at	ZC3H6	0,820160788	-1,234835326	2,054996114
224681_at	GNA12	4,087244545	2,03313004	2,054114505
226861_at	ASB8	3,748268542	1,694547353	2,053721189
237033_at	FAM159A	-0,088974936	-2,142286822	2,053311886
219044_at	THNSL2	-0,088974936	-2,142286822	2,053311886
229255_x_at	-	1,20396005	-0,84928999	2,05325004
223971_at	OR2A20P /// OR2A	-0,043925712	-2,09678811	2,052862398
235734_at	ARFGAP2	-0,043925712	-2,09678811	2,052862398
225369_at	ESAM	-0,043925712	-2,09678811	2,052862398
211372_s_at	IL1R2	-0,043925712	-2,09678811	2,052862398
1557225_at	ASPG	-0,551284523	-2,603471267	2,052186744
216693_x_at	HDGFRP3	3,828857041	1,777135134	2,051721907
1559266_s_at	SKIDA1	-0,135065865	-2,186086603	2,051020738
235485_at	WDR44	-0,135065865	-2,186086603	2,051020738
236838_at	SRCIN1	-0,135065865	-2,186086603	2,051020738
1555980_a_at	LOC100130417	-0,135065865	-2,186086603	2,051020738
205950_s_at	CA1	-0,135065865	-2,186086603	2,051020738
205792_at	WISP2	0,001114523	-2,049747196	2,050861719

238173_at	TCEA2	0,001114523	-2,049747196	2,050861719
233381_at	RUFY1	0,001114523	-2,049747196	2,050861719
216318_at	IGHA1	0,001114523	-2,049747196	2,050861719
238440_at	CLYBL	3,071179046	1,020514937	2,05066411
230041_at	LOC100505639	0,677745787	-1,372734086	2,050479873
204993_at	GNAZ	0,677745787	-1,372734086	2,050479873
203695_s_at	DFNA5	1,960819106	-0,089635713	2,050454819
224519_at	LOC100132167	1,160996507	-0,889222211	2,050218718
214403_x_at	SPDEF	1,493647653	-0,554696666	2,048344319
237423_at	RSPO4	-0,180990326	-2,229233437	2,048243112
207448_at	POFUT2	-0,180990326	-2,229233437	2,048243112
213966_at	HMG20B	-0,180990326	-2,229233437	2,048243112
215949_x_at	IGHM /// LOC1006	1,116462765	-0,931539147	2,048001912
236594_at	LLGL1	2,545254728	0,497424714	2,047830013
37549_g_at	BBS9	1,936562011	-0,111094779	2,04765679
218205_s_at	MKNK2	7,737734381	5,690854598	2,046879784
215718_s_at	PHF3	1,561816652	-0,484558493	2,046375146
206313_at	HLA-DOA	1,528073958	-0,518232988	2,046306947
200927_s_at	RAB14	4,565392441	2,519368159	2,046024282
243083_at	LOC100287704 ///	0,282358733	-1,763664074	2,046022807
223517_at	FBXO44	0,282358733	-1,763664074	2,046022807
207494_s_at	ZNF76	2,560606634	0,515457602	2,045149032
201883_s_at	B4GALT1	3,265594257	1,220470658	2,045123599
217775_s_at	RDH11	4,876721708	2,832036229	2,044685479
232048_at	FAM76B	1,421391637	-0,623254098	2,044645736
206250_x_at	AVPR1A	0,524545436	-1,520022123	2,04456756
1562261_at	AMZ1	0,524545436	-1,520022123	2,04456756
210454_s_at	KCNJ6	-0,384193355	-2,4284334	2,044240045
221238_at	HMGN5	1,071336699	-0,972893339	2,044230037
220943_s_at	C2orf56	2,523100136	0,479089184	2,044010952
1559459_at	LOC613266	-0,49284695	-2,536339282	2,043492331
205833_s_at	PART1	-0,49284695	-2,536339282	2,043492331
219410_at	TMEM45A	0,08619576	-1,956681069	2,042876829
221377_s_at	RBPJL	0,08619576	-1,956681069	2,042876829
219075_at	YIPF2	0,08619576	-1,956681069	2,042876829
206986_at	FGF18	0,08619576	-1,956681069	2,042876829
238104_at	-	1,345611839	-0,697182596	2,042794435
233328_x_at	SLC17A9	0,425036312	-1,616924751	2,041961064
203005_at	LTBR	0,618277321	-1,422257026	2,040534347
234318_x_at	NDOR1	0,618277321	-1,422257026	2,040534347
223548_at	SWT1	2,053550345	0,013190398	2,040359947
205766_at	TCAP	1,024284941	-1,015398016	2,039682957
202340_x_at	NR4A1	1,024284941	-1,015398016	2,039682957
1562017_at	LOC100130654	-0,230432956	-2,27001641	2,039583454
237563_s_at	-	3,228532861	1,189078962	2,039453899
213932_x_at	HLA-A	7,966618222	5,928029655	2,038588567
222108_at	AMIGO2	1,267146969	-0,771340337	2,038487306
1570362_at	-	0,847529938	-1,19085694	2,038386878
229631_at	DNHD1	1,225123479	-0,811769547	2,036893026
228301_x_at	NDUFB10	5,143322491	3,106874588	2,036447903

226763_at	SESTD1	3,636292808	1,601076277	2,035216532
226050_at	TMCO3	3,636292808	1,601076277	2,035216532
222444_at	ARMCX3	2,836219929	0,80114751	2,035072419
226851_at	LYPLAL1	5,092485786	3,05768517	2,034800616
203156_at	AKAP11	3,9635104	1,929092307	2,034418093
216356_x_at	BAIAP3	0,97663406	-1,057301851	2,033935911
201924_at	AFF1	4,355700522	2,323616375	2,032084147
238367_s_at	C1orf228	-0,280273599	-2,312054429	2,03178083
204862_s_at	NME3	3,086998005	1,055317091	2,031680913
1559565_x_at	-	0,167727503	-1,862269442	2,029996945
217254_s_at	EPO	0,167727503	-1,862269442	2,029996945
217224_at	-	0,167727503	-1,862269442	2,029996945
237362_at	ZFP41	0,167727503	-1,862269442	2,029996945
203312_x_at	ARF6	4,270249493	2,24081821	2,029431283
1729_at	TRADD	3,790181405	1,760872722	2,029308683
231069_at	-	1,440265662	-0,589001171	2,029266833
228125_at	ZSCAN30	1,440265662	-0,589001171	2,029266833
219994_at	APBB1IP	-0,437449947	-2,466349414	2,028899467
244089_at	-	-0,437449947	-2,466349414	2,028899467
235961_at	GPR161	0,556428218	-1,471833228	2,028261446
216236_s_at	SLC2A14 /// SLC2A	3,792209911	1,76396664	2,028243272
237159_x_at	AP1S3	0,458905032	-1,568385657	2,027290688
210025_s_at	CARD10	0,458905032	-1,568385657	2,027290688
241669_x_at	PRKD2	0,458905032	-1,568385657	2,027290688
211611_s_at	ATF6B /// TNXB	0,926325262	-1,100943374	2,027268636
210854_x_at	SLC6A8	0,926325262	-1,100943374	2,027268636
242557_at	ZNRD1-AS1	0,791908897	-1,234835326	2,026744224
204254_s_at	VDR	3,081633533	1,055317091	2,026316441
238866_at	C19orf68	1,402495885	-0,623254098	2,025749983
210647_x_at	PLA2G6	1,402495885	-0,623254098	2,025749983
208116_s_at	MAN1A1	2,523100136	0,497424714	2,025675422
209201_x_at	CXCR4	5,899408692	3,874467807	2,024940885
238087_at	RTCA	2,179708195	0,155747281	2,023960914
243377_at	-	1,701542258	-0,322340048	2,023882305
229414_at	PITPNC1	1,326463531	-0,697182596	2,023646127
205775_at	FAM50B	3,300874993	1,278654284	2,022220709
236350_at	-	0,648195588	-1,372734086	2,020929674
208333_at	LHX5	0,648195588	-1,372734086	2,020929674
242145_at	-	0,648195588	-1,372734086	2,020929674
205458_at	MC1R	0,648195588	-1,372734086	2,020929674
202135_s_at	ACTR1B	4,484674718	2,465016194	2,019658524
233580_at	-	0,20740896	-1,811780055	2,019189015
1552917_at	IL29	0,873789652	-1,145057014	2,018846666
1561667_at	-	0,735999505	-1,282520722	2,018520227
228364_at	ZNF784	0,735999505	-1,282520722	2,018520227
223990_at	C4orf17	-0,332405896	-2,350444759	2,018038863
222025_s_at	OPLAH	-0,332405896	-2,350444759	2,018038863
219032_x_at	OPN3	4,200383271	2,182711118	2,017672153
227864_s_at	FAM125A	2,817838865	0,80114751	2,016691355
219401_at	XYLT2	1,20396005	-0,811769547	2,015729597

64486_at	CORO1B	4,110977379	2,097156166	2,013821212
200075_s_at	GUK1	5,822968482	3,809801648	2,013166834
216248_s_at	NR4A2	1,5944803	-0,418248858	2,012729158
218425_at	RNF216	1,528073958	-0,484558493	2,012632452
1556024_at	EME2	0,491906512	-1,520022123	2,011928635
234496_x_at	NYX	0,491906512	-1,520022123	2,011928635
228714_at	-	2,99543179	0,98381571	2,01161608
203909_at	SLC9A6	4,09423887	2,082919929	2,011318941
1553304_at	LSM14B	0,820160788	-1,19085694	2,011017728
210896_s_at	ASPH	0,820160788	-1,19085694	2,011017728
1552311_a_at	RAX2	0,820160788	-1,19085694	2,011017728
36553_at	ASMTL	4,301428548	2,290478717	2,010949831
230388_s_at	KANSL1-AS1	1,65554524	-0,354781582	2,010326821
205784_x_at	ARVCF	1,65554524	-0,354781582	2,010326821
238473_at	LOC100216545	0,58788177	-1,422257026	2,010138797
217165_x_at	MT1F	4,025386557	2,016139502	2,009247055
234340_at	-	-0,49284695	-2,501359113	2,008512163
216336_x_at	LOC100505584 ///	3,931608669	1,923216608	2,008392061
206206_at	CD180	0,244681185	-1,763664074	2,00834526
228669_x_at	PARP10	0,244681185	-1,763664074	2,00834526
224651_at	CCNY	3,228532861	1,220470658	2,008062203
205845_at	CACNA1H	0,39105295	-1,616924751	2,007977702
210263_at	KCNF1	0,39105295	-1,616924751	2,007977702
226190_at	MAP3K13	3,876052392	1,868130962	2,00792143
211540_s_at	RB1	-0,088974936	-2,09678811	2,007813174
228923_at	S100A6	-0,384193355	-2,391863529	2,007670174
223568_s_at	PPAPDC1B	2,719381633	0,711934445	2,007447188
233453_at	-	-0,135065865	-2,142286822	2,007220957
217074_at	SMOX	-0,135065865	-2,142286822	2,007220957
238150_at	-	1,345611839	-0,661178575	2,006790413
212911_at	DNAJC16	2,312738346	0,306454867	2,006283479
210245_at	ABCC8	-0,043925712	-2,049747196	2,005821484
217723_x_at	-	1,116462765	-0,889222211	2,005684975
201308_s_at	40787	-0,180990326	-2,186086603	2,005096277
234910_at	RASAL2	-0,180990326	-2,186086603	2,005096277
236788_at	-	-0,180990326	-2,186086603	2,005096277
228390_at	RAB30	-0,180990326	-2,186086603	2,005096277
204821_at	BTN3A3	3,551632175	1,547376668	2,004255507
217908_s_at	DCAF6	4,204964809	2,20251271	2,0024521
204269_at	PIM2	3,309267982	1,306903969	2,002364012
240091_at	PSMA8	0,045517965	-1,956681069	2,002199034
1561685_a_at	LOC441178	0,045517965	-1,956681069	2,002199034
1553517_at	FERD3L	0,045517965	-1,956681069	2,002199034
216394_x_at	-	0,045517965	-1,956681069	2,002199034
226773_at	-	3,09217775	1,090640407	2,001537343
218418_s_at	KANK2	2,179708195	0,178191865	2,00151633
1558972_s_at	THEMIS	4,744801376	2,743433212	2,001368164
201041_s_at	DUSP1	2,348178434	0,346964736	2,001213699
235126_at	FLVCR1-AS1	3,804904984	1,804519951	2,000385034
211760_s_at	VAMP4	1,910557155	-0,089635713	2,000192868

221833_at	LOC100507577 ///	1,936380191	-0,063377083	1,999757274
230178_s_at	ELP2	1,936380191	-0,063377083	1,999757274
216500_at	-	0,764143511	-1,234835326	1,998978837
228722_at	PRMT2	2,582572508	0,583607503	1,998965005
212654_at	TPM2	-0,230432956	-2,229233437	1,998800482
216041_x_at	GRN	3,828857041	1,830129603	1,998727437
223327_x_at	AGSK1 /// LOC727	2,664625637	0,666486137	1,9981395
233601_at	-	0,08619576	-1,911206569	1,997402329
239268_at	NDUFS1	0,08619576	-1,911206569	1,997402329
242589_x_at	-	1,225123479	-0,771340337	1,996463816
1569961_at	-	0,524545436	-1,471833228	1,996378664
208234_x_at	FGFR2	0,524545436	-1,471833228	1,996378664
209843_s_at	SOX10	0,524545436	-1,471833228	1,996378664
219549_s_at	RTN3	5,708143186	3,713038696	1,99510449
209280_at	MRC2	1,440265662	-0,554696666	1,994962328
209415_at	FZR1	2,127965541	0,133104519	1,994861022
228511_s_at	-	-0,609040214	-2,603471267	1,994431053
229792_at	KLHL17	1,476175815	-0,518232988	1,994408803
229851_s_at	C11orf54	3,668220666	1,674071917	1,99414875
209606_at	CYTIP	4,924288109	2,930238085	1,994050025
214012_at	ERAP1	3,300874993	1,306903969	1,993971024
227420_at	TNFAIP8L1	3,233229368	1,239268196	1,993961172
209235_at	CLCN7	1,671397663	-0,322340048	1,993737711
243364_at	AUTS2	0,425036312	-1,568385657	1,993421969
239796_x_at	TIRAP	0,425036312	-1,568385657	1,993421969
216340_s_at	CYP2A7P1	0,425036312	-1,568385657	1,993421969
238639_x_at	-	0,425036312	-1,568385657	1,993421969
205390_s_at	ANK1	-0,730013898	-2,723114724	1,993100826
226843_s_at	PAPD5	4,091994743	2,099079406	1,992915337
35626_at	SGSH	1,403434313	-0,588568186	1,9920025
205173_x_at	CD58	5,974528004	3,983024602	1,991503402
240002_at	LOC389634	0,618277321	-1,372734086	1,991011407
202674_s_at	LMO7	-0,437449947	-2,4284334	1,990983453
244127_at	-	-0,437449947	-2,4284334	1,990983453
207900_at	CCL17	-0,437449947	-2,4284334	1,990983453
1559103_s_at	-	0,707657549	-1,282520722	1,990178271
231987_at	MIR143HG	0,707657549	-1,282520722	1,990178271
211259_s_at	BMP7	0,707657549	-1,282520722	1,990178271
240311_at	NANOS3	0,707657549	-1,282520722	1,990178271
228278_at	NFIX	0,707657549	-1,282520722	1,990178271
209730_at	SEMA3F	0,707657549	-1,282520722	1,990178271
221497_x_at	EGLN1	3,370643409	1,380552339	1,99009107
213157_s_at	LOC339166 /// WS	0,127732831	-1,862269442	1,990002272
227783_at	CCDC57	2,732422196	0,742671819	1,989750377
213381_at	VSTM4	-0,280273599	-2,27001641	1,989742811
1555888_at	UBR5	1,843966654	-0,144765583	1,988732237
209581_at	PLA2G16	3,166767593	1,178100917	1,988666676
222957_at	NEU4	1,139057614	-0,84928999	1,988347604
226150_at	PPAPDC1B	4,070902947	2,082919929	1,987983019
212614_at	ARID5B	2,410823487	0,424693306	1,986130181

212258_s_at	SMARCA2	0,319287178	-1,665791776	1,985078954
223031_s_at	TRAF7	2,118167848	0,133104519	1,985063329
204994_at	MX2	1,287793882	-0,697182596	1,984976479
226566_at	TRIM11	3,309267982	1,324949741	1,984318241
202934_at	HK2	3,152091004	1,167919222	1,984171782
235745_at	ERN1	1,094118704	-0,889222211	1,983340915
231062_at	DOCK9-AS2	0,791908897	-1,19085694	1,982765837
204076_at	ENTPD4	4,624674869	2,64347734	1,981197528
219852_s_at	MORN1	1,246401134	-0,734355396	1,980756529
1553652_a_at	C18orf54	-0,332405896	-2,312054429	1,979648533
203191_at	ABCB6	-0,332405896	-2,312054429	1,979648533
207309_at	NOS1	0,167727503	-1,811780055	1,979507558
228234_at	TICAM2 /// TMED7	2,476788561	0,497424714	1,979363846
230480_at	PIWIL4	0,458905032	-1,520022123	1,978927155
223549_s_at	ESPN	1,047341799	-0,931539147	1,978880946
219246_s_at	OGFOD2	1,047341799	-0,931539147	1,978880946
219144_at	DUSP26	0,556428218	-1,422257026	1,978685244
209218_at	SQLE	5,994196562	4,016881012	1,97731555
210512_s_at	VEGFA	4,0992582	2,121960601	1,9772976
227127_at	MUSTN1 /// TMEM	1,421391637	-0,554696666	1,976088303
221240_s_at	B3GNT4	1,421391637	-0,554696666	1,976088303
243976_at	-	1,20396005	-0,771340337	1,975300387
35265_at	FXR2	2,417818079	0,442710786	1,975107293
206686_at	PDK1	1,857989965	-0,116767475	1,97475744
226608_at	C16orf87	3,489810047	1,516067292	1,973742756
202381_at	ADAM9	4,125707946	2,151985997	1,973721949
216658_at	-	-0,49284695	-2,466349414	1,973502464
205847_at	PRSS22	1,000459215	-0,972893339	1,973352553
219323_s_at	IL18BP	1,000459215	-0,972893339	1,973352553
228724_at	TTLL7	1,160996507	-0,811769547	1,972766054
1553507_a_at	GPR6	0,355751005	-1,616924751	1,972675756
218208_at	PQLC1	2,908690075	0,936232181	1,972457894
213617_s_at	TPGS2	5,190127413	3,217691783	1,97243563
209500_x_at	TNFSF12 /// TNFSF	0,20740896	-1,763664074	1,971073034
228090_at	NMNAT3	0,20740896	-1,763664074	1,971073034
220077_at	CCDC134	0,20740896	-1,763664074	1,971073034
203965_at	USP20	3,127551022	1,156599976	1,970951045
217743_s_at	TMEM30A	4,343795594	2,373249966	1,970545628
221002_s_at	TSPAN14	2,007220798	0,03784737	1,969373428
208527_x_at	HIST1H2BC /// HIS	4,12127125	2,151985997	1,969285253
1564027_a_at	FAM115C	1,307201325	-0,661178575	1,9683799
213296_at	RER1	2,649978302	0,681874787	1,968103516
210508_s_at	KCNQ2	0,95222556	-1,015398016	1,967623576
244177_at	-	3,03433073	1,067365565	1,966965165
211643_x_at	IGK@ /// IGKC	-0,384193355	-2,350444759	1,966251404
208215_x_at	DRD4	-0,66934516	-2,635549085	1,966203925
223268_at	C11orf54	4,158437147	2,192329358	1,966107789
223390_at	C9orf37	1,116462765	-0,84928999	1,965752755
209326_at	SLC35A2	3,382641505	1,417157926	1,965483579
237203_at	LOC100506546	0,820160788	-1,145057014	1,965217802

217473_x_at	SLC11A1	1,267146969	-0,697182596	1,964329566
564_at	GNA11	1,306455853	-0,657548593	1,964004445
229291_at	-	0,491906512	-1,471833228	1,963739739
235569_at	VPS37D	0,491906512	-1,471833228	1,963739739
227169_at	DNAJC18	0,491906512	-1,471833228	1,963739739
209420_s_at	SMPD1	1,544940605	-0,418248858	1,963189463
223686_at	TPK1	1,510970097	-0,45121326	1,962183357
232051_at	CCDC102A	1,671397663	-0,290694975	1,962092638
1552587_at	CNBD1	-0,135065865	-2,09678811	1,961722245
223855_s_at	MCHR1	-0,135065865	-2,09678811	1,961722245
240604_at	ERI2	-0,135065865	-2,09678811	1,961722245
214063_s_at	TF	-0,135065865	-2,09678811	1,961722245
207741_x_at	TPSAB1	-0,135065865	-2,09678811	1,961722245
242941_x_at	TBX1	-0,135065865	-2,09678811	1,961722245
213451_x_at	TNXA /// TNXB	-0,135065865	-2,09678811	1,961722245
231498_at	-	-0,609040214	-2,570522741	1,961482528
209010_s_at	TRIO	-0,609040214	-2,570522741	1,961482528
209576_at	GNAI1	-0,609040214	-2,570522741	1,961482528
229029_at	CAMK4	3,697863752	1,736450458	1,961413294
1554652_s_at	MAST4	-0,180990326	-2,142286822	1,961296496
1561853_a_at	IL23R	-0,180990326	-2,142286822	1,961296496
224003_at	NCRNA00185 /// T	-0,180990326	-2,142286822	1,961296496
237533_at	-	-0,180990326	-2,142286822	1,961296496
206027_at	S100A3	-0,043925712	-2,005029581	1,961103869
230297_x_at	SYNGAP1	-0,043925712	-2,005029581	1,961103869
1559667_at	-	-0,043925712	-2,005029581	1,961103869
200872_at	S100A10	7,789983917	5,829166898	1,960817018
236449_at	CSTB	-0,088974936	-2,049747196	1,96077226
234366_x_at	CKAP2 /// IGLC1	-0,088974936	-2,049747196	1,96077226
235934_at	NAPA-AS1	0,58788177	-1,372734086	1,960615856
221237_s_at	OSBP2	1,071336699	-0,889222211	1,960558909
209071_s_at	RGS5	0,677745787	-1,282520722	1,960266509
203687_at	CX3CL1	0,677745787	-1,282520722	1,960266509
239608_at	LPCAT4	0,244681185	-1,715576125	1,960257311
231117_at	FAM181A	0,244681185	-1,715576125	1,960257311
231328_s_at	RASGRP4	0,244681185	-1,715576125	1,960257311
202222_s_at	DES	0,244681185	-1,715576125	1,960257311
233016_at	LOC100506546	0,39105295	-1,568385657	1,959438607
231276_at	-	0,39105295	-1,568385657	1,959438607
225057_at	SLC15A4	4,790575561	2,832036229	1,958539332
209202_s_at	EXTL3	1,440265662	-0,518232988	1,958498651
226055_at	ARRDC2	1,440265662	-0,518232988	1,958498651
237078_at	-	0,001114523	-1,956681069	1,957795592
207413_s_at	SCN5A	0,001114523	-1,956681069	1,957795592
232534_at	LIN37	0,001114523	-1,956681069	1,957795592
213702_x_at	ASAH1	5,409254399	3,451657924	1,957596476
223144_s_at	AKIRIN2	4,52000033	2,562621495	1,957378835
207154_at	DIO3	0,900065578	-1,057301851	1,957367429
1554519_at	CD80	0,900065578	-1,057301851	1,957367429
244500_s_at	EVI5L	0,045517965	-1,911206569	1,956724534

1553592_x_at	BCRP3	0,045517965	-1,911206569	1,956724534
220758_s_at	ROBO4	0,045517965	-1,911206569	1,956724534
205616_at	GALNT8 /// KCNA6	0,045517965	-1,911206569	1,956724534
222596_s_at	LGR4	0,045517965	-1,911206569	1,956724534
1558115_at	RECK	1,024284941	-0,931539147	1,955824088
221141_x_at	EPN1	1,024284941	-0,931539147	1,955824088
220024_s_at	PRX	1,024284941	-0,931539147	1,955824088
205460_at	NPAS2	-0,230432956	-2,186086603	1,955653647
215270_at	LFNG	-0,230432956	-2,186086603	1,955653647
204200_s_at	PDGFB	-0,230432956	-2,186086603	1,955653647
221387_at	NPFFR1	0,764143511	-1,19085694	1,955000451
219270_at	CHAC1	0,764143511	-1,19085694	1,955000451
214105_at	SOCS3	-0,437449947	-2,391863529	1,954413581
1561433_at	CCDC74B-AS1	-0,437449947	-2,391863529	1,954413581
205180_s_at	ADAM8	2,339581693	0,385582632	1,953999061
209442_x_at	ANK3	1,364555912	-0,589001171	1,953557083
200700_s_at	KDELR2	4,996753949	3,043944324	1,952809624
209215_at	MFSD10	2,752615529	0,80114751	1,951468019
229971_at	GPR114	3,331722254	1,380552339	1,951169915
209035_at	MDK	1,139057614	-0,811769547	1,950827161
202644_s_at	TNFAIP3	6,410459479	4,460148736	1,950310743
225775_at	TSPAN33	3,786189171	1,836262316	1,949926855
230160_x_at	-	0,97663406	-0,972893339	1,949527398
229318_at	-	1,5944803	-0,354781582	1,949261881
202572_s_at	DLGAP4	1,287793882	-0,661178575	1,948972457
238514_at	TMEM25	-0,280273599	-2,229233437	1,948959838
235066_at	MAP4	-0,280273599	-2,229233437	1,948959838
242023_at	-	-0,280273599	-2,229233437	1,948959838
240441_at	FAM99B	-0,280273599	-2,229233437	1,948959838
220638_s_at	CBLC	-0,280273599	-2,229233437	1,948959838
1562016_at	-	-0,280273599	-2,229233437	1,948959838
217352_at	-	0,847529938	-1,100943374	1,948473312
227090_at	PHF21A	0,847529938	-1,100943374	1,948473312
235933_at	FLJ32154	0,08619576	-1,862269442	1,948465201
243845_at	HSPD1	0,08619576	-1,862269442	1,948465201
232433_at	KIAA1683	0,08619576	-1,862269442	1,948465201
231978_at	TPCN2	0,08619576	-1,862269442	1,948465201
244209_at	FLJ41350	0,282358733	-1,665791776	1,948150509
228687_at	ZNF777	0,282358733	-1,665791776	1,948150509
217573_at	GRIN2C	0,282358733	-1,665791776	1,948150509
232602_at	WFDC3	0,524545436	-1,422257026	1,946802463
205494_at	ZNF821	0,524545436	-1,422257026	1,946802463
205586_x_at	VGF	0,524545436	-1,422257026	1,946802463
212562_s_at	CTSZ	0,524545436	-1,422257026	1,946802463
224898_at	WDR26	2,031158928	0,085534992	1,945623935
212457_at	TFE3	3,886513476	1,941115499	1,945397977
209931_s_at	FKBP1B /// MFSD2	0,425036312	-1,520022123	1,945058436
237242_at	LOC126536	0,425036312	-1,520022123	1,945058436
220163_s_at	HR	0,425036312	-1,520022123	1,945058436
217803_at	GOLPH3	5,002497265	3,05768517	1,944812095

1558784_at	LOC100133089	1,246401134	-0,697182596	1,94358373
201153_s_at	MBNL1	7,144546765	5,201642861	1,942903905
203027_s_at	MVD	1,458255986	-0,484558493	1,94281448
205145_s_at	MYL5	1,458255986	-0,484558493	1,94281448
202756_s_at	GPC1	0,707657549	-1,234835326	1,942492875
235519_at	FOXK1	0,707657549	-1,234835326	1,942492875
218337_at	FAM160B2	2,798156197	0,85655369	1,941602507
204927_at	RASSF7	2,698955954	0,757638486	1,941317468
209361_s_at	PCBP4	2,096248267	0,155747281	1,940500987
210786_s_at	FLI1	3,096793374	1,156599976	1,940193398
214429_at	MTMR6	4,894325047	2,954216214	1,940108834
232480_at	MIRLET7BHG	0,127732831	-1,811780055	1,939512885
229432_at	NAGS	0,127732831	-1,811780055	1,939512885
205003_at	DOCK4	0,127732831	-1,811780055	1,939512885
230704_s_at	ITGB4	0,127732831	-1,811780055	1,939512885
227665_at	-	2,725848647	0,786391549	1,939457098
205196_s_at	AP1S1	3,67542249	1,736450458	1,938972032
239210_at	-	-0,332405896	-2,27001641	1,937610514
230050_at	NACC2	-0,332405896	-2,27001641	1,937610514
223452_s_at	ATL3	2,537731447	0,600385088	1,937346359
212902_at	SEC24A	3,147261986	1,209973561	1,937288424
242587_at	SLC9A9	0,791908897	-1,145057014	1,936965912
234435_at	-	0,319287178	-1,616924751	1,936211929
1558420_at	C14orf180	0,319287178	-1,616924751	1,936211929
214386_at	LOC100653164	0,319287178	-1,616924751	1,936211929
227428_at	GABPA	3,071179046	1,135235995	1,935943051
223318_s_at	ALKBH7	5,250728521	3,315052527	1,935675995
1569099_at	-	-0,49284695	-2,4284334	1,93558645
237360_at	ACTRT2	-0,49284695	-2,4284334	1,93558645
234694_at	CNTROB	-0,49284695	-2,4284334	1,93558645
209526_s_at	HDGFRP3	3,858118781	1,923216608	1,934902173
234485_at	-	1,871521385	-0,063377083	1,934898468
227138_at	CRTAP	1,97249974	0,03784737	1,934652369
220319_s_at	MYLIP	2,817838865	0,883374248	1,934464616
202630_at	APPBP2	2,989672611	1,055317091	1,93435552
235749_at	UGGT2	-0,66934516	-2,603471267	1,934126107
238381_x_at	-	-0,66934516	-2,603471267	1,934126107
209090_s_at	SH3GLB1	4,866513045	2,933477664	1,93303538
225896_at	MPRIP	1,701542258	-0,231045907	1,932588164
1564317_at	-	0,167727503	-1,763664074	1,931391578
211121_s_at	DOK1	2,878891456	0,947673886	1,93121757
1569383_s_at	ZFYVE28	0,873789652	-1,057301851	1,931091503
235620_x_at	ZMYM5	0,873789652	-1,057301851	1,931091503
234918_at	GLTSCR2	0,873789652	-1,057301851	1,931091503
226530_at	BMF	0,873789652	-1,057301851	1,931091503
226878_at	HLA-DOA	2,914752575	0,98381571	1,930936866
238324_at	GATAD2A	0,458905032	-1,471833228	1,930738259
200785_s_at	LRP1	0,458905032	-1,471833228	1,930738259
240294_at	HIPK2	0,458905032	-1,471833228	1,930738259
204916_at	RAMP1	0,648195588	-1,282520722	1,93071631

234977_at	ZADH2	1,307201325	-0,623254098	1,930455424
225796_at	PXK	3,639446351	1,709047448	1,930398903
1556285_s_at	PPA2	6,384386252	4,454638116	1,929748136
211813_x_at	DCN	0,556428218	-1,372734086	1,929162304
230396_at	SAMM50	0,556428218	-1,372734086	1,929162304
216875_x_at	HAB1	0,556428218	-1,372734086	1,929162304
218247_s_at	MEX3C	4,080162946	2,151985997	1,928176949
212830_at	MEGF9	4,016622037	2,088449486	1,928172551
207574_s_at	GADD45B	3,758020365	1,830129603	1,927890762
236673_at	TIFAB	-0,384193355	-2,312054429	1,927861074
211363_s_at	MTAP	-0,384193355	-2,312054429	1,927861074
216157_at	-	-0,384193355	-2,312054429	1,927861074
243623_at	SLC1A7	-0,384193355	-2,312054429	1,927861074
222210_at	KIAA0195	-0,609040214	-2,536339282	1,927299068
221109_at	LOC100506571	0,735999505	-1,19085694	1,926856445
1557458_s_at	SHB	0,735999505	-1,19085694	1,926856445
208251_at	KCNC4	0,735999505	-1,19085694	1,926856445
202544_at	GMFB	4,767552061	2,841176366	1,926375695
241451_s_at	-	0,95222556	-0,972893339	1,925118899
235352_at	MR1	2,33082239	0,405752839	1,925069551
205047_s_at	ASNS	6,474145212	4,549092012	1,925053199
202504_at	TRIM29	1,440265662	-0,484558493	1,924824156
229743_at	ZNF438	1,440265662	-0,484558493	1,924824156
212136_at	ATP2B4	3,996262843	2,071691486	1,924571358
213749_at	MASP1	0,355751005	-1,568385657	1,924136662
211577_s_at	IGF1	0,355751005	-1,568385657	1,924136662
206707_x_at	FAM65B	2,752615529	0,829309537	1,923305992
232160_s_at	TNIP2	3,374552989	1,451507543	1,923045446
229649_at	NRXN3	0,20740896	-1,715576125	1,922985085
227191_at	ITFG1	0,20740896	-1,715576125	1,922985085
242543_at	SH2D6	0,20740896	-1,715576125	1,922985085
230772_at	HNF4A	0,20740896	-1,715576125	1,922985085
234751_s_at	NLGN3	0,20740896	-1,715576125	1,922985085
1554579_a_at	MYO18B	2,229377375	0,306454867	1,922922508
230136_at	LOC400099	1,225123479	-0,697182596	1,922306075
204929_s_at	VAMP5	1,225123479	-0,697182596	1,922306075
219858_s_at	MFSD6	1,983901861	0,061773582	1,922128279
222545_s_at	C10orf57	2,38349585	0,461385738	1,922110113
242668_x_at	SUN5	1,88491011	-0,036543687	1,921453797
219888_at	SPAG4	0,820160788	-1,100943374	1,921104162
242386_x_at	-	0,820160788	-1,100943374	1,921104162
235705_at	-	0,820160788	-1,100943374	1,921104162
212887_at	SEC23A	3,807915835	1,887158627	1,920757208
215372_x_at	-	1,402495885	-0,518232988	1,920728873
225891_at	TPRN	1,775035811	-0,144765583	1,919801394
210232_at	CDC42	2,074841895	0,155747281	1,919094615
1554966_a_at	FILIP1L	3,317909135	1,399283506	1,91862563
225984_at	PRKAA1	2,552836808	0,634301128	1,91853568
229493_at	HOXD-AS2	-0,088974936	-2,005029581	1,916054645
239528_at	PROM2	-0,088974936	-2,005029581	1,916054645

214973_x_at	IGHD	-0,088974936	-2,005029581	1,916054645
233849_s_at	ARHGAP5	3,214269085	1,298349073	1,915920012
207930_at	LCN1	-0,180990326	-2,09678811	1,915797784
1552670_a_at	PPP1R3B	-0,180990326	-2,09678811	1,915797784
242148_at	BCL6B	0,900065578	-1,015398016	1,915463594
235317_at	LOC284454	0,900065578	-1,015398016	1,915463594
238158_at	MEIG1	2,159837409	0,244646697	1,915190712
1560765_a_at	-	-0,551284523	-2,466349414	1,915064891
222426_at	MAPKAP1	2,339581693	0,424693306	1,914888387
206273_at	SLMO1	-0,135065865	-2,049747196	1,914681331
1560151_x_at	SLC29A2	-0,135065865	-2,049747196	1,914681331
229619_at	FOLR2	-0,135065865	-2,049747196	1,914681331
1570167_at	-	-0,135065865	-2,049747196	1,914681331
215185_at	LOC100128077 ///	0,491906512	-1,422257026	1,914163538
238099_at	-	0,491906512	-1,422257026	1,914163538
236807_at	-	0,491906512	-1,422257026	1,914163538
228806_at	RORC	0,491906512	-1,422257026	1,914163538
227630_at	PPP2R5E	2,824160383	0,910483921	1,913676462
212586_at	CAST	5,208677201	3,295596077	1,913081124
234231_at	CASP16	-0,437449947	-2,350444759	1,912994812
236447_at	-	-0,043925712	-1,956681069	1,912755358
211322_s_at	SARDH	-0,043925712	-1,956681069	1,912755358
236457_at	GTDC1	-0,043925712	-1,956681069	1,912755358
213466_at	RAB40C	0,677745787	-1,234835326	1,912581113
224392_s_at	OPN3	2,698955954	0,786391549	1,912564405
220052_s_at	TINF2	2,698955954	0,786391549	1,912564405
227319_at	KLHL36	4,537242248	2,624682607	1,912559642
215045_at	CELF3	0,001114523	-1,911206569	1,912321092
1569256_a_at	FAM43B	0,001114523	-1,911206569	1,912321092
1564319_at	FAM71E2	0,001114523	-1,911206569	1,912321092
218876_at	TPPP3	0,001114523	-1,911206569	1,912321092
221766_s_at	FAM46A	3,171328694	1,259081178	1,912247516
231253_at	SPPL2C	-0,230432956	-2,142286822	1,911853866
224122_at	-	-0,230432956	-2,142286822	1,911853866
224291_at	CACNG6	0,39105295	-1,520022123	1,911075074
1557226_a_at	ASPG	0,39105295	-1,520022123	1,911075074
241927_x_at	-	0,39105295	-1,520022123	1,911075074
210043_at	FRMD8	1,287793882	-0,623254098	1,911047981
233898_s_at	FGFR1OP2	4,64066268	2,730083062	1,910579619
211745_x_at	HBA1 /// HBA2	0,244681185	-1,665791776	1,910472961
234943_at	LOC400927	0,244681185	-1,665791776	1,910472961
237024_at	C3orf45	0,244681185	-1,665791776	1,910472961
1564208_x_at	FLJ35390	3,533714224	1,623485524	1,910228701
226706_at	FLJ23867 /// QSOX	0,764143511	-1,145057014	1,909200525
225970_at	DDHD1	3,925077605	2,016139502	1,908938102
203168_at	ATF6B	1,871521385	-0,036543687	1,908065072
214542_x_at	HIST1H2AG /// HIS	1,871521385	-0,036543687	1,908065072
240786_at	NOTCH4	0,045517965	-1,862269442	1,907787406
206499_s_at	RCC1	3,6787904	1,771157334	1,907633066
203640_at	MBNL2	4,597104084	2,689594859	1,907509225

202571_s_at	DLGAP4	1,094118704	-0,811769547	1,905888251
231905_at	C20orf96	-0,280273599	-2,186086603	1,905813004
231474_at	KHDC3L	-0,280273599	-2,186086603	1,905813004
235994_s_at	PLAC2	-0,280273599	-2,186086603	1,905813004
237091_at	-	-0,280273599	-2,186086603	1,905813004
222042_x_at	MEX3D	-0,280273599	-2,186086603	1,905813004
208779_x_at	DDR1 /// MIR4640	-0,280273599	-2,186086603	1,905813004
215927_at	ARFGEF2	0,847529938	-1,057301851	1,904831789
209314_s_at	HBS1L	3,498020785	1,593605508	1,904415276
212930_at	ATP2B1	1,671397663	-0,231045907	1,902443569
223196_s_at	SESN2	1,671397663	-0,231045907	1,902443569
211807_x_at	PCDHGB5	1,20396005	-0,697182596	1,901142646
215980_s_at	IGHMBP2	1,610353504	-0,290694975	1,901048479
209979_at	ADARB1	0,618277321	-1,282520722	1,900798043
224946_s_at	CCDC115	4,324702656	2,424202743	1,900499913
223894_s_at	AKTIP	2,896717728	0,996453274	1,900264454
218373_at	AKTIP	3,581038515	1,680963921	1,900074594
224743_at	IMPAD1	4,550683059	2,65087728	1,899805779
243400_x_at	LTBR	0,282358733	-1,616924751	1,899283484
226552_at	IER5L	0,926325262	-0,972893339	1,899218601
1554450_s_at	MIER3	0,926325262	-0,972893339	1,899218601
219914_at	ECEL1	0,707657549	-1,19085694	1,898514489
214898_x_at	MUC3B	0,707657549	-1,19085694	1,898514489
236529_at	SRCRB4D	0,707657549	-1,19085694	1,898514489
207843_x_at	CYB5A	5,625570411	3,727191713	1,898378698
201862_s_at	LRRFIP1	5,808735325	3,910368531	1,898366794
233410_at	CYP1B1-AS1	0,08619576	-1,811780055	1,897975814
206270_at	PRKCG	0,08619576	-1,811780055	1,897975814
243255_at	-	0,08619576	-1,811780055	1,897975814
203573_s_at	RABGGTA	2,053550345	0,155747281	1,897803064
235378_at	FAM161B	0,524545436	-1,372734086	1,897279522
241477_at	MIR631 /// NEIL1	0,524545436	-1,372734086	1,897279522
1564626_at	-	0,425036312	-1,471833228	1,89686954
243285_at	LOC283335	0,425036312	-1,471833228	1,89686954
206892_at	AMHR2	0,425036312	-1,471833228	1,89686954
211062_s_at	CPZ /// GPR78	0,425036312	-1,471833228	1,89686954
214073_at	CTTN	0,425036312	-1,471833228	1,89686954
217410_at	AGRN	-0,332405896	-2,229233437	1,896827542
222078_at	PKLR	-0,332405896	-2,229233437	1,896827542
212549_at	STAT5B	4,161221733	2,264929589	1,896292144
224564_s_at	RTN3	4,698307213	2,802594885	1,895712328
222438_at	MED4	1,160996507	-0,734355396	1,895351903
236283_x_at	LOC646214	2,356619952	0,461385738	1,895234214
222068_s_at	DNAAF1	1,476175815	-0,418248858	1,894424673
202208_s_at	ARL4C	3,018305638	1,123905343	1,894400295
235817_at	TMEM184A	1,830468611	-0,063377083	1,893845694
212345_s_at	CREB3L2	4,636598653	2,743433212	1,893165441
235344_at	PPM1A	0,791908897	-1,100943374	1,892852271
226747_at	TXNDC16	1,802733148	-0,089635713	1,89236886
1554251_at	HP1BP3	3,867546929	1,975797592	1,891749337

239256_at	-	0,127732831	-1,763664074	1,891396905
232085_at	MAPK8IP3	0,127732831	-1,763664074	1,891396905
237641_at	-	0,127732831	-1,763664074	1,891396905
229112_at	SIRT5	0,127732831	-1,763664074	1,891396905
222656_at	UBE2W	2,705643918	0,815200271	1,890443647
218983_at	C1RL	1,267146969	-0,623254098	1,890401068
238591_at	HEXDC	1,000459215	-0,889222211	1,889681425
225235_at	TSPAN17	3,023747815	1,135235995	1,88851182
234192_s_at	GKAP1	1,716003175	-0,172194225	1,888197399
226762_at	PURB	3,19484646	1,306903969	1,88794249
235069_at	TATDN3	1,116462765	-0,771340337	1,887803102
240142_at	LOC100652787	0,319287178	-1,568385657	1,887672834
222758_s_at	TMEM132A	0,319287178	-1,568385657	1,887672834
218952_at	PCSK1N	0,319287178	-1,568385657	1,887672834
243567_at	-	0,319287178	-1,568385657	1,887672834
205589_at	MYL3	0,319287178	-1,568385657	1,887672834
205100_at	GFPT2	1,97249974	0,085534992	1,886964747
209321_s_at	ADCY3	3,657457797	1,771157334	1,886300462
210678_s_at	AGPAT2	1,995527416	0,109458907	1,88606851
1569886_a_at	GLB1L3	2,401508182	0,515457602	1,88605058
1564066_at	TMEM151B	-0,384193355	-2,27001641	1,885823055
205156_s_at	ASIC1	2,597647262	0,711934445	1,885712817
212686_at	PPM1H	2,642918178	0,757638486	1,885279692
209312_x_at	HLA-DRB1 /// HLA-	7,219583512	5,334514516	1,885068996
225045_at	CCDC88A	2,685337919	0,80114751	1,884190409
238752_at	GPLD1	0,95222556	-0,931539147	1,883764707
223941_at	FBXO24	0,167727503	-1,715576125	1,883303628
224053_s_at	SLC4A9	0,167727503	-1,715576125	1,883303628
205582_s_at	GGT5	0,167727503	-1,715576125	1,883303628
230627_at	-	0,167727503	-1,715576125	1,883303628
1570243_at	-	0,167727503	-1,715576125	1,883303628
220561_at	IGF2-AS	0,167727503	-1,715576125	1,883303628
206653_at	POLR3G	3,190097062	1,306903969	1,883193092
208417_at	FGF6	1,071336699	-0,811769547	1,883106245
244591_x_at	RNF207	0,648195588	-1,234835326	1,883030914
210228_at	CSF2	0,648195588	-1,234835326	1,883030914
225950_at	SAMD8	1,528073958	-0,354781582	1,88285554
227170_at	ZNF316	1,364555912	-0,518232988	1,8827889
218340_s_at	UBA6	3,050461597	1,167919222	1,882542375
218068_s_at	ZNF672	3,505636113	1,623485524	1,88215059
204641_at	NEK2	4,835812686	2,954216214	1,881596472
221545_x_at	MED16	2,267141818	0,385582632	1,881559187
215193_x_at	HLA-DRB1 /// HLA-	7,130167623	5,248824047	1,881343576
216308_x_at	GRHPR	5,120627777	3,239448352	1,881179425
229807_s_at	-	0,458905032	-1,422257026	1,881162058
216882_s_at	NEBL	0,458905032	-1,422257026	1,881162058
204293_at	SGSH	0,458905032	-1,422257026	1,881162058
210565_at	GCGR	0,458905032	-1,422257026	1,881162058
242810_x_at	-	1,326463531	-0,554696666	1,881160197
1564028_s_at	FAM115C	0,735999505	-1,145057014	1,881056519

203584_at	EMC2	4,693076946	2,812515887	1,88056106
1555989_at	-	2,545254728	0,666486137	1,87876859
222857_s_at	KCNMB4	2,636121902	0,757638486	1,878483415
211008_s_at	UBE2I	0,820160788	-1,057301851	1,877462639
229577_at	AGPAT6	0,820160788	-1,057301851	1,877462639
1556839_s_at	LOC100289090	-0,551284523	-2,4284334	1,877148877
203337_x_at	ITGB1BP1	3,653735827	1,777135134	1,876600693
210332_at	ACHE	0,355751005	-1,520022123	1,875773128
1558728_at	LOC100128881	0,355751005	-1,520022123	1,875773128
225986_x_at	CPSF2	2,75889404	0,883374248	1,875519791
219417_s_at	C17orf59	1,936380191	0,061773582	1,874606609
239485_at	CDH4	-0,437449947	-2,312054429	1,874604482
226439_s_at	NBEA	-0,437449947	-2,312054429	1,874604482
234845_at	DKFZp761P0212	-0,437449947	-2,312054429	1,874604482
233385_x_at	MIA-RAB4B /// RA	3,273854609	1,399283506	1,874571103
238010_at	C1orf174	3,610731271	1,736450458	1,874280813
201531_at	ZFP36	3,389999385	1,516067292	1,873932093
203279_at	EDEM1	3,873276566	1,999354538	1,873922028
223790_at	KATNAL1	3,142467702	1,268569037	1,873898665
230335_at	C9orf50	0,20740896	-1,665791776	1,873200736
1568408_x_at	-	3,102023286	1,2298509	1,872172386
208546_x_at	HIST1H2BH	3,039689025	1,167919222	1,871769803
203568_s_at	TRIM38	3,681878794	1,811004388	1,870874406
242866_x_at	POU2F2	0,58788177	-1,282520722	1,870402492
239789_at	C11orf49	0,58788177	-1,282520722	1,870402492
241389_at	CHRNB2	0,58788177	-1,282520722	1,870402492
202932_at	YES1	2,817838865	0,947673886	1,870164979
228194_s_at	SORCS1	-0,135065865	-2,005029581	1,869963716
206743_s_at	ASGR1	-0,135065865	-2,005029581	1,869963716
219760_at	LIN7B	1,246401134	-0,623254098	1,869655232
243459_x_at	-	1,246401134	-0,623254098	1,869655232
226119_at	PCMTD1	4,580077988	2,710570973	1,869507015
217750_s_at	UBE2Z	4,882191987	3,012928641	1,869263345
1564660_at	LOC100131864	-0,180990326	-2,049747196	1,86875687
228684_at	ZNF503	-0,180990326	-2,049747196	1,86875687
1564435_a_at	KRT72	-0,180990326	-2,049747196	1,86875687
228413_s_at	SFRP1	-0,180990326	-2,049747196	1,86875687
242123_at	PAQR7	-0,180990326	-2,049747196	1,86875687
201722_s_at	GALNT1	3,723597116	1,855279162	1,868317955
224638_at	SPPL3	1,383446753	-0,484558493	1,868005246
210748_at	-	-0,088974936	-1,956681069	1,867706134
233618_at	-	-0,088974936	-1,956681069	1,867706134
217069_at	MLL4	-0,088974936	-1,956681069	1,867706134
227011_at	ZNF672	-0,088974936	-1,956681069	1,867706134
1557676_at	-	-0,043925712	-1,911206569	1,867280858
238925_at	SNTB2	-0,043925712	-1,911206569	1,867280858
1556699_at	-	-0,043925712	-1,911206569	1,867280858
227050_at	ODZ3	-0,043925712	-1,911206569	1,867280858
237092_at	-	-0,043925712	-1,911206569	1,867280858
243045_at	SMYD1	-0,043925712	-1,911206569	1,867280858

209911_x_at	HIST1H2BD	4,68949381	2,822379032	1,867114778
235254_at	-	-0,66934516	-2,536339282	1,866994122
209400_at	SLC12A4	-0,66934516	-2,536339282	1,866994122
243416_at	-	-0,66934516	-2,536339282	1,866994122
206125_s_at	KLK8	-0,230432956	-2,09678811	1,866355154
1568905_at	-	-0,230432956	-2,09678811	1,866355154
206916_x_at	TAT	-0,230432956	-2,09678811	1,866355154
221990_at	PAX8	-0,230432956	-2,09678811	1,866355154
236674_at	LOC388780	-0,230432956	-2,09678811	1,866355154
222741_s_at	SAYSD1	3,001575397	1,135235995	1,866339402
219656_at	PCDH12	1,094118704	-0,771340337	1,865459041
241395_at	NIT1	0,764143511	-1,100943374	1,865086885
230682_x_at	ABCC3	0,764143511	-1,100943374	1,865086885
46142_at	LMF1	0,443538786	-1,42143293	1,864971716
227236_at	TSPAN2	4,193391347	2,328492509	1,864898839
1569069_s_at	TDRD3	3,18076822	1,315897482	1,864870738
235635_at	ARHGAP5	0,491906512	-1,372734086	1,864640597
243387_at	MESP1	0,491906512	-1,372734086	1,864640597
233938_at	C11orf86	0,491906512	-1,372734086	1,864640597
203205_at	KDM4A	2,872644433	1,008210238	1,864434195
221794_at	DOCK6	1,345611839	-0,518232988	1,863844827
208073_x_at	TTC3 /// TTC3P1	5,220937666	3,357361807	1,863575859
218376_s_at	MICAL1	3,152091004	1,28853674	1,863554264
224629_at	LMAN1	5,832975879	3,969510541	1,863465338
239041_at	HIST1H2AK	0,001114523	-1,862269442	1,863383964
1566586_at	-	0,001114523	-1,862269442	1,863383964
221340_at	CDX4	0,001114523	-1,862269442	1,863383964
229148_at	-	0,001114523	-1,862269442	1,863383964
221204_s_at	CRTAC1	0,001114523	-1,862269442	1,863383964
1555831_s_at	LRRC41	3,122148189	1,259081178	1,863067011
204231_s_at	FAAH	0,39105295	-1,471833228	1,862886178
203417_at	MFAP2	0,39105295	-1,471833228	1,862886178
202442_at	AP3S1	7,100423041	5,238309473	1,862113568
242615_at	CCDC37	0,244681185	-1,616924751	1,861605937
219672_at	AHSP	0,244681185	-1,616924751	1,861605937
200962_at	RPL31	3,624986574	1,76396664	1,861019934
226454_at	09.03.15	2,410823487	0,550017041	1,860806446
226496_at	ZCCHC7	4,082216372	2,221565736	1,860650637
226043_at	GPSM1	2,444186596	0,583607503	1,860579093
214965_at	SPATA2L	2,460890032	0,600385088	1,860504944
202545_at	PRKCD	2,374261973	0,515457602	1,858804371
235315_at	TSC22D1	1,871521385	0,013190398	1,858330987
207495_at	RAB28	1,160996507	-0,697182596	1,858179104
239125_at	SLC25A5-AS1	-0,49284695	-2,350444759	1,857597809
217358_at	DNAJC16	-0,49284695	-2,350444759	1,857597809
238063_at	TMEM154	3,349017424	1,491644074	1,85737335
205124_at	MEF2B /// MEF2BI	-0,609040214	-2,466349414	1,8573092
1559083_x_at	LOC284600	-0,609040214	-2,466349414	1,8573092
207689_at	TBX10	0,045517965	-1,811780055	1,857298019
1557166_at	PDCD4	0,045517965	-1,811780055	1,857298019

217138_x_at	IGLC1	0,045517965	-1,811780055	1,857298019
218044_x_at	PTMS	0,045517965	-1,811780055	1,857298019
217660_at	MYH14	0,045517965	-1,811780055	1,857298019
226384_at	PPAPDC1B	3,209439373	1,352622437	1,856816936
213739_at	-	1,625482993	-0,231045907	1,8565289
202259_s_at	N4BP2L2	3,171328694	1,315897482	1,855431212
209320_at	ADCY3	3,132805465	1,278654284	1,854151182
237284_at	DNAJB8	-0,332405896	-2,186086603	1,853680707
230207_s_at	DOCK5	1,816938997	-0,036543687	1,853482684
238114_at	PCMTD1	0,618277321	-1,234835326	1,853112647
242341_x_at	GLYCTK	0,618277321	-1,234835326	1,853112647
1554188_at	C11orf53	0,618277321	-1,234835326	1,853112647
226016_at	CD47	4,891443931	3,038411131	1,8530328
201466_s_at	JUN	2,199941239	0,346964736	1,852976503
230939_at	-	0,707657549	-1,145057014	1,852714563
233642_s_at	HEATR5B	3,852052012	1,999354538	1,852697474
201866_s_at	NR3C1	3,233229368	1,380552339	1,852677029
209893_s_at	FUT4	1,561816652	-0,290694975	1,852511628
204172_at	CPOX	6,272140316	4,419887146	1,85225317
228788_at	YPEL1	0,524545436	-1,32740143	1,851946866
210253_at	HTATIP2	3,939815341	2,088449486	1,851365855
225773_at	RSPRY1	3,414594082	1,563256447	1,851337635
227019_at	C1orf226	0,282358733	-1,568385657	1,85074439
215899_at	-	0,282358733	-1,568385657	1,85074439
237646_x_at	PLEKHG5	0,282358733	-1,568385657	1,85074439
244496_at	-	0,282358733	-1,568385657	1,85074439
202881_x_at	-	0,282358733	-1,568385657	1,85074439
209366_x_at	CYB5A	5,792565538	3,942089645	1,850475893
202711_at	EFNB1	0,08619576	-1,763664074	1,849859834
225122_at	RNF31	0,08619576	-1,763664074	1,849859834
231831_at	COX19	1,000459215	-0,84928999	1,849749205
201036_s_at	HADH	5,663811129	3,814403792	1,849407336
210864_x_at	HFE	0,791908897	-1,057301851	1,849210748
216473_x_at	DUX2 /// DUX4 ///	1,364555912	-0,484558493	1,849114405
202450_s_at	CTSK	1,225123479	-0,623254098	1,848377577
232358_at	KIAA1328	1,225123479	-0,623254098	1,848377577
37986_at	EPOR	1,227288898	-0,620835232	1,84812413
1570105_at	-	0,425036312	-1,422257026	1,847293339
243075_at	-	0,425036312	-1,422257026	1,847293339
207105_s_at	PIK3R2	0,425036312	-1,422257026	1,847293339
205375_at	MDFI	0,425036312	-1,422257026	1,847293339
1563315_s_at	ERICH1	3,362258367	1,516067292	1,846191076
208754_s_at	NAP1L1	6,326241053	4,480622042	1,84561901
225331_at	CCDC50	6,980531731	5,135078464	1,845453266
241513_at	-	-0,384193355	-2,229233437	1,845040083
205557_at	BPI	-0,384193355	-2,229233437	1,845040083
244194_at	ADAM22	1,326463531	-0,518232988	1,844696519
218368_s_at	TNFRSF12A	1,671397663	-0,172194225	1,843591887
224310_s_at	BCL11B	0,127732831	-1,715576125	1,843308956
1560405_at	-	0,127732831	-1,715576125	1,843308956

237858_s_at	MTUS2	0,127732831	-1,715576125	1,843308956
211225_at	FUT5	0,127732831	-1,715576125	1,843308956
228903_at	CES4A	0,127732831	-1,715576125	1,843308956
204046_at	PLCB2	2,285848435	0,443143223	1,842705212
213192_at	THAP3	2,410823487	0,568155442	1,842668044
208877_at	PAK2	4,462797972	2,621003244	1,841794728
203230_at	DVL1	2,107136847	0,265475485	1,841661362
218638_s_at	LOC100130872 ///	0,95222556	-0,889222211	1,841447771
204783_at	MLF1	2,085817135	0,244646697	1,841170437
221351_at	HTR1A	-0,551284523	-2,391863529	1,840579006
227666_at	DCLK2	-0,551284523	-2,391863529	1,840579006
244281_at	REXO1	-0,551284523	-2,391863529	1,840579006
216963_s_at	GAP43	-0,551284523	-2,391863529	1,840579006
240193_at	-	-0,730013898	-2,570522741	1,840508843
225706_at	GLCCI1	4,128096493	2,287674614	1,840421879
219838_at	TTC23	0,319287178	-1,520022123	1,839309301
202764_at	STIM1	1,802733148	-0,036543687	1,839276835
212751_at	UBE2N	4,85214636	3,012928641	1,839217718
228328_at	KLHL28	3,086998005	1,249194639	1,837803366
207667_s_at	MAP2K3	2,949541692	1,112548615	1,836993077
229446_at	-	0,735999505	-1,100943374	1,836942879
226728_at	SLC27A1	1,139057614	-0,697182596	1,836240211
238505_at	ADPRH	1,139057614	-0,697182596	1,836240211
208328_s_at	MEF2A	1,024284941	-0,811769547	1,836054488
227221_at	ZMAT3	2,75889404	0,923203038	1,835691001
222315_at	-	0,820160788	-1,015398016	1,835558804
215265_at	EMX1	0,820160788	-1,015398016	1,835558804
222488_s_at	DCTN4	4,924288109	3,08892491	1,8353632
210010_s_at	SLC25A1	2,745538988	0,910483921	1,835055067
227641_at	FBXL16	3,243218364	1,408220608	1,834997756
206907_at	TNFSF9	2,276736537	0,443143223	1,833593314
1553392_at	EFCAB3	0,167727503	-1,665791776	1,833519279
211560_s_at	ALAS2	0,167727503	-1,665791776	1,833519279
207476_at	LOC100507630	0,167727503	-1,665791776	1,833519279
228625_at	CITED4	0,167727503	-1,665791776	1,833519279
217028_at	CXCR4	6,840820793	5,007355999	1,833464794
201536_at	DUSP3	2,2388565	0,405752839	1,833103661
211822_s_at	NLRP1	2,179708195	0,346964736	1,832743459
224763_at	LOC100506548 ///	4,265403137	2,432792627	1,83261051
225387_at	TSPAN5	3,969866565	2,137342905	1,83252366
209536_s_at	EHD4	3,909506676	2,077419168	1,832087507
228198_s_at	FAHD2B /// FAHD2	1,686984721	-0,144765583	1,831750304
243467_at	-	0,458905032	-1,372734086	1,831639117
234909_at	-	0,458905032	-1,372734086	1,831639117
203705_s_at	FZD7	0,458905032	-1,372734086	1,831639117
206994_at	CST4	0,458905032	-1,372734086	1,831639117
228054_at	TMEM44	2,007220798	0,178191865	1,829028933
200762_at	DPYSL2	6,564132762	4,73510874	1,829024023
213373_s_at	CASP8	4,514001815	2,686001625	1,82800019
1553438_at	C11orf72	1,65554524	-0,172194225	1,827739464

212748_at	MKL1	1,960819106	0,133104519	1,827714587
241246_at	-	0,355751005	-1,471833228	1,827584233
231402_at	-	0,355751005	-1,471833228	1,827584233
1564339_a_at	CHRM3	1,20396005	-0,623254098	1,827214148
207606_s_at	ARHGAP12	1,20396005	-0,623254098	1,827214148
219356_s_at	CHMP5	5,876907286	4,049836996	1,82707029
201237_at	CAPZA2	4,977744402	3,150841611	1,826902791
222389_s_at	WAC	5,645320963	3,818669263	1,8266517
241348_at	ZNF654	2,460890032	0,634301128	1,826588904
204487_s_at	KCNQ1	0,97663406	-0,84928999	1,82592405
230952_at	-	1,307201325	-0,518232988	1,825434313
214010_s_at	ATP9B	1,307201325	-0,518232988	1,825434313
212697_at	FAM134C	3,668220666	1,842852096	1,82536857
227506_at	SLC16A9	2,285848435	0,461385738	1,824462698
228593_at	MTMR9LP	-0,180990326	-2,005029581	1,824039255
205822_s_at	HMGCS1	3,980708208	2,157148588	1,823559619
202646_s_at	CSDE1	6,186092468	4,362853726	1,823238743
227255_at	PDIK1L	3,517785666	1,694547353	1,823238313
212180_at	CRKL	4,661121858	2,83813104	1,822990818
221032_s_at	TMPRSS5	0,677745787	-1,145057014	1,822802801
1557000_at	ESPNL	0,677745787	-1,145057014	1,822802801
213429_at	BICC1	-0,088974936	-1,911206569	1,822231634
227415_at	LOC283508	2,664625637	0,842599219	1,822026418
236960_at	-	-0,135065865	-1,956681069	1,821615204
208299_at	CACNA1I	-0,135065865	-1,956681069	1,821615204
240664_at	-	-0,135065865	-1,956681069	1,821615204
227142_at	PLEKHG5	0,764143511	-1,057301851	1,821445362
208760_at	UBE2I	3,237994092	1,417157926	1,820836165
222452_s_at	GPBP1L1	3,813758784	1,993309863	1,820448921
229430_at	C8orf46	-0,609040214	-2,4284334	1,819393186
210600_s_at	GRK4	-0,230432956	-2,049747196	1,81931424
230971_x_at	GLTPD2	-0,230432956	-2,049747196	1,81931424
220604_x_at	FTCD	-0,230432956	-2,049747196	1,81931424
237434_x_at	MSANTD1	0,491906512	-1,32740143	1,819307942
206889_at	PDIA2	0,491906512	-1,32740143	1,819307942
202700_s_at	TMEM63A	0,491906512	-1,32740143	1,819307942
1568764_x_at	LOC728613 /// PD	-0,49284695	-2,312054429	1,819207479
202377_at	LEPROT	3,389999385	1,571112806	1,818886579
228929_at	DNASE1	1,047341799	-0,771340337	1,818682136
234716_at	ZIC1	-0,043925712	-1,862269442	1,81834373
220177_s_at	TMPRSS3	3,007119335	1,189078962	1,818040373
201928_at	PKP4	4,328738561	2,5107204	1,81801816
209510_at	RNF139	5,037499265	3,220021599	1,817477665
205046_at	CENPE	5,167863921	3,3507559	1,817108021
206619_at	DKK4	-0,280273599	-2,09678811	1,816514511
232191_at	ERVH48-1	-0,280273599	-2,09678811	1,816514511
205314_x_at	SNTB2	-0,280273599	-2,09678811	1,816514511
210343_s_at	SLC22A6	-0,280273599	-2,09678811	1,816514511
1553545_at	ILDR1	-0,280273599	-2,09678811	1,816514511
204728_s_at	WDHD1	1,671397663	-0,144765583	1,816163246

227254_at	POU2F1	1,948628481	0,133104519	1,815523962
206485_at	CD5	2,33082239	0,515457602	1,815364788
204653_at	TFAP2A	2,798156197	0,98381571	1,814340487
239412_at	IRF5	2,798156197	0,98381571	1,814340487
204302_s_at	CTIF	1,116462765	-0,697182596	1,813645361
210905_x_at	POU5F1P4	0,39105295	-1,422257026	1,813309977
207979_s_at	CD8B	0,39105295	-1,422257026	1,813309977
233896_s_at	PAPLN	0,244681185	-1,568385657	1,813066842
244499_at	THAP2	0,001114523	-1,811780055	1,812894578
230107_at	-	0,001114523	-1,811780055	1,812894578
242670_at	LGI4	0,001114523	-1,811780055	1,812894578
244057_s_at	VSTM4	0,001114523	-1,811780055	1,812894578
239215_at	LOC100289495	1,000459215	-0,811769547	1,812228761
244005_at	-	-0,791294935	-2,603471267	1,812176332
218616_at	INTS12	5,547581576	3,735670695	1,81191088
222582_at	PRKAG2	1,775035811	-0,036543687	1,811579498
209244_s_at	KIF1C	1,326463531	-0,484558493	1,811022024
45714_at	HCFC1R1	2,530496634	0,719548765	1,810947869
243809_at	HELQ	-0,332405896	-2,142286822	1,809880926
217467_at	-	-0,332405896	-2,142286822	1,809880926
204989_s_at	ITGB4	-0,332405896	-2,142286822	1,809880926
211411_at	-	-0,332405896	-2,142286822	1,809880926
216942_s_at	CD58	5,08526593	3,275571286	1,809694644
228605_at	UBXN2A	3,639446351	1,830129603	1,809316747
242200_at	ADAMTSL5	0,045517965	-1,763664074	1,809182039
234310_s_at	SUSD2	0,045517965	-1,763664074	1,809182039
227704_at	C19orf12	0,618277321	-1,19085694	1,809134261
1555561_a_at	UGGT2	-0,856259172	-2,665156778	1,808897606
204622_x_at	NR4A2	1,745502609	-0,063377083	1,808879692
206339_at	CARTPT	0,707657549	-1,100943374	1,808600923
207954_at	GATA2	0,707657549	-1,100943374	1,808600923
225305_at	SLC25A29	1,421391637	-0,386896102	1,808287739
227096_at	JOSD2	1,421391637	-0,386896102	1,808287739
202570_s_at	DLGAP4	2,804738618	0,996453274	1,808285344
201059_at	CTTN	3,481948861	1,674071917	1,807876944
215788_at	KIAA1751	0,791908897	-1,015398016	1,807306914
232753_at	ZNF346	0,524545436	-1,282520722	1,807066159
230770_at	-	0,524545436	-1,282520722	1,807066159
218353_at	RGS5	-0,730013898	-2,536339282	1,806325383
205310_at	FBXO46	3,122148189	1,315897482	1,806250707
237163_x_at	LOC390705	1,287793882	-0,518232988	1,80602687
202981_x_at	SIAH1	3,921953088	2,116147513	1,805805575
229549_at	CALU	1,983901861	0,178191865	1,805709996
227326_at	MXRA7	1,544940605	-0,260526297	1,805466902
203252_at	CDK2AP2	5,054431342	3,250322783	1,804108559
202105_at	IGBP1	5,167863921	3,363771078	1,804092843
202314_at	CYP51A1 /// LRRD:	4,537242248	2,733194118	1,804048131
224444_s_at	LINC00467	3,639446351	1,836262316	1,803184034
211828_s_at	TNIK	2,468978138	0,666486137	1,802492001
237764_at	-	0,282358733	-1,520022123	1,802380856

206933_s_at	H6PD	0,282358733	-1,520022123	1,802380856
211498_s_at	NKX3-1	-0,384193355	-2,186086603	1,801893248
236606_at	-	-0,384193355	-2,186086603	1,801893248
238267_s_at	-	0,08619576	-1,715576125	1,801771885
238143_at	LOC646627	0,08619576	-1,715576125	1,801771885
211808_s_at	CREBBP	0,08619576	-1,715576125	1,801771885
1556533_at	C17orf52	0,08619576	-1,715576125	1,801771885
229921_at	-	0,08619576	-1,715576125	1,801771885
208196_x_at	NFATC1	1,510970097	-0,290694975	1,801665072
215807_s_at	PLXNB1	0,95222556	-0,84928999	1,80151555
219028_at	HIPK2	1,910557155	0,109458907	1,801098249
228495_at	CCDC75	3,883929118	2,082919929	1,801009189
236195_x_at	PRKCG	1,139057614	-0,661178575	1,800236189
215026_x_at	SCNN1A	1,139057614	-0,661178575	1,800236189
244389_at	TLK1	-0,551284523	-2,350444759	1,799160237
224949_at	YIPF5	4,010828383	2,212304392	1,79852399
235533_at	COX19	1,476175815	-0,322340048	1,798515863
232034_at	LINC00537	2,063971513	0,265475485	1,798496028
236933_at	EVPLL	0,425036312	-1,372734086	1,797770398
1559478_at	-	0,425036312	-1,372734086	1,797770398
235276_at	EPSTI1	2,276736537	0,479089184	1,797647353
37796_at	LRCH4 /// SAP25	0,967968309	-0,829581071	1,79754938
215572_at	-	-0,66934516	-2,466349414	1,797004254
222718_at	TMEM8A	1,345611839	-0,45121326	1,796825098
201655_s_at	HSPG2	1,440265662	-0,354781582	1,795047244
228519_x_at	CIRBP	2,476788561	0,681874787	1,794913774
203145_at	SPAG5	4,963866728	3,169167796	1,794698931
214683_s_at	CLK1	3,723597116	1,929092307	1,794504809
205877_s_at	ZC3H7B	2,199941239	0,405752839	1,7941884
207126_x_at	UGT1A1 /// UGT1/	0,127732831	-1,665791776	1,793524607
214888_at	CAPN2	0,127732831	-1,665791776	1,793524607
211347_at	CDC14B	0,127732831	-1,665791776	1,793524607
228804_at	DGCR5 /// LOC100	0,127732831	-1,665791776	1,793524607
219101_x_at	ABHD8	0,127732831	-1,665791776	1,793524607
237846_at	-	0,127732831	-1,665791776	1,793524607
1554050_at	SMPDL3B	0,127732831	-1,665791776	1,793524607
241478_at	MICALL2	0,735999505	-1,057301851	1,793301356
1569473_s_at	LOC155060 /// ZNI	0,735999505	-1,057301851	1,793301356
223469_at	PGPEP1	0,820160788	-0,972893339	1,793054126
206791_s_at	PDE4C	0,820160788	-0,972893339	1,793054126
227728_at	PPM1A	3,300874993	1,508107415	1,792767578
222234_s_at	DBNDD1	-0,437449947	-2,229233437	1,79178349
240029_at	C11orf94	-0,437449947	-2,229233437	1,79178349
1555164_at	KIF26B	0,556428218	-1,234835326	1,791263544
232156_at	-	0,556428218	-1,234835326	1,791263544
233812_at	LINC00028	0,556428218	-1,234835326	1,791263544
210874_s_at	NAT6	1,701542258	-0,089635713	1,79117797
1559218_s_at	NFYC	0,319287178	-1,471833228	1,791120405
235605_at	CASZ1	0,319287178	-1,471833228	1,791120405
214301_s_at	DPYSL4	0,319287178	-1,471833228	1,791120405

204985_s_at	TRAPPC6A	4,603614396	2,812515887	1,79109851
229074_at	EHD4	3,190097062	1,399283506	1,790813556
226617_at	ARL5A	4,719538164	2,930238085	1,789300079
234481_at	-	0,900065578	-0,889222211	1,789287789
230172_at	IFI27L1	3,398333742	1,60918327	1,789150471
208860_s_at	ATRX	2,590269697	0,80114751	1,789122187
215286_s_at	PHTF2	2,321828846	0,533258442	1,788570404
206635_at	CHRNB2	2,784898907	0,996453274	1,788445634
217055_x_at	-	0,97663406	-0,811769547	1,788403607
211559_s_at	CCNG2	2,955426542	1,167919222	1,78750732
217967_s_at	FAM129A	5,659001793	3,871747013	1,78725478
242383_at	-	0,458905032	-1,32740143	1,786306462
203344_s_at	RBBP8	5,180080677	3,393801804	1,786278873
203679_at	TMED1	3,102023286	1,315897482	1,786125804
240945_at	-	-0,994186487	-2,780225148	1,786038661
201794_s_at	SMG7	2,920736393	1,135235995	1,785500398
209050_s_at	RALGDS	4,409540371	2,624682607	1,784857765
208014_x_at	-	0,167727503	-1,616924751	1,784652255
225802_at	TOP1MT	3,147261986	1,362900801	1,784361185
1555500_s_at	SLC2A4RG	1,493647653	-0,290694975	1,784342628
202455_at	HDAC5	1,493647653	-0,290694975	1,784342628
1563639_a_at	FHAD1	1,160996507	-0,623254098	1,784250606
214656_x_at	MYO1C	4,052656868	2,269637658	1,78301921
228414_at	KCNMA1	-0,609040214	-2,391863529	1,782823315
207050_at	CACNA2D1	-0,609040214	-2,391863529	1,782823315
213600_at	SIPA1L3	1,047341799	-0,734355396	1,781697195
1559530_at	-	1,047341799	-0,734355396	1,781697195
202219_at	SLC6A8	1,458255986	-0,322340048	1,780596034
235396_at	C22orf25	3,223556135	1,44320939	1,780346745
34858_at	KCTD2	2,507442246	0,727584206	1,77985804
243680_at	LOC100506476	1,225123479	-0,554696666	1,779820145
213374_x_at	HIBCH	4,59885279	2,819209749	1,779643041
1566455_at	LOC100507494	-0,791294935	-2,570522741	1,779227806
211284_s_at	GRN	3,720319513	1,941115499	1,779204014
227290_at	LOC100509498	3,018305638	1,239268196	1,779037442
208519_x_at	GNRH2	0,58788177	-1,19085694	1,77873871
213352_at	TMCC1	0,677745787	-1,100943374	1,778689161
202772_at	HMGCL	4,306090192	2,527778509	1,778311683
243802_at	DNAH12	0,355751005	-1,422257026	1,778008031
1556727_at	PRCD	0,355751005	-1,422257026	1,778008031
219824_at	SLC13A4	0,355751005	-1,422257026	1,778008031
234299_s_at	NIN	0,355751005	-1,422257026	1,778008031
233303_at	-	2,444186596	0,666486137	1,777700459
242442_x_at	TRMT10A	1,326463531	-0,45121326	1,77767679
229407_at	SDK1	-0,49284695	-2,27001641	1,77716946
1562903_at	FLJ10661	-0,49284695	-2,27001641	1,77716946
1555979_at	LOC100130417	-0,49284695	-2,27001641	1,77716946
215863_at	TFR2	-0,49284695	-2,27001641	1,77716946
212690_at	DDHD2	4,308320282	2,531843788	1,776476494
209221_s_at	OSBPL2	1,421391637	-0,354781582	1,776173219

227155_at	LMO4	-0,135065865	-1,911206569	1,776140704
230025_at	GJD3	-0,135065865	-1,911206569	1,776140704
213991_s_at	HS3ST1	-0,135065865	-1,911206569	1,776140704
220565_at	CCR10	-0,135065865	-1,911206569	1,776140704
211974_x_at	RBPJ	7,67595503	5,899849879	1,776105151
210044_s_at	LYL1	1,544940605	-0,231045907	1,775986512
239295_at	SRSF12	0,20740896	-1,568385657	1,775794617
235488_at	RASL10B	0,20740896	-1,568385657	1,775794617
241939_at	IQGAP3	0,20740896	-1,568385657	1,775794617
222084_s_at	SBF1	-0,180990326	-1,956681069	1,775690744
214067_at	TSR3	-0,180990326	-1,956681069	1,775690744
209159_s_at	NDRG4	-0,180990326	-1,956681069	1,775690744
203243_s_at	PDLIM5	3,218770562	1,44320939	1,775561172
218958_at	C19orf60	2,771643417	0,996453274	1,775190144
1563983_at	TPT1-AS1	-0,230432956	-2,005029581	1,774596625
1563081_at	-	-0,230432956	-2,005029581	1,774596625
231803_at	FGF11	-0,230432956	-2,005029581	1,774596625
230080_at	HOXA-AS2	-0,230432956	-2,005029581	1,774596625
228777_at	KBTBD3	2,392346173	0,61779983	1,774546343
222831_at	SAP30L	0,665349482	-1,10917789	1,774527373
227743_at	MYO15B	0,491906512	-1,282520722	1,774427234
219554_at	RHCG	0,491906512	-1,282520722	1,774427234
231189_at	-	0,491906512	-1,282520722	1,774427234
215367_at	KIAA1614	0,491906512	-1,282520722	1,774427234
1552540_s_at	IQCD	0,491906512	-1,282520722	1,774427234
214875_x_at	APLP2	2,560606634	0,786391549	1,774215085
234820_at	MAS1L	-0,088974936	-1,862269442	1,773294506
227661_at	-	-0,088974936	-1,862269442	1,773294506
206094_x_at	UGT1A1 /// UGT1/	-0,088974936	-1,862269442	1,773294506
224072_s_at	KCNK9	-0,088974936	-1,862269442	1,773294506
207690_at	ALX3	-0,088974936	-1,862269442	1,773294506
207883_s_at	TFR2	-0,088974936	-1,862269442	1,773294506
210484_s_at	LOC254896 /// TN	-0,088974936	-1,862269442	1,773294506
242402_x_at	ARAP2	-0,088974936	-1,862269442	1,773294506
210459_at	PSMD4	-0,088974936	-1,862269442	1,773294506
217363_x_at	-	2,484824532	0,711934445	1,772890087
224571_at	IRF2BP2	4,158437147	2,385765892	1,772671255
221902_at	GPR153	2,138605583	0,366031214	1,77257437
211534_x_at	PTPRN2	2,732422196	0,960042218	1,772379978
224674_at	TTYH3	1,65554524	-0,116767475	1,772312714
213138_at	ARID5A	2,961244348	1,189078962	1,772165386
34868_at	SMG5	2,138755115	0,366624915	1,7721302
218723_s_at	RGCC	1,97249974	0,20062106	1,77187868
232406_at	-	1,000459215	-0,771340337	1,771799552
229998_x_at	FAM176B	1,000459215	-0,771340337	1,771799552
233949_s_at	MYH7B	-0,730013898	-2,501359113	1,771345215
205291_at	IL2RB	6,621908998	4,85186468	1,770044318
236803_at	LOC100506214	-0,280273599	-2,049747196	1,769473597
1556808_at	-	-0,280273599	-2,049747196	1,769473597
232428_at	MOGAT2	-0,280273599	-2,049747196	1,769473597

243429_at	-	-0,280273599	-2,049747196	1,769473597
224267_x_at	ZAN	-0,280273599	-2,049747196	1,769473597
206568_at	TNP1	-0,280273599	-2,049747196	1,769473597
213818_x_at	TMEM223	-0,280273599	-2,049747196	1,769473597
243288_at	SMYD2	-0,280273599	-2,049747196	1,769473597
204681_s_at	RAPGEF5	-0,280273599	-2,049747196	1,769473597
221491_x_at	HLA-DRB1 /// HLA-	-0,280273599	-2,049747196	1,769473597
231040_at	-	2,824160383	1,055317091	1,768843291
230504_at	CEACAM19	1,071336699	-0,697182596	1,768519295
212647_at	RRAS	2,074841895	0,306454867	1,768387028
221362_at	HTR5A	-0,043925712	-1,811780055	1,767854343
236496_at	DEGS2	-0,043925712	-1,811780055	1,767854343
216427_at	-	-0,043925712	-1,811780055	1,767854343
214142_at	ZG16	-0,043925712	-1,811780055	1,767854343
204264_at	CPT2	2,966903803	1,199296812	1,767606991
223130_s_at	MYLIP	3,584942056	1,817403386	1,76753867
202014_at	PPP1R15A	1,476175815	-0,290694975	1,76687079
223547_at	JKAMP	1,5944803	-0,172194225	1,766674525
223041_at	CD99L2	2,38349585	0,61779983	1,76569602
206200_s_at	ANXA11	5,716565683	3,951004713	1,76556097
218388_at	PGLS	4,138272593	2,373249966	1,765022627
239334_at	ESYT2	0,707657549	-1,057301851	1,764959399
205575_at	C1QL1	0,707657549	-1,057301851	1,764959399
214074_s_at	CTTN	0,707657549	-1,057301851	1,764959399
224055_x_at	KCNK7	0,707657549	-1,057301851	1,764959399
222169_x_at	SH2D3A	0,791908897	-0,972893339	1,764802236
205539_at	AVIL	0,001114523	-1,763664074	1,764778597
238202_at	-	0,001114523	-1,763664074	1,764778597
237414_at	F7	0,001114523	-1,763664074	1,764778597
243600_at	TPGS1	0,001114523	-1,763664074	1,764778597
232402_at	-	0,001114523	-1,763664074	1,764778597
230548_at	LOC100505942	0,244681185	-1,520022123	1,764703309
1566474_at	-	0,244681185	-1,520022123	1,764703309
202005_at	ST14	0,244681185	-1,520022123	1,764703309
233745_at	PTOV1-AS1	-0,332405896	-2,09678811	1,764382214
206885_x_at	GH1	-0,332405896	-2,09678811	1,764382214
203886_s_at	FBLN2	-0,332405896	-2,09678811	1,764382214
1559765_a_at	LOC286254	0,95222556	-0,811769547	1,763995107
218362_s_at	DIS3	1,345611839	-0,418248858	1,763860697
227281_at	SLC29A4	0,39105295	-1,372734086	1,763787036
205695_at	SDS	0,618277321	-1,145057014	1,763334335
207631_at	NBR2	0,618277321	-1,145057014	1,763334335
220185_at	SPTBN4	0,873789652	-0,889222211	1,763011863
231071_at	-	0,873789652	-0,889222211	1,763011863
204550_x_at	GSTM1	1,440265662	-0,322340048	1,76260571
202933_s_at	YES1	3,278243793	1,516067292	1,762176501
203136_at	RABAC1	5,335944076	3,5744736	1,761470476
211167_s_at	GCK	0,045517965	-1,715576125	1,76109409
213250_at	CCDC85B	0,045517965	-1,715576125	1,76109409
217357_at	-	0,045517965	-1,715576125	1,76109409

203636_at	MID1	0,045517965	-1,715576125	1,76109409
1555503_a_at	TMCC2	0,045517965	-1,715576125	1,76109409
236784_s_at	-	0,045517965	-1,715576125	1,76109409
217989_at	HSD17B11	5,089067923	3,328091364	1,760976559
234933_at	CC2D2A	-0,551284523	-2,312054429	1,760769906
204508_s_at	CA12	-0,551284523	-2,312054429	1,760769906
220928_s_at	PRDM16	-0,551284523	-2,312054429	1,760769906
241261_x_at	-	-0,551284523	-2,312054429	1,760769906
236857_at	SLC38A1	-0,551284523	-2,312054429	1,760769906
213113_s_at	SLC43A3	4,116324812	2,355777584	1,760547228
243626_at	-	0,524545436	-1,234835326	1,759380763
239362_at	NAPA-AS1	0,524545436	-1,234835326	1,759380763
1564314_at	LOC219690	-0,66934516	-2,4284334	1,759088241
207640_x_at	NTN3	-0,66934516	-2,4284334	1,759088241
1569652_at	MLLT3	-0,66934516	-2,4284334	1,759088241
224759_s_at	C12orf23	5,248327634	3,489363371	1,758964263
228857_at	GNL1	1,307201325	-0,45121326	1,758414585
205154_at	LRRN2	-0,384193355	-2,142286822	1,758093467
1557558_s_at	MATN1-AS1	-0,384193355	-2,142286822	1,758093467
239508_x_at	CCDC108	-0,384193355	-2,142286822	1,758093467
231651_at	C17orf57	-0,384193355	-2,142286822	1,758093467
209886_s_at	SMAD6	-0,384193355	-2,142286822	1,758093467
227622_at	PCF11	2,484824532	0,727123109	1,757701423
204401_at	KCNN4	2,836219929	1,078619386	1,757600543
235191_at	-	2,530390387	0,772793615	1,757596772
243259_at	ATXN7	2,063971513	0,306454867	1,757516646
243176_at	ARL5A	2,063971513	0,306454867	1,757516646
201275_at	FDPS	6,464816311	4,707623164	1,757193147
201407_s_at	PPP1CB	5,038901064	3,281812922	1,757088141
212259_s_at	PBXIP1	1,640193838	-0,116767475	1,756961313
211919_s_at	CXCR4	5,862887796	4,105949949	1,756937846
225266_at	ZNF652	2,966903803	1,209973561	1,756930242
205790_at	SKAP1	4,769413297	3,012928641	1,756484655
233825_s_at	CD99L2	2,356619952	0,600385088	1,756234863
207098_s_at	MFN1	2,955426542	1,199296812	1,756129729
201480_s_at	SUPT5H	2,452674045	0,697221375	1,75545267
211952_at	IPO5	1,816938997	0,061773582	1,755165415
212564_at	KCTD2	2,597647262	0,842599219	1,755048043
209436_at	SPON1	0,282358733	-1,471833228	1,754191961
214128_at	DAGLA	0,282358733	-1,471833228	1,754191961
230169_at	THAP6	0,282358733	-1,471833228	1,754191961
216128_at	TBCD	0,282358733	-1,471833228	1,754191961
206793_at	PNMT	0,282358733	-1,471833228	1,754191961
1552761_at	SLC16A11	0,282358733	-1,471833228	1,754191961
219058_x_at	TINAGL1	1,493647653	-0,260526297	1,75417395
210653_s_at	BCKDHB	2,932161212	1,178100917	1,754060295
202708_s_at	HIST2H2BE	2,019346222	0,265475485	1,753870737
201624_at	DARS	5,405175098	3,651987434	1,753187664
212017_at	FAM168B	4,022766247	2,269637658	1,753128589
211938_at	EIF4B /// LOC1006	5,991527772	4,238707962	1,75281981

226072_at	FUK	1,716003175	-0,036543687	1,752546862
234130_at	LDB3	0,425036312	-1,32740143	1,752437742
211002_s_at	TRIM29	0,08619576	-1,665791776	1,751987536
217516_x_at	ARVCF	1,267146969	-0,484558493	1,751705463
237463_at	ZFPM1	0,820160788	-0,931539147	1,751699935
219905_at	ERMAP	1,364555912	-0,386896102	1,751452014
201331_s_at	STAT6	3,861345795	2,110584371	1,750761425
212263_at	QKI	4,153515595	2,403191094	1,750324501
212764_at	ZEB1	4,610466181	2,860202326	1,750263854
229811_at	-	0,648195588	-1,100943374	1,749138962
233157_x_at	CCDC114	0,648195588	-1,100943374	1,749138962
1559091_s_at	FGD2	0,648195588	-1,100943374	1,749138962
228219_s_at	UPB1	1,458255986	-0,290694975	1,748950961
233278_at	-	-0,437449947	-2,186086603	1,748636656
201795_at	LBR	6,619674546	4,871099544	1,748575002
213930_at	ATG12	2,209870035	0,461385738	1,748484298
227577_at	EXOC8	3,414594082	1,666953618	1,747640464
244477_at	-	0,556428218	-1,19085694	1,747285158
214633_at	SOX3	-0,856259172	-2,603471267	1,747212095
226822_at	STOX2	1,760255626	0,013190398	1,747065228
1554704_at	ATP8B3	2,657308324	0,910483921	1,746824403
208792_s_at	CLU	1,544940605	-0,201789721	1,746730326
225578_at	MZT1	5,014397839	3,26868404	1,745713799
223014_at	UBE2R2	5,007345522	3,261989326	1,745356196
214562_at	HIST1H4A /// HIST	-0,791294935	-2,536339282	1,745044346
243633_at	-	0,127732831	-1,616924751	1,744657582
222648_at	TBC1D16	0,127732831	-1,616924751	1,744657582
228758_at	BCL6	0,127732831	-1,616924751	1,744657582
205169_at	RBBP5	2,765070895	1,020514937	1,744555959
226364_at	HIP1	1,421391637	-0,322340048	1,743731685
238004_at	PGBD2	1,421391637	-0,322340048	1,743731685
211028_s_at	KHK	2,007220798	0,265475485	1,741745313
222515_x_at	TMEM165	0,319287178	-1,422257026	1,741544204
231752_at	NPBWR1	0,319287178	-1,422257026	1,741544204
1552383_at	FAM71A	0,319287178	-1,422257026	1,741544204
215682_at	LOC440792	0,458905032	-1,282520722	1,741425754
206922_at	VCY /// VCY1B	0,458905032	-1,282520722	1,741425754
216715_at	-	0,458905032	-1,282520722	1,741425754
223901_at	SYT3	-0,609040214	-2,350444759	1,741404545
1557176_a_at	C14orf37	-0,609040214	-2,350444759	1,741404545
1558398_at	ANKRD24	-0,609040214	-2,350444759	1,741404545
41387_r_at	KDM6B	1,664317239	-0,077001307	1,741318545
37547_at	BBS9	0,319521517	-1,42143293	1,740954447
226352_at	JMY	4,68949381	2,948635566	1,740858245
224682_at	ANKIB1	4,486128467	2,746644067	1,7394844
219770_at	GTDC1	3,147261986	1,408220608	1,739041378
210737_at	TUB	1,287793882	-0,45121326	1,739007142
218788_s_at	SMYD3	4,317231729	2,578283758	1,738947971
227531_at	CLOCK	2,56794744	0,829309537	1,738637903
240841_at	INSM2	0,764143511	-0,972893339	1,73703685

225915_at	CAB39L	0,764143511	-0,972893339	1,73703685
218826_at	SLC35F2	3,934348221	2,197404429	1,736943792
225238_at	MSI2	0,847529938	-0,889222211	1,736752149
206327_s_at	CDH15	0,847529938	-0,889222211	1,736752149
212874_at	APOE	-0,49284695	-2,229233437	1,736386487
208321_s_at	CABP1	-0,49284695	-2,229233437	1,736386487
221993_s_at	C16orf42	-0,49284695	-2,229233437	1,736386487
214312_at	FOXA2	-0,49284695	-2,229233437	1,736386487
220868_s_at	SLC7A10	-0,49284695	-2,229233437	1,736386487
235790_at	CTAGE5	-0,49284695	-2,229233437	1,736386487
214267_s_at	CADM4	-0,49284695	-2,229233437	1,736386487
208432_s_at	CACNA1E	-0,730013898	-2,466349414	1,736335516
214571_at	FGF3	-0,730013898	-2,466349414	1,736335516
238255_at	-	-0,730013898	-2,466349414	1,736335516
243181_at	ANKIB1	0,167727503	-1,568385657	1,73611316
207222_at	LOC100652777 ///	0,677745787	-1,057301851	1,735047638
241237_at	-	0,677745787	-1,057301851	1,735047638
211331_x_at	HFE	0,677745787	-1,057301851	1,735047638
202500_at	DNAJB2	1,671397663	-0,063377083	1,734774746
215016_x_at	DST /// LOC10065	3,936891546	2,20251271	1,734378837
225512_at	ZBTB38	4,140736098	2,406959565	1,733776533
213076_at	ITPKC	0,58788177	-1,145057014	1,732938784
229154_at	WNT10A	0,58788177	-1,145057014	1,732938784
235676_at	-	0,58788177	-1,145057014	1,732938784
228118_x_at	C16orf13	0,58788177	-1,145057014	1,732938784
202802_at	DHPS	3,509819135	1,777135134	1,732684002
237736_at	BSND	1,071336699	-0,661178575	1,732515273
228124_at	ABHD12	1,345611839	-0,386896102	1,732507941
213045_at	MAST3	1,745502609	0,013190398	1,732312211
218530_at	FHOD1	2,229377375	0,497424714	1,731952661
37433_at	PIAS2	0,476108896	-1,254408882	1,730517779
230763_at	SPATA17	-0,180990326	-1,911206569	1,730216244
204094_s_at	TSC22D2	3,493994216	1,76396664	1,730027577
221819_at	RAB35	3,610731271	1,881145546	1,729585725
203116_s_at	FECH	3,137734155	1,408220608	1,729513547
205392_s_at	CCL14 /// CCL14-C	0,355751005	-1,372734086	1,728485091
215681_at	KIAA1654	0,355751005	-1,372734086	1,728485091
239088_at	-	0,355751005	-1,372734086	1,728485091
208009_s_at	ARHGEF16	0,355751005	-1,372734086	1,728485091
208753_s_at	NAP1L1	6,198184816	4,469757829	1,728426987
223247_at	MED10	4,774699135	3,046977905	1,727721231
212371_at	DESI2	4,762900868	3,035366122	1,727534746
212132_at	LSM14A	4,732532909	3,005076416	1,727456493
229814_at	-	0,20740896	-1,520022123	1,727431084
222279_at	HLA-F-AS1	3,282538749	1,555165465	1,727373284
220768_s_at	CSNK1G3	3,783108337	2,055739171	1,727369167
221530_s_at	BHLHE41	-0,135065865	-1,862269442	1,727203577
223919_at	TP53AIP1	-0,135065865	-1,862269442	1,727203577
240862_at	RASGRP4	0,491906512	-1,234835326	1,726741838
228649_at	NOTUM	0,491906512	-1,234835326	1,726741838

234342_at	FAM20C	-0,230432956	-1,956681069	1,726248114
215491_at	MYCL1	-0,230432956	-1,956681069	1,726248114
241317_at	-	-0,230432956	-1,956681069	1,726248114
236753_at	LOC154822	-0,230432956	-1,956681069	1,726248114
218852_at	PPP2R3C	4,859875458	3,134198147	1,725677311
219785_s_at	C16orf95	2,452674045	0,727123109	1,725550936
227985_at	LOC100506098	4,05770789	2,332940758	1,724767132
242757_at	MDGA1	-0,280273599	-2,005029581	1,724755982
234912_at	-	-0,280273599	-2,005029581	1,724755982
228712_at	WNK1	-0,280273599	-2,005029581	1,724755982
229904_at	-	-0,280273599	-2,005029581	1,724755982
223458_at	SEZ6L2	-0,280273599	-2,005029581	1,724755982
230668_at	NKAIN4	-0,280273599	-2,005029581	1,724755982
229056_at	-	-0,280273599	-2,005029581	1,724755982
206816_s_at	SPAG8	-0,280273599	-2,005029581	1,724755982
225637_at	DEF8	2,031158928	0,306454867	1,724704061
230944_at	C6orf223	2,436165655	0,711934445	1,72423121
209457_at	DUSP5	0,95222556	-0,771340337	1,723565897
225338_at	ZYG11B	3,166767593	1,44320939	1,723558203
237467_at	-	0,791908897	-0,931539147	1,723448044
200673_at	LAPTM4A	6,177517926	4,454638116	1,72287981
241976_at	TCEA3	-0,088974936	-1,811780055	1,722805119
206450_at	DBH	-0,088974936	-1,811780055	1,722805119
208102_s_at	PSD	-0,088974936	-1,811780055	1,722805119
235921_at	-	-0,66934516	-2,391863529	1,722518369
1558400_x_at	ANKRD24	-0,66934516	-2,391863529	1,722518369
1560782_at	-	-0,66934516	-2,391863529	1,722518369
232706_s_at	TRABD	2,21987062	0,497424714	1,722445906
203384_s_at	GOLGA1	1,760255626	0,03784737	1,722408255
227414_at	RHBDD1	1,760255626	0,03784737	1,722408255
1567079_at	CLN6	1,20396005	-0,518232988	1,722193038
227408_s_at	SNX25	2,605420261	0,883374248	1,722046012
209379_s_at	FAM190B	2,321828846	0,600385088	1,721443758
211658_at	PRDX2	4,235548209	2,514786608	1,7207616
37590_g_at	-	2,200039202	0,479517756	1,720521446
219327_s_at	GPRC5C	-0,043925712	-1,763664074	1,719738362
1565696_at	-	-0,043925712	-1,763664074	1,719738362
213148_at	C2orf72	-0,043925712	-1,763664074	1,719738362
238325_s_at	ODF3B	-0,043925712	-1,763664074	1,719738362
213870_at	COL11A2	-0,043925712	-1,763664074	1,719738362
231018_at	PALM3	-0,043925712	-1,763664074	1,719738362
236690_at	RHBDD1	-0,043925712	-1,763664074	1,719738362
204378_at	BCAS1	2,798156197	1,078619386	1,719536811
228199_at	FAHD2B /// FAHD2	0,618277321	-1,100943374	1,719220695
217148_x_at	CKAP2 /// IGLC1	0,618277321	-1,100943374	1,719220695
222187_x_at	G3BP1	0,618277321	-1,100943374	1,719220695
234381_at	-	0,618277321	-1,100943374	1,719220695
209952_s_at	MAP2K7	-0,551284523	-2,27001641	1,718731887
207694_at	POU3F4	-0,551284523	-2,27001641	1,718731887
207134_x_at	TPSB2	-0,551284523	-2,27001641	1,718731887

209575_at	IL10RB	4,138272593	2,419753897	1,718518696
216076_at	L3MBTL1	0,39105295	-1,32740143	1,71845438
223611_s_at	LNX1	1,730929079	0,013190398	1,717738681
1553663_a_at	NPB	1,730929079	0,013190398	1,717738681
206881_s_at	LILRA3 /// LOC100	-1,06282519	-2,780225148	1,717399958
231161_x_at	-	1,094118704	-0,623254098	1,717372802
225847_at	NCEH1	1,094118704	-0,623254098	1,717372802
218801_at	UGGT2	-0,332405896	-2,049747196	1,7173413
221463_at	CCL24	-0,332405896	-2,049747196	1,7173413
231278_at	-	-0,332405896	-2,049747196	1,7173413
224739_at	PIM3	4,996753949	3,279515225	1,717238723
225956_at	CREBRF	1,544940605	-0,172194225	1,71713483
224443_at	LINC00467	3,521615383	1,804519951	1,717095433
209204_at	LMO4	2,063971513	0,346964736	1,717006777
234003_at	ENOX2	0,001114523	-1,715576125	1,716690648
242079_at	RGS12	0,001114523	-1,715576125	1,716690648
234021_at	EML2	0,001114523	-1,715576125	1,716690648
242804_at	POLN	0,001114523	-1,715576125	1,716690648
210313_at	LILRA4	0,001114523	-1,715576125	1,716690648
232620_at	WDR93	0,244681185	-1,471833228	1,716514413
1558529_s_at	LOC100508047	0,244681185	-1,471833228	1,716514413
241787_at	-	0,244681185	-1,471833228	1,716514413
226325_at	ADSSL1	0,244681185	-1,471833228	1,716514413
239386_at	HAVCR1P1	0,244681185	-1,471833228	1,716514413
236749_at	MNT	0,244681185	-1,471833228	1,716514413
219398_at	CIDEC	0,244681185	-1,471833228	1,716514413
205601_s_at	HOXB5	0,244681185	-1,471833228	1,716514413
205750_at	BPHL	2,75889404	1,043469112	1,715424928
1568623_a_at	SLC35E4	0,524545436	-1,19085694	1,715402376
217348_x_at	ARHGEF15	0,524545436	-1,19085694	1,715402376
208730_x_at	RAB2A	2,229377375	0,515457602	1,713919773
203285_s_at	HS2ST1 /// LOC33	2,138605583	0,424693306	1,713912278
212958_x_at	PAM	4,116324812	2,403191094	1,713133718
229517_at	PTPDC1	2,019346222	0,306454867	1,712891355
209635_at	AP1S1	4,022766247	2,309925448	1,712840799
224788_at	ARF6	5,975811812	4,263096467	1,712715346
1556872_s_at	IQSEC3 /// LOC10	-0,384193355	-2,09678811	1,712594755
205899_at	CCNA1	-0,384193355	-2,09678811	1,712594755
234818_at	TMEM108	-0,384193355	-2,09678811	1,712594755
211407_at	NDUFB7	-0,384193355	-2,09678811	1,712594755
206564_at	OPRL1	-0,384193355	-2,09678811	1,712594755
223177_at	NT5DC1	4,550683059	2,83813104	1,712552019
218217_at	SCPEP1	3,228532861	1,516067292	1,71246557
202562_s_at	C14orf1	4,839270685	3,127281098	1,711989586
230322_at	NFAM1	0,900065578	-0,811769547	1,711835125
230075_at	RAB39B	2,824160383	1,112548615	1,711611768
231361_at	NLGN1	-0,924000698	-2,635549085	1,711548387
1552370_at	C4orf33	2,56794744	0,85655369	1,71139375
201787_at	FBLN1	0,045517965	-1,665791776	1,711309741
240025_x_at	-	0,045517965	-1,665791776	1,711309741

216685_s_at	MTAP	0,045517965	-1,665791776	1,711309741
242159_at	LOC100506684	0,045517965	-1,665791776	1,711309741
222646_s_at	ERO1L	5,012877957	3,301884701	1,710993255
201545_s_at	BCL2L2-PABPN1 //	3,218770562	1,508107415	1,710663146
207183_at	GPR19	3,273854609	1,563256447	1,710598162
1553007_a_at	ODZ1	-0,791294935	-2,501359113	1,710064178
204582_s_at	KLK3	-0,791294935	-2,501359113	1,710064178
214230_at	CDC42	1,910557155	0,20062106	1,709936096
217776_at	RDH11	5,892935274	4,183386016	1,709549258
237882_at	-	0,820160788	-0,889222211	1,709382999
231963_at	ANKRD33B	3,577387089	1,868130962	1,709256128
212254_s_at	DST /// LOC10065	3,668220666	1,9592579	1,708962767
202255_s_at	SIPA1L1	1,745502609	0,03784737	1,707655238
244049_at	-	0,425036312	-1,282520722	1,707557035
228197_at	C1orf86	0,425036312	-1,282520722	1,707557035
215485_s_at	ICAM1	0,425036312	-1,282520722	1,707557035
217041_at	NPTXR	1,476175815	-0,231045907	1,707221722
230685_at	FLJ33630	2,053550345	0,346964736	1,706585609
206855_s_at	HYAL2	2,053550345	0,346964736	1,706585609
224696_s_at	DCAF5	1,287793882	-0,418248858	1,70604274
202393_s_at	KLF10	4,26103332	2,55501164	1,706021679
226857_at	ARHGEF19	1,383446753	-0,322340048	1,7057868
1559205_s_at	-	0,648195588	-1,057301851	1,705497439
205774_at	F12	2,62869474	0,923203038	1,705491701
238167_at	C17orf51 /// FAM	1,116462765	-0,589001171	1,705463936
235639_at	CDH19	-0,437449947	-2,142286822	1,704836874
202285_s_at	TACSTD2	-0,437449947	-2,142286822	1,704836874
229320_at	C2orf55	-0,437449947	-2,142286822	1,704836874
231128_at	FAM181B	-0,437449947	-2,142286822	1,704836874
241822_at	-	0,282358733	-1,422257026	1,704615759
217661_x_at	SIX5	0,282358733	-1,422257026	1,704615759
229608_at	FAM212B	0,282358733	-1,422257026	1,704615759
220894_x_at	PRDM12	0,282358733	-1,422257026	1,704615759
204848_x_at	HBG1 /// HBG2 ///	0,282358733	-1,422257026	1,704615759
206827_s_at	TRPV6	0,282358733	-1,422257026	1,704615759
202284_s_at	CDKN1A	2,771643417	1,067365565	1,704277852
200081_s_at	RPS6	8,461535343	6,757833832	1,703701511
1560916_a_at	DPY19L1	1,640193838	-0,063377083	1,703570921
228024_at	VPS37A	2,127965541	0,424693306	1,703272235
244085_at	ZNF653	0,08619576	-1,616924751	1,703120511
210150_s_at	LAMA5	0,08619576	-1,616924751	1,703120511
220433_at	PRRG3	0,08619576	-1,616924751	1,703120511
220186_s_at	CDHR2	0,08619576	-1,616924751	1,703120511
234349_at	SSPO	0,08619576	-1,616924751	1,703120511
232928_at	-	-0,609040214	-2,312054429	1,703014215
1552854_a_at	VWA5B1	-0,609040214	-2,312054429	1,703014215
208587_s_at	OR1E1 /// OR1E2	-0,609040214	-2,312054429	1,703014215
205668_at	LY75	2,804738618	1,101780212	1,702958406
218023_s_at	FAM53C	3,614400403	1,911502437	1,702897966
213341_at	FEM1C	2,444186596	0,742671819	1,701514777

241277_x_at	ZNF837	0,556428218	-1,145057014	1,701485232
205685_at	CD86	0,556428218	-1,145057014	1,701485232
1555974_a_at	-	2,530390387	0,829309537	1,70108085
215903_s_at	MAST2	1,92317941	0,222432814	1,700746596
238529_at	-	1,528073958	-0,172194225	1,700268183
202210_x_at	GSK3A	1,528073958	-0,172194225	1,700268183
1560439_at	LINGO3	1,610353504	-0,089635713	1,699989217
224912_at	TTC7A	2,484824532	0,786391549	1,698432983
231345_s_at	DHRS12	-0,730013898	-2,4284334	1,698419502
203078_at	CUL2	0,926325262	-0,771340337	1,697665599
239431_at	TICAM2 /// TMED7	0,926325262	-0,771340337	1,697665599
226574_at	PSPC1	4,070902947	2,373249966	1,697652981
214428_x_at	C4A /// C4B /// LO	1,000459215	-0,697182596	1,697641811
1553826_a_at	C16orf11	1,000459215	-0,697182596	1,697641811
219766_at	B9D2	1,246401134	-0,45121326	1,697614393
235218_x_at	THAP6	1,246401134	-0,45121326	1,697614393
213809_x_at	TCF3	0,847529938	-0,84928999	1,696819928
215490_at	IBA57	0,847529938	-0,84928999	1,696819928
215701_at	-	0,847529938	-0,84928999	1,696819928
216439_at	TNK2	0,847529938	-0,84928999	1,696819928
236454_at	RNF212	0,847529938	-0,84928999	1,696819928
211328_x_at	HFE	0,847529938	-0,84928999	1,696819928
221164_x_at	CHST5	0,127732831	-1,568385657	1,696118487
236541_at	ARSA	0,127732831	-1,568385657	1,696118487
205819_at	MARCO	0,127732831	-1,568385657	1,696118487
201871_s_at	UBXN1	5,4071539	3,711313563	1,695840336
1566454_at	LOC100507494	0,764143511	-0,931539147	1,695682658
221100_at	C6orf15	0,764143511	-0,931539147	1,695682658
228587_at	FAM83G	0,764143511	-0,931539147	1,695682658
227941_at	LOC339803	3,996262843	2,300902761	1,695360082
224092_at	BARHL1	1,071336699	-0,623254098	1,694590797
205946_at	VIPR2	1,071336699	-0,623254098	1,694590797
201894_s_at	SSR1	4,847889357	3,153389283	1,694500074
219847_at	HDAC11	1,307201325	-0,386896102	1,694097427
206220_s_at	RASA3	1,139057614	-0,554696666	1,69375428
205561_at	KCTD17	1,139057614	-0,554696666	1,69375428
217367_s_at	ZHX3	0,458905032	-1,234835326	1,693740358
239300_at	PIK3C3	0,458905032	-1,234835326	1,693740358
210226_at	NR4A1	0,458905032	-1,234835326	1,693740358
235908_at	MMP11	0,458905032	-1,234835326	1,693740358
227962_at	ACOX1	2,590269697	0,896834102	1,693435595
243278_at	FOXP2	1,871521385	0,178191865	1,69332952
244155_x_at	TOR2A	-0,49284695	-2,186086603	1,693239653
236490_at	NEDD4L	1,402495885	-0,290694975	1,69319086
240897_at	-	0,677745787	-1,015398016	1,693143803
201064_s_at	PABPC4	6,004173488	4,311836632	1,692336856
226700_at	U2AF1L4	1,65554524	-0,036543687	1,692088927
1562211_a_at	ZNF491	0,319287178	-1,372734086	1,692021264
1570078_a_at	DOCK5	0,319287178	-1,372734086	1,692021264
237774_at	PENK	0,319287178	-1,372734086	1,692021264

1553813_s_at	TLE6	0,319287178	-1,372734086	1,692021264
1564658_at	NAT16	0,319287178	-1,372734086	1,692021264
224810_s_at	ANKRD13A	5,81676174	4,124822547	1,691939193
225385_s_at	HNRPLL	3,322661262	1,630734622	1,69192664
216976_s_at	RYK	1,544940605	-0,144765583	1,689706188
227639_at	PIGK	3,156891301	1,46719264	1,689698662
225289_at	STAT3	3,370643409	1,680963921	1,689679489
225411_at	TMEM87B	3,132805465	1,44320939	1,689596075
224347_x_at	UBE2J2	1,775035811	0,085534992	1,689500819
226519_s_at	AGXT2L2	2,685337919	0,996453274	1,688884645
207547_s_at	FAM107A /// LOC1	0,58788177	-1,100943374	1,688825144
203144_s_at	KIAA0040	0,58788177	-1,100943374	1,688825144
244182_at	-	0,58788177	-1,100943374	1,688825144
208176_at	DUX1	1,20396005	-0,484558493	1,688518543
203538_at	CAMLG	5,399531502	3,711313563	1,688217939
236399_at	-	0,167727503	-1,520022123	1,687749627
222890_at	CCDC113	0,167727503	-1,520022123	1,687749627
216046_at	-	0,167727503	-1,520022123	1,687749627
239112_at	-	0,167727503	-1,520022123	1,687749627
228269_x_at	KCNIP3	0,167727503	-1,520022123	1,687749627
202250_s_at	DCAF8	2,597647262	0,910483921	1,687163341
212356_at	KHNYN	3,265594257	1,578991006	1,686603251
221053_s_at	TDRKH	0,95222556	-0,734355396	1,686580956
210130_s_at	TM7SF2	2,444186596	0,757638486	1,68654811
203455_s_at	SAT1	4,886957485	3,20060887	1,686348615
227199_at	DIP2A	3,509819135	1,823960141	1,685858995
205376_at	INPP4B	3,233229368	1,547376668	1,6858527
235911_at	MFI2	3,233229368	1,547376668	1,6858527
35820_at	GM2A	2,879034131	1,193392819	1,685641312
1558148_x_at	FLJ90757	0,873789652	-0,811769547	1,685559199
241800_x_at	-	1,024284941	-0,661178575	1,685463516
223237_x_at	AP2A1	1,024284941	-0,661178575	1,685463516
231542_at	SPAG5-AS1	1,267146969	-0,418248858	1,685395827
202623_at	EAPP	4,027733188	2,34250725	1,685225938
222553_x_at	OXR1	4,228647054	2,543655878	1,684991176
218224_at	PNMA1	4,179494373	2,49476365	1,684730723
225352_at	SEC62	3,966733349	2,283169614	1,683563735
1568605_at	JRK	0,355751005	-1,32740143	1,683152435
232536_at	LRRC3DN	0,355751005	-1,32740143	1,683152435
210977_s_at	HSF4	1,094118704	-0,589001171	1,683119875
222048_at	CRYBB2P1	1,094118704	-0,589001171	1,683119875
221756_at	PIK3IP1	2,552836808	0,869824768	1,68301204
216426_at	-	0,491906512	-1,19085694	1,682763452
227345_at	TNFRSF10D	0,491906512	-1,19085694	1,682763452
210240_s_at	CDKN2D	3,261582786	1,578991006	1,682591781
212454_x_at	HNRPDL	4,653725229	2,971193817	1,682531412
233234_at	KCTD16	-0,180990326	-1,862269442	1,681279116
1560977_a_at	BCL2L13	-0,180990326	-1,862269442	1,681279116
203541_s_at	KLF9	-0,180990326	-1,862269442	1,681279116
219042_at	LZTS1	-0,180990326	-1,862269442	1,681279116

214399_s_at	KRT4	-0,180990326	-1,862269442	1,681279116
206213_at	WNT10B	0,791908897	-0,889222211	1,681131108
1566086_at	-	-0,66934516	-2,350444759	1,6810996
232851_at	FBXO3	-0,66934516	-2,350444759	1,6810996
234416_at	-	-0,66934516	-2,350444759	1,6810996
209179_s_at	MBOAT7	3,107338728	1,426280426	1,681058302
217034_at	-	-0,230432956	-1,911206569	1,680773614
231388_at	-	-0,230432956	-1,911206569	1,680773614
216270_at	ILVBL	-0,230432956	-1,911206569	1,680773614
205477_s_at	AMBP	-0,230432956	-1,911206569	1,680773614
243053_x_at	LOC100128079	-0,230432956	-1,911206569	1,680773614
238345_at	SLC38A10	-0,230432956	-1,911206569	1,680773614
243079_x_at	-	-0,230432956	-1,911206569	1,680773614
226344_at	ZMAT1	0,707657549	-0,972893339	1,680550887
219675_s_at	UXS1	4,191105499	2,5107204	1,680385098
207580_at	MAGEB4	-0,856259172	-2,536339282	1,68008011
204664_at	ALPP	-0,856259172	-2,536339282	1,68008011
221884_at	MECOM	-0,856259172	-2,536339282	1,68008011
213505_s_at	SUGP2	3,147261986	1,46719264	1,680069346
214780_s_at	MYO9B	1,857989965	0,178191865	1,6797981
1561401_at	LOC285627	-0,924000698	-2,603471267	1,679470569
224286_at	C1QTNF5 /// MFRI	1,788922638	0,109458907	1,679463731
229308_at	ANKRD29	0,20740896	-1,471833228	1,679242188
234177_at	-	0,20740896	-1,471833228	1,679242188
226856_at	MUSTN1 /// TMEN	0,20740896	-1,471833228	1,679242188
206894_at	APOA4	0,20740896	-1,471833228	1,679242188
231268_at	LOC645895 /// MY	0,20740896	-1,471833228	1,679242188
211142_x_at	HLA-DOA	0,20740896	-1,471833228	1,679242188
203104_at	CSF1R	0,20740896	-1,471833228	1,679242188
226577_at	PSEN1	2,507817181	0,829309537	1,678507644
235891_at	DNM3OS /// MIR2	-0,551284523	-2,229233437	1,677948915
207969_x_at	ACRV1	-0,551284523	-2,229233437	1,677948915
1567320_at	-	-0,551284523	-2,229233437	1,677948915
232081_at	-	-0,551284523	-2,229233437	1,677948915
202801_at	PRKACA	1,640193838	-0,036543687	1,676737525
238767_at	-	-0,135065865	-1,811780055	1,67671419
240347_at	-	-0,135065865	-1,811780055	1,67671419
231834_at	RBM33	-0,135065865	-1,811780055	1,67671419
230671_at	-	-0,135065865	-1,811780055	1,67671419
201044_x_at	DUSP1	-0,135065865	-1,811780055	1,67671419
233983_at	TGM6	-0,135065865	-1,811780055	1,67671419
1563252_at	ERBB3	-0,135065865	-1,811780055	1,67671419
209874_x_at	CNNM2	-0,135065865	-1,811780055	1,67671419
212606_at	WDFY3	-0,135065865	-1,811780055	1,67671419
243133_at	TSC22D1	-0,135065865	-1,811780055	1,67671419
220015_at	CASZ1	2,209870035	0,533258442	1,676611594
223131_s_at	TRIM8	-0,280273599	-1,956681069	1,67640747
239335_at	ZNF710	-0,280273599	-1,956681069	1,67640747
206516_at	AMH	-0,280273599	-1,956681069	1,67640747
242211_x_at	WDR90	-0,280273599	-1,956681069	1,67640747

213417_at	TBX2	-0,280273599	-1,956681069	1,67640747
244834_at	RSG1	1,225123479	-0,45121326	1,676336739
212135_s_at	ATP2B4	3,657457797	1,981728801	1,675728996
207685_at	CRYBB3	0,618277321	-1,057301851	1,675579172
226944_at	HTRA3	0,618277321	-1,057301851	1,675579172
237801_at	-	-0,791294935	-2,466349414	1,675054479
207084_at	POU3F2	-0,791294935	-2,466349414	1,675054479
1555905_a_at	C3orf23	1,830468611	0,155747281	1,674721331
1557167_at	HCG11	1,760255626	0,085534992	1,674720633
209512_at	HSDL2	3,975619308	2,300902761	1,674716547
236423_at	-	-0,088974936	-1,763664074	1,674689139
237840_at	LOC388948	-0,088974936	-1,763664074	1,674689139
210100_s_at	ABCA2	-0,088974936	-1,763664074	1,674689139
227681_at	ZFP36L2	-0,088974936	-1,763664074	1,674689139
225320_at	MCU	2,460890032	0,786391549	1,674498483
232937_at	-	1,383446753	-0,290694975	1,674141728
213066_at	RUSC2	0,39105295	-1,282520722	1,673573673
243738_at	NMNAT3	0,39105295	-1,282520722	1,673573673
1553174_at	JPH2	0,39105295	-1,282520722	1,673573673
212234_at	ASXL1	3,045068376	1,371634653	1,673433723
206508_at	CD70	4,244034996	2,570802332	1,673232664
233647_s_at	CDADC1	3,296548503	1,623485524	1,673062979
204163_at	EMILIN1	-0,332405896	-2,005029581	1,672623685
211673_s_at	MOCS1	-0,332405896	-2,005029581	1,672623685
232488_at	AGXT2L2	2,582572508	0,910483921	1,672088587
232096_x_at	FOXP1-IT1	-0,043925712	-1,715576125	1,671650413
228552_s_at	ISYNA1	-0,043925712	-1,715576125	1,671650413
226100_at	MLL5	3,681878794	2,010406245	1,671472549
215670_s_at	SCAND2	0,900065578	-0,771340337	1,671405915
225709_at	ARL6IP6	0,900065578	-0,771340337	1,671405915
215548_s_at	SCFD1	4,228647054	2,558652146	1,669994908
242569_at	STAM2	0,524545436	-1,145057014	1,669602451
215844_at	TNPO2	0,524545436	-1,145057014	1,669602451
225111_s_at	NAPB	2,552836808	0,883374248	1,66946256
235827_at	LOC100506472 ///	0,820160788	-0,84928999	1,669450778
212907_at	SLC30A1	4,416093706	2,746644067	1,669449638
224783_at	FAM100B	4,891443931	3,222133192	1,669310739
212605_s_at	-	3,223556135	1,555165465	1,66839067
211991_s_at	HLA-DPA1	6,663402768	4,995602626	1,667800141
243604_at	-	0,244681185	-1,422257026	1,666938212
228800_x_at	AURKAIP1	0,244681185	-1,422257026	1,666938212
239993_at	LOC390705 /// LO	0,244681185	-1,422257026	1,666938212
221717_at	-	0,001114523	-1,665791776	1,666906299
204443_at	ARSA	0,001114523	-1,665791776	1,666906299
244617_at	GPR26	0,001114523	-1,665791776	1,666906299
227930_at	EIF2C4	3,252865595	1,586121802	1,666743794
217795_s_at	TMEM43	4,221194173	2,55501164	1,666182533
224357_s_at	MS4A4A	-0,384193355	-2,049747196	1,665553841
217283_at	-	-0,384193355	-2,049747196	1,665553841
1554735_a_at	TRIM7	-0,384193355	-2,049747196	1,665553841

232624_at	ABTB2	-0,384193355	-2,049747196	1,665553841
230275_at	ARSI	-0,384193355	-2,049747196	1,665553841
225151_at	RTKN	-0,384193355	-2,049747196	1,665553841
226406_at	C18orf25	2,943764123	1,278654284	1,66510984
202955_s_at	ARFGEF1	2,575362572	0,910483921	1,664878651
36564_at	RNF19B	1,561517414	-0,103243933	1,664761346
209448_at	HTATIP2	6,069496364	4,405241507	1,664254857
215280_s_at	PPFIA3	0,648195588	-1,015398016	1,663593604
213171_s_at	MMP24	0,648195588	-1,015398016	1,663593604
225478_at	MFHAS1	4,679156164	3,01591662	1,663239544
242594_at	BOD1L1	0,045517965	-1,616924751	1,662442716
1553235_at	PCDHAC1	0,045517965	-1,616924751	1,662442716
233589_x_at	TOR4A	0,045517965	-1,616924751	1,662442716
239925_at	-	0,045517965	-1,616924751	1,662442716
205611_at	TNFSF12 /// TNFSF	0,045517965	-1,616924751	1,662442716
202988_s_at	RGS1	4,669720908	3,007560601	1,662160307
227590_at	C22orf40	1,625482993	-0,036543687	1,66202668
212253_x_at	DST /// LOC10065:	1,307201325	-0,354781582	1,661982907
204729_s_at	STX1A	1,307201325	-0,354781582	1,661982907
218910_at	ANO10	1,307201325	-0,354781582	1,661982907
234406_at	RGMA	-0,730013898	-2,391863529	1,661849631
220312_at	FAM83E	-0,730013898	-2,391863529	1,661849631
222423_at	NDFIP1	3,498020785	1,836262316	1,661758468
214223_at	-	1,000459215	-0,661178575	1,661637789
226978_at	PPARA	1,816938997	0,155747281	1,661191717
202704_at	TOB1	4,778036024	3,117017558	1,661018467
236917_at	LRRC34	-0,609040214	-2,27001641	1,660976196
1558847_at	LINC00565	-0,609040214	-2,27001641	1,660976196
221106_at	SLC22A17	-0,609040214	-2,27001641	1,660976196
205724_at	PKP1	-0,609040214	-2,27001641	1,660976196
230126_s_at	KDM4B	-0,609040214	-2,27001641	1,660976196
234002_at	ABHD16B	-0,609040214	-2,27001641	1,660976196
207726_at	ESRRB	-0,609040214	-2,27001641	1,660976196
209544_at	RIPK2	0,926325262	-0,734355396	1,660680658
234999_at	C19orf47	0,926325262	-0,734355396	1,660680658
220538_at	ADM2	1,071336699	-0,589001171	1,66033787
212285_s_at	AGRN	1,071336699	-0,589001171	1,66033787
202419_at	KDSR	3,726612406	2,066329863	1,660282543
220012_at	ERO1LB	1,458255986	-0,201789721	1,660045707
223241_at	SNX8	0,425036312	-1,234835326	1,659871639
1552627_a_at	ARHGAP5	0,425036312	-1,234835326	1,659871639
207719_x_at	CEP170	5,105521533	3,445797592	1,659723941
1558470_at	VWA3B	-0,437449947	-2,09678811	1,659338162
243978_at	FAM65C	-0,437449947	-2,09678811	1,659338162
203876_s_at	MMP11	-0,437449947	-2,09678811	1,659338162
219503_s_at	TMEM40	-0,437449947	-2,09678811	1,659338162
235499_at	LOC728743	-0,437449947	-2,09678811	1,659338162
242435_at	PDE4A	-0,437449947	-2,09678811	1,659338162
1554867_a_at	PRR16	-0,437449947	-2,09678811	1,659338162
238405_at	-	-0,437449947	-2,09678811	1,659338162

206544_x_at	SMARCA2	4,214272326	2,55501164	1,659260686
227065_at	RNF216	1,92317941	0,265475485	1,657703925
214957_at	ACTL8	0,556428218	-1,100943374	1,657371592
240342_at	TRIM61	1,139057614	-0,518232988	1,657290602
224492_s_at	ZNF627	2,955426542	1,298349073	1,657077468
244730_x_at	LOC100129195	1,364555912	-0,290694975	1,655250887
236702_at	-	0,282358733	-1,372734086	1,655092819
222928_s_at	FAM160B2	0,08619576	-1,568385657	1,654581416
213533_at	D4S234E	0,08619576	-1,568385657	1,654581416
210051_at	RAPGEF3	0,08619576	-1,568385657	1,654581416
235106_at	MAML2	0,08619576	-1,568385657	1,654581416
211444_at	-	1,716003175	0,061773582	1,654229592
1555697_at	KLK4	1,716003175	0,061773582	1,654229592
230947_at	41153	0,764143511	-0,889222211	1,653365722
243887_at	MRPL30	2,410823487	0,757638486	1,653185
209004_s_at	FBXL5	4,458703813	2,805545859	1,653157954
1554452_a_at	HILPDA	5,741384422	4,090064274	1,651320148
224718_at	YY1	4,049824219	2,398910172	1,650914047
218661_at	NAA60	1,760255626	0,109458907	1,650796719
1552607_at	FAM223A	0,677745787	-0,972893339	1,650639126
221655_x_at	EPS8L1	0,677745787	-0,972893339	1,650639126
222784_at	SMOC1	0,677745787	-0,972893339	1,650639126
218172_s_at	DERL1	4,267962641	2,617393502	1,650569139
208925_at	CLDND1	5,751266263	4,101453764	1,649812499
212972_x_at	APBB2	0,458905032	-1,19085694	1,649761971
234880_x_at	KRTAP1-3	0,458905032	-1,19085694	1,649761971
226982_at	ELL2	3,614400403	1,964670566	1,649729837
1564584_at	LOC100506557	-0,49284695	-2,142286822	1,649439871
205635_at	KALRN	-0,49284695	-2,142286822	1,649439871
227845_s_at	SHD	-0,49284695	-2,142286822	1,649439871
217217_at	IGHA1 /// IGHA2 /,	-0,49284695	-2,142286822	1,649439871
202986_at	ARNT2	-0,49284695	-2,142286822	1,649439871
234891_at	DKFZP547L112	-0,49284695	-2,142286822	1,649439871
228762_at	LFNG	1,871521385	0,222432814	1,649088571
1559926_at	LOC728353	0,127732831	-1,520022123	1,647754954
1556773_at	-	0,127732831	-1,520022123	1,647754954
221271_at	IL21	0,127732831	-1,520022123	1,647754954
206093_x_at	TNXA /// TNXB	0,127732831	-1,520022123	1,647754954
216272_x_at	SYDE1	1,024284941	-0,623254098	1,647539039
65770_at	RHOT2	2,949795736	1,302688568	1,647107168
225576_at	C6orf72	5,100432184	3,453397238	1,647034946
233380_s_at	RUFY1	2,530390387	0,883374248	1,647016138
240113_at	-	0,319287178	-1,32740143	1,646688608
231336_at	CPNE4	-0,924000698	-2,570522741	1,646522044
207965_at	NEUROG3	-0,924000698	-2,570522741	1,646522044
231030_at	ZRANB2-AS1	-0,924000698	-2,570522741	1,646522044
208931_s_at	ILF3	3,362258367	1,715863686	1,646394682
218019_s_at	PDXK	2,51558601	0,869824768	1,645761241
211500_at	MAPK11	2,031158928	0,385582632	1,645576296
1552286_at	ATP6V1E2 /// FLJ4	2,031158928	0,385582632	1,645576296

217825_s_at	UBE2J1	3,928402242	2,283169614	1,645232628
230719_at	ING3	0,58788177	-1,057301851	1,645183621
237109_at	TOM1L2	0,58788177	-1,057301851	1,645183621
206807_s_at	ADD2	0,58788177	-1,057301851	1,645183621
231263_at	ARMC12	0,873789652	-0,771340337	1,645129989
233068_at	-	0,873789652	-0,771340337	1,645129989
1556014_at	MESP2	0,873789652	-0,771340337	1,645129989
213065_at	ZFC3H1	3,493994216	1,848871029	1,645123188
1557740_a_at	-	-0,856259172	-2,501359113	1,645099941
211108_s_at	JAK3	-0,856259172	-2,501359113	1,645099941
204784_s_at	MLF1	3,792209911	2,147194726	1,645015185
202653_s_at	07.03.15	4,45671768	2,812515887	1,644201793
209524_at	HDGFRP3	3,694479488	2,050286491	1,644192997
224880_at	RALA	5,446817871	3,802659695	1,644158175
203299_s_at	AP1S2	3,473999051	1,830129603	1,643869448
238770_at	KIAA1456	-0,66934516	-2,312054429	1,64270927
223541_at	HAS3	-0,66934516	-2,312054429	1,64270927
1566803_at	-	-0,66934516	-2,312054429	1,64270927
215166_at	IGSF9B	-0,66934516	-2,312054429	1,64270927
221001_at	-	-0,66934516	-2,312054429	1,64270927
227038_at	SGMS2	-0,66934516	-2,312054429	1,64270927
205042_at	GNE	3,378744007	1,736450458	1,642293549
226729_at	USP37	1,578719607	-0,063377083	1,642096689
217741_s_at	ZFAND5	4,550683059	2,908944587	1,641738472
223617_x_at	ATAD3B	3,117098466	1,475630127	1,641468338
236504_x_at	C6orf52	0,791908897	-0,84928999	1,641198887
219060_at	WDYHV1	4,510408465	2,869317249	1,641091216
217673_x_at	GNAS	4,698307213	3,05768517	1,640622043
230306_at	VPS26B	2,25755351	0,61779983	1,63975368
1566038_at	DGCR7	0,167727503	-1,471833228	1,639560731
230513_at	BAG6	0,167727503	-1,471833228	1,639560731
235208_at	CCDC112	0,167727503	-1,471833228	1,639560731
220126_at	PRSS50	0,167727503	-1,471833228	1,639560731
218821_at	NPEPL1 /// STX16-	0,707657549	-0,931539147	1,639196696
202402_s_at	CARS	4,133352155	2,49476365	1,638588505
210628_x_at	LTBP4	0,355751005	-1,282520722	1,638271727
1555833_a_at	IRGQ	2,507817181	0,869824768	1,637992413
238406_x_at	SEZ6L2	0,97663406	-0,661178575	1,637812634
220016_at	AHNAK	2,739067176	1,101780212	1,637286964
241255_at	-	-0,791294935	-2,4284334	1,637138465
220579_at	-	-0,791294935	-2,4284334	1,637138465
244850_at	-	-0,791294935	-2,4284334	1,637138465
226024_at	COMMD1	4,971128895	3,334057854	1,637071042
220951_s_at	A1CF	0,491906512	-1,145057014	1,636963526
242651_at	-	0,491906512	-1,145057014	1,636963526
1570330_at	-	0,491906512	-1,145057014	1,636963526
202163_s_at	CNOT8	3,969866565	2,332940758	1,636925807
1563549_a_at	ANO8	1,047341799	-0,589001171	1,63634297
224466_s_at	MAFG	1,345611839	-0,290694975	1,636306814
1555366_at	NSAP11	-0,551284523	-2,186086603	1,63480208

237296_at	-	-0,551284523	-2,186086603	1,63480208
207991_x_at	ACRV1	-0,551284523	-2,186086603	1,63480208
216847_at	-	-0,551284523	-2,186086603	1,63480208
238492_at	-	-0,551284523	-2,186086603	1,63480208
1552877_s_at	LINC00334	-0,551284523	-2,186086603	1,63480208
226192_at	AR	-0,551284523	-2,186086603	1,63480208
242503_at	CHST13	-0,551284523	-2,186086603	1,63480208
222469_s_at	TOLLIP	0,900065578	-0,734355396	1,634420974
229142_s_at	BBS1	0,900065578	-0,734355396	1,634420974
227095_at	LEPROT	2,476788561	0,842599219	1,634189341
209920_at	BMPR2	1,182925501	-0,45121326	1,634138761
204156_at	SIK3	1,182925501	-0,45121326	1,634138761
207827_x_at	SNCA	1,182925501	-0,45121326	1,634138761
203761_at	SLA	4,582263053	2,948635566	1,633627487
230142_s_at	CIRBP	1,402495885	-0,231045907	1,633541791
218010_x_at	PPDPF	1,246401134	-0,386896102	1,633297236
202812_at	GAA	1,246401134	-0,386896102	1,633297236
218175_at	CCDC92	3,349017424	1,715863686	1,633153738
1554755_a_at	MTUS2	-1,06282519	-2,695640852	1,632815662
205170_at	STAT2	0,820160788	-0,811769547	1,631930335
222842_at	EIF2C4	0,820160788	-0,811769547	1,631930335
232495_x_at	-	0,820160788	-0,811769547	1,631930335
226523_at	TAGLN	0,820160788	-0,811769547	1,631930335
215395_x_at	PRSS3P2	-0,230432956	-1,862269442	1,631836486
234379_at	FLT4	-0,230432956	-1,862269442	1,631836486
205853_at	ZBTB7B	-0,230432956	-1,862269442	1,631836486
240350_at	-	-0,230432956	-1,862269442	1,631836486
1570241_at	SPATA21	-0,230432956	-1,862269442	1,631836486
218872_at	TESC	3,039689025	1,408220608	1,631468417
203026_at	ZBTB5	3,422378845	1,790914523	1,631464322
210452_x_at	CYP4F2 /// CYP4F3	-0,280273599	-1,911206569	1,63093297
208465_at	GRM2	-0,280273599	-1,911206569	1,63093297
214798_at	ATP2C2	-0,280273599	-1,911206569	1,63093297
229489_at	-	-0,280273599	-1,911206569	1,63093297
219195_at	PPARGC1A	-0,180990326	-1,811780055	1,630789729
239952_at	ZEB1	-0,180990326	-1,811780055	1,630789729
1556356_at	ERICH1	-0,180990326	-1,811780055	1,630789729
236091_at	HMGB2	-0,180990326	-1,811780055	1,630789729
230726_at	MRPL38	-0,180990326	-1,811780055	1,630789729
208020_s_at	CACNA1C	-0,180990326	-1,811780055	1,630789729
215741_x_at	AKAP8L	1,716003175	0,085534992	1,630468182
219435_at	CTC1	2,248155141	0,61779983	1,630355311
219664_s_at	DECR2	2,908690075	1,278654284	1,630035791
235309_at	RPS15A	3,161901883	1,531877749	1,630024134
202784_s_at	NNT	3,161901883	1,531877749	1,630024134
227113_at	ADHFE1	1,936380191	0,306454867	1,629925324
201526_at	ARF5	4,239531398	2,609615156	1,629916242
214440_at	NAT1	4,0992582	2,469496389	1,629761811
219796_s_at	CDHR5	0,20740896	-1,422257026	1,629665987
211824_x_at	NLRP1	2,356619952	0,727123109	1,629496843

221344_at	OR12D2	-0,135065865	-1,763664074	1,628598209
244751_at	PCP2	-0,135065865	-1,763664074	1,628598209
206338_at	ELAVL3	-0,135065865	-1,763664074	1,628598209
235045_at	RBM7	2,107136847	0,479089184	1,628047662
1569171_a_at	-	-0,088974936	-1,715576125	1,626601189
219508_at	GCNT3	-0,088974936	-1,715576125	1,626601189
237714_at	-	-0,088974936	-1,715576125	1,626601189
207418_s_at	DDO	-0,088974936	-1,715576125	1,626601189
224672_x_at	MESDC2	-0,088974936	-1,715576125	1,626601189
210411_s_at	GRIN2B	-0,088974936	-1,715576125	1,626601189
242378_at	MYADML2	-0,088974936	-1,715576125	1,626601189
244707_at	-	-0,088974936	-1,715576125	1,626601189
218774_at	DCPS	4,586396217	2,960011739	1,626384478
205078_at	PIGF	4,288236521	2,662083808	1,626152713
230633_at	TMEM102	0,39105295	-1,234835326	1,625888277
1553872_at	C17orf103	0,39105295	-1,234835326	1,625888277
209769_s_at	SEPT5-GP1BB	0,524545436	-1,100943374	1,62548881
232787_at	PRIC285	0,735999505	-0,889222211	1,625221716
243196_s_at	TRAFD1	1,686984721	0,061773582	1,625211139
222409_at	CORO1C	4,744801376	3,119733444	1,625067932
237411_at	ADAMTS6	-0,332405896	-1,956681069	1,624275174
1570349_at	-	-0,332405896	-1,956681069	1,624275174
220074_at	CDHR5	-0,332405896	-1,956681069	1,624275174
206705_at	TULP1	-0,332405896	-1,956681069	1,624275174
233299_at	RIMS4	-0,332405896	-1,956681069	1,624275174
229072_at	RAB30	-0,332405896	-1,956681069	1,624275174
215353_at	-	-0,332405896	-1,956681069	1,624275174
218551_at	MIIP	3,107338728	1,483698568	1,62364016
224271_x_at	FRMD8P1	1,139057614	-0,484558493	1,623616108
240172_at	ERGIC2	0,926325262	-0,697182596	1,623507859
229774_at	CXXC4	2,25755351	0,634301128	1,623252382
243477_at	ATG10	1,421391637	-0,201789721	1,623181358
202743_at	PIK3R3	3,754428453	2,132145748	1,622282705
218167_at	AMZ2	6,05484923	4,432698346	1,622150884
238582_at	C21orf2	-0,043925712	-1,665791776	1,621866064
217046_s_at	AGER	-0,043925712	-1,665791776	1,621866064
226942_at	PHF20L1	3,237994092	1,616354037	1,621640055
220602_s_at	AGSK1 /// LOC388	1,730929079	0,109458907	1,621470172
36829_at	PER1	1,235815484	-0,385338132	1,621153616
213807_x_at	MET	0,648195588	-0,972893339	1,621088927
204519_s_at	PLLP	0,648195588	-0,972893339	1,621088927
216842_x_at	-	0,648195588	-0,972893339	1,621088927
238022_at	CRNDE	3,768156719	2,147194726	1,620961992
213280_at	RAP1GAP2	-0,384193355	-2,005029581	1,620836226
236317_at	-	-0,384193355	-2,005029581	1,620836226
206604_at	OVOL1	-0,384193355	-2,005029581	1,620836226
215176_x_at	LOC642838	-0,384193355	-2,005029581	1,620836226
205779_at	RAMP2	-0,384193355	-2,005029581	1,620836226
215689_s_at	SHBG	-0,384193355	-2,005029581	1,620836226
230369_at	GPR161	-0,730013898	-2,350444759	1,620430861

1570432_at	-	-0,730013898	-2,350444759	1,620430861
232328_at	ZNF552	-0,730013898	-2,350444759	1,620430861
209697_at	LOC100507088	-0,609040214	-2,229233437	1,620193224
236915_at	C4orf47	-0,609040214	-2,229233437	1,620193224
208545_x_at	MIR1257 /// TAF4	-0,609040214	-2,229233437	1,620193224
230265_at	SEL1L	3,370643409	1,750483134	1,620160276
232066_x_at	CTBP1-AS1	1,88491011	0,265475485	1,619434626
215505_s_at	STRN3	0,847529938	-0,771340337	1,618870275
37577_at	ARHGAP19	3,278284371	1,659453925	1,618830446
238353_at	RASL11A	0,001114523	-1,616924751	1,618039274
227557_at	SCARF2	0,001114523	-1,616924751	1,618039274
204532_x_at	UGT1A1 /// UGT1/	0,001114523	-1,616924751	1,618039274
237993_at	CHCHD5	0,001114523	-1,616924751	1,618039274
205708_s_at	TRPM2	0,001114523	-1,616924751	1,618039274
207384_at	PGLYRP1	0,001114523	-1,616924751	1,618039274
243837_x_at	-	3,517785666	1,899778623	1,618007043
225013_at	LZTS2	0,244681185	-1,372734086	1,617415271
216925_s_at	TAL1	0,244681185	-1,372734086	1,617415271
222960_at	CACNA1H	0,244681185	-1,372734086	1,617415271
232619_at	ACTL10	0,244681185	-1,372734086	1,617415271
228565_at	KIAA1804	2,932161212	1,315897482	1,61626373
241899_at	LOC553103	0,425036312	-1,19085694	1,615893252
1563404_at	-	0,425036312	-1,19085694	1,615893252
237744_at	-	0,425036312	-1,19085694	1,615893252
219979_s_at	C11orf73	4,372691748	2,757131856	1,615559892
222445_at	SLC39A9	4,308320282	2,693044596	1,615275686
231727_s_at	MIF4GD	2,739067176	1,123905343	1,615161833
238991_at	ASB1	-1,137208348	-2,752097204	1,614888855
227182_at	SUSD3	2,99543179	1,380552339	1,614879451
1559009_at	-	0,045517965	-1,568385657	1,613903621
205993_s_at	TBX2	0,045517965	-1,568385657	1,613903621
240806_at	RPL15	0,556428218	-1,057301851	1,613730069
236560_at	LOC100129884	0,556428218	-1,057301851	1,613730069
219518_s_at	ELL3	0,556428218	-1,057301851	1,613730069
229815_at	TMEM161B-AS1	0,95222556	-0,661178575	1,613404135
228845_at	PLBD2	1,024284941	-0,589001171	1,613286112
208104_s_at	TSC22D4	1,094118704	-0,518232988	1,612351692
1561420_a_at	-	-0,924000698	-2,536339282	1,612338584
236183_at	DYNC1H1	-0,924000698	-2,536339282	1,612338584
1558501_at	DNM3	-0,437449947	-2,049747196	1,612297249
234460_at	Ndufaf4	-0,437449947	-2,049747196	1,612297249
217664_at	-	-0,437449947	-2,049747196	1,612297249
1554382_at	LOC200261	-0,437449947	-2,049747196	1,612297249
241894_at	VMO1	-0,437449947	-2,049747196	1,612297249
231126_at	C2orf70	-0,437449947	-2,049747196	1,612297249
238456_at	LOC100289230	-0,437449947	-2,049747196	1,612297249
243029_at	KREMEN1	-0,437449947	-2,049747196	1,612297249
223839_s_at	SCD	2,983826368	1,371634653	1,612191715
222631_at	LOC285540 /// PI4	4,918218598	3,306167675	1,612050923
1557293_at	LOC440993	1,225123479	-0,386896102	1,612019581

227494_at	LOC253842 /// NR	1,225123479	-0,386896102	1,612019581
202638_s_at	ICAM1	2,811067138	1,199296812	1,611770325
217640_x_at	SKA1	1,898032047	0,286279868	1,61175218
213786_at	TAX1BP1	3,291641956	1,680963921	1,610678035
229944_at	OPRK1	-0,856259172	-2,466349414	1,610090242
244144_at	SYNE1	-0,856259172	-2,466349414	1,610090242
223045_at	EGLN1	-0,856259172	-2,466349414	1,610090242
216454_at	TRMT1	-0,856259172	-2,466349414	1,610090242
1555967_at	-	0,282358733	-1,32740143	1,609760163
227298_at	FLJ37798	2,107136847	0,497424714	1,609712132
207686_s_at	CASP8	2,410823487	0,80114751	1,609675977
205479_s_at	PLAU	1,671397663	0,061773582	1,609624081
217964_at	TTC19	5,383324257	3,774776136	1,608548121
241427_x_at	-	0,873789652	-0,734355396	1,608145048
214226_at	PRSS53	0,873789652	-0,734355396	1,608145048
200639_s_at	YWHAZ	5,923704447	4,31603046	1,607673987
201312_s_at	SH3BGRL	6,262526809	4,655367664	1,607159145
205038_at	IKZF1	3,628556015	2,021531611	1,607024404
225672_at	GOLGA2	2,719381633	1,112548615	1,606833018
238601_at	PHKB	1,97249974	0,366031214	1,606468526
223879_s_at	OXR1	2,590269697	0,98381571	1,606453987
203429_s_at	C1orf9	3,200057698	1,593605508	1,606452189
240918_at	-	0,08619576	-1,520022123	1,606217883
238888_at	-	0,08619576	-1,520022123	1,606217883
227888_at	RRBP1	0,08619576	-1,520022123	1,606217883
219268_at	ETNK2	0,08619576	-1,520022123	1,606217883
208262_x_at	MEFV	0,08619576	-1,520022123	1,606217883
213345_at	NFATC4	0,08619576	-1,520022123	1,606217883
210726_at	CYP3A4	0,08619576	-1,520022123	1,606217883
220741_s_at	PPA2	6,499221118	4,89320292	1,606018198
37953_s_at	ASIC1	2,213956148	0,608026145	1,605930003
221877_at	IRGQ	2,41945542	0,815200271	1,604255149
200053_at	SPAG7	4,936312488	3,332103994	1,604208494
1564757_a_at	CCDC148	-0,49284695	-2,09678811	1,60394116
227717_at	ARHGEF37	-0,49284695	-2,09678811	1,60394116
217369_at	IGHG1 /// LOC100!	-0,49284695	-2,09678811	1,60394116
232802_at	SYT8	-0,49284695	-2,09678811	1,60394116
219343_at	CDC37L1	2,824160383	1,220470658	1,603689725
207490_at	TUBA4B	0,791908897	-0,811769547	1,603678444
237319_at	C2orf53	0,58788177	-1,015398016	1,603279786
205012_s_at	HAGH	3,931608669	2,328492509	1,60311616
208239_at	FOXE1	-1,06282519	-2,665156778	1,602331588
237212_at	-	-1,06282519	-2,665156778	1,602331588
203282_at	GBE1	4,267962641	2,665727652	1,602234989
220199_s_at	AIDA	4,65719538	3,05501254	1,60218284
218940_at	METTL21D	3,261582786	1,659532492	1,602050295
1553840_a_at	CCDC149	0,319287178	-1,282520722	1,6018079
213439_x_at	RUNDC3A	0,319287178	-1,282520722	1,6018079
1568377_x_at	DEFB124	1,116462765	-0,484558493	1,601021258
215812_s_at	LOC653562 /// SLC	1,116462765	-0,484558493	1,601021258

1552885_a_at	NKX6-3	1,116462765	-0,484558493	1,601021258
233011_at	ANXA1	-0,66934516	-2,27001641	1,600671251
213495_s_at	-	-0,66934516	-2,27001641	1,600671251
217210_at	-	-0,66934516	-2,27001641	1,600671251
1552493_s_at	CYP11B1	-0,66934516	-2,27001641	1,600671251
1567027_at	SH3GL1P2	-0,66934516	-2,27001641	1,600671251
218004_at	BSDC1	2,621163856	1,020514937	1,600648919
225905_s_at	ST3GAL3	1,510970097	-0,089635713	1,60060581
205999_x_at	CYP3A4	-0,791294935	-2,391863529	1,600568594
208300_at	PTPRH	-0,791294935	-2,391863529	1,600568594
1556643_at	LOC100507535	2,829929672	1,2298509	1,600078772
229484_at	PPM1J	0,127732831	-1,471833228	1,599566058
238225_at	MIR146A	0,127732831	-1,471833228	1,599566058
220238_s_at	KLHL7	2,199941239	0,600385088	1,599556151
238523_at	KLHL36	2,149438457	0,550017041	1,599421417
213616_at	TPGS2	4,216509688	2,617393502	1,599116187
1556121_at	NAP1L1	2,798156197	1,199296812	1,598859385
204257_at	FADS3	1,88491011	0,286279868	1,598630243
218869_at	MLYCD	1,983901861	0,385582632	1,598319229
237977_at	-	0,900065578	-0,697182596	1,597248174
205007_s_at	CIB2	0,707657549	-0,889222211	1,596879759
231792_at	MYLK2	0,707657549	-0,889222211	1,596879759
217503_at	STK17B	2,179708195	0,583607503	1,596100692
228801_at	ORMDL1	2,492739983	0,896834102	1,595905881
209531_at	GSTZ1	3,6787904	2,082919929	1,595870472
227180_at	ELOVL7	1,364555912	-0,231045907	1,595601819
207232_s_at	DZIP3	1,816938997	0,222432814	1,594506183
224968_at	CCDC104	4,744801376	3,150841611	1,593959765
224345_x_at	FAM162A	7,088622845	5,494711522	1,593911323
226596_x_at	LOC729852	1,421391637	-0,172194225	1,593585862
221857_s_at	TJAP1	1,421391637	-0,172194225	1,593585862
212845_at	SAMD4A	1,421391637	-0,172194225	1,593585862
201366_at	ANXA7	5,70657598	4,113313626	1,593262354
240398_at	-	0,491906512	-1,100943374	1,592849886
226696_at	RBBP9	3,521615383	1,929092307	1,592523076
229238_at	C17orf97	0,820160788	-0,771340337	1,591501125
238668_at	-	3,942941653	2,351470274	1,591471379
214658_at	TMED7 /// TMED7	3,618399139	2,026957145	1,591441994
210954_s_at	TSC22D2	-0,551284523	-2,142286822	1,591002299
229907_at	-	-0,551284523	-2,142286822	1,591002299
229788_s_at	-	-0,551284523	-2,142286822	1,591002299
208191_x_at	PSG4	-0,551284523	-2,142286822	1,591002299
215515_at	-	-0,551284523	-2,142286822	1,591002299
211301_at	KCND3	-0,551284523	-2,142286822	1,591002299
225488_at	DERL1	-0,551284523	-2,142286822	1,591002299
1556397_at	-	-0,551284523	-2,142286822	1,591002299
222197_s_at	-	-0,551284523	-2,142286822	1,591002299
213249_at	FBXL7	-0,551284523	-2,142286822	1,591002299
203507_at	CD68	0,355751005	-1,234835326	1,590586331
206905_s_at	MATN1	0,355751005	-1,234835326	1,590586331

235663_at	-	0,355751005	-1,234835326	1,590586331
240587_x_at	LOC100506219	0,355751005	-1,234835326	1,590586331
229561_at	LRRC16B	0,355751005	-1,234835326	1,590586331
221306_at	GPR27	0,355751005	-1,234835326	1,590586331
202245_at	LSS	4,45671768	2,86626857	1,59044911
203987_at	FZD6	3,701002444	2,110584371	1,590418074
209144_s_at	CBFA2T2	0,167727503	-1,422257026	1,58998453
242891_at	ADCY5	0,167727503	-1,422257026	1,58998453
237678_at	-	0,167727503	-1,422257026	1,58998453
207692_s_at	ACAN	0,167727503	-1,422257026	1,58998453
205693_at	TNNT3	0,167727503	-1,422257026	1,58998453
233679_at	LOC100506472	0,167727503	-1,422257026	1,58998453
243475_at	CBL	0,167727503	-1,422257026	1,58998453
211180_x_at	LOC100506403 ///	0,167727503	-1,422257026	1,58998453
238388_x_at	-	0,167727503	-1,422257026	1,58998453
1560739_a_at	-	1,578719607	-0,011001236	1,589720842
231000_at	ROR2	1,000459215	-0,589001171	1,589460386
203280_at	SAFB2	1,000459215	-0,589001171	1,589460386
221490_at	UBAP1	2,597647262	1,008210238	1,589437024
201055_s_at	HNRNPA0	4,274951805	2,686001625	1,58895018
222731_at	ZDHHC2	4,049824219	2,460974054	1,588850165
234994_at	TMEM200A	4,793738098	3,205255599	1,588482499
225047_at	NUPL1	2,085817135	0,497424714	1,58839242
214500_at	H2AFY	2,866549889	1,278654284	1,587895605
220610_s_at	LRRFIP2	3,562907192	1,975797592	1,5871096
221744_at	DCAF7	3,551632175	1,964670566	1,586961609
213461_at	NUDT21	3,642593015	2,055739171	1,586853844
218041_x_at	SLC38A2	5,581288285	3,995185753	1,586102532
205889_s_at	JAKMIP2	-1,137208348	-2,723114724	1,585906376
233900_at	-	-1,137208348	-2,723114724	1,585906376
234296_s_at	TEX11	-1,137208348	-2,723114724	1,585906376
215997_s_at	CUL4B	1,830468611	0,244646697	1,585821914
238622_at	RAP2B	0,735999505	-0,84928999	1,585289495
220339_s_at	TPSG1	0,735999505	-0,84928999	1,585289495
222558_at	RPRD1A	1,383446753	-0,201789721	1,585236474
230239_at	ROCK1	1,383446753	-0,201789721	1,585236474
212625_at	STX10	3,171328694	1,586121802	1,585206893
1553755_at	NXNL1	1,440265662	-0,144765583	1,585031245
221864_at	ORAI3	1,440265662	-0,144765583	1,585031245
200867_at	RNF114	4,330688003	2,746644067	1,584043936
212899_at	CDK19	3,843767728	2,260052727	1,583715001
228026_at	SIKE1	3,889146356	2,305621062	1,583525294
238920_at	-	1,493647653	-0,089635713	1,583283366
234414_at	FAM22F	1,493647653	-0,089635713	1,583283366
222869_s_at	ELAC1	1,716003175	0,133104519	1,582898655
1555737_a_at	KLK4	-0,180990326	-1,763664074	1,582673748
239021_at	TLR6	-0,180990326	-1,763664074	1,582673748
214543_x_at	QKI	2,649978302	1,067365565	1,582612737
215287_at	STRN	1,760255626	0,178191865	1,58206376
205054_at	NEB	-0,730013898	-2,312054429	1,582040531

230635_at	-	-0,730013898	-2,312054429	1,582040531
228025_s_at	PPP2R2C	-0,730013898	-2,312054429	1,582040531
240437_at	CASP9	-0,730013898	-2,312054429	1,582040531
215538_at	LARGE	-0,730013898	-2,312054429	1,582040531
221259_s_at	TEX11	-0,730013898	-2,312054429	1,582040531
237731_at	LOC154092	-0,730013898	-2,312054429	1,582040531
231790_at	DMGDH	-0,280273599	-1,862269442	1,581995843
206331_at	CALCRL	-0,280273599	-1,862269442	1,581995843
215327_at	-	-0,280273599	-1,862269442	1,581995843
229177_at	C16orf89	-0,280273599	-1,862269442	1,581995843
228409_at	PLIN4	-0,280273599	-1,862269442	1,581995843
230929_s_at	-	-0,280273599	-1,862269442	1,581995843
234555_at	EPPK1	-0,280273599	-1,862269442	1,581995843
208129_x_at	LOC100506403 ///	-0,280273599	-1,862269442	1,581995843
213769_at	KSR1	-0,280273599	-1,862269442	1,581995843
225981_at	C17orf28	0,39105295	-1,19085694	1,58190989
235525_at	-	0,39105295	-1,19085694	1,58190989
230615_at	DUOXA2	0,847529938	-0,734355396	1,581885334
237081_at	-	0,524545436	-1,057301851	1,581847287
1566830_at	LOC440028	0,524545436	-1,057301851	1,581847287
1553222_at	OXER1	2,248155141	0,666486137	1,581669004
225024_at	RPRD1B	3,954683435	2,373249966	1,581433469
227247_at	PLEKHA8	3,204847647	1,623485524	1,581362123
214502_at	HIST1H2BJ	-0,230432956	-1,811780055	1,581347099
1563008_at	LOC100287877	-0,230432956	-1,811780055	1,581347099
240056_at	NSMAF	-0,230432956	-1,811780055	1,581347099
224305_s_at	NPC1L1	-0,230432956	-1,811780055	1,581347099
212012_at	PXDN	-0,230432956	-1,811780055	1,581347099
224540_at	LACRT	-0,230432956	-1,811780055	1,581347099
236576_at	-	-0,135065865	-1,715576125	1,58051026
235624_at	HDLBP	-0,135065865	-1,715576125	1,58051026
239643_at	LOC100129516	-0,135065865	-1,715576125	1,58051026
233767_at	HHLA1	-0,135065865	-1,715576125	1,58051026
236213_at	-	0,20740896	-1,372734086	1,580143046
237344_at	LOC100505474	0,20740896	-1,372734086	1,580143046
208505_s_at	FUT2	0,20740896	-1,372734086	1,580143046
215214_at	IGLC1	0,20740896	-1,372734086	1,580143046
1554937_x_at	EXOC3L1	0,20740896	-1,372734086	1,580143046
216332_at	POU6F1	0,20740896	-1,372734086	1,580143046
1564713_a_at	FOXN4	0,20740896	-1,372734086	1,580143046
1556737_at	LOC388387	0,648195588	-0,931539147	1,579734735
1570523_s_at	ATG10	2,321828846	0,742671819	1,579157027
225263_at	HS6ST1	2,229377375	0,65030602	1,579071355
204746_s_at	PICK1	1,024284941	-0,554696666	1,578981607
236186_x_at	IL17RE	-0,332405896	-1,911206569	1,578800674
217634_at	SVIL	-0,332405896	-1,911206569	1,578800674
236821_at	-	-0,332405896	-1,911206569	1,578800674
229448_at	CERS1 /// GDF1	-0,332405896	-1,911206569	1,578800674
227337_at	ANKRD37	3,209439373	1,630734622	1,578704751
223923_at	C21orf62	1,094118704	-0,484558493	1,578677197

222021_x_at	SDHAP1 /// SDHAF	5,342396052	3,763882826	1,578513226
203493_s_at	CEP57	4,183891989	2,605630202	1,578261787
231219_at	CMTM1	1,983901861	0,405752839	1,578149022
234659_at	-	-0,924000698	-2,501359113	1,577358416
244338_at	LDLRAD3	-0,924000698	-2,501359113	1,577358416
232653_at	-	-0,924000698	-2,501359113	1,577358416
214361_s_at	RGS12	-0,609040214	-2,186086603	1,577046389
210363_s_at	SCN2B	-0,609040214	-2,186086603	1,577046389
224551_s_at	SPTBN4	-0,609040214	-2,186086603	1,577046389
237990_x_at	C19orf29	-0,609040214	-2,186086603	1,577046389
209999_x_at	SOCS1	-0,609040214	-2,186086603	1,577046389
215180_at	-	-0,088974936	-1,665791776	1,57681684
241496_at	BTNL9	-0,088974936	-1,665791776	1,57681684
221292_at	PTCH2	-0,088974936	-1,665791776	1,57681684
240752_at	LOC100287808	-0,088974936	-1,665791776	1,57681684
236136_at	CYTH3	-0,088974936	-1,665791776	1,57681684
242763_at	-	-0,994186487	-2,570522741	1,576336254
226564_at	ZFAT	1,92317941	0,346964736	1,576214674
209431_s_at	PATZ1	2,019346222	0,443143223	1,576202999
205081_at	CRIP1	6,372447182	4,796421511	1,576025672
222061_at	CD58	0,764143511	-0,811769547	1,575913058
211634_x_at	IGHM	0,764143511	-0,811769547	1,575913058
204811_s_at	CACNA2D2	0,764143511	-0,811769547	1,575913058
230999_at	-	0,764143511	-0,811769547	1,575913058
214116_at	BTD	0,764143511	-0,811769547	1,575913058
223492_s_at	LRRFIP1	3,291641956	1,715863686	1,57577827
228738_at	D2HGDH	2,348178434	0,772793615	1,57538482
221749_at	YTHDF3	4,674825018	3,099629381	1,575195637
1554444_s_at	C2orf18	1,730929079	0,155747281	1,575181798
1553974_at	C22orf39	3,6071174	2,03313004	1,57398736
237393_at	TPBGL	-0,043925712	-1,616924751	1,572999039
240517_at	CBS	-0,043925712	-1,616924751	1,572999039
205827_at	CCK	-0,043925712	-1,616924751	1,572999039
229727_x_at	C2CD4B	-0,043925712	-1,616924751	1,572999039
241767_at	-	-0,384193355	-1,956681069	1,572487715
236865_at	-	-0,384193355	-1,956681069	1,572487715
221031_s_at	APOLD1	3,127551022	1,555165465	1,572385557
232131_at	LOC100506874	-0,856259172	-2,4284334	1,572174228
1553874_a_at	ZSCAN10	-0,856259172	-2,4284334	1,572174228
232531_at	EMX2OS	-0,856259172	-2,4284334	1,572174228
206798_x_at	DLEC1	0,244681185	-1,32740143	1,572082615
230813_at	LEPREL1	0,556428218	-1,015398016	1,571826234
212978_at	LRRC8B	2,649978302	1,078619386	1,571358916
234339_s_at	GLTSCR2	4,769413297	3,198229785	1,571183511
211976_at	-	3,071179046	1,500059916	1,57111913
203842_s_at	MAPRE3	0,873789652	-0,697182596	1,570972248
214709_s_at	KTN1	6,021252979	4,45105157	1,570201409
1556937_at	-	0,425036312	-1,145057014	1,570093327
215138_s_at	KAZN	0,425036312	-1,145057014	1,570093327
216061_x_at	PDGFB	0,425036312	-1,145057014	1,570093327

223001_at	OSTC	6,668083557	5,098112469	1,569971088
236266_at	RORA	1,182925501	-0,386896102	1,569821603
235008_at	-	0,001114523	-1,568385657	1,569500179
215173_at	DNAAF1	0,001114523	-1,568385657	1,569500179
220080_at	FBXL8	0,001114523	-1,568385657	1,569500179
236069_at	-	0,001114523	-1,568385657	1,569500179
1563225_a_at	-	0,001114523	-1,568385657	1,569500179
220629_at	KCNQ1DN	0,001114523	-1,568385657	1,569500179
223259_at	ORMDL3	4,306090192	2,736663738	1,569426454
229908_s_at	UNKL	2,452674045	0,883374248	1,569299797
208810_at	DNAJB6 /// TMEM	4,355700522	2,786807892	1,56889263
213562_s_at	SQLE	3,430413158	1,861868165	1,568544994
238477_at	KIF1C	2,38349585	0,815200271	1,56829558
230893_at	DNAJC21	2,51558601	0,947673886	1,567912124
236664_at	AKT2	1,307201325	-0,260526297	1,567727622
220753_s_at	CRYL1	1,307201325	-0,260526297	1,567727622
228405_at	RHPN1	1,307201325	-0,260526297	1,567727622
209477_at	EMD	2,836219929	1,268569037	1,567650891
217181_at	-	-0,437449947	-2,005029581	1,567579634
206628_at	SLC5A1	-0,437449947	-2,005029581	1,567579634
232245_at	SLC25A34	-0,437449947	-2,005029581	1,567579634
214348_at	TACR2	-0,437449947	-2,005029581	1,567579634
1570375_at	LOC100506585	-0,437449947	-2,005029581	1,567579634
210728_s_at	CALCA	-0,437449947	-2,005029581	1,567579634
204694_at	AFP	-0,437449947	-2,005029581	1,567579634
1552649_a_at	RAD51L3-RFFL ///	0,677745787	-0,889222211	1,566967998
238230_x_at	MAN2C1	0,677745787	-0,889222211	1,566967998
225101_s_at	SNX14	4,36640141	2,799505781	1,566895629
1561112_at	-	-1,289805289	-2,856570137	1,566764848
223566_s_at	BCOR	3,501655014	1,935144844	1,56651017
210132_at	EFNA3	1,364555912	-0,201789721	1,566345633
211198_s_at	ICOSLG	0,97663406	-0,589001171	1,565635231
231379_at	LOC100507560	0,045517965	-1,520022123	1,565540088
233165_at	NCKIPSD	0,045517965	-1,520022123	1,565540088
244615_x_at	TARSL2	0,045517965	-1,520022123	1,565540088
206110_at	HIST1H3H	1,871521385	0,306454867	1,565066518
212757_s_at	CAMK2G	3,209439373	1,644429915	1,565009458
1568248_x_at	SNORA71B	0,282358733	-1,282520722	1,564879455
1559766_at	ALDH1L1-AS2	0,282358733	-1,282520722	1,564879455
214504_at	ABO	0,282358733	-1,282520722	1,564879455
1553727_at	B4GALNT3	0,282358733	-1,282520722	1,564879455
201360_at	CST3	0,282358733	-1,282520722	1,564879455
212164_at	TMEM183A /// TM	2,276736537	0,711934445	1,564802092
202837_at	TRAFD1	2,276736537	0,711934445	1,564802092
210685_s_at	UBE4B	2,276736537	0,711934445	1,564802092
37201_at	ITIH4	0,529059535	-1,035498147	1,564557682
202557_at	HSPA13	4,648252012	3,084123325	1,564128686
227261_at	KLF12	2,365121417	0,80114751	1,563973907
220989_s_at	AMN	0,791908897	-0,771340337	1,563249234
206752_s_at	DFFB	1,948628481	0,385582632	1,563045849

223437_at	PPARA	2,096248267	0,533258442	1,562989826
201105_at	LGALS1	8,107885665	6,544920261	1,562965404
219282_s_at	TRPV2	3,442765386	1,881145546	1,561619839
200947_s_at	GLUD1	5,265546262	3,704101387	1,561444874
221206_at	PMS2 /// PMS2CL	2,685337919	1,123905343	1,561432576
200715_x_at	RPL13A /// SNORD	7,892831324	6,331586277	1,561245047
240269_at	-	0,900065578	-0,661178575	1,561244153
228106_at	DCAF16	3,012642182	1,451507543	1,561134639
203635_at	DSCR3	4,402175336	2,841176366	1,56099897
226583_at	C12orf76	3,161901883	1,601076277	1,560825607
200940_s_at	RERE	3,269673646	1,709047448	1,560626198
203173_s_at	C16orf62	3,843767728	2,283169614	1,560598114
222421_at	UBE2H	3,296548503	1,736450458	1,560098045
208555_x_at	CST2	-0,66934516	-2,229233437	1,559888278
237113_at	-	-0,66934516	-2,229233437	1,559888278
213565_s_at	SMAD6	-0,66934516	-2,229233437	1,559888278
233149_at	-	0,458905032	-1,100943374	1,559848406
217896_s_at	FAM192A	2,209870035	0,65030602	1,559564015
235333_at	B4GALT6	3,581038515	2,021531611	1,559506904
237610_at	-	-0,791294935	-2,350444759	1,559149824
207823_s_at	AIF1	-0,791294935	-2,350444759	1,559149824
216327_s_at	SIGLEC8	-0,791294935	-2,350444759	1,559149824
211203_s_at	CNTN1	-0,791294935	-2,350444759	1,559149824
215871_at	PLA2G5	-0,791294935	-2,350444759	1,559149824
213032_at	NFIB	-0,791294935	-2,350444759	1,559149824
200905_x_at	HLA-E	6,900833989	5,341743685	1,559090305
201691_s_at	TPD52	1,20396005	-0,354781582	1,558741631
225371_at	GLE1	4,813757418	3,255452586	1,558304832
235515_at	SYNE4	0,08619576	-1,471833228	1,558028987
237765_at	SLC25A47	0,08619576	-1,471833228	1,558028987
219422_at	ESPN	0,08619576	-1,471833228	1,558028987
243950_at	-	0,08619576	-1,471833228	1,558028987
214637_at	OSM	0,08619576	-1,471833228	1,558028987
202187_s_at	PPP2R5A	4,925763575	3,367769667	1,557993908
206438_x_at	TCTN2	2,019346222	0,461385738	1,557960484
230141_at	ARID4A	1,326463531	-0,231045907	1,557509438
213693_s_at	MUC1	1,326463531	-0,231045907	1,557509438
224059_s_at	NUMBL	0,707657549	-0,84928999	1,556947539
228921_at	SLC25A42	-0,49284695	-2,049747196	1,556900246
230693_at	ATP2A1	-0,49284695	-2,049747196	1,556900246
209639_s_at	RGS12	-0,49284695	-2,049747196	1,556900246
206682_at	CLEC10A	-0,49284695	-2,049747196	1,556900246
229597_s_at	WDFY4	-0,49284695	-2,049747196	1,556900246
216055_at	PDGFB	-0,49284695	-2,049747196	1,556900246
209293_x_at	ID4	-0,49284695	-2,049747196	1,556900246
239358_at	-	1,5944803	0,03784737	1,55663293
202164_s_at	CNOT8	4,546768622	2,990421269	1,556347353
218760_at	COQ6	2,657308324	1,101780212	1,555528112
243004_at	-	1,000459215	-0,554696666	1,555155881
229090_at	ZEB1-AS1	1,960819106	0,405752839	1,555066268

243069_at	-	0,820160788	-0,734355396	1,554516184
202752_x_at	SLC7A8	0,820160788	-0,734355396	1,554516184
221095_s_at	KCNE2	0,820160788	-0,734355396	1,554516184
218744_s_at	PACSIN3	0,319287178	-1,234835326	1,554122504
221163_s_at	MLXIPL	0,319287178	-1,234835326	1,554122504
1563946_at	-	0,319287178	-1,234835326	1,554122504
222942_s_at	LOC100505519 ///	2,878891456	1,324949741	1,553941715
222811_at	FTSJD1	3,481948861	1,929092307	1,552856554
222830_at	GRHL1	1,995527416	0,443143223	1,552384193
226104_at	RNF170	3,233229368	1,680963921	1,552265447
203560_at	GGH	5,304531188	3,7524971	1,552034088
224767_at	LOC100506548 ///	4,118748355	2,56688207	1,551866284
225698_at	EPB41L4A-AS1	5,116796324	3,564966474	1,551829851
226483_at	TMEM68	2,771643417	1,220470658	1,551172759
209939_x_at	CFLAR	5,708143186	4,157029702	1,551113484
218771_at	PANK4	3,252865595	1,701800759	1,551064836
234954_at	-	3,883929118	2,332940758	1,55098836
223193_x_at	FAM162A	7,056734911	5,50581282	1,550922092
243483_at	TRPM8	0,127732831	-1,422257026	1,549989857
239637_at	RAB18	0,127732831	-1,422257026	1,549989857
1556058_s_at	SPEN	0,127732831	-1,422257026	1,549989857
214094_at	FUBP1	0,127732831	-1,422257026	1,549989857
1563048_at	-	0,127732831	-1,422257026	1,549989857
224133_at	-	0,127732831	-1,422257026	1,549989857
1555543_a_at	CLCC1	2,365121417	0,815200271	1,549921146
233814_at	EFNA5	0,618277321	-0,931539147	1,549816468
221672_s_at	TRAPPC9	0,618277321	-0,931539147	1,549816468
235264_at	HCFC2	0,926325262	-0,623254098	1,549579361
1562836_at	DDX6	0,926325262	-0,623254098	1,549579361
1569949_at	GRK5	0,926325262	-0,623254098	1,549579361
205936_s_at	HK3	0,491906512	-1,057301851	1,549208363
203021_at	SLPI	0,491906512	-1,057301851	1,549208363
236701_at	GSG1L	0,491906512	-1,057301851	1,549208363
227429_at	EFCAB4A	0,491906512	-1,057301851	1,549208363
224807_at	GRAMD1A	2,855122714	1,306903969	1,548218744
202629_at	APPBP2	3,039689025	1,491644074	1,548044951
221507_at	TNPO2	2,507817181	0,960042218	1,547774963
241357_at	MAPK15	0,735999505	-0,811769547	1,547769052
214481_at	HIST1H2AM	1,510970097	-0,036543687	1,547513784
217205_at	-	1,510970097	-0,036543687	1,547513784
204701_s_at	STOML1	1,510970097	-0,036543687	1,547513784
241396_at	NEDD4L	0,355751005	-1,19085694	1,546607945
208277_at	PITX3	0,355751005	-1,19085694	1,546607945
217658_at	THAP3	0,355751005	-1,19085694	1,546607945
243710_at	LOC100506175	0,355751005	-1,19085694	1,546607945
203186_s_at	S100A4	7,44853233	5,902287619	1,546244711
1552417_a_at	NEDD1	2,972517974	1,426280426	1,546237548
211059_s_at	GOLGA2	2,096248267	0,550017041	1,546231227
242617_at	TMED8	3,748268542	2,20251271	1,545755832
231872_at	LRRCC1	2,25755351	0,711934445	1,545619065

234539_at	ADARB1	-0,551284523	-2,09678811	1,545503587
237447_at	C22orf45	-0,551284523	-2,09678811	1,545503587
1570013_at	-	-0,551284523	-2,09678811	1,545503587
206222_at	TNFRSF10C	-0,551284523	-2,09678811	1,545503587
216937_s_at	RS1	-0,551284523	-2,09678811	1,545503587
227272_at	C15orf52	-0,551284523	-2,09678811	1,545503587
201654_s_at	HSPG2	-0,551284523	-2,09678811	1,545503587
230686_s_at	SLC13A3	-0,551284523	-2,09678811	1,545503587
241378_at	MIB2	-0,551284523	-2,09678811	1,545503587
203575_at	CSNK2A2	3,707586205	2,162541631	1,545044574
239161_at	FDX1	1,745502609	0,20062106	1,544881549
205961_s_at	PSIP1	5,533034735	3,988637612	1,544397122
238974_at	C2orf69	4,907682208	3,363771078	1,54391113
213958_at	CD6	4,79864476	3,255452586	1,543192174
205039_s_at	IKZF1	4,550683059	3,007560601	1,543122457
202339_at	SYMPK	1,024284941	-0,518232988	1,542517929
208260_at	AVPR1B	1,024284941	-0,518232988	1,542517929
217767_at	C3	1,024284941	-0,518232988	1,542517929
235187_s_at	-	-0,924000698	-2,466349414	1,542348716
1565856_at	-	-0,924000698	-2,466349414	1,542348716
214883_at	THRA	-0,924000698	-2,466349414	1,542348716
227692_at	GNAI1	-0,924000698	-2,466349414	1,542348716
1562029_at	-	-1,289805289	-2,832036647	1,542231358
1569853_at	-	-0,994186487	-2,536339282	1,542152795
1562240_at	PLXNA4	-0,994186487	-2,536339282	1,542152795
216701_at	C1orf68	-0,994186487	-2,536339282	1,542152795
224457_at	MGC12982	-0,994186487	-2,536339282	1,542152795
211986_at	AHNAK	5,213520537	3,671698545	1,541821992
1553730_x_at	LRRC43	0,95222556	-0,589001171	1,541226731
203130_s_at	KIF5C	2,410823487	0,869824768	1,540998718
218520_at	TBK1	4,20258728	2,662083808	1,540503472
211485_s_at	FGF18	0,167727503	-1,372734086	1,540461589
229102_at	HTATIP2	0,167727503	-1,372734086	1,540461589
200852_x_at	GNB2	4,381466431	2,841176366	1,540290065
1565554_at	LOC127841	-0,730013898	-2,27001641	1,540002512
209529_at	PPAP2C	-0,730013898	-2,27001641	1,540002512
1553600_at	TMIE	-0,730013898	-2,27001641	1,540002512
206315_at	CRLF1	-0,730013898	-2,27001641	1,540002512
1554842_at	SLC12A1	-0,730013898	-2,27001641	1,540002512
1563407_x_at	ATP4B	-0,730013898	-2,27001641	1,540002512
225350_s_at	ZYG11B	3,771154436	2,23115548	1,539998956
226699_at	FCHSD1	0,524545436	-1,015398016	1,539943453
230017_at	-	0,524545436	-1,015398016	1,539943453
216138_at	-	0,524545436	-1,015398016	1,539943453
243636_s_at	-	-1,212652659	-2,752097204	1,539444544
201006_at	PRDX2	1,307201325	-0,231045907	1,538247232
228035_at	STK33	1,88491011	0,346964736	1,537945375
206823_at	L3MBTL1	0,648195588	-0,889222211	1,537417799
1568667_s_at	PLIN5	0,648195588	-0,889222211	1,537417799
222721_at	CNIH4	0,39105295	-1,145057014	1,536109965

241443_at	-	0,39105295	-1,145057014	1,536109965
241138_at	-	0,39105295	-1,145057014	1,536109965
213114_at	-	0,39105295	-1,145057014	1,536109965
224652_at	CCNY	2,692288672	1,156599976	1,535688695
1569133_x_at	ARSK	-0,856259172	-2,391863529	1,535604357
217154_s_at	EDN3	-0,856259172	-2,391863529	1,535604357
238019_at	PLIN5	-0,856259172	-2,391863529	1,535604357
224836_at	TP53INP2	2,16969527	0,634301128	1,535394142
224924_at	TTC7A	0,873789652	-0,661178575	1,534968226
227151_at	SNX33	0,873789652	-0,661178575	1,534968226
222934_s_at	CLEC4E	0,20740896	-1,32740143	1,53481039
204442_x_at	LTBP4	1,116462765	-0,418248858	1,534711623
234376_at	MYCN	-0,180990326	-1,715576125	1,534585799
222802_at	EDN1	-0,180990326	-1,715576125	1,534585799
228896_at	-	-0,180990326	-1,715576125	1,534585799
207670_at	KRT85	-0,180990326	-1,715576125	1,534585799
203070_at	SEMA3B	-0,180990326	-1,715576125	1,534585799
216137_s_at	MAPK8IP3	-0,180990326	-1,715576125	1,534585799
213972_at	-	-0,180990326	-1,715576125	1,534585799
211448_s_at	RGS6	-0,180990326	-1,715576125	1,534585799
1567068_at	OR4D1	-0,180990326	-1,715576125	1,534585799
209831_x_at	DNASE2	3,858118781	2,323616375	1,534502406
225793_at	LIX1L	4,618041557	3,084123325	1,533918231
201619_at	PRDX3	6,428462634	4,894556606	1,533906028
239339_at	-	-0,609040214	-2,142286822	1,533246608
224385_s_at	MOV10L1	-0,609040214	-2,142286822	1,533246608
223942_x_at	CHST5	-0,609040214	-2,142286822	1,533246608
208105_at	GIPR	-0,609040214	-2,142286822	1,533246608
241783_at	-	-0,609040214	-2,142286822	1,533246608
229932_at	-	-0,609040214	-2,142286822	1,533246608
237430_at	-	-0,609040214	-2,142286822	1,533246608
221674_s_at	CHRD	-0,609040214	-2,142286822	1,533246608
233687_s_at	KLK9	-0,609040214	-2,142286822	1,533246608
231479_at	TTC33	-0,609040214	-2,142286822	1,533246608
229046_s_at	PLEKHB1	-0,230432956	-1,763664074	1,533231118
243076_x_at	GLI4	-0,230432956	-1,763664074	1,533231118
221449_s_at	ITFG1	3,852052012	2,31904917	1,533002843
218197_s_at	OXR1	3,783108337	2,250228409	1,532879928
207568_at	CHRNA6	4,335356502	2,802594885	1,532761617
221498_at	SNX27	2,884644843	1,352622437	1,532022406
207578_s_at	HTR4	1,544940605	0,013190398	1,531750207
240933_at	-	-0,280273599	-1,811780055	1,531506456
1570414_x_at	FLJ13197	-0,280273599	-1,811780055	1,531506456
202327_s_at	PKD1	-0,280273599	-1,811780055	1,531506456
236661_at	IQCF6	-0,280273599	-1,811780055	1,531506456
1553190_s_at	PARD3B	-0,280273599	-1,811780055	1,531506456
1554376_s_at	PTPLA	-0,280273599	-1,811780055	1,531506456
205372_at	PLAG1	1,686984721	0,155747281	1,53123744
201263_at	TARS	5,759034395	4,227865102	1,531169294
223954_x_at	NECAB3	1,640193838	0,109458907	1,530734932

206179_s_at	TPPP	-0,135065865	-1,665791776	1,530725911
244379_at	-	-0,135065865	-1,665791776	1,530725911
223464_at	OSBPL5	-0,135065865	-1,665791776	1,530725911
215621_s_at	IGHD	-0,135065865	-1,665791776	1,530725911
209726_at	CA11	-0,135065865	-1,665791776	1,530725911
242124_at	CHST1	-0,135065865	-1,665791776	1,530725911
208352_x_at	ANK1	-0,135065865	-1,665791776	1,530725911
208319_s_at	RBM3	6,346028824	4,815598205	1,530430619
211425_x_at	SSX4 /// SSX4B	-0,332405896	-1,862269442	1,529863546
237695_at	-	-0,332405896	-1,862269442	1,529863546
234350_at	IGLC1	-0,332405896	-1,862269442	1,529863546
235279_at	PARVA	-0,332405896	-1,862269442	1,529863546
1566973_at	-	-0,332405896	-1,862269442	1,529863546
1556909_at	LOC100505853	-0,332405896	-1,862269442	1,529863546
232954_at	-	-0,332405896	-1,862269442	1,529863546
203726_s_at	LAMA3	2,872644433	1,343200523	1,529443909
228727_at	ANXA11	1,97249974	0,443143223	1,529356517
202637_s_at	ICAM1	2,778527892	1,249194639	1,529333253
217285_at	DGCR14 /// TSSK2	0,556428218	-0,972893339	1,529321557
219807_x_at	MIA-RAB4B /// RA	2,739067176	1,209973561	1,529093614
230032_at	OSGEPL1	3,012642182	1,483698568	1,528943614
227609_at	EPSTI1	4,179494373	2,65087728	1,528617093
1555781_at	PQLC2	1,326463531	-0,201789721	1,528253252
217937_s_at	HDAC7	2,007220798	0,479089184	1,528131614
207281_x_at	VCX2	-0,088974936	-1,616924751	1,527949816
224985_at	NRAS	5,127954735	3,600063903	1,527890832
1554019_s_at	CEP57L1	0,244681185	-1,282520722	1,527201908
244348_at	-	0,244681185	-1,282520722	1,527201908
218416_s_at	SLC48A1	0,244681185	-1,282520722	1,527201908
222261_at	KIAA1609	0,244681185	-1,282520722	1,527201908
215841_at	GUCA1B	0,244681185	-1,282520722	1,527201908
230842_at	-	0,244681185	-1,282520722	1,527201908
244184_at	-	-0,384193355	-1,911206569	1,527013215
209955_s_at	FAP	-0,384193355	-1,911206569	1,527013215
1567297_at	OR13C4	-0,384193355	-1,911206569	1,527013215
241670_x_at	LOC729177	-0,384193355	-1,911206569	1,527013215
214320_x_at	CYP2A6	-0,384193355	-1,911206569	1,527013215
224409_s_at	TSSK6	-0,384193355	-1,911206569	1,527013215
207576_x_at	OXT	-0,384193355	-1,911206569	1,527013215
216141_at	-	-0,384193355	-1,911206569	1,527013215
221373_x_at	PSPN	-0,384193355	-1,911206569	1,527013215
226348_at	-	2,943764123	1,417157926	1,526606197
233451_at	SLC52A3	1,20396005	-0,322340048	1,526300098
202908_at	WFS1	1,20396005	-0,322340048	1,526300098
234351_x_at	TRPS1	0,791908897	-0,734355396	1,526264293
228034_x_at	ALKBH5 /// LOC10	0,425036312	-1,100943374	1,525979687
229237_s_at	-	0,425036312	-1,100943374	1,525979687
1566110_at	PIEZO1	0,425036312	-1,100943374	1,525979687
213766_x_at	GNA11	1,139057614	-0,386896102	1,525953716
231216_at	TMEM179	-0,043925712	-1,568385657	1,524459945

205418_at	FES	-0,043925712	-1,568385657	1,524459945
226226_at	TMEM45B	-0,043925712	-1,568385657	1,524459945
206046_at	ADAM23	-0,043925712	-1,568385657	1,524459945
208583_x_at	HIST1H2AJ	2,507817181	0,98381571	1,524001471
200710_at	ACADVL	4,891443931	3,367769667	1,523674264
201412_at	LRP10	4,209562243	2,686001625	1,523560618
211569_s_at	HADH	5,245449884	3,722097358	1,523352526
219314_s_at	ZNF219	1,071336699	-0,45121326	1,522549958
210296_s_at	PEX2	4,737491615	3,215288307	1,522203308
227963_at	-	2,530390387	1,008210238	1,522180149
213384_x_at	PLCB3	1,510970097	-0,011001236	1,521971333
213118_at	UHRF1BP1L	2,884644843	1,362900801	1,521744042
226040_at	-	2,920736393	1,399283506	1,521452887
236011_at	FOXK1	0,001114523	-1,520022123	1,521136646
223706_at	C22orf23	0,001114523	-1,520022123	1,521136646
234373_x_at	-	0,001114523	-1,520022123	1,521136646
229080_at	EMID2	0,001114523	-1,520022123	1,521136646
230111_at	-	-0,791294935	-2,312054429	1,520759494
244156_at	-	-0,791294935	-2,312054429	1,520759494
230455_at	PPP1R9B	-0,791294935	-2,312054429	1,520759494
205623_at	ALDH3A1	-0,791294935	-2,312054429	1,520759494
237221_at	-	-0,791294935	-2,312054429	1,520759494
236479_at	-	-0,791294935	-2,312054429	1,520759494
215756_at	LOC730227	0,707657549	-0,811769547	1,519427095
220375_s_at	-	0,707657549	-0,811769547	1,519427095
206955_at	AQP7 /// LOC1005	0,58788177	-0,931539147	1,519420917
225561_at	SELT	3,618399139	2,099079406	1,519319733
208266_at	C8orf17	-0,437449947	-1,956681069	1,519231122
1554231_a_at	ZNF396	-0,437449947	-1,956681069	1,519231122
1562651_at	ZSCAN1	-0,437449947	-1,956681069	1,519231122
211884_s_at	CIITA	-0,437449947	-1,956681069	1,519231122
231900_at	ZDHHC18	-0,437449947	-1,956681069	1,519231122
233829_at	C20orf118	-0,437449947	-1,956681069	1,519231122
1556962_at	-	-0,437449947	-1,956681069	1,519231122
216729_at	-	-0,437449947	-1,956681069	1,519231122
224211_at	FOXP3	-0,437449947	-1,956681069	1,519231122
233443_at	PSMG4	-0,437449947	-1,956681069	1,519231122
243021_at	-	-0,437449947	-1,956681069	1,519231122
234015_at	-	-0,437449947	-1,956681069	1,519231122
234875_at	-	3,466076444	1,946903052	1,519173392
202962_at	KIF13B	1,88491011	0,366031214	1,518878897
210540_s_at	B4GALT4	2,348178434	0,829309537	1,518868898
1562896_at	TNRC18	1,287793882	-0,231045907	1,518839789
209973_at	NFKBIL1	1,345611839	-0,172194225	1,517806063
218579_s_at	DHX35	2,118167848	0,600385088	1,51778276
242281_at	GLUL	0,045517965	-1,471833228	1,517351192
227189_at	CPNE5	0,045517965	-1,471833228	1,517351192
204150_at	STAB1	0,045517965	-1,471833228	1,517351192
1561390_at	FAM41AY1 /// FAM	0,045517965	-1,471833228	1,517351192
203443_at	EML3	0,045517965	-1,471833228	1,517351192

1553719_s_at	ZNF548	0,820160788	-0,697182596	1,517343384
232438_at	EPS15L1	0,820160788	-0,697182596	1,517343384
208978_at	CRIP2	0,282358733	-1,234835326	1,517194059
238259_at	ADSSL1	0,282358733	-1,234835326	1,517194059
232214_x_at	ZNF554	1,578719607	0,061773582	1,516946025
236973_at	LOC100131662	-0,66934516	-2,186086603	1,516741443
206598_at	INS	-0,66934516	-2,186086603	1,516741443
207679_at	PAX3	-0,66934516	-2,186086603	1,516741443
223718_at	ACRBP	-0,66934516	-2,186086603	1,516741443
1555044_a_at	KBTBD5	-0,66934516	-2,186086603	1,516741443
221585_at	CACNG4	-0,66934516	-2,186086603	1,516741443
1565751_at	FGD2	-0,66934516	-2,186086603	1,516741443
241104_at	-	-0,66934516	-2,186086603	1,516741443
213354_s_at	NR2F6	-1,289805289	-2,8064286	1,516623311
222483_at	EFHD2	2,50022283	0,98381571	1,516407121
205906_at	FOXJ1	0,458905032	-1,057301851	1,516206882
209957_s_at	NPPA	0,458905032	-1,057301851	1,516206882
234922_s_at	NHSL1	0,458905032	-1,057301851	1,516206882
231280_at	-	0,458905032	-1,057301851	1,516206882
221909_at	RNFT2	1,625482993	0,109458907	1,516024087
244074_at	LOC100129104	1,225123479	-0,290694975	1,515818454
235078_at	-	1,160996507	-0,354781582	1,515778089
221230_s_at	ARID4B	4,597104084	3,081435405	1,515668679
225244_at	LOC100130093 ///	4,885515979	3,36994189	1,515574089
204735_at	PDE4A	2,966903803	1,451507543	1,515396261
210499_s_at	PQBP1	0,926325262	-0,589001171	1,515326433
225980_at	C14orf43	3,291641956	1,777135134	1,514506822
202061_s_at	SEL1L	4,435374233	2,921154239	1,514219994
209407_s_at	DEAF1	2,752615529	1,239268196	1,513347333
225931_s_at	RNF213	3,710746795	2,197404429	1,513342365
222601_at	UBA6	3,09217775	1,578991006	1,513186744
220510_at	RHBG	1,476175815	-0,036543687	1,512719502
203010_at	STAT5A	3,374552989	1,861868165	1,512684824
234790_at	-	-0,49284695	-2,005029581	1,512182631
1567612_at	-	-0,49284695	-2,005029581	1,512182631
209496_at	RARRES2	-0,49284695	-2,005029581	1,512182631
221359_at	GDNF	-0,49284695	-2,005029581	1,512182631
237080_at	-	-0,49284695	-2,005029581	1,512182631
220635_at	PSORS1C2	-0,49284695	-2,005029581	1,512182631
1555339_at	RAP1A	-0,49284695	-2,005029581	1,512182631
233307_x_at	LOC100505828 ///	1,936380191	0,424693306	1,511686885
236388_at	STRN	2,613382867	1,101780212	1,511602655
213480_at	VAMP4	3,309267982	1,797682937	1,511585044
223569_at	PPAPDC1B	2,657308324	1,146010634	1,51129769
227017_at	ERICH1	3,668220666	2,157148588	1,511072078
224824_at	COX20	4,013714191	2,503049671	1,51066452
244095_at	ANKFN1	0,319287178	-1,19085694	1,510144118
208871_at	ATN1	0,319287178	-1,19085694	1,510144118
236913_at	-	0,319287178	-1,19085694	1,510144118
223129_x_at	MYLIP	2,843031657	1,333668715	1,509362943

219577_s_at	ABCA7	1,364555912	-0,144765583	1,509321495
229388_at	LOC100507212	2,392346173	0,883374248	1,508971925
223465_at	COL4A3BP	2,41945542	0,910483921	1,508971499
212111_at	STX12	3,969866565	2,460974054	1,508892511
211263_s_at	PCSK6	0,847529938	-0,661178575	1,508708512
204605_at	CGRRF1	2,621163856	1,112548615	1,508615241
213471_at	NPHP4	3,426291319	1,917743773	1,508547546
233522_at	-	0,08619576	-1,422257026	1,508452786
1556304_s_at	LOC100271722	0,08619576	-1,422257026	1,508452786
207360_s_at	NTSR1	0,08619576	-1,422257026	1,508452786
228957_at	ZNF362	0,08619576	-1,422257026	1,508452786
216892_at	IGHG1	0,08619576	-1,422257026	1,508452786
1553843_at	C10orf67	0,08619576	-1,422257026	1,508452786
227946_at	OSBPL7	2,190140919	0,681874787	1,508266133
222905_s_at	TMEM143	2,444186596	0,936232181	1,507954415
241056_at	ZC3H3	-1,06282519	-2,570522741	1,507697552
1562044_at	-	-1,06282519	-2,570522741	1,507697552
241059_at	-	-1,06282519	-2,570522741	1,507697552
236357_at	-	0,618277321	-0,889222211	1,507499532
241285_at	-	0,618277321	-0,889222211	1,507499532
233841_s_at	SUDS3	2,51558601	1,008210238	1,507375772
221968_s_at	ZNF771	0,735999505	-0,771340337	1,507339842
226167_at	SYT7	0,735999505	-0,771340337	1,507339842
244214_at	LOC100505554	0,735999505	-0,771340337	1,507339842
244160_at	-	0,491906512	-1,015398016	1,507304528
225952_at	FLYWCH1	0,491906512	-1,015398016	1,507304528
1560047_s_at	-	0,491906512	-1,015398016	1,507304528
222846_at	RAB8B	2,685337919	1,178100917	1,507237001
219388_at	GRHL2	-0,994186487	-2,501359113	1,507172626
231080_at	CDAN1	-0,994186487	-2,501359113	1,507172626
207013_s_at	MMP16	-0,994186487	-2,501359113	1,507172626
230070_at	CNIH2	1,544940605	0,03784737	1,507093235
202636_at	RNF103	3,362258367	1,855279162	1,506979206
218319_at	PELI1	2,705643918	1,199296812	1,506347106
48106_at	SLC48A1	2,138806815	0,633221821	1,505584994
206470_at	PLXNC1	1,182925501	-0,322340048	1,505265549
224301_x_at	H2AFJ	4,07586392	2,570802332	1,505061588
225509_at	SAP30L	3,366614157	1,861868165	1,504745992
242488_at	CHRM3	2,401508182	0,896834102	1,50467408
236823_at	IDS	-0,924000698	-2,4284334	1,504432702
1568719_s_at	SLC22A23	-0,924000698	-2,4284334	1,504432702
1554864_a_at	SDC3	-0,924000698	-2,4284334	1,504432702
217430_x_at	COL1A1	2,138605583	0,634301128	1,504304455
238612_at	-	2,811067138	1,306903969	1,504163168
212214_at	OPA1	3,485638812	1,981728801	1,503910011
220182_at	SLC25A23	1,830468611	0,326777209	1,503691402
230334_at	LOC100507291	1,830468611	0,326777209	1,503691402
224207_x_at	MMP28	1,440265662	-0,063377083	1,503642745
227761_at	MYO5A	2,605420261	1,101780212	1,503640049
220650_s_at	SLC9A5	1,116462765	-0,386896102	1,503358867

209533_s_at	PLAA	3,739061547	2,235857731	1,503203816
203814_s_at	NQO2	2,791258167	1,28853674	1,502721427
222175_s_at	MED15	2,085817135	0,583607503	1,502209632
212509_s_at	MXRA7	4,895653873	3,393801804	1,501852069
222787_s_at	TMEM106B	2,545254728	1,043469112	1,501785615
238574_at	SLC25A51	1,610353504	0,109458907	1,500894597
214177_s_at	PBXIP1	2,636121902	1,135235995	1,500885907
215867_x_at	CA12	0,355751005	-1,145057014	1,500808019
205874_at	ITPKA	0,355751005	-1,145057014	1,500808019
201247_at	SREBF2	2,56794744	1,067365565	1,500581875
207860_at	NCR1	0,127732831	-1,372734086	1,500466916
231667_at	SLC39A5	0,127732831	-1,372734086	1,500466916
231352_at	SLC22A8	0,127732831	-1,372734086	1,500466916
214891_at	FBXO21	0,127732831	-1,372734086	1,500466916
208353_x_at	ANK1	0,127732831	-1,372734086	1,500466916
220838_at	EXD3	0,127732831	-1,372734086	1,500466916
205272_s_at	PRH1 /// PRH2	0,127732831	-1,372734086	1,500466916
235542_at	TET3	3,257413106	1,757155322	1,500257785
47550_at	LZTS1	0,107916381	-1,391992302	1,499908683
232892_at	C20orf166	1,65554524	0,155747281	1,499797959
220235_s_at	LRIF1	3,716897585	2,2171284	1,499769185
204236_at	FLI1	4,359963018	2,860202326	1,499760691
1555753_x_at	ERVH-6	-0,730013898	-2,229233437	1,499219539
228428_at	FAM102A	-0,730013898	-2,229233437	1,499219539
243386_at	CASZ1	-0,730013898	-2,229233437	1,499219539
213829_x_at	RTEL1	1,326463531	-0,172194225	1,498657756
237592_at	ZC2HC1B	-0,551284523	-2,049747196	1,498462673
207255_at	LEPR	-0,551284523	-2,049747196	1,498462673
1563327_a_at	CXorf31	-0,551284523	-2,049747196	1,498462673
1553769_at	SLAMF9	-0,551284523	-2,049747196	1,498462673
1556542_a_at	-	-0,551284523	-2,049747196	1,498462673
1557620_a_at	CCDC38	-0,551284523	-2,049747196	1,498462673
1569909_at	KRT79	-0,551284523	-2,049747196	1,498462673
204390_at	-	-0,551284523	-2,049747196	1,498462673
1563638_at	FAM18A	-0,551284523	-2,049747196	1,498462673
216928_at	TAL1	-0,551284523	-2,049747196	1,498462673
233855_at	SCIMP	-0,551284523	-2,049747196	1,498462673
205015_s_at	TGFA	-0,551284523	-2,049747196	1,498462673
1567540_at	-	-0,551284523	-2,049747196	1,498462673
1563247_at	-	-0,551284523	-2,049747196	1,498462673
211049_at	TLX2	-0,551284523	-2,049747196	1,498462673
207836_s_at	RBPMS	-0,551284523	-2,049747196	1,498462673
225072_at	ZCCHC3	-1,137208348	-2,635549085	1,498340736
218387_s_at	PGLS	4,258822873	2,760632255	1,498190618
201307_at	40787	3,248151517	1,750483134	1,497668383
240598_at	-	0,524545436	-0,972893339	1,497438775
236477_at	PRPF40A	0,524545436	-0,972893339	1,497438775
239681_at	C17orf56	0,873789652	-0,623254098	1,49704375
202792_s_at	PPP6R2	0,873789652	-0,623254098	1,49704375
204574_s_at	MMP19	0,873789652	-0,623254098	1,49704375

222937_s_at	MMP28	0,873789652	-0,623254098	1,49704375
219709_x_at	FAM173A	2,642918178	1,146010634	1,496907545
204075_s_at	CEP104	2,575362572	1,078619386	1,496743186
203402_at	KCNAB2	3,478193934	1,981728801	1,496465133
209882_at	RIT1	3,57383031	2,077419168	1,496411141
225376_at	C20orf11	3,628556015	2,132145748	1,496410266
211456_x_at	MT1P2	4,972743529	3,47651952	1,496224009
208914_at	GGA2	2,2388565	0,742671819	1,496184681
226206_at	MAFK	2,063971513	0,568155442	1,495816071
227168_at	MIAT	2,705643918	1,209973561	1,495670356
216262_s_at	TGIF2	3,081633533	1,586121802	1,495511731
213134_x_at	BTG3	5,447596473	3,952350674	1,495245798
209414_at	FZR1	0,167727503	-1,32740143	1,495128933
1562689_at	LOC151484	0,167727503	-1,32740143	1,495128933
202229_s_at	CHERP	0,167727503	-1,32740143	1,495128933
224697_at	DCAF5	0,167727503	-1,32740143	1,495128933
210215_at	TFR2	0,167727503	-1,32740143	1,495128933
209331_s_at	MAX	3,03433073	1,539450683	1,494880047
215202_at	GUSBP11	0,97663406	-0,518232988	1,494867048
1554428_s_at	NLGN2	1,458255986	-0,036543687	1,494799673
202366_at	ACADS	1,458255986	-0,036543687	1,494799673
219882_at	TTLL7	1,760255626	0,265475485	1,494780141
228231_at	SNX8	1,20396005	-0,290694975	1,494655025
223039_at	C22orf13	3,795218988	2,300902761	1,494316227
1553413_at	FLJ13744	-0,856259172	-2,350444759	1,494185587
228036_s_at	FBXO2	-0,856259172	-2,350444759	1,494185587
242179_s_at	TSPAN16	-0,856259172	-2,350444759	1,494185587
230474_at	UBIAD1	-0,856259172	-2,350444759	1,494185587
237324_s_at	HKDC1	-0,856259172	-2,350444759	1,494185587
224725_at	MIB1	2,159837409	0,666486137	1,493351272
200849_s_at	AHCYL1	4,389611304	2,896872114	1,49273919
201534_s_at	UBL3	3,039689025	1,547376668	1,492312357
212807_s_at	SORT1	2,943764123	1,451507543	1,492256581
208188_at	KRT9	0,39105295	-1,100943374	1,491996325
240767_x_at	VPS13A-AS1	0,39105295	-1,100943374	1,491996325
225004_at	TMEM101	2,926639205	1,434743608	1,491895597
225005_at	PHF13	4,123563902	2,63203137	1,491532532
223450_s_at	COG3	2,427650693	0,936232181	1,491418512
223252_at	HDGFRP2	2,074841895	0,583607503	1,491234392
34726_at	CACNB3	0,793183896	-0,697303073	1,490486969
234748_x_at	KIF20B	-1,289805289	-2,780225148	1,490419859
226989_at	RGMB	1,345611839	-0,144765583	1,490377422
1556227_at	VCPIP1	0,20740896	-1,282520722	1,489929682
210364_at	SCN2B	0,20740896	-1,282520722	1,489929682
239263_at	LOC100506813	0,20740896	-1,282520722	1,489929682
207504_at	CA7	0,20740896	-1,282520722	1,489929682
238431_at	-	2,989672611	1,500059916	1,489612695
222865_x_at	TMEM204	0,677745787	-0,811769547	1,489515334
244307_s_at	LOC100507539	0,791908897	-0,697182596	1,489091494
234761_at	-	0,900065578	-0,589001171	1,489066749

204479_at	OSTF1	4,448374788	2,960011739	1,488363049
212858_at	PAQR4	2,138605583	0,65030602	1,488299564
212908_at	DNAJC16	3,470020347	1,981728801	1,488291546
230851_x_at	C16orf13	0,556428218	-0,931539147	1,487967365
221825_at	ANGEL2	4,500752401	3,012928641	1,487823759
218623_at	HMP19	-0,609040214	-2,09678811	1,487747896
238326_at	ODF3B	-0,609040214	-2,09678811	1,487747896
234291_s_at	SLC6A20	-0,609040214	-2,09678811	1,487747896
1558502_s_at	DNM3	-0,609040214	-2,09678811	1,487747896
1556970_at	-	-0,609040214	-2,09678811	1,487747896
213656_s_at	KLC1	4,586396217	3,099629381	1,486766836
234984_at	NEDD1	4,790575561	3,304296123	1,486279438
1561286_a_at	DIP2A	1,225123479	-0,260526297	1,485649776
201824_at	RNF14	3,551632175	2,066329863	1,485302311
1557154_at	-	-0,230432956	-1,715576125	1,485143169
1553429_at	FLJ31713	-0,230432956	-1,715576125	1,485143169
237823_at	-	-0,230432956	-1,715576125	1,485143169
242713_at	-	-0,230432956	-1,715576125	1,485143169
214832_at	HNF4A	-0,230432956	-1,715576125	1,485143169
241629_at	-	-0,230432956	-1,715576125	1,485143169
221215_s_at	RIPK4	-0,230432956	-1,715576125	1,485143169
228816_at	ATP6AP1L /// FLJ4	1,000459215	-0,484558493	1,485017708
215182_x_at	-	1,000459215	-0,484558493	1,485017708
220648_at	ADARB2	-0,180990326	-1,665791776	1,48480145
1553875_s_at	ZSCAN10	-0,180990326	-1,665791776	1,48480145
231838_at	PABPC1L	-0,180990326	-1,665791776	1,48480145
241600_at	WIPF3	-0,180990326	-1,665791776	1,48480145
216706_x_at	IGHG1 /// LOC642	-0,180990326	-1,665791776	1,48480145
233743_x_at	S1PR5	-0,180990326	-1,665791776	1,48480145
236794_at	-	-0,180990326	-1,665791776	1,48480145
240418_at	-	-0,180990326	-1,665791776	1,48480145
241633_x_at	-	-0,180990326	-1,665791776	1,48480145
208898_at	ATP6V1D	4,779371898	3,295596077	1,483775821
212665_at	TIPARP	5,358104722	3,874467807	1,483636915
211512_s_at	OGFR	1,830468611	0,346964736	1,483503876
220447_at	HRH3	-0,280273599	-1,763664074	1,483390475
206744_s_at	ZMYM5	-0,280273599	-1,763664074	1,483390475
1553316_at	GPR82	-0,280273599	-1,763664074	1,483390475
239127_at	LOC100288860	-0,280273599	-1,763664074	1,483390475
211495_x_at	TNFSF12 /// TNFSF	-0,280273599	-1,763664074	1,483390475
208604_s_at	HOXA3	-0,280273599	-1,763664074	1,483390475
1568627_at	SMEK2	1,160996507	-0,322340048	1,483336555
212689_s_at	KDM3A	4,614537809	3,131713063	1,482824747
241992_at	DRAM1	0,425036312	-1,057301851	1,482338163
221315_s_at	FGF22	0,425036312	-1,057301851	1,482338163
243596_at	-	0,425036312	-1,057301851	1,482338163
213942_at	MEGF6	0,425036312	-1,057301851	1,482338163
223788_at	GTPBP2	0,425036312	-1,057301851	1,482338163
218927_s_at	CHST12	3,732463367	2,250228409	1,482234958
1554067_at	C12orf66	3,45766549	1,975797592	1,481867898

229918_at	CCDC40	-0,135065865	-1,616924751	1,481858886
207316_at	HAS1	-0,135065865	-1,616924751	1,481858886
208507_at	OR7C2	-0,135065865	-1,616924751	1,481858886
212128_s_at	DAG1	-0,135065865	-1,616924751	1,481858886
230994_at	FAM181B	-0,135065865	-1,616924751	1,481858886
224485_s_at	SLC30A2	-0,135065865	-1,616924751	1,481858886
222339_x_at	-	-0,135065865	-1,616924751	1,481858886
210624_s_at	ILVBL	2,605420261	1,123905343	1,481514918
220246_at	CAMK1D	1,364555912	-0,116767475	1,481323387
213216_at	OTUD3	1,364555912	-0,116767475	1,481323387
207159_x_at	CRTC1	0,926325262	-0,554696666	1,481021928
224859_at	CD276	1,094118704	-0,386896102	1,481014806
203566_s_at	AGL	4,669720908	3,188806254	1,480914654
212398_at	RDX	2,582572508	1,101780212	1,480792296
226474_at	NLRC5	3,768156719	2,287674614	1,480482105
1554559_at	GPR62	0,244681185	-1,234835326	1,479516512
215153_at	NOS1AP	0,244681185	-1,234835326	1,479516512
216608_at	DKFZP434A062	0,244681185	-1,234835326	1,479516512
216599_x_at	SLC22A6	-0,088974936	-1,568385657	1,479410721
221336_at	ATOH1	-0,088974936	-1,568385657	1,479410721
234451_at	-	-0,088974936	-1,568385657	1,479410721
216269_s_at	ELN	-0,088974936	-1,568385657	1,479410721
228639_at	-	1,307201325	-0,172194225	1,47939555
231212_x_at	-	1,307201325	-0,172194225	1,47939555
227839_at	MBD5	-0,332405896	-1,811780055	1,479374159
210304_at	PDE6B	-0,332405896	-1,811780055	1,479374159
1569422_at	FAM129C	-0,332405896	-1,811780055	1,479374159
214065_s_at	CIB2	-0,332405896	-1,811780055	1,479374159
216707_at	-	-0,332405896	-1,811780055	1,479374159
207323_s_at	MBP	-0,332405896	-1,811780055	1,479374159
227484_at	SRGAP1	0,707657549	-0,771340337	1,478997885
238501_at	-	0,707657549	-0,771340337	1,478997885
227399_at	VGLL3	-0,791294935	-2,27001641	1,478721475
235243_at	CLIP3	-0,791294935	-2,27001641	1,478721475
244064_at	-	-0,791294935	-2,27001641	1,478721475
231525_at	IQCF5	-0,791294935	-2,27001641	1,478721475
1559092_at	SLC9A5	-0,791294935	-2,27001641	1,478721475
201428_at	CLDN4	-0,384193355	-1,862269442	1,478076087
1563119_at	HP09025	-0,384193355	-1,862269442	1,478076087
214286_at	GNAT1	-0,384193355	-1,862269442	1,478076087
239013_at	SEC22C	-0,384193355	-1,862269442	1,478076087
227914_s_at	UBE3B	-0,384193355	-1,862269442	1,478076087
210408_s_at	CPNE6	-0,384193355	-1,862269442	1,478076087
210456_at	PCYT1B	-0,384193355	-1,862269442	1,478076087
223258_s_at	G2E3	2,590269697	1,112548615	1,477721082
219591_at	CEND1	1,246401134	-0,231045907	1,47744704
227302_s_at	FLII /// LLGL1	0,58788177	-0,889222211	1,477103981
203933_at	RAB11FIP3	1,440265662	-0,036543687	1,476809349
230165_at	SGOL2	3,570321555	2,093850742	1,476470813
206649_s_at	TFE3	1,561816652	0,085534992	1,47628166

241650_x_at	HMCN2 /// LOC10	1,561816652	0,085534992	1,47628166
220167_s_at	LOC729264 /// TP!	-0,043925712	-1,520022123	1,476096412
230665_at	GUCY1A2	-0,043925712	-1,520022123	1,476096412
1563224_at	-	-0,043925712	-1,520022123	1,476096412
1563783_a_at	ZNF333	-0,043925712	-1,520022123	1,476096412
230941_at	LOC728537	-0,043925712	-1,520022123	1,476096412
206072_at	UCN	1,802733148	0,326777209	1,475955938
209580_s_at	MBD4	4,705123614	3,229534127	1,475589487
210257_x_at	CUL4B	2,507817181	1,032267596	1,475549585
1558342_x_at	DIXDC1	1,024284941	-0,45121326	1,475498201
218466_at	TBC1D17	1,024284941	-0,45121326	1,475498201
226745_at	CYP4V2	1,97249974	0,497424714	1,475075025
227252_at	LRP10	1,97249974	0,497424714	1,475075025
208954_s_at	LARP4B	2,642918178	1,167919222	1,474998957
205136_s_at	NUFIP1	1,936380191	0,461385738	1,474994454
225084_at	EXOC5	4,337778276	2,86314199	1,474636286
216388_s_at	LTB4R	0,458905032	-1,015398016	1,474303048
244889_at	LOC388210	0,458905032	-1,015398016	1,474303048
214308_s_at	HGD	-0,437449947	-1,911206569	1,473756622
230807_at	CCDC151	-0,437449947	-1,911206569	1,473756622
231414_at	-	-0,437449947	-1,911206569	1,473756622
217258_x_at	IGLV1-44	-0,437449947	-1,911206569	1,473756622
1567030_at	SH3GL1P2	-0,437449947	-1,911206569	1,473756622
243989_at	-	-0,437449947	-1,911206569	1,473756622
214556_at	SSTR4	-0,437449947	-1,911206569	1,473756622
202434_s_at	CYP1B1	-0,437449947	-1,911206569	1,473756622
229755_x_at	DLL3	-0,437449947	-1,911206569	1,473756622
234736_at	JPH3	-0,437449947	-1,911206569	1,473756622
234319_at	STOX2	-0,437449947	-1,911206569	1,473756622
225789_at	AGAP3	2,575362572	1,101780212	1,47358236
244739_at	RDX	-1,06282519	-2,536339282	1,473514092
234550_at	-	-1,06282519	-2,536339282	1,473514092
217582_at	-	-1,06282519	-2,536339282	1,473514092
214304_x_at	-	-1,06282519	-2,536339282	1,473514092
233797_s_at	CST11	-1,06282519	-2,536339282	1,473514092
1554830_a_at	STEAP3	-1,06282519	-2,536339282	1,473514092
1552319_a_at	KLK8	-1,06282519	-2,536339282	1,473514092
1554610_at	ANKMY1	-1,06282519	-2,536339282	1,473514092
1559400_s_at	PAPPA	-1,06282519	-2,536339282	1,473514092
235010_at	LOC729013	2,890618076	1,417157926	1,473460149
208732_at	RAB2A	2,38349585	0,910483921	1,47301193
204961_s_at	NCF1 /// NCF1B //,	0,001114523	-1,471833228	1,47294775
1564431_a_at	LCNL1	0,001114523	-1,471833228	1,47294775
211818_s_at	PDE4C	0,001114523	-1,471833228	1,47294775
239918_at	-	0,001114523	-1,471833228	1,47294775
233403_x_at	TM6SF2	0,001114523	-1,471833228	1,47294775
239473_x_at	MAP6D1	0,001114523	-1,471833228	1,47294775
234846_at	DKFZp761P0212	0,001114523	-1,471833228	1,47294775
234395_at	-	-0,66934516	-2,142286822	1,472941662
229836_s_at	NUDT4P1	-0,66934516	-2,142286822	1,472941662

210930_s_at	ERBB2	-0,66934516	-2,142286822	1,472941662
231140_at	TPPP2	-0,66934516	-2,142286822	1,472941662
227734_s_at	ZNHIT2	-0,66934516	-2,142286822	1,472941662
1566527_at	-	-0,66934516	-2,142286822	1,472941662
229945_at	CYHR1	-0,66934516	-2,142286822	1,472941662
225069_at	PCYT1A	2,199941239	0,727123109	1,47281813
209984_at	KDM4C	3,081633533	1,60918327	1,472450262
224538_s_at	PARD6G	-0,994186487	-2,466349414	1,472162927
209354_at	TNFRSF14	2,62869474	1,156599976	1,472094763
39729_at	PRDX2	6,786137854	5,314161327	1,471976527
203605_at	SRP54	4,429046471	2,957089023	1,471957448
212906_at	GRAMD1B	2,41945542	0,947673886	1,471781534
204097_s_at	RBMX2	2,778527892	1,306903969	1,471623922
204974_at	RAB3A	1,116462765	-0,354781582	1,471244346
216458_at	PQLC3	0,847529938	-0,623254098	1,470784036
224431_s_at	SUV420H2	0,847529938	-0,623254098	1,470784036
220213_at	TSHZ2	0,847529938	-0,623254098	1,470784036
223911_at	LOC440330	0,95222556	-0,518232988	1,470458549
213260_at	FOXC1	0,95222556	-0,518232988	1,470458549
203955_at	PPP1R26	0,95222556	-0,518232988	1,470458549
212276_at	LPIN1	4,321477036	2,851300913	1,470176123
203172_at	FXR2	1,816938997	0,346964736	1,469974261
38447_at	ADRBK1	1,974819539	0,505060597	1,469758942
225675_at	C14orf101	2,339581693	0,869824768	1,469756925
221751_at	PANK3	4,855289601	3,385678356	1,469611246
202296_s_at	RER1	5,341264459	3,871747013	1,469517446
1555973_at	-	1,578719607	0,109458907	1,4692607
207350_s_at	VAMP4	1,458255986	-0,011001236	1,469257222
210422_x_at	SLC11A1	1,267146969	-0,201789721	1,46893669
202465_at	PCOLCE	1,267146969	-0,201789721	1,46893669
223265_at	SH3BP5L	1,983901861	0,515457602	1,468444259
218640_s_at	PLEKHF2	3,142467702	1,674071917	1,468395786
1566990_x_at	ARID1B	2,678352494	1,209973561	1,468378932
214512_s_at	SUB1	6,95567265	5,487366217	1,468306433
1565674_at	FCGR2A /// FCGR2	-0,924000698	-2,391863529	1,467862831
215277_at	PCDH1	-0,924000698	-2,391863529	1,467862831
241895_at	LOC440905	-0,924000698	-2,391863529	1,467862831
234514_at	SRMS	-0,924000698	-2,391863529	1,467862831
1570480_s_at	ART1	-0,924000698	-2,391863529	1,467862831
208211_s_at	ALK	-0,924000698	-2,391863529	1,467862831
221722_x_at	LZTS1	-0,924000698	-2,391863529	1,467862831
240171_at	-	-0,924000698	-2,391863529	1,467862831
1554649_at	-	0,045517965	-1,422257026	1,467774991
211809_x_at	COL13A1	0,045517965	-1,422257026	1,467774991
233612_at	-	0,045517965	-1,422257026	1,467774991
242335_at	SLC25A37	0,618277321	-0,84928999	1,467567311
206778_at	CRYBB2	0,618277321	-0,84928999	1,467567311
204600_at	EPHB3	0,618277321	-0,84928999	1,467567311
201827_at	SMARCD2	3,599444854	2,132145748	1,467299105
200843_s_at	EPRS	5,602635435	4,135643482	1,466991953

229503_at	SLC15A4	-1,137208348	-2,603471267	1,466262918
209652_s_at	PGF	-1,137208348	-2,603471267	1,466262918
229113_s_at	C1orf86	3,132805465	1,666953618	1,465851848
202313_at	PPP2R2A	5,473061201	4,007339482	1,465721718
209714_s_at	CDKN3	5,321230734	3,855575208	1,465655526
236410_x_at	-	1,047341799	-0,418248858	1,465590657
1565894_at	-	0,491906512	-0,972893339	1,46479985
202928_s_at	PHF1	0,491906512	-0,972893339	1,46479985
223726_at	KCNH3	0,491906512	-0,972893339	1,46479985
239248_at	LOC100507495	0,319287178	-1,145057014	1,464344192
240387_at	UBASH3B	0,319287178	-1,145057014	1,464344192
208404_x_at	KCNJ5	0,319287178	-1,145057014	1,464344192
229617_x_at	AP2A1	0,319287178	-1,145057014	1,464344192
228079_at	C3orf58	-0,49284695	-1,956681069	1,463834119
230824_at	10.03.15	-0,49284695	-1,956681069	1,463834119
231246_at	-	-0,49284695	-1,956681069	1,463834119
205073_at	CYP2J2	-0,49284695	-1,956681069	1,463834119
221668_s_at	DNAI2	-0,49284695	-1,956681069	1,463834119
227225_at	ZNF503	-0,49284695	-1,956681069	1,463834119
232518_at	PRIC285	-0,49284695	-1,956681069	1,463834119
218063_s_at	CDC42EP4	-0,49284695	-1,956681069	1,463834119
1554681_a_at	MGC50722	-0,49284695	-1,956681069	1,463834119
227266_s_at	FYB	3,541158018	2,077419168	1,463738849
204522_at	DOM3Z	0,873789652	-0,589001171	1,462790823
209413_at	B4GALT2	1,345611839	-0,116767475	1,462379313
239754_at	C17orf76-AS1 /// S	1,345611839	-0,116767475	1,462379313
203996_s_at	C21orf2	1,345611839	-0,116767475	1,462379313
223214_s_at	ZHX1	2,978070706	1,516067292	1,462003415
209689_at	CCDC93	1,640193838	0,178191865	1,462001973
204742_s_at	PDS5B	1,640193838	0,178191865	1,462001973
228460_at	ZNF319	1,5944803	0,133104519	1,461375781
225487_at	TMEM18	1,5944803	0,133104519	1,461375781
1570026_at	CPXM2	0,764143511	-0,697182596	1,461326107
244429_at	-	0,764143511	-0,697182596	1,461326107
212602_at	WDFY3	0,764143511	-0,697182596	1,461326107
208185_x_at	-	0,97663406	-0,484558493	1,461192553
210024_s_at	UBE2E3	5,502101814	4,041201812	1,460900001
202360_at	MAML1	3,720319513	2,260052727	1,460266786
228521_s_at	MIA-RAB4B /// RA	3,837502555	2,37755543	1,459947125
214075_at	NENF	3,406503548	1,946903052	1,459600496
211404_s_at	APLP2	2,41945542	0,960042218	1,459413201
211018_at	LSS	1,544940605	0,085534992	1,459405613
221495_s_at	TCF25	3,892081305	2,432792627	1,459288678
232487_at	SFT2D1	0,08619576	-1,372734086	1,458929845
1562853_x_at	-	0,08619576	-1,372734086	1,458929845
213843_x_at	SLC6A8	0,08619576	-1,372734086	1,458929845
228136_s_at	C17orf70	0,08619576	-1,372734086	1,458929845
241985_at	JMY	1,843966654	0,385582632	1,458384022
226693_at	SDHAP1 /// SDHAF	3,45766549	1,999354538	1,458310952
208852_s_at	CANX	4,718186291	3,259878841	1,45830745

237046_x_at	IL34	1,071336699	-0,386896102	1,458232801
200976_s_at	TAX1BP1	6,122457274	4,665039799	1,457417475
241361_at	-	0,355751005	-1,100943374	1,456694379
209051_s_at	RALGDS	2,229377375	0,772793615	1,456583761
208494_at	SLC6A7	0,524545436	-0,931539147	1,456084583
243267_x_at	EFNA2	0,524545436	-0,931539147	1,456084583
229161_at	-	0,524545436	-0,931539147	1,456084583
214856_at	SPTBN1	-0,730013898	-2,186086603	1,456072705
201324_at	EMP1	-0,730013898	-2,186086603	1,456072705
222049_s_at	RBP4	-0,730013898	-2,186086603	1,456072705
221332_at	BMP15	-0,730013898	-2,186086603	1,456072705
218934_s_at	HSPB7	-0,730013898	-2,186086603	1,456072705
210327_s_at	AGXT	-0,730013898	-2,186086603	1,456072705
216074_x_at	WWC1	-0,730013898	-2,186086603	1,456072705
1559315_s_at	SOCS2-AS1	-0,730013898	-2,186086603	1,456072705
1558154_at	-	-0,730013898	-2,186086603	1,456072705
221334_s_at	FOXP3	-0,730013898	-2,186086603	1,456072705
203311_s_at	ARF6	3,900805316	2,444951949	1,455853367
214977_at	-	-0,856259172	-2,312054429	1,455795257
222224_at	NACA2	-0,856259172	-2,312054429	1,455795257
217032_at	FOXD4 /// FOXD4L	-0,856259172	-2,312054429	1,455795257
216261_at	ITGB3	-0,856259172	-2,312054429	1,455795257
227954_at	ITPRIPL2	-0,856259172	-2,312054429	1,455795257
220091_at	SLC2A6	2,523100136	1,067365565	1,455734571
223168_at	RHOU	2,523100136	1,067365565	1,455734571
225002_s_at	SUMF2	4,113919949	2,65824137	1,455678578
211835_at	IGK@ /// IGKC	0,127732831	-1,32740143	1,455134261
221748_s_at	TNS1	0,127732831	-1,32740143	1,455134261
1552271_at	PRR22	0,127732831	-1,32740143	1,455134261
1554129_a_at	ADIG	0,127732831	-1,32740143	1,455134261
220453_at	PQLC2	0,127732831	-1,32740143	1,455134261
201809_s_at	ENG	0,127732831	-1,32740143	1,455134261
230916_at	NODAL	0,127732831	-1,32740143	1,455134261
231403_at	TRIO	0,127732831	-1,32740143	1,455134261
209958_s_at	BBS9	1,898032047	0,443143223	1,454888824
208165_s_at	PRSS16	0,900065578	-0,554696666	1,454762244
238709_at	ELP2	0,900065578	-0,554696666	1,454762244
201502_s_at	NFKBIA	5,860194893	4,40626232	1,453932573
203193_at	ESRRA	1,760255626	0,306454867	1,453800759
224388_s_at	COL25A1	-0,551284523	-2,005029581	1,453745058
231078_at	SLC25A37	-0,551284523	-2,005029581	1,453745058
231413_at	-	-0,551284523	-2,005029581	1,453745058
217382_at	-	-0,551284523	-2,005029581	1,453745058
217507_at	SLC11A1	-0,551284523	-2,005029581	1,453745058
243893_at	KCNC3	-0,551284523	-2,005029581	1,453745058
230568_x_at	DLL3	-0,551284523	-2,005029581	1,453745058
229158_at	WNK4	-0,551284523	-2,005029581	1,453745058
242199_at	-	-0,551284523	-2,005029581	1,453745058
231054_at	SPACA4	-0,551284523	-2,005029581	1,453745058
1560189_at	-	-0,551284523	-2,005029581	1,453745058

212508_at	MOAP1	5,28227713	3,829184964	1,453092166
232076_at	ZNF707	0,791908897	-0,661178575	1,453087472
209433_s_at	PPAT	4,149350729	2,696595318	1,452755412
1569542_at	-	-1,212652659	-2,665156778	1,452504119
1559843_s_at	-	-1,212652659	-2,665156778	1,452504119
213540_at	HSD17B8	3,473999051	2,021531611	1,45246744
223400_s_at	PBRM1	1,561816652	0,109458907	1,452357746
205223_at	DEPDC5	1,307201325	-0,144765583	1,451966908
1564117_at	HMCN2 /// LOC10	1,160996507	-0,290694975	1,451691482
1556042_s_at	LOC338799	1,160996507	-0,290694975	1,451691482
210982_s_at	HLA-DRA	6,853975414	5,402471334	1,451504079
218048_at	COMMD3	5,813425326	4,361943785	1,451481541
223441_at	SLC17A5	2,784898907	1,333668715	1,451230193
212359_s_at	KIAA0913	1,983901861	0,533258442	1,450643419
232467_at	KIRREL	0,167727503	-1,282520722	1,450248226
220047_at	SIRT4	0,167727503	-1,282520722	1,450248226
230491_at	-	0,167727503	-1,282520722	1,450248226
225262_at	FOSL2	0,167727503	-1,282520722	1,450248226
202089_s_at	SLC39A6	4,070902947	2,621003244	1,449899703
205482_x_at	SNX15	3,537317882	2,088449486	1,448868396
206443_at	RORB	0,39105295	-1,057301851	1,448354801
1569385_s_at	TET2	0,39105295	-1,057301851	1,448354801
211004_s_at	ALDH3B1	0,39105295	-1,057301851	1,448354801
221425_s_at	ISCA1	2,38349585	0,936232181	1,447263669
223262_s_at	FGFR1OP2	3,190097062	1,743236669	1,446860392
216986_s_at	IRF4	1,383446753	-0,063377083	1,446823835
209860_s_at	ANXA7	6,283938077	4,837330315	1,446607762
209383_at	DDIT3	3,422378845	1,975797592	1,446581253
223227_at	BBS2	2,127965541	0,681874787	1,446090754
211423_s_at	SC5DL	3,8107521	2,364676067	1,446076033
223157_at	NOA1	4,348261798	2,902771765	1,445490033
201473_at	JUNB	2,50022283	1,055317091	1,444905739
228445_at	AIFM2	1,730929079	0,286279868	1,444649211
221982_x_at	LAGE3	1,730929079	0,286279868	1,444649211
203747_at	AQP3	0,926325262	-0,518232988	1,44455825
1552811_at	WFIKKN1	0,926325262	-0,518232988	1,44455825
215030_at	GRSF1	0,926325262	-0,518232988	1,44455825
211742_s_at	EVI2B	4,409540371	2,965609445	1,443930927
210789_x_at	CEACAM3	0,820160788	-0,623254098	1,443414886
204316_at	RGS10	1,326463531	-0,116767475	1,443231006
200953_s_at	CCND2	6,409190805	4,966480369	1,442710437
238551_at	FUT11	1,528073958	0,085534992	1,442538966
226376_at	UNK	1,024284941	-0,418248858	1,442533799
232212_at	PLEKHA8	0,20740896	-1,234835326	1,442244286
224113_at	CARD14	0,20740896	-1,234835326	1,442244286
243411_at	POLDIP3	0,20740896	-1,234835326	1,442244286
231505_s_at	SFXN4	0,20740896	-1,234835326	1,442244286
1557073_s_at	TTBK2	0,707657549	-0,734355396	1,442012945
218906_x_at	KLC2	2,949541692	1,508107415	1,441434277
1560116_a_at	NEDD1	5,146801687	3,70595963	1,440842056

217124_at	IQCE	-0,609040214	-2,049747196	1,440706982
241399_at	FAM19A2	-0,609040214	-2,049747196	1,440706982
221157_s_at	FBXO24	-0,609040214	-2,049747196	1,440706982
229486_s_at	TMEM132A	-0,609040214	-2,049747196	1,440706982
226261_at	ZNRF2	2,107136847	0,666486137	1,440650709
233586_s_at	KLK12	0,425036312	-1,015398016	1,440434329
229581_at	ELFN1	0,425036312	-1,015398016	1,440434329
205276_s_at	GTPBP1	0,425036312	-1,015398016	1,440434329
228964_at	PRDM1	2,866549889	1,426280426	1,440269462
226056_at	ARHGAP31	1,267146969	-0,172194225	1,439341194
202165_at	PPP1R2	3,694479488	2,255165935	1,439313553
231536_at	-	-1,06282519	-2,501359113	1,438533923
1559114_a_at	CXCR7	-1,06282519	-2,501359113	1,438533923
220392_at	EBF2	-1,06282519	-2,501359113	1,438533923
229254_at	MFSD4	-1,06282519	-2,501359113	1,438533923
212608_s_at	-	4,799977847	3,361641325	1,438336522
1554969_x_at	DIP2A	-0,791294935	-2,229233437	1,437938502
237869_at	A2LD1	-0,791294935	-2,229233437	1,437938502
216066_at	ABCA1	-0,791294935	-2,229233437	1,437938502
223627_at	MEX3B	-0,791294935	-2,229233437	1,437938502
231575_at	EGR4	-0,791294935	-2,229233437	1,437938502
220053_at	GDF3	-0,791294935	-2,229233437	1,437938502
222009_at	CEMP1	-0,791294935	-2,229233437	1,437938502
215576_at	-	-0,791294935	-2,229233437	1,437938502
218060_s_at	C16orf57	2,348178434	0,910483921	1,437694514
204639_at	ADA	5,710011704	4,272475625	1,43753608
222572_at	PDP1	3,081633533	1,644429915	1,437203618
226650_at	ZFAND2A	3,513985141	2,077419168	1,436565973
234969_s_at	EPC1	3,628556015	2,192329358	1,436226657
1558052_at	TMED4	-0,180990326	-1,616924751	1,435934425
242754_at	-	-0,180990326	-1,616924751	1,435934425
238321_at	TEAD2	-0,180990326	-1,616924751	1,435934425
206834_at	HBD	-0,180990326	-1,616924751	1,435934425
1564352_at	CECR3	-0,180990326	-1,616924751	1,435934425
228940_at	NDUFB4	-0,180990326	-1,616924751	1,435934425
238667_at	TCF20	-0,180990326	-1,616924751	1,435934425
213740_s_at	LOC100130348	-0,180990326	-1,616924751	1,435934425
213636_at	KIAA1045	-0,180990326	-1,616924751	1,435934425
241008_at	-	0,244681185	-1,19085694	1,435538125
217166_at	-	0,244681185	-1,19085694	1,435538125
1553285_s_at	RAD9B	-0,230432956	-1,665791776	1,43535882
215826_x_at	ZNF835	-0,230432956	-1,665791776	1,43535882
232948_at	-	-0,230432956	-1,665791776	1,43535882
207087_x_at	ANK1	-0,230432956	-1,665791776	1,43535882
209885_at	RHOD	-0,280273599	-1,715576125	1,435302526
241076_at	-	-0,280273599	-1,715576125	1,435302526
1563181_a_at	-	-0,280273599	-1,715576125	1,435302526
234764_x_at	CKAP2 /// IGLC1 //	-0,994186487	-2,4284334	1,434246913
1559125_at	-	-0,994186487	-2,4284334	1,434246913
211393_at	PATZ1	-0,994186487	-2,4284334	1,434246913

216372_at	-	-0,994186487	-2,4284334	1,434246913
230694_at	SLC35E2B	-0,994186487	-2,4284334	1,434246913
217641_at	GPR135	-0,994186487	-2,4284334	1,434246913
233345_at	EFCAB6	-0,994186487	-2,4284334	1,434246913
1565320_at	RBMY3AP	-0,994186487	-2,4284334	1,434246913
225328_at	FBXO32	1,047341799	-0,386896102	1,434237901
223071_at	IER3IP1	5,243853763	3,809801648	1,434052115
219900_s_at	ZNF446	1,760255626	0,326777209	1,433478416
221218_s_at	TPK1	2,356619952	0,923203038	1,433416913
220241_at	TMCO3	-0,135065865	-1,568385657	1,433319792
220280_s_at	ANKMY1	-0,135065865	-1,568385657	1,433319792
228895_s_at	-	-0,135065865	-1,568385657	1,433319792
211266_s_at	GPR4	-0,135065865	-1,568385657	1,433319792
1555809_at	CRISPLD2	-0,135065865	-1,568385657	1,433319792
231462_at	TMEM114	-0,135065865	-1,568385657	1,433319792
231522_at	C20orf144	-1,137208348	-2,570522741	1,433314393
1553531_at	PCSK6	-1,137208348	-2,570522741	1,433314393
231961_at	RBPMS	-1,289805289	-2,723114724	1,433309435
239086_at	-	0,735999505	-0,697182596	1,433182101
225271_at	TMEM63B	1,948628481	0,515457602	1,433170879
208654_s_at	CD164	5,471924989	4,038837464	1,433087526
219297_at	WDR44	2,932161212	1,500059916	1,432101296
207822_at	FGFR1	0,458905032	-0,972893339	1,43179837
239501_at	-	-0,332405896	-1,763664074	1,431258178
233151_s_at	TTTY7	-0,332405896	-1,763664074	1,431258178
243774_at	MUC20	-0,332405896	-1,763664074	1,431258178
243112_at	-	-0,332405896	-1,763664074	1,431258178
1554895_a_at	RHBDL2	-0,332405896	-1,763664074	1,431258178
1561502_x_at	CTU2	-0,332405896	-1,763664074	1,431258178
1557185_at	TPCN1	-0,332405896	-1,763664074	1,431258178
229596_at	AMDHD1	-0,088974936	-1,520022123	1,431047188
206185_at	CRYBB1	-0,088974936	-1,520022123	1,431047188
206368_at	CPLX2	-0,088974936	-1,520022123	1,431047188
230475_at	C15orf59	-0,088974936	-1,520022123	1,431047188
214546_s_at	P2RY11 /// PPAN-F	-0,088974936	-1,520022123	1,431047188
235671_at	-	-0,088974936	-1,520022123	1,431047188
212210_at	INTS1	-0,088974936	-1,520022123	1,431047188
220733_at	SLC26A1	-0,088974936	-1,520022123	1,431047188
227781_x_at	FAM57B	-0,088974936	-1,520022123	1,431047188
225214_at	LOC100129034	2,127965541	0,697221375	1,430744166
242157_at	CHD9	0,618277321	-0,811769547	1,430046868
232248_at	LOC100652768 ///	0,618277321	-0,811769547	1,430046868
206257_at	CCDC9	0,618277321	-0,811769547	1,430046868
214174_s_at	PDLIM4	0,618277321	-0,811769547	1,430046868
241748_x_at	DGCR14	0,618277321	-0,811769547	1,430046868
206405_x_at	USP6	2,285848435	0,85655369	1,429294746
209389_x_at	DBI	6,993937096	5,564856596	1,4290805
204690_at	STX8	4,816454496	3,387778112	1,428676384
205718_at	ITGB7	3,771154436	2,34250725	1,428647186
234948_at	SLC27A5	0,873789652	-0,554696666	1,428486318

201750_s_at	ECE1	0,873789652	-0,554696666	1,428486318
214402_s_at	SFI1	0,873789652	-0,554696666	1,428486318
238423_at	SYTL3	4,900499467	3,472563131	1,427936337
221643_s_at	RERE	-0,043925712	-1,471833228	1,427907516
243297_at	-	-0,043925712	-1,471833228	1,427907516
214406_s_at	SLC7A4	-0,043925712	-1,471833228	1,427907516
232272_at	ZNF624	-0,043925712	-1,471833228	1,427907516
240837_at	FNDC7	-0,043925712	-1,471833228	1,427907516
1557569_at	MPDU1	-0,043925712	-1,471833228	1,427907516
242219_at	-	-0,043925712	-1,471833228	1,427907516
204375_at	CLSTN3	0,97663406	-0,45121326	1,427847319
238509_at	CUL1	0,97663406	-0,45121326	1,427847319
204528_s_at	NAP1L1	6,840222093	5,412468476	1,427753617
215776_at	INSRR	-0,384193355	-1,811780055	1,4275867
211237_s_at	FGFR4	-0,384193355	-1,811780055	1,4275867
206124_s_at	LLGL1	-0,384193355	-1,811780055	1,4275867
229409_s_at	-	-0,384193355	-1,811780055	1,4275867
238518_x_at	GLYCTK	-0,384193355	-1,811780055	1,4275867
219629_at	FAM118A	1,960819106	0,533258442	1,427560665
226073_at	TMEM218	3,086998005	1,659532492	1,427465513
214952_at	NCAM1	-0,66934516	-2,09678811	1,42744295
220414_at	CALML5	-0,66934516	-2,09678811	1,42744295
208482_at	SSTR1	-0,66934516	-2,09678811	1,42744295
228618_at	PEAR1	-0,66934516	-2,09678811	1,42744295
216749_at	-	-0,66934516	-2,09678811	1,42744295
221460_at	OR2C1	-0,66934516	-2,09678811	1,42744295
1559616_x_at	ZNF626	-0,66934516	-2,09678811	1,42744295
239865_at	-	-0,66934516	-2,09678811	1,42744295
224299_x_at	FTCD	-0,66934516	-2,09678811	1,42744295
236144_at	CPXM2	-0,66934516	-2,09678811	1,42744295
1561726_s_at	-	-0,66934516	-2,09678811	1,42744295
240194_at	LOC441204	-0,66934516	-2,09678811	1,42744295
216759_at	HRASLS2	-0,66934516	-2,09678811	1,42744295
217325_at	KRT3	-0,66934516	-2,09678811	1,42744295
236874_at	-	-0,66934516	-2,09678811	1,42744295
1552594_at	TMEM190	0,282358733	-1,145057014	1,427415747
221350_at	HOXC8	0,282358733	-1,145057014	1,427415747
220404_at	GPR97	0,282358733	-1,145057014	1,427415747
239821_at	FLJ30064	0,282358733	-1,145057014	1,427415747
243894_at	SLC41A2	0,282358733	-1,145057014	1,427415747
220420_at	LMAN1L	0,282358733	-1,145057014	1,427415747
233894_x_at	EMID2	0,282358733	-1,145057014	1,427415747
217922_at	MAN1A2	3,804904984	2,37755543	1,427349554
234241_at	SCAMP5	1,440265662	0,013190398	1,427075264
1561179_s_at	AMZ1	1,225123479	-0,201789721	1,4269132
228252_at	PIF1	1,225123479	-0,201789721	1,4269132
1569054_at	SLC1A3	-0,924000698	-2,350444759	1,426444062
1554663_a_at	NUMA1	-0,924000698	-2,350444759	1,426444062
218892_at	DCHS1	-0,924000698	-2,350444759	1,426444062
236324_at	MBP	-0,924000698	-2,350444759	1,426444062

223540_at	PVRL4	-0,924000698	-2,350444759	1,426444062
227810_at	ZNF558	2,468978138	1,043469112	1,425509026
1555565_s_at	TAPBP	1,510970097	0,085534992	1,425435105
219865_at	LINC00339	3,209439373	1,784182261	1,425257112
226529_at	TMEM106B	3,798440091	2,373249966	1,425190125
224240_s_at	CCL28	1,625482993	0,20062106	1,424861934
232767_at	CADM1	-0,437449947	-1,862269442	1,424819494
242084_at	LOC339316	-0,437449947	-1,862269442	1,424819494
221175_at	C3orf36	-0,437449947	-1,862269442	1,424819494
233459_at	POLR3E	-0,437449947	-1,862269442	1,424819494
236232_at	-	-0,437449947	-1,862269442	1,424819494
239599_at	-	-0,437449947	-1,862269442	1,424819494
238242_at	-	-0,437449947	-1,862269442	1,424819494
221355_at	CHRNG	-0,437449947	-1,862269442	1,424819494
219870_at	ATF7IP2	-0,437449947	-1,862269442	1,424819494
226568_at	FAM102B	3,228532861	1,804519951	1,424012911
244192_x_at	USP4	1,307201325	-0,116767475	1,4239688
242555_at	C16orf87	0,491906512	-0,931539147	1,423445659
234256_at	SEBOX	0,491906512	-0,931539147	1,423445659
221710_x_at	FAM176B	0,491906512	-0,931539147	1,423445659
215379_x_at	IGLV1-44	0,491906512	-0,931539147	1,423445659
230155_x_at	MSL1	0,491906512	-0,931539147	1,423445659
204432_at	SOX12	0,491906512	-0,931539147	1,423445659
232762_at	KIAA1217	0,001114523	-1,422257026	1,423371549
1567166_at	-	0,001114523	-1,422257026	1,423371549
238867_at	TMEM182	0,001114523	-1,422257026	1,423371549
205334_at	S100A1	0,001114523	-1,422257026	1,423371549
227342_s_at	MYEOV	0,001114523	-1,422257026	1,423371549
212389_at	SBF1	0,001114523	-1,422257026	1,423371549
209683_at	FAM49A	-1,212652659	-2,635549085	1,422896426
217211_at	-	2,621163856	1,199296812	1,421867044
212974_at	DENND3	1,160996507	-0,260526297	1,421522804
222543_at	DERL1	4,878355601	3,457327669	1,421027932
220933_s_at	ZCCHC6	3,171328694	1,750483134	1,420845561
203212_s_at	MTMR2	2,745538988	1,324949741	1,420589247
202916_s_at	FAM20B	2,983826368	1,563256447	1,420569921
228604_at	FAM76A	1,458255986	0,03784737	1,420408616
204255_s_at	VDR	2,452674045	1,032267596	1,420406449
206234_s_at	MMP17	0,319287178	-1,100943374	1,420230552
202322_s_at	GGPS1	4,873110637	3,453397238	1,4197134
200678_x_at	GRN	4,005144537	2,585619776	1,419524761
217877_s_at	GPBP1L1	3,942941653	2,523551252	1,419390401
233810_x_at	-	1,246401134	-0,172194225	1,418595358
1554367_at	CALHM1	-0,49284695	-1,911206569	1,418359619
1557761_s_at	LOC400794	-0,49284695	-1,911206569	1,418359619
237909_at	ADAM6	-0,49284695	-1,911206569	1,418359619
228576_s_at	MXRA8	-0,49284695	-1,911206569	1,418359619
205972_at	SLC38A3	-0,49284695	-1,911206569	1,418359619
237338_at	B3GNT8	-0,49284695	-1,911206569	1,418359619
207150_at	SLC18A3	-0,49284695	-1,911206569	1,418359619

230084_at	SLC30A2	-0,49284695	-1,911206569	1,418359619
1553094_at	FLJ45513 /// TAC4	-0,49284695	-1,911206569	1,418359619
216679_at	DDR1-AS1	-0,49284695	-1,911206569	1,418359619
222927_s_at	CPLX3	-0,49284695	-1,911206569	1,418359619
227347_x_at	HES4	-0,49284695	-1,911206569	1,418359619
205939_at	CYP3A7	0,045517965	-1,372734086	1,41825205
221901_at	KIAA1644	0,045517965	-1,372734086	1,41825205
230691_at	STX1B	0,045517965	-1,372734086	1,41825205
236222_at	C3orf15 /// LOC10	0,045517965	-1,372734086	1,41825205
203071_at	SEMA3B	0,045517965	-1,372734086	1,41825205
219827_at	UCP3	0,045517965	-1,372734086	1,41825205
218825_at	EGFL7	0,045517965	-1,372734086	1,41825205
1552395_at	TSSK3	1,802733148	0,385582632	1,417150516
219893_at	CCDC71	1,094118704	-0,322340048	1,416458752
1553361_x_at	FBXL18	1,5944803	0,178191865	1,416288435
239670_at	WNK2	0,791908897	-0,623254098	1,415162996
240826_at	-	0,791908897	-0,623254098	1,415162996
228391_at	CYP4V2	1,476175815	0,061773582	1,414402233
219382_at	SERTAD3	1,476175815	0,061773582	1,414402233
238039_at	LOC728769	3,185443259	1,771157334	1,414285925
227998_at	S100A16	1,182925501	-0,231045907	1,413971408
203613_s_at	NDUFB6	5,922262115	4,508358398	1,413903717
213276_at	CAMK2B	0,524545436	-0,889222211	1,413767647
232400_at	-	0,524545436	-0,889222211	1,413767647
229717_at	AMIGO3 /// GMPP	0,524545436	-0,889222211	1,413767647
235205_at	OXR1	0,524545436	-0,889222211	1,413767647
203812_at	SLIT3	0,524545436	-0,889222211	1,413767647
231308_at	-	-0,856259172	-2,27001641	1,413757238
216674_at	HES2	-0,856259172	-2,27001641	1,413757238
233242_at	WDR73	-0,856259172	-2,27001641	1,413757238
234708_at	SMUG1	-0,856259172	-2,27001641	1,413757238
211838_x_at	PCDHA5	-0,856259172	-2,27001641	1,413757238
216133_at	YME1L1	-0,856259172	-2,27001641	1,413757238
1564525_at	GSN	0,08619576	-1,32740143	1,41359719
239642_at	-	0,08619576	-1,32740143	1,41359719
216412_x_at	CKAP2 /// IGLC1	0,08619576	-1,32740143	1,41359719
240727_s_at	-	0,08619576	-1,32740143	1,41359719
226512_at	ZMYM2	1,402495885	-0,011001236	1,41349712
218290_at	PLEKHJ1	3,434684504	2,021531611	1,413152894
230049_at	TAF6L	0,355751005	-1,057301851	1,413052856
234746_at	-	0,355751005	-1,057301851	1,413052856
217054_at	-	0,355751005	-1,057301851	1,413052856
212623_at	TMEM41B	4,155757614	2,743433212	1,412324402
204708_at	MAPK4	-0,730013898	-2,142286822	1,412272923
1563120_at	LOC100129029	-0,730013898	-2,142286822	1,412272923
210348_at	38231	-0,730013898	-2,142286822	1,412272923
222750_s_at	SRD5A3	2,590269697	1,178100917	1,41216878
218029_at	FAM65A	1,267146969	-0,144765583	1,411912552
226952_at	EAF1	3,505636113	2,093850742	1,411785371
203065_s_at	CAV1	4,538810963	3,127281098	1,411529865

217043_s_at	MFN1	3,055618382	1,644429915	1,411188467
227924_at	INO80D	0,926325262	-0,484558493	1,410883756
227698_s_at	RAB40C	2,267141818	0,85655369	1,410588129
225643_at	MAPK1IP1L	4,007783021	2,597466271	1,41031675
227381_at	CERCAM	0,127732831	-1,282520722	1,410253553
203623_at	PLXNA3	0,127732831	-1,282520722	1,410253553
1566231_at	-	0,127732831	-1,282520722	1,410253553
207028_at	MYCNOS	0,127732831	-1,282520722	1,410253553
215502_at	-	0,127732831	-1,282520722	1,410253553
240849_at	-	0,127732831	-1,282520722	1,410253553
204511_at	FARP2	0,820160788	-0,589001171	1,409161959
228641_at	CARD8	3,389999385	1,981728801	1,408270584
201932_at	LRRC41	3,581038515	2,173028091	1,408010423
201354_s_at	BAZ2A	2,33082239	0,923203038	1,407619351
201207_at	TNFAIP1	2,705643918	1,298349073	1,407294844
227545_at	BARD1	3,382641505	1,975797592	1,406843913
223421_at	CYHR1	2,007220798	0,600385088	1,40683571
235496_at	HRCT1	0,39105295	-1,015398016	1,406450967
219500_at	CLCF1	0,39105295	-1,015398016	1,406450967
200654_at	P4HB	5,608763656	4,202330793	1,406432863
207813_s_at	FDXR	1,561816652	0,155747281	1,406069372
201685_s_at	TOX4	3,007119335	1,601076277	1,406043059
208811_s_at	DNAJB6 /// TMEM	4,06801876	2,662083808	1,405934952
219499_at	SEC61A2	1,671397663	0,265475485	1,405922178
1567358_at	NAV2	-1,289805289	-2,695640852	1,405835563
226542_at	-	1,20396005	-0,201789721	1,405749771
217700_at	CNPY4	0,556428218	-0,84928999	1,405718208
1556718_s_at	-	0,556428218	-0,84928999	1,405718208
239612_at	LOC100240734	-0,551284523	-1,956681069	1,405396547
238125_at	ADAMTS16	-0,551284523	-1,956681069	1,405396547
224139_at	SHANK2-AS3	-0,551284523	-1,956681069	1,405396547
241149_at	-	-0,551284523	-1,956681069	1,405396547
211916_s_at	MYO1A	-0,551284523	-1,956681069	1,405396547
223653_x_at	CELF4	-0,551284523	-1,956681069	1,405396547
224134_at	MGC10814	-0,551284523	-1,956681069	1,405396547
220942_x_at	FAM162A	7,032223777	5,627045388	1,405178389
210255_at	RAD51B	0,707657549	-0,697182596	1,404840145
224730_at	DCAF7	3,386206804	1,981728801	1,404478003
235113_at	LRR1	4,631436665	3,227213858	1,404222808
222412_s_at	SSR3	3,951538845	2,54746241	1,404076435
1563606_a_at	LOC286359	-1,06282519	-2,466349414	1,403524224
1553422_s_at	RBFOX1	-1,06282519	-2,466349414	1,403524224
216024_at	DNM2	-1,06282519	-2,466349414	1,403524224
207106_s_at	LTK	-1,06282519	-2,466349414	1,403524224
230749_s_at	CAMK2D	-1,06282519	-2,466349414	1,403524224
233018_at	TMEM134	-1,06282519	-2,466349414	1,403524224
207123_s_at	MATN4	0,95222556	-0,45121326	1,40343882
212565_at	STK38L	2,053550345	0,65030602	1,403244325
201973_s_at	CCZ1 /// CCZ1B	6,585606305	5,182769407	1,402836898
1554613_a_at	KIAA0226	0,167727503	-1,234835326	1,40256283

214955_at	TMPRSS6	1,440265662	0,03784737	1,402418292
1569868_s_at	EME2	1,047341799	-0,354781582	1,40212338
217789_at	SNX6	3,983349065	2,582046772	1,401302293
230966_at	IL4I1	1,364555912	-0,036543687	1,401099599
209150_s_at	TM9SF1	2,860630824	1,459545271	1,401085553
219628_at	ZMAT3	2,908690075	1,508107415	1,40058266
213633_at	SH3BP1	1,983901861	0,583607503	1,400294358
237591_at	LINC00173	2,229377375	0,829309537	1,400067838
226133_s_at	TBC1D10A	2,752615529	1,352622437	1,399993091
242957_at	VWCE	0,58788177	-0,811769547	1,399651317
209890_at	TSPAN5	4,37922506	2,979585083	1,399639977
225758_s_at	TUBGCP6	1,139057614	-0,260526297	1,399583911
238550_at	RUFY2	-1,137208348	-2,536339282	1,399130933
1553482_at	C15orf32	-1,137208348	-2,536339282	1,399130933
1560021_at	-	-1,137208348	-2,536339282	1,399130933
217333_at	-	-1,137208348	-2,536339282	1,399130933
215561_s_at	IL1R1	-1,137208348	-2,536339282	1,399130933
1557550_at	LOC148145	-1,137208348	-2,536339282	1,399130933
239454_at	SCARF2	-1,137208348	-2,536339282	1,399130933
239081_at	-	2,41945542	1,020514937	1,398940483
213752_at	KAZN	0,20740896	-1,19085694	1,3982659
1553594_a_at	INSL3	0,20740896	-1,19085694	1,3982659
213407_at	PHLPP2	2,657308324	1,259081178	1,398227146
237437_s_at	ADARB2	0,425036312	-0,972893339	1,397929651
209660_at	TTR	0,425036312	-0,972893339	1,397929651
233111_at	PTCSC1	0,425036312	-0,972893339	1,397929651
209921_at	SLC7A11	2,824160383	1,426280426	1,397879957
242342_at	GUCY1A2	-0,994186487	-2,391863529	1,397677042
236893_at	HOXB-AS3	-0,994186487	-2,391863529	1,397677042
205713_s_at	COMP	-0,994186487	-2,391863529	1,397677042
228163_at	ST6GALNAC4	-0,994186487	-2,391863529	1,397677042
207381_at	ALOX12B	-0,994186487	-2,391863529	1,397677042
205293_x_at	BAIAP2	-0,994186487	-2,391863529	1,397677042
242753_x_at	AP1AR	2,063971513	0,666486137	1,397485376
221769_at	SPSB3	1,225123479	-0,172194225	1,397317704
238613_at	ZAK	1,225123479	-0,172194225	1,397317704
224111_x_at	KLF16	1,225123479	-0,172194225	1,397317704
216822_x_at	-	0,735999505	-0,661178575	1,39717808
207765_s_at	FAM214B	0,735999505	-0,661178575	1,39717808
224243_at	APOA5	0,735999505	-0,661178575	1,39717808
218588_s_at	FAM114A2	2,685337919	1,28853674	1,396801179
227066_at	MOB3C	1,458255986	0,061773582	1,396482404
241962_at	-	-0,609040214	-2,005029581	1,395989367
1559550_s_at	-	-0,609040214	-2,005029581	1,395989367
216398_at	GYPB	-0,609040214	-2,005029581	1,395989367
1557943_at	CNP	-0,609040214	-2,005029581	1,395989367
221401_at	CACNG5	-0,609040214	-2,005029581	1,395989367
207214_at	SPINK4	-0,609040214	-2,005029581	1,395989367
207758_at	-	-0,609040214	-2,005029581	1,395989367
1569465_at	FAM160A1	-0,609040214	-2,005029581	1,395989367

224300_x_at	FTCD	-0,609040214	-2,005029581	1,395989367
210350_x_at	ING1	2,392346173	0,996453274	1,3958929
223376_s_at	BRI3	4,405838675	3,010094234	1,395744441
226665_at	AHSA2	2,427650693	1,032267596	1,395383097
229618_at	SNX16	2,507817181	1,112548615	1,395268566
213657_s_at	-	3,018305638	1,623485524	1,394820114
209467_s_at	MKNK1	3,018305638	1,623485524	1,394820114
1553403_at	RLN3	-0,791294935	-2,186086603	1,394791668
204894_s_at	AOC3	-0,791294935	-2,186086603	1,394791668
237003_at	BEST3	-0,791294935	-2,186086603	1,394791668
222226_at	SAA3P	-0,791294935	-2,186086603	1,394791668
244242_at	-	-0,791294935	-2,186086603	1,394791668
214198_s_at	DGCR2	2,33082239	0,936232181	1,394590208
55583_at	DOCK6	0,835052349	-0,55913998	1,394192328
45749_at	FAM65A	3,255788498	1,861704656	1,394083841
48612_at	N4BP1	3,473998897	2,080163963	1,393834933
225251_at	RAB24	2,642918178	1,249194639	1,39372354
224708_at	KIAA2013	3,726612406	2,332940758	1,393671648
228622_s_at	DNAJC4	2,460890032	1,067365565	1,393524467
208841_s_at	G3BP2	5,036276071	3,643334026	1,392942045
222431_at	SPIN1	5,255368165	3,862709873	1,392658292
225302_at	TMX3	3,442765386	2,050286491	1,392478895
227422_at	STRN	3,086998005	1,694547353	1,392450651
206180_x_at	ZNF747	2,042564225	0,65030602	1,392258205
231130_at	FKBP7	0,873789652	-0,518232988	1,39202264
234254_at	-	0,873789652	-0,518232988	1,39202264
219432_at	EVC	2,791258167	1,399283506	1,391974661
233632_s_at	XRN1	3,618399139	2,226451905	1,391947234
223580_at	SPSB2	2,339581693	0,947673886	1,391907807
223283_s_at	TSHZ1	3,029186714	1,637482551	1,391704164
205770_at	GSR	3,029186714	1,637482551	1,391704164
218107_at	WDR26	5,657188578	4,265652291	1,391536287
208166_at	MMP16	-1,212652659	-2,603471267	1,390818607
216572_at	FOXL1	-1,212652659	-2,603471267	1,390818607
229346_at	NES	-1,212652659	-2,603471267	1,390818607
221479_s_at	BNIP3L	4,93324721	3,542765782	1,390481427
230616_at	LAMB2P1	0,458905032	-0,931539147	1,390444178
1562002_at	MYOZ3	0,458905032	-0,931539147	1,390444178
224434_s_at	WDR83	1,65554524	0,265475485	1,390069755
235502_at	MIR3661 /// PPP2(	0,244681185	-1,145057014	1,3897382
208354_s_at	SLC12A3	0,244681185	-1,145057014	1,3897382
232091_s_at	ZDHHC24	0,244681185	-1,145057014	1,3897382
214164_x_at	CA12	0,244681185	-1,145057014	1,3897382
243409_at	FOXL1	0,244681185	-1,145057014	1,3897382
234669_x_at	C11orf30	0,244681185	-1,145057014	1,3897382
1567035_at	-	0,244681185	-1,145057014	1,3897382
227795_at	NDUFV1	0,244681185	-1,145057014	1,3897382
223967_at	ANGPTL6	0,618277321	-0,771340337	1,389617658
226222_at	KIAA1432	2,085817135	0,697221375	1,38859576
205474_at	CRLF3	5,794713976	4,40626232	1,388451655

240487_at	-	-0,924000698	-2,312054429	1,388053731
241239_at	EPN2-IT1	-0,924000698	-2,312054429	1,388053731
238153_at	PDE6B	-0,924000698	-2,312054429	1,388053731
207897_at	CRHR2	-0,924000698	-2,312054429	1,388053731
241952_at	SLC16A11	-0,924000698	-2,312054429	1,388053731
205077_s_at	PIGF	5,095686705	3,707798923	1,387887782
240449_at	ZNF341	0,764143511	-0,623254098	1,387397609
216317_x_at	RHCE	0,764143511	-0,623254098	1,387397609
1568718_at	SLC22A23	0,764143511	-0,623254098	1,387397609
237607_at	-	0,764143511	-0,623254098	1,387397609
243766_s_at	TEAD2	0,764143511	-0,623254098	1,387397609
219223_at	CACFD1	0,764143511	-0,623254098	1,387397609
215789_s_at	AJAP1	-0,180990326	-1,568385657	1,387395331
216289_at	GPR144	-0,180990326	-1,568385657	1,387395331
1569234_at	-	-0,180990326	-1,568385657	1,387395331
211223_at	PROP1	-0,180990326	-1,568385657	1,387395331
215799_at	-	-0,180990326	-1,568385657	1,387395331
209359_x_at	LOC100506403 ///	-0,180990326	-1,568385657	1,387395331
205320_at	APC2	-0,180990326	-1,568385657	1,387395331
200784_s_at	LRP1	1,000459215	-0,386896102	1,387355317
208219_at	ACVR1B	-0,230432956	-1,616924751	1,386491795
232732_at	LOC100652999 ///	-0,230432956	-1,616924751	1,386491795
244034_at	-	-0,230432956	-1,616924751	1,386491795
211624_s_at	DRD2	-0,230432956	-1,616924751	1,386491795
1559443_s_at	IL21R-AS1	-0,230432956	-1,616924751	1,386491795
234120_at	-	-0,230432956	-1,616924751	1,386491795
222982_x_at	SLC38A2	6,230927896	4,844933938	1,385993958
223110_at	KIAA1429	3,7799838	2,394276989	1,385706811
240473_at	-	-0,280273599	-1,665791776	1,385518177
227975_at	GPRIN1	-0,280273599	-1,665791776	1,385518177
228721_at	KRBOX1	-0,280273599	-1,665791776	1,385518177
243849_at	TMEM37	-0,280273599	-1,665791776	1,385518177
212307_s_at	OGT	3,754428453	2,36899666	1,385431793
218299_at	C11orf24	1,671397663	0,286279868	1,385117795
201723_s_at	GALNT1	3,668220666	2,283169614	1,385051052
1555020_a_at	ARHGAP20	-0,135065865	-1,520022123	1,384956258
213059_at	CREB3L1	-0,135065865	-1,520022123	1,384956258
202312_s_at	COL1A1	-0,135065865	-1,520022123	1,384956258
205155_s_at	SPTBN2	-0,135065865	-1,520022123	1,384956258
208609_s_at	TNXB	-0,135065865	-1,520022123	1,384956258
220628_s_at	SDK2	-0,135065865	-1,520022123	1,384956258
1569073_x_at	SMARCA4	-0,135065865	-1,520022123	1,384956258
238590_x_at	TMEM107	4,306090192	2,921154239	1,384935953
213560_at	GADD45B	1,182925501	-0,201789721	1,384715222
235484_at	PTAR1	1,182925501	-0,201789721	1,384715222
230647_at	TMEM53	0,282358733	-1,100943374	1,383302107
205271_s_at	CDK20	0,282358733	-1,100943374	1,383302107
228188_at	FOSL2	0,282358733	-1,100943374	1,383302107
224340_at	-	-0,332405896	-1,715576125	1,383170229
231704_at	CYP3A4	-0,332405896	-1,715576125	1,383170229

204625_s_at	ITGB3	-0,332405896	-1,715576125	1,383170229
228892_at	SH3RF2	-0,332405896	-1,715576125	1,383170229
241525_at	LOC200772	-0,332405896	-1,715576125	1,383170229
231659_at	ST3GAL3	-0,332405896	-1,715576125	1,383170229
224198_at	CELA1	-0,332405896	-1,715576125	1,383170229
1566760_at	FLJ34208	-0,332405896	-1,715576125	1,383170229
1565659_at	FUT6	-0,332405896	-1,715576125	1,383170229
236225_at	GGT6	-0,332405896	-1,715576125	1,383170229
228418_at	EXOC5	2,914752575	1,531877749	1,382874826
206913_at	BAAT	-0,088974936	-1,471833228	1,382858292
211003_x_at	TGM2	-0,088974936	-1,471833228	1,382858292
233966_at	-	-0,088974936	-1,471833228	1,382858292
214208_at	KLHL35	-0,088974936	-1,471833228	1,382858292
238864_at	VWA3A	-0,088974936	-1,471833228	1,382858292
216584_at	-	-0,088974936	-1,471833228	1,382858292
202213_s_at	CUL4B	2,725848647	1,343200523	1,382648124
206471_s_at	PLXNC1	0,648195588	-0,734355396	1,382550984
242944_at	FAM83A	0,648195588	-0,734355396	1,382550984
1570384_at	TAS2R19	-1,369772723	-2,752097204	1,382324481
1555221_at	-	-1,369772723	-2,752097204	1,382324481
225125_at	MMGT1	3,889146356	2,506994817	1,382151539
1554153_a_at	PHF21A	2,063971513	0,681874787	1,382096726
211985_s_at	CALM1 /// CALM2	3,892081305	2,5107204	1,381360905
218037_at	FAM134A	2,537731447	1,156599976	1,381131471
209532_at	PLAA	0,791908897	-0,589001171	1,380910068
219722_s_at	GDPD3	0,791908897	-0,589001171	1,380910068
209873_s_at	PKP3	0,791908897	-0,589001171	1,380910068
212189_s_at	COG4	2,966903803	1,586121802	1,380782001
222888_at	CCNJ	-0,66934516	-2,049747196	1,380402037
1557826_at	LOC338817	-0,66934516	-2,049747196	1,380402037
219152_at	PODXL2	-0,66934516	-2,049747196	1,380402037
237308_at	-	-0,66934516	-2,049747196	1,380402037
234774_at	R3HDML	-0,66934516	-2,049747196	1,380402037
239401_at	-	-0,66934516	-2,049747196	1,380402037
238703_at	FAM207A	-0,66934516	-2,049747196	1,380402037
206784_at	AQP8	-0,384193355	-1,763664074	1,379470719
230774_at	PTGR2	-0,384193355	-1,763664074	1,379470719
229825_at	PLK3	-0,384193355	-1,763664074	1,379470719
211432_s_at	TYRO3	-0,384193355	-1,763664074	1,379470719
228187_at	-	-0,384193355	-1,763664074	1,379470719
224194_at	FCRL2	-0,384193355	-1,763664074	1,379470719
234568_at	SCAMP5	-0,384193355	-1,763664074	1,379470719
1554222_at	MGC45922	-0,384193355	-1,763664074	1,379470719
232977_x_at	MYH14	-0,384193355	-1,763664074	1,379470719
201791_s_at	DHCR7	3,209439373	1,830129603	1,37930977
201975_at	CLIP1	2,778527892	1,399283506	1,379244386
214455_at	HIST1H2BC /// HIS	1,024284941	-0,354781582	1,379066523
235607_at	-	1,024284941	-0,354781582	1,379066523
205018_s_at	MBNL2	1,440265662	0,061773582	1,37849208
224008_s_at	KCNK7	-0,043925712	-1,422257026	1,378331314

244713_at	-	-0,043925712	-1,422257026	1,378331314
207355_at	SLC1A7	-0,043925712	-1,422257026	1,378331314
241181_x_at	-	-0,043925712	-1,422257026	1,378331314
39249_at	AQP3	1,663963286	0,285665482	1,378297804
1553858_at	ZBTB3	1,802733148	0,424693306	1,378039842
217940_s_at	CARKD	4,546768622	3,169167796	1,377600826
212474_at	AVL9	3,584942056	2,207440269	1,377501787
220181_x_at	SLC30A5	1,910557155	0,533258442	1,377298714
225933_at	CCDC137	2,21987062	0,842599219	1,377271401
219421_at	TTC33	2,21987062	0,842599219	1,377271401
217168_s_at	HERPUD1	5,697127066	4,320423334	1,376703732
227239_at	FAM126A	3,978219531	2,601822502	1,376397029
221572_s_at	SLC26A6	1,20396005	-0,172194225	1,376154275
213555_at	RWDD2A	1,20396005	-0,172194225	1,376154275
1558540_s_at	SLC2A11	1,364555912	-0,011001236	1,375557148
203494_s_at	CEP57	4,672908132	3,297572471	1,375335661
218165_at	MEAF6	4,607153971	3,231908248	1,375245723
223370_at	PLEKHA3	2,961244348	1,586121802	1,375122546
220975_s_at	C1QTNF1	0,677745787	-0,697182596	1,374928383
1561651_s_at	TAL1	0,677745787	-0,697182596	1,374928383
226444_at	-	0,677745787	-0,697182596	1,374928383
211786_at	TNFRSF9	0,820160788	-0,554696666	1,374857454
228181_at	SLC30A1	1,701542258	0,326777209	1,374765048
201991_s_at	KIF5B	5,329807155	3,955384072	1,374423083
211565_at	SH3GL3	-0,437449947	-1,811780055	1,374330107
214269_at	MFSD7	-0,437449947	-1,811780055	1,374330107
211451_s_at	KCNJ4	-0,437449947	-1,811780055	1,374330107
233514_x_at	TEX11	-0,437449947	-1,811780055	1,374330107
201982_s_at	PAPPA	-0,437449947	-1,811780055	1,374330107
221426_s_at	OR3A3	-0,437449947	-1,811780055	1,374330107
206699_x_at	NPAS1	-0,437449947	-1,811780055	1,374330107
232381_s_at	DNAH5	-0,437449947	-1,811780055	1,374330107
222502_s_at	UFM1	2,321828846	0,947673886	1,37415496
222864_s_at	ZNF219	0,001114523	-1,372734086	1,373848608
231649_at	-	0,001114523	-1,372734086	1,373848608
222144_at	KIF17	0,001114523	-1,372734086	1,373848608
240104_at	-	0,001114523	-1,372734086	1,373848608
239158_at	-	0,001114523	-1,372734086	1,373848608
230273_at	C6orf165	0,001114523	-1,372734086	1,373848608
241464_s_at	-	-0,856259172	-2,229233437	1,372974265
237604_at	-	-0,856259172	-2,229233437	1,372974265
230458_at	SLC45A1	-0,856259172	-2,229233437	1,372974265
231196_x_at	LINC00202	-0,856259172	-2,229233437	1,372974265
205388_at	TNNC2	0,045517965	-1,32740143	1,372919395
213863_s_at	OAZ3	0,045517965	-1,32740143	1,372919395
217235_x_at	IGLL5	0,045517965	-1,32740143	1,372919395
244284_at	-	0,045517965	-1,32740143	1,372919395
210924_at	OLFM1	0,045517965	-1,32740143	1,372919395
204164_at	SIPA1	1,458255986	0,085534992	1,372720994
227385_at	PPAPDC2	2,295108393	0,923203038	1,371905355

202944_at	NAGA	2,392346173	1,020514937	1,371831237
224849_at	TTC17	4,237642543	2,86626857	1,371373973
210788_s_at	DHRS7	4,498569607	3,127281098	1,371288508
201604_s_at	PPP1R12A	3,807915835	2,436714502	1,371201333
235681_at	-	0,355751005	-1,015398016	1,371149021
214145_s_at	SPTB	0,355751005	-1,015398016	1,371149021
234338_s_at	ZBTB47	0,355751005	-1,015398016	1,371149021
208031_s_at	RFX2	0,355751005	-1,015398016	1,371149021
202566_s_at	SVIL	1,307201325	-0,063377083	1,370578408
227738_s_at	ARMC5	0,95222556	-0,418248858	1,370474418
226480_at	-	4,405838675	3,035366122	1,370472553
222605_at	RCOR3	3,147261986	1,777135134	1,370126852
203248_at	ZNF24	1,139057614	-0,231045907	1,370103521
200858_s_at	RPS8 /// SNORD38	7,779196605	6,409176395	1,37002021
223326_s_at	AGSK1 /// LOC642	1,225123479	-0,144765583	1,369889062
219150_s_at	ADAP1	1,047341799	-0,322340048	1,369681846
208789_at	PTRF	1,047341799	-0,322340048	1,369681846
221803_s_at	NRBF2	3,729417893	2,359892448	1,369525445
233057_at	HSPB8	-0,49284695	-1,862269442	1,369422491
205075_at	SERPINF2	-0,49284695	-1,862269442	1,369422491
243151_at	LOC100506282	-0,49284695	-1,862269442	1,369422491
244554_at	-	-0,49284695	-1,862269442	1,369422491
219874_at	SLC12A8	-0,49284695	-1,862269442	1,369422491
221737_at	GNA12	1,716003175	0,346964736	1,369038439
212122_at	RHOQ	0,08619576	-1,282520722	1,368716482
230476_at	-	0,08619576	-1,282520722	1,368716482
213914_s_at	SPTBN1	0,08619576	-1,282520722	1,368716482
213723_s_at	IDUA	0,08619576	-1,282520722	1,368716482
244721_at	TP53INP1	0,08619576	-1,282520722	1,368716482
238038_at	-	0,08619576	-1,282520722	1,368716482
220412_x_at	KCNK7	0,08619576	-1,282520722	1,368716482
1554074_s_at	SLFNL1	0,08619576	-1,282520722	1,368716482
1558619_at	SNORA43	0,08619576	-1,282520722	1,368716482
233895_at	ANKRD24	0,08619576	-1,282520722	1,368716482
230189_x_at	NELF	0,08619576	-1,282520722	1,368716482
212738_at	ARHGAP19	3,867546929	2,498884538	1,368662391
32699_s_at	PVR	0,466558398	-0,901844472	1,368402869
214042_s_at	RPL22	6,884320934	5,516048191	1,368272743
241233_x_at	ANKRD20A11P	0,556428218	-0,811769547	1,368197765
229686_at	P2RY8	3,185443259	1,817403386	1,368039873
219648_at	MREG	4,439413216	3,071469991	1,367943225
240566_at	-	-0,730013898	-2,09678811	1,366774212
237763_at	-	-0,730013898	-2,09678811	1,366774212
231480_at	SLC6A19	-0,730013898	-2,09678811	1,366774212
203483_at	SEMA4G	-0,730013898	-2,09678811	1,366774212
229162_s_at	ABTB1	-0,730013898	-2,09678811	1,366774212
220863_at	MIP	-0,730013898	-2,09678811	1,366774212
223757_at	DIO3OS	-0,730013898	-2,09678811	1,366774212
238849_at	ACY1	-0,730013898	-2,09678811	1,366774212
1554045_at	ZNF24	-0,730013898	-2,09678811	1,366774212

1563805_a_at	FAM83C	-0,730013898	-2,09678811	1,366774212
207001_x_at	TSC22D3	2,866549889	1,500059916	1,366489972
212591_at	ARID4B /// RBM34	5,301285773	3,934892246	1,366393526
201254_x_at	RPS6	8,529302121	7,16343813	1,36586399
201355_s_at	BAZ2A	0,847529938	-0,518232988	1,365762926
227605_at	AIMP1	3,370643409	2,004917973	1,365725437
228732_at	GUCY1A2	-1,06282519	-2,4284334	1,36560821
1552338_at	GSC	-1,06282519	-2,4284334	1,36560821
1554268_at	MORN1	-1,06282519	-2,4284334	1,36560821
238248_at	-	-1,06282519	-2,4284334	1,36560821
1562589_at	-	-1,06282519	-2,4284334	1,36560821
214978_s_at	PPFIA4	-1,06282519	-2,4284334	1,36560821
225184_at	ARID1B	1,948628481	0,583607503	1,365020978
203728_at	BAK1	1,948628481	0,583607503	1,365020978
203115_at	FECH	1,730929079	0,366031214	1,364897865
225574_at	RWDD4	3,942941653	2,578283758	1,364657895
210082_at	ABCA4	1,402495885	0,03784737	1,364648514
217256_x_at	-	6,729536176	5,36517843	1,364357747
202427_s_at	BRP44	5,532010443	4,167778161	1,364232282
209711_at	SLC35D1	4,09423887	2,730083062	1,364155808
241459_at	-	-1,137208348	-2,501359113	1,364150765
1558607_at	PAPPA	-1,137208348	-2,501359113	1,364150765
241570_at	-	-1,137208348	-2,501359113	1,364150765
241665_x_at	-	-1,137208348	-2,501359113	1,364150765
236643_s_at	-	-1,137208348	-2,501359113	1,364150765
207752_x_at	PRB1	1,246401134	-0,116767475	1,363168608
57163_at	ELOVL1	4,385427564	3,022297406	1,363130158
1555896_a_at	ADAM15	2,149438457	0,786391549	1,363046908
212266_s_at	SRSF5	5,840112192	4,477070446	1,363041746
207982_at	HIST1H1T	0,127732831	-1,234835326	1,362568157
1555681_at	-	0,127732831	-1,234835326	1,362568157
220573_at	KLK14	0,127732831	-1,234835326	1,362568157
220819_at	FRMD1	0,127732831	-1,234835326	1,362568157
220427_at	OBSCN	1,071336699	-0,290694975	1,362031674
212043_at	TGOLN2	5,985129809	4,623451961	1,361677848
227687_at	HYLS1	3,86465425	2,503049671	1,36160458
223325_at	TXNDC11	2,484824532	1,123905343	1,360919189
1558345_a_at	LOC439911	1,493647653	0,133104519	1,360543134
211162_x_at	SCD	1,493647653	0,133104519	1,360543134
213044_at	ROCK1	4,988355462	3,628062877	1,360292584
221826_at	ANGEL2	2,62869474	1,268569037	1,360125702
227354_at	PAG1	3,804904984	2,444951949	1,359953035
219739_at	RNF186	-0,551284523	-1,911206569	1,359922047
231144_at	SMARCD3	-0,551284523	-1,911206569	1,359922047
208335_s_at	DARC	-0,551284523	-1,911206569	1,359922047
222285_at	IGHD	-0,551284523	-1,911206569	1,359922047
228411_at	PARD3B	-0,551284523	-1,911206569	1,359922047
216222_s_at	MYO10	-0,551284523	-1,911206569	1,359922047
215695_s_at	GYG2	-0,551284523	-1,911206569	1,359922047
222828_at	IL20RA	-0,551284523	-1,911206569	1,359922047

235842_at	-	-0,551284523	-1,911206569	1,359922047
206063_x_at	PPIL2	1,745502609	0,385582632	1,359919977
227853_at	-	2,229377375	0,869824768	1,359552607
205311_at	DDC	0,735999505	-0,623254098	1,359253604
236463_at	ADAD2	0,735999505	-0,623254098	1,359253604
242327_x_at	-	0,735999505	-0,623254098	1,359253604
219991_at	SLC2A9	0,58788177	-0,771340337	1,359222107
48659_at	MIIP	3,87362669	2,514644121	1,358982569
225925_s_at	USP48	4,888321596	3,52937772	1,358943876
226901_at	C17orf58	4,790575561	3,431831208	1,358744353
232395_x_at	AGBL3	0,167727503	-1,19085694	1,358584443
233267_at	-	0,167727503	-1,19085694	1,358584443
229451_at	GALNT9	0,167727503	-1,19085694	1,358584443
235275_at	BMP8B	0,167727503	-1,19085694	1,358584443
210952_at	AP4S1	0,873789652	-0,484558493	1,358348145
201916_s_at	SEC63	4,193391347	2,835336991	1,358054356
243036_at	CCDC30	-1,212652659	-2,570522741	1,357870082
1563559_at	FMN1 /// LOC1006	-1,212652659	-2,570522741	1,357870082
239832_at	-	-1,212652659	-2,570522741	1,357870082
200814_at	PSME1	6,69739962	5,33979754	1,35760208
220104_at	ZC3HAV1	1,267146969	-0,089635713	1,356782682
222347_at	-	0,425036312	-0,931539147	1,356575459
227411_at	WTIP	0,425036312	-0,931539147	1,356575459
1556176_at	TAF8	0,425036312	-0,931539147	1,356575459
230946_at	FMN2	0,425036312	-0,931539147	1,356575459
1554641_a_at	TET3	0,425036312	-0,931539147	1,356575459
202239_at	PARP4	4,819724179	3,463284238	1,35643994
224871_at	TPRG1L	3,592169709	2,235857731	1,356311978
224071_at	IL20	-0,994186487	-2,350444759	1,356258272
1559534_at	-	-0,994186487	-2,350444759	1,356258272
239087_at	ANKS4B	-0,994186487	-2,350444759	1,356258272
229063_s_at	CCDC107	1,816938997	0,461385738	1,35555326
229958_at	CLN8	1,000459215	-0,354781582	1,355240796
229789_at	TIGD3	1,000459215	-0,354781582	1,355240796
1553359_at	FBXL18	1,000459215	-0,354781582	1,355240796
229741_at	MAVS	1,510970097	0,155747281	1,355222817
33778_at	TBC1D22A	3,988830662	2,633910481	1,354920181
214335_at	RPL18	1,094118704	-0,260526297	1,354645001
201093_x_at	SDHA	5,677838297	4,323231013	1,354607284
218477_at	TMEM14A	6,289725135	4,935841172	1,353883963
202271_at	FBXO28	3,353217129	1,999354538	1,353862591
229960_at	MAP3K6	-1,369772723	-2,723114724	1,353342002
210491_at	-	-1,369772723	-2,723114724	1,353342002
225851_at	CHURC1-FNTB ///	2,752615529	1,399283506	1,353332023
212443_at	NBEAL2	2,209870035	0,85655369	1,353316345
217761_at	ADI1	5,437566312	4,084338814	1,353227498
202328_s_at	PKD1	0,764143511	-0,589001171	1,353144682
223013_at	TBL1XR1	5,634172732	4,281354736	1,352817997
226092_at	MPP5	1,936380191	0,583607503	1,352772688
243912_x_at	APOBEC3F	0,618277321	-0,734355396	1,352632717

243825_at	BCL6B	0,618277321	-0,734355396	1,352632717
203756_at	ARHGEF17	0,618277321	-0,734355396	1,352632717
221211_s_at	C21orf7	0,20740896	-1,145057014	1,352465974
230798_at	-	0,20740896	-1,145057014	1,352465974
207252_at	INE1	0,20740896	-1,145057014	1,352465974
221947_at	-	0,20740896	-1,145057014	1,352465974
217771_at	GOLM1	2,989672611	1,637482551	1,35219006
230446_at	LOC100506130	2,41945542	1,067365565	1,352089855
213858_at	ZNF250	1,88491011	0,533258442	1,351651669
208899_x_at	ATP6V1D	4,138272593	2,786807892	1,351464701
238001_at	KCTD6	1,364555912	0,013190398	1,351365514
221956_at	LRCH4	0,900065578	-0,45121326	1,351278838
224002_s_at	FKBP7	1,287793882	-0,063377083	1,351170965
1553955_at	PPP1R21	4,817982599	3,466845432	1,351137168
238993_at	MATR3	-0,791294935	-2,142286822	1,350991886
1553288_a_at	NYAP1	-0,791294935	-2,142286822	1,350991886
230159_at	C1orf115	-0,791294935	-2,142286822	1,350991886
219845_at	BARX1	-0,791294935	-2,142286822	1,350991886
229458_s_at	GALK1	-0,791294935	-2,142286822	1,350991886
225448_at	NAPG	3,422378845	2,071691486	1,350687359
222858_s_at	DAPP1	3,562907192	2,212304392	1,3506028
207238_s_at	PTPRC	4,797158735	3,447692163	1,349466572
1553728_at	LRRC43	2,031158928	0,681874787	1,349284141
223152_at	PPP1R12C	1,65554524	0,306454867	1,349090373
224909_s_at	PREX1	4,102051194	2,753767314	1,34828388
234316_x_at	KLK12	0,458905032	-0,889222211	1,348127242
234882_at	-	0,458905032	-0,889222211	1,348127242
218930_s_at	TMEM106B	2,719381633	1,371634653	1,34774698
214284_s_at	FGF18	-0,609040214	-1,956681069	1,347640856
211897_s_at	CRHR1	-0,609040214	-1,956681069	1,347640856
240756_at	-	-0,609040214	-1,956681069	1,347640856
1564679_at	ASB15	-0,609040214	-1,956681069	1,347640856
235544_x_at	FAM171A2	-0,609040214	-1,956681069	1,347640856
1560784_x_at	-	-0,609040214	-1,956681069	1,347640856
225725_at	ZMAT3	3,209439373	1,861868165	1,347571208
202762_at	ROCK2	4,337778276	2,990421269	1,347357007
225609_at	GSR	3,639446351	2,292099323	1,347347028
1556126_s_at	-	1,024284941	-0,322340048	1,346624989
223348_x_at	MUM1	1,843966654	0,497424714	1,34654194
227396_at	LOC100287223 ///	3,642593015	2,296310714	1,346282301
226334_s_at	AHSA2	3,723597116	2,37755543	1,346041686
1569128_at	C3orf38	-0,924000698	-2,27001641	1,346015712
240796_at	-	-0,924000698	-2,27001641	1,346015712
209978_s_at	LPA /// PLG	-0,924000698	-2,27001641	1,346015712
219533_at	CDKN1C	-0,924000698	-2,27001641	1,346015712
227285_at	C1orf51	-0,924000698	-2,27001641	1,346015712
222242_s_at	KLK5	-0,924000698	-2,27001641	1,346015712
206178_at	PLA2G5	-0,924000698	-2,27001641	1,346015712
216264_s_at	LAMB2	-0,924000698	-2,27001641	1,346015712
1552890_a_at	CABP4	-0,924000698	-2,27001641	1,346015712

240889_at	CDRT15L2	-0,924000698	-2,27001641	1,346015712
1566884_at	-	-0,924000698	-2,27001641	1,346015712
209722_s_at	SERPINB9	-0,924000698	-2,27001641	1,346015712
224496_s_at	TMEM107	1,788922638	0,443143223	1,345779415
1556851_at	LOC100506609	-1,289805289	-2,635549085	1,345743796
212489_at	COL5A1	-1,289805289	-2,635549085	1,345743796
236340_at	C7orf13 /// LOC10	0,244681185	-1,100943374	1,34562456
1561082_at	NID1	0,244681185	-1,100943374	1,34562456
1557362_at	PHIP	0,244681185	-1,100943374	1,34562456
216993_s_at	COL11A2	0,648195588	-0,697182596	1,345378184
220290_at	AIM1L	0,648195588	-0,697182596	1,345378184
223735_at	ARL6	0,648195588	-0,697182596	1,345378184
213691_at	-	0,648195588	-0,697182596	1,345378184
209650_s_at	TBC1D22A	3,102023286	1,757155322	1,344867965
218093_s_at	ANKRD10	3,999234177	2,654387125	1,344847052
208806_at	CHD3	0,926325262	-0,418248858	1,34457412
230055_at	KHDC1	1,544940605	0,20062106	1,344319545
201598_s_at	INPPL1	3,086998005	1,743236669	1,343761335
222682_s_at	FAM114A2	2,890618076	1,547376668	1,343241408
201899_s_at	UBE2A	5,844522351	4,501663989	1,342858361
201542_at	SAR1A	5,210892881	3,868513789	1,342379092
211702_s_at	USP32	2,41945542	1,078619386	1,340836034
202667_s_at	SLC39A7	2,276736537	0,936232181	1,340504356
229624_at	OPA3	1,745502609	0,405752839	1,33974977
240991_at	-	0,282358733	-1,057301851	1,339660584
1554520_at	LOC283861	0,282358733	-1,057301851	1,339660584
217934_x_at	STUB1	3,366614157	2,026957145	1,339657012
242864_at	ZNF554	-0,180990326	-1,520022123	1,339031798
229719_s_at	DERL3	-0,180990326	-1,520022123	1,339031798
243983_at	-	-0,180990326	-1,520022123	1,339031798
208397_x_at	KCNJ5	-0,180990326	-1,520022123	1,339031798
231185_at	KIAA1161	-0,180990326	-1,520022123	1,339031798
208577_at	HIST1H3A /// HIST	0,677745787	-0,661178575	1,338924361
1554286_at	FLJ25758	0,677745787	-0,661178575	1,338924361
211172_x_at	AKAP7	0,677745787	-0,661178575	1,338924361
206437_at	S1PR4	3,012642182	1,674071917	1,338570265
212705_x_at	PNPLA2	0,820160788	-0,518232988	1,338393776
213923_at	RAP2B	4,179494373	2,841176366	1,338318007
235320_at	ARL6	1,047341799	-0,290694975	1,338036774
207137_at	TONSL	-0,230432956	-1,568385657	1,337952701
233585_at	SDK2	-0,230432956	-1,568385657	1,337952701
1562632_at	LOC285191	-0,230432956	-1,568385657	1,337952701
211396_at	FCGR2C	-0,230432956	-1,568385657	1,337952701
206571_s_at	MAP4K4	2,908690075	1,571112806	1,337577269
217466_x_at	RPS2 /// SNORA64	6,455529871	5,118000298	1,337529573
202542_s_at	AIMP1	5,941023555	4,603515312	1,337508243
224577_at	ERGIC1	2,138605583	0,80114751	1,337458074
212746_s_at	CEP170	3,843767728	2,506994817	1,336772911
232550_at	ABHD1	-0,135065865	-1,471833228	1,336767363
207321_s_at	ABCB9	-0,135065865	-1,471833228	1,336767363

237718_at	EIF4E	-0,135065865	-1,471833228	1,336767363
220584_at	FLJ22184	-0,135065865	-1,471833228	1,336767363
243186_at	-	-0,135065865	-1,471833228	1,336767363
223582_at	GPR98	-0,135065865	-1,471833228	1,336767363
243022_at	-	-0,135065865	-1,471833228	1,336767363
243848_at	-	-0,280273599	-1,616924751	1,336651152
224356_x_at	MS4A6A	-0,280273599	-1,616924751	1,336651152
239111_at	PRDM8	-0,280273599	-1,616924751	1,336651152
214536_at	SLURP1	-0,280273599	-1,616924751	1,336651152
217514_at	-	-0,280273599	-1,616924751	1,336651152
214103_s_at	RAP2A	-0,280273599	-1,616924751	1,336651152
1563919_a_at	CEP89	-0,280273599	-1,616924751	1,336651152
230530_at	-	-0,280273599	-1,616924751	1,336651152
61734_at	RCN3	0,442070058	-0,894483297	1,336553354
1563405_at	ATP4B	0,524545436	-0,811769547	1,336314983
229408_at	HDAC5	0,524545436	-0,811769547	1,336314983
208894_at	HLA-DRA	7,307372297	5,971060562	1,336311735
226505_x_at	USP32	4,290445502	2,954216214	1,336229288
202956_at	ARFGEF1	3,446505209	2,110584371	1,335920839
229294_at	JPH3	-0,66934516	-2,005029581	1,335684422
1559605_a_at	LOC285043	-0,66934516	-2,005029581	1,335684422
221367_at	MOS	-0,66934516	-2,005029581	1,335684422
215274_at	SLC12A3	-0,66934516	-2,005029581	1,335684422
235910_x_at	ZNF316	-0,66934516	-2,005029581	1,335684422
216322_at	CD58	-0,66934516	-2,005029581	1,335684422
242083_at	ZNF81	-0,66934516	-2,005029581	1,335684422
244321_at	PGAP1	-0,66934516	-2,005029581	1,335684422
212543_at	AIM1	4,486128467	3,150841611	1,335286856
224587_at	SUB1	5,582065707	4,246936542	1,335129166
201724_s_at	GALNT1	2,972517974	1,637482551	1,335035423
218979_at	RMI1	3,978219531	2,64347734	1,33474219
205653_at	CTSG	0,319287178	-1,015398016	1,334685194
228279_s_at	-	0,319287178	-1,015398016	1,334685194
221309_at	RBM17	0,319287178	-1,015398016	1,334685194
229612_at	-	0,319287178	-1,015398016	1,334685194
227056_at	KIAA0141	2,33082239	0,996453274	1,334369116
218020_s_at	ZFAND3	2,33082239	0,996453274	1,334369116
227390_at	MEG3	-0,332405896	-1,665791776	1,33338588
230589_at	TRAF3IP2-AS1	-0,332405896	-1,665791776	1,33338588
215479_at	-	-0,332405896	-1,665791776	1,33338588
223587_s_at	AMN	-0,332405896	-1,665791776	1,33338588
215738_at	LINC00563	-0,332405896	-1,665791776	1,33338588
213874_at	SERPINA4	-0,332405896	-1,665791776	1,33338588
208172_s_at	KCNB2	-0,332405896	-1,665791776	1,33338588
220807_at	HBQ1	-0,332405896	-1,665791776	1,33338588
230705_at	SLC2A5	-0,332405896	-1,665791776	1,33338588
227676_at	FAM3D	-0,088974936	-1,422257026	1,333282091
216530_at	-	-0,088974936	-1,422257026	1,333282091
231596_at	-	-0,088974936	-1,422257026	1,333282091
215827_x_at	CROCCP3	-0,088974936	-1,422257026	1,333282091

215005_at	NECAB2	-0,088974936	-1,422257026	1,333282091
229017_s_at	DSTYK	-0,088974936	-1,422257026	1,333282091
208309_s_at	MALT1	3,175897496	1,842852096	1,333045399
216300_x_at	RARA	1,830468611	0,497424714	1,333043897
201628_s_at	RRAGA	3,991087145	2,65824137	1,332845775
203988_s_at	FUT8	4,106824781	2,774037376	1,332787405
1569157_s_at	ZNF846	1,510970097	0,178191865	1,332778232
201764_at	TMEM106C	5,938043502	4,605332091	1,332711411
224410_s_at	LMBR1	3,642593015	2,309925448	1,332667567
213057_at	ATPAF2	1,345611839	0,013190398	1,332421441
212550_at	STAT5B	2,983826368	1,651676651	1,332149717
1559808_at	-	1,071336699	-0,260526297	1,331862995
218176_at	MAGEF1	2,791258167	1,459545271	1,331712896
221299_at	GPR173	-0,384193355	-1,715576125	1,33138277
1553967_at	ADAT3	-0,384193355	-1,715576125	1,33138277
232806_s_at	FAM131A	-0,384193355	-1,715576125	1,33138277
223543_at	PDZD4	-0,384193355	-1,715576125	1,33138277
209425_at	AMACR /// C1QTN	-0,384193355	-1,715576125	1,33138277
213620_s_at	ICAM2	4,803736169	3,472563131	1,331173038
37028_at	PPP1R15A	2,271659866	0,940676135	1,330983731
225513_at	SCRN2	0,707657549	-0,623254098	1,330911647
229201_at	-	2,159837409	0,829309537	1,330527872
1554149_at	CLDND1	5,930297053	4,600158152	1,330138901
222350_at	-	-0,856259172	-2,186086603	1,329827431
232069_at	KIF26A	-0,856259172	-2,186086603	1,329827431
1570339_x_at	-	-0,856259172	-2,186086603	1,329827431
215035_at	IGLV6-57	-0,856259172	-2,186086603	1,329827431
232389_at	WIPF3	-0,856259172	-2,186086603	1,329827431
207560_at	SLC28A1	-0,856259172	-2,186086603	1,329827431
225337_at	ABHD2	2,908690075	1,578991006	1,329699069
212131_at	LSM14A	5,170477358	3,84088223	1,329595128
225771_at	AP1G1	3,322661262	1,993309863	1,329351399
213813_x_at	-	2,719381633	1,390070295	1,329311338
1566767_at	-	-1,137208348	-2,466349414	1,329141066
1553202_at	STOX1	-1,137208348	-2,466349414	1,329141066
242395_at	-	-1,137208348	-2,466349414	1,329141066
213824_at	OLIG2	-1,137208348	-2,466349414	1,329141066
201451_x_at	RHEB	-1,137208348	-2,466349414	1,329141066
213751_at	PPP1R37	-1,137208348	-2,466349414	1,329141066
230889_at	LOC645321	-1,137208348	-2,466349414	1,329141066
243614_s_at	PRODH2	-1,137208348	-2,466349414	1,329141066
214961_at	MTUS2	-1,06282519	-2,391863529	1,329038339
216015_s_at	NLRP3	-1,06282519	-2,391863529	1,329038339
235364_at	TSC22D3	-1,06282519	-2,391863529	1,329038339
208187_s_at	-	-1,06282519	-2,391863529	1,329038339
206400_at	LGALS7 /// LGALS7	-1,06282519	-2,391863529	1,329038339
240273_at	-	-1,06282519	-2,391863529	1,329038339
1553427_at	ADAMTS15	-1,06282519	-2,391863529	1,329038339
224986_s_at	PDPK1	4,389611304	3,060577077	1,329034227
221013_s_at	APOL2	1,5944803	0,265475485	1,329004815

210246_s_at	ABCC8	-0,043925712	-1,372734086	1,328808374
1560353_at	-	-0,043925712	-1,372734086	1,328808374
216568_x_at	-	-0,043925712	-1,372734086	1,328808374
220106_at	NPC1L1	-0,043925712	-1,372734086	1,328808374
238083_at	PARP10	-0,043925712	-1,372734086	1,328808374
239583_x_at	PSG5	-0,043925712	-1,372734086	1,328808374
1561619_at	-	-0,043925712	-1,372734086	1,328808374
210405_x_at	TNFRSF10B	-0,043925712	-1,372734086	1,328808374
207205_at	CEACAM4	0,355751005	-0,972893339	1,328644344
208269_s_at	ADAM28	0,355751005	-0,972893339	1,328644344
240340_at	SPATA3	0,355751005	-0,972893339	1,328644344
221662_s_at	SLC22A7	0,355751005	-0,972893339	1,328644344
1557644_at	-	0,001114523	-1,32740143	1,328515953
205665_at	TSPAN9	0,001114523	-1,32740143	1,328515953
234092_s_at	TM6SF2	0,001114523	-1,32740143	1,328515953
1553802_a_at	SOX3	0,001114523	-1,32740143	1,328515953
234816_at	LINC00521	0,001114523	-1,32740143	1,328515953
229948_at	-	0,001114523	-1,32740143	1,328515953
240306_at	LINC00312	0,001114523	-1,32740143	1,328515953
229370_at	-	2,671492308	1,343200523	1,328291784
1556110_at	-	0,045517965	-1,282520722	1,328038687
241512_at	SPATC1	0,045517965	-1,282520722	1,328038687
241577_at	-	0,045517965	-1,282520722	1,328038687
220780_at	PLA2G3	0,045517965	-1,282520722	1,328038687
227083_at	B3GALTL	0,556428218	-0,771340337	1,327768555
1569341_at	-	-1,452713826	-2,780225148	1,327511322
216293_at	CLTA	1,528073958	0,20062106	1,327452899
202113_s_at	SNX2	4,413844848	3,086398747	1,327446101
224804_s_at	FAM219B	2,890618076	1,563256447	1,327361629
222006_at	LETM1	2,725848647	1,399283506	1,326565141
240911_at	NOS1	-0,437449947	-1,763664074	1,326214127
1560827_at	-	-0,437449947	-1,763664074	1,326214127
210684_s_at	DLG4	-0,437449947	-1,763664074	1,326214127
1555206_at	LOC100287210	-0,437449947	-1,763664074	1,326214127
214278_s_at	NDRG2	-0,437449947	-1,763664074	1,326214127
1555197_a_at	C21orf58	-0,437449947	-1,763664074	1,326214127
218553_s_at	KCTD15	-0,437449947	-1,763664074	1,326214127
239029_at	TMCC1	-0,437449947	-1,763664074	1,326214127
202238_s_at	NNMT	-0,437449947	-1,763664074	1,326214127
228302_x_at	CAMK2N1	-1,369772723	-2,695640852	1,325868129
221560_at	MARK4	2,460890032	1,135235995	1,325654037
238824_at	-	1,094118704	-0,231045907	1,325164611
231249_at	SZT2	0,873789652	-0,45121326	1,325002911
1566151_at	-	0,735999505	-0,589001171	1,325000676
233645_s_at	C1RL	0,735999505	-0,589001171	1,325000676
203848_at	AKAP8	2,392346173	1,067365565	1,324980608
224981_at	TMEM219	3,723597116	2,398910172	1,324686944
218383_at	HAUS4 /// MIR470	4,1816995	2,857256661	1,324442839
226770_at	MAGI3	1,287793882	-0,036543687	1,324337569
230320_at	TBRG1	1,287793882	-0,036543687	1,324337569

221924_at	ZMIZ2	1,287793882	-0,036543687	1,324337569
1552332_at	TRIOBP	1,610353504	0,286279868	1,324073636
223313_s_at	MAGED4 /// MAGI	-1,212652659	-2,536339282	1,323686622
209066_x_at	UQCRB	6,881195055	5,557694213	1,323500842
203879_at	PIK3CD	5,265546262	3,942089645	1,323456617
216885_s_at	DCAF8	2,179708195	0,85655369	1,323154505
244126_at	PEX11G	0,39105295	-0,931539147	1,322592097
243449_at	-	0,39105295	-0,931539147	1,322592097
218377_s_at	RWDD2B	3,209439373	1,887158627	1,322280746
233657_at	OPN5	0,58788177	-0,734355396	1,322237166
1554077_a_at	TMEM53	0,58788177	-0,734355396	1,322237166
226647_at	TMEM25	0,58788177	-0,734355396	1,322237166
205843_x_at	CRAT	1,97249974	0,65030602	1,32219372
39582_at	CYLD	3,122837626	1,800796705	1,322040921
243_g_at	MAP4	4,442341926	3,120398629	1,321943297
242356_at	VTI1A	1,383446753	0,061773582	1,321673171
214372_x_at	ERN2	1,383446753	0,061773582	1,321673171
203599_s_at	WBP4	2,99543179	1,674071917	1,321359873
242655_at	BARD1	2,063971513	0,742671819	1,321299694
240805_at	-	0,08619576	-1,234835326	1,321031086
238854_at	LOC100506668	0,08619576	-1,234835326	1,321031086
229262_at	PPP1R37	0,08619576	-1,234835326	1,321031086
216363_at	-	0,08619576	-1,234835326	1,321031086
207230_at	CDON	0,08619576	-1,234835326	1,321031086
206242_at	TM4SF5	0,08619576	-1,234835326	1,321031086
239395_at	ARRDC3-AS1	0,08619576	-1,234835326	1,321031086
212187_x_at	PTGDS	0,08619576	-1,234835326	1,321031086
227150_at	MTF1	2,972517974	1,651676651	1,320841323
230738_at	-	1,20396005	-0,116767475	1,320727525
236194_at	-	1,20396005	-0,116767475	1,320727525
225921_at	NIN	4,013714191	2,693044596	1,320669595
222212_s_at	CERS2	5,166702612	3,846857035	1,319845577
203963_at	CA12	-0,730013898	-2,049747196	1,319733298
1564308_a_at	MPP7	-0,730013898	-2,049747196	1,319733298
1569668_at	-	-0,730013898	-2,049747196	1,319733298
243628_at	COPS7B	-0,730013898	-2,049747196	1,319733298
231286_at	C9orf47	-0,730013898	-2,049747196	1,319733298
213236_at	SASH1	-0,730013898	-2,049747196	1,319733298
231732_at	SMPD3	-0,730013898	-2,049747196	1,319733298
216348_at	-	4,2463808	2,927197469	1,319183331
32094_at	CHST3	-0,417870544	-1,736940109	1,319069564
206959_s_at	UPF3A	1,625482993	0,306454867	1,319028126
1553910_at	NBPF4	-0,49284695	-1,811780055	1,318933104
238330_s_at	MPRIP	-0,49284695	-1,811780055	1,318933104
243947_s_at	-	-0,49284695	-1,811780055	1,318933104
231746_at	MIXL1	-0,49284695	-1,811780055	1,318933104
232764_at	CCNB2	-0,49284695	-1,811780055	1,318933104
228637_at	ZDHHC1	-0,49284695	-1,811780055	1,318933104
1559821_at	-	-0,49284695	-1,811780055	1,318933104
204095_s_at	ELL	0,764143511	-0,554696666	1,318840177

209081_s_at	COL18A1	0,127732831	-1,19085694	1,318589771
221051_s_at	NMRK2	0,127732831	-1,19085694	1,318589771
219830_at	RAI1	0,127732831	-1,19085694	1,318589771
233412_x_at	-	0,127732831	-1,19085694	1,318589771
204601_at	N4BP1	1,936380191	0,61779983	1,318580361
226361_at	TMEM42	2,698955954	1,380552339	1,318403615
226597_at	REEP6	0,900065578	-0,418248858	1,318314436
230819_at	C2CD4C	0,900065578	-0,418248858	1,318314436
202002_at	ACAA2	0,900065578	-0,418248858	1,318314436
223438_s_at	PPARA	1,116462765	-0,201789721	1,318252486
237117_at	-	-0,994186487	-2,312054429	1,317867942
1553818_x_at	LOC100653171 ///	-0,994186487	-2,312054429	1,317867942
214865_at	DOT1L	-0,994186487	-2,312054429	1,317867942
210020_x_at	CALML3	-0,994186487	-2,312054429	1,317867942
211966_at	COL4A2	-0,994186487	-2,312054429	1,317867942
208702_x_at	APLP2	2,817838865	1,500059916	1,317778948
202031_s_at	WIPI2	3,204847647	1,887158627	1,31768902
219166_at	DNAAF2	3,060716358	1,743236669	1,317479689
222573_s_at	SAV1	3,147261986	1,830129603	1,317132382
208803_s_at	SRP72	3,228532861	1,911502437	1,317030425
1556172_at	-	1,402495885	0,085534992	1,316960892
204155_s_at	SIK3	1,402495885	0,085534992	1,316960892
210563_x_at	CFLAR	5,36737436	4,051142838	1,316231522
1554479_a_at	CARD8	3,671843723	2,355777584	1,316066139
226155_at	FAM160B1	3,349017424	2,03313004	1,315887384
222691_at	SLC35B3	4,402175336	3,086398747	1,315776589
217696_at	FUT7	0,618277321	-0,697182596	1,315459918
226253_at	LRRC45	1,493647653	0,178191865	1,315455788
204426_at	TMED2	3,45766549	2,142313983	1,315351507
223964_x_at	-	2,427650693	1,112548615	1,315102078
213324_at	SRC	1,024284941	-0,290694975	1,314979916
229887_at	ALS2CL	1,024284941	-0,290694975	1,314979916
238077_at	KCTD6	3,657457797	2,34250725	1,314950546
241745_at	LOC100507557	0,425036312	-0,889222211	1,314258523
207684_at	TBX6	0,425036312	-0,889222211	1,314258523
240576_at	TBC1D26 /// ZNF2	0,425036312	-0,889222211	1,314258523
203759_at	ST3GAL4	0,425036312	-0,889222211	1,314258523
229979_x_at	-	0,425036312	-0,889222211	1,314258523
226165_at	C8orf59	5,964736786	4,650762375	1,313974411
241594_at	-	-1,289805289	-2,603471267	1,313665978
235945_at	SRD5A2	-1,289805289	-2,603471267	1,313665978
236789_at	-	-1,289805289	-2,603471267	1,313665978
215120_s_at	SAMD4A	-1,289805289	-2,603471267	1,313665978
241127_at	-	-1,289805289	-2,603471267	1,313665978
1554850_at	VWA5B2	-1,289805289	-2,603471267	1,313665978
227736_at	C10orf99	-1,289805289	-2,603471267	1,313665978
208474_at	CLDN6	0,926325262	-0,386896102	1,313221364
238404_x_at	SEZ6L2	0,926325262	-0,386896102	1,313221364
1559438_at	C21orf58	0,926325262	-0,386896102	1,313221364
1561270_at	-	0,167727503	-1,145057014	1,312784518

204813_at	MAPK10	0,167727503	-1,145057014	1,312784518
211605_s_at	RARA	0,167727503	-1,145057014	1,312784518
243458_at	-	0,167727503	-1,145057014	1,312784518
206611_at	C2orf27A	0,167727503	-1,145057014	1,312784518
217645_at	COX16 /// SYNJ2BI	2,127965541	0,815200271	1,31276527
212217_at	PREPL	3,142467702	1,830129603	1,312338099
227797_x_at	LYRM2	1,421391637	0,109458907	1,311932731
60084_at	CYLD	-0,135065865	-1,446832185	1,31176632
218516_s_at	IMPAD1	2,778527892	1,46719264	1,311335252
205614_x_at	MST1	1,139057614	-0,172194225	1,311251839
220101_x_at	-	-0,551284523	-1,862269442	1,310984919
239323_at	LOC100216546	-0,551284523	-1,862269442	1,310984919
204038_s_at	LPAR1	-0,551284523	-1,862269442	1,310984919
234864_s_at	TRPM6	-0,551284523	-1,862269442	1,310984919
231231_at	-	-0,551284523	-1,862269442	1,310984919
238607_at	ZNF296	-0,551284523	-1,862269442	1,310984919
1553268_at	RHBDL3	-0,551284523	-1,862269442	1,310984919
222293_at	CADM4	-0,551284523	-1,862269442	1,310984919
213186_at	DZIP3	3,060716358	1,750483134	1,310233225
233179_x_at	SIRT6	2,007220798	0,697221375	1,309999423
227854_at	-	2,25755351	0,947673886	1,309879624
1555900_at	-	1,246401134	-0,063377083	1,309778216
241957_x_at	LIN7B	0,648195588	-0,661178575	1,309374163
203464_s_at	EPN2	0,648195588	-0,661178575	1,309374163
230205_at	ZNF561	1,65554524	0,346964736	1,308580504
211979_at	GPR107	1,65554524	0,346964736	1,308580504
1562728_at	-	0,20740896	-1,100943374	1,308352334
208373_s_at	P2RY6	0,20740896	-1,100943374	1,308352334
234360_at	-	0,20740896	-1,100943374	1,308352334
220451_s_at	BIRC7	0,20740896	-1,100943374	1,308352334
38290_at	RGS14	3,172564982	1,864295957	1,308269025
1556355_x_at	RGL3	0,458905032	-0,84928999	1,308195022
236046_at	FLJ44896	0,458905032	-0,84928999	1,308195022
228757_at	HSD11B1L	0,458905032	-0,84928999	1,308195022
202088_at	SLC39A6	5,724396888	4,416511981	1,307884906
213178_s_at	MAPK8IP3	1,047341799	-0,260526297	1,307868096
219453_at	KLHL36	1,047341799	-0,260526297	1,307868096
239324_at	-	1,345611839	0,03784737	1,307764468
241682_at	KLHL23	2,374261973	1,067365565	1,306896408
225989_at	HERC4	2,452674045	1,146010634	1,306663411
217819_at	GOLGA7	5,986343899	4,679852812	1,306491087
225506_at	KIAA1468	1,730929079	0,424693306	1,306235773
200836_s_at	MAP4	2,884644843	1,578991006	1,305653837
228200_at	ZNF252P	1,528073958	0,222432814	1,305641145
218584_at	TCTN1	2,51558601	1,209973561	1,305612449
205856_at	SLC14A1	-0,791294935	-2,09678811	1,305493175
237071_at	-	-0,791294935	-2,09678811	1,305493175
235082_at	PCNX	-0,791294935	-2,09678811	1,305493175
240998_at	-	-0,791294935	-2,09678811	1,305493175
206322_at	SYN3	-0,791294935	-2,09678811	1,305493175

230828_at	GRAMD2	-0,791294935	-2,09678811	1,305493175
207671_s_at	BEST1	-0,791294935	-2,09678811	1,305493175
224877_s_at	MRPS5	2,621163856	1,315897482	1,305266374
207973_x_at	ACRV1	-0,924000698	-2,229233437	1,30523274
206696_at	GPR143	-0,924000698	-2,229233437	1,30523274
243384_at	-	-0,924000698	-2,229233437	1,30523274
240646_at	GIMAP8	-0,924000698	-2,229233437	1,30523274
216290_x_at	-	-0,924000698	-2,229233437	1,30523274
237425_at	LOC100505890	-0,924000698	-2,229233437	1,30523274
1556224_a_at	LOC155060 /// ZNI	-0,924000698	-2,229233437	1,30523274
244191_at	RPLP1	-0,924000698	-2,229233437	1,30523274
202206_at	ARL4C	3,076375717	1,771157334	1,305218383
214634_at	HIST1H4A /// HIST	0,820160788	-0,484558493	1,304719281
231812_x_at	PHAX	2,312738346	1,008210238	1,304528108
212402_at	ZC3H13	3,398333742	2,093850742	1,304483
200605_s_at	PRKAR1A	6,084541703	4,780167437	1,304374266
215096_s_at	ESD	6,311467565	5,007355999	1,304111566
219689_at	SEMA3G	0,491906512	-0,811769547	1,303676058
229258_at	KIF12	0,491906512	-0,811769547	1,303676058
214790_at	SENP6	2,159837409	0,85655369	1,303283719
209305_s_at	GADD45B	2,523100136	1,220470658	1,302629478
230380_at	THAP2	1,458255986	0,155747281	1,302508706
224184_s_at	BOC	1,071336699	-0,231045907	1,302382605
220924_s_at	SLC38A2	5,364029837	4,06178694	1,302242897
211129_x_at	EDA	-0,609040214	-1,911206569	1,302166356
219434_at	TREM1	-0,609040214	-1,911206569	1,302166356
1564883_a_at	TAS1R1	-0,609040214	-1,911206569	1,302166356
211823_s_at	PXN	-0,609040214	-1,911206569	1,302166356
220130_x_at	LTB4R2	-0,609040214	-1,911206569	1,302166356
1558631_at	PPARA	-0,609040214	-1,911206569	1,302166356
215348_at	ADCY1	-0,609040214	-1,911206569	1,302166356
231198_at	CDK6	-0,609040214	-1,911206569	1,302166356
204581_at	CD22	-0,609040214	-1,911206569	1,302166356
240584_at	-	0,244681185	-1,057301851	1,301983036
1553511_at	GJD3	0,244681185	-1,057301851	1,301983036
1568728_s_at	RNF207	0,244681185	-1,057301851	1,301983036
212970_at	APBB2	0,244681185	-1,057301851	1,301983036
208579_x_at	H2BFS /// HIST1H2	4,62997918	3,328091364	1,301887816
221776_s_at	BRD7	3,883929118	2,582046772	1,301882346
218955_at	BRF2	3,19484646	1,893444329	1,30140213
235812_at	CNEP1R1	3,691400566	2,390281999	1,301118567
201933_at	CHMP1A	2,99543179	1,694547353	1,300884437
227959_at	-	1,544940605	0,244646697	1,300293908
208532_x_at	KRTAP5-8	1,544940605	0,244646697	1,300293908
218819_at	INTS6	4,116324812	2,81616114	1,300163672
214149_s_at	LOC100652765	3,071179046	1,771157334	1,300021712
1556507_at	LOC100507274	2,401508182	1,101780212	1,29972797
235917_at	-	1,182925501	-0,116767475	1,299692976
234749_s_at	POC1A	1,760255626	0,461385738	1,298869888
215568_x_at	LYPLA2	1,760255626	0,461385738	1,298869888

230264_s_at	AP1S2	5,272133841	3,973269509	1,298864332
205211_s_at	RIN1	0,847529938	-0,45121326	1,298743197
220465_at	LOC80054	0,847529938	-0,45121326	1,298743197
202009_at	TWF2	3,273854609	1,975797592	1,298057017
204769_s_at	TAP2	2,765070895	1,46719264	1,297878256
227295_at	IKBIP	3,581038515	2,283169614	1,297868901
230228_at	SSC5D	0,282358733	-1,015398016	1,297756749
237041_x_at	RCOR1	0,282358733	-1,015398016	1,297756749
239628_at	-	0,282358733	-1,015398016	1,297756749
214079_at	DHRS2	0,282358733	-1,015398016	1,297756749
226088_at	ZDHHC12	2,365121417	1,067365565	1,297755852
223586_at	ARNTL2	3,081633533	1,784182261	1,297451272
213735_s_at	COX5B	6,511307451	5,213891167	1,297416284
225572_at	CREB1	3,6071174	2,309925448	1,297191952
228094_at	AMICA1	4,473867663	3,176868665	1,296998998
215622_x_at	PHF7	0,707657549	-0,589001171	1,29665872
217346_at	-	1,561816652	0,265475485	1,296341168
213521_at	PTPN18	1,775035811	0,479089184	1,295946627
235394_at	PLAA	1,094118704	-0,201789721	1,295908425
235067_at	MKLN1	2,267141818	0,971607464	1,295534354
241858_at	TNNI3K	-1,369772723	-2,665156778	1,295384055
211274_at	TBX1	-1,369772723	-2,665156778	1,295384055
213470_s_at	HNRNPH1	3,045068376	1,750483134	1,294585242
218696_at	EIF2AK3	3,461795772	2,167793428	1,294002345
217266_at	RPL15	4,823280506	3,52937772	1,293902786
224563_at	WASF2	2,552836808	1,259081178	1,29375563
229746_x_at	LOC100505876	3,67542249	2,381744287	1,293678203
218966_at	MYO5C	2,348178434	1,055317091	1,292861343
203300_x_at	AP1S2	4,381466431	3,08892491	1,292541521
239115_at	-	0,319287178	-0,972893339	1,292180516
218779_x_at	EPS8L1	0,319287178	-0,972893339	1,292180516
230989_s_at	TSSK6	0,319287178	-0,972893339	1,292180516
206092_x_at	RTEL1	0,873789652	-0,418248858	1,29203851
227135_at	NAAA	2,75889404	1,46719264	1,2917014
1553004_at	PKD1L1	-1,137208348	-2,4284334	1,291225052
216510_x_at	IGHA1 /// IGHG1 /,	-1,137208348	-2,4284334	1,291225052
216831_s_at	RUNX1T1	-1,137208348	-2,4284334	1,291225052
236952_at	-	-1,137208348	-2,4284334	1,291225052
208449_s_at	FGF8	-1,137208348	-2,4284334	1,291225052
1564242_at	-	-1,137208348	-2,4284334	1,291225052
209412_at	TRAPPC10	3,214269085	1,923216608	1,291052477
240549_at	-	-0,180990326	-1,471833228	1,290842902
216145_at	-	-0,180990326	-1,471833228	1,290842902
219319_at	HIF3A	-0,180990326	-1,471833228	1,290842902
214303_x_at	MUC5AC	-0,180990326	-1,471833228	1,290842902
215389_s_at	TNNT2	-0,180990326	-1,471833228	1,290842902
233997_at	LOC100287166	-0,180990326	-1,471833228	1,290842902
239854_at	SYCE3	-0,180990326	-1,471833228	1,290842902
224079_at	IL17C	0,735999505	-0,554696666	1,290696171
226004_at	CABLES2	3,152091004	1,861868165	1,290222839

204670_x_at	HLA-DRB1 /// HLA-	6,42919573	5,139270174	1,289925556
231375_at	-	-0,230432956	-1,520022123	1,289589168
1552549_a_at	BSND	-0,230432956	-1,520022123	1,289589168
206350_at	APCS	-0,230432956	-1,520022123	1,289589168
218913_s_at	GMIP	1,65554524	0,366031214	1,289514026
225010_at	CCDC6	4,393737464	3,104562113	1,289175351
1569876_at	C17orf57	-1,212652659	-2,501359113	1,288706454
227419_x_at	PLAC9	-1,212652659	-2,501359113	1,288706454
1556476_at	LOC283485	-1,212652659	-2,501359113	1,288706454
1569293_x_at	CROCCP2	1,116462765	-0,172194225	1,288656989
214742_at	AZI1	1,116462765	-0,172194225	1,288656989
203469_s_at	CDK10	1,326463531	0,03784737	1,288616161
227658_s_at	PLEKHA3	1,326463531	0,03784737	1,288616161
217977_at	MSRB1	3,89798617	2,609615156	1,288371013
219287_at	KCNMB4	1,421391637	0,133104519	1,288287118
211531_x_at	PRB1	1,421391637	0,133104519	1,288287118
227102_at	TRIM35	2,248155141	0,960042218	1,288112923
205540_s_at	RRAGB	-0,280273599	-1,568385657	1,288112058
236416_at	ARHGEF7	-0,280273599	-1,568385657	1,288112058
236725_at	WWC1	-0,280273599	-1,568385657	1,288112058
213217_at	ADCY2	-0,280273599	-1,568385657	1,288112058
229452_at	TMEM88	-0,280273599	-1,568385657	1,288112058
210908_s_at	PFDN5	6,69739962	5,4095074	1,28789222
1556896_at	LOC284751	-1,06282519	-2,350444759	1,287619569
217000_at	-	-1,06282519	-2,350444759	1,287619569
1559502_s_at	LRRC25	-1,06282519	-2,350444759	1,287619569
1560806_at	LOC646743 /// TIS	-1,06282519	-2,350444759	1,287619569
239727_at	DIO3OS /// MIR12	-1,06282519	-2,350444759	1,287619569
210560_at	GBX2	-1,06282519	-2,350444759	1,287619569
1560576_at	-	-1,06282519	-2,350444759	1,287619569
217995_at	SQRDL	5,696307299	4,408785731	1,287521568
232484_at	-	-0,66934516	-1,956681069	1,28733591
1569887_a_at	LOC286135	-0,66934516	-1,956681069	1,28733591
237800_at	-	-0,66934516	-1,956681069	1,28733591
1552910_at	SIGLEC11	-0,66934516	-1,956681069	1,28733591
206009_at	ITGA9	-0,66934516	-1,956681069	1,28733591
1556269_at	MYT1	-0,66934516	-1,956681069	1,28733591
243735_at	ELP2	-0,66934516	-1,956681069	1,28733591
234643_x_at	-	-0,135065865	-1,422257026	1,287191161
237624_at	-	-0,135065865	-1,422257026	1,287191161
1559563_at	-	-0,135065865	-1,422257026	1,287191161
234063_at	-	-0,135065865	-1,422257026	1,287191161
215683_at	LOC100506070	-0,135065865	-1,422257026	1,287191161
203488_at	LPHN1	-0,135065865	-1,422257026	1,287191161
1567410_at	ZNF135	0,900065578	-0,386896102	1,28696168
204435_at	NUPL1	3,685074281	2,398910172	1,286164109
231526_at	-	-0,856259172	-2,142286822	1,28602765
240740_at	LOC100507443	-0,856259172	-2,142286822	1,28602765
239136_at	LOC728978	-0,856259172	-2,142286822	1,28602765
234004_at	TTC28	-0,856259172	-2,142286822	1,28602765

211315_s_at	CACNA1G	-0,856259172	-2,142286822	1,28602765
215386_at	-	-0,856259172	-2,142286822	1,28602765
206655_s_at	GP1BB /// SEPT5 /,	-0,856259172	-2,142286822	1,28602765
209934_s_at	ATP2C1	3,122148189	1,836262316	1,285885873
223250_at	KLHL7	3,096793374	1,811004388	1,285788985
211178_s_at	PSTPIP1	2,51558601	1,2298509	1,28573511
227625_s_at	STUB1	5,368322292	4,083008429	1,285313863
222710_at	AMIGO3 /// GMPP	0,58788177	-0,697182596	1,285064367
206739_at	HOXC5	0,58788177	-0,697182596	1,285064367
220498_at	ACTL7B	-0,332405896	-1,616924751	1,284518855
230723_at	SPATA18	-0,332405896	-1,616924751	1,284518855
237370_at	-	-0,332405896	-1,616924751	1,284518855
1557774_at	-	-0,332405896	-1,616924751	1,284518855
213611_at	AQP5	-0,332405896	-1,616924751	1,284518855
230812_at	LOC100499467	-0,332405896	-1,616924751	1,284518855
200724_at	RPL10	-0,332405896	-1,616924751	1,284518855
1562214_at	LOC151171	-0,332405896	-1,616924751	1,284518855
216878_x_at	HAB1	-0,332405896	-1,616924751	1,284518855
230622_at	MLLT4	-0,332405896	-1,616924751	1,284518855
213721_at	SOX2	-0,332405896	-1,616924751	1,284518855
207457_s_at	LY6G6D	-0,332405896	-1,616924751	1,284518855
229059_at	FAM225A /// FAM	-0,332405896	-1,616924751	1,284518855
224346_at	-	1,345611839	0,061773582	1,283838257
226079_at	FLYWCH2	1,139057614	-0,144765583	1,283823197
242632_at	FGD2	-0,088974936	-1,372734086	1,28375915
228448_at	MAP6	-0,088974936	-1,372734086	1,28375915
211253_x_at	PYY	-0,088974936	-1,372734086	1,28375915
229191_at	TBCD	-0,088974936	-1,372734086	1,28375915
1556021_at	GPR180	-0,088974936	-1,372734086	1,28375915
231516_at	LOC100507146	-0,088974936	-1,372734086	1,28375915
219668_at	GDAP1L1	0,001114523	-1,282520722	1,283635245
224141_at	FLJ38668	0,001114523	-1,282520722	1,283635245
210673_x_at	NKX2-1	0,001114523	-1,282520722	1,283635245
213868_s_at	DHRS7	0,001114523	-1,282520722	1,283635245
227573_s_at	OBSL1	0,001114523	-1,282520722	1,283635245
205384_at	FXYD1	0,001114523	-1,282520722	1,283635245
213840_s_at	MRPS12	0,001114523	-1,282520722	1,283635245
240886_at	CASR	0,001114523	-1,282520722	1,283635245
206874_s_at	SLK	3,060716358	1,777135134	1,283581225
217608_at	SREK1IP1	1,610353504	0,326777209	1,283576294
221725_at	WASF2	3,248151517	1,964670566	1,283480951
222472_at	AFTPH	3,248151517	1,964670566	1,283480951
206090_s_at	DISC1 /// TSNAX-D	-0,043925712	-1,32740143	1,283475718
241802_x_at	GRM2	-0,043925712	-1,32740143	1,283475718
216980_s_at	SPN	-0,043925712	-1,32740143	1,283475718
234536_at	SARDH	-0,043925712	-1,32740143	1,283475718
1555084_at	-	-0,043925712	-1,32740143	1,283475718
1570483_at	-	-0,043925712	-1,32740143	1,283475718
235776_x_at	LINC00475	-0,043925712	-1,32740143	1,283475718
223811_s_at	GET4 /// SUN1	1,246401134	-0,036543687	1,282944821

211546_x_at	SNCA	1,246401134	-0,036543687	1,282944821
218965_s_at	TUT1	1,246401134	-0,036543687	1,282944821
221992_at	NPIPL2	0,764143511	-0,518232988	1,282376499
201414_s_at	NAP1L4	4,393737464	3,111774082	1,281963382
225414_at	RNF149	4,972743529	3,691084872	1,281658657
239481_at	FAM133A	-0,384193355	-1,665791776	1,281598421
206963_s_at	NAT8 /// NAT8B	-0,384193355	-1,665791776	1,281598421
230997_at	TTC21A	-0,384193355	-1,665791776	1,281598421
216900_s_at	CHRNA4	-0,384193355	-1,665791776	1,281598421
219597_s_at	DUOX1	-0,384193355	-1,665791776	1,281598421
244725_at	DMRTA1	-0,384193355	-1,665791776	1,281598421
213586_at	CDKN2D	-0,384193355	-1,665791776	1,281598421
1558458_at	LOC401320	-0,384193355	-1,665791776	1,281598421
208032_s_at	GRIA3	-0,384193355	-1,665791776	1,281598421
219322_s_at	WRAP73	3,438642427	2,157148588	1,281493838
222243_s_at	TOB2	2,804738618	1,523429058	1,281309559
65493_at	HEATR6	1,578017757	0,296712595	1,281305162
230521_at	C9orf100	0,926325262	-0,354781582	1,281106844
231783_at	CHRM1	-1,289805289	-2,570522741	1,280717453
1552609_s_at	IL28A /// IL28B	-1,289805289	-2,570522741	1,280717453
206146_s_at	RHAG	0,045517965	-1,234835326	1,280353291
216562_at	-	0,045517965	-1,234835326	1,280353291
209768_s_at	GP1BB /// SEPT5-G	0,045517965	-1,234835326	1,280353291
233206_at	SLC22A23	0,39105295	-0,889222211	1,280275161
224096_at	MIR4755	0,39105295	-0,889222211	1,280275161
223956_at	TMPRSS13	0,39105295	-0,889222211	1,280275161
233128_at	-	0,39105295	-0,889222211	1,280275161
202583_s_at	RANBP9	3,562907192	2,283169614	1,279737578
218050_at	UFM1	4,696534086	3,416886612	1,279647474
205934_at	PLCL1	0,618277321	-0,661178575	1,279455896
228644_s_at	SLC12A4	0,618277321	-0,661178575	1,279455896
226198_at	TOM1L2	0,618277321	-0,661178575	1,279455896
227469_at	PTEN	0,618277321	-0,661178575	1,279455896
220258_s_at	WRAP53	1,364555912	0,085534992	1,27902092
1555656_at	CD300LG	1,960819106	0,681874787	1,27894432
207799_x_at	ARMCX4	-0,437449947	-1,715576125	1,278126178
224497_x_at	HSD17B14	-0,437449947	-1,715576125	1,278126178
1554685_a_at	KIAA1199	-0,437449947	-1,715576125	1,278126178
1557540_at	LOC100507403	-0,437449947	-1,715576125	1,278126178
235839_at	C22orf34	-0,437449947	-1,715576125	1,278126178
240116_at	-	-0,437449947	-1,715576125	1,278126178
1562368_at	CARD11	-0,437449947	-1,715576125	1,278126178
228016_s_at	-	-0,437449947	-1,715576125	1,278126178
204833_at	ATG12	3,132805465	1,855279162	1,277526304
228864_at	ZNF653	0,08619576	-1,19085694	1,2770527
205654_at	C4BPA	0,08619576	-1,19085694	1,2770527
221929_at	-	0,08619576	-1,19085694	1,2770527
1569008_at	FAM201B /// LOC1	0,08619576	-1,19085694	1,2770527
209134_s_at	RPS6	8,371902615	7,094933662	1,276968953
208823_s_at	CDK16	1,701542258	0,424693306	1,276848952

231967_at	PHF20L1	2,784898907	1,508107415	1,276791492
233350_s_at	TEX264	2,019346222	0,742671819	1,276674403
213420_at	DHX57	2,019346222	0,742671819	1,276674403
236922_at	-	0,791908897	-0,484558493	1,276467391
226146_at	LOC100859930	3,983349065	2,707013102	1,276335963
227977_at	ZADH2	1,910557155	0,634301128	1,276256027
216697_at	TRIO	-0,994186487	-2,27001641	1,275829923
1553134_s_at	C9orf72	-0,994186487	-2,27001641	1,275829923
1553623_at	MGC15705	-0,994186487	-2,27001641	1,275829923
1568999_at	PSG4	-0,994186487	-2,27001641	1,275829923
216763_at	-	-0,994186487	-2,27001641	1,275829923
203953_s_at	CLDN3	-0,994186487	-2,27001641	1,275829923
243276_at	ALS2CL	-0,994186487	-2,27001641	1,275829923
219584_at	PLA1A	-0,994186487	-2,27001641	1,275829923
225985_at	PRKAA1	1,476175815	0,20062106	1,275554755
208829_at	TAPBP	4,413844848	3,138547521	1,275297327
205684_s_at	DENND4C	2,75889404	1,483698568	1,275195472
218170_at	ISOC1	4,714814978	3,439619824	1,275195154
203790_s_at	HRSP12	4,116324812	2,841176366	1,275148446
231908_at	ZDHHC18	-0,730013898	-2,005029581	1,275015683
204486_at	KCNQ1OT1	-0,730013898	-2,005029581	1,275015683
242658_at	-	-0,730013898	-2,005029581	1,275015683
215881_x_at	SSX2 /// SSX2B ///	-0,730013898	-2,005029581	1,275015683
1553212_at	KRT78	-0,730013898	-2,005029581	1,275015683
221614_s_at	RPH3AL	-0,730013898	-2,005029581	1,275015683
209617_s_at	CTNND2	-0,730013898	-2,005029581	1,275015683
1569461_at	KCNT1	-0,730013898	-2,005029581	1,275015683
233771_at	-	-0,730013898	-2,005029581	1,275015683
202659_at	PSMB10	5,234483294	3,959534682	1,274948613
227944_at	PTPN3	2,484824532	1,209973561	1,274850971
1554341_a_at	HELQ	1,287793882	0,013190398	1,274603484
219267_at	GLTP	2,725848647	1,451507543	1,274341104
1557372_at	ATP6V1E2 /// FLJ4	0,425036312	-0,84928999	1,274326302
209578_s_at	POFUT2	1,640193838	0,366031214	1,274162625
1568649_at	-	1,383446753	0,109458907	1,273987846
219433_at	BCOR	3,378744007	2,104873932	1,273870075
218446_s_at	FAM18B1	3,513985141	2,24081821	1,273166931
1558882_at	HTATSF1P2	1,716003175	0,443143223	1,272859952
228518_at	IGHG1 /// IGHM	0,127732831	-1,145057014	1,272789845
1562601_at	UNQ6975	0,127732831	-1,145057014	1,272789845
213422_s_at	MXRA8	0,127732831	-1,145057014	1,272789845
235467_s_at	KCNC4	0,127732831	-1,145057014	1,272789845
222037_at	MCM4	2,932161212	1,659532492	1,272628721
218456_at	CAPRIN2	2,374261973	1,101780212	1,272481762
225840_at	TEF	1,578719607	0,306454867	1,27226474
202463_s_at	MBD3	1,578719607	0,306454867	1,27226474
204930_s_at	BNIP1	2,21987062	0,947673886	1,272196734
228120_at	EIF2C1	2,304041045	1,032267596	1,271773449
210477_x_at	MAPK8	0,648195588	-0,623254098	1,271449687
218522_s_at	MAP1S	0,648195588	-0,623254098	1,271449687

218245_at	TSKU	0,648195588	-0,623254098	1,271449687
221044_s_at	TRIM34 /// TRIM6	2,127965541	0,85655369	1,271411851
213383_at	SBF1	2,127965541	0,85655369	1,271411851
236413_at	-	0,820160788	-0,45121326	1,271374047
242842_at	-	0,820160788	-0,45121326	1,271374047
204899_s_at	SAP30	2,705643918	1,434743608	1,270900309
216716_at	ABO	-0,49284695	-1,763664074	1,270817124
221446_at	ADAM30	-0,49284695	-1,763664074	1,270817124
230711_at	-	-0,49284695	-1,763664074	1,270817124
1566786_at	-	-0,49284695	-1,763664074	1,270817124
220505_at	C9orf53	-0,49284695	-1,763664074	1,270817124
207656_s_at	ACOX1	-0,49284695	-1,763664074	1,270817124
239964_at	TCL6	-0,49284695	-1,763664074	1,270817124
1553060_at	PSKH2	-0,49284695	-1,763664074	1,270817124
235265_at	UBR3	-0,49284695	-1,763664074	1,270817124
226674_at	SHISA4	-0,49284695	-1,763664074	1,270817124
208412_s_at	RARB	-0,49284695	-1,763664074	1,270817124
207257_at	EPO	-0,49284695	-1,763664074	1,270817124
232300_at	LOC100133190	-0,49284695	-1,763664074	1,270817124
235095_at	CCDC64B	-0,49284695	-1,763664074	1,270817124
203426_s_at	IGFBP5	-0,49284695	-1,763664074	1,270817124
237583_at	-	-0,49284695	-1,763664074	1,270817124
222022_at	DTX3	0,458905032	-0,811769547	1,270674578
235221_at	CBLN3	0,458905032	-0,811769547	1,270674578
239390_at	GTDC1	0,458905032	-0,811769547	1,270674578
201332_s_at	STAT6	0,458905032	-0,811769547	1,270674578
224776_at	AGPAT6	3,876052392	2,605630202	1,27042219
228763_at	MDP1 /// NEDD8-I	2,678352494	1,408220608	1,270131886
207170_s_at	LETMD1	4,244034996	2,973964204	1,270070792
1556000_s_at	BTBD7	1,65554524	0,385582632	1,269962608
204411_at	KIF21B	3,481948861	2,212304392	1,269644468
213109_at	TNIK	2,312738346	1,043469112	1,269269234
202839_s_at	NDUFB7	4,393737464	3,124666253	1,269071212
228970_at	ZBTB8OS	4,66447449	3,395519718	1,268954772
215272_at	OGG1	0,167727503	-1,100943374	1,268670877
234677_at	-	0,167727503	-1,100943374	1,268670877
209756_s_at	MYCN	0,167727503	-1,100943374	1,268670877
1566136_at	-	0,167727503	-1,100943374	1,268670877
208034_s_at	PROZ	0,167727503	-1,100943374	1,268670877
213919_at	DNAJC4	0,167727503	-1,100943374	1,268670877
208535_x_at	COL13A1	0,167727503	-1,100943374	1,268670877
215205_x_at	NCOR2	0,167727503	-1,100943374	1,268670877
234732_s_at	EFCAB6	-1,537851782	-2,8064286	1,268576817
218542_at	CEP55	4,701786435	3,433779805	1,26800663
46270_at	UBAP1	2,335063796	1,067191317	1,267872479
220566_at	PIK3R5	0,97663406	-0,290694975	1,267329035
227901_at	LOC648987	0,97663406	-0,290694975	1,267329035
209342_s_at	IKBKB	1,948628481	0,681874787	1,266753694
214449_s_at	RHOQ	1,510970097	0,244646697	1,2663234
237419_at	-	1,094118704	-0,172194225	1,266312929

224413_s_at	TM2D2	4,357852544	3,091548197	1,266304347
226966_at	PRPF40B	0,847529938	-0,418248858	1,265778796
221525_at	ZMIZ2	0,847529938	-0,418248858	1,265778796
240618_at	-	-1,369772723	-2,635549085	1,265776362
216567_at	-	-1,369772723	-2,635549085	1,265776362
229745_x_at	DACT3	-1,369772723	-2,635549085	1,265776362
209027_s_at	ABI1	4,776409588	3,510668356	1,265741232
213864_s_at	NAP1L1	7,4713349	6,205905918	1,265428982
219233_s_at	GSDMB	2,285848435	1,020514937	1,265333499
226034_at	DUSP4	4,644330782	3,379204729	1,265126053
227444_at	ARMCX4	0,20740896	-1,057301851	1,264710811
227163_at	GSTO2	0,20740896	-1,057301851	1,264710811
224494_x_at	HSD17B14	0,20740896	-1,057301851	1,264710811
236744_at	PHPT1	0,20740896	-1,057301851	1,264710811
214490_at	ARSF	0,20740896	-1,057301851	1,264710811
230541_at	LOC149134	0,20740896	-1,057301851	1,264710811
1553644_at	SYNE3	0,20740896	-1,057301851	1,264710811
1561127_at	ADARB2-AS1	0,20740896	-1,057301851	1,264710811
230973_at	SH2D5	0,20740896	-1,057301851	1,264710811
219021_at	RNF121	2,007220798	0,742671819	1,264548979
218325_s_at	DIDO1	3,228532861	1,964670566	1,263862295
221789_x_at	RHOT2	1,960819106	0,697221375	1,263597732
239076_at	-	0,491906512	-0,771340337	1,263246849
223389_s_at	ZNF581	2,849132494	1,586121802	1,263010693
160020_at	MMP14	0,743770596	-0,519201391	1,262971988
204117_at	PREP	4,468222748	3,205255599	1,262967149
216228_s_at	WDHD1	1,528073958	0,265475485	1,262598474
229925_at	SLC6A17	0,707657549	-0,554696666	1,262354215
206416_at	ZNF205	0,707657549	-0,554696666	1,262354215
241129_at	-	-0,924000698	-2,186086603	1,262085905
241341_at	-	-0,924000698	-2,186086603	1,262085905
211191_at	CD84	-0,924000698	-2,186086603	1,262085905
216053_x_at	FAM182A /// FAM	-0,924000698	-2,186086603	1,262085905
209587_at	PITX1	-0,924000698	-2,186086603	1,262085905
1560943_s_at	PGD	-0,924000698	-2,186086603	1,262085905
240660_at	-	-0,924000698	-2,186086603	1,262085905
213339_at	TP73-AS1	-0,924000698	-2,186086603	1,262085905
214990_at	PIGO	-0,924000698	-2,186086603	1,262085905
230639_at	ICA1	-0,924000698	-2,186086603	1,262085905
229656_s_at	EML6	1,440265662	0,178191865	1,262073797
1557117_at	-	1,440265662	0,178191865	1,262073797
208800_at	SRP72	4,512206259	3,250322783	1,261883475
219186_at	ZBTB7A	1,225123479	-0,036543687	1,261667166
216505_x_at	-	5,710011704	4,448392964	1,261618741
224691_at	UHMK1	5,738671649	4,477070446	1,261601203
209913_x_at	AP5Z1	1,116462765	-0,144765583	1,261228348
228014_at	PTRH1	1,116462765	-0,144765583	1,261228348
223226_x_at	SSBP4	2,71254166	1,451507543	1,261034117
225451_at	GRIPAP1	2,752615529	1,491644074	1,260971455
206047_at	GNB3	0,873789652	-0,386896102	1,260685754

221442_at	MC3R	-0,551284523	-1,811780055	1,260495532
216370_s_at	TKTL1	-0,551284523	-1,811780055	1,260495532
1559324_at	USP32P2	-0,551284523	-1,811780055	1,260495532
230575_at	MSRB2	-0,551284523	-1,811780055	1,260495532
216470_x_at	PRSS2	-0,551284523	-1,811780055	1,260495532
1562563_at	HCN1	-0,551284523	-1,811780055	1,260495532
238319_at	LOC644090	-0,551284523	-1,811780055	1,260495532
216209_at	LOC400084	-0,551284523	-1,811780055	1,260495532
217174_s_at	APC2	-0,551284523	-1,811780055	1,260495532
212713_at	MFAP4	-0,551284523	-1,811780055	1,260495532
240404_at	-	-0,551284523	-1,811780055	1,260495532
232186_at	FITM2	-0,551284523	-1,811780055	1,260495532
206266_at	GPLD1	-0,551284523	-1,811780055	1,260495532
210821_x_at	CENPA	3,64600241	2,385765892	1,260236518
214616_at	HIST1H3A /// HIST	0,244681185	-1,015398016	1,260079202
240954_at	-	0,244681185	-1,015398016	1,260079202
243699_at	LOC100507006	0,244681185	-1,015398016	1,260079202
233918_at	DCDC2B	0,244681185	-1,015398016	1,260079202
228003_at	RAB30	0,244681185	-1,015398016	1,260079202
228456_s_at	CDS2	3,892081305	2,63203137	1,260049935
225722_at	MIR3658 /// UCK2	1,775035811	0,515457602	1,259578209
226063_at	VAV2	1,625482993	0,366031214	1,25945178
205140_at	FPGT	1,625482993	0,366031214	1,25945178
41644_at	SASH1	-0,306104283	-1,565555436	1,259451153
218065_s_at	TMEM9B	3,928402242	2,669456704	1,258945538
239920_at	UBTF	0,524545436	-0,734355396	1,258900832
235893_at	-	0,524545436	-0,734355396	1,258900832
239532_at	-	0,524545436	-0,734355396	1,258900832
226077_at	RNF145	5,663100961	4,404237854	1,258863107
209900_s_at	SLC16A1	4,308320282	3,049587049	1,258733233
234312_s_at	ACSS2	1,544940605	0,286279868	1,258660737
219640_at	CLDN15	2,507817181	1,249194639	1,258622542
228580_at	HTRA3	-0,791294935	-2,049747196	1,258452261
1561712_at	-	-0,791294935	-2,049747196	1,258452261
240571_at	-	-0,791294935	-2,049747196	1,258452261
220626_at	SERPINA10	-0,791294935	-2,049747196	1,258452261
227697_at	SOCS3	-0,791294935	-2,049747196	1,258452261
211061_s_at	MGAT2	4,05770789	2,799505781	1,258202109
200612_s_at	AP2B1	3,939815341	2,682180306	1,257635036
205189_s_at	FANCC	1,246401134	-0,011001236	1,257402369
226243_at	PTRHD1	6,106065323	4,849624422	1,256440901
209057_x_at	CDC5L	2,476788561	1,220470658	1,256317902
226078_at	RPUSD1	1,139057614	-0,116767475	1,255825089
226105_at	C1GALT1	1,139057614	-0,116767475	1,255825089
203734_at	FOXJ2	2,739067176	1,483698568	1,255368608
226682_at	RORA	4,299310603	3,043944324	1,255366279
1553034_at	SDCCAG8	1,024284941	-0,231045907	1,255330848
1556944_at	-	-1,624955693	-2,880284116	1,255328422
221961_at	CLCN7	0,282358733	-0,972893339	1,255252072
237331_s_at	MAPT-AS1	0,282358733	-0,972893339	1,255252072

1568629_s_at	PIK3R2	2,444186596	1,189078962	1,255107634
224687_at	ANKIB1	3,86465425	2,609615156	1,255039094
238451_at	MPP7	-1,137208348	-2,391863529	1,25465518
1552269_at	SPATA17	-1,137208348	-2,391863529	1,25465518
230197_s_at	TPPP	-1,137208348	-2,391863529	1,25465518
207579_at	MAGEB3	-1,137208348	-2,391863529	1,25465518
229690_at	FAM109A	0,735999505	-0,518232988	1,254232493
1563297_s_at	-	-1,212652659	-2,466349414	1,253696755
230814_at	C19orf81	-1,212652659	-2,466349414	1,253696755
217157_x_at	IGK@ /// IGKC	-1,212652659	-2,466349414	1,253696755
1555694_a_at	KCNIP3	0,556428218	-0,697182596	1,253610814
216446_at	-	0,556428218	-0,697182596	1,253610814
234238_at	MAP1LC3B2	-0,609040214	-1,862269442	1,253229228
206224_at	CST1	-0,609040214	-1,862269442	1,253229228
223791_at	FAM27A /// FAM2	-0,609040214	-1,862269442	1,253229228
224178_s_at	SOX6	-0,609040214	-1,862269442	1,253229228
218686_s_at	RHBDF1	-0,609040214	-1,862269442	1,253229228
1570140_at	ANXA8 /// ANXA8L	-0,609040214	-1,862269442	1,253229228
214292_at	ITGB4	-0,609040214	-1,862269442	1,253229228
230743_at	HOXB-AS3	-0,609040214	-1,862269442	1,253229228
219535_at	HUNK	-0,609040214	-1,862269442	1,253229228
228784_at	ST3GAL2	-0,609040214	-1,862269442	1,253229228
229188_s_at	ZNRF2	-0,609040214	-1,862269442	1,253229228
214568_at	TPSD1	-0,609040214	-1,862269442	1,253229228
223729_at	CECR2	-0,609040214	-1,862269442	1,253229228
243171_at	-	-0,609040214	-1,862269442	1,253229228
217843_s_at	MED4	4,013714191	2,760632255	1,253081936
211070_x_at	DBI	7,313006172	6,060876949	1,252129223
244110_at	MLL	2,41945542	1,167919222	1,251536198
202815_s_at	HEXIM1	1,948628481	0,697221375	1,251407106
222555_s_at	MRPL44	3,001575397	1,750483134	1,251092264
1555865_at	LOC255512	0,319287178	-0,931539147	1,250826325
233846_at	CCDC85A	0,319287178	-0,931539147	1,250826325
230386_at	-	0,319287178	-0,931539147	1,250826325
206148_at	IL3RA	0,319287178	-0,931539147	1,250826325
212795_at	KIAA1033	3,720319513	2,469496389	1,250823124
236667_at	TMEM120B	1,160996507	-0,089635713	1,25063222
234807_x_at	-	2,107136847	0,85655369	1,250583157
204427_s_at	TMED2	4,195854652	2,945366812	1,25048784
238026_at	RPL35A	2,613382867	1,362900801	1,250482066
239873_at	-	1,65554524	0,405752839	1,249792401
226860_at	TMEM19	3,996262843	2,746644067	1,249618776
203656_at	FIG4	3,282538749	2,03313004	1,249408709
226196_s_at	IFT43	3,305120269	2,055739171	1,249381099
1569159_at	LZTS1	-1,06282519	-2,312054429	1,249229239
220912_at	-	-1,06282519	-2,312054429	1,249229239
213686_at	VPS13A	1,047341799	-0,201789721	1,24913152
1566670_at	PDXK	1,047341799	-0,201789721	1,24913152
217117_x_at	MUC3A	0,58788177	-0,661178575	1,249060345
216385_at	LOC220077	0,58788177	-0,661178575	1,249060345

244561_at	SLC9A1	0,58788177	-0,661178575	1,249060345
235586_at	-	0,58788177	-0,661178575	1,249060345
230403_at	RFX3	1,493647653	0,244646697	1,249000956
213955_at	MYOZ3	0,764143511	-0,484558493	1,248702004
1552995_at	IL27	0,764143511	-0,484558493	1,248702004
231635_x_at	RNF31	0,764143511	-0,484558493	1,248702004
221108_at	C22orf43	0,926325262	-0,322340048	1,24866531
202038_at	UBE4A	5,184742504	3,936585452	1,248157051
201435_s_at	EIF4E	4,600469559	3,352762478	1,247707081
229362_at	PUS10	2,476788561	1,2298509	1,24693766
235291_s_at	FLJ32255	1,671397663	0,424693306	1,246704357
224749_at	ITFG3	1,671397663	0,424693306	1,246704357
1554378_a_at	PDE1C	-1,289805289	-2,536339282	1,246533993
207972_at	GLRA1	-1,289805289	-2,536339282	1,246533993
207899_at	GIP	-1,289805289	-2,536339282	1,246533993
1559948_at	-	-1,289805289	-2,536339282	1,246533993
218794_s_at	TXNL4B	2,552836808	1,306903969	1,245932839
222459_at	AKIRIN1	4,144849571	2,899658146	1,245191425
243394_at	-	0,355751005	-0,889222211	1,244973216
224758_at	C7orf73	1,760255626	0,515457602	1,244798024
1552510_at	SLC34A3	2,752615529	1,508107415	1,244508113
227015_at	ASPHD2	1,610353504	0,366031214	1,24432229
219986_s_at	ACAD10	1,071336699	-0,172194225	1,243530923
226336_at	LOC100288602 ///	5,476974716	4,233509532	1,243465184
1561093_at	SLC22A25	0,791908897	-0,45121326	1,243122157
213166_x_at	MIR4784 /// MZT2	6,37966935	5,136610842	1,243058508
217489_s_at	IL6R	-1,452713826	-2,695640852	1,242927026
212006_at	UBXN4	5,151467688	3,908597431	1,242870257
224922_at	CSNK2A2	3,498020785	2,255165935	1,24285485
211752_s_at	NDUFS7	4,686080913	3,443730948	1,242349965
219029_at	C5orf28	3,720319513	2,478123977	1,242195536
1553684_at	PPIL6	-0,66934516	-1,911206569	1,24186141
229372_at	GOLT1A	-0,66934516	-1,911206569	1,24186141
208547_at	HIST1H2BB	-0,66934516	-1,911206569	1,24186141
239072_at	EMB	-0,66934516	-1,911206569	1,24186141
1559291_at	LINC00032	-0,66934516	-1,911206569	1,24186141
236315_at	-	-0,66934516	-1,911206569	1,24186141
236425_at	-	-0,66934516	-1,911206569	1,24186141
206126_at	CXCR5	-0,66934516	-1,911206569	1,24186141
228616_at	POLRMTP1	-0,66934516	-1,911206569	1,24186141
220441_at	DNAJC22	-0,66934516	-1,911206569	1,24186141
236102_at	LOC100126784	-0,66934516	-1,911206569	1,24186141
229524_at	KIAA0494	-0,66934516	-1,911206569	1,24186141
240424_s_at	LOC441204	-0,66934516	-1,911206569	1,24186141
240276_at	-	-0,66934516	-1,911206569	1,24186141
221885_at	DENND2A	-0,66934516	-1,911206569	1,24186141
1553515_at	MYEOV2	-0,66934516	-1,911206569	1,24186141
226722_at	FAM20C	-0,66934516	-1,911206569	1,24186141
233181_at	-	-0,66934516	-1,911206569	1,24186141
226710_at	C8orf82	2,530390387	1,28853674	1,241853647

203643_at	ERF	0,618277321	-0,623254098	1,24153142
214635_at	CLDN9	0,618277321	-0,623254098	1,24153142
226125_at	LOC100288152	0,618277321	-0,623254098	1,24153142
217983_s_at	RNASET2	5,202180782	3,960716395	1,241464387
1559696_at	-	-0,230432956	-1,471833228	1,241400272
206878_at	DAO	-0,230432956	-1,471833228	1,241400272
234897_s_at	LY6G6D /// LY6G6I	-0,230432956	-1,471833228	1,241400272
230367_at	SMTNL1	-0,230432956	-1,471833228	1,241400272
236082_at	PHLDB3	-0,230432956	-1,471833228	1,241400272
231728_at	CAPS	-0,230432956	-1,471833228	1,241400272
220727_at	KCNK10	-0,230432956	-1,471833228	1,241400272
226111_s_at	ZNF385A	-0,230432956	-1,471833228	1,241400272
226018_at	C7orf41	-0,180990326	-1,422257026	1,241266701
221122_at	HRASLS2	-0,180990326	-1,422257026	1,241266701
239947_at	-	-0,180990326	-1,422257026	1,241266701
213559_s_at	ZNF467	-0,180990326	-1,422257026	1,241266701
1554228_a_at	C8orf56	-0,180990326	-1,422257026	1,241266701
240760_at	CDRT15	-0,180990326	-1,422257026	1,241266701
205911_at	PTH1R	-0,180990326	-1,422257026	1,241266701
233324_at	TRERF1	-0,180990326	-1,422257026	1,241266701
213965_s_at	CHD5	-0,180990326	-1,422257026	1,241266701
220155_s_at	BRD9	2,365121417	1,123905343	1,241216075
215737_x_at	USF2	3,175897496	1,935144844	1,240752652
204898_at	SAP30	-0,856259172	-2,09678811	1,240528938
241161_at	-	-0,856259172	-2,09678811	1,240528938
237677_at	-	-0,856259172	-2,09678811	1,240528938
1552911_at	SIGLEC11	-0,856259172	-2,09678811	1,240528938
235426_at	GATM	-0,856259172	-2,09678811	1,240528938
242026_at	MYCN	-0,856259172	-2,09678811	1,240528938
1569614_s_at	LCN8	-0,856259172	-2,09678811	1,240528938
235087_at	UNKL	-0,856259172	-2,09678811	1,240528938
211694_at	TSSK1B	-0,856259172	-2,09678811	1,240528938
230956_at	C11orf92	-0,856259172	-2,09678811	1,240528938
210329_s_at	SGCD	-0,856259172	-2,09678811	1,240528938
229378_at	STOX1	0,39105295	-0,84928999	1,24034294
223995_at	SLC12A9	0,39105295	-0,84928999	1,24034294
240278_at	RASSF1	0,39105295	-0,84928999	1,24034294
240774_at	-	0,39105295	-0,84928999	1,24034294
230381_at	C1orf186 /// LOC1	0,39105295	-0,84928999	1,24034294
236159_x_at	-	0,39105295	-0,84928999	1,24034294
235110_at	PLA2G16	0,39105295	-0,84928999	1,24034294
218765_at	SIDT2	1,857989965	0,61779983	1,240190135
212171_x_at	VEGFA	1,625482993	0,385582632	1,239900361
64488_at	IRGQ	2,075211776	0,835462955	1,239748821
237681_at	-	-0,280273599	-1,520022123	1,239748524
1561591_at	-	-0,280273599	-1,520022123	1,239748524
1553640_at	XKR6	-0,280273599	-1,520022123	1,239748524
1553837_at	PGAM5	-0,280273599	-1,520022123	1,239748524
217017_at	OSBPL10	-0,280273599	-1,520022123	1,239748524
210365_at	LOC100506403 ///	-0,280273599	-1,520022123	1,239748524

223717_s_at	ACRBP	-0,280273599	-1,520022123	1,239748524
222289_at	KCNC2	-0,280273599	-1,520022123	1,239748524
1556228_a_at	VCPIP1	2,096248267	0,85655369	1,239694578
218435_at	DNAJC15	5,701040555	4,46141139	1,239629166
200864_s_at	RAB11A	2,582572508	1,343200523	1,239371985
206820_at	AGFG2	-0,043925712	-1,282520722	1,23859501
228900_at	SPECC1	-0,043925712	-1,282520722	1,23859501
233067_at	PRDM11	-0,043925712	-1,282520722	1,23859501
210944_s_at	CAPN3	-0,043925712	-1,282520722	1,23859501
1566084_at	-	-0,088974936	-1,32740143	1,238426494
234857_at	-	-0,088974936	-1,32740143	1,238426494
228474_s_at	KLF9	-0,088974936	-1,32740143	1,238426494
212831_at	MEGF9	-0,088974936	-1,32740143	1,238426494
237503_at	SLC5A8	-0,088974936	-1,32740143	1,238426494
215266_at	DNAH3	0,820160788	-0,418248858	1,238409646
228327_x_at	MEIS3	0,820160788	-0,418248858	1,238409646
209303_at	NDUFS4	4,75100323	3,512665066	1,238338164
240291_at	-	-0,135065865	-1,372734086	1,237668221
238046_x_at	PWWP2B	-0,135065865	-1,372734086	1,237668221
211619_s_at	ALPP /// ALPPL2	-0,135065865	-1,372734086	1,237668221
216425_at	-	-0,135065865	-1,372734086	1,237668221
243098_at	-	-0,135065865	-1,372734086	1,237668221
230525_at	LINC00514 /// LOC	-0,135065865	-1,372734086	1,237668221
216601_at	AOC4	-0,135065865	-1,372734086	1,237668221
1555257_a_at	MYO3B	-0,135065865	-1,372734086	1,237668221
212412_at	PDLIM5	3,822956033	2,585619776	1,237336257
226159_at	C5orf51	3,374552989	2,137342905	1,237210084
1556645_s_at	-	0,648195588	-0,589001171	1,237196759
228304_at	RBM43	0,648195588	-0,589001171	1,237196759
1559524_at	-	0,425036312	-0,811769547	1,236805859
207025_at	GJC2	0,425036312	-0,811769547	1,236805859
221750_at	HMGCS1	5,014397839	3,77783183	1,236566009
238802_at	TYSND1	0,045517965	-1,19085694	1,236374905
1566581_at	-	0,045517965	-1,19085694	1,236374905
220998_s_at	UNC93B1	0,045517965	-1,19085694	1,236374905
210185_at	CACNB1	0,045517965	-1,19085694	1,236374905
1555037_a_at	IDH1	4,372691748	3,136334485	1,236357263
1569496_s_at	LOC100130872	1,345611839	0,109458907	1,236152932
205446_s_at	ATF2	1,345611839	0,109458907	1,236152932
214799_at	NFASC	-0,332405896	-1,568385657	1,235979761
243116_at	PIP5KL1	-0,332405896	-1,568385657	1,235979761
202877_s_at	CD93	-0,332405896	-1,568385657	1,235979761
1562034_at	LINC00163	-0,332405896	-1,568385657	1,235979761
233980_s_at	VWF	-0,332405896	-1,568385657	1,235979761
239702_x_at	-	-0,332405896	-1,568385657	1,235979761
1555550_at	ZACN	-0,332405896	-1,568385657	1,235979761
225966_at	C17orf89	-0,332405896	-1,568385657	1,235979761
230034_x_at	MRPL41	-0,332405896	-1,568385657	1,235979761
216924_s_at	DRD2	-0,332405896	-1,568385657	1,235979761
228296_at	YPEL1	0,001114523	-1,234835326	1,235949849

234052_at	-	0,001114523	-1,234835326	1,235949849
207491_at	MOGAT2	0,001114523	-1,234835326	1,235949849
225631_at	EEPD1	0,001114523	-1,234835326	1,235949849
229961_x_at	YJEFN3	0,001114523	-1,234835326	1,235949849
220326_s_at	ARHGEF40	0,001114523	-1,234835326	1,235949849
244175_at	LINC00577	0,001114523	-1,234835326	1,235949849
1563030_at	-	0,001114523	-1,234835326	1,235949849
217182_at	MUC5AC	0,001114523	-1,234835326	1,235949849
225282_at	SMAP2	4,603614396	3,367769667	1,235844729
216036_x_at	WDTC1	1,458255986	0,222432814	1,235823172
215139_at	ARHGEF10	-1,712732543	-2,948507524	1,235774981
207180_s_at	HTATIP2	5,092485786	3,856800376	1,235685411
229536_at	REC8	-0,994186487	-2,229233437	1,23504695
237944_at	-	-0,994186487	-2,229233437	1,23504695
208522_s_at	PTCH1	-0,994186487	-2,229233437	1,23504695
1559138_a_at	SPATA19	-0,994186487	-2,229233437	1,23504695
225280_x_at	ARSD	-0,994186487	-2,229233437	1,23504695
209819_at	HABP4	-0,994186487	-2,229233437	1,23504695
222377_at	TBX10	-0,994186487	-2,229233437	1,23504695
206359_at	SOCS3	-0,994186487	-2,229233437	1,23504695
218491_s_at	THYN1	5,22510269	3,990095334	1,235007356
1562022_s_at	LOC100130987 ///	2,908690075	1,674071917	1,234618159
216834_at	RGS1	6,641740374	5,407136961	1,234603413
225856_at	CLOCK	1,802733148	0,568155442	1,234577705
244672_at	WDR1	0,847529938	-0,386896102	1,23442604
244467_at	SHISA8	-1,369772723	-2,603471267	1,233698544
232197_x_at	ARSB	1,730929079	0,497424714	1,233504364
225573_at	ACAD11 /// NPHP3	1,730929079	0,497424714	1,233504364
230048_at	IFRD1	1,116462765	-0,116767475	1,233230239
226580_at	BRMS1L	2,692288672	1,459545271	1,232743401
207208_at	RBMXL2	-0,384193355	-1,616924751	1,232731396
234422_at	-	-0,384193355	-1,616924751	1,232731396
217681_at	WNT7B	-0,384193355	-1,616924751	1,232731396
230965_at	USP2	-0,384193355	-1,616924751	1,232731396
226395_at	HOOK3	2,955426542	1,722747778	1,232678764
207650_x_at	PTGER1	0,677745787	-0,554696666	1,232442453
238476_at	CREBRF	0,677745787	-0,554696666	1,232442453
241733_at	C18orf54	0,677745787	-0,554696666	1,232442453
1568488_at	-	-1,624955693	-2,856570137	1,231614444
202253_s_at	DNM2	1,476175815	0,244646697	1,231529118
240114_s_at	TMEM174	1,000459215	-0,231045907	1,231505121
202785_at	NDUFA7	5,167863921	3,936585452	1,231278469
208576_s_at	HIST1H3A /// HIST	0,08619576	-1,145057014	1,231252774
1552468_a_at	DSCR10	0,08619576	-1,145057014	1,231252774
233905_at	SUN5	0,08619576	-1,145057014	1,231252774
205781_at	C16orf7	0,08619576	-1,145057014	1,231252774
220782_x_at	KLK12	0,08619576	-1,145057014	1,231252774
231619_at	-	0,08619576	-1,145057014	1,231252774
239564_at	LOC100128198	0,08619576	-1,145057014	1,231252774
236499_at	C1orf86	0,08619576	-1,145057014	1,231252774

1555193_a_at	ZNF277	3,541158018	2,309925448	1,23123257
213351_s_at	TMCC1	2,2388565	1,008210238	1,230646262
212040_at	TGOLN2	3,313347235	2,082919929	1,230427306
226455_at	CREB3L4	2,41945542	1,189078962	1,230376457
203338_at	PPP2R5E	4,433194302	3,202942917	1,230251385
239775_at	-	0,458905032	-0,771340337	1,230245368
216852_x_at	IGLJ3	0,458905032	-0,771340337	1,230245368
204381_at	LRP3	0,458905032	-0,771340337	1,230245368
219113_x_at	HSD17B14	0,458905032	-0,771340337	1,230245368
229931_at	ZNF775	0,458905032	-0,771340337	1,230245368
224591_at	HP1BP3	6,29526566	5,065162801	1,230102858
226095_s_at	ATXN1L	2,190140919	0,960042218	1,230098701
213220_at	BBIP1	3,628556015	2,398910172	1,229645843
219517_at	ELL3	1,267146969	0,03784737	1,229299599
217847_s_at	THRAP3	1,139057614	-0,089635713	1,228693327
242012_at	-	0,127732831	-1,100943374	1,228676205
216344_at	NPHP4	0,127732831	-1,100943374	1,228676205
225355_at	NEURL1B	0,127732831	-1,100943374	1,228676205
202389_s_at	HTT	2,427650693	1,199296812	1,228353881
214926_at	SPTAN1	-0,437449947	-1,665791776	1,228341829
241374_at	TMEM39A	-0,437449947	-1,665791776	1,228341829
1566814_at	FGF22	-0,437449947	-1,665791776	1,228341829
244158_at	-	-0,437449947	-1,665791776	1,228341829
237901_at	-	-0,437449947	-1,665791776	1,228341829
1556066_at	KDM6B	-0,437449947	-1,665791776	1,228341829
219365_s_at	CAMKV	-0,437449947	-1,665791776	1,228341829
204100_at	THRA	-0,437449947	-1,665791776	1,228341829
215341_at	DNAH6	-0,437449947	-1,665791776	1,228341829
227097_at	-	-0,437449947	-1,665791776	1,228341829
225666_at	TMTC4	2,199941239	0,971607464	1,228333774
217461_x_at	-	1,671397663	0,443143223	1,22825444
225156_at	ELOF1	2,943764123	1,715863686	1,227900438
208919_s_at	NADK	3,570321555	2,34250725	1,227814305
229120_s_at	CDC42SE1	5,199716022	3,971987733	1,227728289
219079_at	CYB5R4	3,668220666	2,440833194	1,227387473
210031_at	CD247	5,766181318	4,538823943	1,227357375
35846_at	THRA	-0,122628793	-1,349889709	1,227260915
208682_s_at	MAGED2	2,878891456	1,651676651	1,227214805
217533_x_at	-	-0,730013898	-1,956681069	1,226667171
232026_at	HERC4	-0,730013898	-1,956681069	1,226667171
237409_at	-	-0,730013898	-1,956681069	1,226667171
204956_at	MTAP	-0,730013898	-1,956681069	1,226667171
231135_at	LOC151174	-0,730013898	-1,956681069	1,226667171
1568734_a_at	HS1BP3	-0,730013898	-1,956681069	1,226667171
230045_at	CNTN2	-0,730013898	-1,956681069	1,226667171
1568894_at	-	-0,730013898	-1,956681069	1,226667171
238998_x_at	OTUD1	-0,730013898	-1,956681069	1,226667171
231027_at	-	-0,730013898	-1,956681069	1,226667171
240336_at	HBM	-0,730013898	-1,956681069	1,226667171
208747_s_at	C1S	-0,730013898	-1,956681069	1,226667171

214987_at	GAB1	-0,730013898	-1,956681069	1,226667171
236895_at	-	-0,730013898	-1,956681069	1,226667171
1562468_at	-	-0,730013898	-1,956681069	1,226667171
1566776_at	DNAH1	-0,730013898	-1,956681069	1,226667171
206595_at	CST6	-0,730013898	-1,956681069	1,226667171
216329_at	-	-0,730013898	-1,956681069	1,226667171
221321_s_at	KCNIP2	-0,730013898	-1,956681069	1,226667171
1566633_at	-	-0,730013898	-1,956681069	1,226667171
207613_s_at	CAMK2A	-0,730013898	-1,956681069	1,226667171
201510_at	ELF3	-0,730013898	-1,956681069	1,226667171
237039_at	LOC100506088	-0,730013898	-1,956681069	1,226667171
244252_at	LOC399884	-0,730013898	-1,956681069	1,226667171
1558421_a_at	C14orf180	-0,730013898	-1,956681069	1,226667171
213987_s_at	CDK13	-0,730013898	-1,956681069	1,226667171
205518_s_at	CMAHP	4,72953846	3,502959177	1,226579282
221506_s_at	TNPO2	3,67542249	2,449078728	1,226343762
218436_at	SIL1	1,983901861	0,757638486	1,226263374
219020_at	HS1BP3	2,436165655	1,209973561	1,226192094
204973_at	GJB1	1,024284941	-0,201789721	1,226074662
209525_at	HDGFRP3	1,024284941	-0,201789721	1,226074662
217052_x_at	-	1,024284941	-0,201789721	1,226074662
239601_at	-	1,024284941	-0,201789721	1,226074662
219673_at	MCM9	3,190097062	1,964670566	1,225426496
206493_at	ITGA2B	0,167727503	-1,057301851	1,225029354
238950_at	TNFRSF9	0,167727503	-1,057301851	1,225029354
209337_at	PSIP1	6,044722928	4,819940636	1,224782292
223272_s_at	NTPCR	4,263008992	3,038411131	1,224597861
212050_at	WIPF2	2,890618076	1,666953618	1,223664458
218896_s_at	C17orf85	2,21987062	0,996453274	1,223417347
233070_at	ZNF197	0,20740896	-1,015398016	1,222806976
211234_x_at	ESR1	0,20740896	-1,015398016	1,222806976
212937_s_at	COL6A1	0,20740896	-1,015398016	1,222806976
1558875_at	SREBF1	0,20740896	-1,015398016	1,222806976
239772_x_at	DHX30	0,20740896	-1,015398016	1,222806976
244771_at	KBTBD12	-0,49284695	-1,715576125	1,222729175
223561_at	NEK6	-0,49284695	-1,715576125	1,222729175
238141_s_at	-	-0,49284695	-1,715576125	1,222729175
230839_at	PRMT8	-0,49284695	-1,715576125	1,222729175
216995_x_at	MKRN2	-0,49284695	-1,715576125	1,222729175
227472_at	DDA1	0,900065578	-0,322340048	1,222405626
227467_at	RDH10	0,900065578	-0,322340048	1,222405626
228786_at	LOC387647	0,524545436	-0,697182596	1,221728033
218144_s_at	INF2	0,524545436	-0,697182596	1,221728033
237052_x_at	GIGYF2	1,307201325	0,085534992	1,221666333
241904_at	-	1,528073958	0,306454867	1,221619092
225256_at	-	3,603313992	2,381744287	1,221569705
214114_x_at	FASTK	3,418470674	2,197404429	1,221066245
212103_at	KPNA6 /// LOC100	2,829929672	1,60918327	1,220746402
232092_at	SLC25A51	0,735999505	-0,484558493	1,220557998
235431_s_at	PELI3	0,735999505	-0,484558493	1,220557998

217347_at	-	2,41945542	1,199296812	1,220158607
223650_s_at	NRBF2	3,710746795	2,490640503	1,220106292
217810_x_at	LARS	3,291641956	2,071691486	1,21995047
226784_at	TWISTNB	2,983826368	1,76396664	1,219859728
202993_at	ILVBL	1,625482993	0,405752839	1,219730155
219313_at	GRAMD1C	1,047341799	-0,172194225	1,219536023
214877_at	CDKAL1	1,182925501	-0,036543687	1,219469188
1555301_a_at	DIP2A	1,182925501	-0,036543687	1,219469188
216042_at	TNFRSF25	1,182925501	-0,036543687	1,219469188
225462_at	TMEM128	4,195854652	2,97688045	1,218974203
235432_at	NPHP3	1,716003175	0,497424714	1,21857846
202204_s_at	AMFR	2,285848435	1,067365565	1,21848287
225012_at	HDLBP	2,99543179	1,777135134	1,218296656
215953_at	DKFZP564C196	-0,924000698	-2,142286822	1,218286124
229799_s_at	NCAM1	-0,924000698	-2,142286822	1,218286124
220443_s_at	VAX2	-0,924000698	-2,142286822	1,218286124
221003_s_at	CAB39L	0,244681185	-0,972893339	1,217574524
1555393_s_at	C21orf67	0,244681185	-0,972893339	1,217574524
240617_at	LOC100287098	0,244681185	-0,972893339	1,217574524
1561937_x_at	-	0,926325262	-0,290694975	1,217020237
232841_at	-	1,326463531	0,109458907	1,217004624
222994_at	PRDX5	5,659001793	4,442339631	1,216662162
204407_at	TTF2	4,398126622	3,181622175	1,216504447
220239_at	KLHL7	3,378744007	2,162541631	1,216202376
238165_at	PDZRN3	-1,212652659	-2,4284334	1,215780741
235105_at	-	-1,212652659	-2,4284334	1,215780741
215830_at	SHANK2	-1,212652659	-2,4284334	1,215780741
220511_s_at	DLC1	-1,212652659	-2,4284334	1,215780741
240720_at	-	-1,212652659	-2,4284334	1,215780741
235720_at	CRIP3	-1,212652659	-2,4284334	1,215780741
227346_at	IKZF1	5,502934249	4,287157115	1,215777135
241799_x_at	-	1,640193838	0,424693306	1,215500532
215778_x_at	HAB1	0,764143511	-0,45121326	1,215356771
224352_s_at	CFL2	1,20396005	-0,011001236	1,214961286
212798_s_at	ANKMY2	4,18872659	2,973964204	1,214762387
1569042_at	LRP1	-1,537851782	-2,752097204	1,214245421
229889_at	FAM211A	0,282358733	-0,931539147	1,21389788
235768_at	SH3RF2	-0,791294935	-2,005029581	1,213734646
233074_at	-	-0,791294935	-2,005029581	1,213734646
228360_at	LYPD6B	-0,791294935	-2,005029581	1,213734646
210510_s_at	NRP1	-0,791294935	-2,005029581	1,213734646
233777_at	-	-0,791294935	-2,005029581	1,213734646
206716_at	UMOD	-0,791294935	-2,005029581	1,213734646
243084_at	CALD1	-0,791294935	-2,005029581	1,213734646
204503_at	EVPL	-0,791294935	-2,005029581	1,213734646
230103_at	VSTM4	-0,791294935	-2,005029581	1,213734646
240399_at	-	-0,791294935	-2,005029581	1,213734646
1566156_at	-	-0,791294935	-2,005029581	1,213734646
200884_at	CKB	2,209870035	0,996453274	1,213416762
212897_at	CDK19	2,304041045	1,090640407	1,213400639

234240_at	-	-1,137208348	-2,350444759	1,213236411
234266_at	-	-1,137208348	-2,350444759	1,213236411
202953_at	C1QB	-1,137208348	-2,350444759	1,213236411
204879_at	PDPN	-1,137208348	-2,350444759	1,213236411
1557665_at	-	-1,137208348	-2,350444759	1,213236411
215410_at	LOC100132832 ///	-1,137208348	-2,350444759	1,213236411
233747_at	-	-1,137208348	-2,350444759	1,213236411
217826_s_at	UBE2J1	4,543207821	3,330174376	1,213033445
238092_at	LRFN4	0,95222556	-0,260526297	1,212751857
209402_s_at	SLC12A4	1,345611839	0,133104519	1,21250732
228169_s_at	-	-1,452713826	-2,665156778	1,212442952
231492_at	LOC100506034	-0,551284523	-1,763664074	1,212379551
230433_at	LOC729970	-0,551284523	-1,763664074	1,212379551
1555185_x_at	TERF2	-0,551284523	-1,763664074	1,212379551
234781_at	-	-0,551284523	-1,763664074	1,212379551
229866_at	STK32A	-0,551284523	-1,763664074	1,212379551
216700_at	TRIO	-0,551284523	-1,763664074	1,212379551
230700_at	RTN4RL1	-0,551284523	-1,763664074	1,212379551
205409_at	FOSL2	-0,551284523	-1,763664074	1,212379551
218960_at	TMPRSS4	-0,551284523	-1,763664074	1,212379551
1570185_at	-	1,745502609	0,533258442	1,212244167
204859_s_at	APAF1	2,955426542	1,743236669	1,212189872
217788_s_at	GALNT2	3,813758784	2,601822502	1,211936282
219730_at	MED18	1,225123479	0,013190398	1,211933081
219283_at	C1GALT1C1	3,584942056	2,373249966	1,21169209
205725_at	SCGB1A1	-1,289805289	-2,501359113	1,211553824
1560859_at	-	-1,289805289	-2,501359113	1,211553824
242716_at	SLC30A1	-1,289805289	-2,501359113	1,211553824
240999_at	LOC100287704	-1,289805289	-2,501359113	1,211553824
212112_s_at	STX12	3,77412549	2,562621495	1,211503995
212108_at	FAF2	4,402175336	3,191011385	1,211163951
243819_at	-	0,58788177	-0,623254098	1,211135869
220632_s_at	POMT2	0,58788177	-0,623254098	1,211135869
223167_s_at	USP25	2,312738346	1,101780212	1,210958134
219405_at	TRIM68	2,926639205	1,715863686	1,210775519
202362_at	RAP1A	6,845041927	5,634574899	1,210467028
212212_s_at	INTS1	2,107136847	0,896834102	1,210302745
227651_at	NACC1	2,590269697	1,380552339	1,209717358
210740_s_at	ITPK1	4,708268083	3,498658433	1,20960965
37226_at	BNIP1	1,80988752	0,600778976	1,209108544
225384_at	DOCK7	2,817838865	1,60918327	1,208655594
239533_at	GPR155	0,319287178	-0,889222211	1,208509389
209114_at	TSPAN1	0,319287178	-0,889222211	1,208509389
243523_at	LOC100128644	0,319287178	-0,889222211	1,208509389
207442_at	CSF3	0,319287178	-0,889222211	1,208509389
1555523_a_at	FYCO1	0,319287178	-0,889222211	1,208509389
219102_at	RCN3	0,319287178	-0,889222211	1,208509389
214298_x_at	38961	3,710746795	2,503049671	1,207697124
225632_s_at	ISY1-RAB43 /// RA	0,97663406	-0,231045907	1,207679967
211590_x_at	TBXA2R	0,618277321	-0,589001171	1,207278492

227841_at	AMDHD2 /// CEMI	0,618277321	-0,589001171	1,207278492
1561559_at	-	-1,06282519	-2,27001641	1,20719122
244077_at	C10orf113	-1,06282519	-2,27001641	1,20719122
231482_at	-	-1,06282519	-2,27001641	1,20719122
223973_at	MIR7-3HG	-1,06282519	-2,27001641	1,20719122
1558706_a_at	ATOH8	-1,06282519	-2,27001641	1,20719122
1553882_at	-	-1,06282519	-2,27001641	1,20719122
223837_at	GULP1	-1,06282519	-2,27001641	1,20719122
211109_at	JAK3	-1,06282519	-2,27001641	1,20719122
236771_at	RIPPLY2	-1,06282519	-2,27001641	1,20719122
1559313_at	-	-1,06282519	-2,27001641	1,20719122
224100_s_at	DPYSL5	-1,06282519	-2,27001641	1,20719122
236801_at	-	-1,06282519	-2,27001641	1,20719122
210938_at	PDX1	-1,06282519	-2,27001641	1,20719122
223537_s_at	WNT5B	-1,06282519	-2,27001641	1,20719122
234294_x_at	GATAD2A	3,414594082	2,207440269	1,207153813
241360_at	CCDC15	0,820160788	-0,386896102	1,20705689
1554160_a_at	ZNF446	0,820160788	-0,386896102	1,20705689
221888_at	CC2D1A	0,820160788	-0,386896102	1,20705689
207668_x_at	PDIA6	7,132034872	5,925603914	1,206430958
206846_s_at	HDAC6	2,436165655	1,2298509	1,206314755
230082_at	-	1,116462765	-0,089635713	1,206098477
214126_at	-	2,007220798	0,80114751	1,206073288
228719_at	ZSWIM7	1,267146969	0,061773582	1,205373387
238120_at	RPH3AL	0,355751005	-0,84928999	1,205040995
1555682_at	OTUB2	0,355751005	-0,84928999	1,205040995
228967_at	EIF1	2,878891456	1,674071917	1,204819539
201603_at	PPP1R12A	4,834334905	3,629733221	1,204601683
224658_x_at	PACS1	1,610353504	0,405752839	1,204600665
202125_s_at	TRAK2	2,492739983	1,28853674	1,204203243
218923_at	CTBS	3,517785666	2,314488991	1,203296676
201133_s_at	PJA2	4,241697258	3,038411131	1,203286128
218690_at	PDLIM4	0,648195588	-0,554696666	1,202892254
228110_x_at	-	0,39105295	-0,811769547	1,202822497
226578_s_at	DUSP1	0,39105295	-0,811769547	1,202822497
238071_at	LCN10 /// LCN6	0,39105295	-0,811769547	1,202822497
228889_at	ARHGAP5-AS1	0,39105295	-0,811769547	1,202822497
1558195_at	LINC00592	-0,609040214	-1,811780055	1,202739841
239704_at	RNF144B	-0,609040214	-1,811780055	1,202739841
243616_at	-	-0,609040214	-1,811780055	1,202739841
231103_at	KCNN3	-0,609040214	-1,811780055	1,202739841
221389_at	PLA2G2E	-0,609040214	-1,811780055	1,202739841
237742_at	-	-0,609040214	-1,811780055	1,202739841
240588_at	-	-0,609040214	-1,811780055	1,202739841
216043_x_at	-	-0,609040214	-1,811780055	1,202739841
1553178_a_at	SSTR3	1,139057614	-0,063377083	1,202434697
225704_at	FBRSL1	2,62869474	1,426280426	1,202414313
1562004_x_at	MYOZ3	0,847529938	-0,354781582	1,202311519
220968_s_at	TSPAN9	0,847529938	-0,354781582	1,202311519
217635_s_at	POLG	1,000459215	-0,201789721	1,202248936

225053_at	CNOT7	6,025413739	4,823639792	1,201773947
202260_s_at	STXBP1	3,374552989	2,173028091	1,201524897
224572_s_at	IRF2BP2	2,972517974	1,771157334	1,20136064
225953_at	RPRD1A	4,125707946	2,924357093	1,201350853
202079_s_at	TRAK1	1,528073958	0,326777209	1,201296749
212646_at	RFTN1	6,018567889	4,817293385	1,201274503
204157_s_at	SIK3	1,898032047	0,697221375	1,200810672
229950_s_at	-	-1,369772723	-2,570522741	1,200750019
1563098_at	IGFN1	-1,369772723	-2,570522741	1,200750019
206759_at	FCER2	-1,369772723	-2,570522741	1,200750019
211964_at	COL4A2	-1,369772723	-2,570522741	1,200750019
218310_at	KCTD7 /// RABGEF	3,603313992	2,403191094	1,200122898
200046_at	DAD1	6,125045416	4,925509668	1,199535748
225928_at	VTI1B	2,21987062	1,020514937	1,199355684
204799_at	ZBED4	3,286844008	2,088449486	1,198394522
202426_s_at	RXRA	1,983901861	0,786391549	1,197510312
229210_at	RNASEH2B	2,605420261	1,408220608	1,197199653
237337_at	-	1,024284941	-0,172194225	1,196479166
227219_x_at	MAP1LC3A	1,024284941	-0,172194225	1,196479166
231637_at	LOC100499194	0,425036312	-0,771340337	1,196376649
222871_at	KLHDC8A	0,425036312	-0,771340337	1,196376649
242786_at	SBF2-AS1	0,677745787	-0,518232988	1,195978775
239774_at	-	0,677745787	-0,518232988	1,195978775
204399_s_at	EML2	1,561816652	0,366031214	1,195785439
226121_at	DHRS13	2,972517974	1,777135134	1,19538284
212774_at	ZNF238	3,147261986	1,953032682	1,194229303
225421_at	PM20D2	5,212245235	4,018230367	1,194014868
210592_s_at	SAT1	4,795549676	3,601940721	1,193608956
236311_at	LOH12CR2	-0,088974936	-1,282520722	1,193545787
1553350_at	FAM123C	-0,088974936	-1,282520722	1,193545787
241087_at	-	-0,088974936	-1,282520722	1,193545787
235257_at	ODF3B	-0,088974936	-1,282520722	1,193545787
1559533_at	-	-0,088974936	-1,282520722	1,193545787
1564207_at	FLJ35390	3,729417893	2,535878988	1,193538905
242579_at	BMPR1B	-0,856259172	-2,049747196	1,193488024
220422_at	UBQLN3	-0,856259172	-2,049747196	1,193488024
232766_at	BANF2	-0,856259172	-2,049747196	1,193488024
1552853_at	VWA5B1	-0,856259172	-2,049747196	1,193488024
1560489_at	-	-0,856259172	-2,049747196	1,193488024
214088_s_at	FUT3	-0,856259172	-2,049747196	1,193488024
244319_at	-	-0,856259172	-2,049747196	1,193488024
229599_at	LOC440335	-0,856259172	-2,049747196	1,193488024
217128_s_at	CAMK1G	-0,856259172	-2,049747196	1,193488024
230456_at	-	-0,856259172	-2,049747196	1,193488024
1565857_at	-	-0,856259172	-2,049747196	1,193488024
226067_at	BPIFB1	-0,856259172	-2,049747196	1,193488024
1562482_at	-	-0,856259172	-2,049747196	1,193488024
237140_x_at	-	-0,856259172	-2,049747196	1,193488024
237785_at	ASB14	-0,856259172	-2,049747196	1,193488024
211834_s_at	TP63	-0,856259172	-2,049747196	1,193488024

236932_s_at	GATAD2A	-0,856259172	-2,049747196	1,193488024
224762_at	SERINC2	-0,856259172	-2,049747196	1,193488024
1560149_at	SLC29A2	-0,856259172	-2,049747196	1,193488024
211668_s_at	PLAU	0,458905032	-0,734355396	1,193260427
239535_at	-	0,458905032	-0,734355396	1,193260427
202607_at	NDST1	0,458905032	-0,734355396	1,193260427
212428_at	KIAA0368	4,422748524	3,229534127	1,193214397
239273_s_at	MMP28	-0,66934516	-1,862269442	1,192924282
224458_at	TMEM246	-0,66934516	-1,862269442	1,192924282
208106_x_at	PSG6	-0,66934516	-1,862269442	1,192924282
232143_at	DNM1P41	-0,66934516	-1,862269442	1,192924282
1557400_at	-	-0,66934516	-1,862269442	1,192924282
231139_at	-	-0,66934516	-1,862269442	1,192924282
220596_at	GPATCH4	-0,66934516	-1,862269442	1,192924282
208592_s_at	CD1E	-0,66934516	-1,862269442	1,192924282
236279_at	-	-0,66934516	-1,862269442	1,192924282
235682_s_at	HSPB7	-0,66934516	-1,862269442	1,192924282
239961_at	-	-0,66934516	-1,862269442	1,192924282
1569536_at	FLVCR2	-0,66934516	-1,862269442	1,192924282
1556256_a_at	LOC100505908	-0,66934516	-1,862269442	1,192924282
212682_s_at	LMF2	2,636121902	1,44320939	1,192912512
203602_s_at	ZBTB17	2,771643417	1,578991006	1,192652412
203220_s_at	TLE1	-0,135065865	-1,32740143	1,192335565
213004_at	ANGPTL2	-0,135065865	-1,32740143	1,192335565
205714_s_at	ZMYND10	-0,135065865	-1,32740143	1,192335565
235583_at	ILDR1	-0,135065865	-1,32740143	1,192335565
220003_at	LRRC36	0,707657549	-0,484558493	1,192216042
224829_at	CPEB4	0,707657549	-0,484558493	1,192216042
215916_at	CHRNE	0,001114523	-1,19085694	1,191971463
242081_at	ACAP1	0,001114523	-1,19085694	1,191971463
239523_at	TUSC5	0,001114523	-1,19085694	1,191971463
216045_at	CCDC144A	-0,994186487	-2,186086603	1,191900116
205166_at	CAPN5	-0,994186487	-2,186086603	1,191900116
204895_x_at	MUC4	-0,994186487	-2,186086603	1,191900116
215432_at	ACSM1	-0,994186487	-2,186086603	1,191900116
228470_at	RNF187	-0,994186487	-2,186086603	1,191900116
230410_at	-	-0,994186487	-2,186086603	1,191900116
206712_at	GRTP1	-0,994186487	-2,186086603	1,191900116
240699_at	SEC14L3	-0,994186487	-2,186086603	1,191900116
210310_s_at	FGF5	-0,994186487	-2,186086603	1,191900116
224070_at	TAKR	-0,230432956	-1,422257026	1,191824071
234506_at	-	-0,230432956	-1,422257026	1,191824071
244529_at	-	-0,230432956	-1,422257026	1,191824071
230990_at	KIF13A	-0,230432956	-1,422257026	1,191824071
1557406_s_at	LOC100130111	-0,230432956	-1,422257026	1,191824071
224262_at	IL1F10	-0,230432956	-1,422257026	1,191824071
214401_at	PAX1	-0,230432956	-1,422257026	1,191824071
232025_at	SYT7	-0,230432956	-1,422257026	1,191824071
1562624_at	-	-0,230432956	-1,422257026	1,191824071
242987_x_at	LOC100506553	-0,230432956	-1,422257026	1,191824071

205617_at	PRRG2	-0,180990326	-1,372734086	1,19174376
234888_at	CACHD1	-0,180990326	-1,372734086	1,19174376
206622_at	TRH	-0,180990326	-1,372734086	1,19174376
206317_s_at	ABCB8	-0,180990326	-1,372734086	1,19174376
220965_s_at	RSPH6A	-0,180990326	-1,372734086	1,19174376
220398_at	GNPTAB	-0,180990326	-1,372734086	1,19174376
241398_at	-	-0,180990326	-1,372734086	1,19174376
207641_at	TNFRSF13B	-0,180990326	-1,372734086	1,19174376
1556584_at	-	-0,180990326	-1,372734086	1,19174376
214554_at	HIST1H2AG /// HIS	-0,280273599	-1,471833228	1,191559629
220217_x_at	SPANXC	-0,280273599	-1,471833228	1,191559629
1560911_at	LOC100133461	-0,280273599	-1,471833228	1,191559629
233794_at	-	-0,280273599	-1,471833228	1,191559629
213866_at	SAMD14	-0,280273599	-1,471833228	1,191559629
213731_s_at	TCF3	-0,280273599	-1,471833228	1,191559629
204606_at	CCL21	-0,280273599	-1,471833228	1,191559629
226595_at	SNX21	-0,280273599	-1,471833228	1,191559629
210318_at	RBP3	-0,280273599	-1,471833228	1,191559629
204214_s_at	RAB32	-0,280273599	-1,471833228	1,191559629
215063_x_at	LRRC40	1,857989965	0,666486137	1,191503828
238686_at	FBXO3	1,775035811	0,583607503	1,191428308
212651_at	RHOBTB1	-0,043925712	-1,234835326	1,190909614
211772_x_at	CHRNA3	-0,043925712	-1,234835326	1,190909614
206328_at	CDH15	-0,043925712	-1,234835326	1,190909614
229867_at	BTBD9	-0,043925712	-1,234835326	1,190909614
204336_s_at	RGS19	3,761702872	2,570802332	1,190900541
231184_at	DHX8	0,045517965	-1,145057014	1,190574979
214570_x_at	LOC100294020 ///	0,045517965	-1,145057014	1,190574979
216333_x_at	TNXA /// TNXB	0,045517965	-1,145057014	1,190574979
241990_at	RHOV	0,045517965	-1,145057014	1,190574979
203907_s_at	IQSEC1	2,25755351	1,067365565	1,190187945
231876_at	TRIM56	2,961244348	1,771157334	1,190087014
225619_at	SLAIN1	4,435374233	3,246064373	1,18930986
1565817_at	IKZF1	0,491906512	-0,697182596	1,189089108
205842_s_at	JAK2	1,5944803	0,405752839	1,188727461
214273_x_at	NPRL3	1,788922638	0,600385088	1,18853755
221511_x_at	CCPG1 /// DYX1C1	1,88491011	0,697221375	1,187688736
211652_s_at	LBP	-0,332405896	-1,520022123	1,187616227
1570065_at	-	-0,332405896	-1,520022123	1,187616227
230544_at	RPS6KA4	-0,332405896	-1,520022123	1,187616227
1553063_at	GPR78	-0,332405896	-1,520022123	1,187616227
206646_at	GLI1	-0,332405896	-1,520022123	1,187616227
242692_at	-	-0,332405896	-1,520022123	1,187616227
1564511_a_at	FSTL4	-0,332405896	-1,520022123	1,187616227
244440_at	-	-0,332405896	-1,520022123	1,187616227
213917_at	PAX8	-0,332405896	-1,520022123	1,187616227
1560035_at	CXXC11	-0,332405896	-1,520022123	1,187616227
227128_s_at	TACSTD2	-0,332405896	-1,520022123	1,187616227
216968_at	MASP2	-0,332405896	-1,520022123	1,187616227
203315_at	NCK2	4,784078567	3,596611499	1,187467068

213553_x_at	APOC1	0,735999505	-0,45121326	1,187212765
218524_at	E4F1	1,493647653	0,306454867	1,187192786
236565_s_at	LARP6	0,08619576	-1,100943374	1,187139134
206008_at	TGM1	0,08619576	-1,100943374	1,187139134
211831_s_at	THPO	0,08619576	-1,100943374	1,187139134
244481_at	-	0,08619576	-1,100943374	1,187139134
1554264_at	CKAP2	0,08619576	-1,100943374	1,187139134
1556739_at	GOLGA8IP	0,08619576	-1,100943374	1,187139134
240232_at	-	0,926325262	-0,260526297	1,186851559
204070_at	RARRES3	3,394274216	2,207440269	1,186833947
229590_at	RPL13 /// SNORD6	3,378744007	2,192329358	1,186414649
212308_at	CLASP2	1,364555912	0,178191865	1,186364047
224637_at	OST4	6,773742252	5,587424959	1,186317292
225663_at	ACBD5	4,357852544	3,171631256	1,186221289
205599_at	TRAF1	4,2463808	3,060577077	1,185803723
1554588_a_at	TTC30B	0,524545436	-0,661178575	1,185724011
226956_at	MTMR3	1,610353504	0,424693306	1,185660198
77508_r_at	RABEP2	1,307258264	0,121960385	1,185297879
215328_at	EFR3B	0,127732831	-1,057301851	1,185034681
219593_at	SLC15A3	0,127732831	-1,057301851	1,185034681
205987_at	CD1C	0,127732831	-1,057301851	1,185034681
214302_x_at	GJC2	0,127732831	-1,057301851	1,185034681
231875_at	KIF21A	1,802733148	0,61779983	1,184933318
208631_s_at	HADHA	5,69800487	4,51307582	1,18492905
218636_s_at	MAN1B1	2,836219929	1,651676651	1,184543278
206484_s_at	XPNPEP2	-0,384193355	-1,568385657	1,184192302
227130_s_at	TLE1	-0,384193355	-1,568385657	1,184192302
216869_at	PDE1C	-0,384193355	-1,568385657	1,184192302
204239_s_at	NNAT	-0,384193355	-1,568385657	1,184192302
216119_s_at	SPEF1	-0,384193355	-1,568385657	1,184192302
221017_s_at	LRRC3	-0,384193355	-1,568385657	1,184192302
230687_at	SLC13A3	-0,384193355	-1,568385657	1,184192302
239367_at	BDNF	-0,384193355	-1,568385657	1,184192302
201957_at	PPP1R12B	1,094118704	-0,089635713	1,183754417
211692_s_at	BBC3	1,094118704	-0,089635713	1,183754417
201482_at	QSOX1	1,910557155	0,727123109	1,183434046
232950_s_at	PITPNM2	0,95222556	-0,231045907	1,183271467
236695_at	STK4-AS1	0,95222556	-0,231045907	1,183271467
214491_at	SSTR3	0,95222556	-0,231045907	1,183271467
204611_s_at	PPP2R5B	2,179708195	0,996453274	1,183254921
225902_at	PPIG	2,179708195	0,996453274	1,183254921
203392_s_at	CTBP1	4,718186291	3,534956018	1,183230273
206906_at	ICAM5	0,167727503	-1,015398016	1,18312552
1560115_a_at	KIAA1217	0,167727503	-1,015398016	1,18312552
1558463_s_at	LMF1	0,167727503	-1,015398016	1,18312552
1562677_at	-	-1,452713826	-2,635549085	1,182835259
1555711_x_at	-	-1,452713826	-2,635549085	1,182835259
1564786_at	LOC338667	-1,452713826	-2,635549085	1,182835259
209269_s_at	SYK	-1,452713826	-2,635549085	1,182835259
238649_at	PITPNC1	0,764143511	-0,418248858	1,182392369

214072_x_at	NENF	0,764143511	-0,418248858	1,182392369
229568_at	MOB3B	-0,730013898	-1,911206569	1,181192671
216956_s_at	ITGA2B	-0,730013898	-1,911206569	1,181192671
244167_at	-	-0,730013898	-1,911206569	1,181192671
233885_at	ARHGAP33	-0,730013898	-1,911206569	1,181192671
206575_at	CDKL5	-0,730013898	-1,911206569	1,181192671
204541_at	SEC14L2	-0,730013898	-1,911206569	1,181192671
231229_at	HILS1	-0,730013898	-1,911206569	1,181192671
203430_at	HEBP2	5,634172732	4,453586789	1,180585943
226639_at	SFT2D3	3,185443259	2,004917973	1,180525287
213676_at	TMEM151B	0,20740896	-0,972893339	1,180302299
213556_at	LOC390940	0,20740896	-0,972893339	1,180302299
207876_s_at	FLNC	0,20740896	-0,972893339	1,180302299
203783_x_at	POLRMT	0,20740896	-0,972893339	1,180302299
213499_at	CLCN2	0,20740896	-0,972893339	1,180302299
212283_at	AGRN	0,20740896	-0,972893339	1,180302299
226657_at	C17orf103	0,20740896	-0,972893339	1,180302299
223733_s_at	PPP4R1L	0,20740896	-0,972893339	1,180302299
223806_s_at	NAPSA	0,20740896	-0,972893339	1,180302299
218067_s_at	ARGLU1	5,296107648	4,116251724	1,179855924
1569895_at	LOC100133612	1,116462765	-0,063377083	1,179839848
212891_s_at	GADD45GIP1	3,517785666	2,338020736	1,17976493
208058_s_at	MGAT3	0,556428218	-0,623254098	1,179682316
200603_at	PRKAR1A	6,464236781	5,28465842	1,179578361
1560207_at	RAD21-AS1	-0,437449947	-1,616924751	1,179474804
210199_at	CRYAA	-0,437449947	-1,616924751	1,179474804
233256_at	-	-0,437449947	-1,616924751	1,179474804
243444_at	SRD5A3	-0,437449947	-1,616924751	1,179474804
235332_at	FAM22A /// FAM2	-0,437449947	-1,616924751	1,179474804
201585_s_at	SFPQ	4,705123614	3,525687129	1,179436485
218954_s_at	BRF2	2,199941239	1,020514937	1,179426302
230506_at	C6orf164	-1,212652659	-2,391863529	1,17921087
1559252_a_at	-	-1,212652659	-2,391863529	1,17921087
204921_at	GAS8	1,544940605	0,366031214	1,178909391
216647_at	TCF3	0,791908897	-0,386896102	1,178804999
235659_at	-	0,791908897	-0,386896102	1,178804999
213755_s_at	-	0,791908897	-0,386896102	1,178804999
223190_s_at	MLL5	4,230697503	3,052211048	1,178486455
213223_at	RPL28	3,389999385	2,212304392	1,177694992
215714_s_at	SMARCA4	2,732422196	1,555165465	1,177256731
222856_at	APLN	0,58788177	-0,589001171	1,176882941
229891_x_at	KIAA1704	2,836219929	1,659532492	1,176687437
222129_at	FAM134A	2,636121902	1,459545271	1,176576631
221066_at	RXFP3	-1,289805289	-2,466349414	1,176544125
232657_at	-	-1,289805289	-2,466349414	1,176544125
241284_at	-	-1,289805289	-2,466349414	1,176544125
242089_at	-	-1,289805289	-2,466349414	1,176544125
238376_at	LOC100505564	-1,289805289	-2,466349414	1,176544125
214922_at	ZNF484	-1,289805289	-2,466349414	1,176544125
236894_at	L1TD1	-1,289805289	-2,466349414	1,176544125

209987_s_at	ASCL1	-1,289805289	-2,466349414	1,176544125
1559642_a_at	-	-1,289805289	-2,466349414	1,176544125
207422_at	ADAM20	0,244681185	-0,931539147	1,176220332
224495_at	TMEM107	0,244681185	-0,931539147	1,176220332
219752_at	RASAL1	0,244681185	-0,931539147	1,176220332
230374_at	-	0,244681185	-0,931539147	1,176220332
204213_at	PIGR	0,244681185	-0,931539147	1,176220332
211406_at	IER3IP1	2,575362572	1,399283506	1,176079066
231713_s_at	ELP2	3,876052392	2,700070732	1,17598166
202697_at	NUDT21	4,913150425	3,737342734	1,175807691
214679_x_at	GNA11	1,139057614	-0,036543687	1,175601301
211252_x_at	PTCRA	1,139057614	-0,036543687	1,175601301
243529_at	MARS2	2,085817135	0,910483921	1,175333214
227430_at	ZC3H10	0,820160788	-0,354781582	1,174942369
1554967_at	DIP2A	-1,137208348	-2,312054429	1,174846081
237380_at	-	-1,137208348	-2,312054429	1,174846081
234826_at	-	-1,137208348	-2,312054429	1,174846081
243446_at	AJUBA	-1,137208348	-2,312054429	1,174846081
1563822_at	LOC100131763	-1,137208348	-2,312054429	1,174846081
1558769_s_at	DNAH1	-1,137208348	-2,312054429	1,174846081
244668_at	-	-1,137208348	-2,312054429	1,174846081
239356_at	DOCK9-AS2	-1,137208348	-2,312054429	1,174846081
1553157_at	LHX4	-1,137208348	-2,312054429	1,174846081
1553079_at	TRIM40	-1,137208348	-2,312054429	1,174846081
1569926_s_at	SLC34A3	-1,137208348	-2,312054429	1,174846081
201143_s_at	EIF2S1	4,16769828	2,993331554	1,174366726
218664_at	MECR	2,452674045	1,278654284	1,174019762
224744_at	IMPAD1	2,937809196	1,76396664	1,173842557
202174_s_at	PCM1	5,294944875	4,121221299	1,173723576
212001_at	SUGP2	2,096248267	0,923203038	1,173045229
1560460_at	-	-0,49284695	-1,665791776	1,172944826
223739_at	PADI1	-0,49284695	-1,665791776	1,172944826
1563654_at	C8orf66	-0,49284695	-1,665791776	1,172944826
236631_at	LINC00319	-0,49284695	-1,665791776	1,172944826
206321_at	RFX1	-0,49284695	-1,665791776	1,172944826
221454_at	-	-0,49284695	-1,665791776	1,172944826
233416_at	-	-0,49284695	-1,665791776	1,172944826
215815_at	BUD31	-0,49284695	-1,665791776	1,172944826
203549_s_at	LPL	-0,49284695	-1,665791776	1,172944826
220593_s_at	CCDC40	-0,49284695	-1,665791776	1,172944826
217495_x_at	CALCA	-0,49284695	-1,665791776	1,172944826
222882_s_at	C17orf59	-0,49284695	-1,665791776	1,172944826
1566301_at	PPP1R11	-0,924000698	-2,09678811	1,172787412
220222_at	RBM12B-AS1	-0,924000698	-2,09678811	1,172787412
223687_s_at	LY6K	-0,924000698	-2,09678811	1,172787412
204996_s_at	CDK5R1	-0,924000698	-2,09678811	1,172787412
216501_at	VAC14	-0,924000698	-2,09678811	1,172787412
220644_at	-	-0,924000698	-2,09678811	1,172787412
241636_x_at	-	-0,924000698	-2,09678811	1,172787412
237523_at	-	-0,924000698	-2,09678811	1,172787412

208312_s_at	PRAMEF1 /// PRAI	-0,924000698	-2,09678811	1,172787412
239255_at	-	-0,924000698	-2,09678811	1,172787412
202357_s_at	CFB	-0,924000698	-2,09678811	1,172787412
231052_at	GLOD5	-0,924000698	-2,09678811	1,172787412
1554805_at	CLDN19	-0,924000698	-2,09678811	1,172787412
210553_x_at	LOC100507472 ///	-0,924000698	-2,09678811	1,172787412
1563235_at	-	-0,924000698	-2,09678811	1,172787412
210081_at	AGER	-0,924000698	-2,09678811	1,172787412
219588_s_at	NCAPG2	4,969542968	3,796883855	1,172659113
238990_x_at	TRIM61	1,000459215	-0,172194225	1,172653439
204374_s_at	GALK1	1,000459215	-0,172194225	1,172653439
211504_x_at	ROCK2	1,000459215	-0,172194225	1,172653439
216434_at	TTC38	1,160996507	-0,011001236	1,171997743
216320_x_at	MST1	1,458255986	0,286279868	1,171976119
228097_at	MYLIP	0,282358733	-0,889222211	1,171580944
205387_s_at	CGB /// CGB5 /// C	0,282358733	-0,889222211	1,171580944
209648_x_at	SOCS5	1,686984721	0,515457602	1,171527119
213152_s_at	SRSF8	2,99543179	1,823960141	1,171471649
31846_at	RHOD	3,092498026	1,921331294	1,171166733
225957_at	CREBRF	1,898032047	0,727123109	1,170908938
1552528_at	C21orf67	1,326463531	0,155747281	1,17071625
220964_s_at	RAB1B	4,155757614	2,98507484	1,170682774
228535_at	RAD1	2,605420261	1,434743608	1,170676652
228862_at	TSNARE1	0,847529938	-0,322340048	1,169869986
208390_s_at	GLP1R	0,847529938	-0,322340048	1,169869986
1553992_s_at	NBR2	0,847529938	-0,322340048	1,169869986
1569409_x_at	-	0,847529938	-0,322340048	1,169869986
209940_at	PARP3	1,5944803	0,424693306	1,169786994
43977_at	TMEM161A	3,298099562	2,128666848	1,169432713
210968_s_at	RTN4	6,343295352	5,173898758	1,169396594
212708_at	MSL1	4,087244545	2,918087241	1,169157304
207408_at	SLC22A14	1,024284941	-0,144765583	1,169050524
212940_at	COL6A1	0,319287178	-0,84928999	1,168577168
220743_at	-	0,319287178	-0,84928999	1,168577168
225343_at	TMED8	5,499561841	4,331027554	1,168534287
216481_at	GRIP2	0,355751005	-0,811769547	1,167520552
229677_at	SLC39A3	0,355751005	-0,811769547	1,167520552
228943_at	MAP6	0,355751005	-0,811769547	1,167520552
211135_x_at	LILRA6 /// LILRB3	0,355751005	-0,811769547	1,167520552
202996_at	POLD4	3,029186714	1,861868165	1,16731855
202830_s_at	SLC37A4	2,804738618	1,637482551	1,167256067
222514_at	RRAGC	3,450420588	2,283169614	1,167250974
219484_at	HCFC2	2,138605583	0,971607464	1,166998119
210277_at	AP4S1	1,816938997	0,65030602	1,166632977
235664_at	-	-1,369772723	-2,536339282	1,166566559
1552814_a_at	KLF14	-1,369772723	-2,536339282	1,166566559
204910_s_at	TRIM3	-1,369772723	-2,536339282	1,166566559
205106_at	MTCP1 /// MTCP1	0,648195588	-0,518232988	1,166428576
1562678_at	-	-1,06282519	-2,229233437	1,166408248
237442_at	-	-1,06282519	-2,229233437	1,166408248

1555912_at	ST7-AS1	-1,06282519	-2,229233437	1,166408248
206939_at	DCC	-1,06282519	-2,229233437	1,166408248
233811_at	RIN2	-1,06282519	-2,229233437	1,166408248
237200_at	-	-1,06282519	-2,229233437	1,166408248
1570354_s_at	ZNF169	-1,06282519	-2,229233437	1,166408248
242501_at	-	-1,06282519	-2,229233437	1,166408248
1562895_at	-	-1,06282519	-2,229233437	1,166408248
211146_at	-	-1,06282519	-2,229233437	1,166408248
207378_at	TREH	-1,06282519	-2,229233437	1,166408248
240213_at	-	-1,06282519	-2,229233437	1,166408248
202485_s_at	MBD2	-1,06282519	-2,229233437	1,166408248
242832_at	PER1	-1,06282519	-2,229233437	1,166408248
204803_s_at	RRAD	-1,06282519	-2,229233437	1,166408248
212900_at	SEC24A	4,413844848	3,248126831	1,165718017
242403_at	-	2,989672611	1,823960141	1,16571247
226981_at	MLL	3,751261994	2,585619776	1,165642218
227982_at	SEPSECS	1,92317941	0,757638486	1,165540924
207762_at	LPAL2	-0,791294935	-1,956681069	1,165386134
1565406_a_at	LHX9	-0,791294935	-1,956681069	1,165386134
228877_at	RGL3	-0,791294935	-1,956681069	1,165386134
217447_at	MAG	-0,791294935	-1,956681069	1,165386134
237570_x_at	-	-0,791294935	-1,956681069	1,165386134
217439_at	-	-0,791294935	-1,956681069	1,165386134
215709_at	LOC100653079 ///	-0,791294935	-1,956681069	1,165386134
231046_at	HP07349 /// LOC1(	-0,791294935	-1,956681069	1,165386134
206517_at	CDH16	-0,791294935	-1,956681069	1,165386134
224062_x_at	KLK4	-0,791294935	-1,956681069	1,165386134
230208_at	HCN4	-0,791294935	-1,956681069	1,165386134
1553115_at	NKD1	-0,791294935	-1,956681069	1,165386134
210587_at	INHBE	-0,791294935	-1,956681069	1,165386134
225570_at	SLC41A1	2,007220798	0,842599219	1,164621579
201201_at	CSTB	6,040048444	4,875621102	1,164427342
236113_at	-	-0,551284523	-1,715576125	1,164291602
228874_at	PHLDB3	-0,551284523	-1,715576125	1,164291602
220480_at	HAND2	-0,551284523	-1,715576125	1,164291602
205960_at	PDK4	-0,551284523	-1,715576125	1,164291602
232199_at	-	-0,551284523	-1,715576125	1,164291602
234520_at	-	-0,551284523	-1,715576125	1,164291602
1555082_a_at	NEK11	-0,551284523	-1,715576125	1,164291602
234721_s_at	CYP26B1	-0,551284523	-1,715576125	1,164291602
239139_at	CPNE9	-0,551284523	-1,715576125	1,164291602
215251_at	-	-0,551284523	-1,715576125	1,164291602
1565734_at	-	-0,551284523	-1,715576125	1,164291602
242411_at	ARL10	-0,551284523	-1,715576125	1,164291602
232966_at	LPIN3	-0,551284523	-1,715576125	1,164291602
215675_at	-	-0,551284523	-1,715576125	1,164291602
241980_at	MAP6	-0,551284523	-1,715576125	1,164291602
240595_at	-	-0,551284523	-1,715576125	1,164291602
227365_at	ATCAY	-0,551284523	-1,715576125	1,164291602
212741_at	MAOA	-0,551284523	-1,715576125	1,164291602

204648_at	NPR1	1,047341799	-0,116767475	1,164109273
238179_at	LOC100506268	1,625482993	0,461385738	1,164097256
203080_s_at	BAZ2B	1,364555912	0,20062106	1,163934852
202447_at	DECR1	5,132261918	3,968349836	1,163912082
210312_s_at	IFT20	4,66447449	3,500705275	1,163769215
203515_s_at	PMVK	3,691400566	2,527778509	1,163622057
235798_at	TMEM170B	2,51558601	1,352622437	1,162963573
230729_at	-	0,39105295	-0,771340337	1,162393287
210192_at	ATP8A1	0,39105295	-0,771340337	1,162393287
221270_s_at	QTRT1	1,528073958	0,366031214	1,162042745
208764_s_at	ATP5G2	6,822618184	5,660606387	1,162011797
225232_at	MTMR12	3,720319513	2,558652146	1,161667367
218945_at	METTL22	2,339581693	1,178100917	1,161480776
1558522_at	-	1,383446753	0,222432814	1,161013939
203891_s_at	DAPK3	1,246401134	0,085534992	1,160866141
203943_at	KIF3B	2,937809196	1,777135134	1,160674063
217990_at	GMPR2	3,67542249	2,514786608	1,160635881
239435_x_at	SHROOM1	0,900065578	-0,260526297	1,160591875
214800_x_at	BTF3	7,059492058	5,898959934	1,160532125
212049_at	WIPF2	2,791258167	1,630734622	1,160523545
209434_s_at	PPAT	3,362258367	2,20251271	1,159745658
226195_at	IFT43	2,56794744	1,408220608	1,159726832
212155_at	RNF187	4,693076946	3,533373095	1,159703851
210300_at	REM1	0,425036312	-0,734355396	1,159391708
218849_s_at	PPP1R13L	0,425036312	-0,734355396	1,159391708
1563302_at	-	0,425036312	-0,734355396	1,159391708
206875_s_at	SLK	2,99543179	1,836262316	1,159169473
211168_s_at	UPF1	2,427650693	1,268569037	1,159081656
211635_x_at	IGHA1 /// IGHA2 /,	0,707657549	-0,45121326	1,158870808
214122_at	PDLIM7	0,707657549	-0,45121326	1,158870808
215499_at	MAP2K3	3,748268542	2,589579418	1,158689124
214246_x_at	MINK1	3,076375717	1,917743773	1,158631944
225930_at	NKIRAS1	2,649978302	1,491644074	1,158334229
204344_s_at	SEC23A	1,65554524	0,497424714	1,158120525
222466_s_at	MRPL42	3,993377193	2,835336991	1,158040201
234715_at	GOLGA2P2Y /// GC	-1,537851782	-2,695640852	1,15778907
208141_s_at	DOHH	1,88491011	0,727123109	1,157787002
34846_at	CAMK2B	1,559286101	0,401899213	1,157386887
222602_at	UBA6	3,822956033	2,665727652	1,157228381
225245_x_at	H2AFJ	4,087244545	2,930238085	1,15700646
205548_s_at	BTG3	4,155757614	2,999351239	1,156406376
214327_x_at	TPT1	7,807583456	6,651394102	1,156189354
206672_at	AQP2	0,458905032	-0,697182596	1,156087628
202258_s_at	N4BP2L2	4,574442657	3,41869868	1,155743976
212418_at	ELF1	4,666256427	3,510668356	1,15558807
1553957_at	ZNF564	1,898032047	0,742671819	1,155360228
203391_at	FKBP2	3,798440091	2,64347734	1,15496275
224741_x_at	GAS5 /// SNORD44	6,872008502	5,717118836	1,154889666
212773_s_at	TOMM20	6,38059034	5,225781968	1,154808372
227009_at	-	1,287793882	0,133104519	1,154689363

203051_at	BAHD1	1,287793882	0,133104519	1,154689363
220448_at	KCNK12	-0,609040214	-1,763664074	1,15462386
213674_x_at	IGHD	-0,609040214	-1,763664074	1,15462386
240289_at	PDLIM3	-0,609040214	-1,763664074	1,15462386
212524_x_at	H2AFX	-0,609040214	-1,763664074	1,15462386
1556581_at	ZNF778	-0,609040214	-1,763664074	1,15462386
216727_at	STK38	-0,609040214	-1,763664074	1,15462386
217652_at	MAU2	-0,609040214	-1,763664074	1,15462386
207658_s_at	FOXG1	-0,609040214	-1,763664074	1,15462386
232719_at	CELF4	-0,609040214	-1,763664074	1,15462386
207269_at	DEFA4	-0,609040214	-1,763664074	1,15462386
229172_at	HSPA12B	-0,609040214	-1,763664074	1,15462386
235832_at	NKX6-2	-0,609040214	-1,763664074	1,15462386
211100_x_at	LILRA2	-0,609040214	-1,763664074	1,15462386
243493_at	-	-0,609040214	-1,763664074	1,15462386
228065_at	BCL9L	-0,609040214	-1,763664074	1,15462386
217515_s_at	CACNA1S	-0,609040214	-1,763664074	1,15462386
220209_at	PYY2	-0,609040214	-1,763664074	1,15462386
210689_at	CLDN14	-0,609040214	-1,763664074	1,15462386
225104_at	ZNF598	2,267141818	1,112548615	1,154593203
218789_s_at	C11orf71	2,452674045	1,298349073	1,154324972
238746_at	PXMP4	0,735999505	-0,418248858	1,154248363
208255_s_at	FKBP8	0,735999505	-0,418248858	1,154248363
224015_s_at	MRPS25	1,578719607	0,424693306	1,154026301
235399_at	-	1,578719607	0,424693306	1,154026301
212803_at	NAB2	0,95222556	-0,201789721	1,154015281
239824_s_at	TMEM107	3,867546929	2,713775735	1,153771194
212737_at	GM2A	3,807915835	2,654387125	1,15352871
220553_s_at	PRPF39	3,370643409	2,2171284	1,153515009
202080_s_at	TRAK1	3,03433073	1,881145546	1,153185183
216988_s_at	PTP4A2	5,837570158	4,684465192	1,153104967
1560587_s_at	PRDX5	6,196847579	5,043770318	1,153077261
217871_s_at	MIF	7,71230311	6,55925063	1,15305248
224808_s_at	GET4	1,995527416	0,842599219	1,152928197
216640_s_at	PDIA6	6,716628729	5,563990112	1,152638617
228832_at	FLJ20021	1,701542258	0,550017041	1,151525217
235799_at	NSL1	0,764143511	-0,386896102	1,151039613
200065_s_at	ARF1 /// MIR3620	6,515372034	5,364508945	1,150863089
228853_at	STYX	2,229377375	1,078619386	1,150757989
1563012_x_at	-	-1,452713826	-2,603471267	1,150757441
1557402_at	-	-1,452713826	-2,603471267	1,150757441
216898_s_at	COL4A3	-1,452713826	-2,603471267	1,150757441
233494_at	ERBB4	-1,452713826	-2,603471267	1,150757441
225518_x_at	MTHFD1L	-1,452713826	-2,603471267	1,150757441
208341_x_at	CSH2	-1,452713826	-2,603471267	1,150757441
240085_at	-	-1,452713826	-2,603471267	1,150757441
224542_s_at	NFATC2	-1,452713826	-2,603471267	1,150757441
218449_at	UFSP2	3,050461597	1,899778623	1,150682974
227167_s_at	RASSF3	4,346015214	3,195836661	1,150178553
202304_at	FNDC3A	3,771154436	2,621003244	1,150151192

226644_at	MIB2	1,139057614	-0,011001236	1,15005885
37950_at	PREP	3,553449752	2,40350368	1,149946072
203882_at	IRF9	3,171328694	2,021531611	1,149797084
210980_s_at	ASAH1	4,070902947	2,921154239	1,149748708
225550_at	CNST	2,085817135	0,936232181	1,149584953
223112_s_at	NDUFB10	6,33719734	5,18829749	1,14889985
1560285_at	-	-0,856259172	-2,005029581	1,148770409
232782_at	-	-0,856259172	-2,005029581	1,148770409
205678_at	AP3B2	-0,856259172	-2,005029581	1,148770409
220818_s_at	TRPC4	-0,856259172	-2,005029581	1,148770409
205473_at	ATP6V1B1	-0,856259172	-2,005029581	1,148770409
1562549_at	-	-0,856259172	-2,005029581	1,148770409
237036_at	FBXO10	-0,856259172	-2,005029581	1,148770409
237318_at	-	-0,856259172	-2,005029581	1,148770409
1554469_at	ZBTB44	-0,856259172	-2,005029581	1,148770409
238351_x_at	-	-0,856259172	-2,005029581	1,148770409
223049_at	GRB2	4,31266827	3,163941505	1,148726765
219842_at	ARL15	-0,994186487	-2,142286822	1,148100335
208028_s_at	GPX5	-0,994186487	-2,142286822	1,148100335
238304_at	-	-0,994186487	-2,142286822	1,148100335
216401_x_at	-	-0,994186487	-2,142286822	1,148100335
210015_s_at	MAP2	-0,994186487	-2,142286822	1,148100335
230857_s_at	ZNF497	-0,994186487	-2,142286822	1,148100335
206647_at	HBZ	-0,994186487	-2,142286822	1,148100335
1562222_at	-	-0,994186487	-2,142286822	1,148100335
211398_at	FGFR2	-0,994186487	-2,142286822	1,148100335
238621_at	FMN1 /// LOC1006	-0,994186487	-2,142286822	1,148100335
223600_s_at	KIAA1683	-0,994186487	-2,142286822	1,148100335
215552_s_at	ESR1	-0,994186487	-2,142286822	1,148100335
230445_at	BTBD17	-0,994186487	-2,142286822	1,148100335
1562764_at	LOC100507351	-0,994186487	-2,142286822	1,148100335
234898_at	-	-0,994186487	-2,142286822	1,148100335
222899_at	ITGA11	-0,994186487	-2,142286822	1,148100335
1555871_at	TTC28-AS1	-0,994186487	-2,142286822	1,148100335
228779_at	LOC146880	-0,994186487	-2,142286822	1,148100335
241697_at	-	-0,994186487	-2,142286822	1,148100335
215377_at	CTBP2	-0,994186487	-2,142286822	1,148100335
206394_at	MYBPC2	-0,994186487	-2,142286822	1,148100335
225435_at	SSR1	3,533714224	2,385765892	1,147948332
212821_at	PLEKHG3	0,524545436	-0,623254098	1,147799535
205265_s_at	SPEG	0,524545436	-0,623254098	1,147799535
222275_at	MRPS30	2,031158928	0,883374248	1,147784679
231497_at	ZBTB20-AS1	-0,135065865	-1,282520722	1,147454857
210925_at	CIITA	-0,135065865	-1,282520722	1,147454857
232698_at	BPIFB2	-0,135065865	-1,282520722	1,147454857
227457_at	DDA1	-0,135065865	-1,282520722	1,147454857
211111_at	HGC6.3	-0,135065865	-1,282520722	1,147454857
1555713_at	PNKD	-0,135065865	-1,282520722	1,147454857
232462_s_at	A1BG-AS1	-0,135065865	-1,282520722	1,147454857
206128_at	ADRA2C	-0,135065865	-1,282520722	1,147454857

1559672_a_at	CCDC171	-0,043925712	-1,19085694	1,146931228
232043_at	GNG7	-0,043925712	-1,19085694	1,146931228
1563903_x_at	-	-0,043925712	-1,19085694	1,146931228
217476_at	NR1D1 /// THRA	-0,043925712	-1,19085694	1,146931228
210030_at	-	-0,043925712	-1,19085694	1,146931228
205754_at	F2	-0,043925712	-1,19085694	1,146931228
214663_at	DSTYK	-0,043925712	-1,19085694	1,146931228
1552671_a_at	SLC9A7	-0,043925712	-1,19085694	1,146931228
206539_s_at	CYP4F12	-0,043925712	-1,19085694	1,146931228
220536_at	VRTN	-0,043925712	-1,19085694	1,146931228
212878_s_at	KLC1	3,936891546	2,790111983	1,146779563
218752_at	ZMAT5	2,118167848	0,971607464	1,146560384
229721_x_at	DERL3	0,045517965	-1,100943374	1,146461339
238322_s_at	TEAD2	0,045517965	-1,100943374	1,146461339
222173_s_at	TBC1D2	0,045517965	-1,100943374	1,146461339
216023_at	KDM4B	0,045517965	-1,100943374	1,146461339
221795_at	NTRK2	0,045517965	-1,100943374	1,146461339
216695_s_at	TNKS	-0,180990326	-1,32740143	1,146411104
231922_at	ZNF276	-0,180990326	-1,32740143	1,146411104
214400_at	INSL3	-0,180990326	-1,32740143	1,146411104
214084_x_at	NCF1C	-0,180990326	-1,32740143	1,146411104
234367_x_at	TMPRSS6	-0,180990326	-1,32740143	1,146411104
234265_at	PPP1R12C	-0,180990326	-1,32740143	1,146411104
233454_at	POLN	-0,180990326	-1,32740143	1,146411104
1569095_at	LOC731424	-0,180990326	-1,32740143	1,146411104
217691_x_at	SLC16A3	-0,180990326	-1,32740143	1,146411104
204174_at	ALOX5AP	6,858067149	5,711720386	1,146346763
235282_at	LOC100506295	2,732422196	1,586121802	1,146300395
228626_at	URM1	0,001114523	-1,145057014	1,146171537
204356_at	LIMK1	0,001114523	-1,145057014	1,146171537
240919_at	-	0,001114523	-1,145057014	1,146171537
209552_at	PAX8	0,001114523	-1,145057014	1,146171537
233266_at	-	0,001114523	-1,145057014	1,146171537
240692_at	MGC34796	0,001114523	-1,145057014	1,146171537
1563772_a_at	LAMA3	-0,088974936	-1,234835326	1,145860391
220971_at	IL25	-0,088974936	-1,234835326	1,145860391
227886_at	IFITM10	-0,088974936	-1,234835326	1,145860391
217395_at	MT4	-0,088974936	-1,234835326	1,145860391
227339_at	RGMB	-0,088974936	-1,234835326	1,145860391
226571_s_at	PTPRS	0,556428218	-0,589001171	1,145429389
237532_at	-	1,000459215	-0,144765583	1,145224798
202147_s_at	IFRD1	3,57383031	2,428719338	1,145110972
222548_s_at	MAP4K4	2,312738346	1,167919222	1,144819124
219281_at	MSRA	2,552836808	1,408220608	1,1446162
212666_at	SMURF1	2,127965541	0,98381571	1,144149831
202722_s_at	GFPT1	3,529682801	2,385765892	1,143916909
1555263_at	-	-1,712732543	-2,856570137	1,143837594
210576_at	CYP4F8	-1,712732543	-2,856570137	1,143837594
215823_x_at	PABPC1 /// RLIM	7,671807879	6,528148628	1,143659251
211473_s_at	COL4A6	0,08619576	-1,057301851	1,143497611

210803_at	TXNRD2	0,08619576	-1,057301851	1,143497611
208603_s_at	MAPK8IP2	0,08619576	-1,057301851	1,143497611
208953_at	LARP4B	2,872644433	1,72935563	1,143288803
210795_s_at	-	0,127732831	-1,015398016	1,143130847
229737_at	FAM46A	0,127732831	-1,015398016	1,143130847
240780_at	-	0,127732831	-1,015398016	1,143130847
216694_at	-	0,127732831	-1,015398016	1,143130847
230077_at	LOC220729 /// SDI	3,426291319	2,283169614	1,143121705
1554007_at	-	0,58788177	-0,554696666	1,142578436
230120_s_at	PLGLB1 /// PLGLB2	0,820160788	-0,322340048	1,142500836
207868_at	CHRNA2	0,820160788	-0,322340048	1,142500836
179_at	DTX2P1-UPK3BP1-	2,688123115	1,545674989	1,142448126
210960_at	ADRA1D	-0,66934516	-1,811780055	1,142434895
216376_x_at	-	-0,66934516	-1,811780055	1,142434895
211057_at	ROR1	-0,66934516	-1,811780055	1,142434895
232519_at	NSFL1C	-0,66934516	-1,811780055	1,142434895
239613_at	-	-0,66934516	-1,811780055	1,142434895
202551_s_at	CRIM1	-0,66934516	-1,811780055	1,142434895
1562264_at	LOC339685	-0,66934516	-1,811780055	1,142434895
220423_at	PLA2G2D	-0,66934516	-1,811780055	1,142434895
220795_s_at	BEGAIN	-0,66934516	-1,811780055	1,142434895
234364_at	CKAP2	-0,66934516	-1,811780055	1,142434895
229381_at	C1orf64	-0,66934516	-1,811780055	1,142434895
1564868_a_at	FAM117B	-0,66934516	-1,811780055	1,142434895
228212_at	ISM2	-0,66934516	-1,811780055	1,142434895
230181_at	PQLC1	-0,66934516	-1,811780055	1,142434895
1569792_a_at	METTL20	-0,230432956	-1,372734086	1,14230113
233020_at	-	-0,230432956	-1,372734086	1,14230113
240436_at	MIPEPP3	-0,230432956	-1,372734086	1,14230113
1558256_at	LOC148189	-0,230432956	-1,372734086	1,14230113
212701_at	TLN2	-0,230432956	-1,372734086	1,14230113
213900_at	FAM189A2	-0,230432956	-1,372734086	1,14230113
214425_at	AMBP	-0,230432956	-1,372734086	1,14230113
242921_at	SGSM1	-0,230432956	-1,372734086	1,14230113
215869_at	-	-0,230432956	-1,372734086	1,14230113
213997_at	FAM189A1	-0,230432956	-1,372734086	1,14230113
203658_at	SLC25A20	3,912706782	2,770696222	1,14201056
238696_at	-	-0,280273599	-1,422257026	1,141983427
237799_at	SLC22A12	-0,280273599	-1,422257026	1,141983427
233474_at	LOC284240	-0,280273599	-1,422257026	1,141983427
234573_at	-	-0,280273599	-1,422257026	1,141983427
221226_s_at	ASIC4	-0,280273599	-1,422257026	1,141983427
223371_s_at	DNAJC4	-0,280273599	-1,422257026	1,141983427
205785_at	ITGAM	-0,280273599	-1,422257026	1,141983427
215119_at	MYO16	-0,280273599	-1,422257026	1,141983427
211336_x_at	LILRB1	-0,280273599	-1,422257026	1,141983427
225820_at	PHF17	2,33082239	1,189078962	1,141743428
227767_at	CSNK1G3	3,544559557	2,403191094	1,141368463
211025_x_at	COX5B	6,216679593	5,075579242	1,141100351
209748_at	SPAST	4,089697574	2,948635566	1,141062008

225191_at	CIRBP	1,024284941	-0,116767475	1,141052416
229894_s_at	RAB43	0,167727503	-0,972893339	1,140620842
1555198_x_at	C21orf58	0,167727503	-0,972893339	1,140620842
217264_s_at	SCNN1A	0,167727503	-0,972893339	1,140620842
1565662_at	-	0,167727503	-0,972893339	1,140620842
222000_at	C1orf174	4,870498291	3,730373954	1,140124337
221692_s_at	MRPL34	4,537242248	3,39740877	1,139833479
205628_at	PRIM2	2,791258167	1,651676651	1,139581516
222112_at	EPS15L1	-0,332405896	-1,471833228	1,139427332
211920_at	CFB	-0,332405896	-1,471833228	1,139427332
211413_s_at	PADI4	-0,332405896	-1,471833228	1,139427332
211712_s_at	ANXA9	-0,332405896	-1,471833228	1,139427332
209962_at	EPOR	-0,332405896	-1,471833228	1,139427332
208594_x_at	LILRA6	-0,332405896	-1,471833228	1,139427332
234707_x_at	IGLV1-44	-0,332405896	-1,471833228	1,139427332
210720_s_at	NECAB3	1,544940605	0,405752839	1,139187766
1558685_a_at	LOC158960	3,649735397	2,5107204	1,139014997
221314_at	GDF9	0,20740896	-0,931539147	1,138948107
214104_at	GPR161	0,20740896	-0,931539147	1,138948107
1564897_at	TNR	0,20740896	-0,931539147	1,138948107
234644_x_at	TNFRSF10C	0,20740896	-0,931539147	1,138948107
221882_s_at	TMEM8A	1,383446753	0,244646697	1,138800055
224915_x_at	ZNFX1-AS1	6,622777271	5,484104366	1,138672905
1566764_at	MACC1	-1,289805289	-2,4284334	1,138628111
1554742_at	PMS1	-1,289805289	-2,4284334	1,138628111
220388_at	FER1L4	-1,289805289	-2,4284334	1,138628111
222079_at	ERG	-1,289805289	-2,4284334	1,138628111
208253_at	SIGLEC8	-1,289805289	-2,4284334	1,138628111
1553009_s_at	REXO1L1	-1,289805289	-2,4284334	1,138628111
207514_s_at	GNAT1	-1,289805289	-2,4284334	1,138628111
209513_s_at	HSDL2	5,018947904	3,880345837	1,138602067
217717_s_at	YWHAB	6,101407175	4,963197954	1,138209221
229317_at	KPNA5	1,671397663	0,533258442	1,138139221
1552708_a_at	DUSP19	-1,212652659	-2,350444759	1,1377921
240490_at	-	-1,212652659	-2,350444759	1,1377921
1555291_at	TRPV3	-1,212652659	-2,350444759	1,1377921
217072_at	CD300A	-1,212652659	-2,350444759	1,1377921
229747_x_at	LOC146880	-1,212652659	-2,350444759	1,1377921
231365_at	HOXA-AS4 /// MIR	-1,212652659	-2,350444759	1,1377921
207592_s_at	HCN2	-1,212652659	-2,350444759	1,1377921
206824_at	CES1P1	-1,212652659	-2,350444759	1,1377921
206859_s_at	PAEP	-1,212652659	-2,350444759	1,1377921
1552494_at	TAF8	-1,212652659	-2,350444759	1,1377921
1556777_a_at	-	-1,212652659	-2,350444759	1,1377921
52731_at	AMBRA1	2,318008416	1,180219373	1,137789043
228743_at	TXNDC17	2,007220798	0,869824768	1,13739603
212467_at	DNAJC13	2,811067138	1,674071917	1,136995221
225883_at	ATG16L2	1,047341799	-0,089635713	1,136977511
230150_at	BCAP29	1,047341799	-0,089635713	1,136977511
213944_x_at	GNA11	1,246401134	0,109458907	1,136942227

204167_at	BTD	0,618277321	-0,518232988	1,136510309
216282_x_at	POLR2C	0,618277321	-0,518232988	1,136510309
204425_at	ARHGAP4	1,802733148	0,666486137	1,13624701
217249_x_at	-	5,109492158	3,973269509	1,136222649
239940_at	-	-0,384193355	-1,520022123	1,135828768
1570169_at	CSMD2	-0,384193355	-1,520022123	1,135828768
204428_s_at	LCAT	-0,384193355	-1,520022123	1,135828768
240975_x_at	RBFOX3	-0,384193355	-1,520022123	1,135828768
222005_s_at	GNG3	-0,384193355	-1,520022123	1,135828768
215955_x_at	ARHGAP26	-0,384193355	-1,520022123	1,135828768
210084_x_at	TPSAB1	-0,384193355	-1,520022123	1,135828768
220337_at	NGB	-0,384193355	-1,520022123	1,135828768
1552501_a_at	GPBAR1	-0,384193355	-1,520022123	1,135828768
229097_at	DIAPH3	3,029186714	1,893444329	1,135742385
204483_at	ENO3	2,552836808	1,417157926	1,135678882
203113_s_at	EEF1D	7,007530727	5,872112033	1,135418694
218095_s_at	TMEM165	3,807915835	2,672497494	1,135418341
224818_at	SORT1	3,418470674	2,283169614	1,13530106
200623_s_at	CALM1 /// CALM2	3,327128828	2,192329358	1,13479947
227040_at	NHLRC3	3,422378845	2,287674614	1,134704231
240644_at	-	0,873789652	-0,260526297	1,134315949
233961_at	-	0,873789652	-0,260526297	1,134315949
217389_s_at	ATF5	0,873789652	-0,260526297	1,134315949
204350_s_at	MED7	2,884644843	1,750483134	1,134161709
221499_s_at	STX16	3,233229368	2,099079406	1,134149962
222730_s_at	ZDHHC2	3,086998005	1,953032682	1,133965322
1564277_a_at	LOC100133920 ///	0,244681185	-0,889222211	1,133903396
227436_at	OTUD7B	1,440265662	0,306454867	1,133810795
209447_at	SYNE1	3,001575397	1,868130962	1,133444436
201494_at	PRCP	3,636292808	2,503049671	1,133243138
216326_s_at	HDAC3	4,500752401	3,367769667	1,132982733
222654_at	IMPAD1	3,09217775	1,9592579	1,13291985
1598_g_at	GAS6	1,708925889	0,576102752	1,132823138
1566123_at	FABP6	-1,137208348	-2,27001641	1,132808062
217360_x_at	IGHA1 /// IGHG1 /,	-1,137208348	-2,27001641	1,132808062
241115_at	-	-1,137208348	-2,27001641	1,132808062
205033_s_at	DEFA1 /// DEFA1B	-1,137208348	-2,27001641	1,132808062
243095_at	-	-1,137208348	-2,27001641	1,132808062
225292_at	COL27A1	0,648195588	-0,484558493	1,132754081
204687_at	PARM1	0,648195588	-0,484558493	1,132754081
222749_at	SUFU	0,648195588	-0,484558493	1,132754081
235422_at	-	0,648195588	-0,484558493	1,132754081
225200_at	DPH3	4,010828383	2,878165848	1,132662535
204638_at	ACP5	-0,730013898	-1,862269442	1,132255543
236767_at	IQCF2	-0,730013898	-1,862269442	1,132255543
239569_at	FLJ31485	-0,730013898	-1,862269442	1,132255543
1570090_at	-	-0,730013898	-1,862269442	1,132255543
229683_s_at	KCTD15	-0,730013898	-1,862269442	1,132255543
215880_at	NAGLU	-0,730013898	-1,862269442	1,132255543
1553796_at	LOC100652811 ///	-0,730013898	-1,862269442	1,132255543

228878_s_at	CFC1B	-0,730013898	-1,862269442	1,132255543
207024_at	CHRND	-0,730013898	-1,862269442	1,132255543
204647_at	HOMER3	-0,730013898	-1,862269442	1,132255543
206162_x_at	SYT5	-0,730013898	-1,862269442	1,132255543
244400_at	VPS33A	-0,730013898	-1,862269442	1,132255543
214115_at	VAMP5	-0,730013898	-1,862269442	1,132255543
214433_s_at	SELENBP1	-0,730013898	-1,862269442	1,132255543
229621_x_at	EBF3	-0,730013898	-1,862269442	1,132255543
226238_at	MCEE	3,305120269	2,173028091	1,132092178
204081_at	NRGN	0,282358733	-0,84928999	1,131648723
238437_at	ZNF805	0,282358733	-0,84928999	1,131648723
206908_s_at	CLDN11	0,282358733	-0,84928999	1,131648723
228419_at	METRN	0,282358733	-0,84928999	1,131648723
238643_at	-	0,282358733	-0,84928999	1,131648723
222076_at	HBEGF	-1,369772723	-2,501359113	1,13158639
235853_at	C3orf58	-1,369772723	-2,501359113	1,13158639
207653_at	FOXD2	-1,369772723	-2,501359113	1,13158639
1569141_a_at	PPARGC1A	-1,369772723	-2,501359113	1,13158639
1563114_at	-	-1,369772723	-2,501359113	1,13158639
207336_at	SOX5	-1,369772723	-2,501359113	1,13158639
1560872_at	-	-1,369772723	-2,501359113	1,13158639
1569975_at	-	-1,369772723	-2,501359113	1,13158639
1570546_a_at	TACC2	-1,369772723	-2,501359113	1,13158639
232274_at	CCNL2	1,458255986	0,326777209	1,131478777
218797_s_at	SIRT7	3,214269085	2,082919929	1,131349156
212292_at	SLC7A1	0,900065578	-0,231045907	1,131111485
204801_s_at	DHRS12	0,319287178	-0,811769547	1,131056725
228849_at	NTRK3	0,319287178	-0,811769547	1,131056725
1554954_at	C21orf90	-0,437449947	-1,568385657	1,130935709
214275_at	MED12	-0,437449947	-1,568385657	1,130935709
205258_at	INHBB	-0,437449947	-1,568385657	1,130935709
208044_s_at	PPARD	-0,437449947	-1,568385657	1,130935709
208697_s_at	EIF3E	7,754779518	6,623868702	1,130910816
219462_at	TMEM53	1,094118704	-0,036543687	1,130662391
223350_x_at	LIN7C	3,883929118	2,753767314	1,130161804
224368_s_at	NDRG3	2,21987062	1,090640407	1,129230214
226555_at	INO80D	2,21987062	1,090640407	1,129230214
229270_x_at	SSBP4	1,307201325	0,178191865	1,12900946
201807_at	VPS26A	5,062183897	3,933228921	1,128954976
207625_s_at	CBFA2T2	1,871521385	0,742671819	1,128849566
216705_s_at	ADA	4,972743529	3,843963198	1,128780331
223073_at	HIATL1	4,718186291	3,589866014	1,128320278
212092_at	PEG10	0,926325262	-0,201789721	1,128114983
202207_at	ARL4C	3,729417893	2,601822502	1,127595391
224961_at	SCYL2	3,09217775	1,964670566	1,127507184
244732_at	-	1,116462765	-0,011001236	1,127464
200945_s_at	SEC31A	4,590648292	3,463284238	1,127364054
225431_x_at	PM20D2	4,418104951	3,290762066	1,127342885
217213_at	SLC6A2	-1,537851782	-2,665156778	1,127304996
231706_s_at	EVX1	-1,537851782	-2,665156778	1,127304996

233377_at	-	-1,624955693	-2,752097204	1,127141511
235874_at	PRSS35	-1,624955693	-2,752097204	1,127141511
222205_x_at	FAM182B	-1,624955693	-2,752097204	1,127141511
224574_at	C17orf49 /// RNAS	5,670103015	4,542978929	1,127124086
211768_at	LAT2	0,355751005	-0,771340337	1,127091342
207022_s_at	LDHC	0,355751005	-0,771340337	1,127091342
213380_x_at	MST1P9	0,355751005	-0,771340337	1,127091342
226288_s_at	NLGN2	0,355751005	-0,771340337	1,127091342
205013_s_at	ADORA2A	2,552836808	1,426280426	1,126556382
215645_at	FLCN	0,707657549	-0,418248858	1,125906407
231296_at	-	0,707657549	-0,418248858	1,125906407
225592_at	NRM	3,876052392	2,750185881	1,125866511
205904_at	MICA	2,085817135	0,960042218	1,125774916
214463_x_at	HIST1H4J	2,855122714	1,72935563	1,125767084
1561034_at	-	-0,924000698	-2,049747196	1,125746498
208294_x_at	CSHL1	-0,924000698	-2,049747196	1,125746498
214495_at	CACNG2	-0,924000698	-2,049747196	1,125746498
208563_x_at	POU3F3	-0,924000698	-2,049747196	1,125746498
236678_at	-	-0,924000698	-2,049747196	1,125746498
240722_at	LOC100505838	-0,924000698	-2,049747196	1,125746498
232178_at	ZNF503	-0,924000698	-2,049747196	1,125746498
219468_s_at	CUEDC1	-0,924000698	-2,049747196	1,125746498
229239_x_at	SLCO4A1	-0,924000698	-2,049747196	1,125746498
1557783_at	LOC100133991	-0,924000698	-2,049747196	1,125746498
1562227_at	PDE5A	-0,924000698	-2,049747196	1,125746498
241165_at	-	-0,924000698	-2,049747196	1,125746498
204736_s_at	CSPG4	-0,924000698	-2,049747196	1,125746498
1561320_at	-	-0,924000698	-2,049747196	1,125746498
227956_at	ITPRIPL2	-0,924000698	-2,049747196	1,125746498
228313_at	GPRC5B	-0,924000698	-2,049747196	1,125746498
217849_s_at	CDC42BPB	-0,924000698	-2,049747196	1,125746498
230359_at	KNDC1	-0,924000698	-2,049747196	1,125746498
214047_s_at	MBD4	5,253358403	4,127687813	1,12567059
221868_at	PAIP2B	0,39105295	-0,734355396	1,125408346
230353_at	LOC284112 /// MI	0,39105295	-0,734355396	1,125408346
209542_x_at	IGF1	0,39105295	-0,734355396	1,125408346
1553845_x_at	C10orf67	0,39105295	-0,734355396	1,125408346
202575_at	CRABP2	0,39105295	-0,734355396	1,125408346
243843_at	N4BP2L1	1,510970097	0,385582632	1,125387466
230023_at	NSUN4	2,56794744	1,44320939	1,12473805
204253_s_at	VDR	0,95222556	-0,172194225	1,124419785
201954_at	ARPC1B	6,283938077	5,159569795	1,124368282
244082_at	-	-0,49284695	-1,616924751	1,124077801
235714_at	GANC	-0,49284695	-1,616924751	1,124077801
233756_at	-	-0,49284695	-1,616924751	1,124077801
240129_at	-	-0,49284695	-1,616924751	1,124077801
206232_s_at	B4GALT6	-0,49284695	-1,616924751	1,124077801
223030_at	TRAF7	-0,49284695	-1,616924751	1,124077801
221281_at	SRC	-0,49284695	-1,616924751	1,124077801
206972_s_at	GPR161	-0,49284695	-1,616924751	1,124077801

207217_s_at	NOX1	-0,49284695	-1,616924751	1,124077801
224805_s_at	FAM219B	-0,49284695	-1,616924751	1,124077801
231924_at	LOC100506305	-0,49284695	-1,616924751	1,124077801
220314_at	HSFX1 /// HSFX2	-0,49284695	-1,616924751	1,124077801
217763_s_at	RAB31	-0,49284695	-1,616924751	1,124077801
214629_x_at	RTN4	6,432047535	5,308078354	1,123969181
244345_at	CADM1	-1,06282519	-2,186086603	1,123261413
227830_at	GABRB3	-1,06282519	-2,186086603	1,123261413
202008_s_at	NID1	-1,06282519	-2,186086603	1,123261413
1562818_at	NMNAT2	-1,06282519	-2,186086603	1,123261413
1563165_at	-	-1,06282519	-2,186086603	1,123261413
208213_s_at	KCNAB1	-1,06282519	-2,186086603	1,123261413
231349_at	GPR150	-1,06282519	-2,186086603	1,123261413
1556067_a_at	KDM6B	-1,06282519	-2,186086603	1,123261413
241919_x_at	WDR31	1,345611839	0,222432814	1,123179025
204215_at	C7orf23	4,918218598	3,795295041	1,122923557
235608_at	-	0,735999505	-0,386896102	1,122895607
239315_at	FAM115C	0,735999505	-0,386896102	1,122895607
226934_at	CPSF6	3,161901883	2,039063441	1,122838442
225826_at	MMAB	3,909506676	2,786807892	1,122698784
1555754_s_at	ATN1	2,321828846	1,199296812	1,122532034
216215_s_at	RBFOX2	2,25755351	1,135235995	1,122317515
239290_at	FRMPD4	0,425036312	-0,697182596	1,122218909
221134_at	ANGPT4	0,425036312	-0,697182596	1,122218909
229269_x_at	SSBP4	2,613382867	1,491644074	1,121738793
223530_at	TDRKH	2,267141818	1,146010634	1,121131185
225033_at	ST3GAL1	2,41945542	1,298349073	1,121106346
226730_s_at	USP37	2,427650693	1,306903969	1,120746724
203521_s_at	ZNF318	1,544940605	0,424693306	1,120247299
227685_at	TMF1	2,276736537	1,156599976	1,120136561
210244_at	CAMP	0,458905032	-0,661178575	1,120083606
219057_at	RABEP2	0,458905032	-0,661178575	1,120083606
222390_at	WAC	5,740542093	4,62057199	1,119970104
208526_at	OR2F1	-0,791294935	-1,911206569	1,119911634
243814_at	LOC100505963	-0,791294935	-1,911206569	1,119911634
221167_s_at	CCDC70	-0,791294935	-1,911206569	1,119911634
221576_at	LOC100653010	-0,791294935	-1,911206569	1,119911634
206149_at	CHP2	-0,791294935	-1,911206569	1,119911634
222917_s_at	TBX3	-0,791294935	-1,911206569	1,119911634
236216_at	-	-0,791294935	-1,911206569	1,119911634
243180_at	-	-0,791294935	-1,911206569	1,119911634
1553372_at	-	-0,791294935	-1,911206569	1,119911634
216881_x_at	PRB4	-0,791294935	-1,911206569	1,119911634
222594_s_at	SPATS2	1,364555912	0,244646697	1,119909215
202235_at	SLC16A1	2,642918178	1,523429058	1,11948912
202726_at	LIG1	3,331722254	2,212304392	1,119417862
207750_at	-	-1,712732543	-2,832036647	1,119304104
218187_s_at	C8orf33	4,684174558	3,564966474	1,119208085
215584_at	HECW1	0,764143511	-0,354781582	1,118925092
212299_at	NEK9	3,707586205	2,589579418	1,118006787

222636_at	MED28	1,383446753	0,265475485	1,117971268
213906_at	MYBL1	3,156891301	2,039063441	1,11782786
213479_at	NPTX2	-1,452713826	-2,570522741	1,117808915
237461_at	NLRP7	-1,452713826	-2,570522741	1,117808915
215199_at	CALD1	-1,452713826	-2,570522741	1,117808915
205735_s_at	AFF3	-1,452713826	-2,570522741	1,117808915
229440_at	RBM47	-1,452713826	-2,570522741	1,117808915
228401_at	ATAD2	3,999234177	2,881480309	1,117753868
203492_x_at	CEP57	4,00240646	2,884677311	1,117729149
208639_x_at	PDIA6	7,157025168	6,039309312	1,117715856
209076_s_at	WDR45L	4,785633175	3,668324222	1,117308953
223314_at	TSPAN14	1,000459215	-0,116767475	1,117226689
207375_s_at	IL15RA	2,804738618	1,687645398	1,11709322
209507_at	RPA3	5,666121193	4,549092012	1,117029181
203410_at	AP3M2	3,541158018	2,424202743	1,116955274
1555758_a_at	CDKN3	5,226175876	4,109561281	1,116614596
226867_at	DENND4C	3,362258367	2,245685006	1,116573361
200883_at	UQCRC2	6,287395528	5,171108889	1,116286639
212826_s_at	SLC25A6	6,570932844	5,454667593	1,116265251
223531_x_at	GPR89A /// GPR89	3,798440091	2,682180306	1,116259785
237034_at	-	1,402495885	0,286279868	1,116216017
212752_at	CLASP1	3,4540469	2,338020736	1,116026164
218572_at	CHMP4A /// TM9S	3,505636113	2,390281999	1,115354114
202699_s_at	TMEM63A	0,491906512	-0,623254098	1,11516061
212877_at	KLC1	2,074841895	0,960042218	1,114799677
1556019_at	GPR180	-0,551284523	-1,665791776	1,114507253
229244_at	LSAMP	-0,551284523	-1,665791776	1,114507253
206674_at	FLT3	-0,551284523	-1,665791776	1,114507253
216358_at	-	-0,551284523	-1,665791776	1,114507253
238238_at	-	-0,551284523	-1,665791776	1,114507253
227750_at	KALRN	-0,551284523	-1,665791776	1,114507253
204246_s_at	DCTN3	5,223881232	4,109561281	1,114319952
227043_at	CCDC159	0,791908897	-0,322340048	1,114248945
206419_at	RORC	0,791908897	-0,322340048	1,114248945
228225_at	PEX2	2,685337919	1,571112806	1,114225113
206958_s_at	UPF3A	3,900805316	2,786807892	1,113997424
223628_at	TMEM191A	1,024284941	-0,089635713	1,113920654
213379_at	COQ2	1,871521385	0,757638486	1,113882899
224280_s_at	FAM54B	2,392346173	1,278654284	1,11369189
219528_s_at	BCL11B	3,945594088	2,832036229	1,11355786
229919_at	-	0,524545436	-0,589001171	1,113546607
222760_at	ZNF703	0,524545436	-0,589001171	1,113546607
233892_at	GRIN3B	0,524545436	-0,589001171	1,113546607
1553464_at	FLJ40288	0,524545436	-0,589001171	1,113546607
1555238_at	PTH2	0,524545436	-0,589001171	1,113546607
210116_at	SH2D1A	3,795218988	2,682180306	1,113038682
224664_at	ANAPC16	6,203931152	5,091161037	1,112770116
204779_s_at	HOXB7	2,849132494	1,736450458	1,112682036
224640_at	SPPL3	3,353217129	2,24081821	1,112398919
225226_at	FAM40A	3,228532861	2,116147513	1,112385349

229174_at	C3orf38	3,614400403	2,503049671	1,111350732
217998_at	PHLDA1	1,458255986	0,346964736	1,111291251
219729_at	PRRX2	1,047341799	-0,063377083	1,110718882
224923_at	TTC7A	2,107136847	0,996453274	1,110683573
1570253_a_at	RHEBL1	2,444186596	1,333668715	1,110517881
217985_s_at	BAZ1A	3,951538845	2,841176366	1,110362479
208655_at	CCNI	5,773378356	4,663463224	1,109915132
203414_at	MMD	4,631436665	3,521590309	1,109846356
228031_at	TTPAL	3,175897496	2,066329863	1,109567632
61874_at	CACFD1	2,069603004	0,960293	1,109310005
221190_s_at	C18orf8	3,77412549	2,665727652	1,108397838
222916_s_at	HDLBP	0,847529938	-0,260526297	1,108056235
218523_at	LHPP	0,847529938	-0,260526297	1,108056235
203102_s_at	MGAT2	5,130891823	4,024017564	1,106874259
204513_s_at	ELMO1	4,283600482	3,176868665	1,106731817
222468_at	KIAA0319L	3,065977723	1,9592579	1,106719823
227502_at	-	1,307201325	0,20062106	1,106580265
1556969_at	CCDC108	-0,609040214	-1,715576125	1,106535911
242576_x_at	-	-0,609040214	-1,715576125	1,106535911
216122_at	-	-0,609040214	-1,715576125	1,106535911
220125_at	DNAI1	-0,609040214	-1,715576125	1,106535911
244833_at	CCDC63	-0,609040214	-1,715576125	1,106535911
223385_at	CYP2S1	-0,609040214	-1,715576125	1,106535911
1556012_at	KLHDC7A	-0,609040214	-1,715576125	1,106535911
1559991_s_at	-	-0,609040214	-1,715576125	1,106535911
224438_at	-	-0,609040214	-1,715576125	1,106535911
228476_at	KIAA1407	0,58788177	-0,518232988	1,106114759
210430_x_at	RHD	0,58788177	-0,518232988	1,106114759
226099_at	ELL2	3,649735397	2,543655878	1,10607952
221669_s_at	ACAD8	2,896717728	1,790914523	1,105803205
201256_at	COX7A2L	6,39662724	5,290992034	1,105635206
221216_s_at	SCMH1	1,65554524	0,550017041	1,105528199
218742_at	NARFL	1,094118704	-0,011001236	1,10511994
225949_at	LOC100653301 ///	1,094118704	-0,011001236	1,10511994
240742_at	-	0,873789652	-0,231045907	1,104835558
203122_at	TRAPPC12	3,282538749	2,177710147	1,104828603
225553_at	-	3,282538749	2,177710147	1,104828603
209688_s_at	CCDC93	2,908690075	1,804519951	1,104170125
201393_s_at	IGF2R	3,286844008	2,182711118	1,10413289
1562460_at	CNDP2	1,528073958	0,424693306	1,103380653
207035_at	SLC30A3	1,528073958	0,424693306	1,103380653
226405_s_at	ARRDC1	1,116462765	0,013190398	1,103272367
238700_at	PIAS2	1,116462765	0,013190398	1,103272367
201721_s_at	LAPTM5	5,659001793	4,555968307	1,103033486
237013_at	-	0,618277321	-0,484558493	1,102835814
204536_s_at	-	0,045517965	-1,057301851	1,102819816
236246_x_at	LOC653160	0,045517965	-1,057301851	1,102819816
243007_at	TTC5	0,045517965	-1,057301851	1,102819816
230860_at	CEP19	3,218770562	2,116147513	1,102623049
230908_at	TACR1	-0,994186487	-2,09678811	1,102601623

1558295_a_at	PPFIA2	-0,994186487	-2,09678811	1,102601623
229639_s_at	SLC19A1	-0,994186487	-2,09678811	1,102601623
1564471_at	LOC100130815	-0,994186487	-2,09678811	1,102601623
205312_at	SPI1	-0,994186487	-2,09678811	1,102601623
233344_x_at	KIAA1875	-0,994186487	-2,09678811	1,102601623
241493_at	-	-0,994186487	-2,09678811	1,102601623
229646_at	SOBP	-0,994186487	-2,09678811	1,102601623
1569925_at	LOC100507386	-0,994186487	-2,09678811	1,102601623
1556905_at	ZNF577	-0,994186487	-2,09678811	1,102601623
209792_s_at	KLK10	-0,994186487	-2,09678811	1,102601623
236965_at	UBQLNL	-0,994186487	-2,09678811	1,102601623
1561607_at	-	-0,994186487	-2,09678811	1,102601623
243365_s_at	AUTS2	-0,994186487	-2,09678811	1,102601623
1557491_at	LOC100240728	-0,994186487	-2,09678811	1,102601623
1557123_a_at	CHADL	-0,994186487	-2,09678811	1,102601623
215444_s_at	TRIM31	-0,994186487	-2,09678811	1,102601623
225659_at	SPOPL	2,16969527	1,067365565	1,102329705
217879_at	CDC27	3,934348221	2,832036229	1,102311993
222127_s_at	EXOC1	4,038663603	2,936370826	1,102292777
233040_at	PLEKHA5	-1,289805289	-2,391863529	1,10205824
238364_x_at	GLI4	-1,289805289	-2,391863529	1,10205824
231520_at	SLC35F3	-1,289805289	-2,391863529	1,10205824
1566194_at	-	-1,289805289	-2,391863529	1,10205824
1568762_at	LOC728613 /// PD	-1,289805289	-2,391863529	1,10205824
208501_at	GFI1B	-1,289805289	-2,391863529	1,10205824
1568606_at	C11orf88	-1,289805289	-2,391863529	1,10205824
1560472_at	LOC338588	0,001114523	-1,100943374	1,102057897
237979_at	-	0,001114523	-1,100943374	1,102057897
215232_at	ARHGAP44	0,001114523	-1,100943374	1,102057897
237343_at	LOC100505964	0,001114523	-1,100943374	1,102057897
206360_s_at	SOCS3	0,001114523	-1,100943374	1,102057897
1559075_s_at	BAHCC1	-0,088974936	-1,19085694	1,101882004
228651_at	VWA1	-0,088974936	-1,19085694	1,101882004
234935_at	NDOR1	-0,088974936	-1,19085694	1,101882004
208457_at	GABRD	-0,088974936	-1,19085694	1,101882004
238054_at	ADPRHL1	-0,088974936	-1,19085694	1,101882004
239617_at	GHRLOS	-0,088974936	-1,19085694	1,101882004
214423_x_at	ALDOB	-0,088974936	-1,19085694	1,101882004
213563_s_at	TUBGCP2	-0,088974936	-1,19085694	1,101882004
227119_at	CNOT6L	0,900065578	-0,201789721	1,101855299
228324_at	C9orf41	2,732422196	1,630734622	1,101687575
202428_x_at	DBI	7,28406687	6,182425262	1,101641608
1567274_at	-	0,08619576	-1,015398016	1,101593776
240373_at	CACTIN	0,08619576	-1,015398016	1,101593776
219332_at	MICALL2	0,08619576	-1,015398016	1,101593776
206410_at	NR0B2	0,08619576	-1,015398016	1,101593776
204018_x_at	HBA1 /// HBA2	0,08619576	-1,015398016	1,101593776
232640_at	COMMD5	0,08619576	-1,015398016	1,101593776
213173_at	PCNX	0,08619576	-1,015398016	1,101593776
235038_at	KRR1	-0,180990326	-1,282520722	1,101530396

234264_at	-	-0,180990326	-1,282520722	1,101530396
232761_at	COX4I2	-0,180990326	-1,282520722	1,101530396
217035_at	SKAP2	-0,180990326	-1,282520722	1,101530396
228226_s_at	ZNF775	1,139057614	0,03784737	1,101210244
203502_at	BPGM	1,701542258	0,600385088	1,10115717
241429_at	-	-0,043925712	-1,145057014	1,101131302
1555614_at	SUGT1P1	-0,043925712	-1,145057014	1,101131302
201187_s_at	ITPR3	-0,043925712	-1,145057014	1,101131302
240199_x_at	ZNF345	-0,043925712	-1,145057014	1,101131302
229682_at	MAPRE3	-0,043925712	-1,145057014	1,101131302
219781_s_at	ZNF771	1,345611839	0,244646697	1,100965141
209572_s_at	EED	5,440973988	4,340225042	1,100748945
229313_at	ANO5	0,127732831	-0,972893339	1,100626169
229343_at	GTSE1	0,127732831	-0,972893339	1,100626169
1569986_x_at	TNNT3	0,127732831	-0,972893339	1,100626169
205429_s_at	MPP6	3,023747815	1,923216608	1,100531207
214579_at	NIPAL3	1,983901861	0,883374248	1,100527612
234633_at	KRTAP4-11	-0,856259172	-1,956681069	1,100421898
232082_x_at	SPRR3	-0,856259172	-1,956681069	1,100421898
226961_at	PRR15	-0,856259172	-1,956681069	1,100421898
206812_at	ADRB3	-0,856259172	-1,956681069	1,100421898
231330_at	LINC00445	-0,856259172	-1,956681069	1,100421898
1566137_s_at	-	-0,856259172	-1,956681069	1,100421898
224490_s_at	KANSL1	-0,856259172	-1,956681069	1,100421898
232917_at	-	-0,856259172	-1,956681069	1,100421898
217435_x_at	-	-0,856259172	-1,956681069	1,100421898
234010_at	-	-0,856259172	-1,956681069	1,100421898
226801_s_at	AIDA	5,916351685	4,816504597	1,099847088
242505_at	-	-0,135065865	-1,234835326	1,099769461
238684_at	SETDB2	-0,135065865	-1,234835326	1,099769461
229124_at	PROK1	-0,135065865	-1,234835326	1,099769461
230982_at	SOX1	-0,135065865	-1,234835326	1,099769461
204431_at	TLE2	-0,135065865	-1,234835326	1,099769461
236540_at	-	-0,135065865	-1,234835326	1,099769461
223676_at	TMEM234	-0,135065865	-1,234835326	1,099769461
1553812_at	TLE6	-0,135065865	-1,234835326	1,099769461
223293_at	WDR24	0,648195588	-0,45121326	1,099408848
205723_at	CNTFR	0,648195588	-0,45121326	1,099408848
222836_at	NAA15	-1,212652659	-2,312054429	1,09940177
209541_at	IGF1	-1,212652659	-2,312054429	1,09940177
216827_at	-	-1,212652659	-2,312054429	1,09940177
1561015_at	-	-1,212652659	-2,312054429	1,09940177
1563611_at	-	-1,212652659	-2,312054429	1,09940177
1563057_at	-	-1,212652659	-2,312054429	1,09940177
215527_at	KHDRBS2	-1,212652659	-2,312054429	1,09940177
242661_x_at	ALS2CR12	-1,212652659	-2,312054429	1,09940177
1553341_at	UROC1	-1,212652659	-2,312054429	1,09940177
237585_at	C4orf47	-1,212652659	-2,312054429	1,09940177
235824_at	-	-1,212652659	-2,312054429	1,09940177
1557818_x_at	-	0,167727503	-0,931539147	1,09926665

203149_at	PVRL2	0,167727503	-0,931539147	1,09926665
233511_at	-	0,167727503	-0,931539147	1,09926665
207391_s_at	PIP5K1A	3,739061547	2,639836674	1,099224873
228675_at	USP30-AS1	1,160996507	0,061773582	1,099222925
219241_x_at	SSH3	1,160996507	0,061773582	1,099222925
218372_at	MED9	1,364555912	0,265475485	1,099080427
201132_at	HNRNPH2 /// RPL3	4,895653873	3,796883855	1,098770017
211825_s_at	FLI1	1,871521385	0,772793615	1,09872777
228272_at	DNLZ	3,137734155	2,039063441	1,098670713
230450_at	-	0,926325262	-0,172194225	1,098519487
231188_at	ZSCAN2	1,88491011	0,786391549	1,098518561
212310_at	MIA3	1,716003175	0,61779983	1,098203344
227092_at	LOC100507448	1,716003175	0,61779983	1,098203344
1553561_at	TAS2R50	-1,624955693	-2,723114724	1,098159031
202749_at	WRB	4,642573329	3,544644421	1,097928908
1553710_at	FAM218A	-1,537851782	-2,635549085	1,097697302
1553142_at	LACC1	-1,537851782	-2,635549085	1,097697302
1562864_at	-	-1,537851782	-2,635549085	1,097697302
211489_at	ADRA1A	-1,537851782	-2,635549085	1,097697302
241691_at	SCARF2	-1,537851782	-2,635549085	1,097697302
233879_at	-	-1,537851782	-2,635549085	1,097697302
223391_at	SGPP1	2,972517974	1,874839058	1,097678916
238909_at	S100A10	0,95222556	-0,144765583	1,096991143
227263_at	C8orf58	0,95222556	-0,144765583	1,096991143
236885_at	MEX3A	0,95222556	-0,144765583	1,096991143
214763_at	ACOT11	0,95222556	-0,144765583	1,096991143
204782_at	-	-0,230432956	-1,32740143	1,096968474
240069_at	-	-0,230432956	-1,32740143	1,096968474
1566473_a_at	-	-0,230432956	-1,32740143	1,096968474
228756_at	SLC6A6	0,20740896	-0,889222211	1,096631171
231927_at	ATF6	1,730929079	0,634301128	1,096627951
1559030_a_at	-	-1,369772723	-2,466349414	1,096576691
229199_at	SCN9A	-1,369772723	-2,466349414	1,096576691
222166_at	C9orf16	-1,369772723	-2,466349414	1,096576691
240354_at	C12orf54	-1,369772723	-2,466349414	1,096576691
231805_at	PRLHR	-1,369772723	-2,466349414	1,096576691
240715_at	TBX5	-1,369772723	-2,466349414	1,096576691
1557734_s_at	LOC100130548	-1,369772723	-2,466349414	1,096576691
234045_x_at	BHLHE23	-1,369772723	-2,466349414	1,096576691
242869_at	-	-1,369772723	-2,466349414	1,096576691
209554_at	CD36	-1,369772723	-2,466349414	1,096576691
243940_at	TSHZ2	-1,369772723	-2,466349414	1,096576691
39763_at	HPX	0,741248221	-0,354781582	1,096029803
236478_at	IFNAR1	0,677745787	-0,418248858	1,095994645
216241_s_at	TCEA1	6,40207937	5,306244827	1,095834543
229342_at	C17orf85	3,007119335	1,911502437	1,095616899
35617_at	MAPK7	0,779095143	-0,316324892	1,095420036
204241_at	ACOX3	1,745502609	0,65030602	1,095196589
230980_x_at	-	1,610353504	0,515457602	1,094895902
238332_at	ANKRD29	1,421391637	0,326777209	1,094614428

218749_s_at	SLC24A6	0,707657549	-0,386896102	1,094553651
1552588_a_at	CNBD1	-0,66934516	-1,763664074	1,094318915
235272_at	SBSN	-0,66934516	-1,763664074	1,094318915
240206_at	TARS	-0,66934516	-1,763664074	1,094318915
215403_at	-	-0,66934516	-1,763664074	1,094318915
220598_at	CARD14	-0,66934516	-1,763664074	1,094318915
231903_x_at	ARHGAP23	-0,66934516	-1,763664074	1,094318915
208620_at	PCBP1	5,402600718	4,308463489	1,094137229
217344_at	-	0,282358733	-0,811769547	1,09412828
244488_at	LSM14B	0,282358733	-0,811769547	1,09412828
221450_x_at	PCDHB13	0,282358733	-0,811769547	1,09412828
205332_at	RCE1	0,282358733	-0,811769547	1,09412828
218834_s_at	TMEM132A	0,282358733	-0,811769547	1,09412828
226711_at	FOXN2	4,846478638	3,7524971	1,093981538
234403_at	OR1I1	0,244681185	-0,84928999	1,093971175
239149_at	-	0,244681185	-0,84928999	1,093971175
236087_at	ABLIM2	0,244681185	-0,84928999	1,093971175
235998_at	RHPN1	0,244681185	-0,84928999	1,093971175
225648_at	STK35	1,936380191	0,842599219	1,093780972
224906_at	ANO6	4,193391347	3,099629381	1,093761966
208097_s_at	TMX1	4,125707946	3,032393954	1,093313992
225756_at	CSNK1E	1,775035811	0,681874787	1,093161025
1566831_at	SBF2-AS1	-0,280273599	-1,372734086	1,092460487
230995_at	CMBL	-0,280273599	-1,372734086	1,092460487
244207_at	-	-0,280273599	-1,372734086	1,092460487
241050_at	-	-0,280273599	-1,372734086	1,092460487
1554566_at	KCTD17	-0,280273599	-1,372734086	1,092460487
211231_x_at	CYP4A11	-0,280273599	-1,372734086	1,092460487
214191_at	ICA1	-0,280273599	-1,372734086	1,092460487
206771_at	UPK3A	-0,280273599	-1,372734086	1,092460487
1554683_a_at	MGC50722	-0,280273599	-1,372734086	1,092460487
214939_x_at	MLLT4	-0,280273599	-1,372734086	1,092460487
224936_at	EIF2S3	5,350868606	4,258615677	1,092252929
1559026_at	LOC100506599	-1,137208348	-2,229233437	1,092025089
236188_s_at	NAP1L4	-1,137208348	-2,229233437	1,092025089
243187_at	-	-1,137208348	-2,229233437	1,092025089
205553_s_at	CSRP3	-1,137208348	-2,229233437	1,092025089
242821_at	CCDC171	-1,137208348	-2,229233437	1,092025089
1569828_at	-	-1,137208348	-2,229233437	1,092025089
237534_at	-	-1,137208348	-2,229233437	1,092025089
227643_at	TPPP	-1,137208348	-2,229233437	1,092025089
205738_s_at	FABP3	-1,137208348	-2,229233437	1,092025089
219661_at	RANBP17	-1,137208348	-2,229233437	1,092025089
239542_at	ITPR3	-1,137208348	-2,229233437	1,092025089
214493_s_at	INADL	-1,137208348	-2,229233437	1,092025089
35666_at	SEMA3F	2,112100655	1,020379916	1,091720739
200850_s_at	AHCYL1	4,957022369	3,865466305	1,091556064
202922_at	GCLC	3,147261986	2,055739171	1,091522815
202324_s_at	ACBD3	3,855094415	2,76399677	1,091097645
214630_at	CYP11B2	0,735999505	-0,354781582	1,090781087

210307_s_at	KLHL25	0,735999505	-0,354781582	1,090781087
1554101_a_at	TMTC4	0,735999505	-0,354781582	1,090781087
34206_at	ARAP1	2,132244931	1,04158527	1,090659661
235042_at	XIRP1	0,319287178	-0,771340337	1,090627515
227504_s_at	-	0,319287178	-0,771340337	1,090627515
219559_at	SLC17A9	0,319287178	-0,771340337	1,090627515
213463_s_at	FAM149B1	1,476175815	0,385582632	1,090593183
218924_s_at	CTBS	3,117098466	2,026957145	1,090141321
208057_s_at	GLI2	0,355751005	-0,734355396	1,090106401
221023_s_at	KCNH6	0,355751005	-0,734355396	1,090106401
244326_at	-	0,355751005	-0,734355396	1,090106401
235930_at	KCNMB4	0,355751005	-0,734355396	1,090106401
211125_x_at	GRIN1	0,355751005	-0,734355396	1,090106401
226615_at	XPR1	0,355751005	-0,734355396	1,090106401
235039_x_at	LIN9	1,000459215	-0,089635713	1,090094927
1563369_at	LINC00173	-0,332405896	-1,422257026	1,08985113
203238_s_at	NOTCH3	-0,332405896	-1,422257026	1,08985113
207946_at	MAML3	-0,332405896	-1,422257026	1,08985113
235814_at	-	-0,332405896	-1,422257026	1,08985113
213231_at	DMWD	-0,332405896	-1,422257026	1,08985113
230188_at	NIPAL4	-0,332405896	-1,422257026	1,08985113
212114_at	ATXN7L3B	3,825885656	2,736663738	1,089221919
230460_at	MYLK-AS1	1,267146969	0,178191865	1,088955104
221726_at	RPL22	6,636408544	5,547586867	1,088821677
221268_s_at	SGPP1	2,096248267	1,008210238	1,08803803
217986_s_at	BAZ1A	3,8018113	2,713775735	1,088035565
217313_at	-	2,824160383	1,736450458	1,087709925
207715_at	CRYGB	-0,384193355	-1,471833228	1,087639873
219963_at	DUSP13	-0,384193355	-1,471833228	1,087639873
1556429_a_at	WDR67	-0,384193355	-1,471833228	1,087639873
226573_at	DIRAS1	-0,384193355	-1,471833228	1,087639873
215771_x_at	RET	-0,384193355	-1,471833228	1,087639873
243157_at	-	-0,384193355	-1,471833228	1,087639873
222015_at	CSNK1E	1,65554524	0,568155442	1,087389797
204034_at	ETHE1	4,193391347	3,106874588	1,086516759
207838_x_at	PBXIP1	0,764143511	-0,322340048	1,086483559
51200_at	C19orf60	3,463264966	2,376813739	1,086451227
209102_s_at	HBP1	3,273854609	2,187622928	1,086231681
234037_s_at	-	0,425036312	-0,661178575	1,086214887
232870_at	HDAC10	0,425036312	-0,661178575	1,086214887
223563_at	GNB1L	0,425036312	-0,661178575	1,086214887
202172_at	VEZF1	3,414594082	2,328492509	1,086101573
219494_at	FSBP /// RAD54B	3,233229368	2,147194726	1,086034641
229265_at	SKI	2,836219929	1,750483134	1,085736795
209702_at	FTO	3,248151517	2,162541631	1,085609886
219594_at	NINJ2	3,951538845	2,86626857	1,085270275
207244_x_at	CYP2A6	1,528073958	0,443143223	1,084930736
216650_at	-	1,307201325	0,222432814	1,084768511
222805_at	MANEA	3,8018113	2,717094254	1,084717046
229384_at	-	2,127965541	1,043469112	1,084496429

222839_s_at	PAPOLG	3,161901883	2,077419168	1,084482715
202300_at	HBXIP	6,535246628	5,451001979	1,084244649
217844_at	CTDSP1	3,007119335	1,923216608	1,083902727
204243_at	RLF	3,001575397	1,917743773	1,083831624
218577_at	LRRC40	5,289992607	4,206178	1,083814607
238813_at	APEX2	1,701542258	0,61779983	1,083742428
227207_x_at	ZNF213	1,701542258	0,61779983	1,083742428
1554086_at	TUBGCP3	2,575362572	1,491644074	1,083718498
236584_at	C1orf86	-1,452713826	-2,536339282	1,083625456
233778_at	-	-1,452713826	-2,536339282	1,083625456
221682_s_at	PCDHGB6	-1,452713826	-2,536339282	1,083625456
1570291_at	CLDN10-AS1	-1,452713826	-2,536339282	1,083625456
209547_s_at	SUGP1	1,544940605	0,461385738	1,083554867
202798_at	SEC24B	5,419478694	4,336213543	1,083265151
201285_at	MKRN1	3,402275492	2,31904917	1,083226322
218213_s_at	C11orf10	6,736718568	5,653935083	1,082783485
237536_at	MAPT-AS1	0,791908897	-0,290694975	1,082603872
1553603_s_at	ATL2	0,791908897	-0,290694975	1,082603872
240402_at	KIRREL3	-0,437449947	-1,520022123	1,082572176
241565_at	COL4A4	-0,437449947	-1,520022123	1,082572176
214344_at	FP588	-0,437449947	-1,520022123	1,082572176
234121_at	LOC100129069	-0,437449947	-1,520022123	1,082572176
235297_at	CELF1	-0,437449947	-1,520022123	1,082572176
243292_at	FAM132A	-0,437449947	-1,520022123	1,082572176
212938_at	COL6A1	-0,437449947	-1,520022123	1,082572176
207950_s_at	ANK3	-0,437449947	-1,520022123	1,082572176
1560908_at	-	-0,437449947	-1,520022123	1,082572176
213146_at	KDM6B	-0,437449947	-1,520022123	1,082572176
229087_s_at	-	-0,437449947	-1,520022123	1,082572176
217349_s_at	PRICKLE3	-0,437449947	-1,520022123	1,082572176
1566957_at	-	-0,437449947	-1,520022123	1,082572176
204420_at	FOSL1	-0,437449947	-1,520022123	1,082572176
226757_at	IFIT2	1,071336699	-0,011001236	1,082337934
209329_x_at	HIGD2A	5,239212617	4,157029702	1,082182915
230640_at	-	0,458905032	-0,623254098	1,08215913
220332_at	CLDN16	0,458905032	-0,623254098	1,08215913
204968_at	C6orf47	1,326463531	0,244646697	1,081816834
243516_at	-	-0,730013898	-1,811780055	1,081766156
244783_at	YAF2	-0,730013898	-1,811780055	1,081766156
240691_at	-	-0,730013898	-1,811780055	1,081766156
1553945_at	GPHB5	-0,730013898	-1,811780055	1,081766156
214851_at	HNF4A	-0,730013898	-1,811780055	1,081766156
236586_at	-	-0,730013898	-1,811780055	1,081766156
211639_x_at	IGH@ /// IGHA1 //	-0,730013898	-1,811780055	1,081766156
233962_at	-	-0,730013898	-1,811780055	1,081766156
243144_at	-	-0,730013898	-1,811780055	1,081766156
202177_at	GAS6	-0,730013898	-1,811780055	1,081766156
1565903_at	-	-0,730013898	-1,811780055	1,081766156
238112_at	LOC283177	-0,730013898	-1,811780055	1,081766156
229463_at	NTRK2	-0,730013898	-1,811780055	1,081766156

202565_s_at	SVIL	2,159837409	1,078619386	1,081218023
212785_s_at	LARP7	5,344203926	4,263096467	1,081107459
1557531_a_at	C10orf55	-0,924000698	-2,005029581	1,081028883
216732_at	-	-0,924000698	-2,005029581	1,081028883
227183_at	MIR143HG	-0,924000698	-2,005029581	1,081028883
229652_s_at	FOXN3	-0,924000698	-2,005029581	1,081028883
1566672_at	-	-0,924000698	-2,005029581	1,081028883
215049_x_at	CD163	-0,924000698	-2,005029581	1,081028883
1552738_a_at	ST7L	-0,924000698	-2,005029581	1,081028883
203877_at	MMP11	-0,924000698	-2,005029581	1,081028883
239145_at	ZNF414	-0,924000698	-2,005029581	1,081028883
208252_s_at	CHST3	-0,924000698	-2,005029581	1,081028883
1565974_at	-	-0,924000698	-2,005029581	1,081028883
233597_at	PNPLA5	-0,924000698	-2,005029581	1,081028883
217963_s_at	NGFRAP1	-0,924000698	-2,005029581	1,081028883
206017_at	KIAA0319	0,491906512	-0,589001171	1,080907683
221246_x_at	TNS1	0,491906512	-0,589001171	1,080907683
213667_at	LOC100862671 ///	0,491906512	-0,589001171	1,080907683
214188_at	-	0,491906512	-0,589001171	1,080907683
218216_x_at	ARL6IP4	4,475284297	3,395519718	1,079764579
234069_at	-	-1,06282519	-2,142286822	1,079461632
1555664_at	-	-1,06282519	-2,142286822	1,079461632
211903_s_at	MPL	-1,06282519	-2,142286822	1,079461632
203631_s_at	GPRC5B	-1,06282519	-2,142286822	1,079461632
214381_at	LOC441601	-1,06282519	-2,142286822	1,079461632
238731_at	SETDB2	-1,06282519	-2,142286822	1,079461632
232743_at	-	-1,06282519	-2,142286822	1,079461632
224074_at	VSX1	-1,06282519	-2,142286822	1,079461632
210204_s_at	CNOT4	-1,06282519	-2,142286822	1,079461632
211618_s_at	ALPI	-1,06282519	-2,142286822	1,079461632
209988_s_at	ASCL1	-1,06282519	-2,142286822	1,079461632
215712_s_at	IGFALS	-1,06282519	-2,142286822	1,079461632
1566630_at	PPP2R3B	-1,06282519	-2,142286822	1,079461632
235495_at	CCDC97	0,524545436	-0,554696666	1,079242103
206821_x_at	AGFG2	0,524545436	-0,554696666	1,079242103
213698_at	ZMYM6 /// ZMYM	4,632969749	3,553756974	1,079212775
220659_s_at	C7orf43	0,847529938	-0,231045907	1,078575844
238105_x_at	WNT7B	0,847529938	-0,231045907	1,078575844
239004_at	SQSTM1	0,847529938	-0,231045907	1,078575844
227611_at	TARSL2	2,074841895	0,996453274	1,078388622
216862_s_at	MTCP1NB	4,080162946	3,002270677	1,077892269
55065_at	MARK4	3,406492124	2,328908558	1,077583566
213104_at	TSR3	2,476788561	1,399283506	1,077505055
216305_s_at	GCFC2	3,269673646	2,192329358	1,077344288
213573_at	-	4,2463808	3,169167796	1,077213004
211352_s_at	NCOA3	1,610353504	0,533258442	1,077095062
200889_s_at	SSR1	4,272544367	3,195836661	1,076707705
206542_s_at	SMARCA2	4,533526699	3,457327669	1,07619903
208752_x_at	NAP1L1	7,540950452	6,464841067	1,076109385
204346_s_at	RASSF1	3,470020347	2,394276989	1,075743358

203855_at	WDR47	3,278243793	2,20251271	1,075731083
212410_at	EFHA1	4,64066268	3,564966474	1,075696207
244487_at	-	0,873789652	-0,201789721	1,075579373
226267_at	JDP2	-0,49284695	-1,568385657	1,075538706
229184_at	CCNT2	-0,49284695	-1,568385657	1,075538706
220698_at	MGC4294	-0,49284695	-1,568385657	1,075538706
234586_at	SARDH	-0,49284695	-1,568385657	1,075538706
1560074_at	PRKCA	-0,49284695	-1,568385657	1,075538706
233673_at	-	-0,49284695	-1,568385657	1,075538706
1570281_at	-	-0,49284695	-1,568385657	1,075538706
1555415_at	-	-0,49284695	-1,568385657	1,075538706
215488_at	-	-0,49284695	-1,568385657	1,075538706
202432_at	PPP3CB	5,115580177	4,040084244	1,075495932
223115_at	MED17	1,625482993	0,550017041	1,075465953
237287_at	HMGA1P4	1,160996507	0,085534992	1,075461515
211320_s_at	PTPRU	1,160996507	0,085534992	1,075461515
205849_s_at	UQCRB	6,914635777	5,839493279	1,075142498
207700_s_at	NCOA3	3,544559557	2,469496389	1,075063168
228300_at	CCDC103 /// FAM1	0,556428218	-0,518232988	1,074661206
241408_at	CRIPAK	0,556428218	-0,518232988	1,074661206
236286_at	PROCA1	0,556428218	-0,518232988	1,074661206
243026_x_at	XIAP	1,816938997	0,742671819	1,074267178
222349_x_at	RNF126P1	1,440265662	0,366031214	1,074234449
220323_at	CNTD2	1,182925501	0,109458907	1,073466595
219149_x_at	DBR1	2,890618076	1,817403386	1,073214689
211678_s_at	RNF114	5,329807155	4,25661911	1,073188045
200685_at	SRSF11	3,720319513	2,64735888	1,072960633
208796_s_at	CCNG1	6,468340697	5,395494602	1,072846095
222245_s_at	FER1L4	0,58788177	-0,484558493	1,072440264
1562852_at	-	0,58788177	-0,484558493	1,072440264
231931_at	PRDM15	0,58788177	-0,484558493	1,072440264
227228_s_at	CCDC88C	2,836219929	1,76396664	1,072253289
213000_at	MORC3	2,836219929	1,76396664	1,072253289
223776_x_at	TINF2	3,414594082	2,34250725	1,072086831
228452_at	C17orf39	1,640193838	0,568155442	1,072038396
227464_at	ACSF3	0,926325262	-0,144765583	1,071090845
219853_at	FKRP	-0,791294935	-1,862269442	1,070974506
1559828_at	FAM170B	-0,791294935	-1,862269442	1,070974506
1556409_a_at	-	-0,791294935	-1,862269442	1,070974506
233959_at	ADCY10P1	-0,791294935	-1,862269442	1,070974506
230247_at	-	-0,791294935	-1,862269442	1,070974506
214498_at	ASIP	-0,791294935	-1,862269442	1,070974506
220123_at	SLC35F5	-0,791294935	-1,862269442	1,070974506
227631_at	ABI2	-0,791294935	-1,862269442	1,070974506
207204_at	FSCN2	-0,791294935	-1,862269442	1,070974506
208201_at	DUX2 /// DUX3 ///	-0,791294935	-1,862269442	1,070974506
234456_at	MIR3656 /// TRAP	-0,791294935	-1,862269442	1,070974506
1556266_a_at	C20orf202	-0,791294935	-1,862269442	1,070974506
231724_at	MED26	-0,791294935	-1,862269442	1,070974506
206973_at	PPFIA2	-0,791294935	-1,862269442	1,070974506

236040_at	XAGE3	-0,791294935	-1,862269442	1,070974506
221680_s_at	ETV7	-0,791294935	-1,862269442	1,070974506
218146_at	GLT8D1	5,234483294	4,163558413	1,070924881
216201_at	-	-1,624955693	-2,695640852	1,070685159
1552731_at	ABRA	-1,624955693	-2,695640852	1,070685159
1561527_at	-	-1,624955693	-2,695640852	1,070685159
224223_s_at	PDE11A	-1,624955693	-2,695640852	1,070685159
1566740_at	PLCE1	-1,624955693	-2,695640852	1,070685159
204208_at	RNGTT	3,77412549	2,703479652	1,070645838
208791_at	CLU	1,225123479	0,155747281	1,069376198
221485_at	B4GALT5	4,672908132	3,604010628	1,068897504
217823_s_at	UBE2J1	4,523665259	3,45525776	1,068407499
231530_s_at	C11orf1	3,855094415	2,786807892	1,068286523
217122_s_at	SLC35E2 /// SLC35	4,741350888	3,673370032	1,067980857
238389_s_at	-	2,267141818	1,199296812	1,067845006
1556959_at	ANHX	1,701542258	0,634301128	1,06724113
227786_at	MED30	3,473999051	2,406959565	1,067039486
225804_at	CYB5D2	2,276736537	1,209973561	1,066762976
233748_x_at	PRKAG2	1,92317941	0,85655369	1,06662572
242704_at	ZNF821	1,267146969	0,20062106	1,06652591
226993_at	-	3,132805465	2,066329863	1,066475602
204922_at	C11orf80	0,648195588	-0,418248858	1,066444446
208160_at	-	0,648195588	-0,418248858	1,066444446
823_at	CX3CL1	-0,865682305	-1,931884399	1,066202095
36084_at	CUL7	1,615965689	0,549934923	1,066030766
207071_s_at	ACO1	2,613382867	1,547376668	1,066006199
220039_s_at	CDKAL1	-0,551284523	-1,616924751	1,065640229
230816_at	FAM163B	-0,551284523	-1,616924751	1,065640229
216265_x_at	MYH7	-0,551284523	-1,616924751	1,065640229
207887_s_at	CALCR	-0,551284523	-1,616924751	1,065640229
204628_s_at	ITGB3	-0,551284523	-1,616924751	1,065640229
1569032_at	LOC642852	-0,551284523	-1,616924751	1,065640229
214935_at	IL4I1 /// NUP62 //,	-0,551284523	-1,616924751	1,065640229
219393_s_at	AKT3	-0,551284523	-1,616924751	1,065640229
229922_at	GPRIN1	-0,551284523	-1,616924751	1,065640229
229379_at	AHDC1	-0,551284523	-1,616924751	1,065640229
205636_at	SH3GL3	-0,551284523	-1,616924751	1,065640229
215520_at	PYGO1	-1,537851782	-2,603471267	1,065619484
1569259_at	-	-1,537851782	-2,603471267	1,065619484
223678_s_at	SFTPA1 /// SFTPA2	-1,537851782	-2,603471267	1,065619484
243930_x_at	-	-1,537851782	-2,603471267	1,065619484
211045_s_at	KCNH6	-1,537851782	-2,603471267	1,065619484
241709_s_at	DOCK1	-1,537851782	-2,603471267	1,065619484
224076_s_at	WHSC1L1	3,296548503	2,23115548	1,065393023
213598_at	-	4,270249493	3,205255599	1,064993894
224716_at	MIR4647 /// SLC35	3,942941653	2,878165848	1,064775805
221653_x_at	APOL2	2,926639205	1,861868165	1,06477104
210406_s_at	RAB6A /// RAB6C /	5,114525352	4,049836996	1,064688356
222109_at	GNL3L	0,677745787	-0,386896102	1,064641889
229488_at	OTUD7B	0,677745787	-0,386896102	1,064641889

238530_at	NNT	1,730929079	0,666486137	1,064442942
211139_s_at	NAB1	1,730929079	0,666486137	1,064442942
201319_at	MYL12A	6,376569858	5,31217691	1,064392948
52741_at	TRMT61A	1,151364986	0,086976588	1,064388397
219618_at	IRAK4	2,649978302	1,586121802	1,063856501
209619_at	CD74	6,420970662	5,358390441	1,062580221
205469_s_at	IRF5	1,307201325	0,244646697	1,062554628
203994_s_at	C21orf2	0,707657549	-0,354781582	1,06243913
205308_at	ZC2HC1A	3,551632175	2,490640503	1,060991672
213502_x_at	GUSBP11	1,326463531	0,265475485	1,060988046
238316_at	ZNF567	0,045517965	-1,015398016	1,060915981
204473_s_at	ZNF592	1,024284941	-0,036543687	1,060828628
1565603_at	PWP2	1,024284941	-0,036543687	1,060828628
239527_at	RAB3GAP1	-1,289805289	-2,350444759	1,06063947
1553823_a_at	RTP1	-1,289805289	-2,350444759	1,06063947
1566427_at	-	-1,289805289	-2,350444759	1,06063947
228554_at	PGR	-1,289805289	-2,350444759	1,06063947
209387_s_at	TM4SF1	-1,289805289	-2,350444759	1,06063947
1559254_at	LINC00162	-1,289805289	-2,350444759	1,06063947
235943_at	-	-1,289805289	-2,350444759	1,06063947
233725_at	-	-1,289805289	-2,350444759	1,06063947
231656_x_at	OSBPL10	-1,289805289	-2,350444759	1,06063947
227723_at	LOH12CR1	2,138605583	1,078619386	1,059986197
236247_at	NSUN4	1,995527416	0,936232181	1,059295235
210990_s_at	LAMA4	0,127732831	-0,931539147	1,059271978
243900_at	WDR38	0,127732831	-0,931539147	1,059271978
240124_at	-	0,127732831	-0,931539147	1,059271978
224514_x_at	IL17RC	0,127732831	-0,931539147	1,059271978
201812_s_at	C4orf46 /// TOMM	6,540152997	5,480900532	1,059252465
1553301_a_at	TMEM182	0,08619576	-0,972893339	1,059089098
1570225_at	TMEM150B	0,08619576	-0,972893339	1,059089098
206896_s_at	GNG7	0,08619576	-0,972893339	1,059089098
1559917_a_at	CBR3-AS1	0,08619576	-0,972893339	1,059089098
235699_at	REM2	0,08619576	-0,972893339	1,059089098
208138_at	GAST	0,08619576	-0,972893339	1,059089098
228922_at	SHF	0,08619576	-0,972893339	1,059089098
238187_at	C2orf57	0,08619576	-0,972893339	1,059089098
223019_at	FAM129B	0,08619576	-0,972893339	1,059089098
209808_x_at	ING1	2,836219929	1,777135134	1,059084795
210018_x_at	MALT1	3,023747815	1,964670566	1,05907725
218157_x_at	CDC42SE1	5,205487521	4,146536585	1,058950935
228174_at	SCAI	2,829929672	1,771157334	1,058772338
230567_at	KIAA1430	-1,369772723	-2,4284334	1,058660677
242979_at	IRS1	-1,369772723	-2,4284334	1,058660677
205969_at	AADAC	-1,369772723	-2,4284334	1,058660677
1559732_at	NUB1	-1,369772723	-2,4284334	1,058660677
207424_at	MYF5	-1,369772723	-2,4284334	1,058660677
207250_at	SIX6	-1,369772723	-2,4284334	1,058660677
219498_s_at	BCL11A	-1,369772723	-2,4284334	1,058660677
229993_at	ZCCHC3	-1,369772723	-2,4284334	1,058660677

241552_at	AA06	-1,369772723	-2,4284334	1,058660677
200914_x_at	KTN1	4,521773976	3,463284238	1,058489738
218879_s_at	MTHFSD	0,001114523	-1,057301851	1,058416374
211901_s_at	PDE4A	0,001114523	-1,057301851	1,058416374
230295_at	LOC100507047 ///	0,001114523	-1,057301851	1,058416374
223467_at	RASD1	0,001114523	-1,057301851	1,058416374
226411_at	EVI5L	0,735999505	-0,322340048	1,058339553
218045_x_at	PTMS	0,735999505	-0,322340048	1,058339553
221452_s_at	TMEM14B	5,989276395	4,931222868	1,058053527
212176_at	PNISR	3,969866565	2,912181979	1,057684586
235492_at	RNF217	-1,212652659	-2,27001641	1,057363751
235995_at	PUM2	-1,212652659	-2,27001641	1,057363751
244681_at	NUP210P1	-1,212652659	-2,27001641	1,057363751
240303_at	TMC5	-1,212652659	-2,27001641	1,057363751
1554648_a_at	DUOXA1	-1,212652659	-2,27001641	1,057363751
240950_s_at	CCDC155	-1,212652659	-2,27001641	1,057363751
204468_s_at	TIE1	-1,212652659	-2,27001641	1,057363751
1560654_at	FLJ37201	-1,212652659	-2,27001641	1,057363751
243468_at	PATE1	-1,212652659	-2,27001641	1,057363751
233685_at	-	-1,212652659	-2,27001641	1,057363751
240909_at	-	-1,212652659	-2,27001641	1,057363751
244800_x_at	-	-1,212652659	-2,27001641	1,057363751
211910_at	-	-1,212652659	-2,27001641	1,057363751
219300_s_at	CNTNAP2	-1,212652659	-2,27001641	1,057363751
1567252_at	OR10D3	-1,212652659	-2,27001641	1,057363751
223801_s_at	APOL4	-1,212652659	-2,27001641	1,057363751
201127_s_at	ACLY	3,584942056	2,527778509	1,057163547
235084_x_at	TRIM38	2,053550345	0,996453274	1,057097071
230187_s_at	LOC100507100	-0,043925712	-1,100943374	1,057017662
206286_s_at	TDGF1 /// TDGF1P	-0,043925712	-1,100943374	1,057017662
234285_at	SYNGAP1	-0,043925712	-1,100943374	1,057017662
242255_at	WDR37	-0,043925712	-1,100943374	1,057017662
201538_s_at	DUSP3	-0,043925712	-1,100943374	1,057017662
210446_at	GATA1	-0,043925712	-1,100943374	1,057017662
238792_at	PCNX	-0,043925712	-1,100943374	1,057017662
211527_x_at	VEGFA	-0,043925712	-1,100943374	1,057017662
224911_s_at	DCBLD2	0,167727503	-0,889222211	1,056949714
244728_at	-	0,167727503	-0,889222211	1,056949714
229496_at	CLP1	0,167727503	-0,889222211	1,056949714
1555215_a_at	HRH3	0,167727503	-0,889222211	1,056949714
233418_at	LOC91450	0,167727503	-0,889222211	1,056949714
221935_s_at	EOGT	1,857989965	0,80114751	1,056842456
1566851_at	TRIM42	-0,609040214	-1,665791776	1,056751562
233097_x_at	-	-0,609040214	-1,665791776	1,056751562
1569156_at	ZNF846	-0,609040214	-1,665791776	1,056751562
1565729_at	-	-0,609040214	-1,665791776	1,056751562
206040_s_at	MAPK11	-0,609040214	-1,665791776	1,056751562
221631_at	CACNA1I	-0,609040214	-1,665791776	1,056751562
206161_s_at	SYT5	-0,609040214	-1,665791776	1,056751562
206304_at	MYBPH	-0,609040214	-1,665791776	1,056751562

1555277_a_at	SLC4A5	-0,609040214	-1,665791776	1,056751562
237792_at	-	-0,609040214	-1,665791776	1,056751562
220929_at	GALNT8	-0,609040214	-1,665791776	1,056751562
207289_at	MMP25	-0,609040214	-1,665791776	1,056751562
219305_x_at	FBXO2	-0,609040214	-1,665791776	1,056751562
1555486_a_at	PRR5L	-0,609040214	-1,665791776	1,056751562
205468_s_at	IRF5	-0,609040214	-1,665791776	1,056751562
1565661_x_at	FUT6	0,20740896	-0,84928999	1,05669895
234960_at	HIST1H4A /// HIST	0,20740896	-0,84928999	1,05669895
203372_s_at	SOCS2	0,244681185	-0,811769547	1,056450732
218379_at	RBM7	4,283600482	3,227213858	1,056386624
230243_at	TRMT10A	1,094118704	0,03784737	1,056271334
221131_at	A4GNT	-0,088974936	-1,145057014	1,056082079
205982_x_at	SFTPC	-0,088974936	-1,145057014	1,056082079
234356_at	DKFZP434K028	-0,088974936	-1,145057014	1,056082079
215454_x_at	SFTPC	-0,088974936	-1,145057014	1,056082079
239844_x_at	C1orf228	-0,088974936	-1,145057014	1,056082079
214334_x_at	DAZAP2	5,432035227	4,376059321	1,055975905
215540_at	-	-0,135065865	-1,19085694	1,055791075
1560870_a_at	-	-0,135065865	-1,19085694	1,055791075
1570239_a_at	CLNK	-0,135065865	-1,19085694	1,055791075
211826_s_at	AFF1	-0,135065865	-1,19085694	1,055791075
229289_at	FAM71E1	-0,135065865	-1,19085694	1,055791075
207788_s_at	SORBS3	-0,135065865	-1,19085694	1,055791075
1554175_at	CD300LB	-0,135065865	-1,19085694	1,055791075
220193_at	SH3D21	-0,135065865	-1,19085694	1,055791075
220296_at	-	-0,135065865	-1,19085694	1,055791075
1559265_at	SKIDA1	-0,994186487	-2,049747196	1,055560709
220541_at	MMP26	-0,994186487	-2,049747196	1,055560709
236743_at	AGPAT6	-0,994186487	-2,049747196	1,055560709
241406_at	FAM83H	-0,994186487	-2,049747196	1,055560709
231519_at	-	-0,994186487	-2,049747196	1,055560709
222321_at	AGTR2	-0,994186487	-2,049747196	1,055560709
1560896_a_at	-	-0,994186487	-2,049747196	1,055560709
242129_at	-	-0,994186487	-2,049747196	1,055560709
1556400_at	-	-0,994186487	-2,049747196	1,055560709
230911_at	SIX1	-0,994186487	-2,049747196	1,055560709
201380_at	CRTAP /// LOC100	1,898032047	0,842599219	1,055432828
213542_at	ZNF710	1,421391637	0,366031214	1,055360424
238968_at	-	-0,856259172	-1,911206569	1,054947397
1554496_at	RAD51B	-0,856259172	-1,911206569	1,054947397
1559169_at	LOC100507013	-0,856259172	-1,911206569	1,054947397
215733_x_at	CTAG2	-0,856259172	-1,911206569	1,054947397
1558965_at	PHF21A	-0,856259172	-1,911206569	1,054947397
235665_at	PTOV1	-0,856259172	-1,911206569	1,054947397
240279_at	-	-0,856259172	-1,911206569	1,054947397
210311_at	FGF5	-0,856259172	-1,911206569	1,054947397
216557_x_at	IGHA1 /// IGHD //	-0,856259172	-1,911206569	1,054947397
1559491_at	-	-0,856259172	-1,911206569	1,054947397
244827_at	-	-0,856259172	-1,911206569	1,054947397

213978_at	MTSS1L	-0,856259172	-1,911206569	1,054947397
240220_at	-	-0,856259172	-1,911206569	1,054947397
238216_at	LOC100506258	-0,856259172	-1,911206569	1,054947397
1562743_at	ZNF33B	-0,856259172	-1,911206569	1,054947397
238400_at	ENDOV	-0,856259172	-1,911206569	1,054947397
206229_x_at	PAX2	-0,856259172	-1,911206569	1,054947397
241212_at	-	-0,856259172	-1,911206569	1,054947397
222449_at	PMEPA1	-0,856259172	-1,911206569	1,054947397
232349_x_at	DCAF6	2,190140919	1,135235995	1,054904924
227568_at	HECTD2	0,764143511	-0,290694975	1,054838486
226569_s_at	CHTF18	1,116462765	0,061773582	1,054689183
206709_x_at	GPT	1,116462765	0,061773582	1,054689183
221606_s_at	HMGN5	4,666256427	3,612221266	1,054035161
213194_at	ROBO1	-1,802716385	-2,856570137	1,053853752
221133_s_at	CLDN18	-0,180990326	-1,234835326	1,053845
235797_x_at	HMCN2	-0,180990326	-1,234835326	1,053845
224265_s_at	ZAN	-0,180990326	-1,234835326	1,053845
218931_at	RAB17	-0,180990326	-1,234835326	1,053845
1555853_at	LOC100507463	-0,180990326	-1,234835326	1,053845
205915_x_at	GRIN1	-0,180990326	-1,234835326	1,053845
216152_at	-	-0,180990326	-1,234835326	1,053845
223693_s_at	RADIL	-0,180990326	-1,234835326	1,053845
1558825_s_at	LOC100653178 ///	-0,180990326	-1,234835326	1,053845
210419_at	BARX2	-0,180990326	-1,234835326	1,053845
217156_at	-	0,282358733	-0,771340337	1,05369907
243891_at	-	0,282358733	-0,771340337	1,05369907
242332_at	FOXF1-AS1	0,282358733	-0,771340337	1,05369907
213871_s_at	C6orf108	0,282358733	-0,771340337	1,05369907
216176_at	HCRP1	0,282358733	-0,771340337	1,05369907
1556971_a_at	-	0,319287178	-0,734355396	1,053642574
1558116_x_at	RECK	0,319287178	-0,734355396	1,053642574
1557120_at	EEF1A1	0,319287178	-0,734355396	1,053642574
220576_at	PGAP1	0,319287178	-0,734355396	1,053642574
238953_at	LOC100506325	1,671397663	0,61779983	1,053597833
224720_at	MIB1	3,513985141	2,460974054	1,053011087
40359_at	RASSF7	2,719190887	1,666816979	1,052373909
208100_x_at	SEMA6C	0,39105295	-0,661178575	1,052231525
209559_at	HIP1R	0,39105295	-0,661178575	1,052231525
226408_at	TEAD2	-0,230432956	-1,282520722	1,052087766
229443_at	MNF1	-0,230432956	-1,282520722	1,052087766
231926_at	EPS15L1	-0,230432956	-1,282520722	1,052087766
244666_at	-	-0,230432956	-1,282520722	1,052087766
230826_at	MMD2	-0,230432956	-1,282520722	1,052087766
228203_at	B3GNT1	-0,230432956	-1,282520722	1,052087766
223603_at	RNF112	-0,230432956	-1,282520722	1,052087766
217171_at	SMPD1	-0,230432956	-1,282520722	1,052087766
239304_at	MFSD4	-0,230432956	-1,282520722	1,052087766
234023_s_at	CENPJ	-0,230432956	-1,282520722	1,052087766
218419_s_at	TMUB2	1,476175815	0,424693306	1,051482509
1566177_at	-	1,476175815	0,424693306	1,051482509

231004_s_at	H1FX	0,820160788	-0,231045907	1,051206694
238035_at	SP3	2,752615529	1,701800759	1,050814769
213877_x_at	TCEB2	2,118167848	1,067365565	1,050802283
231550_at	XYLT2	1,493647653	0,443143223	1,05050443
235253_at	RAD1	2,75889404	1,709047448	1,049846592
227424_x_at	C21orf119	2,127965541	1,078619386	1,049346155
220096_at	-	0,847529938	-0,201789721	1,049319659
226964_at	TTBK2	0,847529938	-0,201789721	1,049319659
232609_at	CRB3	0,847529938	-0,201789721	1,049319659
200752_s_at	CAPN1	1,97249974	0,923203038	1,049296701
201576_s_at	GLB1	5,197470834	4,148511394	1,048959441
241942_at	PXDNL	-1,137208348	-2,186086603	1,048878255
214183_s_at	TKTL1	-1,137208348	-2,186086603	1,048878255
234558_at	-	-1,137208348	-2,186086603	1,048878255
204697_s_at	CHGA	-1,137208348	-2,186086603	1,048878255
210657_s_at	38231	-1,137208348	-2,186086603	1,048878255
1554694_at	CCNY	-1,137208348	-2,186086603	1,048878255
1561257_at	LOC286083	-1,137208348	-2,186086603	1,048878255
226141_at	CCDC149	-1,137208348	-2,186086603	1,048878255
1561373_at	PACRG-AS1	-1,137208348	-2,186086603	1,048878255
205336_at	PVALB	-1,137208348	-2,186086603	1,048878255
214477_at	MLLT1	-1,137208348	-2,186086603	1,048878255
210019_at	CALML3	-1,137208348	-2,186086603	1,048878255
229340_at	NKAPL	-1,137208348	-2,186086603	1,048878255
234511_at	ANKRD60	-1,137208348	-2,186086603	1,048878255
243758_at	CCDC37	-1,137208348	-2,186086603	1,048878255
210911_at	ID2B	-1,137208348	-2,186086603	1,048878255
216940_x_at	YBX1	-1,137208348	-2,186086603	1,048878255
213049_at	RALGAPA1	3,426291319	2,37755543	1,048735889
236637_at	TAS2R14	-1,452713826	-2,501359113	1,048645287
210302_s_at	MAB21L2	-1,452713826	-2,501359113	1,048645287
1564840_at	-	-1,452713826	-2,501359113	1,048645287
208421_at	-	-1,452713826	-2,501359113	1,048645287
1562761_at	NMRK1	-1,452713826	-2,501359113	1,048645287
204830_x_at	PSG5	-1,452713826	-2,501359113	1,048645287
216160_at	-	-1,452713826	-2,501359113	1,048645287
203597_s_at	WBP4	2,784898907	1,736450458	1,04844845
230800_at	ADCY4	0,425036312	-0,623254098	1,048290411
227296_at	MFSD3	0,425036312	-0,623254098	1,048290411
233103_at	-	0,425036312	-0,623254098	1,048290411
219347_at	NUDT15	3,798440091	2,750185881	1,04825421
219218_at	BAHCC1	0,458905032	-0,589001171	1,047906203
219810_at	VCPIP1	0,458905032	-0,589001171	1,047906203
224934_at	YIPF5	5,067458371	4,019608479	1,047849892
202724_s_at	FOXO1	2,019346222	0,971607464	1,047738757
228620_at	-	2,678352494	1,630734622	1,047617872
227973_at	C2orf69	3,353217129	2,305621062	1,047596067
215628_x_at	-	2,031158928	0,98381571	1,047343218
1555569_a_at	KCTD7 /// RABGEF	-0,280273599	-1,32740143	1,047127831
219318_x_at	MED31	-0,280273599	-1,32740143	1,047127831

232811_x_at	PRICKLE1	-0,280273599	-1,32740143	1,047127831
236689_at	RNF151	-0,280273599	-1,32740143	1,047127831
219107_at	BCAN	-0,280273599	-1,32740143	1,047127831
1555953_at	SLC19A1	-0,280273599	-1,32740143	1,047127831
207215_at	GSTTP1	-0,280273599	-1,32740143	1,047127831
206122_at	SOX15	-0,280273599	-1,32740143	1,047127831
220233_at	FBXO17 /// SARS2	-0,280273599	-1,32740143	1,047127831
225212_at	SLC25A25	2,427650693	1,380552339	1,047098354
223684_s_at	SMUG1	2,267141818	1,220470658	1,04667116
221953_s_at	-	1,561816652	0,515457602	1,04635905
239932_at	-	-0,66934516	-1,715576125	1,046230966
216934_at	-	-0,66934516	-1,715576125	1,046230966
240996_at	-	-0,66934516	-1,715576125	1,046230966
219140_s_at	RBP4	-0,66934516	-1,715576125	1,046230966
240624_x_at	LOC100129463	-0,66934516	-1,715576125	1,046230966
240479_at	HS3ST5	-0,66934516	-1,715576125	1,046230966
213768_s_at	ASCL1	-0,66934516	-1,715576125	1,046230966
1555678_at	ST3GAL3	-0,66934516	-1,715576125	1,046230966
1561917_at	-	-0,66934516	-1,715576125	1,046230966
244874_at	CHST15	-0,66934516	-1,715576125	1,046230966
232173_at	CLEC2L	-0,66934516	-1,715576125	1,046230966
214688_at	TLE4	-0,66934516	-1,715576125	1,046230966
233060_at	ZMIZ1	-0,66934516	-1,715576125	1,046230966
1560686_at	ITGAD	-0,66934516	-1,715576125	1,046230966
225241_at	CCDC80	-0,66934516	-1,715576125	1,046230966
206390_x_at	PF4	-0,66934516	-1,715576125	1,046230966
204673_at	MUC2	-0,66934516	-1,715576125	1,046230966
235616_at	TSHZ2	-0,66934516	-1,715576125	1,046230966
214175_x_at	PDLIM4	-0,66934516	-1,715576125	1,046230966
218740_s_at	CDK5RAP3	4,407522442	3,361641325	1,045881117
226912_at	ZDHHC23	1,246401134	0,20062106	1,045780074
243707_at	-	1,578719607	0,533258442	1,045461165
225483_at	VPS26B	2,545254728	1,500059916	1,045194811
210950_s_at	FDFT1	6,738260235	5,693106425	1,04515381
1563641_a_at	SNX20	0,900065578	-0,144765583	1,044831161
227534_at	AAED1	3,603313992	2,558652146	1,044661846
224754_at	SP1	3,426291319	2,381744287	1,044547032
204527_at	MYO5A	2,179708195	1,135235995	1,0444722
201004_at	SSR4	6,705519037	5,661833529	1,043685508
220192_x_at	SPDEF	0,926325262	-0,116767475	1,043092737
219292_at	THAP1	3,190097062	2,147194726	1,042902335
220991_s_at	RNF32	0,524545436	-0,518232988	1,042778425
225426_at	PPP6C	2,613382867	1,571112806	1,042270061
205323_s_at	MTF1	1,610353504	0,568155442	1,042198062
217092_x_at	-	6,655134208	5,613228455	1,041905754
212475_at	AVL9	1,625482993	0,583607503	1,04187549
211587_x_at	CHRNA3	0,95222556	-0,089635713	1,041861273
238859_at	-	0,95222556	-0,089635713	1,041861273
214625_s_at	MINK1	0,556428218	-0,484558493	1,040986711
207353_s_at	HMX1	0,556428218	-0,484558493	1,040986711

205449_at	SAC3D1	4,377039749	3,336180482	1,040859267
222138_s_at	WDR13	2,804738618	1,76396664	1,040771978
223166_x_at	RABL6	2,356619952	1,315897482	1,040722469
233258_at	-	-0,332405896	-1,372734086	1,04032819
1561749_at	-	-0,332405896	-1,372734086	1,04032819
234722_x_at	OBP2B	-0,332405896	-1,372734086	1,04032819
214701_s_at	FN1	-0,332405896	-1,372734086	1,04032819
210301_at	XDH	-0,332405896	-1,372734086	1,04032819
239036_at	EIF2C1	-0,332405896	-1,372734086	1,04032819
230745_s_at	TOX3	-0,332405896	-1,372734086	1,04032819
226450_at	INSR	-0,332405896	-1,372734086	1,04032819
1552631_a_at	MAP3K6	-0,332405896	-1,372734086	1,04032819
213885_at	TRIM3	-0,332405896	-1,372734086	1,04032819
209113_s_at	HMG20B	2,38349585	1,343200523	1,040295327
206106_at	MAPK12	1,326463531	0,286279868	1,040183663
212860_at	ZDHHC18	2,365121417	1,324949741	1,040171676
225692_at	CAMTA1	5,080169333	4,040084244	1,040085089
220386_s_at	EML4	2,2388565	1,199296812	1,039559688
230730_at	SGCD	-1,712732543	-2,752097204	1,039364661
204737_s_at	MYH6 /// MYH7	-1,712732543	-2,752097204	1,039364661
1555689_at	CD80	-1,712732543	-2,752097204	1,039364661
227293_at	-	2,530390387	1,491644074	1,038746313
205063_at	GEMIN2	2,530390387	1,491644074	1,038746313
203039_s_at	NDUFS1	4,400128426	3,361641325	1,038487101
223068_at	EML4	4,207481512	3,169167796	1,038313715
58994_at	CC2D1A	0,188886738	-0,84928999	1,038176728
230581_at	-	-0,384193355	-1,422257026	1,038063671
208593_x_at	CRHR1	-0,384193355	-1,422257026	1,038063671
206763_at	FKBP6	-0,384193355	-1,422257026	1,038063671
205459_s_at	NPAS2	-0,384193355	-1,422257026	1,038063671
215995_x_at	-	-0,384193355	-1,422257026	1,038063671
210912_x_at	GSTM4	1,364555912	0,326777209	1,037778703
236384_at	C17orf85	1,364555912	0,326777209	1,037778703
230945_at	-	1,364555912	0,326777209	1,037778703
217966_s_at	FAM129A	4,214272326	3,176868665	1,037403661
221771_s_at	MPHOSPH8	2,127965541	1,090640407	1,037325134
231251_at	WIPF2	1,000459215	-0,036543687	1,037002902
203617_x_at	ELK1	2,285848435	1,249194639	1,036653797
222975_s_at	CSDE1	6,942086845	5,905690662	1,036396182
224660_at	PIGY	5,743695407	4,707623164	1,036072243
203303_at	DYNLT3	4,895653873	3,859612828	1,036041045
210266_s_at	TRIM33	4,561620178	3,525687129	1,035933049
224980_at	LEMD2	1,421391637	0,385582632	1,035809005
1554451_s_at	DNAJC14	1,421391637	0,385582632	1,035809005
203448_s_at	TERF1	2,884644843	1,848871029	1,035773814
1555906_s_at	C3orf23	3,6787904	2,64347734	1,03531306
211509_s_at	RTN4	6,250064314	5,214762496	1,035301817
212849_at	AXIN1	1,024284941	-0,011001236	1,035286177
230382_at	SLC11A1	1,024284941	-0,011001236	1,035286177
225849_s_at	SFT2D1	6,338040866	5,302902418	1,035138448

1556046_a_at	LINC00599 /// MIR	0,648195588	-0,386896102	1,03509169
1559705_s_at	PHKA2	0,648195588	-0,386896102	1,03509169
211433_x_at	FAM214B	0,648195588	-0,386896102	1,03509169
244278_at	-	-0,437449947	-1,471833228	1,03438328
236912_at	-	-0,437449947	-1,471833228	1,03438328
1560785_at	DYRK3	-0,437449947	-1,471833228	1,03438328
233212_at	-	-0,437449947	-1,471833228	1,03438328
1552456_a_at	MBD3L2	-0,437449947	-1,471833228	1,03438328
213412_at	TJP3	-0,437449947	-1,471833228	1,03438328
224001_at	FAM135B	-0,437449947	-1,471833228	1,03438328
221301_at	VWA7	-0,437449947	-1,471833228	1,03438328
206454_s_at	RHO	-0,437449947	-1,471833228	1,03438328
207389_at	GP1BA	-0,437449947	-1,471833228	1,03438328
230358_at	ZER1	-0,437449947	-1,471833228	1,03438328
1558334_a_at	C22orf15	-0,437449947	-1,471833228	1,03438328
211175_at	GPR45	-0,437449947	-1,471833228	1,03438328
229636_at	LOC100506802	-0,437449947	-1,471833228	1,03438328
230222_at	-	-0,437449947	-1,471833228	1,03438328
206358_at	PRM1	-0,437449947	-1,471833228	1,03438328
234580_at	TMEM106A	-0,437449947	-1,471833228	1,03438328
220152_at	C10orf95	-0,437449947	-1,471833228	1,03438328
238044_at	-	-0,437449947	-1,471833228	1,03438328
218905_at	INTS8	5,090512962	4,056211353	1,034301609
243601_at	LOC285957	2,791258167	1,757155322	1,034102845
212597_s_at	HMGXB4	3,055618382	2,021531611	1,034086771
237957_at	-	-1,06282519	-2,09678811	1,03396292
1554210_at	ZCCHC13	-1,06282519	-2,09678811	1,03396292
244017_at	-	-1,06282519	-2,09678811	1,03396292
236745_at	CCDC78	-1,06282519	-2,09678811	1,03396292
200930_s_at	VCL	-1,06282519	-2,09678811	1,03396292
214419_s_at	CYP2C9	-1,06282519	-2,09678811	1,03396292
227695_at	GLYATL1 /// LOC1(	-1,06282519	-2,09678811	1,03396292
204734_at	KRT15	-1,06282519	-2,09678811	1,03396292
221948_s_at	KLHL22	-1,06282519	-2,09678811	1,03396292
207955_at	CCL27	-1,06282519	-2,09678811	1,03396292
216556_x_at	-	-1,06282519	-2,09678811	1,03396292
206736_x_at	CHRNA4	-1,06282519	-2,09678811	1,03396292
219167_at	RASL12	-1,06282519	-2,09678811	1,03396292
1555746_at	CD79B	-1,06282519	-2,09678811	1,03396292
202863_at	SP100	3,269673646	2,235857731	1,033815915
1562772_a_at	DAND5	-0,730013898	-1,763664074	1,033650176
211848_s_at	CEACAM7	-0,730013898	-1,763664074	1,033650176
241812_at	SPATS2L	-0,730013898	-1,763664074	1,033650176
233733_at	-	-0,730013898	-1,763664074	1,033650176
227343_at	-	-0,730013898	-1,763664074	1,033650176
207473_at	MLN	-0,730013898	-1,763664074	1,033650176
217593_at	ZSCAN18	-0,730013898	-1,763664074	1,033650176
203898_at	CRCP	2,649978302	1,616354037	1,033624266
222494_at	FOXN3	2,804738618	1,771157334	1,033581284
202101_s_at	RALB	2,920736393	1,887158627	1,033577766

1569110_x_at	LOC728613	4,080162946	3,046977905	1,033185041
223150_s_at	PTPN23	1,476175815	0,443143223	1,033032592
208322_s_at	ST3GAL1	3,065977723	2,03313004	1,032847683
235303_at	TRMT10B	-0,924000698	-1,956681069	1,032680372
244445_at	-	-0,924000698	-1,956681069	1,032680372
206001_at	NPY	-0,924000698	-1,956681069	1,032680372
214466_at	GJA5	-0,924000698	-1,956681069	1,032680372
220875_at	-	-0,924000698	-1,956681069	1,032680372
1557682_a_at	LOC284688	-0,924000698	-1,956681069	1,032680372
215426_at	ZCCHC14	-0,924000698	-1,956681069	1,032680372
1560633_a_at	C21orf91-OT1	-0,924000698	-1,956681069	1,032680372
202237_at	NNMT	-0,924000698	-1,956681069	1,032680372
236819_at	PTBP1	-0,924000698	-1,956681069	1,032680372
238284_at	LOC100506154	-1,537851782	-2,570522741	1,032670959
1562541_at	-	-1,537851782	-2,570522741	1,032670959
237971_at	-	-1,537851782	-2,570522741	1,032670959
203407_at	PPL	-1,537851782	-2,570522741	1,032670959
1559303_at	LOC100505835	-1,537851782	-2,570522741	1,032670959
215759_at	ANKRD53	-1,537851782	-2,570522741	1,032670959
244504_x_at	ARF1 /// MIR3620	0,677745787	-0,354781582	1,032527369
219579_at	RAB3IL1	0,677745787	-0,354781582	1,032527369
223576_at	C6orf203	3,603313992	2,570802332	1,032511661
208963_x_at	FADS1 /// MIR190	2,33082239	1,298349073	1,032473316
200749_at	RAN	5,13915997	4,107243663	1,031916307
221486_at	ENSA	3,378744007	2,346979857	1,03176415
209545_s_at	RIPK2	3,228532861	2,197404429	1,031128432
229861_at	-	2,374261973	1,343200523	1,03106145
229913_at	C7orf61	1,116462765	0,085534992	1,030927772
202146_at	IFRD1	4,490240735	3,459443789	1,030796946
207435_s_at	SRRM2	2,21987062	1,189078962	1,030791658
231901_at	C19orf52	2,229377375	1,199296812	1,030080563
223898_at	ZNF670	1,139057614	0,109458907	1,029598708
238547_at	HEXIM2	1,139057614	0,109458907	1,029598708
208802_at	SRP72	3,592169709	2,562621495	1,029548214
230413_s_at	-	3,03433073	2,004917973	1,029412757
244714_at	-	-1,802716385	-2,832036647	1,029320262
224034_at	-	-1,802716385	-2,832036647	1,029320262
1565805_at	-	-1,802716385	-2,832036647	1,029320262
223300_s_at	CCDC82	3,983349065	2,954216214	1,029132852
208581_x_at	MT1X	4,913150425	3,884351449	1,028798976
227684_at	S1PR2	1,578719607	0,550017041	1,028702566
202781_s_at	INPP5K	2,436165655	1,408220608	1,027945047
229888_at	C12orf60	1,160996507	0,133104519	1,027891988
209274_s_at	ISCA1	4,116324812	3,08892491	1,027399902
244410_at	PKHD1	-0,49284695	-1,520022123	1,027175173
1555321_at	ACOT11	-0,49284695	-1,520022123	1,027175173
1553977_a_at	CYP39A1	-0,49284695	-1,520022123	1,027175173
213498_at	CREB3L1	-0,49284695	-1,520022123	1,027175173
1553761_at	PRR14L	-0,49284695	-1,520022123	1,027175173
234214_at	-	-0,49284695	-1,520022123	1,027175173

220455_at	SLC16A8	-0,49284695	-1,520022123	1,027175173
1561626_at	-	-0,49284695	-1,520022123	1,027175173
227329_at	ZBTB46	-0,49284695	-1,520022123	1,027175173
212265_at	QKI	5,149366368	4,122411329	1,026955039
204182_s_at	ZBTB43	1,610353504	0,583607503	1,026746001
224785_at	FAM100B	3,336134047	2,309925448	1,026208599
1554239_s_at	ZADH2	3,076375717	2,050286491	1,026089227
208113_x_at	PABPC3	7,966618222	6,940925463	1,025692759
200085_s_at	TCEB2	6,600735382	5,575543013	1,025192369
201238_s_at	CAPZA2	5,452880877	4,427996682	1,024884196
226790_at	MORN2	1,97249974	0,947673886	1,024825854
223171_at	DYM	2,705643918	1,680963921	1,024679997
237075_at	LOC100506762	1,225123479	0,20062106	1,024502419
219364_at	DHX58	1,225123479	0,20062106	1,024502419
216210_x_at	TRIOBP	3,493994216	2,469496389	1,024497827
224685_at	MLLT4	3,023747815	1,999354538	1,024393277
238021_s_at	CRNDE	5,308935994	4,28465024	1,024285754
222785_x_at	C11orf1	3,957687264	2,933477664	1,0242096
201848_s_at	BNIP3	2,507817181	1,483698568	1,024118613
222065_s_at	FLII	3,551632175	2,527778509	1,023853666
220495_s_at	TXNDC15	4,200383271	3,176868665	1,023514606
214048_at	MBD4	2,348178434	1,324949741	1,023228693
217427_s_at	HIRA	4,550683059	3,527465373	1,023217686
205321_at	EIF2S3	4,613033266	3,589866014	1,023167253
1562729_at	-	0,791908897	-0,231045907	1,022954804
236172_at	LTB4R	0,791908897	-0,231045907	1,022954804
202664_at	WIPF1	5,28227713	4,25950843	1,0227687
234997_x_at	-	3,200057698	2,177710147	1,022347551
202861_at	PER1	1,287793882	0,265475485	1,022318397
240910_at	-	-1,289805289	-2,312054429	1,02224914
207935_s_at	KRT13	-1,289805289	-2,312054429	1,02224914
234689_at	PTCHD4	-1,289805289	-2,312054429	1,02224914
234429_at	LINC00476	-1,289805289	-2,312054429	1,02224914
237623_at	CST3	-1,289805289	-2,312054429	1,02224914
1553070_a_at	MYOZ3	-1,289805289	-2,312054429	1,02224914
223935_at	TRPM5	-1,289805289	-2,312054429	1,02224914
206363_at	MAF	-1,289805289	-2,312054429	1,02224914
234438_at	-	-1,289805289	-2,312054429	1,02224914
1570162_at	-	-1,369772723	-2,391863529	1,022090806
237743_at	-	-1,369772723	-2,391863529	1,022090806
242846_at	-	-1,369772723	-2,391863529	1,022090806
244316_at	C17orf98	-1,369772723	-2,391863529	1,022090806
215598_at	TTC12	-1,369772723	-2,391863529	1,022090806
206947_at	B3GALT5	-1,369772723	-2,391863529	1,022090806
1559065_a_at	CLEC4G	-1,369772723	-2,391863529	1,022090806
242132_x_at	-	-1,369772723	-2,391863529	1,022090806
210921_at	-	-1,369772723	-2,391863529	1,022090806
206312_at	GUCY2C	-1,369772723	-2,391863529	1,022090806
1557296_at	FLJ12825	-1,369772723	-2,391863529	1,022090806
1557709_at	-	-1,369772723	-2,391863529	1,022090806

232292_at	-	-1,369772723	-2,391863529	1,022090806
221636_s_at	MARC2	-1,369772723	-2,391863529	1,022090806
243227_at	-	-1,369772723	-2,391863529	1,022090806
223708_at	C1QTNF4	-1,369772723	-2,391863529	1,022090806
239141_at	LOC100126784	-1,369772723	-2,391863529	1,022090806
234116_at	GJD4	-1,369772723	-2,391863529	1,022090806
213985_s_at	C19orf6	-1,369772723	-2,391863529	1,022090806
213349_at	TMCC1	0,820160788	-0,201789721	1,021950509
211217_s_at	KCNQ1	0,820160788	-0,201789721	1,021950509
218620_s_at	HEMK1	2,537731447	1,516067292	1,021664156
213062_at	NTAN1	3,223556135	2,20251271	1,021043425
223187_s_at	ORMDL1	4,631436665	3,610508368	1,020928297
202343_x_at	COX5B	6,286057073	5,265393386	1,020663687
215460_x_at	BRD1	2,943764123	1,923216608	1,020547515
213289_at	APOOL	2,063971513	1,043469112	1,020502401
1554615_at	-	-0,791294935	-1,811780055	1,02048512
239220_at	-	-0,791294935	-1,811780055	1,02048512
238377_s_at	-	-0,791294935	-1,811780055	1,02048512
206863_x_at	-	-0,791294935	-1,811780055	1,02048512
220499_at	FNDC8	-0,791294935	-1,811780055	1,02048512
241196_at	-	-0,791294935	-1,811780055	1,02048512
210735_s_at	CA12	-0,791294935	-1,811780055	1,02048512
233432_at	LINC00598	-0,791294935	-1,811780055	1,02048512
230709_x_at	ZBTB7A	-0,791294935	-1,811780055	1,02048512
228720_at	SORCS2	-0,791294935	-1,811780055	1,02048512
208435_s_at	AQP6	-0,791294935	-1,811780055	1,02048512
216984_x_at	CKAP2 /// IGLC1 //	-0,791294935	-1,811780055	1,02048512
202878_s_at	CD93	-0,791294935	-1,811780055	1,02048512
206801_at	NPPB	-0,791294935	-1,811780055	1,02048512
1554541_a_at	GPRIN2	-0,791294935	-1,811780055	1,02048512
239689_at	-	-0,791294935	-1,811780055	1,02048512
200638_s_at	YWHAZ	6,841994896	5,821589498	1,020405398
228539_at	-	0,847529938	-0,172194225	1,019724162
1560316_s_at	GLCCI1	0,847529938	-0,172194225	1,019724162
1566974_at	-	0,847529938	-0,172194225	1,019724162
238027_at	SPATA24	0,847529938	-0,172194225	1,019724162
216186_at	-	0,847529938	-0,172194225	1,019724162
1552892_at	TNFRSF13C	0,20740896	-0,811769547	1,019178507
231422_x_at	TYSND1	0,20740896	-0,811769547	1,019178507
226630_at	MIS18BP1	3,029186714	2,010406245	1,01878047
228572_at	-	0,873789652	-0,144765583	1,018555235
1560213_at	HCP5B	0,045517965	-0,972893339	1,018411303
226704_at	UBE2J2	0,045517965	-0,972893339	1,018411303
233696_at	-	0,045517965	-0,972893339	1,018411303
232897_at	FLJ20444	0,045517965	-0,972893339	1,018411303
230035_at	BOC	0,045517965	-0,972893339	1,018411303
212435_at	TRIM33	3,945594088	2,927197469	1,018396619
218747_s_at	TAPBPL	2,444186596	1,426280426	1,01790617
206921_at	GLE1	0,08619576	-0,931539147	1,017734907
214324_at	GP2	0,08619576	-0,931539147	1,017734907

219011_at	PLEKHA4	0,08619576	-0,931539147	1,017734907
1556111_s_at	-	0,08619576	-0,931539147	1,017734907
235603_at	HNRNPU	0,08619576	-0,931539147	1,017734907
232557_at	UBAP1L	0,08619576	-0,931539147	1,017734907
205144_at	MFSD7	0,08619576	-0,931539147	1,017734907
224606_at	KLF6	3,710746795	2,693044596	1,017702199
212147_at	SMG5	1,760255626	0,742671819	1,017583807
202394_s_at	ABCF3	1,760255626	0,742671819	1,017583807
202650_s_at	KIAA0195	1,775035811	0,757638486	1,017397325
204598_at	UBOX5	1,775035811	0,757638486	1,017397325
218791_s_at	C15orf29	1,775035811	0,757638486	1,017397325
234934_at	-	-0,551284523	-1,568385657	1,017101134
231451_s_at	-	-0,551284523	-1,568385657	1,017101134
206428_s_at	-	-0,551284523	-1,568385657	1,017101134
1566824_at	-	-0,551284523	-1,568385657	1,017101134
233146_at	KNDC1	-0,551284523	-1,568385657	1,017101134
241325_at	LOC100507408	-0,551284523	-1,568385657	1,017101134
1555377_at	OR4D2	-0,551284523	-1,568385657	1,017101134
1559477_s_at	MEIS1	-0,551284523	-1,568385657	1,017101134
208572_at	HIST3H3	-0,551284523	-1,568385657	1,017101134
211130_x_at	EDA	-0,551284523	-1,568385657	1,017101134
222083_at	GLYAT	-0,551284523	-1,568385657	1,017101134
207880_at	ADAM11	-0,551284523	-1,568385657	1,017101134
206904_at	MATN1	-0,551284523	-1,568385657	1,017101134
205567_at	CHST1	-0,551284523	-1,568385657	1,017101134
225977_at	PCDH18	-0,551284523	-1,568385657	1,017101134
231419_at	LOC100505555	-0,551284523	-1,568385657	1,017101134
228615_at	LOC286161	-0,551284523	-1,568385657	1,017101134
214024_s_at	DGCR6L	-0,551284523	-1,568385657	1,017101134
231691_at	C3P1	-0,551284523	-1,568385657	1,017101134
236876_at	H1FNT	-0,551284523	-1,568385657	1,017101134
1555078_at	ZNF843	-0,551284523	-1,568385657	1,017101134
216490_x_at	-	0,167727503	-0,84928999	1,017017493
1554749_s_at	CLCNKB	0,167727503	-0,84928999	1,017017493
241556_at	-	0,167727503	-0,84928999	1,017017493
1562603_at	RAD51L3-RFFL ///	0,167727503	-0,84928999	1,017017493
213103_at	STARD13	0,167727503	-0,84928999	1,017017493
1568760_at	MYH11	0,127732831	-0,889222211	1,016955041
213770_at	KSR1	0,127732831	-0,889222211	1,016955041
222012_at	DCAF15	0,127732831	-0,889222211	1,016955041
1556468_at	-	0,127732831	-0,889222211	1,016955041
239627_at	-	0,127732831	-0,889222211	1,016955041
233230_s_at	SLAIN2	0,127732831	-0,889222211	1,016955041
206341_at	IL2RA	0,127732831	-0,889222211	1,016955041
222892_s_at	TMEM40	0,355751005	-0,661178575	1,01692958
230311_s_at	PRDM6	0,900065578	-0,116767475	1,016833053
230351_at	LOC283481	0,282358733	-0,734355396	1,016714129
1556928_at	-	0,282358733	-0,734355396	1,016714129
1560430_at	NKPD1	0,282358733	-0,734355396	1,016714129
226506_at	THSD4	0,282358733	-0,734355396	1,016714129

216949_s_at	PKD1	0,282358733	-0,734355396	1,016714129
243986_at	LINC00355	-1,212652659	-2,229233437	1,016580778
228715_at	ZCCHC12	-1,212652659	-2,229233437	1,016580778
234817_at	-	-1,212652659	-2,229233437	1,016580778
234478_at	-	-1,212652659	-2,229233437	1,016580778
240844_at	-	-1,212652659	-2,229233437	1,016580778
240045_at	-	-1,212652659	-2,229233437	1,016580778
207116_s_at	GAPDHS	-1,212652659	-2,229233437	1,016580778
220049_s_at	PDCD1LG2	-1,212652659	-2,229233437	1,016580778
216059_at	PAX3	-1,212652659	-2,229233437	1,016580778
240293_at	CCDC153	-1,212652659	-2,229233437	1,016580778
208208_at	MYH13	-1,212652659	-2,229233437	1,016580778
1553817_at	LOC100653171 ///	-1,212652659	-2,229233437	1,016580778
230154_at	WAC	0,001114523	-1,015398016	1,016512539
1552293_at	TMEM196	0,001114523	-1,015398016	1,016512539
234773_x_at	-	0,001114523	-1,015398016	1,016512539
216739_at	-	0,001114523	-1,015398016	1,016512539
236199_at	-	0,001114523	-1,015398016	1,016512539
203766_s_at	LMOD1	0,001114523	-1,015398016	1,016512539
239658_at	-	0,001114523	-1,015398016	1,016512539
216143_at	-	0,001114523	-1,015398016	1,016512539
1567627_at	CD74	0,001114523	-1,015398016	1,016512539
228691_at	-	0,001114523	-1,015398016	1,016512539
230019_s_at	PTCHD2	0,001114523	-1,015398016	1,016512539
213269_at	ZNF248	2,107136847	1,090640407	1,01649644
1565672_at	-	0,319287178	-0,697182596	1,016469774
221052_at	TDRKH	0,319287178	-0,697182596	1,016469774
204928_s_at	SLC10A3	2,118167848	1,101780212	1,016387637
226902_at	USP13	2,507817181	1,491644074	1,016173108
208647_at	FDFT1	7,03840558	6,022248861	1,016156718
201864_at	GDI1	3,060716358	2,044580955	1,016135403
217637_at	-	0,244681185	-0,771340337	1,016021522
207968_s_at	MEF2C	0,244681185	-0,771340337	1,016021522
222220_s_at	TSNAXIP1	0,244681185	-0,771340337	1,016021522
201962_s_at	RNF41	1,816938997	0,80114751	1,015791487
212349_at	POFUT1	1,830468611	0,815200271	1,01526834
230509_at	SNX22	0,39105295	-0,623254098	1,014307049
235226_at	CDK19	0,425036312	-0,589001171	1,014037484
201297_s_at	MOB1A	3,273854609	2,260052727	1,013801882
216085_at	DKFZP434C153	-1,452713826	-2,466349414	1,013635588
1570528_at	XYLT2	-1,452713826	-2,466349414	1,013635588
1553072_at	BNIPL	-1,452713826	-2,466349414	1,013635588
1553651_at	C18orf54	-1,452713826	-2,466349414	1,013635588
1566832_at	TOP1P2	-1,452713826	-2,466349414	1,013635588
224487_at	-	-1,452713826	-2,466349414	1,013635588
210913_at	CDH20	-1,452713826	-2,466349414	1,013635588
1557446_x_at	TREML3P	-1,452713826	-2,466349414	1,013635588
217327_at	-	-1,452713826	-2,466349414	1,013635588
231409_at	-	-1,452713826	-2,466349414	1,013635588
233352_at	-	-1,452713826	-2,466349414	1,013635588

208177_at	SLC34A1	0,458905032	-0,554696666	1,013601698
222370_x_at	-	0,458905032	-0,554696666	1,013601698
226792_s_at	KIFC2	1,510970097	0,497424714	1,013545383
228379_at	LOC128322 /// NU	2,356619952	1,343200523	1,013419428
243131_x_at	-	-0,043925712	-1,057301851	1,013376139
236772_s_at	-	-0,043925712	-1,057301851	1,013376139
223333_s_at	ANGPTL4	-0,043925712	-1,057301851	1,013376139
203826_s_at	PITPNM1	1,960819106	0,947673886	1,01314522
209273_s_at	ISCA1	3,327128828	2,314488991	1,012639837
218561_s_at	LYRM4	5,038901064	4,026485972	1,012415091
203276_at	LMNB1	4,774699135	3,762464514	1,012234621
230285_at	SVIP	3,209439373	2,197404429	1,012034943
216142_at	-	-0,088974936	-1,100943374	1,011968439
219674_s_at	HDLBP	-0,088974936	-1,100943374	1,011968439
223552_at	LRRC4	-0,088974936	-1,100943374	1,011968439
203052_at	C2	-0,088974936	-1,100943374	1,011968439
237887_at	-	1,000459215	-0,011001236	1,01146045
218335_x_at	TNIP2	3,228532861	2,2171284	1,011404461
206343_s_at	NRG1	-1,893506789	-2,904470894	1,010964105
1553084_at	STARD6	-0,994186487	-2,005029581	1,010843094
241080_at	-	-0,994186487	-2,005029581	1,010843094
228021_at	-	-0,994186487	-2,005029581	1,010843094
234049_at	FLJ10661	-0,994186487	-2,005029581	1,010843094
216004_s_at	PKNOX1	-0,994186487	-2,005029581	1,010843094
203535_at	S100A9	-0,994186487	-2,005029581	1,010843094
1558368_s_at	C1orf187	-0,994186487	-2,005029581	1,010843094
233890_at	-	-0,994186487	-2,005029581	1,010843094
1567286_at	OR5L2	-0,994186487	-2,005029581	1,010843094
1562330_s_at	CSMD1	-0,994186487	-2,005029581	1,010843094
220542_s_at	BPIFA1	-0,994186487	-2,005029581	1,010843094
224287_at	-	-0,994186487	-2,005029581	1,010843094
1564122_at	LINC00514 /// LOC	-0,994186487	-2,005029581	1,010843094
217255_at	-	-0,994186487	-2,005029581	1,010843094
220902_at	-	-0,994186487	-2,005029581	1,010843094
216725_at	DCLK2	-0,994186487	-2,005029581	1,010843094
208488_s_at	CR1	-0,994186487	-2,005029581	1,010843094
1553591_at	BCRP3	-0,994186487	-2,005029581	1,010843094
1555245_s_at	RP1L1	-0,994186487	-2,005029581	1,010843094
223663_at	CCDC88B	2,031158928	1,020514937	1,010643991
1556964_s_at	-	-1,624955693	-2,635549085	1,010593392
215911_x_at	ATP2B3	-1,624955693	-2,635549085	1,010593392
1570630_at	-	-1,624955693	-2,635549085	1,010593392
231459_at	LOC100506397	-1,624955693	-2,635549085	1,010593392
1557888_at	ZNF787	-1,624955693	-2,635549085	1,010593392
214860_at	SLC9A7	-1,624955693	-2,635549085	1,010593392
211032_at	COBLL1	-1,712732543	-2,723114724	1,010382181
233792_at	-	-1,712732543	-2,723114724	1,010382181
1552770_s_at	ZNF563	-1,712732543	-2,723114724	1,010382181
216714_at	CCL13	-1,712732543	-2,723114724	1,010382181
235556_at	CREBRF	0,491906512	-0,518232988	1,0101395

229423_at	CHEK1	0,491906512	-0,518232988	1,0101395
210357_s_at	SMOX	0,491906512	-0,518232988	1,0101395
1552822_at	TMX3	-0,135065865	-1,145057014	1,009991149
237404_at	-	-0,135065865	-1,145057014	1,009991149
237016_at	TMEM217	-0,135065865	-1,145057014	1,009991149
228214_at	SOX6	-0,135065865	-1,145057014	1,009991149
206630_at	TYR	-0,135065865	-1,145057014	1,009991149
243058_at	-	-0,135065865	-1,145057014	1,009991149
241540_at	-	-0,135065865	-1,145057014	1,009991149
235033_at	NPEPL1	-0,135065865	-1,145057014	1,009991149
233734_s_at	OSBPL5	-0,135065865	-1,145057014	1,009991149
219762_s_at	RPL36	7,353038745	6,343050251	1,009988494
214014_at	CDC42EP2	-0,180990326	-1,19085694	1,009866614
244595_at	-	-0,180990326	-1,19085694	1,009866614
210623_at	UBXN1	-0,180990326	-1,19085694	1,009866614
1562944_at	ZNF707	-0,180990326	-1,19085694	1,009866614
213270_at	MPP2	-0,180990326	-1,19085694	1,009866614
231928_at	HES2	-0,180990326	-1,19085694	1,009866614
204015_s_at	DUSP4	5,132261918	4,122411329	1,009850588
226581_at	ZFYVE20	2,739067176	1,72935563	1,009711546
224843_at	SLAIN2	1,071336699	0,061773582	1,009563116
217480_x_at	LOC100287723	1,047341799	0,03784737	1,009494428
219506_at	C1orf54	0,524545436	-0,484558493	1,00910393
1569745_at	LOC100505783	0,524545436	-0,484558493	1,00910393
223251_s_at	ANKRD10	4,855289601	3,846857035	1,008432567
212115_at	HN1L	4,272544367	3,264254379	1,008289988
61297_at	CASKIN2	-0,582787029	-1,591066665	1,008279636
221341_s_at	OR1D5	-0,609040214	-1,616924751	1,007884537
242582_at	-	-0,609040214	-1,616924751	1,007884537
1566835_at	-	-0,609040214	-1,616924751	1,007884537
205957_at	PLXNB3	-0,609040214	-1,616924751	1,007884537
205058_at	SLC26A1	-0,609040214	-1,616924751	1,007884537
206014_at	ACTL6B	-0,609040214	-1,616924751	1,007884537
208155_x_at	GAGE1 /// GAGE12	-0,609040214	-1,616924751	1,007884537
228890_at	ATOH8	-0,609040214	-1,616924751	1,007884537
207003_at	GUCA2A	-0,609040214	-1,616924751	1,007884537
229104_s_at	GPR39	-0,609040214	-1,616924751	1,007884537
233641_s_at	FAM167A	-0,609040214	-1,616924751	1,007884537
240748_at	-	-0,609040214	-1,616924751	1,007884537
209296_at	PPM1B	4,918218598	3,910368531	1,007850066
225287_s_at	TMEM55B	2,972517974	1,964670566	1,007847408
222559_s_at	RPRD1A	2,784898907	1,777135134	1,007763774
217929_s_at	KIAA0319L	2,507817181	1,500059916	1,007757265
218173_s_at	WHSC1L1	0,556428218	-0,45121326	1,007641478
224477_s_at	NUDT16L1	2,074841895	1,067365565	1,00747633
226914_at	ARPC5L	4,18872659	3,181622175	1,007104416
205214_at	STK17B	3,237994092	2,23115548	1,006838612
224809_x_at	TINF2	3,777053516	2,770696222	1,006357294
212124_at	ZMIZ1	3,716897585	2,710570973	1,006326612
1554411_at	CTNNB1	0,58788177	-0,418248858	1,006130628

227664_at	FLJ37453	0,58788177	-0,418248858	1,006130628
244858_at	TGIF1	0,58788177	-0,418248858	1,006130628
222256_s_at	JMJD7 /// JMJD7-F	0,58788177	-0,418248858	1,006130628
221176_x_at	-	0,58788177	-0,418248858	1,006130628
206054_at	KNG1	-0,856259172	-1,862269442	1,00601027
211156_at	CDKN2A	-0,856259172	-1,862269442	1,00601027
1570307_s_at	ST18	-0,856259172	-1,862269442	1,00601027
1566433_at	PLD4	-0,856259172	-1,862269442	1,00601027
1559458_at	LTBR	-0,856259172	-1,862269442	1,00601027
213905_x_at	BGN	-0,856259172	-1,862269442	1,00601027
221116_at	-	-0,856259172	-1,862269442	1,00601027
236914_at	LOC100506790	-0,856259172	-1,862269442	1,00601027
203423_at	RBP1	-0,856259172	-1,862269442	1,00601027
208537_at	S1PR2	-0,856259172	-1,862269442	1,00601027
212029_s_at	-	-0,856259172	-1,862269442	1,00601027
230658_at	SLC7A2	-0,856259172	-1,862269442	1,00601027
206397_x_at	CERS1 /// GDF1	-0,856259172	-1,862269442	1,00601027
236101_at	-	-0,856259172	-1,862269442	1,00601027
209897_s_at	SLIT2	-0,856259172	-1,862269442	1,00601027
215497_s_at	WDTC1	1,139057614	0,133104519	1,005953095
227037_at	PLD6	2,339581693	1,333668715	1,005912978
214779_s_at	SGSM3	1,640193838	0,634301128	1,00589271
221984_s_at	FAM134A	3,993377193	2,987805085	1,005572107
218487_at	ALAD	2,636121902	1,630734622	1,00538728
219653_at	LSM14B	2,107136847	1,101780212	1,005356635
226698_at	FCHSD1	0,618277321	-0,386896102	1,005173423
244190_at	THAP5	-1,137208348	-2,142286822	1,005078473
208360_s_at	ERVH-4	-1,137208348	-2,142286822	1,005078473
1561990_at	LOC157931	-1,137208348	-2,142286822	1,005078473
244571_s_at	-	-1,137208348	-2,142286822	1,005078473
229478_x_at	BIVM	-1,137208348	-2,142286822	1,005078473
241224_x_at	DSCR8	-1,137208348	-2,142286822	1,005078473
237970_at	-	-1,137208348	-2,142286822	1,005078473
213744_at	ATRNL1	-1,137208348	-2,142286822	1,005078473
203438_at	STC2	-1,137208348	-2,142286822	1,005078473
229306_at	C2CD4B	-1,137208348	-2,142286822	1,005078473
234461_at	RNF215	-1,137208348	-2,142286822	1,005078473
236418_at	TTLL10	-1,137208348	-2,142286822	1,005078473
240283_at	-	-1,137208348	-2,142286822	1,005078473
231354_at	LOC780529	-1,137208348	-2,142286822	1,005078473
210798_x_at	MASP2	-1,137208348	-2,142286822	1,005078473
231221_at	CLEC16A	-1,137208348	-2,142286822	1,005078473
232061_at	SDK2	-1,137208348	-2,142286822	1,005078473
205593_s_at	PDE9A	-1,137208348	-2,142286822	1,005078473
1555783_x_at	PQLC2	1,182925501	0,178191865	1,004733636
208656_s_at	CCNI	6,211851421	5,207201735	1,004649685
244189_at	TTC28-AS1	3,473999051	2,469496389	1,004502662
231681_x_at	HIST3H2A	-0,230432956	-1,234835326	1,00440237
207184_at	SLC6A13	-0,230432956	-1,234835326	1,00440237
229040_at	LOC100505746	-0,230432956	-1,234835326	1,00440237

217227_x_at	IGLV1-44	-0,230432956	-1,234835326	1,00440237
219835_at	PRDM8	-0,230432956	-1,234835326	1,00440237
231761_at	FFAR1	-0,230432956	-1,234835326	1,00440237
213085_s_at	WWC1	-0,230432956	-1,234835326	1,00440237
1562456_at	-	-0,230432956	-1,234835326	1,00440237
243677_at	GORASP1	-0,230432956	-1,234835326	1,00440237
226962_at	ZBTB41	2,605420261	1,601076277	1,004343984
36907_at	MVK	3,164128058	2,15984763	1,004280428
201832_s_at	USO1	5,037499265	4,033281307	1,004217957
1557472_a_at	FLJ30838	-1,802716385	-2,8064286	1,003712215
215506_s_at	DIRAS3	-1,802716385	-2,8064286	1,003712215
200789_at	ECH1	5,268057349	4,264419981	1,003637368
221867_at	N4BP1	1,20396005	0,20062106	1,00333899
234733_s_at	FANCM	1,20396005	0,20062106	1,00333899
204319_s_at	RGS10	4,976515905	3,973269509	1,003246395
216699_s_at	KLK1	0,648195588	-0,354781582	1,00297717
213244_at	SCAMP4	0,648195588	-0,354781582	1,00297717
1555650_at	KLHL17	0,648195588	-0,354781582	1,00297717
223378_at	GLIS2	1,745502609	0,742671819	1,00283079
209471_s_at	FNTA	5,182433607	4,179683441	1,002750166
226565_at	TMEM99	2,949541692	1,946903052	1,00263864
225738_at	RAPGEF1	2,410823487	1,408220608	1,002602879
211943_x_at	TPT1	8,607320174	7,60499906	1,002321114
1559715_at	LOC100507391	-0,280273599	-1,282520722	1,002247123
215247_at	-	-0,280273599	-1,282520722	1,002247123
234191_at	BCL2L14	-0,280273599	-1,282520722	1,002247123
231050_at	HRASLS5	-0,280273599	-1,282520722	1,002247123
205405_at	SEMA5A	-0,280273599	-1,282520722	1,002247123
234712_at	LINC00470	-0,280273599	-1,282520722	1,002247123
207763_at	S100A5	-0,280273599	-1,282520722	1,002247123
228074_at	ITPRIPL2	-0,280273599	-1,282520722	1,002247123
201126_s_at	MGAT1	2,401508182	1,399283506	1,002224676
212459_x_at	SUCLG2	4,109088344	3,106874588	1,002213756
202185_at	PLOD3	3,007119335	2,004917973	1,002201363
223626_x_at	IFI27L2	3,007119335	2,004917973	1,002201363
208912_s_at	CNP	3,366614157	2,364676067	1,00193809
210283_x_at	PAIP1	3,434684504	2,432792627	1,001891878
225605_at	TP53I13	1,267146969	0,265475485	1,001671484
47083_at	C7orf26	2,564523022	1,562873589	1,001649433
230486_at	-	1,802733148	0,80114751	1,001585638
211563_s_at	URI1	3,614400403	2,613615641	1,000784762
44673_at	SIGLEC1	0,048627298	-0,952068073	1,000695371
216100_s_at	TOR1AIP1	2,199941239	1,199296812	1,000644427
225121_at	TBC1D23	2,824160383	1,823960141	1,000200242
208370_s_at	RCAN1	1,936380191	0,936232181	1,00014801
1553046_s_at	GAL3ST2	0,677745787	-0,322340048	1,000085835
228700_at	CXorf38	2,855122714	1,855279162	0,999843552
230570_at	EIF3H	2,829929672	1,830129603	0,999800069
214437_s_at	SHMT2	4,471846553	3,472563131	0,999283422
229402_at	SAMD13	2,007220798	1,008210238	0,99901056

204565_at	ACOT13	4,706701013	3,707798923	0,99890209
205417_s_at	DAG1	3,081633533	2,082919929	0,998713604
223928_s_at	GUCA1C	-1,537851782	-2,536339282	0,998487499
1552752_a_at	CADM2	-1,537851782	-2,536339282	0,998487499
224020_at	MGC4473	-1,537851782	-2,536339282	0,998487499
234368_at	-	-1,537851782	-2,536339282	0,998487499
1556365_at	LY86-AS1	-1,537851782	-2,536339282	0,998487499
207959_s_at	DNAH9	-1,537851782	-2,536339282	0,998487499
209399_at	HLCS	0,707657549	-0,290694975	0,998352524
1553264_a_at	SYN1	0,707657549	-0,290694975	0,998352524
221634_at	LOC100287195 ///	3,340803206	2,34250725	0,998295956
225636_at	STAT2	3,529682801	2,531843788	0,997839013
48825_at	ING4	2,675018947	1,677522034	0,997496913
204108_at	NFYA	2,671492308	1,674071917	0,997420391
231378_at	-	1,440265662	0,443143223	0,997122439
200688_at	SF3B3	1,458255986	0,461385738	0,996870249
222489_s_at	WRNIP1	3,77412549	2,777528602	0,996596887
231317_at	-	0,735999505	-0,260526297	0,996525802
239565_at	LOC100128292	0,735999505	-0,260526297	0,996525802
214264_s_at	EFCAB11	3,012642182	2,016139502	0,99650268
205862_at	GREB1	-0,66934516	-1,665791776	0,996446616
1561219_x_at	-	-0,66934516	-1,665791776	0,996446616
244579_at	-	-0,66934516	-1,665791776	0,996446616
236452_at	-	-0,66934516	-1,665791776	0,996446616
214778_at	MEGF8	-0,66934516	-1,665791776	0,996446616
234365_at	-	-0,66934516	-1,665791776	0,996446616
217033_x_at	NTRK3	-0,66934516	-1,665791776	0,996446616
221284_s_at	SRC	-0,66934516	-1,665791776	0,996446616
226903_s_at	SLC6A10P /// SLC6	-0,66934516	-1,665791776	0,996446616
1564984_at	-	-0,66934516	-1,665791776	0,996446616
205253_at	PBX1	-0,66934516	-1,665791776	0,996446616
1569452_at	LOC692247	-0,66934516	-1,665791776	0,996446616
229226_at	-	-0,66934516	-1,665791776	0,996446616
1563657_at	PLXND1	-0,66934516	-1,665791776	0,996446616
224905_at	WDR26	3,804904984	2,809015785	0,995889199
214201_x_at	PRRC2A	2,085817135	1,090640407	0,995176728
1555363_s_at	LOC284440	-0,332405896	-1,32740143	0,994995534
231510_at	-	-0,332405896	-1,32740143	0,994995534
1562411_at	MYLK3	-0,332405896	-1,32740143	0,994995534
242902_at	CYMP	-0,332405896	-1,32740143	0,994995534
234386_s_at	-	-0,332405896	-1,32740143	0,994995534
240943_at	-	-0,332405896	-1,32740143	0,994995534
206868_at	STARD8	-0,332405896	-1,32740143	0,994995534
211846_s_at	PVRL1	-0,332405896	-1,32740143	0,994995534
207562_at	DGKQ	-0,332405896	-1,32740143	0,994995534
215651_at	-	-0,332405896	-1,32740143	0,994995534
200798_x_at	MCL1	4,290445502	3,295596077	0,994849425
228266_s_at	HDGFRP3	2,107136847	1,112548615	0,994588231
202003_s_at	ACAA2	5,137769258	4,143962533	0,993806725
231933_at	08.03.15	0,791908897	-0,201789721	0,993698618

240673_at	-	0,791908897	-0,201789721	0,993698618
1552256_a_at	SCARB1	3,171328694	2,177710147	0,993618548
208580_x_at	HIST1H4A /// HIST	2,38349585	1,390070295	0,993425555
202020_s_at	LANCL1	5,022025255	4,02889383	0,993131424
217191_x_at	-	0,820160788	-0,172194225	0,992355013
226928_x_at	SLC25A37	0,820160788	-0,172194225	0,992355013
231768_at	USF1	0,847529938	-0,144765583	0,992295521
212523_s_at	KIAA0146	0,847529938	-0,144765583	0,992295521
206463_s_at	DHRS2	0,847529938	-0,144765583	0,992295521
213225_at	PPM1B	3,570321555	2,578283758	0,992037797
217992_s_at	EFHD2	4,155757614	3,163941505	0,99181611
201145_at	HAX1	5,715818957	4,724593891	0,991225065
201986_at	MED13	3,402275492	2,411206536	0,991068956
227139_s_at	HPS3	4,52000033	3,52937772	0,99062261
224513_s_at	UBQLN4	3,023747815	2,03313004	0,990617776
214783_s_at	ANXA11	2,21987062	1,2298509	0,99001972
212925_at	C19orf21	0,900065578	-0,089635713	0,989701291
222733_x_at	RRP1	0,900065578	-0,089635713	0,989701291
240592_at	LCORL	2,732422196	1,743236669	0,989185527
201465_s_at	JUN	1,716003175	0,727123109	0,988880066
1555478_at	C17orf82	0,95222556	-0,036543687	0,988769247
235478_at	DCLRE1C	1,936380191	0,947673886	0,988706305
226409_at	TBC1D20	1,936380191	0,947673886	0,988706305
238656_at	-	1,936380191	0,947673886	0,988706305
216973_s_at	HOXB7	2,752615529	1,76396664	0,988648889
229567_at	FITM1	-0,384193355	-1,372734086	0,988540731
1569270_at	LOC100134368	-0,384193355	-1,372734086	0,988540731
239931_at	-	-0,384193355	-1,372734086	0,988540731
240929_at	LOC100128750	-0,384193355	-1,372734086	0,988540731
211644_x_at	IGK@ /// IGKC	-0,384193355	-1,372734086	0,988540731
216821_at	-	-0,384193355	-1,372734086	0,988540731
216736_at	TM6SF2	-0,384193355	-1,372734086	0,988540731
230870_at	CFL1	-0,384193355	-1,372734086	0,988540731
210616_s_at	SEC31A	4,460674444	3,472563131	0,988111313
244687_at	DBT	1,802733148	0,815200271	0,987532877
225129_at	CPNE2	1,843966654	0,85655369	0,987412964
47571_at	ZNF236	1,989823233	1,002499198	0,987324034
218171_at	VPS4B	3,697863752	2,710570973	0,987292778
231371_at	TDRD10	-0,924000698	-1,911206569	0,987205872
1555540_at	TGFB3	-0,924000698	-1,911206569	0,987205872
211079_s_at	DYRK1A	-0,924000698	-1,911206569	0,987205872
230933_at	DSTN	-0,924000698	-1,911206569	0,987205872
237777_at	PFKFB1	-0,924000698	-1,911206569	0,987205872
237021_at	LOC144486	-0,924000698	-1,911206569	0,987205872
1566779_at	-	-0,924000698	-1,911206569	0,987205872
226804_at	FAM20A	-0,924000698	-1,911206569	0,987205872
233143_at	C20orf173	-0,924000698	-1,911206569	0,987205872
238905_at	RHOJ	-0,924000698	-1,911206569	0,987205872
221433_at	FGF21	-0,924000698	-1,911206569	0,987205872
211174_s_at	CCKAR	-0,924000698	-1,911206569	0,987205872

232434_at	DIRC3	-0,924000698	-1,911206569	0,987205872
241431_at	LOC100507077	-0,924000698	-1,911206569	0,987205872
229482_at	DDX51	-0,924000698	-1,911206569	0,987205872
229117_s_at	JUND	-0,924000698	-1,911206569	0,987205872
217115_at	MKRN7P	-0,924000698	-1,911206569	0,987205872
215198_s_at	CALD1	-0,924000698	-1,911206569	0,987205872
223508_at	NOTCH1	-0,924000698	-1,911206569	0,987205872
210388_at	PLCB2	-0,924000698	-1,911206569	0,987205872
1554148_a_at	SLC33A1	2,019346222	1,032267596	0,987078625
234947_s_at	FAM204A	1,92317941	0,936232181	0,986947229
1554022_at	CCDC13	-1,06282519	-2,049747196	0,986922006
241511_at	-	-1,06282519	-2,049747196	0,986922006
228791_at	LOC100129502	-1,06282519	-2,049747196	0,986922006
244673_at	-	-1,06282519	-2,049747196	0,986922006
236577_at	-	-1,06282519	-2,049747196	0,986922006
237964_at	-	-1,06282519	-2,049747196	0,986922006
212909_at	LYPD1	-1,06282519	-2,049747196	0,986922006
1559622_at	-	-1,06282519	-2,049747196	0,986922006
1560498_at	-	-1,06282519	-2,049747196	0,986922006
234345_at	-	-1,06282519	-2,049747196	0,986922006
1553470_at	DNAH17	-1,06282519	-2,049747196	0,986922006
207964_x_at	IFNA4	-1,06282519	-2,049747196	0,986922006
206375_s_at	HSPB3	-1,06282519	-2,049747196	0,986922006
220534_at	TRIM48	-1,06282519	-2,049747196	0,986922006
234307_s_at	KIF26A	-1,06282519	-2,049747196	0,986922006
215961_at	F12	-1,06282519	-2,049747196	0,986922006
229330_at	-	-1,06282519	-2,049747196	0,986922006
233218_at	-	-1,06282519	-2,049747196	0,986922006
233709_at	-	-1,06282519	-2,049747196	0,986922006
243044_at	-	-1,06282519	-2,049747196	0,986922006
231757_at	TAS2R5	-1,06282519	-2,049747196	0,986922006
221383_at	NMUR1	-1,06282519	-2,049747196	0,986922006
224830_at	NUDT21	5,068598441	4,081788629	0,986809812
225344_at	NCOA7	5,072080132	4,085667974	0,986412158
212231_at	FBXO21	3,132805465	2,147194726	0,985610739
208250_s_at	DMBT1	1,047341799	0,061773582	0,985568217
207094_at	CXCR1	1,047341799	0,061773582	0,985568217
236169_at	-	-0,730013898	-1,715576125	0,985562227
209502_s_at	BAIAP2	-0,730013898	-1,715576125	0,985562227
229613_at	-	-0,730013898	-1,715576125	0,985562227
1557610_at	LOC100507034	-0,730013898	-1,715576125	0,985562227
215115_x_at	NTRK3	-0,730013898	-1,715576125	0,985562227
227823_at	RGAG4	-0,730013898	-1,715576125	0,985562227
242177_at	-	-0,730013898	-1,715576125	0,985562227
229215_at	ASCL2	-0,730013898	-1,715576125	0,985562227
208596_s_at	UGT1A1 /// UGT1/	-0,730013898	-1,715576125	0,985562227
234730_s_at	RIPK4	-0,730013898	-1,715576125	0,985562227
237073_at	-	-0,730013898	-1,715576125	0,985562227
213265_at	PGA3 /// PGA4 ///	-0,730013898	-1,715576125	0,985562227
237572_at	UGT3A1	-0,730013898	-1,715576125	0,985562227

202755_s_at	GPC1	-0,730013898	-1,715576125	0,985562227
240126_x_at	-	-0,730013898	-1,715576125	0,985562227
206086_x_at	HFE	-0,730013898	-1,715576125	0,985562227
1554044_a_at	MRAP	-0,730013898	-1,715576125	0,985562227
205777_at	DUSP9	-0,730013898	-1,715576125	0,985562227
215243_s_at	GJB3	-0,730013898	-1,715576125	0,985562227
209800_at	KRT16	-0,730013898	-1,715576125	0,985562227
220489_s_at	SERINC2	-0,730013898	-1,715576125	0,985562227
234195_at	TNFRSF10C	-0,730013898	-1,715576125	0,985562227
241852_at	-	-0,437449947	-1,422257026	0,984807079
1555212_at	OR8B8	-0,437449947	-1,422257026	0,984807079
1557621_at	KCP	-0,437449947	-1,422257026	0,984807079
235649_at	ADAMTS8	-0,437449947	-1,422257026	0,984807079
219779_at	ZFHX4	-0,437449947	-1,422257026	0,984807079
243160_at	-	-0,437449947	-1,422257026	0,984807079
1561919_at	-	-0,437449947	-1,422257026	0,984807079
203760_s_at	SLA	3,745344686	2,760632255	0,984712431
217993_s_at	MAT2B	6,012349102	5,027653658	0,984695444
202234_s_at	SLC16A1	3,559161816	2,574574485	0,984587331
53912_at	SNX11	3,593370554	2,609437222	0,983933332
207801_s_at	RNF10	3,577387089	2,593698508	0,983688582
241234_at	LOC100506797	1,139057614	0,155747281	0,983310334
226723_at	CCDC23	3,481948861	2,498884538	0,983064323
223526_at	C18orf21	3,386206804	2,403191094	0,983015709
244830_at	RASIP1	-1,712732543	-2,695640852	0,982908309
208283_at	GAGE1	-1,712732543	-2,695640852	0,982908309
211137_s_at	ATP2C1	3,485638812	2,503049671	0,982589141
226449_at	CEP120	3,541158018	2,558652146	0,982505872
222781_s_at	C9orf40	4,09423887	3,111774082	0,982464788
204340_at	TMEM187	1,182925501	0,20062106	0,982304442
238015_at	C4orf46	2,75889404	1,777135134	0,981758906
204745_x_at	MT1G	4,618041557	3,636386625	0,981654931
223355_at	ALG1	1,20396005	0,222432814	0,981527236
208725_at	EIF2S2	2,149438457	1,167919222	0,981519235
209551_at	YIPF4	2,590269697	1,60918327	0,981086427
64438_at	C17orf101	1,731197603	0,750120044	0,981077559
201849_at	BNIP3	3,566680842	2,585619776	0,981061067
1553003_at	PKHD1	-1,369772723	-2,350444759	0,980672036
220997_s_at	DIAPH3	-1,369772723	-2,350444759	0,980672036
1554375_a_at	NR1H4	-1,369772723	-2,350444759	0,980672036
220986_s_at	TIGD6	-1,369772723	-2,350444759	0,980672036
230022_at	CLEC18A	-1,369772723	-2,350444759	0,980672036
235637_s_at	-	-1,369772723	-2,350444759	0,980672036
211381_x_at	SPAG11B	-1,369772723	-2,350444759	0,980672036
207206_s_at	ALOX12	-1,369772723	-2,350444759	0,980672036
233247_x_at	ZDHHC21	-1,369772723	-2,350444759	0,980672036
1566127_at	-	-1,369772723	-2,350444759	0,980672036
1554290_at	HERC3	-1,369772723	-2,350444759	0,980672036
218800_at	SRD5A3	-1,369772723	-2,350444759	0,980672036
1562934_at	-	-1,369772723	-2,350444759	0,980672036

243722_at	PYDC1	-1,369772723	-2,350444759	0,980672036
226417_at	RHOB	-1,369772723	-2,350444759	0,980672036
226427_s_at	B3GALT6	-1,369772723	-2,350444759	0,980672036
236185_at	NHLRC2	-1,369772723	-2,350444759	0,980672036
206423_at	ANGPTL7	-1,369772723	-2,350444759	0,980672036
1552440_at	GPR182	-1,369772723	-2,350444759	0,980672036
215384_s_at	MAP1A	-1,369772723	-2,350444759	0,980672036
235417_at	SPOCD1	-1,369772723	-2,350444759	0,980672036
235325_at	SPG7	0,319287178	-0,661178575	0,980465752
241640_at	BCAP29	0,319287178	-0,661178575	0,980465752
218725_at	SLC25A22	1,307201325	0,326777209	0,980424116
239739_at	SNX24	-1,289805289	-2,27001641	0,980211121
232361_s_at	EHF	-1,289805289	-2,27001641	0,980211121
211843_x_at	CYP3A7-CYP3AP1	-1,289805289	-2,27001641	0,980211121
231744_at	CELF5	-1,289805289	-2,27001641	0,980211121
211103_at	MYO7A	-1,289805289	-2,27001641	0,980211121
1567054_at	OR1C1	-1,289805289	-2,27001641	0,980211121
242336_at	GSK3B	-1,289805289	-2,27001641	0,980211121
240678_at	-	-1,289805289	-2,27001641	0,980211121
216605_s_at	CEACAM21	-1,289805289	-2,27001641	0,980211121
214497_s_at	NHLH2	-1,289805289	-2,27001641	0,980211121
210998_s_at	HGF	-1,289805289	-2,27001641	0,980211121
244590_at	-	-1,289805289	-2,27001641	0,980211121
237190_at	-	-1,289805289	-2,27001641	0,980211121
239743_at	SP8	0,39105295	-0,589001171	0,980054122
223740_at	AGPAT4-IT1	0,39105295	-0,589001171	0,980054122
223768_at	SSR4P1	0,39105295	-0,589001171	0,980054122
219265_at	MOB3B	0,39105295	-0,589001171	0,980054122
213403_at	-	0,39105295	-0,589001171	0,980054122
212166_at	XPO7	4,12127125	3,141266133	0,980005117
225641_at	MEF2D	2,2388565	1,259081178	0,979775322
220755_s_at	C6orf48	6,231886017	5,252127909	0,979758108
234012_at	-	0,425036312	-0,554696666	0,979732979
235479_at	CPEB2	0,425036312	-0,554696666	0,979732979
226795_at	LRCH1	2,248155141	1,268569037	0,979586104
237431_at	LOC100506882	0,282358733	-0,697182596	0,979541329
219719_at	HIGD1B	0,167727503	-0,811769547	0,97949705
1564498_at	-	0,167727503	-0,811769547	0,97949705
220357_s_at	SGK2	0,167727503	-0,811769547	0,97949705
232004_at	HNRNPR	1,458255986	0,479089184	0,979166802
243308_at	-	0,244681185	-0,734355396	0,979036581
213329_at	SRGAP2	0,244681185	-0,734355396	0,979036581
231305_at	-	0,244681185	-0,734355396	0,979036581
235293_at	-	0,355751005	-0,623254098	0,979005103
230901_x_at	PCSK4	0,355751005	-0,623254098	0,979005103
238948_at	TM9SF1	-0,49284695	-1,471833228	0,978986277
238872_at	LOC100128239	-0,49284695	-1,471833228	0,978986277
215323_at	LUZP2	-0,49284695	-1,471833228	0,978986277
204707_s_at	MAPK4	-0,49284695	-1,471833228	0,978986277
1563131_at	-	-0,49284695	-1,471833228	0,978986277

231741_at	S1PR3	-0,49284695	-1,471833228	0,978986277
232526_at	ITPKB	-0,49284695	-1,471833228	0,978986277
207005_s_at	BCL2	-0,49284695	-1,471833228	0,978986277
223499_at	C1QTNF5 /// MFRI	-0,49284695	-1,471833228	0,978986277
210801_at	DIMT1	0,20740896	-0,771340337	0,978749297
240962_at	-	0,20740896	-0,771340337	0,978749297
209586_s_at	PRUNE	3,045068376	2,066329863	0,978738513
225090_at	SYVN1	2,267141818	1,28853674	0,978605078
241280_at	ALDOB	-1,624955693	-2,603471267	0,978515574
211585_at	NPAT	-1,624955693	-2,603471267	0,978515574
1561554_at	-	-1,624955693	-2,603471267	0,978515574
220673_s_at	PPP4R4	-1,624955693	-2,603471267	0,978515574
1555729_a_at	CD209	-1,624955693	-2,603471267	0,978515574
226796_at	ABHD15	1,578719607	0,600385088	0,978334519
225146_at	FAM219A	1,421391637	0,443143223	0,978248414
221779_at	MICALL1	1,528073958	0,550017041	0,978056918
232596_at	DIAPH3	1,510970097	0,533258442	0,977711656
219704_at	YBX2	1,383446753	0,405752839	0,977693914
214603_at	MAGEA2 /// MAGI	-1,802716385	-2,780225148	0,977508763
1569760_at	-	-1,802716385	-2,780225148	0,977508763
230784_at	PRAC	-1,802716385	-2,780225148	0,977508763
228318_s_at	CRIPAK	2,460890032	1,483698568	0,977191464
240030_at	COMMD6	0,458905032	-0,518232988	0,97713802
1564021_at	LOC100130456	0,458905032	-0,518232988	0,97713802
204720_s_at	DNAJC6	0,458905032	-0,518232988	0,97713802
229616_s_at	GRAMD2	0,045517965	-0,931539147	0,977057112
203901_at	TAB1	0,045517965	-0,931539147	0,977057112
233195_at	DNAI1	0,045517965	-0,931539147	0,977057112
240374_at	-	0,045517965	-0,931539147	0,977057112
242855_at	KCP	0,045517965	-0,931539147	0,977057112
212700_x_at	PLEKHM1 /// PLEK	0,045517965	-0,931539147	0,977057112
233729_at	-	0,045517965	-0,931539147	0,977057112
232642_at	VWA5B2	0,045517965	-0,931539147	0,977057112
241876_at	-	0,127732831	-0,84928999	0,977022821
231026_at	EFHC1	0,127732831	-0,84928999	0,977022821
213854_at	SYNGR1	0,127732831	-0,84928999	0,977022821
228713_s_at	HSD17B14	0,127732831	-0,84928999	0,977022821
206402_s_at	NPFF	0,127732831	-0,84928999	0,977022821
203003_at	MEF2D	0,127732831	-0,84928999	0,977022821
236426_at	KIF19	0,491906512	-0,484558493	0,976465005
215137_at	-	0,491906512	-0,484558493	0,976465005
239996_x_at	ATP2A2	0,491906512	-0,484558493	0,976465005
206734_at	JRKL	2,692288672	1,715863686	0,976424986
224989_at	-	1,936380191	0,960042218	0,976337973
1555752_at	STH	0,524545436	-0,45121326	0,975758696
230638_at	-	0,524545436	-0,45121326	0,975758696
244011_at	PPM1K	0,524545436	-0,45121326	0,975758696
232448_at	LINC00543	-1,452713826	-2,4284334	0,975719574
1566219_at	LOC338651	-1,452713826	-2,4284334	0,975719574
1556253_s_at	-	-1,452713826	-2,4284334	0,975719574

237272_at	LOC100506907	-1,452713826	-2,4284334	0,975719574
242528_at	HOXA-AS2	-1,452713826	-2,4284334	0,975719574
216225_at	-	-1,452713826	-2,4284334	0,975719574
231950_at	LOC653501 /// ZNI	-1,452713826	-2,4284334	0,975719574
233462_at	TBC1D28	-1,452713826	-2,4284334	0,975719574
229259_at	GFAP	-1,452713826	-2,4284334	0,975719574
208338_at	P2RX3	-1,452713826	-2,4284334	0,975719574
222189_at	-	-1,452713826	-2,4284334	0,975719574
207851_s_at	INSR	-1,452713826	-2,4284334	0,975719574
1563658_a_at	SYT9	-1,452713826	-2,4284334	0,975719574
240394_at	-	-1,452713826	-2,4284334	0,975719574
230378_at	SCGB3A1	0,08619576	-0,889222211	0,975417971
220207_at	YIF1B	0,08619576	-0,889222211	0,975417971
1558393_at	KRT7	0,08619576	-0,889222211	0,975417971
1556477_a_at	LOC283485	0,08619576	-0,889222211	0,975417971
238594_x_at	DUSP8	1,625482993	0,65030602	0,975176973
221818_at	INTS5	1,625482993	0,65030602	0,975176973
201805_at	PRKAG1	4,111472006	3,136334485	0,97513752
207585_s_at	RPL36AL	6,919024087	5,943921541	0,975102546
218179_s_at	TRAPPC11	2,739067176	1,76396664	0,975100536
1316_at	THRA	0,086824888	-0,888214478	0,975039367
222696_at	AXIN2	2,053550345	1,078619386	0,974930959
212967_x_at	NAP1L1	7,408486499	6,433607738	0,974878762
221556_at	CDC14B	2,811067138	1,836262316	0,974804821
213221_s_at	SIK2	0,58788177	-0,386896102	0,974777872
235640_at	-	1,871521385	0,896834102	0,974687283
242487_at	CC2D1B	0,556428218	-0,418248858	0,974677076
226401_at	PARP10	0,556428218	-0,418248858	0,974677076
209061_at	NCOA3	2,560606634	1,586121802	0,974484832
220255_at	FANCE	1,701542258	0,727123109	0,974419149
212557_at	ZNF451	3,517785666	2,543655878	0,974129789
208060_at	PAX7	0,001114523	-0,972893339	0,974007861
231454_at	PLAC4	0,001114523	-0,972893339	0,974007861
229601_at	SCYL1	0,001114523	-0,972893339	0,974007861
235840_at	ZKSCAN3	0,001114523	-0,972893339	0,974007861
243956_at	SUSD3	0,001114523	-0,972893339	0,974007861
1555715_a_at	HRH3	0,001114523	-0,972893339	0,974007861
244759_x_at	RNF207	0,001114523	-0,972893339	0,974007861
229628_s_at	C9orf174 /// LOC1	0,001114523	-0,972893339	0,974007861
232807_at	FAM131A	0,001114523	-0,972893339	0,974007861
215798_at	ALDH1L1	0,001114523	-0,972893339	0,974007861
229319_at	-	1,830468611	0,85655369	0,973914922
218840_s_at	NADSYN1	2,902957057	1,929092307	0,973864751
213518_at	PRKCI	2,791258167	1,817403386	0,97385478
231051_at	M1	-1,212652659	-2,186086603	0,973433944
215259_s_at	CADM4	-1,212652659	-2,186086603	0,973433944
243209_at	KCNQ4	-1,212652659	-2,186086603	0,973433944
214511_x_at	FCGR1B	-1,212652659	-2,186086603	0,973433944
1570644_at	-	-1,212652659	-2,186086603	0,973433944
234533_at	BCL2L14	-1,212652659	-2,186086603	0,973433944

237265_at	C16orf73 /// LINC(	-1,212652659	-2,186086603	0,973433944
220515_at	DUSP21	-1,212652659	-2,186086603	0,973433944
206677_at	KRT31	-1,212652659	-2,186086603	0,973433944
243518_at	-	-1,212652659	-2,186086603	0,973433944
221977_at	TBX2	-1,212652659	-2,186086603	0,973433944
233426_at	-	-1,212652659	-2,186086603	0,973433944
231445_at	PAX1	-1,212652659	-2,186086603	0,973433944
206950_at	SCN9A	-1,212652659	-2,186086603	0,973433944
205337_at	DCT	-1,212652659	-2,186086603	0,973433944
237574_at	-	-1,212652659	-2,186086603	0,973433944
227147_s_at	EGLN2	-1,212652659	-2,186086603	0,973433944
206964_at	NAT8B	-1,212652659	-2,186086603	0,973433944
228898_s_at	SMARCB1	-1,212652659	-2,186086603	0,973433944
224066_s_at	HIPK2	-1,212652659	-2,186086603	0,973433944
232080_at	HECW2	-1,212652659	-2,186086603	0,973433944
201625_s_at	INSIG1	3,426291319	2,45323331	0,973058009
225707_at	ARL6IP6	4,701786435	3,728800162	0,972986273
207165_at	HMMR	6,364388869	5,391561917	0,972826952
223320_s_at	ABCB10	4,452318502	3,479912486	0,972406016
1555535_at	BPIFB6	-0,791294935	-1,763664074	0,972369139
244652_at	-	-0,791294935	-1,763664074	0,972369139
221404_at	IL36A	-0,791294935	-1,763664074	0,972369139
234200_at	-	-0,791294935	-1,763664074	0,972369139
1559674_at	ZNF333	-0,791294935	-1,763664074	0,972369139
206605_at	ENDOU	-0,791294935	-1,763664074	0,972369139
1559800_a_at	LOC100507570	-0,791294935	-1,763664074	0,972369139
225801_at	FBXO32	-0,791294935	-1,763664074	0,972369139
211890_x_at	CAPN3	-0,791294935	-1,763664074	0,972369139
242752_at	-	-0,791294935	-1,763664074	0,972369139
231266_at	LYPD4	-0,791294935	-1,763664074	0,972369139
211670_x_at	SSX3	-0,791294935	-1,763664074	0,972369139
220210_at	CHRNA10	-0,791294935	-1,763664074	0,972369139
232532_at	QRICH2	-0,791294935	-1,763664074	0,972369139
238926_at	LOC100505974	-0,791294935	-1,763664074	0,972369139
235815_at	TSHZ2	-0,791294935	-1,763664074	0,972369139
207573_x_at	ATP5L	7,490161682	6,518068695	0,972092987
239015_at	THAP7-AS1	-0,043925712	-1,015398016	0,971472304
1553062_at	MOGAT1	-0,043925712	-1,015398016	0,971472304
224144_at	SPTBN4	-0,043925712	-1,015398016	0,971472304
1569732_at	-	-0,043925712	-1,015398016	0,971472304
227360_at	RDH13	-0,043925712	-1,015398016	0,971472304
217062_at	DMPK	-0,043925712	-1,015398016	0,971472304
1555475_x_at	TTLL3	-0,043925712	-1,015398016	0,971472304
231782_s_at	KLK4	-0,043925712	-1,015398016	0,971472304
236381_s_at	WRAP73	3,581038515	2,609615156	0,971423358
221563_at	DUSP10	3,7799838	2,809015785	0,970968015
227131_at	MAP3K3	2,159837409	1,189078962	0,970758447
219553_at	NME7	4,218749628	3,248126831	0,970622797
211267_at	HESX1	0,648195588	-0,322340048	0,970535636
1559922_at	-	0,648195588	-0,322340048	0,970535636

216164_at	LRRN2	0,648195588	-0,322340048	0,970535636
226641_at	ANKRD44	3,389999385	2,419753897	0,970245488
209476_at	TMX1	5,548373765	4,578636564	0,969737201
219286_s_at	RBM15	4,684174558	3,714910188	0,96926437
1566989_at	ARID1B	2,25755351	1,28853674	0,96901677
218909_at	RPS6KC1	2,642918178	1,674071917	0,968846262
238232_at	-	-0,551284523	-1,520022123	0,968737601
208486_at	DRD5	-0,551284523	-1,520022123	0,968737601
221333_at	FOXP3	-0,551284523	-1,520022123	0,968737601
1563089_at	-	-0,551284523	-1,520022123	0,968737601
205080_at	RARB	-0,551284523	-1,520022123	0,968737601
240012_at	-	-0,551284523	-1,520022123	0,968737601
230564_at	SIPA1L3	-0,551284523	-1,520022123	0,968737601
219775_s_at	CPLX3	-0,551284523	-1,520022123	0,968737601
237847_at	NTN1	-0,551284523	-1,520022123	0,968737601
233698_at	-	-0,551284523	-1,520022123	0,968737601
235329_at	NOXO1	-0,551284523	-1,520022123	0,968737601
222055_at	FAHD2A /// FAHD2	-0,551284523	-1,520022123	0,968737601
244566_at	-	-0,551284523	-1,520022123	0,968737601
214994_at	APOBEC3F	-0,551284523	-1,520022123	0,968737601
209118_s_at	TUBA1A	7,12239588	6,153902886	0,968492995
228048_at	ZNF503-AS2	-0,088974936	-1,057301851	0,968326915
1562581_at	LOC254028	-0,088974936	-1,057301851	0,968326915
1552930_at	MMEL1	-0,088974936	-1,057301851	0,968326915
241326_at	AK7	-0,088974936	-1,057301851	0,968326915
237754_at	LOC100506559	-0,088974936	-1,057301851	0,968326915
208416_s_at	SPTB	-0,088974936	-1,057301851	0,968326915
208731_at	RAB2A	3,925077605	2,957089023	0,967988581
228614_at	LINC00116	2,943764123	1,975797592	0,967966532
235731_at	AIPL1	0,735999505	-0,231045907	0,967045412
239706_x_at	-	0,735999505	-0,231045907	0,967045412
216181_at	SYNJ2	0,735999505	-0,231045907	0,967045412
202145_at	LY6E	2,590269697	1,623485524	0,966784174
225436_at	FAM108C1	2,552836808	1,586121802	0,966715007
230449_x_at	-	2,374261973	1,408220608	0,966041365
233977_at	GREB1L	-0,135065865	-1,100943374	0,965877509
1569959_at	-	-0,135065865	-1,100943374	0,965877509
204250_s_at	CEP164	-0,135065865	-1,100943374	0,965877509
1556107_at	-	-0,135065865	-1,100943374	0,965877509
210476_s_at	PRLR	-0,135065865	-1,100943374	0,965877509
1560100_at	DLX1	-0,135065865	-1,100943374	0,965877509
48531_at	TNIP2	3,408437341	2,442980521	0,96545682
217188_s_at	C14orf1	4,163636979	3,198229785	0,965407193
218028_at	ELOVL1	3,846827633	2,881480309	0,965347324
210465_s_at	SNAPC3	3,112433079	2,147194726	0,965238352
227799_at	MYO1G	4,842157797	3,876962585	0,965195212
206836_at	SLC6A3	0,820160788	-0,144765583	0,964926371
220864_s_at	NDUFA13	5,828452042	4,863735125	0,964716917
202942_at	ETFB	4,624674869	3,660020626	0,964654242
214349_at	CTCF	0,847529938	-0,116767475	0,964297413

1560327_at	-	0,791908897	-0,172194225	0,964103122
231964_at	BICD1	-0,180990326	-1,145057014	0,964066688
1555509_a_at	SLC25A41	-0,180990326	-1,145057014	0,964066688
241934_at	NTM	-0,180990326	-1,145057014	0,964066688
233164_x_at	RHBDD1	-0,180990326	-1,145057014	0,964066688
240537_s_at	LOC440356	-0,180990326	-1,145057014	0,964066688
1564403_at	C9orf133	-0,180990326	-1,145057014	0,964066688
231007_at	-	-1,537851782	-2,501359113	0,963507331
216744_at	-	-1,537851782	-2,501359113	0,963507331
234280_at	REG3A	-1,537851782	-2,501359113	0,963507331
208040_s_at	MYBPC3	0,873789652	-0,089635713	0,963425365
215354_s_at	PELP1	1,983901861	1,020514937	0,963386924
221483_s_at	ARPP19	5,029786449	4,066469611	0,963316839
216791_at	-	0,95222556	-0,011001236	0,963226796
223921_s_at	GBA2	0,926325262	-0,036543687	0,962868949
220042_x_at	HIVEP3	0,926325262	-0,036543687	0,962868949
221423_s_at	YIPF5	3,322661262	2,359892448	0,962768814
213368_x_at	PPFIA3	1,024284941	0,061773582	0,962511359
214564_s_at	PCDHGC3	-0,994186487	-1,956681069	0,962494583
230631_s_at	LOC100288432	-0,994186487	-1,956681069	0,962494583
223902_at	NEFM	-0,994186487	-1,956681069	0,962494583
235965_at	-	-0,994186487	-1,956681069	0,962494583
208441_at	IGF1R	-0,994186487	-1,956681069	0,962494583
233091_at	ATAD3B	-0,994186487	-1,956681069	0,962494583
239886_at	-	-0,994186487	-1,956681069	0,962494583
211731_x_at	SSX3	-0,994186487	-1,956681069	0,962494583
237922_at	-	-0,994186487	-1,956681069	0,962494583
213884_s_at	TRIM3	-0,994186487	-1,956681069	0,962494583
232290_at	-	-0,994186487	-1,956681069	0,962494583
227283_at	EFR3B	-0,994186487	-1,956681069	0,962494583
211898_s_at	EPHB1	-0,994186487	-1,956681069	0,962494583
227132_at	ZNF706	2,063971513	1,101780212	0,962191302
233021_at	RBM26-AS1	1,898032047	0,936232181	0,961799866
212068_s_at	PRRC2B	2,62869474	1,666953618	0,961741122
223298_s_at	NT5C3	5,100432184	4,139015548	0,961416636
203749_s_at	RARA	1,544940605	0,583607503	0,961333102
230821_at	ZNF148	1,440265662	0,479089184	0,961176478
213622_at	COL9A2	1,440265662	0,479089184	0,961176478
227473_at	CTTN	1,139057614	0,178191865	0,960865749
213526_s_at	LIN37	2,476788561	1,516067292	0,960721269
218183_at	C16orf5	1,267146969	0,306454867	0,960692102
212255_s_at	ATP2C1	3,942941653	2,982339418	0,960602235
221939_at	YIPF2	2,159837409	1,199296812	0,960540597
218745_x_at	TMEM161A	2,427650693	1,46719264	0,960458054
234680_at	KRTAP17-1	-0,230432956	-1,19085694	0,960423984
244025_at	-	-0,230432956	-1,19085694	0,960423984
209661_at	KIFC3	-0,230432956	-1,19085694	0,960423984
207488_at	-	-0,230432956	-1,19085694	0,960423984
227785_at	SDCCAG8	1,816938997	0,85655369	0,960385307
228354_at	MORN4	1,160996507	0,20062106	0,960375448

226429_at	KIAA1704	1,160996507	0,20062106	0,960375448
230440_at	ZNF469	1,160996507	0,20062106	0,960375448
37566_at	KIAA1045	-1,410647317	-2,371005511	0,960358194
1552295_a_at	SLC39A13	1,307201325	0,346964736	0,960236589
38766_at	LOC100862671 ///	1,336703698	0,376505477	0,960198222
219001_s_at	DCAF10	1,802733148	0,842599219	0,960133928
220734_s_at	GLTPD1	1,610353504	0,65030602	0,960047484
241727_x_at	DHFRL1	2,127965541	1,167919222	0,960046319
1560560_at	-	1,225123479	0,265475485	0,959647994
212252_at	CAMKK2	1,788922638	0,829309537	0,959613101
222411_s_at	SSR3	5,652229597	4,692644316	0,959585281
243150_at	-	-1,137208348	-2,09678811	0,959579761
215876_at	-	-1,137208348	-2,09678811	0,959579761
244585_at	IFT140	-1,137208348	-2,09678811	0,959579761
206030_at	ASPA	-1,137208348	-2,09678811	0,959579761
221072_at	GSN-AS1	-1,137208348	-2,09678811	0,959579761
1564379_at	-	-1,137208348	-2,09678811	0,959579761
239058_at	-	-1,137208348	-2,09678811	0,959579761
235835_at	-	-1,137208348	-2,09678811	0,959579761
1553053_at	LINC00521	-1,137208348	-2,09678811	0,959579761
212939_at	COL6A1	-1,137208348	-2,09678811	0,959579761
202834_at	AGT	-1,137208348	-2,09678811	0,959579761
234750_at	CACNG8	-1,137208348	-2,09678811	0,959579761
203325_s_at	COL5A1	-1,137208348	-2,09678811	0,959579761
244250_at	ANXA6	-1,137208348	-2,09678811	0,959579761
1553686_at	C18orf25	-1,137208348	-2,09678811	0,959579761
224336_s_at	DUSP16	-1,137208348	-2,09678811	0,959579761
230561_s_at	KANSL1L	-0,609040214	-1,568385657	0,959345443
216009_at	SLC39A9	-0,609040214	-1,568385657	0,959345443
222281_s_at	C1orf186 /// LOC1	-0,609040214	-1,568385657	0,959345443
221342_at	C6orf25	-0,609040214	-1,568385657	0,959345443
238111_at	SDCCAG3	-0,609040214	-1,568385657	0,959345443
1569305_a_at	-	-0,609040214	-1,568385657	0,959345443
1552914_a_at	CD276	-0,609040214	-1,568385657	0,959345443
1566834_at	-	-0,609040214	-1,568385657	0,959345443
218054_s_at	KXD1	-0,609040214	-1,568385657	0,959345443
238754_at	-	-0,609040214	-1,568385657	0,959345443
1565618_at	KIAA1671	-0,609040214	-1,568385657	0,959345443
1563204_at	-	-0,609040214	-1,568385657	0,959345443
1570607_at	-	-0,609040214	-1,568385657	0,959345443
218634_at	PHLDA3	-0,609040214	-1,568385657	0,959345443
223945_x_at	RP9P	2,530390387	1,571112806	0,959277581
204305_at	MIPEP	1,760255626	0,80114751	0,959108116
205584_at	ALG13	1,760255626	0,80114751	0,959108116
213295_at	CYLD	3,466076444	2,506994817	0,959081627
1555971_s_at	FBXO28	3,771154436	2,812515887	0,958638549
202211_at	ARFGAP3	4,37922506	3,420735804	0,958489256
217997_at	PHLDA1	1,640193838	0,681874787	0,958319051
228059_x_at	MRPS22	4,102051194	3,143844996	0,958206198
209806_at	HIST1H2BK	5,807261671	4,84911965	0,958142021

219351_at	TRAPPC2	3,012642182	2,055739171	0,956903011
224592_x_at	HP1BP3	5,464350223	4,507578586	0,956771637
227840_at	C2orf76	2,926639205	1,970106792	0,956532413
217286_s_at	NDRG3	4,330688003	3,374450349	0,956237654
65591_at	WDR48	2,906317353	1,950776998	0,955540356
221360_s_at	GHSR	-0,856259172	-1,811780055	0,955520883
231346_s_at	-	-0,856259172	-1,811780055	0,955520883
215417_at	EXOC6B	-0,856259172	-1,811780055	0,955520883
210090_at	ARC	-0,856259172	-1,811780055	0,955520883
1566168_at	-	-0,856259172	-1,811780055	0,955520883
229180_at	WWC1	-0,856259172	-1,811780055	0,955520883
1563324_at	LOC100129603	-0,856259172	-1,811780055	0,955520883
232703_at	GLUD1P7	-0,856259172	-1,811780055	0,955520883
1566548_at	-	-0,856259172	-1,811780055	0,955520883
234175_at	-	-0,856259172	-1,811780055	0,955520883
234468_at	-	-0,856259172	-1,811780055	0,955520883
235880_at	SRRM3	-0,856259172	-1,811780055	0,955520883
213722_at	SOX2	-0,856259172	-1,811780055	0,955520883
1554195_a_at	C5orf46	-0,856259172	-1,811780055	0,955520883
1566236_at	DGCR12	-0,856259172	-1,811780055	0,955520883
236061_at	PRDM15	-0,856259172	-1,811780055	0,955520883
216597_at	-	-0,856259172	-1,811780055	0,955520883
236487_at	SCLT1	2,732422196	1,777135134	0,955287063
212461_at	AZIN1	5,061011789	4,105949949	0,955061839
204149_s_at	GSTM4	-0,280273599	-1,234835326	0,954561727
238489_at	PRKAA2	-0,280273599	-1,234835326	0,954561727
222323_at	-	-0,280273599	-1,234835326	0,954561727
210345_s_at	DNAH9	-0,280273599	-1,234835326	0,954561727
215875_at	-	-0,280273599	-1,234835326	0,954561727
203649_s_at	PLA2G2A	-0,280273599	-1,234835326	0,954561727
234337_at	TTLL9	-0,280273599	-1,234835326	0,954561727
1569274_at	-	-0,280273599	-1,234835326	0,954561727
224847_at	CDK6	4,402175336	3,447692163	0,954483173
203966_s_at	PPM1A	3,551632175	2,597466271	0,954165904
205641_s_at	TRADD	3,704211994	2,750185881	0,954026113
214937_x_at	PCM1	4,714814978	3,760893183	0,953921795
1554177_a_at	ATP5S	2,452674045	1,500059916	0,952614129
1552988_at	C11orf65	-1,712732543	-2,665156778	0,952424235
1565673_at	FCGR2A	-1,712732543	-2,665156778	0,952424235
1570476_at	-	-1,712732543	-2,665156778	0,952424235
233023_at	-	-1,712732543	-2,665156778	0,952424235
202288_at	MTOR	2,019346222	1,067365565	0,951980657
218547_at	DHDDS	2,042564225	1,090640407	0,951923818
206975_at	LTA	1,92317941	0,971607464	0,951571946
218117_at	RBX1	6,150226503	5,198764792	0,951461712
209731_at	NTHL1	2,063971513	1,112548615	0,951422898
201416_at	SOX4	4,951709151	4,000298874	0,951410276
221471_at	SERINC3	4,484674718	3,533373095	0,951301623
207831_x_at	DHPS	4,228647054	3,277597597	0,951049457
202475_at	TMEM147	4,900499467	3,949468418	0,951031049

218227_at	NUBP2	2,085817135	1,135235995	0,95058114
204352_at	TRAF5	3,8018113	2,851300913	0,950510387
213527_s_at	ZNF688	1,898032047	0,947673886	0,950358161
224887_at	GNPTG	2,118167848	1,167919222	0,950248627
211908_x_at	IGK@	-0,332405896	-1,282520722	0,950114826
220366_at	ELSPBP1	-0,332405896	-1,282520722	0,950114826
211110_s_at	AR	-0,332405896	-1,282520722	0,950114826
1569648_at	DACT2	-0,332405896	-1,282520722	0,950114826
214839_at	LINC00599 /// MIR	-0,332405896	-1,282520722	0,950114826
229696_at	FECH	-0,332405896	-1,282520722	0,950114826
212755_at	MON2	-0,332405896	-1,282520722	0,950114826
205179_s_at	ADAM8	-0,332405896	-1,282520722	0,950114826
216559_x_at	-	4,611761796	3,66182964	0,949932156
200672_x_at	SPTBN1	2,159837409	1,209973561	0,949863848
225764_at	ETV6	3,559161816	2,609615156	0,94954666
226750_at	LARP1B	2,138605583	1,189078962	0,949526621
1570111_at	LINC00521	-1,802716385	-2,752097204	0,949380819
202035_s_at	SFRP1	-1,802716385	-2,752097204	0,949380819
201845_s_at	RYBP	4,308320282	3,35938193	0,948938352
201800_s_at	OSBP	3,777053516	2,828849722	0,948203794
226625_at	TGFBR3	3,434684504	2,486505579	0,948178926
201232_s_at	PSMD13	4,996753949	4,04859535	0,948158599
216629_at	SRRM2	-0,66934516	-1,616924751	0,947579592
231449_at	-	-0,66934516	-1,616924751	0,947579592
206523_at	CYTH3	-0,66934516	-1,616924751	0,947579592
203815_at	GSTT1	-0,66934516	-1,616924751	0,947579592
206168_at	ZC3H7B	-0,66934516	-1,616924751	0,947579592
1561056_a_at	-	-0,66934516	-1,616924751	0,947579592
236800_at	UCMA	-0,66934516	-1,616924751	0,947579592
208168_s_at	CHIT1	-0,66934516	-1,616924751	0,947579592
235140_at	SHROOM1	-0,66934516	-1,616924751	0,947579592
228527_s_at	SLC25A37	-0,66934516	-1,616924751	0,947579592
241180_at	LOC100129112	-0,66934516	-1,616924751	0,947579592
1561301_at	MAGI1-IT1	-0,66934516	-1,616924751	0,947579592
222008_at	COL9A1	-0,66934516	-1,616924751	0,947579592
236567_at	-	-0,66934516	-1,616924751	0,947579592
1553629_a_at	FAM71B	-0,66934516	-1,616924751	0,947579592
228689_at	NDUFA11	-0,66934516	-1,616924751	0,947579592
1552524_at	ART5	-0,66934516	-1,616924751	0,947579592
217179_x_at	-	-0,66934516	-1,616924751	0,947579592
220616_at	ZNF384	-0,66934516	-1,616924751	0,947579592
203033_x_at	FH	5,700365789	4,753282184	0,947083606
212622_at	TMEM41B	4,142807036	3,195836661	0,946970374
222657_s_at	UBE2W	3,243218364	2,296310714	0,94690765
200779_at	ATF4	7,234349471	6,288258516	0,946090955
237507_at	KRT73	0,39105295	-0,554696666	0,945749617
227902_at	ZFP41	0,39105295	-0,554696666	0,945749617
220208_at	ADAMTS13	0,39105295	-0,554696666	0,945749617
218445_at	H2AFY2	0,39105295	-0,554696666	0,945749617
38398_at	MADD	2,480907981	1,535268376	0,945639605

1557602_at	LOC201617	-1,624955693	-2,570522741	0,945567048
1568826_at	LOC100507173	-1,624955693	-2,570522741	0,945567048
1562091_at	-	-1,624955693	-2,570522741	0,945567048
239398_at	KLHL31	-1,624955693	-2,570522741	0,945567048
1554500_a_at	RGS7	-1,624955693	-2,570522741	0,945567048
1565152_at	-	-1,624955693	-2,570522741	0,945567048
1559567_at	PRDM11	-1,624955693	-2,570522741	0,945567048
211307_s_at	FCAR	-1,624955693	-2,570522741	0,945567048
1569805_at	-	-1,624955693	-2,570522741	0,945567048
220985_s_at	RNF170	2,468978138	1,523429058	0,94554908
226742_at	SAR1B	2,966903803	2,021531611	0,945372193
210695_s_at	WWOX	2,582572508	1,637482551	0,945089957
217858_s_at	ARMCX3	2,955426542	2,010406245	0,945020297
206691_s_at	PDIA2	0,355751005	-0,589001171	0,944752176
206851_at	RNASE3	0,355751005	-0,589001171	0,944752176
234505_at	-	0,355751005	-0,589001171	0,944752176
241715_x_at	ACPT	0,355751005	-0,589001171	0,944752176
218132_s_at	TSEN34	3,175897496	2,23115548	0,944742016
226306_at	C6orf1	1,544940605	0,600385088	0,944555517
211593_s_at	MAST2	1,671397663	0,727123109	0,944274554
219603_s_at	ZNF226	1,610353504	0,666486137	0,943867367
225810_at	MTMR10	1,610353504	0,666486137	0,943867367
204484_at	PIK3C2B	2,878891456	1,935144844	0,943746612
230699_at	PGLS	0,282358733	-0,661178575	0,943537307
236460_at	-	0,282358733	-0,661178575	0,943537307
219135_s_at	LMF1	0,282358733	-0,661178575	0,943537307
228883_at	TUB	0,458905032	-0,484558493	0,943463525
1554614_a_at	PTBP2	0,556428218	-0,386896102	0,94332432
230427_s_at	BAG5	0,556428218	-0,386896102	0,94332432
219891_at	PGPEP1	0,556428218	-0,386896102	0,94332432
223069_s_at	EML4	0,556428218	-0,386896102	0,94332432
212095_s_at	MTUS1	0,425036312	-0,518232988	0,943269301
229150_at	-	-0,384193355	-1,32740143	0,943208075
237403_at	GFI1B	-0,384193355	-1,32740143	0,943208075
201374_x_at	PPP2CB	-0,384193355	-1,32740143	0,943208075
238215_at	SLC6A18	-0,384193355	-1,32740143	0,943208075
215413_at	EXOC7	-0,384193355	-1,32740143	0,943208075
218675_at	SLC22A17	-0,384193355	-1,32740143	0,943208075
234343_s_at	RASAL2	-0,384193355	-1,32740143	0,943208075
220715_at	-	-0,384193355	-1,32740143	0,943208075
208287_at	HCG9	-0,384193355	-1,32740143	0,943208075
224268_x_at	ZAN	-0,384193355	-1,32740143	0,943208075
214233_at	GGA2	-0,384193355	-1,32740143	0,943208075
205243_at	SLC13A3	-0,384193355	-1,32740143	0,943208075
206956_at	BGLAP /// PMF1-B	-0,384193355	-1,32740143	0,943208075
215141_at	NOP14-AS1	-0,384193355	-1,32740143	0,943208075
239241_at	LOC727869	0,491906512	-0,45121326	0,943119771
219521_at	B3GAT1	0,491906512	-0,45121326	0,943119771
222265_at	TNS4	0,491906512	-0,45121326	0,943119771
203326_x_at	-	0,491906512	-0,45121326	0,943119771

231955_s_at	HIBADH	1,640193838	0,697221375	0,942972464
231894_at	SARS	1,476175815	0,533258442	0,942917373
204982_at	GIT2	3,978219531	3,035366122	0,942853409
225279_s_at	C3orf17	0,524545436	-0,418248858	0,942794294
1553713_a_at	RHEBL1	2,836219929	1,893444329	0,9427756
204727_at	WDHD1	0,58788177	-0,354781582	0,942663352
1570402_at	KLC3	0,58788177	-0,354781582	0,942663352
227264_at	TRAF6	0,58788177	-0,354781582	0,942663352
206756_at	CHST7	0,58788177	-0,354781582	0,942663352
216208_s_at	ATF6B	0,319287178	-0,623254098	0,942541276
215107_s_at	TTC22	-1,369772723	-2,312054429	0,942281706
202287_s_at	TACSTD2	-1,369772723	-2,312054429	0,942281706
205485_at	RYR1	-1,369772723	-2,312054429	0,942281706
220931_at	C13orf44	-1,369772723	-2,312054429	0,942281706
230432_at	LOC100422737	-1,369772723	-2,312054429	0,942281706
230552_at	LOC100134317 ///	-1,369772723	-2,312054429	0,942281706
217308_at	OR1F2P	-1,369772723	-2,312054429	0,942281706
208408_at	PTN	-1,369772723	-2,312054429	0,942281706
1555189_a_at	TAT	-1,369772723	-2,312054429	0,942281706
1557465_at	LINC00282	-1,369772723	-2,312054429	0,942281706
243448_at	-	-1,369772723	-2,312054429	0,942281706
240532_at	SLC32A1	-1,369772723	-2,312054429	0,942281706
1555807_a_at	MOG	-1,369772723	-2,312054429	0,942281706
231289_at	-	-1,369772723	-2,312054429	0,942281706
236228_at	LOC100130744	-1,369772723	-2,312054429	0,942281706
1563263_at	-	-1,369772723	-2,312054429	0,942281706
222268_x_at	MUC5B	-1,369772723	-2,312054429	0,942281706
1566186_at	-	-1,06282519	-2,005029581	0,942204391
220224_at	HAO1	-1,06282519	-2,005029581	0,942204391
243351_at	FLJ31485	-1,06282519	-2,005029581	0,942204391
210918_at	-	-1,06282519	-2,005029581	0,942204391
239303_at	PIWIL2	-1,06282519	-2,005029581	0,942204391
235246_at	-	-1,06282519	-2,005029581	0,942204391
215777_at	-	-1,06282519	-2,005029581	0,942204391
240188_at	-	-1,06282519	-2,005029581	0,942204391
238659_at	KIAA0141	-1,06282519	-2,005029581	0,942204391
207695_s_at	IGSF1	-1,06282519	-2,005029581	0,942204391
1555323_at	ABCB9	-1,06282519	-2,005029581	0,942204391
244274_at	-	-1,06282519	-2,005029581	0,942204391
211819_s_at	SORBS1	-1,06282519	-2,005029581	0,942204391
208512_s_at	MLLT4	-1,06282519	-2,005029581	0,942204391
236968_at	CCER1	-1,06282519	-2,005029581	0,942204391
206534_at	GRIN2A	-1,06282519	-2,005029581	0,942204391
207118_s_at	MMP23A /// MMF	-1,06282519	-2,005029581	0,942204391
1553891_at	KLF17	-1,06282519	-2,005029581	0,942204391
242021_at	XBP1	-1,06282519	-2,005029581	0,942204391
207745_at	CABP2	-1,06282519	-2,005029581	0,942204391
205185_at	SPINK5	-1,06282519	-2,005029581	0,942204391
1555634_a_at	LILRA5	-1,06282519	-2,005029581	0,942204391
238857_at	ATE1	-1,06282519	-2,005029581	0,942204391

205613_at	SYT17	-1,06282519	-2,005029581	0,942204391
224878_at	UBFD1	3,570321555	2,628204186	0,942117369
213370_s_at	SFMBT1	2,678352494	1,736450458	0,941902036
207068_at	ZFP37	0,244681185	-0,697182596	0,941863782
238624_at	-	0,244681185	-0,697182596	0,941863782
244084_at	AIFM3	0,244681185	-0,697182596	0,941863782
1561423_at	LINC00535	0,244681185	-0,697182596	0,941863782
203618_at	FAIM2	0,244681185	-0,697182596	0,941863782
36545_s_at	SFI1	2,71254166	1,770732079	0,94180958
217780_at	WDR83OS	4,603614396	3,66182964	0,941784757
227158_at	C14orf126	2,771643417	1,830129603	0,941513814
235387_at	GSTCD	2,739067176	1,797682937	0,941384238
207724_s_at	SPAST	1,402495885	0,461385738	0,941110147
226114_at	ZNF436	0,618277321	-0,322340048	0,940617369
236730_at	GIPC3	0,618277321	-0,322340048	0,940617369
223637_s_at	FAM160A2	2,031158928	1,090640407	0,940518521
212609_s_at	AKT3	2,179708195	1,239268196	0,940439999
210045_at	IDH2	1,267146969	0,326777209	0,94036976
218919_at	ZFAND1	3,870422283	2,930238085	0,940184198
202843_at	DNAJB9	1,345611839	0,405752839	0,939859
209386_at	TM4SF1	0,127732831	-0,811769547	0,939502377
235866_at	C9orf85	0,127732831	-0,811769547	0,939502377
233047_at	FRMD7	-1,289805289	-2,229233437	0,939428149
1555051_at	C10orf53	-1,289805289	-2,229233437	0,939428149
1569767_at	-	-1,289805289	-2,229233437	0,939428149
1561327_at	LINC00242	-1,289805289	-2,229233437	0,939428149
223720_at	SPINK7	-1,289805289	-2,229233437	0,939428149
231722_at	CASP14	-1,289805289	-2,229233437	0,939428149
228807_at	ASPA	-1,289805289	-2,229233437	0,939428149
229674_at	SERTAD4	-1,289805289	-2,229233437	0,939428149
240583_at	RIOK3	-1,289805289	-2,229233437	0,939428149
233171_at	GRIN3A	-1,289805289	-2,229233437	0,939428149
221198_at	SCT	-1,289805289	-2,229233437	0,939428149
239353_at	STK32A	-1,289805289	-2,229233437	0,939428149
208292_at	BMP10	-1,289805289	-2,229233437	0,939428149
1554291_at	UHRF1BP1L	-1,289805289	-2,229233437	0,939428149
239726_at	ANK3	-1,289805289	-2,229233437	0,939428149
200938_s_at	RERE	-1,289805289	-2,229233437	0,939428149
208323_s_at	ANXA13	-1,289805289	-2,229233437	0,939428149
1554377_a_at	CNTNAP4	-1,289805289	-2,229233437	0,939428149
224153_s_at	-	-1,289805289	-2,229233437	0,939428149
237648_x_at	-	-1,452713826	-2,391863529	0,939149703
203650_at	PROCR	-1,452713826	-2,391863529	0,939149703
242430_at	-	-1,452713826	-2,391863529	0,939149703
1562989_at	-	-1,452713826	-2,391863529	0,939149703
1559678_s_at	LOC100652791 ///	-1,452713826	-2,391863529	0,939149703
217424_at	-	-1,452713826	-2,391863529	0,939149703
1558643_s_at	EDIL3	-1,452713826	-2,391863529	0,939149703
216414_at	-	-1,452713826	-2,391863529	0,939149703
1560537_at	LOC100129662	-1,452713826	-2,391863529	0,939149703

1555914_a_at	EXD3	-1,452713826	-2,391863529	0,939149703
243811_at	HOXB1	-1,452713826	-2,391863529	0,939149703
209074_s_at	FAM107A /// LOC1	-1,452713826	-2,391863529	0,939149703
220100_at	SLC22A11	-1,452713826	-2,391863529	0,939149703
238335_at	DNAJC21	0,167727503	-0,771340337	0,93906784
233941_at	C14orf166B	0,167727503	-0,771340337	0,93906784
224630_at	ERLEC1	4,693076946	3,754141744	0,938935202
208451_s_at	C4A /// C4B /// LO	0,648195588	-0,290694975	0,938890563
205193_at	MAFF	0,648195588	-0,290694975	0,938890563
218591_s_at	NOL10	0,707657549	-0,231045907	0,938703455
244002_at	-	0,707657549	-0,231045907	0,938703455
244491_at	-	0,707657549	-0,231045907	0,938703455
219472_at	CENPO	0,707657549	-0,231045907	0,938703455
218759_at	DVL2	1,000459215	0,061773582	0,938685633
238887_at	LOC100630918	1,000459215	0,061773582	0,938685633
213532_at	ADAM17	4,022766247	3,084123325	0,938642922
205035_at	CTDP1	1,160996507	0,222432814	0,938563693
206627_s_at	SSX1	-1,893506789	-2,832036647	0,938529858
236124_at	LOC153546	1,116462765	0,178191865	0,938270899
219894_at	MAGEL2	-0,924000698	-1,862269442	0,938268744
1555602_a_at	ELAVL3	-0,924000698	-1,862269442	0,938268744
207009_at	PHOX2B	-0,924000698	-1,862269442	0,938268744
1564729_at	-	-0,924000698	-1,862269442	0,938268744
239394_at	SLC6A2	-0,924000698	-1,862269442	0,938268744
239327_at	-	-0,924000698	-1,862269442	0,938268744
1563071_at	LOC100289061	-0,924000698	-1,862269442	0,938268744
230796_at	LOC440900	-0,924000698	-1,862269442	0,938268744
233129_at	-	-0,924000698	-1,862269442	0,938268744
243451_at	-	-0,924000698	-1,862269442	0,938268744
221295_at	CIDEA	-0,924000698	-1,862269442	0,938268744
232029_at	-	-0,924000698	-1,862269442	0,938268744
234814_at	-	-0,924000698	-1,862269442	0,938268744
207212_at	SLC9A3	-0,924000698	-1,862269442	0,938268744
208038_at	IL1RL2	-0,924000698	-1,862269442	0,938268744
1565612_at	DYNLRB1	-0,924000698	-1,862269442	0,938268744
205820_s_at	APOC3	-0,924000698	-1,862269442	0,938268744
214242_at	MAN1A2	-0,924000698	-1,862269442	0,938268744
206208_at	CA4	-0,924000698	-1,862269442	0,938268744
229276_at	IGSF9	-0,924000698	-1,862269442	0,938268744
1554331_a_at	LRRC18	-0,924000698	-1,862269442	0,938268744
201431_s_at	DPYSL3	-0,924000698	-1,862269442	0,938268744
208728_s_at	CDC42	5,65533291	4,71743974	0,93789317
215492_x_at	PTCRA	1,047341799	0,109458907	0,937882892
227804_at	TLCD1	0,735999505	-0,201789721	0,937789226
203062_s_at	MDC1	3,936891546	2,999351239	0,937540307
223448_x_at	MRPS22	4,109088344	3,171631256	0,937457088
203129_s_at	KIF5C	0,873789652	-0,063377083	0,937166735
223354_x_at	MFF	3,265594257	2,328492509	0,937101748
203516_at	SNTA1	0,820160788	-0,116767475	0,936928263
211554_s_at	APAF1	0,820160788	-0,116767475	0,936928263

212275_s_at	SRCAP	0,791908897	-0,144765583	0,93667448
213322_at	C6orf130	3,915780568	2,979585083	0,936195484
203513_at	SPG11	3,807915835	2,872127595	0,93578824
214465_at	ORM1 /// ORM2	-0,730013898	-1,665791776	0,935777878
219695_at	SMPD3	-0,730013898	-1,665791776	0,935777878
236363_at	LSM3	-0,730013898	-1,665791776	0,935777878
217484_at	CR1	-0,730013898	-1,665791776	0,935777878
1553045_at	WNT9A	-0,730013898	-1,665791776	0,935777878
244809_at	-	-0,730013898	-1,665791776	0,935777878
232243_at	MCPH1	-0,730013898	-1,665791776	0,935777878
211817_s_at	KCNJ5	-0,730013898	-1,665791776	0,935777878
227883_at	CCDC71L	-0,730013898	-1,665791776	0,935777878
228884_at	LRRC27	-0,730013898	-1,665791776	0,935777878
236176_at	-	-0,730013898	-1,665791776	0,935777878
228098_s_at	MYLIP	3,218770562	2,283169614	0,935600948
214609_at	PHOX2A	0,08619576	-0,84928999	0,93548575
226672_s_at	HDAC10	0,08619576	-0,84928999	0,93548575
202122_s_at	PLIN3	3,132805465	2,197404429	0,935401036
232363_at	-	-0,437449947	-1,372734086	0,935284138
237251_at	LRRC71	-0,437449947	-1,372734086	0,935284138
234086_at	-	-0,437449947	-1,372734086	0,935284138
237929_at	C17orf50	-0,437449947	-1,372734086	0,935284138
214677_x_at	IGLC1	-0,437449947	-1,372734086	0,935284138
231514_at	C1orf94	-0,437449947	-1,372734086	0,935284138
206839_at	C22orf31	-0,437449947	-1,372734086	0,935284138
215501_s_at	DUSP10	3,09217775	2,157148588	0,935029161
211558_s_at	DHPS	3,795218988	2,860202326	0,935016661
207923_x_at	PAX8	0,045517965	-0,889222211	0,934740176
230571_at	-	0,045517965	-0,889222211	0,934740176
207554_x_at	TBXA2R	0,045517965	-0,889222211	0,934740176
1560081_at	LOC100505648	0,045517965	-0,889222211	0,934740176
235256_s_at	GALM	3,562907192	2,628204186	0,934703006
215189_at	KRT86 /// LOC100!	4,274951805	3,340400371	0,934551433
202943_s_at	NAGA	2,642918178	1,709047448	0,933870731
203317_at	PSD4	1,843966654	0,910483921	0,933482734
243634_at	-	0,001114523	-0,931539147	0,93265367
216386_at	LOC220077	0,001114523	-0,931539147	0,93265367
231884_at	CNTROB	0,001114523	-0,931539147	0,93265367
243639_at	-	0,001114523	-0,931539147	0,93265367
204743_at	TAGLN3	0,001114523	-0,931539147	0,93265367
239073_at	ANKFY1	0,001114523	-0,931539147	0,93265367
209403_at	LOC100510707 ///	2,25755351	1,324949741	0,932603769
225565_at	CREB1	2,304041045	1,371634653	0,932406392
208709_s_at	NRD1	4,661121858	3,728800162	0,932321695
201968_s_at	PGM1	4,823280506	3,891666284	0,931614222
207738_s_at	NCKAP1	2,33082239	1,399283506	0,931538884
39705_at	SIN3B	1,364964803	0,433947758	0,931017045
220926_s_at	EDEM3	2,179708195	1,249194639	0,930513556
203655_at	XRCC1	2,365121417	1,434743608	0,930377809
202864_s_at	SP100	3,473999051	2,543655878	0,930343173

203011_at	IMPA1	5,338118798	4,407929795	0,930189002
204068_at	STK3	2,829929672	1,899778623	0,930151049
1552749_a_at	KLC3	1,730929079	0,80114751	0,929781569
202114_at	SNX2	3,09217775	2,162541631	0,929636119
222822_s_at	MMRN2	-1,212652659	-2,142286822	0,929634162
1570393_at	EML5	-1,212652659	-2,142286822	0,929634162
213335_s_at	ST3GAL6	-1,212652659	-2,142286822	0,929634162
1558477_at	LOC100131496	-1,212652659	-2,142286822	0,929634162
234237_s_at	AMBRA1	-1,212652659	-2,142286822	0,929634162
220513_at	KHDC1L	-1,212652659	-2,142286822	0,929634162
1553390_at	ATG9B	-1,212652659	-2,142286822	0,929634162
1552954_at	C5orf17	-1,212652659	-2,142286822	0,929634162
215740_at	LOC100130741	-1,212652659	-2,142286822	0,929634162
217014_s_at	AZGP1 /// AZGP1P	-1,212652659	-2,142286822	0,929634162
221114_at	AMBN	-1,212652659	-2,142286822	0,929634162
234469_at	OR51B4	-1,212652659	-2,142286822	0,929634162
215680_at	KIAA1654	-1,212652659	-2,142286822	0,929634162
205734_s_at	AFF3	-1,212652659	-2,142286822	0,929634162
223529_at	SYT4	-1,212652659	-2,142286822	0,929634162
218154_at	GSDMD	2,51558601	1,586121802	0,929464208
237500_at	-	-0,49284695	-1,422257026	0,929410076
220634_at	TBX4	-0,49284695	-1,422257026	0,929410076
219298_at	ECHDC3	-0,49284695	-1,422257026	0,929410076
205428_s_at	CALB2	-0,49284695	-1,422257026	0,929410076
217342_x_at	FLJ11292	-0,49284695	-1,422257026	0,929410076
1566577_at	-	-0,49284695	-1,422257026	0,929410076
1554782_at	KLHL30-AS1	-0,49284695	-1,422257026	0,929410076
230340_s_at	WASL	-0,49284695	-1,422257026	0,929410076
1566839_at	LOC283674	-0,49284695	-1,422257026	0,929410076
210367_s_at	PTGES	-0,49284695	-1,422257026	0,929410076
229840_at	IQSEC2	-0,49284695	-1,422257026	0,929410076
200070_at	CNPPD1	3,102023286	2,173028091	0,928995195
205230_at	RPH3A	-0,043925712	-0,972893339	0,928967627
1563816_at	GAB4	-0,043925712	-0,972893339	0,928967627
221376_at	FGF17	-0,043925712	-0,972893339	0,928967627
206510_at	SIX2	-0,043925712	-0,972893339	0,928967627
235582_at	E2F2	-0,043925712	-0,972893339	0,928967627
1552948_at	CCDC27	-0,043925712	-0,972893339	0,928967627
210671_x_at	MAPK8	-0,043925712	-0,972893339	0,928967627
216377_x_at	ALPPL2	-0,043925712	-0,972893339	0,928967627
213337_s_at	SOCS1	-0,043925712	-0,972893339	0,928967627
214152_at	CCPG1 /// DYX1C1	1,701542258	0,772793615	0,928748643
1555181_a_at	ST3GAL3	2,063971513	1,135235995	0,928735518
1555522_s_at	DPY30 /// MEMO1	4,165622052	3,236954658	0,928667394
1569689_s_at	GABRB3	-1,537851782	-2,466349414	0,928497632
1554766_s_at	-	-1,537851782	-2,466349414	0,928497632
233524_at	LINC00475	-1,537851782	-2,466349414	0,928497632
231557_at	-	-1,537851782	-2,466349414	0,928497632
221721_s_at	LZTS1	-1,537851782	-2,466349414	0,928497632
243752_s_at	CYTH3	-1,537851782	-2,466349414	0,928497632

243374_x_at	-	-1,537851782	-2,466349414	0,928497632
204520_x_at	BRD1	3,055618382	2,127337555	0,928280827
228834_at	-	1,936380191	1,008210238	0,928169954
204715_at	PANX1	1,528073958	0,600385088	0,92768887
1555922_at	C10orf114	1,544940605	0,61779983	0,927140775
223179_at	YPEL3	1,92317941	0,996453274	0,926726137
207743_at	-	-0,088974936	-1,015398016	0,926423081
234357_at	-	-0,088974936	-1,015398016	0,926423081
216455_at	-	-0,088974936	-1,015398016	0,926423081
223944_at	NLRP12	-0,088974936	-1,015398016	0,926423081
218410_s_at	PGP	-0,088974936	-1,015398016	0,926423081
242242_at	USP6	-0,088974936	-1,015398016	0,926423081
235962_at	AZI2	-0,088974936	-1,015398016	0,926423081
239217_x_at	ABCC3	-0,088974936	-1,015398016	0,926423081
202378_s_at	LEPROT	4,005144537	3,078940895	0,926203642
218234_at	ING4	2,849132494	1,923216608	0,925915886
217949_s_at	VKORC1	4,328738561	3,403106144	0,925632416
233759_s_at	SMEK2	4,66447449	3,738875087	0,925599403
217838_s_at	EVL	4,858503557	3,933228921	0,925274637
221130_s_at	-	-0,791294935	-1,715576125	0,92428119
1552375_at	ZNF333	-0,791294935	-1,715576125	0,92428119
1558299_at	-	-0,791294935	-1,715576125	0,92428119
209373_at	MALL	-0,791294935	-1,715576125	0,92428119
1558849_at	LOC284276	-0,791294935	-1,715576125	0,92428119
205927_s_at	CTSE	-0,791294935	-1,715576125	0,92428119
205720_at	POMC	-0,791294935	-1,715576125	0,92428119
208584_at	SNCG	-0,791294935	-1,715576125	0,92428119
218294_s_at	NUP50	3,521615383	2,597466271	0,924149112
226767_s_at	FAHD1	1,421391637	0,497424714	0,923966923
212704_at	ZCCHC11	2,811067138	1,887158627	0,92390851
226277_at	COL4A3BP	3,498020785	2,574574485	0,9234463
51146_at	PIGV	3,009682686	2,086656468	0,923026218
204645_at	CCNT2	2,285848435	1,362900801	0,922947634
1556262_at	PWRN1	-1,712732543	-2,635549085	0,922816542
1567238_at	OR2L2	-1,712732543	-2,635549085	0,922816542
244522_at	-	-1,712732543	-2,635549085	0,922816542
241307_at	-	-1,712732543	-2,635549085	0,922816542
232888_at	ISM1	-1,712732543	-2,635549085	0,922816542
1561462_at	-	-1,712732543	-2,635549085	0,922816542
202037_s_at	SFRP1	-0,135065865	-1,057301851	0,922235986
202400_s_at	SRF	-0,135065865	-1,057301851	0,922235986
243212_at	LOC100505820	-0,135065865	-1,057301851	0,922235986
242181_at	-	-0,135065865	-1,057301851	0,922235986
1555269_a_at	ANO1	-0,135065865	-1,057301851	0,922235986
1553722_s_at	RNF152	-1,982324124	-2,904470894	0,92214677
222788_s_at	RSBN1	1,383446753	0,461385738	0,922061015
219741_x_at	ZNF552	2,348178434	1,426280426	0,921898008
227072_at	RTTN	2,507817181	1,586121802	0,92169538
204949_at	ICAM3	5,247494373	4,325963114	0,921531259
225693_s_at	CAMTA1	5,299553227	4,378022232	0,921530995

202548_s_at	ARHGEF7	4,677091148	3,755752479	0,921338669
208306_x_at	HLA-DRB1 /// LOC:	6,414538392	5,493299971	0,921238422
1560228_at	SNAI3	-0,551284523	-1,471833228	0,920548705
204911_s_at	TRIM3	-0,551284523	-1,471833228	0,920548705
238583_at	MSRB3	-0,551284523	-1,471833228	0,920548705
227154_at	IGSF21	-0,551284523	-1,471833228	0,920548705
234763_at	-	-0,551284523	-1,471833228	0,920548705
211545_at	GHRHR	-0,551284523	-1,471833228	0,920548705
1566926_at	LINC00527	-0,551284523	-1,471833228	0,920548705
236746_at	GALNT1	-0,551284523	-1,471833228	0,920548705
229217_at	SP3	-0,551284523	-1,471833228	0,920548705
221936_x_at	MRPL41	-0,551284523	-1,471833228	0,920548705
233850_s_at	EBF4	-0,551284523	-1,471833228	0,920548705
244507_at	-	-1,802716385	-2,723114724	0,920398339
1557451_at	DGCR10	-1,802716385	-2,723114724	0,920398339
242773_at	SLC5A1	-0,180990326	-1,100943374	0,919953048
230881_at	CCDC42	-0,180990326	-1,100943374	0,919953048
224506_s_at	PPAPDC3	-0,180990326	-1,100943374	0,919953048
207015_s_at	ALDH1A2	-0,180990326	-1,100943374	0,919953048
203365_s_at	MMP15	-0,180990326	-1,100943374	0,919953048
220284_at	DKKL1	-0,180990326	-1,100943374	0,919953048
210640_s_at	GPER	-0,180990326	-1,100943374	0,919953048
210225_x_at	LILRA6 /// LILRB3	-0,180990326	-1,100943374	0,919953048
210212_x_at	MTCP1NB	3,991087145	3,071469991	0,919617154
209432_s_at	CREB3	3,257413106	2,338020736	0,91939237
219880_at	LOC100507619	3,257413106	2,338020736	0,91939237
223948_s_at	TMPRSS3	1,802733148	0,883374248	0,919358899
226191_at	GSK3B	3,481948861	2,562621495	0,919327366
201292_at	TOP2A	5,496988745	4,577703567	0,919285179
223210_at	CHURC1	3,086998005	2,167793428	0,919204577
225719_s_at	MRPL55	2,824160383	1,905294823	0,918865559
200765_x_at	CTNNA1	2,613382867	1,694547353	0,918835514
215004_s_at	SUGP1	1,775035811	0,85655369	0,918482122
225948_at	APOPT1 /// KLC1	4,258822873	3,340400371	0,918422502
225014_at	C4orf52	4,346015214	3,427694878	0,918320337
207283_at	RPL23AP32	2,063971513	1,146010634	0,917960879
202991_at	STARD3	1,20396005	0,286279868	0,917680182
211733_x_at	SCP2	5,882161375	4,964504886	0,917656489
238057_at	USP45	1,760255626	0,842599219	0,917656406
240064_at	-	1,182925501	0,265475485	0,917450016
205666_at	FMO1	-0,994186487	-1,911206569	0,917020082
237055_at	LOC100506174	-0,994186487	-1,911206569	0,917020082
233112_at	-	-0,994186487	-1,911206569	0,917020082
209663_s_at	ITGA7	-0,994186487	-1,911206569	0,917020082
205620_at	F10	-0,994186487	-1,911206569	0,917020082
228154_at	C19orf44	-0,994186487	-1,911206569	0,917020082
233988_x_at	SCUBE1	-0,994186487	-1,911206569	0,917020082
236549_x_at	-	-0,994186487	-1,911206569	0,917020082
217172_at	-	-0,994186487	-1,911206569	0,917020082
207059_at	PAX9	-0,994186487	-1,911206569	0,917020082

1562272_at	-	-0,994186487	-1,911206569	0,917020082
226950_at	ACVRL1	-0,994186487	-1,911206569	0,917020082
239530_at	ADD2	-0,994186487	-1,911206569	0,917020082
228942_s_at	DAB2IP	-0,994186487	-1,911206569	0,917020082
228730_s_at	SCRN2	1,995527416	1,078619386	0,91690803
206936_x_at	-	1,995527416	1,078619386	0,91690803
223347_at	MUM1	1,139057614	0,222432814	0,9166248
225092_at	RABEP1	3,340803206	2,424202743	0,916600463
223256_at	G2E3	2,590269697	1,674071917	0,916197781
226518_at	KCTD10	3,171328694	2,255165935	0,91616276
206765_at	KCNJ2	1,936380191	1,020514937	0,915865255
222480_at	UBE2Q1	1,116462765	0,20062106	0,915841705
225510_at	OAF	1,116462765	0,20062106	0,915841705
238541_at	C21orf58	1,071336699	0,155747281	0,915589418
244519_at	ASXL1	3,127551022	2,212304392	0,915246629
225230_at	DRAM2	4,091994743	3,176868665	0,915126078
220411_x_at	PODNL1	1,000459215	0,085534992	0,914924222
227571_at	-	1,716003175	0,80114751	0,914855665
220788_s_at	IRF9 /// RNF31	1,024284941	0,109458907	0,914826034
241730_at	MYNN	-0,230432956	-1,145057014	0,914624058
214478_at	SPP2	-0,230432956	-1,145057014	0,914624058
1553239_at	FAM124A	-0,230432956	-1,145057014	0,914624058
236055_at	DQX1	-0,230432956	-1,145057014	0,914624058
217472_at	-	-0,230432956	-1,145057014	0,914624058
205447_s_at	MAP3K12	0,95222556	0,03784737	0,91437819
208662_s_at	TTC3 /// TTC3P1	4,725757944	3,811401547	0,914356397
215498_s_at	MAP2K3	2,537731447	1,623485524	0,914245924
225418_at	PVRL2	1,047341799	0,133104519	0,91423728
232149_s_at	NSMAF	2,492739983	1,578991006	0,913748977
231912_s_at	TECPR1	0,926325262	0,013190398	0,913134864
217135_x_at	-	0,926325262	0,013190398	0,913134864
1553695_a_at	NLRX1	0,926325262	0,013190398	0,913134864
238753_at	NCS1	0,926325262	0,013190398	0,913134864
203919_at	TCEA2	2,21987062	1,306903969	0,912966651
233118_at	-	-1,893506789	-2,8064286	0,912921811
219643_at	LRP1B	-1,893506789	-2,8064286	0,912921811
1567377_at	DNAH1	-1,893506789	-2,8064286	0,912921811
225768_at	NR1D2	1,65554524	0,742671819	0,91287342
227047_x_at	ZBTB4	1,65554524	0,742671819	0,91287342
225068_at	KLHL12	4,600469559	3,687638429	0,91283113
1553626_a_at	C17orf57	-1,137208348	-2,049747196	0,912538848
231367_s_at	-	-1,137208348	-2,049747196	0,912538848
205499_at	SRPX2	-1,137208348	-2,049747196	0,912538848
1563830_a_at	FHAD1	-1,137208348	-2,049747196	0,912538848
1552725_s_at	ADAMTS17	-1,137208348	-2,049747196	0,912538848
1563108_at	ELFN2	-1,137208348	-2,049747196	0,912538848
1553369_at	FAM129C	-1,137208348	-2,049747196	0,912538848
211915_s_at	TUBB7P	-1,137208348	-2,049747196	0,912538848
224588_at	XIST	-1,137208348	-2,049747196	0,912538848
206455_s_at	RHO	-1,137208348	-2,049747196	0,912538848

226915_s_at	ARPC5L	4,335356502	3,422991983	0,912364519
225723_at	CCDC167	4,784078567	3,871747013	0,912331554
204615_x_at	IDI1	5,768810925	4,856717562	0,912093363
202696_at	OXSR1	4,328738561	3,416886612	0,911851949
227332_at	PXN-AS1	2,209870035	1,298349073	0,911520962
204585_s_at	L1CAM	0,524545436	-0,386896102	0,911441538
231674_at	-	-1,624955693	-2,536339282	0,911383588
224941_at	PAPPA	-1,624955693	-2,536339282	0,911383588
1566285_at	-	-1,624955693	-2,536339282	0,911383588
1566441_at	-	-1,624955693	-2,536339282	0,911383588
1556997_a_at	LEPR	-1,624955693	-2,536339282	0,911383588
215752_at	SIK2	-1,624955693	-2,536339282	0,911383588
236177_s_at	-	-1,624955693	-2,536339282	0,911383588
1562309_s_at	LOC100506695 ///	-1,624955693	-2,536339282	0,911383588
205407_at	RECK	0,556428218	-0,354781582	0,911209799
211325_x_at	DSTNP2	2,179708195	1,268569037	0,911139158
209028_s_at	ABI1	4,116324812	3,205255599	0,911069212
1556178_x_at	TAF8	0,900065578	-0,011001236	0,911066814
212339_at	EPB41L1	-0,609040214	-1,520022123	0,91098191
229654_at	-	-0,609040214	-1,520022123	0,91098191
244221_at	-	-0,609040214	-1,520022123	0,91098191
221346_at	OR10J1	-0,609040214	-1,520022123	0,91098191
240421_x_at	SAV1	-0,609040214	-1,520022123	0,91098191
241840_at	-	-0,609040214	-1,520022123	0,91098191
229991_s_at	SYTL4	-0,609040214	-1,520022123	0,91098191
230347_at	ORAI2	-0,609040214	-1,520022123	0,91098191
211260_at	BMP7	1,544940605	0,634301128	0,910639477
214596_at	CHRM3	1,510970097	0,600385088	0,910585009
216206_x_at	MAP2K7	-0,280273599	-1,19085694	0,910583341
214025_at	DDX28	-0,280273599	-1,19085694	0,910583341
210344_at	OSBPL7	-0,280273599	-1,19085694	0,910583341
210773_s_at	FPR2	-0,280273599	-1,19085694	0,910583341
230246_at	PLAC9	-0,280273599	-1,19085694	0,910583341
205778_at	KLK7	-0,280273599	-1,19085694	0,910583341
206324_s_at	DAPK2	-0,280273599	-1,19085694	0,910583341
213369_at	CDHR1	-0,280273599	-1,19085694	0,910583341
233623_at	-	-0,280273599	-1,19085694	0,910583341
1553770_a_at	SLAMF9	-0,280273599	-1,19085694	0,910583341
230649_at	C1orf86	-0,280273599	-1,19085694	0,910583341
227024_s_at	MRPL55	-0,280273599	-1,19085694	0,910583341
211350_s_at	KIF25-AS1	0,355751005	-0,554696666	0,910447671
234062_at	-	0,355751005	-0,554696666	0,910447671
227846_at	GPR176	0,355751005	-0,554696666	0,910447671
206817_x_at	CELF3	0,355751005	-0,554696666	0,910447671
213257_at	SARM1	0,355751005	-0,554696666	0,910447671
237518_at	-	0,355751005	-0,554696666	0,910447671
228823_at	POLR2J2	0,873789652	-0,036543687	0,910333339
201481_s_at	PYGB	2,149438457	1,239268196	0,910170262
235958_at	PLA2G4F	0,491906512	-0,418248858	0,91015537
203918_at	PCDH1	0,491906512	-0,418248858	0,91015537

229354_at	AHRR	4,324702656	3,414783177	0,909919479
232340_at	LOC100507637	0,820160788	-0,089635713	0,909796501
1555562_a_at	ZCCHC7	0,820160788	-0,089635713	0,909796501
226667_x_at	EPN1	0,820160788	-0,089635713	0,909796501
216417_x_at	HOXB9	0,820160788	-0,089635713	0,909796501
217095_x_at	NCR1	0,425036312	-0,484558493	0,909594806
225482_at	KIF1A	0,425036312	-0,484558493	0,909594806
231003_at	SLC35B3	0,707657549	-0,201789721	0,90944727
1566002_at	-	0,39105295	-0,518232988	0,909285939
1559921_at	PECAM1	0,39105295	-0,518232988	0,909285939
211269_s_at	IL2RA	0,39105295	-0,518232988	0,909285939
220731_s_at	NECAP2	3,966733349	3,05768517	0,909048179
204251_s_at	CEP164	0,764143511	-0,144765583	0,908909094
205059_s_at	IDUA	0,648195588	-0,260526297	0,908721885
241201_at	UVSSA	0,648195588	-0,260526297	0,908721885
226640_at	DAGLB	0,648195588	-0,260526297	0,908721885
218145_at	TRIB3	3,081633533	2,173028091	0,908605441
218679_s_at	VPS28	5,069714167	4,161147622	0,908566544
238102_s_at	BTBD19	0,319287178	-0,589001171	0,908288349
212393_at	SBF1	1,458255986	0,550017041	0,908238946
228312_at	PI16	0,735999505	-0,172194225	0,90819373
226249_at	SNX30	0,735999505	-0,172194225	0,90819373
211065_x_at	PFKL	2,692288672	1,784182261	0,908106411
204507_s_at	PPP3R1	3,200057698	2,292099323	0,907958375
204842_x_at	PRKAR2A	3,577387089	2,669456704	0,907930385
1555460_a_at	SLC39A6	2,107136847	1,199296812	0,907840034
230930_at	LOC338620	-0,856259172	-1,763664074	0,907404902
243061_at	C14orf23	-0,856259172	-1,763664074	0,907404902
236218_at	PHOSPHO1	-0,856259172	-1,763664074	0,907404902
239977_at	C12orf42	-0,856259172	-1,763664074	0,907404902
234552_at	EPPK1	-0,856259172	-1,763664074	0,907404902
1554371_at	PKD1L2	-0,856259172	-1,763664074	0,907404902
205563_at	KISS1	-0,856259172	-1,763664074	0,907404902
232189_at	-	-0,856259172	-1,763664074	0,907404902
234421_s_at	EVC	-0,856259172	-1,763664074	0,907404902
221283_at	RUNX2	-0,856259172	-1,763664074	0,907404902
1568616_a_at	-	-0,856259172	-1,763664074	0,907404902
201261_x_at	BGN	-0,856259172	-1,763664074	0,907404902
206237_s_at	NRG1	-0,856259172	-1,763664074	0,907404902
204037_at	LPAR1	-0,856259172	-1,763664074	0,907404902
207311_at	DOC2B	-0,856259172	-1,763664074	0,907404902
203880_at	COX17	5,939590196	5,032749039	0,906841156
38918_at	SOX13	-0,856259172	-1,762876337	0,906617165
217457_s_at	RAP1GDS1	3,309267982	2,403191094	0,906076887
234512_x_at	-	6,748786399	5,842880719	0,90590568
221324_at	TAS2R1	0,244681185	-0,661178575	0,90585976
209791_at	PADI2	0,244681185	-0,661178575	0,90585976
1554531_at	TTC12	0,244681185	-0,661178575	0,90585976
230379_x_at	C2orf56	2,811067138	1,905294823	0,905772314
221605_s_at	PIPOX	0,282358733	-0,623254098	0,905612831

222060_at	KRT8P12	0,282358733	-0,623254098	0,905612831
231035_s_at	-	0,282358733	-0,623254098	0,905612831
216389_s_at	DCAF11	1,983901861	1,078619386	0,905282475
226423_at	PAQR8	3,954683435	3,049587049	0,905096386
202640_s_at	RANBP3	2,295108393	1,390070295	0,905038098
201886_at	DCAF11	2,304041045	1,399283506	0,90475754
219141_s_at	AMBRA1	2,304041045	1,399283506	0,90475754
229957_at	TMEM91	0,20740896	-0,697182596	0,904591557
240813_at	-	0,20740896	-0,697182596	0,904591557
208885_at	LCP1	6,391658904	5,487366217	0,904292687
223411_at	MIF4GD	4,389611304	3,485746794	0,90386451
226835_s_at	ZNFX1-AS1	6,367840843	5,464217567	0,903623276
238756_at	GAS2L3	2,75889404	1,855279162	0,903614878
224841_x_at	GAS5 /// SNORD44	6,469797031	5,566195308	0,903601723
203213_at	CDK1	6,277147611	5,373858803	0,903288808
228861_at	CDS2	1,364555912	0,461385738	0,903170174
208663_s_at	TTC3 /// TTC3P1	4,084416075	3,181622175	0,902793901
221967_at	NXPH4	-0,332405896	-1,234835326	0,90242943
231386_at	FAM228A	-0,332405896	-1,234835326	0,90242943
202063_s_at	SEL1L	-0,332405896	-1,234835326	0,90242943
203670_at	ARPC4-TTLL3 /// T	-0,332405896	-1,234835326	0,90242943
235015_at	ZDHHC9	-0,332405896	-1,234835326	0,90242943
241380_at	ARHGEF37	-0,332405896	-1,234835326	0,90242943
1557757_at	-	-0,332405896	-1,234835326	0,90242943
205607_s_at	SCYL3	3,296548503	2,394276989	0,902271514
223387_at	ZFYVE1	2,849132494	1,946903052	0,902229442
226107_at	C1GALT1	1,287793882	0,385582632	0,90221125
219829_at	ITGB1BP2	0,167727503	-0,734355396	0,902082899
223747_x_at	WWOX	0,167727503	-0,734355396	0,902082899
205970_at	MT3	0,167727503	-0,734355396	0,902082899
206888_s_at	ARHGDIG	0,167727503	-0,734355396	0,902082899
212829_at	PIP4K2A	5,350868606	4,449190565	0,901678041
238600_at	JAKMIP1	4,448374788	3,546728932	0,901645856
225850_at	SFT2D1	6,343295352	5,44197619	0,901319162
202071_at	SDC4	2,698955954	1,797682937	0,901273017
212968_at	RFNG	1,267146969	0,366031214	0,901115756
205865_at	ARID3A	1,88491011	0,98381571	0,901094401
221568_s_at	LIN7C	3,966733349	3,065641037	0,901092313
212796_s_at	TBC1D2B	3,697863752	2,796775008	0,901088743
210768_x_at	TMCO1	3,566680842	2,665727652	0,900953191
214771_x_at	MPRIP	4,030423661	3,129477148	0,900946514
212936_at	FAM172A	2,824160383	1,923216608	0,900943775
212238_at	ASXL1	2,545254728	1,644429915	0,900824813
212982_at	ZDHHC17	2,966903803	2,066329863	0,90057394
235978_at	FABP4	-1,369772723	-2,27001641	0,900243687
214488_at	RAP2B	-1,369772723	-2,27001641	0,900243687
233353_at	FER1L5	-1,369772723	-2,27001641	0,900243687
1559785_at	TPPP2	-1,369772723	-2,27001641	0,900243687
214979_at	-	-1,369772723	-2,27001641	0,900243687
238312_s_at	B4GALT3	-1,369772723	-2,27001641	0,900243687

1560952_at	-	-1,369772723	-2,27001641	0,900243687
1563296_at	LINC00572	-1,369772723	-2,27001641	0,900243687
229655_at	FAM19A5	-1,369772723	-2,27001641	0,900243687
217184_s_at	LTK	-1,369772723	-2,27001641	0,900243687
236750_at	-	-1,369772723	-2,27001641	0,900243687
231758_at	PRM3	-1,369772723	-2,27001641	0,900243687
216916_s_at	DLGAP2	-1,369772723	-2,27001641	0,900243687
236445_at	-	-1,369772723	-2,27001641	0,900243687
211401_s_at	FGFR2	-1,369772723	-2,27001641	0,900243687
226148_at	ZBTB44	3,748268542	2,848029002	0,90023954
1552612_at	CDC42SE2	3,112433079	2,212304392	0,900128686
244103_at	SDE2	4,939708493	4,040084244	0,899624248
217289_s_at	SLC37A4	1,246401134	0,346964736	0,899436398
229132_at	MINA	1,246401134	0,346964736	0,899436398
218364_at	LRRFIP2	3,336134047	2,436714502	0,899419545
207987_s_at	GNRH1	0,127732831	-0,771340337	0,899073168
234885_at	-	0,127732831	-0,771340337	0,899073168
238106_at	-	0,127732831	-0,771340337	0,899073168
239783_at	-	0,127732831	-0,771340337	0,899073168
1563902_at	-	0,127732831	-0,771340337	0,899073168
1556279_at	-	-0,66934516	-1,568385657	0,899040497
234515_at	PCGEM1	-0,66934516	-1,568385657	0,899040497
225249_at	SPPL2B	-0,66934516	-1,568385657	0,899040497
1553740_a_at	IRAK2	-0,66934516	-1,568385657	0,899040497
232639_at	C3orf25	-0,66934516	-1,568385657	0,899040497
214489_at	FSHB	-0,66934516	-1,568385657	0,899040497
233619_at	-	-0,66934516	-1,568385657	0,899040497
239226_at	-	-0,66934516	-1,568385657	0,899040497
224510_s_at	CLPB	-0,66934516	-1,568385657	0,899040497
1565812_at	TRIM36	-0,66934516	-1,568385657	0,899040497
244746_at	SEMA6D	-0,66934516	-1,568385657	0,899040497
231720_s_at	JAM3	-0,66934516	-1,568385657	0,899040497
212413_at	38961	3,081633533	2,182711118	0,898922415
35179_at	B3GAT3	0,913255166	0,014519549	0,898735617
236875_at	LOC100130705	-0,384193355	-1,282520722	0,898327367
240941_at	-	-0,384193355	-1,282520722	0,898327367
229990_at	TSC22D1-AS1	-0,384193355	-1,282520722	0,898327367
235073_at	SCAMP4	-0,384193355	-1,282520722	0,898327367
210099_at	ABCA2	-0,384193355	-1,282520722	0,898327367
207258_at	DSCR4	-0,384193355	-1,282520722	0,898327367
211326_x_at	HFE	-0,384193355	-1,282520722	0,898327367
218088_s_at	RRAGC	4,155757614	3,257790126	0,897967488
237621_at	LOC100507007	0,08619576	-0,811769547	0,897965306
202714_s_at	KIAA0391	0,08619576	-0,811769547	0,897965306
221975_s_at	C21orf2	0,08619576	-0,811769547	0,897965306
237049_at	-	-1,982324124	-2,880284116	0,897959992
230411_at	UBE2V2	1,65554524	0,757638486	0,897906753
213974_at	ADAMTSL3	-1,452713826	-2,350444759	0,897730933
229095_s_at	LIMS3 /// LIMS3-L(	-1,452713826	-2,350444759	0,897730933
235382_at	AQPEP	-1,452713826	-2,350444759	0,897730933

229169_at	TTC18	-1,452713826	-2,350444759	0,897730933
240346_at	-	-1,452713826	-2,350444759	0,897730933
205954_at	RXRG	-1,452713826	-2,350444759	0,897730933
240051_at	TPD52L3	-1,452713826	-2,350444759	0,897730933
216820_at	-	-1,452713826	-2,350444759	0,897730933
225189_s_at	RAPH1	-1,452713826	-2,350444759	0,897730933
212143_s_at	IGFBP3	-1,452713826	-2,350444759	0,897730933
200977_s_at	TAX1BP1	5,923116008	5,025516125	0,897599883
231819_at	LOC100505876	1,640193838	0,742671819	0,897522019
227777_at	-	2,053550345	1,156599976	0,896950369
227284_at	ZNF766	3,156891301	2,260052727	0,896838574
226871_s_at	ATG4D	1,182925501	0,286279868	0,896645634
227278_at	TAF13	4,301645306	3,405078581	0,896566725
217851_s_at	SLMO2	4,488031942	3,591688835	0,896343107
207033_at	GIF	-1,289805289	-2,186086603	0,896281314
231122_x_at	ZDHHC19	-1,289805289	-2,186086603	0,896281314
210670_at	PPY	-1,289805289	-2,186086603	0,896281314
1555304_a_at	KCNH5	-1,289805289	-2,186086603	0,896281314
237565_at	-	-1,289805289	-2,186086603	0,896281314
241316_at	-	-1,289805289	-2,186086603	0,896281314
233582_at	-	-1,289805289	-2,186086603	0,896281314
234963_s_at	FA2H	-1,289805289	-2,186086603	0,896281314
213592_at	APLNR	-1,289805289	-2,186086603	0,896281314
1552915_at	IL28A	-1,289805289	-2,186086603	0,896281314
217438_at	-	-1,289805289	-2,186086603	0,896281314
215418_at	PARVA	-1,289805289	-2,186086603	0,896281314
221410_x_at	PCDHB3	-1,289805289	-2,186086603	0,896281314
209230_s_at	NUPR1	-1,289805289	-2,186086603	0,896281314
228191_at	FLVCR1	4,089697574	3,193461514	0,89623606
203932_at	HLA-DMB	4,065340446	3,169167796	0,89617265
225145_at	NCOA5	2,312738346	1,417157926	0,89558042
219062_s_at	ZCCHC2	2,312738346	1,417157926	0,89558042
200764_s_at	CTNNA1	2,312738346	1,417157926	0,89558042
200023_s_at	EIF3F	7,445052115	6,549477086	0,895575029
201690_s_at	TPD52	4,915049349	4,019608479	0,895440869
218500_at	THEM6	1,561816652	0,666486137	0,895330515
1566720_at	RPS10P7	0,045517965	-0,84928999	0,894807955
220396_at	SLC35E3	0,045517965	-0,84928999	0,894807955
224088_at	NMUR2	0,045517965	-0,84928999	0,894807955
1554119_at	C16orf57	0,045517965	-0,84928999	0,894807955
203764_at	DLGAP5	4,65719538	3,762464514	0,894730865
209152_s_at	TCF3	2,25755351	1,362900801	0,894652709
212082_s_at	MYL6	7,374762773	6,48024867	0,894514104
1556985_at	-	-1,06282519	-1,956681069	0,89385588
237942_at	SNRK-AS1	-1,06282519	-1,956681069	0,89385588
234114_at	ZNRD1	-1,06282519	-1,956681069	0,89385588
238916_at	LOC400027	-1,06282519	-1,956681069	0,89385588
216429_at	-	-1,06282519	-1,956681069	0,89385588
238152_at	C11orf95	-1,06282519	-1,956681069	0,89385588
217020_at	RARB	-1,06282519	-1,956681069	0,89385588

223121_s_at	SFRP2	-1,06282519	-1,956681069	0,89385588
1561868_at	-	-1,06282519	-1,956681069	0,89385588
236509_at	-	-1,06282519	-1,956681069	0,89385588
216811_at	-	-1,06282519	-1,956681069	0,89385588
221577_x_at	GDF15	-1,06282519	-1,956681069	0,89385588
216579_at	GJB4	-1,06282519	-1,956681069	0,89385588
241677_x_at	-	-1,06282519	-1,956681069	0,89385588
232140_at	-	1,528073958	0,634301128	0,89377283
213011_s_at	TPI1	6,866510004	5,972991786	0,893518218
203118_at	PCSK7	2,811067138	1,917743773	0,893323364
1565454_at	XAGE-4	1,493647653	0,600385088	0,893262565
222816_s_at	ZCCHC2	1,983901861	1,090640407	0,893261454
202618_s_at	MECP2	1,510970097	0,61779983	0,893170267
235800_at	-	-1,802716385	-2,695640852	0,892924467
203184_at	FBN2	-1,802716385	-2,695640852	0,892924467
1555567_s_at	LMOD3	-1,802716385	-2,695640852	0,892924467
223573_s_at	PPP2R2C	-1,802716385	-2,695640852	0,892924467
1560175_at	PPP4R1L	-1,802716385	-2,695640852	0,892924467
220597_s_at	ARL6IP4	3,060716358	2,167793428	0,892922931
201762_s_at	PSME2	6,185481357	5,292947145	0,892534212
223407_at	C16orf48	1,802733148	0,910483921	0,892249227
220036_s_at	LMBR1L	1,802733148	0,910483921	0,892249227
225077_at	CHD2	3,142467702	2,250228409	0,892239293
1555743_s_at	ERVH-6	2,190140919	1,298349073	0,891791846
224581_s_at	NUCKS1	4,590648292	3,698866377	0,891781915
204773_at	IL11RA	1,775035811	0,883374248	0,891661563
62987_r_at	CACNG4	3,037342884	2,145737519	0,891605366
228994_at	CCDC24	0,97663406	0,085534992	0,891099067
213448_at	-	0,97663406	0,085534992	0,891099067
227516_at	SF3A1	2,872644433	1,981728801	0,890915632
225278_at	PRKAB2	2,752615529	1,861868165	0,890747364
206204_at	GRB14	-1,712732543	-2,603471267	0,890738724
214640_at	UNC93A	-1,712732543	-2,603471267	0,890738724
236902_at	FLJ43390	-1,712732543	-2,603471267	0,890738724
238302_at	-	-1,712732543	-2,603471267	0,890738724
211638_at	IGH@ /// IGHA1 //	-1,712732543	-2,603471267	0,890738724
1562112_at	-	-1,712732543	-2,603471267	0,890738724
1557807_a_at	-	-1,712732543	-2,603471267	0,890738724
242991_at	TJP2	-1,712732543	-2,603471267	0,890738724
237524_at	-	-1,712732543	-2,603471267	0,890738724
235560_at	NOVA2	-1,712732543	-2,603471267	0,890738724
230291_s_at	NFIB	-1,712732543	-2,603471267	0,890738724
224848_at	CDK6	3,223556135	2,332940758	0,890615377
1559059_s_at	ZNF611	-1,537851782	-2,4284334	0,890581618
231977_at	GRID1	-1,537851782	-2,4284334	0,890581618
1570196_at	-	-1,537851782	-2,4284334	0,890581618
1555214_a_at	CLEC7A	-1,537851782	-2,4284334	0,890581618
242800_at	NHS	-1,537851782	-2,4284334	0,890581618
1555203_s_at	SLC44A4	-1,537851782	-2,4284334	0,890581618
1552548_at	BSND	-1,537851782	-2,4284334	0,890581618

1564836_at	-	-1,537851782	-2,4284334	0,890581618
233730_at	FAM135A	-1,537851782	-2,4284334	0,890581618
231435_at	C7orf34	-1,537851782	-2,4284334	0,890581618
240301_at	DPPA2	-1,537851782	-2,4284334	0,890581618
206545_at	CD28	-1,537851782	-2,4284334	0,890581618
208052_x_at	CEACAM3	0,95222556	0,061773582	0,890451978
214219_x_at	MAP4K1	2,149438457	1,259081178	0,890357279
236918_s_at	LRRC34	0,001114523	-0,889222211	0,890336734
201269_s_at	NUDCD3	0,001114523	-0,889222211	0,890336734
215304_at	-	0,001114523	-0,889222211	0,890336734
240701_at	GSDMB	0,001114523	-0,889222211	0,890336734
231743_at	WNT3	0,001114523	-0,889222211	0,890336734
241958_at	-	0,001114523	-0,889222211	0,890336734
1556163_a_at	IGSF3	0,001114523	-0,889222211	0,890336734
200645_at	GABARAP	5,904369421	5,014191607	0,890177814
240817_at	-	-0,437449947	-1,32740143	0,889951483
1558119_at	TSPAN17	-0,437449947	-1,32740143	0,889951483
240057_at	-	-0,437449947	-1,32740143	0,889951483
232086_at	PIK3C3	-0,437449947	-1,32740143	0,889951483
215858_at	-	-0,437449947	-1,32740143	0,889951483
217581_at	-	-0,437449947	-1,32740143	0,889951483
1569074_at	SRRM3	-0,437449947	-1,32740143	0,889951483
215661_at	MAST2	-0,437449947	-1,32740143	0,889951483
239882_at	-	-0,437449947	-1,32740143	0,889951483
240200_at	SULT1C2	-0,437449947	-1,32740143	0,889951483
1570405_at	-	-0,437449947	-1,32740143	0,889951483
226491_x_at	PTBP1	-0,437449947	-1,32740143	0,889951483
213013_at	LOC644172 /// MA	-0,437449947	-1,32740143	0,889951483
200002_at	RPL35	7,92704604	7,037112754	0,889933287
203647_s_at	FDX1	5,219908865	4,330024694	0,889884171
201210_at	DDX3X	5,663811129	4,77417019	0,889640939
202963_at	RFX5	4,913150425	4,024017564	0,889132861
202388_at	RGS2	3,649735397	2,760632255	0,889103142
222418_s_at	TMEM43	3,076375717	2,187622928	0,888752789
208761_s_at	SUMO1	5,097429074	4,209377651	0,888051424
1556986_at	OR2H1	-0,924000698	-1,811780055	0,887779357
1569337_at	SLC5A9	-0,924000698	-1,811780055	0,887779357
235468_at	RBFOX3	-0,924000698	-1,811780055	0,887779357
209888_s_at	MYL1	-0,924000698	-1,811780055	0,887779357
239085_at	JDP2	-0,924000698	-1,811780055	0,887779357
216891_at	-	-0,924000698	-1,811780055	0,887779357
224061_at	INMT	-0,924000698	-1,811780055	0,887779357
230822_at	TMEM61	-0,924000698	-1,811780055	0,887779357
1562463_at	-	-0,924000698	-1,811780055	0,887779357
237103_at	-	-0,924000698	-1,811780055	0,887779357
214091_s_at	GPX3	-0,924000698	-1,811780055	0,887779357
1566821_at	LOC100505862	-0,924000698	-1,811780055	0,887779357
1554909_at	CCDC36	-0,924000698	-1,811780055	0,887779357
214468_at	MYH6	-0,924000698	-1,811780055	0,887779357
223884_at	OPTC	-0,924000698	-1,811780055	0,887779357

218371_s_at	PSPC1	2,575362572	1,687645398	0,887717174
219296_at	ZDHHC13	3,060716358	2,173028091	0,887688267
1552953_a_at	NEUROD2	-0,043925712	-0,931539147	0,887613435
232345_at	C18orf8	-0,043925712	-0,931539147	0,887613435
235116_at	TRAF1	2,41945542	1,531877749	0,887577671
224800_at	WDFY1	2,671492308	1,784182261	0,887310047
200901_s_at	M6PR	4,326621119	3,439619824	0,887001295
237771_s_at	-	-0,730013898	-1,616924751	0,886910853
1567011_at	-	-0,730013898	-1,616924751	0,886910853
1556661_at	CN5H6.4	-0,730013898	-1,616924751	0,886910853
232079_s_at	PVRL2	-0,730013898	-1,616924751	0,886910853
209664_x_at	NFATC1	-0,730013898	-1,616924751	0,886910853
234226_at	OPN4	-0,730013898	-1,616924751	0,886910853
1553860_at	DCST1	-0,730013898	-1,616924751	0,886910853
208585_at	BTN2A3P	-0,730013898	-1,616924751	0,886910853
1562797_at	-	-0,730013898	-1,616924751	0,886910853
236263_at	SHH	-0,730013898	-1,616924751	0,886910853
240993_at	-	-0,730013898	-1,616924751	0,886910853
216296_at	CLTA	-0,730013898	-1,616924751	0,886910853
217111_at	AMACR	-0,730013898	-1,616924751	0,886910853
243099_at	NFAM1	-0,730013898	-1,616924751	0,886910853
206694_at	PNLIPRP1	-0,730013898	-1,616924751	0,886910853
201287_s_at	SDC1	-0,730013898	-1,616924751	0,886910853
239791_at	HOXB-AS3	-0,730013898	-1,616924751	0,886910853
223820_at	RBP5	-0,730013898	-1,616924751	0,886910853
217231_s_at	MAST1	-0,730013898	-1,616924751	0,886910853
233107_at	-	-0,730013898	-1,616924751	0,886910853
236095_at	NTRK2	-0,730013898	-1,616924751	0,886910853
225900_at	EXOC6B	0,900065578	0,013190398	0,88687518
221590_s_at	ALDH6A1	0,900065578	0,013190398	0,88687518
238065_at	TPM3	0,900065578	0,013190398	0,88687518
231166_at	GPR155	0,900065578	0,013190398	0,88687518
221792_at	RAB6B	0,900065578	0,013190398	0,88687518
209892_at	FUT4	2,902957057	2,016139502	0,886817555
239507_at	LINC00608	-1,893506789	-2,780225148	0,886718359
1556980_at	-	-1,893506789	-2,780225148	0,886718359
219056_at	RNASEH2B	3,237994092	2,351470274	0,886523818
201056_at	GOLGB1	1,701542258	0,815200271	0,886341987
238860_at	C6orf130	2,530390387	1,644429915	0,885960472
213034_at	SIK3	3,200057698	2,314488991	0,885568707
208771_s_at	LTA4H	5,644269945	4,758953214	0,885316731
40489_at	ATN1	0,754945175	-0,130324496	0,885269671
204691_x_at	PLA2G6	1,671397663	0,786391549	0,885006114
218324_s_at	SPATS2	2,621163856	1,736450458	0,884713398
214943_s_at	ARID4B /// RBM34	2,507817181	1,623485524	0,884331658
244210_at	-	-1,212652659	-2,09678811	0,884135451
221623_at	BCAN	-1,212652659	-2,09678811	0,884135451
1561228_at	LOC100506122	-1,212652659	-2,09678811	0,884135451
1552733_at	KLHDC1	-1,212652659	-2,09678811	0,884135451
233975_at	PRNT	-1,212652659	-2,09678811	0,884135451

206832_s_at	SEMA3F	-1,212652659	-2,09678811	0,884135451
1569561_at	-	-1,212652659	-2,09678811	0,884135451
244003_at	-	-1,212652659	-2,09678811	0,884135451
239987_at	-	-1,212652659	-2,09678811	0,884135451
234651_at	-	-1,212652659	-2,09678811	0,884135451
235150_at	SESN3	-1,212652659	-2,09678811	0,884135451
1561508_at	-	-1,212652659	-2,09678811	0,884135451
223372_at	NUDT22	-1,212652659	-2,09678811	0,884135451
208161_s_at	ABCC3	-1,212652659	-2,09678811	0,884135451
220564_at	RNLS	-1,212652659	-2,09678811	0,884135451
243085_at	-	-1,212652659	-2,09678811	0,884135451
1552806_a_at	SIGLEC10	-1,212652659	-2,09678811	0,884135451
204636_at	COL17A1	-1,212652659	-2,09678811	0,884135451
236926_at	TBX1	-1,212652659	-2,09678811	0,884135451
208228_s_at	FGFR2	-1,212652659	-2,09678811	0,884135451
239592_at	-	0,847529938	-0,036543687	0,884073625
224069_x_at	P2RX2	-0,088974936	-0,972893339	0,883918403
217011_at	GBX1	-0,088974936	-0,972893339	0,883918403
201808_s_at	ENG	-0,088974936	-0,972893339	0,883918403
237131_at	RIIAD1	-0,088974936	-0,972893339	0,883918403
213651_at	INPP5J	-0,088974936	-0,972893339	0,883918403
200640_at	YWHAZ	6,292536084	5,408938392	0,883597693
1555981_at	C17orf65	0,820160788	-0,063377083	0,883537871
202099_s_at	DGCR2	1,326463531	0,443143223	0,883320308
212038_s_at	VDAC1	6,499221118	5,616187073	0,883034044
219203_at	EMC9	2,725848647	1,842852096	0,882996551
211717_at	ANKRD40	1,625482993	0,742671819	0,882811174
238937_at	ZNF420	1,307201325	0,424693306	0,882508019
212414_s_at	GLYR1 /// SEPT6	5,113232546	4,230731855	0,882500691
212109_at	HN1L	1,960819106	1,078619386	0,88219972
226236_at	LINC00493	5,473061201	4,590948887	0,882112313
221742_at	CELF1	2,926639205	2,044580955	0,88205825
201643_x_at	KDM3B	3,697863752	2,81616114	0,881702612
216221_s_at	PUM2	5,14119811	4,25950843	0,88168968
1561666_a_at	KIAA1908	0,791908897	-0,089635713	0,88154461
229303_at	-	0,791908897	-0,089635713	0,88154461
1558801_at	-	3,317909135	2,436714502	0,881194633
1555222_a_at	-	0,764143511	-0,116767475	0,880910986
226158_at	KLHL24	0,735999505	-0,144765583	0,880765088
219924_s_at	ZMYM6	4,149350729	3,26868404	0,880666689
202361_at	SEC24C	3,954683435	3,074131603	0,880551832
227421_at	YBEY	3,493994216	2,613615641	0,880378576
221421_s_at	ADAMTS12	-0,135065865	-1,015398016	0,880332151
216082_at	NEU3	-0,135065865	-1,015398016	0,880332151
211484_s_at	DSCAM	-0,135065865	-1,015398016	0,880332151
231360_at	C20orf141	-0,135065865	-1,015398016	0,880332151
49049_at	DTX3	-0,129911888	-1,009988476	0,880076587
229155_at	-	-0,49284695	-1,372734086	0,879887135
1555289_at	SEC16B	-0,49284695	-1,372734086	0,879887135
235867_at	GSTM3	-0,49284695	-1,372734086	0,879887135

202410_x_at	IGF2 /// INS-IGF2	-0,49284695	-1,372734086	0,879887135
206600_s_at	SLC16A5	-0,49284695	-1,372734086	0,879887135
213290_at	COL6A2	-0,49284695	-1,372734086	0,879887135
215857_at	NCLN	-0,49284695	-1,372734086	0,879887135
234908_s_at	DSCAML1	-0,49284695	-1,372734086	0,879887135
222225_at	RPL23AP53	-0,49284695	-1,372734086	0,879887135
1560866_at	WNK2	-0,49284695	-1,372734086	0,879887135
205165_at	CELSR3	0,707657549	-0,172194225	0,879851773
225399_at	TSEN15	4,981149437	4,101453764	0,879695673
205745_x_at	ADAM17	2,878891456	1,999354538	0,879536918
233888_s_at	SRGAP1	0,677745787	-0,201789721	0,879535508
200887_s_at	STAT1	6,595938085	5,716475997	0,879462088
202979_s_at	CREBZF	2,872644433	1,993309863	0,87933457
217544_at	-	0,524545436	-0,354781582	0,879327018
206968_s_at	NFRKB	0,524545436	-0,354781582	0,879327018
206745_at	HOXC11	0,524545436	-0,354781582	0,879327018
1569132_s_at	ARSK	0,524545436	-0,354781582	0,879327018
233339_s_at	ARID1B	0,618277321	-0,260526297	0,878803618
1570257_x_at	-	0,618277321	-0,260526297	0,878803618
231298_at	LOC100507425	0,618277321	-0,260526297	0,878803618
220198_s_at	EIF5A2	0,556428218	-0,322340048	0,878768266
1569294_at	-	0,556428218	-0,322340048	0,878768266
206231_at	KCNN1	0,556428218	-0,322340048	0,878768266
223932_at	-	0,58788177	-0,290694975	0,878576745
219009_at	C14orf93	0,58788177	-0,290694975	0,878576745
202807_s_at	TOM1	0,58788177	-0,290694975	0,878576745
225819_at	TBRG1	1,775035811	0,896834102	0,87820171
225197_at	LOC100505487	2,537731447	1,659532492	0,878198956
212539_at	CHD1L	5,146801687	4,268613406	0,878188281
226468_at	RNF115	3,060716358	2,182711118	0,87800524
219972_s_at	C14orf135	2,312738346	1,434743608	0,877994738
210844_x_at	CTNNA1	3,137734155	2,260052727	0,877681428
231816_s_at	UBE2Q1	0,458905032	-0,418248858	0,87715389
230116_at	KRT8P12	0,458905032	-0,418248858	0,87715389
202317_s_at	UBE4B	3,909506676	3,032393954	0,877112721
209935_at	ATP2C1	3,660947664	2,783936118	0,877011545
222151_s_at	CEP63	1,760255626	0,883374248	0,876881377
228910_at	CD82	2,229377375	1,352622437	0,876754938
212465_at	SETD3	3,934348221	3,05768517	0,876663051
211115_x_at	GEMIN2	3,313347235	2,436714502	0,876632732
234971_x_at	PLCD3	1,182925501	0,306454867	0,876470634
1556239_a_at	-	-1,624955693	-2,501359113	0,87640342
232364_at	LOC100506235	-1,624955693	-2,501359113	0,87640342
1554836_at	USP36	-1,624955693	-2,501359113	0,87640342
1570276_a_at	GATA4	-1,624955693	-2,501359113	0,87640342
239380_at	C5orf27	-1,624955693	-2,501359113	0,87640342
1566222_at	-	-1,624955693	-2,501359113	0,87640342
1565858_at	SNORA71A	-1,624955693	-2,501359113	0,87640342
1568870_at	SLC24A4	-1,624955693	-2,501359113	0,87640342
237839_at	-	-1,624955693	-2,501359113	0,87640342

214345_at	-	-0,180990326	-1,057301851	0,876311525
217377_x_at	NTRK3	-0,180990326	-1,057301851	0,876311525
231508_s_at	-	-0,180990326	-1,057301851	0,876311525
218451_at	CDCP1	-0,180990326	-1,057301851	0,876311525
1552792_at	SOCS4	-0,180990326	-1,057301851	0,876311525
208332_at	LOC100509646 ///	-0,180990326	-1,057301851	0,876311525
244123_at	-	-0,180990326	-1,057301851	0,876311525
206612_at	CACNG1	-0,180990326	-1,057301851	0,876311525
227832_at	MBD6	-0,180990326	-1,057301851	0,876311525
244311_at	-	0,425036312	-0,45121326	0,876249572
1557189_at	DNASE1	0,425036312	-0,45121326	0,876249572
228471_at	ANKRD44	2,920736393	2,044580955	0,876155438
224647_at	CCNY	3,67542249	2,799505781	0,875916709
34689_at	TREX1	3,271563226	2,395667684	0,875895542
217247_at	-	0,39105295	-0,484558493	0,875611444
1566816_at	FGF22	0,39105295	-0,484558493	0,875611444
200008_s_at	GDI2	5,69800487	4,822745519	0,875259351
1773_at	CHURC1-FNTB ///	0,425563601	-0,449518308	0,875081909
35254_at	TRAFD1	2,804216189	1,92927364	0,874942549
1556180_at	LOC729678	2,597647262	1,722747778	0,874899484
229969_at	SEC63	1,160996507	0,286279868	0,87471664
230734_x_at	STRN	3,406503548	2,531843788	0,874659761
231995_at	CAAP1	1,458255986	0,583607503	0,874648483
213028_at	NFRKB	2,042564225	1,167919222	0,874645003
221700_s_at	UBA52	7,39934452	6,524776887	0,874567633
212683_at	SLC25A44	2,031158928	1,156599976	0,874558951
207034_s_at	GLI2	-0,791294935	-1,665791776	0,874496841
221183_at	LOC100507388	-0,791294935	-1,665791776	0,874496841
206733_at	TULP2	-0,791294935	-1,665791776	0,874496841
217018_at	-	-0,791294935	-1,665791776	0,874496841
222676_at	BRI3 /// FLJ30064	-0,791294935	-1,665791776	0,874496841
228246_s_at	SPTBN1	-0,791294935	-1,665791776	0,874496841
1555557_a_at	TNK2	-0,791294935	-1,665791776	0,874496841
232241_at	LOC100272228	-0,791294935	-1,665791776	0,874496841
240931_s_at	-	-0,791294935	-1,665791776	0,874496841
210141_s_at	INHA	-0,791294935	-1,665791776	0,874496841
216491_x_at	IGHM	-0,791294935	-1,665791776	0,874496841
229769_at	TMEM242	-0,791294935	-1,665791776	0,874496841
207101_at	VAMP1	-0,791294935	-1,665791776	0,874496841
214380_at	PRPF31	-0,791294935	-1,665791776	0,874496841
220776_at	KCNJ14	-0,791294935	-1,665791776	0,874496841
224816_at	GET4	-0,791294935	-1,665791776	0,874496841
236636_at	-	-0,791294935	-1,665791776	0,874496841
1560059_at	VPS37C	-0,791294935	-1,665791776	0,874496841
221659_s_at	MYL10	-0,791294935	-1,665791776	0,874496841
204631_at	MYH2 /// MYH4	-1,982324124	-2,856570137	0,874246013
203450_at	CBY1	2,884644843	2,010406245	0,874238598
1558340_at	DIXDC1	0,355751005	-0,518232988	0,873983993
224175_s_at	TRIM34 /// TRIM6	0,355751005	-0,518232988	0,873983993
206814_at	NGF	0,319287178	-0,554696666	0,873983844

213981_at	COMT	0,319287178	-0,554696666	0,873983844
202181_at	KIAA0247	2,678352494	1,804519951	0,873832543
212061_at	U2SURP	2,671492308	1,797682937	0,87380937
218314_s_at	C11orf57	2,582572508	1,709047448	0,87352506
225580_at	MRPL50	2,657308324	1,784182261	0,873126063
210719_s_at	HMG20B	3,843767728	2,971193817	0,87257391
224452_s_at	FAM220A	3,840618883	2,968300712	0,872318171
214835_s_at	SUCLG2	3,694479488	2,822379032	0,872100455
218985_at	SLC2A8	2,41945542	1,547376668	0,872078752
229444_at	LOC100131607	2,943764123	2,071691486	0,872072638
1554065_at	FAM125B	0,282358733	-0,589001171	0,871359904
221113_s_at	WNT16	0,282358733	-0,589001171	0,871359904
226779_at	LMBRD2	3,792209911	2,921154239	0,871055672
244393_x_at	-	-0,551284523	-1,422257026	0,870972504
232326_at	C8orf56	-0,551284523	-1,422257026	0,870972504
242399_at	-	-0,551284523	-1,422257026	0,870972504
217113_at	AMACR	-0,551284523	-1,422257026	0,870972504
231134_at	C20orf79	-0,551284523	-1,422257026	0,870972504
220816_at	LPAR3	-0,551284523	-1,422257026	0,870972504
233435_at	-	-0,551284523	-1,422257026	0,870972504
214572_s_at	INSL3	-0,551284523	-1,422257026	0,870972504
238641_at	C1orf126	-0,551284523	-1,422257026	0,870972504
214294_at	KIAA0485	-0,551284523	-1,422257026	0,870972504
243471_at	-	-0,230432956	-1,100943374	0,870510418
208008_at	TBC1D29	-0,230432956	-1,100943374	0,870510418
221298_s_at	SLC22A8	-0,230432956	-1,100943374	0,870510418
221077_at	ARMC4	-0,230432956	-1,100943374	0,870510418
211541_s_at	DYRK1A	-0,230432956	-1,100943374	0,870510418
231700_at	GUCA1A	-0,230432956	-1,100943374	0,870510418
221785_at	WIZ	-0,230432956	-1,100943374	0,870510418
222692_s_at	FNDC3B	-0,230432956	-1,100943374	0,870510418
211208_s_at	CASK	-0,230432956	-1,100943374	0,870510418
229336_at	ST3GAL2	1,830468611	0,960042218	0,870426393
225489_at	TMEM18	3,562907192	2,693044596	0,869862596
218606_at	ZDHHC7	3,505636113	2,635864822	0,869771291
222228_s_at	ALKBH4	1,047341799	0,178191865	0,869149933
212572_at	STK38L	3,691400566	2,822379032	0,869021534
220642_x_at	GPR89A /// GPR89	3,668220666	2,799505781	0,868714885
204826_at	CCNF	2,285848435	1,417157926	0,868690509
212519_at	UBE2E1	6,758599142	5,889931637	0,868667505
230477_at	LOC100130522	0,20740896	-0,661178575	0,868587535
210783_x_at	CLEC11A	0,20740896	-0,661178575	0,868587535
241964_at	ZNF865	0,20740896	-0,661178575	0,868587535
230558_at	KNDC1	0,20740896	-0,661178575	0,868587535
216938_x_at	DRD2	0,20740896	-0,661178575	0,868587535
234795_at	-	0,20740896	-0,661178575	0,868587535
204482_at	CLDN5	0,20740896	-0,661178575	0,868587535
204173_at	MYL6B	4,913150425	4,044909869	0,868240556
211833_s_at	BAX	3,137734155	2,269637658	0,868096496
235906_at	TAF10	-0,994186487	-1,862269442	0,868082955

220304_s_at	CNGB3	-0,994186487	-1,862269442	0,868082955
223872_at	-	-0,994186487	-1,862269442	0,868082955
220519_s_at	LIM2	-0,994186487	-1,862269442	0,868082955
239967_at	-	-0,994186487	-1,862269442	0,868082955
205925_s_at	RAB3B	-0,994186487	-1,862269442	0,868082955
1554401_a_at	TCTE3	-0,994186487	-1,862269442	0,868082955
207778_at	REG1P	-0,994186487	-1,862269442	0,868082955
1565132_at	RBMY3AP	-0,994186487	-1,862269442	0,868082955
231429_at	TRABD2A	-0,994186487	-1,862269442	0,868082955
215839_at	LILRA5	-0,994186487	-1,862269442	0,868082955
210340_s_at	CSF2RA	-0,994186487	-1,862269442	0,868082955
222973_at	-	-0,994186487	-1,862269442	0,868082955
221429_x_at	TEX13A	-0,994186487	-1,862269442	0,868082955
215052_at	FRMPD4	-0,994186487	-1,862269442	0,868082955
1556113_at	DKFZp451A211	-0,994186487	-1,862269442	0,868082955
231226_at	-	-0,994186487	-1,862269442	0,868082955
244238_at	-	-0,994186487	-1,862269442	0,868082955
208476_s_at	FRMD4A	-0,994186487	-1,862269442	0,868082955
244824_at	-	-0,994186487	-1,862269442	0,868082955
214993_at	ASPHD1	-0,994186487	-1,862269442	0,868082955
232579_at	LOC100134229	-0,994186487	-1,862269442	0,868082955
239683_at	CLYBL	0,244681185	-0,623254098	0,867935284
237378_at	FAM75D1 /// FAM	0,244681185	-0,623254098	0,867935284
211096_at	PBX2	0,244681185	-0,623254098	0,867935284
1555724_s_at	TAGLN	0,244681185	-0,623254098	0,867935284
219617_at	CAMKMT	2,107136847	1,239268196	0,867868651
1553417_at	C11orf44	-1,137208348	-2,005029581	0,867821233
228602_at	SGCD	-1,137208348	-2,005029581	0,867821233
1570171_at	-	-1,137208348	-2,005029581	0,867821233
221386_at	OR3A2	-1,137208348	-2,005029581	0,867821233
1554715_at	LINC00593	-1,137208348	-2,005029581	0,867821233
223987_at	CHRDL2	-1,137208348	-2,005029581	0,867821233
1553998_at	DMRTC1 /// DMRT	-1,137208348	-2,005029581	0,867821233
232512_at	-	-1,137208348	-2,005029581	0,867821233
236498_s_at	C1orf86	-1,137208348	-2,005029581	0,867821233
231337_at	DKFZp686O1327 /,	-1,137208348	-2,005029581	0,867821233
231587_at	APOC3	-1,137208348	-2,005029581	0,867821233
231415_at	WBSCR22	-1,137208348	-2,005029581	0,867821233
1559061_at	CACNA1G-AS1	-1,137208348	-2,005029581	0,867821233
214037_s_at	CCDC22	1,000459215	0,133104519	0,867354696
203906_at	IQSEC1	2,21987062	1,352622437	0,867248183
1555154_a_at	QKI	0,97663406	0,109458907	0,867175153
222779_s_at	C17orf85	2,374261973	1,508107415	0,866154558
210017_at	MALT1	2,374261973	1,508107415	0,866154558
227636_at	THAP5	3,175897496	2,309925448	0,865972048
226881_at	GRPEL2	3,697863752	2,832036229	0,865827523
209623_at	MCCC2	5,103011831	4,237549912	0,865461918
232394_at	-	0,167727503	-0,697182596	0,8649101
231983_at	IBA57	0,167727503	-0,697182596	0,8649101
200811_at	CIRBP	2,824160383	1,9592579	0,864902483

232995_at	-	-0,280273599	-1,145057014	0,864783415
240323_at	-	-0,280273599	-1,145057014	0,864783415
207364_at	TEX28	-0,280273599	-1,145057014	0,864783415
1561039_a_at	ZNF81	-0,280273599	-1,145057014	0,864783415
1552807_a_at	SIGLEC10	-0,280273599	-1,145057014	0,864783415
207172_s_at	CDH11	-0,280273599	-1,145057014	0,864783415
227336_at	DTX1	-0,280273599	-1,145057014	0,864783415
204878_s_at	TAOK2	0,926325262	0,061773582	0,86455168
222410_s_at	SNX6	4,552867541	3,689333494	0,863534048
218586_at	C20orf20	1,760255626	0,896834102	0,863421524
213061_s_at	NTAN1	3,723597116	2,860202326	0,86339479
236075_s_at	LOC100506676	4,237642543	3,374450349	0,863192194
223084_s_at	CCNDBP1	4,508502183	3,645557465	0,862944717
1569644_at	-	-0,609040214	-1,471833228	0,862793014
212526_at	SPG20	-0,609040214	-1,471833228	0,862793014
1557099_at	-	-0,609040214	-1,471833228	0,862793014
229247_at	FBLN7	-0,609040214	-1,471833228	0,862793014
207530_s_at	CDKN2B	-0,609040214	-1,471833228	0,862793014
220226_at	TRPM8	-0,609040214	-1,471833228	0,862793014
203995_at	C21orf2	-0,609040214	-1,471833228	0,862793014
1564697_a_at	LOC400752	-0,609040214	-1,471833228	0,862793014
241476_at	-	-0,609040214	-1,471833228	0,862793014
244630_at	PDPK1	-0,609040214	-1,471833228	0,862793014
202223_at	STT3A	4,27728562	3,414783177	0,862502443
236718_at	MYO10	-1,802716385	-2,665156778	0,862440393
1557432_at	RASAL2	-1,802716385	-2,665156778	0,862440393
240059_at	-	-1,802716385	-2,665156778	0,862440393
1556320_at	STOML1	0,900065578	0,03784737	0,862218208
226948_at	RHBDD1	0,900065578	0,03784737	0,862218208
1558604_a_at	SSBP2	0,127732831	-0,734355396	0,862088227
1558208_at	-	0,127732831	-0,734355396	0,862088227
201045_s_at	RAB6A	0,127732831	-0,734355396	0,862088227
213070_at	PIK3C2A	2,983826368	2,121960601	0,861865767
218344_s_at	RCOR3	2,149438457	1,28853674	0,860901717
221954_at	C20orf111	2,304041045	1,44320939	0,860831656
215015_at	CCDC64	0,873789652	0,013190398	0,860599254
236053_at	LOC100128653	0,873789652	0,013190398	0,860599254
202074_s_at	OPTN	2,392346173	1,531877749	0,860468424
225228_at	DRAM2	2,771643417	1,911502437	0,860140981
215780_s_at	SET /// SETP4	5,514518512	4,654386349	0,860132163
215627_at	-	-1,369772723	-2,229233437	0,859460715
221910_at	ETV1	-1,369772723	-2,229233437	0,859460715
1556558_s_at	ENTPD3-AS1	-1,369772723	-2,229233437	0,859460715
236444_x_at	-	-1,369772723	-2,229233437	0,859460715
1559514_at	LOC100132077	-1,369772723	-2,229233437	0,859460715
1560011_at	JRK	-1,369772723	-2,229233437	0,859460715
239972_at	-	-1,369772723	-2,229233437	0,859460715
213862_at	-	-1,369772723	-2,229233437	0,859460715
240412_s_at	HEATR7B2	-1,369772723	-2,229233437	0,859460715
240611_at	-	-1,369772723	-2,229233437	0,859460715

224539_s_at	PCDHAC2	-1,369772723	-2,229233437	0,859460715
203813_s_at	SLIT3	-1,369772723	-2,229233437	0,859460715
209975_at	CYP2E1	-1,369772723	-2,229233437	0,859460715
1561036_at	-	-1,369772723	-2,229233437	0,859460715
1556250_at	-	-1,369772723	-2,229233437	0,859460715
208430_s_at	DTNA	-1,369772723	-2,229233437	0,859460715
234879_at	-	-1,369772723	-2,229233437	0,859460715
210381_s_at	CCKBR	-1,369772723	-2,229233437	0,859460715
212256_at	GALNT10	1,716003175	0,85655369	0,859449485
1554725_at	RABGAP1L	-1,452713826	-2,312054429	0,859340603
242592_at	GPR137C	-1,452713826	-2,312054429	0,859340603
204926_at	INHBA	-1,452713826	-2,312054429	0,859340603
236015_at	-	-1,452713826	-2,312054429	0,859340603
1570433_at	TMPRSS2	-1,452713826	-2,312054429	0,859340603
210000_s_at	SOCS1	-1,452713826	-2,312054429	0,859340603
231511_at	FRAS1	-1,452713826	-2,312054429	0,859340603
224348_s_at	-	-1,452713826	-2,312054429	0,859340603
1556437_at	LOC253805	-1,452713826	-2,312054429	0,859340603
1564504_at	ASIC5	-1,452713826	-2,312054429	0,859340603
242238_at	-	-1,452713826	-2,312054429	0,859340603
1562537_at	FCER1A	-1,452713826	-2,312054429	0,859340603
231327_at	-	-1,452713826	-2,312054429	0,859340603
208950_s_at	ALDH7A1	-1,452713826	-2,312054429	0,859340603
238372_s_at	-	-0,856259172	-1,715576125	0,859316953
207818_s_at	HTR7	-0,856259172	-1,715576125	0,859316953
218858_at	DEPTOR	-0,856259172	-1,715576125	0,859316953
1560023_x_at	-	-0,856259172	-1,715576125	0,859316953
205783_at	KLK13	-0,856259172	-1,715576125	0,859316953
230234_at	FXN	-0,856259172	-1,715576125	0,859316953
1558850_s_at	LOC284276	-0,856259172	-1,715576125	0,859316953
207586_at	SHH	-0,856259172	-1,715576125	0,859316953
223597_at	ITLN1	-0,856259172	-1,715576125	0,859316953
208275_x_at	UTF1	-0,856259172	-1,715576125	0,859316953
241488_at	-	-0,856259172	-1,715576125	0,859316953
1561598_at	-	-0,856259172	-1,715576125	0,859316953
223657_at	FAM167B	-0,856259172	-1,715576125	0,859316953
234156_at	-	-0,856259172	-1,715576125	0,859316953
210885_s_at	TRIM15	-0,856259172	-1,715576125	0,859316953
225188_at	RAPH1	-0,856259172	-1,715576125	0,859316953
244291_x_at	-	-1,893506789	-2,752097204	0,858590415
1560107_at	-	-1,893506789	-2,752097204	0,858590415
1561616_a_at	DNAH6	0,847529938	-0,011001236	0,858531173
1569838_at	-	-0,332405896	-1,19085694	0,858451044
205577_at	PYGM	-0,332405896	-1,19085694	0,858451044
220959_s_at	OBP2A /// OBP2B	-0,332405896	-1,19085694	0,858451044
211607_x_at	EGFR	-0,332405896	-1,19085694	0,858451044
228848_at	ABTB1	-0,332405896	-1,19085694	0,858451044
243661_at	ZNF273	-0,332405896	-1,19085694	0,858451044
235755_at	TNRC18	-0,332405896	-1,19085694	0,858451044
230338_x_at	GSX2	-0,332405896	-1,19085694	0,858451044

231584_s_at	BCAS4	-0,332405896	-1,19085694	0,858451044
217071_s_at	MTHFR	-0,332405896	-1,19085694	0,858451044
208414_s_at	HOXB3	-0,332405896	-1,19085694	0,858451044
244317_at	KIAA1324L	-0,332405896	-1,19085694	0,858451044
226010_at	SLC25A23	2,745538988	1,887158627	0,858380361
219037_at	RRP15	4,135744086	3,277597597	0,858146489
243155_at	-	-1,712732543	-2,570522741	0,857790199
215943_at	KIAA1661	-1,712732543	-2,570522741	0,857790199
241914_s_at	ACSM2A /// ACSM	-1,712732543	-2,570522741	0,857790199
216769_x_at	-	-1,712732543	-2,570522741	0,857790199
1561387_a_at	NXPE1	-1,712732543	-2,570522741	0,857790199
211909_x_at	PTGER3	-1,712732543	-2,570522741	0,857790199
230215_at	SEC61A2	0,08619576	-0,771340337	0,857536097
221000_s_at	KAZALD1	0,08619576	-0,771340337	0,857536097
220501_at	ACTL7A	0,08619576	-0,771340337	0,857536097
231320_at	EBP	0,08619576	-0,771340337	0,857536097
224065_at	HIPK2	0,08619576	-0,771340337	0,857536097
218421_at	CERK	3,732463367	2,874932245	0,857531122
239105_at	-	0,045517965	-0,811769547	0,857287511
205986_at	AATK	0,045517965	-0,811769547	0,857287511
213833_x_at	NOP16	0,045517965	-0,811769547	0,857287511
216889_s_at	HNF4A	0,045517965	-0,811769547	0,857287511
200822_x_at	TPI1	6,906473044	6,049315624	0,85715742
224635_s_at	BIRC6	4,05770789	3,20060887	0,85709902
212431_at	HMGXB3	2,365121417	1,508107415	0,857014002
219157_at	KLHL2	3,039689025	2,182711118	0,856977907
222804_x_at	DCAF10	2,21987062	1,362900801	0,856969819
209252_at	HARS2	3,701002444	2,844606929	0,856395515
202060_at	CTR9	4,538810963	3,682566532	0,856244432
219548_at	ZNF16	1,671397663	0,815200271	0,856197392
201063_at	RCN1	3,012642182	2,157148588	0,855493594
1560445_x_at	ARHGEF1	0,791908897	-0,063377083	0,85528598
41657_at	STK11	1,601024957	0,746163302	0,854861655
41329_at	SCYL3	3,471313383	2,61649873	0,854814653
223594_at	TMEM117	1,160996507	0,306454867	0,85454164
226607_at	C20orf194	1,65554524	0,80114751	0,85439773
226584_s_at	FAM110A	1,65554524	0,80114751	0,85439773
217917_s_at	DYNLRB1	5,603415323	4,749086149	0,854329174
224890_s_at	C7orf59	4,033407967	3,179283207	0,854124759
241252_at	ESCO2	-1,537851782	-2,391863529	0,854011747
220752_at	LOC51145	-1,537851782	-2,391863529	0,854011747
222247_at	-	-1,537851782	-2,391863529	0,854011747
232119_at	SYNPO2	-1,537851782	-2,391863529	0,854011747
236944_at	-	-1,537851782	-2,391863529	0,854011747
232850_at	-	-1,537851782	-2,391863529	0,854011747
238212_at	-	-1,537851782	-2,391863529	0,854011747
221358_at	NPBWR2	-1,537851782	-2,391863529	0,854011747
1553558_at	TAS2R41	-1,537851782	-2,391863529	0,854011747
1566900_at	-	-1,537851782	-2,391863529	0,854011747
233555_s_at	SULF2	-1,537851782	-2,391863529	0,854011747

234019_at	-	-1,537851782	-2,391863529	0,854011747
205850_s_at	GABRB3	-1,537851782	-2,391863529	0,854011747
213371_at	LDB3	-1,537851782	-2,391863529	0,854011747
210805_x_at	LOC100506403 ///	-1,537851782	-2,391863529	0,854011747
241535_at	LOC100292909	-1,537851782	-2,391863529	0,854011747
241422_at	-	-1,537851782	-2,391863529	0,854011747
220476_s_at	FAM212B	0,764143511	-0,089635713	0,853779224
235920_at	-	0,764143511	-0,089635713	0,853779224
213243_at	VPS13B	3,331722254	2,478123977	0,853598277
200891_s_at	SSR1	6,073926283	5,2204235	0,853502783
204001_at	SNAPC3	2,582572508	1,72935563	0,853216878
213763_at	HIPK2	1,139057614	0,286279868	0,852777747
1558164_s_at	PEX13	0,735999505	-0,116767475	0,85276698
235428_at	LOC100507316	1,625482993	0,772793615	0,852689379
227964_at	FRMD8	4,446069945	3,593410766	0,852659179
202291_s_at	MGP	-1,289805289	-2,142286822	0,852481533
221438_s_at	TEX12	-1,289805289	-2,142286822	0,852481533
209540_at	IGF1	-1,289805289	-2,142286822	0,852481533
220328_at	PHC3	-1,289805289	-2,142286822	0,852481533
1561185_at	TTTY7	-1,289805289	-2,142286822	0,852481533
206465_at	ACSBG1	-1,289805289	-2,142286822	0,852481533
220378_at	TCP11	-1,289805289	-2,142286822	0,852481533
1560724_at	-	-1,289805289	-2,142286822	0,852481533
1555586_at	FAM71B	-1,289805289	-2,142286822	0,852481533
217206_at	-	-1,289805289	-2,142286822	0,852481533
214147_at	HEATR8	-1,289805289	-2,142286822	0,852481533
235049_at	ADCY1	-1,289805289	-2,142286822	0,852481533
207440_at	SLC35A2	-1,289805289	-2,142286822	0,852481533
225763_at	RCSD1	3,837502555	2,98507484	0,852427715
244786_at	SNHG10	0,707657549	-0,144765583	0,852423132
203380_x_at	SRSF5	5,215904344	4,363697406	0,852206938
217906_at	KLHDC2	3,951538845	3,099629381	0,851909464
202930_s_at	SUCLA2	3,867546929	3,01591662	0,851630308
203885_at	RAB21	4,19821572	3,346715127	0,851500592
209680_s_at	KIFC1	3,537317882	2,686001625	0,851316257
204436_at	PLEKHO2	2,285848435	1,434743608	0,851104827
208783_s_at	CD46	5,604295373	4,753282184	0,851013189
211319_at	RAE1	-0,66934516	-1,520022123	0,850676964
1563862_at	TCEANC	-0,66934516	-1,520022123	0,850676964
236626_at	ALG1	-0,66934516	-1,520022123	0,850676964
220907_at	GPR110	-0,66934516	-1,520022123	0,850676964
220245_at	SLC45A2	-0,66934516	-1,520022123	0,850676964
205759_s_at	SULT2B1	-0,66934516	-1,520022123	0,850676964
1556960_a_at	ANHX	-0,66934516	-1,520022123	0,850676964
220555_s_at	PDZD7	-0,66934516	-1,520022123	0,850676964
236658_at	-	-0,66934516	-1,520022123	0,850676964
1554809_at	LOC389199	-0,66934516	-1,520022123	0,850676964
238814_at	SLC35C2	-0,66934516	-1,520022123	0,850676964
1562487_at	-	-0,66934516	-1,520022123	0,850676964
1562232_at	-	-0,66934516	-1,520022123	0,850676964

203290_at	HLA-DQA1	-0,66934516	-1,520022123	0,850676964
239779_at	-	-0,66934516	-1,520022123	0,850676964
213990_s_at	PAK7	-0,66934516	-1,520022123	0,850676964
213630_at	NACAD	-0,384193355	-1,234835326	0,850641971
227652_at	FAM69B	-0,384193355	-1,234835326	0,850641971
1554907_a_at	HYDIN /// HYDIN2	-0,384193355	-1,234835326	0,850641971
236898_at	-	-0,384193355	-1,234835326	0,850641971
230888_at	WDR91	-0,384193355	-1,234835326	0,850641971
216485_s_at	TPSAB1	-0,384193355	-1,234835326	0,850641971
230254_at	FAM26E	-0,384193355	-1,234835326	0,850641971
230855_at	GATA4	-0,384193355	-1,234835326	0,850641971
226522_at	PODN	0,001114523	-0,84928999	0,850404513
1553425_at	WDR65	0,001114523	-0,84928999	0,850404513
218013_x_at	DCTN4	2,705643918	1,855279162	0,850364756
223724_s_at	STAG3L1 /// STAG:	0,677745787	-0,172194225	0,849940012
222536_s_at	ZNF395	0,618277321	-0,231045907	0,849323228
209451_at	TANK	4,346015214	3,496833915	0,849181299
213658_at	-	1,071336699	0,222432814	0,848903885
1559795_at	-	1,071336699	0,222432814	0,848903885
231086_at	-	1,071336699	0,222432814	0,848903885
203992_s_at	KDM6A	2,427650693	1,578991006	0,848659688
228330_at	ZUFSP	2,836219929	1,987810614	0,848409315
206501_x_at	ETV1	0,58788177	-0,260526297	0,848408067
237429_at	-	-1,06282519	-1,911206569	0,84838138
207597_at	ADAM18	-1,06282519	-1,911206569	0,84838138
239326_at	-	-1,06282519	-1,911206569	0,84838138
1562907_at	-	-1,06282519	-1,911206569	0,84838138
1554872_a_at	HMGCLL1	-1,06282519	-1,911206569	0,84838138
242917_at	RASGEF1A	-1,06282519	-1,911206569	0,84838138
1555157_at	-	-1,06282519	-1,911206569	0,84838138
243622_at	LOC145694	-1,06282519	-1,911206569	0,84838138
210727_at	CALCA	-1,06282519	-1,911206569	0,84838138
233197_at	KLHL9	-1,06282519	-1,911206569	0,84838138
222829_s_at	IL20RA	-1,06282519	-1,911206569	0,84838138
231971_at	FANCM	-1,06282519	-1,911206569	0,84838138
224306_at	NPC1L1	-1,06282519	-1,911206569	0,84838138
1552965_a_at	B3GNT7	-1,06282519	-1,911206569	0,84838138
231089_at	LOC100505664	-1,06282519	-1,911206569	0,84838138
237026_at	-	-1,06282519	-1,911206569	0,84838138
220683_at	RDH8	-1,06282519	-1,911206569	0,84838138
1555748_x_at	CD79B	-1,06282519	-1,911206569	0,84838138
236052_at	TRNP1	-1,06282519	-1,911206569	0,84838138
236556_s_at	LONRF1	-1,06282519	-1,911206569	0,84838138
1557879_at	LOC100129175	-1,06282519	-1,911206569	0,84838138
234024_at	CBLN4	-1,06282519	-1,911206569	0,84838138
243202_at	-	-1,06282519	-1,911206569	0,84838138
214283_at	TMEM97	2,829929672	1,981728801	0,848200871
224645_at	EIF4EBP2	1,544940605	0,697221375	0,84771923
218953_s_at	PCYOX1L	3,166767593	2,31904917	0,847718424
212495_at	KDM4B	1,730929079	0,883374248	0,84755483

213628_at	CLCC1	2,75889404	1,911502437	0,847391603
219202_at	RHBDF2	1,326463531	0,479089184	0,847374347
214703_s_at	MAN2B2	2,908690075	2,06133574	0,847354335
59999_at	HIF1AN	1,633451527	0,786102808	0,84734872
208671_at	SERINC1	4,849052846	4,001778634	0,847274212
208991_at	STAT3	5,082707435	4,235529701	0,847177734
210373_at	NPRL2	0,556428218	-0,290694975	0,847123193
203791_at	DMXL1	3,370643409	2,523551252	0,847092157
219362_at	NAA35	2,209870035	1,362900801	0,846969234
238055_at	LOC100505549	0,524545436	-0,322340048	0,846885484
203421_at	TP53I11	0,524545436	-0,322340048	0,846885484
212134_at	PHLDB1	0,524545436	-0,322340048	0,846885484
227201_at	LOC643837	0,491906512	-0,354781582	0,846688093
235418_at	FAHD2A	0,491906512	-0,354781582	0,846688093
1557710_at	-	0,491906512	-0,354781582	0,846688093
201000_at	AARS	4,891443931	4,044909869	0,846534062
235890_at	TBL1XR1	1,024284941	0,178191865	0,846093076
200758_s_at	NFE2L1	3,599444854	2,753767314	0,845677539
214377_s_at	CTRL	-0,043925712	-0,889222211	0,845296499
208124_s_at	SEMA4F	-0,043925712	-0,889222211	0,845296499
211233_x_at	ESR1	-0,043925712	-0,889222211	0,845296499
225778_at	RBMS2	-0,043925712	-0,889222211	0,845296499
219054_at	NPR3	-0,043925712	-0,889222211	0,845296499
234753_x_at	-	-0,043925712	-0,889222211	0,845296499
213767_at	KSR1	-0,043925712	-0,889222211	0,845296499
234355_s_at	PTCHD2	-0,043925712	-0,889222211	0,845296499
218110_at	XAB2	-0,043925712	-0,889222211	0,845296499
229749_at	ANO4	-0,437449947	-1,282520722	0,845070775
222878_s_at	OTUB2	-0,437449947	-1,282520722	0,845070775
229643_at	LOC100287375	-0,437449947	-1,282520722	0,845070775
228072_at	SYT12	-0,437449947	-1,282520722	0,845070775
215925_s_at	CD72	-0,437449947	-1,282520722	0,845070775
1569245_at	C8orf74	-0,437449947	-1,282520722	0,845070775
230390_at	-	-0,437449947	-1,282520722	0,845070775
242683_at	LOC400643	-0,437449947	-1,282520722	0,845070775
232733_s_at	COL20A1	-0,437449947	-1,282520722	0,845070775
1558530_at	LRTM2	-0,437449947	-1,282520722	0,845070775
1563243_at	-	-0,437449947	-1,282520722	0,845070775
234290_x_at	MYH14	-0,437449947	-1,282520722	0,845070775
225281_at	C3orf17	4,747567561	3,902746728	0,844820833
204683_at	ICAM2	3,065977723	2,221565736	0,844411987
216381_x_at	AKR7A3	1,686984721	0,842599219	0,844385501
213509_x_at	CES2	2,849132494	2,004917973	0,844214522
225749_at	C16orf91	2,698955954	1,855279162	0,843676793
1569345_at	-	0,425036312	-0,418248858	0,84328517
1556173_a_at	-	1,910557155	1,067365565	0,84319159
232545_at	LRRC29	-0,49284695	-1,33598831	0,84314136
205433_at	BCHE	4,319579883	3,47651952	0,843060363
218139_s_at	AP5M1	3,954683435	3,111774082	0,842909353
221589_s_at	ALDH6A1	3,362258367	2,519368159	0,842890209

209341_s_at	IKBKB	3,394274216	2,551398837	0,84287538
1552652_at	HPS4	0,95222556	0,109458907	0,842766654
202608_s_at	NDST1	-0,088974936	-0,931539147	0,842564211
216789_at	-	-0,088974936	-0,931539147	0,842564211
216656_at	-	-0,088974936	-0,931539147	0,842564211
229544_at	-	2,685337919	1,842852096	0,842485823
1560292_a_at	TMCO4	0,39105295	-0,45121326	0,84226621
227106_at	TMEM198B	0,39105295	-0,45121326	0,84226621
226317_at	PPP4R2	3,152091004	2,309925448	0,842165556
203468_at	CDK10	1,476175815	0,634301128	0,841874687
208415_x_at	ING1	4,128096493	3,28651066	0,841585833
220277_at	CXXC4	2,427650693	1,586121802	0,841528892
237525_at	-	-1,624955693	-2,466349414	0,841393721
235439_at	RBMS2	-1,624955693	-2,466349414	0,841393721
222535_at	INF2	-1,624955693	-2,466349414	0,841393721
237732_at	PRR9	-1,624955693	-2,466349414	0,841393721
231307_at	-	-1,624955693	-2,466349414	0,841393721
216227_at	-	-1,624955693	-2,466349414	0,841393721
216541_x_at	IGHG1 /// IGHM	-1,624955693	-2,466349414	0,841393721
206815_at	SPAG8	-1,624955693	-2,466349414	0,841393721
211621_at	AR	-1,624955693	-2,466349414	0,841393721
236773_at	-	-1,624955693	-2,466349414	0,841393721
211430_s_at	IGHG1 /// IGHG2 /	-1,624955693	-2,466349414	0,841393721
206979_at	C8B	-1,624955693	-2,466349414	0,841393721
233002_at	PPP4R4	-1,624955693	-2,466349414	0,841393721
219206_x_at	TMBIM4	5,050523265	4,209377651	0,841145614
227100_at	B3GALTL	0,926325262	0,085534992	0,84079027
223269_at	POLR3GL	4,512206259	3,671698545	0,840507714
205583_s_at	ALG13	3,209439373	2,36899666	0,840442713
229297_at	C14orf118	2,25755351	1,417157926	0,840395584
235292_at	FLJ32255	0,355751005	-0,484558493	0,840309498
202438_x_at	IDS	0,355751005	-0,484558493	0,840309498
214516_at	HIST1H4A /// HIST	-0,924000698	-1,763664074	0,839663376
202686_s_at	AXL	-0,924000698	-1,763664074	0,839663376
1558277_at	ZNF740	-0,924000698	-1,763664074	0,839663376
244706_at	PCMTD1	-0,924000698	-1,763664074	0,839663376
1562390_at	-	-0,924000698	-1,763664074	0,839663376
234718_at	ANO2	-0,924000698	-1,763664074	0,839663376
1553237_x_at	PCDHAC1	-0,924000698	-1,763664074	0,839663376
208509_s_at	OR7A17	-0,924000698	-1,763664074	0,839663376
1570568_at	LOC100130502	-0,924000698	-1,763664074	0,839663376
219776_s_at	-	-0,924000698	-1,763664074	0,839663376
216077_s_at	L3MBTL1	-0,924000698	-1,763664074	0,839663376
1555336_a_at	ITGA9	-0,924000698	-1,763664074	0,839663376
231322_at	-	-0,924000698	-1,763664074	0,839663376
217314_at	-	-0,924000698	-1,763664074	0,839663376
244711_at	-	-0,924000698	-1,763664074	0,839663376
203409_at	DDB2	3,296548503	2,456960019	0,839588483
217549_at	-	3,007119335	2,167793428	0,839325908
226370_at	KLHL15	2,21987062	1,380552339	0,839318281

225601_at	HMGB3	2,229377375	1,390070295	0,83930708
203977_at	TAZ	1,640193838	0,80114751	0,839046328
218439_s_at	COMMD10	3,632502157	2,793634788	0,838867369
225549_at	DDX6	4,716606434	3,877875398	0,838731036
225457_s_at	LINC00263 /// PP7	2,56794744	1,72935563	0,83859181
201653_at	CNIH	5,489321938	4,650762375	0,838559563
225761_at	PAPD4	4,077956772	3,239448352	0,83850842
239034_at	CXorf24	-0,730013898	-1,568385657	0,838371758
202198_s_at	MTMR3	-0,730013898	-1,568385657	0,838371758
232895_s_at	-	-0,730013898	-1,568385657	0,838371758
1553946_at	DCD	-0,730013898	-1,568385657	0,838371758
226057_at	ARHGAP31	-0,730013898	-1,568385657	0,838371758
220981_x_at	NXF2 /// NXF2B	-0,730013898	-1,568385657	0,838371758
1553527_at	NLRP9	-0,730013898	-1,568385657	0,838371758
240328_at	-	-0,730013898	-1,568385657	0,838371758
219173_at	MYO15B	-0,730013898	-1,568385657	0,838371758
235011_at	MAP3K2	-0,730013898	-1,568385657	0,838371758
233069_at	PPP4R1L	-0,730013898	-1,568385657	0,838371758
204326_x_at	MT1X	4,466417603	3,628062877	0,838354726
205201_at	GLI3	0,900065578	0,061773582	0,838291996
217943_s_at	MAP7D1	3,166767593	2,328492509	0,838275084
1561572_at	-	-0,135065865	-0,972893339	0,837827474
1560348_at	RGNEF	-0,135065865	-0,972893339	0,837827474
228111_s_at	DNAH1	-0,135065865	-0,972893339	0,837827474
234133_s_at	LOC728543	-0,135065865	-0,972893339	0,837827474
212209_at	MED13L	3,541158018	2,703479652	0,837678365
209349_at	RAD50	1,610353504	0,772793615	0,837559889
221813_at	FBXO42	2,460890032	1,623485524	0,837404509
212971_at	CARS	6,128821297	5,291608124	0,837213173
226414_s_at	ANAPC11	5,922262115	5,085052728	0,837209387
1559270_at	ZFHX4	-1,212652659	-2,049747196	0,837094537
228484_s_at	FOXO1	-1,212652659	-2,049747196	0,837094537
243831_at	-	-1,212652659	-2,049747196	0,837094537
1559586_at	LOC728868	-1,212652659	-2,049747196	0,837094537
221349_at	VPREB1	-1,212652659	-2,049747196	0,837094537
220723_s_at	CWH43	-1,212652659	-2,049747196	0,837094537
209582_s_at	CD200	-1,212652659	-2,049747196	0,837094537
207342_at	CNGB1	-1,212652659	-2,049747196	0,837094537
239509_at	FLJ16779	-1,212652659	-2,049747196	0,837094537
243199_at	LOC100505574	-1,212652659	-2,049747196	0,837094537
217429_at	-	-1,212652659	-2,049747196	0,837094537
236135_at	PNPLA7	-1,212652659	-2,049747196	0,837094537
1569602_at	KANK2	-1,212652659	-2,049747196	0,837094537
244449_at	LOC100506021	-1,212652659	-2,049747196	0,837094537
207432_at	BEST2	-1,212652659	-2,049747196	0,837094537
233281_at	-	-1,212652659	-2,049747196	0,837094537
242441_at	-	-1,212652659	-2,049747196	0,837094537
203562_at	FEZ1	-1,212652659	-2,049747196	0,837094537
239230_at	HES5	-1,212652659	-2,049747196	0,837094537
239011_at	PRKCE	-1,212652659	-2,049747196	0,837094537

211589_at	PML	-1,212652659	-2,049747196	0,837094537
1553082_at	CRYGN	-1,212652659	-2,049747196	0,837094537
210881_s_at	IGF2 /// INS-IGF2	-1,212652659	-2,049747196	0,837094537
1552391_at	C1orf65	-1,212652659	-2,049747196	0,837094537
219942_at	MYL7	-1,212652659	-2,049747196	0,837094537
212226_s_at	PPAP2B	-1,212652659	-2,049747196	0,837094537
207502_at	GUCA2B	-1,212652659	-2,049747196	0,837094537
220804_s_at	TP73	-1,212652659	-2,049747196	0,837094537
1554400_at	TCTE3	-1,212652659	-2,049747196	0,837094537
222670_s_at	MAFB	0,282358733	-0,554696666	0,837055399
218417_s_at	SLC48A1	0,282358733	-0,554696666	0,837055399
226161_at	SLC30A6	3,331722254	2,49476365	0,836958604
224582_s_at	NUCKS1	2,983826368	2,147194726	0,836631641
219982_s_at	SERF1A /// SERF1B	2,085817135	1,249194639	0,836622496
227974_at	-	1,182925501	0,346964736	0,835960765
207511_s_at	CNPPD1	0,873789652	0,03784737	0,835942282
230046_at	-	0,873789652	0,03784737	0,835942282
1557966_x_at	MTERFD2	2,537731447	1,701800759	0,835930688
218434_s_at	AACS	3,489810047	2,654387125	0,835422922
227114_at	RNF214	1,745502609	0,910483921	0,835018688
217866_at	CPSF7	3,624986574	2,790111983	0,834874591
214692_s_at	JRK	1,561816652	0,727123109	0,834693544
212313_at	CHMP7	2,804738618	1,970106792	0,834631825
225633_at	DPY19L3	2,51558601	1,680963921	0,834622089
222316_at	-	-0,49284695	-1,32740143	0,83455448
226418_at	ERGIC2	-0,49284695	-1,32740143	0,83455448
235590_at	FAM178A	-0,49284695	-1,32740143	0,83455448
237053_at	SYNDIG1L	-0,49284695	-1,32740143	0,83455448
232869_at	SRGAP3	-0,49284695	-1,32740143	0,83455448
229820_at	LOC440993	-0,49284695	-1,32740143	0,83455448
1560456_at	PLIN5	-0,49284695	-1,32740143	0,83455448
211699_x_at	HBA1 /// HBA2	-0,49284695	-1,32740143	0,83455448
220808_at	THEG	-0,49284695	-1,32740143	0,83455448
217341_at	DNM1	-0,49284695	-1,32740143	0,83455448
214964_at	-	-0,49284695	-1,32740143	0,83455448
204086_at	PRAME	-0,49284695	-1,32740143	0,83455448
204939_s_at	PLN	-0,180990326	-1,015398016	0,83440769
237286_at	INTS9	-0,180990326	-1,015398016	0,83440769
1555742_at	ERVH-6	-0,180990326	-1,015398016	0,83440769
201907_x_at	DVL3	-0,180990326	-1,015398016	0,83440769
201179_s_at	GNAI3	3,975619308	3,141266133	0,834353175
1565595_at	-	0,847529938	0,013190398	0,83433954
209645_s_at	ALDH1B1	0,847529938	0,013190398	0,83433954
200745_s_at	GNB1	5,80062869	4,966480369	0,834148322
205875_s_at	TREX1	2,507817181	1,674071917	0,833745265
220134_x_at	FAM176B	0,244681185	-0,589001171	0,833682357
230161_at	-	0,244681185	-0,589001171	0,833682357
1554173_at	CD300LB	0,244681185	-0,589001171	0,833682357
221633_at	NCAPH2	0,244681185	-0,589001171	0,833682357
201134_x_at	COX7C	7,506010368	6,672330446	0,833679922

218017_s_at	HGSNAT	2,276736537	1,44320939	0,833527147
235472_at	FUT10	1,383446753	0,550017041	0,833429712
225781_at	MAPK9	2,356619952	1,523429058	0,833190893
227778_at	HEATR7A /// LOC1	1,816938997	0,98381571	0,833123287
1558595_at	-	-1,802716385	-2,635549085	0,8328327
236656_s_at	LOC100288911	-1,802716385	-2,635549085	0,8328327
205952_at	KCNK3	-1,802716385	-2,635549085	0,8328327
240460_at	-	-1,802716385	-2,635549085	0,8328327
214854_at	-	-1,802716385	-2,635549085	0,8328327
222950_at	NIPAL2	-1,802716385	-2,635549085	0,8328327
240804_at	-	-1,802716385	-2,635549085	0,8328327
222575_at	SETD5	1,716003175	0,883374248	0,832628926
218426_s_at	RNF216	1,92317941	1,090640407	0,832539004
211052_s_at	TBCD	2,725848647	1,893444329	0,832404318
213039_at	ARHGEF18	4,142807036	3,31062689	0,832180145
212433_x_at	RPS2 /// SNORA64	8,64416217	7,811995647	0,832166523
220079_s_at	USP48	4,865309532	4,033281307	0,832028225
229167_at	PURA	2,410823487	1,578991006	0,831832481
212696_s_at	RNF4	5,253358403	4,421877167	0,831481236
201423_s_at	CUL4A	3,389999385	2,558652146	0,831347239
200014_s_at	HNRNPC /// LOC10	6,219321649	5,387977657	0,831343992
236125_at	-	0,820160788	-0,011001236	0,831162024
205652_s_at	TTLL1	1,898032047	1,067365565	0,830666482
206706_at	NTF3	0,20740896	-0,623254098	0,830663059
213621_s_at	GUK1	0,20740896	-0,623254098	0,830663059
234286_at	PPP1R12C	0,20740896	-0,623254098	0,830663059
1566775_at	DNAH1	0,20740896	-0,623254098	0,830663059
1563250_at	-	0,20740896	-0,623254098	0,830663059
200821_at	LAMP2	4,930127442	4,099530192	0,83059725
218476_at	POMT1	1,686984721	0,85655369	0,830431031
228769_at	ZSCAN22	1,116462765	0,286279868	0,830182897
201408_at	PPP1CB	4,535582105	3,70595963	0,829622475
224298_s_at	UBAC2	2,127965541	1,298349073	0,829616468
1565978_a_at	-	-1,893506789	-2,723114724	0,829607935
238269_at	-	-1,893506789	-2,723114724	0,829607935
208793_x_at	SMARCA4	1,510970097	0,681874787	0,82909531
230679_at	DCAF10	0,167727503	-0,661178575	0,828906078
1566105_at	MFN2	0,167727503	-0,661178575	0,828906078
228037_at	-	0,167727503	-0,661178575	0,828906078
1563707_at	-	0,167727503	-0,661178575	0,828906078
238729_x_at	SAV1	0,167727503	-0,661178575	0,828906078
1552613_s_at	CDC42SE2	2,884644843	2,055739171	0,828905672
225174_at	DNAJC10	3,765107151	2,936370826	0,828736325
203683_s_at	VEGFB	1,094118704	0,265475485	0,828643219
200652_at	SSR2	6,151594189	5,323084942	0,828509248
230762_at	-	0,791908897	-0,036543687	0,828452584
213130_at	ZNF473	0,791908897	-0,036543687	0,828452584
217509_x_at	GRIK5	0,791908897	-0,036543687	0,828452584
37254_at	ZNF133	1,493587279	0,665369683	0,828217596
201328_at	ETS2	2,855122714	2,026957145	0,828165569

37966_at	PARVB	3,115390868	2,287641377	0,827749491
226448_at	FAM89A /// MIR11	2,096248267	1,268569037	0,82767923
201014_s_at	PAICS	4,721089228	3,893436439	0,827652789
228070_at	PPP2R5E	1,995527416	1,167919222	0,827608194
232103_at	BPNT1	2,949541692	2,121960601	0,827581091
218519_at	SLC35A5	3,039689025	2,212304392	0,827384632
237057_at	-	-0,230432956	-1,057301851	0,826868895
234439_at	-	-0,230432956	-1,057301851	0,826868895
1552450_a_at	DNAJC5G	-0,230432956	-1,057301851	0,826868895
242882_at	RNF207	-0,230432956	-1,057301851	0,826868895
204874_x_at	BAIAP3	-0,230432956	-1,057301851	0,826868895
203567_s_at	TRIM38	3,537317882	2,710570973	0,826746909
214674_at	USP19	1,071336699	0,244646697	0,826690001
214736_s_at	ADD1	3,89798617	3,071469991	0,826516179
222264_at	HNRNPUL2	2,597647262	1,771157334	0,826489928
222547_at	MAP4K4	1,97249974	1,146010634	0,826489106
232983_s_at	SERGEF	2,590269697	1,76396664	0,826303058
206469_x_at	AKR7A3	1,65554524	0,829309537	0,826235703
225504_at	HMBOX1	2,50022283	1,674071917	0,826150914
218250_s_at	CNOT7	5,607747936	4,781745458	0,826002478
201707_at	PEX19	3,19484646	2,36899666	0,825849799
229572_at	ATP6V0A2	2,836219929	2,010406245	0,825813684
218922_s_at	CERS4	0,735999505	-0,089635713	0,825635218
213669_at	FCHO1	0,735999505	-0,089635713	0,825635218
1569374_at	C3orf62	-0,791294935	-1,616924751	0,825629816
207570_at	SHOX	-0,791294935	-1,616924751	0,825629816
242937_at	FOXK2	-0,791294935	-1,616924751	0,825629816
232360_at	EHF	-0,791294935	-1,616924751	0,825629816
216585_at	-	-0,791294935	-1,616924751	0,825629816
223913_s_at	MIR7-3HG	-0,791294935	-1,616924751	0,825629816
240682_at	LINC00293	-0,791294935	-1,616924751	0,825629816
1563607_x_at	LOC286359	-0,791294935	-1,616924751	0,825629816
237900_at	KLHDC4 /// LOC10	-0,791294935	-1,616924751	0,825629816
221462_x_at	KLK15	-0,791294935	-1,616924751	0,825629816
231472_at	FBXO15	-0,791294935	-1,616924751	0,825629816
1568745_at	LOC646268	-0,791294935	-1,616924751	0,825629816
235404_at	ARID5B	-0,791294935	-1,616924751	0,825629816
207567_at	SLC13A2	-0,791294935	-1,616924751	0,825629816
205274_at	GTPBP1	-0,791294935	-1,616924751	0,825629816
215288_at	TRPC2	-0,791294935	-1,616924751	0,825629816
220657_at	KLHL11	-0,791294935	-1,616924751	0,825629816
232276_at	HS6ST3	-0,791294935	-1,616924751	0,825629816
235593_at	ZEB2	-0,791294935	-1,616924751	0,825629816
1558770_a_at	PIK3R6	-0,791294935	-1,616924751	0,825629816
229060_at	YPEL2	-0,791294935	-1,616924751	0,825629816
210483_at	LOC254896 /// TN	-0,791294935	-1,616924751	0,825629816
1555823_at	PACS2	3,660947664	2,835336991	0,825610673
217927_at	SPCS1	7,052529761	6,227040417	0,825489344
211383_s_at	WDR37	2,582572508	1,757155322	0,825417186
200860_s_at	CNOT1	4,821582996	3,996494541	0,825088455

201592_at	EIF3H	6,881195055	6,056250037	0,824945018
210880_s_at	EFS	0,127732831	-0,697182596	0,824915427
209561_at	THBS3	0,127732831	-0,697182596	0,824915427
216537_s_at	SIGLEC7	0,127732831	-0,697182596	0,824915427
1566178_x_at	-	1,047341799	0,222432814	0,824908985
202871_at	TRAF4	2,063971513	1,239268196	0,824703318
223675_s_at	VEZT	3,257413106	2,432792627	0,824620479
226424_at	CAPS	0,707657549	-0,116767475	0,824425023
209405_s_at	FAM3A	0,707657549	-0,116767475	0,824425023
225231_at	CBL	3,614400403	2,790111983	0,82428842
215711_s_at	WEE1	2,62869474	1,804519951	0,824174789
1552760_at	HDAC9	-1,982324124	-2,8064286	0,824104476
211586_s_at	ATP2B2	-1,982324124	-2,8064286	0,824104476
238665_at	ZNF865	1,267146969	0,443143223	0,824003746
241467_at	-	1,267146969	0,443143223	0,824003746
1555620_a_at	PTGIR	1,458255986	0,634301128	0,823954858
224711_at	YY1	4,123563902	3,299705477	0,823858425
217944_at	POMGNT1	1,936380191	1,112548615	0,823831576
213529_at	ZNF688	1,024284941	0,20062106	0,823663881
222287_at	TRDN	-1,712732543	-2,536339282	0,823606739
244116_at	-	-1,712732543	-2,536339282	0,823606739
229085_at	LRRC3B	-1,712732543	-2,536339282	0,823606739
1569062_s_at	IQGAP3	-1,712732543	-2,536339282	0,823606739
211421_s_at	RET	-1,712732543	-2,536339282	0,823606739
238378_at	-	-1,712732543	-2,536339282	0,823606739
1570058_at	SUV420H2	-1,712732543	-2,536339282	0,823606739
207804_s_at	FCN2	-1,712732543	-2,536339282	0,823606739
1563961_at	FHAD1	-1,712732543	-2,536339282	0,823606739
206440_at	LIN7A	-1,712732543	-2,536339282	0,823606739
227644_at	RIMS4	-1,712732543	-2,536339282	0,823606739
242339_at	-	-1,712732543	-2,536339282	0,823606739
1562432_at	-	-1,712732543	-2,536339282	0,823606739
1554780_a_at	PHTF2	2,552836808	1,72935563	0,823481178
218823_s_at	KCTD9	4,20258728	3,379204729	0,82338255
52651_at	COL8A2	-0,953908971	-1,776824313	0,822915342
226006_at	PET100	4,995098967	4,172471673	0,822627294
221622_s_at	TMEM126B	5,432035227	4,609410944	0,822624283
226347_at	FUT11	3,804904984	2,982339418	0,822565566
232591_s_at	TMEM30A	2,401508182	1,578991006	0,822517176
237473_at	PPIEL	0,677745787	-0,144765583	0,82251137
203308_x_at	HPS1	1,440265662	0,61779983	0,822465832
234976_x_at	MTHFD2	1,745502609	0,923203038	0,82229957
220203_at	BMP8A	1,000459215	0,178191865	0,822267349
214540_at	HIST1H2BO	-0,551284523	-1,372734086	0,821449563
214178_s_at	SOX2	-0,551284523	-1,372734086	0,821449563
206353_at	COX6A2	-0,551284523	-1,372734086	0,821449563
241862_x_at	C19orf45	-0,551284523	-1,372734086	0,821449563
235710_at	VPS53	-0,551284523	-1,372734086	0,821449563
207310_s_at	NOS1	-0,551284523	-1,372734086	0,821449563
232677_at	-	-0,551284523	-1,372734086	0,821449563

239128_at	TMEM221	-0,551284523	-1,372734086	0,821449563
239555_at	-	-0,551284523	-1,372734086	0,821449563
243097_x_at	-	-0,551284523	-1,372734086	0,821449563
241671_x_at	LINC00340	-0,551284523	-1,372734086	0,821449563
230092_at	UBXN10	-0,551284523	-1,372734086	0,821449563
241871_at	CAMK4	4,146990808	3,325881269	0,821109539
224624_at	LRRC8A	4,226167036	3,405078581	0,821088454
229754_at	LOC100507297	1,421391637	0,600385088	0,821006549
244418_at	-	-0,280273599	-1,100943374	0,820669775
237025_at	-	-0,280273599	-1,100943374	0,820669775
220708_at	-	-0,280273599	-1,100943374	0,820669775
1552872_at	ASMTL-AS1	-0,280273599	-1,100943374	0,820669775
206407_s_at	CCL13	-0,280273599	-1,100943374	0,820669775
240377_at	LOC100288332 ///	-0,280273599	-1,100943374	0,820669775
214619_at	CRHR1	-0,280273599	-1,100943374	0,820669775
220824_at	-	-0,280273599	-1,100943374	0,820669775
236770_at	TPTE2P5	-0,280273599	-1,100943374	0,820669775
215258_at	CADM4	-0,280273599	-1,100943374	0,820669775
205305_at	FGL1	-0,280273599	-1,100943374	0,820669775
216704_at	-	-0,280273599	-1,100943374	0,820669775
1558705_at	ATOH8	-0,280273599	-1,100943374	0,820669775
230059_at	DEAF1	-0,280273599	-1,100943374	0,820669775
224190_x_at	NOD1	-0,280273599	-1,100943374	0,820669775
230728_at	FKBP14	2,21987062	1,399283506	0,820587114
227391_x_at	LRRFIP1	5,626536098	4,805980808	0,82055529
238006_at	SIN3A	0,08619576	-0,734355396	0,820551156
230648_at	LOC283663	0,08619576	-0,734355396	0,820551156
228216_at	-	0,648195588	-0,172194225	0,820389813
236049_at	WDR90	0,618277321	-0,201789721	0,820067042
1564149_at	-	-1,137208348	-1,956681069	0,819472721
1557759_at	ATP5SL	-1,137208348	-1,956681069	0,819472721
234234_at	-	-1,137208348	-1,956681069	0,819472721
1554214_at	-	-1,137208348	-1,956681069	0,819472721
1553282_at	C21orf128	-1,137208348	-1,956681069	0,819472721
237818_at	LARP6	-1,137208348	-1,956681069	0,819472721
219790_s_at	NPR3	-1,137208348	-1,956681069	0,819472721
206136_at	FZD5	-1,137208348	-1,956681069	0,819472721
211544_s_at	GHRHR	-1,137208348	-1,956681069	0,819472721
243050_at	-	-1,137208348	-1,956681069	0,819472721
211472_at	PLXNB2	-1,137208348	-1,956681069	0,819472721
1559713_at	LOC100507150	-1,137208348	-1,956681069	0,819472721
225557_at	CSRNP1	1,225123479	0,405752839	0,81937064
1553705_a_at	CHRM3	1,716003175	0,896834102	0,819169073
1558103_a_at	-	1,561816652	0,742671819	0,819144833
235013_at	SLC31A1	1,561816652	0,742671819	0,819144833
224248_x_at	FAM192A	2,374261973	1,555165465	0,819096508
238911_at	STARD10	0,58788177	-0,231045907	0,818927677
212090_at	GRINA	0,58788177	-0,231045907	0,818927677
203522_at	CCS	2,817838865	1,999354538	0,818484326
37996_s_at	DMPK	-0,134167544	-0,952068073	0,817900529

216520_s_at	TPT1	8,428008047	7,610161235	0,817846812
1555837_s_at	POLR2B	5,817632533	4,999808362	0,817824171
204384_at	GOLGA2	1,544940605	0,727123109	0,817817496
203264_s_at	ARHGEF9	2,096248267	1,278654284	0,817593984
232497_at	ZNF3	-0,994186487	-1,811780055	0,817593568
232567_at	ARHGAP8 /// PRR5	-0,994186487	-1,811780055	0,817593568
1563728_at	LINC00032	-0,994186487	-1,811780055	0,817593568
220611_at	DAB1	-0,994186487	-1,811780055	0,817593568
214206_at	PPIL6	-0,994186487	-1,811780055	0,817593568
1557146_a_at	SSTR5-AS1	-0,994186487	-1,811780055	0,817593568
237686_at	RNF219	-0,994186487	-1,811780055	0,817593568
237870_at	NQO2	-0,994186487	-1,811780055	0,817593568
1555188_at	MTUS2-AS1	-0,994186487	-1,811780055	0,817593568
223496_s_at	CCDC8	-0,994186487	-1,811780055	0,817593568
216633_s_at	PLCH1	-0,994186487	-1,811780055	0,817593568
244536_at	-	-0,994186487	-1,811780055	0,817593568
230131_x_at	ARSD	-0,994186487	-1,811780055	0,817593568
242192_at	-	-0,994186487	-1,811780055	0,817593568
214438_at	HLX	-0,994186487	-1,811780055	0,817593568
219541_at	LIME1 /// SLC2A4R	2,692288672	1,874839058	0,817449613
221922_at	GPSM2	3,353217129	2,535878988	0,817338141
1554252_a_at	CERS3	-1,452713826	-2,27001641	0,817302584
231915_at	ZSWIM4	-1,452713826	-2,27001641	0,817302584
218736_s_at	PALMD	-1,452713826	-2,27001641	0,817302584
234078_at	ADAMTS9-AS2	-1,452713826	-2,27001641	0,817302584
217359_s_at	NCAM1	-1,452713826	-2,27001641	0,817302584
1555587_at	PDZRN3	-1,452713826	-2,27001641	0,817302584
1569231_x_at	-	-1,452713826	-2,27001641	0,817302584
242427_at	WAC	-1,452713826	-2,27001641	0,817302584
1563241_at	-	-1,452713826	-2,27001641	0,817302584
208359_s_at	KCNJ4	-1,452713826	-2,27001641	0,817302584
227519_at	PLAC4	-1,452713826	-2,27001641	0,817302584
223579_s_at	APOB	-1,452713826	-2,27001641	0,817302584
202995_s_at	FBLN1	-1,452713826	-2,27001641	0,817302584
1570408_at	-	-1,452713826	-2,27001641	0,817302584
237170_at	LOC100507384	-1,452713826	-2,27001641	0,817302584
219524_s_at	NDUFAF5	-1,452713826	-2,27001641	0,817302584
1566505_at	ERVK13-1	-1,452713826	-2,27001641	0,817302584
244051_at	-	-1,452713826	-2,27001641	0,817302584
1565733_at	-	-1,452713826	-2,27001641	0,817302584
205800_at	-	-1,452713826	-2,27001641	0,817302584
243119_at	-	-1,452713826	-2,27001641	0,817302584
1556231_a_at	-	-1,452713826	-2,27001641	0,817302584
240396_at	-	-1,452713826	-2,27001641	0,817302584
230406_at	-	-1,452713826	-2,27001641	0,817302584
223695_s_at	ARSD	-1,452713826	-2,27001641	0,817302584
200989_at	HIF1A	4,886957485	4,069659213	0,817298272
229295_at	IL17RA	1,686984721	0,869824768	0,817159952
223191_at	COX16	5,080169333	4,263096467	0,817072866
222866_s_at	FLVCR2	0,556428218	-0,260526297	0,816954515

226369_at	LOC338799	0,556428218	-0,260526297	0,816954515
219190_s_at	EIF2C4	0,045517965	-0,771340337	0,816858302
233843_at	ZBTB12	0,045517965	-0,771340337	0,816858302
228685_at	IL17RA	0,045517965	-0,771340337	0,816858302
218152_at	HMG20A	2,460890032	1,644429915	0,816460117
220580_at	BICC1	-1,369772723	-2,186086603	0,81631388
1557857_a_at	-	-1,369772723	-2,186086603	0,81631388
208402_at	IL17A	-1,369772723	-2,186086603	0,81631388
233174_at	LOC100287015	-1,369772723	-2,186086603	0,81631388
1558210_at	SRC	-1,369772723	-2,186086603	0,81631388
220830_at	IMPG2	-1,369772723	-2,186086603	0,81631388
1553550_at	VN1R5	-1,369772723	-2,186086603	0,81631388
238568_s_at	-	-1,369772723	-2,186086603	0,81631388
227439_at	ANKS1B	-1,369772723	-2,186086603	0,81631388
216792_at	-	-1,369772723	-2,186086603	0,81631388
234648_s_at	NXF5	-1,369772723	-2,186086603	0,81631388
232409_x_at	FBXL16	-1,369772723	-2,186086603	0,81631388
210940_s_at	GRM1	-1,369772723	-2,186086603	0,81631388
1569065_s_at	C15orf62	-1,369772723	-2,186086603	0,81631388
237262_at	FAM59A	-1,369772723	-2,186086603	0,81631388
211483_x_at	CAMK2B	-1,369772723	-2,186086603	0,81631388
205797_s_at	TCP11L1	1,983901861	1,167919222	0,815982639
213055_at	CD47	0,524545436	-0,290694975	0,815240411
228524_at	ADCK5	0,491906512	-0,322340048	0,81424656
232907_at	UBR4	0,491906512	-0,322340048	0,81424656
216607_s_at	CYP51A1 /// LRRD:	3,45766549	2,64347734	0,81418815
227256_at	USP31	1,160996507	0,346964736	0,814031771
229673_at	C14orf118	1,510970097	0,697221375	0,813748723
213207_s_at	GOSR2	0,458905032	-0,354781582	0,813686613
228078_at	-	-0,609040214	-1,422257026	0,813216813
244365_at	-	-0,609040214	-1,422257026	0,813216813
1566602_at	RPUSD3	-0,609040214	-1,422257026	0,813216813
203724_s_at	RUFY3	-0,609040214	-1,422257026	0,813216813
244245_at	ANKRD9	-0,609040214	-1,422257026	0,813216813
226162_at	SLC30A6	-0,609040214	-1,422257026	0,813216813
206371_at	FOLR3	-0,609040214	-1,422257026	0,813216813
1565848_at	ZNF428	-0,609040214	-1,422257026	0,813216813
219064_at	ITIH5	-0,609040214	-1,422257026	0,813216813
207376_at	VENTX	-0,609040214	-1,422257026	0,813216813
231657_s_at	CCDC74B	-0,609040214	-1,422257026	0,813216813
209783_at	DBP	-0,609040214	-1,422257026	0,813216813
243821_at	MRPS31	-0,609040214	-1,422257026	0,813216813
240493_at	-	0,001114523	-0,811769547	0,81288407
1558586_at	ZNF33B	0,001114523	-0,811769547	0,81288407
238175_at	-	0,001114523	-0,811769547	0,81288407
210021_s_at	CCNO	0,001114523	-0,811769547	0,81288407
1563105_at	-	0,001114523	-0,811769547	0,81288407
1565537_at	NKX1-1	0,001114523	-0,811769547	0,81288407
234695_x_at	FAM22A	0,001114523	-0,811769547	0,81288407
213168_at	SP3	4,57799322	3,765267919	0,812725301

241847_at	-	-0,332405896	-1,145057014	0,812651118
208108_s_at	AVPR2	-0,332405896	-1,145057014	0,812651118
240878_at	SPEM1	-0,332405896	-1,145057014	0,812651118
205670_at	GAL3ST1	-0,332405896	-1,145057014	0,812651118
237230_at	GPHA2	-0,332405896	-1,145057014	0,812651118
204954_s_at	DYRK1B	-0,332405896	-1,145057014	0,812651118
207808_s_at	PROS1	-0,332405896	-1,145057014	0,812651118
1569372_at	LOC100507194	-1,537851782	-2,350444759	0,812592977
224270_at	OCR1	-1,537851782	-2,350444759	0,812592977
216403_at	SP3P	-1,537851782	-2,350444759	0,812592977
208502_s_at	PITX1	-1,537851782	-2,350444759	0,812592977
201462_at	SCRN1	-1,537851782	-2,350444759	0,812592977
237976_at	-	-1,537851782	-2,350444759	0,812592977
211645_x_at	-	-1,537851782	-2,350444759	0,812592977
1562550_at	-	-1,537851782	-2,350444759	0,812592977
237279_at	-	-1,537851782	-2,350444759	0,812592977
216818_s_at	OR2J2	-1,537851782	-2,350444759	0,812592977
1555544_a_at	CADM2	-1,537851782	-2,350444759	0,812592977
215125_s_at	UGT1A1 /// UGT1/	-1,537851782	-2,350444759	0,812592977
237562_at	-	-1,537851782	-2,350444759	0,812592977
217271_at	-	-1,537851782	-2,350444759	0,812592977
211306_s_at	FCAR	-1,537851782	-2,350444759	0,812592977
208589_at	TRPC7	-1,537851782	-2,350444759	0,812592977
1559392_s_at	SYT7	-1,537851782	-2,350444759	0,812592977
1563104_at	-	-1,537851782	-2,350444759	0,812592977
241763_s_at	FBXO32	-1,537851782	-2,350444759	0,812592977
224481_s_at	HECTD1	3,331722254	2,519368159	0,812354095
232018_at	LENG1	1,345611839	0,533258442	0,812353397
225962_at	ZNRF1	2,127965541	1,315897482	0,812068059
223844_at	ADAMTS13	0,425036312	-0,386896102	0,811932415
233235_x_at	-	0,425036312	-0,386896102	0,811932415
240198_at	-	0,425036312	-0,386896102	0,811932415
209128_s_at	SART3	3,374552989	2,562621495	0,811931494
218343_s_at	GTF3C3	3,748268542	2,936370826	0,811897716
214333_x_at	IDH3G	2,118167848	1,306903969	0,811263879
223511_at	SPRTN	1,326463531	0,515457602	0,811005929
225417_at	EPC1	2,983826368	2,173028091	0,810798276
209916_at	DHTKD1	3,200057698	2,390281999	0,809775699
218255_s_at	FBRS	1,476175815	0,666486137	0,809689678
1554462_a_at	DNAJB9	2,190140919	1,380552339	0,80958858
233142_at	-	-0,856259172	-1,665791776	0,809532604
224337_s_at	FZD4	-0,856259172	-1,665791776	0,809532604
216528_at	-	-0,856259172	-1,665791776	0,809532604
239222_at	C9orf9	-0,856259172	-1,665791776	0,809532604
213695_at	PON3	-0,856259172	-1,665791776	0,809532604
242037_at	ASPH	-0,856259172	-1,665791776	0,809532604
228873_at	COL22A1	-0,856259172	-1,665791776	0,809532604
209677_at	PRKCI	-0,856259172	-1,665791776	0,809532604
221317_x_at	PCDHB6	-0,856259172	-1,665791776	0,809532604
1566991_at	ARID1B	-0,856259172	-1,665791776	0,809532604

216760_at	HRASLS2	-0,856259172	-1,665791776	0,809532604
202768_at	FOSB	-0,856259172	-1,665791776	0,809532604
1552717_s_at	CEP170 /// CEP17(	0,39105295	-0,418248858	0,809301808
207871_s_at	ST7 /// ST7-OT3	4,070902947	3,261989326	0,808913621
217408_at	MRPS18B	4,806642953	3,997758123	0,80888483
205756_s_at	F8	3,228532861	2,419753897	0,808778964
204382_at	NAT9	1,910557155	1,101780212	0,808776944
207783_x_at	HUWE1	8,241980831	7,433379268	0,808601563
210110_x_at	HNRNPH3	3,525566827	2,717094254	0,808472573
204692_at	LRCH4 /// SAP25	1,094118704	0,286279868	0,807838836
222955_s_at	FAM45A /// FAM4	1,094118704	0,286279868	0,807838836
229431_at	RFXAP	2,605420261	1,797682937	0,807737323
205928_at	ZNF443	2,085817135	1,278654284	0,807162851
204658_at	TRA2A	4,328738561	3,521590309	0,807148251
207132_x_at	PFDN5	6,480372497	5,673256228	0,807116269
219552_at	SVEP1	-1,289805289	-2,09678811	0,806982821
1554325_at	DOCK2	-1,289805289	-2,09678811	0,806982821
201610_at	ICMT	-1,289805289	-2,09678811	0,806982821
231588_at	PRCP	-1,289805289	-2,09678811	0,806982821
239338_x_at	-	-1,289805289	-2,09678811	0,806982821
231123_at	TRIM36	-1,289805289	-2,09678811	0,806982821
206417_at	CNGA1	-1,289805289	-2,09678811	0,806982821
1561384_a_at	LOC284661	-1,289805289	-2,09678811	0,806982821
232936_at	KCNA7	-1,289805289	-2,09678811	0,806982821
1560286_s_at	-	-1,289805289	-2,09678811	0,806982821
1552402_at	CALML6	-1,289805289	-2,09678811	0,806982821
230963_at	EMX2OS	-1,289805289	-2,09678811	0,806982821
237931_at	-	-1,289805289	-2,09678811	0,806982821
206170_at	ADRB2	-1,289805289	-2,09678811	0,806982821
1553789_a_at	C21orf58	0,355751005	-0,45121326	0,806964265
221476_s_at	RPL15	7,6326125	6,825735573	0,806876927
210815_s_at	CALCRL	-0,384193355	-1,19085694	0,806663585
207602_at	TMPRSS11D	-0,384193355	-1,19085694	0,806663585
212670_at	ELN	-0,384193355	-1,19085694	0,806663585
243833_at	UNC5A	-0,384193355	-1,19085694	0,806663585
205645_at	REPS2	-0,384193355	-1,19085694	0,806663585
91920_at	BCAN	0,634436907	-0,172194225	0,806631132
212876_at	B4GALT4	1,995527416	1,189078962	0,806448454
1560781_at	DNHD1	-2,074223907	-2,880284116	0,806060209
232399_at	DCLK3	-2,074223907	-2,880284116	0,806060209
219691_at	SAMD9	1,578719607	0,772793615	0,805925992
218338_at	PHC1	2,321828846	1,516067292	0,805761554
205197_s_at	ATP7A	1,267146969	0,461385738	0,805761232
202294_at	STAG1	2,752615529	1,946903052	0,805712476
212954_at	DYRK4	2,937809196	2,132145748	0,805663448
229903_x_at	RNPC3	3,055618382	2,250228409	0,805389973
238135_at	AGTRAP	-0,043925712	-0,84928999	0,805364278
238228_at	-	-0,043925712	-0,84928999	0,805364278
243824_at	-	-0,043925712	-0,84928999	0,805364278
1565905_at	FLJ46026	-0,043925712	-0,84928999	0,805364278

203084_at	TGFB1	-0,043925712	-0,84928999	0,805364278
1569048_s_at	LMF1	-0,043925712	-0,84928999	0,805364278
218193_s_at	GOLT1B	3,237994092	2,432792627	0,805201465
222517_at	AP3M1	2,692288672	1,887158627	0,805130045
212862_at	CDS2	2,978070706	2,173028091	0,805042615
202616_s_at	MECP2	1,561816652	0,757638486	0,804178166
222142_at	CYLD	0,319287178	-0,484558493	0,803845671
222567_s_at	MEX3C	0,319287178	-0,484558493	0,803845671
226058_at	B3GNT9	0,319287178	-0,484558493	0,803845671
47069_at	PRR5	0,284552899	-0,519201391	0,80375429
227209_at	CNTN1	-1,624955693	-2,4284334	0,803477707
217246_s_at	DIAPH2	-1,624955693	-2,4284334	0,803477707
224367_at	BEX2	-1,624955693	-2,4284334	0,803477707
1570106_at	-	-1,624955693	-2,4284334	0,803477707
211083_s_at	MAP3K13	-1,624955693	-2,4284334	0,803477707
228310_at	ENAH	-1,624955693	-2,4284334	0,803477707
240641_at	-	-1,624955693	-2,4284334	0,803477707
222913_at	KLF3	-1,624955693	-2,4284334	0,803477707
234646_at	-	-1,624955693	-2,4284334	0,803477707
1570110_at	-	-1,624955693	-2,4284334	0,803477707
218863_s_at	TNS1	-1,624955693	-2,4284334	0,803477707
207337_at	CTAG2	-1,624955693	-2,4284334	0,803477707
1556740_at	EGFLAM-AS2	-1,624955693	-2,4284334	0,803477707
208550_x_at	KCNG2	-1,624955693	-2,4284334	0,803477707
237162_at	KANK1	-1,624955693	-2,4284334	0,803477707
238415_at	-	-1,624955693	-2,4284334	0,803477707
204457_s_at	GAS1	-1,624955693	-2,4284334	0,803477707
220631_at	OSGEPL1	2,042564225	1,239268196	0,803296029
227166_at	DNAJC18	2,21987062	1,417157926	0,802712694
212695_at	CRY2	1,047341799	0,244646697	0,802695101
210823_s_at	PTPRS	-0,66934516	-1,471833228	0,802488068
237187_at	-	-0,66934516	-1,471833228	0,802488068
215877_at	C14orf56	-0,66934516	-1,471833228	0,802488068
229691_at	ZBTB42	-0,66934516	-1,471833228	0,802488068
1553518_at	DEFT1P /// DEFT1I	-0,66934516	-1,471833228	0,802488068
238062_at	GPIHBP1	-0,66934516	-1,471833228	0,802488068
238336_s_at	DNAJC21	-0,66934516	-1,471833228	0,802488068
209597_s_at	PNMA2	-0,66934516	-1,471833228	0,802488068
229023_at	SFT2D3	-0,66934516	-1,471833228	0,802488068
229924_s_at	-	-0,66934516	-1,471833228	0,802488068
213692_s_at	VDR	-0,66934516	-1,471833228	0,802488068
222711_s_at	RHBDF1	-0,66934516	-1,471833228	0,802488068
229203_at	B4GALNT3	-0,66934516	-1,471833228	0,802488068
1565587_at	-	-0,66934516	-1,471833228	0,802488068
239666_at	PYGO2	-0,66934516	-1,471833228	0,802488068
220271_x_at	EFCAB6	-0,66934516	-1,471833228	0,802488068
222474_s_at	TOMM22	4,646299467	3,843963198	0,802336269
237390_at	-	-1,893506789	-2,695640852	0,802134063
230550_at	MS4A6A	-1,893506789	-2,695640852	0,802134063
1566722_a_at	SVEP1	-1,893506789	-2,695640852	0,802134063

1556804_s_at	POLR3B	-1,893506789	-2,695640852	0,802134063
220781_at	01.12.15	-1,893506789	-2,695640852	0,802134063
1552750_at	CIB3	-1,893506789	-2,695640852	0,802134063
1564307_a_at	A2ML1	-1,893506789	-2,695640852	0,802134063
1555862_s_at	MICALL2	-1,893506789	-2,695640852	0,802134063
1553765_a_at	KLHL32	-1,893506789	-2,695640852	0,802134063
224522_s_at	DCAKD	2,365121417	1,563256447	0,801864971
215493_x_at	BTN2A1	2,71254166	1,911502437	0,801039223
227908_at	TBC1D24	1,528073958	0,727123109	0,80095085
218022_at	VRK3	3,398333742	2,597466271	0,800867471
1558871_at	-	-1,802716385	-2,603471267	0,800754882
242252_at	-	-1,802716385	-2,603471267	0,800754882
1568919_at	-	-1,802716385	-2,603471267	0,800754882
241335_at	-	-1,802716385	-2,603471267	0,800754882
1567277_at	CTTN	-1,802716385	-2,603471267	0,800754882
1565740_at	-	-1,802716385	-2,603471267	0,800754882
241949_at	ACOT6	-1,802716385	-2,603471267	0,800754882
237830_at	-	-1,802716385	-2,603471267	0,800754882
1567282_at	OR1J4	-1,802716385	-2,603471267	0,800754882
213272_s_at	TMEM159	0,282358733	-0,518232988	0,800591721
229914_at	FLJ38717	0,282358733	-0,518232988	0,800591721
225907_at	LOC728743	0,282358733	-0,518232988	0,800591721
209441_at	RHOBTB2	0,282358733	-0,518232988	0,800591721
240508_at	-	0,282358733	-0,518232988	0,800591721
213602_s_at	MMP11	-0,088974936	-0,889222211	0,800247275
214367_at	RASGRP2	-0,088974936	-0,889222211	0,800247275
243266_at	-	-0,088974936	-0,889222211	0,800247275
219749_at	SH2D4A	-0,088974936	-0,889222211	0,800247275
222089_s_at	C16orf71	-0,088974936	-0,889222211	0,800247275
219540_at	ZNF267	4,28595496	3,485746794	0,800208167
202721_s_at	GFPT1	3,09217775	2,292099323	0,800078427
203077_s_at	SMAD2	2,804738618	2,004917973	0,799820645
209823_x_at	HLA-DQB1 /// LOC	3,621852128	2,822379032	0,799473095
244294_at	GTF2H5	-1,06282519	-1,862269442	0,799444252
239970_at	-	-1,06282519	-1,862269442	0,799444252
222170_at	-	-1,06282519	-1,862269442	0,799444252
223683_at	ZMYND15	-1,06282519	-1,862269442	0,799444252
1553562_at	CD8B	-1,06282519	-1,862269442	0,799444252
205755_at	ITIH3	-1,06282519	-1,862269442	0,799444252
1559302_at	KIAA1467	-1,06282519	-1,862269442	0,799444252
213424_at	KIAA0895	-1,06282519	-1,862269442	0,799444252
220006_at	CCDC48	-1,06282519	-1,862269442	0,799444252
1553756_at	GLIS3-AS1	-1,06282519	-1,862269442	0,799444252
1563596_at	-	-1,06282519	-1,862269442	0,799444252
205137_x_at	USH1C	-1,06282519	-1,862269442	0,799444252
240371_at	LOC647107	-1,06282519	-1,862269442	0,799444252
205043_at	CFTR	-1,06282519	-1,862269442	0,799444252
231102_at	CROT	0,244681185	-0,554696666	0,799377852
233483_at	TBC1D27	0,244681185	-0,554696666	0,799377852
215938_s_at	PLA2G6	0,244681185	-0,554696666	0,799377852

233236_at	TSPAN16	0,244681185	-0,554696666	0,799377852
236736_at	-	0,244681185	-0,554696666	0,799377852
218282_at	EDEM2	2,179708195	1,380552339	0,799155856
1555847_a_at	LOC284454	2,019346222	1,220470658	0,798875564
218064_s_at	AKAP8L	2,507817181	1,709047448	0,798769733
205070_at	ING3	4,09423887	3,295596077	0,798642792
235647_at	AP4S1	2,321828846	1,523429058	0,798399788
213312_at	C6orf162	2,321828846	1,523429058	0,798399788
218291_at	LAMTOR2	3,936891546	3,138547521	0,798344026
213389_at	ZNF592	1,20396005	0,405752839	0,798207211
235498_at	LRRIQ3	2,16969527	1,371634653	0,798060617
223394_at	SERTAD1	2,16969527	1,371634653	0,798060617
224275_at	GPR98	-1,982324124	-2,780225148	0,797901024
241578_x_at	-	-1,982324124	-2,780225148	0,797901024
1555335_at	ITGA9	-1,982324124	-2,780225148	0,797901024
237949_at	-	-1,982324124	-2,780225148	0,797901024
320_at	PEX6	0,976172871	0,178358633	0,797814237
203262_s_at	FAM50A	3,660947664	2,86314199	0,797805674
202998_s_at	LOXL2	-0,437449947	-1,234835326	0,797385379
230453_s_at	ATP2A3	-0,437449947	-1,234835326	0,797385379
231359_at	APOH	-0,437449947	-1,234835326	0,797385379
211637_x_at	IGH@ /// IGHA1 //	-0,437449947	-1,234835326	0,797385379
229651_at	SEZ6	-0,437449947	-1,234835326	0,797385379
216126_at	-	-0,437449947	-1,234835326	0,797385379
216102_at	PHLDB1	-0,437449947	-1,234835326	0,797385379
241627_x_at	ARHGEF40	-0,437449947	-1,234835326	0,797385379
211872_s_at	RGS11	-0,437449947	-1,234835326	0,797385379
243971_x_at	LOC731789	-0,437449947	-1,234835326	0,797385379
230972_at	ANKRD9	2,38349585	1,586121802	0,797374049
212933_x_at	RPL13 /// SNORD6	7,816082519	7,018736464	0,797346056
218904_s_at	C9orf40	2,007220798	1,209973561	0,797247237
202214_s_at	CUL4B	3,768156719	2,971193817	0,796962902
203459_s_at	VPS16	2,829929672	2,03313004	0,796799632
205031_at	EFNB3	-0,135065865	-0,931539147	0,796473282
1562792_at	NIPAL1	-0,135065865	-0,931539147	0,796473282
216390_at	LCAT	-0,135065865	-0,931539147	0,796473282
236751_at	-	-0,135065865	-0,931539147	0,796473282
241974_at	-	-0,135065865	-0,931539147	0,796473282
234955_at	-	-0,135065865	-0,931539147	0,796473282
219416_at	SCARA3	-0,135065865	-0,931539147	0,796473282
236677_at	NGB	-0,135065865	-0,931539147	0,796473282
209116_x_at	HBB	-0,135065865	-0,931539147	0,796473282
1554837_a_at	CYP4A11 /// CYP4/	-0,135065865	-0,931539147	0,796473282
1569905_at	HSD11B1L	-0,135065865	-0,931539147	0,796473282
209641_s_at	ABCC3	0,20740896	-0,589001171	0,796410131
1557816_a_at	-	0,20740896	-0,589001171	0,796410131
223095_at	MARVELD1	0,20740896	-0,589001171	0,796410131
224990_at	C4orf34	2,983826368	2,187622928	0,79620344
223705_s_at	GPBP1	5,395782629	4,600158152	0,795624477
204402_at	RHBDD3	1,345611839	0,550017041	0,795594798

236611_at	LOC100506676	2,229377375	1,434743608	0,794633767
229850_at	KDSR	1,476175815	0,681874787	0,794301028
222752_s_at	TMEM206	3,843767728	3,049587049	0,794180679
208827_at	PSMB6	6,356536518	5,562600136	0,793936382
212160_at	XPOT	5,024479308	4,230731855	0,793747452
212024_x_at	FLII	3,840618883	3,046977905	0,793640979
209160_at	AKR1C3	2,042564225	1,249194639	0,793369586
218081_at	C20orf27	1,5944803	0,80114751	0,79333279
219342_at	CASD1	3,076375717	2,283169614	0,793206104
209557_s_at	NCDN	0,648195588	-0,144765583	0,792961171
201517_at	NCBP2	4,357852544	3,564966474	0,79288607
222476_at	CNOT6	3,649735397	2,857256661	0,792478736
233492_s_at	LOC728377 /// OR	-1,212652659	-2,005029581	0,792376922
220595_at	PDZRN4	-1,212652659	-2,005029581	0,792376922
242636_at	PRCP	-1,212652659	-2,005029581	0,792376922
1560291_at	RIPPLY1	-1,212652659	-2,005029581	0,792376922
238291_at	UFL1	-1,212652659	-2,005029581	0,792376922
1563007_at	-	-1,212652659	-2,005029581	0,792376922
233477_at	KLK15	-1,212652659	-2,005029581	0,792376922
220543_at	C21orf62	-1,212652659	-2,005029581	0,792376922
1558856_at	DMRTA2	-1,212652659	-2,005029581	0,792376922
1561916_at	-	-1,212652659	-2,005029581	0,792376922
1569617_at	OSBP2	-1,212652659	-2,005029581	0,792376922
207619_at	HCRTR1	-1,212652659	-2,005029581	0,792376922
242456_at	MRE11A	-1,212652659	-2,005029581	0,792376922
231756_at	ZP4	-1,212652659	-2,005029581	0,792376922
1562583_s_at	-	-1,212652659	-2,005029581	0,792376922
233863_at	CASZ1	-1,212652659	-2,005029581	0,792376922
216249_at	PVT1	-1,212652659	-2,005029581	0,792376922
231125_at	-	-1,212652659	-2,005029581	0,792376922
206244_at	CR1	-1,212652659	-2,005029581	0,792376922
207026_s_at	ATP2B3	-1,212652659	-2,005029581	0,792376922
1553449_at	LINC00304	-1,212652659	-2,005029581	0,792376922
221041_s_at	SLC17A5	1,578719607	0,786391549	0,792328058
219092_s_at	IPPK	1,578719607	0,786391549	0,792328058
212346_s_at	MIR4800 /// MXD4	1,139057614	0,346964736	0,792092878
47608_at	TJAP1	3,1274502	2,335379569	0,792070631
200996_at	ACTR3	6,917951515	6,125931888	0,792019628
209151_x_at	TCF3	-0,180990326	-0,972893339	0,791903013
201094_at	RPS29	8,084881799	7,293244353	0,791637446
1566596_at	-	-0,924000698	-1,715576125	0,791575427
232430_at	LOC148696	-0,924000698	-1,715576125	0,791575427
208017_s_at	MCF2	-0,924000698	-1,715576125	0,791575427
237810_at	CLDN6	-0,924000698	-1,715576125	0,791575427
243853_at	-	-0,924000698	-1,715576125	0,791575427
211171_s_at	PDE10A	-0,924000698	-1,715576125	0,791575427
1554853_at	CYP2U1	-0,924000698	-1,715576125	0,791575427
1553175_s_at	PDE5A	-0,924000698	-1,715576125	0,791575427
229938_at	TMEM238	-0,924000698	-1,715576125	0,791575427
228206_at	HS3ST4	-0,924000698	-1,715576125	0,791575427

222367_at	WHAMMP2 /// WI	-0,924000698	-1,715576125	0,791575427
235932_x_at	HMCN2 /// LOC10	-0,924000698	-1,715576125	0,791575427
240940_at	-	-0,924000698	-1,715576125	0,791575427
221697_at	MAP1LC3C	-0,924000698	-1,715576125	0,791575427
210985_s_at	SP100	-0,924000698	-1,715576125	0,791575427
213247_at	SVEP1	-0,924000698	-1,715576125	0,791575427
230587_at	STGC3	-0,924000698	-1,715576125	0,791575427
202718_at	IGFBP2	-0,924000698	-1,715576125	0,791575427
208130_s_at	TBXAS1	-0,924000698	-1,715576125	0,791575427
38069_at	CLCN7	1,864645804	1,073136123	0,791509682
205604_at	HOXD9	0,167727503	-0,623254098	0,790981602
217219_at	DKFZP434A062	0,167727503	-0,623254098	0,790981602
1552596_at	GAS2L2	0,167727503	-0,623254098	0,790981602
214728_x_at	SMARCA4	3,547953548	2,757131856	0,790821692
202480_s_at	DEDD	0,900065578	0,109458907	0,790606671
208544_at	ADRA2B	0,618277321	-0,172194225	0,790471546
210513_s_at	VEGFA	0,618277321	-0,172194225	0,790471546
206747_at	GPRIN2	0,618277321	-0,172194225	0,790471546
1556009_at	PEX13	1,287793882	0,497424714	0,790369168
205965_at	BATF	4,85214636	4,06178694	0,79035942
214462_at	SOCS6	-0,730013898	-1,520022123	0,790008225
206615_s_at	ADAM22	-0,730013898	-1,520022123	0,790008225
232561_at	ZNF771	-0,730013898	-1,520022123	0,790008225
233844_at	CD99L2	-0,730013898	-1,520022123	0,790008225
222211_x_at	SCAND2	-0,730013898	-1,520022123	0,790008225
1564787_at	ELMOD1 /// LOC64	-0,730013898	-1,520022123	0,790008225
220801_s_at	HAO2	-0,730013898	-1,520022123	0,790008225
208167_s_at	MMP16	-0,730013898	-1,520022123	0,790008225
230751_at	WNT4	-0,730013898	-1,520022123	0,790008225
1558176_at	CYAT1 /// IGLC1 //	-0,730013898	-1,520022123	0,790008225
237285_at	SORBS2	-0,730013898	-1,520022123	0,790008225
222003_s_at	DOCK6	-0,730013898	-1,520022123	0,790008225
228165_at	C12orf53	-0,730013898	-1,520022123	0,790008225
1569963_at	-	-0,730013898	-1,520022123	0,790008225
221378_at	CER1	-0,49284695	-1,282520722	0,789673772
1566823_a_at	-	-0,49284695	-1,282520722	0,789673772
240795_at	-	-0,49284695	-1,282520722	0,789673772
204592_at	DLG4	-0,49284695	-1,282520722	0,789673772
237235_at	-	-0,49284695	-1,282520722	0,789673772
204577_s_at	CLUAP1	-0,49284695	-1,282520722	0,789673772
1558412_at	LOC113230	-0,49284695	-1,282520722	0,789673772
220139_at	DNMT3L	-0,49284695	-1,282520722	0,789673772
213525_at	-	-0,49284695	-1,282520722	0,789673772
210781_x_at	GRIN1	-0,49284695	-1,282520722	0,789673772
213159_at	PCNX	0,58788177	-0,201789721	0,789671491
229460_at	FAM126B	2,765070895	1,975797592	0,789273303
204418_x_at	GSTM2	1,561816652	0,772793615	0,789023038
210061_at	ZNF589	0,127732831	-0,661178575	0,788911405
1552846_s_at	RAB42	0,127732831	-0,661178575	0,788911405
229941_at	FAM166B	0,127732831	-0,661178575	0,788911405

220805_at	HRH2	0,127732831	-0,661178575	0,788911405
243720_at	-	0,127732831	-0,661178575	0,788911405
223412_at	KBTBD7	2,41945542	1,630734622	0,788720798
217512_at	KNG1	-1,712732543	-2,501359113	0,78862657
1562472_at	-	-1,712732543	-2,501359113	0,78862657
1562923_at	-	-1,712732543	-2,501359113	0,78862657
222063_s_at	CDS1	-1,712732543	-2,501359113	0,78862657
219782_s_at	ZNF771	-1,712732543	-2,501359113	0,78862657
240545_at	LOC286382	-1,712732543	-2,501359113	0,78862657
241571_at	-	-1,712732543	-2,501359113	0,78862657
1563608_a_at	KCNT1	-1,712732543	-2,501359113	0,78862657
223317_at	ALKBH7	-1,712732543	-2,501359113	0,78862657
216514_at	-	-1,712732543	-2,501359113	0,78862657
1570366_x_at	ZNF709	-1,712732543	-2,501359113	0,78862657
233663_s_at	CDH26	-1,712732543	-2,501359113	0,78862657
228057_at	DDIT4L	-1,712732543	-2,501359113	0,78862657
205933_at	SETBP1	-1,712732543	-2,501359113	0,78862657
233992_x_at	ZNF445	0,873789652	0,085534992	0,788254659
226140_s_at	OTUD1	2,374261973	1,586121802	0,788140172
207387_s_at	GK	1,267146969	0,479089184	0,788057785
64942_at	GPR153	2,335888723	1,547872177	0,788016546
202541_at	AIMP1	4,634811376	3,846857035	0,787954341
37004_at	SFTPB	-0,205499845	-0,993050558	0,787550713
1556709_a_at	LOC283887	0,556428218	-0,231045907	0,787474125
219748_at	TREML2	0,556428218	-0,231045907	0,787474125
202816_s_at	SS18	2,537731447	1,750483134	0,787248314
209256_s_at	KLHDC10	2,365121417	1,578991006	0,786130412
212145_at	MRPS27	4,328738561	3,542765782	0,785972778
217883_at	MMADHC	6,610023317	5,824059887	0,78596343
204516_at	ATXN7	3,340803206	2,55501164	0,785791566
205125_at	PLCD1	0,847529938	0,061773582	0,785756356
223029_s_at	TRAF7	0,847529938	0,061773582	0,785756356
227951_s_at	FAM98C	1,65554524	0,869824768	0,785720471
233287_at	SLC6A17	1,528073958	0,742671819	0,785402139
1557882_at	-	-2,163157732	-2,948507524	0,785349792
1555359_at	PIGQ	0,524545436	-0,260526297	0,785071733
238540_at	LOC401320	0,524545436	-0,260526297	0,785071733
217911_s_at	BAG3	3,378744007	2,593698508	0,785045499
1558855_at	FARP2	-0,230432956	-1,015398016	0,78496506
239638_at	-	-0,230432956	-1,015398016	0,78496506
238579_at	C9orf85	-0,230432956	-1,015398016	0,78496506
1557848_at	-	-0,230432956	-1,015398016	0,78496506
217024_x_at	-	-0,230432956	-1,015398016	0,78496506
239756_at	-	-0,230432956	-1,015398016	0,78496506
226305_at	LYNX1	-0,230432956	-1,015398016	0,78496506
215660_s_at	MAST2	-0,230432956	-1,015398016	0,78496506
231443_at	LOC100505644	-0,230432956	-1,015398016	0,78496506
1552768_at	CAMKK1	-0,230432956	-1,015398016	0,78496506
207531_at	CRYGC	-0,230432956	-1,015398016	0,78496506
228522_at	FBRSL1	-0,230432956	-1,015398016	0,78496506

231092_s_at	-	-0,230432956	-1,015398016	0,78496506
227073_at	MAP3K2	1,402495885	0,61779983	0,784696055
222414_at	MLL3	3,544559557	2,760632255	0,783927302
219544_at	BORA	3,282538749	2,498884538	0,783654212
225703_at	FBRSL1	2,560606634	1,777135134	0,7834715
202374_s_at	AURKAPS1 /// RAB	2,560606634	1,777135134	0,7834715
209907_s_at	ITSN2	2,71254166	1,929092307	0,783449353
396_f_at	EPOR	2,33082239	1,547376668	0,783445722
230792_at	FAAH2	1,97249974	1,189078962	0,783420777
232336_at	ZSWIM5	0,08619576	-0,697182596	0,783378356
235070_at	RBFOX1	0,08619576	-0,697182596	0,783378356
210486_at	ANKMY1	0,08619576	-0,697182596	0,783378356
237766_at	ATP9B	0,08619576	-0,697182596	0,783378356
226688_at	C3orf23	1,383446753	0,600385088	0,783061665
213673_x_at	NENF	0,491906512	-0,290694975	0,782601487
209690_s_at	DOK4	0,491906512	-0,290694975	0,782601487
204104_at	SNAPC2	0,491906512	-0,290694975	0,782601487
1555452_at	RALGPS1	0,820160788	0,03784737	0,782313418
213405_at	RAB22A	3,09217775	2,309925448	0,782252302
224478_s_at	C7orf50	1,493647653	0,711934445	0,781713208
204571_x_at	PIN4	4,177173946	3,395519718	0,781654228
226331_at	BBX	2,468978138	1,687645398	0,78133274
238598_s_at	-	0,458905032	-0,322340048	0,781245079
214830_at	SLC38A6	0,458905032	-0,322340048	0,781245079
218375_at	NUDT9	2,943764123	2,162541631	0,781222493
208652_at	PPP2CA	5,702501466	4,921415169	0,781086297
223239_at	C14orf129	4,784078567	4,003115194	0,780963373
225463_x_at	GPR89A /// GPR89	3,473999051	2,693044596	0,780954455
1560339_s_at	NAP1L4	4,518023232	3,737342734	0,780680498
201595_s_at	ZC3H15	4,619678154	3,839462092	0,780216063
221735_at	WDR48	3,076375717	2,296310714	0,780065003
232382_s_at	PCMTD1	3,233229368	2,45323331	0,779996058
229681_at	-	0,045517965	-0,734355396	0,779873361
242742_at	-	0,045517965	-0,734355396	0,779873361
206246_at	PFKFB4	0,045517965	-0,734355396	0,779873361
212611_at	DTX4	0,425036312	-0,354781582	0,779817894
1560639_at	-	0,425036312	-0,354781582	0,779817894
216231_s_at	B2M	8,567658316	7,788149804	0,779508512
205205_at	RELB	1,5944803	0,815200271	0,779280029
209126_x_at	KRT6B	1,20396005	0,424693306	0,779266744
233775_x_at	LOC100289333	1,476175815	0,697221375	0,77895444
230483_at	-	0,791908897	0,013190398	0,778718499
242151_at	-	0,791908897	0,013190398	0,778718499
212408_at	TOR1AIP1	4,971128895	4,192491623	0,778637272
226838_at	TTC32	4,060218357	3,281812922	0,778405434
32837_at	AGPAT2	2,617078802	1,83889619	0,778182613
208030_s_at	ADD1	4,498569607	3,720600416	0,77796919
235855_at	COX15	0,39105295	-0,386896102	0,777949053
202230_s_at	CHERP	4,18872659	3,410857018	0,777869573
200076_s_at	KXD1	3,09217775	2,314488991	0,777688759

228567_at	MIR4720	1,345611839	0,568155442	0,777456396
219399_at	LIN7C	2,007220798	1,2298509	0,777369898
214420_s_at	CYP2C9	-0,791294935	-1,568385657	0,777090721
239293_at	NRSN1	-0,791294935	-1,568385657	0,777090721
234442_at	-	-0,791294935	-1,568385657	0,777090721
1554261_at	KLHL29	-0,791294935	-1,568385657	0,777090721
1561233_at	LOC283387	-0,791294935	-1,568385657	0,777090721
216540_at	YME1L1	-0,791294935	-1,568385657	0,777090721
1561341_at	-	-0,791294935	-1,568385657	0,777090721
223631_s_at	C19orf33	-0,791294935	-1,568385657	0,777090721
237622_at	-	-0,791294935	-1,568385657	0,777090721
206236_at	GPR4	-0,791294935	-1,568385657	0,777090721
208497_x_at	NEUROG1	-0,791294935	-1,568385657	0,777090721
203138_at	HAT1	5,108108411	4,331027554	0,777080857
242785_at	EML6	-0,280273599	-1,057301851	0,777028252
1558590_at	METTL17	-0,280273599	-1,057301851	0,777028252
234495_at	KLK15	-0,280273599	-1,057301851	0,777028252
218279_s_at	HIST2H2AA3	-0,280273599	-1,057301851	0,777028252
222740_at	ATAD2	4,448374788	3,671698545	0,776676243
208971_at	UROD	2,908690075	2,132145748	0,776544327
236892_s_at	HOXB-AS3	-1,452713826	-2,229233437	0,776519612
203170_at	RRP8	-1,452713826	-2,229233437	0,776519612
220372_at	DNAJC28	-1,452713826	-2,229233437	0,776519612
207229_at	KLRAP1	-1,452713826	-2,229233437	0,776519612
214509_at	HIST1H3A /// HIST	-1,452713826	-2,229233437	0,776519612
215885_at	SSX2	-1,452713826	-2,229233437	0,776519612
243208_x_at	ACTL9	-1,452713826	-2,229233437	0,776519612
228399_at	OSR1	-1,452713826	-2,229233437	0,776519612
211510_s_at	CRHR2	-1,452713826	-2,229233437	0,776519612
207636_at	SERPINI2	-1,452713826	-2,229233437	0,776519612
206897_at	PAGE1	-1,452713826	-2,229233437	0,776519612
215710_at	ST3GAL4	-1,452713826	-2,229233437	0,776519612
214599_at	IVL	-1,452713826	-2,229233437	0,776519612
1562413_at	LINC00167	-1,452713826	-2,229233437	0,776519612
232846_s_at	CDH23 /// LOC100	-1,452713826	-2,229233437	0,776519612
205503_at	PTPN14	-1,452713826	-2,229233437	0,776519612
201046_s_at	RAD23A	3,642593015	2,86626857	0,776324445
229740_at	C17orf109	-0,551284523	-1,32740143	0,776116907
230768_at	-	-0,551284523	-1,32740143	0,776116907
205481_at	ADORA1	-0,551284523	-1,32740143	0,776116907
216576_x_at	IGK@ /// IGKC	-0,551284523	-1,32740143	0,776116907
1569121_at	SLC25A24	-0,551284523	-1,32740143	0,776116907
219652_s_at	CXorf36	-0,551284523	-1,32740143	0,776116907
237643_at	-	-0,551284523	-1,32740143	0,776116907
1553227_s_at	BRWD1	-0,551284523	-1,32740143	0,776116907
228741_s_at	HCN3	-0,551284523	-1,32740143	0,776116907
215979_s_at	SLC7A1	-0,551284523	-1,32740143	0,776116907
217790_s_at	SSR3	2,392346173	1,616354037	0,775992136
226381_at	LOC100506748	2,484824532	1,709047448	0,775777084
221808_at	RAB9A	6,030329426	5,254837816	0,77549161

212869_x_at	TPT1	8,613150536	7,837703334	0,775447201
215429_s_at	ZNF428	2,063971513	1,28853674	0,775434773
1554102_a_at	TMTC4	1,160996507	0,385582632	0,775413875
213187_x_at	FTL	7,998183406	7,22296897	0,775214436
224609_at	SLC44A2	0,764143511	-0,011001236	0,775144747
238597_at	ANKRD13C	0,764143511	-0,011001236	0,775144747
202850_at	ABCD3	5,518430521	4,743674982	0,774755538
208393_s_at	RAD50	3,117098466	2,34250725	0,774591215
200618_at	LASP1	5,340301351	4,565766076	0,774535275
1567361_at	BDNF-AS	-1,537851782	-2,312054429	0,774202647
1552895_a_at	ANKRD30BP2	-1,537851782	-2,312054429	0,774202647
211655_at	IGLC1	-1,537851782	-2,312054429	0,774202647
237340_at	SLC26A8	-1,537851782	-2,312054429	0,774202647
241589_at	-	-1,537851782	-2,312054429	0,774202647
214413_at	TAT	-1,537851782	-2,312054429	0,774202647
238460_at	FAM83A	-1,537851782	-2,312054429	0,774202647
239491_at	CERS5	-1,537851782	-2,312054429	0,774202647
238426_at	TMEM130	-1,537851782	-2,312054429	0,774202647
230316_at	SEC14L2	-1,537851782	-2,312054429	0,774202647
221363_x_at	GPR25	-1,537851782	-2,312054429	0,774202647
233395_at	-	-1,537851782	-2,312054429	0,774202647
207517_at	LAMC2	-1,537851782	-2,312054429	0,774202647
1558885_at	-	-1,537851782	-2,312054429	0,774202647
1566256_s_at	GPR180	-1,537851782	-2,312054429	0,774202647
1553265_at	SLC23A3	-1,537851782	-2,312054429	0,774202647
207444_at	SLC22A13	-1,537851782	-2,312054429	0,774202647
230510_at	HSPB9	-1,537851782	-2,312054429	0,774202647
212573_at	ENDOD1	0,95222556	0,178191865	0,774033695
238329_at	MPRIP	0,355751005	-0,418248858	0,773999863
206838_at	TBX19	0,355751005	-0,418248858	0,773999863
217207_s_at	BTNL3	0,355751005	-0,418248858	0,773999863
216785_at	-	-1,137208348	-1,911206569	0,773998221
207803_s_at	CSN3	-1,137208348	-1,911206569	0,773998221
240253_at	-	-1,137208348	-1,911206569	0,773998221
244249_at	-	-1,137208348	-1,911206569	0,773998221
221252_s_at	GSG1	-1,137208348	-1,911206569	0,773998221
230227_at	-	-1,137208348	-1,911206569	0,773998221
231210_at	C11orf85	-1,137208348	-1,911206569	0,773998221
242971_at	-	-1,137208348	-1,911206569	0,773998221
1561453_at	-	-1,137208348	-1,911206569	0,773998221
223975_at	TRIM51 /// TRIM5	-1,137208348	-1,911206569	0,773998221
1561587_at	LOC284260	-1,137208348	-1,911206569	0,773998221
243952_at	TPTEP1	-1,137208348	-1,911206569	0,773998221
220630_s_at	CHIA	-1,137208348	-1,911206569	0,773998221
237320_at	FAM71F2	-1,137208348	-1,911206569	0,773998221
229909_at	B4GALNT3	-1,137208348	-1,911206569	0,773998221
1561050_a_at	-	-1,137208348	-1,911206569	0,773998221
208437_at	CLCN1	-1,137208348	-1,911206569	0,773998221
214069_at	ACSM2A /// ACSM	-1,137208348	-1,911206569	0,773998221
231729_s_at	CAPS	-1,137208348	-1,911206569	0,773998221

217553_at	STEAP1B	-1,137208348	-1,911206569	0,773998221
209837_at	AP4M1	1,440265662	0,666486137	0,773779525
221938_x_at	MED16	1,440265662	0,666486137	0,773779525
209390_at	TSC1	2,460890032	1,687645398	0,773244634
225096_at	C17orf79	5,037499265	4,264419981	0,773079284
227987_at	VPS13A	1,139057614	0,366031214	0,773026401
220967_s_at	ZNF696	0,735999505	-0,036543687	0,772543192
230517_at	ZNF775	-1,369772723	-2,142286822	0,772514099
221898_at	PDPN	-1,369772723	-2,142286822	0,772514099
237539_at	-	-1,369772723	-2,142286822	0,772514099
241096_at	-	-1,369772723	-2,142286822	0,772514099
1561392_at	-	-1,369772723	-2,142286822	0,772514099
213679_at	TTC30A	-1,369772723	-2,142286822	0,772514099
1565797_at	DUOX1	-1,369772723	-2,142286822	0,772514099
244174_at	-	-1,369772723	-2,142286822	0,772514099
236847_at	C19orf18	-1,369772723	-2,142286822	0,772514099
206924_at	IL11	-1,369772723	-2,142286822	0,772514099
203295_s_at	ATP1A2	-1,369772723	-2,142286822	0,772514099
217676_at	-	-1,369772723	-2,142286822	0,772514099
244748_at	-	-1,369772723	-2,142286822	0,772514099
208387_s_at	MMP24	-1,369772723	-2,142286822	0,772514099
207857_at	LILRA2	-1,369772723	-2,142286822	0,772514099
227642_at	TFCP2L1	-1,369772723	-2,142286822	0,772514099
228656_at	PROX1	-1,369772723	-2,142286822	0,772514099
226210_s_at	MEG3	-1,369772723	-2,142286822	0,772514099
222797_at	DPYSL5	-1,369772723	-2,142286822	0,772514099
210601_at	CDH6	-1,369772723	-2,142286822	0,772514099
243141_at	SGMS2	-1,369772723	-2,142286822	0,772514099
1558437_at	IGHA1	-1,369772723	-2,142286822	0,772514099
204039_at	CEBPA	0,001114523	-0,771340337	0,77245486
230617_at	LOC100507675	0,001114523	-0,771340337	0,77245486
210618_at	RAP1GAP	0,001114523	-0,771340337	0,77245486
201079_at	SYNGR2	4,75100323	3,978556843	0,772446387
206790_s_at	NDUFB1	6,909072121	6,136640748	0,772431373
211998_at	H3F3A /// H3F3B	3,559161816	2,786807892	0,772353924
222645_s_at	KCTD5	1,287793882	0,515457602	0,77233628
206198_s_at	CEACAM7	-1,893506789	-2,665156778	0,771649989
237384_x_at	-	-1,893506789	-2,665156778	0,771649989
231645_s_at	-	-1,893506789	-2,665156778	0,771649989
1558601_at	LSAMP-AS3	-1,893506789	-2,665156778	0,771649989
207893_at	SRY	-1,893506789	-2,665156778	0,771649989
208170_s_at	TRIM31	-1,893506789	-2,665156778	0,771649989
1561617_at	DNAH6	-1,893506789	-2,665156778	0,771649989
227812_at	TNFRSF19	-1,893506789	-2,665156778	0,771649989
201110_s_at	THBS1	-1,893506789	-2,665156778	0,771649989
229101_at	IL17RA	2,179708195	1,408220608	0,771487587
233952_s_at	ZNF295	2,71254166	1,941115499	0,771426161
201035_s_at	HADH	5,523034165	4,751644591	0,771389574
232617_at	CTSS	2,507817181	1,736450458	0,771366723
234660_s_at	DIS3	2,664625637	1,893444329	0,771181308

208942_s_at	SEC62	5,090512962	4,319399526	0,771113437
201148_s_at	TIMP3	0,707657549	-0,063377083	0,771034631
1555470_a_at	PPM1F	0,707657549	-0,063377083	0,771034631
219921_s_at	DOCK5	0,319287178	-0,45121326	0,770500437
229736_at	TMEM86B	0,319287178	-0,45121326	0,770500437
212046_x_at	MAPK3	2,356619952	1,586121802	0,77049815
219613_s_at	SIRT6	1,528073958	0,757638486	0,770435472
235206_at	C20orf152	1,640193838	0,869824768	0,77036907
225717_at	KIAA1715	1,640193838	0,869824768	0,77036907
201433_s_at	PTDSS1	6,189903941	5,419616584	0,770287357
227653_at	TRMT5	2,019346222	1,249194639	0,770151583
201588_at	TXNL1	6,487800531	5,717741859	0,770058671
229980_s_at	SNX5	6,184231288	5,414187153	0,770044135
1562169_at	-	-1,982324124	-2,752097204	0,76977308
239477_at	EFHB	-1,982324124	-2,752097204	0,76977308
230276_at	FAM49A	-1,982324124	-2,752097204	0,76977308
231662_at	ARG1	-1,982324124	-2,752097204	0,76977308
208139_s_at	-	-1,982324124	-2,752097204	0,76977308
224215_s_at	DLL1	-1,982324124	-2,752097204	0,76977308
224665_at	ANAPC16	5,355255298	4,58568697	0,769568328
241531_at	-	-0,994186487	-1,763664074	0,769477587
217384_x_at	IGHV3-48	-0,994186487	-1,763664074	0,769477587
1556084_at	HNRNPM	-0,994186487	-1,763664074	0,769477587
234102_at	RASL11B	-0,994186487	-1,763664074	0,769477587
216130_at	-	-0,994186487	-1,763664074	0,769477587
243890_at	-	-0,994186487	-1,763664074	0,769477587
208053_at	GUCY2F	-0,994186487	-1,763664074	0,769477587
1560449_at	-	-0,994186487	-1,763664074	0,769477587
238031_at	-	-0,994186487	-1,763664074	0,769477587
207858_s_at	PKLR	-0,994186487	-1,763664074	0,769477587
214385_s_at	MUC5AC	-0,994186487	-1,763664074	0,769477587
229048_at	-	-0,994186487	-1,763664074	0,769477587
210804_x_at	SLC8A1	-0,994186487	-1,763664074	0,769477587
1567686_at	CECR9	-0,994186487	-1,763664074	0,769477587
216419_at	CROCC	-0,994186487	-1,763664074	0,769477587
223812_at	FAM178B	-0,994186487	-1,763664074	0,769477587
202382_s_at	GNPDA1	2,348178434	1,578991006	0,769187429
224703_at	DCAF5	2,149438457	1,380552339	0,768886118
202791_s_at	PPP6R2	2,276736537	1,508107415	0,768629122
227027_at	GFPT1	2,829929672	2,06133574	0,768593932
211348_s_at	CDC14B	-0,332405896	-1,100943374	0,768537478
235300_x_at	RCHY1	-0,332405896	-1,100943374	0,768537478
202707_at	UMPS	-0,332405896	-1,100943374	0,768537478
238517_at	KLF7	-0,332405896	-1,100943374	0,768537478
220922_s_at	SPANXA1 /// SPAN	-0,332405896	-1,100943374	0,768537478
242624_at	ABLIM2	-0,332405896	-1,100943374	0,768537478
242858_at	-	-0,332405896	-1,100943374	0,768537478
203592_s_at	FSTL3	-0,332405896	-1,100943374	0,768537478
205639_at	AOAH	-0,332405896	-1,100943374	0,768537478
223364_s_at	DHX37	1,510970097	0,742671819	0,768298278

242149_at	FAM210A	1,510970097	0,742671819	0,768298278
212879_x_at	PIAS4	1,510970097	0,742671819	0,768298278
1553039_a_at	ASB10	1,402495885	0,634301128	0,768194757
218030_at	GIT1	2,074841895	1,306903969	0,767937926
206537_at	XIAP	-0,043925712	-0,811769547	0,767843835
216182_at	SYNJ2	-0,043925712	-0,811769547	0,767843835
224229_s_at	AKT3	-0,043925712	-0,811769547	0,767843835
218778_x_at	EPS8L1	-0,043925712	-0,811769547	0,767843835
230167_at	ADAMTS14	-0,043925712	-0,811769547	0,767843835
230519_at	FAM124A	-0,043925712	-0,811769547	0,767843835
204876_at	ZNF646	-0,043925712	-0,811769547	0,767843835
228840_at	AMOTL1	-0,043925712	-0,811769547	0,767843835
1560973_a_at	-	-1,802716385	-2,570522741	0,767806356
227703_s_at	SYTL4	-1,802716385	-2,570522741	0,767806356
211119_at	ESR2	-1,802716385	-2,570522741	0,767806356
1559688_at	GRAPL	-1,802716385	-2,570522741	0,767806356
1567378_x_at	DNAH1	-1,802716385	-2,570522741	0,767806356
231897_at	PTGR1	-1,802716385	-2,570522741	0,767806356
1553864_at	GPR26	-1,802716385	-2,570522741	0,767806356
226778_at	C8orf42	-1,802716385	-2,570522741	0,767806356
240791_at	-	-1,802716385	-2,570522741	0,767806356
217513_at	MILR1	-1,802716385	-2,570522741	0,767806356
1561501_s_at	CTU2	-1,802716385	-2,570522741	0,767806356
236155_at	ZCCHC6	1,610353504	0,842599219	0,767754284
239269_at	LOC100653017 ///	0,677745787	-0,089635713	0,7673815
202875_s_at	PBX2	1,246401134	0,479089184	0,767311949
1561243_at	TMEM105	1,246401134	0,479089184	0,767311949
221799_at	CHPF2	2,267141818	1,500059916	0,767081902
231824_at	LARP1B	0,282358733	-0,484558493	0,766917226
216555_at	PRR14L	0,282358733	-0,484558493	0,766917226
210841_s_at	NRP2	0,282358733	-0,484558493	0,766917226
1568696_at	CDNF	-1,624955693	-2,391863529	0,766907836
1562727_at	-	-1,624955693	-2,391863529	0,766907836
238358_x_at	-	-1,624955693	-2,391863529	0,766907836
236550_s_at	ZNF311	-1,624955693	-2,391863529	0,766907836
1564706_s_at	GLS2	-1,624955693	-2,391863529	0,766907836
217090_at	-	-1,624955693	-2,391863529	0,766907836
224343_x_at	YME1L1	-1,624955693	-2,391863529	0,766907836
239547_at	HS3ST6	-1,624955693	-2,391863529	0,766907836
1568655_a_at	-	-1,624955693	-2,391863529	0,766907836
233979_s_at	ESPN	-1,624955693	-2,391863529	0,766907836
238984_at	REG4	-1,624955693	-2,391863529	0,766907836
1561612_at	-	-1,624955693	-2,391863529	0,766907836
238286_at	-	-1,624955693	-2,391863529	0,766907836
231935_at	ARPP21	-1,624955693	-2,391863529	0,766907836
1559350_at	-	-1,624955693	-2,391863529	0,766907836
1553188_s_at	PARD3B	-1,624955693	-2,391863529	0,766907836
222921_s_at	HEY2	-1,624955693	-2,391863529	0,766907836
1557261_at	WHAMMP2 /// WI	-1,624955693	-2,391863529	0,766907836
1559976_at	LOC100506314	-1,624955693	-2,391863529	0,766907836

1556502_at	LOC100507654	-1,624955693	-2,391863529	0,766907836
239694_at	TRIM7	-1,624955693	-2,391863529	0,766907836
200086_s_at	COX4I1	6,864321136	6,097514498	0,766806638
204263_s_at	CPT2	2,855122714	2,088449486	0,766673228
1557053_s_at	UBE2G2	2,943764123	2,177710147	0,766053977
211251_x_at	NFYC	2,804738618	2,039063441	0,765675177
226957_x_at	RALBP1	1,701542258	0,936232181	0,765310076
222114_x_at	WDR55	0,648195588	-0,116767475	0,764963063
1556265_at	C20orf202	1,071336699	0,306454867	0,764881832
224688_at	TMEM248	3,362258367	2,597466271	0,764792096
225497_at	ATE1	2,248155141	1,483698568	0,764456573
235093_at	PEX13	2,966903803	2,20251271	0,764391094
212206_s_at	H2AFV	3,735523399	2,971193817	0,764329582
236090_at	-	1,476175815	0,711934445	0,76424137
225588_s_at	TMEM129	1,476175815	0,711934445	0,76424137
215699_x_at	SFI1	1,476175815	0,711934445	0,76424137
222677_x_at	-	-0,609040214	-1,372734086	0,763693872
211679_x_at	GABBR2	-0,609040214	-1,372734086	0,763693872
214580_x_at	KRT6A /// KRT6B /,	-0,609040214	-1,372734086	0,763693872
214667_s_at	TP53I11	-0,609040214	-1,372734086	0,763693872
238066_at	RBP7	-0,609040214	-1,372734086	0,763693872
212889_x_at	GADD45GIP1	-0,609040214	-1,372734086	0,763693872
234828_at	-	-0,609040214	-1,372734086	0,763693872
211839_s_at	CSF1	-0,609040214	-1,372734086	0,763693872
1559376_at	ATP1A1OS	-0,609040214	-1,372734086	0,763693872
217897_at	FXYD6	-0,609040214	-1,372734086	0,763693872
208224_at	HOXB1	-0,609040214	-1,372734086	0,763693872
207892_at	CD40LG	-0,609040214	-1,372734086	0,763693872
240853_at	-	-0,609040214	-1,372734086	0,763693872
208945_s_at	BECN1	4,007783021	3,244090313	0,763692707
221528_s_at	ELMO2	3,09217775	2,328492509	0,763685241
218192_at	IP6K2	3,473999051	2,710570973	0,763428078
241973_x_at	DPP7	0,244681185	-0,518232988	0,762914174
240270_x_at	-	0,244681185	-0,518232988	0,762914174
224503_s_at	ZCCHC2	0,244681185	-0,518232988	0,762914174
235383_at	MYO7B	0,244681185	-0,518232988	0,762914174
218831_s_at	FCGRT	0,244681185	-0,518232988	0,762914174
210371_s_at	RBBP4	5,747305731	4,984398793	0,762906938
1569129_s_at	C3orf38	1,898032047	1,135235995	0,762796052
218111_s_at	CMAS	3,340803206	2,578283758	0,762519448
219304_s_at	PDGFD	0,20740896	-0,554696666	0,762105626
232819_s_at	LTBR	0,20740896	-0,554696666	0,762105626
225560_at	POMT2	0,847529938	0,085534992	0,761994945
203302_at	DCK	4,209562243	3,447692163	0,76187008
202654_x_at	07.03.15	3,624986574	2,86314199	0,761844584
221853_s_at	NOMO1 /// NOMC	5,167863921	4,40626232	0,761601601
218766_s_at	WARS2	2,16969527	1,408220608	0,761474662
227438_at	ALPK1	2,16969527	1,408220608	0,761474662
38043_at	FAM3A	1,402907367	0,641532553	0,761374815
212917_x_at	RECQL	4,146990808	3,385678356	0,761312452

224813_at	WASL	2,636121902	1,874839058	0,761282844
226606_s_at	GTPBP5	1,047341799	0,286279868	0,761061931
225190_x_at	RPL35A	7,823586472	7,062549605	0,761036866
203322_at	ADNP2	3,214269085	2,45323331	0,761035775
233360_at	UBE2I	-0,384193355	-1,145057014	0,760863659
244376_at	METTL7A	-0,384193355	-1,145057014	0,760863659
1560901_at	-	-0,384193355	-1,145057014	0,760863659
215883_at	CTNNA1	-0,384193355	-1,145057014	0,760863659
236284_at	KIAA0146	-0,384193355	-1,145057014	0,760863659
222363_at	-	-0,384193355	-1,145057014	0,760863659
205524_s_at	HAPLN1	-0,384193355	-1,145057014	0,760863659
209072_at	MBP	-0,384193355	-1,145057014	0,760863659
225181_at	ARID1B	3,001575397	2,24081821	0,760757187
226291_at	ALS2	1,561816652	0,80114751	0,760669143
213791_at	PENK	-0,856259172	-1,616924751	0,760665579
207211_at	USP2	-0,856259172	-1,616924751	0,760665579
206927_s_at	GUCY1A2	-0,856259172	-1,616924751	0,760665579
217261_at	TTTY2 /// TTTY2B	-0,856259172	-1,616924751	0,760665579
210682_at	LPO	-0,856259172	-1,616924751	0,760665579
221327_s_at	OPN1LW /// OPN1	-0,856259172	-1,616924751	0,760665579
216910_at	XPNPEP2	-0,856259172	-1,616924751	0,760665579
222897_s_at	ZFP64	-0,856259172	-1,616924751	0,760665579
206393_at	TNNI2	-0,856259172	-1,616924751	0,760665579
234245_at	-	-0,856259172	-1,616924751	0,760665579
221384_at	UCP1	-0,856259172	-1,616924751	0,760665579
217044_s_at	PLEKHG3	-0,856259172	-1,616924751	0,760665579
228294_s_at	ZNF775	-0,856259172	-1,616924751	0,760665579
209356_x_at	EFEMP2	-0,856259172	-1,616924751	0,760665579
239785_at	DZIP1L	-0,088974936	-0,84928999	0,760315054
236369_at	TSPY26P	-0,088974936	-0,84928999	0,760315054
213432_at	MUC5B	-0,088974936	-0,84928999	0,760315054
1565879_at	-	-0,088974936	-0,84928999	0,760315054
205163_at	MYLPF	-0,088974936	-0,84928999	0,760315054
238627_at	TRAPPC2L	0,58788177	-0,172194225	0,760075995
1565610_at	-	-1,289805289	-2,049747196	0,759941907
1560747_at	-	-1,289805289	-2,049747196	0,759941907
233649_at	KATNAL2	-1,289805289	-2,049747196	0,759941907
232144_at	-	-1,289805289	-2,049747196	0,759941907
237480_at	-	-1,289805289	-2,049747196	0,759941907
206107_at	RGS11	-1,289805289	-2,049747196	0,759941907
239574_at	-	-1,289805289	-2,049747196	0,759941907
243035_at	-	-1,289805289	-2,049747196	0,759941907
240938_at	-	-1,289805289	-2,049747196	0,759941907
228265_at	-	-1,289805289	-2,049747196	0,759941907
233310_at	-	-1,289805289	-2,049747196	0,759941907
1564259_at	-	-1,289805289	-2,049747196	0,759941907
216200_at	PLEKHM1	-1,289805289	-2,049747196	0,759941907
237796_at	-	-1,289805289	-2,049747196	0,759941907
241100_at	-	-1,289805289	-2,049747196	0,759941907
1557235_at	-	-1,289805289	-2,049747196	0,759941907

243744_at	FONG	-1,289805289	-2,049747196	0,759941907
1561116_at	-	-1,289805289	-2,049747196	0,759941907
210342_s_at	TPO	-1,289805289	-2,049747196	0,759941907
205057_s_at	IDUA	-1,289805289	-2,049747196	0,759941907
214492_at	SGCD	-1,289805289	-2,049747196	0,759941907
243342_at	-	-1,289805289	-2,049747196	0,759941907
1565562_at	-	-1,289805289	-2,049747196	0,759941907
240559_at	IZUMO2	-1,289805289	-2,049747196	0,759941907
214817_at	UNC13A	-1,289805289	-2,049747196	0,759941907
214966_at	GRIK5	-1,289805289	-2,049747196	0,759941907
215495_s_at	SAMD4A	-1,289805289	-2,049747196	0,759941907
201689_s_at	TPD52	4,221194173	3,461380025	0,759814148
242304_at	WIBG	2,71254166	1,953032682	0,759508977
233427_x_at	-	2,460890032	1,701800759	0,759089273
209805_at	PMS2 /// PMS2CL	1,871521385	1,112548615	0,75897277
224628_at	ERLEC1	4,561620178	3,802659695	0,758960483
217192_s_at	PRDM1	1,024284941	0,265475485	0,758809456
202106_at	GOLGA3	2,51558601	1,757155322	0,758430688
210149_s_at	ATP5H	6,665296854	5,906911884	0,75838497
205260_s_at	ACYP1	4,584332287	3,826007399	0,758324888
241887_at	UBE2W	0,556428218	-0,201789721	0,758217939
203983_at	TSNAX	4,693076946	3,934892246	0,7581847
219289_at	HEATR3	2,613382867	1,855279162	0,758103705
243627_at	-	-2,074223907	-2,832036647	0,75781274
241963_at	ZNF704	-2,074223907	-2,832036647	0,75781274
205126_at	VRK2	3,595895594	2,83813104	0,757764554
40687_at	GJA4	-1,265124126	-2,02278077	0,757656644
203076_s_at	SMAD2	3,934348221	3,176868665	0,757479557
212455_at	YTHDC1	4,705123614	3,947720163	0,757403451
218628_at	CCDC53	5,298547568	4,541228229	0,757319339
214986_x_at	PPIL2	1,307201325	0,550017041	0,757184285
202906_s_at	NBN	3,861345795	3,104562113	0,756783683
1556154_a_at	MGC23284	0,167727503	-0,589001171	0,756728674
237854_at	-	0,167727503	-0,589001171	0,756728674
1562861_at	-	0,167727503	-0,589001171	0,756728674
223929_s_at	-	0,167727503	-0,589001171	0,756728674
220952_s_at	PLEKHA5	2,817838865	2,06133574	0,756503125
208743_s_at	YWHAB	6,038290958	5,282080467	0,756210491
212097_at	CAV1	4,674825018	3,918658606	0,756166412
44617_at	OGFOD2	2,02908267	1,27304216	0,75604051
210586_x_at	RHD	0,524545436	-0,231045907	0,755591343
1556162_at	IGSF3	0,524545436	-0,231045907	0,755591343
203036_s_at	MTSS1	0,524545436	-0,231045907	0,755591343
216266_s_at	ARFGEF1	2,118167848	1,362900801	0,755267047
218292_s_at	PRKAG2	1,160996507	0,405752839	0,755243669
228337_at	PWWP2A	1,160996507	0,405752839	0,755243669
223406_x_at	FAM192A	3,029186714	2,274084398	0,755102316
209404_s_at	TMED7 /// TMED7	3,481948861	2,726850891	0,75509797
209073_s_at	NUMB	2,765070895	2,010406245	0,75466465
228385_at	DDX59	2,99543179	2,24081821	0,754613579

202808_at	WBP1L	2,107136847	1,352622437	0,754514409
201546_at	TRIP12	5,485083996	4,730861472	0,754222524
49051_g_at	DTX3	-1,450526852	-2,204738503	0,754211652
239640_at	LOC401320	-0,135065865	-0,889222211	0,754156346
214622_at	CYP21A2	-0,135065865	-0,889222211	0,754156346
216670_at	KLK13	-0,135065865	-0,889222211	0,754156346
231621_at	MPZL1	-0,135065865	-0,889222211	0,754156346
210079_x_at	KCNAB1	-0,135065865	-0,889222211	0,754156346
236619_at	-	-0,135065865	-0,889222211	0,754156346
225326_at	RBM27	3,966733349	3,212688281	0,754045068
244782_at	PPP3R2	-1,712732543	-2,466349414	0,753616871
237838_at	-	-1,712732543	-2,466349414	0,753616871
1552736_a_at	NETO1	-1,712732543	-2,466349414	0,753616871
224526_at	LOC100132319	-1,712732543	-2,466349414	0,753616871
206344_at	PON1	-1,712732543	-2,466349414	0,753616871
1554929_at	SIK3	-1,712732543	-2,466349414	0,753616871
239578_at	-	-1,712732543	-2,466349414	0,753616871
217057_s_at	GNAS	-1,712732543	-2,466349414	0,753616871
229147_at	RASSF6	-1,712732543	-2,466349414	0,753616871
237484_at	C12orf69	-1,712732543	-2,466349414	0,753616871
1567697_at	-	-1,712732543	-2,466349414	0,753616871
219153_s_at	THSD4	-1,712732543	-2,466349414	0,753616871
1555319_at	STAB1	-1,712732543	-2,466349414	0,753616871
1560769_at	-	-1,712732543	-2,466349414	0,753616871
213953_at	KRT20	-1,712732543	-2,466349414	0,753616871
1556696_s_at	FLJ42709	-1,712732543	-2,466349414	0,753616871
220636_at	DNAI2	-1,712732543	-2,466349414	0,753616871
207739_s_at	GAGE1 /// GAGE1[illegible]	-1,712732543	-2,466349414	0,753616871
1554675_a_at	SYCE1	-1,712732543	-2,466349414	0,753616871
225186_at	RAPH1	-1,712732543	-2,466349414	0,753616871
232546_at	TP73	-1,712732543	-2,466349414	0,753616871
231857_s_at	AGBL5	1,139057614	0,385582632	0,753474982
218937_at	ZNF434	2,179708195	1,426280426	0,753427769
1556284_at	PPA2	-0,437449947	-1,19085694	0,753406992
1561122_a_at	RAD51B	-0,437449947	-1,19085694	0,753406992
233391_at	CDH26	-0,437449947	-1,19085694	0,753406992
1560671_at	KIAA1652	-0,437449947	-1,19085694	0,753406992
214704_at	TCF25	-0,437449947	-1,19085694	0,753406992
237266_at	LOC100289509	-0,437449947	-1,19085694	0,753406992
218610_s_at	CPPED1	-0,437449947	-1,19085694	0,753406992
224145_s_at	SPTBN4	-0,437449947	-1,19085694	0,753406992
214054_at	DOK2	2,530390387	1,777135134	0,753255253
38691_s_at	SFTPC	0,874292943	0,121068226	0,753224717
230326_s_at	C11orf73	5,519395832	4,766405877	0,752989956
244243_at	-	-0,66934516	-1,422257026	0,752911867
238930_at	PPM1N	-0,66934516	-1,422257026	0,752911867
242914_at	-	-0,66934516	-1,422257026	0,752911867
209621_s_at	PDLIM3	-0,66934516	-1,422257026	0,752911867
238373_at	H1FNT	-0,66934516	-1,422257026	0,752911867
222331_at	-	-0,66934516	-1,422257026	0,752911867

204345_at	COL16A1	-0,66934516	-1,422257026	0,752911867
217193_x_at	-	-0,66934516	-1,422257026	0,752911867
226654_at	MUC12	-0,66934516	-1,422257026	0,752911867
244374_at	PLAC2	-0,66934516	-1,422257026	0,752911867
208559_at	PDX1	-0,66934516	-1,422257026	0,752911867
234551_at	OTOP2	-0,66934516	-1,422257026	0,752911867
210443_x_at	OGFR	2,031158928	1,278654284	0,752504644
228303_at	GALNT6	1,267146969	0,515457602	0,751689367
207713_s_at	RBCK1	1,267146969	0,515457602	0,751689367
211112_at	SLC12A4	0,95222556	0,20062106	0,751604501
231835_at	FAM213B	2,878891456	2,127337555	0,751553901
209475_at	USP15	2,817838865	2,066329863	0,751509001
209054_s_at	WHSC1	4,301645306	3,550288791	0,751356515
241836_x_at	CEP97	0,127732831	-0,623254098	0,750986929
1556956_at	KCP	0,127732831	-0,623254098	0,750986929
228139_at	RIPK3	0,127732831	-0,623254098	0,750986929
235245_at	TMEM92	0,127732831	-0,623254098	0,750986929
239823_at	-	0,127732831	-0,623254098	0,750986929
1552664_at	FLCN	1,493647653	0,742671819	0,750975834
227021_at	KDM1B	0,764143511	0,013190398	0,750953113
225677_at	BCAP29	3,521615383	2,770696222	0,750919161
200026_at	RPL34	8,379305196	7,628614599	0,750690597
216337_at	-	-0,180990326	-0,931539147	0,750548821
223994_s_at	SLC12A9	-0,180990326	-0,931539147	0,750548821
1560968_at	-	-0,180990326	-0,931539147	0,750548821
230223_at	ZNF839	-0,180990326	-0,931539147	0,750548821
1552997_a_at	IQCF1	-0,180990326	-0,931539147	0,750548821
232106_s_at	CEP89	-0,180990326	-0,931539147	0,750548821
209322_s_at	SH2B1	1,116462765	0,366031214	0,750431551
46323_at	CANT1	2,606202951	1,855788315	0,750414636
200697_at	HK1	4,49239618	3,742201737	0,750194442
205672_at	XPA	2,605420261	1,855279162	0,750141099
207187_at	JAK3	0,458905032	-0,290694975	0,749600007
225617_at	ODF2	2,719381633	1,970106792	0,749274841
201193_at	IDH1	4,636598653	3,887370283	0,74922837
212633_at	UFL1	3,768156719	3,019022994	0,749133725
209299_x_at	PPIL2	1,246401134	0,497424714	0,748976419
239518_at	-	-1,06282519	-1,811780055	0,748954865
239781_at	LINC00545	-1,06282519	-1,811780055	0,748954865
1557502_at	PCCB	-1,06282519	-1,811780055	0,748954865
240916_x_at	-	-1,06282519	-1,811780055	0,748954865
205547_s_at	TAGLN	-1,06282519	-1,811780055	0,748954865
214520_at	FOXC2	-1,06282519	-1,811780055	0,748954865
216479_at	RPL21 /// RPL21P2	-1,06282519	-1,811780055	0,748954865
235654_at	TMEM218	-1,06282519	-1,811780055	0,748954865
240242_at	-	-1,06282519	-1,811780055	0,748954865
216894_x_at	CDKN1C	-1,06282519	-1,811780055	0,748954865
206541_at	KLKB1	-1,06282519	-1,811780055	0,748954865
217420_s_at	POLR2A	-1,06282519	-1,811780055	0,748954865
226086_at	SYT13	-1,06282519	-1,811780055	0,748954865

239615_at	SLC22A5	-1,06282519	-1,811780055	0,748954865
230255_at	GABRD	-1,06282519	-1,811780055	0,748954865
242912_at	POTEG /// POTEM	-1,06282519	-1,811780055	0,748954865
216906_at	ST14	-1,06282519	-1,811780055	0,748954865
1556414_at	LINC00515	-1,06282519	-1,811780055	0,748954865
240923_at	-	-1,06282519	-1,811780055	0,748954865
220073_s_at	PLEKHG6	-1,06282519	-1,811780055	0,748954865
241148_at	ACTG2	-1,06282519	-1,811780055	0,748954865
230643_at	WNT9A	-1,06282519	-1,811780055	0,748954865
244320_at	NHLRC2	-1,06282519	-1,811780055	0,748954865
234109_x_at	ONECUT3	-1,06282519	-1,811780055	0,748954865
1552617_a_at	RFWD2	3,639446351	2,890758424	0,748687927
231919_at	DBT	0,926325262	0,178191865	0,748133397
226036_x_at	CASP2	0,926325262	0,178191865	0,748133397
226241_s_at	MRPL52	4,69131479	3,943461539	0,74785325
55093_at	CHPF2	2,271816891	1,524221259	0,747595633
237412_at	-	0,425036312	-0,322340048	0,74737636
240117_at	FBN3	0,08619576	-0,661178575	0,747374334
235516_at	SEPSECS	0,08619576	-0,661178575	0,747374334
208257_x_at	PSG1	0,08619576	-0,661178575	0,747374334
241515_at	-	0,08619576	-0,661178575	0,747374334
233006_at	38231	0,08619576	-0,661178575	0,747374334
231203_at	LOC100505478	0,08619576	-0,661178575	0,747374334
205483_s_at	ISG15	4,263008992	3,515907973	0,747101018
218136_s_at	SLC25A37	0,735999505	-0,011001236	0,747000741
213681_at	CYHR1	0,735999505	-0,011001236	0,747000741
217336_at	-	2,427650693	1,680963921	0,746686773
209135_at	ASPH	1,561816652	0,815200271	0,746616382
227352_at	SWSAP1	1,225123479	0,479089184	0,746034295
231124_x_at	LY9	0,39105295	-0,354781582	0,745834532
205944_s_at	CLTCL1	0,39105295	-0,354781582	0,745834532
238995_at	SULT1A1	0,39105295	-0,354781582	0,745834532
242553_at	ABCC3	0,39105295	-0,354781582	0,745834532
204739_at	CENPC1	1,857989965	1,112548615	0,74544135
222990_at	UBQLN1	5,005833942	4,260674392	0,74515955
227981_at	CYB561D1	2,179708195	1,434743608	0,744964587
200915_x_at	KTN1	6,167785615	5,42323161	0,744554004
208638_at	PDIA6	6,986450748	6,242080554	0,744370194
224663_s_at	CFL2	0,900065578	0,155747281	0,744318297
207931_s_at	PFKFB2	0,707657549	-0,036543687	0,744201236
219346_at	LRFN3	0,707657549	-0,036543687	0,744201236
200608_s_at	RAD21	6,629665521	5,885590201	0,74407532
205959_at	MMP13	-1,212652659	-1,956681069	0,74402841
233862_at	-	-1,212652659	-1,956681069	0,74402841
222098_s_at	-	-1,212652659	-1,956681069	0,74402841
1569736_at	-	-1,212652659	-1,956681069	0,74402841
214366_s_at	ALOX5	-1,212652659	-1,956681069	0,74402841
1564783_x_at	OFCC1	-1,212652659	-1,956681069	0,74402841
1558819_at	-	-1,212652659	-1,956681069	0,74402841
1561891_at	-	-1,212652659	-1,956681069	0,74402841

233192_s_at	RUFY2	-1,212652659	-1,956681069	0,74402841
236983_at	TMC5	-1,212652659	-1,956681069	0,74402841
1561408_at	-	-1,212652659	-1,956681069	0,74402841
211117_x_at	ESR2	-1,212652659	-1,956681069	0,74402841
241007_at	-	-1,212652659	-1,956681069	0,74402841
237783_at	PLAC8L1	-1,212652659	-1,956681069	0,74402841
231217_at	-	-1,212652659	-1,956681069	0,74402841
238837_at	PHF8	-1,212652659	-1,956681069	0,74402841
216324_at	-	-1,212652659	-1,956681069	0,74402841
227848_at	PEBP4	-1,212652659	-1,956681069	0,74402841
229731_at	FOXS1	-1,212652659	-1,956681069	0,74402841
223366_at	ZNF704	-1,212652659	-1,956681069	0,74402841
233134_at	RPH3AL	-1,212652659	-1,956681069	0,74402841
230471_at	TMEM30B	-1,212652659	-1,956681069	0,74402841
213116_at	NEK3	1,640193838	0,896834102	0,743359736
225347_at	ARL8A	1,440265662	0,697221375	0,743044288
210581_x_at	PATZ1	0,045517965	-0,697182596	0,742700561
227028_s_at	DGCR2	0,045517965	-0,697182596	0,742700561
209968_s_at	NCAM1	0,045517965	-0,697182596	0,742700561
210431_at	ALPPL2	0,045517965	-0,697182596	0,742700561
238218_at	OOEP	0,045517965	-0,697182596	0,742700561
215158_s_at	DEDD	3,107338728	2,364676067	0,742662661
224121_x_at	PLEKHB2	0,355751005	-0,386896102	0,742647107
215387_x_at	-	0,355751005	-0,386896102	0,742647107
1552558_a_at	RAI1	0,355751005	-0,386896102	0,742647107
203368_at	CRELD1	0,355751005	-0,386896102	0,742647107
244569_at	C8orf37	1,910557155	1,167919222	0,742637933
213754_s_at	PAIP1	3,551632175	2,809015785	0,74261639
1555219_at	RGR	-0,230432956	-0,972893339	0,742460383
243017_at	LOC158572	-0,230432956	-0,972893339	0,742460383
1553544_at	GPR101	-0,230432956	-0,972893339	0,742460383
234784_at	-	-0,230432956	-0,972893339	0,742460383
215085_x_at	DLEC1	-0,230432956	-0,972893339	0,742460383
242887_at	KCMF1	-0,230432956	-0,972893339	0,742460383
230001_at	09.03.15	-0,230432956	-0,972893339	0,742460383
235131_at	RHOJ	-0,230432956	-0,972893339	0,742460383
240229_at	-	-0,230432956	-0,972893339	0,742460383
231355_at	CACNG8	-0,230432956	-0,972893339	0,742460383
201091_s_at	CBX3	5,720385266	4,978165242	0,742220024
218703_at	SEC22A	1,843966654	1,101780212	0,742186443
223012_at	UBXN6	3,102023286	2,359892448	0,742130839
231677_at	LOC100506165	-1,893506789	-2,635549085	0,742042296
208142_at	EDDM3A	-1,893506789	-2,635549085	0,742042296
1560063_a_at	-	-1,893506789	-2,635549085	0,742042296
240533_at	-	-1,893506789	-2,635549085	0,742042296
224043_s_at	UPB1	-1,893506789	-2,635549085	0,742042296
220976_s_at	KRTAP1-1	-1,893506789	-2,635549085	0,742042296
1562653_at	-	-1,893506789	-2,635549085	0,742042296
1552394_a_at	ENTHD1	-1,893506789	-2,635549085	0,742042296
243871_at	LOC100130476	-1,893506789	-2,635549085	0,742042296

214858_at	PP14571	-1,893506789	-2,635549085	0,742042296
229761_at	LOC440173	-1,893506789	-2,635549085	0,742042296
231898_x_at	SOX2-OT	-1,893506789	-2,635549085	0,742042296
1570042_a_at	ADAM9	-0,49284695	-1,234835326	0,741988376
215476_at	-	-0,49284695	-1,234835326	0,741988376
206844_at	FBP2	-0,49284695	-1,234835326	0,741988376
224424_x_at	ACTR3BP2 /// ACT	-0,49284695	-1,234835326	0,741988376
208888_s_at	NCOR2	-0,49284695	-1,234835326	0,741988376
206693_at	IL7	-0,49284695	-1,234835326	0,741988376
210085_s_at	ANXA9	-0,49284695	-1,234835326	0,741988376
1553156_at	LHX4	-0,49284695	-1,234835326	0,741988376
1553367_a_at	COX6B2	-0,49284695	-1,234835326	0,741988376
214541_s_at	QKI	-0,730013898	-1,471833228	0,741819329
229576_s_at	TBX3	-0,730013898	-1,471833228	0,741819329
207925_at	CST5	-0,730013898	-1,471833228	0,741819329
1556536_at	LOC729224	-0,730013898	-1,471833228	0,741819329
239430_at	IGFL1	-0,730013898	-1,471833228	0,741819329
243302_at	-	-0,730013898	-1,471833228	0,741819329
1557518_a_at	-	-0,730013898	-1,471833228	0,741819329
1552685_a_at	GRHL1	-0,730013898	-1,471833228	0,741819329
1555429_at	-	-0,730013898	-1,471833228	0,741819329
210642_at	CCIN	-0,730013898	-1,471833228	0,741819329
211497_x_at	NKX3-1	-0,730013898	-1,471833228	0,741819329
227863_at	IFITM10	-0,730013898	-1,471833228	0,741819329
206329_at	EXTL1	-0,730013898	-1,471833228	0,741819329
1569009_s_at	FAM201B /// LOC1	-0,730013898	-1,471833228	0,741819329
216312_at	ATP2B3	-0,924000698	-1,665791776	0,741791078
210366_at	SLCO1B1	-0,924000698	-1,665791776	0,741791078
1562759_at	-	-0,924000698	-1,665791776	0,741791078
216522_at	OR2B6	-0,924000698	-1,665791776	0,741791078
205385_at	MDM2	-0,924000698	-1,665791776	0,741791078
222274_at	ZDHHC8P1	-0,924000698	-1,665791776	0,741791078
222520_s_at	IFT57	-0,924000698	-1,665791776	0,741791078
216430_x_at	IGLV1-44	-0,924000698	-1,665791776	0,741791078
239100_x_at	PCNX	-0,924000698	-1,665791776	0,741791078
226216_at	INSR	-0,924000698	-1,665791776	0,741791078
206684_s_at	ATF7	-0,924000698	-1,665791776	0,741791078
244202_at	-	-0,924000698	-1,665791776	0,741791078
206996_x_at	CACNB1	-0,924000698	-1,665791776	0,741791078
204579_at	FGFR4	-0,924000698	-1,665791776	0,741791078
205870_at	BDKRB2	-0,924000698	-1,665791776	0,741791078
207894_s_at	TCL6	-0,924000698	-1,665791776	0,741791078
1562173_a_at	LINC00555	-0,924000698	-1,665791776	0,741791078
227463_at	ACE	-0,924000698	-1,665791776	0,741791078
209220_at	GPC3	-0,924000698	-1,665791776	0,741791078
206887_at	CCBP2	-0,924000698	-1,665791776	0,741791078
215574_at	-	-0,924000698	-1,665791776	0,741791078
227222_at	FBXO10	0,677745787	-0,063377083	0,74112287
219348_at	USE1	2,705643918	1,964670566	0,740973352
201898_s_at	UBE2A	5,100432184	4,359468791	0,740963392

230345_at	SEMA7A	1,047341799	0,306454867	0,740886932
1552461_at	FAM46D	-1,982324124	-2,723114724	0,740790601
1552458_at	MBD3L1	-1,982324124	-2,723114724	0,740790601
1556287_a_at	-	-1,982324124	-2,723114724	0,740790601
1553911_at	ZNF663	-1,982324124	-2,723114724	0,740790601
237526_at	-	-1,982324124	-2,723114724	0,740790601
1560864_at	-	-1,982324124	-2,723114724	0,740790601
228707_at	CLDN23	-1,982324124	-2,723114724	0,740790601
234741_at	ATP2B2	-1,982324124	-2,723114724	0,740790601
32069_at	N4BP1	3,456990418	2,716318964	0,740671454
200092_s_at	RPL37	8,28059851	7,539990917	0,740607593
231297_at	DOT1L	1,610353504	0,869824768	0,740528735
226283_at	POC1B	3,422378845	2,682180306	0,740198539
212228_s_at	COQ9	3,86465425	3,124666253	0,739987998
1554868_s_at	PCNP	4,674825018	3,934892246	0,739932772
203454_s_at	ATOX1	4,801719103	4,06178694	0,739932163
228751_at	CLK4	1,760255626	1,020514937	0,739740689
223257_at	G2E3	1,760255626	1,020514937	0,739740689
201186_at	LRPAP1	3,081633533	2,34250725	0,739126282
219003_s_at	MANEA	2,732422196	1,993309863	0,739112334
227672_at	C8orf73	1,307201325	0,568155442	0,739045883
212117_at	RHOQ	3,282538749	2,543655878	0,738882872
202299_s_at	HBXIP	6,286806415	5,548054033	0,738752382
213105_s_at	TSR3	1,948628481	1,209973561	0,738654919
217027_x_at	-	2,890618076	2,151985997	0,738632079
214323_s_at	UPF3A	4,806642953	4,068057231	0,738585723
203447_at	PSMD5	3,438642427	2,700070732	0,738571695
226951_at	C2orf49	2,460890032	1,722747778	0,738142254
1557964_at	EIF4G2	0,847529938	0,109458907	0,738071031
228356_at	ANKRD11	0,847529938	0,109458907	0,738071031
238417_at	PGM2L1	1,5944803	0,85655369	0,73792661
202489_s_at	FXYD3	0,648195588	-0,089635713	0,737831301
204496_at	STRN3	2,75889404	2,021531611	0,737362429
202536_at	CHMP2B	4,172855547	3,435695778	0,737159769
235088_at	C4orf46	1,936380191	1,199296812	0,737083379
201128_s_at	ACLY	5,482272318	4,745386178	0,73688614
212295_s_at	SLC7A1	5,521132257	4,784492053	0,736640204
224899_s_at	MAGT1	4,876721708	4,140132707	0,736589001
228306_at	CNIH4	1,995527416	1,259081178	0,736446238
222586_s_at	OSBPL11	2,50022283	1,76396664	0,736256191
205194_at	PSPH	2,966903803	2,23115548	0,735748323
1487_at	ESRRA	2,318008416	1,582277552	0,735730864
206587_at	CCT6B	2,042564225	1,306903969	0,735660255
203750_s_at	RARA	0,001114523	-0,734355396	0,735469919
1556613_s_at	DPY19L4	1,802733148	1,067365565	0,735367583
1559062_at	-	-0,280273599	-1,015398016	0,735124417
221656_s_at	ARHGEF10L	-0,280273599	-1,015398016	0,735124417
222952_s_at	TLR7	-0,280273599	-1,015398016	0,735124417
244875_at	ASMTL-AS1	-0,280273599	-1,015398016	0,735124417
241795_at	RHEB	0,618277321	-0,116767475	0,735044796

214002_at	MYL6	0,618277321	-0,116767475	0,735044796
201469_s_at	SHC1	0,618277321	-0,116767475	0,735044796
201908_at	DVL3	2,590269697	1,855279162	0,734990536
220341_s_at	C5orf45	1,000459215	0,265475485	0,73498373
201623_s_at	DARS	7,323486612	6,588588284	0,734898328
212019_at	RSL1D1	0,820160788	0,085534992	0,734625795
202197_at	MTMR3	1,730929079	0,996453274	0,734475805
203061_s_at	MDC1	1,857989965	1,123905343	0,734084623
1554014_at	CHD2	2,778527892	2,044580955	0,733946937
224573_at	RNASEK /// RNASE	6,685549754	5,951621401	0,733928353
1554423_a_at	FBXO7	4,849052846	4,11524077	0,733812076
214945_at	FAM153A /// FAM	-1,802716385	-2,536339282	0,733622896
203824_at	TSPAN8	-1,802716385	-2,536339282	0,733622896
210533_at	MSH4	-1,802716385	-2,536339282	0,733622896
238236_at	-	-1,802716385	-2,536339282	0,733622896
240447_at	-	-1,802716385	-2,536339282	0,733622896
211154_at	THPO	-1,802716385	-2,536339282	0,733622896
206457_s_at	DIO1	-1,802716385	-2,536339282	0,733622896
1570601_at	-	-1,802716385	-2,536339282	0,733622896
1555643_s_at	LILRA5	-1,802716385	-2,536339282	0,733622896
241449_at	-	-1,802716385	-2,536339282	0,733622896
220791_x_at	SCN11A	-1,802716385	-2,536339282	0,733622896
1555580_at	-	-1,802716385	-2,536339282	0,733622896
204389_at	MAOA	-1,802716385	-2,536339282	0,733622896
206409_at	TIAM1	0,282358733	-0,45121326	0,733571993
237400_at	-	0,282358733	-0,45121326	0,733571993
1559748_at	ADAMTSL3	0,282358733	-0,45121326	0,733571993
228366_at	-	1,476175815	0,742671819	0,733503996
224777_s_at	PAFAH1B2	4,477030407	3,743575485	0,733454922
216966_at	ITGA2B	-1,452713826	-2,186086603	0,733372777
213960_at	NTRK3	-1,452713826	-2,186086603	0,733372777
234652_at	-	-1,452713826	-2,186086603	0,733372777
1560971_a_at	-	-1,452713826	-2,186086603	0,733372777
1561000_at	-	-1,452713826	-2,186086603	0,733372777
1559901_s_at	LINC00478	-1,452713826	-2,186086603	0,733372777
211616_s_at	HTR2A	-1,452713826	-2,186086603	0,733372777
238107_at	-	-1,452713826	-2,186086603	0,733372777
244671_at	-	-1,452713826	-2,186086603	0,733372777
211116_at	SLC9A2	-1,452713826	-2,186086603	0,733372777
237519_at	LOC100506880	-1,452713826	-2,186086603	0,733372777
217563_at	CLOCK	-1,452713826	-2,186086603	0,733372777
1555322_at	PP2672	-1,452713826	-2,186086603	0,733372777
202376_at	SERPINA3	-1,452713826	-2,186086603	0,733372777
221714_s_at	RRN3P1	-1,452713826	-2,186086603	0,733372777
204596_s_at	STC1	-1,452713826	-2,186086603	0,733372777
1561940_at	LOC100128843	-1,452713826	-2,186086603	0,733372777
1564778_at	-	-1,452713826	-2,186086603	0,733372777
1560030_at	LOC283692	-1,452713826	-2,186086603	0,733372777
243002_at	-	-1,452713826	-2,186086603	0,733372777
232979_at	MIR10A	-1,452713826	-2,186086603	0,733372777

203498_at	RCAN2	-1,452713826	-2,186086603	0,733372777
226875_at	DOCK11	4,731106344	3,997758123	0,733348221
1559776_at	-	2,031158928	1,298349073	0,732809854
210622_x_at	CDK10	0,58788177	-0,144765583	0,732647353
208884_s_at	UBR5	3,816747242	3,084123325	0,732623916
228190_at	ATG4C	2,575362572	1,842852096	0,732510476
226598_s_at	GTPBP5	1,561816652	0,829309537	0,732507116
219065_s_at	DPY30 /// MEMO1	5,385736849	4,653335581	0,732401268
1570207_at	FRRS1	-2,074223907	-2,8064286	0,732204693
1558691_a_at	DOCK4	-2,074223907	-2,8064286	0,732204693
239722_at	C5orf49	-2,074223907	-2,8064286	0,732204693
1566500_at	-	-2,074223907	-2,8064286	0,732204693
211736_at	SP2	-2,074223907	-2,8064286	0,732204693
233868_x_at	ADAM33	1,716003175	0,98381571	0,732187465
1561673_at	-	-1,537851782	-2,27001641	0,732164628
205307_s_at	KMO	-1,537851782	-2,27001641	0,732164628
1562878_at	-	-1,537851782	-2,27001641	0,732164628
1566948_at	-	-1,537851782	-2,27001641	0,732164628
236756_at	CENPVP1 /// CENP	-1,537851782	-2,27001641	0,732164628
215164_at	-	-1,537851782	-2,27001641	0,732164628
216367_at	COL4A3	-1,537851782	-2,27001641	0,732164628
1562251_a_at	LOC574538	-1,537851782	-2,27001641	0,732164628
1562631_at	TEX26-AS1	-1,537851782	-2,27001641	0,732164628
1570100_at	-	-1,537851782	-2,27001641	0,732164628
216622_at	LAMB4	-1,537851782	-2,27001641	0,732164628
1562524_at	-	-1,537851782	-2,27001641	0,732164628
220286_at	MTMR10	-1,537851782	-2,27001641	0,732164628
242394_at	CNKSR3	-1,537851782	-2,27001641	0,732164628
234876_at	-	-1,537851782	-2,27001641	0,732164628
233470_at	PTPN5	-1,537851782	-2,27001641	0,732164628
1556894_at	NT5DC2	-1,537851782	-2,27001641	0,732164628
243510_at	LOC100506418	-1,537851782	-2,27001641	0,732164628
208674_x_at	DDOST	5,713645047	4,981932568	0,731712479
218225_at	ECSIT	2,074841895	1,343200523	0,731641372
222441_x_at	SLMO2	4,806642953	4,075090115	0,731552838
1560227_at	GDPD1	-0,551284523	-1,282520722	0,731236199
231567_s_at	CCDC62	-0,551284523	-1,282520722	0,731236199
213821_s_at	IDS	-0,551284523	-1,282520722	0,731236199
221793_at	DOCK6	-0,551284523	-1,282520722	0,731236199
207228_at	PRKACG	-0,551284523	-1,282520722	0,731236199
224475_at	-	-0,551284523	-1,282520722	0,731236199
1569793_at	SLC25A18	-0,551284523	-1,282520722	0,731236199
209862_s_at	CEP57	3,370643409	2,639836674	0,730806736
213510_x_at	USP32P2	3,498020785	2,767331883	0,730688901
205707_at	IL17RA	2,285848435	1,555165465	0,73068297
203333_at	KIFAP3	4,431230066	3,70056079	0,730669276
204014_at	DUSP4	5,610806763	4,880350779	0,730455983
213610_s_at	KLHL23 /// PHOSPI	3,735523399	3,005076416	0,730446984
224661_at	PIGY	3,233229368	2,503049671	0,730179697
221162_at	HHLA1	0,791908897	0,061773582	0,730135315

232287_at	PGBD3	0,791908897	0,061773582	0,730135315
201987_at	MED13	4,597104084	3,867024218	0,730079866
201945_at	FURIN	0,95222556	0,222432814	0,729792746
226027_at	SWI5	3,107338728	2,37755543	0,729783298
243927_x_at	KIAA1429	1,640193838	0,910483921	0,729709918
228558_at	C14orf80	1,640193838	0,910483921	0,729709918
213982_s_at	RABGAP1L	4,718186291	3,988637612	0,729548679
221830_at	RAP2A	3,628556015	2,899658146	0,728897869
241859_at	PLCL1	-0,791294935	-1,520022123	0,728727188
216063_at	HBBP1	-0,791294935	-1,520022123	0,728727188
206154_at	RLBP1	-0,791294935	-1,520022123	0,728727188
220090_at	CRNN	-0,791294935	-1,520022123	0,728727188
1553209_at	RNFT2	-0,791294935	-1,520022123	0,728727188
232689_at	LOC284561	-0,791294935	-1,520022123	0,728727188
228815_s_at	REPIN1	-0,791294935	-1,520022123	0,728727188
207680_x_at	PAX3	-0,791294935	-1,520022123	0,728727188
229257_at	TNRC18	-0,791294935	-1,520022123	0,728727188
232042_at	TTYH2	-0,791294935	-1,520022123	0,728727188
207998_s_at	CACNA1D	-0,791294935	-1,520022123	0,728727188
215276_at	WFDC8	-0,791294935	-1,520022123	0,728727188
218942_at	PIP4K2C	3,071179046	2,34250725	0,728671796
206413_s_at	TCL1B	0,556428218	-0,172194225	0,728622443
215822_x_at	MYT1	0,556428218	-0,172194225	0,728622443
213393_at	MFSD9	0,556428218	-0,172194225	0,728622443
228904_at	HOXB3	2,053550345	1,324949741	0,728600604
227022_at	GNPDA2	2,920736393	2,192329358	0,728407035
210734_x_at	MAX	1,440265662	0,711934445	0,728331217
233568_x_at	CWF19L1	2,21987062	1,491644074	0,728226547
201031_s_at	HNRNPH1	6,410459479	5,682526267	0,727933212
222809_x_at	CCDC85C	1,225123479	0,497424714	0,727698765
201089_at	ATP6V1B2	3,584942056	2,857256661	0,727685395
1568754_at	-	-0,043925712	-0,771340337	0,727414625
231860_at	BRWD1	-0,043925712	-0,771340337	0,727414625
207458_at	RHPN1-AS1	-0,043925712	-0,771340337	0,727414625
234417_at	-	-0,043925712	-0,771340337	0,727414625
220600_at	C3orf75	-0,043925712	-0,771340337	0,727414625
1552281_at	SLC39A5	-0,043925712	-0,771340337	0,727414625
223160_s_at	SMEK1	3,374552989	2,64735888	0,727194109
224698_at	ESYT2	5,210892881	4,483700773	0,727192108
217872_at	PIH1D1	4,301645306	3,5744736	0,727171706
230721_at	C16orf52	2,436165655	1,709047448	0,727118207
1556913_a_at	GIT2	-1,369772723	-2,09678811	0,727015387
1554232_a_at	-	-1,369772723	-2,09678811	0,727015387
1570623_at	-	-1,369772723	-2,09678811	0,727015387
1554302_s_at	LHFPL3-AS1	-1,369772723	-2,09678811	0,727015387
211353_at	LRIT1	-1,369772723	-2,09678811	0,727015387
240266_at	-	-1,369772723	-2,09678811	0,727015387
231762_at	FGF10	-1,369772723	-2,09678811	0,727015387
243472_at	-	-1,369772723	-2,09678811	0,727015387
233382_at	-	-1,369772723	-2,09678811	0,727015387

221278_at	HOXB8	-1,369772723	-2,09678811	0,727015387
209742_s_at	MYL2	-1,369772723	-2,09678811	0,727015387
231549_at	C1orf158	-1,369772723	-2,09678811	0,727015387
241153_at	-	-1,369772723	-2,09678811	0,727015387
236758_at	LOC100129407 ///	-1,369772723	-2,09678811	0,727015387
214584_x_at	ACACB	-1,369772723	-2,09678811	0,727015387
221171_at	CCDC30	-1,369772723	-2,09678811	0,727015387
236748_at	RASGEF1C	-1,369772723	-2,09678811	0,727015387
1563031_at	-	-1,369772723	-2,09678811	0,727015387
214821_at	SLC25A4	-1,369772723	-2,09678811	0,727015387
234482_at	-	-1,369772723	-2,09678811	0,727015387
215081_at	KIAA1024	-1,369772723	-2,09678811	0,727015387
238268_at	-	-1,369772723	-2,09678811	0,727015387
210397_at	DEFB1	-1,369772723	-2,09678811	0,727015387
1561358_at	TXLNA	-1,369772723	-2,09678811	0,727015387
217096_at	PCLO	-1,369772723	-2,09678811	0,727015387
223993_s_at	CNIH4	5,490289402	4,763290007	0,726999394
214281_s_at	RCHY1	3,366614157	2,639836674	0,726777483
228870_at	FAM84B	0,524545436	-0,201789721	0,726335157
211724_x_at	MIOS	2,685337919	1,9592579	0,726080019
210278_s_at	AP4S1	1,326463531	0,600385088	0,726078443
236403_at	THAP7-AS1	0,20740896	-0,518232988	0,725641949
217630_at	ANGEL2	0,20740896	-0,518232988	0,725641949
242476_at	-	0,20740896	-0,518232988	0,725641949
205536_at	VAV2	0,20740896	-0,518232988	0,725641949
224700_at	STT3B	5,365205123	4,639627194	0,725577928
218848_at	THOC6	1,871521385	1,146010634	0,725510751
1570574_at	-	-1,624955693	-2,350444759	0,725489066
1552489_s_at	MPP4	-1,624955693	-2,350444759	0,725489066
232108_at	SCRN3	-1,624955693	-2,350444759	0,725489066
1563884_at	-	-1,624955693	-2,350444759	0,725489066
1552544_at	SERPINA12	-1,624955693	-2,350444759	0,725489066
216573_at	IGLV1-44	-1,624955693	-2,350444759	0,725489066
237381_at	-	-1,624955693	-2,350444759	0,725489066
222066_at	EPB41L1	-1,624955693	-2,350444759	0,725489066
1559293_x_at	LINC00032	-1,624955693	-2,350444759	0,725489066
237505_at	-	-1,624955693	-2,350444759	0,725489066
220166_at	CNNM1	-1,624955693	-2,350444759	0,725489066
241502_x_at	-	-1,624955693	-2,350444759	0,725489066
208131_s_at	PTGIS	-1,624955693	-2,350444759	0,725489066
215325_x_at	C19orf26	-1,624955693	-2,350444759	0,725489066
244715_at	-	-1,624955693	-2,350444759	0,725489066
1556607_at	EHD4	-1,624955693	-2,350444759	0,725489066
1555286_at	CDKL1	-1,624955693	-2,350444759	0,725489066
225306_s_at	SLC25A29	2,159837409	1,434743608	0,725093801
234894_at	ITIH6	-1,137208348	-1,862269442	0,725061093
230909_at	COX18	-1,137208348	-1,862269442	0,725061093
216788_at	-	-1,137208348	-1,862269442	0,725061093
220361_at	IQCH	-1,137208348	-1,862269442	0,725061093
235669_at	AGXT2L2	-1,137208348	-1,862269442	0,725061093

243343_at	-	-1,137208348	-1,862269442	0,725061093
242945_at	FAM20A	-1,137208348	-1,862269442	0,725061093
1553682_at	FBXL14	-1,137208348	-1,862269442	0,725061093
243905_at	LOC387895	-1,137208348	-1,862269442	0,725061093
210535_at	B9D1	-1,137208348	-1,862269442	0,725061093
207884_at	GUCY2D	-1,137208348	-1,862269442	0,725061093
233556_at	CRYBB2P1	-1,137208348	-1,862269442	0,725061093
211305_x_at	FCAR	-1,137208348	-1,862269442	0,725061093
210159_s_at	TRIM31	-1,137208348	-1,862269442	0,725061093
201109_s_at	THBS1	-1,137208348	-1,862269442	0,725061093
217762_s_at	RAB31	-1,137208348	-1,862269442	0,725061093
228061_at	CCDC126	1,745502609	1,020514937	0,724987672
227094_at	DHTKD1	3,076375717	2,351470274	0,724905444
1555680_a_at	SMOX	-0,332405896	-1,057301851	0,724895955
211863_x_at	HFE	-0,332405896	-1,057301851	0,724895955
230221_at	ABHD16A	-0,332405896	-1,057301851	0,724895955
235473_at	-	-0,332405896	-1,057301851	0,724895955
212948_at	CAMTA2	1,5944803	0,869824768	0,724655532
212669_at	CAMK2G	1,510970097	0,786391549	0,724578548
201825_s_at	SCCPDH	4,991134457	4,26677677	0,724357688
214356_s_at	KIAA0368	4,339857197	3,615671864	0,724185333
212715_s_at	MICAL3	1,802733148	1,078619386	0,724113762
226910_at	COMMD2	3,840618883	3,117017558	0,723601326
242989_at	STRN	1,307201325	0,583607503	0,723593822
216051_x_at	-	1,307201325	0,583607503	0,723593822
223015_at	EIF2A	5,925923524	5,202716986	0,723206538
243258_at	KIAA0664	0,491906512	-0,231045907	0,722952418
219321_at	MPP5	0,491906512	-0,231045907	0,722952418
214755_at	UAP1L1	0,735999505	0,013190398	0,722809107
204287_at	SYNGR1	0,735999505	0,013190398	0,722809107
1554835_a_at	B3GNT5 /// LOC10	-0,088974936	-0,811769547	0,722794611
1555590_a_at	GATA1	-0,088974936	-0,811769547	0,722794611
229037_at	TRIM69	-0,088974936	-0,811769547	0,722794611
227358_at	ZBTB46	-0,088974936	-0,811769547	0,722794611
243326_at	-	-0,088974936	-0,811769547	0,722794611
218389_s_at	APH1A	3,489810047	2,767331883	0,722478164
212703_at	TLN2	0,167727503	-0,554696666	0,72242417
219407_s_at	LAMC3	0,167727503	-0,554696666	0,72242417
232220_at	S100A7A	0,167727503	-0,554696666	0,72242417
217773_s_at	NDUFA4	6,142790811	5,42038983	0,722400981
223538_at	SERF1A /// SERF1B	3,77412549	3,052211048	0,721914442
203028_s_at	CYBA	6,527987595	5,806375144	0,72161245
201418_s_at	SOX4	3,132805465	2,411206536	0,721598929
241977_s_at	RAB3C	-0,994186487	-1,715576125	0,721389638
235490_at	TMEM107	-0,994186487	-1,715576125	0,721389638
238772_at	ZNF207	-0,994186487	-1,715576125	0,721389638
231910_at	NUDT14	-0,994186487	-1,715576125	0,721389638
231430_at	FAM181B	-0,994186487	-1,715576125	0,721389638
240252_at	-	-0,994186487	-1,715576125	0,721389638
1561733_at	-	-0,994186487	-1,715576125	0,721389638

203982_s_at	ABCD4	-0,994186487	-1,715576125	0,721389638
220479_at	CPS1-IT1	-0,994186487	-1,715576125	0,721389638
1560636_a_at	HSPC081	-0,994186487	-1,715576125	0,721389638
1566037_at	DGCR7	-0,994186487	-1,715576125	0,721389638
233062_at	-	-0,994186487	-1,715576125	0,721389638
216230_x_at	SMPD1	-0,994186487	-1,715576125	0,721389638
210080_x_at	CELA3A	-0,994186487	-1,715576125	0,721389638
210934_at	BLK	-0,994186487	-1,715576125	0,721389638
220430_at	FAM110D	-0,994186487	-1,715576125	0,721389638
214127_s_at	SRRT	-0,994186487	-1,715576125	0,721389638
233447_at	PSMG4	-0,994186487	-1,715576125	0,721389638
208483_x_at	KRT33A	-0,994186487	-1,715576125	0,721389638
221179_at	-	-0,994186487	-1,715576125	0,721389638
206223_at	LMTK2	-0,994186487	-1,715576125	0,721389638
238747_at	CACNA1E	-0,994186487	-1,715576125	0,721389638
1556554_at	TRIM50	-0,994186487	-1,715576125	0,721389638
233135_at	LOC100506527	-0,994186487	-1,715576125	0,721389638
201506_at	LOC100652886 ///	-0,994186487	-1,715576125	0,721389638
240716_at	TTC23	-0,994186487	-1,715576125	0,721389638
213567_at	KPNA4	2,771643417	2,050286491	0,721356926
227200_at	ETV3	3,843767728	3,122419381	0,721348347
203075_at	SMAD2	3,861345795	3,141266133	0,720079663
203339_at	SLC25A12	1,843966654	1,123905343	0,720061312
208310_s_at	CCZ1 /// CCZ1B	4,478885385	3,759583763	0,719301623
222702_x_at	CRIPT	4,204964809	3,485746794	0,719218016
217812_at	YTHDF2	4,983489079	4,264419981	0,719069098
228628_at	SRGAP2C	2,118167848	1,399283506	0,718884343
234797_at	-	0,707657549	-0,011001236	0,718658784
1555007_s_at	WDR66	-0,609040214	-1,32740143	0,718361216
214782_at	CTTN	-0,609040214	-1,32740143	0,718361216
235477_at	-	-0,609040214	-1,32740143	0,718361216
237541_at	-	-0,609040214	-1,32740143	0,718361216
241318_at	-	-0,609040214	-1,32740143	0,718361216
1570631_at	-	-0,609040214	-1,32740143	0,718361216
230582_at	HECA	-0,609040214	-1,32740143	0,718361216
221955_at	-	-0,609040214	-1,32740143	0,718361216
240875_at	CTC1	-0,609040214	-1,32740143	0,718361216
231025_at	SYT7	-0,609040214	-1,32740143	0,718361216
214392_at	IRGC	-0,609040214	-1,32740143	0,718361216
221124_s_at	VSX1	-0,609040214	-1,32740143	0,718361216
239261_s_at	CORIN	-0,609040214	-1,32740143	0,718361216
1553660_at	HUS1B	-0,609040214	-1,32740143	0,718361216
226852_at	MTA3	-0,609040214	-1,32740143	0,718361216
223137_at	ZDHHC4	-0,609040214	-1,32740143	0,718361216
225546_at	EEF2K	3,562907192	2,844606929	0,718300263
203460_s_at	PSEN1	3,142467702	2,424202743	0,718264959
212170_at	RBM12	2,16969527	1,451507543	0,718187727
205107_s_at	EFNA4	0,873789652	0,155747281	0,718042371
223274_at	TCF19	3,261582786	2,543655878	0,717926909
211703_s_at	TM2D1	4,019763292	3,301884701	0,71787859

230206_at	DOCK5	1,701542258	0,98381571	0,717726548
1554016_a_at	C16orf57	2,107136847	1,390070295	0,717066551
226183_at	GSK3B	1,640193838	0,923203038	0,7169908
218079_s_at	GGNBP2	4,158437147	3,44162151	0,716815637
237611_at	-	-0,384193355	-1,100943374	0,716750019
244642_at	-	-0,384193355	-1,100943374	0,716750019
211384_s_at	CASR	-0,384193355	-1,100943374	0,716750019
237653_at	-	-0,384193355	-1,100943374	0,716750019
242062_at	SAMD8	-0,384193355	-1,100943374	0,716750019
215677_s_at	BRF1	0,127732831	-0,589001171	0,716734002
236686_at	-	0,127732831	-0,589001171	0,716734002
206725_x_at	BMP1	0,127732831	-0,589001171	0,716734002
1553954_at	ALG14	2,966903803	2,250228409	0,716675394
207777_s_at	SP140	2,692288672	1,975797592	0,71649108
226866_at	ESCO1	3,628556015	2,912181979	0,716374036
213239_at	PIBF1	2,878891456	2,162541631	0,716349825
206509_at	PIP	0,425036312	-0,290694975	0,715731288
1556445_at	-	0,425036312	-0,290694975	0,715731288
1556248_at	FLJ33065	0,425036312	-0,290694975	0,715731288
203517_at	MTX2	4,37922506	3,663499786	0,715725274
205751_at	SH3GL2	-1,712732543	-2,4284334	0,715700857
210297_s_at	MSMB	-1,712732543	-2,4284334	0,715700857
207659_s_at	MOBP	-1,712732543	-2,4284334	0,715700857
206167_s_at	ARHGAP6	-1,712732543	-2,4284334	0,715700857
231031_at	KGFLP2	-1,712732543	-2,4284334	0,715700857
227398_s_at	MIDN	-1,712732543	-2,4284334	0,715700857
222379_at	KCNE4	-1,712732543	-2,4284334	0,715700857
206221_at	RASA3	-1,712732543	-2,4284334	0,715700857
1564144_at	-	-1,712732543	-2,4284334	0,715700857
214297_at	CSPG4	-1,712732543	-2,4284334	0,715700857
227194_at	FAM3B	-1,712732543	-2,4284334	0,715700857
1559540_at	LOC100506526	-1,712732543	-2,4284334	0,715700857
201058_s_at	MYL9	-1,712732543	-2,4284334	0,715700857
230732_s_at	MAPK4	-1,712732543	-2,4284334	0,715700857
231410_at	-	-1,712732543	-2,4284334	0,715700857
242085_at	-	-1,712732543	-2,4284334	0,715700857
236129_at	GALNT5	-1,712732543	-2,4284334	0,715700857
235032_at	DNAJC21	3,282538749	2,56688207	0,715656679
200949_x_at	RPS20 /// SNORD5	8,180220146	7,464683027	0,715537119
240904_at	-	-1,289805289	-2,005029581	0,715224292
235270_at	ZNF397	-1,289805289	-2,005029581	0,715224292
230299_s_at	WNT5B	-1,289805289	-2,005029581	0,715224292
241302_at	-	-1,289805289	-2,005029581	0,715224292
239668_at	-	-1,289805289	-2,005029581	0,715224292
236245_at	ODF3L1	-1,289805289	-2,005029581	0,715224292
1554041_at	TMEM239	-1,289805289	-2,005029581	0,715224292
243437_at	GCC1	-1,289805289	-2,005029581	0,715224292
221455_s_at	WNT3	-1,289805289	-2,005029581	0,715224292
205829_at	HSD17B1	-1,289805289	-2,005029581	0,715224292
211224_s_at	ABCB11	-1,289805289	-2,005029581	0,715224292

223786_at	CHST6	-1,289805289	-2,005029581	0,715224292
1563255_at	LOC100506733	-1,289805289	-2,005029581	0,715224292
237497_at	-	-1,289805289	-2,005029581	0,715224292
241903_at	-	-1,289805289	-2,005029581	0,715224292
208272_at	RANBP3	-1,289805289	-2,005029581	0,715224292
231753_s_at	PCDHGC4	-1,289805289	-2,005029581	0,715224292
229547_s_at	WNK2	-1,289805289	-2,005029581	0,715224292
212598_at	WDFY3	-1,289805289	-2,005029581	0,715224292
204643_s_at	ENOX2	1,625482993	0,910483921	0,714999073
214339_s_at	MAP4K1	1,871521385	1,156599976	0,714921409
205812_s_at	TMED9	5,589081108	4,874253211	0,714827897
202883_s_at	PPP2R1B	2,955426542	2,24081821	0,714608331
201990_s_at	CREBL2	2,582572508	1,868130962	0,714441546
218916_at	ZNF768	1,364555912	0,65030602	0,714249892
238724_at	-	-0,135065865	-0,84928999	0,714224125
225495_x_at	GADD45GIP1	-0,135065865	-0,84928999	0,714224125
1554128_at	ADIG	-0,135065865	-0,84928999	0,714224125
211143_x_at	NR4A1	-0,135065865	-0,84928999	0,714224125
213846_at	COX7C	4,584332287	3,870131307	0,714200981
212215_at	PREPL	3,86465425	3,150841611	0,713812639
217768_at	C14orf166	7,095959579	6,382238195	0,713721384
225157_at	MLXIP	1,610353504	0,896834102	0,713519402
214471_x_at	LHB	0,39105295	-0,322340048	0,713392998
200090_at	FNTA	6,366313939	5,65298827	0,713325669
227441_s_at	ANKS1B	-1,982324124	-2,695640852	0,713316728
208554_at	POU4F3	-1,982324124	-2,695640852	0,713316728
213720_s_at	SMARCA4	3,664722055	2,951484533	0,713237521
212652_s_at	SNX4	4,570504669	3,858181176	0,712323493
1563166_at	-	-0,856259172	-1,568385657	0,712126485
1556090_at	-	-0,856259172	-1,568385657	0,712126485
208049_s_at	TACR1	-0,856259172	-1,568385657	0,712126485
1552405_at	NLRP5	-0,856259172	-1,568385657	0,712126485
217474_at	-	-0,856259172	-1,568385657	0,712126485
241582_at	-	-0,856259172	-1,568385657	0,712126485
1552301_a_at	CORO6	-0,856259172	-1,568385657	0,712126485
237178_at	LOC100506414	-0,856259172	-1,568385657	0,712126485
202897_at	SIRPA	-0,856259172	-1,568385657	0,712126485
231073_at	C1orf168	-0,856259172	-1,568385657	0,712126485
1564463_at	LOC100499221	-0,856259172	-1,568385657	0,712126485
207742_s_at	NR6A1	-0,856259172	-1,568385657	0,712126485
216606_x_at	LYPLA2	1,802733148	1,090640407	0,712092741
228972_at	PITPNA-AS1	3,505636113	2,793634788	0,712001325
226015_at	ZNF12	3,190097062	2,478123977	0,711973084
204900_x_at	SAP30	4,962294442	4,250487736	0,711806706
226752_at	FAM174A	2,849132494	2,137342905	0,71178959
215373_x_at	-	2,312738346	1,601076277	0,711662069
214167_s_at	RPLP0	8,027645176	7,316154454	0,711490721
226813_at	NTPCR	3,243218364	2,531843788	0,711374576
237028_at	ENO1-AS1	0,820160788	0,109458907	0,710701881
221665_s_at	EPS8L1	0,355751005	-0,354781582	0,710532587

229445_at	CYBA	0,355751005	-0,354781582	0,710532587
213963_s_at	SAP30	0,355751005	-0,354781582	0,710532587
202064_s_at	SEL1L	1,730929079	1,020514937	0,710414142
227693_at	WDR20	2,41945542	1,709047448	0,710407972
1560197_at	ZC2HC1A	-1,893506789	-2,603471267	0,709964478
237313_at	LOC100507410	-1,893506789	-2,603471267	0,709964478
1562922_at	-	-1,893506789	-2,603471267	0,709964478
214346_at	C1QL1	-1,893506789	-2,603471267	0,709964478
1565807_at	-	-1,893506789	-2,603471267	0,709964478
1556916_a_at	-	-1,893506789	-2,603471267	0,709964478
243330_at	-	-1,893506789	-2,603471267	0,709964478
244620_at	SLC8A1-AS1	-1,893506789	-2,603471267	0,709964478
222618_at	SMU1	2,209870035	1,500059916	0,709810119
202076_at	BIRC2	5,717593136	5,007828519	0,709764617
220395_at	DNAJA4	1,225123479	0,515457602	0,709665877
213922_at	TTBK2	1,225123479	0,515457602	0,709665877
225529_at	ACAP3	1,421391637	0,711934445	0,709457192
230670_at	IGSF10	0,08619576	-0,623254098	0,709449858
233000_x_at	DPH3P1	0,08619576	-0,623254098	0,709449858
229668_at	-	0,08619576	-0,623254098	0,709449858
224885_s_at	KRTCAP2	5,900685777	5,191464463	0,709221314
227002_at	FAM78A	3,521615383	2,812515887	0,709099496
222637_at	COMMD10	3,322661262	2,613615641	0,709045621
1562378_s_at	PROM2	1,898032047	1,189078962	0,708953085
56197_at	C17orf61-PLSCR3 /	4,524468243	3,815535805	0,708932438
226230_at	SMEK2	4,175145566	3,466845432	0,708300134
208076_at	HIST1H4A /// HIST	-0,180990326	-0,889222211	0,708231885
236145_at	-	-0,180990326	-0,889222211	0,708231885
1553540_a_at	SLC29A2	-0,180990326	-0,889222211	0,708231885
239422_at	GPC2	-0,180990326	-0,889222211	0,708231885
205424_at	TBKBP1	-0,180990326	-0,889222211	0,708231885
1557169_x_at	HCG11	1,65554524	0,947673886	0,707871354
227606_s_at	STAMBPL1	2,582572508	1,874839058	0,70773345
208450_at	LGALS2	-0,437449947	-1,145057014	0,707607067
231068_at	SLC47A2	-0,437449947	-1,145057014	0,707607067
1556822_s_at	ZNF837	-0,437449947	-1,145057014	0,707607067
221015_s_at	CDADC1	-0,437449947	-1,145057014	0,707607067
220849_at	LOC79999	-0,437449947	-1,145057014	0,707607067
231309_at	GNA12	-0,437449947	-1,145057014	0,707607067
233519_at	-	-0,437449947	-1,145057014	0,707607067
219883_at	C11orf20 /// KCNK	-0,437449947	-1,145057014	0,707607067
209657_s_at	HSF2	3,414594082	2,707013102	0,707580979
218850_s_at	LIMD1	0,95222556	0,244646697	0,707578863
203157_s_at	GLS	0,95222556	0,244646697	0,707578863
219487_at	BBS10	1,493647653	0,786391549	0,707256104
227324_at	ADCK4	1,493647653	0,786391549	0,707256104
225903_at	PIGU	3,265594257	2,558652146	0,706942111
235587_at	LOC202781	2,966903803	2,260052727	0,706851076
225988_at	HERC4	2,778527892	2,071691486	0,706836406
237826_at	-	0,045517965	-0,661178575	0,706696539

241386_at	LOC100506691	0,045517965	-0,661178575	0,706696539
209570_s_at	D4S234E	0,045517965	-0,661178575	0,706696539
1560477_a_at	SAMD11	0,045517965	-0,661178575	0,706696539
1564876_s_at	FOXP2	0,045517965	-0,661178575	0,706696539
234158_at	-	0,045517965	-0,661178575	0,706696539
206769_at	TMSB4Y	0,045517965	-0,661178575	0,706696539
227942_s_at	CRIPT	4,368395955	3,66182964	0,706566315
208776_at	PSMD11	2,908690075	2,20251271	0,706177365
1569040_s_at	ANKRD36BP2	-2,074223907	-2,780225148	0,706001241
1562880_at	LOC100288238	-2,074223907	-2,780225148	0,706001241
219607_s_at	MS4A4A	-2,074223907	-2,780225148	0,706001241
1557609_s_at	TBC1D12	-2,074223907	-2,780225148	0,706001241
225319_s_at	FAM104A	4,885515979	4,179683441	0,705832538
227111_at	ZBTB34	2,804738618	2,099079406	0,705659212
224683_at	FBXO18	2,276736537	1,571112806	0,705623731
226594_at	ENTPD5	1,561816652	0,85655369	0,705262963
242377_x_at	THUMPD3	0,58788177	-0,116767475	0,704649245
204360_s_at	NAGLU	0,58788177	-0,116767475	0,704649245
228532_at	C1orf162	2,725848647	2,021531611	0,704317036
227351_at	C16orf52	1,640193838	0,936232181	0,703961657
227820_at	TBC1D25	0,926325262	0,222432814	0,703892448
229293_at	-	0,926325262	0,222432814	0,703892448
1553136_at	KCTD18	1,182925501	0,479089184	0,703836317
227988_s_at	VPS13A	1,182925501	0,479089184	0,703836317
209788_s_at	ERAP1	4,2463808	3,542765782	0,703615018
214665_s_at	CHP1	4,283600482	3,580129335	0,703471147
205120_s_at	SGCB	2,621163856	1,917743773	0,703420083
240577_at	TRAF3IP2-AS1	-0,66934516	-1,372734086	0,703388926
1564630_at	EDN1	-0,66934516	-1,372734086	0,703388926
208466_at	RAB3D	-0,66934516	-1,372734086	0,703388926
1561394_s_at	KIAA1755	-0,66934516	-1,372734086	0,703388926
243897_at	-	-0,66934516	-1,372734086	0,703388926
220563_s_at	SHANK1	-0,66934516	-1,372734086	0,703388926
216345_at	KIAA0913	-0,66934516	-1,372734086	0,703388926
216418_at	-	-0,66934516	-1,372734086	0,703388926
216630_at	-	-0,66934516	-1,372734086	0,703388926
201785_at	RNASE1	-0,66934516	-1,372734086	0,703388926
203381_s_at	APOE	-0,66934516	-1,372734086	0,703388926
209082_s_at	COL18A1	-0,66934516	-1,372734086	0,703388926
225823_at	C19orf70	4,418104951	3,714910188	0,703194763
209452_s_at	VTI1B	4,515915598	3,812818769	0,703096829
236080_at	LOC100507670	0,764143511	0,061773582	0,702369929
208095_s_at	SRP72	5,340301351	4,637996517	0,702304835
218147_s_at	GLT8D1	3,632502157	2,930238085	0,702264072
215772_x_at	SUCLG2	3,906843271	3,205255599	0,701587671
209857_s_at	SPHK2	0,556428218	-0,144765583	0,701193801
239240_at	-	0,556428218	-0,144765583	0,701193801
230062_at	RIMBP3 /// RIMBP	0,556428218	-0,144765583	0,701193801
1561537_at	LOC388906	0,556428218	-0,144765583	0,701193801
222435_s_at	UBE2J1	4,062761482	3,361641325	0,701120157

214768_x_at	IGKC	-0,230432956	-0,931539147	0,701106191
1553081_at	WFDC12	-0,230432956	-0,931539147	0,701106191
211182_x_at	LOC100506403 ///	-0,230432956	-0,931539147	0,701106191
231573_at	FATE1	-0,230432956	-0,931539147	0,701106191
234327_at	-	-0,230432956	-0,931539147	0,701106191
207882_at	-	-0,230432956	-0,931539147	0,701106191
1555491_a_at	C19orf66	-0,230432956	-0,931539147	0,701106191
201071_x_at	SF3B1	6,360070299	5,659002475	0,701067824
206037_at	CCBL1	2,118167848	1,417157926	0,701009922
240343_at	-	-1,06282519	-1,763664074	0,700838884
215445_x_at	-	-1,06282519	-1,763664074	0,700838884
232102_at	METTL6	-1,06282519	-1,763664074	0,700838884
1561686_at	-	-1,06282519	-1,763664074	0,700838884
243960_x_at	-	-1,06282519	-1,763664074	0,700838884
1570186_at	GRASPOS	-1,06282519	-1,763664074	0,700838884
1556299_s_at	C12orf76	-1,06282519	-1,763664074	0,700838884
243703_x_at	-	-1,06282519	-1,763664074	0,700838884
221624_at	TCL6	-1,06282519	-1,763664074	0,700838884
241229_at	-	-1,06282519	-1,763664074	0,700838884
238780_s_at	-	-1,06282519	-1,763664074	0,700838884
233922_at	-	-1,06282519	-1,763664074	0,700838884
1552580_at	TRIML2	-1,06282519	-1,763664074	0,700838884
243806_at	-	-1,06282519	-1,763664074	0,700838884
229077_at	KATNB1	-1,06282519	-1,763664074	0,700838884
238443_at	TFAM	-1,06282519	-1,763664074	0,700838884
228508_at	MAML3	-1,06282519	-1,763664074	0,700838884
204458_at	PLA2G15	0,282358733	-0,418248858	0,700607591
1560300_a_at	DMRTC1 /// DMRT	0,282358733	-0,418248858	0,700607591
214170_x_at	FH	5,468255648	4,767838191	0,700417457
217984_at	RNASET2	4,803736169	4,103620228	0,700115941
216645_at	-	1,160996507	0,461385738	0,69961077
57532_at	DVL2	3,058381915	2,35890611	0,699475805
231952_at	-	0,900065578	0,20062106	0,699444518
201047_x_at	RAB6A	5,00107192	4,301899151	0,699172769
90265_at	ADAP1	1,82270925	1,123997115	0,698712135
1557488_at	CBX3P2	-1,802716385	-2,501359113	0,698642728
243784_s_at	LOC100272217	-1,802716385	-2,501359113	0,698642728
217583_at	PAH	-1,802716385	-2,501359113	0,698642728
236716_at	-	-1,802716385	-2,501359113	0,698642728
224379_at	FTHL17	-1,802716385	-2,501359113	0,698642728
235988_at	GPR110	-1,802716385	-2,501359113	0,698642728
208566_at	KCNJ12 /// KCNJ18	-1,802716385	-2,501359113	0,698642728
242331_x_at	LOC642236	-1,802716385	-2,501359113	0,698642728
224131_at	HCAR1	-1,802716385	-2,501359113	0,698642728
240986_at	-	-1,802716385	-2,501359113	0,698642728
1558649_at	LOC145757	-1,802716385	-2,501359113	0,698642728
234867_at	-	-1,802716385	-2,501359113	0,698642728
240039_at	PLA2R1	-1,802716385	-2,501359113	0,698642728
244109_at	WDR38	-1,802716385	-2,501359113	0,698642728
205903_s_at	KCNN3	-1,802716385	-2,501359113	0,698642728

228501_at	GALNTL2	-1,212652659	-1,911206569	0,69855391
220174_at	LRRC8E	-1,212652659	-1,911206569	0,69855391
240779_at	-	-1,212652659	-1,911206569	0,69855391
236084_at	-	-1,212652659	-1,911206569	0,69855391
1562693_at	-	-1,212652659	-1,911206569	0,69855391
242454_at	-	-1,212652659	-1,911206569	0,69855391
211226_at	GALR2	-1,212652659	-1,911206569	0,69855391
206776_x_at	ACRV1	-1,212652659	-1,911206569	0,69855391
220815_at	CTNNA3	-1,212652659	-1,911206569	0,69855391
236886_at	LOC100049716	-1,212652659	-1,911206569	0,69855391
1565783_at	TNK2	-1,212652659	-1,911206569	0,69855391
237323_at	HKDC1	-1,212652659	-1,911206569	0,69855391
233104_at	PABPC1L	-1,212652659	-1,911206569	0,69855391
204760_s_at	NR1D1 /// THRA	-1,212652659	-1,911206569	0,69855391
1558144_at	MEG3	-1,212652659	-1,911206569	0,69855391
1560856_at	ARHGAP26-AS1	-1,212652659	-1,911206569	0,69855391
210884_s_at	SPAG11A	-1,212652659	-1,911206569	0,69855391
224364_s_at	PPIL3	5,095686705	4,397256845	0,69842986
233546_at	LOC283075	-2,336678463	-3,035090645	0,698412181
1569138_a_at	SRCAP	0,001114523	-0,697182596	0,698297119
205131_x_at	CLEC11A	0,001114523	-0,697182596	0,698297119
235370_at	KREMEN1	0,001114523	-0,697182596	0,698297119
222761_at	BIVM	2,427650693	1,72935563	0,698295064
217297_s_at	MYO9B	0,735999505	0,03784737	0,698152135
222036_s_at	MCM4	3,834426729	3,136334485	0,698092244
224565_at	LOC100653017 ///	4,773165602	4,075090115	0,698075488
230056_at	BPTF	-0,49284695	-1,19085694	0,69800999
239663_x_at	LARP1	-0,49284695	-1,19085694	0,69800999
1563300_at	-	-0,49284695	-1,19085694	0,69800999
241437_s_at	EP400NL	-0,49284695	-1,19085694	0,69800999
1559237_a_at	LOC100507108	-0,49284695	-1,19085694	0,69800999
206289_at	HOXA4	-0,49284695	-1,19085694	0,69800999
219460_s_at	TMEM127	1,843966654	1,146010634	0,69795602
233602_at	-	-2,250580859	-2,948507524	0,697926665
209612_s_at	ADH1B	-2,250580859	-2,948507524	0,697926665
243375_at	-	-2,250580859	-2,948507524	0,697926665
1552889_a_at	EXOC3L2	1,440265662	0,742671819	0,697593843
235302_at	C14orf118	1,440265662	0,742671819	0,697593843
222607_s_at	DIS3	3,493994216	2,796775008	0,697219208
211114_x_at	GEMIN2	3,007119335	2,309925448	0,697193887
225284_at	DNAJC3	2,849132494	2,151985997	0,697146497
222588_s_at	C11orf57	2,590269697	1,893444329	0,696825368
200833_s_at	LOC100506390 ///	6,775070295	6,078468568	0,696601727
222099_s_at	LSM14A	3,156891301	2,460974054	0,695917247
228493_at	LOC100506992	0,244681185	-0,45121326	0,695894445
204476_s_at	PC	0,244681185	-0,45121326	0,695894445
211241_at	ANXA2P3	0,244681185	-0,45121326	0,695894445
209622_at	STK16	2,817838865	2,121960601	0,695878264
200890_s_at	SSR1	3,697863752	3,002270677	0,695593074
39650_s_at	PCNXL2	1,384500799	0,689225775	0,695275024

1554342_s_at	HELQ	1,830468611	1,135235995	0,695232616
203646_at	FDX1	3,691400566	2,99622929	0,695171276
204158_s_at	TCIRG1	3,076375717	2,381744287	0,69463143
206992_s_at	ATP5S	2,019346222	1,324949741	0,694396481
211852_s_at	ATRN	1,421391637	0,727123109	0,694268528
218580_x_at	AURKAIP1	5,346515533	4,652447526	0,694068006
219951_s_at	DZANK1	1,000459215	0,306454867	0,694004348
224919_at	MRPS6	5,490289402	4,796421511	0,693867891
226412_at	PNISR	4,290445502	3,596611499	0,693834003
222460_s_at	FAM192A	3,509819135	2,81616114	0,693657996
201176_s_at	ARCN1	4,490240735	3,796883855	0,69335688
201095_at	DAP	3,986286762	3,293054172	0,69323259
226466_s_at	FAM58A /// FAM5	3,248151517	2,55501164	0,693139876
201276_at	RAB5B	2,248155141	1,555165465	0,692989676
214558_at	GPR12	-0,924000698	-1,616924751	0,692924054
231694_at	-	-0,924000698	-1,616924751	0,692924054
215431_at	SNTB1	-0,924000698	-1,616924751	0,692924054
220149_at	C2orf54	-0,924000698	-1,616924751	0,692924054
204462_s_at	SLC16A2	-0,924000698	-1,616924751	0,692924054
222059_at	ZNF335	-0,924000698	-1,616924751	0,692924054
1566097_at	-	-0,924000698	-1,616924751	0,692924054
231290_at	RCCD1	-0,924000698	-1,616924751	0,692924054
215025_at	NTRK3	-0,924000698	-1,616924751	0,692924054
1562106_at	-	-0,924000698	-1,616924751	0,692924054
217548_at	LOC100129502	-0,924000698	-1,616924751	0,692924054
236206_at	FAM53A	-0,924000698	-1,616924751	0,692924054
242962_at	-	-0,924000698	-1,616924751	0,692924054
225990_at	BOC	-0,924000698	-1,616924751	0,692924054
235446_at	-	-0,924000698	-1,616924751	0,692924054
1554758_a_at	CD99L2	-0,924000698	-1,616924751	0,692924054
201020_at	YWHAH	4,624674869	3,931967762	0,692707107
1563776_at	LOC100507283	-0,280273599	-0,972893339	0,69261974
1562346_at	-	-0,280273599	-0,972893339	0,69261974
221022_s_at	PMFBP1	-0,280273599	-0,972893339	0,69261974
231085_s_at	-	-0,280273599	-0,972893339	0,69261974
205824_at	HSPB2	-0,280273599	-0,972893339	0,69261974
226911_at	EGFLAM	-0,280273599	-0,972893339	0,69261974
208472_at	IKZF4	-0,280273599	-0,972893339	0,69261974
243425_at	LOC100506521	-0,280273599	-0,972893339	0,69261974
1553272_at	SLC36A1	-0,280273599	-0,972893339	0,69261974
221357_at	CHRM4	-0,280273599	-0,972893339	0,69261974
226329_s_at	MITD1	4,646299467	3,953789653	0,692509814
208617_s_at	PTP4A2	6,448385536	5,755933204	0,692452332
1570138_at	-	-0,730013898	-1,422257026	0,692243128
1563069_at	-	-0,730013898	-1,422257026	0,692243128
1570126_at	-	-0,730013898	-1,422257026	0,692243128
205985_x_at	CLCNKB	-0,730013898	-1,422257026	0,692243128
1552503_at	FRAS1	-0,730013898	-1,422257026	0,692243128
202273_at	PDGFRB	-0,730013898	-1,422257026	0,692243128
232838_at	ASXL3	-0,730013898	-1,422257026	0,692243128

1554322_a_at	HDAC4	-0,730013898	-1,422257026	0,692243128
221067_s_at	C12orf39	-0,730013898	-1,422257026	0,692243128
207468_s_at	SFRP5	-0,730013898	-1,422257026	0,692243128
212658_at	LHFPL2	-0,730013898	-1,422257026	0,692243128
208436_s_at	IRF7	1,561816652	0,869824768	0,691991884
220882_at	-	1,561816652	0,869824768	0,691991884
238907_at	ZNF780A	0,20740896	-0,484558493	0,691967454
223668_at	CBLC	0,20740896	-0,484558493	0,691967454
205930_at	GTF2E1	3,309267982	2,617393502	0,69187448
222614_at	RWDD2B	1,225123479	0,533258442	0,691865037
212419_at	ZCCHC24	1,225123479	0,533258442	0,691865037
232147_at	SLX4	1,116462765	0,424693306	0,691769459
238496_at	WHSC1L1	2,961244348	2,269637658	0,691606689
237866_at	PID1	-1,537851782	-2,229233437	0,691381655
207409_at	LECT2	-1,537851782	-2,229233437	0,691381655
242779_at	-	-1,537851782	-2,229233437	0,691381655
1563934_at	-	-1,537851782	-2,229233437	0,691381655
214612_x_at	MAGEA6	-1,537851782	-2,229233437	0,691381655
1553371_at	EPHA10	-1,537851782	-2,229233437	0,691381655
1552496_a_at	COBL	-1,537851782	-2,229233437	0,691381655
242581_at	MAP3K15	-1,537851782	-2,229233437	0,691381655
205863_at	S100A12	-1,537851782	-2,229233437	0,691381655
1554763_at	UBE2DNL	-1,537851782	-2,229233437	0,691381655
232655_at	UGT1A1	-1,537851782	-2,229233437	0,691381655
244120_at	LOC340178	-1,537851782	-2,229233437	0,691381655
205597_at	SLC44A4	-1,537851782	-2,229233437	0,691381655
1560108_at	-	-1,537851782	-2,229233437	0,691381655
217757_at	A2M	-1,537851782	-2,229233437	0,691381655
1553213_a_at	KRT78	-1,537851782	-2,229233437	0,691381655
234160_at	-	-1,537851782	-2,229233437	0,691381655
1566841_at	-	-1,537851782	-2,229233437	0,691381655
210745_at	ONECUT1	-1,537851782	-2,229233437	0,691381655
234856_at	-	-1,537851782	-2,229233437	0,691381655
1560135_at	-	-1,537851782	-2,229233437	0,691381655
1560964_at	-	-1,537851782	-2,229233437	0,691381655
1556958_at	-	-1,537851782	-2,229233437	0,691381655
1563849_at	SH2D4B	-1,537851782	-2,229233437	0,691381655
236189_at	ANKRD50	-1,537851782	-2,229233437	0,691381655
1558963_at	-	-1,537851782	-2,229233437	0,691381655
206981_at	SCN4A	-1,537851782	-2,229233437	0,691381655
242566_at	VASH1	-1,537851782	-2,229233437	0,691381655
219839_x_at	TCL6	-1,537851782	-2,229233437	0,691381655
233985_x_at	PPP1R9A	-1,537851782	-2,229233437	0,691381655
1561574_at	-	-1,537851782	-2,229233437	0,691381655
1554666_at	LOC100130950	-1,537851782	-2,229233437	0,691381655
1555988_a_at	LOC126536	-1,537851782	-2,229233437	0,691381655
231467_at	-	-1,537851782	-2,229233437	0,691381655
1567270_at	-	-1,537851782	-2,229233437	0,691381655
1564067_x_at	TMEM151B	-1,537851782	-2,229233437	0,691381655
202902_s_at	CTSS	2,926639205	2,235857731	0,690781474

204758_s_at	C2CD2L	-0,043925712	-0,734355396	0,690429684
241813_at	MBD1	-0,043925712	-0,734355396	0,690429684
1553688_at	METTL6	-0,043925712	-0,734355396	0,690429684
233467_s_at	TSPAN32	-0,043925712	-0,734355396	0,690429684
235145_at	ZBTB7B	0,97663406	0,286279868	0,690354192
206951_at	HIST1H4A /// HIST	0,458905032	-0,231045907	0,689950938
215233_at	JMJD6	0,458905032	-0,231045907	0,689950938
204177_s_at	KLHL20	2,229377375	1,539450683	0,689926692
223630_at	C7orf13	2,149438457	1,459545271	0,689893186
209029_at	COPS7A	2,642918178	1,953032682	0,689885496
203739_at	ZNF217	4,31266827	3,622794326	0,689873944
223728_at	CENPBD1	1,476175815	0,786391549	0,689784266
224028_at	CPA6	-1,452713826	-2,142286822	0,689572996
231392_at	-	-1,452713826	-2,142286822	0,689572996
1567856_x_at	ZNF29P	-1,452713826	-2,142286822	0,689572996
244066_at	-	-1,452713826	-2,142286822	0,689572996
1560411_at	-	-1,452713826	-2,142286822	0,689572996
216081_at	LAMA4	-1,452713826	-2,142286822	0,689572996
1558293_at	KIAA1107	-1,452713826	-2,142286822	0,689572996
233950_at	CADPS	-1,452713826	-2,142286822	0,689572996
222194_at	FAM66D	-1,452713826	-2,142286822	0,689572996
209638_x_at	RGS12	-1,452713826	-2,142286822	0,689572996
207615_s_at	C16orf3	-1,452713826	-2,142286822	0,689572996
235935_at	LRRC73	-1,452713826	-2,142286822	0,689572996
230913_at	-	-1,452713826	-2,142286822	0,689572996
242000_at	CASD1	-1,452713826	-2,142286822	0,689572996
1556145_a_at	LOC100131170	-1,452713826	-2,142286822	0,689572996
215106_at	TTC22	-1,452713826	-2,142286822	0,689572996
215335_at	-	-1,452713826	-2,142286822	0,689572996
228467_at	PURB	-1,452713826	-2,142286822	0,689572996
231458_at	LOC100507629	-1,452713826	-2,142286822	0,689572996
241037_at	-	-1,452713826	-2,142286822	0,689572996
211981_at	COL4A1	-1,452713826	-2,142286822	0,689572996
200620_at	TMEM59	5,834315089	5,14488611	0,689428979
1569478_s_at	-	1,307201325	0,61779983	0,689401495
201535_at	UBL3	3,286844008	2,597466271	0,689377737
213501_at	ACOX1	1,625482993	0,936232181	0,689250812
206342_x_at	IDS	3,442765386	2,753767314	0,688998072
220988_s_at	C1QTNF3	0,677745787	-0,011001236	0,688747023
244453_at	ANKRD53	0,677745787	-0,011001236	0,688747023
1560496_at	-	0,677745787	-0,011001236	0,688747023
213778_x_at	ZNF276	0,677745787	-0,011001236	0,688747023
234201_x_at	-	0,677745787	-0,011001236	0,688747023
213246_at	C14orf109	3,903941986	3,215288307	0,688653679
244354_at	STRN	1,544940605	0,85655369	0,688386915
235566_at	TMF1	1,094118704	0,405752839	0,688365865
221049_s_at	POLL	1,094118704	0,405752839	0,688365865
216177_at	-	2,267141818	1,578991006	0,688150813
210186_s_at	FKBP1A	3,19484646	2,506994817	0,687851643
219680_at	NLRX1	2,692288672	2,004917973	0,687370699

216310_at	TAOK1	1,610353504	0,923203038	0,687150465
1552592_at	MMP21	-1,624955693	-2,312054429	0,687098736
1569193_at	-	-1,624955693	-2,312054429	0,687098736
221858_at	TBC1D12	-1,624955693	-2,312054429	0,687098736
1568851_at	-	-1,624955693	-2,312054429	0,687098736
1565265_at	-	-1,624955693	-2,312054429	0,687098736
1559036_at	C4A /// C4B /// LO	-1,624955693	-2,312054429	0,687098736
214970_s_at	ST6GAL1	-1,624955693	-2,312054429	0,687098736
210360_s_at	MTSS1	-1,624955693	-2,312054429	0,687098736
229708_at	C9orf167	-1,624955693	-2,312054429	0,687098736
240287_at	IRG1	-1,624955693	-2,312054429	0,687098736
236438_at	-	-1,624955693	-2,312054429	0,687098736
225353_s_at	C1QC	-1,624955693	-2,312054429	0,687098736
206446_s_at	CELA2A /// CELA2E	-1,624955693	-2,312054429	0,687098736
1553232_at	FAM82A1	-1,624955693	-2,312054429	0,687098736
1554810_at	PLA2G4C	-1,624955693	-2,312054429	0,687098736
209590_at	BMP7	-1,624955693	-2,312054429	0,687098736
1565282_at	-	-1,624955693	-2,312054429	0,687098736
204570_at	COX7A1	-1,624955693	-2,312054429	0,687098736
225142_at	JHDM1D	-1,624955693	-2,312054429	0,687098736
203158_s_at	GLS	0,820160788	0,133104519	0,687056269
217978_s_at	UBE2Q1	5,116796324	4,429907797	0,686888527
200624_s_at	MATR3 /// SNHG4	5,496033243	4,809191163	0,686842079
203771_s_at	BLVRA	2,56794744	1,881145546	0,686801893
202373_s_at	AURKAPS1 /// RAB	3,768156719	3,081435405	0,686721314
203944_x_at	BTN2A1	3,029186714	2,34250725	0,686679464
224602_at	C4orf3	4,749149445	4,062923431	0,686226014
217860_at	NDUFA10	5,069714167	4,383687696	0,686026471
240516_at	-	0,167727503	-0,518232988	0,685960492
218049_s_at	LOC100506980 ///	4,72953846	4,043683659	0,685854801
214733_s_at	YIPF1	2,817838865	2,132145748	0,685693116
230257_s_at	TSEN15	4,186291235	3,500705275	0,68558596
215084_s_at	LRRC42	2,71254166	2,026957145	0,685584515
214195_at	TPP1	0,425036312	-0,260526297	0,685562609
204897_at	PTGER4	5,389930424	4,704551849	0,685378576
219419_at	RBFA	2,127965541	1,44320939	0,684756151
228798_x_at	MAZ	0,648195588	-0,036543687	0,684739275
225034_at	ST3GAL1	0,648195588	-0,036543687	0,684739275
218728_s_at	CNIH4	3,957687264	3,27333514	0,684352124
200746_s_at	GNB1	5,428968009	4,744668046	0,684299963
227451_s_at	CCDC90A	2,507817181	1,823960141	0,68385704
226507_at	PAK1	3,132805465	2,449078728	0,683726737
218128_at	NFYB	2,2388565	1,555165465	0,683691035
239039_at	-	-0,551284523	-1,234835326	0,683550803
229168_at	COL23A1	-0,551284523	-1,234835326	0,683550803
209499_x_at	TNFSF12 /// TNFSF	-0,551284523	-1,234835326	0,683550803
222335_at	-	-0,551284523	-1,234835326	0,683550803
221921_s_at	CADM3	-0,551284523	-1,234835326	0,683550803
1566721_at	SVEP1	-0,551284523	-1,234835326	0,683550803
223968_at	ZNF44	-0,551284523	-1,234835326	0,683550803

216582_at	POM121L2	-0,551284523	-1,234835326	0,683550803
231457_at	-	-0,551284523	-1,234835326	0,683550803
230801_at	RPRD1B	-0,551284523	-1,234835326	0,683550803
225437_s_at	BRAT1	1,267146969	0,583607503	0,683539466
212628_at	PKN2	2,118167848	1,434743608	0,68342424
1560369_at	ANKH	-1,668176653	-2,351407175	0,683230522
228060_at	SLC35F1	-0,332405896	-1,015398016	0,68299212
220711_at	-	-0,332405896	-1,015398016	0,68299212
221232_s_at	ANKRD2	-0,332405896	-1,015398016	0,68299212
229856_s_at	PITHD1	-0,332405896	-1,015398016	0,68299212
229936_at	GFRA3	-0,332405896	-1,015398016	0,68299212
1561019_at	-	-0,332405896	-1,015398016	0,68299212
234099_at	-	-0,332405896	-1,015398016	0,68299212
207482_at	TP53TG5	-0,332405896	-1,015398016	0,68299212
231469_at	NTRK3-AS1	-0,332405896	-1,015398016	0,68299212
239524_at	TSSK6	-0,332405896	-1,015398016	0,68299212
213100_at	UNC5B	-0,332405896	-1,015398016	0,68299212
241053_at	LOC730184	-0,332405896	-1,015398016	0,68299212
217621_at	SLC6A2	-1,982324124	-2,665156778	0,682832654
1561860_at	-	-1,982324124	-2,665156778	0,682832654
234616_at	-	-1,982324124	-2,665156778	0,682832654
1561322_at	-	-1,982324124	-2,665156778	0,682832654
231569_at	TMEM31	-1,982324124	-2,665156778	0,682832654
208123_at	KCNB2	-1,982324124	-2,665156778	0,682832654
211866_x_at	HFE	0,127732831	-0,554696666	0,682429497
234070_at	-	0,127732831	-0,554696666	0,682429497
235491_at	ZBTB10	0,127732831	-0,554696666	0,682429497
239213_at	SERPINB1	0,127732831	-0,554696666	0,682429497
206424_at	CYP26A1	-0,088974936	-0,771340337	0,682365401
220264_s_at	GPR107	-0,088974936	-0,771340337	0,682365401
207096_at	SAA2-SAA4 /// SAA	-0,088974936	-0,771340337	0,682365401
228178_s_at	-	-0,088974936	-0,771340337	0,682365401
230484_at	CHDH	-0,088974936	-0,771340337	0,682365401
234076_at	-	-0,088974936	-0,771340337	0,682365401
229729_at	TMEM8B	-0,088974936	-0,771340337	0,682365401
233144_s_at	RASAL1	-0,088974936	-0,771340337	0,682365401
230371_at	HPS6	-0,088974936	-0,771340337	0,682365401
208700_s_at	TKT	6,497299986	5,814969827	0,682330159
226457_at	-	2,229377375	1,547376668	0,682000707
235830_at	-	1,160996507	0,479089184	0,681907323
228785_at	-	3,903941986	3,222133192	0,681808794
226836_at	SFT2D1	0,39105295	-0,290694975	0,681747926
230880_at	KIAA1652	0,618277321	-0,063377083	0,681654404
216646_at	DSCC1	0,618277321	-0,063377083	0,681654404
212202_s_at	TMEM87A	3,122148189	2,440833194	0,681314996
223493_at	FBXO4	1,910557155	1,2298509	0,680706255
233356_at	-	-0,791294935	-1,471833228	0,680538292
208205_at	PCDHA9	-0,791294935	-1,471833228	0,680538292
238117_at	PPOX	-0,791294935	-1,471833228	0,680538292
226988_s_at	MYH14	-0,791294935	-1,471833228	0,680538292

1562447_a_at	LOC100506599	-0,791294935	-1,471833228	0,680538292
206298_at	ARHGAP22	-0,791294935	-1,471833228	0,680538292
1559508_at	-	-0,791294935	-1,471833228	0,680538292
236591_at	LOC100505738	-0,791294935	-1,471833228	0,680538292
228439_at	BATF2	-0,791294935	-1,471833228	0,680538292
1554852_a_at	KIAA1257	-0,791294935	-1,471833228	0,680538292
243798_at	-	-0,791294935	-1,471833228	0,680538292
230463_at	-	-0,791294935	-1,471833228	0,680538292
240541_at	-	-0,791294935	-1,471833228	0,680538292
206403_at	ZNF536	-0,791294935	-1,471833228	0,680538292
244729_at	-	-0,791294935	-1,471833228	0,680538292
221101_at	-	-0,791294935	-1,471833228	0,680538292
227647_at	KCNE3	-0,791294935	-1,471833228	0,680538292
219085_s_at	GEMIN7	-0,791294935	-1,471833228	0,680538292
210799_at	HTR1B	-0,791294935	-1,471833228	0,680538292
204419_x_at	HBG1 /// HBG2 ///	-0,791294935	-1,471833228	0,680538292
212185_x_at	MT2A	5,857960538	5,177692413	0,680268124
202502_at	ACADM	5,541129315	4,86108334	0,680045974
234527_at	-	-1,369772723	-2,049747196	0,679974473
1552991_at	OR5P2	-1,369772723	-2,049747196	0,679974473
1564485_at	LOC100131551	-1,369772723	-2,049747196	0,679974473
1557564_at	LOC100507300	-1,369772723	-2,049747196	0,679974473
204967_at	SHROOM2	-1,369772723	-2,049747196	0,679974473
228546_at	DPP6	-1,369772723	-2,049747196	0,679974473
243110_x_at	NPW	-1,369772723	-2,049747196	0,679974473
1561311_at	-	-1,369772723	-2,049747196	0,679974473
1557104_at	ZSCAN30	-1,369772723	-2,049747196	0,679974473
207915_at	-	-1,369772723	-2,049747196	0,679974473
233363_at	-	-1,369772723	-2,049747196	0,679974473
243321_at	-	-1,369772723	-2,049747196	0,679974473
237470_at	DOCK7	-1,369772723	-2,049747196	0,679974473
203980_at	FABP4	-1,369772723	-2,049747196	0,679974473
236764_at	-	-1,369772723	-2,049747196	0,679974473
221084_at	HTR3B	-1,369772723	-2,049747196	0,679974473
242904_x_at	LOC100653229	-1,369772723	-2,049747196	0,679974473
207872_s_at	LILRA1	-1,369772723	-2,049747196	0,679974473
242988_at	DCST2	-1,369772723	-2,049747196	0,679974473
1569980_x_at	HKR1	-1,369772723	-2,049747196	0,679974473
231663_s_at	ARG1	-1,369772723	-2,049747196	0,679974473
237099_at	BPIFA2	-1,369772723	-2,049747196	0,679974473
211490_at	ADRA1A	-1,369772723	-2,049747196	0,679974473
205502_at	CYP17A1	-1,369772723	-2,049747196	0,679974473
230586_s_at	-	-1,369772723	-2,049747196	0,679974473
237151_s_at	PCDP1	-1,369772723	-2,049747196	0,679974473
230053_at	-	-1,369772723	-2,049747196	0,679974473
213245_at	ADCY1	-1,369772723	-2,049747196	0,679974473
1553579_a_at	SPAG11A /// SPAG	-1,712732543	-2,391863529	0,679130986
234603_at	-	-1,712732543	-2,391863529	0,679130986
231446_at	LOC100506226	-1,712732543	-2,391863529	0,679130986
210548_at	CCL23	-1,712732543	-2,391863529	0,679130986

239713_at	CASC2	-1,712732543	-2,391863529	0,679130986
206692_at	KCNJ10	-1,712732543	-2,391863529	0,679130986
203798_s_at	VSNL1	-1,712732543	-2,391863529	0,679130986
231314_at	-	-1,712732543	-2,391863529	0,679130986
224488_s_at	SPON1	-1,712732543	-2,391863529	0,679130986
1554934_at	RCBTB1	-1,712732543	-2,391863529	0,679130986
240879_x_at	-	-1,712732543	-2,391863529	0,679130986
238862_at	MFSD4	-1,712732543	-2,391863529	0,679130986
1558950_at	-	-1,712732543	-2,391863529	0,679130986
220031_at	OTUD7B	-1,712732543	-2,391863529	0,679130986
1569322_at	LOC439990	-1,712732543	-2,391863529	0,679130986
229476_s_at	THRSP	-1,712732543	-2,391863529	0,679130986
1554374_at	-	-1,712732543	-2,391863529	0,679130986
205028_at	TRO	-1,712732543	-2,391863529	0,679130986
244796_at	-	-1,712732543	-2,391863529	0,679130986
231804_at	RXFP1	-1,712732543	-2,391863529	0,679130986
221099_at	-	0,764143511	0,085534992	0,678608518
225403_at	RPP25L	3,649735397	2,971193817	0,67854158
213489_at	MAPRE2	1,246401134	0,568155442	0,678245691
238675_x_at	BTF3L4	1,745502609	1,067365565	0,678137044
211728_s_at	HYAL3	0,355751005	-0,322340048	0,678091053
219353_at	NHLRC2	0,355751005	-0,322340048	0,678091053
207189_s_at	ZZEF1	0,355751005	-0,322340048	0,678091053
214857_at	LOC100505761	0,355751005	-0,322340048	0,678091053
1561363_a_at	-	-2,074223907	-2,752097204	0,677873297
240955_at	PANX3	-2,074223907	-2,752097204	0,677873297
1556039_s_at	GPR173	-2,074223907	-2,752097204	0,677873297
1561962_at	-	-2,074223907	-2,752097204	0,677873297
208094_s_at	CCDC130	1,625482993	0,947673886	0,677809107
222906_at	FLVCR1	2,739067176	2,06133574	0,677731436
212846_at	RRP1B	5,014397839	4,337375351	0,677022489
239472_at	-	-1,893506789	-2,570522741	0,677015952
1562710_at	-	-1,893506789	-2,570522741	0,677015952
238314_x_at	-	-1,893506789	-2,570522741	0,677015952
230943_at	SOX17	-1,893506789	-2,570522741	0,677015952
1565596_at	-	-1,893506789	-2,570522741	0,677015952
1554161_at	SLC25A27	-1,893506789	-2,570522741	0,677015952
231186_at	FLJ43390	-1,893506789	-2,570522741	0,677015952
1562645_at	LOC401176	-1,893506789	-2,570522741	0,677015952
240884_at	CCDC14	-1,893506789	-2,570522741	0,677015952
217287_s_at	TRPC6	-1,893506789	-2,570522741	0,677015952
240408_at	-	-1,893506789	-2,570522741	0,677015952
1563420_at	XG /// XGPY2	-1,893506789	-2,570522741	0,677015952
217021_at	CYB5A	-1,893506789	-2,570522741	0,677015952
203540_at	GFAP	-1,893506789	-2,570522741	0,677015952
232442_at	BCAR1	-1,893506789	-2,570522741	0,677015952
207080_s_at	PYY	-1,893506789	-2,570522741	0,677015952
214156_at	MYRIP	-1,893506789	-2,570522741	0,677015952
201840_at	NEDD8	5,601579484	4,924704877	0,676874607
230393_at	CUL5	-0,135065865	-0,811769547	0,676703682

243679_at	JPH3	-0,135065865	-0,811769547	0,676703682
1557689_at	-	-0,135065865	-0,811769547	0,676703682
232736_s_at	POM121L9P	-0,135065865	-0,811769547	0,676703682
230754_at	ZBTB38	-0,135065865	-0,811769547	0,676703682
240443_at	-	-0,135065865	-0,811769547	0,676703682
208843_s_at	GORASP2	4,462797972	3,786191905	0,676606067
201761_at	MTHFD2	7,058288222	6,382518316	0,675769906
219374_s_at	ALG9	2,62869474	1,953032682	0,675662057
204590_x_at	VPS33A	1,730929079	1,055317091	0,675611987
238223_at	-	0,08619576	-0,589001171	0,675196931
234870_at	TANC1	0,08619576	-0,589001171	0,675196931
232647_at	PROCA1	0,08619576	-0,589001171	0,675196931
238052_at	-	0,08619576	-0,589001171	0,675196931
227308_x_at	LTBP3	0,08619576	-0,589001171	0,675196931
32402_s_at	SYMPK	0,886987435	0,211801886	0,675185549
221800_s_at	C17orf70	1,671397663	0,996453274	0,674944389
226511_at	DCAF10	1,671397663	0,996453274	0,674944389
220452_x_at	-	1,671397663	0,996453274	0,674944389
229594_at	SPTY2D1	3,233229368	2,558652146	0,674577222
208026_at	HIST1H4A /// HIST	-1,137208348	-1,811780055	0,674571706
205237_at	FCN1	-1,137208348	-1,811780055	0,674571706
231099_at	FKBP15	-1,137208348	-1,811780055	0,674571706
241221_at	SEC14L3	-1,137208348	-1,811780055	0,674571706
206207_at	CLC	-1,137208348	-1,811780055	0,674571706
240913_at	FGFR2	-1,137208348	-1,811780055	0,674571706
230467_at	TMEM52	-1,137208348	-1,811780055	0,674571706
240243_at	-	-1,137208348	-1,811780055	0,674571706
1559880_at	LZTS1-AS1	-1,137208348	-1,811780055	0,674571706
217350_at	KRT19P2 /// MIR4!	-1,137208348	-1,811780055	0,674571706
214576_at	KRT36	-1,137208348	-1,811780055	0,674571706
207639_at	FZD9	-1,137208348	-1,811780055	0,674571706
227938_s_at	DLL1	-1,137208348	-1,811780055	0,674571706
241717_at	LOC285281	-1,137208348	-1,811780055	0,674571706
222253_s_at	POM121L9P	-1,137208348	-1,811780055	0,674571706
223349_s_at	BOK	-1,137208348	-1,811780055	0,674571706
215427_s_at	ZCCHC14	-1,137208348	-1,811780055	0,674571706
234782_at	KLHL31	-1,137208348	-1,811780055	0,674571706
231609_at	C10orf82	-1,137208348	-1,811780055	0,674571706
229805_at	ZDHHC22	-1,137208348	-1,811780055	0,674571706
234916_at	DBH	-1,137208348	-1,811780055	0,674571706
244212_at	-	-1,137208348	-1,811780055	0,674571706
242180_at	TSPAN16	-1,137208348	-1,811780055	0,674571706
233537_at	KRTAP3-1	-1,137208348	-1,811780055	0,674571706
205852_at	CDK5R2	-1,137208348	-1,811780055	0,674571706
232123_at	LOC283174	-1,137208348	-1,811780055	0,674571706
223379_s_at	LATS2	-1,137208348	-1,811780055	0,674571706
200743_s_at	TPP1	3,77412549	3,099629381	0,674496108
1556988_s_at	CHD1L	3,903941986	3,229534127	0,67440786
212080_at	MLL	2,410823487	1,736450458	0,674373029
218059_at	ZNF706	6,120105454	5,445759108	0,674346347

239807_at	-	0,735999505	0,061773582	0,674225923
215218_s_at	WDR62	0,735999505	0,061773582	0,674225923
238800_s_at	ZCCHC6	0,319287178	-0,354781582	0,674068759
226189_at	ITGB8	3,055618382	2,381744287	0,673874095
224726_at	MIB1	3,931608669	3,257790126	0,673818543
212976_at	LRRC8B	-0,609040214	-1,282520722	0,673480508
231058_at	FXYD4	-0,609040214	-1,282520722	0,673480508
1556405_s_at	LOC374890	-0,609040214	-1,282520722	0,673480508
230823_at	-	-0,609040214	-1,282520722	0,673480508
244823_at	LOC100129034	-0,609040214	-1,282520722	0,673480508
233466_at	LOC100652861	-0,609040214	-1,282520722	0,673480508
240654_at	-	-0,609040214	-1,282520722	0,673480508
1552955_at	LINC00208	-0,609040214	-1,282520722	0,673480508
1555231_a_at	C21orf88	-0,609040214	-1,282520722	0,673480508
227753_at	TMEM139	-0,609040214	-1,282520722	0,673480508
213519_s_at	LAMA2	-0,609040214	-1,282520722	0,673480508
237079_at	-	-0,609040214	-1,282520722	0,673480508
231605_at	-	-0,609040214	-1,282520722	0,673480508
228663_x_at	FIZ1	0,556428218	-0,116767475	0,673195693
202796_at	SYNPO	0,873789652	0,20062106	0,673168592
203723_at	ITPKB	0,873789652	0,20062106	0,673168592
1569172_a_at	LOC402160	-0,384193355	-1,057301851	0,673108496
1553488_at	TEKT5	-0,384193355	-1,057301851	0,673108496
237348_at	-	-0,384193355	-1,057301851	0,673108496
243536_x_at	ARHGAP27	-0,384193355	-1,057301851	0,673108496
231177_at	HCFC1	-0,384193355	-1,057301851	0,673108496
233550_s_at	SLC4A11	-0,384193355	-1,057301851	0,673108496
207011_s_at	PTK7	-0,384193355	-1,057301851	0,673108496
232593_at	NEURL3	-0,384193355	-1,057301851	0,673108496
207169_x_at	DDR1 /// MIR4640	-0,384193355	-1,057301851	0,673108496
224965_at	GNG2	1,307201325	0,634301128	0,672900197
217727_x_at	VPS35	5,505011382	4,832394579	0,672616803
209020_at	C20orf111	3,045068376	2,373249966	0,67181841
1553645_at	CCDC141	1,65554524	0,98381571	0,67172953
227262_at	HAPLN3	1,65554524	0,98381571	0,67172953
243785_at	LOC100272217	-0,994186487	-1,665791776	0,671605289
230899_at	SETD4	-0,994186487	-1,665791776	0,671605289
207995_s_at	CLEC4M	-0,994186487	-1,665791776	0,671605289
240319_at	-	-0,994186487	-1,665791776	0,671605289
1558532_at	TPM1	-0,994186487	-1,665791776	0,671605289
1566476_at	-	-0,994186487	-1,665791776	0,671605289
207841_at	SPIN2A	-0,994186487	-1,665791776	0,671605289
230431_at	-	-0,994186487	-1,665791776	0,671605289
244543_s_at	BCDIN3D-AS1	-0,994186487	-1,665791776	0,671605289
211608_at	-	-0,994186487	-1,665791776	0,671605289
240808_at	ESD	-0,994186487	-1,665791776	0,671605289
1562906_at	FAM170A	-0,994186487	-1,665791776	0,671605289
237440_at	-	-0,994186487	-1,665791776	0,671605289
220057_at	XAGE1A /// XAGE1	-0,994186487	-1,665791776	0,671605289
237087_at	-	-0,994186487	-1,665791776	0,671605289

202023_at	EFNA1	-0,994186487	-1,665791776	0,671605289
52159_at	HEMK1	2,511250263	1,839756862	0,6714934
219329_s_at	C2orf28	6,084541703	5,413095764	0,671445939
222235_s_at	CSGALNACT2	3,018305638	2,346979857	0,67132578
200091_s_at	RPS25	8,011325363	7,340465469	0,670859894
222751_at	HERPUD2	4,07586392	3,405078581	0,670785339
225441_x_at	LSMD1	3,214269085	2,543655878	0,670613207
228189_at	BAG4	4,144849571	3,474637573	0,670211997
226703_at	NEURL4	1,094118704	0,424693306	0,669425398
228453_at	EPG5	0,847529938	0,178191865	0,669338073
239135_at	CPPED1	0,847529938	0,178191865	0,669338073
222160_at	AKAP8L	0,282358733	-0,386896102	0,669254835
218270_at	MRPL24	3,707586205	3,038411131	0,669175075
203378_at	PCF11	2,356619952	1,687645398	0,668974553
234057_at	-	-2,163157732	-2,832036647	0,668878915
238196_at	LOC285095	-2,163157732	-2,832036647	0,668878915
208202_s_at	PHF15	0,045517965	-0,623254098	0,668772063
1558641_at	SPATA24	2,597647262	1,929092307	0,668554955
238484_s_at	SSBP2	-0,180990326	-0,84928999	0,668299664
207399_at	BFSP2	-0,180990326	-0,84928999	0,668299664
210552_s_at	RALGPS1	-0,180990326	-0,84928999	0,668299664
1561770_at	-	-0,180990326	-0,84928999	0,668299664
231401_s_at	-	-0,180990326	-0,84928999	0,668299664
238334_at	MTG1	-0,180990326	-0,84928999	0,668299664
239853_at	KLC3	-0,180990326	-0,84928999	0,668299664
218667_at	PJA1	3,142467702	2,47417686	0,668290843
219099_at	C12orf5	3,639446351	2,971193817	0,668252533
226734_at	EIF4E2 /// LOC100	2,159837409	1,491644074	0,668193335
222133_s_at	PHF20L1	1,898032047	1,2298509	0,668181147
214259_s_at	AKR7A2	4,687775278	4,019608479	0,668166798
213902_at	ASAH1	3,537317882	2,869317249	0,668000633
65718_at	GPR124	-0,024015603	-0,691835321	0,667819718
225649_s_at	STK35	4,891443931	4,22363244	0,667811491
223077_at	TMOD3	3,505636113	2,83813104	0,667505073
223607_x_at	ZSWIM1	1,440265662	0,772793615	0,667472048
232581_x_at	HIVEP3	1,182925501	0,515457602	0,667467899
224833_at	ETS1	5,635134943	4,967825818	0,667309126
226054_at	BRD4	3,237994092	2,570802332	0,66719176
221773_at	ELK3	3,966733349	3,299705477	0,667027872
224938_at	NUFIP2	2,99543179	2,328492509	0,666939281
1556158_at	FAM154B	-1,289805289	-1,956681069	0,666875781
205655_at	MDM4	-1,289805289	-1,956681069	0,666875781
1560238_at	LOC100506930	-1,289805289	-1,956681069	0,666875781
231653_at	CCDC129	-1,289805289	-1,956681069	0,666875781
1555958_at	CRTAC1	-1,289805289	-1,956681069	0,666875781
236861_at	-	-1,289805289	-1,956681069	0,666875781
230698_at	CALN1	-1,289805289	-1,956681069	0,666875781
221338_at	LY6G6E	-1,289805289	-1,956681069	0,666875781
239306_at	-	-1,289805289	-1,956681069	0,666875781
244662_at	MBD5	-1,289805289	-1,956681069	0,666875781

224515_at	-	-1,289805289	-1,956681069	0,666875781
208162_s_at	-	-1,289805289	-1,956681069	0,666875781
231440_at	LOC100507206	-1,289805289	-1,956681069	0,666875781
234610_at	HSPA12B	-1,289805289	-1,956681069	0,666875781
239916_at	WDR16	-1,289805289	-1,956681069	0,666875781
216245_at	IL1RN	-1,289805289	-1,956681069	0,666875781
1568593_a_at	NUDT16P1	-1,289805289	-1,956681069	0,666875781
1569647_at	LOC643623	-1,289805289	-1,956681069	0,666875781
207312_at	PHKG1	-1,289805289	-1,956681069	0,666875781
211435_at	-	-1,289805289	-1,956681069	0,666875781
234292_s_at	ZNF167	-1,289805289	-1,956681069	0,666875781
229332_at	HPDL	1,267146969	0,600385088	0,666761881
230200_at	NSUN6	2,304041045	1,637482551	0,666558495
225425_s_at	MRPL41	3,152091004	2,486505579	0,665585426
216689_x_at	ARHGAP1	1,071336699	0,405752839	0,66558386
224765_at	MSL1	3,988952858	3,323485083	0,665467775
200757_s_at	CALU	3,442765386	2,777528602	0,665236783
202171_at	VEZF1	3,493994216	2,828849722	0,665144495
204872_at	TLE4	2,21987062	1,555165465	0,664705155
201097_s_at	ARF4	6,156267609	5,491706717	0,664560892
226168_at	ZFAND2B	2,468978138	1,804519951	0,664458188
217125_at	-	1,493647653	0,829309537	0,664338116
221949_at	UBE2D4	0,491906512	-0,172194225	0,664100736
211677_x_at	CADM3	0,491906512	-0,172194225	0,664100736
203165_s_at	SLC33A1	2,007220798	1,343200523	0,664020275
225179_at	UBE2K	4,418104951	3,754141744	0,663963207
223090_x_at	VEZT	4,027733188	3,363771078	0,66396211
228121_at	TGFB2	-0,856259172	-1,520022123	0,663762951
1560698_a_at	TRHDE-AS1	-0,856259172	-1,520022123	0,663762951
213781_at	PPP1R37	-0,856259172	-1,520022123	0,663762951
238090_at	HGSNAT	-0,856259172	-1,520022123	0,663762951
236818_at	-	-0,856259172	-1,520022123	0,663762951
229207_x_at	RNF187	-0,856259172	-1,520022123	0,663762951
219404_at	EPS8L3	-0,856259172	-1,520022123	0,663762951
1556038_at	GPR173	-0,856259172	-1,520022123	0,663762951
205143_at	NCAN	-0,856259172	-1,520022123	0,663762951
241039_at	-	-0,856259172	-1,520022123	0,663762951
207260_at	FEV	-0,856259172	-1,520022123	0,663762951
221016_s_at	TCF7L1	-0,856259172	-1,520022123	0,663762951
211667_x_at	TRAV12-2	-0,856259172	-1,520022123	0,663762951
1561085_at	LOC153910	-0,856259172	-1,520022123	0,663762951
209851_at	ZC3H13	-0,856259172	-1,520022123	0,663762951
1566138_at	-	-0,856259172	-1,520022123	0,663762951
1552418_at	TTLL10	-0,856259172	-1,520022123	0,663762951
1561928_s_at	ANKUB1	-0,856259172	-1,520022123	0,663762951
232404_at	SHROOM4	-0,856259172	-1,520022123	0,663762951
233388_at	-	-0,856259172	-1,520022123	0,663762951
215267_s_at	SLC8A2	-0,856259172	-1,520022123	0,663762951
221132_at	CLDN18	-0,856259172	-1,520022123	0,663762951
232465_at	-	-0,856259172	-1,520022123	0,663762951

224259_at	WNT8A	-0,856259172	-1,520022123	0,663762951
217684_at	TYMS	-0,856259172	-1,520022123	0,663762951
229242_at	TNFSF15	-0,856259172	-1,520022123	0,663762951
243545_at	-	-0,856259172	-1,520022123	0,663762951
223905_at	CCDC135	-0,856259172	-1,520022123	0,663762951
219103_at	ASAP3	-0,856259172	-1,520022123	0,663762951
229702_at	CSNK1G3	-0,856259172	-1,520022123	0,663762951
240920_at	-	-0,856259172	-1,520022123	0,663762951
1566157_x_at	-	-0,856259172	-1,520022123	0,663762951
240572_s_at	LOC374443	2,427650693	1,76396664	0,663684054
1563941_at	-	-1,802716385	-2,466349414	0,663633029
1562638_at	LOC339874	-1,802716385	-2,466349414	0,663633029
219804_at	SYNPO2L	-1,802716385	-2,466349414	0,663633029
1560154_a_at	-	-1,802716385	-2,466349414	0,663633029
203180_at	ALDH1A3	-1,802716385	-2,466349414	0,663633029
242595_at	TSSK4	-1,802716385	-2,466349414	0,663633029
240187_at	PPP1R3C	-1,802716385	-2,466349414	0,663633029
1563887_at	INTS4L1	-1,802716385	-2,466349414	0,663633029
236633_at	GTPBP10	-1,802716385	-2,466349414	0,663633029
1556401_a_at	-	-1,802716385	-2,466349414	0,663633029
1555876_at	SRGAP1	-1,802716385	-2,466349414	0,663633029
242816_at	-	-1,802716385	-2,466349414	0,663633029
1557343_at	-	-1,802716385	-2,466349414	0,663633029
240967_at	KRTAP19-3	-1,802716385	-2,466349414	0,663633029
241067_at	-	-1,802716385	-2,466349414	0,663633029
219250_s_at	FLRT3	-1,802716385	-2,466349414	0,663633029
214868_at	PIWIL1	-1,802716385	-2,466349414	0,663633029
221353_at	OR3A1	-1,802716385	-2,466349414	0,663633029
232957_x_at	-	-1,802716385	-2,466349414	0,663633029
219383_at	PRR5L	-1,802716385	-2,466349414	0,663633029
227572_at	USP30	1,730929079	1,067365565	0,663563514
244525_at	-	-0,437449947	-1,100943374	0,663493427
233217_at	-	-0,437449947	-1,100943374	0,663493427
228134_at	MYH11	-0,437449947	-1,100943374	0,663493427
237307_at	-	-0,437449947	-1,100943374	0,663493427
232109_at	UBXN10	-0,437449947	-1,100943374	0,663493427
227434_at	WBSCR17	-0,437449947	-1,100943374	0,663493427
208452_x_at	MYO9B	-0,437449947	-1,100943374	0,663493427
1556835_s_at	LOC100652770	-0,437449947	-1,100943374	0,663493427
222812_s_at	RHOF	2,053550345	1,390070295	0,66348005
223301_s_at	CCDC82	2,836219929	2,173028091	0,663191838
216331_at	ITGA7	0,244681185	-0,418248858	0,662930043
231153_at	C16orf86	0,244681185	-0,418248858	0,662930043
242365_at	-	0,244681185	-0,418248858	0,662930043
223242_s_at	MFSD11	2,597647262	1,935144844	0,662502418
241841_at	-	0,001114523	-0,661178575	0,662293097
215849_x_at	TTC18	0,001114523	-0,661178575	0,662293097
1554219_at	-	0,001114523	-0,661178575	0,662293097
210219_at	SP100	0,001114523	-0,661178575	0,662293097
228388_at	NFKBIB	0,001114523	-0,661178575	0,662293097

241003_at	-	0,001114523	-0,661178575	0,662293097
233935_at	TNFSF14	0,001114523	-0,661178575	0,662293097
244865_at	HAX1	0,001114523	-0,661178575	0,662293097
222419_x_at	UBE2H	0,001114523	-0,661178575	0,662293097
200087_s_at	TMED2	5,785429149	5,123206007	0,662223143
209139_s_at	PRKRA	4,123563902	3,461380025	0,662183878
221427_s_at	CCNL2	2,829929672	2,167793428	0,662136244
1554414_a_at	OSGIN2	2,248155141	1,586121802	0,662033339
231953_at	BPTF	1,047341799	0,385582632	0,661759167
227747_at	MPZL3	2,16969527	1,508107415	0,661587854
226967_at	FIZ1	1,544940605	0,883374248	0,661566357
204477_at	RABIF	1,910557155	1,249194639	0,661362516
1555041_a_at	NAGA	2,966903803	2,305621062	0,661282741
218397_at	FANCL	4,548572168	3,887370283	0,661201886
238949_at	RNF145	3,204847647	2,543655878	0,661191769
203109_at	UBE2M	3,87876941	3,217691783	0,661077627
222424_s_at	NUCKS1	4,263008992	3,601940721	0,661068271
1552287_s_at	AFG3L1P	2,312738346	1,651676651	0,661061695
34406_at	PACS2	1,837610486	1,176634449	0,660976037
217830_s_at	NSFL1C	2,127965541	1,46719264	0,660772902
216224_s_at	HDAC6	0,458905032	-0,201789721	0,660694753
210705_s_at	TRIM5	1,716003175	1,055317091	0,660686083
223950_s_at	FLYWCH1	1,716003175	1,055317091	0,660686083
204008_at	DNAL4	1,816938997	1,156599976	0,660339021
218525_s_at	HIF1AN	1,326463531	0,666486137	0,659977394
210006_at	ABHD14A	3,071179046	2,411206536	0,659972511
224856_at	FKBP5	3,720319513	3,060577077	0,659742436
209595_at	GTF2F2	2,085817135	1,426280426	0,659536709
1554577_a_at	PSMD10	4,592740505	3,933228921	0,659511584
229211_at	DUSP28	3,045068376	2,385765892	0,659302484
229086_at	C1orf213	0,648195588	-0,011001236	0,659196824
206034_at	SERPINB8	0,791908897	0,133104519	0,658804378
1563295_at	-	-0,230432956	-0,889222211	0,658789255
223636_at	ZMYND12	-0,230432956	-0,889222211	0,658789255
236530_at	HS1BP3	-0,230432956	-0,889222211	0,658789255
234499_at	-	-0,230432956	-0,889222211	0,658789255
1558897_at	PLK5	-0,230432956	-0,889222211	0,658789255
209855_s_at	KLK2	-0,230432956	-0,889222211	0,658789255
1558975_at	FAM100A	-0,230432956	-0,889222211	0,658789255
205377_s_at	ACHE	-0,230432956	-0,889222211	0,658789255
208386_x_at	DMC1	-0,230432956	-0,889222211	0,658789255
223979_x_at	FTCD	-0,230432956	-0,889222211	0,658789255
219359_at	ATHL1	-0,230432956	-0,889222211	0,658789255
210399_x_at	FUT6	0,20740896	-0,45121326	0,65862222
202224_at	CRK	2,339581693	1,680963921	0,658617772
218911_at	YEATS4	3,248151517	2,589579418	0,658572098
204098_at	RBMX2	2,836219929	2,177710147	0,658509782
205263_at	BCL10	4,005144537	3,346715127	0,658429409
225152_at	ZNF622	3,876052392	3,217691783	0,658360609
1563872_at	LOC284395	1,024284941	0,366031214	0,658253727

225409_at	COA5	2,860630824	2,20251271	0,658118115
205295_at	CKMT2	-0,66934516	-1,32740143	0,65805627
239550_at	RORA	-0,66934516	-1,32740143	0,65805627
239381_at	KLK7	-0,66934516	-1,32740143	0,65805627
1555468_at	NRP2	-0,66934516	-1,32740143	0,65805627
1555632_at	PIK3IP1	-0,66934516	-1,32740143	0,65805627
239632_at	-	-0,66934516	-1,32740143	0,65805627
1566949_at	-	-0,66934516	-1,32740143	0,65805627
224040_at	TTTY5	-0,66934516	-1,32740143	0,65805627
1557850_at	INHBA-AS1	-0,66934516	-1,32740143	0,65805627
228104_at	PLXNA4	-0,66934516	-1,32740143	0,65805627
1562946_at	-	-0,66934516	-1,32740143	0,65805627
204043_at	TCN2	-0,66934516	-1,32740143	0,65805627
231595_at	MRVI1-AS1	-0,66934516	-1,32740143	0,65805627
220426_at	C20orf195	-0,66934516	-1,32740143	0,65805627
216919_at	TP53I11	-0,66934516	-1,32740143	0,65805627
227726_at	RNF166	1,936380191	1,278654284	0,657725908
221732_at	CANT1	3,102023286	2,444951949	0,657071337
212671_s_at	HLA-DQA1 /// HLA	6,476257231	5,819490407	0,656766824
242584_at	FAM161A	2,019346222	1,362900801	0,656445421
202984_s_at	BAG5	2,460890032	1,804519951	0,656370082
212207_at	MED13L	2,460890032	1,804519951	0,656370082
209009_at	ESD	6,294566555	5,638252745	0,656313809
228127_at	KCNK3	0,425036312	-0,231045907	0,656082219
64883_at	MOSPD2	1,864507317	1,208437866	0,65606945
1555705_a_at	CMTM3	3,029186714	2,373249966	0,655936749
212616_at	CHD9	2,920736393	2,264929589	0,655806804
201819_at	SCARB1	2,107136847	1,451507543	0,655629304
221472_at	SERINC3	3,921953088	3,266431559	0,655521529
201790_s_at	DHCR7	3,09217775	2,436714502	0,655463247
203128_at	SPTLC2	0,900065578	0,244646697	0,655418881
1553397_at	CCDC13	0,900065578	0,244646697	0,655418881
1569415_at	-	1,116462765	0,461385738	0,655077027
226513_at	ASB7	2,138605583	1,483698568	0,654907015
228937_at	LACC1	0,618277321	-0,036543687	0,654821008
229879_at	-	0,618277321	-0,036543687	0,654821008
1562081_a_at	LINC00424	0,618277321	-0,036543687	0,654821008
233476_at	-	0,618277321	-0,036543687	0,654821008
204725_s_at	NCK1	3,340803206	2,686001625	0,654801582
239801_at	RNF40	0,764143511	0,109458907	0,654684604
212949_at	NCAPH	3,517785666	2,86314199	0,654643676
212284_x_at	TPT1	8,503789334	7,849231851	0,654557484
213995_at	ATP5S	2,811067138	2,157148588	0,653918549
1568784_at	-	-2,250580859	-2,904470894	0,653890035
1554905_x_at	FRMD8	1,287793882	0,634301128	0,653492754
205167_s_at	CDC25C	2,582572508	1,929092307	0,653480201
201139_s_at	SSB	4,142807036	3,489363371	0,653443664
212449_s_at	LYPLA1	6,010561016	5,357184736	0,65337628
205508_at	SCN1B	-0,043925712	-0,697182596	0,653256885
219621_at	CLSPN	-0,043925712	-0,697182596	0,653256885

244071_at	FBLL1	-0,043925712	-0,697182596	0,653256885
1566003_x_at	-	-0,043925712	-0,697182596	0,653256885
230279_at	LOC100507540	-0,043925712	-0,697182596	0,653256885
206580_s_at	EFEMP2	-0,043925712	-0,697182596	0,653256885
1554930_a_at	FUT8	2,692288672	2,039063441	0,65322523
241200_x_at	-	-1,982324124	-2,635549085	0,653224961
235564_at	ZNF117	-1,982324124	-2,635549085	0,653224961
235465_at	FAM123A	-1,982324124	-2,635549085	0,653224961
206033_s_at	DSC3	-1,982324124	-2,635549085	0,653224961
234815_at	-	-1,982324124	-2,635549085	0,653224961
230343_at	-	-1,982324124	-2,635549085	0,653224961
240614_at	KCNC2	-1,982324124	-2,635549085	0,653224961
221365_at	MLNR	-1,982324124	-2,635549085	0,653224961
226701_at	GJA5	-1,982324124	-2,635549085	0,653224961
241179_at	-	-1,982324124	-2,635549085	0,653224961
1553037_a_at	SYN2	-1,982324124	-2,635549085	0,653224961
236853_at	TEX29	-1,982324124	-2,635549085	0,653224961
215000_s_at	FEZ2	3,446505209	2,793634788	0,652870421
205379_at	CBR3	3,223556135	2,570802332	0,652753803
208391_s_at	GLP1R	-1,06282519	-1,715576125	0,652750935
209454_s_at	TEAD3	-1,06282519	-1,715576125	0,652750935
236595_at	LOC100507307	-1,06282519	-1,715576125	0,652750935
1552261_at	WFDC2	-1,06282519	-1,715576125	0,652750935
237862_at	LOC100506189	-1,06282519	-1,715576125	0,652750935
1563484_at	-	-1,06282519	-1,715576125	0,652750935
1569807_at	-	-1,06282519	-1,715576125	0,652750935
237397_at	-	-1,06282519	-1,715576125	0,652750935
1553058_at	GIPC3	-1,06282519	-1,715576125	0,652750935
1566484_at	-	-1,06282519	-1,715576125	0,652750935
1553044_at	GJA10	-1,06282519	-1,715576125	0,652750935
228533_at	-	-1,06282519	-1,715576125	0,652750935
236970_s_at	BCAP29	-1,06282519	-1,715576125	0,652750935
233283_at	-	-1,06282519	-1,715576125	0,652750935
1555897_at	-	-1,06282519	-1,715576125	0,652750935
244884_at	LOC100507150	-1,06282519	-1,715576125	0,652750935
233987_at	TFAP2D	-1,06282519	-1,715576125	0,652750935
1562765_at	WWTR1-AS1	-1,06282519	-1,715576125	0,652750935
225182_at	TMEM50B	3,442765386	2,790111983	0,652653403
208506_at	HIST1H3A /// HIST	-0,49284695	-1,145057014	0,652210064
236166_at	LOC285147	-0,49284695	-1,145057014	0,652210064
203499_at	EPHA2	-0,49284695	-1,145057014	0,652210064
224393_s_at	CECR6	-0,49284695	-1,145057014	0,652210064
211830_s_at	CACNA1I	-0,49284695	-1,145057014	0,652210064
220179_at	DPEP3	-0,49284695	-1,145057014	0,652210064
227145_at	LOXL4	-0,49284695	-1,145057014	0,652210064
237835_at	-	-0,49284695	-1,145057014	0,652210064
219145_at	LPHN1	-0,49284695	-1,145057014	0,652210064
206138_s_at	PI4KB	3,166767593	2,514786608	0,651980985
228523_at	NANOS1	0,39105295	-0,260526297	0,651579247
1553444_a_at	C1orf127	0,39105295	-0,260526297	0,651579247

235024_at	PHF17	1,910557155	1,259081178	0,651475977
219039_at	SEMA4C	1,910557155	1,259081178	0,651475977
229828_at	CDC73	1,910557155	1,259081178	0,651475977
225658_at	SPOPL	3,771154436	3,119733444	0,651420992
212343_at	YIPF6	1,561816652	0,910483921	0,651332732
244534_at	-	1,561816652	0,910483921	0,651332732
219736_at	TRIM36	-0,280273599	-0,931539147	0,651265548
208423_s_at	MSR1	-0,280273599	-0,931539147	0,651265548
239075_at	-	-0,280273599	-0,931539147	0,651265548
219325_s_at	ELAC1	-0,280273599	-0,931539147	0,651265548
240264_at	-	-0,280273599	-0,931539147	0,651265548
212472_at	MICAL2	-0,280273599	-0,931539147	0,651265548
231302_at	-	0,58788177	-0,063377083	0,651258853
214442_s_at	PIAS2	1,493647653	0,842599219	0,651048434
209478_at	STRA13	4,533526699	3,882991815	0,650534884
219340_s_at	CLN8	0,735999505	0,085534992	0,650464513
209110_s_at	RGL2	1,610353504	0,960042218	0,650311285
229325_at	ZZZ3	1,948628481	1,298349073	0,650279407
206860_s_at	MIOS	2,56794744	1,917743773	0,650203666
237996_at	-	-1,212652659	-1,862269442	0,649616782
1562904_s_at	FLJ10661	-1,212652659	-1,862269442	0,649616782
223453_s_at	ATL3	-1,212652659	-1,862269442	0,649616782
207710_at	LCE2B	-1,212652659	-1,862269442	0,649616782
242720_at	ITIH4	-1,212652659	-1,862269442	0,649616782
231476_at	-	-1,212652659	-1,862269442	0,649616782
243248_at	-	-1,212652659	-1,862269442	0,649616782
1566044_at	-	-1,212652659	-1,862269442	0,649616782
215225_s_at	GPR17	-1,212652659	-1,862269442	0,649616782
205317_s_at	SLC15A2	-1,212652659	-1,862269442	0,649616782
1553752_at	SPATA25	-1,212652659	-1,862269442	0,649616782
244709_at	FAM123C	-1,212652659	-1,862269442	0,649616782
244882_at	TNRC18	-1,212652659	-1,862269442	0,649616782
233125_at	-	-1,212652659	-1,862269442	0,649616782
1555251_a_at	OTOF	-1,212652659	-1,862269442	0,649616782
229927_at	LEMD1	-1,212652659	-1,862269442	0,649616782
230988_at	ADD2	-1,212652659	-1,862269442	0,649616782
234687_x_at	LIMD1	1,267146969	0,61779983	0,649347139
242601_at	HEPACAM2	-2,074223907	-2,723114724	0,648890817
233025_at	PDZD2	-2,074223907	-2,723114724	0,648890817
240120_at	-	-2,074223907	-2,723114724	0,648890817
216328_at	SIGLEC8	-2,074223907	-2,723114724	0,648890817
230420_at	CASZ1	-2,074223907	-2,723114724	0,648890817
208908_s_at	CAST	3,426291319	2,777528602	0,648762717
221763_at	JMJD1C	3,8018113	3,153389283	0,648422017
200068_s_at	CANX	6,272140316	5,62373163	0,648408685
212085_at	SLC25A6	6,648321363	6,00007453	0,648246833
244246_at	MIPOL1	-1,537851782	-2,186086603	0,648234821
239357_at	ATP2B2	-1,537851782	-2,186086603	0,648234821
1556682_s_at	-	-1,537851782	-2,186086603	0,648234821
222564_at	POGK	-1,537851782	-2,186086603	0,648234821

1559203_s_at	KRAS	-1,537851782	-2,186086603	0,648234821
239510_at	-	-1,537851782	-2,186086603	0,648234821
1552979_at	LINC00471	-1,537851782	-2,186086603	0,648234821
1568685_at	-	-1,537851782	-2,186086603	0,648234821
242165_at	LRIG2	-1,537851782	-2,186086603	0,648234821
231179_at	IP6K3	-1,537851782	-2,186086603	0,648234821
1563082_at	LINC00486	-1,537851782	-2,186086603	0,648234821
243962_at	-	-1,537851782	-2,186086603	0,648234821
226899_at	UNC5B	-1,537851782	-2,186086603	0,648234821
211880_x_at	PCDHGA1	-1,537851782	-2,186086603	0,648234821
227995_at	-	-1,537851782	-2,186086603	0,648234821
241766_at	LOC100509303	-1,537851782	-2,186086603	0,648234821
236014_at	MKX	-1,537851782	-2,186086603	0,648234821
220539_at	TTC40	-1,537851782	-2,186086603	0,648234821
230975_at	-	-1,537851782	-2,186086603	0,648234821
215324_at	SEMA3D	-1,537851782	-2,186086603	0,648234821
211390_at	N4BP2L1	-1,537851782	-2,186086603	0,648234821
222783_s_at	SMOC1	-1,537851782	-2,186086603	0,648234821
239492_at	SEC14L4	-1,537851782	-2,186086603	0,648234821
217278_x_at	-	-1,537851782	-2,186086603	0,648234821
216904_at	LOC100652958	-1,537851782	-2,186086603	0,648234821
239455_at	-	-1,537851782	-2,186086603	0,648234821
1557444_at	TREML3P	-1,537851782	-2,186086603	0,648234821
202614_at	SLC30A9	3,945594088	3,297572471	0,648021618
225799_at	LINC00152 /// LOC	4,636598653	3,988637612	0,647961041
208757_at	TMED9	3,89798617	3,250322783	0,647663387
205212_s_at	ACAP1	1,476175815	0,829309537	0,646866278
226227_x_at	ZNFX1-AS1	6,788295624	6,14144262	0,646853004
209375_at	XPC	3,209439373	2,562621495	0,646817878
225559_at	C3orf19	2,063971513	1,417157926	0,646813587
222694_at	LOC100287195 ///	1,071336699	0,424693306	0,646643393
236991_at	-	0,355751005	-0,290694975	0,64644598
1553340_s_at	SLC35G3 /// SLC35	0,355751005	-0,290694975	0,64644598
223982_s_at	PNPLA8	3,190097062	2,543655878	0,646441184
218783_at	INTS7	2,16969527	1,523429058	0,646266211
1559161_at	-	0,556428218	-0,089635713	0,646063931
1565604_at	PWP2	0,127732831	-0,518232988	0,645965819
213204_at	CUL9	1,160996507	0,515457602	0,645538905
207226_at	HIST1H2BN	-0,088974936	-0,734355396	0,64538046
234446_at	-	-0,088974936	-0,734355396	0,64538046
205679_x_at	ACAN	-0,088974936	-0,734355396	0,64538046
1564109_at	LOC284865	-0,088974936	-0,734355396	0,64538046
213096_at	TMCC2	-0,088974936	-0,734355396	0,64538046
202841_x_at	OGFR	2,053550345	1,408220608	0,645329737
1561550_at	-	-1,624955693	-2,27001641	0,645060717
243056_at	C12orf60	-1,624955693	-2,27001641	0,645060717
240086_at	VPS36	-1,624955693	-2,27001641	0,645060717
237844_at	LOC100506639 ///	-1,624955693	-2,27001641	0,645060717
1554108_at	-	-1,624955693	-2,27001641	0,645060717
240275_at	ARMC3	-1,624955693	-2,27001641	0,645060717

1556153_s_at	-	-1,624955693	-2,27001641	0,645060717
224038_at	TIMM23	-1,624955693	-2,27001641	0,645060717
227958_s_at	GSN	-1,624955693	-2,27001641	0,645060717
239894_at	LOC100128511	-1,624955693	-2,27001641	0,645060717
220506_at	GUCY1B2	-1,624955693	-2,27001641	0,645060717
229030_at	CAPN8	-1,624955693	-2,27001641	0,645060717
211648_at	IGHG1 /// IGHM	-1,624955693	-2,27001641	0,645060717
1553465_a_at	CES5A	-1,624955693	-2,27001641	0,645060717
235251_at	-	-1,624955693	-2,27001641	0,645060717
1553089_a_at	WFDC2	-1,624955693	-2,27001641	0,645060717
243220_at	-	-1,624955693	-2,27001641	0,645060717
224550_s_at	MRVI1	-1,624955693	-2,27001641	0,645060717
233200_at	-	-1,624955693	-2,27001641	0,645060717
241117_at	LOXHD1	-1,624955693	-2,27001641	0,645060717
238466_at	-	-1,624955693	-2,27001641	0,645060717
238344_at	-	-1,624955693	-2,27001641	0,645060717
220491_at	HAMP	-1,624955693	-2,27001641	0,645060717
243541_at	IL31RA	-1,624955693	-2,27001641	0,645060717
234408_at	IL17F	-1,624955693	-2,27001641	0,645060717
231712_at	ASCL5	-0,924000698	-1,568385657	0,644384959
207698_at	C6orf123	-0,924000698	-1,568385657	0,644384959
209924_at	CCL18	-0,924000698	-1,568385657	0,644384959
1569269_s_at	SRGAP1	-0,924000698	-1,568385657	0,644384959
233669_s_at	TRIM54	-0,924000698	-1,568385657	0,644384959
1558960_a_at	-	-0,924000698	-1,568385657	0,644384959
230863_at	LRP2	-0,924000698	-1,568385657	0,644384959
233526_at	-	-0,924000698	-1,568385657	0,644384959
1561995_at	-	-0,924000698	-1,568385657	0,644384959
236849_at	VTI1A	-0,924000698	-1,568385657	0,644384959
1564165_at	LOC100630923	-0,924000698	-1,568385657	0,644384959
234449_at	-	-0,924000698	-1,568385657	0,644384959
1563077_at	LOC100289058	-0,924000698	-1,568385657	0,644384959
217865_at	RNF130	-0,924000698	-1,568385657	0,644384959
217875_s_at	PMEPA1	-0,924000698	-1,568385657	0,644384959
236009_at	PERP	-0,924000698	-1,568385657	0,644384959
224614_at	DYNC1LI2	2,127965541	1,483698568	0,644266973
208809_s_at	C6orf62	5,362941058	4,718691404	0,644249654
241739_at	OGFOD1	-1,452713826	-2,09678811	0,644074284
234071_at	DEPTOR	-1,452713826	-2,09678811	0,644074284
213456_at	SOSTDC1	-1,452713826	-2,09678811	0,644074284
232152_at	CEP57L1	-1,452713826	-2,09678811	0,644074284
1559634_at	CHRM3	-1,452713826	-2,09678811	0,644074284
1569699_at	AK7	-1,452713826	-2,09678811	0,644074284
240899_at	-	-1,452713826	-2,09678811	0,644074284
1565635_at	-	-1,452713826	-2,09678811	0,644074284
225817_at	CGNL1	-1,452713826	-2,09678811	0,644074284
1558273_a_at	-	-1,452713826	-2,09678811	0,644074284
206488_s_at	CD36	-1,452713826	-2,09678811	0,644074284
1556578_a_at	FLJ31945	-1,452713826	-2,09678811	0,644074284
216346_at	SEC14L3	-1,452713826	-2,09678811	0,644074284

215080_s_at	AGAP2	-1,452713826	-2,09678811	0,644074284
234060_at	-	-1,452713826	-2,09678811	0,644074284
1554970_at	PDILT	-1,452713826	-2,09678811	0,644074284
235754_at	HFE	-1,452713826	-2,09678811	0,644074284
1552830_at	FBXO39	-1,452713826	-2,09678811	0,644074284
207066_at	HRC	-1,452713826	-2,09678811	0,644074284
237715_at	-	-1,452713826	-2,09678811	0,644074284
240675_at	-	-1,452713826	-2,09678811	0,644074284
1561223_at	-	-1,452713826	-2,09678811	0,644074284
231778_at	DLX3	-1,452713826	-2,09678811	0,644074284
237756_at	KLHL23 /// PHOSPI	-1,452713826	-2,09678811	0,644074284
210339_s_at	KLK2	-1,452713826	-2,09678811	0,644074284
203424_s_at	IGFBP5	-1,452713826	-2,09678811	0,644074284
234470_at	-	-1,452713826	-2,09678811	0,644074284
243152_at	COPS8	-1,452713826	-2,09678811	0,644074284
210359_at	MTSS1	-1,452713826	-2,09678811	0,644074284
221042_s_at	CLMN	-1,452713826	-2,09678811	0,644074284
208946_s_at	BECN1	4,165622052	3,521590309	0,644031742
223852_s_at	STK40	1,745502609	1,101780212	0,643722397
226425_at	CLIP4	1,686984721	1,043469112	0,643515609
219485_s_at	PSMD10	5,36737436	4,723859191	0,64351517
225318_at	-	3,960533133	3,317257552	0,643275581
1560758_at	-	-2,163157732	-2,8064286	0,643270868
241612_at	FOXD3	-2,163157732	-2,8064286	0,643270868
239089_at	-	-2,163157732	-2,8064286	0,643270868
237812_at	-	-1,893506789	-2,536339282	0,642832493
230861_at	DKFZP434L187	-1,893506789	-2,536339282	0,642832493
211766_s_at	PNLIPRP2	-1,893506789	-2,536339282	0,642832493
233335_at	-	-1,893506789	-2,536339282	0,642832493
229118_at	PRRG3	-1,893506789	-2,536339282	0,642832493
236639_at	LOC100507596	-1,893506789	-2,536339282	0,642832493
1565746_at	LOC100132815	-1,893506789	-2,536339282	0,642832493
230817_at	-	-1,893506789	-2,536339282	0,642832493
239787_at	KCTD4	-1,893506789	-2,536339282	0,642832493
203505_at	ABCA1	-1,893506789	-2,536339282	0,642832493
205378_s_at	ACHE	-1,893506789	-2,536339282	0,642832493
206616_s_at	ADAM22	-1,893506789	-2,536339282	0,642832493
206296_x_at	MAP4K1	2,190140919	1,547376668	0,642764251
207500_at	CASP5	-0,730013898	-1,372734086	0,642720187
1553300_a_at	DGKH	-0,730013898	-1,372734086	0,642720187
1554646_at	OSBPL1A	-0,730013898	-1,372734086	0,642720187
1555661_at	OR8D1	-0,730013898	-1,372734086	0,642720187
207280_at	RNF185-AS1	-0,730013898	-1,372734086	0,642720187
237327_at	APH1A	-0,730013898	-1,372734086	0,642720187
239152_at	-	-0,730013898	-1,372734086	0,642720187
242548_x_at	ANKRD37	-0,730013898	-1,372734086	0,642720187
243120_at	-	-0,730013898	-1,372734086	0,642720187
237366_at	-	-0,730013898	-1,372734086	0,642720187
244752_at	ZNF438	-0,730013898	-1,372734086	0,642720187
1557476_at	PFN4	-0,730013898	-1,372734086	0,642720187

1557590_at	PARD6G-AS1	-0,730013898	-1,372734086	0,642720187
234911_at	-	-0,730013898	-1,372734086	0,642720187
1557755_at	CEP128	-0,730013898	-1,372734086	0,642720187
205391_x_at	ANK1	-0,730013898	-1,372734086	0,642720187
225762_x_at	RN45S	2,118167848	1,475630127	0,642537721
203738_at	C5orf22	3,132805465	2,490640503	0,642164962
1554465_s_at	ZNF673 /// ZNF674	0,820160788	0,178191865	0,641968923
203835_at	LRRC32	0,820160788	0,178191865	0,641968923
222980_at	RAB10	5,954907677	5,312950049	0,641957628
203101_s_at	MGAT2	0,319287178	-0,322340048	0,641627226
214840_at	TOM1L2	0,319287178	-0,322340048	0,641627226
229540_at	RBPJ	1,225123479	0,583607503	0,641515976
231895_at	SASS6	1,830468611	1,189078962	0,641389649
227770_at	COG8	1,830468611	1,189078962	0,641389649
215732_s_at	DTX2 /// LOC1006	0,524545436	-0,116767475	0,641312911
214125_s_at	NENF	0,08619576	-0,554696666	0,640892426
229449_at	-	0,08619576	-0,554696666	0,640892426
203435_s_at	MME	0,08619576	-0,554696666	0,640892426
202913_at	ARHGEF11	0,08619576	-0,554696666	0,640892426
217930_s_at	TOLLIP	1,307201325	0,666486137	0,640715188
212000_at	SUGP2	1,307201325	0,666486137	0,640715188
210153_s_at	ME2	4,450465038	3,809801648	0,64066339
216832_at	RUNX1T1	-0,332405896	-0,972893339	0,640487443
1553131_a_at	GATA4	-0,332405896	-0,972893339	0,640487443
1554030_at	ARSB	-0,332405896	-0,972893339	0,640487443
220522_at	CRB1	-0,332405896	-0,972893339	0,640487443
241125_at	-	-0,332405896	-0,972893339	0,640487443
229770_at	GLT1D1	-0,332405896	-0,972893339	0,640487443
228232_s_at	VSIG2	-0,332405896	-0,972893339	0,640487443
207940_x_at	CNR1	-0,332405896	-0,972893339	0,640487443
1553832_at	LINC00315	-0,332405896	-0,972893339	0,640487443
216975_x_at	NPAS1	-0,332405896	-0,972893339	0,640487443
211552_s_at	ALDH4A1	-0,332405896	-0,972893339	0,640487443
215685_s_at	DLX2	-0,332405896	-0,972893339	0,640487443
227068_at	LOC100652805 ///	4,733987652	4,093744039	0,640243613
226181_at	TUBE1	3,442765386	2,802594885	0,640170501
203519_s_at	UPF2	3,521615383	2,881480309	0,640135075
210802_s_at	DIMT1	3,828857041	3,188806254	0,640050786
1569713_at	SEC24B-AS1	0,677745787	0,03784737	0,639898417
223419_at	FBXW9	0,677745787	0,03784737	0,639898417
227937_at	MYPOP	0,677745787	0,03784737	0,639898417
41469_at	PI3	-1,491729473	-2,131609629	0,639880156
226896_at	CHCHD1	5,129300304	4,489549662	0,639750642
208693_s_at	GARS	6,521849682	5,882267754	0,639581928
45687_at	PRR14	3,529012338	2,889432229	0,639580108
236503_at	-	-0,551284523	-1,19085694	0,639572417
236726_at	RGS6	-0,551284523	-1,19085694	0,639572417
236151_at	SRRM4	-0,551284523	-1,19085694	0,639572417
230555_s_at	MED30	-0,551284523	-1,19085694	0,639572417
207352_s_at	GABRB2	-0,551284523	-1,19085694	0,639572417

211868_x_at	IGH@ /// IGHA1 //	-0,551284523	-1,19085694	0,639572417
1556947_at	-	-0,551284523	-1,19085694	0,639572417
216560_x_at	IGLC1	-0,551284523	-1,19085694	0,639572417
237861_at	LOC100506105	-0,551284523	-1,19085694	0,639572417
214535_s_at	ADAMTS2	-0,551284523	-1,19085694	0,639572417
222565_s_at	PRKD3	2,621163856	1,981728801	0,639435055
230592_at	NSL1	1,816938997	1,178100917	0,63883808
226842_at	FBXL20	2,138605583	1,500059916	0,638545667
201520_s_at	GRSF1	4,870498291	4,232076746	0,638421545
218757_s_at	UPF3B	4,68949381	4,051142838	0,638350972
229350_x_at	PARP10	1,936380191	1,298349073	0,638031118
1553715_s_at	FAM195A	3,223556135	2,585619776	0,637936359
234996_at	CALCRL	-1,712732543	-2,350444759	0,637712216
232600_at	-	-1,712732543	-2,350444759	0,637712216
230636_s_at	KLF9	-1,712732543	-2,350444759	0,637712216
1565949_x_at	CHML	-1,712732543	-2,350444759	0,637712216
1558493_at	CSTT	-1,712732543	-2,350444759	0,637712216
211812_s_at	B3GALNT1	-1,712732543	-2,350444759	0,637712216
1558862_at	NBPF5	-1,712732543	-2,350444759	0,637712216
239529_at	C5orf20 /// TIFAB	-1,712732543	-2,350444759	0,637712216
215912_at	GNAO1	-1,712732543	-2,350444759	0,637712216
1570320_at	-	-1,712732543	-2,350444759	0,637712216
219415_at	TTYH1	-1,712732543	-2,350444759	0,637712216
239260_at	CORIN	-1,712732543	-2,350444759	0,637712216
206265_s_at	GPLD1	-1,712732543	-2,350444759	0,637712216
230661_at	-	-1,712732543	-2,350444759	0,637712216
218002_s_at	CXCL14	-1,712732543	-2,350444759	0,637712216
219310_at	SYNDIG1	-1,712732543	-2,350444759	0,637712216
244727_at	KCNQ1OT1	-1,712732543	-2,350444759	0,637712216
1557702_at	-	-1,712732543	-2,350444759	0,637712216
1567443_x_at	PSEN1	-1,712732543	-2,350444759	0,637712216
1566557_at	FLJ90757	-1,712732543	-2,350444759	0,637712216
242204_at	WFDC5	-1,712732543	-2,350444759	0,637712216
210152_at	LILRB4	-1,712732543	-2,350444759	0,637712216
202375_at	SEC24D	1,287793882	0,65030602	0,637487862
1570266_x_at	ERVH-1	1,716003175	1,078619386	0,637383788
236294_at	HUWE1	1,116462765	0,479089184	0,637373581
210883_x_at	EFNB3	0,282358733	-0,354781582	0,637140314
222508_s_at	ARGLU1	2,304041045	1,666953618	0,637087428
228992_at	MED28	4,324702656	3,687638429	0,637064227
229663_at	LONP2	0,491906512	-0,144765583	0,636672095
1557315_a_at	-	-0,135065865	-0,771340337	0,636274472
222248_s_at	SIRT4	-0,135065865	-0,771340337	0,636274472
1557342_a_at	-	-0,135065865	-0,771340337	0,636274472
214000_s_at	RGS10	-0,135065865	-0,771340337	0,636274472
204144_s_at	PIGQ	-0,135065865	-0,771340337	0,636274472
204202_at	IQCE	-0,135065865	-0,771340337	0,636274472
228897_at	DERL3	-0,135065865	-0,771340337	0,636274472
212588_at	PTPRC	5,621221374	4,985027501	0,636193873
219104_at	RNF141	2,229377375	1,593605508	0,635771867

202243_s_at	PSMB4	6,95567265	6,320224106	0,635448545
210140_at	CST7	2,866549889	2,23115548	0,635394409
228131_at	ERCC1	2,62869474	1,993309863	0,635384877
241492_at	-	-1,369772723	-2,005029581	0,635256858
237359_at	-	-1,369772723	-2,005029581	0,635256858
1565998_at	LOC286299	-1,369772723	-2,005029581	0,635256858
243018_at	-	-1,369772723	-2,005029581	0,635256858
238403_at	-	-1,369772723	-2,005029581	0,635256858
228376_at	GGTA1P	-1,369772723	-2,005029581	0,635256858
234810_at	ZFP1	-1,369772723	-2,005029581	0,635256858
231751_at	ABCG8	-1,369772723	-2,005029581	0,635256858
220211_at	FLJ13224	-1,369772723	-2,005029581	0,635256858
236099_at	-	-1,369772723	-2,005029581	0,635256858
236657_at	LOC100288911	-1,369772723	-2,005029581	0,635256858
243137_at	-	-1,369772723	-2,005029581	0,635256858
244232_at	-	-1,369772723	-2,005029581	0,635256858
1561732_at	-	-1,369772723	-2,005029581	0,635256858
1557835_at	-	-1,369772723	-2,005029581	0,635256858
1557208_at	LOC219731	-1,369772723	-2,005029581	0,635256858
213948_x_at	CADM3	-1,369772723	-2,005029581	0,635256858
216781_at	KIAA1751	-1,369772723	-2,005029581	0,635256858
1558687_a_at	FOXN1	-1,369772723	-2,005029581	0,635256858
1559289_at	TADA1	-1,369772723	-2,005029581	0,635256858
227816_at	NTN1	-1,369772723	-2,005029581	0,635256858
222002_at	C7orf26	-1,369772723	-2,005029581	0,635256858
220592_at	CCDC40	-1,369772723	-2,005029581	0,635256858
243980_at	ZNF594	-1,369772723	-2,005029581	0,635256858
236292_at	RNF130	-1,369772723	-2,005029581	0,635256858
215057_at	LOC100272228	-1,369772723	-2,005029581	0,635256858
218959_at	HOXC10	-1,369772723	-2,005029581	0,635256858
204559_s_at	LSM7	5,901453744	5,266214489	0,635239255
233864_s_at	VPS35	4,607153971	3,971987733	0,635166238
225877_at	TYSND1	0,648195588	0,013190398	0,63500519
201162_at	IGFBP7	0,648195588	0,013190398	0,63500519
225499_at	RALGAPA2	1,421391637	0,786391549	0,635000088
1007_s_at	DDR1 /// MIR4640	-0,197616913	-0,83249114	0,634874228
205664_at	KIN	3,278243793	2,64347734	0,634766452
226749_at	MRPS9	3,723597116	3,08892491	0,634672206
231110_at	-	0,045517965	-0,589001171	0,634519136
210264_at	GPR35	0,045517965	-0,589001171	0,634519136
201432_at	CAT	5,380783446	4,746319618	0,634463827
223119_s_at	USP47	1,544940605	0,910483921	0,634456684
208184_s_at	TRAPPC10	1,000459215	0,366031214	0,634428001
224820_at	COX20	5,049246526	4,414859474	0,634387052
220176_at	NUBPL	1,701542258	1,067365565	0,634176693
227645_at	PIK3R5	2,476788561	1,842852096	0,633936464
209927_s_at	CHTOP	2,476788561	1,842852096	0,633936464
203133_at	SEC61B	6,461704497	5,828181789	0,633522707
212219_at	PSME4	3,831656423	3,198229785	0,633426638
212635_at	TNPO1	3,632502157	2,999351239	0,633150918

218571_s_at	CHMP4A	4,207481512	3,5744736	0,633007912
200809_x_at	RPL12	8,306286779	7,673331408	0,632955371
1555461_at	-	1,182925501	0,550017041	0,632908461
201922_at	NSA2	6,394478178	5,761807207	0,632670971
209033_s_at	DYRK1A	4,693076946	4,060510153	0,632566794
208739_x_at	SUMO2	6,758930488	6,126493341	0,632437146
231764_at	CHRAC1	1,910557155	1,278654284	0,631902872
213687_s_at	RPL35A	7,845117237	7,213249242	0,631867994
206559_x_at	-	9,025177497	8,393453878	0,631723619
218036_x_at	NMD3	3,588695979	2,957089023	0,631606956
214643_x_at	BIN1	0,244681185	-0,386896102	0,631577288
1566425_at	-	0,244681185	-0,386896102	0,631577288
206657_s_at	MYOD1	0,244681185	-0,386896102	0,631577288
203289_s_at	NPRL3	0,244681185	-0,386896102	0,631577288
1552985_at	SLC30A8	-0,384193355	-1,015398016	0,631204661
222577_at	CCDC90B	-0,384193355	-1,015398016	0,631204661
1556247_a_at	LOC100506271	-0,384193355	-1,015398016	0,631204661
224998_at	CMTM4	-0,384193355	-1,015398016	0,631204661
230470_at	DSCR9	-0,384193355	-1,015398016	0,631204661
229082_at	CCDC125	-0,384193355	-1,015398016	0,631204661
1562254_at	-	0,458905032	-0,172194225	0,631099256
231115_at	POLH	0,458905032	-0,172194225	0,631099256
227805_at	METAP1D	0,764143511	0,133104519	0,631038992
1568650_a_at	-	-0,791294935	-1,422257026	0,630962091
1563498_s_at	SLC25A45	-0,791294935	-1,422257026	0,630962091
221119_at	ARHGEF38	-0,791294935	-1,422257026	0,630962091
207398_at	HOXD13	-0,791294935	-1,422257026	0,630962091
241158_at	-	-0,791294935	-1,422257026	0,630962091
231055_at	-	-0,791294935	-1,422257026	0,630962091
236352_at	VGLL2	-0,791294935	-1,422257026	0,630962091
239189_at	CASKIN1	-0,791294935	-1,422257026	0,630962091
228236_at	SLC52A3	-0,791294935	-1,422257026	0,630962091
1561918_at	-	-0,791294935	-1,422257026	0,630962091
221380_at	-	-0,791294935	-1,422257026	0,630962091
240705_at	CYP19A1	-0,791294935	-1,422257026	0,630962091
217567_at	TGM4	-0,791294935	-1,422257026	0,630962091
1564823_at	CDC42BPG	-0,791294935	-1,422257026	0,630962091
220069_at	TUBA8	-0,791294935	-1,422257026	0,630962091
222935_x_at	SLC39A8	-0,791294935	-1,422257026	0,630962091
1555262_a_at	MAGI1	-0,791294935	-1,422257026	0,630962091
238247_at	LOC100506942	-0,791294935	-1,422257026	0,630962091
222539_at	CLN6	-0,791294935	-1,422257026	0,630962091
235941_s_at	FAM224A	-0,791294935	-1,422257026	0,630962091
238816_at	PSEN1	-0,791294935	-1,422257026	0,630962091
235323_at	LOC100499489	-0,180990326	-0,811769547	0,630779221
214407_x_at	GYPB	-0,180990326	-0,811769547	0,630779221
244409_at	CCDC154	-0,180990326	-0,811769547	0,630779221
229424_s_at	ARHGAP27	-0,180990326	-0,811769547	0,630779221
1562776_at	LOC339807	-0,180990326	-0,811769547	0,630779221
241896_at	MACF1	-0,180990326	-0,811769547	0,630779221

31799_at	-	-0,383972088	-1,013756213	0,629784126
204308_s_at	TECPR2	1,402495885	0,772793615	0,62970227
216678_at	IFT122	0,97663406	0,346964736	0,629669324
229050_s_at	SNHG7 /// SNORA:	4,317231729	3,687638429	0,6295933
201155_s_at	MFN2	3,713669011	3,084123325	0,629545685
207108_s_at	NIPBL	1,97249974	1,343200523	0,629299216
217216_x_at	MLH3	1,326463531	0,697221375	0,629242156
228211_at	ERCC6L2	0,873789652	0,244646697	0,629142955
209528_s_at	TELO2	0,873789652	0,244646697	0,629142955
227375_at	ANKRD13C	1,625482993	0,996453274	0,62902972
213411_at	ADAM22	1,246401134	0,61779983	0,628601303
1558102_at	-	1,246401134	0,61779983	0,628601303
224598_at	MGAT4B	4,393737464	3,765267919	0,628469545
214309_s_at	C21orf2	-0,759777233	-1,388218972	0,628441738
225587_at	TMEM129	1,071336699	0,443143223	0,628193476
215747_s_at	RCC1	1,816938997	1,189078962	0,627860035
221379_at	-	1,160996507	0,533258442	0,627738066
222032_s_at	USP7	1,160996507	0,533258442	0,627738066
212818_s_at	ASB1	2,053550345	1,426280426	0,627269919
234107_s_at	DTD1	4,99820644	4,371325911	0,62688053
227218_at	RLTPR	0,425036312	-0,201789721	0,626826033
218215_s_at	NR1H2	0,425036312	-0,201789721	0,626826033
228286_at	GEN1	2,725848647	2,099079406	0,626769241
238002_at	GOLIM4	2,507817181	1,881145546	0,626671635
226515_at	CCDC127	3,204847647	2,578283758	0,626563889
1554927_at	LINC00598	-1,137208348	-1,763664074	0,626455726
241481_at	FAM81A	-1,137208348	-1,763664074	0,626455726
1562267_s_at	ZNF709	-1,137208348	-1,763664074	0,626455726
1569758_at	-	-1,137208348	-1,763664074	0,626455726
1561097_at	-	-1,137208348	-1,763664074	0,626455726
215466_at	-	-1,137208348	-1,763664074	0,626455726
235712_at	GAS5-AS1	-1,137208348	-1,763664074	0,626455726
1552425_a_at	KLHL10	-1,137208348	-1,763664074	0,626455726
243906_at	-	-1,137208348	-1,763664074	0,626455726
244218_at	-	-1,137208348	-1,763664074	0,626455726
1560513_at	LOC400568	-1,137208348	-1,763664074	0,626455726
214886_s_at	N4BP2L1	-1,137208348	-1,763664074	0,626455726
206803_at	PDYN	-1,137208348	-1,763664074	0,626455726
244178_at	COMMD7	-1,137208348	-1,763664074	0,626455726
1563078_at	-	-1,137208348	-1,763664074	0,626455726
1552903_at	B4GALNT2	-1,137208348	-1,763664074	0,626455726
206974_at	CXCR6	-1,137208348	-1,763664074	0,626455726
221289_at	DLX6	-1,137208348	-1,763664074	0,626455726
213488_at	SNED1	-1,137208348	-1,763664074	0,626455726
224263_x_at	ZAN	-1,137208348	-1,763664074	0,626455726
234663_at	-	-1,137208348	-1,763664074	0,626455726
242271_at	SLC26A9	-1,137208348	-1,763664074	0,626455726
213604_at	TCEB3	2,537731447	1,911502437	0,626229011
1569940_at	SLC6A16	-0,609040214	-1,234835326	0,625795112
236358_at	-	-0,609040214	-1,234835326	0,625795112

244658_at	-	-0,609040214	-1,234835326	0,625795112
1564784_at	-	-0,609040214	-1,234835326	0,625795112
240573_at	LOC374443	-0,609040214	-1,234835326	0,625795112
237513_at	PRSS58	-0,609040214	-1,234835326	0,625795112
210780_at	ESR2	-0,609040214	-1,234835326	0,625795112
222054_at	PPIEL	-0,609040214	-1,234835326	0,625795112
236406_at	ZNF324B	-0,609040214	-1,234835326	0,625795112
1560550_at	-	-0,609040214	-1,234835326	0,625795112
219589_s_at	TMEM143	-0,609040214	-1,234835326	0,625795112
1554914_at	PLA2G4D	-0,609040214	-1,234835326	0,625795112
235217_at	LOC100216546	-0,609040214	-1,234835326	0,625795112
1555631_at	-	-0,609040214	-1,234835326	0,625795112
1552724_at	RHOXF1	-0,609040214	-1,234835326	0,625795112
230267_at	-	-0,609040214	-1,234835326	0,625795112
225275_at	EDIL3	-1,802716385	-2,4284334	0,625717015
228885_at	MAMDC2	-1,802716385	-2,4284334	0,625717015
240821_at	-	-1,802716385	-2,4284334	0,625717015
215673_at	TEF	-1,802716385	-2,4284334	0,625717015
240386_at	-	-1,802716385	-2,4284334	0,625717015
233542_at	-	-1,802716385	-2,4284334	0,625717015
243577_at	-	-1,802716385	-2,4284334	0,625717015
202992_at	C7	-1,802716385	-2,4284334	0,625717015
234543_at	-	-1,802716385	-2,4284334	0,625717015
1559641_at	-	-1,802716385	-2,4284334	0,625717015
239733_at	DYDC2	-1,802716385	-2,4284334	0,625717015
222255_at	PRX	-1,802716385	-2,4284334	0,625717015
216566_at	CKAP2	-1,802716385	-2,4284334	0,625717015
1554449_at	MIER3	-1,802716385	-2,4284334	0,625717015
219768_at	VTCN1	-1,802716385	-2,4284334	0,625717015
217281_x_at	IGH@ /// IGHA1 //	-1,802716385	-2,4284334	0,625717015
221730_at	COL5A2	-1,802716385	-2,4284334	0,625717015
1567558_at	TREML4	-1,802716385	-2,4284334	0,625717015
242830_at	-	-1,802716385	-2,4284334	0,625717015
219655_at	C7orf10	-1,802716385	-2,4284334	0,625717015
1553148_a_at	SNX13	0,20740896	-0,418248858	0,625657818
205313_at	HNF1B	0,20740896	-0,418248858	0,625657818
1552944_a_at	PANX2	0,20740896	-0,418248858	0,625657818
208661_s_at	TTC3 /// TTC3P1	4,466417603	3,84088223	0,625535374
200693_at	YWHAQ	7,494437779	6,86893882	0,625498959
240854_x_at	-	0,95222556	0,326777209	0,625448351
218125_s_at	CCDC25	3,007119335	2,381744287	0,625375048
228606_at	TCTEX1D2	3,716897585	3,091548197	0,625349388
210088_x_at	MYL4	1,307201325	0,681874787	0,625326538
228517_at	MEAF6	0,847529938	0,222432814	0,625097124
1570070_at	C20orf160	0,847529938	0,222432814	0,625097124
227581_at	TECPR1	1,440265662	0,815200271	0,625065391
213623_at	KIF3A	2,276736537	1,651676651	0,625059886
211185_s_at	SF3B1	6,325730699	5,700836504	0,624894195
221570_s_at	METTL5	5,74282607	5,118000298	0,624825772
218844_at	ACSF2	1,802733148	1,178100917	0,62463223

210826_x_at	RAD17	3,414594082	2,790111983	0,624482098
229865_at	FNDC3B	0,001114523	-0,623254098	0,624368621
215439_x_at	-	0,001114523	-0,623254098	0,624368621
205050_s_at	MAPK8IP2	0,001114523	-0,623254098	0,624368621
1554606_at	CEP120	0,001114523	-0,623254098	0,624368621
233049_x_at	STUB1	3,505636113	2,881480309	0,624155805
225074_at	RAB2B	3,751261994	3,127281098	0,623980896
225113_at	AGPS	1,995527416	1,371634653	0,623892763
209411_s_at	GGA3	2,552836808	1,929092307	0,623744501
214179_s_at	NFE2L1	3,327128828	2,703479652	0,623649175
224180_x_at	WDPCP	1,139057614	0,515457602	0,623600012
217019_at	-	1,139057614	0,515457602	0,623600012
235675_at	DHFRL1	2,107136847	1,483698568	0,623438279
229140_at	ZNF579	1,5944803	0,971607464	0,622872836
1563557_at	-	-0,994186487	-1,616924751	0,622738264
203645_s_at	CD163	-0,994186487	-1,616924751	0,622738264
1554380_at	NEK11	-0,994186487	-1,616924751	0,622738264
210704_at	FEZ2	-0,994186487	-1,616924751	0,622738264
239769_at	CDH11	-0,994186487	-1,616924751	0,622738264
1564369_at	-	-0,994186487	-1,616924751	0,622738264
232246_at	TTC28-AS1	-0,994186487	-1,616924751	0,622738264
1552884_at	NKX6-3	-0,994186487	-1,616924751	0,622738264
205438_at	PTPN21	-0,994186487	-1,616924751	0,622738264
206659_at	-	-0,994186487	-1,616924751	0,622738264
203591_s_at	CSF3R	-0,994186487	-1,616924751	0,622738264
233806_at	-	-0,994186487	-1,616924751	0,622738264
1553281_at	PLCXD2	-0,994186487	-1,616924751	0,622738264
242973_at	CACNA1C	-0,994186487	-1,616924751	0,622738264
238645_at	-	-0,994186487	-1,616924751	0,622738264
239810_at	VASH1	-0,994186487	-1,616924751	0,622738264
204792_s_at	IFT140	-0,994186487	-1,616924751	0,622738264
229284_at	MAT2B	-0,994186487	-1,616924751	0,622738264
211300_s_at	TP53	-0,994186487	-1,616924751	0,622738264
212354_at	SULF1	-0,994186487	-1,616924751	0,622738264
211429_s_at	SERPINA1	-0,994186487	-1,616924751	0,622738264
218474_s_at	KCTD5	3,09217775	2,469496389	0,622681361
1560060_s_at	VPS37C	3,584942056	2,962798728	0,622143328
215616_s_at	KDM4B	0,707657549	0,085534992	0,622122556
1555824_a_at	PACS2	0,39105295	-0,231045907	0,622098857
232177_at	-	0,39105295	-0,231045907	0,622098857
239281_at	ZDHHC14	0,39105295	-0,231045907	0,622098857
218485_s_at	SLC35C1	0,39105295	-0,231045907	0,622098857
211994_at	WNK1	4,478885385	3,856800376	0,62208501
225514_at	NOP9	1,364555912	0,742671819	0,621884093
201493_s_at	PUM2	4,747567561	4,12578963	0,621777931
225622_at	PAG1	2,33082239	1,709047448	0,621774942
212901_s_at	CSTF2T	2,698955954	2,077419168	0,621536786
1561886_a_at	-	-2,074223907	-2,695640852	0,621416945
233886_at	-	-2,074223907	-2,695640852	0,621416945
232113_at	-	-2,074223907	-2,695640852	0,621416945

202935_s_at	SOX9	-2,074223907	-2,695640852	0,621416945
239572_at	GJA3	-2,074223907	-2,695640852	0,621416945
239887_at	-	-1,289805289	-1,911206569	0,621401281
1557126_a_at	PLD1	-1,289805289	-1,911206569	0,621401281
217262_s_at	CELSR1	-1,289805289	-1,911206569	0,621401281
223860_at	-	-1,289805289	-1,911206569	0,621401281
234615_at	-	-1,289805289	-1,911206569	0,621401281
241121_at	LOC100505928	-1,289805289	-1,911206569	0,621401281
1568931_at	-	-1,289805289	-1,911206569	0,621401281
234222_at	-	-1,289805289	-1,911206569	0,621401281
217651_at	-	-1,289805289	-1,911206569	0,621401281
208084_at	ITGB6	-1,289805289	-1,911206569	0,621401281
244541_x_at	-	-1,289805289	-1,911206569	0,621401281
207789_s_at	DPP6	-1,289805289	-1,911206569	0,621401281
233469_at	TPTEP1	-1,289805289	-1,911206569	0,621401281
223754_at	C2orf88	-1,289805289	-1,911206569	0,621401281
1557805_at	-	-1,289805289	-1,911206569	0,621401281
1554787_at	C9orf96	-1,289805289	-1,911206569	0,621401281
241358_at	WFIKKN2	-1,289805289	-1,911206569	0,621401281
229772_at	DEFB123	-1,289805289	-1,911206569	0,621401281
206252_s_at	AVPR1A	-1,289805289	-1,911206569	0,621401281
231363_at	LELP1	-1,289805289	-1,911206569	0,621401281
214595_at	KCNG1	-1,289805289	-1,911206569	0,621401281
234500_at	-	-1,289805289	-1,911206569	0,621401281
217002_s_at	HTR3A	-1,289805289	-1,911206569	0,621401281
241424_at	ELAVL4	-1,289805289	-1,911206569	0,621401281
1553536_at	MBNL2	-1,289805289	-1,911206569	0,621401281
1569044_at	CDC42BPG	-1,289805289	-1,911206569	0,621401281
1561181_at	-	-1,289805289	-1,911206569	0,621401281
1552459_a_at	MBD3L1	-1,289805289	-1,911206569	0,621401281
244037_at	LOC100507520	-1,289805289	-1,911206569	0,621401281
206215_at	OPCML	-1,289805289	-1,911206569	0,621401281
209205_s_at	LMO4	4,441588953	3,820305316	0,621283637
244793_at	-	-1,982324124	-2,603471267	0,621147143
224146_s_at	ABCC11	-1,982324124	-2,603471267	0,621147143
235538_at	-	-1,982324124	-2,603471267	0,621147143
214636_at	CALCB	-1,982324124	-2,603471267	0,621147143
234029_at	PCDHGB8P	-1,982324124	-2,603471267	0,621147143
1557705_a_at	-	-1,982324124	-2,603471267	0,621147143
1559409_a_at	CC2D2A	-1,982324124	-2,603471267	0,621147143
230372_at	HAS2	-1,982324124	-2,603471267	0,621147143
211579_at	ITGB3	-1,982324124	-2,603471267	0,621147143
209618_at	CTNND2	-1,982324124	-2,603471267	0,621147143
226323_at	ZNF830	2,725848647	2,104873932	0,620974715
37943_at	ZFYVE26	1,449043754	0,828711208	0,620332546
212262_at	QKI	2,356619952	1,736450458	0,620169494
37424_at	CCHCR1	2,077410781	1,457445467	0,619965314
239272_at	MMP28	-0,437449947	-1,057301851	0,619851903
207527_at	KCNJ9	-0,437449947	-1,057301851	0,619851903
220413_at	SLC39A2	-0,437449947	-1,057301851	0,619851903

1566653_at	-	-0,437449947	-1,057301851	0,619851903
223953_s_at	ZBTB37	-0,437449947	-1,057301851	0,619851903
234055_s_at	GZF1	-0,437449947	-1,057301851	0,619851903
225776_at	RBMS2	-0,437449947	-1,057301851	0,619851903
1555659_a_at	TREML1	-0,437449947	-1,057301851	0,619851903
220730_at	-	-0,437449947	-1,057301851	0,619851903
226904_at	SLC6A10P /// SLC6	-0,437449947	-1,057301851	0,619851903
207961_x_at	MYH11	-0,437449947	-1,057301851	0,619851903
227802_at	RUFY3	-0,437449947	-1,057301851	0,619851903
1559532_at	C3orf71	0,556428218	-0,063377083	0,619805301
236706_at	LYG1	0,556428218	-0,063377083	0,619805301
1557170_at	NEK8	0,820160788	0,20062106	0,619539728
58916_at	KCTD14 /// NDUFC	-1,608003067	-2,227441258	0,619438191
204599_s_at	MRPL28	3,018305638	2,398910172	0,619395465
207439_s_at	SLC35A2	0,167727503	-0,45121326	0,618940763
232351_at	-	0,167727503	-0,45121326	0,618940763
240934_at	-	0,167727503	-0,45121326	0,618940763
243568_at	-	0,167727503	-0,45121326	0,618940763
244462_at	ZNF224	-0,230432956	-0,84928999	0,618857034
202398_at	AP3S2 /// C15orf3	-0,230432956	-0,84928999	0,618857034
1555270_a_at	WFS1	-0,230432956	-0,84928999	0,618857034
228403_at	ENHO	-0,230432956	-0,84928999	0,618857034
242314_at	TNRC6C	-0,230432956	-0,84928999	0,618857034
1554953_a_at	C21orf90	-0,230432956	-0,84928999	0,618857034
230805_at	-	2,436165655	1,817403386	0,618762269
201709_s_at	NIPSNAP1	4,391815177	3,7731786	0,618636578
219293_s_at	OLA1	6,415301	5,796746461	0,618554539
221493_at	TSPYL1	4,446069945	3,827536095	0,61853385
217580_x_at	-	1,024284941	0,405752839	0,618532102
204497_at	ADCY9	1,345611839	0,727123109	0,61848873
225893_at	RC3H1	2,932161212	2,314488991	0,617672222
227060_at	RELT	1,528073958	0,910483921	0,617590038
225552_x_at	AURKAIP1	5,334249893	4,716711672	0,61753822
229803_s_at	NUDT3 /// RPS10-I	2,955426542	2,338020736	0,617405806
212375_at	EP400	2,401508182	1,784182261	0,617325921
239372_at	-	-0,043925712	-0,661178575	0,617252863
232104_at	C1orf95	-0,043925712	-0,661178575	0,617252863
47553_at	DFNB31	-1,667858949	-2,284938246	0,617079296
1553214_a_at	CCDC7	-2,163157732	-2,780225148	0,617067416
208586_s_at	SSX4 /// SSX4B	-2,163157732	-2,780225148	0,617067416
1555572_at	CA6	-2,163157732	-2,780225148	0,617067416
1565685_at	LOC400940	-2,163157732	-2,780225148	0,617067416
1555313_a_at	MCF2	-2,163157732	-2,780225148	0,617067416
226554_at	ZBTB7A	3,410696467	2,793634788	0,61706168
212600_s_at	UQCRC2	6,725585606	6,108789916	0,61679569
222690_s_at	TMEM39A	2,427650693	1,811004388	0,616646305
221058_s_at	CKLF	4,288236521	3,671698545	0,616537976
207747_s_at	DOK4	0,355751005	-0,260526297	0,616277302
230196_x_at	ARHGAP23	0,355751005	-0,260526297	0,616277302
206097_at	SLC22A18AS	0,355751005	-0,260526297	0,616277302

203847_s_at	AKAP8	1,92317941	1,306903969	0,616275441
226178_at	SOCS4	3,828857041	3,212688281	0,616168759
205998_x_at	CYP3A4	1,402495885	0,786391549	0,616104336
222238_s_at	POLM	0,677745787	0,061773582	0,615972205
229375_at	PPIE	0,677745787	0,061773582	0,615972205
226707_at	NAPRT1	1,458255986	0,842599219	0,615656767
239270_at	PLCXD3	-0,856259172	-1,471833228	0,615574056
243014_at	-	-0,856259172	-1,471833228	0,615574056
205151_s_at	TRIL	-0,856259172	-1,471833228	0,615574056
232337_at	B3GNT7	-0,856259172	-1,471833228	0,615574056
210248_at	WNT7A	-0,856259172	-1,471833228	0,615574056
228629_s_at	WWTR1-AS1	-0,856259172	-1,471833228	0,615574056
239953_at	-	-0,856259172	-1,471833228	0,615574056
1554655_a_at	RPRML	-0,856259172	-1,471833228	0,615574056
210967_x_at	CACNB1	-0,856259172	-1,471833228	0,615574056
1554110_at	CDCP1	-0,856259172	-1,471833228	0,615574056
210659_at	CMKLR1	-0,856259172	-1,471833228	0,615574056
240135_x_at	-	-0,856259172	-1,471833228	0,615574056
1556675_s_at	LOC100507584	-0,856259172	-1,471833228	0,615574056
206760_s_at	FCER2	-0,856259172	-1,471833228	0,615574056
205622_at	SMPD2	1,000459215	0,385582632	0,614876583
211439_at	SRSF7	1,000459215	0,385582632	0,614876583
220147_s_at	FAM60A	4,976515905	4,361943785	0,614572119
227416_s_at	ZCRB1	5,853835436	5,239283618	0,614551818
212032_s_at	PTOV1	2,507817181	1,893444329	0,614372852
220081_x_at	HSD17B7	3,948420174	3,334057854	0,61436232
222686_s_at	CPPED1	1,843966654	1,2298509	0,614115754
242073_at	-	0,900065578	0,286279868	0,61378571
224656_s_at	LUZP6 /// MTPN	6,335333297	5,721585079	0,613748218
216711_s_at	TAF1	0,791908897	0,178191865	0,613717032
226093_at	DCP1B	2,560606634	1,946903052	0,613703582
218189_s_at	NANS	4,739280418	4,12578963	0,613490788
216996_s_at	FASTKD2	2,71254166	2,099079406	0,613462254
218643_s_at	CRIPT	3,394274216	2,780838243	0,613435973
207878_at	KRT76	-0,66934516	-1,282520722	0,613175563
213487_at	MAP2K2	-0,66934516	-1,282520722	0,613175563
1564154_at	-	-0,66934516	-1,282520722	0,613175563
222159_at	-	-0,66934516	-1,282520722	0,613175563
1552902_a_at	FOXP2	-0,66934516	-1,282520722	0,613175563
241490_s_at	PGBD2	-0,66934516	-1,282520722	0,613175563
1557866_at	C9orf117	-0,66934516	-1,282520722	0,613175563
1562770_at	-	-0,66934516	-1,282520722	0,613175563
204002_s_at	ICA1	-0,66934516	-1,282520722	0,613175563
225500_x_at	SCAF1	-0,66934516	-1,282520722	0,613175563
239249_at	-	-0,66934516	-1,282520722	0,613175563
233920_at	-	-0,66934516	-1,282520722	0,613175563
1564603_at	C15orf55	-0,66934516	-1,282520722	0,613175563
1567375_at	-	-0,66934516	-1,282520722	0,613175563
212731_at	ANKRD46	2,530390387	1,917743773	0,612646614
219155_at	PITPNC1	3,789279973	3,176868665	0,612411309

203195_s_at	NUP98	2,38349585	1,771157334	0,612338516
1557135_at	-	0,127732831	-0,484558493	0,612291324
238491_at	LOC100506161	1,246401134	0,634301128	0,612100006
228395_at	GLT8D1	1,246401134	0,634301128	0,612100006
201992_s_at	KIF5B	2,127965541	1,516067292	0,611898249
1563001_at	-	-2,336678463	-2,948507524	0,611829061
235215_at	ERCC4	1,160996507	0,550017041	0,610979467
221537_at	PLXNA1	1,440265662	0,829309537	0,610956126
209787_s_at	HMGN4	6,135655793	5,52471291	0,610942884
223306_at	EBPL	4,348261798	3,737342734	0,610919064
214118_x_at	PCM1	3,8018113	3,191011385	0,610799915
222764_at	ASRGL1	1,5944803	0,98381571	0,61066459
231629_x_at	KLK3	1,383446753	0,772793615	0,610653138
234726_s_at	TMEM168	1,383446753	0,772793615	0,610653138
231975_s_at	MIER3	0,97663406	0,366031214	0,610602846
230434_at	PHOSPHO2	0,97663406	0,366031214	0,610602846
201758_at	TSG101	4,823280506	4,21289113	0,610389376
1556479_at	-	0,648195588	0,03784737	0,610348218
202048_s_at	CBX6	3,394274216	2,783936118	0,610338098
223141_at	UCK1	2,339581693	1,72935563	0,610226063
231818_x_at	-	0,319287178	-0,290694975	0,609982153
203792_x_at	PCGF2	0,319287178	-0,290694975	0,609982153
1558914_at	DESI2	1,071336699	0,461385738	0,609950961
226443_at	FAM122A	1,97249974	1,362900801	0,609598939
40560_at	TBX2	0,406970081	-0,202494341	0,609464422
201178_at	FBXO7	4,705123614	4,095792397	0,609331217
48030_i_at	C5orf4	2,771643417	2,162541631	0,609101786
218767_at	REXO4	2,896717728	2,287674614	0,609043114
50374_at	C17orf90	3,16393829	2,554957372	0,608980918
1555529_at	RNH1	-0,280273599	-0,889222211	0,608948612
232059_at	DSCAML1	-0,280273599	-0,889222211	0,608948612
216107_at	PP13	-0,280273599	-0,889222211	0,608948612
236147_at	-	-0,280273599	-0,889222211	0,608948612
208489_at	GJA8	-0,280273599	-0,889222211	0,608948612
239267_at	NEK6	-0,280273599	-0,889222211	0,608948612
216600_x_at	ALDOB	-0,280273599	-0,889222211	0,608948612
228177_at	CREBBP	1,544940605	0,936232181	0,608708424
1553703_at	ZNF791	2,476788561	1,868130962	0,608657599
223282_at	TSHZ1	3,107338728	2,498884538	0,608454191
219831_at	CDKL3	0,873789652	0,265475485	0,608314167
210074_at	CTSL2	0,873789652	0,265475485	0,608314167
213551_x_at	PCGF2	0,873789652	0,265475485	0,608314167
233963_at	H2BFXP	-0,088974936	-0,697182596	0,608207661
1569640_s_at	EEPD1	-0,088974936	-0,697182596	0,608207661
1555553_a_at	SLC22A7	-0,088974936	-0,697182596	0,608207661
216083_s_at	NEU3	-0,088974936	-0,697182596	0,608207661
238946_at	-	-0,49284695	-1,100943374	0,608096424
236086_at	-	-0,49284695	-1,100943374	0,608096424
230112_at	04.03.15	-0,49284695	-1,100943374	0,608096424
1552340_at	SP7	-0,49284695	-1,100943374	0,608096424

234246_at	-	-0,49284695	-1,100943374	0,608096424
217614_at	-	-0,49284695	-1,100943374	0,608096424
244368_x_at	-	-0,49284695	-1,100943374	0,608096424
1555378_at	DBF4B	-0,49284695	-1,100943374	0,608096424
214676_x_at	MUC3B	-0,49284695	-1,100943374	0,608096424
239676_x_at	FBRSL1 /// LOC10(	-0,49284695	-1,100943374	0,608096424
214797_s_at	CDK18	-0,49284695	-1,100943374	0,608096424
232636_at	SLITRK4	-1,893506789	-2,501359113	0,607852324
1563475_s_at	METTL20	-1,893506789	-2,501359113	0,607852324
234903_at	OR2B3	-1,893506789	-2,501359113	0,607852324
243081_at	LOC100652770	-1,893506789	-2,501359113	0,607852324
234390_x_at	-	-1,893506789	-2,501359113	0,607852324
1560728_at	-	-1,893506789	-2,501359113	0,607852324
1563897_at	CRABP1	-1,893506789	-2,501359113	0,607852324
223977_s_at	LINC00470	-1,893506789	-2,501359113	0,607852324
216638_s_at	PRLR	-1,893506789	-2,501359113	0,607852324
1561418_at	-	-1,893506789	-2,501359113	0,607852324
244244_at	-	-1,893506789	-2,501359113	0,607852324
222725_s_at	PALMD	-1,893506789	-2,501359113	0,607852324
232553_at	PCYT1B	-1,893506789	-2,501359113	0,607852324
203001_s_at	STMN2	-1,893506789	-2,501359113	0,607852324
234348_at	-	-1,893506789	-2,501359113	0,607852324
240439_at	-	-1,893506789	-2,501359113	0,607852324
1557495_at	-	-1,893506789	-2,501359113	0,607852324
226294_x_at	FAM91A1	3,6787904	3,071469991	0,607320409
204853_at	ORC2	2,582572508	1,975797592	0,606774916
227313_at	CNPY4	2,38349585	1,777135134	0,606360717
238585_at	GTDC1	1,476175815	0,869824768	0,606351047
237202_at	PGPEP1	1,421391637	0,815200271	0,606191366
228566_at	RPRD1A	1,421391637	0,815200271	0,606191366
208065_at	ST8SIA3	-2,250580859	-2,856570137	0,605989278
208529_at	BTF3P11	-2,250580859	-2,856570137	0,605989278
212216_at	PREPL	2,649978302	2,044580955	0,605397348
238996_x_at	ALDOA	4,170262037	3,564966474	0,605295563
228469_at	PPID	0,618277321	0,013190398	0,605086923
218878_s_at	SIRT1	2,285848435	1,680963921	0,604884515
206444_at	PDE1B	0,282358733	-0,322340048	0,604698781
241010_x_at	-	0,282358733	-0,322340048	0,604698781
222736_s_at	TMEM38B	1,843966654	1,239268196	0,604698458
210627_s_at	MOGS	2,159837409	1,555165465	0,604671944
234840_s_at	OR5V1	-1,537851782	-2,142286822	0,604435039
216687_x_at	UGT2B15	-1,537851782	-2,142286822	0,604435039
240953_at	-	-1,537851782	-2,142286822	0,604435039
1564662_at	ZNF852	-1,537851782	-2,142286822	0,604435039
240949_x_at	-	-1,537851782	-2,142286822	0,604435039
205287_s_at	TFAP2C	-1,537851782	-2,142286822	0,604435039
232368_at	BET3L	-1,537851782	-2,142286822	0,604435039
222456_s_at	LIMA1	-1,537851782	-2,142286822	0,604435039
229441_at	PRSS23	-1,537851782	-2,142286822	0,604435039
216764_at	-	-1,537851782	-2,142286822	0,604435039

229125_at	KANK4	-1,537851782	-2,142286822	0,604435039
236415_at	DENND5B	-1,537851782	-2,142286822	0,604435039
228782_at	SCGB3A2	-1,537851782	-2,142286822	0,604435039
217561_at	CALCA	-1,537851782	-2,142286822	0,604435039
1561345_at	-	-1,537851782	-2,142286822	0,604435039
238138_at	CANX	-1,537851782	-2,142286822	0,604435039
1562953_s_at	WDFY3-AS2	-1,537851782	-2,142286822	0,604435039
222134_at	DDO	-1,537851782	-2,142286822	0,604435039
236877_at	-	-1,537851782	-2,142286822	0,604435039
222908_at	PIEZO2	-1,537851782	-2,142286822	0,604435039
233715_at	-	-1,537851782	-2,142286822	0,604435039
206597_at	NRL	-1,537851782	-2,142286822	0,604435039
203000_at	STMN2	-1,537851782	-2,142286822	0,604435039
239874_at	PDE8A	-1,537851782	-2,142286822	0,604435039
216184_s_at	RIMS1	-1,537851782	-2,142286822	0,604435039
1567272_at	OR2K2	-1,537851782	-2,142286822	0,604435039
220263_at	SMAD5-AS1	-1,537851782	-2,142286822	0,604435039
244489_at	LOC100129268 ///	-1,537851782	-2,142286822	0,604435039
236377_at	TMEM132D	-1,537851782	-2,142286822	0,604435039
206865_at	HRK	-1,537851782	-2,142286822	0,604435039
233737_s_at	LOC284561	-1,537851782	-2,142286822	0,604435039
221070_s_at	KIAA1967	-1,537851782	-2,142286822	0,604435039
221280_s_at	PARD3	-1,537851782	-2,142286822	0,604435039
214822_at	FAM5B	-1,537851782	-2,142286822	0,604435039
203457_at	STX7	0,08619576	-0,518232988	0,604428748
222793_at	DDX58	0,08619576	-0,518232988	0,604428748
232499_at	ITPKB	0,08619576	-0,518232988	0,604428748
205873_at	PIGL	0,08619576	-0,518232988	0,604428748
217147_s_at	TRAT1	2,597647262	1,993309863	0,604337399
222700_at	ATL2	4,608929726	4,00463588	0,604293846
231433_at	LOC100506929	-1,624955693	-2,229233437	0,604277744
1552929_at	GRK7	-1,624955693	-2,229233437	0,604277744
240018_at	-	-1,624955693	-2,229233437	0,604277744
214647_s_at	HFE	-1,624955693	-2,229233437	0,604277744
1554689_a_at	NLGN4X	-1,624955693	-2,229233437	0,604277744
1554892_a_at	MS4A3	-1,624955693	-2,229233437	0,604277744
231606_at	-	-1,624955693	-2,229233437	0,604277744
241549_at	-	-1,624955693	-2,229233437	0,604277744
208006_at	FOXI1	-1,624955693	-2,229233437	0,604277744
239452_at	-	-1,624955693	-2,229233437	0,604277744
243379_at	-	-1,624955693	-2,229233437	0,604277744
1560109_s_at	-	-1,624955693	-2,229233437	0,604277744
244388_at	-	-1,624955693	-2,229233437	0,604277744
202888_s_at	ANPEP	-1,624955693	-2,229233437	0,604277744
231624_s_at	-	-1,624955693	-2,229233437	0,604277744
222361_at	TUBBP5	-1,624955693	-2,229233437	0,604277744
1556161_a_at	LOC100506102	-1,624955693	-2,229233437	0,604277744
243048_at	CECR7	-1,624955693	-2,229233437	0,604277744
217411_s_at	RREB1	-1,624955693	-2,229233437	0,604277744
220419_s_at	USP25	3,918896157	3,315052527	0,603843631

226389_s_at	RAPGEF1	2,484824532	1,881145546	0,603678985
225993_at	EARS2	0,458905032	-0,144765583	0,603670615
217042_at	RDH11	-1,06282519	-1,665791776	0,602966586
228085_at	LOC100507419 ///	-1,06282519	-1,665791776	0,602966586
242659_at	-	-1,06282519	-1,665791776	0,602966586
1553673_at	STK35	-1,06282519	-1,665791776	0,602966586
210271_at	NEUROD2	-1,06282519	-1,665791776	0,602966586
215934_at	-	-1,06282519	-1,665791776	0,602966586
210247_at	SYN2	-1,06282519	-1,665791776	0,602966586
238241_at	LOC100144602	-1,06282519	-1,665791776	0,602966586
228802_at	RBPMS2	-1,06282519	-1,665791776	0,602966586
209266_s_at	SLC39A8	-1,06282519	-1,665791776	0,602966586
232821_at	GTSF1L	-1,06282519	-1,665791776	0,602966586
217029_at	-	-1,06282519	-1,665791776	0,602966586
206209_s_at	CA4	-1,06282519	-1,665791776	0,602966586
236238_at	-	-1,06282519	-1,665791776	0,602966586
210545_at	ITSN2	-1,06282519	-1,665791776	0,602966586
221361_at	OMP	-1,06282519	-1,665791776	0,602966586
237913_at	LOC100506348	-1,06282519	-1,665791776	0,602966586
217685_at	SLC16A3	-1,06282519	-1,665791776	0,602966586
224220_x_at	TRPC4	-1,06282519	-1,665791776	0,602966586
220002_at	KIF26B	-1,06282519	-1,665791776	0,602966586
1561785_at	MUC4	-1,06282519	-1,665791776	0,602966586
220231_at	PPP1R17	-1,06282519	-1,665791776	0,602966586
232797_at	-	-1,06282519	-1,665791776	0,602966586
208924_at	RNF11	3,966733349	3,363771078	0,602962271
230100_x_at	PAK1	1,871521385	1,268569037	0,602952347
1569105_at	SETD5	1,871521385	1,268569037	0,602952347
232095_at	LOC100509683	1,345611839	0,742671819	0,60294002
223083_s_at	EGLN2 /// RAB4B-I	0,735999505	0,133104519	0,602894986
217419_x_at	AGRN	0,847529938	0,244646697	0,60288324
242725_at	-	0,847529938	0,244646697	0,60288324
200595_s_at	EIF3A	4,628242806	4,025360264	0,602882542
240383_at	UBE2D3	1,936380191	1,333668715	0,602711477
200017_at	RPS27A	8,281473246	7,679276356	0,60219689
201370_s_at	CUL3	2,085817135	1,483698568	0,602118567
209372_x_at	TUBB2A /// TUBB2	2,053550345	1,451507543	0,602042802
1555841_at	MSANTD3	3,071179046	2,469496389	0,601682657
201897_s_at	CKS1B	6,387600678	5,786351479	0,601249199
224668_at	SYS1	1,97249974	1,371634653	0,600865087
212710_at	CAMSAP1	2,468978138	1,868130962	0,600847177
217827_s_at	SPG21	4,2463808	3,645557465	0,600823334
225388_at	TSPAN5	2,798156197	2,197404429	0,600751768
233929_x_at	WASH3P	2,752615529	2,151985997	0,600629532
210582_s_at	LIMK2	1,510970097	0,910483921	0,600486177
225652_at	-	3,029186714	2,428719338	0,600467376
237315_at	-	0,045517965	-0,554696666	0,600214631
236997_at	LOC100505554	0,045517965	-0,554696666	0,600214631
236629_at	IBA57	0,045517965	-0,554696666	0,600214631
234305_s_at	GSDMC	0,045517965	-0,554696666	0,600214631

200093_s_at	HINT1	7,420132533	6,820407231	0,599725302
221248_s_at	WHSC1L1	1,024284941	0,424693306	0,599591635
203878_s_at	MMP11	0,244681185	-0,354781582	0,599462767
239991_at	-	0,244681185	-0,354781582	0,599462767
219515_at	PRDM10	2,042564225	1,44320939	0,599354835
241676_x_at	-	-1,712732543	-2,312054429	0,599321886
243542_at	PREPL	-1,712732543	-2,312054429	0,599321886
219732_at	LPPR1	-1,712732543	-2,312054429	0,599321886
1561658_at	-	-1,712732543	-2,312054429	0,599321886
215770_at	OR7E2P	-1,712732543	-2,312054429	0,599321886
1554739_at	IPP	-1,712732543	-2,312054429	0,599321886
1556898_at	LOC200830	-1,712732543	-2,312054429	0,599321886
234083_at	-	-1,712732543	-2,312054429	0,599321886
227763_at	LYPD6	-1,712732543	-2,312054429	0,599321886
239636_at	MCF2L	-1,712732543	-2,312054429	0,599321886
233611_at	-	-1,712732543	-2,312054429	0,599321886
242212_at	RGS16	-1,712732543	-2,312054429	0,599321886
241635_at	-	-1,712732543	-2,312054429	0,599321886
1570296_at	-	-1,712732543	-2,312054429	0,599321886
206373_at	ZIC1	-1,712732543	-2,312054429	0,599321886
1565927_s_at	-	-1,712732543	-2,312054429	0,599321886
239344_at	-	-1,712732543	-2,312054429	0,599321886
1553838_at	C4orf45	-1,712732543	-2,312054429	0,599321886
1563587_at	CDH4	-1,712732543	-2,312054429	0,599321886
205309_at	SMPDL3B	-1,712732543	-2,312054429	0,599321886
238133_at	-	-1,712732543	-2,312054429	0,599321886
228233_at	FREM1	-1,712732543	-2,312054429	0,599321886
208367_x_at	CYP3A4	-1,712732543	-2,312054429	0,599321886
236747_at	LOC100507066	-1,712732543	-2,312054429	0,599321886
202052_s_at	RAI14	-1,712732543	-2,312054429	0,599321886
204802_at	RRAD	-1,712732543	-2,312054429	0,599321886
218914_at	RRNAD1	1,182925501	0,583607503	0,599317998
236980_at	STRN	-0,135065865	-0,734355396	0,599289531
230737_s_at	LOC387647	-0,135065865	-0,734355396	0,599289531
215530_at	FANCA	-0,135065865	-0,734355396	0,599289531
206610_s_at	F11	-0,135065865	-0,734355396	0,599289531
216047_x_at	SEZ6L	-0,135065865	-0,734355396	0,599289531
235349_at	FAM82A1	-0,332405896	-0,931539147	0,599133251
243731_at	-	-0,332405896	-0,931539147	0,599133251
239400_at	FLJ45513	-0,332405896	-0,931539147	0,599133251
1559996_s_at	SAMD14	-0,332405896	-0,931539147	0,599133251
1553778_at	WBSCR27	-0,332405896	-0,931539147	0,599133251
1553997_a_at	ASPHD1	-0,332405896	-0,931539147	0,599133251
220095_at	CNTLN	-1,212652659	-1,811780055	0,599127396
233644_at	KATNAL2	-1,212652659	-1,811780055	0,599127396
243583_at	-	-1,212652659	-1,811780055	0,599127396
243540_at	-	-1,212652659	-1,811780055	0,599127396
236252_at	-	-1,212652659	-1,811780055	0,599127396
238660_at	WDFY3	-1,212652659	-1,811780055	0,599127396
204309_at	CYP11A1	-1,212652659	-1,811780055	0,599127396

236096_at	SIPA1L3	-1,212652659	-1,811780055	0,599127396
228047_at	SNORA72	-1,212652659	-1,811780055	0,599127396
1569681_at	-	-1,212652659	-1,811780055	0,599127396
1561635_at	-	-1,212652659	-1,811780055	0,599127396
221405_at	-	-1,212652659	-1,811780055	0,599127396
207589_at	ADRA1B	-1,212652659	-1,811780055	0,599127396
206259_at	PROC	-1,212652659	-1,811780055	0,599127396
1566229_a_at	-	-1,212652659	-1,811780055	0,599127396
206573_at	KCNQ3	-1,212652659	-1,811780055	0,599127396
1562153_a_at	MIR1204 /// PVT1	-1,212652659	-1,811780055	0,599127396
207377_at	PPP1R2P9	-1,212652659	-1,811780055	0,599127396
203691_at	PI3	-1,212652659	-1,811780055	0,599127396
228004_at	LINC00261	-1,212652659	-1,811780055	0,599127396
237336_at	ADD2	-1,212652659	-1,811780055	0,599127396
208657_s_at	40057	2,849132494	2,250228409	0,598904085
1556809_a_at	-	0,58788177	-0,011001236	0,598883006
236719_at	-	0,58788177	-0,011001236	0,598883006
212469_at	NIPBL	2,908690075	2,309925448	0,598764627
209949_at	NCF2	3,331722254	2,733194118	0,598528136
212742_at	RNF115	3,498020785	2,899658146	0,598362638
226935_s_at	CLPTM1L	3,498020785	2,899658146	0,598362638
211797_s_at	NFYC	2,664625637	2,066329863	0,598295774
230626_at	TSPAN12	-0,730013898	-1,32740143	0,597387532
242860_at	-	-0,730013898	-1,32740143	0,597387532
231347_at	-	-0,730013898	-1,32740143	0,597387532
1554839_at	CIDECP	-0,730013898	-1,32740143	0,597387532
215669_at	HLA-DRB4	-0,730013898	-1,32740143	0,597387532
216800_at	-	-0,730013898	-1,32740143	0,597387532
1567519_at	PLXNA3	-0,730013898	-1,32740143	0,597387532
1565728_at	LOC284630	-0,730013898	-1,32740143	0,597387532
238648_at	-	-0,730013898	-1,32740143	0,597387532
243117_at	-	-0,730013898	-1,32740143	0,597387532
1558984_at	MAP3K11	-0,730013898	-1,32740143	0,597387532
206800_at	MTHFR	-0,730013898	-1,32740143	0,597387532
237254_at	SLC5A11	-0,730013898	-1,32740143	0,597387532
230996_at	LPP	-0,730013898	-1,32740143	0,597387532
229002_at	FAM69B	-0,730013898	-1,32740143	0,597387532
211869_at	-	-0,730013898	-1,32740143	0,597387532
208111_at	AVPR2	-0,730013898	-1,32740143	0,597387532
1569416_at	-	-0,730013898	-1,32740143	0,597387532
237916_at	-	-0,730013898	-1,32740143	0,597387532
220554_at	SLC22A7	-0,730013898	-1,32740143	0,597387532
211811_s_at	PCDHA6	-1,452713826	-2,049747196	0,59703337
1562720_at	-	-1,452713826	-2,049747196	0,59703337
240638_at	-	-1,452713826	-2,049747196	0,59703337
234171_at	-	-1,452713826	-2,049747196	0,59703337
223999_at	PPIL2	-1,452713826	-2,049747196	0,59703337
1566709_at	-	-1,452713826	-2,049747196	0,59703337
217222_at	IGHG1	-1,452713826	-2,049747196	0,59703337
244113_at	KCNJ9	-1,452713826	-2,049747196	0,59703337

240468_at	-	-1,452713826	-2,049747196	0,59703337
1556420_s_at	YPEL2	-1,452713826	-2,049747196	0,59703337
228396_at	PRKG1	-1,452713826	-2,049747196	0,59703337
1563034_at	GPD1	-1,452713826	-2,049747196	0,59703337
237734_s_at	LOC100506907	-1,452713826	-2,049747196	0,59703337
229225_at	NRP2	-1,452713826	-2,049747196	0,59703337
230137_at	TMEM155	-1,452713826	-2,049747196	0,59703337
220614_s_at	ADGB	-1,452713826	-2,049747196	0,59703337
213160_at	DOCK2	5,31516215	4,718173606	0,596988544
203107_x_at	RPS2 /// SNORA64	8,955051068	8,358145701	0,596905366
208238_x_at	-	2,312738346	1,715863686	0,59687466
204349_at	MED7	2,739067176	2,142313983	0,596753193
220097_s_at	TMEM104	1,094118704	0,497424714	0,59669399
221788_at	PGM3	1,094118704	0,497424714	0,59669399
1560878_at	SYT15	1,094118704	0,497424714	0,59669399
213394_at	MAPKBP1	1,816938997	1,220470658	0,596468339
209553_at	LOC100505729 ///	2,21987062	1,623485524	0,596385097
228600_x_at	FAM221A	1,88491011	1,28853674	0,59637337
226251_at	ASXL2	1,88491011	1,28853674	0,59637337
217799_x_at	UBE2H	2,507817181	1,911502437	0,596314745
223319_at	GPHN	2,096248267	1,500059916	0,596188351
201966_at	NDUFS2	5,188683224	4,59257978	0,596103444
240530_at	-	-0,924000698	-1,520022123	0,596021426
205866_at	FCN3	-0,924000698	-1,520022123	0,596021426
221424_s_at	OR51E2	-0,924000698	-1,520022123	0,596021426
202525_at	PRSS8	-0,924000698	-1,520022123	0,596021426
234693_at	-	-0,924000698	-1,520022123	0,596021426
207557_s_at	RYR2	-0,924000698	-1,520022123	0,596021426
237654_at	PPP1R36	-0,924000698	-1,520022123	0,596021426
216886_at	CHRNA4	-0,924000698	-1,520022123	0,596021426
236810_at	-	-0,924000698	-1,520022123	0,596021426
206227_at	CILP	-0,924000698	-1,520022123	0,596021426
234484_s_at	ACSS1	-0,924000698	-1,520022123	0,596021426
1565737_at	SCTR	-0,924000698	-1,520022123	0,596021426
228537_at	GLI2	-0,924000698	-1,520022123	0,596021426
237097_at	-	-0,924000698	-1,520022123	0,596021426
230423_at	-	-0,924000698	-1,520022123	0,596021426
46256_at	SPSB3	3,50971064	2,913762824	0,595947816
213803_at	-	4,642573329	4,047300286	0,595273043
228680_at	KIF3A	3,161901883	2,56688207	0,595019813
206263_at	FMO4	1,000459215	0,405752839	0,594706376
219063_at	C1orf35	1,910557155	1,315897482	0,594659673
203219_s_at	APRT	3,562907192	2,968300712	0,59460648
212788_x_at	FTL	8,347200846	7,752750892	0,594449954
207467_x_at	CAST	4,383685466	3,789333093	0,594352374
217007_s_at	ADAM15	0,20740896	-0,386896102	0,594305062
203241_at	UVRAG	2,649978302	2,055739171	0,594239132
208630_at	HADHA	5,623297131	5,029330929	0,593966201
226337_at	GORAB	2,365121417	1,771157334	0,593964083
204766_s_at	NUDT1	3,327128828	2,733194118	0,59393471

226943_at	C12orf73	3,64600241	3,052211048	0,593791362
214055_x_at	PRRC2C	4,033407967	3,439619824	0,593788143
206741_at	C3orf32	-0,551284523	-1,145057014	0,593772492
1555129_at	-	-0,551284523	-1,145057014	0,593772492
1561978_at	LOC284798	-0,551284523	-1,145057014	0,593772492
229996_s_at	PCGF5	-0,551284523	-1,145057014	0,593772492
1552272_a_at	PRR22	-0,551284523	-1,145057014	0,593772492
208377_s_at	CACNA1F	-0,551284523	-1,145057014	0,593772492
213201_s_at	TNNT1	-0,551284523	-1,145057014	0,593772492
221706_s_at	USE1	2,295108393	1,701800759	0,593307634
218607_s_at	SDAD1	1,936380191	1,343200523	0,593179668
217710_x_at	ITPK1	0,556428218	-0,036543687	0,592971905
211603_s_at	ETV4	0,39105295	-0,201789721	0,592842671
223910_at	ERN2	0,39105295	-0,201789721	0,592842671
226268_at	RAB21	0,39105295	-0,201789721	0,592842671
235625_at	VPS41	1,476175815	0,883374248	0,592801567
209593_s_at	TOR1B	3,636292808	3,043944324	0,592348484
223445_at	DTNBP1	2,920736393	2,328492509	0,592243884
232794_at	LOC153682	0,677745787	0,085534992	0,592210795
211708_s_at	SCD	1,421391637	0,829309537	0,5920821
218898_at	FAM57A	1,528073958	0,936232181	0,591841777
203991_s_at	KDM6A	1,364555912	0,772793615	0,591762298
219303_at	RNF219	3,273854609	2,682180306	0,591674303
203523_at	LSP1	3,485638812	2,894109605	0,591529206
37232_at	KIAA0586	1,183225281	0,592020685	0,591204597
214532_x_at	POU5F1B	0,97663406	0,385582632	0,591051428
230847_at	WRNIP1	0,97663406	0,385582632	0,591051428
240921_at	-	-2,074223907	-2,665156778	0,590932871
239120_at	C1orf126	-2,074223907	-2,665156778	0,590932871
213516_at	AKAP13	-2,074223907	-2,665156778	0,590932871
231339_at	TSPYL6	-2,074223907	-2,665156778	0,590932871
240966_at	SH3TC2	-2,074223907	-2,665156778	0,590932871
238566_at	-	-2,074223907	-2,665156778	0,590932871
1563392_at	-	-2,074223907	-2,665156778	0,590932871
1559172_at	-	-2,074223907	-2,665156778	0,590932871
243803_at	LOC643037	-2,074223907	-2,665156778	0,590932871
240680_at	-	-2,074223907	-2,665156778	0,590932871
205290_s_at	BMP2	-2,074223907	-2,665156778	0,590932871
218550_s_at	LRRC20	1,225123479	0,634301128	0,590822351
223047_at	CMTM6	4,948102662	4,357501567	0,590601095
223086_x_at	MRPL51	6,153179494	5,562600136	0,590579358
200932_s_at	DCTN2	4,895653873	4,305252091	0,590401782
239800_at	UMPS	-0,180990326	-0,771340337	0,590350011
232938_at	SFMBT2	-0,180990326	-0,771340337	0,590350011
210086_at	HR	-0,180990326	-0,771340337	0,590350011
233993_at	MUC3	-0,180990326	-0,771340337	0,590350011
239012_at	RNF144B	-0,180990326	-0,771340337	0,590350011
204413_at	TRAF2	1,561816652	0,971607464	0,590209188
232003_at	PNMAL2	0,001114523	-0,589001171	0,590115694
225095_at	SPTLC2	1,610353504	1,020514937	0,589838567

207563_s_at	OGT	2,705643918	2,116147513	0,589496405
223554_s_at	RANGRF	1,857989965	1,268569037	0,589420928
233784_at	-	-1,802716385	-2,391863529	0,589147144
225983_s_at	VWA1	-1,802716385	-2,391863529	0,589147144
1568970_at	ADAM18	-1,802716385	-2,391863529	0,589147144
224350_at	-	-1,802716385	-2,391863529	0,589147144
1561895_at	-	-1,802716385	-2,391863529	0,589147144
204471_at	GAP43	-1,802716385	-2,391863529	0,589147144
230794_at	PELI2	-1,802716385	-2,391863529	0,589147144
1562973_at	-	-1,802716385	-2,391863529	0,589147144
1556504_at	-	-1,802716385	-2,391863529	0,589147144
1565597_at	-	-1,802716385	-2,391863529	0,589147144
201650_at	KRT19	-1,802716385	-2,391863529	0,589147144
205471_s_at	DACH1	-1,802716385	-2,391863529	0,589147144
1559928_at	PAPPA	-1,802716385	-2,391863529	0,589147144
207207_at	RBMXL2	-1,802716385	-2,391863529	0,589147144
1568689_at	LOC100631378	-1,802716385	-2,391863529	0,589147144
243393_at	C17orf64	-1,802716385	-2,391863529	0,589147144
242520_s_at	C1orf228	-1,802716385	-2,391863529	0,589147144
237152_at	PCDP1	-1,802716385	-2,391863529	0,589147144
1560679_at	LOC100506328	-1,802716385	-2,391863529	0,589147144
1554833_at	MCTP2	-1,802716385	-2,391863529	0,589147144
217305_s_at	ADCY10	-1,802716385	-2,391863529	0,589147144
240010_at	-	-1,802716385	-2,391863529	0,589147144
1563287_at	-	-1,802716385	-2,391863529	0,589147144
214270_s_at	MAPRE3	-1,802716385	-2,391863529	0,589147144
214575_s_at	AZU1	-1,802716385	-2,391863529	0,589147144
226068_at	SYK	-1,802716385	-2,391863529	0,589147144
1555994_at	DIAPH3-AS1	-1,802716385	-2,391863529	0,589147144
210487_at	DNTT	-1,802716385	-2,391863529	0,589147144
1565565_at	-	-2,163157732	-2,752097204	0,588939472
1561414_at	LOC401497	-2,163157732	-2,752097204	0,588939472
243277_x_at	MECOM	-2,163157732	-2,752097204	0,588939472
1553475_at	PRO1483	-2,163157732	-2,752097204	0,588939472
1569100_a_at	-	-2,163157732	-2,752097204	0,588939472
1559827_at	LOC401074	-2,163157732	-2,752097204	0,588939472
212352_s_at	TMED10	5,56221414	4,973363861	0,588850278
91816_f_at	MEX3D	0,926019989	0,337318436	0,588701552
223510_at	NRP2	-0,384193355	-0,972893339	0,588699984
209365_s_at	ECM1	-0,384193355	-0,972893339	0,588699984
1555542_at	AFAP1L1	-0,384193355	-0,972893339	0,588699984
213715_s_at	KANK3	-0,384193355	-0,972893339	0,588699984
227779_at	-	-0,384193355	-0,972893339	0,588699984
214762_at	ATP6V1G2	-0,384193355	-0,972893339	0,588699984
237031_at	-	-0,384193355	-0,972893339	0,588699984
203074_at	ANXA8 /// ANXA8L	-0,384193355	-0,972893339	0,588699984
213124_at	ZNF473	1,458255986	0,869824768	0,588431218
214194_at	DIS3	1,458255986	0,869824768	0,588431218
218241_at	GOLGA5	3,993377193	3,405078581	0,588298611
1553129_at	SVEP1	-1,982324124	-2,570522741	0,588198618

1564128_at	-	-1,982324124	-2,570522741	0,588198618
1564688_a_at	RAD9B	-1,982324124	-2,570522741	0,588198618
234686_at	SUGT1P1	-1,982324124	-2,570522741	0,588198618
214159_at	PLCE1	-1,982324124	-2,570522741	0,588198618
238313_at	-	-1,982324124	-2,570522741	0,588198618
230602_at	ACMSD	-1,982324124	-2,570522741	0,588198618
1562139_a_at	FOXP2	-1,982324124	-2,570522741	0,588198618
1569570_at	AGBL4	-1,982324124	-2,570522741	0,588198618
219285_s_at	NIN	-1,982324124	-2,570522741	0,588198618
1556259_at	-	-1,982324124	-2,570522741	0,588198618
207410_s_at	TLX2	-1,982324124	-2,570522741	0,588198618
240694_at	-	-1,982324124	-2,570522741	0,588198618
1553322_s_at	TEAD1	-1,982324124	-2,570522741	0,588198618
228056_s_at	NAPSB	-1,982324124	-2,570522741	0,588198618
1570364_at	ZNF709	-1,982324124	-2,570522741	0,588198618
214351_x_at	RPL13 /// SNORD6	8,084030212	7,495847708	0,588182503
225766_s_at	TNPO1	3,185443259	2,597466271	0,587976989
219999_at	MAN2A2	0,524545436	-0,063377083	0,587922519
201900_s_at	AKR1A1	5,737003362	5,149142454	0,587860907
208107_s_at	LOC81691	2,392346173	1,804519951	0,587826223
219833_s_at	EFHC1	1,510970097	0,923203038	0,587767059
203569_s_at	OFD1	4,00240646	3,414783177	0,587623283
214698_at	PTBP3	0,873789652	0,286279868	0,587509784
1555575_a_at	KDELR1	4,733987652	4,146536585	0,587451066
211614_at	ERVH-6	-1,369772723	-1,956681069	0,586908347
217228_s_at	ASB4	-1,369772723	-1,956681069	0,586908347
1559045_at	LOC100128288	-1,369772723	-1,956681069	0,586908347
243260_x_at	XKR6	-1,369772723	-1,956681069	0,586908347
236230_at	-	-1,369772723	-1,956681069	0,586908347
218454_at	PLBD1	-1,369772723	-1,956681069	0,586908347
233664_at	-	-1,369772723	-1,956681069	0,586908347
243538_at	-	-1,369772723	-1,956681069	0,586908347
234324_at	NHSL1	-1,369772723	-1,956681069	0,586908347
1560955_at	-	-1,369772723	-1,956681069	0,586908347
231477_at	-	-1,369772723	-1,956681069	0,586908347
237952_at	-	-1,369772723	-1,956681069	0,586908347
222100_at	CYP2E1	-1,369772723	-1,956681069	0,586908347
1558794_at	LOC728190	-1,369772723	-1,956681069	0,586908347
226695_at	PRRX1	-1,369772723	-1,956681069	0,586908347
235650_at	CDHR3 /// LOC100	-1,369772723	-1,956681069	0,586908347
226028_at	ROBO4	-1,369772723	-1,956681069	0,586908347
233624_at	-	-1,369772723	-1,956681069	0,586908347
216120_s_at	ATP2B2	-1,369772723	-1,956681069	0,586908347
220871_at	-	-1,369772723	-1,956681069	0,586908347
206837_at	ALX1	-1,369772723	-1,956681069	0,586908347
233717_x_at	-	-1,369772723	-1,956681069	0,586908347
241663_at	C3orf23	-1,369772723	-1,956681069	0,586908347
214362_at	RGS12	-1,369772723	-1,956681069	0,586908347
244596_at	-	-1,369772723	-1,956681069	0,586908347
221217_s_at	RBFOX1	-1,369772723	-1,956681069	0,586908347

1565880_at	-	-1,369772723	-1,956681069	0,586908347
230121_at	C1orf133	-1,369772723	-1,956681069	0,586908347
1563840_at	-	-1,369772723	-1,956681069	0,586908347
1560378_at	GRIK1-AS2	-1,369772723	-1,956681069	0,586908347
1557052_at	-	-1,369772723	-1,956681069	0,586908347
204945_at	PTPRN	-1,369772723	-1,956681069	0,586908347
202466_at	PAPD7	3,35759121	2,770696222	0,586894987
200946_x_at	GLUD1	4,37922506	3,792342525	0,586882535
204530_s_at	TOX	0,355751005	-0,231045907	0,586796912
233487_s_at	LRRC8A	0,355751005	-0,231045907	0,586796912
1568191_at	-	0,355751005	-0,231045907	0,586796912
219431_at	ARHGAP10	3,039689025	2,45323331	0,586455715
206152_at	AGAP2	0,648195588	0,061773582	0,586422006
236834_at	SCFD2	0,648195588	0,061773582	0,586422006
218288_s_at	CCDC90B	4,241697258	3,655528924	0,586168334
206477_s_at	NOVA2	1,20396005	0,61779983	0,58616022
218764_at	PRKCH	3,001575397	2,415420763	0,586154635
201311_s_at	SH3BGRL	4,667936611	4,081788629	0,586147982
211056_s_at	SRD5A1	2,295108393	1,709047448	0,586060945
222895_s_at	BCL11B	4,792135367	4,206178	0,585957367
203242_s_at	PDLIM5	1,047341799	0,461385738	0,585956061
219725_at	TREM2	0,764143511	0,178191865	0,585951646
218521_s_at	UBE2W	2,085817135	1,500059916	0,585757218
218495_at	UXT	5,820147197	5,234391931	0,585755267
212242_at	TUBA4A	4,274951805	3,689333494	0,585618311
212355_at	KHNYN	2,321828846	1,736450458	0,585378388
225530_at	MOB3A	3,77412549	3,188806254	0,585319235
227079_at	DHX8	1,686984721	1,101780212	0,585204509
235410_at	NPHP3	1,843966654	1,259081178	0,584885476
200960_x_at	CLTA	5,543354832	4,958563897	0,584790935
223238_s_at	PBRM1	3,925077605	3,340400371	0,584677233
222743_s_at	C11orf71	2,019346222	1,434743608	0,584602613
238773_at	METTL15	2,019346222	1,434743608	0,584602613
232432_s_at	SLC30A5	3,732463367	3,14842776	0,584035607
217873_at	CAB39	4,949910672	4,366031382	0,58387929
238199_x_at	COX3	4,060218357	3,47651952	0,583698836
210453_x_at	ATP5L	7,549321564	6,965638259	0,583683305
225708_at	MED29	2,16969527	1,586121802	0,583573468
207877_s_at	NVL	3,831656423	3,248126831	0,583529592
218265_at	SECISBP2	1,871521385	1,28853674	0,582984645
227536_at	ZC3H13	3,023747815	2,440833194	0,582914622
220966_x_at	ARPC5L	5,684686261	5,101837964	0,582848297
211599_x_at	MET	1,182925501	0,600385088	0,582540413
203731_s_at	ZKSCAN5	1,182925501	0,600385088	0,582540413
221221_s_at	KLHL3	1,578719607	0,996453274	0,582266333
225330_at	IGF1R	1,898032047	1,315897482	0,582134565
202849_x_at	GRK6	2,523100136	1,941115499	0,581984637
206625_at	PRPH2	-0,609040214	-1,19085694	0,581816726
210169_at	SEC14L5	-0,609040214	-1,19085694	0,581816726
241410_at	-	-0,609040214	-1,19085694	0,581816726

233409_at	RHBDL3	-0,609040214	-1,19085694	0,581816726
214458_at	TRAF3IP1	-0,609040214	-1,19085694	0,581816726
222185_at	PKNOX2	-0,609040214	-1,19085694	0,581816726
242521_at	LOC100505812	3,581038515	2,999351239	0,581687276
216199_s_at	MAP3K4	4,109088344	3,527465373	0,581622971
204089_x_at	MAP3K4	3,076375717	2,49476365	0,581612068
241209_at	DCAF6	-2,250580859	-2,832036647	0,581455788
240356_s_at	-	-2,250580859	-2,832036647	0,581455788
241281_at	-	-0,791294935	-1,372734086	0,581439151
1553043_a_at	CD300LF	-0,791294935	-1,372734086	0,581439151
226690_at	ADCYAP1R1	-0,791294935	-1,372734086	0,581439151
237880_at	LOC100506457	-0,791294935	-1,372734086	0,581439151
206171_at	ADORA3	-0,791294935	-1,372734086	0,581439151
236832_at	ADCY10P1	-0,791294935	-1,372734086	0,581439151
206569_at	IL24	-0,791294935	-1,372734086	0,581439151
242567_at	ABTB1	-0,791294935	-1,372734086	0,581439151
233306_at	-	-0,791294935	-1,372734086	0,581439151
1562425_at	FARP1	-0,791294935	-1,372734086	0,581439151
211798_x_at	IGLJ3	-0,791294935	-1,372734086	0,581439151
211663_x_at	PTGDS	-0,791294935	-1,372734086	0,581439151
216285_at	DGCR14 /// TSSK2	-0,791294935	-1,372734086	0,581439151
1565583_at	ZSCAN5A	-0,791294935	-1,372734086	0,581439151
1558053_s_at	TMED4	-0,230432956	-0,811769547	0,581336591
238100_at	AAK1	-0,230432956	-0,811769547	0,581336591
1565483_at	EGFR	-0,230432956	-0,811769547	0,581336591
208023_at	TNFRSF4	-0,230432956	-0,811769547	0,581336591
223468_s_at	RGMA	-0,230432956	-0,811769547	0,581336591
236806_at	-	-0,230432956	-0,811769547	0,581336591
212237_at	ASXL1	3,317909135	2,736663738	0,581245398
202130_at	RIOK3	4,348261798	3,767104697	0,581157101
238975_at	MMAB	1,024284941	0,443143223	0,581141718
238628_s_at	TRAPPC2L	1,024284941	0,443143223	0,581141718
218252_at	CKAP2	4,341822193	3,760893183	0,58092901
203555_at	PTPN18	1,716003175	1,135235995	0,58076718
217822_at	WBP11	4,856885829	4,276165612	0,580720218
208913_at	GGA2	2,127965541	1,547376668	0,580588873
223124_s_at	PITHD1	4,452318502	3,871747013	0,580571489
218962_s_at	TMEM168	2,949541692	2,36899666	0,580545032
209571_at	CIR1	1,528073958	0,947673886	0,580400073
226080_at	SSH2	1,960819106	1,380552339	0,580266767
201927_s_at	PKP4	1,92317941	1,343200523	0,579978887
1554117_at	CCDC60	0,319287178	-0,260526297	0,579813475
222495_at	TMEM167B	4,584332287	4,00463588	0,579696407
201771_at	SCAMP3	4,025386557	3,445797592	0,579588966
212880_at	WDR7	2,267141818	1,687645398	0,57949642
215531_s_at	GABRA5	-2,495152701	-3,074492332	0,579339632
220507_s_at	UPB1	-0,043925712	-0,623254098	0,579328387
1560713_a_at	ELFN2	-0,043925712	-0,623254098	0,579328387
243594_x_at	SPIRE2	-0,043925712	-0,623254098	0,579328387
1570420_at	STXBP2	-0,043925712	-0,623254098	0,579328387

1556056_at	SIK2	-0,043925712	-0,623254098	0,579328387
226260_x_at	ZNF358	-0,043925712	-0,623254098	0,579328387
1569426_at	-	-0,043925712	-0,623254098	0,579328387
207113_s_at	TNF	-0,043925712	-0,623254098	0,579328387
205298_s_at	BTN2A2	2,295108393	1,715863686	0,579244708
204429_s_at	SLC2A5	0,127732831	-0,45121326	0,57894609
204717_s_at	SLC29A2	0,127732831	-0,45121326	0,57894609
215369_at	-	0,127732831	-0,45121326	0,57894609
1561611_at	-	0,127732831	-0,45121326	0,57894609
231291_at	-	0,127732831	-0,45121326	0,57894609
226357_at	USP19	2,560606634	1,981728801	0,578877833
222887_s_at	TMEM127	1,421391637	0,842599219	0,578792418
219633_at	TTPAL	2,179708195	1,601076277	0,578631918
209146_at	MSMO1	5,163360593	4,58477881	0,578581783
1560995_s_at	-	-1,137208348	-1,715576125	0,578367777
242259_at	FAM187B	-1,137208348	-1,715576125	0,578367777
238270_x_at	-	-1,137208348	-1,715576125	0,578367777
231010_at	AP1AR	-1,137208348	-1,715576125	0,578367777
232943_at	-	-1,137208348	-1,715576125	0,578367777
217169_at	IGHA1 /// IGHG1 /,	-1,137208348	-1,715576125	0,578367777
1563208_s_at	C3orf65	-1,137208348	-1,715576125	0,578367777
239736_at	-	-1,137208348	-1,715576125	0,578367777
1556494_at	-	-1,137208348	-1,715576125	0,578367777
237125_at	-	-1,137208348	-1,715576125	0,578367777
1556201_at	RNASET2	-1,137208348	-1,715576125	0,578367777
224120_at	-	-1,137208348	-1,715576125	0,578367777
215124_at	ZNF550	-1,137208348	-1,715576125	0,578367777
227453_at	UNC13A	-1,137208348	-1,715576125	0,578367777
1553565_s_at	DDAH1	-1,137208348	-1,715576125	0,578367777
1567254_at	OR10D1P	-1,137208348	-1,715576125	0,578367777
212488_at	COL5A1	-1,137208348	-1,715576125	0,578367777
231197_at	PPP1R3F	-1,137208348	-1,715576125	0,578367777
234251_at	-	-1,137208348	-1,715576125	0,578367777
234353_at	CATSPERG	-1,137208348	-1,715576125	0,578367777
230940_at	LOC100288123	-1,137208348	-1,715576125	0,578367777
243470_at	-	-1,137208348	-1,715576125	0,578367777
215890_at	GM2A	-1,137208348	-1,715576125	0,578367777
1557389_at	LOC100505839	-1,137208348	-1,715576125	0,578367777
205319_at	PSCA	-1,137208348	-1,715576125	0,578367777
225830_at	PDZD8	-1,137208348	-1,715576125	0,578367777
1554938_a_at	ACOT11	-1,137208348	-1,715576125	0,578367777
227423_at	LRRC28	1,561816652	0,98381571	0,578000943
207859_s_at	CHRNB3	-0,437449947	-1,015398016	0,577948069
213961_s_at	-	-0,437449947	-1,015398016	0,577948069
236891_at	-	-0,437449947	-1,015398016	0,577948069
1556480_a_at	-	-0,437449947	-1,015398016	0,577948069
1565814_at	TRIM36	-0,437449947	-1,015398016	0,577948069
226097_at	FNDC5	-0,437449947	-1,015398016	0,577948069
1562924_at	LOC340357	-0,437449947	-1,015398016	0,577948069
1561481_at	-	-0,437449947	-1,015398016	0,577948069

205236_x_at	SOD3	-0,437449947	-1,015398016	0,577948069
1561847_at	NUDT17	-0,437449947	-1,015398016	0,577948069
230950_at	CNDP2	-0,437449947	-1,015398016	0,577948069
234590_x_at	-	-0,437449947	-1,015398016	0,577948069
241610_x_at	-	-0,437449947	-1,015398016	0,577948069
204136_at	COL7A1	-0,437449947	-1,015398016	0,577948069
222394_at	PDCD6IP	1,816938997	1,239268196	0,577670802
203358_s_at	EZH2	5,256557379	4,678997829	0,57755955
203547_at	CD4	2,401508182	1,823960141	0,577548041
215424_s_at	SNW1	4,211840302	3,634723971	0,577116331
226736_at	CHURC1	2,50022283	1,923216608	0,577006222
208979_at	NCOA6	2,983826368	2,406959565	0,576866803
225273_at	WWC3	2,949541692	2,373249966	0,576291726
211152_s_at	HTRA2	3,161901883	2,585619776	0,576282107
204629_at	PARVB	2,019346222	1,44320939	0,576136832
205631_at	KIAA0586	2,019346222	1,44320939	0,576136832
203600_s_at	FAM193A	2,16969527	1,593605508	0,576089762
224655_at	AK3	6,078826967	5,502741595	0,576085371
203017_s_at	SSX2IP	1,775035811	1,199296812	0,575738999
212213_x_at	OPA1	3,353217129	2,777528602	0,575688526
1569321_at	-	0,458905032	-0,116767475	0,575672506
218948_at	QRSL1	0,458905032	-0,116767475	0,575672506
238797_at	TRIM11	0,458905032	-0,116767475	0,575672506
233541_at	LIMD1-AS1	0,458905032	-0,116767475	0,575672506
236467_at	ERICH1 /// FLJ002	0,458905032	-0,116767475	0,575672506
209334_s_at	PSMD9	0,820160788	0,244646697	0,575514091
243807_at	NCOA7	0,820160788	0,244646697	0,575514091
222401_s_at	TMEM50A	6,105191431	5,530302217	0,574889215
223025_s_at	AP1M1	1,458255986	0,883374248	0,574881738
213072_at	CYHR1	1,225123479	0,65030602	0,574817459
202128_at	KIAA0317	2,685337919	2,110584371	0,574753548
229573_at	USP9X	0,58788177	0,013190398	0,574691372
229935_s_at	MLL	-0,994186487	-1,568385657	0,57419917
1566734_at	LOC283454	-0,994186487	-1,568385657	0,57419917
207249_s_at	SLC28A2	-0,994186487	-1,568385657	0,57419917
221403_s_at	INSL6	-0,994186487	-1,568385657	0,57419917
241842_x_at	C19orf45	-0,994186487	-1,568385657	0,57419917
1561089_at	-	-0,994186487	-1,568385657	0,57419917
244592_at	-	-0,994186487	-1,568385657	0,57419917
238892_at	-	-0,994186487	-1,568385657	0,57419917
1561277_at	LOC339298	-0,994186487	-1,568385657	0,57419917
239325_at	DNAJC27-AS1	-0,994186487	-1,568385657	0,57419917
206596_s_at	NRL	-0,994186487	-1,568385657	0,57419917
227860_at	CPXM1	-0,994186487	-1,568385657	0,57419917
1563949_at	-	-0,994186487	-1,568385657	0,57419917
1557693_at	LOC100507299	-0,994186487	-1,568385657	0,57419917
232454_at	-	-0,994186487	-1,568385657	0,57419917
217022_s_at	IGH@ /// IGHA1 //	-0,994186487	-1,568385657	0,57419917
210382_at	SCTR	-0,994186487	-1,568385657	0,57419917
214456_x_at	SAA1 /// SAA2	-0,994186487	-1,568385657	0,57419917

221974_at	IPW /// LOC10050	-0,994186487	-1,568385657	0,57419917
224360_s_at	PACSIN1	-0,994186487	-1,568385657	0,57419917
204392_at	CAMK1	-0,994186487	-1,568385657	0,57419917
1555132_at	-	-0,994186487	-1,568385657	0,57419917
238177_at	SLC6A19	-0,994186487	-1,568385657	0,57419917
244692_at	CYP4F22	-0,994186487	-1,568385657	0,57419917
228461_at	SH3RF3	-0,994186487	-1,568385657	0,57419917
1562788_at	LOC254099	-0,994186487	-1,568385657	0,57419917
210779_x_at	GEMIN2	2,590269697	2,016139502	0,574130195
202216_x_at	NFYC	2,791258167	2,2171284	0,574129766
205329_s_at	SNX4	4,409540371	3,83554191	0,573998461
214151_s_at	CCPG1 /// DYX1C1	1,5944803	1,020514937	0,573965363
201022_s_at	DSTN	6,068019211	5,49410554	0,573913671
213812_s_at	CAMKK2	4,211840302	3,638116884	0,573723418
200788_s_at	PEA15	3,485638812	2,912181979	0,573456833
32540_at	PPP3CC	-1,019124414	-1,592451078	0,573326664
235692_at	SH3KBP1	3,649735397	3,0766152	0,573120198
215148_s_at	APBA3	1,898032047	1,324949741	0,573082306
203233_at	IL4R	1,898032047	1,324949741	0,573082306
229107_at	-	0,282358733	-0,290694975	0,573053708
236321_at	FAM200B	1,802733148	1,2298509	0,572882248
234904_x_at	ELAVL4	-1,893506789	-2,466349414	0,572842625
1570222_at	NDST4	-1,893506789	-2,466349414	0,572842625
1557645_at	-	-1,893506789	-2,466349414	0,572842625
1570114_at	MDGA2	-1,893506789	-2,466349414	0,572842625
224107_at	-	-1,893506789	-2,466349414	0,572842625
217316_at	OR7A10	-1,893506789	-2,466349414	0,572842625
240161_s_at	CDC20B	-1,893506789	-2,466349414	0,572842625
1558034_s_at	CP	-1,893506789	-2,466349414	0,572842625
229554_at	LUM	-1,893506789	-2,466349414	0,572842625
1553296_at	GPR128	-1,893506789	-2,466349414	0,572842625
223737_x_at	CHST9	-1,893506789	-2,466349414	0,572842625
240706_at	-	-1,893506789	-2,466349414	0,572842625
243732_at	LOC100506629	-1,893506789	-2,466349414	0,572842625
1565897_at	-	-1,893506789	-2,466349414	0,572842625
1555454_at	LITAF	-1,893506789	-2,466349414	0,572842625
1569716_at	-	-1,893506789	-2,466349414	0,572842625
1568930_at	EFCAB1	-1,893506789	-2,466349414	0,572842625
1570283_at	-	-1,893506789	-2,466349414	0,572842625
241236_at	-	-1,893506789	-2,466349414	0,572842625
209930_s_at	NFE2	-1,893506789	-2,466349414	0,572842625
228595_at	HSD17B1	1,640193838	1,067365565	0,572828273
223183_at	AGPAT3	2,096248267	1,523429058	0,572819209
228374_at	R3HCC1L	1,345611839	0,772793615	0,572818224
204825_at	MELK	4,533526699	3,960716395	0,572810304
201803_at	POLR2B	5,157548518	4,58477881	0,572769707
218587_s_at	POGLUT1	3,309267982	2,736663738	0,572604244
226106_at	RNF141	2,649978302	2,077419168	0,572559134
1565527_x_at	TCP11L2	-1,289805289	-1,862269442	0,572464153
207322_at	ITSN1	-1,289805289	-1,862269442	0,572464153

202526_at	SMAD4	-1,289805289	-1,862269442	0,572464153
1569490_at	FNDC3B	-1,289805289	-1,862269442	0,572464153
216410_at	-	-1,289805289	-1,862269442	0,572464153
242950_x_at	-	-1,289805289	-1,862269442	0,572464153
231626_at	TPH1	-1,289805289	-1,862269442	0,572464153
1555273_at	GALNTL6	-1,289805289	-1,862269442	0,572464153
237687_at	LOC283737	-1,289805289	-1,862269442	0,572464153
221300_at	NPAP1	-1,289805289	-1,862269442	0,572464153
232830_at	RNF32	-1,289805289	-1,862269442	0,572464153
233484_at	-	-1,289805289	-1,862269442	0,572464153
1566080_at	-	-1,289805289	-1,862269442	0,572464153
236868_at	-	-1,289805289	-1,862269442	0,572464153
207179_at	TLX1	-1,289805289	-1,862269442	0,572464153
1560844_at	-	-1,289805289	-1,862269442	0,572464153
207092_at	LEP	-1,289805289	-1,862269442	0,572464153
230373_at	LOC100505483	-1,289805289	-1,862269442	0,572464153
228133_s_at	MYH11	-1,289805289	-1,862269442	0,572464153
215362_at	-	-1,289805289	-1,862269442	0,572464153
206256_at	CPN1	-1,289805289	-1,862269442	0,572464153
1557077_a_at	-	-1,289805289	-1,862269442	0,572464153
233301_at	OXCT2	-1,289805289	-1,862269442	0,572464153
208484_at	HIST1H1A	-1,289805289	-1,862269442	0,572464153
1562717_at	LINC00299	-1,289805289	-1,862269442	0,572464153
216959_x_at	NRCAM	-1,289805289	-1,862269442	0,572464153
236008_at	LOC100128909	-1,289805289	-1,862269442	0,572464153
1557319_at	-	-0,088974936	-0,661178575	0,572203639
205275_at	GTPBP1	-0,088974936	-0,661178575	0,572203639
1554752_a_at	-	-0,088974936	-0,661178575	0,572203639
234928_x_at	RUNX3	-0,088974936	-0,661178575	0,572203639
1555165_a_at	PGPEP1	-0,088974936	-0,661178575	0,572203639
237487_at	-	-0,088974936	-0,661178575	0,572203639
229282_at	GATA6	-0,088974936	-0,661178575	0,572203639
224789_at	DCAF12	2,798156197	2,226451905	0,571704292
225398_at	RPUSD4	3,895112363	3,323485083	0,57162728
1569139_s_at	FAM53A	0,08619576	-0,484558493	0,570754253
233396_s_at	CSRP2BP	0,08619576	-0,484558493	0,570754253
229499_at	CAPN13	0,08619576	-0,484558493	0,570754253
234026_at	LOC100287177	0,08619576	-0,484558493	0,570754253
216958_s_at	IVD	1,493647653	0,923203038	0,570444615
203610_s_at	TRIM38	2,575362572	2,004917973	0,570444599
226467_at	TMCO7	1,625482993	1,055317091	0,570165902
235158_at	TMEM209	3,792209911	3,222133192	0,570076719
201339_s_at	SCP2	5,569095514	4,99912348	0,569972034
214614_at	MNX1	2,053550345	1,483698568	0,569851777
227031_at	SNX13	1,20396005	0,634301128	0,569658922
225032_at	FNDC3B	2,312738346	1,743236669	0,569501677
201409_s_at	PPP1CB	4,749149445	4,179683441	0,569466004
234950_s_at	RFWD2	3,64600241	3,0766152	0,569387211
224600_at	CGGBP1	3,948420174	3,379204729	0,569215444
206196_s_at	RUNDC3A	-0,280273599	-0,84928999	0,569016391

239505_at	-	-0,280273599	-0,84928999	0,569016391
206183_s_at	HERC3	-0,280273599	-0,84928999	0,569016391
229881_at	KLF12	-0,280273599	-0,84928999	0,569016391
1558584_at	UBL4B	-0,280273599	-0,84928999	0,569016391
243553_x_at	TRAF3IP2-AS1	-0,280273599	-0,84928999	0,569016391
232211_at	PPP1R3F	-0,280273599	-0,84928999	0,569016391
224646_x_at	H19 /// MIR675	-0,280273599	-0,84928999	0,569016391
208478_s_at	BAX	2,878891456	2,309925448	0,568966008
209229_s_at	PPP6R1	2,843031657	2,274084398	0,568947259
46665_at	SEMA4C	3,063419693	2,494687142	0,568732551
202392_s_at	PISD	1,788922638	1,220470658	0,56845198
238504_at	C6orf57	2,392346173	1,823960141	0,568386032
205074_at	SLC22A5	0,677745787	0,109458907	0,56828688
209089_at	RAB5A	5,22851938	4,660441801	0,568077579
1556592_at	-	-2,336678463	-2,904470894	0,567792431
225274_at	PCYOX1	3,107338728	2,539809146	0,567529582
228835_at	-	0,556428218	-0,011001236	0,567429454
231914_at	NUDT14	0,556428218	-0,011001236	0,567429454
212931_at	TCF20	3,614400403	3,046977905	0,567422498
234359_at	-	0,873789652	0,306454867	0,567334785
207320_x_at	STAU1	4,919780177	4,35249495	0,567285227
218712_at	C1orf109	2,248155141	1,680963921	0,56719122
207595_s_at	BMP1	0,244681185	-0,322340048	0,567021233
222709_at	ATG7	0,244681185	-0,322340048	0,567021233
242317_at	HIGD1A	2,042564225	1,475630127	0,566934097
208883_at	UBR5	2,33082239	1,76396664	0,56685575
212512_s_at	CARM1	1,983901861	1,417157926	0,566743935
201078_at	TM9SF2	5,369272323	4,802891201	0,566381122
212890_at	SLC38A10	1,701542258	1,135235995	0,566306263
203660_s_at	PCNT	3,378744007	2,812515887	0,56622812
217185_s_at	ZNF259 /// ZNF259	2,943764123	2,37755543	0,566208693
1570454_at	EIF4EBP2	-0,856259172	-1,422257026	0,565997854
221364_at	GRID2	-0,856259172	-1,422257026	0,565997854
216558_x_at	IGHA1 /// IGHD //	-0,856259172	-1,422257026	0,565997854
207587_at	CRYGA	-0,856259172	-1,422257026	0,565997854
214134_at	C2orf55	-0,856259172	-1,422257026	0,565997854
207423_s_at	ADAM20	-0,856259172	-1,422257026	0,565997854
1563003_at	ITGAX	-0,856259172	-1,422257026	0,565997854
226658_at	PDPN	-0,856259172	-1,422257026	0,565997854
218033_s_at	SNN	-0,856259172	-1,422257026	0,565997854
207085_x_at	CSF2RA	-0,856259172	-1,422257026	0,565997854
1563709_at	LOC100127955 ///	-0,856259172	-1,422257026	0,565997854
1561459_at	-	-0,856259172	-1,422257026	0,565997854
211248_s_at	CHRD	-0,856259172	-1,422257026	0,565997854
1553303_at	C16orf46	-0,856259172	-1,422257026	0,565997854
216482_x_at	ZNF79	-0,856259172	-1,422257026	0,565997854
216663_s_at	ZMYND10	-0,856259172	-1,422257026	0,565997854
242951_at	-	-0,856259172	-1,422257026	0,565997854
227759_at	PCSK9	-0,856259172	-1,422257026	0,565997854
1566931_at	TFB2M	-0,856259172	-1,422257026	0,565997854

1566932_x_at	TFB2M	-0,856259172	-1,422257026	0,565997854
207199_at	TERT	-0,856259172	-1,422257026	0,565997854
1555725_a_at	RGS5	-0,66934516	-1,234835326	0,565490167
203439_s_at	STC2	-0,66934516	-1,234835326	0,565490167
242010_at	-	-0,66934516	-1,234835326	0,565490167
233762_at	-	-0,66934516	-1,234835326	0,565490167
209706_at	NKX3-1	-0,66934516	-1,234835326	0,565490167
210762_s_at	DLC1	-0,66934516	-1,234835326	0,565490167
227389_x_at	-	-0,66934516	-1,234835326	0,565490167
203684_s_at	BCL2	-0,66934516	-1,234835326	0,565490167
207048_at	SLC6A11	-0,66934516	-1,234835326	0,565490167
201951_at	ALCAM	2,621163856	2,055739171	0,565424685
1554627_a_at	ASCC1	3,434684504	2,869317249	0,565367255
212241_at	GCOM1 /// MYZAF	1,561816652	0,996453274	0,565363379
212181_s_at	NUDT4 /// NUDT4I	3,513985141	2,948635566	0,565349576
210276_s_at	TRIOBP	1,843966654	1,278654284	0,565312371
202333_s_at	UBE2B	4,837645002	4,272475625	0,565169378
221834_at	LOC100507577 ///	1,182925501	0,61779983	0,565125671
211177_s_at	TXNRD2	1,182925501	0,61779983	0,565125671
224748_at	DCAF7	4,046717183	3,481966713	0,56475047
225886_at	DDX5	2,597647262	2,03313004	0,564517222
210567_s_at	SKP2	2,843031657	2,278572464	0,564459194
244562_s_at	-	-0,49284695	-1,057301851	0,564454901
239216_at	TEKT1	-0,49284695	-1,057301851	0,564454901
210072_at	CCL19	-0,49284695	-1,057301851	0,564454901
1561421_a_at	-	-0,49284695	-1,057301851	0,564454901
239463_at	-	-0,49284695	-1,057301851	0,564454901
1555404_a_at	DUOXA1	-0,49284695	-1,057301851	0,564454901
232863_at	ZNF815P /// ZNF8(	-0,49284695	-1,057301851	0,564454901
232299_at	C2orf82	-0,49284695	-1,057301851	0,564454901
231561_s_at	APOC2	-0,49284695	-1,057301851	0,564454901
205071_x_at	XRCC4	2,096248267	1,531877749	0,564370518
203427_at	ASF1A	4,721089228	4,157029702	0,564059526
231992_x_at	-	2,031158928	1,46719264	0,563966288
228149_at	C7orf60	2,063971513	1,500059916	0,563911597
207427_at	ACR	0,045517965	-0,518232988	0,563750953
240325_x_at	-	0,045517965	-0,518232988	0,563750953
1559863_a_at	AFG3L1P	0,045517965	-0,518232988	0,563750953
1558066_s_at	TBC1D16	0,045517965	-0,518232988	0,563750953
1553613_s_at	FOXC1	0,045517965	-0,518232988	0,563750953
243777_at	RAB7L1	0,045517965	-0,518232988	0,563750953
222635_s_at	MED28	2,468978138	1,905294823	0,563683315
236555_at	TRAF3IP2-AS1	1,364555912	0,80114751	0,563408402
202594_at	LEPROTL1	3,991087145	3,427694878	0,563392267
201618_x_at	GPAA1	2,705643918	2,142313983	0,563329935
208746_x_at	ATP5L	7,554021775	6,990755117	0,563266658
229695_at	-	0,39105295	-0,172194225	0,563247175
239720_at	ZGLP1	0,39105295	-0,172194225	0,563247175
239973_at	-	0,39105295	-0,172194225	0,563247175
208646_at	-	7,415164557	6,852086029	0,563078528

33494_at	ETFDH	1,855836377	1,292848271	0,562988106
218915_at	NF2	2,817838865	2,255165935	0,56267293
1554201_at	CABP4	0,648195588	0,085534992	0,562660596
222999_s_at	CCNL2	4,256620804	3,694322866	0,562297938
225623_at	KIAA1737	1,5944803	1,032267596	0,562212704
200855_at	NCOR1	1,5944803	1,032267596	0,562212704
241817_at	C3orf62 /// MIR42	0,20740896	-0,354781582	0,562190542
206590_x_at	DRD2	0,20740896	-0,354781582	0,562190542
241006_at	-	-0,135065865	-0,697182596	0,562116731
211176_s_at	PAX4	-0,135065865	-0,697182596	0,562116731
1552355_s_at	C19orf26	-0,135065865	-0,697182596	0,562116731
244656_at	RASL10B	-0,135065865	-0,697182596	0,562116731
224406_s_at	FCRL5	-0,135065865	-0,697182596	0,562116731
1556213_a_at	BTG3	-0,135065865	-0,697182596	0,562116731
241637_at	-	-0,135065865	-0,697182596	0,562116731
228705_at	CAPN12	-0,135065865	-0,697182596	0,562116731
210827_s_at	ELF3	-0,135065865	-0,697182596	0,562116731
235545_at	DEPDC1	3,581038515	3,019022994	0,562015521
215210_s_at	DLST	4,153515595	3,591688835	0,561826761
39854_r_at	PNPLA2	3,171268117	2,609490599	0,561777518
220194_at	NSUN7	-2,074223907	-2,635549085	0,561325178
243373_at	-	-2,074223907	-2,635549085	0,561325178
241055_at	-	-2,074223907	-2,635549085	0,561325178
235075_at	DSG3	-2,074223907	-2,635549085	0,561325178
1555581_a_at	TP63	-2,074223907	-2,635549085	0,561325178
207259_at	LINC00483	-2,074223907	-2,635549085	0,561325178
220205_at	TPTE	-2,074223907	-2,635549085	0,561325178
224821_at	ABHD14B	2,560606634	1,999354538	0,561252096
236528_at	-	0,847529938	0,286279868	0,56125007
229507_at	FAM212A	0,847529938	0,286279868	0,56125007
212458_at	SPRED2	2,955426542	2,394276989	0,561149553
1566201_at	-	-1,624955693	-2,186086603	0,56113091
238287_at	SLC7A13	-1,624955693	-2,186086603	0,56113091
241384_x_at	GK5	-1,624955693	-2,186086603	0,56113091
231230_at	KCNK10	-1,624955693	-2,186086603	0,56113091
1563531_at	-	-1,624955693	-2,186086603	0,56113091
1563379_at	-	-1,624955693	-2,186086603	0,56113091
207063_at	NCRNA00185 /// T	-1,624955693	-2,186086603	0,56113091
237000_at	-	-1,624955693	-2,186086603	0,56113091
239244_at	LOC100507616	-1,624955693	-2,186086603	0,56113091
1559244_at	FMN2	-1,624955693	-2,186086603	0,56113091
235656_s_at	-	-1,624955693	-2,186086603	0,56113091
220276_at	RERGL	-1,624955693	-2,186086603	0,56113091
209108_at	TSPAN6	-1,624955693	-2,186086603	0,56113091
210049_at	SERPINC1	-1,624955693	-2,186086603	0,56113091
217236_x_at	IGH@ /// IGHA1 //	-1,624955693	-2,186086603	0,56113091
1555028_at	BRD3	-1,624955693	-2,186086603	0,56113091
243770_at	ZNF483	-1,624955693	-2,186086603	0,56113091
234638_at	-	-1,624955693	-2,186086603	0,56113091
234848_at	-	-1,624955693	-2,186086603	0,56113091

243347_at	-	-1,624955693	-2,186086603	0,56113091
237454_at	-	-1,624955693	-2,186086603	0,56113091
234141_s_at	LOC286059	-1,624955693	-2,186086603	0,56113091
234039_at	TANC1	-1,624955693	-2,186086603	0,56113091
238450_at	PFKFB2	-1,624955693	-2,186086603	0,56113091
227992_s_at	LINC00085	-1,624955693	-2,186086603	0,56113091
214890_s_at	FAM149A	-1,624955693	-2,186086603	0,56113091
206283_s_at	TAL1	-1,624955693	-2,186086603	0,56113091
210593_at	KIAA0913 /// SAT1	-1,624955693	-2,186086603	0,56113091
237014_at	MYBL1	-1,624955693	-2,186086603	0,56113091
1566576_at	-	-1,624955693	-2,186086603	0,56113091
210321_at	GZMH	-1,624955693	-2,186086603	0,56113091
1555268_a_at	GRID1	-1,624955693	-2,186086603	0,56113091
217557_s_at	CPM	-1,624955693	-2,186086603	0,56113091
219836_at	ZBED2	-1,624955693	-2,186086603	0,56113091
221925_s_at	CSPP1	0,524545436	-0,036543687	0,561089123
229988_at	-	0,524545436	-0,036543687	0,561089123
213512_at	C14orf79	0,524545436	-0,036543687	0,561089123
228787_s_at	BCAS4	0,524545436	-0,036543687	0,561089123
242307_at	ZNF789	1,760255626	1,199296812	0,560958813
209175_at	SEC23IP	1,995527416	1,434743608	0,560783808
202226_s_at	CRK	2,21987062	1,659532492	0,560338129
207198_s_at	LIMS1	4,529665206	3,969510541	0,560154664
226739_at	RNF169	2,692288672	2,132145748	0,560142923
221895_at	MOSPD2	3,161901883	2,601822502	0,560079382
1564007_at	-	-2,163157732	-2,723114724	0,559956993
241177_at	-	-2,163157732	-2,723114724	0,559956993
1553497_at	LINC00615	-2,163157732	-2,723114724	0,559956993
228979_at	SFTA3	-2,163157732	-2,723114724	0,559956993
1558494_at	-	-2,163157732	-2,723114724	0,559956993
219556_at	C16orf59	1,402495885	0,842599219	0,559896665
218968_s_at	ZFP64	2,878891456	2,31904917	0,559842286
225098_at	ABI2	2,33082239	1,771157334	0,559665056
202512_s_at	ATG5	1,857989965	1,298349073	0,559640892
202939_at	ZMPSTE24	4,381466431	3,82195864	0,559507791
212048_s_at	YARS	5,181001846	4,621587556	0,55941429
207507_s_at	ATP5G3	6,756191228	6,197058119	0,559133109
234952_s_at	PHRF1	1,788922638	1,2298509	0,559071738
204003_s_at	NUPL2	2,356619952	1,797682937	0,558937014
205150_s_at	TRIL	-1,537851782	-2,09678811	0,558936328
237606_at	CD53	-1,537851782	-2,09678811	0,558936328
1561455_at	LOC284294	-1,537851782	-2,09678811	0,558936328
243746_at	IGHMBP2	-1,537851782	-2,09678811	0,558936328
233769_at	-	-1,537851782	-2,09678811	0,558936328
239877_at	LOC157740	-1,537851782	-2,09678811	0,558936328
1555265_at	ABCC13	-1,537851782	-2,09678811	0,558936328
205373_at	CTNNA2	-1,537851782	-2,09678811	0,558936328
1561268_at	-	-1,537851782	-2,09678811	0,558936328
203400_s_at	TF	-1,537851782	-2,09678811	0,558936328
234896_at	-	-1,537851782	-2,09678811	0,558936328

240981_at	-	-1,537851782	-2,09678811	0,558936328
237120_at	KRT77	-1,537851782	-2,09678811	0,558936328
230957_at	PCDHB19P	-1,537851782	-2,09678811	0,558936328
214029_at	SLC25A42	-1,537851782	-2,09678811	0,558936328
241173_at	-	-1,537851782	-2,09678811	0,558936328
233804_at	LOC440131	-1,537851782	-2,09678811	0,558936328
207073_at	CDKL2	-1,537851782	-2,09678811	0,558936328
1559034_at	SIRPB2	-1,537851782	-2,09678811	0,558936328
243828_at	-	-1,537851782	-2,09678811	0,558936328
241383_at	ZNF385C	-1,537851782	-2,09678811	0,558936328
238128_at	TMEM200C	-1,537851782	-2,09678811	0,558936328
207354_at	CCL16	-1,537851782	-2,09678811	0,558936328
214350_at	SNTB2	-1,537851782	-2,09678811	0,558936328
1569588_x_at	-	-1,537851782	-2,09678811	0,558936328
1563036_at	-	-1,537851782	-2,09678811	0,558936328
1564421_at	LOC100507435	-1,537851782	-2,09678811	0,558936328
241256_at	LOC100131283	-1,537851782	-2,09678811	0,558936328
242452_at	-	-1,537851782	-2,09678811	0,558936328
241071_at	-	-1,537851782	-2,09678811	0,558936328
216330_s_at	POU6F1	-1,537851782	-2,09678811	0,558936328
235858_at	CREBBP	-1,537851782	-2,09678811	0,558936328
201981_at	PAPPA	-1,537851782	-2,09678811	0,558936328
217422_s_at	CD22	-1,537851782	-2,09678811	0,558936328
225247_at	C19orf6	2,042564225	1,483698568	0,558865657
226396_at	TEN1 /// TEN1-CDI	1,671397663	1,112548615	0,558849047
238337_s_at	DNAJC21	4,341822193	3,783043925	0,558778268
219239_s_at	ZNF654	2,295108393	1,736450458	0,558657936
221562_s_at	SIRT3	1,225123479	0,666486137	0,558637342
202795_x_at	TRIOBP	2,523100136	1,964670566	0,558429571
219446_at	RIC8B	2,2388565	1,680963921	0,557892579
213026_at	ATG12	4,65719538	4,099530192	0,557665188
1557415_s_at	LETM2	0,355751005	-0,201789721	0,557540726
231543_at	PLEKHA8	0,355751005	-0,201789721	0,557540726
223892_s_at	TMBIM4	5,685228499	5,127891846	0,557336653
237551_at	-	-1,712732543	-2,27001641	0,557283867
228946_at	INTU	-1,712732543	-2,27001641	0,557283867
1570168_at	-	-1,712732543	-2,27001641	0,557283867
235182_at	ISM1	-1,712732543	-2,27001641	0,557283867
232316_at	SAMD15	-1,712732543	-2,27001641	0,557283867
1562399_at	-	-1,712732543	-2,27001641	0,557283867
216452_at	TRPM3	-1,712732543	-2,27001641	0,557283867
219930_at	KLF8	-1,712732543	-2,27001641	0,557283867
242275_at	-	-1,712732543	-2,27001641	0,557283867
238197_at	GATA5	-1,712732543	-2,27001641	0,557283867
207012_at	MMP16	-1,712732543	-2,27001641	0,557283867
207393_at	HCRTR2	-1,712732543	-2,27001641	0,557283867
208027_s_at	TLL2	-1,712732543	-2,27001641	0,557283867
230806_s_at	FAM65A	-1,712732543	-2,27001641	0,557283867
230153_at	NEK9	-1,712732543	-2,27001641	0,557283867
1570490_at	-	-1,712732543	-2,27001641	0,557283867

219945_at	DDX25	-1,712732543	-2,27001641	0,557283867
210122_at	PRM2	-1,712732543	-2,27001641	0,557283867
241593_x_at	-	-1,712732543	-2,27001641	0,557283867
217532_x_at	-	-1,712732543	-2,27001641	0,557283867
205493_s_at	DPYSL4	-1,712732543	-2,27001641	0,557283867
235936_at	LOC254559	-1,712732543	-2,27001641	0,557283867
1556192_x_at	-	-1,712732543	-2,27001641	0,557283867
205112_at	PLCE1	-1,712732543	-2,27001641	0,557283867
211659_at	GPR135	-1,712732543	-2,27001641	0,557283867
228033_at	E2F7	2,908690075	2,351470274	0,557219801
225770_at	RSPRY1	3,142467702	2,585619776	0,556847926
242421_at	-	-0,332405896	-0,889222211	0,556816315
224712_x_at	C19orf42	-0,332405896	-0,889222211	0,556816315
229275_at	IGFN1	-0,332405896	-0,889222211	0,556816315
236571_at	-	0,618277321	0,061773582	0,556503739
221907_at	TRMT61A	1,528073958	0,971607464	0,556466494
226242_at	C1orf131	4,693076946	4,136904703	0,556172244
222832_s_at	MFF	5,181001846	4,624879928	0,556121918
217713_x_at	-	1,071336699	0,515457602	0,555879096
1553526_at	NLRP8	0,001114523	-0,554696666	0,555811189
220076_at	ANKH	0,001114523	-0,554696666	0,555811189
221065_s_at	CHST8	0,001114523	-0,554696666	0,555811189
221406_s_at	MSH5 /// MSH5-S/	0,001114523	-0,554696666	0,555811189
1560278_at	LOC221122	0,001114523	-0,554696666	0,555811189
242614_at	-	0,001114523	-0,554696666	0,555811189
234881_at	-	0,001114523	-0,554696666	0,555811189
200043_at	ERH	6,511307451	5,955591086	0,555716365
201250_s_at	SLC2A1	2,199941239	1,644429915	0,555511324
210415_s_at	ODF2	1,843966654	1,28853674	0,555429914
219931_s_at	KLHL12	1,843966654	1,28853674	0,555429914
203091_at	FUBP1	3,336134047	2,780838243	0,555295804
228917_at	-	0,491906512	-0,063377083	0,555283595
213091_at	CRTC1	1,267146969	0,711934445	0,555212524
218855_at	TPRA1	1,610353504	1,055317091	0,555036412
218893_at	ISOC2	2,436165655	1,881145546	0,555020108
227796_at	ZFP62	2,914752575	2,359892448	0,554860128
201213_at	PPP1R7	1,898032047	1,343200523	0,554831524
1562731_s_at	MDS2	0,167727503	-0,386896102	0,554623605
241978_at	-	0,167727503	-0,386896102	0,554623605
203111_s_at	PTK2B	0,167727503	-0,386896102	0,554623605
221461_at	TAS2R9	-1,06282519	-1,616924751	0,554099561
241063_at	-	-1,06282519	-1,616924751	0,554099561
238033_at	HEXDC	-1,06282519	-1,616924751	0,554099561
237237_at	-	-1,06282519	-1,616924751	0,554099561
1561400_at	-	-1,06282519	-1,616924751	0,554099561
241154_x_at	-	-1,06282519	-1,616924751	0,554099561
216339_s_at	TNXA /// TNXB	-1,06282519	-1,616924751	0,554099561
237535_x_at	NRXN1	-1,06282519	-1,616924751	0,554099561
1559017_at	LOC100507351	-1,06282519	-1,616924751	0,554099561
1568770_at	MYLK	-1,06282519	-1,616924751	0,554099561

241615_x_at	-	-1,06282519	-1,616924751	0,554099561
240944_at	-	-1,06282519	-1,616924751	0,554099561
226926_at	DMKN	-1,06282519	-1,616924751	0,554099561
224408_at	MCHR2	-1,06282519	-1,616924751	0,554099561
1561449_at	-	-1,06282519	-1,616924751	0,554099561
235156_at	BRWD3	-1,06282519	-1,616924751	0,554099561
237260_at	LOC100505978	-1,06282519	-1,616924751	0,554099561
238226_at	FAM70B	-1,06282519	-1,616924751	0,554099561
242497_at	TRAFD1	-1,06282519	-1,616924751	0,554099561
231466_at	FAM71F1	-1,06282519	-1,616924751	0,554099561
1569339_s_at	LOC100287558	-1,06282519	-1,616924751	0,554099561
215420_at	IHH	-1,06282519	-1,616924751	0,554099561
1558930_at	LINC00460	-1,06282519	-1,616924751	0,554099561
223066_at	SNAPIN	4,389611304	3,83554191	0,554069394
217705_at	PRKD1	-1,982324124	-2,536339282	0,554015158
216795_at	-	-1,982324124	-2,536339282	0,554015158
243317_at	-	-1,982324124	-2,536339282	0,554015158
233688_at	KCNIP3	-1,982324124	-2,536339282	0,554015158
1560373_a_at	-	-1,982324124	-2,536339282	0,554015158
239827_at	RGCC	-1,982324124	-2,536339282	0,554015158
214685_at	NOP14-AS1	-1,982324124	-2,536339282	0,554015158
1556531_at	-	-1,982324124	-2,536339282	0,554015158
225728_at	SORBS2	-1,982324124	-2,536339282	0,554015158
1564937_at	-	-1,982324124	-2,536339282	0,554015158
1553450_s_at	LINC00304	-1,982324124	-2,536339282	0,554015158
222384_at	DKFZP564C196	-1,982324124	-2,536339282	0,554015158
240915_at	IGHV1-69	-1,982324124	-2,536339282	0,554015158
233026_s_at	PDZD2	-1,982324124	-2,536339282	0,554015158
233508_at	MAP3K13	-1,982324124	-2,536339282	0,554015158
242963_at	SGMS2	-1,982324124	-2,536339282	0,554015158
215517_at	PYGO1	-1,982324124	-2,536339282	0,554015158
201430_s_at	DPYSL3	-1,982324124	-2,536339282	0,554015158
226435_at	PAPLN	-1,982324124	-2,536339282	0,554015158
217918_at	DYNLRB1	5,906218976	5,352285605	0,553933371
204179_at	MB	1,65554524	1,101780212	0,553765028
222571_at	ST6GALNAC6	1,326463531	0,772793615	0,553669916
224375_at	-	1,20396005	0,65030602	0,55365403
203064_s_at	FOXK2	2,248155141	1,694547353	0,553607788
212120_at	RHOQ	2,552836808	1,999354538	0,55348227
1565484_x_at	EGFR	-0,180990326	-0,734355396	0,55336507
220765_s_at	LIMS2	-0,180990326	-0,734355396	0,55336507
1554962_a_at	FGFR4	-0,180990326	-0,734355396	0,55336507
203530_s_at	STX4	3,694479488	3,141266133	0,553213355
65884_at	MAN1B1	2,801080877	2,247886737	0,553194141
212399_s_at	VGLL4	3,296548503	2,743433212	0,55311529
238557_at	LOC100144603	0,900065578	0,346964736	0,553100842
219565_at	CYP20A1	1,476175815	0,923203038	0,552972777
200662_s_at	TOMM20	6,173768592	5,621070956	0,552697636
205971_s_at	CTRB1 /// CTRB2	-0,730013898	-1,282520722	0,552506824
236167_at	-	-0,730013898	-1,282520722	0,552506824

238374_at	H1FNT	-0,730013898	-1,282520722	0,552506824
225815_at	CPLX2	-0,730013898	-1,282520722	0,552506824
222549_at	CLDN1	-0,730013898	-1,282520722	0,552506824
222278_at	-	-0,730013898	-1,282520722	0,552506824
1554318_at	LOC541473	-0,730013898	-1,282520722	0,552506824
243371_at	-	-0,730013898	-1,282520722	0,552506824
1553584_at	CXorf36	-0,730013898	-1,282520722	0,552506824
206073_at	COLQ	-0,730013898	-1,282520722	0,552506824
222724_at	VWA1	-0,730013898	-1,282520722	0,552506824
207412_x_at	CELP	-0,730013898	-1,282520722	0,552506824
220827_at	-	-0,730013898	-1,282520722	0,552506824
1569991_at	KY	-0,730013898	-1,282520722	0,552506824
217695_x_at	-	-0,730013898	-1,282520722	0,552506824
232933_at	KIAA1656	-0,730013898	-1,282520722	0,552506824
220571_at	PRDM11	-0,730013898	-1,282520722	0,552506824
227885_at	LOC400236	-0,730013898	-1,282520722	0,552506824
221944_at	FLJ42627	-0,730013898	-1,282520722	0,552506824
237342_at	TOLLIP	-0,730013898	-1,282520722	0,552506824
213167_s_at	SLC5A3	-0,730013898	-1,282520722	0,552506824
213438_at	NFASC	-1,452713826	-2,005029581	0,552315755
1563913_at	-	-1,452713826	-2,005029581	0,552315755
215632_at	NEUROG2	-1,452713826	-2,005029581	0,552315755
244040_at	KCNN3	-1,452713826	-2,005029581	0,552315755
235602_at	TP53INP1	-1,452713826	-2,005029581	0,552315755
1561010_a_at	MAOB	-1,452713826	-2,005029581	0,552315755
239786_at	-	-1,452713826	-2,005029581	0,552315755
210889_s_at	FCGR2B	-1,452713826	-2,005029581	0,552315755
1567241_at	OR2L1P	-1,452713826	-2,005029581	0,552315755
234913_at	TTTY4 /// TTTY4B /	-1,452713826	-2,005029581	0,552315755
237790_at	SCN8A	-1,452713826	-2,005029581	0,552315755
234153_at	-	-1,452713826	-2,005029581	0,552315755
1564960_at	KRTAP7-1	-1,452713826	-2,005029581	0,552315755
240524_x_at	-	-1,452713826	-2,005029581	0,552315755
237368_at	C3orf35	-1,452713826	-2,005029581	0,552315755
242896_at	-	-1,452713826	-2,005029581	0,552315755
233787_at	C6orf163	-1,452713826	-2,005029581	0,552315755
1570409_x_at	-	-1,452713826	-2,005029581	0,552315755
211148_s_at	ANGPT2	-1,452713826	-2,005029581	0,552315755
206856_at	LILRB5	-1,452713826	-2,005029581	0,552315755
229166_s_at	GATAD2A	-1,452713826	-2,005029581	0,552315755
1555320_a_at	STAB1	-1,452713826	-2,005029581	0,552315755
241292_at	-	-1,452713826	-2,005029581	0,552315755
210770_s_at	CACNA1A	-1,452713826	-2,005029581	0,552315755
232317_at	PLXNA4	-1,452713826	-2,005029581	0,552315755
206275_s_at	MICAL2	-1,452713826	-2,005029581	0,552315755
205578_at	ROR2	-1,452713826	-2,005029581	0,552315755
222845_x_at	TMBIM4	5,728849214	5,176647348	0,552201866
214252_s_at	CLN5	2,019346222	1,46719264	0,552153582
225480_at	C1orf122	3,137734155	2,585619776	0,552114379
222302_at	LOC100507009	0,97663406	0,424693306	0,551940754

202138_x_at	AIMP2	4,911353661	4,359468791	0,55188487
217039_x_at	ELK2AP /// LOC10(	1,421391637	0,869824768	0,551566869
222125_s_at	P4HTM	3,086998005	2,535878988	0,551119017
204977_at	DDX10	2,983826368	2,432792627	0,551033741
236197_at	-	-1,212652659	-1,763664074	0,551011415
241107_at	-	-1,212652659	-1,763664074	0,551011415
224137_at	CACNG7	-1,212652659	-1,763664074	0,551011415
231444_at	LOC100506216	-1,212652659	-1,763664074	0,551011415
210462_at	BLZF1	-1,212652659	-1,763664074	0,551011415
215105_at	CG030	-1,212652659	-1,763664074	0,551011415
1568643_a_at	-	-1,212652659	-1,763664074	0,551011415
1552662_a_at	PCDHGB7	-1,212652659	-1,763664074	0,551011415
1561045_a_at	-	-1,212652659	-1,763664074	0,551011415
224166_at	SLC25A2	-1,212652659	-1,763664074	0,551011415
234534_at	-	-1,212652659	-1,763664074	0,551011415
242184_s_at	-	-1,212652659	-1,763664074	0,551011415
1560448_at	PRR7-AS1	-1,212652659	-1,763664074	0,551011415
1564241_at	ATP1A4	-1,212652659	-1,763664074	0,551011415
231707_at	-	-1,212652659	-1,763664074	0,551011415
211613_s_at	GPD2	-1,212652659	-1,763664074	0,551011415
1560935_s_at	LOC284669	-1,212652659	-1,763664074	0,551011415
216708_x_at	CKAP2	-1,212652659	-1,763664074	0,551011415
220454_s_at	SEMA6A	-1,212652659	-1,763664074	0,551011415
220346_at	MTHFD2L	-1,212652659	-1,763664074	0,551011415
213515_x_at	HBG1 /// HBG2 ///	-1,212652659	-1,763664074	0,551011415
224387_at	COMMD5	2,042564225	1,491644074	0,550920151
200005_at	EIF3D	6,607744993	6,056885584	0,550859409
241869_at	APOL6	0,319287178	-0,231045907	0,550333085
217960_s_at	TOMM22	3,939815341	3,389993505	0,549821836
234530_s_at	ZNRD1	-0,551284523	-1,100943374	0,549658851
1561460_at	-	-0,551284523	-1,100943374	0,549658851
234811_at	CENPN	-0,551284523	-1,100943374	0,549658851
235372_at	FCRLA	-0,551284523	-1,100943374	0,549658851
236903_at	-	-0,551284523	-1,100943374	0,549658851
205114_s_at	CCL3 /// CCL3L1 //	-0,551284523	-1,100943374	0,549658851
201561_s_at	CLSTN1	2,312738346	1,76396664	0,548771706
215952_s_at	OAZ1	7,580980985	7,032344819	0,548636167
225360_at	TRABD	2,229377375	1,680963921	0,548413454
215991_s_at	EMC1	-0,924000698	-1,471833228	0,54783253
212962_at	SYDE1	-0,924000698	-1,471833228	0,54783253
220594_at	OGT	-0,924000698	-1,471833228	0,54783253
1569523_a_at	-	-0,924000698	-1,471833228	0,54783253
1554385_a_at	PADI2	-0,924000698	-1,471833228	0,54783253
216159_s_at	-	-0,924000698	-1,471833228	0,54783253
237421_at	-	-0,924000698	-1,471833228	0,54783253
216927_at	MAU2	-0,924000698	-1,471833228	0,54783253
244864_at	LOC100505562	-0,924000698	-1,471833228	0,54783253
233525_s_at	LINC00475	-0,924000698	-1,471833228	0,54783253
243420_at	-	-0,924000698	-1,471833228	0,54783253
234425_at	-	-0,924000698	-1,471833228	0,54783253

206160_at	APOBEC2	-0,924000698	-1,471833228	0,54783253
237004_at	-	-0,924000698	-1,471833228	0,54783253
219647_at	POPDC2	-0,924000698	-1,471833228	0,54783253
217518_at	MYOF	-0,924000698	-1,471833228	0,54783253
202018_s_at	LTF	-0,924000698	-1,471833228	0,54783253
1562845_at	EP400NL	-0,924000698	-1,471833228	0,54783253
1562219_at	FLJ41649	-0,924000698	-1,471833228	0,54783253
208385_at	NR2E3	-0,924000698	-1,471833228	0,54783253
223943_s_at	-	-0,924000698	-1,471833228	0,54783253
229358_at	IHH	-0,924000698	-1,471833228	0,54783253
206903_at	EXOG	-0,924000698	-1,471833228	0,54783253
1568732_at	-	-1,802716385	-2,350444759	0,547728374
237921_at	-	-1,802716385	-2,350444759	0,547728374
238318_at	-	-1,802716385	-2,350444759	0,547728374
243163_at	-	-1,802716385	-2,350444759	0,547728374
237991_at	-	-1,802716385	-2,350444759	0,547728374
244820_at	-	-1,802716385	-2,350444759	0,547728374
214897_at	-	-1,802716385	-2,350444759	0,547728374
231142_at	-	-1,802716385	-2,350444759	0,547728374
204677_at	CDH5	-1,802716385	-2,350444759	0,547728374
230809_at	-	-1,802716385	-2,350444759	0,547728374
238361_s_at	-	-1,802716385	-2,350444759	0,547728374
237385_at	LOC100506126	-1,802716385	-2,350444759	0,547728374
234392_at	-	-1,802716385	-2,350444759	0,547728374
1564327_at	FLJ32790	-1,802716385	-2,350444759	0,547728374
237981_at	CMYA5	-1,802716385	-2,350444759	0,547728374
240874_at	-	-1,802716385	-2,350444759	0,547728374
240322_at	-	-1,802716385	-2,350444759	0,547728374
234018_s_at	SEL1L2	-1,802716385	-2,350444759	0,547728374
229923_at	ZDHHC21	-1,802716385	-2,350444759	0,547728374
206024_at	HPD	-1,802716385	-2,350444759	0,547728374
1560770_at	PABPC1	-1,802716385	-2,350444759	0,547728374
231586_at	SRG7	-1,802716385	-2,350444759	0,547728374
1552932_at	NLRP6	-1,802716385	-2,350444759	0,547728374
243795_s_at	LOC440900	-1,802716385	-2,350444759	0,547728374
1553196_a_at	FCRL3	-1,802716385	-2,350444759	0,547728374
230348_at	LATS2	-1,802716385	-2,350444759	0,547728374
231456_at	-	-1,802716385	-2,350444759	0,547728374
213946_s_at	OBSL1	-1,802716385	-2,350444759	0,547728374
212814_at	AHCYL2	2,276736537	1,72935563	0,547380907
230269_at	-	-0,384193355	-0,931539147	0,547345792
237495_at	MPP7	-0,384193355	-0,931539147	0,547345792
237355_at	-	-0,384193355	-0,931539147	0,547345792
1556766_at	ERICH1	-0,384193355	-0,931539147	0,547345792
238059_at	-	-0,384193355	-0,931539147	0,547345792
1561539_at	LOC100506368	-0,384193355	-0,931539147	0,547345792
221747_at	TNS1	-0,384193355	-0,931539147	0,547345792
208738_x_at	SUMO2	6,698014565	6,150734511	0,547280054
208745_at	ATP5L	4,862309887	4,315068752	0,547241135
201823_s_at	RNF14	2,613382867	2,066329863	0,547053004

224926_at	EXOC4	3,273854609	2,726850891	0,547003718
225108_at	AGPS	2,33082239	1,784182261	0,546640129
238475_at	ALG10B	1,578719607	1,032267596	0,54645201
222699_s_at	PLEKHF2	1,578719607	1,032267596	0,54645201
208881_x_at	IDI1	5,585166528	5,038980358	0,54618617
1555348_at	TFAP2E	0,127732831	-0,418248858	0,545981689
205400_at	WAS	0,127732831	-0,418248858	0,545981689
223316_at	CCDC3	0,127732831	-0,418248858	0,545981689
224750_at	RNF185	2,492739983	1,946903052	0,54583693
203117_s_at	PAN2	2,190140919	1,644429915	0,545711004
204837_at	MTMR9	2,896717728	2,351470274	0,545247454
226609_at	DCBLD1	1,287793882	0,742671819	0,545122063
224432_at	SH3GLB2	1,287793882	0,742671819	0,545122063
218395_at	ACTR6	4,803736169	4,258615677	0,545120492
1557457_at	-	-0,043925712	-0,589001171	0,545075459
1553893_at	CCDC105	-0,043925712	-0,589001171	0,545075459
1563867_at	LOC283194	-0,043925712	-0,589001171	0,545075459
234661_at	CCDC57	-0,043925712	-0,589001171	0,545075459
206901_at	C19orf57	-0,043925712	-0,589001171	0,545075459
213140_s_at	SS18L1	3,039689025	2,49476365	0,544925375
228583_at	LIN52	3,055618382	2,5107204	0,544897981
235474_at	-	2,321828846	1,777135134	0,544693712
218053_at	PRPF40A	4,949910672	4,405241507	0,544669165
227418_at	MSANTD4	3,945594088	3,401110525	0,544483563
204544_at	HPS5	3,876052392	3,332103994	0,543948398
1555866_a_at	HEXDC	1,802733148	1,259081178	0,543651969
206529_x_at	SLC26A4	-2,336678463	-2,880284116	0,543605652
208313_s_at	SF1	3,816747242	3,27333514	0,543412102
222540_s_at	RSF1	3,071179046	2,527778509	0,543400537
1560553_at	TIAF1	0,556428218	0,013190398	0,54323782
242994_at	-	0,556428218	0,013190398	0,54323782
218420_s_at	PROSER1	2,705643918	2,162541631	0,543102287
213457_at	MFHAS1	4,096676757	3,553756974	0,542919783
1552804_a_at	TIRAP	0,282358733	-0,260526297	0,54288503
239464_at	-	0,282358733	-0,260526297	0,54288503
235546_at	SPINT1	0,282358733	-0,260526297	0,54288503
218704_at	RNF43	2,042564225	1,500059916	0,542504308
209647_s_at	SOCS5	1,857989965	1,315897482	0,542092483
209850_s_at	CDC42EP2	0,425036312	-0,116767475	0,541803787
218688_at	DAK	0,764143511	0,222432814	0,541710697
234806_at	ORF1	-1,369772723	-1,911206569	0,541433847
1560148_at	-	-1,369772723	-1,911206569	0,541433847
1560797_s_at	LOC100507404	-1,369772723	-1,911206569	0,541433847
237932_at	-	-1,369772723	-1,911206569	0,541433847
1560455_at	LOC339166 /// WS	-1,369772723	-1,911206569	0,541433847
239305_at	-	-1,369772723	-1,911206569	0,541433847
240978_at	-	-1,369772723	-1,911206569	0,541433847
217233_at	-	-1,369772723	-1,911206569	0,541433847
1566656_a_at	LOC100507654	-1,369772723	-1,911206569	0,541433847
241810_at	LOC100131180	-1,369772723	-1,911206569	0,541433847

235829_at	CAHM	-1,369772723	-1,911206569	0,541433847
217112_at	PDGFB	-1,369772723	-1,911206569	0,541433847
236465_at	RNF175	-1,369772723	-1,911206569	0,541433847
1561688_at	-	-1,369772723	-1,911206569	0,541433847
219271_at	GALNT14	-1,369772723	-1,911206569	0,541433847
243344_at	-	-1,369772723	-1,911206569	0,541433847
1559144_x_at	LOC100130581	-1,369772723	-1,911206569	0,541433847
214417_s_at	FETUB	-1,369772723	-1,911206569	0,541433847
206886_x_at	GH1	-1,369772723	-1,911206569	0,541433847
1560118_at	-	-1,369772723	-1,911206569	0,541433847
231965_at	PCED1A	-1,369772723	-1,911206569	0,541433847
243914_at	-	-1,369772723	-1,911206569	0,541433847
215487_x_at	FAM182B	-1,369772723	-1,911206569	0,541433847
232040_at	LOC157860	-1,369772723	-1,911206569	0,541433847
1555049_at	TSPEAR	-1,369772723	-1,911206569	0,541433847
231559_at	LOC100506941	-1,369772723	-1,911206569	0,541433847
216880_at	RAD51B	-1,369772723	-1,911206569	0,541433847
231097_at	-	-1,369772723	-1,911206569	0,541433847
230641_at	LOC100505938	-1,369772723	-1,911206569	0,541433847
1558589_at	C21orf15	-1,369772723	-1,911206569	0,541433847
221047_s_at	MARK1	-1,369772723	-1,911206569	0,541433847
225237_s_at	MSI2	1,561816652	1,020514937	0,541301716
1556006_s_at	CSNK1A1	0,847529938	0,306454867	0,541075071
231883_at	FBXW8	-0,230432956	-0,771340337	0,540907381
231705_at	HRSP12	-0,230432956	-0,771340337	0,540907381
224132_at	-	-0,230432956	-0,771340337	0,540907381
235271_s_at	ZNF397	-0,230432956	-0,771340337	0,540907381
222644_s_at	GLT25D1	-0,230432956	-0,771340337	0,540907381
239941_at	-	-0,230432956	-0,771340337	0,540907381
228814_at	RBBP6	-0,230432956	-0,771340337	0,540907381
236257_at	CD2AP	-0,230432956	-0,771340337	0,540907381
224659_at	SEPN1	-0,230432956	-0,771340337	0,540907381
212296_at	PSMD14	5,817632533	5,277094857	0,540537676
213654_at	TAF5L	2,692288672	2,151985997	0,540302675
221604_s_at	PEX16	2,468978138	1,929092307	0,539885831
203269_at	NSMAF	4,741350888	4,201469562	0,539881326
209005_at	FBXL5	2,401508182	1,861868165	0,539640017
209600_s_at	ACOX1	2,649978302	2,110584371	0,539393932
235536_at	SNORD89	1,510970097	0,971607464	0,539362633
219040_at	CORO7	1,510970097	0,971607464	0,539362633
204189_at	RARG	1,000459215	0,461385738	0,539073477
215873_x_at	ABCC10	1,910557155	1,371634653	0,538922502
235434_at	-	2,21987062	1,680963921	0,538906699
239020_at	FUT10	0,648195588	0,109458907	0,538736681
219805_at	CXorf56	0,648195588	0,109458907	0,538736681
212163_at	KIDINS220	3,331722254	2,793634788	0,538087466
203419_at	MLL4	1,421391637	0,883374248	0,538017389
204778_x_at	HOXB7	2,374261973	1,836262316	0,537999657
225853_at	GNPNAT1	2,295108393	1,757155322	0,537953072
213080_x_at	RPL5 /// SNORD21	8,325665787	7,787770916	0,537894871

232420_x_at	LOC100289341	2,267141818	1,72935563	0,537786189
227814_at	WDR53	2,267141818	1,72935563	0,537786189
227787_s_at	MED30	4,552867541	4,015353114	0,537514427
203174_s_at	ARFRP1	1,20396005	0,666486137	0,537473913
236076_at	LOC257396	0,08619576	-0,45121326	0,537409019
243042_at	FAM73A	0,08619576	-0,45121326	0,537409019
208037_s_at	MADCAM1	0,08619576	-0,45121326	0,537409019
229917_at	AGAP2	0,08619576	-0,45121326	0,537409019
236066_at	-	0,08619576	-0,45121326	0,537409019
210849_s_at	VPS41	2,664625637	2,127337555	0,537288082
225339_at	SPAG9	2,664625637	2,127337555	0,537288082
53071_s_at	C17orf101	3,446502002	2,909293697	0,537208305
201102_s_at	PFKL	2,392346173	1,855279162	0,537067012
200097_s_at	HNRNPK /// MIR7-	5,339236189	4,802203076	0,537033113
213047_x_at	SET	6,42919573	5,892447193	0,536748536
212485_at	GPATCH8	2,552836808	2,016139502	0,536697306
215963_x_at	RPL3	8,134010603	7,597699176	0,536311427
239329_at	RAB21	1,671397663	1,135235995	0,536161668
1568558_x_at	-	-0,791294935	-1,32740143	0,536106495
1559551_at	-	-0,791294935	-1,32740143	0,536106495
217338_at	KRT19P2	-0,791294935	-1,32740143	0,536106495
203797_at	VSNL1	-0,791294935	-1,32740143	0,536106495
232590_at	-	-0,791294935	-1,32740143	0,536106495
240324_at	-	-0,791294935	-1,32740143	0,536106495
216890_at	-	-0,791294935	-1,32740143	0,536106495
217471_at	-	-0,791294935	-1,32740143	0,536106495
210583_at	POLDIP3	-0,791294935	-1,32740143	0,536106495
219856_at	C1orf116	-0,791294935	-1,32740143	0,536106495
1564785_at	C14orf183	-0,791294935	-1,32740143	0,536106495
220279_at	TRIM17	-0,791294935	-1,32740143	0,536106495
211695_x_at	MUC1	-0,791294935	-1,32740143	0,536106495
238023_at	-	-0,791294935	-1,32740143	0,536106495
1561413_at	-	-0,791294935	-1,32740143	0,536106495
236212_at	-	-0,791294935	-1,32740143	0,536106495
228052_x_at	TCF3	-0,791294935	-1,32740143	0,536106495
217079_at	-	-0,791294935	-1,32740143	0,536106495
234630_at	-	-0,791294935	-1,32740143	0,536106495
1554952_s_at	NLRP12	-0,791294935	-1,32740143	0,536106495
1555490_s_at	PDZD3	-0,791294935	-1,32740143	0,536106495
202977_s_at	CREBZF	-0,791294935	-1,32740143	0,536106495
229170_s_at	TTC18	-0,609040214	-1,145057014	0,5360168
234791_at	GSX1	-0,609040214	-1,145057014	0,5360168
216535_at	CADM3	-0,609040214	-1,145057014	0,5360168
242240_at	-	-0,609040214	-1,145057014	0,5360168
218554_s_at	ASH1L	-0,609040214	-1,145057014	0,5360168
1553216_at	ZNF41	-0,609040214	-1,145057014	0,5360168
206062_at	GUCA1A	-0,609040214	-1,145057014	0,5360168
240612_at	-	-0,609040214	-1,145057014	0,5360168
214793_at	DUSP7	-0,609040214	-1,145057014	0,5360168
237851_at	-	-0,609040214	-1,145057014	0,5360168

224018_s_at	SCD5	-0,609040214	-1,145057014	0,5360168
231020_at	ARX	-0,609040214	-1,145057014	0,5360168
78330_at	ZNF335	-0,035757978	-0,571746959	0,535988981
201829_at	NET1	3,156891301	2,621003244	0,535888057
1553366_s_at	ANKRD23 /// ANKI	0,39105295	-0,144765583	0,535818533
225428_s_at	DDX54	0,39105295	-0,144765583	0,535818533
205558_at	TRAF6	2,019346222	1,483698568	0,535647654
218056_at	BFAR	3,214269085	2,678678228	0,535590857
1555895_at	DNM2	0,524545436	-0,011001236	0,535546672
1553732_s_at	CEP89	0,524545436	-0,011001236	0,535546672
210005_at	GART	1,745502609	1,209973561	0,535529047
233951_at	-	-0,437449947	-0,972893339	0,535443391
204069_at	MEIS1	-0,437449947	-0,972893339	0,535443391
1565559_at	MAGIX	-0,437449947	-0,972893339	0,535443391
1558512_at	-	-0,437449947	-0,972893339	0,535443391
220256_s_at	OXCT2	-0,437449947	-0,972893339	0,535443391
1552498_at	ZSCAN20	-0,437449947	-0,972893339	0,535443391
227714_s_at	-	-0,437449947	-0,972893339	0,535443391
232517_s_at	PRIC285	-0,437449947	-0,972893339	0,535443391
218151_x_at	SLC52A2	2,908690075	2,373249966	0,535440109
1569933_at	-	0,735999505	0,20062106	0,535378445
223462_at	TMEM175	0,735999505	0,20062106	0,535378445
236867_at	-	0,735999505	0,20062106	0,535378445
206926_s_at	IL11	0,244681185	-0,290694975	0,535376161
221328_at	CLDN17	0,244681185	-0,290694975	0,535376161
235826_at	-	0,244681185	-0,290694975	0,535376161
241617_x_at	-	0,244681185	-0,290694975	0,535376161
220950_s_at	KANSL3	0,244681185	-0,290694975	0,535376161
237085_x_at	-	0,244681185	-0,290694975	0,535376161
209153_s_at	TCF3	4,550683059	4,015353114	0,535329944
1552615_at	ACACB	1,578719607	1,043469112	0,535250494
220885_s_at	CENPJ	1,578719607	1,043469112	0,535250494
212423_at	ZCCHC24	1,364555912	0,829309537	0,535246375
201197_at	AMD1	4,648252012	4,113313626	0,534938385
1561486_at	-	-1,893506789	-2,4284334	0,534926611
1570194_x_at	-	-1,893506789	-2,4284334	0,534926611
244403_at	CRB1	-1,893506789	-2,4284334	0,534926611
213197_at	ASTN1	-1,893506789	-2,4284334	0,534926611
208240_s_at	FGF1	-1,893506789	-2,4284334	0,534926611
242744_s_at	CASR	-1,893506789	-2,4284334	0,534926611
240657_at	-	-1,893506789	-2,4284334	0,534926611
237945_at	-	-1,893506789	-2,4284334	0,534926611
207981_s_at	ESRRG	-1,893506789	-2,4284334	0,534926611
236939_at	PTPLAD2	-1,893506789	-2,4284334	0,534926611
234233_s_at	-	-1,893506789	-2,4284334	0,534926611
243200_at	-	-1,893506789	-2,4284334	0,534926611
1556938_a_at	-	-1,893506789	-2,4284334	0,534926611
238632_at	LOC100505946	-1,893506789	-2,4284334	0,534926611
1557861_at	-	-1,893506789	-2,4284334	0,534926611
242509_at	-	-1,893506789	-2,4284334	0,534926611

1561237_at	-	-1,893506789	-2,4284334	0,534926611
1560225_at	CNR1	-1,893506789	-2,4284334	0,534926611
209655_s_at	TMEM47	-1,893506789	-2,4284334	0,534926611
216731_s_at	-	-1,893506789	-2,4284334	0,534926611
234336_s_at	TTLL9	-1,893506789	-2,4284334	0,534926611
222881_at	HPSE	-1,893506789	-2,4284334	0,534926611
206640_x_at	GAGE12B /// GAGI	-1,893506789	-2,4284334	0,534926611
238646_at	-	1,246401134	0,711934445	0,534466688
228778_at	MCPH1	1,307201325	0,772793615	0,534407711
222777_s_at	WHSC1	2,127965541	1,593605508	0,534360033
1556552_a_at	ACSF3	-0,088974936	-0,623254098	0,534279163
240310_at	-	-0,088974936	-0,623254098	0,534279163
1565731_at	ALDH3B1	-0,088974936	-0,623254098	0,534279163
220846_s_at	-	-0,088974936	-0,623254098	0,534279163
224579_at	SLC38A1	5,370186375	4,835912357	0,534274018
225904_at	CCSAP	4,801719103	4,267566192	0,534152911
225887_at	PROSER1	4,195854652	3,66182964	0,534025013
233528_s_at	GATSL3	0,820160788	0,286279868	0,53388092
200600_at	MSN	5,551081232	5,017311715	0,533769517
201443_s_at	ATP6AP2	5,277109976	4,743674982	0,533434994
212051_at	WIPF2	2,745538988	2,212304392	0,533234595
217478_s_at	HLA-DMA	4,043821727	3,510668356	0,533153371
212870_at	SOS2	1,92317941	1,390070295	0,533109115
235338_s_at	SETDB2	1,182925501	0,65030602	0,532619481
230804_at	NKAPL	-2,163157732	-2,695640852	0,53248312
211680_at	PDLIM5	-2,163157732	-2,695640852	0,53248312
207664_at	ADAM2	-2,163157732	-2,695640852	0,53248312
241077_at	-	-2,163157732	-2,695640852	0,53248312
240082_s_at	C17orf74	-2,163157732	-2,695640852	0,53248312
1564985_a_at	SLC8A1	-2,163157732	-2,695640852	0,53248312
1570316_at	-	-2,163157732	-2,695640852	0,53248312
212230_at	PPAP2B	-2,163157732	-2,695640852	0,53248312
203024_s_at	C5orf15	4,299310603	3,767104697	0,532205907
209709_s_at	HMMR	5,061011789	4,528833812	0,532177977
1559382_at	C19orf42	1,047341799	0,515457602	0,531884197
214487_s_at	RAP2A /// RAP2B	1,047341799	0,515457602	0,531884197
1554706_at	OR2L13	-2,416625063	-2,948507524	0,531882461
221727_at	SUB1	3,67542249	3,143844996	0,531577494
1555137_a_at	FGD6	-0,280273599	-0,811769547	0,531495948
231868_at	HOMEZ	-0,280273599	-0,811769547	0,531495948
1569470_a_at	FRMD5	-0,280273599	-0,811769547	0,531495948
225258_at	FBLIM1	-0,280273599	-0,811769547	0,531495948
201235_s_at	BTG2	-0,280273599	-0,811769547	0,531495948
203651_at	ZFYVE16	1,948628481	1,417157926	0,531470554
222229_x_at	-	7,197664206	6,666241176	0,531423031
229322_at	PPP2R5E	3,03433073	2,503049671	0,531281059
203126_at	IMPA2	2,295108393	1,76396664	0,531141754
218912_at	GCC1	2,321828846	1,790914523	0,530914323
218025_s_at	ECI2	4,693076946	4,162193397	0,530883549
223092_at	ANKH	5,161840645	4,631007841	0,530832804

202961_s_at	ATP5J2	6,456784072	5,92599188	0,530792192
226714_at	SAMD4B	1,287793882	0,757638486	0,530155396
230005_at	SVIP	2,872644433	2,34250725	0,530137182
205662_at	B9D1	0,045517965	-0,484558493	0,530076458
211918_x_at	PAPPA2	0,20740896	-0,322340048	0,529749008
1556296_at	-	0,20740896	-0,322340048	0,529749008
240469_at	-	-2,250580859	-2,780225148	0,529644289
1558121_at	CSRNP3	-2,250580859	-2,780225148	0,529644289
1567100_at	-	-2,250580859	-2,780225148	0,529644289
236489_at	GPR110	-2,250580859	-2,780225148	0,529644289
239140_at	-	-2,250580859	-2,780225148	0,529644289
1566846_at	-	-2,250580859	-2,780225148	0,529644289
1563179_at	-	-2,250580859	-2,780225148	0,529644289
1553383_at	ARHGAP42	-2,250580859	-2,780225148	0,529644289
1563107_at	-	-2,250580859	-2,780225148	0,529644289
224089_at	-	-2,250580859	-2,780225148	0,529644289
227156_at	CASK	-2,250580859	-2,780225148	0,529644289
207956_x_at	PDS5B	3,903941986	3,374450349	0,529491638
234134_at	-	-2,074223907	-2,603471267	0,52924736
234800_at	-	-2,074223907	-2,603471267	0,52924736
1560018_at	ARPP21	-2,074223907	-2,603471267	0,52924736
206157_at	PTX3	-2,074223907	-2,603471267	0,52924736
237150_at	-	-2,074223907	-2,603471267	0,52924736
1556921_at	-	-2,074223907	-2,603471267	0,52924736
204006_s_at	FCGR3A /// FCGR3	-2,074223907	-2,603471267	0,52924736
211155_s_at	THPO	-2,074223907	-2,603471267	0,52924736
212787_at	YLPM1	3,322661262	2,793634788	0,529026474
224702_at	TMEM167A	3,939815341	3,410857018	0,528958324
211795_s_at	FYB	3,060716358	2,531843788	0,528872571
216342_x_at	-	7,446945419	6,918245724	0,528699695
230101_at	CXCL2	-1,137208348	-1,665791776	0,528583428
1570033_at	WIPI2	-1,137208348	-1,665791776	0,528583428
204762_s_at	GNAO1	-1,137208348	-1,665791776	0,528583428
220832_at	TLR8	-1,137208348	-1,665791776	0,528583428
1569824_at	-	-1,137208348	-1,665791776	0,528583428
215762_at	-	-1,137208348	-1,665791776	0,528583428
216817_s_at	OR2H1	-1,137208348	-1,665791776	0,528583428
223924_at	TTC25	-1,137208348	-1,665791776	0,528583428
234642_at	-	-1,137208348	-1,665791776	0,528583428
226621_at	OSMR	-1,137208348	-1,665791776	0,528583428
208455_at	PVRL1	-1,137208348	-1,665791776	0,528583428
210991_s_at	RIMS3	-1,137208348	-1,665791776	0,528583428
208458_at	SCNN1D	-1,137208348	-1,665791776	0,528583428
207617_at	DDX3X	-1,137208348	-1,665791776	0,528583428
1556327_a_at	-	-1,137208348	-1,665791776	0,528583428
220331_at	CYP46A1	-1,137208348	-1,665791776	0,528583428
232120_at	-	-1,137208348	-1,665791776	0,528583428
232129_s_at	LZTS2	-1,137208348	-1,665791776	0,528583428
237986_at	-	-1,137208348	-1,665791776	0,528583428
1553385_at	LOC84931	-1,137208348	-1,665791776	0,528583428

210922_at	-	-1,137208348	-1,665791776	0,528583428
218506_x_at	GLYR1	3,137734155	2,609615156	0,528118998
223176_at	KCTD20	3,792209911	3,264254379	0,527955533
208664_s_at	TTC3 /// TTC3P1	1,225123479	0,697221375	0,527902104
202265_at	BMI1 /// COMMD3	5,062183897	4,534332507	0,527851389
211375_s_at	ILF3	3,723597116	3,195836661	0,527760455
231017_at	STK11	2,019346222	1,491644074	0,527702148
65086_at	YIPF2	1,216668779	0,68938925	0,527279529
230224_at	ZCCHC18	1,024284941	0,497424714	0,526860227
244129_at	-	0,873789652	0,346964736	0,526824916
212995_x_at	MZT2A /// MZT2B	6,167084521	5,640293154	0,526791366
236140_at	GCLM	2,642918178	2,116147513	0,526770666
227268_at	RNFT1	2,642918178	2,116147513	0,526770666
204031_s_at	PCBP2	6,797502927	6,270764185	0,526738742
207565_s_at	MR1	1,160996507	0,634301128	0,526695379
223061_at	CHID1	2,401508182	1,874839058	0,526669124
222593_s_at	SPATS2	3,233229368	2,707013102	0,526216266
207344_at	AKAP3	-0,135065865	-0,661178575	0,526112709
235554_x_at	PACRGL	-0,135065865	-0,661178575	0,526112709
209747_at	TGFB3	-0,135065865	-0,661178575	0,526112709
233860_s_at	OBP2A /// OBP2B	-0,135065865	-0,661178575	0,526112709
228666_at	C15orf38	-0,135065865	-0,661178575	0,526112709
214699_x_at	WIPI2	-0,135065865	-0,661178575	0,526112709
238331_at	SPRN	-0,135065865	-0,661178575	0,526112709
232727_at	UCKL1	-0,135065865	-0,661178575	0,526112709
232643_at	POLR2F	-0,135065865	-0,661178575	0,526112709
222352_at	-	0,58788177	0,061773582	0,526108188
214404_x_at	SPDEF	0,58788177	0,061773582	0,526108188
203142_s_at	AP3B1	3,161901883	2,635864822	0,526037061
221073_s_at	NOD1	1,094118704	0,568155442	0,525963262
212369_at	ZNF384	2,552836808	2,026957145	0,525879663
232473_at	-	-0,994186487	-1,520022123	0,525835636
214928_at	OBSL1	-0,994186487	-1,520022123	0,525835636
207552_at	ATP5G2	-0,994186487	-1,520022123	0,525835636
215365_at	CACNB2	-0,994186487	-1,520022123	0,525835636
201372_s_at	CUL3	-0,994186487	-1,520022123	0,525835636
219666_at	MS4A6A	-0,994186487	-1,520022123	0,525835636
214951_at	SLC26A10	-0,994186487	-1,520022123	0,525835636
215748_at	KLHL35	-0,994186487	-1,520022123	0,525835636
206773_at	LY6H	-0,994186487	-1,520022123	0,525835636
206114_at	EPHA4	-0,994186487	-1,520022123	0,525835636
224039_at	FAM186B	-0,994186487	-1,520022123	0,525835636
1557628_s_at	LOC283745	-0,994186487	-1,520022123	0,525835636
208568_at	MC2R	-0,994186487	-1,520022123	0,525835636
1553057_at	SERPINB12	-0,994186487	-1,520022123	0,525835636
227321_at	GATS	-0,994186487	-1,520022123	0,525835636
207985_at	-	-0,994186487	-1,520022123	0,525835636
220532_s_at	TMEM176B	-0,994186487	-1,520022123	0,525835636
216854_at	GDF11	-0,994186487	-1,520022123	0,525835636
200862_at	DHCR24	3,988952858	3,463284238	0,52566862

201533_at	CTNNB1	4,158437147	3,632850102	0,525587045
32502_at	GDPD5	1,687646107	1,162083318	0,525562788
200982_s_at	ANXA6	5,587966078	5,062440867	0,525525211
202579_x_at	HMGN4	6,102108167	5,577203867	0,524904299
218992_at	PLGRKT	3,442765386	2,918087241	0,524678145
202848_s_at	GRK6	2,118167848	1,593605508	0,52456234
227768_at	ZNF407	2,118167848	1,593605508	0,52456234
205282_at	LRP8	2,961244348	2,436714502	0,524529845
221911_at	ETV1	1,983901861	1,459545271	0,52435659
230006_s_at	SVIP	3,291641956	2,767331883	0,524310073
218549_s_at	FAM82B	4,221194173	3,697224187	0,523969986
218262_at	RMND5B	1,625482993	1,101780212	0,523702782
226428_at	TNPO2	1,625482993	1,101780212	0,523702782
203089_s_at	HTRA2	4,250892641	3,727191713	0,523700928
224304_x_at	NIN	2,007220798	1,483698568	0,52352223
200885_at	RHOC	4,671297742	4,148511394	0,522786349
221203_s_at	YEATS2	2,649978302	2,127337555	0,522640748
208315_x_at	TRAF3	2,085817135	1,563256447	0,522560688
242734_x_at	GALT	-0,49284695	-1,015398016	0,522551066
235480_at	LOC100506472	-0,49284695	-1,015398016	0,522551066
220548_at	PKDREJ	-0,49284695	-1,015398016	0,522551066
205350_at	CRABP1	-0,49284695	-1,015398016	0,522551066
217378_x_at	LOC100130100	-0,49284695	-1,015398016	0,522551066
206563_s_at	OPRL1	-0,49284695	-1,015398016	0,522551066
213706_at	GPD1	-0,49284695	-1,015398016	0,522551066
1557613_at	FLJ39534	-0,49284695	-1,015398016	0,522551066
207136_at	ARR3	-0,49284695	-1,015398016	0,522551066
237455_at	-	-0,49284695	-1,015398016	0,522551066
235585_at	-	0,167727503	-0,354781582	0,522509085
232756_at	KALRN	0,167727503	-0,354781582	0,522509085
1563853_at	LOC283045	0,167727503	-0,354781582	0,522509085
213321_at	BCKDHB	2,159837409	1,637482551	0,522354858
227312_at	SNTB2	0,458905032	-0,063377083	0,522282114
221928_at	ACACB	0,677745787	0,155747281	0,521998506
1561503_at	MYLK4	-1,289805289	-1,811780055	0,521974766
232713_at	-	-1,289805289	-1,811780055	0,521974766
236640_at	LOC100507165	-1,289805289	-1,811780055	0,521974766
232815_at	FBXO3	-1,289805289	-1,811780055	0,521974766
1553306_at	CASP8	-1,289805289	-1,811780055	0,521974766
1553325_at	POU5F2	-1,289805289	-1,811780055	0,521974766
1562659_at	LOC400548	-1,289805289	-1,811780055	0,521974766
215843_s_at	TLL2	-1,289805289	-1,811780055	0,521974766
241012_at	-	-1,289805289	-1,811780055	0,521974766
204942_s_at	ALDH3B2	-1,289805289	-1,811780055	0,521974766
1557025_a_at	-	-1,289805289	-1,811780055	0,521974766
230736_at	LOC387647	-1,289805289	-1,811780055	0,521974766
1556750_at	LOC153577	-1,289805289	-1,811780055	0,521974766
236434_at	PES1	-1,289805289	-1,811780055	0,521974766
234711_s_at	BCORL1	-1,289805289	-1,811780055	0,521974766
208048_at	TACR1	-1,289805289	-1,811780055	0,521974766

222081_at	SIRT5	-1,289805289	-1,811780055	0,521974766
216423_at	-	-1,289805289	-1,811780055	0,521974766
1553589_a_at	PDZK1IP1	-1,289805289	-1,811780055	0,521974766
234719_at	LAMA3	-1,289805289	-1,811780055	0,521974766
237377_at	-	-1,289805289	-1,811780055	0,521974766
230114_at	HOXB8	-1,289805289	-1,811780055	0,521974766
209775_x_at	SLC19A1	-1,289805289	-1,811780055	0,521974766
223904_at	PRKAG3	-1,289805289	-1,811780055	0,521974766
240794_at	NPAS4	-1,289805289	-1,811780055	0,521974766
236848_s_at	TEX13A	-1,289805289	-1,811780055	0,521974766
204595_s_at	STC1	-1,289805289	-1,811780055	0,521974766
1559753_at	-	-1,289805289	-1,811780055	0,521974766
229312_s_at	GKAP1	1,364555912	0,842599219	0,521956693
208078_s_at	SIK1	1,364555912	0,842599219	0,521956693
213699_s_at	YWHAQ	7,90515835	7,383343417	0,521814933
218573_at	MAGEH1	2,817838865	2,296310714	0,52152815
1552793_at	C8orf31	-0,66934516	-1,19085694	0,52151178
244269_at	-	-0,66934516	-1,19085694	0,52151178
228550_at	RTN4R	-0,66934516	-1,19085694	0,52151178
1562945_at	-	-0,66934516	-1,19085694	0,52151178
1554992_at	RASGRF1	-0,66934516	-1,19085694	0,52151178
1566182_at	-	-0,66934516	-1,19085694	0,52151178
1558848_at	LOC100653281	-0,66934516	-1,19085694	0,52151178
228808_s_at	LOXL2	-0,66934516	-1,19085694	0,52151178
211550_at	EGFR	-0,66934516	-1,19085694	0,52151178
220747_at	HSPC072	-0,66934516	-1,19085694	0,52151178
1568957_x_at	SRGAP2 /// SRGAP	-0,66934516	-1,19085694	0,52151178
1553030_a_at	SUOX	-0,66934516	-1,19085694	0,52151178
228292_at	-	1,071336699	0,550017041	0,521319658
221580_s_at	MIR1304 /// SNOR	5,615365095	5,094182557	0,521182538
37022_at	PRELP	-0,229045972	-0,750127882	0,52108191
232022_at	-	0,319287178	-0,201789721	0,521076899
1559326_at	DIO3OS	0,319287178	-0,201789721	0,521076899
202067_s_at	LDLR	1,307201325	0,786391549	0,520809776
234873_x_at	RPL7A /// SNORD2	7,707182638	7,186514471	0,520668166
229419_at	FBXW7	1,910557155	1,390070295	0,52048686
1559003_a_at	CCDC163P	1,788922638	1,268569037	0,520353601
229758_at	TIGD5	1,65554524	1,135235995	0,520309245
227873_at	TXNDC15	2,884644843	2,364676067	0,519968776
1569983_at	LINC00566	-2,336678463	-2,856570137	0,519891673
215355_at	POU2F3	-2,336678463	-2,856570137	0,519891673
229219_s_at	-	0,764143511	0,244646697	0,519496814
214909_s_at	DDAH2	0,764143511	0,244646697	0,519496814
218780_at	HOOK2	0,764143511	0,244646697	0,519496814
43934_at	GPR137	1,129296022	0,609815384	0,519480638
239866_at	-	0,001114523	-0,518232988	0,519347511
209438_at	PHKA2	0,001114523	-0,518232988	0,519347511
1552555_at	PRSS36	0,001114523	-0,518232988	0,519347511
232350_x_at	GPR161	0,001114523	-0,518232988	0,519347511
236152_at	PAGE5	0,001114523	-0,518232988	0,519347511

218448_at	C20orf11	3,688415399	3,169167796	0,519247602
227788_at	USP13	1,686984721	1,167919222	0,519065499
226727_at	CISD3	3,147261986	2,628204186	0,5190578
1555345_at	SLC38A4	-1,982324124	-2,501359113	0,51903499
1570596_at	-	-1,982324124	-2,501359113	0,51903499
1565653_at	HEATR7A	-1,982324124	-2,501359113	0,51903499
1569759_at	-	-1,982324124	-2,501359113	0,51903499
237476_at	-	-1,982324124	-2,501359113	0,51903499
241287_x_at	-	-1,982324124	-2,501359113	0,51903499
1561516_at	-	-1,982324124	-2,501359113	0,51903499
1553394_a_at	TFAP2B	-1,982324124	-2,501359113	0,51903499
1561478_at	LINC00560	-1,982324124	-2,501359113	0,51903499
234418_x_at	CD44	-1,982324124	-2,501359113	0,51903499
223753_s_at	CFC1 /// CFC1B	-1,982324124	-2,501359113	0,51903499
1567078_x_at	CLN6	-1,982324124	-2,501359113	0,51903499
234940_s_at	-	-1,982324124	-2,501359113	0,51903499
207561_s_at	ABCB8 /// ASIC3	-1,982324124	-2,501359113	0,51903499
211737_x_at	LOC100287705 ///	-1,982324124	-2,501359113	0,51903499
206475_x_at	CSH1	-1,982324124	-2,501359113	0,51903499
208548_at	IFNA6	-1,982324124	-2,501359113	0,51903499
204518_s_at	PPIC	-1,982324124	-2,501359113	0,51903499
38707_r_at	E2F4	2,594041613	2,07504239	0,518999223
221534_at	C11orf68	2,199941239	1,680963921	0,518977318
226692_at	C15orf63 /// MIR1	2,127965541	1,60918327	0,518782271
210819_x_at	DIO2	0,556428218	0,03784737	0,518580848
209778_at	TRIP11	0,556428218	0,03784737	0,518580848
224852_at	TTC17	2,590269697	2,071691486	0,518578211
1555832_s_at	KLF6	3,758020365	3,239448352	0,518572013
200942_s_at	HSBP1	4,801719103	4,283545167	0,518173935
204320_at	COL11A1	-1,624955693	-2,142286822	0,517331129
216636_at	-	-1,624955693	-2,142286822	0,517331129
1558660_at	LOC100507059	-1,624955693	-2,142286822	0,517331129
206013_s_at	ACTL6B	-1,624955693	-2,142286822	0,517331129
239174_at	LOC100505912	-1,624955693	-2,142286822	0,517331129
1559571_a_at	ATP13A4	-1,624955693	-2,142286822	0,517331129
235951_s_at	ZNF688	-1,624955693	-2,142286822	0,517331129
1562523_at	-	-1,624955693	-2,142286822	0,517331129
215332_s_at	CD8B	-1,624955693	-2,142286822	0,517331129
206713_at	NTNG1	-1,624955693	-2,142286822	0,517331129
242406_at	-	-1,624955693	-2,142286822	0,517331129
234270_at	-	-1,624955693	-2,142286822	0,517331129
233972_s_at	FEZF2	-1,624955693	-2,142286822	0,517331129
1569005_at	-	-1,624955693	-2,142286822	0,517331129
237297_at	FAM53B	-1,624955693	-2,142286822	0,517331129
240755_at	-	-1,624955693	-2,142286822	0,517331129
216797_at	-	-1,624955693	-2,142286822	0,517331129
1560358_at	-	-1,624955693	-2,142286822	0,517331129
1557776_at	-	-1,624955693	-2,142286822	0,517331129
233311_at	LOC145845	-1,624955693	-2,142286822	0,517331129
229241_at	LDHD	-1,624955693	-2,142286822	0,517331129

206447_at	CELA2A /// CELA2E	-1,624955693	-2,142286822	0,517331129
230773_at	ZNF385D	-1,624955693	-2,142286822	0,517331129
1553861_at	TCP11L2	-1,624955693	-2,142286822	0,517331129
223998_at	TTLL2	-1,624955693	-2,142286822	0,517331129
208607_s_at	SAA1 /// SAA2 ///	-1,624955693	-2,142286822	0,517331129
241948_at	-	-1,624955693	-2,142286822	0,517331129
1564444_at	LOC100130264	-1,624955693	-2,142286822	0,517331129
234855_at	ORF1	-1,624955693	-2,142286822	0,517331129
206779_s_at	ASMT	-1,624955693	-2,142286822	0,517331129
241166_at	-	-0,332405896	-0,84928999	0,516884094
207697_x_at	LILRB2	-0,332405896	-0,84928999	0,516884094
243430_at	SEZ6	-0,332405896	-0,84928999	0,516884094
222720_x_at	C1orf27	-0,332405896	-0,84928999	0,516884094
1552419_s_at	TTLL10	-0,332405896	-0,84928999	0,516884094
202298_at	NDUFA1	6,476257231	5,95959334	0,516663891
240631_at	GPR98	-1,712732543	-2,229233437	0,516500895
1555444_a_at	PPP1R12B	-1,712732543	-2,229233437	0,516500895
1561558_at	LOC100506939	-1,712732543	-2,229233437	0,516500895
1569064_at	C15orf62	-1,712732543	-2,229233437	0,516500895
1553199_at	DCAF4L2	-1,712732543	-2,229233437	0,516500895
205363_at	BBOX1	-1,712732543	-2,229233437	0,516500895
207155_at	TBX5	-1,712732543	-2,229233437	0,516500895
207262_at	APOF	-1,712732543	-2,229233437	0,516500895
1554004_a_at	RGNEF	-1,712732543	-2,229233437	0,516500895
1568638_a_at	IDO2	-1,712732543	-2,229233437	0,516500895
242309_at	-	-1,712732543	-2,229233437	0,516500895
1563064_at	-	-1,712732543	-2,229233437	0,516500895
238217_at	-	-1,712732543	-2,229233437	0,516500895
224339_s_at	ANGPTL1	-1,712732543	-2,229233437	0,516500895
232572_at	PCA3	-1,712732543	-2,229233437	0,516500895
1562701_at	-	-1,712732543	-2,229233437	0,516500895
216598_s_at	CCL2	-1,712732543	-2,229233437	0,516500895
1566140_at	HOPX	-1,712732543	-2,229233437	0,516500895
235319_at	LOC100216546	-1,712732543	-2,229233437	0,516500895
231344_at	-	-1,712732543	-2,229233437	0,516500895
220654_at	PPY2	-1,712732543	-2,229233437	0,516500895
1555624_a_at	SSH1	-1,712732543	-2,229233437	0,516500895
1555151_s_at	TDH	-1,712732543	-2,229233437	0,516500895
241070_at	-	-1,712732543	-2,229233437	0,516500895
239698_at	-	-1,712732543	-2,229233437	0,516500895
1558982_at	ANKRD20A12P	-1,712732543	-2,229233437	0,516500895
239653_at	-	-1,712732543	-2,229233437	0,516500895
205289_at	BMP2	-1,712732543	-2,229233437	0,516500895
217669_s_at	AKAP6	-1,712732543	-2,229233437	0,516500895
239211_at	LOC100506251	-0,856259172	-1,372734086	0,516474914
1556398_a_at	-	-0,856259172	-1,372734086	0,516474914
234914_at	ZNF7	-0,856259172	-1,372734086	0,516474914
228844_at	SLC13A5	-0,856259172	-1,372734086	0,516474914
234796_at	CABP7	-0,856259172	-1,372734086	0,516474914
1556281_at	-	-0,856259172	-1,372734086	0,516474914

215451_s_at	AFF1	-0,856259172	-1,372734086	0,516474914
232501_at	-	-0,856259172	-1,372734086	0,516474914
224543_at	SVEP1	-0,856259172	-1,372734086	0,516474914
1556382_a_at	NAA15	-0,856259172	-1,372734086	0,516474914
244683_at	-	-0,856259172	-1,372734086	0,516474914
214135_at	CLDN18	-0,856259172	-1,372734086	0,516474914
228358_at	SOX12	-0,856259172	-1,372734086	0,516474914
1554259_at	GPSM1	-0,856259172	-1,372734086	0,516474914
229032_at	WSCD2	-0,856259172	-1,372734086	0,516474914
240885_at	-	-0,856259172	-1,372734086	0,516474914
223632_s_at	-	-0,856259172	-1,372734086	0,516474914
241433_at	RCOR3	-0,180990326	-0,697182596	0,516192271
242373_at	-	-0,180990326	-0,697182596	0,516192271
211181_x_at	LOC100506403 ///	-0,180990326	-0,697182596	0,516192271
207890_s_at	MMP25	-0,180990326	-0,697182596	0,516192271
238572_at	STK16	1,116462765	0,600385088	0,516077677
210958_s_at	MAST4	1,116462765	0,600385088	0,516077677
202166_s_at	PPP1R2	5,100432184	4,58477881	0,515653373
205432_at	OVGP1	0,97663406	0,461385738	0,515248322
239366_at	-	0,648195588	0,133104519	0,515091069
226286_at	ELMOD3	0,648195588	0,133104519	0,515091069
1553263_at	USH1G	0,648195588	0,133104519	0,515091069
241394_at	-	1,287793882	0,772793615	0,515000268
201214_s_at	PPP1R7	4,49239618	3,977561363	0,514834817
207842_s_at	CASC3	3,533714224	3,019022994	0,51469123
229859_at	-	0,425036312	-0,089635713	0,514672025
244647_at	-	0,425036312	-0,089635713	0,514672025
1557203_at	PABPC1L2A /// PA	0,127732831	-0,386896102	0,514628933
217353_at	-	0,127732831	-0,386896102	0,514628933
1554880_at	DKFZP434K028	0,127732831	-0,386896102	0,514628933
1560136_at	ARL5C	0,127732831	-0,386896102	0,514628933
235896_s_at	SMCR7	1,047341799	0,533258442	0,514083357
227116_at	MON1B	4,301645306	3,787622346	0,51402296
235059_at	RAB12	0,820160788	0,306454867	0,513705921
218997_at	POLR1E	2,937809196	2,424202743	0,513606453
223174_at	BTBD10	3,541158018	3,027559376	0,513598641
227815_at	-	2,75889404	2,245685006	0,513209033
230407_at	SBNO1	2,62869474	2,116147513	0,512547227
200083_at	USP22	4,626492955	4,114266588	0,512226368
213293_s_at	TRIM22	2,545254728	2,03313004	0,512124688
1553736_at	ZFC3H1	-1,537851782	-2,049747196	0,511895414
239929_at	PM20D1	-1,537851782	-2,049747196	0,511895414
212797_at	SORT1	-1,537851782	-2,049747196	0,511895414
1565557_at	-	-1,537851782	-2,049747196	0,511895414
237219_at	PHKA1-AS1	-1,537851782	-2,049747196	0,511895414
208570_at	WNT1	-1,537851782	-2,049747196	0,511895414
204517_at	PPIC	-1,537851782	-2,049747196	0,511895414
231515_at	-	-1,537851782	-2,049747196	0,511895414
211188_at	CD84	-1,537851782	-2,049747196	0,511895414
206155_at	ABCC2	-1,537851782	-2,049747196	0,511895414

206969_at	KRT34 /// LOC100(	-1,537851782	-2,049747196	0,511895414
235774_at	LOC100422737	-1,537851782	-2,049747196	0,511895414
1569316_at	TRIM24	-1,537851782	-2,049747196	0,511895414
206203_at	RCVRN	-1,537851782	-2,049747196	0,511895414
229403_at	B4GALT1	-1,537851782	-2,049747196	0,511895414
203889_at	SCG5	-1,537851782	-2,049747196	0,511895414
220362_at	PSORS1C1	-1,537851782	-2,049747196	0,511895414
211722_s_at	HDAC6	-1,537851782	-2,049747196	0,511895414
244355_at	AVL9	-1,537851782	-2,049747196	0,511895414
223895_s_at	EPN3	-1,537851782	-2,049747196	0,511895414
1559877_at	LOC100289094	-1,537851782	-2,049747196	0,511895414
240543_at	-	-1,537851782	-2,049747196	0,511895414
1568635_at	-	-1,537851782	-2,049747196	0,511895414
240507_at	-	-1,537851782	-2,049747196	0,511895414
236844_at	DHX30	-1,537851782	-2,049747196	0,511895414
207978_s_at	NR4A3	-1,537851782	-2,049747196	0,511895414
200751_s_at	HNRNPC /// LOC1(	5,758208547	5,246483322	0,511725224
1560288_at	-	-2,416625063	-2,928183529	0,511558466
225644_at	CCDC117	2,884644843	2,373249966	0,511394877
1566471_at	-	0,524545436	0,013190398	0,511355038
203839_s_at	TNK2	1,326463531	0,815200271	0,51126326
201817_at	UBE3C	3,410696467	2,899658146	0,511038321
222273_at	PAPOLG	2,321828846	1,811004388	0,510824458
240023_at	-	-0,043925712	-0,554696666	0,510770954
220050_at	C9orf9	1,160996507	0,65030602	0,510690487
202424_at	MAP2K2	3,461795772	2,951484533	0,510311239
212293_at	HIPK1	4,153515595	3,643334026	0,51018157
211561_x_at	MAPK14	2,267141818	1,757155322	0,509986497
217733_s_at	TMSB10	8,527698034	8,017799203	0,509898831
211505_s_at	STAU1	3,107338728	2,597466271	0,509872457
47773_at	FBXO42	1,183225281	0,673496747	0,509728534
213508_at	SPTSSA	2,692288672	2,182711118	0,509577554
235035_at	SLC35E1	1,267146969	0,757638486	0,509508483
202622_s_at	ATXN2	2,2388565	1,72935563	0,50950087
1556677_at	-	-1,802716385	-2,312054429	0,509338044
1562512_at	SMPDL3A	-1,802716385	-2,312054429	0,509338044
1558571_at	LOC100505963	-1,802716385	-2,312054429	0,509338044
1561776_at	-	-1,802716385	-2,312054429	0,509338044
1569230_at	-	-1,802716385	-2,312054429	0,509338044
216255_s_at	GRM8	-1,802716385	-2,312054429	0,509338044
219476_at	C1orf116	-1,802716385	-2,312054429	0,509338044
1570043_at	-	-1,802716385	-2,312054429	0,509338044
237193_s_at	-	-1,802716385	-2,312054429	0,509338044
216021_s_at	GLRA3	-1,802716385	-2,312054429	0,509338044
216134_at	FRMD4B	-1,802716385	-2,312054429	0,509338044
207271_x_at	SPAG11B	-1,802716385	-2,312054429	0,509338044
207913_at	CYP2F1	-1,802716385	-2,312054429	0,509338044
233527_at	LOC100129069	-1,802716385	-2,312054429	0,509338044
201496_x_at	MYH11	-1,802716385	-2,312054429	0,509338044
1563319_at	-	-1,802716385	-2,312054429	0,509338044

1560909_x_at	-	-1,802716385	-2,312054429	0,509338044
237697_at	LOC100506403 ///	-1,802716385	-2,312054429	0,509338044
206476_s_at	NOVA2	-1,802716385	-2,312054429	0,509338044
234745_at	-	-1,802716385	-2,312054429	0,509338044
220287_at	ADAMTS9	-1,802716385	-2,312054429	0,509338044
1560582_a_at	-	-1,802716385	-2,312054429	0,509338044
1560410_at	-	-1,802716385	-2,312054429	0,509338044
1560230_at	-	-1,802716385	-2,312054429	0,509338044
231733_at	CARD18	-1,802716385	-2,312054429	0,509338044
232777_s_at	C6orf118	-1,802716385	-2,312054429	0,509338044
233871_at	-	-1,802716385	-2,312054429	0,509338044
232837_at	KIF13A	-1,802716385	-2,312054429	0,509338044
1562600_at	-	-1,802716385	-2,312054429	0,509338044
214357_at	C1orf105	-1,802716385	-2,312054429	0,509338044
216355_at	PCDHB17	-1,802716385	-2,312054429	0,509338044
1569315_s_at	LOC100272228	-1,802716385	-2,312054429	0,509338044
1557031_at	RNF212	-1,802716385	-2,312054429	0,509338044
1563341_at	-	-1,802716385	-2,312054429	0,509338044
208471_at	HPR	-1,802716385	-2,312054429	0,509338044
205893_at	NLGN1	-1,802716385	-2,312054429	0,509338044
203166_at	CFDP1	3,060716358	2,551398837	0,509317522
213018_at	GATAD1	1,960819106	1,451507543	0,509311564
219238_at	PIGV	3,19484646	2,686001625	0,508844835
232702_at	RABGAP1L	1,024284941	0,515457602	0,508827339
240233_at	LOC100506714	0,618277321	0,109458907	0,508818415
1557712_x_at	-	0,618277321	0,109458907	0,508818415
209444_at	RAP1GDS1	4,288236521	3,779479375	0,508757145
223304_at	SLC37A3	2,285848435	1,777135134	0,508713302
202121_s_at	CHMP2A	4,829437568	4,321202891	0,508234677
201007_at	HADHB	5,709175578	5,201078294	0,508097284
224969_at	ATXN7L3	1,898032047	1,390070295	0,507961752
217809_at	BZW2	5,661294005	5,153407556	0,507886449
1560939_at	PCOLCE-AS1	0,39105295	-0,116767475	0,507820425
205657_at	HAAO	0,39105295	-0,116767475	0,507820425
238973_s_at	TSNAX	0,39105295	-0,116767475	0,507820425
213632_at	DHODH	0,873789652	0,366031214	0,507758438
228263_at	GRASP	0,707657549	0,20062106	0,507036489
204572_s_at	PIN4	1,20396005	0,697221375	0,506738675
52164_at	C11orf24	2,468838846	1,962348736	0,50649011
240333_at	-	-0,551284523	-1,057301851	0,506017328
206673_at	GPR176	-0,551284523	-1,057301851	0,506017328
232089_at	FLJ39639 /// ZNF2	-0,551284523	-1,057301851	0,506017328
213882_at	TM2D1	-0,551284523	-1,057301851	0,506017328
1566923_at	-	-0,551284523	-1,057301851	0,506017328
236713_at	-	-0,551284523	-1,057301851	0,506017328
236986_at	LINC00582	-0,551284523	-1,057301851	0,506017328
1555704_at	CMTM3	-0,551284523	-1,057301851	0,506017328
205487_s_at	VGLL1	-0,551284523	-1,057301851	0,506017328
206738_at	APOC2 /// APOC4 ,	-0,551284523	-1,057301851	0,506017328
204623_at	TFF3	-0,551284523	-1,057301851	0,506017328

229795_at	-	-0,551284523	-1,057301851	0,506017328
1563222_at	TMBIM4	-0,551284523	-1,057301851	0,506017328
221407_at	GJD2	-0,551284523	-1,057301851	0,506017328
228947_x_at	TMEM204	-0,551284523	-1,057301851	0,506017328
217801_at	ATP5E	6,800124054	6,294327586	0,505796468
1557232_at	-	0,791908897	0,286279868	0,50562903
1557047_at	YEATS2	0,791908897	0,286279868	0,50562903
207224_s_at	SIGLEC7	-1,06282519	-1,568385657	0,505560467
1553169_at	LRRN4	-1,06282519	-1,568385657	0,505560467
1555968_a_at	-	-1,06282519	-1,568385657	0,505560467
1566735_at	-	-1,06282519	-1,568385657	0,505560467
243235_at	-	-1,06282519	-1,568385657	0,505560467
236993_at	-	-1,06282519	-1,568385657	0,505560467
214923_at	ATP6V1D	-1,06282519	-1,568385657	0,505560467
216710_x_at	ZNF287	-1,06282519	-1,568385657	0,505560467
1556823_s_at	-	-1,06282519	-1,568385657	0,505560467
1557658_at	-	-1,06282519	-1,568385657	0,505560467
1567060_at	OR8G1	-1,06282519	-1,568385657	0,505560467
220249_at	HYAL4	-1,06282519	-1,568385657	0,505560467
1555309_a_at	CARD14	-1,06282519	-1,568385657	0,505560467
1558193_at	-	-1,06282519	-1,568385657	0,505560467
1560507_at	AWAT1	-1,06282519	-1,568385657	0,505560467
214454_at	ADAMTS2	-1,06282519	-1,568385657	0,505560467
202737_s_at	LSM4	4,57799322	4,07248815	0,50550507
201772_at	AZIN1	4,42686741	3,921474513	0,505392897
214808_at	-	1,857989965	1,352622437	0,505367528
208374_s_at	CAPZA1	6,732041862	6,226713971	0,50532789
212323_s_at	VPS13D	1,97249974	1,46719264	0,5053071
201698_s_at	GATC /// SRSF9	4,985871078	4,480622042	0,505249036
220308_at	CCDC19	0,244681185	-0,260526297	0,505207482
232374_s_at	-	0,244681185	-0,260526297	0,505207482
220583_at	-	0,244681185	-0,260526297	0,505207482
239059_at	DNAH1	-0,384193355	-0,889222211	0,505028856
242368_at	-	-0,384193355	-0,889222211	0,505028856
234498_at	C2orf65	-0,384193355	-0,889222211	0,505028856
234048_s_at	EPG5	-0,384193355	-0,889222211	0,505028856
207853_s_at	SNCB	-0,384193355	-0,889222211	0,505028856
215705_at	PPP5C	-0,384193355	-0,889222211	0,505028856
210480_s_at	MYO6	-0,384193355	-0,889222211	0,505028856
214529_at	TSHB	-0,730013898	-1,234835326	0,504821428
236531_at	-	-0,730013898	-1,234835326	0,504821428
228668_x_at	CCDC71L	-0,730013898	-1,234835326	0,504821428
227190_at	TMEM37	-0,730013898	-1,234835326	0,504821428
211553_x_at	APAF1	-0,730013898	-1,234835326	0,504821428
242045_at	-	-0,730013898	-1,234835326	0,504821428
213319_s_at	CSDA	-0,730013898	-1,234835326	0,504821428
206880_at	P2RX6	-0,730013898	-1,234835326	0,504821428
224171_at	LSM14B	-0,730013898	-1,234835326	0,504821428
1552432_at	MFSD6L	-0,730013898	-1,234835326	0,504821428
233384_at	-	-0,730013898	-1,234835326	0,504821428

229411_at	PNCK	-0,730013898	-1,234835326	0,504821428
241769_at	-	-0,730013898	-1,234835326	0,504821428
211048_s_at	PDIA4	3,729417893	3,224658402	0,504759491
1569631_at	NMNAT1	1,139057614	0,634301128	0,504756486
210692_s_at	SLC43A3	2,537731447	2,03313004	0,504601408
210775_x_at	CASP9	1,476175815	0,971607464	0,504568351
201641_at	BST2	3,434684504	2,930238085	0,504446419
1557679_at	ERICH1-AS1	0,08619576	-0,418248858	0,504444618
216488_s_at	ATP11A	0,08619576	-0,418248858	0,504444618
204952_at	LYPD3	0,08619576	-0,418248858	0,504444618
227070_at	GLT8D2	-1,452713826	-1,956681069	0,503967244
1557354_at	SOS1	-1,452713826	-1,956681069	0,503967244
207328_at	ALOX15	-1,452713826	-1,956681069	0,503967244
1558577_at	LOC148709	-1,452713826	-1,956681069	0,503967244
205764_at	CSNK1A1	-1,452713826	-1,956681069	0,503967244
1561556_at	-	-1,452713826	-1,956681069	0,503967244
215192_at	PMS2P4	-1,452713826	-1,956681069	0,503967244
239945_at	-	-1,452713826	-1,956681069	0,503967244
237769_at	-	-1,452713826	-1,956681069	0,503967244
1555990_at	C22orf42	-1,452713826	-1,956681069	0,503967244
226632_at	CYGB	-1,452713826	-1,956681069	0,503967244
1560091_a_at	LOC100506695 ///	-1,452713826	-1,956681069	0,503967244
244540_at	-	-1,452713826	-1,956681069	0,503967244
1559979_at	SYF2	-1,452713826	-1,956681069	0,503967244
239182_at	HOXD-AS1	-1,452713826	-1,956681069	0,503967244
206494_s_at	ITGA2B	-1,452713826	-1,956681069	0,503967244
216829_at	IGK@ /// IGKC ///	-1,452713826	-1,956681069	0,503967244
215838_at	LILRA5	-1,452713826	-1,956681069	0,503967244
240534_at	-	-1,452713826	-1,956681069	0,503967244
243775_at	-	-1,452713826	-1,956681069	0,503967244
238198_at	-	-1,452713826	-1,956681069	0,503967244
1568869_at	-	-1,452713826	-1,956681069	0,503967244
224527_at	CDH23 /// LOC100	-1,452713826	-1,956681069	0,503967244
244812_at	-	-1,452713826	-1,956681069	0,503967244
238307_at	-	-1,452713826	-1,956681069	0,503967244
242823_at	ADAMTS18	-1,452713826	-1,956681069	0,503967244
233998_x_at	-	-1,452713826	-1,956681069	0,503967244
1569763_at	-	-1,452713826	-1,956681069	0,503967244
210068_s_at	AQP4	-1,452713826	-1,956681069	0,503967244
1553064_at	H1FOO	-1,452713826	-1,956681069	0,503967244
232686_at	SIGLEC17P	-1,452713826	-1,956681069	0,503967244
218980_at	FHOD3	-1,452713826	-1,956681069	0,503967244
217088_s_at	NCR1	-0,230432956	-0,734355396	0,50392244
226047_at	MRVI1	-0,230432956	-0,734355396	0,50392244
241004_at	-	-0,230432956	-0,734355396	0,50392244
209696_at	FBP1	-0,230432956	-0,734355396	0,50392244
232988_at	KIAA0182	-0,230432956	-0,734355396	0,50392244
215607_x_at	-	-0,230432956	-0,734355396	0,50392244
202692_s_at	UBTF	1,5944803	1,090640407	0,503839893
1554442_at	BEST1	1,246401134	0,742671819	0,503729314

201611_s_at	ICMT	3,442765386	2,939187671	0,503577715
212199_at	MRFAP1L1	4,368395955	3,865466305	0,502929651
231785_at	NTF4	-1,212652659	-1,715576125	0,502923466
238385_at	C6orf58	-1,212652659	-1,715576125	0,502923466
1553711_a_at	FAM218A	-1,212652659	-1,715576125	0,502923466
1561561_x_at	-	-1,212652659	-1,715576125	0,502923466
208601_s_at	TUBB1	-1,212652659	-1,715576125	0,502923466
1555445_at	CA8	-1,212652659	-1,715576125	0,502923466
230216_at	C12orf51	-1,212652659	-1,715576125	0,502923466
215118_s_at	IGHA1	-1,212652659	-1,715576125	0,502923466
1561668_at	-	-1,212652659	-1,715576125	0,502923466
239132_at	NOS1	-1,212652659	-1,715576125	0,502923466
216955_at	TAF1	-1,212652659	-1,715576125	0,502923466
1553847_a_at	SPERT	-1,212652659	-1,715576125	0,502923466
229290_at	DAPL1	-1,212652659	-1,715576125	0,502923466
229386_at	ID4	-1,212652659	-1,715576125	0,502923466
206843_at	CRYBA4	-1,212652659	-1,715576125	0,502923466
243580_at	GNA14	-1,212652659	-1,715576125	0,502923466
216617_s_at	MAG	-1,212652659	-1,715576125	0,502923466
229626_at	C12orf68	-1,212652659	-1,715576125	0,502923466
223634_at	RASD2	-1,212652659	-1,715576125	0,502923466
205736_at	PGAM2	-1,212652659	-1,715576125	0,502923466
209983_s_at	NRXN2	-1,212652659	-1,715576125	0,502923466
243107_at	-	-1,212652659	-1,715576125	0,502923466
223779_at	AFAP1-AS1	-1,212652659	-1,715576125	0,502923466
223727_at	KCNIP2	0,491906512	-0,011001236	0,502907747
200080_s_at	H3F3A /// H3F3AP	7,637746004	7,134862821	0,502883183
200775_s_at	HNRNPK /// MIR7-	6,726693218	6,22412579	0,502567427
225963_at	KLHDC5	2,190140919	1,687645398	0,502495521
243501_at	ATP5F1	0,58788177	0,085534992	0,502346778
230637_at	SFXN4	0,58788177	0,085534992	0,502346778
211962_s_at	ZFP36L1	1,910557155	1,408220608	0,502336547
214765_s_at	NAAA	1,701542258	1,199296812	0,502245445
1554133_at	RUFY2	-2,163157732	-2,665156778	0,501999046
241083_at	-	-2,163157732	-2,665156778	0,501999046
1564807_at	-	-2,163157732	-2,665156778	0,501999046
240972_at	-	-2,163157732	-2,665156778	0,501999046
234453_s_at	C14orf166B	-2,163157732	-2,665156778	0,501999046
217078_s_at	CD300A	-2,163157732	-2,665156778	0,501999046
213591_at	ALDH7A1	-2,163157732	-2,665156778	0,501999046
234789_at	-	-2,163157732	-2,665156778	0,501999046
1563362_at	D21S2090E	-2,163157732	-2,665156778	0,501999046
225534_at	C8orf40	5,475251296	4,973363861	0,501887434
1438_at	EPHB3	-0,492240857	-0,993989149	0,501748292
227656_at	C6orf70	2,41945542	1,917743773	0,501711646
213396_s_at	AKAP10	1,936380191	1,434743608	0,501636583
219333_s_at	CAPN10	0,926325262	0,424693306	0,501631956
1568704_a_at	CHERP	1,625482993	1,123905343	0,501577651
1560543_at	GRK4	-2,250580859	-2,752097204	0,501516345
243936_x_at	-	-2,250580859	-2,752097204	0,501516345

236870_at	IQCF4	-2,250580859	-2,752097204	0,501516345
240970_x_at	-	-2,250580859	-2,752097204	0,501516345
206254_at	EGF	-2,250580859	-2,752097204	0,501516345
221054_s_at	TCL6	-2,250580859	-2,752097204	0,501516345
230910_s_at	LOC100288181	-2,250580859	-2,752097204	0,501516345
218626_at	EIF4ENIF1	2,829929672	2,328492509	0,501437163
240175_at	-	1,287793882	0,786391549	0,501402333
208286_x_at	POU5F1 /// POU5F	1,182925501	0,681874787	0,501050714
1556854_at	LOC283501	0,355751005	-0,144765583	0,500516588
230756_at	ZNF683	0,355751005	-0,144765583	0,500516588
213626_at	CBR4	3,071179046	2,570802332	0,500376715
209158_s_at	CYTH2	2,765070895	2,264929589	0,500141306
202698_x_at	COX4I1	7,421069736	6,920946373	0,500123363
1569200_at	-	1,578719607	1,078619386	0,500100221
224378_x_at	MAP1LC3A	1,383446753	0,883374248	0,500072504
216509_x_at	MLLT10	-0,088974936	-0,589001171	0,500026236
243389_at	PRH1 /// PRH1-PRI	-0,088974936	-0,589001171	0,500026236
216183_at	TGM2	-0,088974936	-0,589001171	0,500026236
208439_s_at	FCN2	-0,088974936	-0,589001171	0,500026236
229959_at	CYP4V2	-0,088974936	-0,589001171	0,500026236
204936_at	MAP4K2	-0,088974936	-0,589001171	0,500026236
212965_at	HIC2	-0,088974936	-0,589001171	0,500026236
1559167_x_at	MPV17L	1,871521385	1,371634653	0,499886732
223079_s_at	GLS	1,871521385	1,371634653	0,499886732
203373_at	SOCS2	2,452674045	1,953032682	0,499641363
200759_x_at	NFE2L1	3,768156719	3,26868404	0,499472679
222707_s_at	ACTR8	2,884644843	2,385765892	0,49887895
226309_at	DNAL1	0,764143511	0,265475485	0,498668026
214202_at	-	0,764143511	0,265475485	0,498668026
212727_at	DLG3	0,764143511	0,265475485	0,498668026
219910_at	FICD	1,116462765	0,61779983	0,498662935
203671_at	TPMT	1,116462765	0,61779983	0,498662935
207909_x_at	DAZ1 /// DAZ2 ///	-1,893506789	-2,391863529	0,49835674
240101_at	-	-1,893506789	-2,391863529	0,49835674
1558373_s_at	-	-1,893506789	-2,391863529	0,49835674
216786_at	FAM224A	-1,893506789	-2,391863529	0,49835674
243571_at	-	-1,893506789	-2,391863529	0,49835674
1561205_at	-	-1,893506789	-2,391863529	0,49835674
240330_at	-	-1,893506789	-2,391863529	0,49835674
224426_s_at	ACTR3BP2 /// ACT	-1,893506789	-2,391863529	0,49835674
220318_at	EPN3	-1,893506789	-2,391863529	0,49835674
1567374_at	-	-1,893506789	-2,391863529	0,49835674
1557277_a_at	LOC100507584	-1,893506789	-2,391863529	0,49835674
231334_at	-	-1,893506789	-2,391863529	0,49835674
237550_at	-	-1,893506789	-2,391863529	0,49835674
1560048_at	-	-1,893506789	-2,391863529	0,49835674
216660_at	MYO7B	-1,893506789	-2,391863529	0,49835674
1555224_at	CCDC148-AS1	-1,893506789	-2,391863529	0,49835674
1566873_at	-	-1,893506789	-2,391863529	0,49835674
240973_s_at	-	-1,893506789	-2,391863529	0,49835674

215320_at	FAM75C2	-1,893506789	-2,391863529	0,49835674
231981_at	PRLR	-1,893506789	-2,391863529	0,49835674
233668_at	-	-1,893506789	-2,391863529	0,49835674
242667_at	-	-1,893506789	-2,391863529	0,49835674
207635_s_at	KCNH1	-1,893506789	-2,391863529	0,49835674
238718_at	-	-1,893506789	-2,391863529	0,49835674
205044_at	GABRP	-1,893506789	-2,391863529	0,49835674
1556487_a_at	C3orf15 /// LOC10	-1,893506789	-2,391863529	0,49835674
243415_at	-	-1,893506789	-2,391863529	0,49835674
1553879_a_at	GOT1L1	-1,893506789	-2,391863529	0,49835674
228718_at	ZNF44	-1,893506789	-2,391863529	0,49835674
232818_at	-	-1,893506789	-2,391863529	0,49835674
211832_s_at	MDM2	-1,893506789	-2,391863529	0,49835674
237141_x_at	-	-1,893506789	-2,391863529	0,49835674
200871_s_at	PSAP	4,866513045	4,368188721	0,498324324
1558183_at	ZNF17	-0,924000698	-1,422257026	0,498256329
231945_at	FILIP1	-0,924000698	-1,422257026	0,498256329
1570511_at	ARHGEF10L	-0,924000698	-1,422257026	0,498256329
239803_at	-	-0,924000698	-1,422257026	0,498256329
211896_s_at	DCN	-0,924000698	-1,422257026	0,498256329
225008_at	ASPH	-0,924000698	-1,422257026	0,498256329
230523_at	QSOX1	-0,924000698	-1,422257026	0,498256329
1557279_at	ITSN1	-0,924000698	-1,422257026	0,498256329
1558168_at	-	-0,924000698	-1,422257026	0,498256329
216741_at	-	-0,924000698	-1,422257026	0,498256329
233433_at	-	-0,924000698	-1,422257026	0,498256329
220354_at	MCF2L-AS1	-0,924000698	-1,422257026	0,498256329
221197_s_at	CHAT	-0,924000698	-1,422257026	0,498256329
214852_x_at	VPS13A	-0,924000698	-1,422257026	0,498256329
210155_at	MYOC	0,20740896	-0,290694975	0,498103935
218570_at	KBTBD4 /// PTPMT	3,137734155	2,639836674	0,497897481
1555514_a_at	PIAS2	0,97663406	0,479089184	0,497544876
219161_s_at	CKLF /// CKLF-CMT	4,52000033	4,022483924	0,497516406
211666_x_at	RNU86 /// RPL3 //	7,884264257	7,386763368	0,497500889
224593_at	ZNF664	4,85385304	4,356398115	0,497454925
229418_at	FAM222B	1,047341799	0,550017041	0,497324758
214306_at	OPA1	2,365121417	1,868130962	0,496990456
212229_s_at	FBXO21	3,257413106	2,760632255	0,496780851
219821_s_at	GFOD1	0,045517965	-0,45121326	0,496731224
221652_s_at	ASUN	5,004299749	4,507578586	0,496721163
227052_at	-	3,200057698	2,703479652	0,496578045
213727_x_at	MPPE1	2,99543179	2,498884538	0,496547252
218131_s_at	GATAD2A	3,007119335	2,5107204	0,496398935
239999_at	LINC00478	-2,074223907	-2,570522741	0,496298834
219962_at	ACE2	-2,074223907	-2,570522741	0,496298834
1554994_at	RAG1	-2,074223907	-2,570522741	0,496298834
215094_at	SPICE1	-2,074223907	-2,570522741	0,496298834
215469_at	-	-2,074223907	-2,570522741	0,496298834
216233_at	CD163	-2,074223907	-2,570522741	0,496298834
236287_at	-	-2,074223907	-2,570522741	0,496298834

244098_at	-	-2,074223907	-2,570522741	0,496298834
1553032_at	IL31RA	-2,074223907	-2,570522741	0,496298834
233600_at	EFCAB6	-2,074223907	-2,570522741	0,496298834
238964_at	FIGN	-2,074223907	-2,570522741	0,496298834
1565722_at	-	-2,074223907	-2,570522741	0,496298834
220674_at	CD22	-2,074223907	-2,570522741	0,496298834
243823_at	-	-2,074223907	-2,570522741	0,496298834
216515_x_at	-	6,650171508	6,153902886	0,496268622
224692_at	PPP1R15B	4,019763292	3,523783625	0,495979666
219409_at	SNIP1	1,528073958	1,032267596	0,495806362
212789_at	NCAPD3	3,825885656	3,330174376	0,49571128
1559924_at	-	0,458905032	-0,036543687	0,495448719
1558480_at	LOC100507032	-2,336678463	-2,832036647	0,495358184
211601_at	CATR1	-2,336678463	-2,832036647	0,495358184
237205_at	LINC00238	-2,336678463	-2,832036647	0,495358184
1562611_at	-	-2,336678463	-2,832036647	0,495358184
215290_at	-	-2,336678463	-2,832036647	0,495358184
223560_s_at	C2orf56	3,228532861	2,733194118	0,495338743
226472_at	PPIL4	2,855122714	2,359892448	0,495230266
221883_at	PKNOX1	2,042564225	1,547376668	0,495187557
203087_s_at	KIF2A	4,719538164	4,224659944	0,49487822
225920_at	LOC148413	3,18076822	2,686001625	0,494766595
71933_at	WNT6	1,035859431	0,541159555	0,494699876
242675_x_at	-	0,556428218	0,061773582	0,494654636
244587_at	ATF7	0,556428218	0,061773582	0,494654636
212146_at	PLEKHM2	1,160996507	0,666486137	0,49451037
37278_at	TAZ	2,375089713	1,880585087	0,494504627
209171_at	ITPA	4,288236521	3,793786385	0,494450135
219679_s_at	WAC	2,884644843	2,390281999	0,494362844
210878_s_at	KDM3B	3,076375717	2,582046772	0,494328945
1559226_x_at	LCE1E	0,900065578	0,405752839	0,494312739
228084_at	PLA2G12A	2,582572508	2,088449486	0,494123022
201693_s_at	EGR1	-0,437449947	-0,931539147	0,494089199
216234_s_at	PRKACA	-0,437449947	-0,931539147	0,494089199
205913_at	PLIN1	-0,437449947	-0,931539147	0,494089199
231014_at	TRIM50	-0,437449947	-0,931539147	0,494089199
233646_at	-	-0,437449947	-0,931539147	0,494089199
219385_at	SLAMF8	-0,437449947	-0,931539147	0,494089199
203009_at	BCAM	-0,437449947	-0,931539147	0,494089199
237506_at	TMEM177	-0,437449947	-0,931539147	0,494089199
228447_at	AKAP17A	-0,437449947	-0,931539147	0,494089199
244088_at	-	-0,437449947	-0,931539147	0,494089199
216577_at	-	-0,437449947	-0,931539147	0,494089199
225542_at	ACAP3	-0,437449947	-0,931539147	0,494089199
219082_at	AMDHD2 /// CEMI	-0,437449947	-0,931539147	0,494089199
1553919_at	C9orf62	-0,437449947	-0,931539147	0,494089199
218303_x_at	KRCC1	4,133352155	3,639810352	0,493541803
218891_at	C10orf76	0,820160788	0,326777209	0,493383578
202026_at	SDHD	5,356892072	4,863735125	0,493156947
221746_at	UBL4A	2,685337919	2,192329358	0,493008561

202754_at	R3HDM1	4,636598653	4,143962533	0,49263612
1570120_at	-	-1,369772723	-1,862269442	0,492496719
234567_at	-	-1,369772723	-1,862269442	0,492496719
206381_at	SCN2A	-1,369772723	-1,862269442	0,492496719
241574_s_at	IGF2BP1	-1,369772723	-1,862269442	0,492496719
1555305_at	FOXJ2	-1,369772723	-1,862269442	0,492496719
239968_at	LINC00313	-1,369772723	-1,862269442	0,492496719
230163_at	GFRA1	-1,369772723	-1,862269442	0,492496719
243422_at	-	-1,369772723	-1,862269442	0,492496719
204537_s_at	GABRE /// MIR224	-1,369772723	-1,862269442	0,492496719
215010_s_at	BRSK2	-1,369772723	-1,862269442	0,492496719
232576_at	-	-1,369772723	-1,862269442	0,492496719
207379_at	EDIL3	-1,369772723	-1,862269442	0,492496719
228557_at	L3MBTL4	-1,369772723	-1,862269442	0,492496719
241442_at	-	-1,369772723	-1,862269442	0,492496719
240201_at	-	-1,369772723	-1,862269442	0,492496719
240882_at	LOC100506289	-1,369772723	-1,862269442	0,492496719
216334_s_at	CYP2A7P1	-1,369772723	-1,862269442	0,492496719
213033_s_at	NFIB	-1,369772723	-1,862269442	0,492496719
1554443_s_at	BEST1	-1,369772723	-1,862269442	0,492496719
231670_at	-	-1,369772723	-1,862269442	0,492496719
244225_x_at	LMNA	-1,369772723	-1,862269442	0,492496719
1556771_a_at	LOC415056	-1,369772723	-1,862269442	0,492496719
222450_at	PMEPA1	-1,369772723	-1,862269442	0,492496719
212157_at	SDC2	-1,369772723	-1,862269442	0,492496719
232891_at	SIRPD	-1,369772723	-1,862269442	0,492496719
1552842_at	HS6ST3 /// LOC10(	-1,369772723	-1,862269442	0,492496719
221690_s_at	NLRP2	-1,369772723	-1,862269442	0,492496719
221552_at	ABHD6	0,648195588	0,155747281	0,492448308
202452_at	ZER1	0,648195588	0,155747281	0,492448308
201786_s_at	ADAR	5,744560023	5,252127909	0,492432114
205087_at	RWDD3	3,707586205	3,215288307	0,492297898
203557_s_at	PCBD1	1,983901861	1,491644074	0,492257787
219089_s_at	ZNF576	1,816938997	1,324949741	0,491989256
239802_at	SAP30L	-0,609040214	-1,100943374	0,49190316
243447_at	-	-0,609040214	-1,100943374	0,49190316
1562289_at	-	-0,609040214	-1,100943374	0,49190316
237735_at	LOC100506907	-0,609040214	-1,100943374	0,49190316
206802_at	PAX5	-0,609040214	-1,100943374	0,49190316
214538_x_at	RGS6	-0,609040214	-1,100943374	0,49190316
1568598_at	KAZALD1	-0,609040214	-1,100943374	0,49190316
234267_at	LOC100507584	-0,609040214	-1,100943374	0,49190316
241624_at	LOC389834	-0,609040214	-1,100943374	0,49190316
238971_at	-	-0,609040214	-1,100943374	0,49190316
242782_x_at	TMEM198	-0,609040214	-1,100943374	0,49190316
220641_at	NOX5	-0,609040214	-1,100943374	0,49190316
217796_s_at	NPLOC4	3,685074281	3,193461514	0,491612767
235914_at	SYNPO	0,319287178	-0,172194225	0,491481403
208347_at	-	0,319287178	-0,172194225	0,491481403
204227_s_at	TK2	0,319287178	-0,172194225	0,491481403

204397_at	EML2	0,319287178	-0,172194225	0,491481403
208799_at	PSMB5	4,335356502	3,843963198	0,491393304
1561509_at	-	-0,791294935	-1,282520722	0,491225787
214082_at	CA5B	-0,791294935	-1,282520722	0,491225787
234027_at	CCDC129	-0,791294935	-1,282520722	0,491225787
220406_at	TGFB2	-0,791294935	-1,282520722	0,491225787
243961_at	LOC100505622	-0,791294935	-1,282520722	0,491225787
239795_at	-	-0,791294935	-1,282520722	0,491225787
232776_at	-	-0,791294935	-1,282520722	0,491225787
238826_x_at	-	-0,791294935	-1,282520722	0,491225787
236897_at	-	-0,791294935	-1,282520722	0,491225787
240430_at	KCMF1	-0,791294935	-1,282520722	0,491225787
222362_at	AGFG2	-0,791294935	-1,282520722	0,491225787
238235_at	-	-0,791294935	-1,282520722	0,491225787
216301_at	-	-0,791294935	-1,282520722	0,491225787
52255_s_at	COL5A3	-0,318621864	-0,809711768	0,491089905
234061_at	-	-0,280273599	-0,771340337	0,491066738
213837_at	L3MBTL1	-0,280273599	-0,771340337	0,491066738
219480_at	SNAI1	-0,280273599	-0,771340337	0,491066738
205451_at	FOXO4	-0,280273599	-0,771340337	0,491066738
1554616_at	SERPINB8	-0,280273599	-0,771340337	0,491066738
205598_at	TRAIP	1,024284941	0,533258442	0,491026499
213102_at	ACTR3	6,095517202	5,604491695	0,491025508
221539_at	EIF4EBP1	3,03433073	2,543655878	0,490674852
210337_s_at	ACLY	3,915780568	3,425365487	0,49041508
222798_at	PTER	3,813758784	3,323485083	0,490273701
232880_at	-	0,167727503	-0,322340048	0,490067551
216696_s_at	PRODH2	0,167727503	-0,322340048	0,490067551
237234_at	CYTH2	0,167727503	-0,322340048	0,490067551
222338_x_at	LOC646808	3,029186714	2,539809146	0,489377568
217804_s_at	ILF3	2,817838865	2,328492509	0,489346356
207040_s_at	ST13	6,369925206	5,880694091	0,489231115
217840_at	DDX41	3,171328694	2,682180306	0,489148389
218150_at	ARL5A	5,016204642	4,527225386	0,488979256
1559201_a_at	-	1,139057614	0,65030602	0,488751594
217322_x_at	-	1,139057614	0,65030602	0,488751594
224502_s_at	KIAA1191	4,19821572	3,709577987	0,488637732
213897_s_at	MRPL23	4,878355601	4,389718133	0,488637468
232419_at	TMEM132A	0,425036312	-0,063377083	0,488413395
226627_at	39692	2,2388565	1,750483134	0,488373366
226044_at	TDP1	2,190140919	1,701800759	0,48834016
222554_s_at	NOL6	0,873789652	0,385582632	0,48820702
1555731_a_at	AP1S3	-0,135065865	-0,623254098	0,488188233
244667_at	-	-0,135065865	-0,623254098	0,488188233
200913_at	PPM1G	3,906843271	3,41869868	0,48814459
227940_at	LOC339803	1,578719607	1,090640407	0,4880792
235192_at	TP53RK	1,578719607	1,090640407	0,4880792
1554018_at	GPNMB	-2,416625063	-2,904470894	0,487845831
1569406_at	-	-2,416625063	-2,904470894	0,487845831
204148_s_at	POMZP3 /// ZP3	1,071336699	0,583607503	0,487729195

218527_at	APTX	2,752615529	2,264929589	0,48768594
1552473_at	GAMT	1,65554524	1,167919222	0,487626018
201988_s_at	CREBL2	2,019346222	1,531877749	0,487468473
203577_at	GTF2H4	2,042564225	1,555165465	0,487398759
207907_at	TNFSF14	1,830468611	1,343200523	0,487268088
208734_x_at	RAB2A	4,258822873	3,771650587	0,487172287
232700_at	-	0,524545436	0,03784737	0,486698066
219111_s_at	DDX54	1,287793882	0,80114751	0,486646372
238519_at	DDI2 /// RSC1A1	2,304041045	1,817403386	0,486637659
227990_at	SLU7	3,282538749	2,796775008	0,485763741
244179_x_at	GGT1 /// GGT2 ///	0,001114523	-0,484558493	0,485673016
241984_at	FOXN3	0,001114523	-0,484558493	0,485673016
223253_at	EPDR1	3,873276566	3,387778112	0,485498454
208801_at	SRP72	5,541810369	5,05640944	0,485400929
210013_at	HPX	0,618277321	0,133104519	0,485172802
1554538_at	RHOF	0,618277321	0,133104519	0,485172802
221797_at	C17orf90	2,444186596	1,9592579	0,484928697
212208_at	MED13L	2,817838865	2,332940758	0,484898107
207163_s_at	AKT1	2,692288672	2,207440269	0,484848403
238623_at	-	2,248155141	1,76396664	0,484188502
221071_at	-	0,282358733	-0,201789721	0,484148454
1556127_at	DIP2A	0,282358733	-0,201789721	0,484148454
216492_at	KIR3DX1	0,282358733	-0,201789721	0,484148454
1557823_s_at	LOC401134	-1,982324124	-2,466349414	0,48402529
217126_at	-	-1,982324124	-2,466349414	0,48402529
241986_at	BMPER	-1,982324124	-2,466349414	0,48402529
222246_at	-	-1,982324124	-2,466349414	0,48402529
205696_s_at	GFRA1	-1,982324124	-2,466349414	0,48402529
220860_at	PURG	-1,982324124	-2,466349414	0,48402529
215601_at	-	-1,982324124	-2,466349414	0,48402529
237834_at	SNCAIP	-1,982324124	-2,466349414	0,48402529
243865_x_at	-	-1,982324124	-2,466349414	0,48402529
1552599_at	PXT1	-1,982324124	-2,466349414	0,48402529
1562655_at	-	-1,982324124	-2,466349414	0,48402529
234559_at	-	-1,982324124	-2,466349414	0,48402529
242006_at	LCA5	-1,982324124	-2,466349414	0,48402529
1561054_a_at	CCDC14	-1,982324124	-2,466349414	0,48402529
1557541_at	FAM201A	-1,982324124	-2,466349414	0,48402529
204298_s_at	LOX	-1,982324124	-2,466349414	0,48402529
237238_at	WWC1	-1,982324124	-2,466349414	0,48402529
1553448_at	FLJ34503	-1,982324124	-2,466349414	0,48402529
1554413_s_at	SNX29P1 /// SNX2	-1,982324124	-2,466349414	0,48402529
219026_s_at	RASAL2	-1,982324124	-2,466349414	0,48402529
1561170_at	GOLGA6L1 /// GOL	-1,982324124	-2,466349414	0,48402529
241452_at	-	-1,982324124	-2,466349414	0,48402529
208346_at	PPBPP2	-1,982324124	-2,466349414	0,48402529
240822_at	TDP1	-1,982324124	-2,466349414	0,48402529
205560_at	PCSK5	-1,982324124	-2,466349414	0,48402529
237522_at	FAS-AS1	-1,982324124	-2,466349414	0,48402529
243726_at	-	-1,982324124	-2,466349414	0,48402529

217649_at	ZFAND5	-1,982324124	-2,466349414	0,48402529
225683_x_at	PHPT1	1,326463531	0,842599219	0,483864312
218961_s_at	PNKP	1,983901861	1,500059916	0,483841944
218166_s_at	RSF1	1,983901861	1,500059916	0,483841944
206039_at	RAB33A	4,030423661	3,546728932	0,48369473
79005_at	SLC35E1	2,069603004	1,586172329	0,483430675
217957_at	C16orf80	5,222661752	4,739375943	0,483285809
235567_at	RORA	0,926325262	0,443143223	0,483182039
202073_at	OPTN	0,926325262	0,443143223	0,483182039
1553677_a_at	TIPRL	4,027733188	3,544644421	0,483088767
225317_at	ACBD6	3,282538749	2,799505781	0,483032969
224944_at	TMPO	5,648666752	5,165943912	0,48272284
235443_at	LOC100131067	2,312738346	1,830129603	0,482608743
1566135_at	CARHSP1	0,127732831	-0,354781582	0,482514412
1569879_a_at	MEGF11	0,127732831	-0,354781582	0,482514412
219413_at	ACBD4	0,127732831	-0,354781582	0,482514412
213896_x_at	FAM149B1	1,225123479	0,742671819	0,48245166
212916_at	PHF8	1,225123479	0,742671819	0,48245166
207730_x_at	-	2,492739983	2,010406245	0,482333738
212961_x_at	CXorf40B	3,732463367	3,250322783	0,482140584
201273_s_at	SRP9	7,252311781	6,77019399	0,482117791
224333_s_at	MRPS5	3,889146356	3,407104418	0,482041937
221564_at	PRMT2	3,185443259	2,703479652	0,481963607
200034_s_at	RPL6	7,738486181	7,256598332	0,481887849
209422_at	PHF20	4,918218598	4,436438184	0,481780413
223527_s_at	CDADC1	1,760255626	1,278654284	0,481601342
213037_x_at	STAU1	5,061011789	4,579462624	0,481549165
228380_at	SENP2	0,847529938	0,366031214	0,481498724
218258_at	POLR1D	5,666877479	5,185688448	0,48118903
201392_s_at	IGF2R	2,190140919	1,709047448	0,481093471
231101_at	-	0,39105295	-0,089635713	0,480688663
221519_at	FBXW4	2,118167848	1,637482551	0,480685298
200919_at	PHC2	3,577387089	3,096957539	0,48042955
220142_at	HAPLN2	-0,180990326	-0,661178575	0,480188249
211402_x_at	NR6A1	-0,180990326	-0,661178575	0,480188249
216888_at	LDB3	-0,180990326	-0,661178575	0,480188249
223381_at	NUF2	4,716606434	4,236528682	0,480077752
241922_at	LMO4	-0,49284695	-0,972893339	0,480046388
223601_at	OLFM2	-0,49284695	-0,972893339	0,480046388
228172_at	-	-0,49284695	-0,972893339	0,480046388
228528_at	-	-0,49284695	-0,972893339	0,480046388
44702_at	SYDE1	1,230065286	0,750077127	0,479988159
1556505_at	LINC00605	-1,137208348	-1,616924751	0,479716403
233209_at	LOC200609	-1,137208348	-1,616924751	0,479716403
1561844_at	-	-1,137208348	-1,616924751	0,479716403
216075_at	-	-1,137208348	-1,616924751	0,479716403
205947_s_at	VIPR2	-1,137208348	-1,616924751	0,479716403
1569885_at	LOC100302640	-1,137208348	-1,616924751	0,479716403
215939_at	-	-1,137208348	-1,616924751	0,479716403
240087_at	-	-1,137208348	-1,616924751	0,479716403

1553155_x_at	ATP6V0D2	-1,137208348	-1,616924751	0,479716403
226139_at	CCDC149	-1,137208348	-1,616924751	0,479716403
233064_at	ZFR2	-1,137208348	-1,616924751	0,479716403
230603_at	COL27A1	-1,137208348	-1,616924751	0,479716403
208135_at	HNF1B	-1,137208348	-1,616924751	0,479716403
242350_s_at	LOC100128098	-1,137208348	-1,616924751	0,479716403
208473_s_at	GP2	-1,137208348	-1,616924751	0,479716403
1556633_at	C1orf204	-1,137208348	-1,616924751	0,479716403
244122_at	MGST3	-1,137208348	-1,616924751	0,479716403
1566456_at	-	-1,137208348	-1,616924751	0,479716403
204249_s_at	LMO2	-1,137208348	-1,616924751	0,479716403
204303_s_at	CTIF	-1,137208348	-1,616924751	0,479716403
220490_at	SERINC2	-1,137208348	-1,616924751	0,479716403
225345_s_at	FBXO32	-1,137208348	-1,616924751	0,479716403
208962_s_at	FADS1 /// MIR190	2,590269697	2,110584371	0,479685326
215482_s_at	EIF2B4	3,57383031	3,094388414	0,479441896
235408_x_at	ZNF117	-0,332405896	-0,811769547	0,479363651
237300_at	PSMA3	-0,332405896	-0,811769547	0,479363651
231224_x_at	PRKAG2	-0,332405896	-0,811769547	0,479363651
1553137_s_at	KLF11	-0,332405896	-0,811769547	0,479363651
211696_x_at	HBB	-0,332405896	-0,811769547	0,479363651
244471_x_at	PANX2	-0,332405896	-0,811769547	0,479363651
219625_s_at	COL4A3BP	3,03433073	2,55501164	0,47931909
236614_at	LOC729683	0,97663406	0,497424714	0,479209346
222420_s_at	UBE2H	3,430413158	2,951484533	0,478928625
225348_at	SRSF10	2,932161212	2,45323331	0,478927902
210162_s_at	NFATC1	0,491906512	0,013190398	0,478716114
206970_at	CNTN2	0,491906512	0,013190398	0,478716114
219793_at	SNX16	0,764143511	0,286279868	0,477863643
210597_x_at	PRB1	0,764143511	0,286279868	0,477863643
40640_at	NCAPH2	0,834298672	0,356529472	0,4777692
221262_s_at	SLC2A11	-0,994186487	-1,471833228	0,477646741
220066_at	NOD2	-0,994186487	-1,471833228	0,477646741
244494_at	-	-0,994186487	-1,471833228	0,477646741
236854_at	LINC00494	-0,994186487	-1,471833228	0,477646741
217038_at	-	-0,994186487	-1,471833228	0,477646741
222072_at	LOC100505933	-0,994186487	-1,471833228	0,477646741
224032_x_at	SPANXA1 /// SPAN	-0,994186487	-1,471833228	0,477646741
233131_at	CCDC147	-0,994186487	-1,471833228	0,477646741
217688_at	-	-0,994186487	-1,471833228	0,477646741
204685_s_at	ATP2B2	-0,994186487	-1,471833228	0,477646741
1558654_at	PPM1H	-0,994186487	-1,471833228	0,477646741
211845_at	PVRL1	-0,994186487	-1,471833228	0,477646741
202510_s_at	TNFAIP2	-0,994186487	-1,471833228	0,477646741
223085_at	RNF19A	3,362258367	2,884677311	0,477581056
223195_s_at	SESN2	1,65554524	1,178100917	0,477444322
226308_at	HAUS8	1,578719607	1,101780212	0,476939395
226307_at	CRTC2	1,578719607	1,101780212	0,476939395
221736_at	RALGAPB	2,436165655	1,9592579	0,476907755
229763_at	FOXP4	1,20396005	0,727123109	0,476836941

201415_at	GSS	3,64600241	3,169167796	0,476834614
200794_x_at	DAZAP2	5,664955538	5,18829749	0,476658049
200729_s_at	ACTR2	5,809852611	5,333251113	0,476601499
212898_at	TTI1	3,370643409	2,894109605	0,476533804
221502_at	KPNA3	4,109088344	3,632850102	0,476238242
208308_s_at	GPI	6,162552061	5,686391844	0,476160218
212863_x_at	CTBP1	4,93324721	4,45742068	0,47582653
216092_s_at	SLC7A8	0,244681185	-0,231045907	0,475727092
233449_at	-	-0,66934516	-1,145057014	0,475711855
239705_at	-	-0,66934516	-1,145057014	0,475711855
223930_at	-	-0,66934516	-1,145057014	0,475711855
1554549_a_at	WDR20	-0,66934516	-1,145057014	0,475711855
242246_x_at	MEG3	-0,66934516	-1,145057014	0,475711855
220257_x_at	NXF2 /// NXF2B	-0,66934516	-1,145057014	0,475711855
227032_at	PLXNA2	-0,66934516	-1,145057014	0,475711855
205918_at	SLC4A3	-0,66934516	-1,145057014	0,475711855
239221_at	GPR123	-0,66934516	-1,145057014	0,475711855
232046_at	KIAA1217	-0,66934516	-1,145057014	0,475711855
241392_at	TMEM39A	-0,66934516	-1,145057014	0,475711855
215856_at	SIGLEC15	-0,66934516	-1,145057014	0,475711855
231682_at	-	-0,66934516	-1,145057014	0,475711855
223169_s_at	RHOU	-0,66934516	-1,145057014	0,475711855
202740_at	ABHD14A-ACY1 //,	2,597647262	2,121960601	0,475686661
211073_x_at	RNU86 /// RPL3 //,	8,231728811	7,7561143	0,475614512
201021_s_at	DSTN	5,205487521	4,730136072	0,475351449
207607_at	ASCL2	-2,495152701	-2,970378591	0,47522589
206503_x_at	PML	1,610353504	1,135235995	0,475117509
211971_s_at	LRPPRC	5,280168491	4,805199352	0,474969139
200728_at	ACTR2	6,388603216	5,913910779	0,474692437
210621_s_at	RASA1	3,340803206	2,86626857	0,474534636
232072_at	-	-0,043925712	-0,518232988	0,474307276
1569782_at	-	-1,289805289	-1,763664074	0,473858785
223761_at	FGF19	-1,289805289	-1,763664074	0,473858785
243717_at	EPHA10	-1,289805289	-1,763664074	0,473858785
1556078_at	-	-1,289805289	-1,763664074	0,473858785
241830_at	C20orf112	-1,289805289	-1,763664074	0,473858785
205901_at	PNOC	-1,289805289	-1,763664074	0,473858785
210862_s_at	SARDH	-1,289805289	-1,763664074	0,473858785
241923_x_at	NANS	-1,289805289	-1,763664074	0,473858785
204130_at	HSD11B2	-1,289805289	-1,763664074	0,473858785
233928_at	ADAMTS9-AS1	-1,289805289	-1,763664074	0,473858785
243237_at	-	-1,289805289	-1,763664074	0,473858785
240672_at	WDR74	-1,289805289	-1,763664074	0,473858785
1569300_at	MFAP3L	-1,289805289	-1,763664074	0,473858785
1569462_x_at	KCNT1	-1,289805289	-1,763664074	0,473858785
1560753_at	-	-1,289805289	-1,763664074	0,473858785
206675_s_at	SKIL	-1,289805289	-1,763664074	0,473858785
217101_at	TTC39A	-1,289805289	-1,763664074	0,473858785
230527_at	-	-1,289805289	-1,763664074	0,473858785
206372_at	MYF6	-1,289805289	-1,763664074	0,473858785

219477_s_at	MRPS31P3 /// THS	1,421391637	0,947673886	0,473717751
208911_s_at	PDHB	4,326621119	3,852972046	0,473649072
215948_x_at	ZMYM5	1,246401134	0,772793615	0,473607519
222140_s_at	GPR89A /// GPR89	3,086998005	2,613615641	0,473382364
209816_at	PTCH1	-1,712732543	-2,186086603	0,47335406
240308_at	-	-1,712732543	-2,186086603	0,47335406
1555116_s_at	SLC11A1	-1,712732543	-2,186086603	0,47335406
1556350_a_at	EIF4A2	-1,712732543	-2,186086603	0,47335406
1553426_at	C5orf64	-1,712732543	-2,186086603	0,47335406
1557211_a_at	FAM181A-AS1	-1,712732543	-2,186086603	0,47335406
217082_at	-	-1,712732543	-2,186086603	0,47335406
241022_at	-	-1,712732543	-2,186086603	0,47335406
216905_s_at	ST14	-1,712732543	-2,186086603	0,47335406
215219_at	DOPEY2	-1,712732543	-2,186086603	0,47335406
241579_at	-	-1,712732543	-2,186086603	0,47335406
1569751_at	TBC1D26	-1,712732543	-2,186086603	0,47335406
220117_at	ZNF385D	-1,712732543	-2,186086603	0,47335406
1552843_at	SLC26A1	-1,712732543	-2,186086603	0,47335406
1561342_at	LOC150005	-1,712732543	-2,186086603	0,47335406
234965_at	-	-1,712732543	-2,186086603	0,47335406
243556_at	NGEF	-1,712732543	-2,186086603	0,47335406
1566543_at	-	-1,712732543	-2,186086603	0,47335406
235684_s_at	SESN3	-1,712732543	-2,186086603	0,47335406
1564490_at	LOC100128830	-1,712732543	-2,186086603	0,47335406
235571_at	-	-1,712732543	-2,186086603	0,47335406
206139_at	PI4KB	0,95222556	0,479089184	0,473136376
1569268_at	GRIN2C	0,08619576	-0,386896102	0,473091862
236132_at	TLN1	0,08619576	-0,386896102	0,473091862
233565_s_at	FKBP1A-SDCBP2 //	0,08619576	-0,386896102	0,473091862
234106_s_at	FLYWCH1	0,08619576	-0,386896102	0,473091862
1553260_s_at	ALS2CR11	-2,250580859	-2,723114724	0,472533865
1563721_at	-	-2,250580859	-2,723114724	0,472533865
208508_s_at	OR2J2	-2,250580859	-2,723114724	0,472533865
208098_at	OR12D3 /// OR5V1	-2,250580859	-2,723114724	0,472533865
243850_at	-	-2,250580859	-2,723114724	0,472533865
236544_at	-	-2,250580859	-2,723114724	0,472533865
237998_at	-	-2,250580859	-2,723114724	0,472533865
1555299_s_at	ERVW-1	-2,250580859	-2,723114724	0,472533865
214566_at	SMR3A	-2,250580859	-2,723114724	0,472533865
244760_at	-	-2,250580859	-2,723114724	0,472533865
1561951_at	SLC5A12	-2,250580859	-2,723114724	0,472533865
1564905_at	-	-2,250580859	-2,723114724	0,472533865
1564198_a_at	C10orf90	-2,250580859	-2,723114724	0,472533865
1562292_at	ANKRD30B	-2,250580859	-2,723114724	0,472533865
1556511_a_at	-	-2,250580859	-2,723114724	0,472533865
214903_at	SYT2	0,355751005	-0,116767475	0,47251848
203554_x_at	PTTG1	6,553803322	6,081328234	0,472475087
216068_at	-	-2,163157732	-2,635549085	0,472391353
235400_at	FCRLA	-2,163157732	-2,635549085	0,472391353
219934_s_at	SULT1E1	-2,163157732	-2,635549085	0,472391353

1555433_at	SLC39A14	-2,163157732	-2,635549085	0,472391353
1561324_at	-	-2,163157732	-2,635549085	0,472391353
205572_at	ANGPT2	-2,163157732	-2,635549085	0,472391353
1561271_at	CCDC144C	-2,163157732	-2,635549085	0,472391353
231593_at	-	-2,163157732	-2,635549085	0,472391353
1553052_at	WFDC13	-2,163157732	-2,635549085	0,472391353
1568513_x_at	-	1,871521385	1,399283506	0,472237879
201923_at	PRDX4	5,718535483	5,246483322	0,472052161
203073_at	COG2	3,300874993	2,828849722	0,472025271
1557763_at	LPAR6	-1,624955693	-2,09678811	0,471832417
213967_at	RALYL	-1,624955693	-2,09678811	0,471832417
1559837_at	-	-1,624955693	-2,09678811	0,471832417
1553069_at	BRWD1-IT2	-1,624955693	-2,09678811	0,471832417
1559277_at	FLJ35700	-1,624955693	-2,09678811	0,471832417
211153_s_at	TNFSF11	-1,624955693	-2,09678811	0,471832417
1559018_at	PTPRE	-1,624955693	-2,09678811	0,471832417
215795_at	MYH7B	-1,624955693	-2,09678811	0,471832417
208126_s_at	CYP2C18	-1,624955693	-2,09678811	0,471832417
241328_at	ZMAT1	-1,624955693	-2,09678811	0,471832417
244330_at	-	-1,624955693	-2,09678811	0,471832417
224942_at	PAPPA	-1,624955693	-2,09678811	0,471832417
205892_s_at	FABP1	-1,624955693	-2,09678811	0,471832417
213443_at	TRADD	-1,624955693	-2,09678811	0,471832417
223782_s_at	TINAG	-1,624955693	-2,09678811	0,471832417
205980_s_at	ARHGAP8 /// PRR5	-1,624955693	-2,09678811	0,471832417
215642_at	-	-1,624955693	-2,09678811	0,471832417
231590_at	GATM	-1,624955693	-2,09678811	0,471832417
239162_at	DAPK1-IT1	-1,624955693	-2,09678811	0,471832417
1561728_a_at	LOC400238	-1,624955693	-2,09678811	0,471832417
206717_at	MYH8	-1,624955693	-2,09678811	0,471832417
205549_at	PCP4	-1,624955693	-2,09678811	0,471832417
234444_at	-	-1,624955693	-2,09678811	0,471832417
240209_at	C16orf78	-1,624955693	-2,09678811	0,471832417
239620_at	TTC23L	-1,624955693	-2,09678811	0,471832417
217805_at	ILF3	2,804738618	2,332940758	0,47179786
213021_at	GOSR1	3,122148189	2,65087728	0,471270909
1569728_at	LINC00423	-0,856259172	-1,32740143	0,471142258
209779_at	LLPH	-0,856259172	-1,32740143	0,471142258
1556564_at	HHIPL1	-0,856259172	-1,32740143	0,471142258
236904_x_at	TECTA	-0,856259172	-1,32740143	0,471142258
1552773_at	CLEC4D	-0,856259172	-1,32740143	0,471142258
231994_at	CHDH	-0,856259172	-1,32740143	0,471142258
225354_s_at	SH3BGRL2	-0,856259172	-1,32740143	0,471142258
1568828_at	LOC730441	-0,856259172	-1,32740143	0,471142258
1569891_at	ATP5A1	-0,856259172	-1,32740143	0,471142258
226129_at	FAM83H	-0,856259172	-1,32740143	0,471142258
1555267_at	GRID1	-0,856259172	-1,32740143	0,471142258
1562733_at	LINC00092	-0,856259172	-1,32740143	0,471142258
212400_at	FAM102A	3,060716358	2,589579418	0,47113694
204738_s_at	KRIT1	1,182925501	0,711934445	0,470991056

217816_s_at	PCNP	5,401573936	4,930612834	0,470961102
202564_x_at	ARL2	3,697863752	3,227213858	0,470649894
226715_at	FOXK1	1,5944803	1,123905343	0,470574957
229405_at	KIF7	0,735999505	0,265475485	0,47052402
218707_at	ZNF444	0,735999505	0,265475485	0,47052402
36742_at	TRIM15	-0,328217035	-0,798423011	0,470205975
205914_s_at	GRIN1	-1,06392583	-1,533994857	0,470069026
221844_x_at	SPCS3	4,42686741	3,956802611	0,470064799
227122_at	-	0,648195588	0,178191865	0,470003723
238115_at	DNAJC18	0,458905032	-0,011001236	0,469906267
1557849_at	-	-2,336678463	-2,8064286	0,469750136
1559949_at	-	-2,336678463	-2,8064286	0,469750136
240599_x_at	-	-2,336678463	-2,8064286	0,469750136
206251_s_at	AVPR1A	-2,336678463	-2,8064286	0,469750136
215692_s_at	MPPED2	-2,336678463	-2,8064286	0,469750136
209487_at	RBPMS	-2,336678463	-2,8064286	0,469750136
211080_s_at	NEK2	2,21987062	1,750483134	0,469387487
209358_at	TAF11	4,204964809	3,735670695	0,469294114
202702_at	TRIM26	2,884644843	2,415420763	0,46922408
223261_at	POLK	3,386206804	2,918087241	0,468119563
238963_at	-	1,476175815	1,008210238	0,467965577
201248_s_at	SREBF2	1,857989965	1,390070295	0,46791967
203022_at	RNASEH2A	4,404121066	3,936585452	0,467535614
37117_at	ARHGAP8 /// PRR5	-1,289074806	-1,75657516	0,467500353
1567855_at	ZNF29P	-1,802716385	-2,27001641	0,467300025
238502_at	-	-1,802716385	-2,27001641	0,467300025
1558977_at	LOC100130992	-1,802716385	-2,27001641	0,467300025
224169_at	NPFFR2	-1,802716385	-2,27001641	0,467300025
206685_at	HCG4	-1,802716385	-2,27001641	0,467300025
244883_at	-	-1,802716385	-2,27001641	0,467300025
204884_s_at	HUS1	-1,802716385	-2,27001641	0,467300025
209560_s_at	DLK1	-1,802716385	-2,27001641	0,467300025
1566277_at	OR5E1P	-1,802716385	-2,27001641	0,467300025
230802_at	ARHGAP24	-1,802716385	-2,27001641	0,467300025
237636_at	PPP2R2A	-1,802716385	-2,27001641	0,467300025
1552554_a_at	NLRC4	-1,802716385	-2,27001641	0,467300025
206377_at	FOXF2	-1,802716385	-2,27001641	0,467300025
208307_at	RBMY1A1 /// RBM	-1,802716385	-2,27001641	0,467300025
238258_at	WBSCR28	-1,802716385	-2,27001641	0,467300025
242541_at	ABCA9	-1,802716385	-2,27001641	0,467300025
207569_at	ROS1	-1,802716385	-2,27001641	0,467300025
228534_s_at	-	-1,802716385	-2,27001641	0,467300025
206156_at	GJB5	-1,802716385	-2,27001641	0,467300025
219902_at	BHMT2	-1,802716385	-2,27001641	0,467300025
240407_at	LOC100126784	-1,802716385	-2,27001641	0,467300025
1563978_at	LOC728690	-1,802716385	-2,27001641	0,467300025
204066_s_at	AGAP1	-1,802716385	-2,27001641	0,467300025
1554194_at	-	-1,802716385	-2,27001641	0,467300025
240825_at	-	-1,802716385	-2,27001641	0,467300025
1561323_at	LOC339975	-1,802716385	-2,27001641	0,467300025

234553_at	-	-1,802716385	-2,27001641	0,467300025
1570491_at	-	-1,802716385	-2,27001641	0,467300025
240426_at	FAM47B	-1,802716385	-2,27001641	0,467300025
222020_s_at	LOC100653217 ///	-1,802716385	-2,27001641	0,467300025
212353_at	SULF1	-1,802716385	-2,27001641	0,467300025
213284_at	ZFP36L1	-1,537851782	-2,005029581	0,467177799
242856_at	-	-1,537851782	-2,005029581	0,467177799
230237_at	ADCYAP1	-1,537851782	-2,005029581	0,467177799
202493_x_at	CSH1	-1,537851782	-2,005029581	0,467177799
219976_at	HOOK1	-1,537851782	-2,005029581	0,467177799
239809_at	-	-1,537851782	-2,005029581	0,467177799
240743_at	-	-1,537851782	-2,005029581	0,467177799
237132_at	TJP2	-1,537851782	-2,005029581	0,467177799
208575_at	HIST1H3A /// HIST	-1,537851782	-2,005029581	0,467177799
1561353_at	-	-1,537851782	-2,005029581	0,467177799
239345_at	SLC19A3	-1,537851782	-2,005029581	0,467177799
216534_at	-	-1,537851782	-2,005029581	0,467177799
1563132_at	-	-1,537851782	-2,005029581	0,467177799
1554514_at	ACSM5	-1,537851782	-2,005029581	0,467177799
1560757_at	TOP1MT	-1,537851782	-2,005029581	0,467177799
207369_at	BRS3	-1,537851782	-2,005029581	0,467177799
1554372_at	-	-1,537851782	-2,005029581	0,467177799
232982_at	SYNRG	-1,537851782	-2,005029581	0,467177799
1558549_s_at	VNN1	-1,537851782	-2,005029581	0,467177799
216011_at	SLC39A9	-1,537851782	-2,005029581	0,467177799
1569750_at	-	-1,537851782	-2,005029581	0,467177799
232157_at	SPRY3	-1,537851782	-2,005029581	0,467177799
239986_at	-	-1,537851782	-2,005029581	0,467177799
1556513_at	ZNF573	-1,537851782	-2,005029581	0,467177799
216946_at	HLA-DOA	-1,537851782	-2,005029581	0,467177799
222328_x_at	-	-1,537851782	-2,005029581	0,467177799
231141_at	LOC100506983	-1,537851782	-2,005029581	0,467177799
205941_s_at	COL10A1	-1,537851782	-2,005029581	0,467177799
201107_s_at	THBS1	-1,537851782	-2,005029581	0,467177799
229834_at	NFIX	-1,537851782	-2,005029581	0,467177799
221181_at	-	-1,537851782	-2,005029581	0,467177799
227530_at	AKAP12	-1,537851782	-2,005029581	0,467177799
230487_at	C6orf99	-0,230432956	-0,697182596	0,466749641
1553248_at	CCDC57	-0,230432956	-0,697182596	0,466749641
233956_at	-	-0,230432956	-0,697182596	0,466749641
205757_at	ENTPD5	-0,230432956	-0,697182596	0,466749641
234494_x_at	-	-0,230432956	-0,697182596	0,466749641
203861_s_at	ACTN2	-0,230432956	-0,697182596	0,466749641
241943_at	C18orf61	-0,230432956	-0,697182596	0,466749641
234321_x_at	NHSL1	-0,230432956	-0,697182596	0,466749641
210392_x_at	NR6A1	-0,230432956	-0,697182596	0,466749641
226572_at	SOCS7	2,321828846	1,855279162	0,466549685
210142_x_at	FLOT1	3,777053516	3,31062689	0,466426626
232946_s_at	NADSYN1	2,436165655	1,970106792	0,466058862
1555504_at	TYR	-2,569309811	-3,035090645	0,465780833

220529_at	FLJ11710	-0,088974936	-0,554696666	0,465721731
217275_at	TSSK2	-0,088974936	-0,554696666	0,465721731
218038_at	ATP5SL	2,597647262	2,132145748	0,465501514
204297_at	PIK3C3	2,798156197	2,332940758	0,465215439
210070_s_at	CHKB-CPT1B /// CI	0,791908897	0,326777209	0,465131688
43544_at	MED16	2,891198586	2,426080816	0,465117769
1554943_at	VIL1	-0,384193355	-0,84928999	0,465096635
211447_s_at	PDE4A	-0,384193355	-0,84928999	0,465096635
237445_at	-	-0,384193355	-0,84928999	0,465096635
1559909_a_at	TIMM17B	-0,384193355	-0,84928999	0,465096635
244627_at	DAK	-0,384193355	-0,84928999	0,465096635
206129_s_at	ARSB	-0,384193355	-0,84928999	0,465096635
210838_s_at	ACVRL1	-0,384193355	-0,84928999	0,465096635
238445_x_at	MGAT5B	-0,384193355	-0,84928999	0,465096635
213867_x_at	ACTB /// LOC1005(	7,964181448	7,499578239	0,464603209
47105_at	DUS2L	1,71749024	1,252915052	0,464575189
216675_at	-	-0,551284523	-1,015398016	0,464113493
217270_s_at	DYRK1B	-0,551284523	-1,015398016	0,464113493
228579_at	KCNQ3	-0,551284523	-1,015398016	0,464113493
215859_at	NCLN	-0,551284523	-1,015398016	0,464113493
231537_at	-	-0,551284523	-1,015398016	0,464113493
238245_at	ENPP7	-0,551284523	-1,015398016	0,464113493
240274_at	-	-0,551284523	-1,015398016	0,464113493
230499_at	BIRC3	2,811067138	2,346979857	0,46408728
212277_at	MTMR4	3,588695979	3,124666253	0,464029726
229456_s_at	DDAH1	0,045517965	-0,418248858	0,463766823
211199_s_at	ICOSLG	0,045517965	-0,418248858	0,463766823
1557892_at	LOC100144597	0,045517965	-0,418248858	0,463766823
234729_at	PHKG1	0,045517965	-0,418248858	0,463766823
216271_x_at	SYDE1	0,045517965	-0,418248858	0,463766823
222286_at	SNAPC3	0,045517965	-0,418248858	0,463766823
201956_s_at	GNPAT	5,216996489	4,753282184	0,463714305
206830_at	SLC4A10	-2,416625063	-2,880284116	0,463659052
234426_x_at	PIH2	-2,416625063	-2,880284116	0,463659052
241194_at	-	-2,416625063	-2,880284116	0,463659052
241655_at	-	-2,416625063	-2,880284116	0,463659052
234632_x_at	-	-2,416625063	-2,880284116	0,463659052
224447_s_at	MIEN1	4,133352155	3,669940122	0,463412033
201688_s_at	TPD52	3,713669011	3,250322783	0,463346228
228569_at	PAPOLA	3,190097062	2,726850891	0,463246171
241878_at	-	0,524545436	0,061773582	0,462771854
33197_at	MYO7A	-0,958666825	-1,42143293	0,462766105
202886_s_at	PPP2R1B	1,730929079	1,268569037	0,462360041
218287_s_at	EIF2C1	2,085817135	1,623485524	0,462331611
201384_s_at	NBR1	3,978219531	3,515907973	0,462311557
202244_at	PSMB4	7,294866687	6,832625105	0,462241582
226916_x_at	DPP9	1,345611839	0,883374248	0,46223759
1560495_at	-	-2,074223907	-2,536339282	0,462115375
239984_at	-	-2,074223907	-2,536339282	0,462115375
237997_s_at	-	-2,074223907	-2,536339282	0,462115375

227241_at	MUC15	-2,074223907	-2,536339282	0,462115375
240739_at	-	-2,074223907	-2,536339282	0,462115375
233739_at	-	-2,074223907	-2,536339282	0,462115375
221202_at	-	-2,074223907	-2,536339282	0,462115375
237269_at	-	-2,074223907	-2,536339282	0,462115375
205699_at	MAP2K6	-2,074223907	-2,536339282	0,462115375
1554941_at	KLHL14	-2,074223907	-2,536339282	0,462115375
244392_at	-	-2,074223907	-2,536339282	0,462115375
207036_x_at	GRIN2D	-2,074223907	-2,536339282	0,462115375
238354_x_at	-	-2,074223907	-2,536339282	0,462115375
242152_at	-	-2,074223907	-2,536339282	0,462115375
1569235_a_at	-	-2,074223907	-2,536339282	0,462115375
231486_x_at	-	-2,074223907	-2,536339282	0,462115375
236021_at	-	-2,074223907	-2,536339282	0,462115375
238510_at	ZNF720	3,777053516	3,315052527	0,462000989
202559_x_at	CHTOP	4,362103226	3,900176622	0,461926605
235750_at	-	1,458255986	0,996453274	0,461802713
214093_s_at	FUBP1	3,370643409	2,908944587	0,461698823
214172_x_at	RYK	1,671397663	1,209973561	0,461424101
217407_x_at	PPIL2	1,20396005	0,742671819	0,461288231
226626_at	THOC2	3,473999051	3,012928641	0,461070409
206985_at	HSD17B3	-0,730013898	-1,19085694	0,460843042
238285_at	SOX5	-0,730013898	-1,19085694	0,460843042
221186_at	LOC100131532	-0,730013898	-1,19085694	0,460843042
222337_at	-	-0,730013898	-1,19085694	0,460843042
241603_at	ATP11A	-0,730013898	-1,19085694	0,460843042
214384_s_at	DCTN2	-0,730013898	-1,19085694	0,460843042
226138_s_at	-	-0,730013898	-1,19085694	0,460843042
222346_at	LAMA1	-0,730013898	-1,19085694	0,460843042
233362_at	ZNF341	-0,730013898	-1,19085694	0,460843042
214534_at	HIST1H1B	-0,730013898	-1,19085694	0,460843042
240033_at	PLG	-0,730013898	-1,19085694	0,460843042
231531_at	C3orf24	-0,730013898	-1,19085694	0,460843042
89948_at	PCIF1	1,420069595	0,959280565	0,46078903
235159_at	-	1,936380191	1,475630127	0,460750064
201441_at	COX6B1	6,404469458	5,943921541	0,460547917
212315_s_at	NUP210	2,657308324	2,197404429	0,459903894
217173_s_at	LDLR	1,094118704	0,634301128	0,459817576
218231_at	NAGK	4,211840302	3,7524971	0,459343202
53987_at	RANBP10	0,806538976	0,34725273	0,459286246
218124_at	RETSAT	2,295108393	1,836262316	0,458846077
1555594_a_at	MBNL1	1,830468611	1,371634653	0,458833958
228991_at	CDK13	2,118167848	1,659532492	0,458635357
212042_x_at	RPL7	8,219241199	7,760716055	0,458525144
214593_at	PIAS2	-1,452713826	-1,911206569	0,458492743
235876_at	TMEM218	-1,452713826	-1,911206569	0,458492743
1558323_at	TMEM72	-1,452713826	-1,911206569	0,458492743
240320_at	C14orf164	-1,452713826	-1,911206569	0,458492743
219771_at	TBC1D8B	-1,452713826	-1,911206569	0,458492743
1559109_a_at	VPS53	-1,452713826	-1,911206569	0,458492743

242396_at	LOC644192	-1,452713826	-1,911206569	0,458492743
1558523_at	FAM184A	-1,452713826	-1,911206569	0,458492743
1557664_at	LOC340239	-1,452713826	-1,911206569	0,458492743
235820_at	ERC1	-1,452713826	-1,911206569	0,458492743
228893_at	-	-1,452713826	-1,911206569	0,458492743
216808_at	MIR622	-1,452713826	-1,911206569	0,458492743
220621_at	FOXE3	-1,452713826	-1,911206569	0,458492743
240151_at	HOXB-AS3	-1,452713826	-1,911206569	0,458492743
244530_at	-	-1,452713826	-1,911206569	0,458492743
1563062_at	LINC00460	-1,452713826	-1,911206569	0,458492743
211163_s_at	TNFRSF10C	-1,452713826	-1,911206569	0,458492743
220932_at	-	-1,452713826	-1,911206569	0,458492743
242954_at	-	-1,452713826	-1,911206569	0,458492743
211562_s_at	LMOD1	-1,452713826	-1,911206569	0,458492743
1564514_at	SNRK	-1,452713826	-1,911206569	0,458492743
1552769_at	ZNF625	-1,452713826	-1,911206569	0,458492743
205439_at	GSTT2	-1,452713826	-1,911206569	0,458492743
231163_at	C1orf111	-1,452713826	-1,911206569	0,458492743
1566218_at	KRTAP5-2	-1,452713826	-1,911206569	0,458492743
210518_at	CDH8	-1,452713826	-1,911206569	0,458492743
224822_at	DLC1	-1,452713826	-1,911206569	0,458492743
237196_at	LOC100506558 ///	-1,452713826	-1,911206569	0,458492743
228055_at	NAPSB	-1,452713826	-1,911206569	0,458492743
1567255_at	OR10D1P	0,167727503	-0,290694975	0,458422478
232167_at	SLC2A11	0,167727503	-0,290694975	0,458422478
217807_s_at	GLTSCR2	7,189952435	6,731976212	0,457976222
228273_at	PRR11	6,525932318	6,067994833	0,457937484
239417_x_at	C6orf52	0,764143511	0,306454867	0,457688644
227660_at	ANTXR1	0,764143511	0,306454867	0,457688644
1569673_at	-	-1,06282519	-1,520022123	0,457196934
239989_at	CNTLN	-1,06282519	-1,520022123	0,457196934
244399_at	-	-1,06282519	-1,520022123	0,457196934
204724_s_at	COL9A3	-1,06282519	-1,520022123	0,457196934
231760_at	LINC00029	-1,06282519	-1,520022123	0,457196934
240074_at	-	-1,06282519	-1,520022123	0,457196934
241522_at	-	-1,06282519	-1,520022123	0,457196934
215163_at	-	-1,06282519	-1,520022123	0,457196934
1566739_at	PLCE1	-1,06282519	-1,520022123	0,457196934
1560065_at	PAIP2	-1,06282519	-1,520022123	0,457196934
1552857_a_at	HTR6	-1,06282519	-1,520022123	0,457196934
1553215_s_at	CCDC7	-1,06282519	-1,520022123	0,457196934
234599_at	-	-1,06282519	-1,520022123	0,457196934
229760_at	VEPH1	-1,06282519	-1,520022123	0,457196934
1559864_at	LCN6	-1,06282519	-1,520022123	0,457196934
220677_s_at	ADAMTS8	-1,06282519	-1,520022123	0,457196934
1553458_at	VSTM4	-1,06282519	-1,520022123	0,457196934
234946_at	ENTPD6	-1,06282519	-1,520022123	0,457196934
233802_at	-	-1,06282519	-1,520022123	0,457196934
228761_at	SCRT1	-1,06282519	-1,520022123	0,457196934
204854_at	LEPREL2	-1,06282519	-1,520022123	0,457196934

1555107_a_at	UVSSA	-1,06282519	-1,520022123	0,457196934
208429_x_at	HNF4A	-1,06282519	-1,520022123	0,457196934
222241_at	-	-1,06282519	-1,520022123	0,457196934
203383_s_at	GOLGA1	1,686984721	1,2298509	0,457133821
225724_at	FLJ31306	1,948628481	1,491644074	0,456984407
202155_s_at	NUP214	2,179708195	1,722747778	0,456960417
216443_at	-	-1,893506789	-2,350444759	0,45693797
222947_at	LOC100379224	-1,893506789	-2,350444759	0,45693797
220435_at	SLC30A10	-1,893506789	-2,350444759	0,45693797
207220_at	ART4	-1,893506789	-2,350444759	0,45693797
227794_at	GLYATL1 /// LOC1(	-1,893506789	-2,350444759	0,45693797
210147_at	ART3	-1,893506789	-2,350444759	0,45693797
1561078_at	-	-1,893506789	-2,350444759	0,45693797
205765_at	CYP3A5	-1,893506789	-2,350444759	0,45693797
1553672_at	ENAH	-1,893506789	-2,350444759	0,45693797
219436_s_at	EMCN	-1,893506789	-2,350444759	0,45693797
201150_s_at	TIMP3	-1,893506789	-2,350444759	0,45693797
1560834_a_at	RMST	-1,893506789	-2,350444759	0,45693797
236378_at	CIB4	-1,893506789	-2,350444759	0,45693797
233365_at	-	-1,893506789	-2,350444759	0,45693797
230067_at	FAM124A	-1,893506789	-2,350444759	0,45693797
1560025_at	-	-1,893506789	-2,350444759	0,45693797
206376_at	SLC6A15	-1,893506789	-2,350444759	0,45693797
1567064_at	OR1Q1	-1,893506789	-2,350444759	0,45693797
240225_at	-	-1,893506789	-2,350444759	0,45693797
235356_at	NHLRC2	-1,893506789	-2,350444759	0,45693797
221209_s_at	OTOR	-1,893506789	-2,350444759	0,45693797
221719_s_at	LZTS1	-1,893506789	-2,350444759	0,45693797
229669_at	LOC100507263	-1,893506789	-2,350444759	0,45693797
227237_x_at	ATAD3B	2,523100136	2,066329863	0,456770273
213224_s_at	BBIP1	2,199941239	1,743236669	0,456704569
224935_at	EIF2S3	5,758208547	5,30195934	0,456249206
1558431_at	NHLRC4	1,024284941	0,568155442	0,456129499
212034_s_at	EXOC7	2,62869474	2,173028091	0,455666648
209625_at	PIGH	1,510970097	1,055317091	0,455653006
218360_at	RAB22A	2,99543179	2,539809146	0,455622644
201597_at	COX7A2	6,98144441	6,525938463	0,455505948
34408_at	RTN2	1,356226003	0,900895716	0,455330287
224612_s_at	DNAJC5	1,578719607	1,123905343	0,454814264
224884_at	AKAP13	0,95222556	0,497424714	0,454800846
214317_x_at	RPS9	6,656486235	6,201938213	0,454548022
1558876_at	-	0,39105295	-0,063377083	0,454430033
237555_at	CARS2	0,39105295	-0,063377083	0,454430033
211392_s_at	PATZ1	0,39105295	-0,063377083	0,454430033
225276_at	GSPT1	2,685337919	2,23115548	0,454182439
237919_at	RFFL	0,820160788	0,366031214	0,454129574
1553900_s_at	LOC100287541 ///	-0,280273599	-0,734355396	0,454081797
1565862_a_at	-	-0,280273599	-0,734355396	0,454081797
215019_x_at	ZNF528	-0,280273599	-0,734355396	0,454081797
211486_s_at	KCNQ2	-0,280273599	-0,734355396	0,454081797

222096_x_at	LOC100507284	-0,280273599	-0,734355396	0,454081797
213108_at	CAMK2A	-0,280273599	-0,734355396	0,454081797
241533_at	LOC731656	-0,280273599	-0,734355396	0,454081797
202043_s_at	SMS	5,050523265	4,596446615	0,45407665
211280_s_at	NRF1	0,491906512	0,03784737	0,454059141
232662_x_at	FAM213A	0,491906512	0,03784737	0,454059141
1552689_at	CASKIN1	0,491906512	0,03784737	0,454059141
221863_at	MIER2	0,491906512	0,03784737	0,454059141
202597_at	IRF6	-0,135065865	-0,589001171	0,453935306
231412_at	LOC100506342	-0,135065865	-0,589001171	0,453935306
235662_at	-	-0,135065865	-0,589001171	0,453935306
236780_at	-	-0,135065865	-0,589001171	0,453935306
229135_at	FASTKD2	-0,135065865	-0,589001171	0,453935306
231998_at	SART1	-0,135065865	-0,589001171	0,453935306
209429_x_at	EIF2B4	3,547953548	3,094388414	0,453565134
209227_at	TUSC3	-2,495152701	-2,948507524	0,453354823
1553326_at	RXFP2	-1,212652659	-1,665791776	0,453139117
220321_s_at	CCDC121	-1,212652659	-1,665791776	0,453139117
220888_s_at	CASS4	-1,212652659	-1,665791776	0,453139117
218990_s_at	SPRR3	-1,212652659	-1,665791776	0,453139117
231592_at	TSIX	-1,212652659	-1,665791776	0,453139117
1555342_a_at	UNC5C	-1,212652659	-1,665791776	0,453139117
243396_at	-	-1,212652659	-1,665791776	0,453139117
241064_at	-	-1,212652659	-1,665791776	0,453139117
1562786_at	-	-1,212652659	-1,665791776	0,453139117
1564017_at	COL18A1-AS1	-1,212652659	-1,665791776	0,453139117
210295_at	MAGEA10	-1,212652659	-1,665791776	0,453139117
221231_s_at	C14orf102	-1,212652659	-1,665791776	0,453139117
217340_at	-	-1,212652659	-1,665791776	0,453139117
224324_at	MRO	-1,212652659	-1,665791776	0,453139117
1564426_x_at	LOC729732	-1,212652659	-1,665791776	0,453139117
1556593_s_at	-	-1,212652659	-1,665791776	0,453139117
1556735_at	-	-1,212652659	-1,665791776	0,453139117
217298_at	RPS14	-1,212652659	-1,665791776	0,453139117
216365_x_at	IGLC1 /// IGLJ3	-1,212652659	-1,665791776	0,453139117
1567036_at	C20orf181	-1,212652659	-1,665791776	0,453139117
221249_s_at	FAM117A	3,846827633	3,393801804	0,453025829
41856_at	UNC5B	1,126016682	0,673496747	0,452519935
228817_at	ALG9	1,701542258	1,249194639	0,452347619
222034_at	GNB2L1 /// LOC10	1,701542258	1,249194639	0,452347619
204979_s_at	SH3BGR	1,225123479	0,772793615	0,452329864
239225_at	-	1,225123479	0,772793615	0,452329864
206274_s_at	CROCC	0,001114523	-0,45121326	0,452327782
1553720_a_at	FAM123A	0,001114523	-0,45121326	0,452327782
230368_at	ERF	0,001114523	-0,45121326	0,452327782
229712_at	SNAPC3	0,001114523	-0,45121326	0,452327782
201087_at	PXN	0,001114523	-0,45121326	0,452327782
209250_at	DEGS1	5,501100021	5,048787948	0,452312073
235347_at	LRCH3	1,730929079	1,278654284	0,452274795
228255_at	TMEM237	1,267146969	0,815200271	0,451946698

237137_at	SCARNA2	-0,437449947	-0,889222211	0,451772263
224633_s_at	GPATCH4	-0,437449947	-0,889222211	0,451772263
231427_at	LOC284648	-0,437449947	-0,889222211	0,451772263
227000_at	C7orf41	-0,437449947	-0,889222211	0,451772263
211403_x_at	VCX2	-0,437449947	-0,889222211	0,451772263
225198_at	VAPA	3,628556015	3,176868665	0,45168735
216044_x_at	FAM69A	2,739067176	2,287674614	0,451392562
226046_at	MAPK8	1,910557155	1,459545271	0,451011884
200844_s_at	PRDX6	6,087616388	5,636827089	0,450789299
224960_at	SCYL2	4,281531373	3,830847157	0,450684216
217491_x_at	COX7C	7,08510634	6,634440906	0,450665434
217915_s_at	RSL24D1	5,446817871	4,996158676	0,450659195
224535_s_at	MRP63	2,949541692	2,498884538	0,450657154
236093_at	-	0,127732831	-0,322340048	0,450072878
211279_at	NRF1	0,127732831	-0,322340048	0,450072878
1568699_at	IFT43	0,127732831	-0,322340048	0,450072878
216371_at	-	0,127732831	-0,322340048	0,450072878
238944_at	LOC100505715	0,127732831	-0,322340048	0,450072878
202617_s_at	MECP2	0,127732831	-0,322340048	0,450072878
221094_s_at	ELP3	2,560606634	2,110584371	0,450022263
242082_at	MMAB	1,116462765	0,666486137	0,449976627
229202_at	-	2,476788561	2,026957145	0,449831415
202001_s_at	NDUFA6	5,811684132	5,362301381	0,449382751
227357_at	TAB3	3,509819135	3,060577077	0,449242058
210927_x_at	JTB	6,061711545	5,612472372	0,449239173
227216_at	RLTPR	0,873789652	0,424693306	0,449096346
1565611_at	DYNLRB1	0,873789652	0,424693306	0,449096346
210293_s_at	SEC23B	4,404121066	3,955384072	0,448736994
235268_at	-	-0,924000698	-1,372734086	0,448733388
1555175_a_at	PBLD	-0,924000698	-1,372734086	0,448733388
235016_at	REEP3	-0,924000698	-1,372734086	0,448733388
235311_at	FKBP14	-0,924000698	-1,372734086	0,448733388
1552289_a_at	CILP2	-0,924000698	-1,372734086	0,448733388
227503_at	-	-0,924000698	-1,372734086	0,448733388
1563063_at	-	-0,924000698	-1,372734086	0,448733388
213030_s_at	PLXNA2	-0,924000698	-1,372734086	0,448733388
1554157_a_at	WFDC8	-0,924000698	-1,372734086	0,448733388
1556122_at	RAB11B-AS1	-0,924000698	-1,372734086	0,448733388
243590_at	-	-0,924000698	-1,372734086	0,448733388
227667_at	CUEDC1	-0,924000698	-1,372734086	0,448733388
202555_s_at	MYLK	-0,924000698	-1,372734086	0,448733388
1554554_at	CCDC57	-0,924000698	-1,372734086	0,448733388
215104_at	NRIP2	-0,924000698	-1,372734086	0,448733388
241143_at	-	-0,924000698	-1,372734086	0,448733388
204920_at	CPS1	-0,924000698	-1,372734086	0,448733388
1552275_s_at	PXK	2,698955954	2,250228409	0,448727545
224784_at	MLLT6	2,348178434	1,899778623	0,448399811
231178_at	SPATA4	-0,609040214	-1,057301851	0,448261637
244263_at	-	-0,609040214	-1,057301851	0,448261637
1552718_at	DTD1	-0,609040214	-1,057301851	0,448261637

232828_at	NALCN-AS1	-0,609040214	-1,057301851	0,448261637
237852_at	-	-0,609040214	-1,057301851	0,448261637
1567440_at	PSEN1	-0,609040214	-1,057301851	0,448261637
233634_at	MARVELD3	-0,609040214	-1,057301851	0,448261637
210420_at	SLC24A1	-0,609040214	-1,057301851	0,448261637
234927_s_at	MAGIX	-0,609040214	-1,057301851	0,448261637
243917_at	CLIC5	-0,609040214	-1,057301851	0,448261637
205467_at	CASP10	-0,609040214	-1,057301851	0,448261637
243881_at	SHC3	-0,609040214	-1,057301851	0,448261637
209270_at	LAMB3	-0,609040214	-1,057301851	0,448261637
225501_at	PHF6	4,64066268	4,192491623	0,448171057
226479_at	KBTBD6	1,995527416	1,547376668	0,448150748
222497_x_at	NMD3	4,626492955	4,178345555	0,4481474
224474_x_at	SMEK2	2,884644843	2,436714502	0,44793034
212274_at	LPIN1	4,953144142	4,505298548	0,447845594
51226_at	-	-1,757023018	-2,204738503	0,447715485
225825_at	C20orf194	0,648195588	0,20062106	0,447574528
229323_at	LOC387723	1,625482993	1,178100917	0,447382076
208786_s_at	MAP1LC3B	4,301645306	3,854386399	0,447258907
209397_at	ME2	5,196271767	4,749086149	0,447185618
1559220_at	-	1,047341799	0,600385088	0,446956711
232786_at	COG6	-1,982324124	-2,4284334	0,446109276
231424_at	SLC5A12	-1,982324124	-2,4284334	0,446109276
236391_at	-	-1,982324124	-2,4284334	0,446109276
231033_at	-	-1,982324124	-2,4284334	0,446109276
206142_at	ZNF135	-1,982324124	-2,4284334	0,446109276
1558394_s_at	KRT7	-1,982324124	-2,4284334	0,446109276
1555173_at	STX19	-1,982324124	-2,4284334	0,446109276
210163_at	CXCL11	-1,982324124	-2,4284334	0,446109276
237135_at	-	-1,982324124	-2,4284334	0,446109276
1563427_at	-	-1,982324124	-2,4284334	0,446109276
1569603_at	-	-1,982324124	-2,4284334	0,446109276
1560315_at	GLCCI1	-1,982324124	-2,4284334	0,446109276
241507_x_at	-	-1,982324124	-2,4284334	0,446109276
216087_at	-	-1,982324124	-2,4284334	0,446109276
206104_at	ISL1	-1,982324124	-2,4284334	0,446109276
242547_at	-	-1,982324124	-2,4284334	0,446109276
238088_at	-	-1,982324124	-2,4284334	0,446109276
244076_at	KIAA2022	-1,982324124	-2,4284334	0,446109276
1565650_at	-	-1,982324124	-2,4284334	0,446109276
234334_s_at	LAMB4	-1,982324124	-2,4284334	0,446109276
1557004_at	LOC100505902 ///	-1,982324124	-2,4284334	0,446109276
223829_at	TKTL2	-1,982324124	-2,4284334	0,446109276
1556700_a_at	-	-1,982324124	-2,4284334	0,446109276
243460_at	-	-1,982324124	-2,4284334	0,446109276
207497_s_at	MS4A2	-1,982324124	-2,4284334	0,446109276
229130_at	-	-1,982324124	-2,4284334	0,446109276
1567237_at	OR2L2	-1,982324124	-2,4284334	0,446109276
221168_at	PRDM13	2,878891456	2,432792627	0,446098829
221787_at	C6orf120	3,096793374	2,65087728	0,445916094

39817_s_at	C6orf108	3,386105376	2,940342062	0,445763314
225671_at	SPNS2	0,458905032	0,013190398	0,445714634
203829_at	ELP4	3,35759121	2,912181979	0,445409231
1559629_at	-	0,355751005	-0,089635713	0,445386718
212319_at	SGSM2	0,355751005	-0,089635713	0,445386718
238794_at	SFR1	0,355751005	-0,089635713	0,445386718
202021_x_at	EIF1	7,162969647	6,71759687	0,445372777
200009_at	GDI2	6,792035235	6,346663328	0,445371907
206492_at	FHIT	1,246401134	0,80114751	0,445253624
1558027_s_at	PRKAB2	1,287793882	0,842599219	0,445194663
219822_at	MTRF1	2,374261973	1,929092307	0,445169666
1566324_a_at	MAF	-2,250580859	-2,695640852	0,445059993
221177_at	MIA2	-2,250580859	-2,695640852	0,445059993
1552849_at	C2orf65	-2,250580859	-2,695640852	0,445059993
1567380_at	-	-2,250580859	-2,695640852	0,445059993
1570506_at	-	-2,250580859	-2,695640852	0,445059993
1553620_at	TRIM42	-2,250580859	-2,695640852	0,445059993
235691_at	-	-2,250580859	-2,695640852	0,445059993
1560962_at	-	-2,250580859	-2,695640852	0,445059993
238751_at	-	-2,250580859	-2,695640852	0,445059993
223185_s_at	BHLHE41	-2,250580859	-2,695640852	0,445059993
1560383_at	LOC100506387	-2,250580859	-2,695640852	0,445059993
1557739_at	-	-2,250580859	-2,695640852	0,445059993
217319_x_at	CYP4A22	-2,250580859	-2,695640852	0,445059993
237556_at	-	-2,250580859	-2,695640852	0,445059993
1569078_at	LOC100294362	-2,250580859	-2,695640852	0,445059993
225733_at	B3GALT6	1,936380191	1,491644074	0,444736118
211955_at	IPO5	4,350356009	3,905727379	0,44462863
200676_s_at	UBE2L3	2,074841895	1,630734622	0,444107274
223774_at	SNHG12 /// SNOR/	3,9635104	3,519587056	0,443923344
242282_at	ZFPM1	1,094118704	0,65030602	0,443812684
203518_at	LYST	2,908690075	2,465016194	0,443673881
224639_at	SPPL3	3,517785666	3,074131603	0,443654063
201826_s_at	SCCPDH	2,248155141	1,804519951	0,443635191
200817_x_at	RPS10	8,46785403	8,02427025	0,44358378
1560404_a_at	ATPBD4	-2,336678463	-2,780225148	0,443546684
231114_at	SPATA22	-2,336678463	-2,780225148	0,443546684
224017_at	TBX22	-2,336678463	-2,780225148	0,443546684
240285_at	AQP2	-2,336678463	-2,780225148	0,443546684
239543_s_at	-	-2,336678463	-2,780225148	0,443546684
236024_at	GPM6A	-0,791294935	-1,234835326	0,443540391
232005_at	DNAH1	-0,791294935	-1,234835326	0,443540391
206105_at	AFF2	-0,791294935	-1,234835326	0,443540391
232844_at	IFT140	-0,791294935	-1,234835326	0,443540391
1562324_a_at	-	-0,791294935	-1,234835326	0,443540391
1553846_at	SPERT	-0,791294935	-1,234835326	0,443540391
234624_at	-	-0,791294935	-1,234835326	0,443540391
1568997_at	POLR1E	-0,791294935	-1,234835326	0,443540391
201217_x_at	RNU86 /// RPL3 //.	8,302336075	7,858836223	0,443499852
230502_s_at	-	0,97663406	0,533258442	0,443375618

1560451_at	-	0,97663406	0,533258442	0,443375618
218071_s_at	MKRN2	4,195854652	3,7524971	0,443357552
225475_at	MIER1	3,461795772	3,019022994	0,442772778
36830_at	MIPEP	1,421504372	0,97898895	0,442515422
201949_x_at	CAPZB	5,291057394	4,848562131	0,442495263
203912_s_at	DNASE1L1	1,402495885	0,960042218	0,442453666
205361_s_at	PFDN4	4,510408465	4,068057231	0,442351234
1562263_at	LOC100507156	-0,180990326	-0,623254098	0,442263773
211894_x_at	SEZ6L	-0,180990326	-0,623254098	0,442263773
244108_at	SYNPO2	-0,180990326	-0,623254098	0,442263773
204713_s_at	F5	-0,180990326	-0,623254098	0,442263773
219866_at	CLIC5	-0,180990326	-0,623254098	0,442263773
213121_at	SNRNP70	-0,180990326	-0,623254098	0,442263773
243138_at	-	-0,180990326	-0,623254098	0,442263773
1555202_a_at	RPRD1A	-0,180990326	-0,623254098	0,442263773
232822_x_at	LOC100506757	-1,369772723	-1,811780055	0,442007332
1562351_at	-	-1,369772723	-1,811780055	0,442007332
238139_at	-	-1,369772723	-1,811780055	0,442007332
229714_at	HS6ST3	-1,369772723	-1,811780055	0,442007332
1569932_at	NHSL2	-1,369772723	-1,811780055	0,442007332
1554503_a_at	OSCAR	-1,369772723	-1,811780055	0,442007332
222954_at	FBXO40	-1,369772723	-1,811780055	0,442007332
216587_s_at	FZD8	-1,369772723	-1,811780055	0,442007332
222739_at	TMEM62	-1,369772723	-1,811780055	0,442007332
1556435_at	LOC400622	-1,369772723	-1,811780055	0,442007332
237325_at	C22orf43	-1,369772723	-1,811780055	0,442007332
207070_at	RGR	-1,369772723	-1,811780055	0,442007332
201616_s_at	CALD1	-1,369772723	-1,811780055	0,442007332
230071_at	40787	-1,369772723	-1,811780055	0,442007332
1560078_at	LAMA3	-1,369772723	-1,811780055	0,442007332
210569_s_at	SIGLEC9	-1,369772723	-1,811780055	0,442007332
1566195_at	-	-1,369772723	-1,811780055	0,442007332
1568249_at	LOC388796 /// SN	-1,369772723	-1,811780055	0,442007332
1567081_x_at	CLN6	0,847529938	0,405752839	0,441777099
227538_at	MED26	3,418470674	2,97688045	0,441590224
201875_s_at	MPZL1	1,364555912	0,923203038	0,441352874
218511_s_at	PNPO	3,257413106	2,81616114	0,441251967
223162_s_at	KIAA1147	3,340803206	2,899658146	0,44114506
208382_s_at	DMC1	0,08619576	-0,354781582	0,440977341
236709_at	-	0,08619576	-0,354781582	0,440977341
210736_x_at	DTNA	0,08619576	-0,354781582	0,440977341
211588_s_at	PML	0,08619576	-0,354781582	0,440977341
1555889_a_at	CRTAP /// LOC100	3,758020365	3,317257552	0,440762813
235739_at	-	-0,043925712	-0,484558493	0,440632782
232735_at	ANKRD34A	-0,043925712	-0,484558493	0,440632782
206614_at	GDF5	-0,043925712	-0,484558493	0,440632782
202193_at	LIMK2	1,948628481	1,508107415	0,440521065
220649_at	AGBL3	-2,163157732	-2,603471267	0,440313535
232049_at	-	-2,163157732	-2,603471267	0,440313535
215240_at	ITGB3	-2,163157732	-2,603471267	0,440313535

231617_at	TEX33	-2,163157732	-2,603471267	0,440313535
242925_at	RNF148	-2,163157732	-2,603471267	0,440313535
207149_at	CDH12	-2,163157732	-2,603471267	0,440313535
1561704_at	-	-2,163157732	-2,603471267	0,440313535
1560723_at	LOC283731	-2,163157732	-2,603471267	0,440313535
235044_at	CYYR1	-2,163157732	-2,603471267	0,440313535
234865_at	IL23A	-2,163157732	-2,603471267	0,440313535
206799_at	SCGB1D2	-2,163157732	-2,603471267	0,440313535
221060_s_at	TLR4	-2,163157732	-2,603471267	0,440313535
208293_x_at	CSHL1	-2,163157732	-2,603471267	0,440313535
1561590_a_at	LOC415056	-2,163157732	-2,603471267	0,440313535
211362_s_at	SERPINB13	-2,163157732	-2,603471267	0,440313535
1557944_s_at	CTNND1 /// TMX2	1,182925501	0,742671819	0,440253682
225323_at	CC2D1B	1,182925501	0,742671819	0,440253682
233411_at	-	0,618277321	0,178191865	0,440085456
1564964_at	-	-2,416625063	-2,856570137	0,439945074
243979_at	-	-2,416625063	-2,856570137	0,439945074
201859_at	SRGN	6,56374816	6,123938318	0,439809842
227378_x_at	C16orf13	2,896717728	2,456960019	0,439757708
40829_at	WDTC1	1,837362828	1,39800773	0,439355097
203733_at	DEXI	2,374261973	1,935144844	0,43911713
223336_s_at	RAB18	3,345120948	2,906008589	0,439112359
31807_at	DDX49	3,349906327	2,910800821	0,439105506
240561_at	LOC100652838	-0,332405896	-0,771340337	0,438934441
238578_at	TMEM182	-0,332405896	-0,771340337	0,438934441
239322_at	CEP41	-0,332405896	-0,771340337	0,438934441
221034_s_at	TEX13B	-0,332405896	-0,771340337	0,438934441
237042_at	LOC100507024	-0,332405896	-0,771340337	0,438934441
239602_at	LOC100652972	-0,332405896	-0,771340337	0,438934441
228315_at	ZMAT3	3,834426729	3,395519718	0,438907011
209628_at	NXT2	3,828857041	3,389993505	0,438863535
232900_at	HERC2P7	-0,49284695	-0,931539147	0,438692197
205175_s_at	KHK	-0,49284695	-0,931539147	0,438692197
217114_at	SNRNP200	-0,49284695	-0,931539147	0,438692197
205344_at	CSPG5	-0,49284695	-0,931539147	0,438692197
217149_x_at	TNK1	-0,49284695	-0,931539147	0,438692197
231877_at	TRMT10A	-0,49284695	-0,931539147	0,438692197
231407_s_at	FOXH1	-0,49284695	-0,931539147	0,438692197
229786_at	-	-0,49284695	-0,931539147	0,438692197
235806_at	ARAP3	-0,49284695	-0,931539147	0,438692197
207699_at	ZFHX2	-0,49284695	-0,931539147	0,438692197
209294_x_at	TNFRSF10B	-0,49284695	-0,931539147	0,438692197
206044_s_at	BRAF	0,900065578	0,461385738	0,43867984
230832_at	RTF1	0,20740896	-0,231045907	0,438454867
205019_s_at	VIPR1	0,20740896	-0,231045907	0,438454867
207844_at	IL13	0,20740896	-0,231045907	0,438454867
229878_at	KIAA1731	2,798156197	2,359892448	0,43826375
222581_at	XPR1	2,798156197	2,359892448	0,43826375
220367_s_at	SAP130	3,362258367	2,924357093	0,437901274
226586_at	ANKS6	1,267146969	0,829309537	0,437837433

218177_at	CHMP1B	1,686984721	1,249194639	0,437790082
212644_s_at	MAPK1IP1L	3,065977723	2,628204186	0,437773537
200773_x_at	PTMA	7,347278232	6,90967651	0,437601722
203379_at	RPS6KA1	1,960819106	1,523429058	0,437390048
203616_at	POLB	5,05583032	4,618481456	0,437348864
218401_s_at	ZNF281	3,03433073	2,597466271	0,436864459
222984_at	PAIP2	5,940250725	5,503396432	0,436854293
225640_at	LOC100506710	3,960533133	3,523783625	0,436749508
214173_x_at	URI1	4,955955491	4,519344963	0,436610528
225356_at	-	3,849373906	3,412768101	0,436605805
222620_s_at	DNAJC1	4,046717183	3,610508368	0,436208815
236400_at	IDH1-AS1	0,319287178	-0,116767475	0,436054653
1557690_x_at	-	0,319287178	-0,116767475	0,436054653
230887_at	CDC14B	0,319287178	-0,116767475	0,436054653
229465_s_at	-	0,319287178	-0,116767475	0,436054653
233357_at	TRIM67	0,425036312	-0,011001236	0,436037548
219095_at	JMJD7 /// JMJD7-F	0,425036312	-0,011001236	0,436037548
218608_at	ATP13A2	0,425036312	-0,011001236	0,436037548
218074_at	FAM96B	5,565534834	5,130022088	0,435512746
226316_at	RBM26	2,642918178	2,207440269	0,43547791
218828_at	C17orf61-PLSCR3 /	1,760255626	1,324949741	0,435305885
205408_at	MLLT10	1,910557155	1,475630127	0,434927028
217707_x_at	SMARCA2	4,409540371	3,974735953	0,434804419
216960_s_at	ZNF133	1,116462765	0,681874787	0,434587978
201989_s_at	CREBL2	3,758020365	3,323485083	0,434535282
221484_at	B4GALT5	3,639446351	3,205255599	0,434190751
224163_s_at	DMAP1	2,636121902	2,20251271	0,433609192
207508_at	ATP5G3	6,117765182	5,684316765	0,433448418
215157_x_at	PABPC1	8,41920719	7,985827786	0,433379404
213881_x_at	SUMO2	7,658421682	7,225357526	0,433064156
224910_at	CARHSP1	1,97249974	1,539450683	0,433049057
207145_at	MSTN	-2,495152701	-2,928183529	0,433030828
211227_s_at	PCDH11X /// PCDH	-2,495152701	-2,928183529	0,433030828
233682_at	-	-2,495152701	-2,928183529	0,433030828
1561110_at	-	-2,495152701	-2,928183529	0,433030828
1559956_at	SYT7	-2,495152701	-2,928183529	0,433030828
200594_x_at	HNRNPU	6,088644787	5,655804314	0,432840473
218011_at	UBL5	5,606731501	5,173898758	0,432832743
1569579_at	-	0,045517965	-0,386896102	0,432414067
207203_s_at	NR1I2	0,045517965	-0,386896102	0,432414067
230623_x_at	USP28	1,544940605	1,112548615	0,43239199
204209_at	PCYT1A	1,510970097	1,078619386	0,432350711
222333_at	ALS2CL	1,000459215	0,568155442	0,432303772
201552_at	LAMP1	3,906843271	3,474637573	0,432205697
200030_s_at	SLC25A3	7,432767711	7,000589529	0,432178182
224000_at	C2orf16	0,58788177	0,155747281	0,43213449
243684_at	-	-0,66934516	-1,100943374	0,431598215
231461_at	KRT71	-0,66934516	-1,100943374	0,431598215
242213_at	-	-0,66934516	-1,100943374	0,431598215
237576_x_at	LOC100506480	-0,66934516	-1,100943374	0,431598215

212743_at	RCHY1	-0,66934516	-1,100943374	0,431598215
211633_x_at	-	-0,66934516	-1,100943374	0,431598215
211329_x_at	HFE	-0,66934516	-1,100943374	0,431598215
203237_s_at	NOTCH3	-0,66934516	-1,100943374	0,431598215
1558581_at	TEX22	-0,66934516	-1,100943374	0,431598215
204948_s_at	FST	-0,66934516	-1,100943374	0,431598215
238912_x_at	C9orf85	3,505636113	3,074131603	0,43150451
226221_at	KIAA1432	2,791258167	2,359892448	0,431365719
1559352_a_at	MAFG-AS1	1,287793882	0,85655369	0,431240192
209998_at	PIGO	1,246401134	0,815200271	0,431200863
240869_at	-	-1,137208348	-1,568385657	0,431177308
234835_at	LOC100506667	-1,137208348	-1,568385657	0,431177308
208261_x_at	IFNA10	-1,137208348	-1,568385657	0,431177308
214646_at	HIST1H3A /// HIST	-1,137208348	-1,568385657	0,431177308
231072_at	MIDN	-1,137208348	-1,568385657	0,431177308
239610_at	-	-1,137208348	-1,568385657	0,431177308
237542_at	-	-1,137208348	-1,568385657	0,431177308
230514_s_at	LYZL1 /// LYZL2	-1,137208348	-1,568385657	0,431177308
236397_at	-	-1,137208348	-1,568385657	0,431177308
1570531_at	-	-1,137208348	-1,568385657	0,431177308
1558780_a_at	H2AFY	-1,137208348	-1,568385657	0,431177308
1562478_at	LOC100652730 ///	-1,137208348	-1,568385657	0,431177308
234737_at	NT5DC3	-1,137208348	-1,568385657	0,431177308
1558118_at	LOC100287576 ///	-1,137208348	-1,568385657	0,431177308
236060_at	-	-1,137208348	-1,568385657	0,431177308
1560141_at	LOC100133039	-1,137208348	-1,568385657	0,431177308
1557394_at	DLGAP4	-1,137208348	-1,568385657	0,431177308
222272_x_at	SCIN	-1,137208348	-1,568385657	0,431177308
216496_s_at	-	-1,137208348	-1,568385657	0,431177308
207662_at	TBX1	-1,137208348	-1,568385657	0,431177308
229911_at	TPM2	-1,137208348	-1,568385657	0,431177308
236389_x_at	-	-1,137208348	-1,568385657	0,431177308
1558647_at	SH3D19	-1,137208348	-1,568385657	0,431177308
1569246_a_at	C8orf74	-1,137208348	-1,568385657	0,431177308
220032_at	CPED1	-1,137208348	-1,568385657	0,431177308
239700_at	ZNF710	-1,137208348	-1,568385657	0,431177308
206297_at	CTRC	-1,137208348	-1,568385657	0,431177308
203441_s_at	CDH2	-1,137208348	-1,568385657	0,431177308
243382_at	CCDC85A	-1,137208348	-1,568385657	0,431177308
211535_s_at	FGFR1	3,402275492	2,971193817	0,431081674
221782_at	DNAJC10	2,248155141	1,817403386	0,430751755
223760_s_at	-	-0,230432956	-0,661178575	0,430745619
214239_x_at	PCGF2	-0,230432956	-0,661178575	0,430745619
203364_s_at	ATG13	2,613382867	2,182711118	0,430671749
226661_at	CDCA2	4,016622037	3,586015261	0,430606776
229983_at	TIGD2	2,118167848	1,687645398	0,43052245
225433_at	GTF2A1	3,614400403	3,18412401	0,430276393
213568_at	OSR2	0,491906512	0,061773582	0,43013293
200801_x_at	ACTB /// LOC1005(	8,003392494	7,573637106	0,429755388
228822_s_at	USP16	3,945594088	3,515907973	0,429686115

240658_at	-	-1,712732543	-2,142286822	0,429554279
1570622_at	-	-1,712732543	-2,142286822	0,429554279
220402_at	TP53AIP1	-1,712732543	-2,142286822	0,429554279
1569790_at	-	-1,712732543	-2,142286822	0,429554279
229560_at	TLR8	-1,712732543	-2,142286822	0,429554279
233312_at	ROPN1L	-1,712732543	-2,142286822	0,429554279
1559513_a_at	FANCC	-1,712732543	-2,142286822	0,429554279
244657_at	-	-1,712732543	-2,142286822	0,429554279
1555605_x_at	BAGE	-1,712732543	-2,142286822	0,429554279
234804_at	PROX2	-1,712732543	-2,142286822	0,429554279
240951_at	RORA	-1,712732543	-2,142286822	0,429554279
1565700_at	-	-1,712732543	-2,142286822	0,429554279
238301_at	-	-1,712732543	-2,142286822	0,429554279
1553319_at	OXGR1	-1,712732543	-2,142286822	0,429554279
210493_s_at	MFAP3L	-1,712732543	-2,142286822	0,429554279
1570116_at	-	-1,712732543	-2,142286822	0,429554279
1552552_s_at	CLEC4C	-1,712732543	-2,142286822	0,429554279
237657_at	-	-1,712732543	-2,142286822	0,429554279
1557727_at	PCBP1-AS1	-1,712732543	-2,142286822	0,429554279
1565816_at	-	-1,712732543	-2,142286822	0,429554279
232252_at	DUSP27	-1,712732543	-2,142286822	0,429554279
235915_at	-	-1,712732543	-2,142286822	0,429554279
1561585_at	RCBTB2	-1,712732543	-2,142286822	0,429554279
222454_s_at	PARVA	-1,712732543	-2,142286822	0,429554279
1561252_at	LOC648691	-1,712732543	-2,142286822	0,429554279
1553746_a_at	OTOGL	-1,712732543	-2,142286822	0,429554279
217177_s_at	PTPRB	-1,712732543	-2,142286822	0,429554279
1566778_at	-	-1,712732543	-2,142286822	0,429554279
213541_s_at	ERG	-1,712732543	-2,142286822	0,429554279
1559149_at	-	-1,712732543	-2,142286822	0,429554279
1556914_at	-	-1,712732543	-2,142286822	0,429554279
223749_at	C1QTNF2	-1,712732543	-2,142286822	0,429554279
1552777_a_at	RAET1E	-1,712732543	-2,142286822	0,429554279
235017_s_at	CSRNP3	-1,712732543	-2,142286822	0,429554279
241382_at	PCP4L1	-1,712732543	-2,142286822	0,429554279
240809_at	ZNF295-AS1	-1,712732543	-2,142286822	0,429554279
232255_at	LOC401321	-1,712732543	-2,142286822	0,429554279
244521_at	TSHZ2	-1,712732543	-2,142286822	0,429554279
236296_x_at	C8orf58	0,735999505	0,306454867	0,429544638
205021_s_at	FOXN3	1,047341799	0,61779983	0,429541969
207814_at	DEFA6	-0,088974936	-0,518232988	0,429258053
231132_at	-	-0,088974936	-0,518232988	0,429258053
239143_x_at	RNF138	2,484824532	2,055739171	0,429085361
206567_s_at	PHF20	3,257413106	2,828849722	0,428563384
209070_s_at	RGS5	0,167727503	-0,260526297	0,4282538
219309_at	C22orf46	0,167727503	-0,260526297	0,4282538
234436_x_at	OBP2A	0,167727503	-0,260526297	0,4282538
211684_s_at	DYNC1I2	4,504407021	4,076293142	0,428113879
238293_at	-	-0,994186487	-1,422257026	0,428070539
242113_at	-	-0,994186487	-1,422257026	0,428070539

240562_at	GSK3B	-0,994186487	-1,422257026	0,428070539
211551_at	EGFR	-0,994186487	-1,422257026	0,428070539
214781_at	-	-0,994186487	-1,422257026	0,428070539
237683_s_at	-	-0,994186487	-1,422257026	0,428070539
223927_at	PCDHB9	-0,994186487	-1,422257026	0,428070539
236320_at	CCDC17	-0,994186487	-1,422257026	0,428070539
233051_at	SLITRK2	-0,994186487	-1,422257026	0,428070539
210165_at	DNASE1	-0,994186487	-1,422257026	0,428070539
227838_at	UNC5C	-0,994186487	-1,422257026	0,428070539
1557148_at	HM13	-0,994186487	-1,422257026	0,428070539
242822_at	MGC39584	-0,994186487	-1,422257026	0,428070539
216448_at	-	-0,994186487	-1,422257026	0,428070539
232836_at	C20orf61	-0,994186487	-1,422257026	0,428070539
221302_at	KLF15	-0,994186487	-1,422257026	0,428070539
234886_at	-	-0,994186487	-1,422257026	0,428070539
208733_at	RAB2A	-0,994186487	-1,422257026	0,428070539
214746_s_at	ZNF467	-0,994186487	-1,422257026	0,428070539
200879_s_at	EPAS1 /// LOC100(	-0,994186487	-1,422257026	0,428070539
224411_at	PLA2G12B	-0,994186487	-1,422257026	0,428070539
243290_at	WWC1	-0,994186487	-1,422257026	0,428070539
207160_at	IL12A	-0,994186487	-1,422257026	0,428070539
212301_at	RTF1	2,860630824	2,432792627	0,427838198
226980_at	DEPDC1B	3,466076444	3,038411131	0,427665313
218392_x_at	SFXN1	3,614400403	3,186758158	0,427642245
202488_s_at	FXYD3	0,39105295	-0,036543687	0,427596637
231932_at	-	0,39105295	-0,036543687	0,427596637
220653_at	ZIM2	-0,384193355	-0,811769547	0,427576192
239633_at	-	-0,384193355	-0,811769547	0,427576192
1559977_a_at	SLC25A34	-0,384193355	-0,811769547	0,427576192
242168_at	NDUFS7	-0,384193355	-0,811769547	0,427576192
212318_at	TNPO3	4,613033266	4,185638086	0,42739518
221855_at	SDHAF1	2,692288672	2,264929589	0,427359082
214271_x_at	RPL12	7,951782133	7,52449895	0,427283184
1562750_at	-	-2,074223907	-2,501359113	0,427135206
1565525_a_at	TCP11L2	-2,074223907	-2,501359113	0,427135206
234194_at	-	-2,074223907	-2,501359113	0,427135206
1565925_at	SLC17A4	-2,074223907	-2,501359113	0,427135206
237939_at	EPHA5	-2,074223907	-2,501359113	0,427135206
1566265_at	-	-2,074223907	-2,501359113	0,427135206
244247_at	-	-2,074223907	-2,501359113	0,427135206
243728_at	LOC100505750	-2,074223907	-2,501359113	0,427135206
237282_s_at	AKAP14	-2,074223907	-2,501359113	0,427135206
237508_at	-	-2,074223907	-2,501359113	0,427135206
1560642_at	-	-2,074223907	-2,501359113	0,427135206
1561846_s_at	CCDC168	-2,074223907	-2,501359113	0,427135206
205876_at	LIFR	-2,074223907	-2,501359113	0,427135206
234230_at	-	-2,074223907	-2,501359113	0,427135206
1559152_at	-	-2,074223907	-2,501359113	0,427135206
206292_s_at	SULT2A1	-2,074223907	-2,501359113	0,427135206
223374_s_at	B3GALNT1	-2,074223907	-2,501359113	0,427135206

1555726_at	GAFA3	-2,074223907	-2,501359113	0,427135206
232731_x_at	LOC100190938	-2,074223907	-2,501359113	0,427135206
208500_x_at	FOXD3	-2,074223907	-2,501359113	0,427135206
232903_at	-	-2,074223907	-2,501359113	0,427135206
1567681_at	SNORA74A	-2,074223907	-2,501359113	0,427135206
216796_s_at	-	-2,074223907	-2,501359113	0,427135206
205239_at	AREG /// AREGB	-2,074223907	-2,501359113	0,427135206
228096_at	MINOS1	0,282358733	-0,144765583	0,427124316
219899_x_at	NDOR1	0,282358733	-0,144765583	0,427124316
221188_s_at	CIDEB	0,282358733	-0,144765583	0,427124316
213254_at	TNRC6B	0,97663406	0,550017041	0,426617019
227739_at	NDOR1	1,561816652	1,135235995	0,426580657
214358_at	ACACA	1,5944803	1,167919222	0,426561078
238237_at	LOC100130964	-1,802716385	-2,229233437	0,426517052
1560763_at	-	-1,802716385	-2,229233437	0,426517052
239336_at	THBS1	-1,802716385	-2,229233437	0,426517052
240167_at	LOC152742	-1,802716385	-2,229233437	0,426517052
233969_at	-	-1,802716385	-2,229233437	0,426517052
1553467_at	FLJ32742	-1,802716385	-2,229233437	0,426517052
240422_at	-	-1,802716385	-2,229233437	0,426517052
1560634_a_at	-	-1,802716385	-2,229233437	0,426517052
1552553_a_at	NLRC4	-1,802716385	-2,229233437	0,426517052
241888_at	-	-1,802716385	-2,229233437	0,426517052
1564235_at	LOC729866	-1,802716385	-2,229233437	0,426517052
244644_at	FAM9C	-1,802716385	-2,229233437	0,426517052
206930_at	GLYAT	-1,802716385	-2,229233437	0,426517052
227300_at	TMEM119	-1,802716385	-2,229233437	0,426517052
1562941_at	-	-1,802716385	-2,229233437	0,426517052
210398_x_at	FUT6	-1,802716385	-2,229233437	0,426517052
236878_at	-	-1,802716385	-2,229233437	0,426517052
1567390_at	-	-1,802716385	-2,229233437	0,426517052
244851_at	-	-1,802716385	-2,229233437	0,426517052
231398_at	SLC22A7	-1,802716385	-2,229233437	0,426517052
1565073_at	-	-1,802716385	-2,229233437	0,426517052
234625_at	-	-1,802716385	-2,229233437	0,426517052
235719_at	CYP4V2	-1,802716385	-2,229233437	0,426517052
1566984_at	-	-1,802716385	-2,229233437	0,426517052
239399_at	-	-1,802716385	-2,229233437	0,426517052
214521_at	HES2	-1,802716385	-2,229233437	0,426517052
240693_at	-	-1,802716385	-2,229233437	0,426517052
1554712_a_at	GLYATL2	-1,802716385	-2,229233437	0,426517052
219466_s_at	APOA2	-1,802716385	-2,229233437	0,426517052
211124_s_at	KITLG	-1,802716385	-2,229233437	0,426517052
210511_s_at	INHBA	-1,802716385	-2,229233437	0,426517052
211948_x_at	PRRC2C	3,798440091	3,372163924	0,426276167
1562440_at	MAP3K13	-0,856259172	-1,282520722	0,42626155
223825_at	KIAA1432	-0,856259172	-1,282520722	0,42626155
238080_at	B4GALNT4	-0,856259172	-1,282520722	0,42626155
202017_at	EPHX1	-0,856259172	-1,282520722	0,42626155
210197_at	ITPK1	-0,856259172	-1,282520722	0,42626155

230727_at	CISD3	-0,856259172	-1,282520722	0,42626155
234951_s_at	COL12A1	-0,856259172	-1,282520722	0,42626155
1555766_a_at	GNG2	-0,856259172	-1,282520722	0,42626155
217303_s_at	ADRB3	-0,856259172	-1,282520722	0,42626155
205368_at	FAM131B	-0,856259172	-1,282520722	0,42626155
225373_at	C10orf54	-0,856259172	-1,282520722	0,42626155
1566452_at	-	-0,856259172	-1,282520722	0,42626155
236812_at	STMN4	-0,856259172	-1,282520722	0,42626155
226233_at	B3GALNT2	3,525566827	3,099629381	0,425937446
208941_s_at	SEPHS1	3,362258367	2,936370826	0,425887541
209877_at	SNCG	0,791908897	0,366031214	0,425877684
1556464_a_at	C2orf72	0,791908897	0,366031214	0,425877684
238685_at	STXBP5-AS1	-1,289805289	-1,715576125	0,425770836
220001_at	PADI4	-1,289805289	-1,715576125	0,425770836
211821_x_at	GYPA	-1,289805289	-1,715576125	0,425770836
206732_at	SLITRK3	-1,289805289	-1,715576125	0,425770836
1566503_at	-	-1,289805289	-1,715576125	0,425770836
216489_at	TRPM3	-1,289805289	-1,715576125	0,425770836
230984_s_at	-	-1,289805289	-1,715576125	0,425770836
224054_at	-	-1,289805289	-1,715576125	0,425770836
230096_at	-	-1,289805289	-1,715576125	0,425770836
215254_at	RCAN1	-1,289805289	-1,715576125	0,425770836
1566837_at	-	-1,289805289	-1,715576125	0,425770836
233889_at	TBX18	-1,289805289	-1,715576125	0,425770836
223893_at	ENAM	-1,289805289	-1,715576125	0,425770836
1560212_a_at	-	-1,289805289	-1,715576125	0,425770836
231632_at	C19orf69	-1,289805289	-1,715576125	0,425770836
229020_x_at	EPS8L2	-1,289805289	-1,715576125	0,425770836
227362_at	SLC2A4RG	-1,289805289	-1,715576125	0,425770836
208276_at	-	-1,289805289	-1,715576125	0,425770836
226777_at	ADAM12	-1,289805289	-1,715576125	0,425770836
216649_at	RREB1	-1,289805289	-1,715576125	0,425770836
238369_s_at	LOC100507186	-1,289805289	-1,715576125	0,425770836
230835_at	KRTDAP	-1,289805289	-1,715576125	0,425770836
243499_at	-	-1,289805289	-1,715576125	0,425770836
209872_s_at	PKP3	-1,289805289	-1,715576125	0,425770836
236508_at	SLC6A5	-1,289805289	-1,715576125	0,425770836
1557007_a_at	-	-1,289805289	-1,715576125	0,425770836
238194_at	DGKK	-1,289805289	-1,715576125	0,425770836
235972_at	TMEM131	-1,289805289	-1,715576125	0,425770836
244254_at	-	-1,289805289	-1,715576125	0,425770836
222043_at	CLU	-1,289805289	-1,715576125	0,425770836
220437_at	C19orf80	-1,289805289	-1,715576125	0,425770836
210252_s_at	MADD	2,401508182	1,975797592	0,42571059
209284_s_at	FAM208A	3,834426729	3,408745454	0,425681275
64371_at	SUGP2	1,484853791	1,059201863	0,425651928
37012_at	CAPZB	5,773426682	5,347946326	0,425480356
206703_at	CHRNB1	1,182925501	0,757638486	0,425287015
205135_s_at	NUFIP1	2,229377375	1,804519951	0,424857425
206464_at	BMX	-1,624955693	-2,049747196	0,424791503

1556368_at	PHKG2	-1,624955693	-2,049747196	0,424791503
1562142_at	SKIV2L2	-1,624955693	-2,049747196	0,424791503
244058_at	VSTM4	-1,624955693	-2,049747196	0,424791503
1564056_at	-	-1,624955693	-2,049747196	0,424791503
207445_s_at	CCR9	-1,624955693	-2,049747196	0,424791503
231304_at	PPP3R2	-1,624955693	-2,049747196	0,424791503
1560512_at	-	-1,624955693	-2,049747196	0,424791503
217375_at	-	-1,624955693	-2,049747196	0,424791503
238671_at	EPHA4	-1,624955693	-2,049747196	0,424791503
242708_at	-	-1,624955693	-2,049747196	0,424791503
219989_s_at	ANKS1B	-1,624955693	-2,049747196	0,424791503
208495_at	TLX3	-1,624955693	-2,049747196	0,424791503
208244_at	BMP3	-1,624955693	-2,049747196	0,424791503
1560162_at	FLJ40606	-1,624955693	-2,049747196	0,424791503
244240_at	-	-1,624955693	-2,049747196	0,424791503
232920_at	CCDC157	-1,624955693	-2,049747196	0,424791503
206899_at	NTSR2	-1,624955693	-2,049747196	0,424791503
208235_x_at	GAGE12F /// GAGE	-1,624955693	-2,049747196	0,424791503
1561141_at	-	-1,624955693	-2,049747196	0,424791503
232193_at	GSTT1	-1,624955693	-2,049747196	0,424791503
208291_s_at	TH	-1,624955693	-2,049747196	0,424791503
1553491_at	KSR2	-1,624955693	-2,049747196	0,424791503
220082_at	PPP1R14D	-1,624955693	-2,049747196	0,424791503
214013_s_at	TBC1D1	-1,624955693	-2,049747196	0,424791503
1556156_at	ESRRB	-1,624955693	-2,049747196	0,424791503
203948_s_at	MPO	-1,624955693	-2,049747196	0,424791503
217404_s_at	COL2A1	-1,624955693	-2,049747196	0,424791503
219788_at	PILRA	-1,624955693	-2,049747196	0,424791503
244787_at	-	-1,624955693	-2,049747196	0,424791503
216416_at	-	-1,624955693	-2,049747196	0,424791503
210289_at	NAT8 /// NAT8B	-1,624955693	-2,049747196	0,424791503
210118_s_at	IL1A	-1,624955693	-2,049747196	0,424791503
200804_at	TMBIM6	6,451848093	6,027680935	0,424167157
218438_s_at	MED28	4,036242149	3,612221266	0,424020883
205804_s_at	TRAF3IP3	3,204847647	2,780838243	0,424009403
226540_at	CCDC42B	1,225123479	0,80114751	0,423975969
219720_s_at	C14orf118	1,307201325	0,883374248	0,423827077
208649_s_at	VCP	4,494493519	4,071220803	0,423272717
219843_at	IPP	1,857989965	1,434743608	0,423246357
203292_s_at	VPS11	1,857989965	1,434743608	0,423246357
203859_s_at	PALM	0,847529938	0,424693306	0,422836632
215260_s_at	TCF3	0,847529938	0,424693306	0,422836632
201088_at	KPNA2	6,427629208	6,005022993	0,422606215
243423_at	TNIP1	1,830468611	1,408220608	0,422248003
207416_s_at	NFATC3	1,671397663	1,249194639	0,422203024
223155_at	HDHD2	3,909506676	3,487551166	0,421955509
224512_s_at	LSMD1	3,349017424	2,927197469	0,421819955
214003_x_at	RPS20 /// SNORD5	8,567091679	8,145373998	0,421717681
224594_x_at	ACTB /// LOC1005	7,994671536	7,572960554	0,421710983
1559372_at	-	-0,551284523	-0,972893339	0,421608816

238288_at	LOC100506838	-0,551284523	-0,972893339	0,421608816
210273_at	PCDH7	-0,551284523	-0,972893339	0,421608816
1559687_at	TMEM221	-0,551284523	-0,972893339	0,421608816
240321_at	-	-0,551284523	-0,972893339	0,421608816
239068_at	GNL1	-0,551284523	-0,972893339	0,421608816
234883_x_at	-	-0,551284523	-0,972893339	0,421608816
1563009_at	LOC284930	-0,551284523	-0,972893339	0,421608816
1557601_s_at	KIAA1257 /// LOC1	-0,551284523	-0,972893339	0,421608816
220516_at	ZSCAN2	-0,551284523	-0,972893339	0,421608816
200866_s_at	PSAP	3,768156719	3,346715127	0,421441591
241093_at	-	0,707657549	0,286279868	0,421377681
205011_at	VWA5A	0,707657549	0,286279868	0,421377681
225861_at	FAM195A	2,890618076	2,469496389	0,421121687
223248_at	HSDL1	2,007220798	1,586121802	0,421098996
218920_at	FAM193B	2,007220798	1,586121802	0,421098996
225713_at	STK11IP	0,458905032	0,03784737	0,421057661
204650_s_at	APBB3	0,458905032	0,03784737	0,421057661
235970_at	LCORL	1,071336699	0,65030602	0,421030679
238896_at	-	0,900065578	0,479089184	0,420976394
209694_at	PTS	3,204847647	2,783936118	0,420911528
227523_s_at	PHF20L1	3,252865595	2,832036229	0,420829367
228041_at	AASDH	1,983901861	1,563256447	0,420645414
205382_s_at	CFD	1,745502609	1,324949741	0,420552868
1557172_x_at	NEK8	1,510970097	1,090640407	0,420329691
202569_s_at	MARK3	1,871521385	1,451507543	0,420013842
206192_at	CDSN	-0,135065865	-0,554696666	0,419630801
243554_at	-	-0,135065865	-0,554696666	0,419630801
201320_at	SMARCC2	2,107136847	1,687645398	0,419491449
233694_at	HSPA1L	0,001114523	-0,418248858	0,419363381
229369_at	VSIG2	0,001114523	-0,418248858	0,419363381
238781_at	SREK1	0,001114523	-0,418248858	0,419363381
219214_s_at	NT5C	2,16969527	1,750483134	0,419212136
236525_at	FBXO36	0,355751005	-0,063377083	0,419128088
215697_at	-	0,355751005	-0,063377083	0,419128088
225814_at	XRN1	3,418470674	2,999351239	0,419119435
229956_at	NR2C1	-1,537851782	-1,956681069	0,418829287
220197_at	ATP6V0A4	-1,537851782	-1,956681069	0,418829287
1563092_at	-	-1,537851782	-1,956681069	0,418829287
208314_at	RRH	-1,537851782	-1,956681069	0,418829287
241001_at	-	-1,537851782	-1,956681069	0,418829287
229133_s_at	ZNF397	-1,537851782	-1,956681069	0,418829287
1569459_a_at	-	-1,537851782	-1,956681069	0,418829287
228193_s_at	RGCC	-1,537851782	-1,956681069	0,418829287
216857_at	IL23A	-1,537851782	-1,956681069	0,418829287
229171_at	CENPBD1	-1,537851782	-1,956681069	0,418829287
214863_at	-	-1,537851782	-1,956681069	0,418829287
241521_at	LOC100507652	-1,537851782	-1,956681069	0,418829287
233386_at	-	-1,537851782	-1,956681069	0,418829287
243547_at	FLJ39639 /// ZNF2	-1,537851782	-1,956681069	0,418829287
237603_at	C1orf100	-1,537851782	-1,956681069	0,418829287

226211_at	MEG3	-1,537851782	-1,956681069	0,418829287
211039_at	CHRNA1	-1,537851782	-1,956681069	0,418829287
1555443_at	CCDC172	-1,537851782	-1,956681069	0,418829287
209594_x_at	PSG9	-1,537851782	-1,956681069	0,418829287
1559195_at	-	-1,537851782	-1,956681069	0,418829287
1560604_at	-	-1,537851782	-1,956681069	0,418829287
241527_at	-	-1,537851782	-1,956681069	0,418829287
1560220_a_at	CBY1	-1,537851782	-1,956681069	0,418829287
241735_at	RASSF8	-1,537851782	-1,956681069	0,418829287
227588_s_at	GET4	-1,537851782	-1,956681069	0,418829287
236634_at	C8orf48	-1,537851782	-1,956681069	0,418829287
213832_at	KCND3	-1,537851782	-1,956681069	0,418829287
228140_s_at	PPP2R2C	-1,537851782	-1,956681069	0,418829287
211980_at	COL4A1	-1,537851782	-1,956681069	0,418829287
225646_at	CTSC	4,281531373	3,862709873	0,4188215
227485_at	DDX26B	1,402495885	0,98381571	0,418680175
1556133_s_at	LOC100169752	-1,893506789	-2,312054429	0,41854764
210525_x_at	EFCAB11	-1,893506789	-2,312054429	0,41854764
239541_at	LOC100505817	-1,893506789	-2,312054429	0,41854764
1558010_s_at	SLC1A2	-1,893506789	-2,312054429	0,41854764
213285_at	TMEM30B	-1,893506789	-2,312054429	0,41854764
1569722_s_at	LOC219731	-1,893506789	-2,312054429	0,41854764
234595_at	CATX-1	-1,893506789	-2,312054429	0,41854764
1555352_at	FOXP2	-1,893506789	-2,312054429	0,41854764
239553_at	LOC729420	-1,893506789	-2,312054429	0,41854764
206633_at	CHRNA1	-1,893506789	-2,312054429	0,41854764
1561064_a_at	-	-1,893506789	-2,312054429	0,41854764
216140_at	-	-1,893506789	-2,312054429	0,41854764
221644_s_at	SLC45A2	-1,893506789	-2,312054429	0,41854764
231648_at	-	-1,893506789	-2,312054429	0,41854764
209684_at	RIN2	-1,893506789	-2,312054429	0,41854764
216413_at	-	-1,893506789	-2,312054429	0,41854764
1560771_at	-	-1,893506789	-2,312054429	0,41854764
220471_s_at	MYCT1	-1,893506789	-2,312054429	0,41854764
234383_x_at	-	-1,893506789	-2,312054429	0,41854764
208574_at	SOX14	-1,893506789	-2,312054429	0,41854764
1555244_at	LOC554207	-1,893506789	-2,312054429	0,41854764
237975_at	LOC100505986	-1,893506789	-2,312054429	0,41854764
234458_at	ANPEP	-1,893506789	-2,312054429	0,41854764
236786_at	FAM135B	-1,893506789	-2,312054429	0,41854764
1553051_s_at	ODF3	-1,893506789	-2,312054429	0,41854764
211886_s_at	TBX5	-1,893506789	-2,312054429	0,41854764
237954_x_at	-	-1,893506789	-2,312054429	0,41854764
213664_at	SLC1A1	-1,893506789	-2,312054429	0,41854764
209079_x_at	PCDHGA1 /// PCDI	0,127732831	-0,290694975	0,418427806
233833_at	-	0,127732831	-0,290694975	0,418427806
200012_x_at	RPL21 /// RPL21P2	8,505428258	8,087013051	0,418415207
217299_s_at	NBN	2,575362572	2,157148588	0,418213984
212534_at	ZNF24	2,575362572	2,157148588	0,418213984
212039_x_at	RNU86 /// RPL3 //.	8,227475197	7,809285531	0,418189666

209065_at	UQCRB	3,327128828	2,908944587	0,418184241
215884_s_at	UBQLN2	4,09423887	3,676186811	0,418052059
201391_at	TRAP1	4,254948633	3,837037831	0,417910802
205331_s_at	REEP2	0,618277321	0,20062106	0,417656261
239277_at	-	1,20396005	0,786391549	0,417568501
203744_at	HMGB3	3,87876941	3,461380025	0,417389385
212130_x_at	EIF1	7,124489263	6,707542264	0,416946999
235091_at	PDE12	-0,280273599	-0,697182596	0,416908997
243432_at	CHL1-AS2	-0,280273599	-0,697182596	0,416908997
222876_s_at	ADAP2	-0,280273599	-0,697182596	0,416908997
236569_at	-	0,244681185	-0,172194225	0,41687541
234216_at	FLJ21408	0,244681185	-0,172194225	0,41687541
243651_at	CPEB3	0,244681185	-0,172194225	0,41687541
212471_at	AVL9	0,244681185	-0,172194225	0,41687541
229575_at	-	1,000459215	0,583607503	0,416851711
225547_at	SNHG6 /// SNORD	7,622054574	7,205280665	0,416773908
202689_at	RBM15B	2,159837409	1,743236669	0,416600739
202918_s_at	HSPE1-MOB4 /// N	5,075740228	4,659183503	0,416556725
202736_s_at	LSM4	4,855289601	4,439305907	0,415983695
1557735_at	-	-2,336678463	-2,752097204	0,41541874
1564567_at	-	-2,336678463	-2,752097204	0,41541874
224052_at	HSFY1 /// HSFY2	-2,336678463	-2,752097204	0,41541874
234825_at	-	-2,336678463	-2,752097204	0,41541874
1553654_at	SYT14	-2,336678463	-2,752097204	0,41541874
1563265_at	-	-2,336678463	-2,752097204	0,41541874
214887_at	N4BP2L1	-2,336678463	-2,752097204	0,41541874
1569208_a_at	-	-2,336678463	-2,752097204	0,41541874
234755_x_at	-	-2,336678463	-2,752097204	0,41541874
241564_at	GLOD5	-2,416625063	-2,832036647	0,415411584
1560161_at	CCNB2	-2,416625063	-2,832036647	0,415411584
232188_at	AKAP13	-2,416625063	-2,832036647	0,415411584
224389_s_at	COL25A1	-2,416625063	-2,832036647	0,415411584
1559543_at	LINC00441	-2,416625063	-2,832036647	0,415411584
212515_s_at	DDX3X	4,359963018	3,944614329	0,415348689
1556864_at	TCTN1	0,524545436	0,109458907	0,41508653
202032_s_at	MAN2A2	1,92317941	1,508107415	0,415071995
232853_at	-	-0,730013898	-1,145057014	0,415043116
1555417_a_at	TAS1R1	-0,730013898	-1,145057014	0,415043116
207027_at	HGFAC	-0,730013898	-1,145057014	0,415043116
206629_at	ADAMTSL2	-0,730013898	-1,145057014	0,415043116
1558525_at	-	-0,730013898	-1,145057014	0,415043116
1563802_at	LOC284551	-0,730013898	-1,145057014	0,415043116
1554205_s_at	ICA1L	-0,730013898	-1,145057014	0,415043116
211771_s_at	POU2F2	-0,730013898	-1,145057014	0,415043116
239133_at	CTDSPL2	1,493647653	1,078619386	0,415028267
215655_at	GRIK2	-2,250580859	-2,665156778	0,414575919
203789_s_at	SEMA3C	-2,250580859	-2,665156778	0,414575919
1563033_x_at	-	-2,250580859	-2,665156778	0,414575919
243678_at	-	-2,250580859	-2,665156778	0,414575919
1558797_at	-	-2,250580859	-2,665156778	0,414575919

237891_at	MDM2	-2,250580859	-2,665156778	0,414575919
220919_s_at	WDR96	-2,250580859	-2,665156778	0,414575919
1566169_at	-	-2,250580859	-2,665156778	0,414575919
226237_at	COL8A1	-2,250580859	-2,665156778	0,414575919
231478_at	PDE4C	-2,250580859	-2,665156778	0,414575919
243949_at	-	-2,250580859	-2,665156778	0,414575919
217412_at	-	-2,250580859	-2,665156778	0,414575919
228492_at	USP9Y	-2,250580859	-2,665156778	0,414575919
208927_at	SPOP	3,513985141	3,099629381	0,41435576
231182_at	WIPF1	1,898032047	1,483698568	0,414333479
227479_at	-	2,530390387	2,116147513	0,414242874
223949_at	TMPRSS3	2,530390387	2,116147513	0,414242874
226941_at	ATF6	3,555373205	3,141266133	0,414107072
212456_at	KIAA0664	2,791258167	2,37755543	0,413702736
223213_s_at	ZHX1	3,618399139	3,205255599	0,413143539
1569004_at	LOC100505812	1,047341799	0,634301128	0,413040671
225969_at	ALKBH6	3,166767593	2,753767314	0,413000279
238122_at	RBM12B	1,671397663	1,259081178	0,412316485
40837_at	TLE2	-0,157970873	-0,570167409	0,412196536
218683_at	PTBP2	2,811067138	2,398910172	0,412156965
228799_at	LOC100507239	3,055618382	2,64347734	0,412141042
217955_at	BCL2L13	2,267141818	1,855279162	0,411862657
1557948_at	PHLDB3	0,425036312	0,013190398	0,411845914
233820_at	-	0,425036312	0,013190398	0,411845914
233250_x_at	FOXRED2	0,425036312	0,013190398	0,411845914
211235_s_at	ESR1	-0,437449947	-0,84928999	0,411840043
217572_at	-	-0,437449947	-0,84928999	0,411840043
220535_at	FAM90A1	-0,437449947	-0,84928999	0,411840043
238397_at	-	-0,437449947	-0,84928999	0,411840043
216801_at	-	-0,437449947	-0,84928999	0,411840043
1569380_a_at	HERPUD1	-0,437449947	-0,84928999	0,411840043
209462_at	APLP1	-0,437449947	-0,84928999	0,411840043
215749_s_at	GORASP1	1,383446753	0,971607464	0,411839288
214095_at	SHMT2	2,657308324	2,245685006	0,411623317
210829_s_at	SSBP2	3,086998005	2,675582704	0,4114153
230566_at	MORC2-AS1	1,610353504	1,199296812	0,411056692
209577_at	PCYT2	1,267146969	0,85655369	0,41059328
236227_at	TMEM161B	1,267146969	0,85655369	0,41059328
201985_at	KIAA0196	2,552836808	2,142313983	0,410522825
220757_s_at	UBXN6	1,307201325	0,896834102	0,410367223
212261_at	GIGYF2	2,019346222	1,60918327	0,410162951
200025_s_at	RPL27	8,215534173	7,805469544	0,410064629
200856_x_at	NCOR1	1,225123479	0,815200271	0,409923208
1558722_at	ZNF252P	-1,452713826	-1,862269442	0,409555616
1553461_at	FAM9B	-1,452713826	-1,862269442	0,409555616
240936_at	LOC100287290	-1,452713826	-1,862269442	0,409555616
1557779_at	-	-1,452713826	-1,862269442	0,409555616
241254_at	-	-1,452713826	-1,862269442	0,409555616
237857_at	-	-1,452713826	-1,862269442	0,409555616
217584_at	NPC1	-1,452713826	-1,862269442	0,409555616

241321_at	-	-1,452713826	-1,862269442	0,409555616
1561534_at	-	-1,452713826	-1,862269442	0,409555616
236047_at	XKR6	-1,452713826	-1,862269442	0,409555616
234893_s_at	DNAH6	-1,452713826	-1,862269442	0,409555616
216923_at	CDKL5	-1,452713826	-1,862269442	0,409555616
227775_at	CELF6	-1,452713826	-1,862269442	0,409555616
236270_at	NFATC4	-1,452713826	-1,862269442	0,409555616
235193_at	-	-1,452713826	-1,862269442	0,409555616
216690_at	OR7C1	-1,452713826	-1,862269442	0,409555616
214168_s_at	TJP1	-1,452713826	-1,862269442	0,409555616
1563894_at	LOC441178	-1,452713826	-1,862269442	0,409555616
202896_s_at	SIRPA	-1,452713826	-1,862269442	0,409555616
210579_s_at	TRIM10	-1,452713826	-1,862269442	0,409555616
234859_at	PLXNA4	-1,452713826	-1,862269442	0,409555616
207007_at	NR1I3	-1,452713826	-1,862269442	0,409555616
243602_at	MGC40069	-1,452713826	-1,862269442	0,409555616
240036_at	SEC14L1	-1,452713826	-1,862269442	0,409555616
1559871_s_at	LOC100129129	-1,452713826	-1,862269442	0,409555616
206193_s_at	CDSN	-1,452713826	-1,862269442	0,409555616
221369_at	MTNR1A	-1,452713826	-1,862269442	0,409555616
1564537_a_at	ASB10	-1,452713826	-1,862269442	0,409555616
213182_x_at	CDKN1C	-1,452713826	-1,862269442	0,409555616
1563086_at	-	-1,452713826	-1,862269442	0,409555616
1560138_at	KIAA0226	-1,452713826	-1,862269442	0,409555616
213792_s_at	INSR	-1,452713826	-1,862269442	0,409555616
223451_s_at	CKLF	4,133352155	3,723797476	0,409554679
214725_at	SBSPON	-1,982324124	-2,391863529	0,409539405
244277_at	-	-1,982324124	-2,391863529	0,409539405
233346_at	-	-1,982324124	-2,391863529	0,409539405
1570470_at	CATSPERB	-1,982324124	-2,391863529	0,409539405
1565924_a_at	-	-1,982324124	-2,391863529	0,409539405
232770_at	TUSC3	-1,982324124	-2,391863529	0,409539405
233569_at	COPG2	-1,982324124	-2,391863529	0,409539405
240712_s_at	-	-1,982324124	-2,391863529	0,409539405
237879_at	-	-1,982324124	-2,391863529	0,409539405
244805_at	-	-1,982324124	-2,391863529	0,409539405
216190_x_at	ITGB1	-1,982324124	-2,391863529	0,409539405
220858_at	SORBS2	-1,982324124	-2,391863529	0,409539405
231731_at	OTX2	-1,982324124	-2,391863529	0,409539405
210138_at	RGS20	-1,982324124	-2,391863529	0,409539405
230478_at	OIT3	-1,982324124	-2,391863529	0,409539405
1553484_at	LINC00477	-1,982324124	-2,391863529	0,409539405
1553912_at	FLJ35424	-1,982324124	-2,391863529	0,409539405
210505_at	ADH7	-1,982324124	-2,391863529	0,409539405
238928_at	-	-1,982324124	-2,391863529	0,409539405
237815_at	-	-1,982324124	-2,391863529	0,409539405
217151_at	-	-1,982324124	-2,391863529	0,409539405
231405_at	SPACA7	-1,982324124	-2,391863529	0,409539405
239573_at	-	-1,982324124	-2,391863529	0,409539405
1558160_at	CCDC171	-1,982324124	-2,391863529	0,409539405

225299_at	MYO5B	-1,982324124	-2,391863529	0,409539405
242269_at	FLJ42875	-1,982324124	-2,391863529	0,409539405
1560790_at	GOLGA6L2	-1,982324124	-2,391863529	0,409539405
211334_at	MRE11A	-1,982324124	-2,391863529	0,409539405
207944_at	OCM2	-1,982324124	-2,391863529	0,409539405
244859_at	-	-1,982324124	-2,391863529	0,409539405
1557790_at	JRKL	-1,982324124	-2,391863529	0,409539405
242436_at	-	-1,982324124	-2,391863529	0,409539405
242323_at	PLA2G12A	3,422378845	3,012928641	0,409450204
226787_at	ZNF18	2,159837409	1,750483134	0,409354275
207437_at	NOVA1	-2,495152701	-2,904470894	0,409318193
237967_at	HAL	-2,495152701	-2,904470894	0,409318193
220128_s_at	NIPAL2	0,735999505	0,326777209	0,409222296
243599_at	LOC100507226	-1,06282519	-1,471833228	0,409008038
203619_s_at	FAIM2	-1,06282519	-1,471833228	0,409008038
203872_at	ACTA1	-1,06282519	-1,471833228	0,409008038
207529_at	DEFA5	-1,06282519	-1,471833228	0,409008038
1562231_at	-	-1,06282519	-1,471833228	0,409008038
1557329_at	-	-1,06282519	-1,471833228	0,409008038
1569915_at	-	-1,06282519	-1,471833228	0,409008038
241203_at	-	-1,06282519	-1,471833228	0,409008038
233196_at	-	-1,06282519	-1,471833228	0,409008038
216018_at	RNF5	-1,06282519	-1,471833228	0,409008038
1558140_at	PLXNA1	-1,06282519	-1,471833228	0,409008038
1557981_at	RPS9	-1,06282519	-1,471833228	0,409008038
231545_at	-	-1,06282519	-1,471833228	0,409008038
1570107_at	-	-1,06282519	-1,471833228	0,409008038
231076_at	C16orf82	-1,06282519	-1,471833228	0,409008038
1559559_at	FAM75E1	-1,06282519	-1,471833228	0,409008038
231568_at	CT47A1 /// CT47A:	-1,06282519	-1,471833228	0,409008038
242817_at	PGLYRP2	-1,06282519	-1,471833228	0,409008038
235001_at	DNAJC21	-1,06282519	-1,471833228	0,409008038
208041_at	GRK1	-1,06282519	-1,471833228	0,409008038
206527_at	ABAT	-1,06282519	-1,471833228	0,409008038
1568675_at	CHRNA10	-1,06282519	-1,471833228	0,409008038
210855_at	GREB1	-1,06282519	-1,471833228	0,409008038
223715_at	BRSK2	-1,06282519	-1,471833228	0,409008038
222650_s_at	SLC2A4RG	0,319287178	-0,089635713	0,408922891
221827_at	RBCK1	4,022766247	3,614020495	0,408745752
203776_at	GPKOW	2,824160383	2,415420763	0,40873962
223677_at	ATG10	2,179708195	1,771157334	0,408550861
236143_at	-	0,08619576	-0,322340048	0,408535808
209727_at	GM2A	0,97663406	0,568155442	0,408478618
214895_s_at	ADAM10	0,97663406	0,568155442	0,408478618
210984_x_at	EGFR	-0,180990326	-0,589001171	0,408010845
1559210_at	-	-0,180990326	-0,589001171	0,408010845
221385_s_at	FFAR3	-0,180990326	-0,589001171	0,408010845
1559261_a_at	LOC100653085 ///	-0,180990326	-0,589001171	0,408010845
241062_at	-	-0,180990326	-0,589001171	0,408010845
230430_at	ENTPD2	-0,180990326	-0,589001171	0,408010845

209981_at	CSDC2	-0,180990326	-0,589001171	0,408010845
207648_at	DRP2	-0,180990326	-0,589001171	0,408010845
1552834_at	B3GNT6	-0,180990326	-0,589001171	0,408010845
234575_at	ZNF71	-0,180990326	-0,589001171	0,408010845
222649_at	XPO4	2,074841895	1,666953618	0,407888278
209157_at	DNAJA2	4,241697258	3,834044437	0,407652821
220772_at	BPESC1	-2,163157732	-2,570522741	0,40736501
1566632_at	-	-2,163157732	-2,570522741	0,40736501
228766_at	CD36	-2,163157732	-2,570522741	0,40736501
232258_at	-	-2,163157732	-2,570522741	0,40736501
240987_at	-	-2,163157732	-2,570522741	0,40736501
1562886_at	-	-2,163157732	-2,570522741	0,40736501
201280_s_at	DAB2	-2,163157732	-2,570522741	0,40736501
244446_at	LOC100505525	-2,163157732	-2,570522741	0,40736501
1567248_at	OR9A1P	-2,163157732	-2,570522741	0,40736501
1557533_at	-	-2,163157732	-2,570522741	0,40736501
242985_x_at	RNF180	-2,163157732	-2,570522741	0,40736501
241290_at	-	-2,163157732	-2,570522741	0,40736501
1563373_at	LOC731223	-2,163157732	-2,570522741	0,40736501
232721_at	TRIM55	-2,163157732	-2,570522741	0,40736501
208331_at	BPY2	-2,163157732	-2,570522741	0,40736501
221420_at	-	-2,163157732	-2,570522741	0,40736501
234169_at	-	-2,163157732	-2,570522741	0,40736501
233285_at	-	-2,163157732	-2,570522741	0,40736501
205651_x_at	RAPGEF4	-2,163157732	-2,570522741	0,40736501
1559450_at	-	-2,163157732	-2,570522741	0,40736501
241536_at	-	-2,163157732	-2,570522741	0,40736501
1560169_at	-	-2,163157732	-2,570522741	0,40736501
244234_at	-	-0,043925712	-0,45121326	0,407287548
220848_x_at	OBP2A	-0,043925712	-0,45121326	0,407287548
225316_at	MFSD2A	-0,043925712	-0,45121326	0,407287548
211628_x_at	FTH1P5	6,336399256	5,929289821	0,407109435
222199_s_at	BIN3	2,642918178	2,235857731	0,407060448
223145_s_at	AKIRIN2	4,033407967	3,626539517	0,406868449
201581_at	TMX4	4,556423731	4,149648657	0,406775075
236174_at	-	0,491906512	0,085534992	0,406371519
229697_at	HIRIP3	0,491906512	0,085534992	0,406371519
218644_at	PLEK2	0,491906512	0,085534992	0,406371519
239097_at	FRRS1L	-0,609040214	-1,015398016	0,406357802
241447_at	ING5	-0,609040214	-1,015398016	0,406357802
216474_x_at	TPSAB1 /// TPSB2	-0,609040214	-1,015398016	0,406357802
202112_at	VWF	-0,609040214	-1,015398016	0,406357802
211958_at	IGFBP5	-0,609040214	-1,015398016	0,406357802
201205_at	-	-0,609040214	-1,015398016	0,406357802
215678_at	LOC440792	-0,609040214	-1,015398016	0,406357802
1564439_a_at	C11orf36	-0,609040214	-1,015398016	0,406357802
1570124_at	-	-0,609040214	-1,015398016	0,406357802
1561102_at	-	-0,609040214	-1,015398016	0,406357802
1553780_at	MGC23270	0,791908897	0,385582632	0,406326266
223606_x_at	KIAA1704	3,03433073	2,628204186	0,406126544

226156_at	AKT2	1,730929079	1,324949741	0,405979338
226797_at	MBTD1	2,365121417	1,9592579	0,405863518
223231_at	TATDN1	4,283600482	3,877875398	0,405725084
202967_at	GSTA4	2,107136847	1,701800759	0,405336087
209780_at	PHTF2	4,010828383	3,605722367	0,405106016
222400_s_at	ADI1	5,806652898	5,401834291	0,404818607
201877_s_at	PPP2R5C	4,680946381	4,276165612	0,404780769
1554429_a_at	DMWD	0,847529938	0,443143223	0,404386715
234094_x_at	DNAJC21	-1,212652659	-1,616924751	0,404272092
1560631_at	CALCOCO2	-1,212652659	-1,616924751	0,404272092
241073_at	-	-1,212652659	-1,616924751	0,404272092
237795_s_at	SP2	-1,212652659	-1,616924751	0,404272092
1565892_at	-	-1,212652659	-1,616924751	0,404272092
1564401_at	LOC400748	-1,212652659	-1,616924751	0,404272092
214627_at	EPX	-1,212652659	-1,616924751	0,404272092
234404_at	CAND2	-1,212652659	-1,616924751	0,404272092
1557608_a_at	LOC284080	-1,212652659	-1,616924751	0,404272092
243902_at	-	-1,212652659	-1,616924751	0,404272092
221391_at	TAS2R14	-1,212652659	-1,616924751	0,404272092
1564851_at	-	-1,212652659	-1,616924751	0,404272092
206258_at	ST8SIA5	-1,212652659	-1,616924751	0,404272092
211388_s_at	-	-1,212652659	-1,616924751	0,404272092
210333_at	NR5A1	-1,212652659	-1,616924751	0,404272092
218814_s_at	TMEM206	-1,212652659	-1,616924751	0,404272092
237186_at	KCNJ5	-1,212652659	-1,616924751	0,404272092
244593_at	C17orf28	-1,212652659	-1,616924751	0,404272092
213976_at	CIZ1	-1,212652659	-1,616924751	0,404272092
238275_at	HAP1	-1,212652659	-1,616924751	0,404272092
241381_at	CXorf36	-1,212652659	-1,616924751	0,404272092
219509_at	MYOZ1	-1,212652659	-1,616924751	0,404272092
1560180_at	-	-1,212652659	-1,616924751	0,404272092
235657_at	-	-1,212652659	-1,616924751	0,404272092
211573_x_at	TGM2	-1,212652659	-1,616924751	0,404272092
224921_at	SCAMP2	1,830468611	1,426280426	0,404188185
200705_s_at	EEF1B2 /// SNORA	7,7364617	7,332458135	0,404003564
210284_s_at	TAB2	3,147261986	2,743433212	0,403828773
212854_x_at	NBPF10	5,092485786	4,688802819	0,403682967
1563828_at	-	-0,924000698	-1,32740143	0,403400732
235420_at	HAPLN4	-0,924000698	-1,32740143	0,403400732
1561897_at	-	-0,924000698	-1,32740143	0,403400732
215816_at	GUSBP11 /// GUSE	-0,924000698	-1,32740143	0,403400732
1560910_at	PPIL6	-0,924000698	-1,32740143	0,403400732
240500_at	-	-0,924000698	-1,32740143	0,403400732
1553539_at	KRT74	-0,924000698	-1,32740143	0,403400732
216001_at	PRAMEF12	-0,924000698	-1,32740143	0,403400732
238340_at	DCAF8	-0,924000698	-1,32740143	0,403400732
205325_at	PHYHIP	-0,924000698	-1,32740143	0,403400732
234283_at	-	-0,924000698	-1,32740143	0,403400732
217432_s_at	IDS	1,20396005	0,80114751	0,40281254
203781_at	MRPL33	5,543354832	5,140853304	0,402501528

203524_s_at	MPST	2,138605583	1,736450458	0,402155126
244052_at	CBR4	2,401508182	1,999354538	0,402153644
239307_at	-	0,39105295	-0,011001236	0,402054186
1558279_a_at	KDSR	0,39105295	-0,011001236	0,402054186
204705_x_at	ALDOB	-0,332405896	-0,734355396	0,4019495
244843_x_at	-	-0,332405896	-0,734355396	0,4019495
204889_s_at	NEURL	-0,332405896	-0,734355396	0,4019495
223338_s_at	ATPIF1	3,668220666	3,266431559	0,401789107
219235_s_at	PHACTR4	2,096248267	1,694547353	0,401700914
212333_at	FAM98A	4,155757614	3,754141744	0,40161587
219448_at	TMEM70	1,88491011	1,483698568	0,401211542
201891_s_at	B2M	8,231275979	7,830124639	0,40115134
1556328_at	-	-2,569309811	-2,970378591	0,401068779
203644_s_at	MON1B	1,843966654	1,44320939	0,400757264
214092_x_at	SUGP2	3,127551022	2,726850891	0,400700131
203043_at	ZBED1	2,427650693	2,026957145	0,400693548
234841_x_at	OBP2A	0,556428218	0,155747281	0,400680937
237010_at	-	0,045517965	-0,354781582	0,400299546
1568845_at	-	0,045517965	-0,354781582	0,400299546
241935_at	SHROOM1	0,045517965	-0,354781582	0,400299546
223744_s_at	SIAE	0,045517965	-0,354781582	0,400299546
218822_s_at	NPEPL1 /// STX16-	0,045517965	-0,354781582	0,400299546
229562_at	RPL10A	0,045517965	-0,354781582	0,400299546
243345_at	RNF14	-0,791294935	-1,19085694	0,399562005
236211_at	RAB40B	-0,791294935	-1,19085694	0,399562005
207429_at	SLC22A2	-0,791294935	-1,19085694	0,399562005
208479_at	KCNA1	-0,791294935	-1,19085694	0,399562005
244524_at	-	-0,791294935	-1,19085694	0,399562005
231554_at	TPD52L3	-0,791294935	-1,19085694	0,399562005
232730_at	C19orf44	-0,791294935	-1,19085694	0,399562005
221318_at	NEUROD4	-0,791294935	-1,19085694	0,399562005
211816_x_at	FCAR	-0,791294935	-1,19085694	0,399562005
242689_at	RALGPS1	-0,791294935	-1,19085694	0,399562005
229108_at	LOC100507278	-0,791294935	-1,19085694	0,399562005
207463_x_at	PRSS3	-0,791294935	-1,19085694	0,399562005
221529_s_at	PLVAP	-0,791294935	-1,19085694	0,399562005
238201_at	LOC100505786	-0,791294935	-1,19085694	0,399562005
203539_s_at	-	-0,791294935	-1,19085694	0,399562005
214510_at	GPR20	-0,791294935	-1,19085694	0,399562005
209693_at	ASTN2	-0,791294935	-1,19085694	0,399562005
1557325_at	-	-0,791294935	-1,19085694	0,399562005
218881_s_at	FOSL2	-0,791294935	-1,19085694	0,399562005
224732_at	CHTF8	4,046717183	3,647229275	0,399487907
225813_at	RC3H2	2,732422196	2,332940758	0,399481439
235239_at	QSOX2	0,282358733	-0,116767475	0,399126208
218298_s_at	C14orf159	3,142467702	2,743433212	0,39903449
228676_at	ORAOV1	0,167727503	-0,231045907	0,39877341
208906_at	BSCL2 /// HNRNPL	3,060716358	2,662083808	0,398632551
211983_x_at	ACTG1	8,437391317	8,038828416	0,398562901
235466_s_at	-	2,042564225	1,644429915	0,39813431

225211_at	PVRL1	0,764143511	0,366031214	0,398112297
204166_at	SBNO2	0,764143511	0,366031214	0,398112297
219019_at	PIDD	2,107136847	1,709047448	0,398089399
223048_at	SDHAF2	3,610731271	3,212688281	0,39804299
201454_s_at	LOC100653042 ///	4,441588953	4,043683659	0,397905294
214812_s_at	MOB1A	4,66447449	4,26677677	0,39769772
213450_s_at	ICOSLG	1,476175815	1,078619386	0,397556429
209267_s_at	SLC39A8	5,963268214	5,565791084	0,397477131
224916_at	TMEM173	1,730929079	1,333668715	0,397260364
202152_x_at	USF2	2,507817181	2,110584371	0,397232811
1555326_a_at	ADAM9	0,458905032	0,061773582	0,397131449
222492_at	PDXK	1,094118704	0,697221375	0,396897329
208904_s_at	RPS28	8,156800905	7,759937137	0,396863767
222052_at	C19orf54	2,752615529	2,355777584	0,396837945
213038_at	RNF19B	1,440265662	1,043469112	0,39679655
203040_s_at	HMBS	2,778527892	2,381744287	0,396783605
206308_at	TRDMT1	1,182925501	0,786391549	0,396533952
215809_at	CYP2D6	1,182925501	0,786391549	0,396533952
209549_s_at	DGUOK	5,273387846	4,876912218	0,396475629
217900_at	IARS2	5,066267042	4,669856971	0,396410071
1562256_at	NLRP1	-0,49284695	-0,889222211	0,396375261
1562012_at	LOC100506730	-0,49284695	-0,889222211	0,396375261
227721_at	CPAMD8	-0,49284695	-0,889222211	0,396375261
206867_at	GCKR	-0,49284695	-0,889222211	0,396375261
214387_x_at	SFTPC	-0,49284695	-0,889222211	0,396375261
211461_at	CSPG4P1Y	-0,49284695	-0,889222211	0,396375261
238072_at	CLK3	-0,49284695	-0,889222211	0,396375261
237228_at	ZDHHC1	-0,49284695	-0,889222211	0,396375261
56821_at	SLC38A7	0,540574899	0,14433843	0,39623647
212427_at	KIAA0368	0,618277321	0,222432814	0,395844507
208199_s_at	ZFP161	0,618277321	0,222432814	0,395844507
203208_s_at	MTFR1	3,142467702	2,746644067	0,395823635
41386_i_at	KDM6B	1,225123479	0,829309537	0,395813942
227844_at	FMNL3	-0,088974936	-0,484558493	0,395583558
232437_at	CPSF3L	-0,088974936	-0,484558493	0,395583558
236813_at	MORN4	-0,088974936	-0,484558493	0,395583558
217189_s_at	SMG7	-0,088974936	-0,484558493	0,395583558
218509_at	LPPR2	-0,088974936	-0,484558493	0,395583558
236988_x_at	ITGB2	-0,088974936	-0,484558493	0,395583558
203601_s_at	ZBTB17	-0,088974936	-0,484558493	0,395583558
225711_at	ARL6IP6	2,179708195	1,784182261	0,395525934
210349_at	CAMK4	2,636121902	2,24081821	0,395303691
217938_s_at	KCMF1	6,149090529	5,754369771	0,394720758
226995_at	LOC642852	0,873789652	0,479089184	0,394700468
222658_s_at	APTX	2,460890032	2,066329863	0,394560169
226270_at	EXOC2	1,910557155	1,516067292	0,394489863
213444_at	ZNF862	1,402495885	1,008210238	0,394285647
235836_at	MXRA7	-1,369772723	-1,763664074	0,393891351
244532_x_at	-	-1,369772723	-1,763664074	0,393891351
1570394_at	XRN1	-1,369772723	-1,763664074	0,393891351

231131_at	FAM133A	-1,369772723	-1,763664074	0,393891351
1563916_at	WDR11-AS1	-1,369772723	-1,763664074	0,393891351
228489_at	TM4SF18	-1,369772723	-1,763664074	0,393891351
217328_at	IL23A	-1,369772723	-1,763664074	0,393891351
204987_at	ITIH2	-1,369772723	-1,763664074	0,393891351
217455_s_at	SSTR2	-1,369772723	-1,763664074	0,393891351
1561221_x_at	LOC728099	-1,369772723	-1,763664074	0,393891351
239180_at	LOC100507419 ///	-1,369772723	-1,763664074	0,393891351
229406_at	RBFOX3	-1,369772723	-1,763664074	0,393891351
207976_at	KLHL18	-1,369772723	-1,763664074	0,393891351
218207_s_at	STMN3	-1,369772723	-1,763664074	0,393891351
239079_at	ADAM7	-1,369772723	-1,763664074	0,393891351
229627_at	C9orf174 /// LOC1	-1,369772723	-1,763664074	0,393891351
215783_s_at	ALPL	-1,369772723	-1,763664074	0,393891351
236805_at	C9orf96	-1,369772723	-1,763664074	0,393891351
211287_x_at	CSF2RA	-1,369772723	-1,763664074	0,393891351
227282_at	PCDH19	-1,369772723	-1,763664074	0,393891351
236682_at	-	-1,369772723	-1,763664074	0,393891351
213386_at	TMEM246	-1,369772723	-1,763664074	0,393891351
244366_at	LOC100506371	-1,369772723	-1,763664074	0,393891351
220512_at	DLC1	-1,369772723	-1,763664074	0,393891351
1553437_at	KLHDC7A	-1,369772723	-1,763664074	0,393891351
228017_s_at	NKAIN4	-1,369772723	-1,763664074	0,393891351
236601_at	LOC100506484	-1,369772723	-1,763664074	0,393891351
204467_s_at	SNCA	-1,369772723	-1,763664074	0,393891351
216654_at	TNXB	-1,369772723	-1,763664074	0,393891351
220599_s_at	CARD14	-1,369772723	-1,763664074	0,393891351
1552694_at	SLC2A13	-2,641232718	-3,035090645	0,393857927
213356_x_at	HNRNPA1 /// HNR	7,707757918	7,314070123	0,393687795
204084_s_at	CLN5	3,055618382	2,662083808	0,393534574
229447_x_at	LOC100506032 ///	5,13429861	4,740776661	0,393521949
221879_at	CALML4	1,88491011	1,491644074	0,393266037
1556291_at	POM121L12	0,97663406	0,583607503	0,393026557
235475_at	SERP1	1,364555912	0,971607464	0,392948448
215452_x_at	SUMO4	5,241506811	4,848562131	0,39294468
41160_at	MBD3	2,938185732	2,545310235	0,392875496
219411_at	ELMO3	-0,230432956	-0,623254098	0,392821143
242359_at	-	-0,230432956	-0,623254098	0,392821143
208395_s_at	URB1	-0,230432956	-0,623254098	0,392821143
204296_at	DCTN1	-0,230432956	-0,623254098	0,392821143
231006_at	SPATA8	-0,230432956	-0,623254098	0,392821143
206216_at	SRPK3	-0,230432956	-0,623254098	0,392821143
223082_at	SH3KBP1	6,480938027	6,08812196	0,392816067
209923_s_at	BRAP	1,671397663	1,278654284	0,392743379
202010_s_at	ZNF410	3,559161816	3,166549502	0,392612314
200815_s_at	PAFAH1B1	4,096676757	3,704101387	0,39257537
213477_x_at	EEF1A1 /// LOC10(	8,602107596	8,209644708	0,392462888
205519_at	WDR76	1,843966654	1,451507543	0,392459112
223446_s_at	DTNBP1	2,285848435	1,893444329	0,392404106
228975_at	SP6	0,355751005	-0,036543687	0,392294692

238592_at	-	-2,074223907	-2,466349414	0,392125507
1558743_at	ZNF620	-2,074223907	-2,466349414	0,392125507
232451_at	-	-2,074223907	-2,466349414	0,392125507
225911_at	NPNT	-2,074223907	-2,466349414	0,392125507
1553191_at	DST /// LOC10065	-2,074223907	-2,466349414	0,392125507
211324_s_at	RGPD3 /// RGPD4	-2,074223907	-2,466349414	0,392125507
211265_at	PTGER3	-2,074223907	-2,466349414	0,392125507
239502_at	-	-2,074223907	-2,466349414	0,392125507
1563371_at	-	-2,074223907	-2,466349414	0,392125507
1555186_at	-	-2,074223907	-2,466349414	0,392125507
220754_at	ARMC9	-2,074223907	-2,466349414	0,392125507
236670_s_at	-	-2,074223907	-2,466349414	0,392125507
1561114_a_at	DEPDC4	-2,074223907	-2,466349414	0,392125507
204304_s_at	PROM1	-2,074223907	-2,466349414	0,392125507
1556508_s_at	LOC100507274	-2,074223907	-2,466349414	0,392125507
1558941_at	ZNF704	-2,074223907	-2,466349414	0,392125507
1562591_a_at	OFCC1	-2,074223907	-2,466349414	0,392125507
228412_at	LOC643072	-2,074223907	-2,466349414	0,392125507
236331_at	CDKL2	-2,074223907	-2,466349414	0,392125507
1562065_at	-	-2,074223907	-2,466349414	0,392125507
210261_at	KCNK2	-2,074223907	-2,466349414	0,392125507
238941_at	TRIM50	-2,074223907	-2,466349414	0,392125507
219896_at	CALY	-2,074223907	-2,466349414	0,392125507
237721_s_at	ASB4	-2,074223907	-2,466349414	0,392125507
222139_at	ERV3-2	-2,074223907	-2,466349414	0,392125507
207225_at	AANAT	-2,074223907	-2,466349414	0,392125507
232503_at	-	-2,074223907	-2,466349414	0,392125507
209749_s_at	ACE	-2,074223907	-2,466349414	0,392125507
227752_at	SPTLC3	-2,074223907	-2,466349414	0,392125507
227929_at	LIN7A	-2,074223907	-2,466349414	0,392125507
230650_at	-	-2,074223907	-2,466349414	0,392125507
209971_x_at	AIMP2	4,859875458	4,46803814	0,391837318
239641_at	FLI1-AS1	0,524545436	0,133104519	0,391440917
229027_at	PPM1A	0,524545436	0,133104519	0,391440917
211802_x_at	CACNA1G	0,524545436	0,133104519	0,391440917
32128_at	CCL18	-1,664214284	-2,055617718	0,391403434
208948_s_at	STAU1	4,57799322	4,186639529	0,391353691
209063_x_at	PAIP1	4,046717183	3,655528924	0,391188258
202332_at	CSNK1E	2,949541692	2,558652146	0,390889546
226970_at	FBXO33	1,816938997	1,426280426	0,390658571
202717_s_at	CDC16	3,577387089	3,186758158	0,390628932
232278_s_at	DEPDC1	3,117098466	2,726850891	0,390247575
221798_x_at	-	8,919140698	8,529058236	0,390082462
202647_s_at	NRAS	3,581038515	3,191011385	0,390027129
220766_at	BTG4	1,024284941	0,634301128	0,389983813
215764_x_at	AP2A2	1,610353504	1,220470658	0,389882846
1564705_at	GLS2	-2,416625063	-2,8064286	0,389803536
1560684_x_at	NBEAP1	-2,416625063	-2,8064286	0,389803536
241880_x_at	-	-2,416625063	-2,8064286	0,389803536
213652_at	PCSK5	-2,416625063	-2,8064286	0,389803536

240414_at	-	-2,416625063	-2,8064286	0,389803536
1561613_at	-	-2,416625063	-2,8064286	0,389803536
223769_x_at	HYI	0,244681185	-0,144765583	0,389446768
213476_x_at	TUBB3	4,565392441	4,175977324	0,389415117
217225_x_at	NOMO1 /// NOMC	5,900006878	5,510609755	0,389397123
213941_x_at	RPS7	8,546532259	8,157176443	0,389355816
212778_at	PACS2	1,116462765	0,727123109	0,389339656
229670_at	-	3,9635104	3,5744736	0,3890368
225295_at	SLC39A10	2,179708195	1,790914523	0,388793672
202277_at	SPTLC1	4,636598653	4,247887799	0,388710855
200931_s_at	VCL	3,204847647	2,81616114	0,388686507
214062_x_at	NFKBIB	1,760255626	1,371634653	0,388620973
224597_at	LOC647979	5,383324257	4,995026007	0,38829825
230852_at	STAC3	0,127732831	-0,260526297	0,388259128
219425_at	SULT4A1	0,127732831	-0,260526297	0,388259128
212358_at	CLIP3	0,127732831	-0,260526297	0,388259128
202473_x_at	HCFC1	0,127732831	-0,260526297	0,388259128
227682_at	-	2,51558601	2,127337555	0,388248455
209122_at	LOC100509484 ///	3,398333742	3,010094234	0,388239507
225187_at	KIAA1967	2,902957057	2,514786608	0,388170449
222388_s_at	VPS35	4,341822193	3,953789653	0,38803254
1567257_at	OR1J2	0,001114523	-0,386896102	0,388010625
208258_s_at	GAS2L1	0,001114523	-0,386896102	0,388010625
1554346_at	GNB5	0,001114523	-0,386896102	0,388010625
222491_at	HGSNAT	0,001114523	-0,386896102	0,388010625
201893_x_at	DCN	-0,66934516	-1,057301851	0,387956691
224442_at	PHF6	-0,66934516	-1,057301851	0,387956691
227842_at	RAB30	-0,66934516	-1,057301851	0,387956691
240465_at	C4orf32	-0,66934516	-1,057301851	0,387956691
230253_at	SCUBE3	-0,66934516	-1,057301851	0,387956691
244544_at	-	-0,66934516	-1,057301851	0,387956691
1562723_at	-	-0,66934516	-1,057301851	0,387956691
1555056_at	CCNG2	-0,66934516	-1,057301851	0,387956691
1560480_at	-	-0,66934516	-1,057301851	0,387956691
209926_at	MEF2BNB	-0,66934516	-1,057301851	0,387956691
224945_at	BTBD7	2,62869474	2,24081821	0,387876529
217718_s_at	YWHAB	7,285486483	6,897703786	0,387782697
226642_s_at	NUDCD2	5,259994403	4,87243534	0,387559063
201567_s_at	GOLGA4	3,336134047	2,948635566	0,387498481
221741_s_at	YTHDF1	3,980708208	3,593410766	0,387297442
1557878_at	-	0,425036312	0,03784737	0,387188942
220165_at	INO80D	0,425036312	0,03784737	0,387188942
1553622_a_at	FSIP1	-0,384193355	-0,771340337	0,387146982
216586_at	-	-0,384193355	-0,771340337	0,387146982
207873_x_at	SEZ6L	-0,384193355	-0,771340337	0,387146982
236880_at	RAD52	-0,384193355	-0,771340337	0,387146982
216564_at	-	-0,384193355	-0,771340337	0,387146982
209327_s_at	NOP16	-0,384193355	-0,771340337	0,387146982
236647_at	-	-0,384193355	-0,771340337	0,387146982
201142_at	EIF2S1	3,8018113	3,414783177	0,387028123

202915_s_at	FAM20B	2,321828846	1,935144844	0,386684002
203207_s_at	MTFR1	2,053550345	1,666953618	0,386596727
204616_at	UCHL3	4,850340179	4,463801018	0,386539161
217797_at	UFC1	5,166702612	4,780167437	0,386535175
1559353_at	-	-2,336678463	-2,723114724	0,386436261
242940_x_at	DLX6	-2,336678463	-2,723114724	0,386436261
1567706_at	-	-2,336678463	-2,723114724	0,386436261
234289_x_at	-	-2,336678463	-2,723114724	0,386436261
1557468_at	-	-2,336678463	-2,723114724	0,386436261
242401_x_at	-	-2,336678463	-2,723114724	0,386436261
1556404_a_at	ZNRF2P1	-2,336678463	-2,723114724	0,386436261
220474_at	SLC25A21	-2,336678463	-2,723114724	0,386436261
243965_at	-	-2,336678463	-2,723114724	0,386436261
225832_s_at	DAGLB	0,791908897	0,405752839	0,386156059
237779_at	LOC100505711	0,791908897	0,405752839	0,386156059
202982_s_at	ACOT1 /// ACOT2	2,664625637	2,278572464	0,386053173
227014_at	ASPHD2	1,802733148	1,417157926	0,385575221
212834_at	DDX52	1,476175815	1,090640407	0,385535408
200661_at	CTSA	3,918896157	3,533373095	0,385523062
241151_at	-	-2,495152701	-2,880284116	0,385131415
1562342_at	-	-2,495152701	-2,880284116	0,385131415
241708_at	DOCK1	-2,495152701	-2,880284116	0,385131415
203543_s_at	KLF9	-2,495152701	-2,880284116	0,385131415
201181_at	GNAI3	2,649978302	2,264929589	0,385048713
1562999_x_at	-	-2,250580859	-2,635549085	0,384968226
209613_s_at	ADH1B	-2,250580859	-2,635549085	0,384968226
231070_at	IYD	-2,250580859	-2,635549085	0,384968226
1562854_at	-	-2,250580859	-2,635549085	0,384968226
235638_at	RASSF6	-2,250580859	-2,635549085	0,384968226
1554199_at	PTPRO	-2,250580859	-2,635549085	0,384968226
233404_at	-	-2,250580859	-2,635549085	0,384968226
206029_at	ANKRD1	-2,250580859	-2,635549085	0,384968226
244289_at	ZNF300P1	-2,250580859	-2,635549085	0,384968226
1564236_at	-	-2,250580859	-2,635549085	0,384968226
238219_at	C12orf50	-2,250580859	-2,635549085	0,384968226
206642_at	DSG1	-2,250580859	-2,635549085	0,384968226
231603_at	RNASE11	-2,250580859	-2,635549085	0,384968226
233463_at	RASSF6	-2,250580859	-2,635549085	0,384968226
237361_at	-	-2,250580859	-2,635549085	0,384968226
214913_at	ADAMTS3	-2,250580859	-2,635549085	0,384968226
1562057_at	-	-2,250580859	-2,635549085	0,384968226
1566101_at	TTLL5	-2,250580859	-2,635549085	0,384968226
241881_at	TRIM58	-2,250580859	-2,635549085	0,384968226
1560418_at	CEP57L1	-2,250580859	-2,635549085	0,384968226
1564244_a_at	LOC100506195	-2,250580859	-2,635549085	0,384968226
207363_at	RS1	-2,250580859	-2,635549085	0,384968226
1569558_at	-	-2,250580859	-2,635549085	0,384968226
237329_at	LOC100509621	-2,250580859	-2,635549085	0,384968226
220854_at	-	-2,250580859	-2,635549085	0,384968226
1556166_x_at	-	-2,250580859	-2,635549085	0,384968226

218981_at	ACN9	2,955426542	2,570802332	0,38462421
224840_at	FKBP5	5,525224248	5,140853304	0,384370944
213170_at	GPX7	2,791258167	2,406959565	0,384298602
1559611_at	TMEM75	-1,712732543	-2,09678811	0,384055567
1570038_at	ZNF595	-1,712732543	-2,09678811	0,384055567
222188_at	C9orf156	-1,712732543	-2,09678811	0,384055567
1559134_a_at	-	-1,712732543	-2,09678811	0,384055567
1561679_at	-	-1,712732543	-2,09678811	0,384055567
233390_at	-	-1,712732543	-2,09678811	0,384055567
237407_at	HS1BP3	-1,712732543	-2,09678811	0,384055567
207235_s_at	GRM5	-1,712732543	-2,09678811	0,384055567
1560595_at	-	-1,712732543	-2,09678811	0,384055567
1566768_at	-	-1,712732543	-2,09678811	0,384055567
1560464_at	TSPAN10	-1,712732543	-2,09678811	0,384055567
221347_at	CHRM5	-1,712732543	-2,09678811	0,384055567
230882_at	DLX6-AS1	-1,712732543	-2,09678811	0,384055567
228249_at	C11orf74	-1,712732543	-2,09678811	0,384055567
235928_at	-	-1,712732543	-2,09678811	0,384055567
230437_s_at	PRKCB	-1,712732543	-2,09678811	0,384055567
235904_at	UGT3A1	-1,712732543	-2,09678811	0,384055567
220983_s_at	LOC100653017 ///	-1,712732543	-2,09678811	0,384055567
237941_at	-	-1,712732543	-2,09678811	0,384055567
207325_x_at	MAGEA1	-1,712732543	-2,09678811	0,384055567
231460_at	LOC100507511	-1,712732543	-2,09678811	0,384055567
234163_at	UBE3A	-1,712732543	-2,09678811	0,384055567
206048_at	OVOL2	-1,712732543	-2,09678811	0,384055567
243239_at	SAMM50	-1,712732543	-2,09678811	0,384055567
1561429_a_at	C3orf15 /// LOC10	-1,712732543	-2,09678811	0,384055567
1569931_at	-	-1,712732543	-2,09678811	0,384055567
228176_at	S1PR3	-1,712732543	-2,09678811	0,384055567
214393_at	RND2	-1,712732543	-2,09678811	0,384055567
242387_at	C8orf42	-1,712732543	-2,09678811	0,384055567
1555298_a_at	VWA3B	-1,712732543	-2,09678811	0,384055567
1553658_at	LOC150197	-1,712732543	-2,09678811	0,384055567
239647_at	CHST13	-1,712732543	-2,09678811	0,384055567
215102_at	DPY19L1P1	-1,712732543	-2,09678811	0,384055567
237791_at	-	-1,712732543	-2,09678811	0,384055567
207510_at	BDKRB1	-1,712732543	-2,09678811	0,384055567
215472_at	PACRG	-1,712732543	-2,09678811	0,384055567
231485_at	LOC100505540	-1,712732543	-2,09678811	0,384055567
214111_at	OPCML	-1,712732543	-2,09678811	0,384055567
205878_at	POU6F1	-1,712732543	-2,09678811	0,384055567
220421_at	BTNL8	-1,712732543	-2,09678811	0,384055567
213938_at	ERC2	-1,712732543	-2,09678811	0,384055567
227446_s_at	DHRS4-AS1	2,085817135	1,701800759	0,384016375
216438_s_at	TMSB4X	8,77066684	8,38684351	0,38382333
201426_s_at	VIM	8,113585763	7,729794967	0,383790796
207177_at	PTGFR	-1,802716385	-2,186086603	0,383370218
207329_at	MMP8	-1,802716385	-2,186086603	0,383370218
1561476_at	-	-1,802716385	-2,186086603	0,383370218

213684_s_at	PDLIM5	-1,802716385	-2,186086603	0,383370218
1566725_at	-	-1,802716385	-2,186086603	0,383370218
220021_at	TMC7	-1,802716385	-2,186086603	0,383370218
243230_at	-	-1,802716385	-2,186086603	0,383370218
1563118_at	-	-1,802716385	-2,186086603	0,383370218
1564790_at	ST7-AS2	-1,802716385	-2,186086603	0,383370218
1559577_at	FCN2	-1,802716385	-2,186086603	0,383370218
1560900_a_at	-	-1,802716385	-2,186086603	0,383370218
1560944_at	SLC25A3P1	-1,802716385	-2,186086603	0,383370218
1567271_at	OR4C1P	-1,802716385	-2,186086603	0,383370218
230468_s_at	C1orf56	-1,802716385	-2,186086603	0,383370218
215442_s_at	TSHR	-1,802716385	-2,186086603	0,383370218
1560657_at	TIE1	-1,802716385	-2,186086603	0,383370218
207454_at	GRIK3	-1,802716385	-2,186086603	0,383370218
214889_at	FAM149A	-1,802716385	-2,186086603	0,383370218
223807_at	IGSF1	-1,802716385	-2,186086603	0,383370218
211632_at	IGHD /// IGHG1 //,	-1,802716385	-2,186086603	0,383370218
206309_at	LECT1	-1,802716385	-2,186086603	0,383370218
211365_s_at	PCDHA2	-1,802716385	-2,186086603	0,383370218
235818_at	VSTM1	-1,802716385	-2,186086603	0,383370218
216259_at	LOC100506699	-1,802716385	-2,186086603	0,383370218
213707_s_at	DLX5	-1,802716385	-2,186086603	0,383370218
1560178_at	-	-1,802716385	-2,186086603	0,383370218
240047_at	-	-1,802716385	-2,186086603	0,383370218
241189_at	-	-1,802716385	-2,186086603	0,383370218
209854_s_at	KLK2	-1,802716385	-2,186086603	0,383370218
232707_at	ISX	-1,802716385	-2,186086603	0,383370218
208487_at	LMX1B	-1,802716385	-2,186086603	0,383370218
224881_at	VKORC1L1	3,076375717	2,693044596	0,383331122
1560928_at	LOC151657	-0,135065865	-0,518232988	0,383167123
1556195_a_at	-	-0,135065865	-0,518232988	0,383167123
1556249_a_at	FLJ33065	-0,135065865	-0,518232988	0,383167123
211215_x_at	DIO2	-0,135065865	-0,518232988	0,383167123
212861_at	MFSD5	2,427650693	2,044580955	0,383069739
209803_s_at	PHLDA2	3,489810047	3,106874588	0,382935459
209758_s_at	MFAP5	-1,137208348	-1,520022123	0,382813775
1559585_at	DDX60L	-1,137208348	-1,520022123	0,382813775
1552491_at	IDI2	-1,137208348	-1,520022123	0,382813775
238682_at	CCDC96	-1,137208348	-1,520022123	0,382813775
204665_at	SIKE1	-1,137208348	-1,520022123	0,382813775
217442_at	LOC100131825	-1,137208348	-1,520022123	0,382813775
217036_at	-	-1,137208348	-1,520022123	0,382813775
244216_at	-	-1,137208348	-1,520022123	0,382813775
237394_at	-	-1,137208348	-1,520022123	0,382813775
241658_at	-	-1,137208348	-1,520022123	0,382813775
221372_s_at	P2RX2	-1,137208348	-1,520022123	0,382813775
229995_at	SYCN	-1,137208348	-1,520022123	0,382813775
239782_at	RBP1	-1,137208348	-1,520022123	0,382813775
1563745_a_at	LOC283050	-1,137208348	-1,520022123	0,382813775
234053_at	-	-1,137208348	-1,520022123	0,382813775

233652_at	-	-1,137208348	-1,520022123	0,382813775
222862_s_at	AK5	-1,137208348	-1,520022123	0,382813775
217102_at	-	-1,137208348	-1,520022123	0,382813775
228586_at	-	-1,137208348	-1,520022123	0,382813775
213949_s_at	DOHH	-1,137208348	-1,520022123	0,382813775
1554711_at	CALHM3	-1,137208348	-1,520022123	0,382813775
1552349_a_at	PRSS33	-1,137208348	-1,520022123	0,382813775
236118_at	LOC100128893	-1,137208348	-1,520022123	0,382813775
231826_at	RALGAPA2	-1,137208348	-1,520022123	0,382813775
235402_at	PPP1R32	-1,137208348	-1,520022123	0,382813775
210380_s_at	CACNA1G	-1,137208348	-1,520022123	0,382813775
218082_s_at	UBP1	3,697863752	3,315052527	0,382811225
219451_at	MSRB2	0,648195588	0,265475485	0,382720103
210910_s_at	POMZP3	0,648195588	0,265475485	0,382720103
235423_at	ORC2	2,159837409	1,777135134	0,382702275
204024_at	OSGIN2	0,319287178	-0,063377083	0,382664261
219536_s_at	ZFP64	0,319287178	-0,063377083	0,382664261
219330_at	VANGL1	1,225123479	0,842599219	0,38252426
211967_at	TMEM123	6,512381658	6,129991113	0,382390545
222734_at	WARS2	1,857989965	1,475630127	0,382359838
238719_at	MIR3661 /// PPP2(	1,857989965	1,475630127	0,382359838
218562_s_at	TMEM57	1,898032047	1,516067292	0,381964755
235507_at	PCMTD1	1,898032047	1,516067292	0,381964755
218042_at	COPS4	4,42686741	4,044909869	0,381957541
213208_at	KIAA0240	1,182925501	0,80114751	0,381777991
205441_at	OCEL1	1,182925501	0,80114751	0,381777991
208929_x_at	RPL13 /// SNORD6	8,077158539	7,695729912	0,381428627
222800_at	TRNAU1AP	1,139057614	0,757638486	0,381419128
209481_at	SNRK	3,422378845	3,041114664	0,381264181
204106_at	TESK1	1,640193838	1,259081178	0,38111266
219341_at	CLN8	-0,280273599	-0,661178575	0,380904975
1564373_a_at	LOC283887	-0,280273599	-0,661178575	0,380904975
207403_at	IRS4	-0,280273599	-0,661178575	0,380904975
203304_at	BAMBI	-0,280273599	-0,661178575	0,380904975
224117_at	LOC284912 /// ME	-0,280273599	-0,661178575	0,380904975
230209_at	ZXDC	-0,280273599	-0,661178575	0,380904975
1563878_a_at	LOC338963	-0,280273599	-0,661178575	0,380904975
242992_at	ZNF551	-0,280273599	-0,661178575	0,380904975
226870_at	COMTD1	0,707657549	0,326777209	0,380880339
239026_x_at	AGAP3	1,047341799	0,666486137	0,380855662
218254_s_at	SAR1B	4,548572168	4,167778161	0,380794007
219481_at	TTC13	3,218770562	2,83813104	0,380639522
203159_at	GLS	2,537731447	2,157148588	0,380582859
1561887_at	-	-0,551284523	-0,931539147	0,380254624
228192_at	MNF1	-0,551284523	-0,931539147	0,380254624
210744_s_at	IL5RA	-0,551284523	-0,931539147	0,380254624
222295_x_at	-	-0,551284523	-0,931539147	0,380254624
240435_at	ALDH1A2	-0,551284523	-0,931539147	0,380254624
221115_s_at	LENEP	-0,551284523	-0,931539147	0,380254624
236073_at	EPHA10	-0,551284523	-0,931539147	0,380254624

1559697_a_at	-	-1,624955693	-2,005029581	0,380073888
1554362_at	BTG4	-1,624955693	-2,005029581	0,380073888
243588_at	-	-1,624955693	-2,005029581	0,380073888
1560798_at	-	-1,624955693	-2,005029581	0,380073888
214796_at	KIAA1456	-1,624955693	-2,005029581	0,380073888
234593_at	-	-1,624955693	-2,005029581	0,380073888
237720_at	ASB4	-1,624955693	-2,005029581	0,380073888
237164_at	-	-1,624955693	-2,005029581	0,380073888
234839_at	-	-1,624955693	-2,005029581	0,380073888
236383_at	-	-1,624955693	-2,005029581	0,380073888
243846_x_at	-	-1,624955693	-2,005029581	0,380073888
215229_at	LOC100129973	-1,624955693	-2,005029581	0,380073888
239113_at	LOC100507468	-1,624955693	-2,005029581	0,380073888
1562383_at	MIR4313	-1,624955693	-2,005029581	0,380073888
1562793_at	-	-1,624955693	-2,005029581	0,380073888
244677_at	-	-1,624955693	-2,005029581	0,380073888
238626_at	ANKS6	-1,624955693	-2,005029581	0,380073888
236068_s_at	C3orf14	-1,624955693	-2,005029581	0,380073888
237436_at	-	-1,624955693	-2,005029581	0,380073888
1553949_at	IQSEC3	-1,624955693	-2,005029581	0,380073888
1555033_a_at	RGS12	-1,624955693	-2,005029581	0,380073888
233147_at	-	-1,624955693	-2,005029581	0,380073888
1561101_at	JAKMIP2-AS1	-1,624955693	-2,005029581	0,380073888
1560241_at	LOC100506559	-1,624955693	-2,005029581	0,380073888
220909_at	TRIM46	-1,624955693	-2,005029581	0,380073888
244266_at	AKR1C1	-1,624955693	-2,005029581	0,380073888
235963_at	-	-1,624955693	-2,005029581	0,380073888
1565776_at	-	-1,624955693	-2,005029581	0,380073888
223710_at	CCL26	-1,624955693	-2,005029581	0,380073888
206243_at	TIMP4	-1,624955693	-2,005029581	0,380073888
211726_s_at	FMO2	-1,624955693	-2,005029581	0,380073888
1560703_at	C17orf108	-1,624955693	-2,005029581	0,380073888
211876_x_at	PCDHGA10 /// PCI	-1,624955693	-2,005029581	0,380073888
206623_at	PDE6A	-1,624955693	-2,005029581	0,380073888
1561984_at	LOC100505583	-1,624955693	-2,005029581	0,380073888
1555140_a_at	BCL2L2	-1,624955693	-2,005029581	0,380073888
1557523_at	ATP6AP1L /// FLJ4	-1,624955693	-2,005029581	0,380073888
1557380_at	AGAP11	-1,624955693	-2,005029581	0,380073888
216549_s_at	TBC1D22B	-1,624955693	-2,005029581	0,380073888
230644_at	LRFN5	-1,624955693	-2,005029581	0,380073888
1566955_at	-	-1,624955693	-2,005029581	0,380073888
213014_at	LOC644172 /// MA	-1,624955693	-2,005029581	0,380073888
1559170_at	-	-1,624955693	-2,005029581	0,380073888
220960_x_at	RPL22	8,073510812	7,693658823	0,379851989
1557119_a_at	ZNF575	1,458255986	1,078619386	0,3796366
206076_at	LRRC23	0,20740896	-0,172194225	0,379603185
1558670_at	-	0,20740896	-0,172194225	0,379603185
202372_at	AURKAPS1 /// RAB	3,353217129	2,973964204	0,379252925
241061_at	-	-2,569309811	-2,948507524	0,379197713
35150_at	CD40	1,664317239	1,285238413	0,379078825

209786_at	HMGN4	4,839270685	4,460645086	0,378625599
239839_at	ZNF555	-0,856259172	-1,234835326	0,378576154
234093_at	ZNF326	-0,856259172	-1,234835326	0,378576154
205040_at	ORM1	-0,856259172	-1,234835326	0,378576154
241178_at	-	-0,856259172	-1,234835326	0,378576154
207019_s_at	AKAP4	-0,856259172	-1,234835326	0,378576154
238338_at	-	-0,856259172	-1,234835326	0,378576154
1563274_at	-	-0,856259172	-1,234835326	0,378576154
1554798_at	SNED1	-0,856259172	-1,234835326	0,378576154
221914_at	SYN1	-0,856259172	-1,234835326	0,378576154
244299_at	-	-0,856259172	-1,234835326	0,378576154
1553258_at	FLJ30679	-0,856259172	-1,234835326	0,378576154
201295_s_at	WSB1	-0,856259172	-1,234835326	0,378576154
1554470_s_at	ZBTB44	0,764143511	0,385582632	0,378560879
1554933_at	PSIP1	-0,994186487	-1,372734086	0,378547599
1556573_s_at	LOC286178	-0,994186487	-1,372734086	0,378547599
239958_at	LOC253039	-0,994186487	-1,372734086	0,378547599
220665_at	LUZP4	-0,994186487	-1,372734086	0,378547599
235633_at	-	-0,994186487	-1,372734086	0,378547599
232996_at	CCDC120	-0,994186487	-1,372734086	0,378547599
231625_at	SLC22A9	-0,994186487	-1,372734086	0,378547599
1561689_at	-	-0,994186487	-1,372734086	0,378547599
204539_s_at	CELSR1	-0,994186487	-1,372734086	0,378547599
216692_at	-	-0,994186487	-1,372734086	0,378547599
243245_at	LOC100506446	-0,994186487	-1,372734086	0,378547599
219657_s_at	KLF3	-0,994186487	-1,372734086	0,378547599
1569894_at	PPP2R3C	-0,994186487	-1,372734086	0,378547599
206720_at	MGAT5	-0,994186487	-1,372734086	0,378547599
1555907_at	LOC100130776	-0,994186487	-1,372734086	0,378547599
204693_at	CDC42EP1	-0,994186487	-1,372734086	0,378547599
206831_s_at	ARSD	-0,994186487	-1,372734086	0,378547599
1561454_at	-	-0,994186487	-1,372734086	0,378547599
215184_at	DAPK2	-0,994186487	-1,372734086	0,378547599
235160_at	ATF7	-0,994186487	-1,372734086	0,378547599
222778_s_at	WHSC1	2,692288672	2,314488991	0,377799681
205401_at	AGPS	2,545254728	2,167793428	0,3774613
223432_at	OSBP2	0,08619576	-0,290694975	0,376890735
216174_at	HCRP1	0,08619576	-0,290694975	0,376890735
209115_at	UBA3	4,94671006	4,570176798	0,376533262
240401_at	-	-1,893506789	-2,27001641	0,376509621
224095_at	LOC100128175	-1,893506789	-2,27001641	0,376509621
208181_at	HIST1H4A /// HIST	-1,893506789	-2,27001641	0,376509621
1569080_at	RNF165	-1,893506789	-2,27001641	0,376509621
231738_at	PCDHB7	-1,893506789	-2,27001641	0,376509621
217454_at	-	-1,893506789	-2,27001641	0,376509621
206000_at	MEP1A	-1,893506789	-2,27001641	0,376509621
244605_at	-	-1,893506789	-2,27001641	0,376509621
227764_at	LYPD6	-1,893506789	-2,27001641	0,376509621
224192_at	FCRL2	-1,893506789	-2,27001641	0,376509621
1559975_at	BTG1	-1,893506789	-2,27001641	0,376509621

227481_at	CNKSR3	-1,893506789	-2,27001641	0,376509621
206189_at	UNC5C	-1,893506789	-2,27001641	0,376509621
213425_at	WNT5A	-1,893506789	-2,27001641	0,376509621
237593_at	-	-1,893506789	-2,27001641	0,376509621
241572_at	PDZD9	-1,893506789	-2,27001641	0,376509621
241141_at	BMP6	-1,893506789	-2,27001641	0,376509621
208247_at	ERC2-IT1	-1,893506789	-2,27001641	0,376509621
206305_s_at	C8A	-1,893506789	-2,27001641	0,376509621
224999_at	EGFR	-1,893506789	-2,27001641	0,376509621
231676_s_at	-	-1,893506789	-2,27001641	0,376509621
1559079_at	LINC00567	-1,893506789	-2,27001641	0,376509621
234385_at	-	-1,893506789	-2,27001641	0,376509621
215702_s_at	CFTR	-1,893506789	-2,27001641	0,376509621
231623_at	TMEM174	-1,893506789	-2,27001641	0,376509621
215935_at	FAM75A1 /// FAM	-1,893506789	-2,27001641	0,376509621
228224_at	PRELP	-1,893506789	-2,27001641	0,376509621
237564_at	-	-1,893506789	-2,27001641	0,376509621
216040_x_at	-	-1,893506789	-2,27001641	0,376509621
1555942_a_at	MIR205HG	-1,893506789	-2,27001641	0,376509621
229005_at	MCTP2	-1,893506789	-2,27001641	0,376509621
226338_at	TMEM55A	1,802733148	1,426280426	0,376452721
216804_s_at	PDLIM5	1,802733148	1,426280426	0,376452721
204892_x_at	EEF1A1 /// LOC10(	9,023558928	8,647318494	0,376240434
239275_at	FRMPD2 /// FRMP	-1,289805289	-1,665791776	0,375986487
1556387_at	LOC100507389	-1,289805289	-1,665791776	0,375986487
1568832_a_at	-	-1,289805289	-1,665791776	0,375986487
216651_s_at	GAD2	-1,289805289	-1,665791776	0,375986487
208600_s_at	GPR39	-1,289805289	-1,665791776	0,375986487
1565730_at	-	-1,289805289	-1,665791776	0,375986487
238817_at	RIMBP2	-1,289805289	-1,665791776	0,375986487
1569789_at	-	-1,289805289	-1,665791776	0,375986487
1569474_at	-	-1,289805289	-1,665791776	0,375986487
223654_s_at	CELF4	-1,289805289	-1,665791776	0,375986487
1555662_s_at	DAOA	-1,289805289	-1,665791776	0,375986487
1561988_at	LOC286068	-1,289805289	-1,665791776	0,375986487
239470_at	C15orf56	-1,289805289	-1,665791776	0,375986487
208564_at	KCNA2	-1,289805289	-1,665791776	0,375986487
230830_at	OSTBETA	-1,289805289	-1,665791776	0,375986487
235748_s_at	-	-1,289805289	-1,665791776	0,375986487
1558001_s_at	ARID5B	-1,289805289	-1,665791776	0,375986487
1553327_a_at	BEND7	-1,289805289	-1,665791776	0,375986487
208533_at	SOX1	-1,289805289	-1,665791776	0,375986487
223127_s_at	C1orf21	-1,289805289	-1,665791776	0,375986487
216787_at	-	-1,289805289	-1,665791776	0,375986487
207274_at	CHRNE	-1,289805289	-1,665791776	0,375986487
240851_at	-	-1,289805289	-1,665791776	0,375986487
1555256_at	EVC2	-1,289805289	-1,665791776	0,375986487
233119_at	PRR5L	-1,289805289	-1,665791776	0,375986487
220119_at	EPB41L4A	-1,289805289	-1,665791776	0,375986487
211788_s_at	TREX2	-1,289805289	-1,665791776	0,375986487

215512_at	06.03.15	-1,289805289	-1,665791776	0,375986487
238014_at	TMEM194B	-1,289805289	-1,665791776	0,375986487
244694_at	IGLON5	-1,289805289	-1,665791776	0,375986487
235002_at	NUDT16	-1,289805289	-1,665791776	0,375986487
200099_s_at	RPS3A /// SNORD7	8,573734986	8,197864821	0,375870165
225460_at	SEC22C	4,588489926	4,21289113	0,375598795
203353_s_at	MBD1	3,35759121	2,982339418	0,375251792
212150_at	EFR3A	4,007783021	3,632850102	0,374932919
218769_s_at	ANKRA2	3,252865595	2,878165848	0,374699748
222663_at	RIOK2	1,898032047	1,523429058	0,374602989
222334_at	C1orf186 /// LOC1	-0,043925712	-0,418248858	0,374323146
217596_at	UPF3A	-0,043925712	-0,418248858	0,374323146
219396_s_at	MIR631 /// NEIL1	-0,043925712	-0,418248858	0,374323146
225854_x_at	IRF2BP2	-0,043925712	-0,418248858	0,374323146
227427_at	ARHGEF25	-0,043925712	-0,418248858	0,374323146
235375_x_at	TTC9B	-0,437449947	-0,811769547	0,374319599
220813_at	CYSLTR2	-0,437449947	-0,811769547	0,374319599
212294_at	GNG12	-0,437449947	-0,811769547	0,374319599
205294_at	BAIAP2	-0,437449947	-0,811769547	0,374319599
1554229_at	CREBRF	-0,437449947	-0,811769547	0,374319599
241230_at	CA12	-0,437449947	-0,811769547	0,374319599
221196_x_at	BRCC3	2,484824532	2,110584371	0,374240161
207911_s_at	TGM5	1,071336699	0,697221375	0,374115324
238227_at	-	-0,180990326	-0,554696666	0,37370634
223659_at	TMPRSS13	-0,180990326	-0,554696666	0,37370634
1554989_at	KIAA0317	-0,180990326	-0,554696666	0,37370634
220823_at	LOC729164	-0,180990326	-0,554696666	0,37370634
234308_at	TUBGCP6	-0,180990326	-0,554696666	0,37370634
237618_at	-	-0,180990326	-0,554696666	0,37370634
206656_s_at	C20orf3	3,581038515	3,207623136	0,373415379
201858_s_at	SRGN	6,322591704	5,949212274	0,37337943
243822_at	-	-1,537851782	-1,911206569	0,373354787
216073_at	ANKRD34C	-1,537851782	-1,911206569	0,373354787
232795_at	-	-1,537851782	-1,911206569	0,373354787
236996_at	-	-1,537851782	-1,911206569	0,373354787
1569832_at	LOC100131655	-1,537851782	-1,911206569	0,373354787
242936_at	-	-1,537851782	-1,911206569	0,373354787
207148_x_at	MYOZ2	-1,537851782	-1,911206569	0,373354787
234819_at	-	-1,537851782	-1,911206569	0,373354787
238934_at	-	-1,537851782	-1,911206569	0,373354787
215331_at	MYH15	-1,537851782	-1,911206569	0,373354787
208399_s_at	EDN3	-1,537851782	-1,911206569	0,373354787
224212_s_at	PCDHA1 /// PCDH/	-1,537851782	-1,911206569	0,373354787
1552538_a_at	KIF6	-1,537851782	-1,911206569	0,373354787
244839_at	TTN	-1,537851782	-1,911206569	0,373354787
213155_at	LOC339166 /// WS	-1,537851782	-1,911206569	0,373354787
216078_at	-	-1,537851782	-1,911206569	0,373354787
238823_at	FMNL3	-1,537851782	-1,911206569	0,373354787
208470_s_at	HP /// HPR	-1,537851782	-1,911206569	0,373354787
207470_at	-	-1,537851782	-1,911206569	0,373354787

239864_at	LOC100130175	-1,537851782	-1,911206569	0,373354787
233367_at	-	-1,537851782	-1,911206569	0,373354787
211357_s_at	ALDOB	-1,537851782	-1,911206569	0,373354787
224239_at	DEFB103A /// DEFI	-1,537851782	-1,911206569	0,373354787
232508_at	ZMIZ1	-1,537851782	-1,911206569	0,373354787
217163_at	ESR1	-1,537851782	-1,911206569	0,373354787
231607_at	LOC100507480	-1,537851782	-1,911206569	0,373354787
216964_at	USP22	-1,537851782	-1,911206569	0,373354787
1563182_at	ACVR1C	-1,537851782	-1,911206569	0,373354787
218246_at	MUL1	2,248155141	1,874839058	0,373316083
218228_s_at	TNKS2	2,642918178	2,269637658	0,37328052
219299_at	TRMT12	2,692288672	2,31904917	0,373239502
232817_at	-	-2,163157732	-2,536339282	0,37318155
211959_at	IGFBP5	-2,163157732	-2,536339282	0,37318155
221886_at	DENND2A	-2,163157732	-2,536339282	0,37318155
1555794_at	ARL17A /// ARL17I	-2,163157732	-2,536339282	0,37318155
215894_at	PTGDR	-2,163157732	-2,536339282	0,37318155
240770_at	TMEM171	-2,163157732	-2,536339282	0,37318155
1559284_at	-	-2,163157732	-2,536339282	0,37318155
237354_at	PDXDC1	-2,163157732	-2,536339282	0,37318155
1555130_at	PER3	-2,163157732	-2,536339282	0,37318155
214515_at	OR1E1	-2,163157732	-2,536339282	0,37318155
234279_at	-	-2,163157732	-2,536339282	0,37318155
238251_at	-	-2,163157732	-2,536339282	0,37318155
1561854_at	-	-2,163157732	-2,536339282	0,37318155
233184_at	EPHA6	-2,163157732	-2,536339282	0,37318155
229964_at	C9orf152	-2,163157732	-2,536339282	0,37318155
234600_at	-	-2,163157732	-2,536339282	0,37318155
217201_at	RASAL2	-2,163157732	-2,536339282	0,37318155
1564139_at	A2M-AS1	-2,163157732	-2,536339282	0,37318155
213068_at	DPT	-2,163157732	-2,536339282	0,37318155
1556945_a_at	-	-2,163157732	-2,536339282	0,37318155
227949_at	PHACTR3	-2,163157732	-2,536339282	0,37318155
203887_s_at	THBD	-2,163157732	-2,536339282	0,37318155
214837_at	ALB	-2,163157732	-2,536339282	0,37318155
1559374_at	-	-2,163157732	-2,536339282	0,37318155
243896_at	WDR96	-2,163157732	-2,536339282	0,37318155
213277_at	ZFP36L1	-2,163157732	-2,536339282	0,37318155
238577_s_at	TSHZ2	-2,163157732	-2,536339282	0,37318155
230425_at	EPHB1	-2,163157732	-2,536339282	0,37318155
219757_s_at	C14orf101	1,716003175	1,343200523	0,372802651
222759_at	SUV420H1	2,678352494	2,305621062	0,372731432
201738_at	EIF1B	5,669337368	5,296615486	0,372721882
40446_at	PHF1	3,639780622	3,267176796	0,372603826
225777_at	SAPCD2	2,978070706	2,605630202	0,372440504
232460_at	HGS	0,282358733	-0,089635713	0,371994446
210358_x_at	GATA2	0,282358733	-0,089635713	0,371994446
221488_s_at	CUTA	5,802420557	5,430470976	0,371949581
40016_g_at	MAST4	0,678232894	0,306312757	0,371920137
211988_at	SMARCE1	5,454674816	5,082835835	0,371838981

218108_at	UBR7	4,308320282	3,936585452	0,37173483
229248_at	UCHL5	1,97249974	1,601076277	0,371423463
204461_x_at	RAD1	3,843767728	3,472563131	0,371204597
234792_x_at	IGHA1 /// IGHV4-3	-0,730013898	-1,100943374	0,370929476
240397_x_at	-	-0,730013898	-1,100943374	0,370929476
238171_at	SLC25A30	-0,730013898	-1,100943374	0,370929476
235718_at	SRL	-0,730013898	-1,100943374	0,370929476
244538_at	KCTD19	-0,730013898	-1,100943374	0,370929476
230522_s_at	C9orf100	-0,730013898	-1,100943374	0,370929476
209509_s_at	DPAGT1	2,492739983	2,121960601	0,370779382
223026_s_at	VPS29	5,770822994	5,400651561	0,370171433
214006_s_at	GGCX	2,725848647	2,355777584	0,370071063
226229_s_at	SSU72	0,735999505	0,366031214	0,369968291
1569501_at	-	0,167727503	-0,201789721	0,369517224
205879_x_at	RET	0,167727503	-0,201789721	0,369517224
202044_at	ARHGAP35	0,167727503	-0,201789721	0,369517224
225545_at	EEF2K	3,065977723	2,696595318	0,369382405
235572_at	SPC24	2,374261973	2,004917973	0,369344001
204675_at	SRD5A1	3,418470674	3,049587049	0,368883625
203553_s_at	MAP4K5	1,88491011	1,516067292	0,368842819
231377_at	CXorf65	0,524545436	0,155747281	0,368798156
212517_at	ATRN	1,225123479	0,85655369	0,368569789
207629_s_at	ARHGEF2	1,225123479	0,85655369	0,368569789
213292_s_at	SNX13	2,042564225	1,674071917	0,368492308
1560854_s_at	ZNF107	0,847529938	0,479089184	0,368440754
218781_at	SMC6	4,574442657	4,206178	0,368264656
1561239_at	-	-1,982324124	-2,350444759	0,368120636
1557139_at	DDX11-AS1	-1,982324124	-2,350444759	0,368120636
211437_at	-	-1,982324124	-2,350444759	0,368120636
240857_at	DNAH9	-1,982324124	-2,350444759	0,368120636
1561881_at	LOC100505718	-1,982324124	-2,350444759	0,368120636
1560772_a_at	-	-1,982324124	-2,350444759	0,368120636
1569641_at	-	-1,982324124	-2,350444759	0,368120636
1570465_at	-	-1,982324124	-2,350444759	0,368120636
221305_s_at	UGT1A8 /// UGT1/	-1,982324124	-2,350444759	0,368120636
1562640_at	HOXA-AS2	-1,982324124	-2,350444759	0,368120636
241244_at	-	-1,982324124	-2,350444759	0,368120636
235981_at	C8orf22	-1,982324124	-2,350444759	0,368120636
1557821_at	LOC283547	-1,982324124	-2,350444759	0,368120636
214090_at	PAWR	-1,982324124	-2,350444759	0,368120636
235161_at	LOC100506451	-1,982324124	-2,350444759	0,368120636
218899_s_at	BAALC	-1,982324124	-2,350444759	0,368120636
207918_s_at	TSPY1 /// TSPY10 /	-1,982324124	-2,350444759	0,368120636
229912_at	SDK1	-1,982324124	-2,350444759	0,368120636
237776_at	LOC100652904	-1,982324124	-2,350444759	0,368120636
242960_at	-	-1,982324124	-2,350444759	0,368120636
219093_at	PID1	-1,982324124	-2,350444759	0,368120636
237748_at	SCGB2B2	-1,982324124	-2,350444759	0,368120636
1554633_a_at	MYT1L	-1,982324124	-2,350444759	0,368120636
1556516_at	-	-1,982324124	-2,350444759	0,368120636

219529_at	CLIC3	-1,982324124	-2,350444759	0,368120636
1556514_at	C12orf74	-1,982324124	-2,350444759	0,368120636
221728_x_at	XIST	-1,982324124	-2,350444759	0,368120636
221681_s_at	DSPP	-1,982324124	-2,350444759	0,368120636
218402_s_at	HPS4	1,802733148	1,434743608	0,367989539
212227_x_at	EIF1	7,178325763	6,810456195	0,367869568
224328_s_at	LCE3D	0,045517965	-0,322340048	0,367858013
208163_s_at	OSBPL7	0,045517965	-0,322340048	0,367858013
234975_at	GSPT1	0,045517965	-0,322340048	0,367858013
231946_at	ZFHX2	0,045517965	-0,322340048	0,367858013
224746_at	KIAA1522	0,045517965	-0,322340048	0,367858013
219907_at	FRS3	0,045517965	-0,322340048	0,367858013
209288_s_at	CDC42EP3	2,118167848	1,750483134	0,367684715
223439_at	NKAP	2,866549889	2,498884538	0,367665351
203927_at	NFKBIE	3,446505209	3,078940895	0,367564314
215464_s_at	P2RX5-TAX1BP3 //	1,983901861	1,616354037	0,367547824
218873_at	GON4L	2,138605583	1,771157334	0,367448249
223198_x_at	COMMD5	3,430413158	3,063097042	0,367316116
222417_s_at	SNX5	5,302218259	4,934989076	0,367229184
218733_at	MSL2	3,374552989	3,007560601	0,366992388
225268_at	KPNA4	4,558198545	4,191278093	0,366920452
223255_at	G2E3	2,096248267	1,72935563	0,366892637
218099_at	TEX2	2,096248267	1,72935563	0,366892637
203974_at	HDHD1	4,372691748	4,005922659	0,366769089
228928_x_at	BANP	3,045068376	2,678678228	0,366390148
225531_at	CABLES1	1,000459215	0,634301128	0,366158087
219966_x_at	BANP	3,710746795	3,344597303	0,366149492
212790_x_at	RPL13A /// RPL13A	8,443957558	8,077865221	0,366092337
208284_x_at	GGT1 /// GGT2 ///	2,671492308	2,305621062	0,365871246
212025_s_at	FLII	2,507817181	2,142313983	0,365503198
1554036_at	ZBTB24	0,58788177	0,222432814	0,365448956
217560_at	GGA1	0,58788177	0,222432814	0,365448956
202759_s_at	AKAP2 /// PALM2-	2,582572508	2,2171284	0,365444108
219505_at	CECR1	2,392346173	2,026957145	0,365389028
225502_at	DOCK8	4,662628176	4,297303611	0,365324564
222537_s_at	CDC42SE1	2,884644843	2,519368159	0,365276684
225917_at	-	2,657308324	2,292099323	0,365209001
206335_at	GALNS	2,966903803	2,601822502	0,365081301
201216_at	ERP29	5,35957938	4,994606965	0,364972415
222179_at	CDC5L	-0,332405896	-0,697182596	0,364776701
231892_at	C9orf100	-0,332405896	-0,697182596	0,364776701
216965_x_at	SPG20	-0,332405896	-0,697182596	0,364776701
220689_at	-	-0,332405896	-0,697182596	0,364776701
208440_at	C3orf27	-0,332405896	-0,697182596	0,364776701
213464_at	SHC2	-0,332405896	-0,697182596	0,364776701
213783_at	MFNG	-0,332405896	-0,697182596	0,364776701
217183_at	LDLR	-0,332405896	-0,697182596	0,364776701
227675_at	LRSAM1	-0,332405896	-0,697182596	0,364776701
1568743_at	ATP10A	-0,332405896	-0,697182596	0,364776701
208232_x_at	NRG1	-0,332405896	-0,697182596	0,364776701

228309_at	-	-0,332405896	-0,697182596	0,364776701
203816_at	DGUOK	3,562907192	3,198229785	0,364677407
222396_at	HN1	5,004299749	4,639627194	0,364672554
224874_at	POLR1D	4,214272326	3,850052829	0,364219497
240648_at	-	-0,609040214	-0,972893339	0,363853125
210444_at	NPY6R	-0,609040214	-0,972893339	0,363853125
238074_at	WDR27	-0,609040214	-0,972893339	0,363853125
215906_at	-	-0,609040214	-0,972893339	0,363853125
1555139_a_at	OTUD7B	-0,609040214	-0,972893339	0,363853125
203106_s_at	VPS41	-0,609040214	-0,972893339	0,363853125
239094_at	LOC730961	-0,609040214	-0,972893339	0,363853125
243670_at	-	-0,609040214	-0,972893339	0,363853125
222695_s_at	AXIN2	-0,609040214	-0,972893339	0,363853125
225089_at	USP40	-0,609040214	-0,972893339	0,363853125
214933_at	CACNA1A	-0,609040214	-0,972893339	0,363853125
211228_s_at	RAD17	3,317909135	2,954216214	0,363692922
212282_at	TMEM97	4,942322973	4,578636564	0,363686409
244183_x_at	PCDHB3	-2,416625063	-2,780225148	0,363600085
222202_at	-	-2,416625063	-2,780225148	0,363600085
224094_at	-	-2,416625063	-2,780225148	0,363600085
1563000_at	-	-2,416625063	-2,780225148	0,363600085
1569254_s_at	INTS4	-2,416625063	-2,780225148	0,363600085
1553546_at	PRO2012	-2,416625063	-2,780225148	0,363600085
1562286_at	-	-2,416625063	-2,780225148	0,363600085
232541_at	-	-2,416625063	-2,780225148	0,363600085
1555492_a_at	BEST3	-2,416625063	-2,780225148	0,363600085
234587_at	-	-2,416625063	-2,780225148	0,363600085
230144_at	GRIA3	-2,416625063	-2,780225148	0,363600085
242028_at	ZNF709	-2,416625063	-2,780225148	0,363600085
231655_x_at	SERPINB6	-2,416625063	-2,780225148	0,363600085
1569371_at	LRRC59	-2,416625063	-2,780225148	0,363600085
205978_at	KL	-2,416625063	-2,780225148	0,363600085
206066_s_at	RAD51C	3,418470674	3,05501254	0,363458134
212988_x_at	ACTG1	8,448597351	8,085177682	0,363419669
223590_at	ZNF700	1,871521385	1,508107415	0,36341397
223009_at	LAMTOR1	4,819724179	4,456437907	0,363286272
209019_s_at	PINK1	1,830468611	1,46719264	0,363275972
243414_at	-	0,425036312	0,061773582	0,36326273
208992_s_at	STAT3	4,377039749	4,013964233	0,363075516
222673_x_at	FAM122B	4,446069945	4,083008429	0,363061516
225843_at	ZFYVE19	1,246401134	0,883374248	0,363026885
229298_at	KBTBD7	1,383446753	1,020514937	0,362931816
202671_s_at	PDXK	2,890618076	2,527778509	0,362839567
218336_at	PFDN2	4,82490062	4,462314713	0,362585907
235747_at	SLC25A16	1,948628481	1,586121802	0,362506679
1556852_a_at	LOC100506609	0,648195588	0,286279868	0,36191572
212918_at	RECQL	4,413844848	4,052245355	0,361599493
222672_at	LYRM4	1,88491011	1,523429058	0,361481052
210218_s_at	SP100	1,88491011	1,523429058	0,361481052
1556715_at	PRPSAP1	0,244681185	-0,116767475	0,36144866

216668_at	-	0,244681185	-0,116767475	0,36144866
1554827_a_at	ADCY7	0,244681185	-0,116767475	0,36144866
224359_s_at	HOOK3	0,244681185	-0,116767475	0,36144866
201806_s_at	ATXN2L	0,244681185	-0,116767475	0,36144866
1552691_at	ARL11	-2,495152701	-2,856570137	0,361417436
219070_s_at	MOSPD3	1,20396005	0,842599219	0,361360831
202062_s_at	SEL1L	1,760255626	1,399283506	0,36097212
218316_at	TIMM9	2,698955954	2,338020736	0,360935218
231723_at	SNX12	0,707657549	0,346964736	0,360692813
202098_s_at	PRMT2	2,771643417	2,411206536	0,360436882
212528_at	DESI1	3,204847647	2,844606929	0,360240718
220408_x_at	FAM48A	3,096793374	2,736663738	0,360129636
217736_s_at	EIF2AK1	4,915049349	4,555087262	0,359962086
205213_at	ACAP1	3,398333742	3,038411131	0,359922611
243782_at	-	-1,06282519	-1,422257026	0,359431836
1561292_at	-	-1,06282519	-1,422257026	0,359431836
216010_x_at	FUT3	-1,06282519	-1,422257026	0,359431836
239813_at	IQCH	-1,06282519	-1,422257026	0,359431836
1564178_at	LOC283033	-1,06282519	-1,422257026	0,359431836
223572_at	HHATL	-1,06282519	-1,422257026	0,359431836
228182_at	ADCY5	-1,06282519	-1,422257026	0,359431836
236833_at	TTC16	-1,06282519	-1,422257026	0,359431836
1558874_a_at	-	-1,06282519	-1,422257026	0,359431836
207301_at	EFNA5	-1,06282519	-1,422257026	0,359431836
243385_at	-	-1,06282519	-1,422257026	0,359431836
1556779_s_at	-	-1,06282519	-1,422257026	0,359431836
242998_at	RDH12	-1,06282519	-1,422257026	0,359431836
1564974_at	KRTAP8-1	-1,06282519	-1,422257026	0,359431836
1552960_at	LRRC15	-1,06282519	-1,422257026	0,359431836
216846_at	CYAT1	-1,06282519	-1,422257026	0,359431836
1554708_s_at	SPATA6L	-1,06282519	-1,422257026	0,359431836
1562940_at	-	-1,06282519	-1,422257026	0,359431836
234415_x_at	-	-1,06282519	-1,422257026	0,359431836
243975_at	-	-1,06282519	-1,422257026	0,359431836
221469_at	GPR32	-1,06282519	-1,422257026	0,359431836
1560006_a_at	LOC646762	-1,06282519	-1,422257026	0,359431836
234199_at	-	-1,06282519	-1,422257026	0,359431836
1561038_at	ZNF81	-1,06282519	-1,422257026	0,359431836
1569443_s_at	-	-1,06282519	-1,422257026	0,359431836
214485_at	ODF1	-1,06282519	-1,422257026	0,359431836
204358_s_at	FLRT2 /// LOC1005	-1,06282519	-1,422257026	0,359431836
222560_at	LANCL2	-1,06282519	-1,422257026	0,359431836
202509_s_at	TNFAIP2	-1,06282519	-1,422257026	0,359431836
1556332_at	-	-1,06282519	-1,422257026	0,359431836
210094_s_at	PARD3	-1,06282519	-1,422257026	0,359431836
218309_at	CAMK2N1	-1,06282519	-1,422257026	0,359431836
64440_at	IL17RC	1,071810942	0,712384349	0,359426594
203307_at	GNL1	1,071336699	0,711934445	0,359402253
218699_at	RAB7L1	4,043821727	3,684444287	0,35937744
212300_at	TXLNA	2,392346173	2,03313004	0,359216133

1560250_s_at	LOC284242	-1,452713826	-1,811780055	0,359066229
1560937_at	RSU1	-1,452713826	-1,811780055	0,359066229
207284_s_at	ASPH	-1,452713826	-1,811780055	0,359066229
207550_at	MPL	-1,452713826	-1,811780055	0,359066229
1565784_at	LOC100128262	-1,452713826	-1,811780055	0,359066229
234377_at	IL23A	-1,452713826	-1,811780055	0,359066229
232723_at	-	-1,452713826	-1,811780055	0,359066229
238503_at	LOC100506354	-1,452713826	-1,811780055	0,359066229
1553500_at	FBXL21	-1,452713826	-1,811780055	0,359066229
232930_at	DOCK1	-1,452713826	-1,811780055	0,359066229
206690_at	ASIC2	-1,452713826	-1,811780055	0,359066229
243967_at	AFF3	-1,452713826	-1,811780055	0,359066229
1557557_at	MATN1-AS1	-1,452713826	-1,811780055	0,359066229
227486_at	NT5E	-1,452713826	-1,811780055	0,359066229
1565846_at	-	-1,452713826	-1,811780055	0,359066229
1561906_at	-	-1,452713826	-1,811780055	0,359066229
242766_at	-	-1,452713826	-1,811780055	0,359066229
232116_at	GRHL3	-1,452713826	-1,811780055	0,359066229
236351_at	LOC389023	-1,452713826	-1,811780055	0,359066229
1570215_at	-	-1,452713826	-1,811780055	0,359066229
1569730_at	HEATR7B2	-1,452713826	-1,811780055	0,359066229
211132_at	INTS3	-1,452713826	-1,811780055	0,359066229
216673_at	TTTY1 /// TTTY1B	-1,452713826	-1,811780055	0,359066229
1564620_at	-	-1,452713826	-1,811780055	0,359066229
1559620_at	LOC441167	-1,452713826	-1,811780055	0,359066229
237819_at	CREB3L2	-1,452713826	-1,811780055	0,359066229
232313_at	TMEM132C	-1,452713826	-1,811780055	0,359066229
243174_at	-	-1,452713826	-1,811780055	0,359066229
228434_at	BTNL9	-1,452713826	-1,811780055	0,359066229
210957_s_at	AFF2	-1,452713826	-1,811780055	0,359066229
207683_at	FOXN1	-1,452713826	-1,811780055	0,359066229
224143_at	TTTY8 /// TTTY8B	-1,452713826	-1,811780055	0,359066229
236456_at	PTPN5	-1,452713826	-1,811780055	0,359066229
1554897_s_at	RHBDL2	-1,452713826	-1,811780055	0,359066229
243132_at	APTX	-1,452713826	-1,811780055	0,359066229
230551_at	KSR2	-1,452713826	-1,811780055	0,359066229
216127_at	PDIA2	-1,452713826	-1,811780055	0,359066229
217016_x_at	TMEM212	-1,452713826	-1,811780055	0,359066229
237841_at	-	-2,336678463	-2,695640852	0,358962388
233839_at	EFCAB6	-2,336678463	-2,695640852	0,358962388
240027_at	LIN7A	-2,336678463	-2,695640852	0,358962388
1555412_at	FBXL21	-2,336678463	-2,695640852	0,358962388
210729_at	NPY2R	-2,336678463	-2,695640852	0,358962388
239218_at	PDE1C	-2,336678463	-2,695640852	0,358962388
1569021_at	PIK3C2A	-2,336678463	-2,695640852	0,358962388
1567656_at	OR2H1	-2,336678463	-2,695640852	0,358962388
1554831_x_at	ALS2CR11	-2,336678463	-2,695640852	0,358962388
216917_s_at	SYCP1	-2,336678463	-2,695640852	0,358962388
1561584_at	-	-2,336678463	-2,695640852	0,358962388
1570593_at	-	-2,336678463	-2,695640852	0,358962388

217175_at	UGT2B15	-2,336678463	-2,695640852	0,358962388
243461_at	-	-2,336678463	-2,695640852	0,358962388
1570054_at	-	-2,336678463	-2,695640852	0,358962388
241870_at	-	-2,336678463	-2,695640852	0,358962388
205118_at	FPR1	-2,336678463	-2,695640852	0,358962388
1564672_at	-	-2,336678463	-2,695640852	0,358962388
231227_at	WNT5A	-2,336678463	-2,695640852	0,358962388
1566162_x_at	-	-2,336678463	-2,695640852	0,358962388
206479_at	TRPM1	-2,336678463	-2,695640852	0,358962388
220270_at	RNF17	-2,336678463	-2,695640852	0,358962388
234868_s_at	GFRA4	-2,336678463	-2,695640852	0,358962388
228794_at	XIRP2	-2,336678463	-2,695640852	0,358962388
216313_at	PCDHB17	-2,336678463	-2,695640852	0,358962388
229839_at	SCARA5	-2,336678463	-2,695640852	0,358962388
203485_at	RTN1	-2,336678463	-2,695640852	0,358962388
239548_at	NEGR1	-2,569309811	-2,928183529	0,358873718
223564_s_at	GNB1L	0,97663406	0,61779983	0,35883423
223847_s_at	ERGIC1	1,116462765	0,757638486	0,358824278
213549_at	PDZD8	1,116462765	0,757638486	0,358824278
233893_s_at	UVSSA	2,452674045	2,093850742	0,358823303
205689_at	PCNXL2	0,491906512	0,133104519	0,358801993
227303_at	ANKS3	0,491906512	0,133104519	0,358801993
202103_at	BRD4	0,127732831	-0,231045907	0,358778737
238708_at	LOC386758	0,127732831	-0,231045907	0,358778737
208226_x_at	ADAM22	0,127732831	-0,231045907	0,358778737
1565641_at	C16orf45	0,127732831	-0,231045907	0,358778737
230108_at	ERCC6	0,127732831	-0,231045907	0,358778737
225586_at	WDR85	1,898032047	1,539450683	0,358581364
240632_at	-	-0,230432956	-0,589001171	0,358568215
229009_at	SIX5	-0,230432956	-0,589001171	0,358568215
202794_at	INPP1	2,149438457	1,790914523	0,358523934
239949_at	THNSL2	-0,924000698	-1,282520722	0,358520024
1566874_at	-	-0,924000698	-1,282520722	0,358520024
244797_at	-	-0,924000698	-1,282520722	0,358520024
222082_at	ZBTB7A	-0,924000698	-1,282520722	0,358520024
1565000_a_at	TCP11L2	-0,924000698	-1,282520722	0,358520024
208562_s_at	ABCC9	-0,924000698	-1,282520722	0,358520024
210923_at	SLC1A7	-0,924000698	-1,282520722	0,358520024
237827_at	-	-0,924000698	-1,282520722	0,358520024
1556205_at	-	-0,924000698	-1,282520722	0,358520024
210636_at	PPARD	-0,924000698	-1,282520722	0,358520024
1566643_a_at	-	-0,924000698	-1,282520722	0,358520024
1558020_at	ERGIC3	-0,924000698	-1,282520722	0,358520024
221402_at	OR1F1	-0,924000698	-1,282520722	0,358520024
1561318_at	-	-0,924000698	-1,282520722	0,358520024
1562586_at	-	-0,924000698	-1,282520722	0,358520024
244353_s_at	SLC2A12	-0,924000698	-1,282520722	0,358520024
237174_at	-	-0,924000698	-1,282520722	0,358520024
1552976_at	-	-0,924000698	-1,282520722	0,358520024
1560879_a_at	SYT15	-0,924000698	-1,282520722	0,358520024

244062_at	DAAM1	0,764143511	0,405752839	0,358390672
217721_at	39326	0,764143511	0,405752839	0,358390672
218158_s_at	APPL1	3,748268542	3,389993505	0,358275037
225476_at	GPANK1	0,926325262	0,568155442	0,35816982
222989_s_at	UBQLN1	2,41945542	2,06133574	0,35811968
219336_s_at	ASCC1	2,920736393	2,562621495	0,358114898
223072_s_at	INO80B /// INO80I	2,560606634	2,20251271	0,358093924
200078_s_at	ATP6V0B	5,086681681	4,728594404	0,358087276
213940_s_at	FNBP1	5,610806763	5,252790612	0,358016151
203164_at	SLC33A1	2,21987062	1,861868165	0,358002455
225523_at	CCDC142 /// MRPI	5,196271767	4,838270435	0,358001332
208101_s_at	URM1	2,339581693	1,981728801	0,357852892
241347_at	RNF213	2,339581693	1,981728801	0,357852892
208641_s_at	RAC1	5,792565538	5,434745697	0,35781984
209489_at	CELF1	3,881435528	3,523783625	0,357651902
212267_at	WAPAL	2,739067176	2,381744287	0,357322889
91682_at	-	0,664020925	0,306700903	0,357320022
225297_at	HAUS1	4,308320282	3,951004713	0,357315569
202029_x_at	RPL38	7,756634669	7,399348569	0,3572861
210667_s_at	C21orf33	2,304041045	1,946903052	0,357137993
218399_s_at	CDCA4	3,278243793	2,921154239	0,357089554
200082_s_at	RPS7	8,350584911	7,993649941	0,35693497
212335_at	GNS	2,401508182	2,044580955	0,356927227
217833_at	SYNCRIP	4,00240646	3,645557465	0,356848994
223186_at	TMEM189 /// TME	2,127965541	1,771157334	0,356808207
240793_at	TTN	-0,49284695	-0,84928999	0,35644304
206530_at	RAB30	-0,49284695	-0,84928999	0,35644304
208528_x_at	SSX5	-0,49284695	-0,84928999	0,35644304
229481_at	NKD1	-0,49284695	-0,84928999	0,35644304
1553566_at	C12orf53	-0,49284695	-0,84928999	0,35644304
222929_at	FAM160B2	-0,49284695	-0,84928999	0,35644304
235312_s_at	NRAP	-0,49284695	-0,84928999	0,35644304
1564544_x_at	-	-0,49284695	-0,84928999	0,35644304
237965_at	-	-0,49284695	-0,84928999	0,35644304
215382_x_at	TPSAB1	-0,49284695	-0,84928999	0,35644304
212102_s_at	KPNA6 /// LOC100	0,001114523	-0,354781582	0,355896104
212067_s_at	C1R	0,001114523	-0,354781582	0,355896104
231796_at	EPHA8	0,001114523	-0,354781582	0,355896104
205634_x_at	ZDHHC24	1,544940605	1,189078962	0,355861643
1569532_a_at	LCN15	0,319287178	-0,036543687	0,355830865
232162_at	ZNF696	0,319287178	-0,036543687	0,355830865
220352_x_at	FLJ42627	0,319287178	-0,036543687	0,355830865
219008_at	C2orf43	3,478193934	3,122419381	0,355774553
216257_at	SERPINB13	-1,212652659	-1,568385657	0,355732997
216754_at	-	-1,212652659	-1,568385657	0,355732997
207213_s_at	USP2	-1,212652659	-1,568385657	0,355732997
1562960_at	KCNQ1-AS1	-1,212652659	-1,568385657	0,355732997
221285_at	ST8SIA2	-1,212652659	-1,568385657	0,355732997
219534_x_at	CDKN1C	-1,212652659	-1,568385657	0,355732997
242462_at	LOC100506930	-1,212652659	-1,568385657	0,355732997

1561135_at	-	-1,212652659	-1,568385657	0,355732997
215988_s_at	DLG1	-1,212652659	-1,568385657	0,355732997
1569737_a_at	CASKIN1	-1,212652659	-1,568385657	0,355732997
216193_at	-	-1,212652659	-1,568385657	0,355732997
219514_at	ANGPTL2	-1,212652659	-1,568385657	0,355732997
1563043_at	LOC285375	-1,212652659	-1,568385657	0,355732997
230935_at	LOC100506798	-1,212652659	-1,568385657	0,355732997
1560384_a_at	LOC100506387	-1,212652659	-1,568385657	0,355732997
206750_at	MAFK	-1,212652659	-1,568385657	0,355732997
228361_at	E2F2	2,804738618	2,449078728	0,35565989
210976_s_at	PFKM	3,816747242	3,461380025	0,355367217
204434_at	SPATA2	2,159837409	1,804519951	0,355317458
223310_x_at	PNPLA8	3,639446351	3,284313623	0,355132728
213214_x_at	ACTG1	8,429872956	8,074740679	0,355132276
1554015_a_at	CHD2	1,948628481	1,593605508	0,355022972
238076_at	GATAD2B	2,209870035	1,855279162	0,354590874
1568852_x_at	-	-2,074223907	-2,4284334	0,354209493
1561593_at	-	-2,074223907	-2,4284334	0,354209493
1570046_at	-	-2,074223907	-2,4284334	0,354209493
1567705_at	-	-2,074223907	-2,4284334	0,354209493
211568_at	BAI3	-2,074223907	-2,4284334	0,354209493
241807_x_at	-	-2,074223907	-2,4284334	0,354209493
206525_at	GABRR1	-2,074223907	-2,4284334	0,354209493
1569287_at	LINC00458	-2,074223907	-2,4284334	0,354209493
1559923_at	-	-2,074223907	-2,4284334	0,354209493
244367_at	-	-2,074223907	-2,4284334	0,354209493
236523_at	LOC285556	-2,074223907	-2,4284334	0,354209493
203939_at	NT5E	-2,074223907	-2,4284334	0,354209493
234090_at	-	-2,074223907	-2,4284334	0,354209493
1553965_x_at	RHOB	-2,074223907	-2,4284334	0,354209493
205951_at	MYH1	-2,074223907	-2,4284334	0,354209493
231439_at	FAM84A	-2,074223907	-2,4284334	0,354209493
236712_at	-	-2,074223907	-2,4284334	0,354209493
229092_at	NR2F2	-2,074223907	-2,4284334	0,354209493
1555869_a_at	LOC100507477	-2,074223907	-2,4284334	0,354209493
1561662_at	-	-2,074223907	-2,4284334	0,354209493
235622_at	-	-2,074223907	-2,4284334	0,354209493
228974_at	-	-2,074223907	-2,4284334	0,354209493
217519_at	-	-2,074223907	-2,4284334	0,354209493
229224_x_at	LOC643085	-2,074223907	-2,4284334	0,354209493
220407_s_at	TGFB2	-2,074223907	-2,4284334	0,354209493
1568687_s_at	ATP8B5P	-2,074223907	-2,4284334	0,354209493
1553608_a_at	LINC00189	-2,074223907	-2,4284334	0,354209493
241576_at	-	-2,074223907	-2,4284334	0,354209493
224528_s_at	KCNIP2	-2,074223907	-2,4284334	0,354209493
1555074_a_at	KCNH5	-2,074223907	-2,4284334	0,354209493
241590_at	-	-2,074223907	-2,4284334	0,354209493
237969_at	-	-2,074223907	-2,4284334	0,354209493
216516_at	-	-2,074223907	-2,4284334	0,354209493
1556221_a_at	-	-2,074223907	-2,4284334	0,354209493

231350_at	-	-2,074223907	-2,4284334	0,354209493
1566726_at	-	-2,074223907	-2,4284334	0,354209493
214321_at	NOV	-2,074223907	-2,4284334	0,354209493
1566161_at	-	-2,074223907	-2,4284334	0,354209493
241122_s_at	MUSK	-2,074223907	-2,4284334	0,354209493
227061_at	-	-2,074223907	-2,4284334	0,354209493
205389_s_at	ANK1	-2,074223907	-2,4284334	0,354209493
231275_at	FLJ42875	-2,074223907	-2,4284334	0,354209493
1553295_at	ABCA13	-2,074223907	-2,4284334	0,354209493
205016_at	TGFA	-2,074223907	-2,4284334	0,354209493
227579_at	FER	2,118167848	1,76396664	0,354201209
229862_x_at	ZBTB45	1,421391637	1,067365565	0,354026072
242263_at	TMED5	2,190140919	1,836262316	0,353878603
212951_at	GPR116	-0,791294935	-1,145057014	0,353762079
236920_at	RHOXF2 /// RHOXI	-0,791294935	-1,145057014	0,353762079
232257_s_at	-	-0,791294935	-1,145057014	0,353762079
242147_at	-	-0,791294935	-1,145057014	0,353762079
231463_at	CNTD1	-0,791294935	-1,145057014	0,353762079
215421_at	LOC100131510	-0,791294935	-1,145057014	0,353762079
1570285_at	-	-0,791294935	-1,145057014	0,353762079
1558766_at	-	-0,791294935	-1,145057014	0,353762079
232423_at	ARSD	-0,791294935	-1,145057014	0,353762079
219339_s_at	EHMT1	-0,791294935	-1,145057014	0,353762079
1560097_at	FHAD1	-0,791294935	-1,145057014	0,353762079
217273_at	PRAMEF10	-0,791294935	-1,145057014	0,353762079
242185_at	-	-0,791294935	-1,145057014	0,353762079
225867_at	VASN	-0,791294935	-1,145057014	0,353762079
201387_s_at	UCHL1	-0,791294935	-1,145057014	0,353762079
236347_at	MMAA	1,182925501	0,829309537	0,353615964
211686_s_at	MAK16	3,837502555	3,483950333	0,353552222
90610_at	LRCH4	3,509785698	3,156480639	0,353305059
215293_s_at	PGAP2	3,117098466	2,76399677	0,353101696
222157_s_at	WDR48	1,88491011	1,531877749	0,353032361
220026_at	CLCA4	-2,250580859	-2,603471267	0,352890408
1570452_at	-	-2,250580859	-2,603471267	0,352890408
222717_at	SDPR	-2,250580859	-2,603471267	0,352890408
210303_at	MAB21L2	-2,250580859	-2,603471267	0,352890408
243401_at	-	-2,250580859	-2,603471267	0,352890408
1557050_at	HOTAIRM1	-2,250580859	-2,603471267	0,352890408
1559685_at	LOC100506379	-2,250580859	-2,603471267	0,352890408
238390_at	-	-2,250580859	-2,603471267	0,352890408
1553126_a_at	SLC39A12	-2,250580859	-2,603471267	0,352890408
1562607_at	-	-2,250580859	-2,603471267	0,352890408
229084_at	CNTN4	-2,250580859	-2,603471267	0,352890408
1569858_at	-	-2,250580859	-2,603471267	0,352890408
241283_at	-	-2,250580859	-2,603471267	0,352890408
220102_at	FOXL2	-2,250580859	-2,603471267	0,352890408
233932_at	-	-2,250580859	-2,603471267	0,352890408
1557604_at	LOC401312	-2,250580859	-2,603471267	0,352890408
240122_at	DIRAS2	-2,250580859	-2,603471267	0,352890408

222291_at	FAM149A	-2,250580859	-2,603471267	0,352890408
1569761_x_at	-	-2,250580859	-2,603471267	0,352890408
207074_s_at	SLC18A1	-2,250580859	-2,603471267	0,352890408
213952_s_at	ALOX5	-2,250580859	-2,603471267	0,352890408
244288_s_at	-	-2,250580859	-2,603471267	0,352890408
238901_at	LOC100505633	-2,250580859	-2,603471267	0,352890408
205535_s_at	PCDH7	-2,250580859	-2,603471267	0,352890408
1568920_at	-	-2,250580859	-2,603471267	0,352890408
209866_s_at	LPHN3	-2,250580859	-2,603471267	0,352890408
201888_s_at	IL13RA1	-2,250580859	-2,603471267	0,352890408
242097_at	-	0,618277321	0,265475485	0,352801836
219992_at	TAC3	0,618277321	0,265475485	0,352801836
223058_at	FAM107B	4,37922506	4,026485972	0,352739088
227585_at	ATAD1	1,139057614	0,786391549	0,352666065
212648_at	DHX29	3,513985141	3,161501127	0,352484015
213934_s_at	ZNF23	2,16969527	1,817403386	0,352291883
219205_at	SRR	2,16969527	1,817403386	0,352291883
225132_at	FBXL3	4,580077988	4,227865102	0,352212886
209504_s_at	PLEKHB1	0,20740896	-0,144765583	0,352174543
1555952_at	SLC19A1	0,20740896	-0,144765583	0,352174543
228941_at	ALG10B	2,074841895	1,722747778	0,352094118
212547_at	BRD3	3,045068376	2,693044596	0,35202378
211464_x_at	CASP6	3,09217775	2,740247037	0,351930713
218127_at	NFYB	3,18076822	2,828849722	0,351918498
223147_s_at	SFT2D3 /// WDR33	0,95222556	0,600385088	0,351840472
219373_at	DPM3	2,829929672	2,478123977	0,351805695
1570208_at	LOC284260	-2,641232718	-2,993014768	0,351782051
1556488_s_at	C3orf15 /// LOC10	-2,641232718	-2,993014768	0,351782051
220661_s_at	ZNF692	2,21987062	1,868130962	0,351739659
236557_at	ZBTB38	1,094118704	0,742671819	0,351446885
212198_s_at	TM9SF4	1,802733148	1,451507543	0,351225605
208909_at	UQCRFS1	6,259605337	5,908399584	0,351205753
219245_s_at	OGFOD2	1,383446753	1,032267596	0,351179156
208984_x_at	RBM10	3,551632175	3,20060887	0,351023305
218195_at	C6orf211	4,486128467	4,135643482	0,350484984
206435_at	B4GALNT1	0,735999505	0,385582632	0,350416873
242376_at	-	-0,384193355	-0,734355396	0,350162041
214825_at	FAM155A	-0,384193355	-0,734355396	0,350162041
238544_at	-	-0,384193355	-0,734355396	0,350162041
217199_s_at	STAT2	-0,384193355	-0,734355396	0,350162041
207061_at	ERN1	-0,384193355	-0,734355396	0,350162041
1562695_at	FOXN4	-0,384193355	-0,734355396	0,350162041
219220_x_at	MRPS22	4,308320282	3,958160592	0,35015969
218630_at	MKS1	1,000459215	0,65030602	0,350153195
221005_s_at	PTDSS2	0,847529938	0,497424714	0,350105223
206188_at	ZNF623	0,900065578	0,550017041	0,350048537
204169_at	IMPDH1	2,824160383	2,47417686	0,349983523
224204_x_at	ARNTL2	2,989672611	2,639836674	0,349835938
201853_s_at	CDC25B	3,67542249	3,325881269	0,349541221
1563229_at	DLEU2	-0,135065865	-0,484558493	0,349492628

242995_at	-	-0,135065865	-0,484558493	0,349492628
219881_s_at	LOC100507619	-0,135065865	-0,484558493	0,349492628
221991_at	NXPH3	-0,135065865	-0,484558493	0,349492628
203486_s_at	ARMC8	2,267141818	1,917743773	0,349398045
209206_at	SEC22B	3,466076444	3,117017558	0,349058886
58308_at	TRIM62	0,374673916	0,025627601	0,349046315
202188_at	NUP93	3,748268542	3,399265962	0,34900258
204191_at	IFNAR1	0,791908897	0,443143223	0,348765674
201437_s_at	EIF4E	3,248151517	2,899658146	0,34849337
212317_at	TNPO3	4,140736098	3,792342525	0,348393573
221820_s_at	KAT8	3,336134047	2,987805085	0,348328962
45633_at	GINS3	1,912351793	1,564341981	0,348009812
209538_at	ZNF32	2,452674045	2,104873932	0,347800113
223056_s_at	XPO5	3,991087145	3,643334026	0,347753119
225678_at	POLR3H	1,493647653	1,146010634	0,347637019
201174_s_at	TERF2IP	4,816454496	4,469013045	0,347441451
200650_s_at	LDHA	7,955760071	7,608521836	0,347238234
228012_at	MATR3 /// SNHG4	1,307201325	0,960042218	0,347159107
221082_s_at	NDRG3	1,307201325	0,960042218	0,347159107
225860_at	-	1,307201325	0,960042218	0,347159107
212334_at	GNS	3,485638812	3,138547521	0,347091291
31861_at	IGHMBP2	2,396940971	2,049986945	0,346954027
226292_at	CAPN5	0,08619576	-0,260526297	0,346722057
229195_at	MESP1	0,08619576	-0,260526297	0,346722057
1560830_a_at	LOC147646	0,08619576	-0,260526297	0,346722057
226638_at	ARHGAP23	0,08619576	-0,260526297	0,346722057
220261_s_at	ZDHHC4	2,56794744	2,221565736	0,346381704
1554483_at	TMEM37	0,524545436	0,178191865	0,346353571
236722_at	LOC100129380	0,524545436	0,178191865	0,346353571
1561446_at	-	-0,66934516	-1,015398016	0,346052857
230771_at	NKAIN4	-0,66934516	-1,015398016	0,346052857
230419_at	FLJ37644	-0,66934516	-1,015398016	0,346052857
215755_at	-	-0,66934516	-1,015398016	0,346052857
219367_s_at	-	-0,66934516	-1,015398016	0,346052857
205431_s_at	BMP5	-0,66934516	-1,015398016	0,346052857
1570338_at	-	-0,66934516	-1,015398016	0,346052857
214443_at	PVR	-0,66934516	-1,015398016	0,346052857
220282_at	RIC3	-0,66934516	-1,015398016	0,346052857
213830_at	YME1L1	-0,66934516	-1,015398016	0,346052857
226359_at	GTPBP1	-0,66934516	-1,015398016	0,346052857
204376_at	VPRBP	-0,66934516	-1,015398016	0,346052857
216637_at	-	-0,66934516	-1,015398016	0,346052857
232139_s_at	KIAA1919	-0,66934516	-1,015398016	0,346052857
209261_s_at	NR2F6	-0,66934516	-1,015398016	0,346052857
234431_at	GSN	-0,66934516	-1,015398016	0,346052857
229240_at	ZDHHC21	-0,66934516	-1,015398016	0,346052857
244149_at	-	-0,66934516	-1,015398016	0,346052857
221393_at	TAAR3	-1,369772723	-1,715576125	0,345803402
215810_x_at	DST	-1,369772723	-1,715576125	0,345803402
1554828_at	PDGFRA	-1,369772723	-1,715576125	0,345803402

230684_at	GTPBP10	-1,369772723	-1,715576125	0,345803402
1563679_at	LOC150577	-1,369772723	-1,715576125	0,345803402
244469_at	-	-1,369772723	-1,715576125	0,345803402
234301_s_at	TFB1M	-1,369772723	-1,715576125	0,345803402
1557724_a_at	LOC285847	-1,369772723	-1,715576125	0,345803402
230193_at	WDR66	-1,369772723	-1,715576125	0,345803402
1563563_at	CCDC40	-1,369772723	-1,715576125	0,345803402
1559676_a_at	-	-1,369772723	-1,715576125	0,345803402
239924_at	GUSBP11	-1,369772723	-1,715576125	0,345803402
210724_at	EMR3	-1,369772723	-1,715576125	0,345803402
210746_s_at	EPB42	-1,369772723	-1,715576125	0,345803402
1562273_at	CNGA4	-1,369772723	-1,715576125	0,345803402
1563260_at	LINC00587	-1,369772723	-1,715576125	0,345803402
229985_at	BTNL9	-1,369772723	-1,715576125	0,345803402
220098_at	HYDIN	-1,369772723	-1,715576125	0,345803402
1562540_at	LOC339978	-1,369772723	-1,715576125	0,345803402
1561487_at	-	-1,369772723	-1,715576125	0,345803402
1553844_a_at	C10orf67	-1,369772723	-1,715576125	0,345803402
237111_at	LOC388942	-1,369772723	-1,715576125	0,345803402
233699_at	-	-1,369772723	-1,715576125	0,345803402
211099_s_at	CNGB1	-1,369772723	-1,715576125	0,345803402
1564157_at	FLJ33544	-1,369772723	-1,715576125	0,345803402
214846_s_at	ALPK3	-1,369772723	-1,715576125	0,345803402
234966_at	KLK15	-1,369772723	-1,715576125	0,345803402
234905_at	DKFZP434H168	-1,369772723	-1,715576125	0,345803402
238359_at	-	-1,369772723	-1,715576125	0,345803402
221729_at	COL5A2	-1,369772723	-1,715576125	0,345803402
1559228_at	CLU	-1,369772723	-1,715576125	0,345803402
201915_at	SEC63	1,160996507	0,815200271	0,345796236
214737_x_at	HNRNPC /// LOC1(	6,44938534	6,103616794	0,345768546
1554430_at	FAM165B	0,282358733	-0,063377083	0,345735816
208645_s_at	RPS14	8,589240181	8,244307787	0,344932393
211942_x_at	RPL13A /// RPL13/	7,934172187	7,589327183	0,344845004
55662_at	C10orf76	0,022601538	-0,321613097	0,344214635
212321_at	SGPL1	1,071336699	0,727123109	0,34421359
1554493_s_at	THADA	1,510970097	1,167919222	0,343050875
238618_at	NF2	-0,280273599	-0,623254098	0,342980499
1552354_at	C19orf26	-0,280273599	-0,623254098	0,342980499
238570_at	-	-0,043925712	-0,386896102	0,34297039
1554274_a_at	SSH1	-0,043925712	-0,386896102	0,34297039
239014_at	CCAR1	-0,043925712	-0,386896102	0,34297039
208405_s_at	CD164	5,837570158	5,494711522	0,342858636
237209_s_at	NFRKB	1,421391637	1,078619386	0,342772251
215690_x_at	GPAA1	2,932161212	2,589579418	0,342581794
226110_at	PTAR1	2,932161212	2,589579418	0,342581794
224149_x_at	LOC100287789 ///	0,355751005	0,013190398	0,342560607
223207_x_at	PHPT1	5,110793201	4,76840397	0,342389231
239084_at	SNAP29	0,97663406	0,634301128	0,342332932
204074_s_at	CEP104	2,642918178	2,300902761	0,342015417
230864_at	NIM1	0,648195588	0,306454867	0,341740721

211748_x_at	PTGDS	0,648195588	0,306454867	0,341740721
213827_at	ARHGAP33	0,707657549	0,366031214	0,341626335
215519_x_at	SGSM3	0,707657549	0,366031214	0,341626335
226464_at	C3orf58	3,045068376	2,703479652	0,341588724
201146_at	NFE2L2	4,858503557	4,517111809	0,341391748
200677_at	PTTG1IP	4,381466431	4,040084244	0,341382187
218348_s_at	ZC3H7A	3,915780568	3,5744736	0,341306968
208403_x_at	MAX	0,820160788	0,479089184	0,341071604
202331_at	BCKDHA	2,705643918	2,364676067	0,340967851
218098_at	ARFGEF2	3,726612406	3,385678356	0,34093405
223050_s_at	FBXW5	2,138605583	1,797682937	0,340922646
203528_at	SEMA4D	3,406503548	3,065641037	0,340862512
1552651_a_at	RAD51L3-RFFL ///	0,873789652	0,533258442	0,34053121
218737_at	SBNO1	2,356619952	2,016139502	0,340480449
200033_at	DDX5	6,788953388	6,448905567	0,340047821
244398_x_at	ZNF684	0,167727503	-0,172194225	0,339921728
1570068_at	-	0,167727503	-0,172194225	0,339921728
235214_at	LURAP1	0,167727503	-0,172194225	0,339921728
219069_at	ANKRD49	3,921953088	3,582050316	0,339902771
225845_at	ZBTB44	3,050461597	2,710570973	0,339890624
1568883_at	-	-1,802716385	-2,142286822	0,339570436
229542_at	C20orf85	-1,802716385	-2,142286822	0,339570436
239770_at	ESYT3	-1,802716385	-2,142286822	0,339570436
207456_at	HNF4G	-1,802716385	-2,142286822	0,339570436
1570263_at	-	-1,802716385	-2,142286822	0,339570436
240088_at	PDE5A	-1,802716385	-2,142286822	0,339570436
240195_at	-	-1,802716385	-2,142286822	0,339570436
205958_x_at	CSHL1	-1,802716385	-2,142286822	0,339570436
234363_at	OR6B1	-1,802716385	-2,142286822	0,339570436
206164_at	CLCA2	-1,802716385	-2,142286822	0,339570436
231257_at	TCERG1L	-1,802716385	-2,142286822	0,339570436
1567183_s_at	-	-1,802716385	-2,142286822	0,339570436
244336_at	-	-1,802716385	-2,142286822	0,339570436
238249_at	-	-1,802716385	-2,142286822	0,339570436
222948_s_at	LOC100509751 ///	-1,802716385	-2,142286822	0,339570436
209347_s_at	MAF	-1,802716385	-2,142286822	0,339570436
232327_at	THSD7B	-1,802716385	-2,142286822	0,339570436
1553938_a_at	STK32A	-1,802716385	-2,142286822	0,339570436
228459_at	FAM84A	-1,802716385	-2,142286822	0,339570436
1557346_a_at	C17orf51 /// FAM2	-1,802716385	-2,142286822	0,339570436
1557223_at	RBPMS	-1,802716385	-2,142286822	0,339570436
205426_s_at	HIP1	-1,802716385	-2,142286822	0,339570436
206280_at	CDH18	-1,802716385	-2,142286822	0,339570436
210842_at	NRP2	-1,802716385	-2,142286822	0,339570436
206602_s_at	HOXD3	-1,802716385	-2,142286822	0,339570436
1555158_at	-	-1,802716385	-2,142286822	0,339570436
216991_at	ZNF224	-1,802716385	-2,142286822	0,339570436
1553927_at	C7orf33	-1,802716385	-2,142286822	0,339570436
221366_at	NKX6-1	-1,802716385	-2,142286822	0,339570436
223648_s_at	FGFRL1	-1,802716385	-2,142286822	0,339570436

206427_s_at	MLANA	-1,802716385	-2,142286822	0,339570436
207663_x_at	GAGE3	-1,802716385	-2,142286822	0,339570436
1553618_at	TRIM43 /// TRIM4:	-1,802716385	-2,142286822	0,339570436
228880_at	NAT8L	0,425036312	0,085534992	0,33950132
203869_at	USP46	0,425036312	0,085534992	0,33950132
229564_at	RRP7A	0,764143511	0,424693306	0,339450205
221538_s_at	PLXNA1	2,890618076	2,551398837	0,339219239
213530_at	RAB3GAP1	1,830468611	1,491644074	0,338824538
238147_at	TRIM46	1,701542258	1,362900801	0,338641457
202492_at	ATG9A	1,440265662	1,101780212	0,338485451
232450_at	LOC149351	-2,163157732	-2,501359113	0,338201382
1564371_a_at	CASC2	-2,163157732	-2,501359113	0,338201382
1556238_at	-	-2,163157732	-2,501359113	0,338201382
241811_x_at	SLC6A4	-2,163157732	-2,501359113	0,338201382
204619_s_at	VCAN	-2,163157732	-2,501359113	0,338201382
242483_at	-	-2,163157732	-2,501359113	0,338201382
235336_at	-	-2,163157732	-2,501359113	0,338201382
1556500_a_at	-	-2,163157732	-2,501359113	0,338201382
1562076_at	-	-2,163157732	-2,501359113	0,338201382
216098_s_at	HTR7 /// HTR7P1	-2,163157732	-2,501359113	0,338201382
1564405_at	-	-2,163157732	-2,501359113	0,338201382
237651_x_at	LINC00518	-2,163157732	-2,501359113	0,338201382
210503_at	MAGEA11	-2,163157732	-2,501359113	0,338201382
1554921_a_at	SCEL	-2,163157732	-2,501359113	0,338201382
1560075_at	-	-2,163157732	-2,501359113	0,338201382
237168_at	-	-2,163157732	-2,501359113	0,338201382
232638_at	COL20A1	-2,163157732	-2,501359113	0,338201382
237602_at	-	-2,163157732	-2,501359113	0,338201382
202280_at	-	-2,163157732	-2,501359113	0,338201382
1560380_at	LOC497256	-2,163157732	-2,501359113	0,338201382
241042_at	-	-2,163157732	-2,501359113	0,338201382
241883_x_at	-	-2,163157732	-2,501359113	0,338201382
1564331_at	ZNF846	-2,163157732	-2,501359113	0,338201382
1568836_at	CLK4	-2,163157732	-2,501359113	0,338201382
1561650_s_at	LOC285692	-2,163157732	-2,501359113	0,338201382
240143_at	-	-2,163157732	-2,501359113	0,338201382
204470_at	CXCL1	-2,163157732	-2,501359113	0,338201382
1568791_s_at	LOC100505518	-2,163157732	-2,501359113	0,338201382
241710_at	LOC728819	-2,163157732	-2,501359113	0,338201382
244070_at	SYNE1	-2,163157732	-2,501359113	0,338201382
207655_s_at	BLNK	-2,163157732	-2,501359113	0,338201382
233259_at	CCDC48	-2,163157732	-2,501359113	0,338201382
240836_at	ZNF19	-2,163157732	-2,501359113	0,338201382
226766_at	ROBO2	-2,163157732	-2,501359113	0,338201382
228053_s_at	TOMM5	5,329807155	4,991615853	0,338191302
212533_at	WEE1	4,871514979	4,533347044	0,338167935
213315_x_at	CXorf40A	3,577387089	3,239448352	0,337938738
1564229_at	LOC729173	-0,551284523	-0,889222211	0,337937688
1554826_at	CDK15	-0,551284523	-0,889222211	0,337937688
1564803_at	KRTAP11-1	-0,551284523	-0,889222211	0,337937688

237256_at	FBXL12	-0,551284523	-0,889222211	0,337937688
1555822_at	FAM138A /// FAM	-0,551284523	-0,889222211	0,337937688
217251_x_at	-	-0,551284523	-0,889222211	0,337937688
1560630_at	-	-0,551284523	-0,889222211	0,337937688
229977_at	RTDR1	-0,551284523	-0,889222211	0,337937688
217162_at	-	-0,551284523	-0,889222211	0,337937688
207306_at	TCF15	-0,551284523	-0,889222211	0,337937688
232294_at	LOC219347	-0,551284523	-0,889222211	0,337937688
221150_at	MEPE	-0,551284523	-0,889222211	0,337937688
220963_s_at	RSG1	-0,551284523	-0,889222211	0,337937688
1552378_s_at	RDH10	-0,551284523	-0,889222211	0,337937688
209046_s_at	GABARAPL2	5,782922641	5,445155798	0,337766843
203316_s_at	SNRPE	6,805568272	6,467827735	0,337740537
204088_at	P2RX4	1,671397663	1,333668715	0,337728948
1553262_a_at	UTS2R	1,671397663	1,333668715	0,337728948
211787_s_at	EIF4A1 /// SENP3-I	6,878962431	6,541534692	0,337427739
200909_s_at	RPLP2 /// SNORA5	8,118920962	7,781550089	0,337370873
221679_s_at	ABHD6	-0,180990326	-0,518232988	0,337242663
227707_at	MYLIP	-0,180990326	-0,518232988	0,337242663
241423_at	ZBED3-AS1	-0,180990326	-0,518232988	0,337242663
216347_s_at	PPP1R13B	-0,180990326	-0,518232988	0,337242663
202131_s_at	RIOK3	2,752615529	2,415420763	0,337194766
205356_at	USP13	3,012642182	2,675582704	0,337059478
1561448_at	-	-1,712732543	-2,049747196	0,337014653
241543_at	-	-1,712732543	-2,049747196	0,337014653
1566771_at	-	-1,712732543	-2,049747196	0,337014653
228195_at	C2orf88	-1,712732543	-2,049747196	0,337014653
1558383_at	KPNA4	-1,712732543	-2,049747196	0,337014653
1560557_at	-	-1,712732543	-2,049747196	0,337014653
235642_at	-	-1,712732543	-2,049747196	0,337014653
220620_at	CRCT1	-1,712732543	-2,049747196	0,337014653
1562443_at	CLVS2	-1,712732543	-2,049747196	0,337014653
1555191_a_at	FHL5	-1,712732543	-2,049747196	0,337014653
1570302_at	-	-1,712732543	-2,049747196	0,337014653
207958_at	UGT2A1 /// UGT2/	-1,712732543	-2,049747196	0,337014653
1569659_at	LOC100506895	-1,712732543	-2,049747196	0,337014653
240042_at	FIBCD1	-1,712732543	-2,049747196	0,337014653
233705_at	PACSIN2	-1,712732543	-2,049747196	0,337014653
232956_at	-	-1,712732543	-2,049747196	0,337014653
206603_at	SLC2A4	-1,712732543	-2,049747196	0,337014653
1556602_at	-	-1,712732543	-2,049747196	0,337014653
207584_at	LPA	-1,712732543	-2,049747196	0,337014653
241752_at	SLC8A1	-1,712732543	-2,049747196	0,337014653
225016_at	APCDD1	-1,712732543	-2,049747196	0,337014653
1566935_at	TYRO3P	-1,712732543	-2,049747196	0,337014653
1561665_at	-	-1,712732543	-2,049747196	0,337014653
211369_at	-	-1,712732543	-2,049747196	0,337014653
242104_at	-	-1,712732543	-2,049747196	0,337014653
1554886_a_at	MLXIP	-1,712732543	-2,049747196	0,337014653
222354_at	F11R	-1,712732543	-2,049747196	0,337014653

227957_at	GSN	-1,712732543	-2,049747196	0,337014653
244069_at	-	-1,712732543	-2,049747196	0,337014653
215904_at	MLLT4	-1,712732543	-2,049747196	0,337014653
243990_at	-	-1,712732543	-2,049747196	0,337014653
201495_x_at	MYH11	-1,712732543	-2,049747196	0,337014653
237472_at	SOX1	-1,712732543	-2,049747196	0,337014653
207896_s_at	DLEC1	-1,712732543	-2,049747196	0,337014653
1561266_at	LOC100507033	-1,712732543	-2,049747196	0,337014653
202554_s_at	GSTM3	-1,712732543	-2,049747196	0,337014653
1562610_at	-	-1,712732543	-2,049747196	0,337014653
233499_at	LRRC7	-2,495152701	-2,832036647	0,336883946
241829_at	FAM124A	-2,495152701	-2,832036647	0,336883946
240619_at	-	-2,495152701	-2,832036647	0,336883946
1554142_at	SUGT1P3	-2,495152701	-2,832036647	0,336883946
207888_at	-	-2,495152701	-2,832036647	0,336883946
231192_at	LPAR3	-2,495152701	-2,832036647	0,336883946
240959_at	-	-2,495152701	-2,832036647	0,336883946
232424_at	PRDM16	-2,495152701	-2,832036647	0,336883946
233261_at	EBF1	-2,495152701	-2,832036647	0,336883946
234930_at	-	-2,495152701	-2,832036647	0,336883946
224601_at	LOC100507246	4,651898655	4,315068752	0,336829903
223711_s_at	THYN1	4,140736098	3,804015818	0,336720279
225870_s_at	TRAPPC5	2,983826368	2,64735888	0,336467488
230799_at	LOC100134259	0,045517965	-0,290694975	0,33621294
235255_at	ATP6V0A2	0,045517965	-0,290694975	0,33621294
215121_x_at	CYAT1 /// IGLC1 //	0,045517965	-0,290694975	0,33621294
209950_s_at	VILL	0,045517965	-0,290694975	0,33621294
211250_s_at	SH3BP2	0,045517965	-0,290694975	0,33621294
227258_at	CACUL1	0,045517965	-0,290694975	0,33621294
1561989_at	-	0,045517965	-0,290694975	0,33621294
234572_at	-	0,491906512	0,155747281	0,336159231
217942_at	MRPS35	4,2463808	3,910368531	0,336012269
209662_at	CETN3	4,773165602	4,437259899	0,335905704
213311_s_at	TCF25	3,442765386	3,106874588	0,335890797
239309_at	DLX6	-1,893506789	-2,229233437	0,335726648
210335_at	RASSF9	-1,893506789	-2,229233437	0,335726648
207371_at	-	-1,893506789	-2,229233437	0,335726648
241645_at	-	-1,893506789	-2,229233437	0,335726648
1562424_at	LOC285889	-1,893506789	-2,229233437	0,335726648
1560821_at	ARHGAP22	-1,893506789	-2,229233437	0,335726648
237261_at	ANGPT2	-1,893506789	-2,229233437	0,335726648
205083_at	AOX1	-1,893506789	-2,229233437	0,335726648
1569554_at	ESR2	-1,893506789	-2,229233437	0,335726648
243032_at	-	-1,893506789	-2,229233437	0,335726648
232187_at	PALMD	-1,893506789	-2,229233437	0,335726648
1566899_at	-	-1,893506789	-2,229233437	0,335726648
244806_at	-	-1,893506789	-2,229233437	0,335726648
1567986_at	-	-1,893506789	-2,229233437	0,335726648
240782_at	-	-1,893506789	-2,229233437	0,335726648
1565325_at	MATR3 /// SNHG4	-1,893506789	-2,229233437	0,335726648

1563568_at	LINC00559	-1,893506789	-2,229233437	0,335726648
1570357_at	STX8	-1,893506789	-2,229233437	0,335726648
244549_at	-	-1,893506789	-2,229233437	0,335726648
240197_at	SYN2	-1,893506789	-2,229233437	0,335726648
205912_at	PNLIP	-1,893506789	-2,229233437	0,335726648
1555391_a_at	ALS2CR8	-1,893506789	-2,229233437	0,335726648
230418_s_at	GALNTL1	-1,893506789	-2,229233437	0,335726648
1554844_at	EYA3	-1,893506789	-2,229233437	0,335726648
1567023_at	OR5AK4P	-1,893506789	-2,229233437	0,335726648
1570537_a_at	-	-1,893506789	-2,229233437	0,335726648
226931_at	TMTC1	-1,893506789	-2,229233437	0,335726648
220822_at	-	-1,893506789	-2,229233437	0,335726648
1563685_at	LOC285422	-1,893506789	-2,229233437	0,335726648
242534_at	EVI5L	-1,893506789	-2,229233437	0,335726648
229471_s_at	SRSF8	-1,893506789	-2,229233437	0,335726648
1564837_at	LOC151760	-1,893506789	-2,229233437	0,335726648
239688_at	SMC1A	-1,893506789	-2,229233437	0,335726648
214604_at	HOXD11	-1,893506789	-2,229233437	0,335726648
232502_at	LOC100652735 ///	-1,893506789	-2,229233437	0,335726648
240219_at	LINC00327	-1,893506789	-2,229233437	0,335726648
215625_at	LOC644450	-1,893506789	-2,229233437	0,335726648
117_at	HSPA6	-1,441132444	-1,776824313	0,335691868
241098_at	CLEC7A	-2,416625063	-2,752097204	0,33547214
1561234_at	-	-2,416625063	-2,752097204	0,33547214
240914_at	-	-2,416625063	-2,752097204	0,33547214
233711_at	-	-2,416625063	-2,752097204	0,33547214
244283_x_at	-	-2,416625063	-2,752097204	0,33547214
1561514_at	LOC400655	-2,416625063	-2,752097204	0,33547214
210194_at	PLA2R1	-2,416625063	-2,752097204	0,33547214
215679_at	-	-2,416625063	-2,752097204	0,33547214
1552742_at	KCNH8	-2,416625063	-2,752097204	0,33547214
205466_s_at	HS3ST1	-2,416625063	-2,752097204	0,33547214
237667_at	-	-2,569309811	-2,904470894	0,335161083
220935_s_at	CDK5RAP2	1,97249974	1,637482551	0,335017189
203012_x_at	RPL23A	8,65677941	8,321789386	0,334990024
221667_s_at	HSPB8	-1,137208348	-1,471833228	0,334624879
242678_at	-	-1,137208348	-1,471833228	0,334624879
219941_at	TMEM19	-1,137208348	-1,471833228	0,334624879
1555264_a_at	LINC00598	-1,137208348	-1,471833228	0,334624879
237644_at	-	-1,137208348	-1,471833228	0,334624879
211441_x_at	CYP3A43	-1,137208348	-1,471833228	0,334624879
206325_at	SERPINA6	-1,137208348	-1,471833228	0,334624879
235973_at	-	-1,137208348	-1,471833228	0,334624879
1558088_a_at	UBE2I	-1,137208348	-1,471833228	0,334624879
203930_s_at	MAPT	-1,137208348	-1,471833228	0,334624879
242525_at	SLC2A5	-1,137208348	-1,471833228	0,334624879
221239_s_at	FCRL2	-1,137208348	-1,471833228	0,334624879
205506_at	VIL1	-1,137208348	-1,471833228	0,334624879
1556994_at	-	-1,137208348	-1,471833228	0,334624879
207962_at	CAPN11	-1,137208348	-1,471833228	0,334624879

208342_x_at	CSH2	-1,137208348	-1,471833228	0,334624879
237739_at	-	-1,137208348	-1,471833228	0,334624879
230417_at	GALNTL1	-1,137208348	-1,471833228	0,334624879
215419_at	ZFR2	-1,137208348	-1,471833228	0,334624879
209867_s_at	LPHN3	-1,137208348	-1,471833228	0,334624879
216512_s_at	DCT	-1,137208348	-1,471833228	0,334624879
223454_at	CXCL16	-1,137208348	-1,471833228	0,334624879
230132_at	LOC100505495	-1,137208348	-1,471833228	0,334624879
233724_at	ARNT	-0,856259172	-1,19085694	0,334597768
206398_s_at	CD19	-0,856259172	-1,19085694	0,334597768
221724_s_at	CLEC4A	-0,856259172	-1,19085694	0,334597768
1565836_at	-	-0,856259172	-1,19085694	0,334597768
219441_s_at	LRRK1	-0,856259172	-1,19085694	0,334597768
1559945_at	RUVBL2	-0,856259172	-1,19085694	0,334597768
240669_at	-	-0,856259172	-1,19085694	0,334597768
1555343_at	MEGF10	-0,856259172	-1,19085694	0,334597768
1554705_at	SCARA5	-0,856259172	-1,19085694	0,334597768
1555480_a_at	FBLIM1	-0,856259172	-1,19085694	0,334597768
219550_at	ROBO3	-0,856259172	-1,19085694	0,334597768
1565639_a_at	-	-0,856259172	-1,19085694	0,334597768
222767_s_at	C12orf49	1,857989965	1,523429058	0,334560907
238180_at	-	0,95222556	0,61779983	0,33442573
217040_x_at	SOX15	0,244681185	-0,089635713	0,334316898
236411_at	-	0,244681185	-0,089635713	0,334316898
1564333_a_at	PSAPL1	0,244681185	-0,089635713	0,334316898
217814_at	CCDC47	3,697863752	3,363771078	0,334092673
205191_at	RP2	2,321828846	1,987810614	0,334018232
215537_x_at	DDAH2	0,556428218	0,222432814	0,333995404
232449_at	BCO2	-0,437449947	-0,771340337	0,333890389
1559449_a_at	-	-0,437449947	-0,771340337	0,333890389
219877_at	ZMAT4	-0,437449947	-0,771340337	0,333890389
1569428_at	WIBG	-0,437449947	-0,771340337	0,333890389
230242_at	NFASC	-0,437449947	-0,771340337	0,333890389
206735_at	CHRNA4	-0,437449947	-0,771340337	0,333890389
227784_s_at	COG1	2,16969527	1,836262316	0,333432953
233661_at	LINC00557	-0,994186487	-1,32740143	0,333214943
235794_at	MOBP	-0,994186487	-1,32740143	0,333214943
1565825_at	-	-0,994186487	-1,32740143	0,333214943
223756_at	KANSL3	-0,994186487	-1,32740143	0,333214943
211438_at	TRHR	-0,994186487	-1,32740143	0,333214943
207421_at	CA5A	-0,994186487	-1,32740143	0,333214943
229039_at	SYN2	-0,994186487	-1,32740143	0,333214943
1555395_at	AKAP12	-0,994186487	-1,32740143	0,333214943
240471_at	-	-0,994186487	-1,32740143	0,333214943
1564460_at	LOC286442	-0,994186487	-1,32740143	0,333214943
1570382_at	-	-0,994186487	-1,32740143	0,333214943
235534_at	-	-0,994186487	-1,32740143	0,333214943
242868_at	-	-0,994186487	-1,32740143	0,333214943
1555923_a_at	C10orf114	-0,994186487	-1,32740143	0,333214943
206483_at	LRRC6	-0,994186487	-1,32740143	0,333214943

244863_at	SH3GLB2	-0,994186487	-1,32740143	0,333214943
228978_at	LOC645722	-0,994186487	-1,32740143	0,333214943
1553588_at	ND3 /// SH3KBP1	8,900622383	8,567490756	0,333131627
212071_s_at	SPTBN1	4,588489926	4,255588638	0,332901288
224680_at	TMED4	3,726612406	3,393801804	0,332810602
57588_at	SLC24A3	-1,937365895	-2,2700497	0,332683805
218478_s_at	ZCCHC8	3,758020365	3,425365487	0,332654878
200903_s_at	AHCY	4,850340179	4,517858152	0,332482027
203537_at	PRPSAP2	4,343795594	4,011554792	0,332240802
218548_x_at	TEX264	2,348178434	2,016139502	0,332038932
227369_at	SERBP1	3,122148189	2,790111983	0,332036206
229089_at	ZBTB49	0,618277321	0,286279868	0,331997454
220962_s_at	PADI1	0,618277321	0,286279868	0,331997454
201329_s_at	ETS2	1,561816652	1,2298509	0,331965752
212518_at	PIP5K1C	0,900065578	0,568155442	0,331910136
236325_at	KIAA1377	-1,624955693	-1,956681069	0,331725376
229857_s_at	KANSL1-AS1	-1,624955693	-1,956681069	0,331725376
216780_at	-	-1,624955693	-1,956681069	0,331725376
220833_at	-	-1,624955693	-1,956681069	0,331725376
237369_at	-	-1,624955693	-1,956681069	0,331725376
231173_at	PYROXD1	-1,624955693	-1,956681069	0,331725376
220738_s_at	RPS6KA6	-1,624955693	-1,956681069	0,331725376
217330_at	DISC1	-1,624955693	-1,956681069	0,331725376
214557_at	PTTG2	-1,624955693	-1,956681069	0,331725376
235555_at	-	-1,624955693	-1,956681069	0,331725376
242504_at	-	-1,624955693	-1,956681069	0,331725376
234183_at	-	-1,624955693	-1,956681069	0,331725376
1562454_at	-	-1,624955693	-1,956681069	0,331725376
1561232_at	LOC100270680	-1,624955693	-1,956681069	0,331725376
228973_at	DLG2	-1,624955693	-1,956681069	0,331725376
227224_at	RALGPS2	-1,624955693	-1,956681069	0,331725376
211431_s_at	TYRO3	-1,624955693	-1,956681069	0,331725376
242455_at	POU3F2	-1,624955693	-1,956681069	0,331725376
233545_at	-	-1,624955693	-1,956681069	0,331725376
205464_at	SCNN1B	-1,624955693	-1,956681069	0,331725376
232584_at	-	-1,624955693	-1,956681069	0,331725376
219034_at	PARP16	1,610353504	1,278654284	0,33169922
218633_x_at	ABHD10	3,261582786	2,930238085	0,331344702
225386_s_at	HNRPLL	5,024479308	4,693359539	0,331119769
213431_x_at	SFI1	1,421391637	1,090640407	0,33075123
229153_at	SLC7A6OS	1,65554524	1,324949741	0,330595499
217749_at	COPG1	2,636121902	2,305621062	0,33050084
121_at	PAX8	2,101577987	1,77116233	0,330415657
1555419_a_at	ASAH1	3,758020365	3,427694878	0,330325488
233072_at	NTNG2	0,319287178	-0,011001236	0,330288414
211019_s_at	LSS	0,319287178	-0,011001236	0,330288414
219358_s_at	ADAP2	0,319287178	-0,011001236	0,330288414
200926_at	RPS23	8,590989104	8,261028586	0,329960518
203823_at	RGS3	1,88491011	1,555165465	0,329744645
1556334_s_at	-	-1,982324124	-2,312054429	0,329730305

243761_at	CLDN12	-1,982324124	-2,312054429	0,329730305
239985_at	-	-1,982324124	-2,312054429	0,329730305
236466_at	-	-1,982324124	-2,312054429	0,329730305
1561678_at	-	-1,982324124	-2,312054429	0,329730305
1554246_at	C1orf210	-1,982324124	-2,312054429	0,329730305
241356_at	-	-1,982324124	-2,312054429	0,329730305
235121_at	ZNF542	-1,982324124	-2,312054429	0,329730305
240627_x_at	MSANTD4	-1,982324124	-2,312054429	0,329730305
233225_at	-	-1,982324124	-2,312054429	0,329730305
241778_at	NT5C2	-1,982324124	-2,312054429	0,329730305
242572_at	-	-1,982324124	-2,312054429	0,329730305
216893_s_at	COL4A3	-1,982324124	-2,312054429	0,329730305
1566585_at	-	-1,982324124	-2,312054429	0,329730305
229472_at	SFT2D3 /// WDR33	-1,982324124	-2,312054429	0,329730305
211782_at	IDS	-1,982324124	-2,312054429	0,329730305
1553878_at	GOT1L1	-1,982324124	-2,312054429	0,329730305
1555651_at	OR10A5	-1,982324124	-2,312054429	0,329730305
1558436_a_at	-	-1,982324124	-2,312054429	0,329730305
241309_at	-	-1,982324124	-2,312054429	0,329730305
236184_at	-	-1,982324124	-2,312054429	0,329730305
237139_at	PDE9A	-1,982324124	-2,312054429	0,329730305
219429_at	FA2H	-1,982324124	-2,312054429	0,329730305
1567224_at	HMGA2	-1,982324124	-2,312054429	0,329730305
228137_s_at	PPP2R2C	-1,982324124	-2,312054429	0,329730305
208061_at	-	-1,982324124	-2,312054429	0,329730305
218326_s_at	LGR4	-1,982324124	-2,312054429	0,329730305
1556521_a_at	-	-1,982324124	-2,312054429	0,329730305
243898_at	-	-1,982324124	-2,312054429	0,329730305
234479_at	PCDHB18	-1,982324124	-2,312054429	0,329730305
207501_s_at	FGF12	-1,982324124	-2,312054429	0,329730305
244206_at	ZFAND4	-1,982324124	-2,312054429	0,329730305
1559316_at	-	-1,982324124	-2,312054429	0,329730305
219073_s_at	OSBPL10	-1,982324124	-2,312054429	0,329730305
216551_x_at	PLCG1	1,578719607	1,249194639	0,329524968
244890_at	-	0,127732831	-0,201789721	0,329522552
230157_at	CDH24	0,127732831	-0,201789721	0,329522552
239388_at	-	0,39105295	0,061773582	0,329279368
214253_s_at	DTNB	0,39105295	0,061773582	0,329279368
242997_at	-	-0,088974936	-0,418248858	0,329273922
229539_at	ABCB8	-0,088974936	-0,418248858	0,329273922
230182_at	PDCD7	-0,088974936	-0,418248858	0,329273922
210769_at	CNGB1	-0,088974936	-0,418248858	0,329273922
1557124_at	TMEM198B	-0,088974936	-0,418248858	0,329273922
224484_s_at	BRMS1L	-0,088974936	-0,418248858	0,329273922
203905_at	PARN	4,381466431	4,052245355	0,329221077
1554466_a_at	C16orf13	2,972517974	2,64347734	0,329040634
218539_at	FBXO34	2,972517974	2,64347734	0,329040634
1566146_x_at	-	-0,332405896	-0,661178575	0,328772679
241972_at	LOC401588	-0,332405896	-0,661178575	0,328772679
1559311_at	FLJ40292	-0,332405896	-0,661178575	0,328772679

216794_at	-	-0,332405896	-0,661178575	0,328772679
1558484_s_at	LRRC27	-0,332405896	-0,661178575	0,328772679
215668_s_at	PLXNB1	-0,332405896	-0,661178575	0,328772679
233971_at	FAM166A	-0,332405896	-0,661178575	0,328772679
223303_at	FERMT3	3,018305638	2,689594859	0,328710779
220607_x_at	TH1L	4,537242248	4,208549876	0,328692372
207119_at	PRKG1	-2,336678463	-2,665156778	0,328478314
244097_at	CR2	-2,336678463	-2,665156778	0,328478314
234080_at	-	-2,336678463	-2,665156778	0,328478314
1556473_at	FLJ38379	-2,336678463	-2,665156778	0,328478314
209909_s_at	TGFB2	-2,336678463	-2,665156778	0,328478314
1564685_a_at	-	-2,336678463	-2,665156778	0,328478314
210383_at	SCN1A	-2,336678463	-2,665156778	0,328478314
209465_x_at	LOC100287705 ///	-2,336678463	-2,665156778	0,328478314
1561196_at	-	-2,336678463	-2,665156778	0,328478314
224022_x_at	WNT16	-2,336678463	-2,665156778	0,328478314
222455_s_at	PARVA	-2,336678463	-2,665156778	0,328478314
231613_at	-	-2,336678463	-2,665156778	0,328478314
1568749_at	-	-2,336678463	-2,665156778	0,328478314
226535_at	ITGB6	-2,336678463	-2,665156778	0,328478314
1563091_at	-	-2,336678463	-2,665156778	0,328478314
1570030_at	ZNF396	-2,336678463	-2,665156778	0,328478314
241170_at	-	-2,336678463	-2,665156778	0,328478314
206757_at	PDE5A	-2,336678463	-2,665156778	0,328478314
228503_at	RPS6KA6	-2,336678463	-2,665156778	0,328478314
238362_at	-	-2,336678463	-2,665156778	0,328478314
208016_s_at	AGTR1	-2,336678463	-2,665156778	0,328478314
1560813_at	-	-2,336678463	-2,665156778	0,328478314
1567912_s_at	CT45A1 /// CT45A:	-2,336678463	-2,665156778	0,328478314
204547_at	RAB40B	1,745502609	1,417157926	0,328344682
222131_x_at	RHOT2	1,745502609	1,417157926	0,328344682
224709_s_at	CDC42SE2	6,141570897	5,813286344	0,328284553
218753_at	XKR8	2,937809196	2,609615156	0,32819404
238893_at	LOC338758	1,440265662	1,112548615	0,327717047
202321_at	GGPS1	2,138605583	1,811004388	0,327601195
224752_at	C7orf73 /// LOC10	4,965350893	4,637996517	0,327354376
230834_at	-	-0,730013898	-1,057301851	0,327287953
232708_at	GALT	-0,730013898	-1,057301851	0,327287953
220056_at	IL22RA1	-0,730013898	-1,057301851	0,327287953
221413_at	KCNAB3	-0,730013898	-1,057301851	0,327287953
207644_at	FOXH1	-0,730013898	-1,057301851	0,327287953
241517_at	-	-0,730013898	-1,057301851	0,327287953
1567341_at	FOXD4	-0,730013898	-1,057301851	0,327287953
1566647_s_at	LOC149086	-0,730013898	-1,057301851	0,327287953
220862_s_at	USP22	-0,730013898	-1,057301851	0,327287953
236940_at	-	-0,730013898	-1,057301851	0,327287953
231602_at	LOC100506231	-0,730013898	-1,057301851	0,327287953
1569250_at	ZNF333	-0,730013898	-1,057301851	0,327287953
222513_s_at	SORBS1	-0,730013898	-1,057301851	0,327287953
1559580_at	LRRC39	-1,289805289	-1,616924751	0,327119462

244848_at	LOC100505794	-1,289805289	-1,616924751	0,327119462
240127_at	-	-1,289805289	-1,616924751	0,327119462
238298_at	LOC100507058	-1,289805289	-1,616924751	0,327119462
232944_at	-	-1,289805289	-1,616924751	0,327119462
1561269_at	RAPGEF5	-1,289805289	-1,616924751	0,327119462
237581_at	-	-1,289805289	-1,616924751	0,327119462
207383_s_at	RHBDL1	-1,289805289	-1,616924751	0,327119462
215370_at	-	-1,289805289	-1,616924751	0,327119462
240119_at	TEPP	-1,289805289	-1,616924751	0,327119462
215053_at	SRCAP	-1,289805289	-1,616924751	0,327119462
233790_at	-	-1,289805289	-1,616924751	0,327119462
224201_s_at	SUFU	-1,289805289	-1,616924751	0,327119462
1570600_at	-	-1,289805289	-1,616924751	0,327119462
228018_at	NKAIN4	-1,289805289	-1,616924751	0,327119462
217668_at	FAM211B	-1,289805289	-1,616924751	0,327119462
1562801_at	-	-1,289805289	-1,616924751	0,327119462
215721_at	IGHG1	-1,289805289	-1,616924751	0,327119462
242381_x_at	-	-1,289805289	-1,616924751	0,327119462
1559049_a_at	-	-1,289805289	-1,616924751	0,327119462
209933_s_at	CD300A	-1,289805289	-1,616924751	0,327119462
227782_at	ZBTB7C	-1,289805289	-1,616924751	0,327119462
220756_s_at	SLC52A1	-1,289805289	-1,616924751	0,327119462
225150_s_at	RTKN	-1,289805289	-1,616924751	0,327119462
204659_s_at	GFER	1,802733148	1,475630127	0,32710302
222541_at	RSF1	1,024284941	0,697221375	0,327063566
215649_s_at	MVK	1,024284941	0,697221375	0,327063566
1553344_at	PCDH15	-2,708365344	-3,035090645	0,326725301
225767_at	RN45S	2,042564225	1,715863686	0,326700539
217100_s_at	UBXN7	2,664625637	2,338020736	0,326604901
37020_at	CRP	-2,292987487	-2,619421023	0,326433536
209370_s_at	SH3BP2	1,182925501	0,85655369	0,326371811
222849_s_at	SCRN3	1,857989965	1,531877749	0,326112216
218589_at	LPAR6	4,109088344	3,783043925	0,326044419
211995_x_at	ACTG1	8,375514501	8,049489124	0,326025377
222471_s_at	KCMF1	0,926325262	0,600385088	0,325940174
227003_at	RAB28	3,915780568	3,589866014	0,325914554
221941_at	PAOX	0,458905032	0,133104519	0,325800512
207937_x_at	FGFR1	1,493647653	1,167919222	0,325728431
201633_s_at	CYB5B	3,697863752	3,372163924	0,325699828
218304_s_at	OSBPL11	3,166767593	2,841176366	0,325591227
224734_at	HMGB1	4,306090192	3,980593719	0,325496473
220633_s_at	HP1BP3	2,537731447	2,212304392	0,325427055
221192_x_at	MFSD11	1,816938997	1,491644074	0,325294924
213165_at	CEP350	3,748268542	3,422991983	0,325276559
212801_at	CIT /// MIR1178	2,096248267	1,771157334	0,325090933
200055_at	TAF10	5,28337073	4,958563897	0,324806834
222415_at	MLL3	3,577387089	3,252850239	0,324536851
221890_at	ZNF335	1,910557155	1,586121802	0,324435354
1562856_at	-	-1,537851782	-1,862269442	0,324417659
205848_at	GAS2	-1,537851782	-1,862269442	0,324417659

219580_s_at	TMC5	-1,537851782	-1,862269442	0,324417659
207575_at	GOLGA6A	-1,537851782	-1,862269442	0,324417659
219727_at	DUOX2	-1,537851782	-1,862269442	0,324417659
1553451_at	CCDC79	-1,537851782	-1,862269442	0,324417659
1553888_at	LDHAL6A	-1,537851782	-1,862269442	0,324417659
242899_at	SESN3	-1,537851782	-1,862269442	0,324417659
213873_at	ST3GAL6	-1,537851782	-1,862269442	0,324417659
208582_s_at	DUX1 /// DUX3 ///	-1,537851782	-1,862269442	0,324417659
214043_at	PTPRD	-1,537851782	-1,862269442	0,324417659
1559936_at	-	-1,537851782	-1,862269442	0,324417659
205279_s_at	GLRB	-1,537851782	-1,862269442	0,324417659
1562068_at	-	-1,537851782	-1,862269442	0,324417659
237639_at	TMEM207	-1,537851782	-1,862269442	0,324417659
231529_at	-	-1,537851782	-1,862269442	0,324417659
233991_at	-	-1,537851782	-1,862269442	0,324417659
221316_at	CATSPERG	-1,537851782	-1,862269442	0,324417659
220188_at	JPH3	-1,537851782	-1,862269442	0,324417659
238256_at	-	-1,537851782	-1,862269442	0,324417659
237527_at	-	-1,537851782	-1,862269442	0,324417659
216935_at	LINC00302	-1,537851782	-1,862269442	0,324417659
1555993_at	CACNA1D	-1,537851782	-1,862269442	0,324417659
229300_at	RAB3C	-1,537851782	-1,862269442	0,324417659
243269_s_at	FAM205A /// FAM	-1,537851782	-1,862269442	0,324417659
243104_at	-	-1,537851782	-1,862269442	0,324417659
215062_at	-	-1,537851782	-1,862269442	0,324417659
217459_at	-	-1,537851782	-1,862269442	0,324417659
224803_s_at	MRPL20	-1,537851782	-1,862269442	0,324417659
215142_at	CXorf27	-1,537851782	-1,862269442	0,324417659
207248_at	KCNA4	-1,537851782	-1,862269442	0,324417659
231685_at	-	-1,537851782	-1,862269442	0,324417659
207620_s_at	CASK	-1,537851782	-1,862269442	0,324417659
220896_at	FBXL18	-0,230432956	-0,554696666	0,32426371
236107_at	UBE2Z	-0,230432956	-0,554696666	0,32426371
213202_at	SETD1A	0,20740896	-0,116767475	0,324176435
213421_x_at	PRSS3	0,20740896	-0,116767475	0,324176435
1554023_s_at	CCDC13	0,20740896	-0,116767475	0,324176435
215672_s_at	AHCYL2	0,20740896	-0,116767475	0,324176435
223466_x_at	COL4A3BP	3,060716358	2,736663738	0,324052621
1553279_at	BTNL9	0,524545436	0,20062106	0,323924377
201557_at	VAMP2	0,524545436	0,20062106	0,323924377
243606_at	NXPE3	1,402495885	1,078619386	0,323876499
219133_at	OXSM	3,18076822	2,857256661	0,323511559
234895_at	CTLA4	0,001114523	-0,322340048	0,323454571
1554094_at	ENTPD5	0,001114523	-0,322340048	0,323454571
235281_x_at	AHNAK	0,001114523	-0,322340048	0,323454571
202045_s_at	ARHGAP35	0,001114523	-0,322340048	0,323454571
1556540_a_at	-	0,001114523	-0,322340048	0,323454571
214228_x_at	TNFRSF4	0,001114523	-0,322340048	0,323454571
203342_at	TIMM17B	2,943764123	2,621003244	0,322760879
224060_s_at	DPH5	2,943764123	2,621003244	0,322760879

1558371_a_at	-	0,820160788	0,497424714	0,322736073
225118_at	SETD8	1,561816652	1,239268196	0,322548457
214316_x_at	CALR	1,561816652	1,239268196	0,322548457
1554773_at	WFIKKN2	-0,609040214	-0,931539147	0,322498933
237352_at	-	-0,609040214	-0,931539147	0,322498933
205402_x_at	PRSS2	-0,609040214	-0,931539147	0,322498933
238986_at	FLJ43663	-0,609040214	-0,931539147	0,322498933
211405_x_at	IFNA17	-0,609040214	-0,931539147	0,322498933
216292_at	-	-0,609040214	-0,931539147	0,322498933
206049_at	SELP	-0,609040214	-0,931539147	0,322498933
1555371_at	ABCB5	-0,609040214	-0,931539147	0,322498933
225165_at	PPP1R1B	-0,609040214	-0,931539147	0,322498933
230707_at	SORL1	-0,609040214	-0,931539147	0,322498933
220288_at	MYO15A	-0,609040214	-0,931539147	0,322498933
238422_at	LBX2-AS1	-0,609040214	-0,931539147	0,322498933
225536_at	TMEM54	-0,609040214	-0,931539147	0,322498933
218259_at	MKL2	2,664625637	2,34250725	0,322118387
201637_s_at	FXR1	6,044722928	5,722712287	0,322010641
205550_s_at	BRE	3,8018113	3,479912486	0,321898814
210216_x_at	RAD1	3,870422283	3,548671762	0,321750521
203533_s_at	CUL5	1,788922638	1,46719264	0,321729998
226276_at	TMEM167A	5,379681577	5,058099094	0,321582482
236104_at	HNRPLL	1,995527416	1,674071917	0,3214555
1557105_a_at	ZSCAN30	0,648195588	0,326777209	0,321418379
206453_s_at	NDRG2	0,648195588	0,326777209	0,321418379
235901_at	-	1,094118704	0,772793615	0,321325089
213528_at	METTL18	2,732422196	2,411206536	0,321215661
228468_at	MASTL	2,138605583	1,817403386	0,321202197
234405_s_at	PHAX	2,2388565	1,917743773	0,321112727
226310_at	RICTOR	1,364555912	1,043469112	0,3210868
209665_at	CYB561D2	2,621163856	2,300902761	0,320261095
241260_at	-	-2,250580859	-2,570522741	0,319941882
217562_at	FAM5C	-2,250580859	-2,570522741	0,319941882
231560_at	-	-2,250580859	-2,570522741	0,319941882
1554592_a_at	SLC1A6	-2,250580859	-2,570522741	0,319941882
1554368_at	NT5C1B	-2,250580859	-2,570522741	0,319941882
1565698_at	HECTD2	-2,250580859	-2,570522741	0,319941882
1561731_at	-	-2,250580859	-2,570522741	0,319941882
241034_at	GLS	-2,250580859	-2,570522741	0,319941882
217037_at	-	-2,250580859	-2,570522741	0,319941882
230496_at	FAM123A	-2,250580859	-2,570522741	0,319941882
234115_s_at	ZNRD1	-2,250580859	-2,570522741	0,319941882
228740_at	-	-2,250580859	-2,570522741	0,319941882
214559_at	DRD3	-2,250580859	-2,570522741	0,319941882
207487_at	-	-2,250580859	-2,570522741	0,319941882
1562216_at	-	-2,250580859	-2,570522741	0,319941882
233491_at	LOC100268168	-2,250580859	-2,570522741	0,319941882
1561707_at	LOC150185	-2,250580859	-2,570522741	0,319941882
240364_at	-	-2,250580859	-2,570522741	0,319941882
228780_at	POU3F3	-2,250580859	-2,570522741	0,319941882

217320_at	-	-2,250580859	-2,570522741	0,319941882
210259_s_at	DLX4	-2,250580859	-2,570522741	0,319941882
216992_s_at	GRM8	-2,250580859	-2,570522741	0,319941882
1553804_a_at	TEX34	-2,250580859	-2,570522741	0,319941882
212211_at	ANKRD17	2,295108393	1,975797592	0,319310802
218583_s_at	DCUN1D1	3,685074281	3,36586621	0,319208071
1552291_at	PIGX	3,470020347	3,150841611	0,319178736
221431_s_at	OR12D3	-0,49284695	-0,811769547	0,318922596
217450_at	AGFG2	-0,49284695	-0,811769547	0,318922596
206780_at	GAD2	-0,49284695	-0,811769547	0,318922596
237710_at	-	-0,49284695	-0,811769547	0,318922596
215256_x_at	ARHGAP33	-0,49284695	-0,811769547	0,318922596
234519_at	NOBOX	-0,49284695	-0,811769547	0,318922596
207006_s_at	CCDC106	-0,49284695	-0,811769547	0,318922596
206411_s_at	ABL2	-0,49284695	-0,811769547	0,318922596
1566646_at	LOC149086	-0,49284695	-0,811769547	0,318922596
238136_at	SHISA7	-0,49284695	-0,811769547	0,318922596
241568_at	LOC100506319	-0,49284695	-0,811769547	0,318922596
222485_at	FKBP3 /// LOC100(	-0,49284695	-0,811769547	0,318922596
220320_at	DOK3	0,282358733	-0,036543687	0,31890242
210895_s_at	CD86	0,282358733	-0,036543687	0,31890242
228859_at	C4orf21	2,199941239	1,881145546	0,318795692
211984_at	CALM1 /// CALM2	3,895112363	3,576411191	0,318701171
235203_at	-	2,374261973	2,055739171	0,318522803
222182_s_at	CNOT2	4,183891989	3,865466305	0,318425684
214005_at	GGCX	3,595895594	3,277597597	0,318297996
219096_at	ARMC7	1,326463531	1,008210238	0,318253293
224914_s_at	SARNP	4,819724179	4,501663989	0,318060189
221277_s_at	PUS3	2,074841895	1,757155322	0,317686574
236600_at	SPG20	-2,074223907	-2,391863529	0,317639622
1564250_at	-	-2,074223907	-2,391863529	0,317639622
231293_at	-	-2,074223907	-2,391863529	0,317639622
1566478_at	C17orf104	-2,074223907	-2,391863529	0,317639622
204052_s_at	SFRP4	-2,074223907	-2,391863529	0,317639622
1553486_a_at	C17orf78	-2,074223907	-2,391863529	0,317639622
232829_at	OR52K3P	-2,074223907	-2,391863529	0,317639622
1567280_at	-	-2,074223907	-2,391863529	0,317639622
1563963_at	-	-2,074223907	-2,391863529	0,317639622
242629_at	RAB3B	-2,074223907	-2,391863529	0,317639622
233304_at	NFIB	-2,074223907	-2,391863529	0,317639622
1563325_at	-	-2,074223907	-2,391863529	0,317639622
1569892_at	-	-2,074223907	-2,391863529	0,317639622
215703_at	CFTR	-2,074223907	-2,391863529	0,317639622
220556_at	ATP1B4	-2,074223907	-2,391863529	0,317639622
1556996_at	-	-2,074223907	-2,391863529	0,317639622
242789_at	PDE1A	-2,074223907	-2,391863529	0,317639622
235185_s_at	LOC388692	-2,074223907	-2,391863529	0,317639622
1567245_at	OR5J2	-2,074223907	-2,391863529	0,317639622
240642_at	ZMYM2	-2,074223907	-2,391863529	0,317639622
241700_at	ZFHX4	-2,074223907	-2,391863529	0,317639622

1552971_at	SGCZ	-2,074223907	-2,391863529	0,317639622
208712_at	CCND1	-2,074223907	-2,391863529	0,317639622
1563157_at	-	-2,074223907	-2,391863529	0,317639622
1560187_at	-	-2,074223907	-2,391863529	0,317639622
228406_at	SMYD1	-2,074223907	-2,391863529	0,317639622
1564072_at	MYH16	-2,074223907	-2,391863529	0,317639622
205498_at	GHR	-2,074223907	-2,391863529	0,317639622
234673_at	HHLA2	-2,074223907	-2,391863529	0,317639622
216154_at	-	-2,074223907	-2,391863529	0,317639622
244022_at	-	-2,074223907	-2,391863529	0,317639622
1563414_at	-	-2,074223907	-2,391863529	0,317639622
227991_x_at	ZBTB43	2,053550345	1,736450458	0,317099887
201225_s_at	SRRM1	5,186219991	4,869549729	0,316670262
218341_at	PPCS	3,768156719	3,451657924	0,316498795
200036_s_at	RPL10A	7,612750352	7,296284344	0,316466008
1552800_at	ABHD11	0,900065578	0,583607503	0,316458075
234471_s_at	NCOA5	0,900065578	0,583607503	0,316458075
202225_at	CRK	2,590269697	2,274084398	0,316185299
205605_at	HOXD9	-0,135065865	-0,45121326	0,316147395
204217_s_at	RTN2	-0,135065865	-0,45121326	0,316147395
228112_at	DNAH1	-0,135065865	-0,45121326	0,316147395
1554360_at	FCHSD2	1,383446753	1,067365565	0,316081188
211953_s_at	IPO5	3,533714224	3,217691783	0,316022441
212627_s_at	EXOSC7	4,533526699	4,217504346	0,316022352
54970_at	ZMIZ2	3,6901267	3,374181869	0,315944831
1564816_at	C14orf178	0,425036312	0,109458907	0,315577406
222395_s_at	UBE2Z	3,422378845	3,106874588	0,315504257
225075_at	PDRG1	3,481948861	3,166549502	0,315399359
204276_at	TK2	1,116462765	0,80114751	0,315315255
202370_s_at	CBFB	5,385736849	5,070665356	0,315071493
226203_at	MYO9A	1,225123479	0,910483921	0,314639558
45526_g_at	NAA60	3,834464315	3,519882492	0,314581823
208695_s_at	LOC100652821 ///	8,556776906	8,242518516	0,31425839
218358_at	CRELD2	6,089984574	5,775811945	0,314172629
223819_x_at	COMMD5	3,382641505	3,068545562	0,314095943
217732_s_at	ITM2B	5,35957938	5,045804029	0,313775351
207291_at	PRRG4	0,491906512	0,178191865	0,313714647
233974_s_at	FAM129B	0,491906512	0,178191865	0,313714647
65521_at	UBE2D4	1,716527266	1,402947749	0,313579517
213452_at	ZNF184	3,19484646	2,881480309	0,313366151
223989_s_at	REXO2	1,788922638	1,475630127	0,313292511
222843_at	FIGNL1	3,701002444	3,387778112	0,313224332
1552470_a_at	ABHD11	-0,384193355	-0,697182596	0,312989242
206540_at	GLB1L	-0,384193355	-0,697182596	0,312989242
214089_at	RPS8	-0,384193355	-0,697182596	0,312989242
211471_s_at	RAB36	-0,384193355	-0,697182596	0,312989242
210816_s_at	CYB561	-0,384193355	-0,697182596	0,312989242
240501_at	-	-0,384193355	-0,697182596	0,312989242
205640_at	ALDH3B1	-0,384193355	-0,697182596	0,312989242
219623_at	ACTR5	1,97249974	1,659532492	0,312967248

1556123_a_at	RAB11B-AS1	0,791908897	0,479089184	0,312819713
239169_at	RDM1	2,339581693	2,026957145	0,312624548
226402_at	CYP2U1	0,167727503	-0,144765583	0,312493086
240359_at	BPIFA3	0,167727503	-0,144765583	0,312493086
227965_at	LOC100506848	1,024284941	0,711934445	0,312350496
209018_s_at	PINK1	1,024284941	0,711934445	0,312350496
213365_at	ERI2	2,811067138	2,498884538	0,3121826
212496_s_at	KDM4B	3,353217129	3,041114664	0,312102465
219204_s_at	SRR	2,096248267	1,784182261	0,312066007
213579_s_at	EP300	1,898032047	1,586121802	0,311910245
211255_x_at	DEDD	0,677745787	0,366031214	0,311714573
1556064_at	LOC284926	1,510970097	1,199296812	0,311673285
200096_s_at	ATP6V0E1	6,234767026	5,923103661	0,311663365
223273_at	C14orf142	3,825885656	3,514270861	0,311614795
204299_at	SRSF10	3,438642427	3,127281098	0,311361328
237984_x_at	-	-2,495152701	-2,8064286	0,311275899
213764_s_at	MFAP5	-2,495152701	-2,8064286	0,311275899
216623_x_at	TOX3	-2,495152701	-2,8064286	0,311275899
232603_at	DCDC5	-2,495152701	-2,8064286	0,311275899
237391_at	LOC100507160	-2,495152701	-2,8064286	0,311275899
240750_at	-	-2,495152701	-2,8064286	0,311275899
1567657_at	OR2H1	-2,495152701	-2,8064286	0,311275899
224731_at	HMGB1	6,719245522	6,408218788	0,311026734
239776_at	TMEM232	-2,569309811	-2,880284116	0,310974304
1563072_at	-	-2,569309811	-2,880284116	0,310974304
1562301_at	C8orf34	-2,569309811	-2,880284116	0,310974304
1553180_at	ADAMTS19	-2,569309811	-2,880284116	0,310974304
1559360_at	-	-1,452713826	-1,763664074	0,310950248
207151_at	ADCYAP1R1	-1,452713826	-1,763664074	0,310950248
205513_at	TCN1	-1,452713826	-1,763664074	0,310950248
1562712_at	LRRC8C	-1,452713826	-1,763664074	0,310950248
244036_at	-	-1,452713826	-1,763664074	0,310950248
236959_s_at	-	-1,452713826	-1,763664074	0,310950248
214650_x_at	MOG	-1,452713826	-1,763664074	0,310950248
1559011_at	FLJ13773	-1,452713826	-1,763664074	0,310950248
239878_at	IPMK	-1,452713826	-1,763664074	0,310950248
241591_at	-	-1,452713826	-1,763664074	0,310950248
1561052_s_at	-	-1,452713826	-1,763664074	0,310950248
234771_at	DKFZp547J222	-1,452713826	-1,763664074	0,310950248
216067_at	-	-1,452713826	-1,763664074	0,310950248
206871_at	ELANE	-1,452713826	-1,763664074	0,310950248
208183_at	TACR3	-1,452713826	-1,763664074	0,310950248
239686_at	-	-1,452713826	-1,763664074	0,310950248
205818_at	DBC1	-1,452713826	-1,763664074	0,310950248
1553506_at	CPO	-1,452713826	-1,763664074	0,310950248
213125_at	OLFML2B	-1,452713826	-1,763664074	0,310950248
215064_at	SC5DL	-1,452713826	-1,763664074	0,310950248
237410_x_at	-	-1,452713826	-1,763664074	0,310950248
1560620_at	KCNK15	-1,452713826	-1,763664074	0,310950248
217013_at	AZGP1P1	-1,452713826	-1,763664074	0,310950248

234006_s_at	TMEM234	-1,452713826	-1,763664074	0,310950248
244607_at	-	-1,452713826	-1,763664074	0,310950248
213455_at	FAM114A1	-1,452713826	-1,763664074	0,310950248
238365_s_at	C1orf228	-1,452713826	-1,763664074	0,310950248
228939_at	OAF	-1,452713826	-1,763664074	0,310950248
224371_at	ADAMTSL1	-1,452713826	-1,763664074	0,310950248
211506_s_at	IL8	-1,452713826	-1,763664074	0,310950248
207042_at	E2F2	-0,043925712	-0,354781582	0,31085587
1557207_s_at	LOC283177	-0,043925712	-0,354781582	0,31085587
213690_s_at	-	-0,043925712	-0,354781582	0,31085587
229637_at	RIC8B	-0,043925712	-0,354781582	0,31085587
232984_at	HYDIN	-0,924000698	-1,234835326	0,310834628
219645_at	CASQ1	-0,924000698	-1,234835326	0,310834628
1552523_a_at	TIGD4	-0,924000698	-1,234835326	0,310834628
232226_at	LRRC4C	-0,924000698	-1,234835326	0,310834628
234252_at	-	-0,924000698	-1,234835326	0,310834628
219438_at	NKAIN1	-0,924000698	-1,234835326	0,310834628
1558378_a_at	AHNAK2	-0,924000698	-1,234835326	0,310834628
219545_at	KCTD14 /// NDUFC	-0,924000698	-1,234835326	0,310834628
207140_at	ALPI	-0,924000698	-1,234835326	0,310834628
217355_at	-	-0,924000698	-1,234835326	0,310834628
222509_s_at	ZNF672	-0,924000698	-1,234835326	0,310834628
1569962_at	-	-0,924000698	-1,234835326	0,310834628
244237_at	FLJ90680	-0,924000698	-1,234835326	0,310834628
237457_at	EIF3B	-0,924000698	-1,234835326	0,310834628
228043_at	UTP15	-0,924000698	-1,234835326	0,310834628
214479_at	GFRA3	-0,924000698	-1,234835326	0,310834628
226907_at	PPP1R14C	-0,924000698	-1,234835326	0,310834628
238366_at	C1orf228	-0,924000698	-1,234835326	0,310834628
1569006_at	LOC284379	-0,924000698	-1,234835326	0,310834628
201419_at	BAP1	2,507817181	2,197404429	0,310412752
202139_at	AKR7A2	4,441588953	4,131291507	0,310297446
238929_at	SRSF8	0,97663406	0,666486137	0,310147923
232329_at	RANBP10	-1,06282519	-1,372734086	0,309908896
244603_at	-	-1,06282519	-1,372734086	0,309908896
238116_at	DYNLRB2	-1,06282519	-1,372734086	0,309908896
240888_at	-	-1,06282519	-1,372734086	0,309908896
210073_at	ST8SIA1	-1,06282519	-1,372734086	0,309908896
1554069_at	EPHA8	-1,06282519	-1,372734086	0,309908896
207372_s_at	ENTPD2	-1,06282519	-1,372734086	0,309908896
232041_at	INADL	-1,06282519	-1,372734086	0,309908896
1553929_at	ACER1	-1,06282519	-1,372734086	0,309908896
206768_at	RPL3L	-1,06282519	-1,372734086	0,309908896
1553071_a_at	MYOZ3	-1,06282519	-1,372734086	0,309908896
208578_at	SCN10A	-1,06282519	-1,372734086	0,309908896
1554677_s_at	CMTM4	-1,06282519	-1,372734086	0,309908896
243214_at	ZBTB46	-1,06282519	-1,372734086	0,309908896
234411_x_at	CD44	-1,06282519	-1,372734086	0,309908896
1555237_at	-	-1,06282519	-1,372734086	0,309908896
232153_at	SPEG	-1,06282519	-1,372734086	0,309908896

223495_at	CCDC8	-1,06282519	-1,372734086	0,309908896
202635_s_at	POLR2K	5,262428718	4,95261581	0,309812908
231813_s_at	COG1	-0,791294935	-1,100943374	0,309648439
217605_at	USP27X	-0,791294935	-1,100943374	0,309648439
1563586_at	LOC401324	-0,791294935	-1,100943374	0,309648439
1557511_at	-	-0,791294935	-1,100943374	0,309648439
239581_at	ARL10	-0,791294935	-1,100943374	0,309648439
1562885_at	-	-0,791294935	-1,100943374	0,309648439
232893_at	LMBRD2	-0,791294935	-1,100943374	0,309648439
237740_at	-	-0,791294935	-1,100943374	0,309648439
205859_at	LY86	-0,791294935	-1,100943374	0,309648439
208153_s_at	FAT2	-0,791294935	-1,100943374	0,309648439
210064_s_at	UPK1B	-0,791294935	-1,100943374	0,309648439
234362_s_at	CTLA4	-0,791294935	-1,100943374	0,309648439
207895_at	NAALADL1	-0,791294935	-1,100943374	0,309648439
233626_at	-	-0,791294935	-1,100943374	0,309648439
1561403_at	SOHLH1	-0,791294935	-1,100943374	0,309648439
217703_x_at	-	1,910557155	1,601076277	0,309480879
203574_at	NFIL3	1,910557155	1,601076277	0,309480879
204759_at	RCBTB2	3,200057698	2,890758424	0,309299274
202675_at	SDHB	4,597104084	4,288281574	0,30882251
237772_at	-	-0,280273599	-0,589001171	0,308727572
242923_at	ZNF678	-0,280273599	-0,589001171	0,308727572
207532_at	CRYGD	-0,280273599	-0,589001171	0,308727572
233570_at	-	-0,280273599	-0,589001171	0,308727572
226724_s_at	-	-0,280273599	-0,589001171	0,308727572
244421_at	-	-0,280273599	-0,589001171	0,308727572
213200_at	SYP	-0,280273599	-0,589001171	0,308727572
201398_s_at	TRAM1	5,171648117	4,863014084	0,308634033
218455_at	NFS1	2,138605583	1,830129603	0,30847598
200651_at	GNB2L1 /// LOC10	8,252654413	7,944557088	0,308097325
221784_at	WIZ	0,244681185	-0,063377083	0,308058268
241393_at	IPP	0,244681185	-0,063377083	0,308058268
215089_s_at	RBM10	3,402275492	3,094388414	0,307887078
203034_s_at	RPL27A /// SNORA	8,540764849	8,232910986	0,307853863
225076_s_at	ZNFX1	2,16969527	1,861868165	0,307827105
219787_s_at	ECT2	3,624986574	3,317257552	0,307729022
229198_at	USP35	1,094118704	0,786391549	0,307727155
225996_at	LONRF2	1,528073958	1,220470658	0,3076033
215956_at	-	-1,212652659	-1,520022123	0,307369464
220322_at	IL36G	-1,212652659	-1,520022123	0,307369464
224251_at	WDR96	-1,212652659	-1,520022123	0,307369464
201743_at	CD14	-1,212652659	-1,520022123	0,307369464
234757_at	LOC100129884	-1,212652659	-1,520022123	0,307369464
1552967_at	ZNF645	-1,212652659	-1,520022123	0,307369464
211674_x_at	CTAG1A /// CTAG1	-1,212652659	-1,520022123	0,307369464
230722_at	BNC2	-1,212652659	-1,520022123	0,307369464
1559073_at	KDM4B	-1,212652659	-1,520022123	0,307369464
1570085_at	LOC100506679	-1,212652659	-1,520022123	0,307369464
228575_at	IL20RB	-1,212652659	-1,520022123	0,307369464

1557669_at	LOC100132005	-1,212652659	-1,520022123	0,307369464
210326_at	AGXT	-1,212652659	-1,520022123	0,307369464
223731_at	MYCBPAP	-1,212652659	-1,520022123	0,307369464
237752_at	-	-1,212652659	-1,520022123	0,307369464
209145_s_at	CBFA2T2	-1,212652659	-1,520022123	0,307369464
244516_at	-	-1,212652659	-1,520022123	0,307369464
218678_at	NES	-1,212652659	-1,520022123	0,307369464
215116_s_at	DNM1	-1,212652659	-1,520022123	0,307369464
205823_at	RGS12	-1,212652659	-1,520022123	0,307369464
1559849_at	ZNF605	-2,641232718	-2,948507524	0,307274806
208742_s_at	SAP18	6,056354289	5,749456124	0,306898164
211698_at	EID1	3,86465425	3,557786554	0,306867696
204752_x_at	PARP2	3,931608669	3,62479531	0,306813359
227075_at	ELP3	3,748268542	3,44162151	0,306647032
212078_s_at	MLL	2,276736537	1,970106792	0,306629745
211594_s_at	MRPL9	3,978219531	3,671698545	0,306520986
220850_at	MORC1	-2,416625063	-2,723114724	0,306489661
1560717_at	-	-2,416625063	-2,723114724	0,306489661
241278_at	-	-2,416625063	-2,723114724	0,306489661
241479_at	-	-2,416625063	-2,723114724	0,306489661
1564707_x_at	GLS2	-2,416625063	-2,723114724	0,306489661
207054_at	IMPG1	-2,416625063	-2,723114724	0,306489661
243958_at	-	-2,416625063	-2,723114724	0,306489661
234679_at	KRTAP9-3	-2,416625063	-2,723114724	0,306489661
237973_at	-	-2,416625063	-2,723114724	0,306489661
1559483_at	PROX1-AS1	-2,416625063	-2,723114724	0,306489661
234684_s_at	KRTAP4-4	-2,416625063	-2,723114724	0,306489661
237395_at	CYP4Z1	-2,416625063	-2,723114724	0,306489661
225250_at	STIM2	0,319287178	0,013190398	0,30609678
216460_at	LOC100288594	0,319287178	0,013190398	0,30609678
244038_at	WDR89	1,326463531	1,020514937	0,305948594
212222_at	PSME4	2,575362572	2,269637658	0,305724914
220947_s_at	TBC1D10B	1,936380191	1,630734622	0,30564557
212844_at	RRP1B	0,873789652	0,568155442	0,30563421
201037_at	PFKP	4,875022718	4,569475269	0,305547449
1559388_a_at	LYNX1	0,39105295	0,085534992	0,305517958
226929_at	MTHFR	0,820160788	0,515457602	0,304703186
1554464_a_at	CRTAP /// LOC100(	1,730929079	1,426280426	0,304648653
201892_s_at	IMPDH2	6,590398336	6,285813876	0,304584461
219254_at	C17orf101	1,160996507	0,85655369	0,304442817
205240_at	GPSM2	3,228532861	2,924357093	0,304175768
225311_at	IVD	2,365121417	2,06133574	0,303785678
223401_at	C17orf48	2,348178434	2,044580955	0,30359748
230029_x_at	UBR3	2,348178434	2,044580955	0,30359748
1552751_a_at	CIB3	-0,180990326	-0,484558493	0,303568168
202661_at	ITPR2	-0,180990326	-0,484558493	0,303568168
232342_at	MTMR14	-0,180990326	-0,484558493	0,303568168
1557795_s_at	NTRK3	-0,180990326	-0,484558493	0,303568168
231143_at	LOC100507421	-0,180990326	-0,484558493	0,303568168
227821_at	LGI4	-0,180990326	-0,484558493	0,303568168

214549_x_at	SPRR1A	-0,180990326	-0,484558493	0,303568168
229301_at	-	-0,66934516	-0,972893339	0,303548179
242473_at	TRAF4	-0,66934516	-0,972893339	0,303548179
222097_at	-	-0,66934516	-0,972893339	0,303548179
211314_at	CACNA1G	-0,66934516	-0,972893339	0,303548179
209589_s_at	EPHB2	-0,66934516	-0,972893339	0,303548179
1569402_at	MEIS3	-0,66934516	-0,972893339	0,303548179
220714_at	PRDM14	-0,66934516	-0,972893339	0,303548179
215061_at	METTL10	-0,66934516	-0,972893339	0,303548179
228589_at	BASP1	-0,66934516	-0,972893339	0,303548179
202325_s_at	ATP5J	6,4442599	6,140894621	0,303365279
224706_at	KIAA2013	3,257413106	2,954216214	0,303196892
232136_s_at	CTTNBP2	-2,163157732	-2,466349414	0,303191682
204469_at	PTPRZ1	-2,163157732	-2,466349414	0,303191682
1560748_at	-	-2,163157732	-2,466349414	0,303191682
239693_at	SNX24	-2,163157732	-2,466349414	0,303191682
236646_at	C12orf59	-2,163157732	-2,466349414	0,303191682
241678_at	-	-2,163157732	-2,466349414	0,303191682
243844_at	-	-2,163157732	-2,466349414	0,303191682
244757_at	-	-2,163157732	-2,466349414	0,303191682
232027_at	SYNE1	-2,163157732	-2,466349414	0,303191682
1560954_at	-	-2,163157732	-2,466349414	0,303191682
238906_s_at	RHOJ	-2,163157732	-2,466349414	0,303191682
238282_at	-	-2,163157732	-2,466349414	0,303191682
1562035_at	-	-2,163157732	-2,466349414	0,303191682
221322_at	NPVF	-2,163157732	-2,466349414	0,303191682
230289_at	EPB41L1	-2,163157732	-2,466349414	0,303191682
211496_s_at	PDC	-2,163157732	-2,466349414	0,303191682
233512_at	SH3RF3-AS1	-2,163157732	-2,466349414	0,303191682
220829_s_at	B3GALT1	-2,163157732	-2,466349414	0,303191682
240601_at	-	-2,163157732	-2,466349414	0,303191682
1552809_at	RFX4	-2,163157732	-2,466349414	0,303191682
1560673_at	-	-2,163157732	-2,466349414	0,303191682
1559404_a_at	LOC100288198	-2,163157732	-2,466349414	0,303191682
217590_s_at	TRPA1	-2,163157732	-2,466349414	0,303191682
208245_at	RAB9BP1	-2,163157732	-2,466349414	0,303191682
214018_at	GRIP1	-2,163157732	-2,466349414	0,303191682
1567457_at	RAC1	-2,163157732	-2,466349414	0,303191682
1558308_at	LOC100292680	-2,163157732	-2,466349414	0,303191682
1555775_a_at	ZAR1	-2,163157732	-2,466349414	0,303191682
1556940_at	LOC283484	-2,163157732	-2,466349414	0,303191682
232318_s_at	LINC00284	-2,163157732	-2,466349414	0,303191682
1558920_at	SLC8A1-AS1	-2,163157732	-2,466349414	0,303191682
205111_s_at	PLCE1	-2,163157732	-2,466349414	0,303191682
215766_at	GSTA1	0,458905032	0,155747281	0,303157751
1553483_at	TSGA10IP	0,458905032	0,155747281	0,303157751
202072_at	HNRNPL	3,537317882	3,234381926	0,302935956
229000_at	ZNF77	1,65554524	1,352622437	0,302922802
201475_x_at	MARS	4,789213414	4,486584749	0,302628665
205246_at	PEX13	2,138605583	1,836262316	0,302343267

40093_at	BCAM	-0,517555391	-0,819887788	0,302332397
223471_at	RAB3IP	2,21987062	1,917743773	0,302126847
227482_at	ADCK1	0,524545436	0,222432814	0,302112622
226321_at	LYSMD3	3,214269085	2,912181979	0,302087106
210106_at	BLOC1S1-RDH5 ///	0,95222556	0,65030602	0,30191954
224586_x_at	SUB1	7,377008376	7,075206904	0,301801471
202418_at	YIF1A	4,193391347	3,891666284	0,301725064
201417_at	SOX4	5,809852611	5,508133647	0,301718964
226262_at	DHX33	2,849132494	2,54746241	0,301670084
213304_at	FAM179B	2,085817135	1,784182261	0,301634874
214338_at	DNAJB12	0,58788177	0,286279868	0,301601903
219504_s_at	RPAP2	0,58788177	0,286279868	0,301601903
223525_at	DLL4	0,58788177	0,286279868	0,301601903
212969_x_at	EML3	0,58788177	0,286279868	0,301601903
200716_x_at	RPL13A /// RPL13A	8,368814617	8,067512921	0,301301696
237291_at	PRORSD1P	1,116462765	0,815200271	0,301262494
218762_at	ZNF574	1,116462765	0,815200271	0,301262494
222874_s_at	CLN8	0,648195588	0,346964736	0,301230852
223309_x_at	PNPLA8	3,489810047	3,188806254	0,301003793
209484_s_at	NSL1	4,793738098	4,493034674	0,300703424
220171_x_at	KIAA1704	1,625482993	1,324949741	0,300533252
217784_at	YKT6	2,339581693	2,039063441	0,300518252
219543_at	PBLD	0,127732831	-0,172194225	0,299927055
205780_at	BIK	1,92317941	1,623485524	0,299693887
209713_s_at	SLC35D1	0,900065578	0,600385088	0,29968049
225679_at	NAA30	2,961244348	2,662083808	0,29916054
203449_s_at	TERF1	3,286844008	2,987805085	0,299038923
244731_at	-	-2,336678463	-2,635549085	0,298870621
205837_s_at	GYPA	-2,336678463	-2,635549085	0,298870621
230061_at	TM4SF18	-2,336678463	-2,635549085	0,298870621
1556786_at	PDE5A	-2,336678463	-2,635549085	0,298870621
210121_at	B3GALT2	-2,336678463	-2,635549085	0,298870621
207496_at	MS4A2	-2,336678463	-2,635549085	0,298870621
1560756_at	-	-2,336678463	-2,635549085	0,298870621
1564357_at	ABHD12B /// MIR4	-2,336678463	-2,635549085	0,298870621
212915_at	PDZRN3	-2,336678463	-2,635549085	0,298870621
234139_s_at	-	-2,336678463	-2,635549085	0,298870621
230447_at	FAXC	-2,336678463	-2,635549085	0,298870621
239558_at	-	-2,336678463	-2,635549085	0,298870621
243015_at	CYP3A5	-2,336678463	-2,635549085	0,298870621
1569783_at	LRRD1	-2,336678463	-2,635549085	0,298870621
228322_at	NAA30	-2,336678463	-2,635549085	0,298870621
221411_at	HOXD12	-2,336678463	-2,635549085	0,298870621
1561158_at	-	-2,336678463	-2,635549085	0,298870621
242664_at	-	-2,336678463	-2,635549085	0,298870621
244750_at	-	-2,336678463	-2,635549085	0,298870621
224199_at	DKK2	-2,336678463	-2,635549085	0,298870621
205501_at	PDE10A	-2,336678463	-2,635549085	0,298870621
200038_s_at	RPL17 /// RPL17-C	7,825311137	7,526890837	0,298420299
201816_s_at	GBAS	4,638516946	4,340225042	0,298291903

202925_s_at	PLAGL2	2,739067176	2,440833194	0,298233982
216020_at	IFIH1	-0,551284523	-0,84928999	0,298005467
219632_s_at	SHPK /// TRPV1	-0,551284523	-0,84928999	0,298005467
1559535_s_at	-	-0,551284523	-0,84928999	0,298005467
1556422_at	-	-0,551284523	-0,84928999	0,298005467
1562028_at	CCND3	-0,551284523	-0,84928999	0,298005467
217337_at	-	-0,551284523	-0,84928999	0,298005467
1556453_at	LOC100506274	-0,551284523	-0,84928999	0,298005467
224090_s_at	TNFRSF19	-0,551284523	-0,84928999	0,298005467
1568807_a_at	RBM26-AS1	-0,088974936	-0,386896102	0,297921167
236392_at	CUL9	-0,088974936	-0,386896102	0,297921167
234842_at	-	-0,088974936	-0,386896102	0,297921167
213614_x_at	EEF1A1 /// LOC10(	8,609655896	8,312109391	0,297546505
53968_at	INTS5	2,829419601	2,531915523	0,297504079
225715_at	RPTOR	1,421391637	1,123905343	0,297486295
239876_at	-	1,024284941	0,727123109	0,297161832
225608_at	SNX29	1,024284941	0,727123109	0,297161832
212857_x_at	SUB1	7,407451311	7,110397395	0,297053916
234738_s_at	KLHDC4 /// LOC10	0,20740896	-0,089635713	0,297044673
244763_at	MTRF1	0,20740896	-0,089635713	0,297044673
240607_at	MIAT	0,20740896	-0,089635713	0,297044673
213215_at	AP3S2 /// C15orf3	0,20740896	-0,089635713	0,297044673
209716_at	CSF1	0,20740896	-0,089635713	0,297044673
205147_x_at	NCF4	1,640193838	1,343200523	0,296993315
214280_x_at	HNRNPA1	5,757392622	5,46047789	0,296914731
215381_at	MTOR	-0,437449947	-0,734355396	0,296905448
244131_at	HNRNPA2B1	-0,437449947	-0,734355396	0,296905448
203784_s_at	DDX28	-0,437449947	-0,734355396	0,296905448
236146_at	SYNCRIP	-0,437449947	-0,734355396	0,296905448
231699_at	LOC100289251	-0,437449947	-0,734355396	0,296905448
1562910_at	SH3PXD2B	-0,437449947	-0,734355396	0,296905448
232357_at	TTLL9	-0,437449947	-0,734355396	0,296905448
241354_at	ASMTL-AS1	-0,437449947	-0,734355396	0,296905448
213685_at	LOC100506963	1,843966654	1,547376668	0,296589986
1559333_at	SRGAP3-AS2	-1,369772723	-1,665791776	0,296019053
1556618_at	-	-1,369772723	-1,665791776	0,296019053
210637_at	TACR1	-1,369772723	-1,665791776	0,296019053
231074_at	-	-1,369772723	-1,665791776	0,296019053
1560432_at	CLRN1-AS1	-1,369772723	-1,665791776	0,296019053
244309_at	-	-1,369772723	-1,665791776	0,296019053
1565909_at	-	-1,369772723	-1,665791776	0,296019053
210711_at	LINC00260	-1,369772723	-1,665791776	0,296019053
237255_at	LOC100506115	-1,369772723	-1,665791776	0,296019053
232965_at	LOC400684	-1,369772723	-1,665791776	0,296019053
244572_at	KY	-1,369772723	-1,665791776	0,296019053
1555235_s_at	IQCF3	-1,369772723	-1,665791776	0,296019053
222296_at	-	-1,369772723	-1,665791776	0,296019053
243428_at	KCNQ1OT1	-1,369772723	-1,665791776	0,296019053
1558682_at	HMGA2	-1,369772723	-1,665791776	0,296019053
233923_at	ARHGAP42	-1,369772723	-1,665791776	0,296019053

237757_at	-	-1,369772723	-1,665791776	0,296019053
221307_at	KCNIP1	-1,369772723	-1,665791776	0,296019053
244360_at	FBXL17	-1,369772723	-1,665791776	0,296019053
1553123_at	WDR62	-1,369772723	-1,665791776	0,296019053
1552277_a_at	MSANTD3	3,029186714	2,733194118	0,295992596
201586_s_at	SFPQ	5,372984776	5,077376387	0,295608389
225378_at	VPS37A	3,127551022	2,832036229	0,295514793
218563_at	NDUFA3	4,718186291	4,422765225	0,295421067
224643_at	PRRC1	3,912706782	3,617435945	0,295270837
212245_at	MCFD2	3,694479488	3,399265962	0,295213526
225684_at	SKA2	5,20416543	4,909309922	0,294855508
203754_s_at	BRF1	0,97663406	0,681874787	0,294759273
221028_s_at	GFOD2	0,97663406	0,681874787	0,294759273
1553704_x_at	ZNF791	2,031158928	1,736450458	0,29470847
207275_s_at	ACSL1	2,410823487	2,116147513	0,294675974
225351_at	FAM45A	1,802733148	1,508107415	0,294625732
226732_at	RBM33	3,257413106	2,962798728	0,294614378
217609_at	LRRC23 /// RPL13F	0,791908897	0,497424714	0,294484183
221208_s_at	MSANTD2	1,610353504	1,315897482	0,294456022
41660_at	CELSR1	-1,531085418	-1,825462445	0,294377027
226986_at	WIPI2	1,440265662	1,146010634	0,294255029
208081_s_at	ZNF442	-1,802716385	-2,09678811	0,294071725
1563145_at	LINC00301	-1,802716385	-2,09678811	0,294071725
1561368_at	-	-1,802716385	-2,09678811	0,294071725
236827_at	-	-1,802716385	-2,09678811	0,294071725
222141_at	KLHL22	-1,802716385	-2,09678811	0,294071725
237802_at	XKR4	-1,802716385	-2,09678811	0,294071725
1562473_at	-	-1,802716385	-2,09678811	0,294071725
215503_at	SPINT3	-1,802716385	-2,09678811	0,294071725
203563_at	AFAP1	-1,802716385	-2,09678811	0,294071725
230287_at	SGSM1	-1,802716385	-2,09678811	0,294071725
207885_at	S100G	-1,802716385	-2,09678811	0,294071725
201547_at	KDM5B	-1,802716385	-2,09678811	0,294071725
241455_at	C6orf132	-1,802716385	-2,09678811	0,294071725
1554779_s_at	PHLDB2	-1,802716385	-2,09678811	0,294071725
231754_at	PCDHGC4	-1,802716385	-2,09678811	0,294071725
1560276_at	LOC283403	-1,802716385	-2,09678811	0,294071725
237656_at	WWC2	-1,802716385	-2,09678811	0,294071725
1555099_at	MPP4	-1,802716385	-2,09678811	0,294071725
1561261_at	-	-1,802716385	-2,09678811	0,294071725
230539_at	FAM182A	-1,802716385	-2,09678811	0,294071725
1563612_at	-	-1,802716385	-2,09678811	0,294071725
229051_at	-	-1,802716385	-2,09678811	0,294071725
224041_at	TTTY6 /// TTTY6B	-1,802716385	-2,09678811	0,294071725
1556619_at	SHISA9	-1,802716385	-2,09678811	0,294071725
217008_s_at	GRM7	-1,802716385	-2,09678811	0,294071725
1561125_at	MTHFD1L	-1,802716385	-2,09678811	0,294071725
1556847_s_at	-	-1,802716385	-2,09678811	0,294071725
208552_at	GRIK4	-1,802716385	-2,09678811	0,294071725
1553585_a_at	MAGEA10-MAGEA	-1,802716385	-2,09678811	0,294071725

1560208_at	-	-1,802716385	-2,09678811	0,294071725
1559000_at	C10orf108	-1,802716385	-2,09678811	0,294071725
242833_at	-	-1,802716385	-2,09678811	0,294071725
241112_at	ZNF517	-1,802716385	-2,09678811	0,294071725
210584_s_at	POLDIP3 /// RRP7E	-1,802716385	-2,09678811	0,294071725
237950_s_at	-	-1,802716385	-2,09678811	0,294071725
238947_at	-	-1,802716385	-2,09678811	0,294071725
1567590_at	-	-1,802716385	-2,09678811	0,294071725
239057_at	LMOD2	-1,802716385	-2,09678811	0,294071725
1554657_a_at	C20orf26	-1,802716385	-2,09678811	0,294071725
214967_at	-	-1,802716385	-2,09678811	0,294071725
200986_at	SERPING1	-1,802716385	-2,09678811	0,294071725
231100_at	RRAD	-1,802716385	-2,09678811	0,294071725
213736_at	COX5B	1,701542258	1,408220608	0,29332165
226738_at	WDR81	1,760255626	1,46719264	0,293062986
210187_at	FKBP1A	1,094118704	0,80114751	0,292971194
239607_at	GPR156	0,735999505	0,443143223	0,292856282
226422_at	ERGIC2	4,592740505	4,29990671	0,292833795
221554_at	STRADA	2,71254166	2,419753897	0,292787763
242438_at	ASXL1	2,678352494	2,385765892	0,292586601
223918_at	ACSL6 /// LOC1005	-1,893506789	-2,186086603	0,292579814
241265_x_at	-	-1,893506789	-2,186086603	0,292579814
1570180_at	-	-1,893506789	-2,186086603	0,292579814
1559470_at	D21S2088E	-1,893506789	-2,186086603	0,292579814
1559412_at	LINC00478	-1,893506789	-2,186086603	0,292579814
224093_at	IFNK	-1,893506789	-2,186086603	0,292579814
1556314_a_at	-	-1,893506789	-2,186086603	0,292579814
1566896_at	-	-1,893506789	-2,186086603	0,292579814
237288_at	TGM7	-1,893506789	-2,186086603	0,292579814
216896_at	COL4A3	-1,893506789	-2,186086603	0,292579814
1553798_a_at	FBXL13	-1,893506789	-2,186086603	0,292579814
208394_x_at	ESM1	-1,893506789	-2,186086603	0,292579814
211273_s_at	TBX1	-1,893506789	-2,186086603	0,292579814
235334_at	ST6GALNAC3	-1,893506789	-2,186086603	0,292579814
239752_at	-	-1,893506789	-2,186086603	0,292579814
228632_at	MEG9	-1,893506789	-2,186086603	0,292579814
213796_at	SPRR1A	-1,893506789	-2,186086603	0,292579814
209738_x_at	PSG6	-1,893506789	-2,186086603	0,292579814
242392_at	UBE2U	-1,893506789	-2,186086603	0,292579814
237787_at	-	-1,893506789	-2,186086603	0,292579814
204915_s_at	SOX11	-1,893506789	-2,186086603	0,292579814
1568821_at	TTC23	-1,893506789	-2,186086603	0,292579814
1555180_at	LOC100132686	-1,893506789	-2,186086603	0,292579814
240974_at	LOC100506851	-1,893506789	-2,186086603	0,292579814
232854_at	DIRAS1	-1,893506789	-2,186086603	0,292579814
240466_at	-	-1,893506789	-2,186086603	0,292579814
1559656_a_at	LOC100507244	-1,893506789	-2,186086603	0,292579814
1552583_s_at	ABCC13	-1,893506789	-2,186086603	0,292579814
243043_at	-	-1,893506789	-2,186086603	0,292579814
1555048_a_at	TSPEAR	-1,893506789	-2,186086603	0,292579814

1560787_at	QDPR	-1,893506789	-2,186086603	0,292579814
242444_at	C1QTNF6	-1,893506789	-2,186086603	0,292579814
232334_at	NXPH2	-1,893506789	-2,186086603	0,292579814
228682_at	-	-1,893506789	-2,186086603	0,292579814
1555053_at	SYT9	-1,893506789	-2,186086603	0,292579814
209772_s_at	CD24	-1,893506789	-2,186086603	0,292579814
202936_s_at	SOX9	-1,893506789	-2,186086603	0,292579814
219301_s_at	CNTNAP2	-1,893506789	-2,186086603	0,292579814
203639_s_at	FGFR2	-1,893506789	-2,186086603	0,292579814
236255_at	PLEKHG4B	-1,893506789	-2,186086603	0,292579814
225660_at	SEMA6A	-1,893506789	-2,186086603	0,292579814
223477_s_at	C12orf65	2,855122714	2,562621495	0,292501219
209053_s_at	WHSC1	3,592169709	3,299705477	0,292464231
225910_at	HELZ	2,348178434	2,055739171	0,292439264
219110_at	GAR1	4,498569607	4,206178	0,292391606
212153_at	POGZ	2,932161212	2,639836674	0,292324539
1559363_at	LOC283587	-1,712732543	-2,005029581	0,292297038
1554176_a_at	C3orf33	-1,712732543	-2,005029581	0,292297038
1564670_at	LOC100190938	-1,712732543	-2,005029581	0,292297038
1562071_at	-	-1,712732543	-2,005029581	0,292297038
205525_at	CALD1	-1,712732543	-2,005029581	0,292297038
206068_s_at	ACADL	-1,712732543	-2,005029581	0,292297038
214217_at	GRM5	-1,712732543	-2,005029581	0,292297038
234402_at	YME1L1	-1,712732543	-2,005029581	0,292297038
1569698_s_at	-	-1,712732543	-2,005029581	0,292297038
234205_at	-	-1,712732543	-2,005029581	0,292297038
232746_at	CXCR7	-1,712732543	-2,005029581	0,292297038
231190_at	LOC100653171 ///	-1,712732543	-2,005029581	0,292297038
1557898_at	-	-1,712732543	-2,005029581	0,292297038
207910_at	SCGB1D1	-1,712732543	-2,005029581	0,292297038
1568730_at	-	-1,712732543	-2,005029581	0,292297038
204380_s_at	FGFR3	-1,712732543	-2,005029581	0,292297038
1562791_at	MYCBPAP	-1,712732543	-2,005029581	0,292297038
1568634_a_at	LRRC66	-1,712732543	-2,005029581	0,292297038
1562217_at	FLJ34521	-1,712732543	-2,005029581	0,292297038
233279_at	-	-1,712732543	-2,005029581	0,292297038
215658_at	LOC100289255	-1,712732543	-2,005029581	0,292297038
213921_at	SST	-1,712732543	-2,005029581	0,292297038
226420_at	MECOM	-1,712732543	-2,005029581	0,292297038
1560774_at	-	-1,712732543	-2,005029581	0,292297038
1558476_at	BEND5	-1,712732543	-2,005029581	0,292297038
1559078_at	-	-1,712732543	-2,005029581	0,292297038
1557515_at	-	-1,712732543	-2,005029581	0,292297038
1560284_at	-	-1,712732543	-2,005029581	0,292297038
211120_x_at	ESR2	-1,712732543	-2,005029581	0,292297038
244816_at	-	-1,712732543	-2,005029581	0,292297038
207234_at	RFX3	-1,712732543	-2,005029581	0,292297038
215782_at	RAB40AL	-1,712732543	-2,005029581	0,292297038
204627_s_at	ITGB3	-1,712732543	-2,005029581	0,292297038
232302_at	PDLIM2	-1,712732543	-2,005029581	0,292297038

244807_at	C17orf76-AS1	-1,712732543	-2,005029581	0,292297038
238060_s_at	B4GALNT4	-1,712732543	-2,005029581	0,292297038
1553880_at	C10orf91	-1,712732543	-2,005029581	0,292297038
1558868_a_at	DSE	-1,712732543	-2,005029581	0,292297038
204083_s_at	TPM2	-1,712732543	-2,005029581	0,292297038
236345_at	TBXAS1	-1,712732543	-2,005029581	0,292297038
231936_at	HOXC9	-1,712732543	-2,005029581	0,292297038
230020_at	NAA38	0,677745787	0,385582632	0,292163155
213970_at	RABL3	1,625482993	1,333668715	0,291814279
238266_at	-	0,001114523	-0,290694975	0,291809498
237947_at	-	0,001114523	-0,290694975	0,291809498
1559997_x_at	SAMD14	0,001114523	-0,290694975	0,291809498
221713_s_at	MAP6D1	0,001114523	-0,290694975	0,291809498
1563800_at	LOC100506870 ///	0,001114523	-0,290694975	0,291809498
228370_at	LOC100506948 ///	2,096248267	1,804519951	0,291728317
227547_at	-	2,664625637	2,373249966	0,291375671
226643_s_at	NUDCD2	1,775035811	1,483698568	0,291337243
225025_at	IGSF8	0,491906512	0,20062106	0,291285452
208270_s_at	RNPEP	3,313347235	3,02208274	0,291264495
1553810_a_at	KIAA1524	1,160996507	0,869824768	0,291171739
219224_x_at	ZNF408	1,830468611	1,539450683	0,291017929
211256_x_at	BTN2A1	1,830468611	1,539450683	0,291017929
219402_s_at	DERL1	4,739280418	4,448392964	0,290887454
215676_at	BRF1	-0,332405896	-0,623254098	0,290848203
1563074_at	LOC255654	-0,332405896	-0,623254098	0,290848203
204805_s_at	H1FX	2,507817181	2,2171284	0,290688781
213892_s_at	APRT	4,223646624	3,933228921	0,290417703
201258_at	RPS16	7,974935507	7,684583857	0,29035165
226179_at	SLC25A37	1,345611839	1,055317091	0,290294747
225794_s_at	C22orf32	4,806642953	4,516440716	0,290202237
205281_s_at	PIGA	1,578719607	1,28853674	0,290182867
212072_s_at	CSNK2A1 /// CSNK	4,030423661	3,740664823	0,289758838
216583_x_at	-	2,138605583	1,848871029	0,289734555
226101_at	PRKCE	4,362103226	4,07248815	0,289615076
229083_at	-	2,692288672	2,403191094	0,289097577
203677_s_at	TARBP2	2,410823487	2,121960601	0,288862886
221781_s_at	DNAJC10	2,410823487	2,121960601	0,288862886
224165_s_at	IQCH	-0,856259172	-1,145057014	0,288797842
238878_at	ARX	-0,856259172	-1,145057014	0,288797842
214287_s_at	CDK13	-0,856259172	-1,145057014	0,288797842
229186_s_at	ZFP64	-0,856259172	-1,145057014	0,288797842
1560974_s_at	NOS1	-0,856259172	-1,145057014	0,288797842
1555904_at	-	-0,856259172	-1,145057014	0,288797842
219121_s_at	ESRP1	-0,856259172	-1,145057014	0,288797842
205864_at	SLC7A4	-0,856259172	-1,145057014	0,288797842
243830_at	-	-0,856259172	-1,145057014	0,288797842
224498_x_at	AXIN2	-0,856259172	-1,145057014	0,288797842
1563529_at	HYDIN2	-0,856259172	-1,145057014	0,288797842
231528_at	-	-0,856259172	-1,145057014	0,288797842
231036_at	LOC100505774	-0,856259172	-1,145057014	0,288797842

1557274_at	LOC100506494	-0,856259172	-1,145057014	0,288797842
231748_at	ULBP3	-0,856259172	-1,145057014	0,288797842
1557697_at	-	-0,856259172	-1,145057014	0,288797842
212655_at	ZCCHC14	-0,856259172	-1,145057014	0,288797842
216901_s_at	IKZF1	-0,856259172	-1,145057014	0,288797842
228230_at	PRIC285	-0,856259172	-1,145057014	0,288797842
218504_at	FAHD2A	3,493994216	3,205255599	0,288738617
210907_s_at	PDCD10	6,073926283	5,785272932	0,288653351
217941_s_at	ERBB2IP	4,398126622	4,109561281	0,288565341
213185_at	KIAA0556	1,000459215	0,711934445	0,28852477
1561723_at	-	-0,994186487	-1,282520722	0,288334235
1559988_at	ZNF483	-0,994186487	-1,282520722	0,288334235
223734_at	MGARP	-0,994186487	-1,282520722	0,288334235
1568698_at	TMEM232	-0,994186487	-1,282520722	0,288334235
1555135_at	-	-0,994186487	-1,282520722	0,288334235
1552687_a_at	C20orf152	-0,994186487	-1,282520722	0,288334235
1569900_at	FLJ16124	-0,994186487	-1,282520722	0,288334235
1566609_at	-	-0,994186487	-1,282520722	0,288334235
1553555_at	TAS2R38	-0,994186487	-1,282520722	0,288334235
242114_at	-	-0,994186487	-1,282520722	0,288334235
1557769_at	-	-0,994186487	-1,282520722	0,288334235
1566210_at	-	-0,994186487	-1,282520722	0,288334235
238777_x_at	ATMIN	-0,994186487	-1,282520722	0,288334235
234074_at	-	-0,994186487	-1,282520722	0,288334235
206620_at	GRAP	-0,994186487	-1,282520722	0,288334235
220870_at	-	-0,994186487	-1,282520722	0,288334235
239820_at	-	-0,994186487	-1,282520722	0,288334235
220749_at	C10orf68	-0,994186487	-1,282520722	0,288334235
1563045_at	-	-0,994186487	-1,282520722	0,288334235
242022_at	-	-0,994186487	-1,282520722	0,288334235
241300_at	-	-0,994186487	-1,282520722	0,288334235
234328_at	-	-0,994186487	-1,282520722	0,288334235
215371_at	MED27	-0,994186487	-1,282520722	0,288334235
239837_at	ADAM11	-0,994186487	-1,282520722	0,288334235
237432_at	-	-0,994186487	-1,282520722	0,288334235
224778_s_at	TAOK1	3,132805465	2,844606929	0,288198536
224768_at	IWS1	3,505636113	3,217691783	0,28794433
205813_s_at	MAT1A	-0,230432956	-0,518232988	0,287800033
211200_s_at	EFCAB2	-0,230432956	-0,518232988	0,287800033
1556364_at	ADAMTS9-AS2	-0,230432956	-0,518232988	0,287800033
1563668_at	MORN1	-0,230432956	-0,518232988	0,287800033
239687_at	SLX4	-0,230432956	-0,518232988	0,287800033
204421_s_at	FGF2	-0,230432956	-0,518232988	0,287800033
207122_x_at	SULT1A2	1,730929079	1,44320939	0,287719689
203831_at	R3HDM2	2,365121417	2,077419168	0,287702249
223818_s_at	RSF1	1,686984721	1,399283506	0,287701215
1566862_at	-	-1,982324124	-2,27001641	0,287692286
237723_at	-	-1,982324124	-2,27001641	0,287692286
215665_at	-	-1,982324124	-2,27001641	0,287692286
241271_at	-	-1,982324124	-2,27001641	0,287692286

237094_at	FAM19A5	-1,982324124	-2,27001641	0,287692286
231725_at	PCDHB2	-1,982324124	-2,27001641	0,287692286
230495_at	LOC150568	-1,982324124	-2,27001641	0,287692286
244159_at	-	-1,982324124	-2,27001641	0,287692286
1561017_at	-	-1,982324124	-2,27001641	0,287692286
232799_at	-	-1,982324124	-2,27001641	0,287692286
229357_at	ADAMTS5	-1,982324124	-2,27001641	0,287692286
1555195_at	FBXO36	-1,982324124	-2,27001641	0,287692286
243884_at	TEX26	-1,982324124	-2,27001641	0,287692286
237672_at	-	-1,982324124	-2,27001641	0,287692286
226069_at	PRICKLE1	-1,982324124	-2,27001641	0,287692286
1560556_a_at	PLEKHA8	-1,982324124	-2,27001641	0,287692286
241592_at	LOC100507594	-1,982324124	-2,27001641	0,287692286
216034_at	ZNF280A	-1,982324124	-2,27001641	0,287692286
215893_x_at	-	-1,982324124	-2,27001641	0,287692286
236409_at	-	-1,982324124	-2,27001641	0,287692286
1568908_at	-	-1,982324124	-2,27001641	0,287692286
240898_at	SPAG16	-1,982324124	-2,27001641	0,287692286
1566783_at	-	-1,982324124	-2,27001641	0,287692286
227984_at	LMF1	-1,982324124	-2,27001641	0,287692286
1561565_at	-	-1,982324124	-2,27001641	0,287692286
236412_at	LOC100505983	-1,982324124	-2,27001641	0,287692286
1555052_a_at	SYT9	-1,982324124	-2,27001641	0,287692286
244581_at	ZBTB20	-1,982324124	-2,27001641	0,287692286
1563522_at	DDX10	-1,982324124	-2,27001641	0,287692286
201976_s_at	MYO10	-1,982324124	-2,27001641	0,287692286
1560628_at	-	-1,982324124	-2,27001641	0,287692286
233188_at	GALNTL4	-1,982324124	-2,27001641	0,287692286
236289_at	LOC100506563	-1,982324124	-2,27001641	0,287692286
230377_s_at	TBC1D2B	-1,982324124	-2,27001641	0,287692286
237867_s_at	PID1	-1,982324124	-2,27001641	0,287692286
1570190_at	LSAMP	-1,982324124	-2,27001641	0,287692286
217477_at	PIP5K1B	-1,982324124	-2,27001641	0,287692286
221684_s_at	NYX	-1,982324124	-2,27001641	0,287692286
220492_s_at	OTOF	-1,982324124	-2,27001641	0,287692286
1555962_at	B3GNT7	-1,982324124	-2,27001641	0,287692286
224006_at	LOC100507377	-1,982324124	-2,27001641	0,287692286
201075_s_at	SMARCC1	2,732422196	2,444951949	0,287470247
220497_at	ZNF214	-2,569309811	-2,856570137	0,287260325
1562998_at	-	-2,569309811	-2,856570137	0,287260325
240463_at	-	-2,569309811	-2,856570137	0,287260325
238178_at	-	-2,569309811	-2,856570137	0,287260325
201361_at	TMEM109	3,566680842	3,279515225	0,287165617
219873_at	COLEC11	1,116462765	0,829309537	0,287153228
230934_at	LOC100506057 ///	1,116462765	0,829309537	0,287153228
219649_at	ALG6	3,765107151	3,478068945	0,287038206
237520_x_at	-	-2,641232718	-2,928183529	0,286950811
210813_s_at	XRCC4	1,307201325	1,020514937	0,286686388
235181_at	TYW5	1,307201325	1,020514937	0,286686388
231001_at	FIBIN	-1,624955693	-1,911206569	0,286250876

1553869_at	SESN3	-1,624955693	-1,911206569	0,286250876
1562822_at	-	-1,624955693	-1,911206569	0,286250876
227400_at	NFIX	-1,624955693	-1,911206569	0,286250876
215347_at	-	-1,624955693	-1,911206569	0,286250876
1556687_a_at	CLDN10	-1,624955693	-1,911206569	0,286250876
203807_x_at	CSH2	-1,624955693	-1,911206569	0,286250876
232994_s_at	RGNEF	-1,624955693	-1,911206569	0,286250876
1566228_at	-	-1,624955693	-1,911206569	0,286250876
243598_at	-	-1,624955693	-1,911206569	0,286250876
236323_at	PVRL3-AS1	-1,624955693	-1,911206569	0,286250876
238576_at	MOCOS	-1,624955693	-1,911206569	0,286250876
228343_at	POU2F2	-1,624955693	-1,911206569	0,286250876
223842_s_at	SCARA3	-1,624955693	-1,911206569	0,286250876
204437_s_at	FOLR1	-1,624955693	-1,911206569	0,286250876
233471_at	PTPN5	-1,624955693	-1,911206569	0,286250876
216879_at	HR44	-1,624955693	-1,911206569	0,286250876
1553637_s_at	TMCO5A	-1,624955693	-1,911206569	0,286250876
223865_at	SOX6	-1,624955693	-1,911206569	0,286250876
226809_at	LOC100216479 ///	-1,624955693	-1,911206569	0,286250876
233317_at	CD9	-1,624955693	-1,911206569	0,286250876
237453_at	ZNF529	-1,624955693	-1,911206569	0,286250876
1556891_at	SORCS1	-1,624955693	-1,911206569	0,286250876
237486_at	-	-1,624955693	-1,911206569	0,286250876
205650_s_at	FGA	-1,624955693	-1,911206569	0,286250876
240589_at	LINC00313	-1,624955693	-1,911206569	0,286250876
230778_at	-	-1,624955693	-1,911206569	0,286250876
230195_at	LOC100131138	-1,624955693	-1,911206569	0,286250876
243987_at	-	-1,624955693	-1,911206569	0,286250876
214207_s_at	CARD10	-1,624955693	-1,911206569	0,286250876
233394_at	NFIB	-1,624955693	-1,911206569	0,286250876
242596_at	-	-1,624955693	-1,911206569	0,286250876
239308_at	DYRK1A	-1,624955693	-1,911206569	0,286250876
219377_at	FAM59A	-1,624955693	-1,911206569	0,286250876
225046_at	LOC389831	3,768156719	3,481966713	0,286190006
217880_at	CDC27	4,005144537	3,719032089	0,286112448
239962_at	-	-2,250580859	-2,536339282	0,285758422
1563032_at	-	-2,250580859	-2,536339282	0,285758422
233332_at	-	-2,250580859	-2,536339282	0,285758422
226302_at	ATP8B1	-2,250580859	-2,536339282	0,285758422
229973_at	C1orf173	-2,250580859	-2,536339282	0,285758422
1553930_at	TAAR1	-2,250580859	-2,536339282	0,285758422
1562932_at	-	-2,250580859	-2,536339282	0,285758422
216669_at	-	-2,250580859	-2,536339282	0,285758422
211298_s_at	ALB	-2,250580859	-2,536339282	0,285758422
1565769_at	-	-2,250580859	-2,536339282	0,285758422
220356_at	CORIN	-2,250580859	-2,536339282	0,285758422
241295_at	-	-2,250580859	-2,536339282	0,285758422
1561294_a_at	LOC100128554	-2,250580859	-2,536339282	0,285758422
231787_at	SLC25A27	-2,250580859	-2,536339282	0,285758422
243794_at	-	-2,250580859	-2,536339282	0,285758422

1569773_at	ATP8A1	-2,250580859	-2,536339282	0,285758422
1562905_at	-	-2,250580859	-2,536339282	0,285758422
242249_at	-	-2,250580859	-2,536339282	0,285758422
206177_s_at	ARG1	-2,250580859	-2,536339282	0,285758422
211741_x_at	PSG3	-2,250580859	-2,536339282	0,285758422
1563070_at	-	-2,250580859	-2,536339282	0,285758422
236443_at	-	-2,250580859	-2,536339282	0,285758422
233032_x_at	-	-2,250580859	-2,536339282	0,285758422
217585_at	NEBL	-2,250580859	-2,536339282	0,285758422
232224_at	MASP1	-2,250580859	-2,536339282	0,285758422
232668_at	-	-2,250580859	-2,536339282	0,285758422
208361_s_at	POLR3D	1,92317941	1,637482551	0,285696859
203458_at	SPR	1,871521385	1,586121802	0,285399583
207064_s_at	AOC2	-0,730013898	-1,015398016	0,285384118
1553347_s_at	KCNA6	-0,730013898	-1,015398016	0,285384118
204311_at	ATP1B2	-0,730013898	-1,015398016	0,285384118
236319_at	-	-0,730013898	-1,015398016	0,285384118
241514_at	-	-0,730013898	-1,015398016	0,285384118
235528_at	GUCA1B	-0,730013898	-1,015398016	0,285384118
233741_at	FTCD	-0,730013898	-1,015398016	0,285384118
218838_s_at	TTC31	2,401508182	2,116147513	0,285360669
200089_s_at	RPL4 /// SNORD16	7,458815796	7,173637883	0,285177913
233789_at	-	-2,495152701	-2,780225148	0,285072447
1559503_a_at	LOC441666	-2,495152701	-2,780225148	0,285072447
231680_at	-	-2,495152701	-2,780225148	0,285072447
242193_at	LOC100130155	-2,495152701	-2,780225148	0,285072447
239006_at	SLC26A7	-2,495152701	-2,780225148	0,285072447
240348_at	-	-2,495152701	-2,780225148	0,285072447
231512_at	-	-2,495152701	-2,780225148	0,285072447
217428_s_at	COL10A1	-2,495152701	-2,780225148	0,285072447
1560884_at	SLC17A1	-2,495152701	-2,780225148	0,285072447
1556289_at	-	-2,495152701	-2,780225148	0,285072447
207014_at	GABRA2	-2,495152701	-2,780225148	0,285072447
1555346_at	CDC20B	-2,495152701	-2,780225148	0,285072447
237580_at	-	-2,495152701	-2,780225148	0,285072447
1555585_a_at	FAM71B	-2,495152701	-2,780225148	0,285072447
1554714_at	RELL1	-1,137208348	-1,422257026	0,285048678
237691_x_at	ENO3	-1,137208348	-1,422257026	0,285048678
237761_at	-	-1,137208348	-1,422257026	0,285048678
208046_at	HIST1H4A /// HIST	-1,137208348	-1,422257026	0,285048678
1557003_at	TTC23L	-1,137208348	-1,422257026	0,285048678
1566524_a_at	-	-1,137208348	-1,422257026	0,285048678
1568636_a_at	-	-1,137208348	-1,422257026	0,285048678
244564_at	-	-1,137208348	-1,422257026	0,285048678
233176_at	LOC100507642	-1,137208348	-1,422257026	0,285048678
241075_at	RIMKLA	-1,137208348	-1,422257026	0,285048678
215414_at	FARS2	-1,137208348	-1,422257026	0,285048678
219525_at	SLC47A1	-1,137208348	-1,422257026	0,285048678
213759_at	ARL4C	-1,137208348	-1,422257026	0,285048678
242050_at	LRRC7	-1,137208348	-1,422257026	0,285048678

237894_at	C3orf22	-1,137208348	-1,422257026	0,285048678
233336_at	-	-1,137208348	-1,422257026	0,285048678
214907_at	CEACAM21	-1,137208348	-1,422257026	0,285048678
1570344_at	-	-1,137208348	-1,422257026	0,285048678
214050_at	-	-1,137208348	-1,422257026	0,285048678
206538_at	MRAS	-1,137208348	-1,422257026	0,285048678
208982_at	PECAM1	-1,137208348	-1,422257026	0,285048678
1558546_at	DNASE1	1,071336699	0,786391549	0,28494515
218719_s_at	GINS3	2,190140919	1,905294823	0,284846096
218355_at	KIF4A	3,261582786	2,97688045	0,284702337
241647_x_at	-	-2,708365344	-2,993014768	0,284649424
1561386_at	-	0,167727503	-0,116767475	0,284494978
229722_at	LOC100270804	0,167727503	-0,116767475	0,284494978
239790_s_at	-	0,167727503	-0,116767475	0,284494978
215760_s_at	SBNO2	0,167727503	-0,116767475	0,284494978
219502_at	NEIL3	3,588695979	3,304296123	0,284399856
222531_s_at	AP5M1	3,434684504	3,150841611	0,283842894
210970_s_at	IBTK	3,739061547	3,45525776	0,283803787
203652_at	MAP3K11	1,440265662	1,156599976	0,283665686
218286_s_at	RNF7	3,858118781	3,5744736	0,283645181
230405_at	C5orf56	2,798156197	2,514786608	0,283369589
218677_at	S100A14	-0,135065865	-0,418248858	0,283182993
226553_at	TMPRSS2	-0,135065865	-0,418248858	0,283182993
210237_at	ARTN	-0,135065865	-0,418248858	0,283182993
202919_at	MOB4	3,228532861	2,945366812	0,283166049
211930_at	HNRNPA3	2,752615529	2,469496389	0,28311914
213847_at	PRPH	1,326463531	1,043469112	0,282994419
222493_s_at	ZFAND3	1,326463531	1,043469112	0,282994419
209332_s_at	MAX	4,10471458	3,82195864	0,28275594
226619_at	SENP1	2,523100136	2,24081821	0,282281926
222674_at	C9orf114	0,900065578	0,61779983	0,282265748
211513_s_at	OGFR	0,648195588	0,366031214	0,282164374
218473_s_at	GLT25D1	1,983901861	1,701800759	0,282101101
203594_at	RTCA	4,927145108	4,645442219	0,281702889
221382_at	-	0,39105295	0,109458907	0,281594044
1567222_x_at	ELOVL5	0,319287178	0,03784737	0,281439808
215130_s_at	IQCK	0,58788177	0,306454867	0,281426903
230018_at	DPP9	0,58788177	0,306454867	0,281426903
1552628_a_at	HERPUD2	3,748268542	3,466845432	0,28142311
217928_s_at	PPP6R3	2,468978138	2,187622928	0,28135521
219294_at	CENPQ	1,671397663	1,390070295	0,281327368
212436_at	TRIM33	2,932161212	2,65087728	0,281283932
220712_at	C8orf60	0,244681185	-0,036543687	0,281224872
232680_at	HDGFL1	0,244681185	-0,036543687	0,281224872
225221_at	ZKSCAN1	3,732463367	3,451657924	0,280805444
219120_at	C2orf44	2,209870035	1,929092307	0,280777728
213507_s_at	KPNB1	6,073076138	5,792306215	0,280769923
219897_at	RNF122	0,458905032	0,178191865	0,280713166
213360_s_at	POM121 /// POM1	4,521773976	4,241210072	0,280563905
212204_at	TMEM87A	3,544559557	3,264254379	0,280305179

237311_at	-	-0,609040214	-0,889222211	0,280181997
221297_at	GPRC5D	-0,609040214	-0,889222211	0,280181997
214365_at	TPM3	-0,609040214	-0,889222211	0,280181997
216848_at	-	-0,609040214	-0,889222211	0,280181997
232227_at	LOC100505976	-0,609040214	-0,889222211	0,280181997
1569634_at	SEPSECS	-0,609040214	-0,889222211	0,280181997
215481_s_at	PEX5	-0,609040214	-0,889222211	0,280181997
243457_s_at	ZNF214	-0,609040214	-0,889222211	0,280181997
230284_at	MYOM3	-0,609040214	-0,889222211	0,280181997
241743_at	-	-0,609040214	-0,889222211	0,280181997
229832_x_at	SH3TC1	-0,609040214	-0,889222211	0,280181997
1552535_at	CLDN19	-0,609040214	-0,889222211	0,280181997
230531_at	KCNC3	-0,609040214	-0,889222211	0,280181997
1563943_at	PPARGC1B	-0,609040214	-0,889222211	0,280181997
224794_s_at	CERCAM /// LOC1(	-0,609040214	-0,889222211	0,280181997
229054_at	ZFP36L1	-0,609040214	-0,889222211	0,280181997
206123_at	LLGL1	0,524545436	0,244646697	0,279898739
231921_at	DCAF17	0,524545436	0,244646697	0,279898739
222503_s_at	WDR41	4,969542968	4,689660397	0,279882571
201206_s_at	RRBP1	1,287793882	1,008210238	0,279583645
223461_at	TBC1D7	3,525566827	3,246064373	0,279502454
204449_at	PDCL	2,267141818	1,987810614	0,279331204
200625_s_at	CAP1	5,628888391	5,349852336	0,279036056
1561094_a_at	SLC22A25	-2,416625063	-2,695640852	0,279015789
233295_at	-	-2,416625063	-2,695640852	0,279015789
222959_at	CNGB3	-2,416625063	-2,695640852	0,279015789
1556351_at	HCN1	-2,416625063	-2,695640852	0,279015789
1554723_x_at	-	-2,416625063	-2,695640852	0,279015789
1553320_s_at	CDC14C	-2,416625063	-2,695640852	0,279015789
219619_at	DIRAS2	-2,416625063	-2,695640852	0,279015789
1560251_at	LOC645485	-2,416625063	-2,695640852	0,279015789
1556447_at	LINC00606	-2,416625063	-2,695640852	0,279015789
237540_at	LOC100506767	-2,416625063	-2,695640852	0,279015789
221127_s_at	DKK3	-2,416625063	-2,695640852	0,279015789
214397_at	MBD2	-2,416625063	-2,695640852	0,279015789
1568736_s_at	-	-2,416625063	-2,695640852	0,279015789
240196_at	-	-2,416625063	-2,695640852	0,279015789
234872_at	-	-2,416625063	-2,695640852	0,279015789
225731_at	ANKRD50	-2,416625063	-2,695640852	0,279015789
205860_x_at	FOLH1 /// FOLH1B	-2,416625063	-2,695640852	0,279015789
236326_at	HDAC7	-1,289805289	-1,568385657	0,278580368
206199_at	CEACAM7	-1,289805289	-1,568385657	0,278580368
1564344_at	ATP9B	-1,289805289	-1,568385657	0,278580368
204586_at	BSN	-1,289805289	-1,568385657	0,278580368
225746_at	RAB11FIP4	-1,289805289	-1,568385657	0,278580368
243676_at	-	-1,289805289	-1,568385657	0,278580368
227899_at	VIT	-1,289805289	-1,568385657	0,278580368
239001_at	MGST1	-1,289805289	-1,568385657	0,278580368
209982_s_at	NRXN2	-1,289805289	-1,568385657	0,278580368
242415_at	FBRSL1	-1,289805289	-1,568385657	0,278580368

237280_at	TCTE1	-1,289805289	-1,568385657	0,278580368
205491_s_at	GJB3	-1,289805289	-1,568385657	0,278580368
237089_at	-	-1,289805289	-1,568385657	0,278580368
1561389_at	-	-1,289805289	-1,568385657	0,278580368
1559891_at	HMGA2	-1,289805289	-1,568385657	0,278580368
235627_at	PFN4	-1,289805289	-1,568385657	0,278580368
241329_s_at	-	-1,289805289	-1,568385657	0,278580368
235596_at	-	-1,289805289	-1,568385657	0,278580368
1566149_at	CALML4	-1,289805289	-1,568385657	0,278580368
216395_at	FBXL18	-1,289805289	-1,568385657	0,278580368
207077_at	CELA2B	-1,289805289	-1,568385657	0,278580368
207866_at	BMP8A	-1,289805289	-1,568385657	0,278580368
243932_at	-	-1,289805289	-1,568385657	0,278580368
218003_s_at	FKBP3 /// LOC100(	4,875022718	4,596446615	0,278576102
1556188_a_at	ZNF555	-0,49284695	-0,771340337	0,278493387
202790_at	CLDN7	-0,49284695	-0,771340337	0,278493387
238882_at	-	-0,49284695	-0,771340337	0,278493387
1559957_a_at	LOC642852	-0,49284695	-0,771340337	0,278493387
223834_at	CD274	-0,49284695	-0,771340337	0,278493387
215217_at	IGKC	-0,043925712	-0,322340048	0,278414336
1563655_at	TNNT2	-0,043925712	-0,322340048	0,278414336
208864_s_at	TXN	6,999352301	6,721281555	0,278070746
232215_x_at	PRR11	4,096676757	3,818669263	0,278007495
217719_at	EIF3L	7,372299836	7,094560077	0,27773976
40524_at	PTPN21	-2,292987487	-2,570522741	0,277535255
200818_at	ATP5O	7,090842101	6,813620071	0,277222031
218428_s_at	REV1	3,418470674	3,141266133	0,277204541
209579_s_at	MBD4	5,508704656	5,231559804	0,277144852
214256_at	ATP10A	-0,384193355	-0,661178575	0,27698522
237494_at	-	-0,384193355	-0,661178575	0,27698522
226244_at	CLEC14A	-0,384193355	-0,661178575	0,27698522
222251_s_at	GMEB2	1,936380191	1,659532492	0,2768477
211230_s_at	PIK3CD	0,045517965	-0,231045907	0,276563871
1559224_at	LCE1E	0,045517965	-0,231045907	0,276563871
209963_s_at	EPOR	0,045517965	-0,231045907	0,276563871
204161_s_at	ENPP4	1,760255626	1,483698568	0,276557058
215405_at	-	-2,074223907	-2,350444759	0,276220852
234889_at	-	-2,074223907	-2,350444759	0,276220852
1556617_a_at	LSAMP-AS4	-2,074223907	-2,350444759	0,276220852
243551_at	-	-2,074223907	-2,350444759	0,276220852
210498_at	CLTC	-2,074223907	-2,350444759	0,276220852
228377_at	KLHL14	-2,074223907	-2,350444759	0,276220852
1553556_at	TAS2R40	-2,074223907	-2,350444759	0,276220852
1569331_at	-	-2,074223907	-2,350444759	0,276220852
244583_at	-	-2,074223907	-2,350444759	0,276220852
239618_at	SEC16B	-2,074223907	-2,350444759	0,276220852
1561699_a_at	-	-2,074223907	-2,350444759	0,276220852
1561098_at	LINC00616	-2,074223907	-2,350444759	0,276220852
1557504_at	-	-2,074223907	-2,350444759	0,276220852
1555646_at	Dbpht2	-2,074223907	-2,350444759	0,276220852

233150_at	GALNTL5	-2,074223907	-2,350444759	0,276220852
216778_s_at	CYLC1	-2,074223907	-2,350444759	0,276220852
233721_x_at	-	-2,074223907	-2,350444759	0,276220852
243572_at	-	-2,074223907	-2,350444759	0,276220852
208148_at	MYH2 /// MYH4	-2,074223907	-2,350444759	0,276220852
237303_at	-	-2,074223907	-2,350444759	0,276220852
207768_at	EGR4	-2,074223907	-2,350444759	0,276220852
237501_at	-	-2,074223907	-2,350444759	0,276220852
206749_at	CD1B	-2,074223907	-2,350444759	0,276220852
204855_at	SERPINB5	-2,074223907	-2,350444759	0,276220852
1561638_at	-	-2,074223907	-2,350444759	0,276220852
229218_at	COL1A2	-2,074223907	-2,350444759	0,276220852
233695_s_at	CECR2	-2,074223907	-2,350444759	0,276220852
1560905_at	-	-2,074223907	-2,350444759	0,276220852
207854_at	GYPE	-2,074223907	-2,350444759	0,276220852
210747_at	HLA-DQB1	-2,074223907	-2,350444759	0,276220852
208085_s_at	ARHGAP6	-2,074223907	-2,350444759	0,276220852
206983_at	CCR6	-2,074223907	-2,350444759	0,276220852
1564449_at	-	-2,074223907	-2,350444759	0,276220852
205242_at	CXCL13	-2,074223907	-2,350444759	0,276220852
1566267_at	-	-2,074223907	-2,350444759	0,276220852
216861_at	-	-2,074223907	-2,350444759	0,276220852
222486_s_at	ADAMTS1	-2,074223907	-2,350444759	0,276220852
231608_at	-	-2,074223907	-2,350444759	0,276220852
213779_at	EMID1	2,019346222	1,743236669	0,276109552
216547_at	-	2,896717728	2,621003244	0,275714484
221520_s_at	CDCA8	3,954683435	3,679352683	0,275330752
226257_x_at	MRPS22	4,082216372	3,806990944	0,275225428
225597_at	SLC45A4	1,246401134	0,971607464	0,274793669
229798_s_at	BRI3	1,246401134	0,971607464	0,274793669
217744_s_at	PERP	3,001575397	2,726850891	0,274724506
201994_at	MORF4L2	5,880953673	5,606237288	0,274716386
200991_s_at	SNX17	4,558198545	4,283545167	0,274653377
225361_x_at	FAM122B	4,323104796	4,04859535	0,274509447
1554017_at	RILPL1 /// SNRNP3	-0,280273599	-0,554696666	0,274423067
200010_at	RPL11	7,965936142	7,691606214	0,274329928
215136_s_at	EXOSC8	4,843588416	4,569475269	0,274113147
212928_at	TSPYL4	3,858118781	3,584031895	0,274086886
224148_at	FYB	-1,537851782	-1,811780055	0,273928273
238521_at	FGF12	-1,537851782	-1,811780055	0,273928273
228581_at	KCNJ10	-1,537851782	-1,811780055	0,273928273
213816_s_at	MET	-1,537851782	-1,811780055	0,273928273
243694_at	-	-1,537851782	-1,811780055	0,273928273
233607_at	-	-1,537851782	-1,811780055	0,273928273
208320_at	CABP1	-1,537851782	-1,811780055	0,273928273
241729_at	DOK6	-1,537851782	-1,811780055	0,273928273
220087_at	BCMO1	-1,537851782	-1,811780055	0,273928273
1562942_at	-	-1,537851782	-1,811780055	0,273928273
238726_at	-	-1,537851782	-1,811780055	0,273928273
1556459_at	ARHGAP22-IT1	-1,537851782	-1,811780055	0,273928273

1568554_x_at	-	-1,537851782	-1,811780055	0,273928273
1570297_at	-	-1,537851782	-1,811780055	0,273928273
229335_at	CADM4	-1,537851782	-1,811780055	0,273928273
207764_s_at	HIPK3	-1,537851782	-1,811780055	0,273928273
1567389_at	-	-1,537851782	-1,811780055	0,273928273
216132_at	-	-1,537851782	-1,811780055	0,273928273
1561226_at	XCR1	-1,537851782	-1,811780055	0,273928273
229073_at	PRTG	-1,537851782	-1,811780055	0,273928273
228938_at	MBP	-1,537851782	-1,811780055	0,273928273
1562032_at	-	-1,537851782	-1,811780055	0,273928273
1555665_at	-	-1,537851782	-1,811780055	0,273928273
234754_at	SLC37A1	-1,537851782	-1,811780055	0,273928273
1563809_a_at	MCF2L	-1,537851782	-1,811780055	0,273928273
1557197_a_at	LGALS3	-1,537851782	-1,811780055	0,273928273
211195_s_at	TP63	-1,537851782	-1,811780055	0,273928273
210089_s_at	LAMA4	-1,537851782	-1,811780055	0,273928273
236724_at	CFC1	-1,537851782	-1,811780055	0,273928273
243688_at	MGC45800	-1,537851782	-1,811780055	0,273928273
240553_at	TESC	-1,537851782	-1,811780055	0,273928273
223392_s_at	TSHZ3	-1,537851782	-1,811780055	0,273928273
203340_s_at	SLC25A12	3,007119335	2,733194118	0,273925217
208837_at	TMED3	3,6787904	3,405078581	0,273711819
224604_at	C4orf3	4,193391347	3,920124421	0,273266926
211927_x_at	EEF1G /// MIR365	8,45808863	8,184873229	0,273215401
1555272_at	RSPH10B /// RSPH	1,960819106	1,687645398	0,273173708
232902_s_at	RARS2	3,175897496	2,902771765	0,273125731
211013_x_at	PML	1,910557155	1,637482551	0,273074604
202050_s_at	ZMYM4	2,21987062	1,946903052	0,272967568
220526_s_at	MRPL20	5,566873789	5,294027118	0,272846671
223334_at	TMEM126A	5,215904344	4,943369689	0,272534655
211021_s_at	RGS14	0,127732831	-0,144765583	0,272498414
222467_s_at	PPP6R3	4,073484114	3,801081113	0,272403
206348_s_at	PDK3	2,096248267	1,823960141	0,272288126
212895_s_at	ABR	2,41945542	2,147194726	0,272260693
206621_s_at	EIF4H	6,42156652	6,149620963	0,271945557
231111_at	-	1,510970097	1,239268196	0,271701902
212825_at	PAXIP1	3,694479488	3,422991983	0,271487505
208433_s_at	LRP8	2,636121902	2,364676067	0,271445835
214853_s_at	SHC1	2,914752575	2,64347734	0,271275235
221926_s_at	IL17RC	0,20740896	-0,063377083	0,270786043
235678_at	GM2A	0,95222556	0,681874787	0,270350773
212494_at	TENC1	-0,180990326	-0,45121326	0,270222934
210561_s_at	WSB1	4,267962641	3,997758123	0,270204517
244861_at	ZNF527	1,071336699	0,80114751	0,270189189
235497_at	LOC643837	0,820160788	0,550017041	0,270143747
224787_s_at	RAB18	3,513985141	3,244090313	0,269894828
208626_s_at	VAT1	2,374261973	2,104873932	0,269388042
217987_at	ASNSD1	4,341822193	4,07248815	0,269334043
37831_at	SIPA1L3	0,425434386	0,15610578	0,269328606
205340_at	ZBTB24	3,243218364	2,973964204	0,26925416

226551_at	RIPK1	1,458255986	1,189078962	0,269177024
210290_at	ZNF174	0,282358733	0,013190398	0,269168335
215048_at	ZNF280B	0,282358733	0,013190398	0,269168335
225701_at	AKNA	3,566680842	3,297572471	0,269108372
211098_x_at	TMCO1	3,855094415	3,586015261	0,269079154
234978_at	SLC36A4	1,760255626	1,491644074	0,268611552
213300_at	ATG2A	2,460890032	2,192329358	0,268560674
203482_at	FAM178A	1,88491011	1,616354037	0,268556074
203229_s_at	CLK2	3,382641505	3,114374925	0,26826658
201648_at	JAK1	4,693076946	4,424811729	0,268265217
223512_at	SAR1B	2,356619952	2,088449486	0,268170465
225732_at	KLHDC5	3,018305638	2,750185881	0,268119757
202978_s_at	CREBZF	2,582572508	2,314488991	0,268083517
204009_s_at	KRAS	3,886513476	3,619206676	0,2673068
1564064_a_at	ATP11B	2,031158928	1,76396664	0,267192288
224566_at	LOC100653017 ///	4,091994743	3,824839773	0,26715497
204912_at	IL10RA	1,610353504	1,343200523	0,267152981
223024_at	AP1M1	2,849132494	2,582046772	0,267085722
214787_at	DENND4A	2,56794744	2,300902761	0,267044678
239024_at	ZNF148	1,345611839	1,078619386	0,266992453
229022_at	ZFX	1,775035811	1,508107415	0,266928396
241614_at	-	-0,924000698	-1,19085694	0,266856242
1557623_at	-	-0,924000698	-1,19085694	0,266856242
1565830_at	-	-0,924000698	-1,19085694	0,266856242
243780_at	-	-0,924000698	-1,19085694	0,266856242
242841_at	LOC100505570	-0,924000698	-1,19085694	0,266856242
235886_at	LOC100505666	-0,924000698	-1,19085694	0,266856242
238839_at	OTX1	-0,924000698	-1,19085694	0,266856242
1566927_at	LINC00527	-0,924000698	-1,19085694	0,266856242
203287_at	LAD1	-0,924000698	-1,19085694	0,266856242
215489_x_at	HOMER3	-0,924000698	-1,19085694	0,266856242
231867_at	ODZ2	-0,924000698	-1,19085694	0,266856242
204021_s_at	PURA	2,966903803	2,700070732	0,266833072
218782_s_at	ATAD2	4,05770789	3,790897218	0,266810672
237489_at	-	-2,336678463	-2,603471267	0,266792803
241469_at	HFM1	-2,336678463	-2,603471267	0,266792803
1563221_at	LOC414300	-2,336678463	-2,603471267	0,266792803
216539_at	ATXN3L	-2,336678463	-2,603471267	0,266792803
1556421_at	LOC286189	-2,336678463	-2,603471267	0,266792803
222342_at	-	-2,336678463	-2,603471267	0,266792803
206434_at	SPOCK3	-2,336678463	-2,603471267	0,266792803
1554996_at	LOC643955 /// ZNI	-2,336678463	-2,603471267	0,266792803
230891_at	TUBE1	-2,336678463	-2,603471267	0,266792803
230962_at	DCLK1	-2,336678463	-2,603471267	0,266792803
1563162_at	-	-2,336678463	-2,603471267	0,266792803
233906_at	-	-2,336678463	-2,603471267	0,266792803
1555687_a_at	CLEC4C	-2,336678463	-2,603471267	0,266792803
234474_x_at	IL6ST	-2,336678463	-2,603471267	0,266792803
243273_at	-	-2,336678463	-2,603471267	0,266792803
227554_at	MAGI2-AS3	-2,336678463	-2,603471267	0,266792803

240864_at	-	-2,336678463	-2,603471267	0,266792803
1553907_a_at	EXD1	-2,336678463	-2,603471267	0,266792803
239822_at	LOC100506795	-2,336678463	-2,603471267	0,266792803
1552763_at	TMEM67	-2,336678463	-2,603471267	0,266792803
231798_at	NOG	-2,336678463	-2,603471267	0,266792803
240575_at	-	-2,336678463	-2,603471267	0,266792803
1569508_at	PRDM5	-2,336678463	-2,603471267	0,266792803
241873_at	-	-2,336678463	-2,603471267	0,266792803
223864_at	ANKRD30A	-2,336678463	-2,603471267	0,266792803
230900_at	CCDC110	-2,336678463	-2,603471267	0,266792803
243713_at	-	-2,336678463	-2,603471267	0,266792803
229635_at	LOC100505702	-2,336678463	-2,603471267	0,266792803
209396_s_at	CHI3L1	-2,336678463	-2,603471267	0,266792803
1561817_at	-	-2,336678463	-2,603471267	0,266792803
1565601_at	-	-2,336678463	-2,603471267	0,266792803
218087_s_at	SORBS1	-2,336678463	-2,603471267	0,266792803
336_at	TBXA2R	-0,919292447	-1,186039887	0,26674744
204902_s_at	ATG4B	1,024284941	0,757638486	0,266646455
1560131_at	LOC100506497	-0,791294935	-1,057301851	0,266006916
1563271_at	-	-0,791294935	-1,057301851	0,266006916
230757_at	-	-0,791294935	-1,057301851	0,266006916
238745_at	-	-0,791294935	-1,057301851	0,266006916
235415_at	RPRD2	-0,791294935	-1,057301851	0,266006916
202827_s_at	MMP14	-0,791294935	-1,057301851	0,266006916
1560588_at	-	-0,791294935	-1,057301851	0,266006916
203108_at	GPRC5A	-0,791294935	-1,057301851	0,266006916
229368_s_at	ZFAND5	-0,791294935	-1,057301851	0,266006916
1556206_at	LOC100652856	-0,791294935	-1,057301851	0,266006916
206151_x_at	CELA3B	-0,791294935	-1,057301851	0,266006916
226126_at	TBCK	3,012642182	2,746644067	0,265998115
218040_at	PRPF38B	2,890618076	2,624682607	0,265935469
205346_at	ST3GAL2	-0,088974936	-0,354781582	0,265806646
221074_at	-	-0,088974936	-0,354781582	0,265806646
213441_x_at	SPDEF	0,900065578	0,634301128	0,26576445
210046_s_at	IDH2	4,323104796	4,057569918	0,265534879
204194_at	BACH1	2,824160383	2,558652146	0,265508237
220750_s_at	LEPRE1	1,788922638	1,523429058	0,26549358
211793_s_at	ABI2	1,65554524	1,390070295	0,265474944
224334_s_at	MRPL51 /// SPTLC	3,529682801	3,264254379	0,265428422
231784_s_at	DCAF13	3,261582786	2,99622929	0,265353497
237751_x_at	-	-2,163157732	-2,4284334	0,265275668
1553646_at	HDX	-2,163157732	-2,4284334	0,265275668
215284_at	-	-2,163157732	-2,4284334	0,265275668
237479_at	-	-2,163157732	-2,4284334	0,265275668
227690_at	GABRB3	-2,163157732	-2,4284334	0,265275668
207864_at	SCN7A	-2,163157732	-2,4284334	0,265275668
239921_at	COL28A1	-2,163157732	-2,4284334	0,265275668
1559529_at	PTK2	-2,163157732	-2,4284334	0,265275668
232606_at	ANK2	-2,163157732	-2,4284334	0,265275668
242777_at	-	-2,163157732	-2,4284334	0,265275668

241119_at	-	-2,163157732	-2,4284334	0,265275668
230303_at	SYNPR	-2,163157732	-2,4284334	0,265275668
1554235_at	CTNNA3	-2,163157732	-2,4284334	0,265275668
1562490_at	THEM5	-2,163157732	-2,4284334	0,265275668
1561998_at	-	-2,163157732	-2,4284334	0,265275668
1558658_at	ZNF391	-2,163157732	-2,4284334	0,265275668
1562927_at	-	-2,163157732	-2,4284334	0,265275668
217067_s_at	DMP1	-2,163157732	-2,4284334	0,265275668
1564152_at	FLJ35816	-2,163157732	-2,4284334	0,265275668
1553243_at	ITIH5	-2,163157732	-2,4284334	0,265275668
238588_at	-	-2,163157732	-2,4284334	0,265275668
1553944_at	CTAGE5 /// MIA2	-2,163157732	-2,4284334	0,265275668
240799_at	SLC35F4	-2,163157732	-2,4284334	0,265275668
1566269_at	RALGAPA1	-2,163157732	-2,4284334	0,265275668
1554707_at	SPATA6L	-2,163157732	-2,4284334	0,265275668
214451_at	TFAP2B	-2,163157732	-2,4284334	0,265275668
241282_at	-	-2,163157732	-2,4284334	0,265275668
204933_s_at	TNFRSF11B	-2,163157732	-2,4284334	0,265275668
202907_s_at	NBN	3,925077605	3,660020626	0,265056978
209286_at	CDC42EP3	1,094118704	0,829309537	0,264809167
201830_s_at	NET1	3,086998005	2,822379032	0,264618972
1552740_at	C2orf15 /// MRPL3	-1,06282519	-1,32740143	0,26457624
1554741_s_at	FGF7 /// KGFLP1 /,	-1,06282519	-1,32740143	0,26457624
1555136_at	FGD6	-1,06282519	-1,32740143	0,26457624
1561258_at	TMEM151B	-1,06282519	-1,32740143	0,26457624
228679_at	-	-1,06282519	-1,32740143	0,26457624
241337_at	-	-1,06282519	-1,32740143	0,26457624
234250_at	LOC100506405	-1,06282519	-1,32740143	0,26457624
231044_at	C1orf194	-1,06282519	-1,32740143	0,26457624
239513_at	C22orf45	-1,06282519	-1,32740143	0,26457624
1561610_at	-	-1,06282519	-1,32740143	0,26457624
1562972_at	LOC503519	-1,06282519	-1,32740143	0,26457624
1553159_at	DNAH11	-1,06282519	-1,32740143	0,26457624
208573_s_at	OR2H2	-1,06282519	-1,32740143	0,26457624
216379_x_at	CD24	-1,06282519	-1,32740143	0,26457624
209751_s_at	TRAPPC2 /// TRAP	3,018305638	2,753767314	0,264538323
228989_at	C18orf56	0,707657549	0,443143223	0,264514326
211773_s_at	ZKSCAN3	0,707657549	0,443143223	0,264514326
227732_at	ATXN7L1	0,707657549	0,443143223	0,264514326
227500_at	FBXL18	0,707657549	0,443143223	0,264514326
225390_s_at	KLF13	3,398333742	3,134198147	0,264135595
217970_s_at	CNOT6	2,75889404	2,49476365	0,26413039
212621_at	TMEM194A	3,327128828	3,063097042	0,264031786
235919_at	CEP78	2,62869474	2,364676067	0,264018673
209002_s_at	CALCOCO1	0,847529938	0,583607503	0,263922435
200869_at	RPL18A	8,240561991	7,976864465	0,263697526
225526_at	MKLN1	2,460890032	2,197404429	0,263485603
204353_s_at	POT1	3,065977723	2,802594885	0,263382838
1557950_at	NEMF	1,802733148	1,539450683	0,263282465
220523_at	EFHC2	-2,641232718	-2,904470894	0,263238176

206664_at	SI	-2,641232718	-2,904470894	0,263238176
222299_x_at	-	-2,641232718	-2,904470894	0,263238176
237351_at	LOC100652994 ///	-1,452713826	-1,715576125	0,262862299
1560002_at	FAM27A	-1,452713826	-1,715576125	0,262862299
237314_at	ENKUR	-1,452713826	-1,715576125	0,262862299
221105_at	-	-1,452713826	-1,715576125	0,262862299
1556175_at	MTSS1L	-1,452713826	-1,715576125	0,262862299
218186_at	RAB25	-1,452713826	-1,715576125	0,262862299
1566727_at	-	-1,452713826	-1,715576125	0,262862299
234354_x_at	ERBB2	-1,452713826	-1,715576125	0,262862299
230690_at	TUBB1	-1,452713826	-1,715576125	0,262862299
235877_at	-	-1,452713826	-1,715576125	0,262862299
233081_at	-	-1,452713826	-1,715576125	0,262862299
229783_at	AKAP13	-1,452713826	-1,715576125	0,262862299
240011_at	TTBK2	-1,452713826	-1,715576125	0,262862299
1566268_at	-	-1,452713826	-1,715576125	0,262862299
236774_at	-	-1,452713826	-1,715576125	0,262862299
1569741_at	-	-1,452713826	-1,715576125	0,262862299
232093_at	LINC00085	-1,452713826	-1,715576125	0,262862299
227831_at	TMEM245	-1,452713826	-1,715576125	0,262862299
1563854_s_at	LOC283045	-1,452713826	-1,715576125	0,262862299
1553418_a_at	CNTNAP5	-1,452713826	-1,715576125	0,262862299
238927_at	-	-1,452713826	-1,715576125	0,262862299
220779_at	PADI3	-1,452713826	-1,715576125	0,262862299
239983_at	SLC30A8	-1,452713826	-1,715576125	0,262862299
1564263_at	LINC00330	-1,452713826	-1,715576125	0,262862299
1561880_a_at	SIGLEC16	-1,452713826	-1,715576125	0,262862299
223149_s_at	PTPN23	-1,452713826	-1,715576125	0,262862299
1566501_at	-	-1,452713826	-1,715576125	0,262862299
243517_at	-	-1,452713826	-1,715576125	0,262862299
1556001_at	LOC284939 /// MA	1,364555912	1,101780212	0,2627757
205421_at	SLC22A3	-2,569309811	-2,832036647	0,262726836
229546_at	FAM84A /// LOC65	-2,569309811	-2,832036647	0,262726836
238234_at	-	-2,569309811	-2,832036647	0,262726836
1559545_at	LOC100506948 ///	-2,569309811	-2,832036647	0,262726836
236458_at	-	0,648195588	0,385582632	0,262612956
236243_at	ZCCHC6	0,648195588	0,385582632	0,262612956
224693_at	FAM210B	0,648195588	0,385582632	0,262612956
204669_s_at	RNF24	1,246401134	0,98381571	0,262585424
222991_s_at	UBQLN1	2,890618076	2,628204186	0,26241389
213677_s_at	PMS1	3,64600241	3,383632552	0,262369859
209798_at	NPAT	3,278243793	3,01591662	0,262327172
201769_at	CLINT1	4,254948633	3,992707333	0,2622413
1553061_at	OR6W1P	-0,66934516	-0,931539147	0,262193987
220825_s_at	KIRREL	-0,66934516	-0,931539147	0,262193987
1557022_at	-	-0,66934516	-0,931539147	0,262193987
218261_at	AP1M2	-0,66934516	-0,931539147	0,262193987
219331_s_at	KLHDC8A	-0,66934516	-0,931539147	0,262193987
239257_at	MOV10L1	-0,66934516	-0,931539147	0,262193987
230833_at	ACRBP	-0,66934516	-0,931539147	0,262193987

215279_at	-	-0,66934516	-0,931539147	0,262193987
244686_at	TCOF1	-0,66934516	-0,931539147	0,262193987
211050_x_at	LOC100134822 ///	-0,66934516	-0,931539147	0,262193987
229018_at	C12orf26	2,339581693	2,077419168	0,262162525
202102_s_at	BRD4	3,039689025	2,777528602	0,262160422
212149_at	EFR3A	4,817982599	4,555968307	0,262014292
1569712_at	-	-2,708365344	-2,970378591	0,262013247
33768_at	DMWD	1,356213841	1,094318034	0,261895807
220472_at	ZCCHC4	0,001114523	-0,260526297	0,26164082
218665_at	FZD4	0,001114523	-0,260526297	0,26164082
230856_at	-	0,001114523	-0,260526297	0,26164082
238401_at	ENDOV /// LOC10(	0,001114523	-0,260526297	0,26164082
63825_at	ABHD2	1,440118716	1,178571695	0,261547021
220335_x_at	CES3	1,88491011	1,623485524	0,261424587
204183_s_at	ADRBK2	1,88491011	1,623485524	0,261424587
204203_at	CEBPG	3,152091004	2,890758424	0,26133258
202853_s_at	RYK	2,621163856	2,359892448	0,261271408
231840_x_at	LYRM7	2,961244348	2,700070732	0,261173616
235651_at	TTC22	0,58788177	0,326777209	0,261104561
1563842_at	PIGG	0,58788177	0,326777209	0,261104561
232237_at	MDGA1	0,58788177	0,326777209	0,261104561
210839_s_at	ENPP2	0,58788177	0,326777209	0,261104561
225059_at	AGTRAP	1,047341799	0,786391549	0,26095025
200857_s_at	NCOR1	2,896717728	2,635864822	0,260852906
215535_s_at	AGPAT1	1,5944803	1,333668715	0,260811585
227678_at	XRCC6BP1	4,500752401	4,239953834	0,260798567
201699_at	PSMC6	5,605561887	5,344800421	0,260761466
214649_s_at	MTMR2	2,705643918	2,444951949	0,260691969
201244_s_at	RAF1	3,67542249	3,414783177	0,260639313
1561025_at	-	-2,774503732	-3,035090645	0,260586912
221707_s_at	VPS53	-0,551284523	-0,811769547	0,260485024
228838_at	-	-0,551284523	-0,811769547	0,260485024
206278_at	PTAFR	-0,551284523	-0,811769547	0,260485024
228126_x_at	CTXN1	-0,551284523	-0,811769547	0,260485024
230553_at	PRDM15	-0,551284523	-0,811769547	0,260485024
221368_at	NEU2	-0,551284523	-0,811769547	0,260485024
215027_at	RAPGEF3	-0,551284523	-0,811769547	0,260485024
236441_at	-	-0,551284523	-0,811769547	0,260485024
222198_at	-	-0,551284523	-0,811769547	0,260485024
232435_at	ALG13	-0,551284523	-0,811769547	0,260485024
205360_at	PFDN4	-0,551284523	-0,811769547	0,260485024
244087_at	-	-0,551284523	-0,811769547	0,260485024
230912_at	ASPDH	-0,551284523	-0,811769547	0,260485024
207152_at	NTRK2	-0,551284523	-0,811769547	0,260485024
201541_s_at	ZNHIT1	4,588489926	4,328059972	0,260429954
203093_s_at	TIMM44	2,365121417	2,104873932	0,260247485
212409_s_at	TOR1AIP1	2,392346173	2,132145748	0,260200425
214263_x_at	POLR2C	4,041098071	3,781121106	0,259976964
218830_at	RPL26L1	5,312271844	5,052393453	0,259878391
210630_s_at	RAD52	-0,437449947	-0,697182596	0,259732649

1553346_a_at	TNRC6A	-0,437449947	-0,697182596	0,259732649
227057_at	ARHGAP27	-0,437449947	-0,697182596	0,259732649
226469_s_at	GGT7	-0,437449947	-0,697182596	0,259732649
225408_at	MBP	-0,437449947	-0,697182596	0,259732649
221778_at	JHDM1D	-0,437449947	-0,697182596	0,259732649
209856_x_at	ABI2	1,182925501	0,923203038	0,259722463
209117_at	WBP2	1,640193838	1,380552339	0,259641499
201593_s_at	ZC3H15	5,005833942	4,746319618	0,259514324
200031_s_at	RPS11	8,526634565	8,26712982	0,259504746
225880_at	TOR1AIP2	2,50022283	2,24081821	0,25940462
203190_at	MIR4691 /// NDUF	5,10188718	4,842575423	0,259311757
238552_at	-	-1,212652659	-1,471833228	0,259180568
207295_at	SCNN1G	-1,212652659	-1,471833228	0,259180568
236872_at	RBM22	-1,212652659	-1,471833228	0,259180568
237082_at	-	-1,212652659	-1,471833228	0,259180568
206971_at	GPR161	-1,212652659	-1,471833228	0,259180568
240964_at	-	-1,212652659	-1,471833228	0,259180568
220365_at	ALLC	-1,212652659	-1,471833228	0,259180568
1561910_at	-	-1,212652659	-1,471833228	0,259180568
224118_at	UBE2B	-1,212652659	-1,471833228	0,259180568
240912_x_at	LINC00277	-1,212652659	-1,471833228	0,259180568
238987_at	B4GALT1	-1,212652659	-1,471833228	0,259180568
216581_at	-	-1,212652659	-1,471833228	0,259180568
201265_at	-	-1,212652659	-1,471833228	0,259180568
221313_at	GPR52	-1,212652659	-1,471833228	0,259180568
207848_at	AVP	-1,212652659	-1,471833228	0,259180568
232456_at	C10orf71	-1,212652659	-1,471833228	0,259180568
240357_at	-	-1,212652659	-1,471833228	0,259180568
228919_at	-	-1,212652659	-1,471833228	0,259180568
228013_at	PPP2R2A	1,995527416	1,736450458	0,259076958
201652_at	COPS5	4,913150425	4,654386349	0,258764076
1553701_a_at	DUSP18	0,791908897	0,533258442	0,258650456
225505_s_at	PCED1A	0,791908897	0,533258442	0,258650456
224504_s_at	BUD13	2,436165655	2,177710147	0,258455508
214896_at	-	0,08619576	-0,172194225	0,258389984
1563577_at	-	0,458905032	0,20062106	0,258283972
203196_at	ABCC4	3,209439373	2,951484533	0,25795484
212707_s_at	RASA4 /// RASA4B	1,000459215	0,742671819	0,257787396
223565_at	MZB1	1,000459215	0,742671819	0,257787396
216218_s_at	PLCL2	1,610353504	1,352622437	0,257731067
244842_x_at	-	1,610353504	1,352622437	0,257731067
212791_at	C1orf216	3,248151517	2,990421269	0,257730248
241541_at	MIB2	0,319287178	0,061773582	0,257513596
228860_at	UBE3B	0,167727503	-0,089635713	0,257363216
229145_at	ANAPC16	3,861345795	3,604010628	0,257335167
212273_x_at	GNAS	7,028520378	6,771284856	0,257235522
223712_at	PCBD2	1,788922638	1,531877749	0,257044889
203830_at	C17orf75	2,983826368	2,726850891	0,256975477
1569591_at	F11	-2,495152701	-2,752097204	0,256944503
234702_x_at	CFTR	-2,495152701	-2,752097204	0,256944503

229057_at	SCN2A	-2,495152701	-2,752097204	0,256944503
225571_at	LIFR	-2,495152701	-2,752097204	0,256944503
241770_x_at	-	-2,495152701	-2,752097204	0,256944503
236793_at	-	-2,495152701	-2,752097204	0,256944503
1568286_at	HMGA2	-2,495152701	-2,752097204	0,256944503
1556583_a_at	SLC8A1	-2,495152701	-2,752097204	0,256944503
243274_x_at	-	-2,495152701	-2,752097204	0,256944503
1569375_at	-	-2,495152701	-2,752097204	0,256944503
243799_x_at	ANGPTL3	-2,495152701	-2,752097204	0,256944503
1557883_a_at	-	-2,495152701	-2,752097204	0,256944503
1561663_at	-	-2,495152701	-2,752097204	0,256944503
1565819_at	-	-2,495152701	-2,752097204	0,256944503
240688_at	MGST1	-2,495152701	-2,752097204	0,256944503
233035_at	-	-2,495152701	-2,752097204	0,256944503
233944_at	-	-2,495152701	-2,752097204	0,256944503
225162_at	SH3D19	-2,495152701	-2,752097204	0,256944503
212802_s_at	GAPVD1	3,03433073	2,777528602	0,256802127
225564_at	SPATA13	3,269673646	3,012928641	0,256745004
210243_s_at	B4GALT3	2,468978138	2,212304392	0,256673746
1566690_at	-	-0,332405896	-0,589001171	0,256595275
209138_x_at	IGLC1	-0,332405896	-0,589001171	0,256595275
233854_x_at	KLK4	-0,332405896	-0,589001171	0,256595275
207776_s_at	CACNB2	-0,332405896	-0,589001171	0,256595275
1563485_at	-	-0,332405896	-0,589001171	0,256595275
235514_at	ASPRV1	-0,332405896	-0,589001171	0,256595275
206944_at	HTR6	-0,332405896	-0,589001171	0,256595275
207104_x_at	LILRB1	-0,332405896	-0,589001171	0,256595275
243383_at	-	-0,332405896	-0,589001171	0,256595275
222164_at	FGFR1	1,402495885	1,146010634	0,256485251
204460_s_at	RAD1	3,233229368	2,97688045	0,256348918
203018_s_at	SSX2IP	1,20396005	0,947673886	0,256286164
214836_x_at	IGK@ /// IGKC ///	3,204847647	2,948635566	0,256212081
236518_at	KIAA1984	0,244681185	-0,011001236	0,255682421
1555819_s_at	SAMD14	0,244681185	-0,011001236	0,255682421
236777_at	LOC100129195	0,244681185	-0,011001236	0,255682421
220685_at	FAM120C	0,244681185	-0,011001236	0,255682421
225811_at	C11orf58	3,313347235	3,05768517	0,255662064
212260_at	GIGYF2	1,287793882	1,032267596	0,255526286
204195_s_at	PKNOX1	1,287793882	1,032267596	0,255526286
222958_s_at	DEPDC1	3,931608669	3,676186811	0,255421858
242289_at	-	1,936380191	1,680963921	0,25541627
204603_at	EXO1	3,570321555	3,315052527	0,255269029
212281_s_at	TMEM97	4,648252012	4,393002019	0,255249993
223490_s_at	EXOSC3	2,817838865	2,562621495	0,25521737
221597_s_at	TMEM208	3,8018113	3,546728932	0,255082368
203709_at	PHKG2	1,561816652	1,306903969	0,254912683
202611_s_at	MED14	2,427650693	2,173028091	0,254622602
228959_at	PDK3	4,062761482	3,808369874	0,254391608
200717_x_at	RPL7	8,594205005	8,339935158	0,254269847
222732_at	TRIM39 /// TRIM3!	1,671397663	1,417157926	0,254239736

218149_s_at	ZNF395	1,88491011	1,630734622	0,254175489
1556063_s_at	RPP30	-0,230432956	-0,484558493	0,254125538
243749_s_at	-	-0,230432956	-0,484558493	0,254125538
1562496_at	LOC339539	-0,230432956	-0,484558493	0,254125538
232976_at	SNORD116-17 /// !	-0,230432956	-0,484558493	0,254125538
208301_at	-	-0,230432956	-0,484558493	0,254125538
203463_s_at	EPN2	-0,230432956	-0,484558493	0,254125538
234466_at	FAM20C	-0,230432956	-0,484558493	0,254125538
228006_at	PTEN	-0,230432956	-0,484558493	0,254125538
203490_at	ELF4	1,948628481	1,694547353	0,254081127
225334_at	C10orf32	1,948628481	1,694547353	0,254081127
201780_s_at	RNF13	3,261582786	3,007560601	0,254022185
236027_at	SFR1	1,225123479	0,971607464	0,253516015
222017_x_at	LRCH4	1,421391637	1,167919222	0,253472415
221607_x_at	ACTG1	8,456851089	8,203789079	0,25306201
1553956_at	TMEM237	2,199941239	1,946903052	0,253038187
217945_at	BTBD1	4,722768554	4,469757829	0,253010725
219016_at	FASTKD5	2,989672611	2,736663738	0,253008873
208648_at	VCP	2,914752575	2,662083808	0,252668768
227983_at	RILPL2	4,949910672	4,69741204	0,252498632
208598_s_at	HUWE1	4,033407967	3,781121106	0,25228686
236097_at	-	0,618277321	0,366031214	0,252246108
1558041_a_at	KIAA0895L	1,760255626	1,508107415	0,25214821
231277_x_at	DTWD2	0,820160788	0,568155442	0,252005346
204838_s_at	MLH3	1,510970097	1,259081178	0,251888919
235174_s_at	LOC100128822	3,340803206	3,08892491	0,251878297
205705_at	ANKRD26	-0,135065865	-0,386896102	0,251830237
234611_at	LGALS8-AS1	-0,135065865	-0,386896102	0,251830237
239375_at	-	-0,135065865	-0,386896102	0,251830237
223064_at	RNF181	5,444742986	5,192917431	0,251825555
227549_x_at	ZDHHC24	1,960819106	1,709047448	0,251771659
205345_at	BARD1	3,029186714	2,777528602	0,251658112
238402_s_at	ENDOV /// LOC10(	1,024284941	0,772793615	0,251491327
204193_at	CHKB	2,312738346	2,06133574	0,251402606
208616_s_at	PTP4A2	6,852734143	6,601528408	0,251205734
229268_at	FAM105B	1,910557155	1,659532492	0,251024663
224611_s_at	DNAJC5	2,267141818	2,016139502	0,251002316
203752_s_at	JUND	4,829437568	4,578636564	0,250801003
1555365_x_at	C11orf58	-2,250580859	-2,501359113	0,250778254
235885_at	P2RY12	-2,250580859	-2,501359113	0,250778254
223823_at	KCNMB2	-2,250580859	-2,501359113	0,250778254
235267_at	MAGI2-AS3	-2,250580859	-2,501359113	0,250778254
216097_at	-	-2,250580859	-2,501359113	0,250778254
204913_s_at	SOX11	-2,250580859	-2,501359113	0,250778254
1562800_at	-	-2,250580859	-2,501359113	0,250778254
1560940_at	SACS-AS1	-2,250580859	-2,501359113	0,250778254
1561154_at	-	-2,250580859	-2,501359113	0,250778254
228949_at	WLS	-2,250580859	-2,501359113	0,250778254
1558999_x_at	LOC283922 /// PD	-2,250580859	-2,501359113	0,250778254
206644_at	NR0B1	-2,250580859	-2,501359113	0,250778254

1552557_a_at	ZDHHC15	-2,250580859	-2,501359113	0,250778254
221182_at	C1orf129	-2,250580859	-2,501359113	0,250778254
1553924_at	FMO9P	-2,250580859	-2,501359113	0,250778254
243243_at	GPC3	-2,250580859	-2,501359113	0,250778254
1561864_at	-	-2,250580859	-2,501359113	0,250778254
217372_at	-	-2,250580859	-2,501359113	0,250778254
1552482_at	RAPH1	-2,250580859	-2,501359113	0,250778254
230867_at	COL6A6	-2,250580859	-2,501359113	0,250778254
1562453_at	-	-2,250580859	-2,501359113	0,250778254
235666_at	ITGA8	-2,250580859	-2,501359113	0,250778254
243701_at	-	-2,250580859	-2,501359113	0,250778254
235050_at	SLC2A12	-2,250580859	-2,501359113	0,250778254
236495_at	-	-2,250580859	-2,501359113	0,250778254
1562670_at	-	-2,250580859	-2,501359113	0,250778254
217056_at	YME1L1	-2,250580859	-2,501359113	0,250778254
207299_s_at	GRM1	-2,250580859	-2,501359113	0,250778254
236316_at	FAM3C	-2,250580859	-2,501359113	0,250778254
211625_s_at	DRD3	-2,250580859	-2,501359113	0,250778254
237890_at	-	-2,250580859	-2,501359113	0,250778254
201177_s_at	UBA2	5,639636349	5,388902314	0,250734035
203520_s_at	ZNF318	1,160996507	0,910483921	0,250512587
1554167_a_at	GOLGA7	4,616350364	4,366031382	0,250318982
231959_at	LIN52	2,452674045	2,20251271	0,250161336
218494_s_at	SLC2A4RG	1,640193838	1,390070295	0,250123543
202507_s_at	SNAP25	0,556428218	0,306454867	0,249973351
222728_s_at	MIR1304 /// SNOR	5,535113886	5,285468634	0,249645252
225699_at	SNHG15	5,046791742	4,797205789	0,249585954
225539_at	ZNF295	2,824160383	2,574574485	0,249585898
202513_s_at	PPP2R5D	2,401508182	2,151985997	0,249522185
217888_s_at	ARFGAP1	1,528073958	1,278654284	0,249419675
213387_at	ATAD2B	2,784898907	2,535878988	0,24901992
212302_at	RTF1	1,857989965	1,60918327	0,248806695
202386_s_at	KIAA0430	3,071179046	2,822379032	0,248800014
213458_at	FAM149B1	-1,893506789	-2,142286822	0,248780033
1566882_at	-	-1,893506789	-2,142286822	0,248780033
216782_at	-	-1,893506789	-2,142286822	0,248780033
243494_at	LOC100506926	-1,893506789	-2,142286822	0,248780033
208231_at	NRG1	-1,893506789	-2,142286822	0,248780033
230797_s_at	LOC440900	-1,893506789	-2,142286822	0,248780033
1570252_at	-	-1,893506789	-2,142286822	0,248780033
1564491_at	SPANXA2-OT1	-1,893506789	-2,142286822	0,248780033
237462_at	-	-1,893506789	-2,142286822	0,248780033
1562069_at	-	-1,893506789	-2,142286822	0,248780033
1556406_at	LOC255025	-1,893506789	-2,142286822	0,248780033
224086_at	-	-1,893506789	-2,142286822	0,248780033
240448_at	SOGA2	-1,893506789	-2,142286822	0,248780033
1568888_at	LOC100507283	-1,893506789	-2,142286822	0,248780033
219909_at	MMP28	-1,893506789	-2,142286822	0,248780033
220259_at	PLEKHH3	-1,893506789	-2,142286822	0,248780033
1552713_a_at	SLC4A1	-1,893506789	-2,142286822	0,248780033

1552508_at	KCNE4	-1,893506789	-2,142286822	0,248780033
242819_at	-	-1,893506789	-2,142286822	0,248780033
237047_at	LINC00281	-1,893506789	-2,142286822	0,248780033
208025_s_at	HMGA2	-1,893506789	-2,142286822	0,248780033
1553636_at	TMCO5A	-1,893506789	-2,142286822	0,248780033
242532_at	-	-1,893506789	-2,142286822	0,248780033
239118_at	KCNA2	-1,893506789	-2,142286822	0,248780033
205444_at	ATP2A1	-1,893506789	-2,142286822	0,248780033
243716_at	FLJ43663	-1,893506789	-2,142286822	0,248780033
209925_at	OCLN	-1,893506789	-2,142286822	0,248780033
243102_at	-	-1,893506789	-2,142286822	0,248780033
237930_at	-	-1,893506789	-2,142286822	0,248780033
228353_x_at	UBASH3B	-1,893506789	-2,142286822	0,248780033
212154_at	SDC2	-1,893506789	-2,142286822	0,248780033
1562827_at	DIO2-AS1	-1,893506789	-2,142286822	0,248780033
232973_at	DOCK1	-1,893506789	-2,142286822	0,248780033
202033_s_at	RB1CC1	3,559161816	3,31062689	0,248534926
1553362_at	DNAH6	-2,416625063	-2,665156778	0,248531715
241796_x_at	-	-2,416625063	-2,665156778	0,248531715
210919_at	PHLPP1	-2,416625063	-2,665156778	0,248531715
220799_at	GCM2	-2,416625063	-2,665156778	0,248531715
241120_s_at	CDC20B	-2,416625063	-2,665156778	0,248531715
1560676_at	SIAH3	-2,416625063	-2,665156778	0,248531715
1555018_at	OR2C3	-2,416625063	-2,665156778	0,248531715
206145_at	RHAG	-2,416625063	-2,665156778	0,248531715
238584_at	IQCA1	-2,416625063	-2,665156778	0,248531715
228141_at	GPX8	-2,416625063	-2,665156778	0,248531715
237290_at	-	-2,416625063	-2,665156778	0,248531715
219850_s_at	EHF	-2,416625063	-2,665156778	0,248531715
233046_at	-	-2,416625063	-2,665156778	0,248531715
216469_at	LOC441666	-2,416625063	-2,665156778	0,248531715
244545_at	LOC100652770	-2,416625063	-2,665156778	0,248531715
1569610_at	IQCH	-2,416625063	-2,665156778	0,248531715
1557914_s_at	ERVFH21-1	-2,416625063	-2,665156778	0,248531715
1552754_a_at	CADM2	-2,416625063	-2,665156778	0,248531715
204235_s_at	GULP1	-2,416625063	-2,665156778	0,248531715
36019_at	STK19	1,978212065	1,729850735	0,248361329
223135_s_at	BBX	2,159837409	1,911502437	0,248334972
1554001_at	TRIM37	1,458255986	1,209973561	0,248282425
222538_s_at	APPL1	3,493994216	3,246064373	0,247929844
211317_s_at	CFLAR	2,692288672	2,444951949	0,247336722
1563945_at	LOC284100	0,045517965	-0,201789721	0,247307686
215654_at	BCAT2	0,045517965	-0,201789721	0,247307686
235966_at	DAB2IP	0,045517965	-0,201789721	0,247307686
212144_at	SUN2	3,18076822	2,933477664	0,247290556
220762_s_at	GNB1L	1,730929079	1,483698568	0,247230511
217250_s_at	CHD5	-1,369772723	-1,616924751	0,247152028
233533_at	KRTAP1-5	-1,369772723	-1,616924751	0,247152028
219091_s_at	MMRN2	-1,369772723	-1,616924751	0,247152028
1553435_at	C18orf15	-1,369772723	-1,616924751	0,247152028

240569_at	-	-1,369772723	-1,616924751	0,247152028
1565738_at	SCTR	-1,369772723	-1,616924751	0,247152028
235392_at	-	-1,369772723	-1,616924751	0,247152028
214031_s_at	KRT7	-1,369772723	-1,616924751	0,247152028
231256_at	LOC727944	-1,369772723	-1,616924751	0,247152028
241313_at	DOCK4	-1,369772723	-1,616924751	0,247152028
235715_at	SPRNP1	-1,369772723	-1,616924751	0,247152028
232722_at	RNASET2	-1,369772723	-1,616924751	0,247152028
207542_s_at	AQP1	-1,369772723	-1,616924751	0,247152028
222174_at	-	-1,369772723	-1,616924751	0,247152028
230090_at	GDNF	-1,369772723	-1,616924751	0,247152028
229698_at	-	-1,369772723	-1,616924751	0,247152028
220987_s_at	AKIP1 /// NUAK2	-1,369772723	-1,616924751	0,247152028
1565671_a_at	-	-1,369772723	-1,616924751	0,247152028
221418_s_at	MED16	0,847529938	0,600385088	0,24714485
213264_at	PCBP2	2,492739983	2,245685006	0,247054976
220380_at	DNASE2B	-1,802716385	-2,049747196	0,247030811
221394_at	TAAR2	-1,802716385	-2,049747196	0,247030811
242641_at	COL6A6	-1,802716385	-2,049747196	0,247030811
1566638_at	-	-1,802716385	-2,049747196	0,247030811
233213_at	-	-1,802716385	-2,049747196	0,247030811
234507_at	-	-1,802716385	-2,049747196	0,247030811
242166_at	-	-1,802716385	-2,049747196	0,247030811
242001_at	-	-1,802716385	-2,049747196	0,247030811
236005_at	-	-1,802716385	-2,049747196	0,247030811
220737_at	RPS6KA6	-1,802716385	-2,049747196	0,247030811
231916_at	NOS1	-1,802716385	-2,049747196	0,247030811
241420_at	-	-1,802716385	-2,049747196	0,247030811
235506_at	-	-1,802716385	-2,049747196	0,247030811
1557434_at	LOC100506497	-1,802716385	-2,049747196	0,247030811
221026_s_at	SCRT1	-1,802716385	-2,049747196	0,247030811
1555505_a_at	TYR	-1,802716385	-2,049747196	0,247030811
232695_at	KIF6	-1,802716385	-2,049747196	0,247030811
241897_at	-	-1,802716385	-2,049747196	0,247030811
243645_at	NFASC	-1,802716385	-2,049747196	0,247030811
205627_at	CDA	-1,802716385	-2,049747196	0,247030811
217639_at	-	-1,802716385	-2,049747196	0,247030811
207361_at	HBP1	-1,802716385	-2,049747196	0,247030811
215837_x_at	-	-1,802716385	-2,049747196	0,247030811
233650_at	CEP63	-1,802716385	-2,049747196	0,247030811
243497_at	-	-1,802716385	-2,049747196	0,247030811
234756_at	CACNG8	-1,802716385	-2,049747196	0,247030811
211104_s_at	MYO7A	-1,802716385	-2,049747196	0,247030811
208514_at	KCNE1	-1,802716385	-2,049747196	0,247030811
1559537_at	RNF130	-1,802716385	-2,049747196	0,247030811
229976_at	MORN5	-1,802716385	-2,049747196	0,247030811
1556639_at	LOC439914	-1,802716385	-2,049747196	0,247030811
211072_x_at	LOC100288366 ///	8,081326667	7,834303702	0,247022965
229461_x_at	NEGR1	-1,982324124	-2,229233437	0,246909314
214984_at	LOC100271836 ///	-1,982324124	-2,229233437	0,246909314

215851_at	MECOM	-1,982324124	-2,229233437	0,246909314
210004_at	OLR1	-1,982324124	-2,229233437	0,246909314
216805_at	-	-1,982324124	-2,229233437	0,246909314
1553513_at	VNN3	-1,982324124	-2,229233437	0,246909314
236811_at	DMRTC2	-1,982324124	-2,229233437	0,246909314
1554908_at	HYDIN	-1,982324124	-2,229233437	0,246909314
1569492_at	-	-1,982324124	-2,229233437	0,246909314
1558968_at	-	-1,982324124	-2,229233437	0,246909314
220360_at	THAP9	-1,982324124	-2,229233437	0,246909314
1560038_at	LOC100506071	-1,982324124	-2,229233437	0,246909314
1552449_a_at	SCGB1C1	-1,982324124	-2,229233437	0,246909314
233594_at	LOC100506025	-1,982324124	-2,229233437	0,246909314
1557345_at	LOC283516	-1,982324124	-2,229233437	0,246909314
215187_at	FLJ11292	-1,982324124	-2,229233437	0,246909314
1568924_a_at	IQUB	-1,982324124	-2,229233437	0,246909314
235982_at	FCRL1	-1,982324124	-2,229233437	0,246909314
201348_at	GPX3	-1,982324124	-2,229233437	0,246909314
216657_at	ATXN3	-1,982324124	-2,229233437	0,246909314
234589_at	TMEM106A	-1,982324124	-2,229233437	0,246909314
242849_at	-	-1,982324124	-2,229233437	0,246909314
206532_at	-	-1,982324124	-2,229233437	0,246909314
1557548_at	C10orf108	-1,982324124	-2,229233437	0,246909314
1559617_at	LOC100505565	-1,982324124	-2,229233437	0,246909314
239595_at	GPX2	-1,982324124	-2,229233437	0,246909314
1560960_at	-	-1,982324124	-2,229233437	0,246909314
244138_at	-	-1,982324124	-2,229233437	0,246909314
203698_s_at	FRZB	-1,982324124	-2,229233437	0,246909314
1552526_at	FAM71C	-1,982324124	-2,229233437	0,246909314
239634_at	-	-1,982324124	-2,229233437	0,246909314
1561091_at	-	-1,982324124	-2,229233437	0,246909314
219427_at	FAT4	-1,982324124	-2,229233437	0,246909314
216799_at	-	-1,982324124	-2,229233437	0,246909314
241553_at	GSG1	-1,982324124	-2,229233437	0,246909314
206650_at	IQCC	0,425036312	0,178191865	0,246844447
242738_s_at	ZFHX3	0,425036312	0,178191865	0,246844447
219072_at	BCL7C	2,127965541	1,881145546	0,246819995
1568683_at	MGC23284	-0,043925712	-0,290694975	0,246769263
217559_at	RPL10L	-0,043925712	-0,290694975	0,246769263
1564112_at	FAM71A	-0,043925712	-0,290694975	0,246769263
235673_at	-	1,182925501	0,936232181	0,24669332
227922_x_at	LOC441124	1,267146969	1,020514937	0,246632033
217779_s_at	PNRC2	5,822968482	5,576607767	0,246360715
230678_at	RGS5	0,355751005	0,109458907	0,246292098
225613_at	MAST4	0,707657549	0,461385738	0,246271811
204295_at	SURF1	5,161840645	4,915922766	0,245917879
219306_at	KIF15	3,493994216	3,248126831	0,245867386
225006_x_at	TH1L	4,496331177	4,250487736	0,245843441
211970_x_at	ACTG1	8,30285477	8,05703949	0,24581528
235918_x_at	CEP97	1,745502609	1,500059916	0,245442692
224959_at	SLC26A2	1,995527416	1,750483134	0,245044282

1562162_at	-	-0,856259172	-1,100943374	0,244684202
1558841_at	CWC27	-0,856259172	-1,100943374	0,244684202
1559755_at	C21orf49	-0,856259172	-1,100943374	0,244684202
224246_at	-	-0,856259172	-1,100943374	0,244684202
215974_at	-	-0,856259172	-1,100943374	0,244684202
210963_s_at	GYG2	-0,856259172	-1,100943374	0,244684202
228631_s_at	ZNF688	-0,856259172	-1,100943374	0,244684202
1559002_at	LOC340544	-0,856259172	-1,100943374	0,244684202
243408_at	-	-0,856259172	-1,100943374	0,244684202
211377_x_at	MYCN	-0,856259172	-1,100943374	0,244684202
1554940_a_at	LOC388882	-0,856259172	-1,100943374	0,244684202
206396_at	SLC1A1	-0,856259172	-1,100943374	0,244684202
42361_g_at	CCHCR1	1,384741891	1,140219523	0,244522368
235248_at	BTBD9	0,282358733	0,03784737	0,244511363
206130_s_at	ASGR2	0,127732831	-0,116767475	0,244500305
232949_at	-	0,127732831	-0,116767475	0,244500305
1556015_a_at	MESP2	0,926325262	0,681874787	0,244450475
226463_at	ATP6V1C1	1,830468611	1,586121802	0,24434681
201658_at	ARL1	3,296548503	3,052211048	0,244337455
221743_at	CELF1	5,159334259	4,91512695	0,244207309
201334_s_at	ARHGEF12	3,366614157	3,122419381	0,244194776
209040_s_at	PSMB8	5,610806763	5,366734111	0,244072652
206997_s_at	HS6ST1	0,20740896	-0,036543687	0,243952647
228520_s_at	APLP2	0,20740896	-0,036543687	0,243952647
240539_at	-	-1,712732543	-1,956681069	0,243948527
209651_at	TGFB1I1	-1,712732543	-1,956681069	0,243948527
224295_at	-	-1,712732543	-1,956681069	0,243948527
225871_at	STEAP2	-1,712732543	-1,956681069	0,243948527
243265_at	GAD2	-1,712732543	-1,956681069	0,243948527
1569917_at	-	-1,712732543	-1,956681069	0,243948527
242220_at	SPTBN1	-1,712732543	-1,956681069	0,243948527
215922_at	REPS1	-1,712732543	-1,956681069	0,243948527
1562585_at	LOC284263	-1,712732543	-1,956681069	0,243948527
1562629_a_at	KRT40	-1,712732543	-1,956681069	0,243948527
1562475_at	DKFZp686O1327 /,	-1,712732543	-1,956681069	0,243948527
1566981_at	-	-1,712732543	-1,956681069	0,243948527
244372_at	-	-1,712732543	-1,956681069	0,243948527
220487_at	SNTG2	-1,712732543	-1,956681069	0,243948527
243167_at	ABCB5	-1,712732543	-1,956681069	0,243948527
233253_at	-	-1,712732543	-1,956681069	0,243948527
233075_at	HERC2P7	-1,712732543	-1,956681069	0,243948527
1564168_a_at	LOC93444	-1,712732543	-1,956681069	0,243948527
1569219_at	-	-1,712732543	-1,956681069	0,243948527
1565778_at	ABCA8	-1,712732543	-1,956681069	0,243948527
244688_at	-	-1,712732543	-1,956681069	0,243948527
227522_at	CMBL	-1,712732543	-1,956681069	0,243948527
1561128_at	-	-1,712732543	-1,956681069	0,243948527
231535_x_at	ROPN1	-1,712732543	-1,956681069	0,243948527
206934_at	SIRPB1	-1,712732543	-1,956681069	0,243948527
221448_s_at	TEX15	-1,712732543	-1,956681069	0,243948527

230014_at	-	-1,712732543	-1,956681069	0,243948527
1564004_at	-	-1,712732543	-1,956681069	0,243948527
233370_at	-	-1,712732543	-1,956681069	0,243948527
242671_at	-	-1,712732543	-1,956681069	0,243948527
226769_at	FIBIN	-1,712732543	-1,956681069	0,243948527
241931_at	XG /// XGPY2	-1,712732543	-1,956681069	0,243948527
230584_at	LOC100287728 ///	-1,712732543	-1,956681069	0,243948527
1569802_at	-	-1,712732543	-1,956681069	0,243948527
205916_at	S100A7	-1,712732543	-1,956681069	0,243948527
231692_at	PIGG	-1,712732543	-1,956681069	0,243948527
1555014_x_at	-	-1,712732543	-1,956681069	0,243948527
1570372_at	-	-1,712732543	-1,956681069	0,243948527
207110_at	KCNJ12 /// LOC10(	-1,712732543	-1,956681069	0,243948527
215406_at	-	-1,712732543	-1,956681069	0,243948527
230217_at	CLVS1	-1,712732543	-1,956681069	0,243948527
1562844_at	LOC339822	-1,712732543	-1,956681069	0,243948527
242825_at	LPPR5	-1,712732543	-1,956681069	0,243948527
235968_at	AGAP1	-1,712732543	-1,956681069	0,243948527
239517_at	ITGB6	-1,712732543	-1,956681069	0,243948527
1556654_at	CDK12	-1,712732543	-1,956681069	0,243948527
236348_at	TMEM176B	-1,712732543	-1,956681069	0,243948527
204105_s_at	NRCAM	-1,712732543	-1,956681069	0,243948527
208766_s_at	HNRNPR	6,173273036	5,929700757	0,243572278
228465_at	-	1,775035811	1,531877749	0,243158062
213738_s_at	ATP5A1	7,543418955	7,300361587	0,243057368
212581_x_at	GAPDH	8,664540251	8,421511261	0,243028991
234476_at	DNAH7	-0,730013898	-0,972893339	0,24287944
215551_at	ESR1	-0,730013898	-0,972893339	0,24287944
1552942_at	LOC149373	-0,730013898	-0,972893339	0,24287944
1562950_at	-	-0,730013898	-0,972893339	0,24287944
229121_at	CMKLR1	-0,730013898	-0,972893339	0,24287944
227553_at	PIK3R5	-0,730013898	-0,972893339	0,24287944
239204_at	ZNF75A	-0,730013898	-0,972893339	0,24287944
244280_at	LOC100507254	-0,730013898	-0,972893339	0,24287944
218345_at	TMEM176A	-0,730013898	-0,972893339	0,24287944
240887_at	LOC100506470	-0,730013898	-0,972893339	0,24287944
238716_at	LOC100506990	-0,730013898	-0,972893339	0,24287944
1559126_at	RRP12	-0,730013898	-0,972893339	0,24287944
216217_at	PLCL2	-0,730013898	-0,972893339	0,24287944
225686_at	SKA2	3,668220666	3,425365487	0,242855179
207769_s_at	PQBP1	3,912706782	3,669940122	0,24276666
203356_at	CAPN7	2,932161212	2,689594859	0,242566354
239711_at	ADAL	0,648195588	0,405752839	0,242442749
225676_s_at	DCAF13	4,898999808	4,6568995	0,242100309
243880_at	GOSR2	0,791908897	0,550017041	0,241891857
207812_s_at	GORASP2	4,087244545	3,84538632	0,241858225
205484_at	SIT1	2,560606634	2,31904917	0,241557465
1559728_at	ZBTB40	-0,49284695	-0,734355396	0,241508446
215261_at	-	-0,49284695	-0,734355396	0,241508446
232604_at	ZNF541	-0,49284695	-0,734355396	0,241508446

203134_at	PICALM	-0,49284695	-0,734355396	0,241508446
235437_at	-	-0,49284695	-0,734355396	0,241508446
1556292_s_at	POM121L12	-0,49284695	-0,734355396	0,241508446
235983_at	-	-0,49284695	-0,734355396	0,241508446
225229_at	AFF4	2,51558601	2,274084398	0,241501612
229650_s_at	C19orf42	2,537731447	2,296310714	0,241420733
1555961_a_at	HINT1	7,634600059	7,393337744	0,241262315
208845_at	VDAC3	6,741906764	6,500806896	0,241099868
222976_s_at	TPM3	6,864321136	6,623336571	0,240984564
202789_at	PLCG1	3,122148189	2,881480309	0,240667881
238956_at	EFNA2	-0,994186487	-1,234835326	0,240648839
208140_s_at	LRRC48	-0,994186487	-1,234835326	0,240648839
243624_at	PIAS2	-0,994186487	-1,234835326	0,240648839
243397_at	-	-0,994186487	-1,234835326	0,240648839
206938_at	SRD5A2	-0,994186487	-1,234835326	0,240648839
239606_at	-	-0,994186487	-1,234835326	0,240648839
242222_at	LOC151009 /// LO(	-0,994186487	-1,234835326	0,240648839
205721_at	GFRA2	-0,994186487	-1,234835326	0,240648839
1563863_x_at	TCEANC	-0,994186487	-1,234835326	0,240648839
1560942_at	PGD	-0,994186487	-1,234835326	0,240648839
206387_at	CDX2	-0,994186487	-1,234835326	0,240648839
1570282_at	-	-0,994186487	-1,234835326	0,240648839
227397_at	TPM2	-0,994186487	-1,234835326	0,240648839
205348_s_at	DYNC1I1	-0,994186487	-1,234835326	0,240648839
229052_at	ANKRD23 /// ANKI	-0,994186487	-1,234835326	0,240648839
204583_x_at	KLK3	-0,994186487	-1,234835326	0,240648839
1555016_at	IL16	-0,994186487	-1,234835326	0,240648839
217414_x_at	HBA1 /// HBA2	-0,994186487	-1,234835326	0,240648839
212426_s_at	YWHAQ	7,521818436	7,281197992	0,240620444
233079_at	-	-0,609040214	-0,84928999	0,240249776
235809_at	LIN54	-0,609040214	-0,84928999	0,240249776
232407_at	MUC17	-0,609040214	-0,84928999	0,240249776
208278_s_at	-	-0,609040214	-0,84928999	0,240249776
222799_at	WDR91	-0,609040214	-0,84928999	0,240249776
1553586_at	FBXL19-AS1	-0,609040214	-0,84928999	0,240249776
227250_at	KREMEN1	-0,609040214	-0,84928999	0,240249776
227380_x_at	C16orf13	-0,609040214	-0,84928999	0,240249776
227186_s_at	MRPL41	4,179494373	3,939331359	0,240163014
200071_at	SMNDC1	4,049824219	3,809801648	0,240022571
223042_s_at	FUNDC2	4,28595496	4,046112122	0,239842839
206526_at	RIBC2	1,307201325	1,067365565	0,23983576
218393_s_at	SMU1	2,209870035	1,970106792	0,239763243
232221_x_at	RSBN1L	2,209870035	1,970106792	0,239763243
222518_at	ARFGEF2	2,545254728	2,305621062	0,239633666
219242_at	CEP63	3,096793374	2,857256661	0,239536713
218130_at	C17orf62	3,305120269	3,065641037	0,239479233
210069_at	CHKB-CPT1B /// CI	1,730929079	1,491644074	0,239285005
232225_at	-	-0,384193355	-0,623254098	0,239060744
231116_at	-	-0,384193355	-0,623254098	0,239060744
218558_s_at	MRPL39	4,916700014	4,677643598	0,239056417

203090_at	SDF2	2,692288672	2,45323331	0,239055361
1557180_at	C11orf87	-2,641232718	-2,880284116	0,239051398
242955_x_at	-	-2,641232718	-2,880284116	0,239051398
200896_x_at	HDGF	5,109492158	4,871099544	0,238392614
204676_at	TMEM186	2,552836808	2,314488991	0,238347818
228753_at	LOC100128737	1,246401134	1,008210238	0,238190896
236433_at	LOC100506713 ///	-0,280273599	-0,518232988	0,237959389
232522_at	-	-0,280273599	-0,518232988	0,237959389
215860_at	SYT12	-0,280273599	-0,518232988	0,237959389
214506_at	GPR182	-0,280273599	-0,518232988	0,237959389
237304_at	SYCE2	-0,280273599	-0,518232988	0,237959389
208542_x_at	ZNF208	-2,074223907	-2,312054429	0,237830522
239691_at	C12orf77	-2,074223907	-2,312054429	0,237830522
1564841_at	-	-2,074223907	-2,312054429	0,237830522
237700_at	-	-2,074223907	-2,312054429	0,237830522
1569407_at	-	-2,074223907	-2,312054429	0,237830522
1562613_at	-	-2,074223907	-2,312054429	0,237830522
1567249_at	OR9A1P	-2,074223907	-2,312054429	0,237830522
220170_at	FHL5	-2,074223907	-2,312054429	0,237830522
1570448_at	-	-2,074223907	-2,312054429	0,237830522
1563673_a_at	ALS2CR11	-2,074223907	-2,312054429	0,237830522
242047_at	RTKN2	-2,074223907	-2,312054429	0,237830522
240244_at	-	-2,074223907	-2,312054429	0,237830522
235079_at	ZNF704	-2,074223907	-2,312054429	0,237830522
232477_at	-	-2,074223907	-2,312054429	0,237830522
224272_at	RACGAP1P	-2,074223907	-2,312054429	0,237830522
1553468_at	HYDIN2	-2,074223907	-2,312054429	0,237830522
1553574_at	IFNE	-2,074223907	-2,312054429	0,237830522
1563581_at	RPL34-AS1	-2,074223907	-2,312054429	0,237830522
209774_x_at	CXCL2	-2,074223907	-2,312054429	0,237830522
1561540_at	LINC00343	-2,074223907	-2,312054429	0,237830522
207294_at	AGTR2	-2,074223907	-2,312054429	0,237830522
1558795_at	LOC728052	-2,074223907	-2,312054429	0,237830522
1562121_at	-	-2,074223907	-2,312054429	0,237830522
238280_at	CYB5RL	-2,074223907	-2,312054429	0,237830522
241463_at	EIF2AK3	-2,074223907	-2,312054429	0,237830522
239665_at	LOC441179	-2,074223907	-2,312054429	0,237830522
1563466_at	MYLK	-2,074223907	-2,312054429	0,237830522
1569182_at	-	-2,074223907	-2,312054429	0,237830522
1556099_at	LINC00290	-2,074223907	-2,312054429	0,237830522
229105_at	GPR39	-2,074223907	-2,312054429	0,237830522
241047_at	LINC00340	-2,074223907	-2,312054429	0,237830522
1562754_at	LOC339260	-2,074223907	-2,312054429	0,237830522
227443_at	LURAP1L	-2,074223907	-2,312054429	0,237830522
237134_at	-	-2,074223907	-2,312054429	0,237830522
1552669_at	PPP1R3B	-2,074223907	-2,312054429	0,237830522
1559029_at	-	-2,074223907	-2,312054429	0,237830522
208427_s_at	ELAVL2	-2,074223907	-2,312054429	0,237830522
208772_at	ANKHD1 /// ANKH	3,599444854	3,361641325	0,237803529
232909_s_at	BPTF /// LOC1468	3,309267982	3,071469991	0,237797991

230836_at	ST8SIA4	2,410823487	2,173028091	0,237795395
228257_at	ANKRD52	1,458255986	1,220470658	0,237785328
221711_s_at	BABAM1	3,642593015	3,405078581	0,237514434
212156_at	VPS39	1,745502609	1,508107415	0,237395193
243886_at	-	-1,624955693	-1,862269442	0,237313749
220108_at	GNA14	-1,624955693	-1,862269442	0,237313749
208378_x_at	FGF5	-1,624955693	-1,862269442	0,237313749
239229_at	PHEX	-1,624955693	-1,862269442	0,237313749
1566951_at	-	-1,624955693	-1,862269442	0,237313749
229137_at	FUCA1	-1,624955693	-1,862269442	0,237313749
219789_at	NPR3	-1,624955693	-1,862269442	0,237313749
242653_at	DCC	-1,624955693	-1,862269442	0,237313749
1564299_at	LOC100127940	-1,624955693	-1,862269442	0,237313749
1570270_at	-	-1,624955693	-1,862269442	0,237313749
237587_at	-	-1,624955693	-1,862269442	0,237313749
1564545_a_at	-	-1,624955693	-1,862269442	0,237313749
1566499_at	-	-1,624955693	-1,862269442	0,237313749
230596_at	-	-1,624955693	-1,862269442	0,237313749
216178_x_at	ITGB1	-1,624955693	-1,862269442	0,237313749
205846_at	PTPRB	-1,624955693	-1,862269442	0,237313749
243122_at	-	-1,624955693	-1,862269442	0,237313749
241499_at	ZNF621	-1,624955693	-1,862269442	0,237313749
232494_at	CYP8B1	-1,624955693	-1,862269442	0,237313749
237934_at	-	-1,624955693	-1,862269442	0,237313749
205741_s_at	DTNA	-1,624955693	-1,862269442	0,237313749
232717_at	KALRN	-1,624955693	-1,862269442	0,237313749
241131_at	-	-1,624955693	-1,862269442	0,237313749
1565358_at	RARA	-1,624955693	-1,862269442	0,237313749
1562926_at	-	-1,624955693	-1,862269442	0,237313749
244542_at	BCDIN3D-AS1	-1,624955693	-1,862269442	0,237313749
1564200_at	LINC00607	-1,624955693	-1,862269442	0,237313749
216926_s_at	MAU2	-1,624955693	-1,862269442	0,237313749
227589_at	PITPNC1	-1,624955693	-1,862269442	0,237313749
217415_at	POLR2A	-1,624955693	-1,862269442	0,237313749
238296_at	GLIPR1L1	-1,624955693	-1,862269442	0,237313749
205069_s_at	ARHGAP26	-1,624955693	-1,862269442	0,237313749
1563171_at	-	-1,624955693	-1,862269442	0,237313749
229842_at	ELF3	-1,624955693	-1,862269442	0,237313749
241171_at	-	-1,624955693	-1,862269442	0,237313749
212013_at	PXDN	-1,624955693	-1,862269442	0,237313749
235838_at	NLGN2	-1,624955693	-1,862269442	0,237313749
208401_s_at	GLP1R	-1,624955693	-1,862269442	0,237313749
1557907_x_at	MUC12	-0,180990326	-0,418248858	0,237258532
209260_at	SFN	-0,180990326	-0,418248858	0,237258532
1562223_at	LOC642426	-2,569309811	-2,8064286	0,237118788
236538_at	GRIA2	-2,569309811	-2,8064286	0,237118788
1556170_at	-	-2,569309811	-2,8064286	0,237118788
1568926_x_at	MYLK3	-2,569309811	-2,8064286	0,237118788
1562329_at	CSMD1	-2,569309811	-2,8064286	0,237118788
1557506_a_at	-	-2,569309811	-2,8064286	0,237118788

1561562_at	-	-2,569309811	-2,8064286	0,237118788
1554640_at	PALM2	-2,569309811	-2,8064286	0,237118788
226968_at	KIF1B	2,085817135	1,848871029	0,236946106
201815_s_at	TBC1D5	1,476175815	1,239268196	0,236907619
227582_at	KLHDC9	0,458905032	0,222432814	0,236472218
200899_s_at	MGEA5	3,555373205	3,319388768	0,235984437
204568_at	ATG14	1,326463531	1,090640407	0,235823124
210101_x_at	SH3GLB1	4,377039749	4,141402272	0,235637477
209178_at	DHX38	1,92317941	1,687645398	0,235534012
235986_at	-	-1,137208348	-1,372734086	0,235525737
1563621_at	-	-1,137208348	-1,372734086	0,235525737
236642_at	C5orf63	-1,137208348	-1,372734086	0,235525737
1553641_a_at	TSGA13	-1,137208348	-1,372734086	0,235525737
238569_at	GABBR1	-1,137208348	-1,372734086	0,235525737
214325_at	GP2	-1,137208348	-1,372734086	0,235525737
1563290_at	DNAH3	-1,137208348	-1,372734086	0,235525737
211030_s_at	SLC6A6	-1,137208348	-1,372734086	0,235525737
223957_at	-	-1,137208348	-1,372734086	0,235525737
216431_at	-	-1,137208348	-1,372734086	0,235525737
214199_at	SFTPD	-1,137208348	-1,372734086	0,235525737
242465_at	LOC100505592	-1,137208348	-1,372734086	0,235525737
236592_at	-	-1,137208348	-1,372734086	0,235525737
214480_at	ETV3	-1,137208348	-1,372734086	0,235525737
201847_at	LIPA /// LOC10050	4,161221733	3,925716571	0,235505161
225067_at	ULK3	1,625482993	1,390070295	0,235412698
235117_at	CHAC2	4,170262037	3,934892246	0,235369791
211972_x_at	RPLP0	8,379779726	8,144668085	0,23511164
223356_s_at	MTIF3	3,966733349	3,732068422	0,234664927
209340_at	UAP1	3,852052012	3,617435945	0,234616067
244063_at	BTN2A1	0,677745787	0,443143223	0,234602564
221253_s_at	MUTED-TXNDC5 //	5,030908502	4,796421511	0,234486991
203925_at	GCLM	4,175145566	3,940688263	0,234457302
204280_at	RGS14	1,701542258	1,46719264	0,234349618
1562235_s_at	-	-2,336678463	-2,570522741	0,233844278
206007_at	PRG4	-2,336678463	-2,570522741	0,233844278
1561521_at	S100B	-2,336678463	-2,570522741	0,233844278
1560851_at	C10orf136	-2,336678463	-2,570522741	0,233844278
1561105_at	CLDN11	-2,336678463	-2,570522741	0,233844278
220817_at	TRPC4	-2,336678463	-2,570522741	0,233844278
1552721_a_at	FGF1	-2,336678463	-2,570522741	0,233844278
240208_at	-	-2,336678463	-2,570522741	0,233844278
1555079_at	SPATA6L	-2,336678463	-2,570522741	0,233844278
1567698_x_at	-	-2,336678463	-2,570522741	0,233844278
238160_at	ACOT12	-2,336678463	-2,570522741	0,233844278
1560104_at	-	-2,336678463	-2,570522741	0,233844278
231499_s_at	-	-2,336678463	-2,570522741	0,233844278
1563333_at	-	-2,336678463	-2,570522741	0,233844278
234588_at	-	-2,336678463	-2,570522741	0,233844278
242197_x_at	CD36	-2,336678463	-2,570522741	0,233844278
211642_at	-	-2,336678463	-2,570522741	0,233844278

234844_at	ZNF407	-2,336678463	-2,570522741	0,233844278
1558828_s_at	-	-2,336678463	-2,570522741	0,233844278
237305_at	-	-2,336678463	-2,570522741	0,233844278
243652_at	-	-2,336678463	-2,570522741	0,233844278
234582_at	-	-2,336678463	-2,570522741	0,233844278
1560419_at	LOC100127974	-2,336678463	-2,570522741	0,233844278
208477_at	KCNC1	-2,336678463	-2,570522741	0,233844278
220037_s_at	LYVE1	-2,336678463	-2,570522741	0,233844278
242880_at	NALCN	-2,336678463	-2,570522741	0,233844278
217145_at	IGK@ /// IGKC	-2,336678463	-2,570522741	0,233844278
1553105_s_at	DSG2	-2,336678463	-2,570522741	0,233844278
1558599_at	-	-2,336678463	-2,570522741	0,233844278
215835_at	LOC100653174	-2,336678463	-2,570522741	0,233844278
232010_at	FSTL5	-2,336678463	-2,570522741	0,233844278
1557383_a_at	-	0,319287178	0,085534992	0,233752185
240098_at	RIF1	0,319287178	0,085534992	0,233752185
225555_x_at	AURKAIP1	0,319287178	0,085534992	0,233752185
200725_x_at	RPL10 /// SNORA7	8,215078121	7,981542298	0,233535823
216755_at	OSBPL10	-0,088974936	-0,322340048	0,233365112
227931_at	INO80D	1,88491011	1,651676651	0,233233459
206352_s_at	PEX10	1,116462765	0,883374248	0,233088516
236923_x_at	-	1,116462765	0,883374248	0,233088516
210571_s_at	CMAHP	3,521615383	3,288532747	0,233082636
200873_s_at	CCT8	7,366000965	7,133670309	0,232330656
240683_at	-	0,001114523	-0,231045907	0,232160429
205102_at	TMPRSS2	0,001114523	-0,231045907	0,232160429
1567010_at	-	0,001114523	-0,231045907	0,232160429
214272_at	CYLD	0,001114523	-0,231045907	0,232160429
242039_at	ARAP1	0,001114523	-0,231045907	0,232160429
234388_at	-	0,001114523	-0,231045907	0,232160429
1556149_at	ARVCF	0,001114523	-0,231045907	0,232160429
207131_x_at	GGT1 /// GGT2 ///	2,537731447	2,305621062	0,232110386
234982_at	UBR3	2,248155141	2,016139502	0,232015639
200981_x_at	GNAS	6,264261931	6,032263037	0,231998893
224814_at	DPP7	2,444186596	2,212304392	0,231882204
218178_s_at	CHMP1B	3,873276566	3,641397622	0,231878944
212316_at	NUP210	3,286844008	3,05501254	0,231831468
208826_x_at	HINT1	7,498782089	7,267042392	0,231739697
230659_at	-	1,802733148	1,571112806	0,231620342
215966_x_at	GK3P	0,244681185	0,013190398	0,231490787
1568877_a_at	ACBD5	0,244681185	0,013190398	0,231490787
218323_at	RHOT1	3,603313992	3,372163924	0,231150069
204077_x_at	ENTPD4	0,167727503	-0,063377083	0,231104586
217647_at	-	0,167727503	-0,063377083	0,231104586
222681_at	POGLUT1	2,476788561	2,245685006	0,231103554
226014_at	EIF3F	0,08619576	-0,144765583	0,230961343
215834_x_at	SCARB1	0,08619576	-0,144765583	0,230961343
214117_s_at	BTD	0,08619576	-0,144765583	0,230961343
217979_at	TSPAN13	0,764143511	0,533258442	0,230885069
218361_at	GOLPH3L	3,001575397	2,770696222	0,230879175

203253_s_at	PPIP5K2	3,710746795	3,479912486	0,230834309
203620_s_at	FCHSD2	3,45766549	3,227213858	0,230451632
1558622_a_at	ZNF548	-1,289805289	-1,520022123	0,230216834
206058_at	SLC6A12	-1,289805289	-1,520022123	0,230216834
213680_at	KRT6B	-1,289805289	-1,520022123	0,230216834
233574_at	BCORL1	-1,289805289	-1,520022123	0,230216834
236954_at	BOLL	-1,289805289	-1,520022123	0,230216834
228143_at	CP	-1,289805289	-1,520022123	0,230216834
233155_at	UPP2	-1,289805289	-1,520022123	0,230216834
241322_at	-	-1,289805289	-1,520022123	0,230216834
239239_at	BANCR	-1,289805289	-1,520022123	0,230216834
1554719_at	NDUFA10	-1,289805289	-1,520022123	0,230216834
205722_s_at	GFRA2	-1,289805289	-1,520022123	0,230216834
233643_at	FAM86B1 /// FAM	-1,289805289	-1,520022123	0,230216834
231740_at	KCNJ11	-1,289805289	-1,520022123	0,230216834
1556538_at	MFI2	-1,289805289	-1,520022123	0,230216834
207675_x_at	ARTN	-1,289805289	-1,520022123	0,230216834
1553041_at	HTR3C	-1,289805289	-1,520022123	0,230216834
225994_at	CPSF2	3,386206804	3,155995136	0,230211667
223996_s_at	MRPL30	3,327128828	3,096957539	0,230171288
221135_s_at	ASTE1	2,285848435	2,055739171	0,230109265
203092_at	TIMM44	2,199941239	1,970106792	0,229834446
226800_at	EFCAB7	2,460890032	2,23115548	0,229734552
219344_at	SLC29A3	0,847529938	0,61779983	0,229730108
211385_x_at	SULT1A2	1,830468611	1,601076277	0,229392335
209947_at	UBAP2L	1,830468611	1,601076277	0,229392335
204321_at	NEO1	1,364555912	1,135235995	0,229319917
228282_at	MFSD8	1,65554524	1,426280426	0,229264813
209698_at	CCHCR1	2,507817181	2,278572464	0,229244717
212363_x_at	ACTG1	8,230818886	8,001760029	0,229058857
51192_at	SSH3	1,182925501	0,954028247	0,228897255
233329_s_at	KRCC1	3,03433073	2,805545859	0,228784871
219811_at	DGCR8 /// MIR130	1,071336699	0,842599219	0,228737479
220897_at	-	-2,163157732	-2,391863529	0,228705797
240992_at	-	-2,163157732	-2,391863529	0,228705797
1566780_at	-	-2,163157732	-2,391863529	0,228705797
1564169_at	-	-2,163157732	-2,391863529	0,228705797
1559770_at	-	-2,163157732	-2,391863529	0,228705797
244067_x_at	-	-2,163157732	-2,391863529	0,228705797
217084_at	IGHA1 /// IGHG1 /,	-2,163157732	-2,391863529	0,228705797
220025_at	TBR1	-2,163157732	-2,391863529	0,228705797
1562011_at	LOC100506413	-2,163157732	-2,391863529	0,228705797
243733_at	-	-2,163157732	-2,391863529	0,228705797
1558894_a_at	CCDC67	-2,163157732	-2,391863529	0,228705797
223796_at	CNTNAP3 /// CNTN	-2,163157732	-2,391863529	0,228705797
237938_at	-	-2,163157732	-2,391863529	0,228705797
206349_at	LGI1	-2,163157732	-2,391863529	0,228705797
222171_s_at	PKNOX2	-2,163157732	-2,391863529	0,228705797
237148_at	-	-2,163157732	-2,391863529	0,228705797
242791_at	FBXO3	-2,163157732	-2,391863529	0,228705797

1565424_at	LINC00529	-2,163157732	-2,391863529	0,228705797
241101_at	-	-2,163157732	-2,391863529	0,228705797
1553655_at	CDC20B	-2,163157732	-2,391863529	0,228705797
1569923_s_at	LINC00491	-2,163157732	-2,391863529	0,228705797
229532_at	ZNF502	-2,163157732	-2,391863529	0,228705797
235578_at	ABCC9	-2,163157732	-2,391863529	0,228705797
208083_s_at	ITGB6	-2,163157732	-2,391863529	0,228705797
208383_s_at	PCK1	-2,163157732	-2,391863529	0,228705797
1557787_at	-	-2,163157732	-2,391863529	0,228705797
236297_at	-	-2,163157732	-2,391863529	0,228705797
1566517_at	-	-2,163157732	-2,391863529	0,228705797
243412_at	-	-2,163157732	-2,391863529	0,228705797
231156_at	-	-2,163157732	-2,391863529	0,228705797
229209_at	SNHG10	-2,163157732	-2,391863529	0,228705797
222932_at	EHF	-2,163157732	-2,391863529	0,228705797
209830_s_at	SLC9A3R2	-2,163157732	-2,391863529	0,228705797
227858_at	PCNXL3	1,139057614	0,910483921	0,228573694
1559960_x_at	SYCE1L	0,707657549	0,479089184	0,228568364
229615_at	-	2,321828846	2,093850742	0,227978104
215653_at	-	-2,495152701	-2,723114724	0,227962024
232056_at	SCEL	-2,495152701	-2,723114724	0,227962024
1557843_at	-	-2,495152701	-2,723114724	0,227962024
1568673_s_at	EAF2	-2,495152701	-2,723114724	0,227962024
234022_at	-	-2,495152701	-2,723114724	0,227962024
1559722_at	-	-2,495152701	-2,723114724	0,227962024
235599_at	LOC339535	-2,495152701	-2,723114724	0,227962024
216406_at	-	-2,495152701	-2,723114724	0,227962024
231348_s_at	LMO3	-2,495152701	-2,723114724	0,227962024
1553374_at	PRO2949	-2,495152701	-2,723114724	0,227962024
1567687_at	CECR9	-2,495152701	-2,723114724	0,227962024
1554296_at	CYP19A1	-2,495152701	-2,723114724	0,227962024
213574_s_at	-	5,352422261	5,124487446	0,227934814
215691_x_at	HSPB11	5,88707333	5,659281871	0,227791459
223178_s_at	NT5DC1	3,096793374	2,869317249	0,227476125
226330_s_at	FAM48A	3,336134047	3,109293915	0,226840132
201630_s_at	ACP1	5,10427949	4,877484654	0,226794836
212501_at	CEBPB	3,300874993	3,074131603	0,22674339
212920_at	REST	2,031158928	1,804519951	0,226638977
209943_at	FBXL4	2,248155141	2,021531611	0,22662353
237059_at	-	0,491906512	0,265475485	0,226431027
218647_s_at	YRDC	3,701002444	3,474637573	0,226364871
56748_at	TRIM10	-1,869103657	-2,095275158	0,226171501
37152_at	PPARD	2,531852263	2,305757418	0,226094845
201550_x_at	ACTG1	8,457595341	8,231511801	0,22608354
207425_s_at	40057	1,701542258	1,475630127	0,22591213
207268_x_at	ABI2	1,701542258	1,475630127	0,22591213
1552486_s_at	LACTB	1,701542258	1,475630127	0,22591213
200851_s_at	IST1	5,210892881	4,985027501	0,22586538
231208_at	-	-1,537851782	-1,763664074	0,225812292
1553410_a_at	ABCC12	-1,537851782	-1,763664074	0,225812292

207400_at	NPY5R	-1,537851782	-1,763664074	0,225812292
1555144_at	ARL17A /// ARL17I	-1,537851782	-1,763664074	0,225812292
217699_at	-	-1,537851782	-1,763664074	0,225812292
240679_at	-	-1,537851782	-1,763664074	0,225812292
1564621_a_at	MEI1	-1,537851782	-1,763664074	0,225812292
239584_at	-	-1,537851782	-1,763664074	0,225812292
1570221_at	-	-1,537851782	-1,763664074	0,225812292
244484_at	-	-1,537851782	-1,763664074	0,225812292
1552758_at	HDAC9	-1,537851782	-1,763664074	0,225812292
1559479_at	LOC285540	-1,537851782	-1,763664074	0,225812292
241465_at	-	-1,537851782	-1,763664074	0,225812292
240902_at	LOC283624	-1,537851782	-1,763664074	0,225812292
208790_s_at	PTRF	-1,537851782	-1,763664074	0,225812292
236645_at	LOC100506312	-1,537851782	-1,763664074	0,225812292
215987_at	RAPGEF2	-1,537851782	-1,763664074	0,225812292
231641_at	EBLN2	-1,537851782	-1,763664074	0,225812292
1570014_at	-	-1,537851782	-1,763664074	0,225812292
1562007_at	-	-1,537851782	-1,763664074	0,225812292
236057_at	KCTD1	-1,537851782	-1,763664074	0,225812292
1566571_at	-	-1,537851782	-1,763664074	0,225812292
1552938_at	ZIC5	-1,537851782	-1,763664074	0,225812292
217566_s_at	TGM4	-1,537851782	-1,763664074	0,225812292
217318_x_at	KIR2DL1 /// KIR2D	-1,537851782	-1,763664074	0,225812292
236684_at	RQCD1	-1,537851782	-1,763664074	0,225812292
230283_at	NEURL2	-1,537851782	-1,763664074	0,225812292
237512_at	-	-1,537851782	-1,763664074	0,225812292
211549_s_at	HPGD	-1,537851782	-1,763664074	0,225812292
240623_at	-	-1,537851782	-1,763664074	0,225812292
1556240_at	-	-1,537851782	-1,763664074	0,225812292
227425_at	REPS2	-1,537851782	-1,763664074	0,225812292
1555757_at	C7orf34	-1,537851782	-1,763664074	0,225812292
213520_at	RECQL4	1,788922638	1,563256447	0,225666191
204847_at	ZBTB11	3,685074281	3,459443789	0,225630492
227465_at	MAU2	2,811067138	2,585619776	0,225447362
219175_s_at	SLC41A3	2,460890032	2,235857731	0,225032302
1553569_at	COX2	8,407398621	8,182528732	0,224869889
212482_at	RMND5A	3,282538749	3,05768517	0,224853579
204843_s_at	PRKAR2A	0,425036312	0,20062106	0,224415253
1558675_s_at	NEMF	2,552836808	2,328492509	0,2243443
228944_at	-	1,094118704	0,869824768	0,224293936
235810_at	ZNF182	1,094118704	0,869824768	0,224293936
209084_s_at	RAB28	2,312738346	2,088449486	0,22428886
1553552_at	TAAR8	-0,791294935	-1,015398016	0,224103081
238200_at	-	-0,791294935	-1,015398016	0,224103081
210442_at	IL1RL1	-0,791294935	-1,015398016	0,224103081
205470_s_at	KLK11	-0,791294935	-1,015398016	0,224103081
220904_at	LINC00574	-0,791294935	-1,015398016	0,224103081
231540_at	LOC100130691	-0,791294935	-1,015398016	0,224103081
240048_at	STRC	-0,791294935	-1,015398016	0,224103081
212793_at	DAAM2	-0,791294935	-1,015398016	0,224103081

215998_at	-	-0,791294935	-1,015398016	0,224103081
1561530_at	-	-0,791294935	-1,015398016	0,224103081
206890_at	IL12RB1	-0,791294935	-1,015398016	0,224103081
237098_at	C1orf21	-0,791294935	-1,015398016	0,224103081
243565_at	CCDC150	-0,791294935	-1,015398016	0,224103081
216295_s_at	CLTA	5,112028025	4,888115253	0,223912772
200016_x_at	HNRNPA1	7,89891387	7,675078294	0,223835575
219483_s_at	PORCN	0,791908897	0,568155442	0,223753455
201820_at	KRT5	-0,437449947	-0,661178575	0,223728627
226622_at	MUC20	-0,437449947	-0,661178575	0,223728627
236178_at	C6orf162	-0,437449947	-0,661178575	0,223728627
230321_at	TOR1AIP2	-0,437449947	-0,661178575	0,223728627
207986_x_at	CYB561	-0,437449947	-0,661178575	0,223728627
227327_at	MEGF8	1,267146969	1,043469112	0,223677857
201972_at	ATP6V1A	5,286023994	5,062440867	0,223583127
243943_x_at	C6orf52	0,648195588	0,424693306	0,223502282
60528_at	JMJD7 /// JMJD7-F	0,988156191	0,764697289	0,223458902
234974_at	GALM	2,492739983	2,269637658	0,223102324
203803_at	PCYOX1	1,182925501	0,960042218	0,222883283
230779_at	TNRC6B	1,510970097	1,28853674	0,222433357
212450_at	SECISBP2L	1,510970097	1,28853674	0,222433357
207825_s_at	GHRHR	-0,332405896	-0,554696666	0,22229077
215399_s_at	OS9	1,421391637	1,199296812	0,222094825
212749_s_at	RCHY1	3,237994092	3,01591662	0,222077471
225402_at	TP53RK	2,71254166	2,490640503	0,221901157
201751_at	JOSD1	4,411729887	4,190235418	0,221494469
35201_at	HNRNPL	4,369679237	4,148206345	0,221472892
217734_s_at	WDR6	3,081633533	2,860202326	0,221431206
212476_at	ACAP2	3,065977723	2,844606929	0,221370794
241666_at	C3orf23	1,528073958	1,306903969	0,221169989
225685_at	CDC42EP3	1,528073958	1,306903969	0,221169989
1558942_at	ZNF765	2,304041045	2,082919929	0,221121117
237426_at	SP100	-0,924000698	-1,145057014	0,221056317
205924_at	RAB3B	-0,924000698	-1,145057014	0,221056317
241314_at	-	-0,924000698	-1,145057014	0,221056317
241000_at	-	-0,924000698	-1,145057014	0,221056317
205737_at	KCNQ2	-0,924000698	-1,145057014	0,221056317
211640_x_at	IGHG1 /// IGHM	-0,924000698	-1,145057014	0,221056317
243094_at	-	-0,924000698	-1,145057014	0,221056317
243653_at	SHROOM3	-0,924000698	-1,145057014	0,221056317
234011_at	-	-0,924000698	-1,145057014	0,221056317
234585_at	-	-0,924000698	-1,145057014	0,221056317
214347_s_at	DDC	-0,924000698	-1,145057014	0,221056317
1554847_at	ATP6V1B1	-0,924000698	-1,145057014	0,221056317
1555063_at	USP6	-0,924000698	-1,145057014	0,221056317
234834_at	-	-0,924000698	-1,145057014	0,221056317
223194_s_at	SLC22A23	-0,924000698	-1,145057014	0,221056317
237350_at	TTC36	-0,924000698	-1,145057014	0,221056317
214750_at	PLAC4	-0,924000698	-1,145057014	0,221056317
1556034_s_at	MTMR11	-0,924000698	-1,145057014	0,221056317

208863_s_at	SRSF1	4,398126622	4,177087169	0,221039453
212161_at	AP2A2	-0,230432956	-0,45121326	0,220780304
235219_at	C5orf55	-0,230432956	-0,45121326	0,220780304
1557970_s_at	RPS6KA2	-0,230432956	-0,45121326	0,220780304
1554920_at	SCEL	-2,834722577	-3,055403285	0,220680708
238861_at	SSBP2	0,282358733	0,061773582	0,220585151
1553815_a_at	TCEANC	0,282358733	0,061773582	0,220585151
229286_at	MAGEE1	0,282358733	0,061773582	0,220585151
238093_at	LOC100129722	0,282358733	0,061773582	0,220585151
213176_s_at	LTBP4	0,282358733	0,061773582	0,220585151
223324_s_at	TRPM7	1,936380191	1,715863686	0,220516506
211975_at	ARFGAP2	2,719381633	2,498884538	0,220497095
215596_s_at	LTN1	3,291641956	3,071469991	0,220171965
206240_s_at	ZNF136	1,20396005	0,98381571	0,22014434
202169_s_at	AASDHPPT	4,991134457	4,771059175	0,220075282
219273_at	CCNK	-0,551284523	-0,771340337	0,220055814
241058_at	-	-0,551284523	-0,771340337	0,220055814
232845_at	CDH23 /// LOC100	-0,551284523	-0,771340337	0,220055814
208560_at	KCNA10	-0,551284523	-0,771340337	0,220055814
239350_at	MARVELD3	-0,551284523	-0,771340337	0,220055814
206077_at	KEL	-0,551284523	-0,771340337	0,220055814
1555617_x_at	-	-0,66934516	-0,889222211	0,219877051
228350_at	UNC13D	-0,66934516	-0,889222211	0,219877051
232077_s_at	YPEL3	-0,66934516	-0,889222211	0,219877051
219455_at	C7orf63	-0,66934516	-0,889222211	0,219877051
1564338_at	-	-0,66934516	-0,889222211	0,219877051
215036_at	IGLC1	-0,66934516	-0,889222211	0,219877051
203954_x_at	CLDN3	-0,66934516	-0,889222211	0,219877051
239842_x_at	-	-0,66934516	-0,889222211	0,219877051
232142_at	ISY1	-0,135065865	-0,354781582	0,219715716
1553990_at	C16orf79	-0,135065865	-0,354781582	0,219715716
217947_at	CMTM6	5,392490003	5,172780458	0,219709545
1554662_at	C17orf47	-1,06282519	-1,282520722	0,219695532
210341_at	MYT1	-1,06282519	-1,282520722	0,219695532
243913_at	-	-1,06282519	-1,282520722	0,219695532
1562748_at	LOC253044	-1,06282519	-1,282520722	0,219695532
229416_at	CLPTM1L	-1,06282519	-1,282520722	0,219695532
207385_at	TFDP3	-1,06282519	-1,282520722	0,219695532
243352_at	ALPK1	-1,06282519	-1,282520722	0,219695532
237382_at	GLT25D2	-1,06282519	-1,282520722	0,219695532
232533_at	METTL8	-1,06282519	-1,282520722	0,219695532
240368_at	-	-1,06282519	-1,282520722	0,219695532
1562497_at	-	-1,06282519	-1,282520722	0,219695532
208092_s_at	FAM49A	-1,06282519	-1,282520722	0,219695532
211058_x_at	LOC100288366 ///	8,121879105	7,90222401	0,219655095
222221_x_at	EHD1	2,392346173	2,173028091	0,219318082
211060_x_at	GPAA1	2,908690075	2,689594859	0,219095216
219447_s_at	SLC35C2	1,458255986	1,239268196	0,218987791
1554072_s_at	CCDC67	-2,416625063	-2,635549085	0,218924021
1569995_at	LOC152586	-2,416625063	-2,635549085	0,218924021

243390_at	-	-2,416625063	-2,635549085	0,218924021
217443_at	-	-2,416625063	-2,635549085	0,218924021
240564_x_at	-	-2,416625063	-2,635549085	0,218924021
228504_at	SCN7A	-2,416625063	-2,635549085	0,218924021
220324_at	LINC00472	-2,416625063	-2,635549085	0,218924021
237149_at	-	-2,416625063	-2,635549085	0,218924021
237804_at	DNAH11	-2,416625063	-2,635549085	0,218924021
240137_at	-	-2,416625063	-2,635549085	0,218924021
233073_at	-	-2,416625063	-2,635549085	0,218924021
1554418_s_at	SPOCK3	-2,416625063	-2,635549085	0,218924021
242101_at	-	-2,416625063	-2,635549085	0,218924021
207195_at	CNTN6	-2,416625063	-2,635549085	0,218924021
1563312_at	-	-2,416625063	-2,635549085	0,218924021
220111_s_at	ANO2	-2,416625063	-2,635549085	0,218924021
231675_s_at	ADH4	-2,416625063	-2,635549085	0,218924021
231470_at	LOC400680	-2,416625063	-2,635549085	0,218924021
217272_s_at	SERPINB13	-2,416625063	-2,635549085	0,218924021
1568844_at	-	-2,416625063	-2,635549085	0,218924021
1558397_at	PECAM1	-2,416625063	-2,635549085	0,218924021
242468_at	-	-2,416625063	-2,635549085	0,218924021
236336_at	BOLA3-AS1	-2,416625063	-2,635549085	0,218924021
242025_at	-	-2,416625063	-2,635549085	0,218924021
203565_s_at	MNAT1	2,492739983	2,274084398	0,218655585
228530_at	MZT1	0,900065578	0,681874787	0,218190791
227592_at	ALDH16A1	1,047341799	0,829309537	0,218032262
204510_at	CDC7	3,592169709	3,374450349	0,21771936
201778_s_at	KIAA0494	3,632502157	3,414783177	0,21771898
222938_x_at	ENPP3	0,045517965	-0,172194225	0,217712189
228349_at	KIAA1958	0,045517965	-0,172194225	0,217712189
242154_x_at	LRR1	0,045517965	-0,172194225	0,217712189
225865_x_at	TH1L	4,448374788	4,230731855	0,217642932
209384_at	PROSC	4,538810963	4,321202891	0,217608072
1555062_s_at	GTPBP3	0,127732831	-0,089635713	0,217368543
235135_at	TAF10	0,127732831	-0,089635713	0,217368543
1556274_at	-	0,127732831	-0,089635713	0,217368543
206050_s_at	RNH1	3,501655014	3,284313623	0,217341391
227818_at	CEP85	3,007119335	2,790111983	0,217007352
210336_x_at	MZF1	1,225123479	1,008210238	0,216913241
223604_at	GARNL3	-0,043925712	-0,260526297	0,216600585
239450_at	-	-0,043925712	-0,260526297	0,216600585
224562_at	WASF2	3,513985141	3,297572471	0,216412671
1552541_at	TAGAP	0,677745787	0,461385738	0,216360049
216550_x_at	ANKRD12	1,910557155	1,694547353	0,216009802
224653_at	EIF4EBP2	3,265594257	3,049587049	0,216007208
214080_x_at	PRKCSH	2,778527892	2,562621495	0,215906397
223165_s_at	IP6K2	1,816938997	1,601076277	0,215862721
202272_s_at	FBXO28	3,792209911	3,576411191	0,21579872
1561343_a_at	LOC150005	-2,250580859	-2,466349414	0,215768555
239150_at	SNTN	-2,250580859	-2,466349414	0,215768555
1570594_at	-	-2,250580859	-2,466349414	0,215768555

229331_at	SPATA18	-2,250580859	-2,466349414	0,215768555
240068_at	LINC00323	-2,250580859	-2,466349414	0,215768555
217392_at	-	-2,250580859	-2,466349414	0,215768555
1555341_at	UNC5C	-2,250580859	-2,466349414	0,215768555
1556867_at	-	-2,250580859	-2,466349414	0,215768555
234700_s_at	RNASE7	-2,250580859	-2,466349414	0,215768555
224242_at	GALP	-2,250580859	-2,466349414	0,215768555
231665_at	ARG1	-2,250580859	-2,466349414	0,215768555
238634_x_at	-	-2,250580859	-2,466349414	0,215768555
224533_s_at	-	-2,250580859	-2,466349414	0,215768555
1552393_at	ENTHD1	-2,250580859	-2,466349414	0,215768555
213994_s_at	SPON1	-2,250580859	-2,466349414	0,215768555
1557439_at	-	-2,250580859	-2,466349414	0,215768555
1560846_at	-	-2,250580859	-2,466349414	0,215768555
1558683_a_at	HMGA2	-2,250580859	-2,466349414	0,215768555
208448_x_at	IFNA16	-2,250580859	-2,466349414	0,215768555
1557107_at	SLC26A4-AS1	-2,250580859	-2,466349414	0,215768555
214884_at	MCF2	-2,250580859	-2,466349414	0,215768555
1561276_at	DOCK5	-2,250580859	-2,466349414	0,215768555
1565578_at	-	-2,250580859	-2,466349414	0,215768555
243862_at	RASEF	-2,250580859	-2,466349414	0,215768555
1563677_at	CDON	-2,250580859	-2,466349414	0,215768555
229494_s_at	PHLDA2	-2,250580859	-2,466349414	0,215768555
234215_at	-	-2,250580859	-2,466349414	0,215768555
1564158_a_at	LOC100130894	-2,250580859	-2,466349414	0,215768555
239506_s_at	LINC00608	-2,250580859	-2,466349414	0,215768555
1557583_at	ST18	-2,250580859	-2,466349414	0,215768555
229779_at	COL4A4	-2,250580859	-2,466349414	0,215768555
1561948_at	COQ10B	-2,250580859	-2,466349414	0,215768555
234727_at	DNAH7	-2,250580859	-2,466349414	0,215768555
206382_s_at	BDNF	-2,250580859	-2,466349414	0,215768555
207089_at	NRAP	-2,250580859	-2,466349414	0,215768555
1564642_at	RUNX1T1	-2,250580859	-2,466349414	0,215768555
220844_at	TCEB3B	-2,250580859	-2,466349414	0,215768555
1560946_at	-	-2,250580859	-2,466349414	0,215768555
223867_at	TEKT3	-2,250580859	-2,466349414	0,215768555
208396_s_at	PDE1A	-2,250580859	-2,466349414	0,215768555
1566916_at	HPYR1	-2,250580859	-2,466349414	0,215768555
1570360_s_at	DDX3Y	-2,250580859	-2,466349414	0,215768555
231067_s_at	-	-2,250580859	-2,466349414	0,215768555
1569608_x_at	-	-2,250580859	-2,466349414	0,215768555
208640_at	RAC1	5,437566312	5,221822966	0,215743346
218741_at	CENPM	3,461795772	3,246064373	0,215731399
1552858_at	MAGEB6	-2,641232718	-2,856570137	0,215337419
1568662_at	PWRN2	-2,641232718	-2,856570137	0,215337419
1563110_at	-	-2,641232718	-2,856570137	0,215337419
228993_s_at	BBIP1	2,932161212	2,717094254	0,215066958
204711_at	KIAA0753	2,21987062	2,004917973	0,214952648
222369_at	NAA40	2,392346173	2,177710147	0,214636027
201708_s_at	NIPSNAP1	3,386206804	3,171631256	0,214575548

224845_s_at	SLAIN2	2,190140919	1,975797592	0,214343328
218142_s_at	CRBN	2,149438457	1,935144844	0,214293614
220150_s_at	FAM184A	1,326463531	1,112548615	0,213914916
234995_at	SPICE1	1,640193838	1,426280426	0,213913412
202842_s_at	DNAJB9	3,007119335	2,793634788	0,213484547
237040_at	CWF19L2	1,402495885	1,189078962	0,213416922
220054_at	IL23A	1,160996507	0,947673886	0,213322621
228148_at	ZNF584	1,160996507	0,947673886	0,213322621
225026_at	CHD6	0,847529938	0,634301128	0,21322881
219919_s_at	SSH3	0,847529938	0,634301128	0,21322881
1556730_at	LOC652993	-1,452713826	-1,665791776	0,21307795
231129_at	LOC728012	-1,452713826	-1,665791776	0,21307795
224404_s_at	FCRL5	-1,452713826	-1,665791776	0,21307795
243270_at	FAM205A	-1,452713826	-1,665791776	0,21307795
214262_at	TRIP6	-1,452713826	-1,665791776	0,21307795
222834_s_at	GNG12	-1,452713826	-1,665791776	0,21307795
237204_at	-	-1,452713826	-1,665791776	0,21307795
232321_at	MUC17	-1,452713826	-1,665791776	0,21307795
205221_at	HGD	-1,452713826	-1,665791776	0,21307795
202563_at	C14orf1	-1,452713826	-1,665791776	0,21307795
239885_at	-	-1,452713826	-1,665791776	0,21307795
241831_at	-	-1,452713826	-1,665791776	0,21307795
1564931_at	-	-1,452713826	-1,665791776	0,21307795
237166_at	-	-1,452713826	-1,665791776	0,21307795
205475_at	SCRG1	-1,452713826	-1,665791776	0,21307795
235524_at	RALGAPA1	-1,452713826	-1,665791776	0,21307795
210123_s_at	CHRFAM7A /// CH	-1,452713826	-1,665791776	0,21307795
1562078_at	-	-1,452713826	-1,665791776	0,21307795
235856_at	-	-1,452713826	-1,665791776	0,21307795
239429_at	-	-1,452713826	-1,665791776	0,21307795
235726_at	ZBTB10	-1,452713826	-1,665791776	0,21307795
220013_at	EPHX3	-1,452713826	-1,665791776	0,21307795
220706_at	ADAMTS7	-1,452713826	-1,665791776	0,21307795
1560874_at	EFCAB4B	-1,452713826	-1,665791776	0,21307795
226828_s_at	HEYL	-1,452713826	-1,665791776	0,21307795
238097_at	GAS6-AS1	-1,452713826	-1,665791776	0,21307795
231652_at	-	-1,452713826	-1,665791776	0,21307795
1554364_at	PPP2R5C	-1,452713826	-1,665791776	0,21307795
242665_at	FMNL2	-1,452713826	-1,665791776	0,21307795
218533_s_at	UCKL1	2,159837409	1,946903052	0,212934357
200819_s_at	RPS15	8,415285794	8,202380816	0,212904977
225638_at	C1orf31	4,144849571	3,931967762	0,212881809
225134_at	SPRYD3	1,510970097	1,298349073	0,212621024
209666_s_at	CHUK	2,698955954	2,486505579	0,212450376
209565_at	RNF113A	3,64600241	3,433779805	0,212222605
217770_at	PIGT	2,817838865	2,605630202	0,212208662
218051_s_at	NT5DC2	2,476788561	2,264929589	0,211858971
211977_at	GPR107	1,267146969	1,055317091	0,211829878
207525_s_at	GIPC1	1,267146969	1,055317091	0,211829878
203466_at	MPV17	3,117098466	2,906008589	0,211089877

209214_s_at	EWSR1	4,954619423	4,743674982	0,21094444
1560997_at	-	-2,569309811	-2,780225148	0,210915336
1553868_a_at	KIAA0825	-2,569309811	-2,780225148	0,210915336
220014_at	PRR16	-2,569309811	-2,780225148	0,210915336
1554298_a_at	WDR49	-2,569309811	-2,780225148	0,210915336
234335_s_at	FAM84A	-2,569309811	-2,780225148	0,210915336
244742_at	PAH	-2,569309811	-2,780225148	0,210915336
1563649_at	-	-2,569309811	-2,780225148	0,210915336
211169_s_at	PPP1R3A	-2,569309811	-2,780225148	0,210915336
217343_at	-	-2,569309811	-2,780225148	0,210915336
1555623_at	-	-2,569309811	-2,780225148	0,210915336
1552359_at	MCMDC2	-2,569309811	-2,780225148	0,210915336
242496_at	ART4	-2,569309811	-2,780225148	0,210915336
221891_x_at	HSPA8 /// SNORD1	7,996818083	7,786328861	0,210489222
212326_at	VPS13D	0,707657549	0,497424714	0,210232834
210672_s_at	NPRL3	0,707657549	0,497424714	0,210232834
223205_s_at	L3MBTL2	2,642918178	2,432792627	0,210125552
200095_x_at	RPS10	8,489319322	8,279481758	0,209837564
225797_at	MRPL54	3,828857041	3,619206676	0,209650365
237130_at	-	-1,212652659	-1,422257026	0,209604367
242526_at	-	-1,212652659	-1,422257026	0,209604367
205216_s_at	APOH	-1,212652659	-1,422257026	0,209604367
1566208_at	TCEA1	-1,212652659	-1,422257026	0,209604367
1553447_at	AGBL1	-1,212652659	-1,422257026	0,209604367
223605_at	SLC25A18	-1,212652659	-1,422257026	0,209604367
1552453_a_at	WDR88	-1,212652659	-1,422257026	0,209604367
240997_at	-	-1,212652659	-1,422257026	0,209604367
243464_at	-	-1,212652659	-1,422257026	0,209604367
232045_at	PHACTR1	-1,212652659	-1,422257026	0,209604367
210602_s_at	CDH6	-1,212652659	-1,422257026	0,209604367
243895_x_at	-	-1,212652659	-1,422257026	0,209604367
240661_at	LOC100506459	-1,212652659	-1,422257026	0,209604367
210964_s_at	GYG2	-1,212652659	-1,422257026	0,209604367
223752_at	CFC1	-1,212652659	-1,422257026	0,209604367
1569387_at	CSGALNACT1	-1,212652659	-1,422257026	0,209604367
221447_s_at	GLT8D2	-1,212652659	-1,422257026	0,209604367
210497_x_at	SSX2 /// SSX2B	-1,212652659	-1,422257026	0,209604367
217315_s_at	KLK13	-1,212652659	-1,422257026	0,209604367
239055_at	-	-1,212652659	-1,422257026	0,209604367
204134_at	PDE2A	-1,212652659	-1,422257026	0,209604367
1562488_at	PIEZO2	-1,212652659	-1,422257026	0,209604367
1552799_at	TSNARE1	-1,212652659	-1,422257026	0,209604367
233840_at	LOC100130950	-1,212652659	-1,422257026	0,209604367
236044_at	PPAPDC1A	-1,212652659	-1,422257026	0,209604367
213716_s_at	SECTM1	-1,212652659	-1,422257026	0,209604367
240557_at	-	-1,212652659	-1,422257026	0,209604367
227780_s_at	ECSCR	-1,212652659	-1,422257026	0,209604367
228298_at	PCED1B	0,556428218	0,346964736	0,209463482
210011_s_at	EWSR1	3,517785666	3,308395318	0,209390348
213787_s_at	EBP	4,886957485	4,677643598	0,209313887

213583_x_at	EEF1A1 /// LOC10(	8,492899328	8,283837211	0,209062117
200062_s_at	RPL30	8,304382341	8,095435298	0,208947043
222580_at	ZNF644	4,036242149	3,827536095	0,208706055
230416_at	-	2,063971513	1,855279162	0,208692352
214030_at	CRYBG3	2,063971513	1,855279162	0,208692352
231271_x_at	NMRAL1	2,99543179	2,786807892	0,208623898
1552377_s_at	FAM18B2	0,791908897	0,583607503	0,208301394
218222_x_at	ARNT	0,791908897	0,583607503	0,208301394
226255_at	ZBTB33	3,804904984	3,596611499	0,208293485
226434_at	PPP1R35	3,322661262	3,114374925	0,208286337
238034_at	CANX	4,230697503	4,022483924	0,20821358
202282_at	HSD17B10	4,969542968	4,761725557	0,207817411
215603_x_at	GGT1 /// GGT2 ///	2,107136847	1,899778623	0,207358223
1558330_x_at	TONSL	0,244681185	0,03784737	0,206833815
204829_s_at	FOLR2	0,244681185	0,03784737	0,206833815
212929_s_at	FAM21A /// FAM2	2,725848647	2,519368159	0,206480488
225304_s_at	FUT5 /// NDUFA11	5,448444206	5,242026313	0,206417894
211416_x_at	GGTLC1	1,116462765	0,910483921	0,205978844
233727_at	-	-0,180990326	-0,386896102	0,205905776
1560089_at	LOC100289019	1,307201325	1,101780212	0,205421113
225440_at	AGPAT3	3,446505209	3,24169614	0,204809069
215322_at	LONRF1	-0,384193355	-0,589001171	0,204807816
230184_at	LOC100506421	-0,384193355	-0,589001171	0,204807816
205647_at	RAD52	-0,384193355	-0,589001171	0,204807816
1566249_at	-	-0,384193355	-0,589001171	0,204807816
230442_at	MTHFSD	-0,384193355	-0,589001171	0,204807816
216943_at	-	-0,384193355	-0,589001171	0,204807816
1560409_at	-	-0,384193355	-0,589001171	0,204807816
211542_x_at	RPS10	8,464219533	8,259525097	0,204694436
223431_at	CNO	2,890618076	2,686001625	0,204616451
218908_at	ASPSCR1	1,225123479	1,020514937	0,204608542
242076_at	-	-0,49284695	-0,697182596	0,204335646
221422_s_at	MIR600 /// MIR60	-0,49284695	-0,697182596	0,204335646
238782_at	-	-0,49284695	-0,697182596	0,204335646
207642_at	HCRT	-0,49284695	-0,697182596	0,204335646
208491_s_at	PGM5	-0,49284695	-0,697182596	0,204335646
1570328_s_at	C20orf62	-0,49284695	-0,697182596	0,204335646
219154_at	TMEM120B	-0,49284695	-0,697182596	0,204335646
207390_s_at	SMTN	-0,49284695	-0,697182596	0,204335646
211518_s_at	BMP4	-0,49284695	-0,697182596	0,204335646
238347_at	SLC38A10	-0,49284695	-0,697182596	0,204335646
1561107_at	-	-0,49284695	-0,697182596	0,204335646
237451_x_at	-	-0,280273599	-0,484558493	0,204284894
210723_x_at	-	-0,280273599	-0,484558493	0,204284894
1563217_at	-	-0,280273599	-0,484558493	0,204284894
241202_at	-	-0,280273599	-0,484558493	0,204284894
1555734_x_at	AP1S3	-0,280273599	-0,484558493	0,204284894
1560625_s_at	-	-0,280273599	-0,484558493	0,204284894
212195_at	IL6ST	2,817838865	2,613615641	0,204223224
221764_at	R3HDM4	2,75889404	2,55501164	0,2038824

208635_x_at	NACA	8,176763959	7,972903014	0,203860945
233766_at	SOBP	-1,982324124	-2,186086603	0,203762479
238899_at	-	-1,982324124	-2,186086603	0,203762479
223669_at	HEMGN	-1,982324124	-2,186086603	0,203762479
233714_at	-	-1,982324124	-2,186086603	0,203762479
216921_s_at	KRT35	-1,982324124	-2,186086603	0,203762479
1557405_at	LOC100130111	-1,982324124	-2,186086603	0,203762479
1559021_at	C3orf52	-1,982324124	-2,186086603	0,203762479
1555148_a_at	C2orf65	-1,982324124	-2,186086603	0,203762479
241960_at	CSMD1	-1,982324124	-2,186086603	0,203762479
216428_x_at	KIR3DX1	-1,982324124	-2,186086603	0,203762479
221801_x_at	NEFL	-1,982324124	-2,186086603	0,203762479
237985_at	-	-1,982324124	-2,186086603	0,203762479
1561560_at	-	-1,982324124	-2,186086603	0,203762479
238103_at	LOC100505989	-1,982324124	-2,186086603	0,203762479
1570289_at	LOC646736	-1,982324124	-2,186086603	0,203762479
223970_at	RETNLB	-1,982324124	-2,186086603	0,203762479
241126_at	-	-1,982324124	-2,186086603	0,203762479
244608_at	-	-1,982324124	-2,186086603	0,203762479
234281_at	ESPN /// ESPNP	-1,982324124	-2,186086603	0,203762479
1563495_at	SLC9C2	-1,982324124	-2,186086603	0,203762479
1569978_x_at	-	-1,982324124	-2,186086603	0,203762479
240409_at	TPTE2P5	-1,982324124	-2,186086603	0,203762479
1553080_at	CSN1S2AP	-1,982324124	-2,186086603	0,203762479
237906_at	-	-1,982324124	-2,186086603	0,203762479
1552730_at	DHH	-1,982324124	-2,186086603	0,203762479
207345_at	FST	-1,982324124	-2,186086603	0,203762479
236342_at	-	-1,982324124	-2,186086603	0,203762479
244465_at	-	-1,982324124	-2,186086603	0,203762479
238958_at	UBE2E1	-1,982324124	-2,186086603	0,203762479
1558949_at	TNRC18	-1,982324124	-2,186086603	0,203762479
215480_at	KIAA0509	-1,982324124	-2,186086603	0,203762479
231091_x_at	-	-1,982324124	-2,186086603	0,203762479
216994_s_at	RUNX2	-1,982324124	-2,186086603	0,203762479
1558608_a_at	PAPPA	-1,982324124	-2,186086603	0,203762479
222910_s_at	PEX5L	-1,982324124	-2,186086603	0,203762479
242978_x_at	-	1,960819106	1,757155322	0,203663785
204529_s_at	TOX	4,183891989	3,980593719	0,20329827
1560988_a_at	LINC00556	-1,893506789	-2,09678811	0,203281321
207775_at	MGC4859	-1,893506789	-2,09678811	0,203281321
220930_s_at	C13orf44	-1,893506789	-2,09678811	0,203281321
233162_at	-	-1,893506789	-2,09678811	0,203281321
231160_at	-	-1,893506789	-2,09678811	0,203281321
1568805_at	NCOA7	-1,893506789	-2,09678811	0,203281321
1553293_at	MRGPRX3	-1,893506789	-2,09678811	0,203281321
210917_at	YES1	-1,893506789	-2,09678811	0,203281321
244170_at	RAB3C	-1,893506789	-2,09678811	0,203281321
207441_at	SMR3B	-1,893506789	-2,09678811	0,203281321
1565890_at	-	-1,893506789	-2,09678811	0,203281321
244510_at	-	-1,893506789	-2,09678811	0,203281321

1569826_at	-	-1,893506789	-2,09678811	0,203281321
226841_at	MPEG1	-1,893506789	-2,09678811	0,203281321
220317_at	LRAT	-1,893506789	-2,09678811	0,203281321
206522_at	MGAM	-1,893506789	-2,09678811	0,203281321
1566691_at	-	-1,893506789	-2,09678811	0,203281321
1560246_at	-	-1,893506789	-2,09678811	0,203281321
239732_x_at	FAM47C	-1,893506789	-2,09678811	0,203281321
237927_at	-	-1,893506789	-2,09678811	0,203281321
1556004_at	LOC100506777	-1,893506789	-2,09678811	0,203281321
226311_at	ADAMTS2	-1,893506789	-2,09678811	0,203281321
240419_at	SLC6A15	-1,893506789	-2,09678811	0,203281321
1557681_s_at	SAMD15	-1,893506789	-2,09678811	0,203281321
210173_at	PTPRJ	-1,893506789	-2,09678811	0,203281321
216494_at	-	-1,893506789	-2,09678811	0,203281321
213462_at	NPAS2	-1,893506789	-2,09678811	0,203281321
1559624_at	STK32A	-1,893506789	-2,09678811	0,203281321
215248_at	GRB10	-1,893506789	-2,09678811	0,203281321
1557514_a_at	-	-1,893506789	-2,09678811	0,203281321
244831_at	-	-1,893506789	-2,09678811	0,203281321
211442_x_at	CYP3A43	-1,893506789	-2,09678811	0,203281321
223816_at	SLC46A2	-1,893506789	-2,09678811	0,203281321
204284_at	PPP1R3C	-1,893506789	-2,09678811	0,203281321
1561006_at	-	-1,893506789	-2,09678811	0,203281321
228441_s_at	-	-1,893506789	-2,09678811	0,203281321
238003_at	HEPACAM /// HEPI	-1,893506789	-2,09678811	0,203281321
236440_at	NETO1	-1,893506789	-2,09678811	0,203281321
241563_at	-	-1,893506789	-2,09678811	0,203281321
216771_at	-	-1,893506789	-2,09678811	0,203281321
207709_at	PRKAA2	-1,893506789	-2,09678811	0,203281321
232887_at	PIRT	-1,893506789	-2,09678811	0,203281321
223550_s_at	CA10	-1,893506789	-2,09678811	0,203281321
227084_at	DTNA	-1,893506789	-2,09678811	0,203281321
201591_s_at	NISCH	3,713669011	3,510668356	0,203000654
233214_at	-	0,08619576	-0,116767475	0,202963235
204505_s_at	EPB49	0,08619576	-0,116767475	0,202963235
225217_s_at	BRPF3	0,08619576	-0,116767475	0,202963235
206487_at	SUN1	0,001114523	-0,201789721	0,202904244
236110_at	-	0,001114523	-0,201789721	0,202904244
215836_s_at	PCDHGA1 /// PCDI	0,001114523	-0,201789721	0,202904244
238655_at	ACAD10	0,001114523	-0,201789721	0,202904244
228239_at	FAM165B	0,900065578	0,697221375	0,202844203
223544_at	TMEM79	1,139057614	0,936232181	0,202825433
212784_at	CIC	1,139057614	0,936232181	0,202825433
224971_at	C2orf15 /// MRPL3	4,211840302	4,009037164	0,202803138
216071_x_at	MED12	1,788922638	1,586121802	0,202800836
201317_s_at	PSMA2	6,79503252	6,592256716	0,202775805
1554743_x_at	PMS1	-0,609040214	-0,811769547	0,202729333
1570230_at	-	-0,609040214	-0,811769547	0,202729333
232601_at	-	-0,609040214	-0,811769547	0,202729333
217365_at	PRAMEF11	-0,609040214	-0,811769547	0,202729333

244575_at	POLA2	-0,609040214	-0,811769547	0,202729333
235029_at	GINS4	0,425036312	0,222432814	0,202603498
216125_s_at	RANBP9	2,149438457	1,946903052	0,202535405
224585_x_at	ACTG1	8,452815917	8,250383361	0,202432556
232644_x_at	OCIAD1	3,102023286	2,899658146	0,20236514
231988_x_at	ZNF490	0,820160788	0,61779983	0,202360958
231781_s_at	LRRC2	-1,802716385	-2,005029581	0,202313196
1552933_at	AKNAD1	-1,802716385	-2,005029581	0,202313196
216724_at	DCLK2	-1,802716385	-2,005029581	0,202313196
1555603_at	BAGE	-1,802716385	-2,005029581	0,202313196
1556912_at	GIT2	-1,802716385	-2,005029581	0,202313196
1569072_s_at	ABCB5	-1,802716385	-2,005029581	0,202313196
1569061_at	IQGAP3	-1,802716385	-2,005029581	0,202313196
1562690_at	-	-1,802716385	-2,005029581	0,202313196
220869_at	UBA6	-1,802716385	-2,005029581	0,202313196
1560957_at	-	-1,802716385	-2,005029581	0,202313196
207241_at	C4orf6	-1,802716385	-2,005029581	0,202313196
237413_at	MAPK10	-1,802716385	-2,005029581	0,202313196
1563659_at	HERC6	-1,802716385	-2,005029581	0,202313196
1559309_at	MCFD2	-1,802716385	-2,005029581	0,202313196
1555403_a_at	CDH19	-1,802716385	-2,005029581	0,202313196
240935_at	-	-1,802716385	-2,005029581	0,202313196
1561055_at	LOC100507534	-1,802716385	-2,005029581	0,202313196
226498_at	FLT1	-1,802716385	-2,005029581	0,202313196
234261_at	-	-1,802716385	-2,005029581	0,202313196
1560727_at	HEATR4	-1,802716385	-2,005029581	0,202313196
230674_at	LGR4	-1,802716385	-2,005029581	0,202313196
1556856_at	-	-1,802716385	-2,005029581	0,202313196
237339_at	-	-1,802716385	-2,005029581	0,202313196
220184_at	NANOG	-1,802716385	-2,005029581	0,202313196
227048_at	LAMA1	-1,802716385	-2,005029581	0,202313196
216812_at	-	-1,802716385	-2,005029581	0,202313196
234443_at	-	-1,802716385	-2,005029581	0,202313196
1555722_at	SCAMPER	-1,802716385	-2,005029581	0,202313196
206163_at	MAB21L1	-1,802716385	-2,005029581	0,202313196
238306_at	-	-1,802716385	-2,005029581	0,202313196
242799_at	-	-1,802716385	-2,005029581	0,202313196
1567682_x_at	SNORA74A	-1,802716385	-2,005029581	0,202313196
1555774_at	ZAR1	-1,802716385	-2,005029581	0,202313196
244279_at	SOBP	-1,802716385	-2,005029581	0,202313196
1570474_s_at	LOC338579	-1,802716385	-2,005029581	0,202313196
207043_s_at	SLC6A9	-1,802716385	-2,005029581	0,202313196
226304_at	HSPB6	-1,802716385	-2,005029581	0,202313196
233581_at	-	-1,802716385	-2,005029581	0,202313196
236720_at	-	-1,802716385	-2,005029581	0,202313196
1561251_at	LOC285577	-1,802716385	-2,005029581	0,202313196
240380_at	LOC728040	-1,802716385	-2,005029581	0,202313196
231218_at	-	-1,802716385	-2,005029581	0,202313196
1561249_a_at	DNM1P35	-1,802716385	-2,005029581	0,202313196
210222_s_at	RTN1	-1,802716385	-2,005029581	0,202313196

238463_at	-	-1,802716385	-2,005029581	0,202313196
1559940_s_at	-	-1,802716385	-2,005029581	0,202313196
223170_at	TMEM98	-1,802716385	-2,005029581	0,202313196
221123_x_at	FBXO16 /// ZNF39!	0,58788177	0,385582632	0,202299139
208706_s_at	EIF5	4,429046471	4,226860102	0,202186369
221775_x_at	RPL22	8,176343309	7,974195164	0,202148145
222516_at	AP3M1	3,624986574	3,422991983	0,201994591
226131_s_at	RPS16	8,618353634	8,416389827	0,201963807
223946_at	MED23	-0,088974936	-0,290694975	0,20172004
238061_at	LGI3	-0,088974936	-0,290694975	0,20172004
229134_at	VANGL1	-0,088974936	-0,290694975	0,20172004
234877_x_at	-	-0,088974936	-0,290694975	0,20172004
1569144_a_at	C9orf169	-0,088974936	-0,290694975	0,20172004
218569_s_at	KBTBD4	1,802733148	1,601076277	0,201656871
202921_s_at	ANK2	-0,730013898	-0,931539147	0,201525249
225873_at	TYSND1	-0,730013898	-0,931539147	0,201525249
241646_s_at	BCAP29	-0,730013898	-0,931539147	0,201525249
243370_at	CAPRIN1	-0,730013898	-0,931539147	0,201525249
1552798_a_at	TLR4	-0,730013898	-0,931539147	0,201525249
1559982_s_at	AKR1E2	-0,730013898	-0,931539147	0,201525249
238487_at	GNL1	-0,730013898	-0,931539147	0,201525249
240818_at	-	-0,730013898	-0,931539147	0,201525249
229392_s_at	PIK3R2	-0,730013898	-0,931539147	0,201525249
214678_x_at	ZFX	1,701542258	1,500059916	0,201482341
232262_at	PIGL	-0,856259172	-1,057301851	0,201042679
216402_at	SEC14L4	-0,856259172	-1,057301851	0,201042679
215169_at	SLC35E2	-0,856259172	-1,057301851	0,201042679
1552924_a_at	PITPNM2	-0,856259172	-1,057301851	0,201042679
241215_at	-	-0,856259172	-1,057301851	0,201042679
229185_at	LIFR	-0,856259172	-1,057301851	0,201042679
210324_at	C8G	-0,856259172	-1,057301851	0,201042679
239677_at	-	-0,856259172	-1,057301851	0,201042679
224283_x_at	IL18BP	-0,856259172	-1,057301851	0,201042679
207767_s_at	EGR4	-0,856259172	-1,057301851	0,201042679
1555483_x_at	FBLIM1	-0,856259172	-1,057301851	0,201042679
230985_at	C9orf131	-0,856259172	-1,057301851	0,201042679
240547_at	-	-0,856259172	-1,057301851	0,201042679
230818_at	-	-0,856259172	-1,057301851	0,201042679
209951_s_at	MAP2K7	-0,856259172	-1,057301851	0,201042679
1554404_a_at	-	-0,856259172	-1,057301851	0,201042679
1553657_at	VWA3A	-0,856259172	-1,057301851	0,201042679
229439_s_at	RBM47	-0,856259172	-1,057301851	0,201042679
236630_at	AQP2	-0,856259172	-1,057301851	0,201042679
200816_s_at	PAFAH1B1	4,228647054	4,027690404	0,20095665
221219_s_at	KLHDC4 /// LOC10	2,460890032	2,260052727	0,200837305
207186_s_at	BPTF /// LOC1468!	4,070902947	3,870131307	0,20077164
41858_at	PGAP2	2,580050689	2,379293262	0,200757428
213801_x_at	RPSA /// RPSAP19	8,643513143	8,442795268	0,200717876
212733_at	KIAA0226	2,798156197	2,597466271	0,200689926
242033_at	RNF180	-2,495152701	-2,695640852	0,200488151

228692_at	PREX2	-2,495152701	-2,695640852	0,200488151
1561564_at	-	-2,495152701	-2,695640852	0,200488151
1568487_x_at	-	-2,495152701	-2,695640852	0,200488151
243734_x_at	-	-2,495152701	-2,695640852	0,200488151
233821_at	-	-2,495152701	-2,695640852	0,200488151
1563186_at	-	-2,495152701	-2,695640852	0,200488151
1560996_at	-	-2,495152701	-2,695640852	0,200488151
205523_at	HAPLN1	-2,495152701	-2,695640852	0,200488151
216839_at	LAMA2	-2,495152701	-2,695640852	0,200488151
216258_s_at	SERPINB13	-2,495152701	-2,695640852	0,200488151
240480_at	TINAG	-2,495152701	-2,695640852	0,200488151
1556026_at	LOC100131434	-2,495152701	-2,695640852	0,200488151
1561383_at	LOC284661	-2,495152701	-2,695640852	0,200488151
207120_at	ZNF667	-2,495152701	-2,695640852	0,200488151
1556187_at	ZNF555	-2,495152701	-2,695640852	0,200488151
201617_x_at	CALD1	-2,495152701	-2,695640852	0,200488151
1559814_at	-	-2,495152701	-2,695640852	0,200488151
1557456_a_at	-	-2,495152701	-2,695640852	0,200488151
226259_at	EXOC6	2,074841895	1,874839058	0,200002837
225170_at	WDR5	1,936380191	1,736450458	0,199929733
220786_s_at	SLC38A4	-2,336678463	-2,536339282	0,199660818
220700_at	-	-2,336678463	-2,536339282	0,199660818
240751_at	-	-2,336678463	-2,536339282	0,199660818
1561151_a_at	-	-2,336678463	-2,536339282	0,199660818
1563797_at	-	-2,336678463	-2,536339282	0,199660818
226256_at	COPB2	-2,336678463	-2,536339282	0,199660818
1559285_at	-	-2,336678463	-2,536339282	0,199660818
215117_at	RAG2	-2,336678463	-2,536339282	0,199660818
242792_at	-	-2,336678463	-2,536339282	0,199660818
1565936_a_at	LMO3	-2,336678463	-2,536339282	0,199660818
1563254_a_at	LOC100506733	-2,336678463	-2,536339282	0,199660818
240847_at	-	-2,336678463	-2,536339282	0,199660818
1561123_at	-	-2,336678463	-2,536339282	0,199660818
1570395_a_at	FAM66C	-2,336678463	-2,536339282	0,199660818
241257_at	-	-2,336678463	-2,536339282	0,199660818
220540_at	KCNK15	-2,336678463	-2,536339282	0,199660818
1569840_at	-	-2,336678463	-2,536339282	0,199660818
1554874_at	MITF	-2,336678463	-2,536339282	0,199660818
205029_s_at	FABP7	-2,336678463	-2,536339282	0,199660818
1556706_at	-	-2,336678463	-2,536339282	0,199660818
240741_x_at	-	-2,336678463	-2,536339282	0,199660818
1562634_at	-	-2,336678463	-2,536339282	0,199660818
1561718_at	-	-2,336678463	-2,536339282	0,199660818
1560903_at	-	-2,336678463	-2,536339282	0,199660818
1553524_at	DGKB	-2,336678463	-2,536339282	0,199660818
1561577_at	-	-2,336678463	-2,536339282	0,199660818
213662_at	-	-2,336678463	-2,536339282	0,199660818
232771_at	NRK	-2,336678463	-2,536339282	0,199660818
1559377_at	-	-2,336678463	-2,536339282	0,199660818
205931_s_at	CREB5 /// LOC401	-2,336678463	-2,536339282	0,199660818

1558375_at	LRRC38	-2,336678463	-2,536339282	0,199660818
232233_at	SLC22A16	-2,336678463	-2,536339282	0,199660818
240489_at	-	-2,336678463	-2,536339282	0,199660818
1564635_a_at	FHAD1	-2,336678463	-2,536339282	0,199660818
1552424_at	KLHL10	-2,336678463	-2,536339282	0,199660818
203548_s_at	LPL	-2,336678463	-2,536339282	0,199660818
202081_at	IER2	3,77412549	3,5744736	0,19965189
221812_at	FBXO42	1,345611839	1,146010634	0,199601205
225206_s_at	MTRF1L	1,345611839	1,146010634	0,199601205
207243_s_at	CALM1 /// CALM2	7,732809296	7,533456914	0,199352381
230492_s_at	GPCPD1	0,926325262	0,727123109	0,199202153
212373_at	FEM1B	0,926325262	0,727123109	0,199202153
203621_at	NDUFB5	5,990294152	5,791148102	0,19914605
204757_s_at	C2CD2L	1,182925501	0,98381571	0,199109791
213535_s_at	UBE2I	5,068598441	4,869549729	0,199048712
228373_at	C16orf72	2,401508182	2,20251271	0,198995472
228207_at	LOC100499489	0,677745787	0,479089184	0,198656603
233178_at	TGIF2LY	-1,369772723	-1,568385657	0,198612934
241211_at	-	-1,369772723	-1,568385657	0,198612934
220876_at	-	-1,369772723	-1,568385657	0,198612934
237153_at	-	-1,369772723	-1,568385657	0,198612934
227673_at	ZNRD1	-1,369772723	-1,568385657	0,198612934
1559481_at	CHIC1	-1,369772723	-1,568385657	0,198612934
234900_at	-	-1,369772723	-1,568385657	0,198612934
244718_at	MTMR9LP	-1,369772723	-1,568385657	0,198612934
225759_x_at	CLMN	-1,369772723	-1,568385657	0,198612934
237452_at	-	-1,369772723	-1,568385657	0,198612934
243334_at	CACNA1D	-1,369772723	-1,568385657	0,198612934
1567065_at	OR1Q1	-1,369772723	-1,568385657	0,198612934
214524_at	GHRH	-1,369772723	-1,568385657	0,198612934
204988_at	FGB	-1,369772723	-1,568385657	0,198612934
219630_at	PDZK1IP1	-1,369772723	-1,568385657	0,198612934
219721_at	-	-1,369772723	-1,568385657	0,198612934
234297_at	RGS8 /// SDHAP3	-1,369772723	-1,568385657	0,198612934
232783_at	-	-1,369772723	-1,568385657	0,198612934
207146_at	KRT32	-1,369772723	-1,568385657	0,198612934
240454_at	-	-1,369772723	-1,568385657	0,198612934
231176_at	PRR19	-1,369772723	-1,568385657	0,198612934
205177_at	TNNI1	-1,369772723	-1,568385657	0,198612934
1569001_at	BMP1	-1,369772723	-1,568385657	0,198612934
204597_x_at	STC1	-1,369772723	-1,568385657	0,198612934
233816_at	-	-1,369772723	-1,568385657	0,198612934
1558113_at	FAM78B	-1,369772723	-1,568385657	0,198612934
237726_at	-	-1,369772723	-1,568385657	0,198612934
220051_at	PRSS21	-1,369772723	-1,568385657	0,198612934
218950_at	ARAP3	-1,369772723	-1,568385657	0,198612934
234907_x_at	POLB	-1,712732543	-1,911206569	0,198474027
240216_at	-	-1,712732543	-1,911206569	0,198474027
241598_at	NUDT10	-1,712732543	-1,911206569	0,198474027
242334_at	NLRP4	-1,712732543	-1,911206569	0,198474027

237517_at	-	-1,712732543	-1,911206569	0,198474027
1559348_a_at	LOC100507568	-1,712732543	-1,911206569	0,198474027
211428_at	SERPINA1	-1,712732543	-1,911206569	0,198474027
1556456_at	FLJ39739	-1,712732543	-1,911206569	0,198474027
1555092_at	VASH2	-1,712732543	-1,911206569	0,198474027
1552982_a_at	FGF4	-1,712732543	-1,911206569	0,198474027
211820_x_at	GYPA	-1,712732543	-1,911206569	0,198474027
1555456_at	-	-1,712732543	-1,911206569	0,198474027
1562604_at	-	-1,712732543	-1,911206569	0,198474027
220664_at	SPRR2C	-1,712732543	-1,911206569	0,198474027
233544_at	GNL1	-1,712732543	-1,911206569	0,198474027
234545_at	OR14J1	-1,712732543	-1,911206569	0,198474027
206333_at	MSI1	-1,712732543	-1,911206569	0,198474027
1556837_a_at	-	-1,712732543	-1,911206569	0,198474027
238658_at	-	-1,712732543	-1,911206569	0,198474027
1556496_a_at	-	-1,712732543	-1,911206569	0,198474027
1566842_at	-	-1,712732543	-1,911206569	0,198474027
235166_at	ZNF148	-1,712732543	-1,911206569	0,198474027
224075_s_at	VSX1	-1,712732543	-1,911206569	0,198474027
236100_at	FBXO10	-1,712732543	-1,911206569	0,198474027
211829_s_at	GPER	-1,712732543	-1,911206569	0,198474027
237612_at	-	-1,712732543	-1,911206569	0,198474027
210550_s_at	RASGRF1	-1,712732543	-1,911206569	0,198474027
241199_x_at	DPPA4	-1,712732543	-1,911206569	0,198474027
210656_at	EED	-1,712732543	-1,911206569	0,198474027
224119_at	-	-1,712732543	-1,911206569	0,198474027
240593_x_at	-	-1,712732543	-1,911206569	0,198474027
243340_at	-	-1,712732543	-1,911206569	0,198474027
1570635_at	-	-1,712732543	-1,911206569	0,198474027
231180_at	LOC100506591	-1,712732543	-1,911206569	0,198474027
204335_at	CCDC94	1,857989965	1,659532492	0,198457474
44146_at	GMEB2	2,388715083	2,190458663	0,19825642
219053_s_at	VPS37C	2,476788561	2,278572464	0,198216097
226314_at	CHST14	1,578719607	1,380552339	0,198167268
224917_at	MIR21	3,657457797	3,459443789	0,198014007
227932_at	ARIH2	2,575362572	2,37755543	0,197807142
209008_x_at	KRT8	0,524545436	0,326777209	0,197768227
220488_s_at	BCAS3	0,524545436	0,326777209	0,197768227
224689_at	MANBAL	2,914752575	2,717094254	0,197658321
224617_at	PTBP3	5,442088257	5,244521252	0,197567005
223418_x_at	ANKRD13C	2,791258167	2,593698508	0,197559659
215009_s_at	THAP9-AS1	2,452674045	2,255165935	0,19750811
236250_at	AFG3L1P	0,847529938	0,65030602	0,197223918
217751_at	GSTK1	4,381466431	4,184570502	0,19689593
229753_at	POU2F1	0,282358733	0,085534992	0,196823741
240615_at	PTOV1-AS1	-0,994186487	-1,19085694	0,196670453
235704_at	DAZAP2	-0,994186487	-1,19085694	0,196670453
1566403_at	SNORA68	-0,994186487	-1,19085694	0,196670453
222050_at	-	-0,994186487	-1,19085694	0,196670453
217628_at	CLIC5	-0,994186487	-1,19085694	0,196670453

221128_at	ADAM19	-0,994186487	-1,19085694	0,196670453
231264_at	TMCO2	-0,994186487	-1,19085694	0,196670453
221148_at	-	-0,994186487	-1,19085694	0,196670453
1552436_a_at	CDH23 /// LOC100	-0,994186487	-1,19085694	0,196670453
215161_at	CAMK1G	-0,994186487	-1,19085694	0,196670453
228430_at	BOLA3-AS1	-0,994186487	-1,19085694	0,196670453
215610_at	ALG1L2	-0,994186487	-1,19085694	0,196670453
209097_s_at	JAG1	-0,994186487	-1,19085694	0,196670453
1561439_at	-	-0,994186487	-1,19085694	0,196670453
238629_x_at	-	-0,994186487	-1,19085694	0,196670453
226855_at	PDP2	1,364555912	1,167919222	0,19663669
200780_x_at	GNAS	7,017419652	6,821003485	0,196416168
225634_at	ZC3HAV1	4,082216372	3,885830539	0,196385834
1556889_s_at	-	-2,708365344	-2,904470894	0,19610555
1554700_at	CDH7	-2,708365344	-2,904470894	0,19610555
211630_s_at	GSS	3,394274216	3,198229785	0,196044431
227488_at	MGC16121 /// MI	0,764143511	0,568155442	0,195988069
59375_at	MYO15B	-0,204050947	-0,399982787	0,19593184
216974_at	-	-2,774503732	-2,970378591	0,195874858
204036_at	LPAR1	-2,074223907	-2,27001641	0,195792503
207481_at	-	-2,074223907	-2,27001641	0,195792503
210492_at	MFAP3L	-2,074223907	-2,27001641	0,195792503
211567_at	-	-2,074223907	-2,27001641	0,195792503
234081_at	-	-2,074223907	-2,27001641	0,195792503
243737_at	ATP1B4	-2,074223907	-2,27001641	0,195792503
1564610_at	-	-2,074223907	-2,27001641	0,195792503
242164_s_at	LRIG2	-2,074223907	-2,27001641	0,195792503
1561877_at	-	-2,074223907	-2,27001641	0,195792503
1566644_at	-	-2,074223907	-2,27001641	0,195792503
1555368_x_at	ZNF479	-2,074223907	-2,27001641	0,195792503
237549_at	-	-2,074223907	-2,27001641	0,195792503
1566040_at	-	-2,074223907	-2,27001641	0,195792503
226913_s_at	SOX8	-2,074223907	-2,27001641	0,195792503
237889_s_at	LOC100422737	-2,074223907	-2,27001641	0,195792503
234497_s_at	-	-2,074223907	-2,27001641	0,195792503
233740_at	-	-2,074223907	-2,27001641	0,195792503
1554403_a_at	-	-2,074223907	-2,27001641	0,195792503
1559789_a_at	ZNF781	-2,074223907	-2,27001641	0,195792503
1554558_at	DCAF5	-2,074223907	-2,27001641	0,195792503
244888_at	-	-2,074223907	-2,27001641	0,195792503
233130_at	-	-2,074223907	-2,27001641	0,195792503
210558_at	AKR1C4	-2,074223907	-2,27001641	0,195792503
1562296_at	-	-2,074223907	-2,27001641	0,195792503
1569971_at	LOC100288721	-2,074223907	-2,27001641	0,195792503
242196_at	ARHGAP32	-2,074223907	-2,27001641	0,195792503
215790_at	AJAP1	-2,074223907	-2,27001641	0,195792503
241656_at	-	-2,074223907	-2,27001641	0,195792503
224102_at	P2RY12	-2,074223907	-2,27001641	0,195792503
210435_at	ARL17A /// ARL17	-2,074223907	-2,27001641	0,195792503
1557881_at	ZNF32-AS3	-2,074223907	-2,27001641	0,195792503

217050_at	EPAG	-2,074223907	-2,27001641	0,195792503
222178_s_at	-	-2,074223907	-2,27001641	0,195792503
1553834_at	COL6A5	-2,074223907	-2,27001641	0,195792503
232074_at	PRSS27	-2,074223907	-2,27001641	0,195792503
211877_s_at	PCDHGA11 /// PCI	-2,074223907	-2,27001641	0,195792503
241026_at	-	-2,074223907	-2,27001641	0,195792503
236360_at	FLJ42875	-2,074223907	-2,27001641	0,195792503
1559691_at	-	-2,074223907	-2,27001641	0,195792503
211878_at	-	-2,074223907	-2,27001641	0,195792503
207906_at	IL3	-2,074223907	-2,27001641	0,195792503
205183_at	-	-2,074223907	-2,27001641	0,195792503
241319_at	-	-2,074223907	-2,27001641	0,195792503
202946_s_at	BTBD3	-2,074223907	-2,27001641	0,195792503
211149_at	UTY	-2,074223907	-2,27001641	0,195792503
1567009_at	-	-2,074223907	-2,27001641	0,195792503
1564721_at	-	-2,074223907	-2,27001641	0,195792503
1563970_at	MRPS27	-2,074223907	-2,27001641	0,195792503
1569355_at	KCNIP3	-2,074223907	-2,27001641	0,195792503
224197_s_at	C1QTNF1	-2,074223907	-2,27001641	0,195792503
223567_at	SEMA6B	-2,074223907	-2,27001641	0,195792503
237837_at	-	-2,074223907	-2,27001641	0,195792503
221029_s_at	WNT5B	-2,074223907	-2,27001641	0,195792503
224461_s_at	AIFM2	1,20396005	1,008210238	0,195749812
227093_at	USP36	2,019346222	1,823960141	0,195386081
226426_at	ADNP	2,784898907	2,589579418	0,195319489
201204_s_at	RRBP1	1,493647653	1,298349073	0,19529858
203211_s_at	MTMR2	4,033407967	3,838267489	0,195140478
224812_at	HIBADH	2,972517974	2,777528602	0,194989372
218243_at	RUFY1	3,336134047	3,141266133	0,194867914
201170_s_at	BHLHE40	5,191320822	4,99665521	0,194665612
206194_at	HOXC4	0,95222556	0,757638486	0,194587074
219817_at	MAPKAPK5-AS1	2,321828846	2,127337555	0,194491292
200802_at	SARS	5,195433974	5,001026816	0,194407158
203550_s_at	FAM189B	2,2388565	2,044580955	0,194275545
212634_at	UFL1	2,978070706	2,783936118	0,194134588
200656_s_at	P4HB	3,77412549	3,580129335	0,193996155
200719_at	SKP1	2,209870035	2,016139502	0,193730533
1554145_a_at	PPP1R21	2,410823487	2,2171284	0,193695086
209453_at	SLC9A1	0,618277321	0,424693306	0,193584015
1554021_a_at	ZNF12	1,802733148	1,60918327	0,193549877
1552484_at	SMCR8	0,458905032	0,265475485	0,193429547
209154_at	P2RX5-TAX1BP3 //	3,951538845	3,758449036	0,19308981
219599_at	EIF4B /// LOC1006	3,086998005	2,894109605	0,192888399
227593_at	FLJ37453	3,018305638	2,825679048	0,19262659
219052_at	HPS6	1,716003175	1,523429058	0,192574116
227825_at	NAIF1	1,402495885	1,209973561	0,192522323
224369_s_at	FBXO38	3,621852128	3,429874957	0,191977171
219390_at	FKBP14	0,873789652	0,681874787	0,191914865
212303_x_at	KHSRP	0,873789652	0,681874787	0,191914865
227598_at	C7orf29	0,791908897	0,600385088	0,191523809

204206_at	MNT	3,127551022	2,936370826	0,191180195
228138_at	ZNF498	1,440265662	1,249194639	0,191071023
210458_s_at	TANK	1,995527416	1,804519951	0,191007466
201890_at	RRM2	6,812092549	6,621214875	0,190877674
203416_at	CD53	5,595764877	5,404914562	0,190850315
1555364_at	C11orf58	-2,641232718	-2,832036647	0,190803929
1567246_at	OR5H1	-2,641232718	-2,832036647	0,190803929
241760_x_at	RORA	-2,641232718	-2,832036647	0,190803929
1565228_s_at	ALB	-2,641232718	-2,832036647	0,190803929
1566288_at	OR2M4	-2,641232718	-2,832036647	0,190803929
222498_at	AZI2	2,961244348	2,770696222	0,190548125
230298_at	MBLAC2	3,470020347	3,279515225	0,190505121
228500_at	THAP8	0,39105295	0,20062106	0,190431891
224863_at	GNAQ	0,556428218	0,366031214	0,190397004
208237_x_at	ADAM22	0,556428218	0,366031214	0,190397004
235128_at	SYNPO	0,045517965	-0,144765583	0,190283548
204259_at	MMP7	-1,137208348	-1,32740143	0,190193082
1559544_s_at	LINC00441	-1,137208348	-1,32740143	0,190193082
240652_at	-	-1,137208348	-1,32740143	0,190193082
232955_at	FLJ41170	-1,137208348	-1,32740143	0,190193082
207652_s_at	CMKLR1	-1,137208348	-1,32740143	0,190193082
1552569_a_at	RTP3	-1,137208348	-1,32740143	0,190193082
234239_at	-	-1,137208348	-1,32740143	0,190193082
241854_at	DNASE1	-1,137208348	-1,32740143	0,190193082
234919_s_at	SNTG1	-1,137208348	-1,32740143	0,190193082
242959_at	-	-1,137208348	-1,32740143	0,190193082
205744_at	DOC2A	-1,137208348	-1,32740143	0,190193082
242367_at	-	-1,137208348	-1,32740143	0,190193082
209991_x_at	GABBR2	-1,137208348	-1,32740143	0,190193082
207537_at	PFKFB1	-1,137208348	-1,32740143	0,190193082
241377_s_at	MIB2	-1,137208348	-1,32740143	0,190193082
219819_s_at	MRPS28	4,458703813	4,268613406	0,190090407
222907_x_at	TMEM50B	2,321828846	2,132145748	0,189683098
213514_s_at	DIAPH1	1,960819106	1,771157334	0,189661772
235390_at	SREK1IP1	2,356619952	2,167793428	0,188826524
205084_at	BCAP29	2,33082239	2,142313983	0,188508407
225209_s_at	UBE2J2	4,087244545	3,898781056	0,188463489
212711_at	CAMSAP1	3,023747815	2,835336991	0,188410824
227546_x_at	AURKAIP1	3,237994092	3,049587049	0,188407042
229582_at	INO80C	2,276736537	2,088449486	0,188287051
218226_s_at	NDUFB4	6,889369304	6,701173367	0,188195937
202606_s_at	TLK1	2,884644843	2,696595318	0,188049525
233558_s_at	TRAPPC11	2,62869474	2,440833194	0,187861546
201013_s_at	PAICS	5,626536098	5,438709741	0,187826357
229589_x_at	BIVM	-0,230432956	-0,418248858	0,187815902
230534_at	ZNF678	-0,230432956	-0,418248858	0,187815902
244222_at	-	-0,230432956	-0,418248858	0,187815902
226984_at	FGD5	-0,230432956	-0,418248858	0,187815902
204666_s_at	SIKE1	-0,230432956	-0,418248858	0,187815902
219018_s_at	CCDC85C	-0,230432956	-0,418248858	0,187815902

205119_s_at	FPR1	-0,230432956	-0,418248858	0,187815902
238679_at	MESDC2	-0,230432956	-0,418248858	0,187815902
217798_at	CNOT2	3,555373205	3,367769667	0,187603538
215631_s_at	BRMS1	3,081633533	2,894109605	0,187523927
241147_at	-	-2,163157732	-2,350444759	0,187287028
1554492_at	THADA	-2,163157732	-2,350444759	0,187287028
1559992_a_at	LOC100505967	-2,163157732	-2,350444759	0,187287028
1569755_at	LINC00276	-2,163157732	-2,350444759	0,187287028
231755_at	IL36B	-2,163157732	-2,350444759	0,187287028
241712_at	CBY3	-2,163157732	-2,350444759	0,187287028
242893_at	LOC100506267	-2,163157732	-2,350444759	0,187287028
1566203_at	-	-2,163157732	-2,350444759	0,187287028
237989_at	IGFBP1	-2,163157732	-2,350444759	0,187287028
1564285_at	HFM1	-2,163157732	-2,350444759	0,187287028
239503_at	C10orf114	-2,163157732	-2,350444759	0,187287028
1552522_at	TIGD4	-2,163157732	-2,350444759	0,187287028
1567664_at	-	-2,163157732	-2,350444759	0,187287028
1555217_at	UBE2W	-2,163157732	-2,350444759	0,187287028
208282_x_at	DAZ1 /// DAZ2 ///	-2,163157732	-2,350444759	0,187287028
223500_at	CPLX1	-2,163157732	-2,350444759	0,187287028
234556_at	-	-2,163157732	-2,350444759	0,187287028
1560111_at	-	-2,163157732	-2,350444759	0,187287028
1556167_at	HEATR7B1	-2,163157732	-2,350444759	0,187287028
1560643_x_at	-	-2,163157732	-2,350444759	0,187287028
231029_at	F5	-2,163157732	-2,350444759	0,187287028
1562338_at	01.03.15	-2,163157732	-2,350444759	0,187287028
1565107_x_at	-	-2,163157732	-2,350444759	0,187287028
204726_at	CDH13	-2,163157732	-2,350444759	0,187287028
216408_at	OR2B2	-2,163157732	-2,350444759	0,187287028
237828_at	SRRM4	-2,163157732	-2,350444759	0,187287028
217623_at	MYLK3	-2,163157732	-2,350444759	0,187287028
1562919_at	FAM45A	-2,163157732	-2,350444759	0,187287028
1564000_at	ANKRD31	-2,163157732	-2,350444759	0,187287028
219414_at	CLSTN2	-2,163157732	-2,350444759	0,187287028
215077_at	-	-2,163157732	-2,350444759	0,187287028
241005_at	-	-2,163157732	-2,350444759	0,187287028
211861_x_at	CD28	-2,163157732	-2,350444759	0,187287028
240790_at	-	-2,163157732	-2,350444759	0,187287028
241232_x_at	-	-2,163157732	-2,350444759	0,187287028
1566204_at	-	-2,163157732	-2,350444759	0,187287028
211491_at	ADRA1A	-2,163157732	-2,350444759	0,187287028
1555230_a_at	KCNIP2	-2,163157732	-2,350444759	0,187287028
1559573_at	LOC100506229	-2,163157732	-2,350444759	0,187287028
200088_x_at	LOC648771 /// NB	8,343367335	8,156081132	0,187286203
225109_at	OGFOD1	-0,135065865	-0,322340048	0,187274183
217588_at	CATSPER2 /// CATS	-0,135065865	-0,322340048	0,187274183
216571_at	-	-0,135065865	-0,322340048	0,187274183
224176_s_at	AXIN2	-0,135065865	-0,322340048	0,187274183
206722_s_at	LPAR2	-0,135065865	-0,322340048	0,187274183
201556_s_at	VAMP2	-0,135065865	-0,322340048	0,187274183

216207_x_at	IGKC /// LOC6428:	-0,135065865	-0,322340048	0,187274183
203310_at	STXBP3	4,471846553	4,28465024	0,187196313
1569086_at	LOC100287177	-0,043925712	-0,231045907	0,187120195
204543_at	RAPGEF1	-0,043925712	-0,231045907	0,187120195
213671_s_at	MARS	5,332289905	5,145291217	0,186998688
232171_x_at	KLHDC4	1,686984721	1,500059916	0,186924804
53720_at	C19orf66	1,738299065	1,551452052	0,186847014
229831_at	CNTN3	-2,416625063	-2,603471267	0,186846203
1557424_at	LOC100505878	-2,416625063	-2,603471267	0,186846203
1562396_at	-	-2,416625063	-2,603471267	0,186846203
1558388_a_at	UG0898H09	-2,416625063	-2,603471267	0,186846203
1561053_at	-	-2,416625063	-2,603471267	0,186846203
240713_s_at	-	-2,416625063	-2,603471267	0,186846203
1553799_at	FAM227B	-2,416625063	-2,603471267	0,186846203
237123_x_at	KLHL9	-2,416625063	-2,603471267	0,186846203
242513_x_at	KIAA2018	-2,416625063	-2,603471267	0,186846203
1553474_at	LOC100288966 ///	-2,416625063	-2,603471267	0,186846203
234583_at	CHL1	-2,416625063	-2,603471267	0,186846203
217528_at	CLCA2	-2,416625063	-2,603471267	0,186846203
232804_at	LOC100506385	-2,416625063	-2,603471267	0,186846203
1567859_at	-	-2,416625063	-2,603471267	0,186846203
230876_at	ZNF883	-2,416625063	-2,603471267	0,186846203
236234_at	PDE1A	-2,416625063	-2,603471267	0,186846203
231551_at	LOC729059	-2,416625063	-2,603471267	0,186846203
237528_at	-	-2,416625063	-2,603471267	0,186846203
209672_s_at	MIOS	-2,416625063	-2,603471267	0,186846203
1565105_at	-	-2,416625063	-2,603471267	0,186846203
212775_at	OBSL1	-2,416625063	-2,603471267	0,186846203
240133_x_at	-	-2,416625063	-2,603471267	0,186846203
215892_at	ZNF440	-2,416625063	-2,603471267	0,186846203
215074_at	MYO1B	-2,416625063	-2,603471267	0,186846203
242127_at	-	-2,416625063	-2,603471267	0,186846203
240218_at	DSCAM	-2,416625063	-2,603471267	0,186846203
243742_at	LOC100505797	-2,416625063	-2,603471267	0,186846203
235746_s_at	PLA2R1	-2,416625063	-2,603471267	0,186846203
219908_at	DKK2	-2,416625063	-2,603471267	0,186846203
1552457_a_at	ADAMTSL1	-1,624955693	-1,811780055	0,186824362
1567223_at	HMGA2	-1,624955693	-1,811780055	0,186824362
213714_at	CACNB2	-1,624955693	-1,811780055	0,186824362
1553355_at	-	-1,624955693	-1,811780055	0,186824362
233457_at	-	-1,624955693	-1,811780055	0,186824362
234613_at	-	-1,624955693	-1,811780055	0,186824362
206566_at	SLC7A1	-1,624955693	-1,811780055	0,186824362
244282_at	-	-1,624955693	-1,811780055	0,186824362
244628_at	-	-1,624955693	-1,811780055	0,186824362
210699_at	-	-1,624955693	-1,811780055	0,186824362
1562499_at	-	-1,624955693	-1,811780055	0,186824362
215234_at	-	-1,624955693	-1,811780055	0,186824362
229400_at	HOXD10	-1,624955693	-1,811780055	0,186824362
217237_at	-	-1,624955693	-1,811780055	0,186824362

226526_s_at	CKAP4	-1,624955693	-1,811780055	0,186824362
238008_at	PRR18	-1,624955693	-1,811780055	0,186824362
239066_at	LOC100506966	-1,624955693	-1,811780055	0,186824362
231687_at	-	-1,624955693	-1,811780055	0,186824362
237220_at	-	-1,624955693	-1,811780055	0,186824362
229214_at	-	-1,624955693	-1,811780055	0,186824362
1552973_at	WNT9B	-1,624955693	-1,811780055	0,186824362
1562979_at	-	-1,624955693	-1,811780055	0,186824362
221927_s_at	ABHD11	2,460890032	2,274084398	0,186805634
222403_at	MTCH2	2,765070895	2,578283758	0,186787137
238436_s_at	ZNF805	1,493647653	1,306903969	0,186743684
223188_at	SMG9	1,92317941	1,736450458	0,186728953
213024_at	TMF1	2,374261973	2,187622928	0,186639045
227920_at	BEND3	1,182925501	0,996453274	0,186472228
200886_s_at	PGAM1	6,90307769	6,716676026	0,186401663
235327_x_at	UBXN2A	4,698307213	4,511971409	0,186335804
235036_at	LIX1L	0,319287178	0,133104519	0,186182659
225878_at	KIF1B	2,896717728	2,710570973	0,186146755
230300_at	PSMA5	1,287793882	1,101780212	0,186013671
228345_at	CHIC1	1,287793882	1,101780212	0,186013671
218046_s_at	LOC100652993 ///	4,138272593	3,952350674	0,185921918
223428_s_at	ISY1	1,936380191	1,750483134	0,185897057
239678_at	-	0,820160788	0,634301128	0,18585966
202652_at	APBB1	-0,332405896	-0,518232988	0,185827092
242556_at	-	-0,332405896	-0,518232988	0,185827092
1561624_at	-	-0,437449947	-0,623254098	0,185804151
239181_at	-	-0,437449947	-0,623254098	0,185804151
241084_x_at	DYNC1H1	-0,437449947	-0,623254098	0,185804151
234528_at	-	-0,437449947	-0,623254098	0,185804151
225423_x_at	MRPL41	-0,437449947	-0,623254098	0,185804151
1569206_at	TCP11L2	-0,437449947	-0,623254098	0,185804151
235787_at	CDC37L1	0,491906512	0,306454867	0,185451645
207721_x_at	HINT1	7,45344403	7,268077703	0,185366327
207949_s_at	ICA1	1,000459215	0,815200271	0,185258944
202576_s_at	DDX19A /// DDX19	3,668220666	3,483950333	0,184270333
227246_at	PLRG1	1,383446753	1,199296812	0,18414994
227869_at	FAM104B /// LOC1	1,610353504	1,426280426	0,184073078
219439_at	C1GALT1	2,159837409	1,975797592	0,184039817
214197_s_at	SETDB1	1,094118704	0,910483921	0,183634783
56919_at	WDR48	2,331103491	2,147595459	0,183508032
208669_s_at	EID1	4,699939684	4,516440716	0,183498968
226478_at	TM7SF3	1,20396005	1,020514937	0,183445113
208615_s_at	PTP4A2	5,252085383	5,068805029	0,183280355
1554303_at	HNMT	-0,551284523	-0,734355396	0,183070873
216163_at	-	-0,551284523	-0,734355396	0,183070873
1558820_a_at	C18orf34	-0,551284523	-0,734355396	0,183070873
235097_at	TRMT44	-0,551284523	-0,734355396	0,183070873
219592_at	MCPH1	-0,551284523	-0,734355396	0,183070873
243355_at	LOC100131316	-0,551284523	-0,734355396	0,183070873
234054_at	LOC143188	-0,551284523	-0,734355396	0,183070873

223409_at	-	-0,551284523	-0,734355396	0,183070873
224557_x_at	P2RX2	-0,551284523	-0,734355396	0,183070873
216411_s_at	GALK2	-0,551284523	-0,734355396	0,183070873
217161_x_at	ACAN	-0,551284523	-0,734355396	0,183070873
236193_at	HIST1H2BC	0,244681185	0,061773582	0,182907603
222318_at	ZNF324B	0,244681185	0,061773582	0,182907603
211815_s_at	GGA3	0,244681185	0,061773582	0,182907603
238652_at	-	0,244681185	0,061773582	0,182907603
209182_s_at	C10orf10	0,244681185	0,061773582	0,182907603
1553883_at	ZNF99	-2,569309811	-2,752097204	0,182787392
1563465_at	PKD1L1	-2,569309811	-2,752097204	0,182787392
1561213_at	-	-2,569309811	-2,752097204	0,182787392
1552837_at	ICK	-2,569309811	-2,752097204	0,182787392
1562480_at	-	-2,569309811	-2,752097204	0,182787392
1566428_at	-	-2,569309811	-2,752097204	0,182787392
208175_s_at	DMP1	-2,569309811	-2,752097204	0,182787392
1561721_a_at	C12orf40	-2,569309811	-2,752097204	0,182787392
1553963_at	RHOB	-2,569309811	-2,752097204	0,182787392
1564965_at	-	-2,569309811	-2,752097204	0,182787392
208143_s_at	EDDM3A	-2,569309811	-2,752097204	0,182787392
214389_at	SLC5A12	-2,569309811	-2,752097204	0,182787392
1568871_at	-	-2,569309811	-2,752097204	0,182787392
208147_s_at	CYP2C8	-2,569309811	-2,752097204	0,182787392
1561290_at	LOC339622	-2,569309811	-2,752097204	0,182787392
206810_at	ZNF44	-2,569309811	-2,752097204	0,182787392
207029_at	KITLG	-2,569309811	-2,752097204	0,182787392
208195_at	TTN	1,625482993	1,44320939	0,182273603
218427_at	SDCCAG3	2,56794744	2,385765892	0,182181547
222297_x_at	-	1,898032047	1,715863686	0,182168362
227591_at	LOC100505696	0,58788177	0,405752839	0,182128932
210974_s_at	AP3D1	1,421391637	1,239268196	0,182123442
221286_s_at	MZB1	1,421391637	1,239268196	0,182123442
209392_at	ENPP2	1,421391637	1,239268196	0,182123442
225834_at	FAM72A /// FAM7	5,927618166	5,745523205	0,182094961
200013_at	RPL24	8,200813467	8,018767312	0,182046156
1552705_at	DUSP19	-1,289805289	-1,471833228	0,182027939
1569580_a_at	-	-1,289805289	-1,471833228	0,182027939
208515_at	HIST1H2BM	-1,289805289	-1,471833228	0,182027939
217351_at	-	-1,289805289	-1,471833228	0,182027939
1555804_a_at	YSK4	-1,289805289	-1,471833228	0,182027939
1561134_at	-	-1,289805289	-1,471833228	0,182027939
206320_s_at	SMAD9	-1,289805289	-1,471833228	0,182027939
1564423_a_at	SEC16B	-1,289805289	-1,471833228	0,182027939
207158_at	APOBEC1	-1,289805289	-1,471833228	0,182027939
231206_at	-	-1,289805289	-1,471833228	0,182027939
1562661_at	-	-1,289805289	-1,471833228	0,182027939
207138_at	PHF2	-1,289805289	-1,471833228	0,182027939
1563137_at	MGAT5B	-1,289805289	-1,471833228	0,182027939
206755_at	CYP2B6	-1,289805289	-1,471833228	0,182027939
224344_at	COX6A1	-1,289805289	-1,471833228	0,182027939

1552481_s_at	MAN1A2	-1,289805289	-1,471833228	0,182027939
228161_at	RAB32	-1,289805289	-1,471833228	0,182027939
217143_s_at	YME1L1	-1,289805289	-1,471833228	0,182027939
224051_at	-	-1,289805289	-1,471833228	0,182027939
232927_at	-	-1,289805289	-1,471833228	0,182027939
1561967_at	FLNB-AS1	-1,289805289	-1,471833228	0,182027939
242187_s_at	TNRC18	-1,289805289	-1,471833228	0,182027939
238166_s_at	C17orf51 /// FAM2	-1,289805289	-1,471833228	0,182027939
1559351_at	-	-1,289805289	-1,471833228	0,182027939
210037_s_at	NOS2	-1,289805289	-1,471833228	0,182027939
207832_at	BAIAP2	-1,289805289	-1,471833228	0,182027939
242606_at	-	-1,289805289	-1,471833228	0,182027939
208103_s_at	ANP32E	4,343795594	4,162193397	0,181602197
1557789_at	-	-0,791294935	-0,972893339	0,181598403
1554642_at	RNF32	-0,791294935	-0,972893339	0,181598403
232335_at	-	-0,791294935	-0,972893339	0,181598403
235169_at	FBXO27	-0,791294935	-0,972893339	0,181598403
1562778_at	FAM166A	-0,791294935	-0,972893339	0,181598403
1570627_at	TCEB3	-0,791294935	-0,972893339	0,181598403
239563_at	-	-0,791294935	-0,972893339	0,181598403
207770_x_at	CSH2	-0,791294935	-0,972893339	0,181598403
241480_at	RNF213	-0,791294935	-0,972893339	0,181598403
201615_x_at	CALD1	-0,791294935	-0,972893339	0,181598403
205546_s_at	TYK2	2,468978138	2,287674614	0,181303524
201029_s_at	CD99	5,383324257	5,202716986	0,180607271
219372_at	IFT81	1,326463531	1,146010634	0,180452897
222360_at	DPH5	0,425036312	0,244646697	0,180389615
1568691_at	-	0,677745787	0,497424714	0,180321073
225752_at	NIPA1	1,116462765	0,936232181	0,180230583
226965_at	FAM116A	3,243218364	3,063097042	0,180121322
201098_at	COPB2	5,030908502	4,850963371	0,179945131
230827_at	-	-0,66934516	-0,84928999	0,17994483
240684_at	-	-0,66934516	-0,84928999	0,17994483
216472_at	-	-0,66934516	-0,84928999	0,17994483
234031_at	C20orf112	-0,66934516	-0,84928999	0,17994483
234770_at	-	-0,66934516	-0,84928999	0,17994483
201185_at	HTRA1	-0,66934516	-0,84928999	0,17994483
226549_at	SBK1	-0,66934516	-0,84928999	0,17994483
234208_at	-	-0,66934516	-0,84928999	0,17994483
1552646_at	IL11RA	-0,66934516	-0,84928999	0,17994483
227392_at	NISCH	-0,66934516	-0,84928999	0,17994483
220684_at	TBX21	-0,66934516	-0,84928999	0,17994483
200032_s_at	RPL9	8,339506942	8,159649868	0,179857074
201560_at	CLIC4	4,383685466	4,20412215	0,179563316
227181_at	LNP1	1,578719607	1,399283506	0,179436101
224951_at	CERS5	3,653735827	3,474637573	0,179098253
211963_s_at	ARPC5	6,61780432	6,438813814	0,178990506
212162_at	KIDINS220	2,295108393	2,116147513	0,178960881
222507_s_at	TMEM9B	2,878891456	2,700070732	0,178820724
203959_s_at	ZBTB40	1,830468611	1,651676651	0,17879196

215909_x_at	MINK1	0,167727503	-0,011001236	0,178728739
205142_x_at	ABCD1	0,167727503	-0,011001236	0,178728739
209216_at	WDR45	2,159837409	1,981728801	0,178108608
222427_s_at	LARS	4,049824219	3,871747013	0,178077207
203654_s_at	COIL	2,771643417	2,593698508	0,177944909
1553607_at	BACH1 /// GRIK1-A	-2,250580859	-2,4284334	0,177852541
1552747_a_at	PP2D1	-2,250580859	-2,4284334	0,177852541
1556624_at	-	-2,250580859	-2,4284334	0,177852541
1561867_at	-	-2,250580859	-2,4284334	0,177852541
1561596_at	-	-2,250580859	-2,4284334	0,177852541
243613_at	DENND5B	-2,250580859	-2,4284334	0,177852541
1566950_at	-	-2,250580859	-2,4284334	0,177852541
1553086_at	C11orf40	-2,250580859	-2,4284334	0,177852541
1561129_at	-	-2,250580859	-2,4284334	0,177852541
1565638_at	-	-2,250580859	-2,4284334	0,177852541
223629_at	PCDHB5	-2,250580859	-2,4284334	0,177852541
215981_at	LOC100505534	-2,250580859	-2,4284334	0,177852541
242426_at	NRG4	-2,250580859	-2,4284334	0,177852541
1562803_at	LRRC37A5P	-2,250580859	-2,4284334	0,177852541
242086_at	SPATA6	-2,250580859	-2,4284334	0,177852541
1561165_a_at	DEFB108B	-2,250580859	-2,4284334	0,177852541
1566597_at	-	-2,250580859	-2,4284334	0,177852541
214598_at	CLDN8	-2,250580859	-2,4284334	0,177852541
1563351_at	-	-2,250580859	-2,4284334	0,177852541
240977_at	-	-2,250580859	-2,4284334	0,177852541
213382_at	MST1 /// MST1P2	-2,250580859	-2,4284334	0,177852541
1555038_at	EPB41L4A	-2,250580859	-2,4284334	0,177852541
240820_at	-	-2,250580859	-2,4284334	0,177852541
1557802_at	-	-2,250580859	-2,4284334	0,177852541
243892_at	-	-2,250580859	-2,4284334	0,177852541
233547_x_at	PDE1A	-2,250580859	-2,4284334	0,177852541
1552940_at	TEDDM1	-2,250580859	-2,4284334	0,177852541
1556571_at	LOC100506538 ///	-2,250580859	-2,4284334	0,177852541
1562909_at	C1orf98	-2,250580859	-2,4284334	0,177852541
236010_at	-	-2,250580859	-2,4284334	0,177852541
1566715_at	-	-2,250580859	-2,4284334	0,177852541
205064_at	SPRR1B	-2,250580859	-2,4284334	0,177852541
231220_at	CELF4	-2,250580859	-2,4284334	0,177852541
1557297_at	-	-2,250580859	-2,4284334	0,177852541
1557280_s_at	ITSN1	-2,250580859	-2,4284334	0,177852541
237806_s_at	LOC284757 /// LO	-2,250580859	-2,4284334	0,177852541
242559_at	-	-2,250580859	-2,4284334	0,177852541
235453_at	TOR1AIP2	-2,250580859	-2,4284334	0,177852541
225140_at	KLF3	-2,250580859	-2,4284334	0,177852541
1555233_at	RHOJ	-2,250580859	-2,4284334	0,177852541
1559737_at	-	-2,250580859	-2,4284334	0,177852541
209591_s_at	BMP7	-2,250580859	-2,4284334	0,177852541
213808_at	ADAM23	-2,250580859	-2,4284334	0,177852541
1553211_at	ANKFN1	-2,250580859	-2,4284334	0,177852541
236984_at	C4orf26	-2,250580859	-2,4284334	0,177852541

225412_at	TMEM87B	1,493647653	1,315897482	0,177750171
216728_at	-	-1,537851782	-1,715576125	0,177724343
240022_at	ZC3H4	-1,537851782	-1,715576125	0,177724343
1570158_at	-	-1,537851782	-1,715576125	0,177724343
216155_at	-	-1,537851782	-1,715576125	0,177724343
215174_at	FMO6P	-1,537851782	-1,715576125	0,177724343
241315_at	-	-1,537851782	-1,715576125	0,177724343
1563513_at	SYTL4	-1,537851782	-1,715576125	0,177724343
1554528_at	C3orf15 /// LOC10	-1,537851782	-1,715576125	0,177724343
224099_at	KCNH7	-1,537851782	-1,715576125	0,177724343
234122_at	-	-1,537851782	-1,715576125	0,177724343
244007_at	ZNF462	-1,537851782	-1,715576125	0,177724343
231889_at	UBR4	-1,537851782	-1,715576125	0,177724343
242172_at	-	-1,537851782	-1,715576125	0,177724343
210016_at	MYT1L	-1,537851782	-1,715576125	0,177724343
221091_at	INSL5	-1,537851782	-1,715576125	0,177724343
241581_at	-	-1,537851782	-1,715576125	0,177724343
236826_at	-	-1,537851782	-1,715576125	0,177724343
244231_at	BPI	-1,537851782	-1,715576125	0,177724343
242729_at	LOC100506866	-1,537851782	-1,715576125	0,177724343
208280_at	CDRT1	-1,537851782	-1,715576125	0,177724343
1570451_at	C1orf61	-1,537851782	-1,715576125	0,177724343
217366_at	-	-1,537851782	-1,715576125	0,177724343
1569839_s_at	LRRC37A5P	-1,537851782	-1,715576125	0,177724343
1561175_at	LOC283482	-1,537851782	-1,715576125	0,177724343
1559630_at	-	-1,537851782	-1,715576125	0,177724343
207401_at	PROX1	-1,537851782	-1,715576125	0,177724343
1566924_at	-	-1,537851782	-1,715576125	0,177724343
204575_s_at	MMP19	-1,537851782	-1,715576125	0,177724343
228621_at	HFE2	-1,537851782	-1,715576125	0,177724343
221345_at	FFAR2	-1,537851782	-1,715576125	0,177724343
1552566_at	BTBD16	-1,537851782	-1,715576125	0,177724343
208243_s_at	CNR1	-1,537851782	-1,715576125	0,177724343
238028_at	C6orf132	-1,537851782	-1,715576125	0,177724343
232989_s_at	CCDC136	-1,537851782	-1,715576125	0,177724343
207493_x_at	SSX2 /// SSX2B	-1,537851782	-1,715576125	0,177724343
237406_at	NEK6	-1,537851782	-1,715576125	0,177724343
1567285_at	OR5L2	-1,537851782	-1,715576125	0,177724343
212841_s_at	PPFIBP2	-1,537851782	-1,715576125	0,177724343
213849_s_at	PPP2R2B	-1,537851782	-1,715576125	0,177724343
214057_at	MCL1	1,345611839	1,167919222	0,177692617
212990_at	SYNJ1	2,276736537	2,099079406	0,177657131
209472_at	CCBL2	3,739061547	3,561438401	0,177623146
206911_at	TRIM25	0,524545436	0,346964736	0,177580701
220470_at	BET1L	0,355751005	0,178191865	0,17755914
240013_at	-	0,355751005	0,178191865	0,17755914
222280_at	LOC100506469	1,047341799	0,869824768	0,17751703
32029_at	PDPK1	2,938156718	2,760811921	0,177344798
241721_at	-	1,160996507	0,98381571	0,177180797
239282_at	CCDC41	-0,924000698	-1,100943374	0,176942676

224014_at	ZNF160	-0,924000698	-1,100943374	0,176942676
1555598_a_at	DUSP19	-0,924000698	-1,100943374	0,176942676
207733_x_at	PSG9	-0,924000698	-1,100943374	0,176942676
242278_at	-	-0,924000698	-1,100943374	0,176942676
1569557_at	ZNF248	-0,924000698	-1,100943374	0,176942676
233567_at	IPO9-AS1	-0,924000698	-1,100943374	0,176942676
214969_at	MAP3K9	-0,924000698	-1,100943374	0,176942676
237845_at	-	-0,924000698	-1,100943374	0,176942676
229890_at	LOC100507547 ///	-0,924000698	-1,100943374	0,176942676
230927_at	-	-0,924000698	-1,100943374	0,176942676
242449_at	-	-0,924000698	-1,100943374	0,176942676
241828_x_at	LOC339240	-0,924000698	-1,100943374	0,176942676
1565694_at	DTYMK	-0,924000698	-1,100943374	0,176942676
244430_at	PLD6	-0,924000698	-1,100943374	0,176942676
227468_at	CPT1C	-0,924000698	-1,100943374	0,176942676
231564_at	-	-0,924000698	-1,100943374	0,176942676
1554564_a_at	SPPL3	-0,924000698	-1,100943374	0,176942676
205690_s_at	BUD31	4,858503557	4,681569691	0,176933866
49679_s_at	-	0,286439462	0,109515809	0,176923653
202228_s_at	NPTN	3,948420174	3,771650587	0,176769587
202154_x_at	TUBB3	4,281531373	4,104828001	0,176703372
212645_x_at	BRE	3,327128828	3,150841611	0,176287217
202090_s_at	UQCR11	6,111461908	5,935337309	0,176124599
225620_at	RAB35	2,436165655	2,260052727	0,176112928
205436_s_at	H2AFX	4,832741146	4,6568995	0,175841646
220041_at	PIGZ	0,08619576	-0,089635713	0,175831473
220588_at	BCAS4	0,08619576	-0,089635713	0,175831473
228228_at	DACT3	0,08619576	-0,089635713	0,175831473
224023_s_at	BRK1	1,528073958	1,352622437	0,175451521
208687_x_at	HSPA8 /// SNORD1	7,989783083	7,814462319	0,175320764
216593_s_at	LOC100505991 ///	2,21987062	2,044580955	0,175289665
213054_at	HAUS5	1,898032047	1,722747778	0,175284269
202307_s_at	TAP1	5,077218663	4,902370015	0,174848648
229664_at	MAPK8	1,97249974	1,797682937	0,174816802
225702_at	C8orf76	2,725848647	2,551398837	0,17444981
240131_at	-	0,707657549	0,533258442	0,174399107
212184_s_at	TAB2	4,1816995	4,007339482	0,174360018
201353_s_at	BAZ2A	0,791908897	0,61779983	0,174109067
220189_s_at	MGAT4B	1,910557155	1,736450458	0,174106697
202676_x_at	FASTK	1,910557155	1,736450458	0,174106697
1564272_a_at	KLHDC1	-2,774503732	-2,948507524	0,174003792
1558387_at	UG0898H09	-2,774503732	-2,948507524	0,174003792
1561789_at	-	-2,774503732	-2,948507524	0,174003792
1561030_at	TMC7	-2,774503732	-2,948507524	0,174003792
224745_x_at	OTUD5	2,356619952	2,182711118	0,173908834
200626_s_at	MATR3 /// SNHG4	5,952214192	5,778352051	0,17386214
44111_at	VPS33B	2,864307955	2,690475573	0,173832381
240327_at	-	-0,180990326	-0,354781582	0,173791256
217232_x_at	HBB	-0,180990326	-0,354781582	0,173791256
209075_s_at	ISCU	4,600469559	4,426930782	0,173538777

219264_s_at	PPP2R3B	0,001114523	-0,172194225	0,173308748
208204_s_at	CAV3	0,001114523	-0,172194225	0,173308748
205256_at	ZBTB39	0,001114523	-0,172194225	0,173308748
208492_at	RFXAP	0,900065578	0,727123109	0,172942469
235295_at	-	0,282358733	0,109458907	0,172899826
225257_at	CCDC97	1,92317941	1,750483134	0,172696277
202529_at	PRPSAP1	3,942941653	3,770303326	0,172638327
210076_x_at	SERBP1	3,67542249	3,502959177	0,172463312
232625_at	TLN2	-1,06282519	-1,234835326	0,172010136
1552879_a_at	ATOH7	-1,06282519	-1,234835326	0,172010136
210687_at	CPT1A	-1,06282519	-1,234835326	0,172010136
229508_at	U2AF2	-1,06282519	-1,234835326	0,172010136
1565680_at	DIP2C	-1,06282519	-1,234835326	0,172010136
214942_at	RBM34	-1,06282519	-1,234835326	0,172010136
228882_at	TUB	-1,06282519	-1,234835326	0,172010136
240136_at	-	-1,06282519	-1,234835326	0,172010136
1570293_at	TBL1X	-1,06282519	-1,234835326	0,172010136
1564334_at	LINC00304	-1,06282519	-1,234835326	0,172010136
207209_at	CETN1	-1,06282519	-1,234835326	0,172010136
205507_at	ARHGEF15	-1,06282519	-1,234835326	0,172010136
1553318_at	RIBC1	-1,06282519	-1,234835326	0,172010136
1561599_at	-	-1,06282519	-1,234835326	0,172010136
226645_at	KLF2	-1,06282519	-1,234835326	0,172010136
220068_at	VPREB3	-1,06282519	-1,234835326	0,172010136
241243_at	-	-1,06282519	-1,234835326	0,172010136
207404_s_at	HTR1E	-1,06282519	-1,234835326	0,172010136
1562392_at	MRPL23	-1,06282519	-1,234835326	0,172010136
221434_s_at	SLIRP	5,461739019	5,289808844	0,171930175
244016_at	-	-2,708365344	-2,880284116	0,171918772
226976_at	KPNA6 /// LOC100	2,50022283	2,328492509	0,171730322
219116_s_at	DCUN1D2	1,20396005	1,032267596	0,171692453
1557121_s_at	-	1,20396005	1,032267596	0,171692453
209929_s_at	IKBKG	2,523100136	2,351470274	0,171629863
243719_at	STK19	-0,088974936	-0,260526297	0,171551361
216302_at	HNRNPCL1 /// LOC	-0,088974936	-0,260526297	0,171551361
225286_at	ARSD	-0,088974936	-0,260526297	0,171551361
238856_s_at	PANK2	1,000459215	0,829309537	0,171149678
206205_at	MPHOSPH9	2,636121902	2,465016194	0,171105707
219620_x_at	TOR4A	-0,280273599	-0,45121326	0,170939661
231118_at	ANKRD35	-0,280273599	-0,45121326	0,170939661
238096_at	LOC284023	-0,280273599	-0,45121326	0,170939661
1566432_at	-	-0,280273599	-0,45121326	0,170939661
218251_at	MID1IP1	1,830468611	1,659532492	0,17093612
227187_at	CBLL1	3,175897496	3,005076416	0,17082108
200853_at	H2AFZ	7,187996724	7,017320773	0,170675951
244028_at	USP15	-0,384193355	-0,554696666	0,170503311
223906_s_at	TEX101	-0,384193355	-0,554696666	0,170503311
206728_at	ECE2	-0,384193355	-0,554696666	0,170503311
225379_at	MAPT	-0,384193355	-0,554696666	0,170503311
214871_x_at	LOC100287590	-0,384193355	-0,554696666	0,170503311

210646_x_at	RPL13A /// RPL13A	8,479198171	8,308750874	0,170447297
202866_at	DNAJB12	2,16969527	1,999354538	0,170340732
200018_at	LOC100508408 ///	8,220639676	8,050595148	0,170044527
242118_x_at	-	-2,495152701	-2,665156778	0,170004077
1565851_at	-	-2,495152701	-2,665156778	0,170004077
243878_at	-	-2,495152701	-2,665156778	0,170004077
244118_at	GABRA1	-2,495152701	-2,665156778	0,170004077
240995_at	-	-2,495152701	-2,665156778	0,170004077
1559725_at	-	-2,495152701	-2,665156778	0,170004077
1555718_x_at	-	-2,495152701	-2,665156778	0,170004077
1558226_a_at	-	-2,495152701	-2,665156778	0,170004077
234808_x_at	-	-2,495152701	-2,665156778	0,170004077
1555517_at	GABRG3	-2,495152701	-2,665156778	0,170004077
218469_at	GREM1	-2,495152701	-2,665156778	0,170004077
1566600_at	-	-2,495152701	-2,665156778	0,170004077
1552325_at	CCDC11	-2,495152701	-2,665156778	0,170004077
1567304_at	-	-2,495152701	-2,665156778	0,170004077
1560885_x_at	SLC17A1	-2,495152701	-2,665156778	0,170004077
1567174_at	-	-2,495152701	-2,665156778	0,170004077
216167_at	LRRN2	-2,495152701	-2,665156778	0,170004077
1562815_at	EFCAB6	-2,495152701	-2,665156778	0,170004077
220724_at	CWH43	-2,495152701	-2,665156778	0,170004077
200006_at	PARK7	7,274779392	7,104837894	0,169941498
229466_at	TRIM66	0,820160788	0,65030602	0,169854768
202588_at	AK1	0,820160788	0,65030602	0,169854768
200048_s_at	JTB	6,309811726	6,140086422	0,169725304
203309_s_at	HPS1	0,20740896	0,03784737	0,16956159
201487_at	CTSC	4,0992582	3,929758078	0,169500122
200044_at	GATC /// SRSF9	5,094148055	4,924704877	0,169443178
218988_at	SLC35E3	2,75889404	2,589579418	0,169314621
231870_s_at	NMD3	5,097429074	4,928184329	0,169244745
225346_at	MTERFD3	1,88491011	1,715863686	0,169046425
210959_s_at	SRD5A1	1,716003175	1,547376668	0,168626507
225527_at	CEBPG	3,450420588	3,281812922	0,168607666
202292_x_at	LYPLA2	2,51558601	2,346979857	0,168606153
207405_s_at	RAD17	4,038663603	3,870131307	0,168532296
226312_at	RICTOR	3,142467702	2,973964204	0,168503499
209832_s_at	CDT1	2,138605583	1,970106792	0,168498791
225419_at	MPLKIP	4,46459746	4,296136603	0,168460857
217209_at	CEACAM3	-0,49284695	-0,661178575	0,168331624
214066_x_at	NPR2	-0,49284695	-0,661178575	0,168331624
220469_at	COPE	-0,49284695	-0,661178575	0,168331624
206388_at	PDE3A	-0,49284695	-0,661178575	0,168331624
236820_at	-	-0,49284695	-0,661178575	0,168331624
1559136_s_at	IDS /// LOC100272	-0,49284695	-0,661178575	0,168331624
210749_x_at	DDR1 /// MIR4640	-0,49284695	-0,661178575	0,168331624
243916_x_at	UBLCP1	3,327128828	3,158887039	0,168241789
201663_s_at	SMC4	4,686080913	4,517858152	0,168222761
210466_s_at	SERBP1	6,348866152	6,180787207	0,168078945
239515_at	-	0,735999505	0,568155442	0,167844063

225370_at	PYGO2	0,735999505	0,568155442	0,167844063
233943_x_at	PLEKHA8	1,246401134	1,078619386	0,167781747
204640_s_at	SPOP	3,991087145	3,823361714	0,167725431
210588_x_at	HNRNPH3	4,267962641	4,100577438	0,167385203
204354_at	POT1	3,685074281	3,517892726	0,167181555
201518_at	CBX1	4,809922892	4,642835395	0,167087497
213668_s_at	SOX4	2,199941239	2,03313004	0,166811199
206074_s_at	HMGA1	3,957687264	3,790897218	0,166790046
201530_x_at	EIF4A1 /// SENP3-I	6,627793424	6,461054502	0,166738922
222504_s_at	EMC8	1,745502609	1,578991006	0,166511603
208842_s_at	GORASP2	3,89798617	3,732068422	0,165917747
223365_at	DHX37	1,775035811	1,60918327	0,165852541
202561_at	TNKS	1,775035811	1,60918327	0,165852541
1558445_at	-	0,95222556	0,786391549	0,165834011
235314_at	RPL32P3	0,95222556	0,786391549	0,165834011
206583_at	ZNF673	2,312738346	2,147194726	0,16554362
204342_at	SLC25A24	3,889146356	3,723797476	0,165348879
1561959_x_at	-	-2,641232718	-2,8064286	0,165195882
212592_at	IGJ	-2,641232718	-2,8064286	0,165195882
243938_x_at	DNAH5	-2,641232718	-2,8064286	0,165195882
1556697_at	GPRIN3	-2,641232718	-2,8064286	0,165195882
1554507_at	NAALAD2	-2,641232718	-2,8064286	0,165195882
211873_s_at	PCDHGA9	0,491906512	0,326777209	0,165129302
241150_at	SPTAN1	0,491906512	0,326777209	0,165129302
40612_at	DOPEY1	0,190330798	0,025627601	0,164703197
1569592_a_at	F11	-2,336678463	-2,501359113	0,16468065
1569218_at	-	-2,336678463	-2,501359113	0,16468065
1562737_at	-	-2,336678463	-2,501359113	0,16468065
242128_at	OTX2	-2,336678463	-2,501359113	0,16468065
1553321_a_at	SULT1C4	-2,336678463	-2,501359113	0,16468065
1561016_at	-	-2,336678463	-2,501359113	0,16468065
233166_at	DEFB126	-2,336678463	-2,501359113	0,16468065
235401_s_at	FCRLA	-2,336678463	-2,501359113	0,16468065
204965_at	GC	-2,336678463	-2,501359113	0,16468065
230865_at	LIX1	-2,336678463	-2,501359113	0,16468065
1558411_at	EGFEM1P	-2,336678463	-2,501359113	0,16468065
244039_x_at	-	-2,336678463	-2,501359113	0,16468065
234793_at	-	-2,336678463	-2,501359113	0,16468065
207053_at	SLC8A1	-2,336678463	-2,501359113	0,16468065
244223_at	-	-2,336678463	-2,501359113	0,16468065
1557534_at	LOC339862	-2,336678463	-2,501359113	0,16468065
211533_at	PDGFRA	-2,336678463	-2,501359113	0,16468065
234861_at	LOC93463	-2,336678463	-2,501359113	0,16468065
1553330_at	C7orf45	-2,336678463	-2,501359113	0,16468065
239519_at	-	-2,336678463	-2,501359113	0,16468065
237898_at	-	-2,336678463	-2,501359113	0,16468065
1554960_at	C1orf110	-2,336678463	-2,501359113	0,16468065
234517_at	-	-2,336678463	-2,501359113	0,16468065
1569881_at	DRP2	-2,336678463	-2,501359113	0,16468065
1562492_at	LOC340090	-2,336678463	-2,501359113	0,16468065

1560678_at	-	-2,336678463	-2,501359113	0,16468065
236313_at	CDKN2B	-2,336678463	-2,501359113	0,16468065
1554643_at	RGS11	-2,336678463	-2,501359113	0,16468065
208075_s_at	CCL7	-2,336678463	-2,501359113	0,16468065
229515_at	PAWR	-2,336678463	-2,501359113	0,16468065
234961_at	-	-2,336678463	-2,501359113	0,16468065
216447_at	-	-2,336678463	-2,501359113	0,16468065
210067_at	AQP4	-2,336678463	-2,501359113	0,16468065
1560253_at	LHX9	-2,336678463	-2,501359113	0,16468065
203817_at	GUCY1B3	-2,336678463	-2,501359113	0,16468065
209348_s_at	MAF	-2,336678463	-2,501359113	0,16468065
201400_at	PSMB3	6,416864503	6,252225061	0,164639442
243299_at	-	1,160996507	0,996453274	0,164543234
204238_s_at	C6orf108	3,317909135	3,153389283	0,164519853
201090_x_at	LOC100288366 ///	7,818509201	7,654041356	0,164467844
201180_s_at	GNAI3	5,27108412	5,106686237	0,164397883
1494_f_at	CYP2A6	-0,13424916	-0,298533662	0,164284502
211480_s_at	SLCO1A2	-1,452713826	-1,616924751	0,164210925
1568802_at	NADSYN1	-1,452713826	-1,616924751	0,164210925
1556465_at	-	-1,452713826	-1,616924751	0,164210925
244419_at	FRZB	-1,452713826	-1,616924751	0,164210925
214905_at	RASGRF1	-1,452713826	-1,616924751	0,164210925
1558565_at	AKNA	-1,452713826	-1,616924751	0,164210925
222911_s_at	CXorf36	-1,452713826	-1,616924751	0,164210925
242834_at	-	-1,452713826	-1,616924751	0,164210925
215818_at	NUDT7	-1,452713826	-1,616924751	0,164210925
233584_at	C20orf62	-1,452713826	-1,616924751	0,164210925
205834_s_at	PART1	-1,452713826	-1,616924751	0,164210925
220131_at	FXYD7	-1,452713826	-1,616924751	0,164210925
1570533_at	CMPK1	-1,452713826	-1,616924751	0,164210925
210472_at	MT1G	-1,452713826	-1,616924751	0,164210925
204445_s_at	ALOX5	-1,452713826	-1,616924751	0,164210925
227933_at	LINGO1	-1,452713826	-1,616924751	0,164210925
217702_at	IL27RA	-1,452713826	-1,616924751	0,164210925
216688_at	-	-1,452713826	-1,616924751	0,164210925
232671_at	SLFNL1-AS1	-1,452713826	-1,616924751	0,164210925
210551_s_at	ASMT	-1,452713826	-1,616924751	0,164210925
226355_at	POC1A	2,829929672	2,665727652	0,16420202
203606_at	NDUFS6	5,744560023	5,580721056	0,163838968
207436_x_at	-	2,817838865	2,654387125	0,163451739
52940_at	SIGIRR	2,944701303	2,78170612	0,162995183
213398_s_at	SDR39U1	3,175897496	3,012928641	0,162968854
209630_s_at	FBXW2	3,688415399	3,525687129	0,16272827
223197_s_at	SMARCAD1	3,086998005	2,924357093	0,162640912
243405_at	-	1,871521385	1,709047448	0,162473937
35436_at	GOLGA2	2,60664099	2,444218179	0,162422811
226786_at	RFX1	1,421391637	1,259081178	0,162310459
230987_at	-	-0,609040214	-0,771340337	0,162300123
1565771_at	DKFZp434E1119	-0,609040214	-0,771340337	0,162300123
241249_at	LOC253039	-0,609040214	-0,771340337	0,162300123

224307_x_at	MAGI3	-0,609040214	-0,771340337	0,162300123
225828_at	DAGLB	-0,609040214	-0,771340337	0,162300123
1558327_at	ZDHHC18	-0,609040214	-0,771340337	0,162300123
1556976_s_at	-	-0,609040214	-0,771340337	0,162300123
223689_at	IGF2BP1	0,045517965	-0,116767475	0,16228544
211222_s_at	HAP1	0,045517965	-0,116767475	0,16228544
204524_at	PDPK1	3,122148189	2,960011739	0,16213645
229031_at	-	1,701542258	1,539450683	0,162091575
201397_at	PHGDH	2,636121902	2,47417686	0,161945042
208741_at	SAP18	2,149438457	1,987810614	0,161627843
213672_at	MARS	0,97663406	0,815200271	0,161433789
225037_at	SLC35C2	2,530390387	2,36899666	0,161393727
225926_at	VTI1B	1,307201325	1,146010634	0,161190691
226524_at	C3orf38	3,4540469	3,293054172	0,160992727
203152_at	MRPL40	4,352240682	4,191278093	0,160962589
203531_at	CUL5	3,657457797	3,496833915	0,160623882
33760_at	PEX14	3,225725073	3,065217829	0,160507244
201076_at	NHP2L1	3,660947664	3,500705275	0,160242389
222254_at	-	-1,212652659	-1,372734086	0,160081426
242316_at	TMOD3	-1,212652659	-1,372734086	0,160081426
235563_at	GPRC5A	-1,212652659	-1,372734086	0,160081426
244384_at	-	-1,212652659	-1,372734086	0,160081426
233024_at	-	-1,212652659	-1,372734086	0,160081426
1553489_a_at	TEKT5	-1,212652659	-1,372734086	0,160081426
201958_s_at	PPP1R12B	-1,212652659	-1,372734086	0,160081426
239626_x_at	TM9SF3	-1,212652659	-1,372734086	0,160081426
229531_at	-	-1,212652659	-1,372734086	0,160081426
206277_at	P2RY2	-1,212652659	-1,372734086	0,160081426
237811_at	-	-1,212652659	-1,372734086	0,160081426
202914_s_at	ARHGEF11	-1,212652659	-1,372734086	0,160081426
234257_at	TNS3	-1,212652659	-1,372734086	0,160081426
236025_at	NCOR2	-1,212652659	-1,372734086	0,160081426
240375_at	-	-1,212652659	-1,372734086	0,160081426
212077_at	CALD1	-1,212652659	-1,372734086	0,160081426
242004_x_at	-	-1,212652659	-1,372734086	0,160081426
235108_at	KCNK3	-1,212652659	-1,372734086	0,160081426
237568_at	-	-1,212652659	-1,372734086	0,160081426
240506_at	-	-1,212652659	-1,372734086	0,160081426
213590_at	SLC16A5	-1,212652659	-1,372734086	0,160081426
1553412_at	AGBL4	-1,212652659	-1,372734086	0,160081426
214719_at	SLC46A3	1,910557155	1,750483134	0,160074021
218499_at	MST4	5,246614709	5,086553178	0,160061531
226831_at	SLC25A46	4,060218357	3,900176622	0,160041735
226753_at	FAM76B	2,460890032	2,300902761	0,159987271
238398_s_at	-	-1,982324124	-2,142286822	0,159962698
1561073_at	-	-1,982324124	-2,142286822	0,159962698
1558587_at	-	-1,982324124	-2,142286822	0,159962698
228170_at	OLIG1	-1,982324124	-2,142286822	0,159962698
1561676_at	PRICKLE2-AS3	-1,982324124	-2,142286822	0,159962698
229387_at	LRCH3	-1,982324124	-2,142286822	0,159962698

1569929_at	-	-1,982324124	-2,142286822	0,159962698
1561229_at	-	-1,982324124	-2,142286822	0,159962698
220878_at	-	-1,982324124	-2,142286822	0,159962698
217479_at	-	-1,982324124	-2,142286822	0,159962698
205357_s_at	AGTR1	-1,982324124	-2,142286822	0,159962698
1564856_s_at	LOC727924	-1,982324124	-2,142286822	0,159962698
1559288_at	-	-1,982324124	-2,142286822	0,159962698
1562371_s_at	VWA3B	-1,982324124	-2,142286822	0,159962698
240881_at	-	-1,982324124	-2,142286822	0,159962698
217026_at	CFTR	-1,982324124	-2,142286822	0,159962698
1570422_at	SNTG2	-1,982324124	-2,142286822	0,159962698
241204_at	-	-1,982324124	-2,142286822	0,159962698
213895_at	EMP1	-1,982324124	-2,142286822	0,159962698
208305_at	PGR	-1,982324124	-2,142286822	0,159962698
216420_at	-	-1,982324124	-2,142286822	0,159962698
1562365_at	LOC286177	-1,982324124	-2,142286822	0,159962698
239604_at	-	-1,982324124	-2,142286822	0,159962698
1554099_a_at	SPIN3	-1,982324124	-2,142286822	0,159962698
231668_x_at	IGHG1	-1,982324124	-2,142286822	0,159962698
1561474_at	-	-1,982324124	-2,142286822	0,159962698
1566082_at	-	-1,982324124	-2,142286822	0,159962698
205330_at	MN1	-1,982324124	-2,142286822	0,159962698
231082_at	-	-1,982324124	-2,142286822	0,159962698
236407_at	KCNE1	-1,982324124	-2,142286822	0,159962698
237259_at	-	-1,982324124	-2,142286822	0,159962698
1569910_at	-	-1,982324124	-2,142286822	0,159962698
236543_at	-	-1,982324124	-2,142286822	0,159962698
1560019_at	MGC11082	-1,982324124	-2,142286822	0,159962698
241078_at	SLC35E4	-1,982324124	-2,142286822	0,159962698
1553859_at	TPH1	-1,982324124	-2,142286822	0,159962698
1562633_at	RMST	-1,982324124	-2,142286822	0,159962698
235100_at	-	-1,982324124	-2,142286822	0,159962698
1555027_at	P4HA2	-1,982324124	-2,142286822	0,159962698
216062_at	LOC100507164	-1,982324124	-2,142286822	0,159962698
233397_at	-	-1,982324124	-2,142286822	0,159962698
224349_at	-	-1,982324124	-2,142286822	0,159962698
203888_at	THBD	-1,982324124	-2,142286822	0,159962698
1563339_at	-	-1,982324124	-2,142286822	0,159962698
1569063_at	-	-1,982324124	-2,142286822	0,159962698
240901_at	-	-1,982324124	-2,142286822	0,159962698
205669_at	NCAM2	-1,982324124	-2,142286822	0,159962698
1552772_at	CLEC4D	-1,982324124	-2,142286822	0,159962698
207603_at	SLC4A7	-1,982324124	-2,142286822	0,159962698
1553363_at	C6orf195	-1,982324124	-2,142286822	0,159962698
234013_at	YME1L1	-1,982324124	-2,142286822	0,159962698
1554294_s_at	TTBK2	-1,982324124	-2,142286822	0,159962698
237399_at	-	-1,982324124	-2,142286822	0,159962698
213524_s_at	G0S2	-1,982324124	-2,142286822	0,159962698
1557287_at	-	-1,982324124	-2,142286822	0,159962698
232539_at	-	-1,982324124	-2,142286822	0,159962698

231341_at	SLC35D3	-1,982324124	-2,142286822	0,159962698
225805_at	HNRNPU	1,730929079	1,571112806	0,159816273
228168_at	ATP5G3	1,5944803	1,434743608	0,159736692
54037_at	HPS4	1,394100906	1,23437528	0,159725626
223416_at	SF3B14	6,456784072	6,297066767	0,159717305
224433_s_at	DDX54	0,425036312	0,265475485	0,159560828
235598_at	SMAD2	0,425036312	0,265475485	0,159560828
236274_at	EIF3B	0,425036312	0,265475485	0,159560828
242205_at	-	-0,730013898	-0,889222211	0,159208313
1553078_at	OR5P3	-0,730013898	-0,889222211	0,159208313
235864_at	ATP6V1D	-0,730013898	-0,889222211	0,159208313
236137_at	-	-0,730013898	-0,889222211	0,159208313
232011_s_at	MAP1LC3A	-0,730013898	-0,889222211	0,159208313
1557236_at	APOL6	-0,730013898	-0,889222211	0,159208313
232016_at	-	-0,730013898	-0,889222211	0,159208313
1560488_at	LCNL1	-0,730013898	-0,889222211	0,159208313
226205_at	ANKRD13D	0,244681185	0,085534992	0,159146193
216733_s_at	GATM	-0,856259172	-1,015398016	0,159138844
243219_x_at	MRPL50	-0,856259172	-1,015398016	0,159138844
236334_at	-	-0,856259172	-1,015398016	0,159138844
1570192_at	-	-0,856259172	-1,015398016	0,159138844
231295_at	ME3	-0,856259172	-1,015398016	0,159138844
244164_at	-	-0,856259172	-1,015398016	0,159138844
215400_x_at	ADCY9	-0,856259172	-1,015398016	0,159138844
236596_at	-	-0,856259172	-1,015398016	0,159138844
203240_at	FCGBP	-0,856259172	-1,015398016	0,159138844
1553207_at	ARL10	-0,856259172	-1,015398016	0,159138844
240784_at	NAT16	-0,856259172	-1,015398016	0,159138844
1566980_at	-	-0,856259172	-1,015398016	0,159138844
201425_at	ALDH2	-0,856259172	-1,015398016	0,159138844
237236_x_at	LOC731789	-0,856259172	-1,015398016	0,159138844
205121_at	SGCB	-0,856259172	-1,015398016	0,159138844
207967_at	VPS45	-0,856259172	-1,015398016	0,159138844
200813_s_at	PAFAH1B1	2,063971513	1,905294823	0,15867669
222634_s_at	TBL1XR1	2,007220798	1,848871029	0,158349769
225115_at	HIPK2	1,510970097	1,352622437	0,15834766
222477_s_at	TM7SF3	3,855094415	3,697224187	0,157870228
238790_at	LOC374443	-0,043925712	-0,201789721	0,157864009
242809_at	IL1RL1	-0,043925712	-0,201789721	0,157864009
1553943_at	NS3BP	-0,043925712	-0,201789721	0,157864009
230414_s_at	TMEM170A	-0,043925712	-0,201789721	0,157864009
1552307_a_at	TTC39C	1,000459215	0,842599219	0,157859995
201754_at	COX6C	7,30625837	7,148463049	0,157795321
222612_at	PSPC1	0,707657549	0,550017041	0,157640508
201264_at	COPE	2,989672611	2,832036229	0,157636382
233378_at	-	0,791908897	0,634301128	0,157607769
225532_at	CABLES1	2,778527892	2,621003244	0,157524648
227979_at	RBM4	2,778527892	2,621003244	0,157524648
226128_at	BROX	3,792209911	3,634723971	0,15748594
201575_at	SNW1	5,148110767	4,990646361	0,157464407

215659_at	GSDMB	0,618277321	0,461385738	0,156891584
225254_at	CCDC97	0,618277321	0,461385738	0,156891584
225159_s_at	ELK4	3,577387089	3,420735804	0,156651286
44563_at	WRAP53	2,542537547	2,385940436	0,156597112
205022_s_at	FOXN3	2,444186596	2,287674614	0,156511982
230186_at	TMEM136	-0,230432956	-0,386896102	0,156463146
231660_at	-	-0,230432956	-0,386896102	0,156463146
229955_at	FBXO3	-0,230432956	-0,386896102	0,156463146
232133_at	ADAMTS10	-0,230432956	-0,386896102	0,156463146
216833_x_at	GYPB	-0,230432956	-0,386896102	0,156463146
217053_x_at	ETV1	-0,230432956	-0,386896102	0,156463146
208632_at	RNF10	1,528073958	1,371634653	0,156439305
240457_at	-	-1,893506789	-2,049747196	0,156240407
224115_at	-	-1,893506789	-2,049747196	0,156240407
232073_at	PPFIA2	-1,893506789	-2,049747196	0,156240407
226084_at	MAP1B	-1,893506789	-2,049747196	0,156240407
1563279_at	-	-1,893506789	-2,049747196	0,156240407
231326_s_at	LOC100506234	-1,893506789	-2,049747196	0,156240407
1554999_at	RASGEF1B	-1,893506789	-2,049747196	0,156240407
1552629_a_at	JMJD6	-1,893506789	-2,049747196	0,156240407
1559735_at	-	-1,893506789	-2,049747196	0,156240407
223890_at	-	-1,893506789	-2,049747196	0,156240407
218963_s_at	KRT23	-1,893506789	-2,049747196	0,156240407
237292_at	DPYSL3	-1,893506789	-2,049747196	0,156240407
207626_s_at	SLC7A2	-1,893506789	-2,049747196	0,156240407
1552463_at	SERPINB11	-1,893506789	-2,049747196	0,156240407
205559_s_at	PCSK5	-1,893506789	-2,049747196	0,156240407
215319_at	ATP8B3	-1,893506789	-2,049747196	0,156240407
1563794_s_at	IFT122	-1,893506789	-2,049747196	0,156240407
232720_at	LINGO2	-1,893506789	-2,049747196	0,156240407
1568723_at	ATP9B	-1,893506789	-2,049747196	0,156240407
214265_at	ITGA8	-1,893506789	-2,049747196	0,156240407
233293_at	NPAS3	-1,893506789	-2,049747196	0,156240407
238845_at	SLC30A4	-1,893506789	-2,049747196	0,156240407
229004_at	ADAMTS15	-1,893506789	-2,049747196	0,156240407
220268_at	C11orf67	-1,893506789	-2,049747196	0,156240407
240860_at	LOC100506288	-1,893506789	-2,049747196	0,156240407
208007_at	-	-1,893506789	-2,049747196	0,156240407
220136_s_at	CRYBA2	-1,893506789	-2,049747196	0,156240407
1568633_a_at	-	-1,893506789	-2,049747196	0,156240407
230317_x_at	TMCO3	-1,893506789	-2,049747196	0,156240407
235565_at	ZNF425	-1,893506789	-2,049747196	0,156240407
1567167_at	-	-1,893506789	-2,049747196	0,156240407
235843_at	-	-1,893506789	-2,049747196	0,156240407
202832_at	GCC2	2,978070706	2,822379032	0,155691674
235581_at	-	-0,135065865	-0,290694975	0,15562911
242885_at	-	-0,135065865	-0,290694975	0,15562911
214991_s_at	PIGO	-0,135065865	-0,290694975	0,15562911
1554379_a_at	TP73	-0,135065865	-0,290694975	0,15562911
204119_s_at	ADK	4,552867541	4,397256845	0,155610697

212004_at	C1orf144	2,872644433	2,717094254	0,155550178
223311_s_at	MTA3	1,65554524	1,500059916	0,155485323
208768_x_at	RPL22	8,115541908	7,960057366	0,155484543
225213_at	PPTC7	4,817982599	4,662634779	0,15534782
226805_at	FITM2	1,139057614	0,98381571	0,155241904
211720_x_at	RPLP0	8,468327627	8,313123757	0,15520387
205355_at	ACADSB	0,355751005	0,20062106	0,155129945
1557480_a_at	PPP1R27	0,355751005	0,20062106	0,155129945
242536_at	-	-2,074223907	-2,229233437	0,15500953
233109_at	COL12A1	-2,074223907	-2,229233437	0,15500953
1557363_a_at	PHIP	-2,074223907	-2,229233437	0,15500953
226834_at	-	-2,074223907	-2,229233437	0,15500953
243605_at	-	-2,074223907	-2,229233437	0,15500953
240585_at	-	-2,074223907	-2,229233437	0,15500953
232538_at	-	-2,074223907	-2,229233437	0,15500953
233942_at	LOC613126	-2,074223907	-2,229233437	0,15500953
241436_at	SCNN1G	-2,074223907	-2,229233437	0,15500953
204750_s_at	DSC2	-2,074223907	-2,229233437	0,15500953
216793_x_at	-	-2,074223907	-2,229233437	0,15500953
203903_s_at	HEPH	-2,074223907	-2,229233437	0,15500953
1561576_a_at	-	-2,074223907	-2,229233437	0,15500953
244879_at	-	-2,074223907	-2,229233437	0,15500953
209278_s_at	TFPI2	-2,074223907	-2,229233437	0,15500953
230923_at	FAM19A1	-2,074223907	-2,229233437	0,15500953
1557195_at	-	-2,074223907	-2,229233437	0,15500953
1566840_at	LOC283674	-2,074223907	-2,229233437	0,15500953
1557829_at	-	-2,074223907	-2,229233437	0,15500953
1559394_a_at	-	-2,074223907	-2,229233437	0,15500953
1570505_at	ABCB4	-2,074223907	-2,229233437	0,15500953
241218_at	LOC100506387	-2,074223907	-2,229233437	0,15500953
203399_x_at	PSG3	-2,074223907	-2,229233437	0,15500953
1561627_at	-	-2,074223907	-2,229233437	0,15500953
211422_at	TRPM3	-2,074223907	-2,229233437	0,15500953
232482_at	OR51E2	-2,074223907	-2,229233437	0,15500953
237474_at	-	-2,074223907	-2,229233437	0,15500953
241308_at	-	-2,074223907	-2,229233437	0,15500953
1560610_at	-	-2,074223907	-2,229233437	0,15500953
210643_at	TNFSF11	-2,074223907	-2,229233437	0,15500953
243311_at	DEFB132	-2,074223907	-2,229233437	0,15500953
1570020_at	AACSP1	-2,074223907	-2,229233437	0,15500953
204359_at	FLRT2 /// LOC1005	-2,074223907	-2,229233437	0,15500953
230037_at	CD8B	-2,074223907	-2,229233437	0,15500953
1561882_at	SYTL3	-2,074223907	-2,229233437	0,15500953
228375_at	IGSF11	-2,074223907	-2,229233437	0,15500953
216612_x_at	-	-2,074223907	-2,229233437	0,15500953
237905_at	KRT25	-2,074223907	-2,229233437	0,15500953
216148_at	-	-2,074223907	-2,229233437	0,15500953
235262_at	ZNF585B	-2,074223907	-2,229233437	0,15500953
1563770_at	ZBTB8B	-2,074223907	-2,229233437	0,15500953
227612_at	ELAVL3	-2,074223907	-2,229233437	0,15500953

1561543_at	-	-2,074223907	-2,229233437	0,15500953
1555179_at	IGHV7-81	-2,074223907	-2,229233437	0,15500953
1560912_at	LOC389043	-2,074223907	-2,229233437	0,15500953
239246_at	FARP1	-2,074223907	-2,229233437	0,15500953
1554163_at	TWIST2	-2,074223907	-2,229233437	0,15500953
237038_at	CXCL14	-2,074223907	-2,229233437	0,15500953
237631_at	-	-2,074223907	-2,229233437	0,15500953
212325_at	LIMCH1	-2,074223907	-2,229233437	0,15500953
202952_s_at	ADAM12	-2,074223907	-2,229233437	0,15500953
233126_s_at	OLAH	-2,074223907	-2,229233437	0,15500953
203534_at	LSM1	4,784078567	4,629244987	0,15483358
213798_s_at	CAP1	6,641740374	6,48708155	0,154658824
204212_at	ACOT8	2,932161212	2,777528602	0,15463261
203774_at	MTR	3,804904984	3,650351707	0,154553278
1557116_at	APOL6	1,024284941	0,869824768	0,154460173
213084_x_at	RPL23A /// SNORD	8,758749464	8,604436003	0,15431346
203306_s_at	SLC35A1	3,562907192	3,408745454	0,154161738
219973_at	ARSJ	-1,802716385	-1,956681069	0,153964684
1562073_at	-	-1,802716385	-1,956681069	0,153964684
215571_at	-	-1,802716385	-1,956681069	0,153964684
1562455_at	-	-1,802716385	-1,956681069	0,153964684
233222_at	-	-1,802716385	-1,956681069	0,153964684
219965_s_at	MAGIX	-1,802716385	-1,956681069	0,153964684
210614_at	TTPA	-1,802716385	-1,956681069	0,153964684
1557542_at	-	-1,802716385	-1,956681069	0,153964684
223883_s_at	STK31	-1,802716385	-1,956681069	0,153964684
213543_at	SGCD	-1,802716385	-1,956681069	0,153964684
1559507_at	LOC100130357	-1,802716385	-1,956681069	0,153964684
1564310_a_at	PARP15	-1,802716385	-1,956681069	0,153964684
1556881_at	ARHGEF33	-1,802716385	-1,956681069	0,153964684
220835_s_at	ZNF407	-1,802716385	-1,956681069	0,153964684
221330_at	CHRM2	-1,802716385	-1,956681069	0,153964684
244258_at	-	-1,802716385	-1,956681069	0,153964684
241518_at	-	-1,802716385	-1,956681069	0,153964684
236376_at	RIMKLA	-1,802716385	-1,956681069	0,153964684
234161_at	-	-1,802716385	-1,956681069	0,153964684
216772_at	-	-1,802716385	-1,956681069	0,153964684
215914_at	-	-1,802716385	-1,956681069	0,153964684
208606_s_at	WNT4	-1,802716385	-1,956681069	0,153964684
1570279_at	ABCA1	-1,802716385	-1,956681069	0,153964684
237471_at	LOC154822	-1,802716385	-1,956681069	0,153964684
1564536_at	ASB10	-1,802716385	-1,956681069	0,153964684
240182_at	-	-1,802716385	-1,956681069	0,153964684
216661_x_at	CYP2C9	-1,802716385	-1,956681069	0,153964684
213050_at	COBL	-1,802716385	-1,956681069	0,153964684
235180_at	STYX	-1,802716385	-1,956681069	0,153964684
220879_at	-	-1,802716385	-1,956681069	0,153964684
1553583_a_at	THRSP	-1,802716385	-1,956681069	0,153964684
241664_x_at	-	-2,416625063	-2,570522741	0,153897678
1562079_at	-	-2,416625063	-2,570522741	0,153897678

234103_at	KCNT2	-2,416625063	-2,570522741	0,153897678
1568646_x_at	ZNF208	-2,416625063	-2,570522741	0,153897678
216775_at	USP53	-2,416625063	-2,570522741	0,153897678
240160_x_at	-	-2,416625063	-2,570522741	0,153897678
1554932_at	ZSWIM2	-2,416625063	-2,570522741	0,153897678
1570141_at	MYO5B	-2,416625063	-2,570522741	0,153897678
231797_at	SIX4	-2,416625063	-2,570522741	0,153897678
215643_at	SEMA3D	-2,416625063	-2,570522741	0,153897678
216819_at	-	-2,416625063	-2,570522741	0,153897678
240341_at	-	-2,416625063	-2,570522741	0,153897678
240815_at	-	-2,416625063	-2,570522741	0,153897678
215473_at	-	-2,416625063	-2,570522741	0,153897678
227242_s_at	EBF3	-2,416625063	-2,570522741	0,153897678
241332_at	-	-2,416625063	-2,570522741	0,153897678
241142_at	-	-2,416625063	-2,570522741	0,153897678
206143_at	SLC26A3	-2,416625063	-2,570522741	0,153897678
206253_at	DLG2	-2,416625063	-2,570522741	0,153897678
220771_at	LINC00328	-2,416625063	-2,570522741	0,153897678
221086_s_at	FEZF2	-2,416625063	-2,570522741	0,153897678
233392_at	-	-2,416625063	-2,570522741	0,153897678
237488_at	-	-2,416625063	-2,570522741	0,153897678
220906_at	-	-2,416625063	-2,570522741	0,153897678
204414_at	LARGE	-2,416625063	-2,570522741	0,153897678
211517_s_at	IL5RA	-2,416625063	-2,570522741	0,153897678
237547_at	-	-2,416625063	-2,570522741	0,153897678
208571_at	ANP32D	-2,416625063	-2,570522741	0,153897678
208271_at	PAPOLB	-2,416625063	-2,570522741	0,153897678
1570206_at	-	-2,416625063	-2,570522741	0,153897678
244736_at	-	-2,416625063	-2,570522741	0,153897678
212338_at	MYO1D	-2,416625063	-2,570522741	0,153897678
242681_at	-	-2,416625063	-2,570522741	0,153897678
213624_at	SMPDL3A	-2,416625063	-2,570522741	0,153897678
204618_s_at	GABPB1	2,636121902	2,482273566	0,153848336
219263_at	RNF128	-2,569309811	-2,723114724	0,153804913
220810_at	CLCA3P	-2,569309811	-2,723114724	0,153804913
234374_at	-	-2,569309811	-2,723114724	0,153804913
232656_at	-	-2,569309811	-2,723114724	0,153804913
215793_at	MTMR7	-2,569309811	-2,723114724	0,153804913
1555103_s_at	FGF7	-2,569309811	-2,723114724	0,153804913
1566694_at	-	-2,569309811	-2,723114724	0,153804913
235144_at	RASEF	-2,569309811	-2,723114724	0,153804913
238490_at	KIAA2026	0,820160788	0,666486137	0,153674651
235012_at	LRCH1	0,926325262	0,772793615	0,153531648
51228_at	RBM12B	1,633451527	1,480085027	0,1533665
212186_at	ACACA	3,18076822	3,027559376	0,153208843
222119_s_at	FBXO11	3,218770562	3,065641037	0,153129525
228077_at	MRI1	3,588695979	3,435695778	0,153000201
202397_at	LOC128322 /// NU	5,535113886	5,382450951	0,152662935
209016_s_at	KRT7	3,768156719	3,615671864	0,152484855
228308_at	FKBP11	0,458905032	0,306454867	0,152450165

204317_at	TRMU	0,735999505	0,583607503	0,152392002
206870_at	PPARA	-0,332405896	-0,484558493	0,152152597
242848_x_at	-	-0,332405896	-0,484558493	0,152152597
1555792_a_at	CCDC116	-0,332405896	-0,484558493	0,152152597
238042_at	-	-0,332405896	-0,484558493	0,152152597
205570_at	PIP4K2A	1,745502609	1,593605508	0,1518971
201804_x_at	TBCB	4,790575561	4,638750201	0,15182536
217861_s_at	PREB	3,156891301	3,005076416	0,151814886
226391_at	-	2,074841895	1,923216608	0,151625287
1557886_at	CEP112	-0,437449947	-0,589001171	0,151551224
222782_s_at	GMIP	-0,437449947	-0,589001171	0,151551224
211705_s_at	SORBS1	-0,437449947	-0,589001171	0,151551224
220172_at	DCAF17	-0,437449947	-0,589001171	0,151551224
228760_at	SRSF8	2,401508182	2,250228409	0,151279773
203514_at	MAP3K3	1,476175815	1,324949741	0,151226074
237449_at	SP8	-0,994186487	-1,145057014	0,150870527
240777_at	SYNE2	-0,994186487	-1,145057014	0,150870527
220601_at	C16orf70	-0,994186487	-1,145057014	0,150870527
232498_at	HEATR7A /// LOC1	-0,994186487	-1,145057014	0,150870527
1567611_at	-	-0,994186487	-1,145057014	0,150870527
244717_x_at	SV2B	-0,994186487	-1,145057014	0,150870527
232785_at	RGAG1	-0,994186487	-1,145057014	0,150870527
205730_s_at	ABLIM3	-0,994186487	-1,145057014	0,150870527
216655_s_at	-	-0,994186487	-1,145057014	0,150870527
205082_s_at	AOX1	-0,994186487	-1,145057014	0,150870527
237095_at	ASXL2	-0,994186487	-1,145057014	0,150870527
1566558_x_at	FLJ90757	-0,994186487	-1,145057014	0,150870527
227311_at	SNX25	-0,994186487	-1,145057014	0,150870527
220385_at	JPH2	-0,994186487	-1,145057014	0,150870527
231801_at	NFATC2	-0,994186487	-1,145057014	0,150870527
243145_at	-	-0,994186487	-1,145057014	0,150870527
219160_s_at	PAPOLG	-0,994186487	-1,145057014	0,150870527
234465_at	EME1	2,348178434	2,197404429	0,150774005
202218_s_at	FADS2	0,648195588	0,497424714	0,150770874
222617_s_at	FAM204A	2,914752575	2,76399677	0,150755805
213860_x_at	CSNK1A1	5,583598186	5,432873543	0,150724643
232421_at	SCARB1	0,556428218	0,405752839	0,150675379
1554183_s_at	TRIM73 /// TRIM7	0,556428218	0,405752839	0,150675379
212851_at	DCUN1D4	1,307201325	1,156599976	0,150601349
212473_s_at	MICAL2	1,307201325	1,156599976	0,150601349
200054_at	ZNF259	3,389999385	3,239448352	0,150551033
218115_at	ASF1B	3,132805465	2,982339418	0,150466047
1566664_at	CCDC168	-1,369772723	-1,520022123	0,150249401
1564760_at	-	-1,369772723	-1,520022123	0,150249401
220528_at	VNN3	-1,369772723	-1,520022123	0,150249401
210504_at	KLF1	-1,369772723	-1,520022123	0,150249401
220340_at	GREB1L	-1,369772723	-1,520022123	0,150249401
231686_at	GATM	-1,369772723	-1,520022123	0,150249401
1556679_at	-	-1,369772723	-1,520022123	0,150249401
1565795_at	DUOX1	-1,369772723	-1,520022123	0,150249401

228227_at	ITGB1BP1	-1,369772723	-1,520022123	0,150249401
217500_at	TIAL1	-1,369772723	-1,520022123	0,150249401
232247_at	ZNF502	-1,369772723	-1,520022123	0,150249401
222639_s_at	PLBD1	-1,369772723	-1,520022123	0,150249401
240203_at	-	-1,369772723	-1,520022123	0,150249401
222727_s_at	SLC24A6	-1,369772723	-1,520022123	0,150249401
213834_at	IQSEC3 /// LOC10(	-1,369772723	-1,520022123	0,150249401
215140_at	-	-1,369772723	-1,520022123	0,150249401
227655_at	LOC100505806	-1,369772723	-1,520022123	0,150249401
232678_at	-	-1,369772723	-1,520022123	0,150249401
1560511_at	-	-1,369772723	-1,520022123	0,150249401
205564_at	PAGE4	-1,369772723	-1,520022123	0,150249401
208389_s_at	SLC1A2	-1,369772723	-1,520022123	0,150249401
204347_at	AK4 /// LOC10050	-1,369772723	-1,520022123	0,150249401
243166_at	SLC30A5	0,08619576	-0,063377083	0,149572843
243757_at	-	0,08619576	-0,063377083	0,149572843
1553205_at	C20orf166-AS1	0,08619576	-0,063377083	0,149572843
242013_at	BCL2L15	-1,712732543	-1,862269442	0,149536899
1564282_a_at	LINC00491	-1,712732543	-1,862269442	0,149536899
237557_at	-	-1,712732543	-1,862269442	0,149536899
210429_at	RHD	-1,712732543	-1,862269442	0,149536899
1556134_a_at	B3GNT5 /// LOC10	-1,712732543	-1,862269442	0,149536899
223652_at	AS3MT	-1,712732543	-1,862269442	0,149536899
217463_s_at	C11orf9	-1,712732543	-1,862269442	0,149536899
233349_at	TLK2	-1,712732543	-1,862269442	0,149536899
215391_at	MAP1A	-1,712732543	-1,862269442	0,149536899
209686_at	S100B	-1,712732543	-1,862269442	0,149536899
236711_at	ZIC4	-1,712732543	-1,862269442	0,149536899
1562865_at	-	-1,712732543	-1,862269442	0,149536899
237349_at	-	-1,712732543	-1,862269442	0,149536899
237427_at	-	-1,712732543	-1,862269442	0,149536899
239570_at	RAB1A	-1,712732543	-1,862269442	0,149536899
221467_at	MC4R	-1,712732543	-1,862269442	0,149536899
237782_at	-	-1,712732543	-1,862269442	0,149536899
1569306_at	-	-1,712732543	-1,862269442	0,149536899
231368_at	BOD1L2	-1,712732543	-1,862269442	0,149536899
244722_at	-	-1,712732543	-1,862269442	0,149536899
1570542_a_at	PMPCB	-1,712732543	-1,862269442	0,149536899
1564548_at	LOC642620	-1,712732543	-1,862269442	0,149536899
1557840_at	EFHB	-1,712732543	-1,862269442	0,149536899
233989_at	-	-1,712732543	-1,862269442	0,149536899
238838_at	TCERG1	-1,712732543	-1,862269442	0,149536899
241222_at	-	-1,712732543	-1,862269442	0,149536899
206794_at	ERBB4	-1,712732543	-1,862269442	0,149536899
222693_at	FNDC3B	-1,712732543	-1,862269442	0,149536899
228770_at	GPR146 /// LOC10	-1,712732543	-1,862269442	0,149536899
212344_at	SULF1	-1,712732543	-1,862269442	0,149536899
242938_s_at	FOXK2	0,282358733	0,133104519	0,149254214
229611_at	LMLN	0,282358733	0,133104519	0,149254214
1570055_at	-	0,282358733	0,133104519	0,149254214

225216_at	FAM199X	2,042564225	1,893444329	0,149119896
242977_at	LOC100506128	-2,163157732	-2,312054429	0,148896697
239712_at	CCDC171	-2,163157732	-2,312054429	0,148896697
234623_x_at	-	-2,163157732	-2,312054429	0,148896697
1559280_a_at	LOC100506013	-2,163157732	-2,312054429	0,148896697
240472_at	-	-2,163157732	-2,312054429	0,148896697
1553905_at	CXorf22	-2,163157732	-2,312054429	0,148896697
239352_at	SLC6A15	-2,163157732	-2,312054429	0,148896697
233659_at	-	-2,163157732	-2,312054429	0,148896697
1561461_at	-	-2,163157732	-2,312054429	0,148896697
1560735_s_at	LOC727924 /// OR	-2,163157732	-2,312054429	0,148896697
234783_at	-	-2,163157732	-2,312054429	0,148896697
234396_at	-	-2,163157732	-2,312054429	0,148896697
221076_at	-	-2,163157732	-2,312054429	0,148896697
217546_at	MT1M	-2,163157732	-2,312054429	0,148896697
241264_at	-	-2,163157732	-2,312054429	0,148896697
1560337_at	LOC286184	-2,163157732	-2,312054429	0,148896697
1561452_at	-	-2,163157732	-2,312054429	0,148896697
1563591_at	LOC100506403 ///	-2,163157732	-2,312054429	0,148896697
236421_at	ANKRD45	-2,163157732	-2,312054429	0,148896697
244635_s_at	-	-2,163157732	-2,312054429	0,148896697
240337_at	RHOA	-2,163157732	-2,312054429	0,148896697
224537_at	PCDHGC5	-2,163157732	-2,312054429	0,148896697
238985_at	-	-2,163157732	-2,312054429	0,148896697
222383_s_at	ALOXE3	-2,163157732	-2,312054429	0,148896697
244621_x_at	-	-2,163157732	-2,312054429	0,148896697
219302_s_at	CNTNAP2	-2,163157732	-2,312054429	0,148896697
1552448_a_at	C8orf12	-2,163157732	-2,312054429	0,148896697
236710_at	C1orf87	-2,163157732	-2,312054429	0,148896697
211907_s_at	PARD6B	-2,163157732	-2,312054429	0,148896697
235881_at	FMNL2	-2,163157732	-2,312054429	0,148896697
215046_at	KANSL1L	-2,163157732	-2,312054429	0,148896697
241279_at	-	-2,163157732	-2,312054429	0,148896697
231356_at	LOC100652917	-2,163157732	-2,312054429	0,148896697
243907_at	-	-2,163157732	-2,312054429	0,148896697
240423_at	LOC441204	-2,163157732	-2,312054429	0,148896697
215039_at	HS2ST1 /// LOC33	-2,163157732	-2,312054429	0,148896697
236871_s_at	IQCF3 /// IQCF4	-2,163157732	-2,312054429	0,148896697
222810_s_at	RASAL2	-2,163157732	-2,312054429	0,148896697
1564789_at	-	-2,163157732	-2,312054429	0,148896697
214044_at	RYR2	-2,163157732	-2,312054429	0,148896697
219564_at	KCNJ16	-2,163157732	-2,312054429	0,148896697
1555845_at	-	2,597647262	2,449078728	0,148568534
212360_at	AMPD2	2,613382867	2,465016194	0,148366673
206578_at	NKX2-5	1,326463531	1,178100917	0,148362614
1561765_at	-	-2,708365344	-2,856570137	0,148204793
218541_s_at	C8orf4	-2,708365344	-2,856570137	0,148204793
1569719_at	BCL2L14	-2,708365344	-2,856570137	0,148204793
1561136_at	GYPE	-2,708365344	-2,856570137	0,148204793
1565026_a_at	OFCC1	-2,708365344	-2,856570137	0,148204793

224933_s_at	JMJD1C	2,642918178	2,49476365	0,148154529
212361_s_at	ATP2A2	2,884644843	2,736663738	0,147981105
200916_at	TAGLN2	5,963268214	5,815423292	0,147844923
208856_x_at	RPLP0	8,392968128	8,245499284	0,147468844
205245_at	PARD6A	0,97663406	0,829309537	0,147324523
223076_s_at	NSUN2	3,156891301	3,010094234	0,146797067
226802_s_at	LOC96610	1,995527416	1,848871029	0,146656387
210183_x_at	PNN	5,88707333	5,740442694	0,146630636
203055_s_at	ARHGEF1	1,225123479	1,078619386	0,146504093
239490_at	COA1	0,39105295	0,244646697	0,146406253
240700_at	TOR1AIP2	0,39105295	0,244646697	0,146406253
1566171_at	RFFL	0,39105295	0,244646697	0,146406253
222918_at	RAB9B	1,345611839	1,199296812	0,146315026
218031_s_at	FOXN3	2,063971513	1,917743773	0,14622774
205395_s_at	MRE11A	3,478193934	3,332103994	0,146089939
208144_s_at	-	-0,551284523	-0,697182596	0,145898074
214171_s_at	U2AF2	-0,551284523	-0,697182596	0,145898074
244624_at	RPS27A	-0,551284523	-0,697182596	0,145898074
235558_at	RBMS2	-0,551284523	-0,697182596	0,145898074
215951_at	-	-0,551284523	-0,697182596	0,145898074
210134_x_at	SHOX2	-0,551284523	-0,697182596	0,145898074
1559474_at	SPEG	-0,551284523	-0,697182596	0,145898074
229566_at	LOC645638	-0,551284523	-0,697182596	0,145898074
216768_x_at	TTC38	-0,551284523	-0,697182596	0,145898074
215055_at	B3GNTL1	-0,551284523	-0,697182596	0,145898074
205727_at	TEP1	0,001114523	-0,144765583	0,145880106
1559870_at	LOC100129129	0,001114523	-0,144765583	0,145880106
1554631_at	ATM	0,20740896	0,061773582	0,145635378
229605_at	CYB5RL	0,20740896	0,061773582	0,145635378
1558739_at	-	-1,137208348	-1,282520722	0,145312374
221439_at	RBBP9	-1,137208348	-1,282520722	0,145312374
234570_at	ZNF71	-1,137208348	-1,282520722	0,145312374
229015_at	LOC286367	-1,137208348	-1,282520722	0,145312374
216195_at	ANK2	-1,137208348	-1,282520722	0,145312374
231475_at	TBC1D21	-1,137208348	-1,282520722	0,145312374
244293_at	UQCRB	-1,137208348	-1,282520722	0,145312374
222117_s_at	ADCK2	-1,137208348	-1,282520722	0,145312374
241609_at	FOXD3	-1,137208348	-1,282520722	0,145312374
208274_at	OCLM	-1,137208348	-1,282520722	0,145312374
240746_s_at	-	-1,137208348	-1,282520722	0,145312374
1554280_a_at	C9orf43	-1,137208348	-1,282520722	0,145312374
208431_s_at	TUB	-1,137208348	-1,282520722	0,145312374
1569833_at	-	-1,137208348	-1,282520722	0,145312374
208889_s_at	NCOR2	-1,137208348	-1,282520722	0,145312374
1556972_at	-	-1,137208348	-1,282520722	0,145312374
216311_at	OVOL3	-1,137208348	-1,282520722	0,145312374
229645_at	FAM69C	0,491906512	0,346964736	0,144941776
1563111_a_at	PIGX	4,130786109	3,985936256	0,144849853
218776_s_at	TMEM62	0,677745787	0,533258442	0,144487345
217753_s_at	RPS26	7,66774724	7,523309162	0,144438078

201189_s_at	ITPR3	2,884644843	2,740247037	0,144397806
221998_s_at	VRK3	3,378744007	3,234381926	0,144362081
213996_at	YPEL1	1,000459215	0,85655369	0,143905525
225048_at	PHF10	2,745538988	2,601822502	0,143716486
216954_x_at	ATP5O	4,540832283	4,397256845	0,143575438
1553570_x_at	COX2	8,393704428	8,250383361	0,143321067
213323_s_at	ZC3H7B	1,267146969	1,123905343	0,143241627
208867_s_at	CSNK1A1	2,866549889	2,723341187	0,143208701
208736_at	ARPC3	6,418239563	6,27503843	0,143201133
230031_at	HSPA5	1,830468611	1,687645398	0,142823213
213414_s_at	RPS19	8,349397649	8,206617601	0,142780048
201757_at	NDUFS5	6,745039797	6,602280782	0,142759015
205448_s_at	MAP3K12	1,139057614	0,996453274	0,142604341
211296_x_at	UBC	8,400144815	8,257663061	0,142481755
209944_at	ZNF410	4,799977847	4,657538356	0,142439491
236277_at	-	-0,66934516	-0,811769547	0,142424387
236039_at	LYPD5	-0,66934516	-0,811769547	0,142424387
1555236_a_at	PGC	-0,66934516	-0,811769547	0,142424387
221308_at	FRS2	-0,66934516	-0,811769547	0,142424387
209776_s_at	SLC19A1	-0,66934516	-0,811769547	0,142424387
1565660_at	FUT6	-0,66934516	-0,811769547	0,142424387
221260_s_at	CSRNP2	1,458255986	1,315897482	0,142358504
223363_at	PSMG3	3,18076822	3,038411131	0,142357089
239939_at	-	-0,088974936	-0,231045907	0,142070971
223384_s_at	TRIM4	3,378744007	3,236954658	0,141789349
242590_at	-	-0,180990326	-0,322340048	0,141349722
236239_at	XPNPEP1	-0,180990326	-0,322340048	0,141349722
212865_s_at	COL14A1	-2,250580859	-2,391863529	0,14128267
230309_at	BHMT2	-2,250580859	-2,391863529	0,14128267
224101_x_at	MRS2	-2,250580859	-2,391863529	0,14128267
224362_at	LOC100128922	-2,250580859	-2,391863529	0,14128267
1569124_at	LRRC63	-2,250580859	-2,391863529	0,14128267
1563054_at	-	-2,250580859	-2,391863529	0,14128267
1562321_at	PDK4	-2,250580859	-2,391863529	0,14128267
233102_at	-	-2,250580859	-2,391863529	0,14128267
234690_at	-	-2,250580859	-2,391863529	0,14128267
1564909_at	-	-2,250580859	-2,391863529	0,14128267
206515_at	CYP4F3	-2,250580859	-2,391863529	0,14128267
207647_at	CDY1	-2,250580859	-2,391863529	0,14128267
205442_at	MFAP3L	-2,250580859	-2,391863529	0,14128267
1556901_s_at	LOC149773	-2,250580859	-2,391863529	0,14128267
1569768_at	-	-2,250580859	-2,391863529	0,14128267
241753_at	-	-2,250580859	-2,391863529	0,14128267
234067_at	-	-2,250580859	-2,391863529	0,14128267
241032_at	ANKRD40	-2,250580859	-2,391863529	0,14128267
1555118_at	ENTPD3	-2,250580859	-2,391863529	0,14128267
1558451_at	LOC285500	-2,250580859	-2,391863529	0,14128267
1570099_at	-	-2,250580859	-2,391863529	0,14128267
1558964_at	FAT3	-2,250580859	-2,391863529	0,14128267
220736_at	SLC19A3	-2,250580859	-2,391863529	0,14128267

1564078_at	ADAM29	-2,250580859	-2,391863529	0,14128267
1557044_at	LOC100506930	-2,250580859	-2,391863529	0,14128267
227892_at	PRKAA2	-2,250580859	-2,391863529	0,14128267
1557030_at	GAB1	-2,250580859	-2,391863529	0,14128267
228431_at	NFYA	-2,250580859	-2,391863529	0,14128267
205747_at	CBLN1	-2,250580859	-2,391863529	0,14128267
243974_at	-	-2,250580859	-2,391863529	0,14128267
238204_at	-	-2,250580859	-2,391863529	0,14128267
234699_at	RNASE7	-2,250580859	-2,391863529	0,14128267
1553873_at	KLHL34	-2,250580859	-2,391863529	0,14128267
244770_at	-	-2,250580859	-2,391863529	0,14128267
214218_s_at	XIST	-2,250580859	-2,391863529	0,14128267
238050_at	ANTXR2	-2,250580859	-2,391863529	0,14128267
241473_at	-	-2,250580859	-2,391863529	0,14128267
215448_at	-	-2,250580859	-2,391863529	0,14128267
228683_s_at	KCTD15	-2,250580859	-2,391863529	0,14128267
1555035_a_at	CLRN1	-2,250580859	-2,391863529	0,14128267
239673_at	-	-2,250580859	-2,391863529	0,14128267
214591_at	KLHL4	-2,250580859	-2,391863529	0,14128267
239110_s_at	-	-2,250580859	-2,391863529	0,14128267
1557924_s_at	ALPL	-2,250580859	-2,391863529	0,14128267
210906_x_at	AQP4	-2,250580859	-2,391863529	0,14128267
222465_at	RSL24D1	5,451079563	5,30981955	0,141260013
205412_at	ACAT1	4,439413216	4,298213993	0,141199223
218964_at	ARID3B	2,410823487	2,269637658	0,141185828
1553338_at	SDE2	0,319287178	0,178191865	0,141095313
204229_at	SLC17A7	0,319287178	0,178191865	0,141095313
202162_s_at	CNOT8	3,252865595	3,111774082	0,141091514
201501_s_at	GRSF1	3,243218364	3,1021766	0,141041764
1554510_s_at	GHITM	4,06801876	3,927118341	0,140900419
221650_s_at	MED18	1,898032047	1,757155322	0,140876725
207127_s_at	HNRNPH3	3,694479488	3,553756974	0,140722514
227301_at	CCT6P1 /// CCT6P3	2,605420261	2,465016194	0,140404066
1561156_at	-	-2,495152701	-2,635549085	0,140396384
207461_at	-	-2,495152701	-2,635549085	0,140396384
1561021_at	-	-2,495152701	-2,635549085	0,140396384
1561415_at	-	-2,495152701	-2,635549085	0,140396384
1563187_at	-	-2,495152701	-2,635549085	0,140396384
237670_at	LOC284801	-2,495152701	-2,635549085	0,140396384
1554524_a_at	OLFM3	-2,495152701	-2,635549085	0,140396384
1554354_at	SIAE	-2,495152701	-2,635549085	0,140396384
216124_at	-	-2,495152701	-2,635549085	0,140396384
1561340_at	-	-2,495152701	-2,635549085	0,140396384
1568603_at	CADPS	-2,495152701	-2,635549085	0,140396384
213006_at	CEBPD	-2,495152701	-2,635549085	0,140396384
1562850_at	-	-2,495152701	-2,635549085	0,140396384
206535_at	SLC2A2	-2,495152701	-2,635549085	0,140396384
242502_at	KCNH5	-2,495152701	-2,635549085	0,140396384
211520_s_at	GRIA1	-2,495152701	-2,635549085	0,140396384
205789_at	CD1D	-2,495152701	-2,635549085	0,140396384

1552521_a_at	TMEM74	-2,495152701	-2,635549085	0,140396384
215231_at	PRKAG2	-2,495152701	-2,635549085	0,140396384
208146_s_at	CPVL	-2,495152701	-2,635549085	0,140396384
208673_s_at	SRSF3	3,883929118	3,743575485	0,140353633
233507_at	-	-0,791294935	-0,931539147	0,140244212
237088_at	-	-0,791294935	-0,931539147	0,140244212
239437_at	-	-0,791294935	-0,931539147	0,140244212
217711_at	TEK	-0,791294935	-0,931539147	0,140244212
207462_at	GLRA2	-0,791294935	-0,931539147	0,140244212
216116_at	NCKIPSD	-0,791294935	-0,931539147	0,140244212
243453_at	-	-0,791294935	-0,931539147	0,140244212
235501_at	-	-0,791294935	-0,931539147	0,140244212
215618_at	RSU1	-0,791294935	-0,931539147	0,140244212
1567319_at	-	-0,791294935	-0,931539147	0,140244212
206658_at	UPK3B	-0,791294935	-0,931539147	0,140244212
204626_s_at	ITGB3	-0,791294935	-0,931539147	0,140244212
206362_x_at	MAP3K10	0,926325262	0,786391549	0,139933713
224436_s_at	NIPSNAP3A	3,096793374	2,957089023	0,139704351
218605_at	TFB2M	3,991087145	3,851518797	0,139568348
200655_s_at	CALM1 /// CALM2	6,127604791	5,988110608	0,139494183
203135_at	TBP	3,132805465	2,993331554	0,139473912
218615_s_at	TMEM39A	1,910557155	1,771157334	0,139399821
219180_s_at	PEX26	1,307201325	1,167919222	0,139282103
241470_x_at	-	-2,641232718	-2,780225148	0,13899243
1560998_x_at	-	-2,641232718	-2,780225148	0,13899243
1561553_at	-	-2,641232718	-2,780225148	0,13899243
1557762_at	-	-2,641232718	-2,780225148	0,13899243
1570235_at	MGC27382	-2,641232718	-2,780225148	0,13899243
1555519_at	-	-2,641232718	-2,780225148	0,13899243
216151_at	-	-2,641232718	-2,780225148	0,13899243
232099_at	PCDHB16	-2,641232718	-2,780225148	0,13899243
1560918_at	-	-2,641232718	-2,780225148	0,13899243
217306_at	-	-2,641232718	-2,780225148	0,13899243
214268_s_at	MTMR4	3,171328694	3,032393954	0,13893474
217855_x_at	SDF4	3,551632175	3,412768101	0,138864074
218113_at	TMEM2	0,425036312	0,286279868	0,138756445
1556722_a_at	C20orf203	0,425036312	0,286279868	0,138756445
232915_at	DDX49	0,127732831	-0,011001236	0,138734066
231658_x_at	LOC100289079	-1,624955693	-1,763664074	0,138708381
243068_at	-	-1,624955693	-1,763664074	0,138708381
240378_at	-	-1,624955693	-1,763664074	0,138708381
229003_x_at	FAM69B	-1,624955693	-1,763664074	0,138708381
232664_at	FLJ12334	-1,624955693	-1,763664074	0,138708381
1564595_at	-	-1,624955693	-1,763664074	0,138708381
1562794_at	-	-1,624955693	-1,763664074	0,138708381
1567022_at	OR5AK4P	-1,624955693	-1,763664074	0,138708381
1558560_s_at	BLZF1	-1,624955693	-1,763664074	0,138708381
238048_at	-	-1,624955693	-1,763664074	0,138708381
1556963_at	-	-1,624955693	-1,763664074	0,138708381
221465_at	OR6A2	-1,624955693	-1,763664074	0,138708381

216093_at	-	-1,624955693	-1,763664074	0,138708381
232158_x_at	NIPAL1	-1,624955693	-1,763664074	0,138708381
213719_s_at	SMARCA4	-1,624955693	-1,763664074	0,138708381
1553249_at	SMC1B	-1,624955693	-1,763664074	0,138708381
243769_at	-	-1,624955693	-1,763664074	0,138708381
205814_at	GRM3	-1,624955693	-1,763664074	0,138708381
238244_at	LINC00535	-1,624955693	-1,763664074	0,138708381
244060_at	-	-1,624955693	-1,763664074	0,138708381
1561472_at	LOC728805	-1,624955693	-1,763664074	0,138708381
228462_at	IRX2	-1,624955693	-1,763664074	0,138708381
234434_at	-	-1,624955693	-1,763664074	0,138708381
234620_at	-	-1,624955693	-1,763664074	0,138708381
243903_at	-	-1,624955693	-1,763664074	0,138708381
242746_at	KLHDC10	-1,624955693	-1,763664074	0,138708381
234612_at	-	-1,624955693	-1,763664074	0,138708381
237531_at	-	-1,624955693	-1,763664074	0,138708381
210404_x_at	CAMK2B	-1,624955693	-1,763664074	0,138708381
230007_at	JMJD1C	-1,624955693	-1,763664074	0,138708381
243621_at	SPACA3	-1,624955693	-1,763664074	0,138708381
204422_s_at	FGF2	-1,624955693	-1,763664074	0,138708381
211564_s_at	PDLIM4	-1,624955693	-1,763664074	0,138708381
215794_x_at	GLUD2	2,575362572	2,436714502	0,13864807
200614_at	CLTC	6,241036142	6,102467837	0,138568305
213019_at	RANBP6	3,001575397	2,86314199	0,138433407
227934_at	KPNA5	2,159837409	2,021531611	0,138305798
212532_s_at	LSM12	3,291641956	3,153389283	0,138252673
221603_at	PEX16	-0,280273599	-0,418248858	0,137975259
222660_s_at	RNF38	-0,280273599	-0,418248858	0,137975259
208749_x_at	FLOT1	3,614400403	3,47651952	0,137880883
217398_x_at	GAPDH	8,671367746	8,533650469	0,137717277
202266_at	TDP2	5,340301351	5,202716986	0,137584365
201106_at	GPX4	6,001584555	5,86426443	0,137320125
213689_x_at	FAM69A	2,636121902	2,498884538	0,137237364
202852_s_at	AAGAB	3,122148189	2,98507484	0,137073349
203805_s_at	FANCA	0,95222556	0,815200271	0,137025289
234979_at	BCDIN3D	1,960819106	1,823960141	0,136858966
233625_x_at	CPSF3L	3,096793374	2,960011739	0,136781635
200024_at	RPS5	8,046150005	7,90979076	0,136359245
211342_x_at	MED12	1,745502609	1,60918327	0,136319338
202148_s_at	PYCR1	2,896717728	2,760632255	0,136085473
208623_s_at	EZR	4,927145108	4,791165786	0,135979322
235668_at	PRDM1	1,830468611	1,694547353	0,135921258
212632_at	STX7	1,578719607	1,44320939	0,135510217
232185_at	C20orf132	0,244681185	0,109458907	0,135222279
236485_at	LOC100507111	0,045517965	-0,089635713	0,135153678
229929_at	SPSB4	0,045517965	-0,089635713	0,135153678
1554977_at	C20orf201	0,045517965	-0,089635713	0,135153678
233851_s_at	TOR3A	1,610353504	1,475630127	0,134723376
233056_x_at	DLGAP4	2,621163856	2,486505579	0,134658277
229748_x_at	LOC100233156 ///	1,225123479	1,090640407	0,134483072

220651_s_at	MCM10	3,166767593	3,032393954	0,134373639
229551_x_at	ZNF367	3,843767728	3,709577987	0,13418974
55692_at	ELMO2	3,423707841	3,289658014	0,134049827
1558233_s_at	ATF1	2,365121417	2,23115548	0,133965937
239516_at	-	1,402495885	1,268569037	0,133926847
201856_s_at	ZFR	2,285848435	2,151985997	0,133862438
1555803_a_at	C11orf57	1,440265662	1,306903969	0,133361693
218651_s_at	LARP6	-0,924000698	-1,057301851	0,133301153
240892_at	-	-0,924000698	-1,057301851	0,133301153
243706_at	CDO1	-0,924000698	-1,057301851	0,133301153
1566145_s_at	LOC644450	-0,924000698	-1,057301851	0,133301153
216615_s_at	HTR3A	-0,924000698	-1,057301851	0,133301153
233523_at	BPIFB4	-0,924000698	-1,057301851	0,133301153
215744_at	FUS	-0,924000698	-1,057301851	0,133301153
235269_at	FAM83F	-0,924000698	-1,057301851	0,133301153
218978_s_at	SLC25A37	-0,924000698	-1,057301851	0,133301153
244176_at	-	-0,924000698	-1,057301851	0,133301153
215715_at	SLC6A2	-0,924000698	-1,057301851	0,133301153
1566870_at	-	-0,924000698	-1,057301851	0,133301153
230905_at	-	-0,924000698	-1,057301851	0,133301153
228887_x_at	CCDC61	-0,924000698	-1,057301851	0,133301153
233315_at	-	-0,924000698	-1,057301851	0,133301153
45828_at	ATP5SL	3,977592013	3,844399544	0,133192469
212197_x_at	MPRIP	3,716897585	3,584031895	0,13286569
218112_at	MRPS34	3,086998005	2,954216214	0,132781791
218729_at	LXN	2,843031657	2,710570973	0,132460684
1557825_at	-	-1,289805289	-1,422257026	0,132451737
208461_at	HIC1	-1,289805289	-1,422257026	0,132451737
234822_at	-	-1,289805289	-1,422257026	0,132451737
227497_at	SOX6	-1,289805289	-1,422257026	0,132451737
240060_at	-	-1,289805289	-1,422257026	0,132451737
230068_s_at	PEG3-AS1	-1,289805289	-1,422257026	0,132451737
236171_at	ATP2C2	-1,289805289	-1,422257026	0,132451737
234631_at	KRTAP4-8	-1,289805289	-1,422257026	0,132451737
1556850_at	CEP290	-1,289805289	-1,422257026	0,132451737
1564377_at	SHANK2	-1,289805289	-1,422257026	0,132451737
240610_at	LOC100507193	-1,289805289	-1,422257026	0,132451737
221121_at	CXorf48	-1,289805289	-1,422257026	0,132451737
208379_x_at	NPY2R	-1,289805289	-1,422257026	0,132451737
1554659_at	NEK4	-1,289805289	-1,422257026	0,132451737
217657_at	-	-1,289805289	-1,422257026	0,132451737
1563178_at	-	-1,289805289	-1,422257026	0,132451737
210328_at	GNMT	-1,289805289	-1,422257026	0,132451737
237335_at	ZP1	-1,289805289	-1,422257026	0,132451737
202186_x_at	PPP2R5A	-1,289805289	-1,422257026	0,132451737
220745_at	IL19	-1,289805289	-1,422257026	0,132451737
1563106_at	-	-1,289805289	-1,422257026	0,132451737
1560001_at	LOC100131581	-1,289805289	-1,422257026	0,132451737
202895_s_at	SIRPA	-1,289805289	-1,422257026	0,132451737
234720_s_at	TOB2	-1,289805289	-1,422257026	0,132451737

1564399_a_at	ARMCX4	-1,289805289	-1,422257026	0,132451737
1555716_a_at	CXADR	-1,289805289	-1,422257026	0,132451737
226846_at	PHYHD1	-1,289805289	-1,422257026	0,132451737
221969_at	PAX5	-1,289805289	-1,422257026	0,132451737
1559522_at	LOC100505918	-1,289805289	-1,422257026	0,132451737
226051_at	SELM	-1,289805289	-1,422257026	0,132451737
227947_at	PHACTR2	2,209870035	2,077419168	0,132450867
202929_s_at	DDT	3,786189171	3,653742074	0,132447097
217794_at	PRR13	4,646299467	4,514056284	0,132243183
220028_at	ACVR2B	0,458905032	0,326777209	0,132127822
1555388_s_at	SNX25	0,458905032	0,326777209	0,132127822
224742_at	ABHD12	1,640193838	1,508107415	0,132086423
201704_at	ENTPD6	1,671397663	1,539450683	0,13194698
217881_s_at	CDC27	2,560606634	2,428719338	0,131887296
230875_s_at	ATP11A	2,401508182	2,269637658	0,131870523
232172_at	CD99P1	0,556428218	0,424693306	0,131734912
202151_s_at	UBAC1	3,521615383	3,389993505	0,131621878
205453_at	HOXB2	2,21987062	2,088449486	0,131421134
235837_at	SNIP1	1,287793882	1,156599976	0,131193906
201104_x_at	LOC100506032 ///	4,267962641	4,136904703	0,131057938
207856_s_at	LOC150776 /// SM	2,468978138	2,338020736	0,130957402
32625_at	NPR1	-1,028097676	-1,158892565	0,130794888
226354_at	LACTB	3,426291319	3,295596077	0,130695242
228690_s_at	NDUFA11	5,936040515	5,805363703	0,130676812
1557836_at	ELMOD2	-0,49284695	-0,623254098	0,130407148
221642_at	-	-0,49284695	-0,623254098	0,130407148
243939_at	-	-0,49284695	-0,623254098	0,130407148
216475_at	-	-0,49284695	-0,623254098	0,130407148
230877_at	IGHD	-0,49284695	-0,623254098	0,130407148
230346_x_at	-	-0,49284695	-0,623254098	0,130407148
230667_at	FAM204A	-0,49284695	-0,623254098	0,130407148
211166_at	FAM153A	-0,49284695	-0,623254098	0,130407148
208569_at	HIST1H2AB	-0,49284695	-0,623254098	0,130407148
215449_at	TSPO2	-0,49284695	-0,623254098	0,130407148
1558438_a_at	IGHA1	-0,49284695	-0,623254098	0,130407148
203425_s_at	IGFBP5	-0,49284695	-0,623254098	0,130407148
204978_at	CLASRP	2,25755351	2,127337555	0,130215956
217229_at	ASB4	-2,774503732	-2,904470894	0,129967162
1566448_at	CD6	0,167727503	0,03784737	0,129880133
238923_at	SPOP	0,167727503	0,03784737	0,129880133
216836_s_at	ERBB2	0,167727503	0,03784737	0,129880133
223833_at	WDR55	0,764143511	0,634301128	0,129842383
211862_x_at	CFLAR	2,545254728	2,415420763	0,129833965
217878_s_at	CDC27	4,272544367	4,142787092	0,129757274
228293_at	DEPDC7	-2,336678463	-2,466349414	0,129670951
1561076_at	-	-2,336678463	-2,466349414	0,129670951
244891_x_at	-	-2,336678463	-2,466349414	0,129670951
1560528_at	-	-2,336678463	-2,466349414	0,129670951
1565633_at	-	-2,336678463	-2,466349414	0,129670951
236575_at	ARHGEF26-AS1	-2,336678463	-2,466349414	0,129670951

1564547_x_at	-	-2,336678463	-2,466349414	0,129670951
1560833_at	LOC643648	-2,336678463	-2,466349414	0,129670951
209656_s_at	TMEM47	-2,336678463	-2,466349414	0,129670951
228915_at	DACH1	-2,336678463	-2,466349414	0,129670951
220005_at	P2RY13	-2,336678463	-2,466349414	0,129670951
210661_at	GLRA3	-2,336678463	-2,466349414	0,129670951
206295_at	IL18	-2,336678463	-2,466349414	0,129670951
242964_at	-	-2,336678463	-2,466349414	0,129670951
220759_at	EDDM3B	-2,336678463	-2,466349414	0,129670951
1560174_at	SPAG16	-2,336678463	-2,466349414	0,129670951
1561204_at	-	-2,336678463	-2,466349414	0,129670951
242631_x_at	DLC1	-2,336678463	-2,466349414	0,129670951
231717_s_at	ZNF226	-2,336678463	-2,466349414	0,129670951
230573_at	SGK2	-2,336678463	-2,466349414	0,129670951
1560533_at	-	-2,336678463	-2,466349414	0,129670951
243935_at	FRAS1	-2,336678463	-2,466349414	0,129670951
233579_at	-	-2,336678463	-2,466349414	0,129670951
1569297_at	LOC731779	-2,336678463	-2,466349414	0,129670951
1564389_at	-	-2,336678463	-2,466349414	0,129670951
243193_at	-	-2,336678463	-2,466349414	0,129670951
1560494_a_at	CPXCR1	-2,336678463	-2,466349414	0,129670951
1553335_x_at	LOC285696	-2,336678463	-2,466349414	0,129670951
1564097_at	-	-2,336678463	-2,466349414	0,129670951
1557545_s_at	RNF165	-2,336678463	-2,466349414	0,129670951
241182_at	-	-2,336678463	-2,466349414	0,129670951
1562501_at	LOC255177	-2,336678463	-2,466349414	0,129670951
1553310_at	PLB1	-2,336678463	-2,466349414	0,129670951
239594_at	LOC145837	-2,336678463	-2,466349414	0,129670951
224302_s_at	MRPS36	4,413844848	4,28465024	0,129194607
203572_s_at	TAF6	2,804738618	2,675582704	0,129155914
55705_at	R3HDM4	3,453864381	3,325550084	0,128314297
218921_at	SIGIRR	1,871521385	1,743236669	0,128284715
228473_at	MSX1	-0,043925712	-0,172194225	0,128268513
1561518_at	LOC283914	-0,043925712	-0,172194225	0,128268513
1566906_at	LOC100129722	-0,043925712	-0,172194225	0,128268513
219940_s_at	PCID2	4,060218357	3,931967762	0,128250595
242993_at	-	-1,06282519	-1,19085694	0,12803175
208422_at	MSR1	-1,06282519	-1,19085694	0,12803175
1552970_s_at	ZMYM6	-1,06282519	-1,19085694	0,12803175
239176_at	-	-1,06282519	-1,19085694	0,12803175
233984_at	-	-1,06282519	-1,19085694	0,12803175
1557875_at	-	-1,06282519	-1,19085694	0,12803175
238989_at	C1GALT1C1	-1,06282519	-1,19085694	0,12803175
207787_at	KRT33B	-1,06282519	-1,19085694	0,12803175
233736_at	-	-1,06282519	-1,19085694	0,12803175
235386_at	-	-1,06282519	-1,19085694	0,12803175
240511_at	-	-1,06282519	-1,19085694	0,12803175
220067_at	SPTBN5	-1,06282519	-1,19085694	0,12803175
234832_at	ANTXR1	-1,06282519	-1,19085694	0,12803175
205492_s_at	DPYSL4	-1,06282519	-1,19085694	0,12803175

226788_at	CENPT	-1,06282519	-1,19085694	0,12803175
219464_at	CA14	-1,06282519	-1,19085694	0,12803175
219887_at	CCDC87	-1,06282519	-1,19085694	0,12803175
214015_at	SOCS7	-1,06282519	-1,19085694	0,12803175
205808_at	ASPH	-1,06282519	-1,19085694	0,12803175
217160_at	TSPY1	-1,06282519	-1,19085694	0,12803175
230072_at	TBC1D15	-1,06282519	-1,19085694	0,12803175
205580_s_at	HRH1	-1,06282519	-1,19085694	0,12803175
204252_at	CDK2	3,895112363	3,767104697	0,128007666
201379_s_at	TPD52L2	3,209439373	3,081435405	0,128003968
234901_at	-	-1,537851782	-1,665791776	0,127939994
1566665_at	CCDC168	-1,537851782	-1,665791776	0,127939994
1561278_at	-	-1,537851782	-1,665791776	0,127939994
1561756_at	-	-1,537851782	-1,665791776	0,127939994
215613_at	ADAM12	-1,537851782	-1,665791776	0,127939994
1566538_at	-	-1,537851782	-1,665791776	0,127939994
243169_at	-	-1,537851782	-1,665791776	0,127939994
220585_at	HKDC1	-1,537851782	-1,665791776	0,127939994
224170_s_at	TULP4	-1,537851782	-1,665791776	0,127939994
224422_x_at	PMCHL2	-1,537851782	-1,665791776	0,127939994
230040_at	ADAMTS18	-1,537851782	-1,665791776	0,127939994
234526_at	OR51B6	-1,537851782	-1,665791776	0,127939994
204931_at	TCF21	-1,537851782	-1,665791776	0,127939994
1565242_at	TNXB	-1,537851782	-1,665791776	0,127939994
230266_at	RAB7B	-1,537851782	-1,665791776	0,127939994
239829_at	-	-1,537851782	-1,665791776	0,127939994
229978_at	SHISA9	-1,537851782	-1,665791776	0,127939994
237685_at	LOC339760	-1,537851782	-1,665791776	0,127939994
240400_at	-	-1,537851782	-1,665791776	0,127939994
1560563_at	-	-1,537851782	-1,665791776	0,127939994
1563073_at	-	-1,537851782	-1,665791776	0,127939994
208711_s_at	CCND1	-1,537851782	-1,665791776	0,127939994
228421_s_at	EFEMP1	-1,537851782	-1,665791776	0,127939994
234639_x_at	KRTAP9-8	-1,537851782	-1,665791776	0,127939994
243205_at	-	-1,537851782	-1,665791776	0,127939994
220105_at	RTDR1	-1,537851782	-1,665791776	0,127939994
240110_at	HMGCS2	-1,537851782	-1,665791776	0,127939994
215042_at	BMP6	-1,537851782	-1,665791776	0,127939994
231814_at	MUC12	-1,537851782	-1,665791776	0,127939994
219786_at	MTL5	-1,537851782	-1,665791776	0,127939994
212099_at	RHOB	-1,537851782	-1,665791776	0,127939994
236655_at	TPD52	1,544940605	1,417157926	0,127782679
212687_at	LIMS1	5,175469101	5,047761287	0,127707814
201410_at	PLEKHB2	4,258822873	4,131291507	0,127531366
217862_at	PIAS1	2,339581693	2,212304392	0,127277301
222126_at	AGFG2	0,900065578	0,772793615	0,127271963
1552426_a_at	TM2D3	3,688415399	3,561438401	0,126976998
225220_at	SNHG8	4,735706923	4,608785884	0,12692104
222824_at	NUDT5	3,986286762	3,859612828	0,126673934
1568925_at	MYLK3	-2,569309811	-2,695640852	0,12633104

1563396_x_at	-	-2,569309811	-2,695640852	0,12633104
224080_at	-	-2,569309811	-2,695640852	0,12633104
220837_at	-	-2,569309811	-2,695640852	0,12633104
1562566_at	TRAF3IP2-AS1	-2,569309811	-2,695640852	0,12633104
1568686_at	ATP8B5P	-2,569309811	-2,695640852	0,12633104
213880_at	LGR5	-2,569309811	-2,695640852	0,12633104
242910_x_at	-	-2,569309811	-2,695640852	0,12633104
1561362_at	-	-2,569309811	-2,695640852	0,12633104
1561264_at	-	-2,569309811	-2,695640852	0,12633104
244203_at	-	-2,569309811	-2,695640852	0,12633104
1553261_x_at	ALS2CR11	-2,569309811	-2,695640852	0,12633104
1563061_at	-	-2,569309811	-2,695640852	0,12633104
1567181_x_at	-	-2,569309811	-2,695640852	0,12633104
1562288_at	-	-2,569309811	-2,695640852	0,12633104
1569504_at	LILRB4	-2,569309811	-2,695640852	0,12633104
241926_s_at	ERG	-2,569309811	-2,695640852	0,12633104
237745_at	TSC22D1-AS1	-2,569309811	-2,695640852	0,12633104
207307_at	HTR2C	-2,569309811	-2,695640852	0,12633104
1562588_at	-	-2,569309811	-2,695640852	0,12633104
229916_at	ENPP6	-2,569309811	-2,695640852	0,12633104
229951_x_at	-	-2,569309811	-2,695640852	0,12633104
36612_at	FAM168A	1,493312008	1,367027643	0,126284365
227607_at	STAMBPL1	3,657457797	3,531489068	0,125968728
201096_s_at	ARF4	4,826403949	4,700442403	0,125961546
227903_x_at	TPGS1	0,491906512	0,366031214	0,125875298
243228_at	GTF3C4	0,491906512	0,366031214	0,125875298
233779_x_at	-	0,39105295	0,265475485	0,125577466
222236_s_at	ASAP3	-0,135065865	-0,260526297	0,125460432
230815_at	LOC389765	-0,135065865	-0,260526297	0,125460432
229716_at	-	0,791908897	0,666486137	0,12542276
1555972_s_at	FBXO28	-0,609040214	-0,734355396	0,125315182
210391_at	NR6A1	-0,609040214	-0,734355396	0,125315182
1556855_a_at	LOC283501	-0,609040214	-0,734355396	0,125315182
225380_at	PKDCC	-0,609040214	-0,734355396	0,125315182
233742_at	METTL22	-0,609040214	-0,734355396	0,125315182
244426_at	-	-0,609040214	-0,734355396	0,125315182
231547_at	ZBTB37	-0,609040214	-0,734355396	0,125315182
220008_at	PEAK1	-0,609040214	-0,734355396	0,125315182
222675_s_at	BAIAP2L1	-0,609040214	-0,734355396	0,125315182
232183_at	SERAC1	0,926325262	0,80114751	0,125177752
229592_at	LOC100507501	0,926325262	0,80114751	0,125177752
203428_s_at	ASF1A	5,231074726	5,106012574	0,125062152
89476_r_at	NPEPL1	0,510623698	0,385699907	0,12492379
225136_at	PLEKHA2	5,122611534	4,99775782	0,124853714
201002_s_at	TMEM189 /// TME	5,145580394	5,020855028	0,124725366
234322_at	-	-0,230432956	-0,354781582	0,124348626
220216_at	C8orf44	-0,230432956	-0,354781582	0,124348626
1563344_at	-	-0,230432956	-0,354781582	0,124348626
212341_at	YIPF6	3,374552989	3,250322783	0,124230206
1562013_a_at	LOC100506730	1,640193838	1,516067292	0,124126547

216944_s_at	ITPR1	2,552836808	2,428719338	0,12411747
201371_s_at	CUL3	5,170477358	5,046390176	0,124087182
217597_x_at	RAB40B	0,707657549	0,583607503	0,124050045
235831_at	-	-2,708365344	-2,832036647	0,123671303
1560424_at	-	-2,708365344	-2,832036647	0,123671303
1566337_x_at	CHML	-2,708365344	-2,832036647	0,123671303
212191_x_at	RPL13 /// SNORD6	8,101573382	7,977931234	0,123642149
222029_x_at	PFDN6	3,636292808	3,512665066	0,123627743
217848_s_at	PPA1	7,219992498	7,096664191	0,123328307
202167_s_at	MMS19	2,285848435	2,162541631	0,123306804
232032_x_at	SDF4	4,070902947	3,947720163	0,123182785
210570_x_at	MAPK9	3,029186714	2,906008589	0,123178125
225556_at	VMA21	4,038663603	3,915686746	0,122976857
224303_x_at	NIN	0,820160788	0,697221375	0,122939413
211157_at	-	0,08619576	-0,036543687	0,122739447
1556449_at	-	0,08619576	-0,036543687	0,122739447
227393_at	ANO9	0,08619576	-0,036543687	0,122739447
224950_at	PTGFRN	0,08619576	-0,036543687	0,122739447
209817_at	PPP3CB	2,392346173	2,269637658	0,122708515
213607_x_at	NADK	1,094118704	0,971607464	0,122511239
203495_at	LRRC14	1,094118704	0,971607464	0,122511239
208392_x_at	SP110	1,094118704	0,971607464	0,122511239
219183_s_at	CYTH4	1,246401134	1,123905343	0,122495791
203947_at	CSTF3	3,694479488	3,572465199	0,122014289
213453_x_at	GAPDH	8,383976149	8,262416008	0,121560141
225837_at	C12orf32	3,735523399	3,614020495	0,121502904
212168_at	RBM12	3,7799838	3,658492103	0,121491697
233208_x_at	CPSF2	2,51558601	2,394276989	0,121309021
209285_s_at	FAM208A	3,257413106	3,136334485	0,121078621
228363_at	XIAP	3,35759121	3,236954658	0,120636552
226300_at	MED19	3,537317882	3,416886612	0,12043127
209919_x_at	GGT1 /// GGT2 ///	2,671492308	2,551398837	0,120093471
213878_at	PYROXD1	3,127551022	3,007560601	0,119990421
224129_s_at	DPY30	5,525224248	5,405404754	0,119819495
223420_at	DNAJC14	2,452674045	2,332940758	0,119733287
228695_at	C8orf46	-2,416625063	-2,536339282	0,119714218
228598_at	DPP10	-2,416625063	-2,536339282	0,119714218
232578_at	CLDN18	-2,416625063	-2,536339282	0,119714218
207901_at	IL12B	-2,416625063	-2,536339282	0,119714218
1560746_at	NEXN-AS1	-2,416625063	-2,536339282	0,119714218
241296_at	-	-2,416625063	-2,536339282	0,119714218
201505_at	LAMB1	-2,416625063	-2,536339282	0,119714218
1568660_a_at	LOC100506172	-2,416625063	-2,536339282	0,119714218
230344_x_at	-	-2,416625063	-2,536339282	0,119714218
244680_at	GLRB	-2,416625063	-2,536339282	0,119714218
1556529_a_at	LOC285326	-2,416625063	-2,536339282	0,119714218
1563690_at	PPP4R1	-2,416625063	-2,536339282	0,119714218
237911_at	-	-2,416625063	-2,536339282	0,119714218
1570396_at	FAM66C	-2,416625063	-2,536339282	0,119714218
235892_at	-	-2,416625063	-2,536339282	0,119714218

1558813_at	TMED5	-2,416625063	-2,536339282	0,119714218
206884_s_at	SCEL	-2,416625063	-2,536339282	0,119714218
241432_at	SLIT2-IT1	-2,416625063	-2,536339282	0,119714218
1563456_at	CCDC141	-2,416625063	-2,536339282	0,119714218
234678_at	KRTAP4-3	-2,416625063	-2,536339282	0,119714218
224112_at	-	-2,416625063	-2,536339282	0,119714218
231438_x_at	-	-2,416625063	-2,536339282	0,119714218
240858_at	-	-2,416625063	-2,536339282	0,119714218
234540_at	GCNT7	-2,416625063	-2,536339282	0,119714218
224274_at	FSCB	-2,416625063	-2,536339282	0,119714218
1560330_at	PCDH15	-2,416625063	-2,536339282	0,119714218
1552816_at	NXNL2	-2,416625063	-2,536339282	0,119714218
239461_at	GALNTL2	-2,416625063	-2,536339282	0,119714218
230746_s_at	-	-2,416625063	-2,536339282	0,119714218
207109_at	POU2F3	-2,416625063	-2,536339282	0,119714218
213436_at	CNR1	-2,416625063	-2,536339282	0,119714218
207168_s_at	H2AFY	6,114380779	5,994718012	0,119662766
209607_x_at	SLX1A-SULT1A3 //,	2,031158928	1,911502437	0,119656491
203465_at	MRPL19	3,816747242	3,697224187	0,119523055
219375_at	CEPT1	3,886513476	3,767104697	0,11940878
212579_at	SMCHD1	3,278243793	3,158887039	0,119356754
243407_at	MFSD8	-0,730013898	-0,84928999	0,119276092
234890_at	LOC100288175	-0,730013898	-0,84928999	0,119276092
1565556_at	LOC127841	-0,730013898	-0,84928999	0,119276092
235021_at	KIAA2026	-0,730013898	-0,84928999	0,119276092
223850_at	-	-0,730013898	-0,84928999	0,119276092
204310_s_at	NPR2	-0,730013898	-0,84928999	0,119276092
1556806_at	-	-0,730013898	-0,84928999	0,119276092
228076_s_at	NOL6	-0,730013898	-0,84928999	0,119276092
234626_at	OR51I1	-0,730013898	-0,84928999	0,119276092
233686_at	ASXL3	-0,730013898	-0,84928999	0,119276092
1553525_at	NLRP13	-0,730013898	-0,84928999	0,119276092
239276_at	-	-0,730013898	-0,84928999	0,119276092
1560897_a_at	KRTAP10-11	-0,730013898	-0,84928999	0,119276092
53991_at	DENND2A	-1,847397512	-1,966324048	0,118926536
204904_at	GJA4	-0,332405896	-0,45121326	0,118807364
202701_at	BMP1	-0,332405896	-0,45121326	0,118807364
219624_at	BAG4	0,524545436	0,405752839	0,118792598
230853_at	-	0,524545436	0,405752839	0,118792598
223321_s_at	FGFRL1	0,524545436	0,405752839	0,118792598
204651_at	NRF1	1,544940605	1,426280426	0,118660179
227649_s_at	SRGAP2	1,139057614	1,020514937	0,118542678
202449_s_at	RXRA	2,460890032	2,34250725	0,118382782
203598_s_at	WBP4	2,392346173	2,274084398	0,118261775
232367_x_at	ZNF598	0,735999505	0,61779983	0,118199675
201833_at	HDAC2	5,219908865	5,101837964	0,1180709
209517_s_at	ASH2L	4,584332287	4,466269902	0,118062386
225252_at	SRXN1	2,62869474	2,5107204	0,117974339
217144_at	-	3,345120948	3,227213858	0,11790709
219699_at	LGI2	0,001114523	-0,116767475	0,117881998

204022_at	WWP2	1,625482993	1,508107415	0,117375578
235125_x_at	FAM73A	1,625482993	1,508107415	0,117375578
201782_s_at	AIP	4,409540371	4,292243424	0,117296947
241474_at	-	-0,437449947	-0,554696666	0,117246719
1555380_at	ADAMTS4	-0,437449947	-0,554696666	0,117246719
211469_s_at	CXCR6	-0,437449947	-0,554696666	0,117246719
235298_at	WDR27	-0,437449947	-0,554696666	0,117246719
222345_at	-	-0,437449947	-0,554696666	0,117246719
1557589_a_at	-	-0,437449947	-0,554696666	0,117246719
215910_s_at	FNDC3A	-0,437449947	-0,554696666	0,117246719
216019_x_at	PHLDB1	-0,437449947	-0,554696666	0,117246719
202634_at	POLR2K	3,909506676	3,792342525	0,117164151
231241_at	-	1,000459215	0,883374248	0,117084966
1557055_s_at	LOC643837	3,029186714	2,912181979	0,117004736
202568_s_at	MARK3	3,029186714	2,912181979	0,117004736
202954_at	UBE2C	5,676287296	5,559411291	0,116876005
223053_x_at	SSU72	3,517785666	3,401110525	0,116675141
216467_s_at	-	-0,856259172	-0,972893339	0,116634167
240164_at	-	-0,856259172	-0,972893339	0,116634167
230979_at	-	-0,856259172	-0,972893339	0,116634167
244438_at	-	-0,856259172	-0,972893339	0,116634167
1554911_at	ABCC11	-0,856259172	-0,972893339	0,116634167
239582_at	PML	-0,856259172	-0,972893339	0,116634167
224449_at	DDI2 /// RSC1A1	-0,856259172	-0,972893339	0,116634167
205595_at	DSG3	-0,856259172	-0,972893339	0,116634167
234747_at	TRAPPC11	-0,856259172	-0,972893339	0,116634167
1558594_at	ZNF219	-0,856259172	-0,972893339	0,116634167
211107_s_at	AURKC	-0,856259172	-0,972893339	0,116634167
239590_x_at	FAM95B1 /// LOC1	-0,856259172	-0,972893339	0,116634167
242750_at	MMAA	-0,856259172	-0,972893339	0,116634167
218185_s_at	ARMC1	4,466417603	4,349844515	0,116573088
218386_x_at	USP16	3,881435528	3,765267919	0,116167609
226137_at	ZFHX3	1,345611839	1,2298509	0,115760939
225261_x_at	TH1L	4,355700522	4,239953834	0,115746688
201282_at	OGDH	2,085817135	1,970106792	0,115710342
208265_at	C2orf83	-1,452713826	-1,568385657	0,115671831
230953_at	LOC100286922	-1,452713826	-1,568385657	0,115671831
238976_at	-	-1,452713826	-1,568385657	0,115671831
238834_at	MYLK3	-1,452713826	-1,568385657	0,115671831
235083_at	LOC151009	-1,452713826	-1,568385657	0,115671831
234809_at	ZC4H2	-1,452713826	-1,568385657	0,115671831
1563553_at	-	-1,452713826	-1,568385657	0,115671831
1559148_at	-	-1,452713826	-1,568385657	0,115671831
237679_at	TRIM66	-1,452713826	-1,568385657	0,115671831
224191_x_at	ROPN1	-1,452713826	-1,568385657	0,115671831
228329_at	DAB1	-1,452713826	-1,568385657	0,115671831
208256_at	EFNA2	-1,452713826	-1,568385657	0,115671831
1562802_at	LINC00210	-1,452713826	-1,568385657	0,115671831
229296_at	LOC100506119	-1,452713826	-1,568385657	0,115671831
210353_s_at	SLC6A2	-1,452713826	-1,568385657	0,115671831

231601_at	LOC100507224	-1,452713826	-1,568385657	0,115671831
1565768_at	ZNF268	-1,452713826	-1,568385657	0,115671831
233495_at	EXOSC3	-1,452713826	-1,568385657	0,115671831
240842_at	-	-1,452713826	-1,568385657	0,115671831
1570089_at	-	-1,452713826	-1,568385657	0,115671831
1569615_at	-	-1,452713826	-1,568385657	0,115671831
1564150_a_at	LOC256021	-1,452713826	-1,568385657	0,115671831
229688_at	PRDM11	-1,452713826	-1,568385657	0,115671831
208330_at	ALX4	-1,452713826	-1,568385657	0,115671831
237478_at	FTH1	-1,452713826	-1,568385657	0,115671831
200003_s_at	RPL28	8,373557928	8,258161394	0,115396534
228642_at	HOTAIRM1	1,364555912	1,249194639	0,115361273
202396_at	TCERG1	4,339857197	4,224659944	0,115197254
1554575_a_at	BPNT1	1,816938997	1,701800759	0,115138238
217950_at	NOSIP	3,691400566	3,576411191	0,114989375
219561_at	COPZ2	0,648195588	0,533258442	0,114937146
224876_at	C5orf24	3,886513476	3,771650587	0,11486289
224027_at	CCL28	-1,212652659	-1,32740143	0,114748771
1561375_at	-	-1,212652659	-1,32740143	0,114748771
1564407_a_at	-	-1,212652659	-1,32740143	0,114748771
233232_at	RHBDL3	-1,212652659	-1,32740143	0,114748771
1567101_at	-	-1,212652659	-1,32740143	0,114748771
206260_at	TGM4	-1,212652659	-1,32740143	0,114748771
231171_at	-	-1,212652659	-1,32740143	0,114748771
238815_at	LRRTM1	-1,212652659	-1,32740143	0,114748771
1561499_at	-	-1,212652659	-1,32740143	0,114748771
209637_s_at	RGS12	-1,212652659	-1,32740143	0,114748771
237824_at	-	-1,212652659	-1,32740143	0,114748771
231162_at	CLDND2	-1,212652659	-1,32740143	0,114748771
234333_at	KIAA2022	-1,212652659	-1,32740143	0,114748771
207083_s_at	CC2D1A	-1,212652659	-1,32740143	0,114748771
233145_at	CSMD2	-1,212652659	-1,32740143	0,114748771
232942_at	PYROXD2	-1,212652659	-1,32740143	0,114748771
237030_at	ACPP	-1,212652659	-1,32740143	0,114748771
221723_s_at	SLC4A5	-1,212652659	-1,32740143	0,114748771
232289_at	KCNJ12	-1,212652659	-1,32740143	0,114748771
240696_at	-	-1,212652659	-1,32740143	0,114748771
233592_at	ANKRD18B	-1,212652659	-1,32740143	0,114748771
207278_s_at	CD209	-1,212652659	-1,32740143	0,114748771
204092_s_at	AURKA	4,677091148	4,562409099	0,114682049
208980_s_at	UBC	7,229363929	7,11475656	0,114607368
231517_at	ZYG11A	1,730929079	1,616354037	0,114575042
232463_at	PPP2R3B-AS1	0,127732831	0,013190398	0,114542433
211313_s_at	BAZ1B	0,127732831	0,013190398	0,114542433
203586_s_at	ARL4D	0,127732831	0,013190398	0,114542433
231487_at	COX8C	-1,982324124	-2,09678811	0,114463986
1563203_at	-	-1,982324124	-2,09678811	0,114463986
209395_at	CHI3L1	-1,982324124	-2,09678811	0,114463986
1570076_at	-	-1,982324124	-2,09678811	0,114463986
226484_at	ZBTB47	-1,982324124	-2,09678811	0,114463986

237066_at	-	-1,982324124	-2,09678811	0,114463986
232575_at	PCA3	-1,982324124	-2,09678811	0,114463986
216084_at	FAM205B	-1,982324124	-2,09678811	0,114463986
1569203_at	CXCL2	-1,982324124	-2,09678811	0,114463986
1560087_a_at	-	-1,982324124	-2,09678811	0,114463986
221396_at	TAS2R7	-1,982324124	-2,09678811	0,114463986
242845_at	RUNX1T1	-1,982324124	-2,09678811	0,114463986
208329_at	PBOV1	-1,982324124	-2,09678811	0,114463986
1552514_at	WBP2NL	-1,982324124	-2,09678811	0,114463986
239121_at	-	-1,982324124	-2,09678811	0,114463986
1564253_at	LOC285766	-1,982324124	-2,09678811	0,114463986
214068_at	BEAN1	-1,982324124	-2,09678811	0,114463986
1552412_a_at	DEFB106A /// DEFI	-1,982324124	-2,09678811	0,114463986
235129_at	PPP1R1A	-1,982324124	-2,09678811	0,114463986
1561250_at	-	-1,982324124	-2,09678811	0,114463986
1556190_s_at	LOC100507516 ///	-1,982324124	-2,09678811	0,114463986
217280_x_at	GABRA5	-1,982324124	-2,09678811	0,114463986
207464_at	AHCYL1	-1,982324124	-2,09678811	0,114463986
222484_s_at	CXCL14	-1,982324124	-2,09678811	0,114463986
234197_at	ACSM1	-1,982324124	-2,09678811	0,114463986
1567250_at	OR10A3	-1,982324124	-2,09678811	0,114463986
240930_at	CRAMP1L	-1,982324124	-2,09678811	0,114463986
208207_at	-	-1,982324124	-2,09678811	0,114463986
226575_at	ZNF462	-1,982324124	-2,09678811	0,114463986
1569944_at	-	-1,982324124	-2,09678811	0,114463986
229345_at	RTN4RL2	-1,982324124	-2,09678811	0,114463986
200665_s_at	LOC100505813 ///	-1,982324124	-2,09678811	0,114463986
241850_at	-	-1,982324124	-2,09678811	0,114463986
238095_at	GATA5	-1,982324124	-2,09678811	0,114463986
1553508_at	MDS2	-1,982324124	-2,09678811	0,114463986
228609_at	MIR1302-10 /// M	-1,982324124	-2,09678811	0,114463986
244853_at	-	-1,982324124	-2,09678811	0,114463986
243455_at	-	-1,982324124	-2,09678811	0,114463986
223997_at	FNIP1	-1,982324124	-2,09678811	0,114463986
1554612_at	KIAA0226	-1,982324124	-2,09678811	0,114463986
240896_at	-	-1,982324124	-2,09678811	0,114463986
1554692_at	SLC23A2	-1,982324124	-2,09678811	0,114463986
243466_at	-	-1,982324124	-2,09678811	0,114463986
221661_at	SLC22A7	-1,982324124	-2,09678811	0,114463986
232864_s_at	AFF4	-1,982324124	-2,09678811	0,114463986
1563154_at	-	-1,982324124	-2,09678811	0,114463986
232745_x_at	SPEF2	-1,982324124	-2,09678811	0,114463986
201471_s_at	SQSTM1	3,498020785	3,383632552	0,114388233
228999_at	CHD2	1,871521385	1,757155322	0,114366063
203201_at	PMM2	2,276736537	2,162541631	0,114194906
220355_s_at	PBRM1	3,588695979	3,474637573	0,114058405
232504_at	LOC285628 /// MI	2,41945542	2,305621062	0,113834358
238020_at	PSMC2	2,657308324	2,543655878	0,113652446
219828_at	RABL6	0,556428218	0,443143223	0,113284995
202157_s_at	CELF2	5,501100021	5,387977657	0,113122364

31835_at	HRG	-1,800290068	-1,913212441	0,112922373
201784_s_at	C11orf58	5,466491247	5,353578761	0,112912486
242640_at	C19orf55	-0,088974936	-0,201789721	0,112814785
220617_s_at	ZNF532	-0,088974936	-0,201789721	0,112814785
239253_at	-	-0,088974936	-0,201789721	0,112814785
1562323_at	-	-0,088974936	-0,201789721	0,112814785
205717_x_at	PCDHGA1 /// PCDI	-0,088974936	-0,201789721	0,112814785
211865_s_at	FZR1	-0,088974936	-0,201789721	0,112814785
217994_x_at	CPSF3L	2,966903803	2,854304969	0,112598835
209253_at	SORBS3	1,225123479	1,112548615	0,112574864
34031_i_at	KRIT1	2,784898907	2,672497494	0,112401414
225872_at	SLC35F5	2,295108393	2,182711118	0,112397275
216484_x_at	HDGF	3,6071174	3,494805562	0,112311838
218100_s_at	IFT57	3,592169709	3,479912486	0,112257223
210417_s_at	PI4KB	1,92317941	1,811004388	0,112175022
38964_r_at	WAS	4,930088702	4,818108589	0,111980113
1557186_s_at	TPCN1	0,458905032	0,346964736	0,111940296
243875_at	CCDC158	-2,074223907	-2,186086603	0,111862696
206426_at	MLANA	-2,074223907	-2,186086603	0,111862696
228502_at	RPS6KA6	-2,074223907	-2,186086603	0,111862696
220793_at	SAGE1	-2,074223907	-2,186086603	0,111862696
1561297_at	WARS2	-2,074223907	-2,186086603	0,111862696
1555011_at	ZFYVE16	-2,074223907	-2,186086603	0,111862696
202966_at	CAPN6	-2,074223907	-2,186086603	0,111862696
210979_at	ASAH1	-2,074223907	-2,186086603	0,111862696
1553876_at	SAMD3	-2,074223907	-2,186086603	0,111862696
221174_at	-	-2,074223907	-2,186086603	0,111862696
238184_at	-	-2,074223907	-2,186086603	0,111862696
207200_at	OTC	-2,074223907	-2,186086603	0,111862696
220530_at	-	-2,074223907	-2,186086603	0,111862696
1570189_at	LOC388387	-2,074223907	-2,186086603	0,111862696
1570024_at	-	-2,074223907	-2,186086603	0,111862696
242328_at	RAB3C	-2,074223907	-2,186086603	0,111862696
207008_at	CXCR2	-2,074223907	-2,186086603	0,111862696
236797_at	-	-2,074223907	-2,186086603	0,111862696
1557856_at	-	-2,074223907	-2,186086603	0,111862696
224077_at	WHSC1L1	-2,074223907	-2,186086603	0,111862696
214577_at	MAP1B	-2,074223907	-2,186086603	0,111862696
1562669_at	-	-2,074223907	-2,186086603	0,111862696
243515_at	-	-2,074223907	-2,186086603	0,111862696
1566124_a_at	FABP6	-2,074223907	-2,186086603	0,111862696
216007_at	-	-2,074223907	-2,186086603	0,111862696
239906_at	-	-2,074223907	-2,186086603	0,111862696
215034_s_at	TM4SF1	-2,074223907	-2,186086603	0,111862696
1562752_at	-	-2,074223907	-2,186086603	0,111862696
1559322_at	LOC727916	-2,074223907	-2,186086603	0,111862696
208173_at	IFNB1	-2,074223907	-2,186086603	0,111862696
236491_at	BCL2L10	-2,074223907	-2,186086603	0,111862696
230422_at	FPR3	-2,074223907	-2,186086603	0,111862696
215808_at	KLK10	-2,074223907	-2,186086603	0,111862696

1570534_a_at	ZNF483	-2,074223907	-2,186086603	0,111862696
1553541_at	LMX1A	-2,074223907	-2,186086603	0,111862696
238168_at	TM4SF1	-2,074223907	-2,186086603	0,111862696
1567240_x_at	OR2L2	-2,074223907	-2,186086603	0,111862696
1557674_s_at	EFCAB2	-2,074223907	-2,186086603	0,111862696
229401_at	IL17RE	-2,074223907	-2,186086603	0,111862696
230258_at	GLIS3	-2,074223907	-2,186086603	0,111862696
218545_at	CCDC91	2,978070706	2,86626857	0,111802136
228183_s_at	RPAIN	3,822956033	3,711313563	0,11164247
211345_x_at	EEF1G /// MIR365	8,094277974	7,982681107	0,111596866
228130_at	ZSCAN30	0,244681185	0,133104519	0,111576666
225234_at	CBL	2,484824532	2,373249966	0,111574566
242274_x_at	SLC25A42	-1,893506789	-2,005029581	0,111522792
1562380_at	-	-1,893506789	-2,005029581	0,111522792
1553228_at	CCDC89	-1,893506789	-2,005029581	0,111522792
1555894_s_at	MTSS1L	-1,893506789	-2,005029581	0,111522792
1558661_at	LOC100506207	-1,893506789	-2,005029581	0,111522792
243087_at	WDR63	-1,893506789	-2,005029581	0,111522792
216989_at	SPAM1	-1,893506789	-2,005029581	0,111522792
1569731_at	-	-1,893506789	-2,005029581	0,111522792
238411_x_at	NREP	-1,893506789	-2,005029581	0,111522792
227310_at	ADSS	-1,893506789	-2,005029581	0,111522792
1558163_at	PEX13	-1,893506789	-2,005029581	0,111522792
216592_at	MAGEC3	-1,893506789	-2,005029581	0,111522792
229724_at	GABRB3	-1,893506789	-2,005029581	0,111522792
244188_at	-	-1,893506789	-2,005029581	0,111522792
1561677_at	MACROD2	-1,893506789	-2,005029581	0,111522792
232692_at	TDRD6	-1,893506789	-2,005029581	0,111522792
226612_at	UBE2QL1	-1,893506789	-2,005029581	0,111522792
216635_at	-	-1,893506789	-2,005029581	0,111522792
217103_at	LDLR	-1,893506789	-2,005029581	0,111522792
215847_at	HERC2P3	-1,893506789	-2,005029581	0,111522792
1570135_at	ZNF230	-1,893506789	-2,005029581	0,111522792
239460_at	LOC100652954	-1,893506789	-2,005029581	0,111522792
1556402_at	-	-1,893506789	-2,005029581	0,111522792
244452_at	ERLEC1	-1,893506789	-2,005029581	0,111522792
232122_s_at	VEPH1	-1,893506789	-2,005029581	0,111522792
215264_at	EMX1	-1,893506789	-2,005029581	0,111522792
1561407_at	ARAP2	-1,893506789	-2,005029581	0,111522792
231214_at	-	-1,893506789	-2,005029581	0,111522792
236759_at	-	-1,893506789	-2,005029581	0,111522792
206514_s_at	CYP4F2 /// CYP4F3	-1,893506789	-2,005029581	0,111522792
1553375_at	BTBD9	-1,893506789	-2,005029581	0,111522792
241821_at	-	-1,893506789	-2,005029581	0,111522792
210026_s_at	CARD10	-1,893506789	-2,005029581	0,111522792
220436_at	LOC441402	-1,893506789	-2,005029581	0,111522792
1564949_at	-	-1,893506789	-2,005029581	0,111522792
243034_at	-	-1,893506789	-2,005029581	0,111522792
1565780_at	ABCA8	-1,893506789	-2,005029581	0,111522792
243033_at	TWF1	-1,893506789	-2,005029581	0,111522792

239126_at	CIRBP-AS1	-1,893506789	-2,005029581	0,111522792
1562316_at	-	-1,893506789	-2,005029581	0,111522792
240495_at	-	-1,893506789	-2,005029581	0,111522792
1555515_a_at	FAM189B	-1,893506789	-2,005029581	0,111522792
238134_at	-	-1,893506789	-2,005029581	0,111522792
1554337_at	FARP2	-1,893506789	-2,005029581	0,111522792
244297_at	ANKRD18A	-1,893506789	-2,005029581	0,111522792
242535_at	-	-1,893506789	-2,005029581	0,111522792
228872_at	FAM108B1	-1,893506789	-2,005029581	0,111522792
211399_at	FGFR2	-1,893506789	-2,005029581	0,111522792
223557_s_at	TMEFF2	-1,893506789	-2,005029581	0,111522792
217961_at	SLC25A38	3,340803206	3,229534127	0,11126908
229984_at	DTWD1	0,355751005	0,244646697	0,111104308
223106_at	TMEM14C	5,399531502	5,288621916	0,110909586
201103_x_at	LOC100506032 ///	5,483874276	5,372993635	0,110880641
217132_at	-	-2,641232718	-2,752097204	0,110864486
219995_s_at	ZNF750	-2,641232718	-2,752097204	0,110864486
244567_at	-	-2,641232718	-2,752097204	0,110864486
1555939_at	-	-2,641232718	-2,752097204	0,110864486
206893_at	SALL1	-2,641232718	-2,752097204	0,110864486
1552906_at	FMR1NB	-2,641232718	-2,752097204	0,110864486
1554526_at	OLFM3	-2,641232718	-2,752097204	0,110864486
228335_at	CLDN11	-2,641232718	-2,752097204	0,110864486
237566_at	-	-2,641232718	-2,752097204	0,110864486
215494_at	-	-2,641232718	-2,752097204	0,110864486
1556497_a_at	-	-2,641232718	-2,752097204	0,110864486
1554506_x_at	NAALAD2	-2,641232718	-2,752097204	0,110864486
230554_at	ACSM2A	-2,641232718	-2,752097204	0,110864486
225114_at	AGPS	2,71254166	2,601822502	0,110719158
223478_at	TIMM8B	2,765070895	2,654387125	0,11068377
235878_at	TAF1B	1,267146969	1,156599976	0,110546993
228830_s_at	ATF7	1,561816652	1,451507543	0,11030911
238468_at	TNRC6B	1,094118704	0,98381571	0,110302994
212340_at	YIPF6	1,094118704	0,98381571	0,110302994
1554472_a_at	PHF20L1	2,074841895	1,964670566	0,110171329
212342_at	YIPF6	0,791908897	0,681874787	0,110034111
205688_at	TFAP4	0,791908897	0,681874787	0,110034111
1554537_at	TMEM126B	-0,551284523	-0,661178575	0,109894052
236271_at	-	-0,551284523	-0,661178575	0,109894052
209766_at	PRDX3	-0,551284523	-0,661178575	0,109894052
240590_at	LOC348761	-0,551284523	-0,661178575	0,109894052
202445_s_at	NOTCH2	-0,551284523	-0,661178575	0,109894052
204372_s_at	KHSRP	3,988952858	3,879188394	0,109764464
218135_at	ERGIC2	4,514001815	4,404237854	0,109763961
1557754_at	LOC401068	-0,180990326	-0,290694975	0,109704649
231306_at	LYZL4	-0,180990326	-0,290694975	0,109704649
234188_at	-	-0,180990326	-0,290694975	0,109704649
1553642_at	C9orf163	-0,180990326	-0,290694975	0,109704649
221870_at	EHD2	-0,180990326	-0,290694975	0,109704649
214041_x_at	RPL37A	2,745538988	2,635864822	0,109674166

224164_at	TPM3	0,045517965	-0,063377083	0,108895048
226144_at	REXO1	0,045517965	-0,063377083	0,108895048
212639_x_at	LOC100288366 ///	7,984936617	7,876102246	0,10883437
1559633_a_at	CHRM3	0,58788177	0,479089184	0,108792586
210210_at	MPZL1	0,58788177	0,479089184	0,108792586
232019_at	ZKSCAN2	0,58788177	0,479089184	0,108792586
203926_x_at	ATP5D	1,760255626	1,651676651	0,108578975
224623_at	LOC728554 /// TH	3,966733349	3,858181176	0,108552173
232429_at	-	-1,802716385	-1,911206569	0,108490184
239580_at	GUCY1A3	-1,802716385	-1,911206569	0,108490184
221337_s_at	ADAM29	-1,802716385	-1,911206569	0,108490184
1561146_at	VPS35	-1,802716385	-1,911206569	0,108490184
1554813_at	-	-1,802716385	-1,911206569	0,108490184
1559595_at	LOC728084	-1,802716385	-1,911206569	0,108490184
235018_at	CSRNP3	-1,802716385	-1,911206569	0,108490184
230088_at	-	-1,802716385	-1,911206569	0,108490184
207062_at	IAPP	-1,802716385	-1,911206569	0,108490184
1555749_at	SF1	-1,802716385	-1,911206569	0,108490184
228699_at	NRP2	-1,802716385	-1,911206569	0,108490184
233640_x_at	KRTAP9-4	-1,802716385	-1,911206569	0,108490184
238085_at	LOC100507653	-1,802716385	-1,911206569	0,108490184
241258_at	-	-1,802716385	-1,911206569	0,108490184
215133_s_at	FAM153A /// FAM	-1,802716385	-1,911206569	0,108490184
1568882_at	LRTOMT	-1,802716385	-1,911206569	0,108490184
207632_at	MUSK	-1,802716385	-1,911206569	0,108490184
233716_at	-	-1,802716385	-1,911206569	0,108490184
202619_s_at	PLOD2	-1,802716385	-1,911206569	0,108490184
234146_at	B3GAT2	-1,802716385	-1,911206569	0,108490184
215918_s_at	SPTBN1	-1,802716385	-1,911206569	0,108490184
1570272_at	-	-1,802716385	-1,911206569	0,108490184
230162_s_at	MAPK8IP3	-1,802716385	-1,911206569	0,108490184
243870_at	-	-1,802716385	-1,911206569	0,108490184
226900_at	GABPB1-AS1	-1,802716385	-1,911206569	0,108490184
233294_at	DENND2C	-1,802716385	-1,911206569	0,108490184
219744_at	FN3K	-1,802716385	-1,911206569	0,108490184
1563635_at	-	-1,802716385	-1,911206569	0,108490184
1561348_at	LOC339874	-1,802716385	-1,911206569	0,108490184
215314_at	-	-1,802716385	-1,911206569	0,108490184
241015_at	-	-1,802716385	-1,911206569	0,108490184
229487_at	EBF1	-1,802716385	-1,911206569	0,108490184
1564559_at	LOC728073	-1,802716385	-1,911206569	0,108490184
1555340_x_at	RAP1A	-1,802716385	-1,911206569	0,108490184
208726_s_at	EIF2S2	6,739757106	6,631279991	0,108477115
208541_x_at	TFAM	2,339581693	2,23115548	0,108426213
49327_at	SIRT3	1,742538245	1,634157171	0,108381074
218274_s_at	ANKZF1	1,995527416	1,887158627	0,108368789
1561280_at	LEMD1-AS1	-2,495152701	-2,603471267	0,108318566
1565799_at	RAB3IP	-2,495152701	-2,603471267	0,108318566
1567182_at	-	-2,495152701	-2,603471267	0,108318566
206418_at	NOX1	-2,495152701	-2,603471267	0,108318566

238399_x_at	-	-2,495152701	-2,603471267	0,108318566
233793_at	-	-2,495152701	-2,603471267	0,108318566
228128_x_at	PAPPA	-2,495152701	-2,603471267	0,108318566
227803_at	ENPP5	-2,495152701	-2,603471267	0,108318566
241698_at	RFTN2	-2,495152701	-2,603471267	0,108318566
244314_at	-	-2,495152701	-2,603471267	0,108318566
240787_at	-	-2,495152701	-2,603471267	0,108318566
214586_at	GPR37	-2,495152701	-2,603471267	0,108318566
1563133_at	-	-2,495152701	-2,603471267	0,108318566
224237_at	-	-2,495152701	-2,603471267	0,108318566
232132_at	PARD6G	-2,495152701	-2,603471267	0,108318566
215303_at	DCLK1	-2,495152701	-2,603471267	0,108318566
240865_at	-	-2,495152701	-2,603471267	0,108318566
226847_at	FST	-2,495152701	-2,603471267	0,108318566
235617_x_at	LOC100507537	-2,495152701	-2,603471267	0,108318566
234185_at	-	-2,495152701	-2,603471267	0,108318566
238983_at	NSUN7	-2,495152701	-2,603471267	0,108318566
243562_at	KNG1	-2,495152701	-2,603471267	0,108318566
1563188_at	-	-2,495152701	-2,603471267	0,108318566
235373_at	LOC100506314	-2,495152701	-2,603471267	0,108318566
213936_x_at	SFTPB	0,820160788	0,711934445	0,108226343
200721_s_at	ACTR1A	1,802733148	1,694547353	0,108185794
228674_s_at	EML4	2,590269697	2,482273566	0,107996131
241364_at	TMEM57	1,788922638	1,680963921	0,107958717
218700_s_at	RAB7L1	1,788922638	1,680963921	0,107958717
217729_s_at	AES	2,732422196	2,624682607	0,10773959
226945_at	RHBDD1	1,830468611	1,722747778	0,107720834
217765_at	NRBP1	2,436165655	2,328492509	0,107673146
229678_at	LOC728431	0,707657549	0,600385088	0,107272461
201118_at	PGD	4,92247179	4,815598205	0,106873585
202989_at	RGS1	-2,163157732	-2,27001641	0,106858678
223838_at	TSGA10	-2,163157732	-2,27001641	0,106858678
1557717_at	LINC00550	-2,163157732	-2,27001641	0,106858678
237832_at	-	-2,163157732	-2,27001641	0,106858678
232263_at	SLC6A15	-2,163157732	-2,27001641	0,106858678
241998_at	C2orf80	-2,163157732	-2,27001641	0,106858678
234691_at	KRTAP2-1	-2,163157732	-2,27001641	0,106858678
1560453_at	-	-2,163157732	-2,27001641	0,106858678
1559879_a_at	-	-2,163157732	-2,27001641	0,106858678
238877_at	EYA4	-2,163157732	-2,27001641	0,106858678
1562307_at	-	-2,163157732	-2,27001641	0,106858678
211196_at	DBT	-2,163157732	-2,27001641	0,106858678
235284_s_at	LOC388692	-2,163157732	-2,27001641	0,106858678
244643_at	LOC100506652	-2,163157732	-2,27001641	0,106858678
233901_at	-	-2,163157732	-2,27001641	0,106858678
1562299_at	LOC100652860 ///	-2,163157732	-2,27001641	0,106858678
214618_at	CFLAR	-2,163157732	-2,27001641	0,106858678
220298_s_at	SPATA6	-2,163157732	-2,27001641	0,106858678
1552856_at	LINC00311	-2,163157732	-2,27001641	0,106858678
232202_at	-	-2,163157732	-2,27001641	0,106858678

1563742_at	LOC643650	-2,163157732	-2,27001641	0,106858678
228538_at	ZNF662	-2,163157732	-2,27001641	0,106858678
237298_at	FLJ26850	-2,163157732	-2,27001641	0,106858678
217245_at	EPAG	-2,163157732	-2,27001641	0,106858678
242897_at	-	-2,163157732	-2,27001641	0,106858678
1562811_at	-	-2,163157732	-2,27001641	0,106858678
240728_at	-	-2,163157732	-2,27001641	0,106858678
225792_at	HOOK1	-2,163157732	-2,27001641	0,106858678
241604_at	ATP11A	-2,163157732	-2,27001641	0,106858678
242980_at	COMMD10	-2,163157732	-2,27001641	0,106858678
230323_s_at	TMEM45B	-2,163157732	-2,27001641	0,106858678
226755_at	MIR205HG	-2,163157732	-2,27001641	0,106858678
1556377_s_at	LMO7	-2,163157732	-2,27001641	0,106858678
1552401_a_at	BACH1 /// GRIK1-A	-2,163157732	-2,27001641	0,106858678
223329_x_at	SUGT1	5,256557379	5,149745997	0,106811383
237128_at	-	-0,994186487	-1,100943374	0,106756887
211029_x_at	FGF18	-0,994186487	-1,100943374	0,106756887
221375_at	OR1G1	-0,994186487	-1,100943374	0,106756887
1563542_a_at	SCML4	-0,994186487	-1,100943374	0,106756887
1561436_at	-	-0,994186487	-1,100943374	0,106756887
1561409_at	-	-0,994186487	-1,100943374	0,106756887
231566_at	NOXRED1	-0,994186487	-1,100943374	0,106756887
231604_at	-	-0,994186487	-1,100943374	0,106756887
232610_at	PARP14	-0,994186487	-1,100943374	0,106756887
217405_x_at	GPLD1	-0,994186487	-1,100943374	0,106756887
235098_at	PEX26	-0,994186487	-1,100943374	0,106756887
215614_at	-	-0,994186487	-1,100943374	0,106756887
233613_x_at	REXO2	-0,994186487	-1,100943374	0,106756887
1558369_at	MPHOSPH9	2,2388565	2,132145748	0,106710752
244461_at	SPECC1	-0,280273599	-0,386896102	0,106622503
1569368_at	LOC283693	-0,280273599	-0,386896102	0,106622503
203937_s_at	TAF1C	-0,280273599	-0,386896102	0,106622503
204682_at	LTBP2	-0,280273599	-0,386896102	0,106622503
209588_at	EPHB2	-0,280273599	-0,386896102	0,106622503
1561571_at	LOC730139	-0,280273599	-0,386896102	0,106622503
212678_at	NF1	-0,280273599	-0,386896102	0,106622503
220691_at	-	0,491906512	0,385582632	0,10632388
219913_s_at	CRNKL1	2,649978302	2,543655878	0,106322425
200593_s_at	HNRNPU	4,45671768	4,350661368	0,106056312
91580_at	LRTM1	-2,480934008	-2,586902946	0,105968937
233703_x_at	-	-2,774503732	-2,880284116	0,105780383
238067_at	TBC1D8B	-2,774503732	-2,880284116	0,105780383
242802_x_at	-	-2,774503732	-2,880284116	0,105780383
1561450_at	-	-2,774503732	-2,880284116	0,105780383
224819_at	TCEAL8	1,92317941	1,817403386	0,105776024
1557352_at	SQLE	1,160996507	1,055317091	0,105679416
227074_at	LOC100131564	3,282538749	3,176868665	0,105670085
229129_at	HNRNPD	1,458255986	1,352622437	0,105633549
229468_at	CDK3 /// TEN1-CDI	1,364555912	1,259081178	0,105474734
219248_at	THUMPD2	2,38349585	2,278572464	0,104923387

210449_x_at	MAPK14	2,149438457	2,044580955	0,104857502
235967_at	LOC100127891	0,39105295	0,286279868	0,104773083
233506_at	-	0,39105295	0,286279868	0,104773083
209784_s_at	JAG2	0,282358733	0,178191865	0,104166868
203745_at	HCCS	2,427650693	2,323616375	0,104034319
209449_at	LSM2	3,825885656	3,722097358	0,103788298
221189_s_at	TARS2	2,21987062	2,116147513	0,103723108
226776_at	ENY2	1,000459215	0,896834102	0,103625113
226177_at	GLTP	4,510408465	4,407124345	0,10328412
201049_s_at	RPS18	8,493385064	8,390296134	0,10308893
205457_at	C6orf106	1,983901861	1,881145546	0,102756314
211759_x_at	TBCB	4,873110637	4,770476599	0,102634039
229410_at	SLC35E1	2,179708195	2,077419168	0,102289027
241731_x_at	ZNF440	-1,369772723	-1,471833228	0,102060505
225664_at	COL12A1	-1,369772723	-1,471833228	0,102060505
1570383_at	BMP1	-1,369772723	-1,471833228	0,102060505
232651_at	LOC100287813	-1,369772723	-1,471833228	0,102060505
208063_s_at	CAPN9	-1,369772723	-1,471833228	0,102060505
1561480_a_at	-	-1,369772723	-1,471833228	0,102060505
240332_at	-	-1,369772723	-1,471833228	0,102060505
244531_at	NNT	-1,369772723	-1,471833228	0,102060505
236394_at	A2MP1	-1,369772723	-1,471833228	0,102060505
236982_at	-	-1,369772723	-1,471833228	0,102060505
214081_at	PLXDC1	-1,369772723	-1,471833228	0,102060505
208064_s_at	ST8SIA3	-1,369772723	-1,471833228	0,102060505
1560279_a_at	LOC221122	-1,369772723	-1,471833228	0,102060505
233752_s_at	ZFHX3	-1,369772723	-1,471833228	0,102060505
227532_at	LRRC39	-1,369772723	-1,471833228	0,102060505
241797_at	-	-1,369772723	-1,471833228	0,102060505
1562933_at	-	-1,369772723	-1,471833228	0,102060505
231811_at	LOC100134445 ///	-1,369772723	-1,471833228	0,102060505
235751_s_at	VMO1	-1,369772723	-1,471833228	0,102060505
232871_at	-	-1,369772723	-1,471833228	0,102060505
208446_s_at	ZFYVE9	-1,369772723	-1,471833228	0,102060505
233286_at	-	-1,369772723	-1,471833228	0,102060505
1568843_at	TTLL13	-1,369772723	-1,471833228	0,102060505
216090_x_at	-	-1,369772723	-1,471833228	0,102060505
228102_at	-	-1,369772723	-1,471833228	0,102060505
234477_at	IGHA1 /// IGHV4-3	-1,369772723	-1,471833228	0,102060505
212776_s_at	OBSL1	-1,369772723	-1,471833228	0,102060505
209466_x_at	LOC100287705 ///	-1,369772723	-1,471833228	0,102060505
1555271_a_at	TERT	-1,369772723	-1,471833228	0,102060505
204439_at	IFI44L	-1,369772723	-1,471833228	0,102060505
1558544_at	C1orf86	-1,369772723	-1,471833228	0,102060505
1554006_a_at	LLGL2	-1,369772723	-1,471833228	0,102060505
210095_s_at	IGFBP3	-1,369772723	-1,471833228	0,102060505
226997_at	ADAMTS12	-0,66934516	-0,771340337	0,101995177
205697_at	SCGN	-0,66934516	-0,771340337	0,101995177
208267_at	TRPV5	-0,66934516	-0,771340337	0,101995177
1560062_at	SLC29A2	-0,66934516	-0,771340337	0,101995177

1552657_a_at	TXNDC2	-0,66934516	-0,771340337	0,101995177
227918_s_at	ZYG11B	-0,66934516	-0,771340337	0,101995177
202829_s_at	VAMP7	4,448374788	4,346466111	0,101908677
218351_at	COMMD8	5,645320963	5,543483425	0,101837538
224889_at	FOXO3	2,878891456	2,777528602	0,101362853
226633_at	RAB8B	4,460674444	4,359468791	0,101205653
225193_at	KIAA1967	1,745502609	1,644429915	0,101072694
202320_at	GTF3C1	1,745502609	1,644429915	0,101072694
220482_s_at	SERGEF	0,873789652	0,772793615	0,100996037
218726_at	HJURP	3,751261994	3,650351707	0,100910288
206006_s_at	KIAA1009	-0,043925712	-0,144765583	0,100839871
1569791_at	STK4	-0,043925712	-0,144765583	0,100839871
217393_x_at	UBE2NL	4,570504669	4,469757829	0,10074684
214753_at	N4BP2L2	1,640193838	1,539450683	0,100743155
205235_s_at	KIF20B	1,843966654	1,743236669	0,100729985
202232_s_at	EIF3M	5,664955538	5,564365119	0,10059042
1555760_a_at	RBM15	2,890618076	2,790111983	0,100506092
1553168_at	GRIK5	-0,384193355	-0,484558493	0,100365139
209133_s_at	COMMD4	-0,384193355	-0,484558493	0,100365139
213777_s_at	-	-0,384193355	-0,484558493	0,100365139
224491_at	APOL4	-0,384193355	-0,484558493	0,100365139
229473_at	MAMDC4	-0,384193355	-0,484558493	0,100365139
205244_s_at	SLC13A3	-0,384193355	-0,484558493	0,100365139
232814_x_at	APOPT1	2,51558601	2,415420763	0,100165247
217756_x_at	C15orf63 /// MIR1	5,875369475	5,775347831	0,100021645
208720_s_at	RBM39	5,183311485	5,083432658	0,099878827
1554593_s_at	SLC1A6	-2,250580859	-2,350444759	0,0998639
219949_at	LRRC2	-2,250580859	-2,350444759	0,0998639
1563385_at	LINC00307	-2,250580859	-2,350444759	0,0998639
224423_x_at	PMCHL2	-2,250580859	-2,350444759	0,0998639
1562102_at	-	-2,250580859	-2,350444759	0,0998639
223861_at	HORMAD1	-2,250580859	-2,350444759	0,0998639
211636_at	IGHA1 /// IGHA2 /,	-2,250580859	-2,350444759	0,0998639
230993_s_at	C6orf118	-2,250580859	-2,350444759	0,0998639
241225_at	-	-2,250580859	-2,350444759	0,0998639
233273_at	-	-2,250580859	-2,350444759	0,0998639
1556388_a_at	LOC100507389	-2,250580859	-2,350444759	0,0998639
237755_s_at	WDR16	-2,250580859	-2,350444759	0,0998639
231966_at	PPP1R9A	-2,250580859	-2,350444759	0,0998639
217238_s_at	ALDOB	-2,250580859	-2,350444759	0,0998639
239624_at	-	-2,250580859	-2,350444759	0,0998639
232523_at	MEGF10	-2,250580859	-2,350444759	0,0998639
215591_at	SATB2	-2,250580859	-2,350444759	0,0998639
1566825_at	-	-2,250580859	-2,350444759	0,0998639
237808_at	ST7-AS2	-2,250580859	-2,350444759	0,0998639
1552957_at	DNAH6	-2,250580859	-2,350444759	0,0998639
240788_at	-	-2,250580859	-2,350444759	0,0998639
217215_s_at	-	-2,250580859	-2,350444759	0,0998639
1569638_at	SOX5	-2,250580859	-2,350444759	0,0998639
1561849_at	PKD1L2	-2,250580859	-2,350444759	0,0998639

233947_s_at	TBX5-AS1	-2,250580859	-2,350444759	0,0998639
1556648_a_at	C10orf40	-2,250580859	-2,350444759	0,0998639
237277_at	-	-2,250580859	-2,350444759	0,0998639
224253_at	-	-2,250580859	-2,350444759	0,0998639
235942_at	FAM224A /// FAM	-2,250580859	-2,350444759	0,0998639
1558603_at	PLGLB2	-2,250580859	-2,350444759	0,0998639
1552477_a_at	IRF6	-2,250580859	-2,350444759	0,0998639
224341_x_at	TLR4	-2,250580859	-2,350444759	0,0998639
219497_s_at	BCL11A	-2,250580859	-2,350444759	0,0998639
219871_at	FLJ13197	-2,250580859	-2,350444759	0,0998639
210945_at	COL4A6	-2,250580859	-2,350444759	0,0998639
1563099_at	LOC100507053	-2,250580859	-2,350444759	0,0998639
1566947_at	-	-2,250580859	-2,350444759	0,0998639
222963_s_at	IL1RAPL1	-2,250580859	-2,350444759	0,0998639
239747_s_at	-	-2,250580859	-2,350444759	0,0998639
233298_at	CCDC169	-2,250580859	-2,350444759	0,0998639
1560185_at	-	-2,250580859	-2,350444759	0,0998639
229868_s_at	LOC100653010	-2,250580859	-2,350444759	0,0998639
1558136_s_at	TAF11	3,309267982	3,210051096	0,099216886
200912_s_at	EIF4A2 /// MIR124	7,626283832	7,52720758	0,099076253
222972_at	VSX1	-1,712732543	-1,811780055	0,099047512
241082_at	ASNS	-1,712732543	-1,811780055	0,099047512
224532_at	USP26	-1,712732543	-1,811780055	0,099047512
1554776_at	ZFP42	-1,712732543	-1,811780055	0,099047512
242322_at	-	-1,712732543	-1,811780055	0,099047512
230214_at	MRVI1	-1,712732543	-1,811780055	0,099047512
240613_at	JAK1	-1,712732543	-1,811780055	0,099047512
209351_at	KRT14	-1,712732543	-1,811780055	0,099047512
236337_at	SYCP2L	-1,712732543	-1,811780055	0,099047512
1555108_at	SLC10A7	-1,712732543	-1,811780055	0,099047512
1553573_s_at	EFNA2	-1,712732543	-1,811780055	0,099047512
230803_s_at	ARHGAP24	-1,712732543	-1,811780055	0,099047512
244079_at	LRRC72	-1,712732543	-1,811780055	0,099047512
233513_at	-	-1,712732543	-1,811780055	0,099047512
203808_at	-	-1,712732543	-1,811780055	0,099047512
1560176_s_at	PPP4R1L	-1,712732543	-1,811780055	0,099047512
1564749_at	LOC100289045	-1,712732543	-1,811780055	0,099047512
216433_s_at	PRDM2	-1,712732543	-1,811780055	0,099047512
240390_at	GALNT5	-1,712732543	-1,811780055	0,099047512
1554668_a_at	FAM151A	-1,712732543	-1,811780055	0,099047512
244781_x_at	-	-1,712732543	-1,811780055	0,099047512
204456_s_at	GAS1	-1,712732543	-1,811780055	0,099047512
1553442_a_at	CNTNAP4	-1,712732543	-1,811780055	0,099047512
1554262_s_at	KLHL29	-1,712732543	-1,811780055	0,099047512
232359_at	RDH11	-1,712732543	-1,811780055	0,099047512
204042_at	WASF3	-1,712732543	-1,811780055	0,099047512
203799_at	CD302 /// LY75-CD	-1,712732543	-1,811780055	0,099047512
228764_s_at	MDP1 /// NEDD8-I	2,339581693	2,24081821	0,098763483
210041_s_at	PGM3	1,287793882	1,189078962	0,09871492
220658_s_at	ARNTL2	2,872644433	2,774037376	0,098607056

211754_s_at	SLC25A17	2,914752575	2,81616114	0,098591436
201268_at	NME1-NME2 /// N	6,867734668	6,769224796	0,098509872
200059_s_at	RHOA	7,216261307	7,117818697	0,09844261
208896_at	DDX18	2,920736393	2,822379032	0,09835736
208873_s_at	REEP5	4,036242149	3,937994353	0,098247796
233486_at	-	0,648195588	0,550017041	0,098178548
223034_s_at	C1orf43	6,437445553	6,339345265	0,098100288
241674_s_at	-	-2,708365344	-2,8064286	0,098063256
211379_x_at	B3GALNT1	-2,708365344	-2,8064286	0,098063256
237302_at	-	-2,708365344	-2,8064286	0,098063256
211093_at	PDE6C	-2,708365344	-2,8064286	0,098063256
1564631_at	-	-2,708365344	-2,8064286	0,098063256
1557871_at	LOC253573	-2,708365344	-2,8064286	0,098063256
230401_at	-	0,20740896	0,109458907	0,097950054
217863_at	PIAS1	2,348178434	2,250228409	0,097950026
236956_at	-	-0,791294935	-0,889222211	0,097927276
1566535_at	-	-0,791294935	-0,889222211	0,097927276
237146_at	-	-0,791294935	-0,889222211	0,097927276
241771_at	RIMBP2	-0,791294935	-0,889222211	0,097927276
1565758_at	-	-0,791294935	-0,889222211	0,097927276
228451_at	LOC100128071	-0,791294935	-0,889222211	0,097927276
244018_at	-	-0,791294935	-0,889222211	0,097927276
244712_at	MEX3D	-0,791294935	-0,889222211	0,097927276
1566504_at	-	-0,791294935	-0,889222211	0,097927276
218332_at	BEX1	-0,791294935	-0,889222211	0,097927276
209918_at	GGTLC2	-0,791294935	-0,889222211	0,097927276
236653_at	-	-0,791294935	-0,889222211	0,097927276
1554635_a_at	NPAS3	-0,791294935	-0,889222211	0,097927276
203445_s_at	CTDSP2	3,87876941	3,781121106	0,097648304
230199_at	-	-1,137208348	-1,234835326	0,097626978
241597_at	-	-1,137208348	-1,234835326	0,097626978
241379_at	APLF	-1,137208348	-1,234835326	0,097626978
1553255_at	ERVV-1	-1,137208348	-1,234835326	0,097626978
227055_at	METTL7B	-1,137208348	-1,234835326	0,097626978
231572_at	PRSS37	-1,137208348	-1,234835326	0,097626978
232239_at	LOC643529	-1,137208348	-1,234835326	0,097626978
240670_at	-	-1,137208348	-1,234835326	0,097626978
239019_at	KLF12	-1,137208348	-1,234835326	0,097626978
215326_at	PAK4	-1,137208348	-1,234835326	0,097626978
238500_at	EMP2	-1,137208348	-1,234835326	0,097626978
210451_at	PKLR	-1,137208348	-1,234835326	0,097626978
233008_at	LOC100507284	-1,137208348	-1,234835326	0,097626978
240872_at	-	-1,137208348	-1,234835326	0,097626978
216362_at	-	-1,137208348	-1,234835326	0,097626978
234698_at	N6AMT1	-1,137208348	-1,234835326	0,097626978
232941_at	TMPRSS6	-1,137208348	-1,234835326	0,097626978
1566580_at	-	-1,137208348	-1,234835326	0,097626978
243889_at	-	-1,137208348	-1,234835326	0,097626978
1552410_at	CLEC4F	-1,137208348	-1,234835326	0,097626978
205186_at	DNALI1	-1,137208348	-1,234835326	0,097626978

208380_at	LBX1	-1,137208348	-1,234835326	0,097626978
1561850_at	LOC100133669	-1,137208348	-1,234835326	0,097626978
217469_at	IGHA1	-1,137208348	-1,234835326	0,097626978
223881_at	ICA1L	-1,137208348	-1,234835326	0,097626978
225400_at	TSEN15	3,122148189	3,024624257	0,097523933
210214_s_at	BMPR2	0,08619576	-0,011001236	0,097196996
228755_at	GIGYF1	0,08619576	-0,011001236	0,097196996
202775_s_at	SFSWAP	1,440265662	1,343200523	0,097065139
205530_at	ETFDH	1,440265662	1,343200523	0,097065139
222436_s_at	CHMP3 /// RNF10:	2,890618076	2,793634788	0,096983288
218220_at	C12orf10	3,076375717	2,979585083	0,096790634
220999_s_at	CYFIP2	1,421391637	1,324949741	0,096441896
212005_at	C1orf144	2,410823487	2,314488991	0,096334496
216774_at	-	-0,49284695	-0,589001171	0,096154221
242032_at	KIDINS220	-0,49284695	-0,589001171	0,096154221
213252_at	SH3PXD2A	-0,49284695	-0,589001171	0,096154221
214411_x_at	CTRB2	-0,49284695	-0,589001171	0,096154221
1566134_at	CARHSP1	-0,49284695	-0,589001171	0,096154221
226824_at	CPXM2	-0,49284695	-0,589001171	0,096154221
235765_at	TLE4	-0,49284695	-0,589001171	0,096154221
208653_s_at	CD164	3,466076444	3,36994189	0,096134554
224931_at	SLC41A3	2,392346173	2,296310714	0,096035459
232098_at	DST /// LOC10065:	-0,135065865	-0,231045907	0,095980042
242533_at	LOC100130051	-0,135065865	-0,231045907	0,095980042
242701_at	TBRG1	-0,135065865	-0,231045907	0,095980042
226629_at	SLC43A2	-0,135065865	-0,231045907	0,095980042
217793_at	RAB11B	-0,135065865	-0,231045907	0,095980042
1568751_at	RGS13	-2,569309811	-2,665156778	0,095846966
221037_s_at	SLC25A31	-2,569309811	-2,665156778	0,095846966
232235_at	DSEL	-2,569309811	-2,665156778	0,095846966
201744_s_at	LUM	-2,569309811	-2,665156778	0,095846966
243028_x_at	-	-2,569309811	-2,665156778	0,095846966
1569494_at	MIPOL1	-2,569309811	-2,665156778	0,095846966
217698_at	-	-2,569309811	-2,665156778	0,095846966
243421_at	-	-2,569309811	-2,665156778	0,095846966
1563486_at	-	-2,569309811	-2,665156778	0,095846966
217194_at	-	-2,569309811	-2,665156778	0,095846966
241782_at	NEBL	-2,569309811	-2,665156778	0,095846966
1561777_at	-	-2,569309811	-2,665156778	0,095846966
230319_at	-	-2,569309811	-2,665156778	0,095846966
216158_at	-	-2,569309811	-2,665156778	0,095846966
1563332_at	-	-2,569309811	-2,665156778	0,095846966
1570391_at	-	-2,569309811	-2,665156778	0,095846966
230578_at	-	-2,569309811	-2,665156778	0,095846966
237974_at	ABHD12B /// MIR4	-2,569309811	-2,665156778	0,095846966
1566115_at	-	-2,569309811	-2,665156778	0,095846966
1566437_at	-	-2,569309811	-2,665156778	0,095846966
237961_at	-	-2,569309811	-2,665156778	0,095846966
216016_at	NLRP3	-2,569309811	-2,665156778	0,095846966
232468_at	-	-2,569309811	-2,665156778	0,095846966

235801_at	TUSC3	-2,569309811	-2,665156778	0,095846966
229327_s_at	-	-2,569309811	-2,665156778	0,095846966
213465_s_at	PPP1R7	5,137769258	5,041971841	0,095797416
213490_s_at	MAP2K2	0,95222556	0,85655369	0,095671871
230426_at	DLD	1,476175815	1,380552339	0,095623476
203503_s_at	PEX14	1,402495885	1,306903969	0,095591915
213945_s_at	NUP210	1,402495885	1,306903969	0,095591915
242364_x_at	LOC100131096	1,139057614	1,043469112	0,095588502
219261_at	C7orf26	1,458255986	1,362900801	0,095355185
210605_s_at	MFGE8	0,556428218	0,461385738	0,09504248
222679_s_at	DCUN1D1	2,33082239	2,235857731	0,094964659
218598_at	RINT1	2,692288672	2,597466271	0,094822401
223111_x_at	ARID4B	2,966903803	2,872127595	0,094776209
226049_at	ERC1	0,791908897	0,697221375	0,094687523
213546_at	DKFZP586I1420	2,597647262	2,503049671	0,094597591
201455_s_at	LOC100653042 ///	3,096793374	3,002270677	0,094522697
217221_x_at	RBM10	3,132805465	3,038411131	0,094394335
213682_at	NUP50	4,747567561	4,653335581	0,09423198
219236_at	PAQR6	0,677745787	0,583607503	0,094138284
203143_s_at	KIAA0040	0,677745787	0,583607503	0,094138284
32088_at	BLZF1	-0,22731151	-0,321039072	0,093727561
213857_s_at	CD47	5,39823584	5,304533528	0,093702312
202215_s_at	NFYC	1,936380191	1,842852096	0,093528095
224631_at	ZFP91	2,159837409	2,066329863	0,093507546
201188_s_at	ITPR3	1,544940605	1,451507543	0,093433063
205526_s_at	KATNA1	4,118748355	4,025360264	0,093388091
222148_s_at	RHOT1	3,685074281	3,591688835	0,093385446
208807_s_at	CHD3	1,686984721	1,593605508	0,093379213
201995_at	EXT1	1,686984721	1,593605508	0,093379213
225913_at	PEAK1	1,948628481	1,855279162	0,093349319
233093_s_at	BIRC6	4,060218357	3,966959907	0,09325845
221809_at	RANBP10	0,820160788	0,727123109	0,093037679
202539_s_at	HMGCR	3,86465425	3,771650587	0,093003664
209624_s_at	MCCC2	2,771643417	2,678678228	0,092965189
205425_at	HIP1	0,458905032	0,366031214	0,092873818
244478_at	LRRC37A3	0,458905032	0,366031214	0,092873818
207079_s_at	MED6	2,836219929	2,743433212	0,092786717
207564_x_at	OGT	2,537731447	2,444951949	0,092779498
632_at	GSK3A	1,502199165	1,409486045	0,092713121
202051_s_at	ZMYM4	3,001575397	2,908944587	0,092630811
227144_at	KIAA0930	1,182925501	1,090640407	0,092285095
241373_at	-	1,182925501	1,090640407	0,092285095
204504_s_at	HIRIP3	3,127551022	3,035366122	0,0921849
213980_s_at	CTBP1	4,175145566	4,083008429	0,092137137
226327_at	ZNF507	2,545254728	2,45323331	0,092021417
229579_s_at	DISP2	-0,230432956	-0,322340048	0,091907092
225630_at	EEPD1	-0,230432956	-0,322340048	0,091907092
1563138_at	-	-0,230432956	-0,322340048	0,091907092
226313_at	C10orf35	-0,230432956	-0,322340048	0,091907092
203415_at	PDCD6	4,45671768	4,364860928	0,091856752

1557079_at	ITGBL1	-2,336678463	-2,4284334	0,091754937
223681_s_at	INADL	-2,336678463	-2,4284334	0,091754937
1561287_at	LINC00561	-2,336678463	-2,4284334	0,091754937
232452_at	LOC148824	-2,336678463	-2,4284334	0,091754937
1558578_a_at	C7orf73 /// LOC10	-2,336678463	-2,4284334	0,091754937
237499_at	-	-2,336678463	-2,4284334	0,091754937
226930_at	FNDC1	-2,336678463	-2,4284334	0,091754937
1563367_at	MAPT-AS1	-2,336678463	-2,4284334	0,091754937
1555940_a_at	-	-2,336678463	-2,4284334	0,091754937
236103_at	-	-2,336678463	-2,4284334	0,091754937
1569817_at	-	-2,336678463	-2,4284334	0,091754937
243328_at	-	-2,336678463	-2,4284334	0,091754937
225207_at	PDK4	-2,336678463	-2,4284334	0,091754937
231464_at	-	-2,336678463	-2,4284334	0,091754937
233260_at	-	-2,336678463	-2,4284334	0,091754937
220880_at	-	-2,336678463	-2,4284334	0,091754937
1561633_at	HMGA2	-2,336678463	-2,4284334	0,091754937
206153_at	CYP4F11	-2,336678463	-2,4284334	0,091754937
1552266_at	ADAM32	-2,336678463	-2,4284334	0,091754937
210914_at	-	-2,336678463	-2,4284334	0,091754937
228704_s_at	CLDN23	-2,336678463	-2,4284334	0,091754937
1565693_at	DTYMK	-2,336678463	-2,4284334	0,091754937
1567031_at	ZNF160	-2,336678463	-2,4284334	0,091754937
1553434_at	CYP4Z2P	-2,336678463	-2,4284334	0,091754937
220619_at	CHD7	-2,336678463	-2,4284334	0,091754937
236734_at	SLITRK1	-2,336678463	-2,4284334	0,091754937
215516_at	LAMB4	-2,336678463	-2,4284334	0,091754937
230680_at	SLITRK4	-2,336678463	-2,4284334	0,091754937
1557890_at	STXBP5-AS1	-2,336678463	-2,4284334	0,091754937
224050_s_at	-	-2,336678463	-2,4284334	0,091754937
240725_at	-	-2,336678463	-2,4284334	0,091754937
239030_at	-	-2,336678463	-2,4284334	0,091754937
1570484_at	-	-2,336678463	-2,4284334	0,091754937
211170_s_at	PDE10A	-2,336678463	-2,4284334	0,091754937
1564855_at	LOC727924	-2,336678463	-2,4284334	0,091754937
230130_at	SLIT2	-2,336678463	-2,4284334	0,091754937
241457_at	-	-2,336678463	-2,4284334	0,091754937
1552766_at	HS6ST2	-2,336678463	-2,4284334	0,091754937
225050_at	ZNF512	3,983349065	3,891666284	0,091682782
219862_s_at	NARF	4,590648292	4,499058841	0,091589451
213883_s_at	TM2D1	4,16769828	4,076293142	0,091405137
244013_at	-	-0,924000698	-1,015398016	0,091397318
209986_at	PAH	-0,924000698	-1,015398016	0,091397318
219947_at	CLEC4A	-0,924000698	-1,015398016	0,091397318
220283_at	HHIPL2	-0,924000698	-1,015398016	0,091397318
230438_at	TBX15	-0,924000698	-1,015398016	0,091397318
229578_at	JPH2	-0,924000698	-1,015398016	0,091397318
238497_at	TMEM136	-0,924000698	-1,015398016	0,091397318
227876_at	ARHGAP39	-0,924000698	-1,015398016	0,091397318
205610_at	MYOM1	-0,924000698	-1,015398016	0,091397318

1554474_a_at	MOXD1	-0,924000698	-1,015398016	0,091397318
231893_at	KIAA1755	-0,924000698	-1,015398016	0,091397318
207682_s_at	KIF25	-0,924000698	-1,015398016	0,091397318
220847_x_at	ZNF221	-0,924000698	-1,015398016	0,091397318
241794_at	ACTR6	-0,924000698	-1,015398016	0,091397318
1570057_x_at	-	-0,924000698	-1,015398016	0,091397318
229901_at	ZNF488	-0,924000698	-1,015398016	0,091397318
211665_s_at	SOS2	-0,924000698	-1,015398016	0,091397318
215990_s_at	BCL6	-0,924000698	-1,015398016	0,091397318
224864_at	SRA1	2,745538988	2,654387125	0,091151863
228114_x_at	C16orf13	2,476788561	2,385765892	0,091022668
1567458_s_at	RAC1	3,618399139	3,527465373	0,090933766
217752_s_at	CNDP2	4,140736098	4,049836996	0,090899102
243052_at	MOB3C	0,001114523	-0,089635713	0,090750236
204610_s_at	CCDC85B	3,6787904	3,588157836	0,090632564
211481_at	SLCO1A2	-1,624955693	-1,715576125	0,090620432
1561212_at	-	-1,624955693	-1,715576125	0,090620432
237493_at	IL22RA2	-1,624955693	-1,715576125	0,090620432
1558680_s_at	PDE1A	-1,624955693	-1,715576125	0,090620432
240856_at	O3FAR1	-1,624955693	-1,715576125	0,090620432
1557661_at	-	-1,624955693	-1,715576125	0,090620432
205935_at	FOXF1	-1,624955693	-1,715576125	0,090620432
206585_at	MKRN3	-1,624955693	-1,715576125	0,090620432
1554328_at	STXBP4	-1,624955693	-1,715576125	0,090620432
242420_at	-	-1,624955693	-1,715576125	0,090620432
1558320_at	-	-1,624955693	-1,715576125	0,090620432
240565_at	ZFP28	-1,624955693	-1,715576125	0,090620432
236652_at	LOC149703	-1,624955693	-1,715576125	0,090620432
231170_at	-	-1,624955693	-1,715576125	0,090620432
219605_at	ZNF3	-1,624955693	-1,715576125	0,090620432
207368_at	HTR1D	-1,624955693	-1,715576125	0,090620432
1563540_at	-	-1,624955693	-1,715576125	0,090620432
211238_at	ADAM7	-1,624955693	-1,715576125	0,090620432
213697_at	HIPK3	-1,624955693	-1,715576125	0,090620432
242494_at	-	-1,624955693	-1,715576125	0,090620432
1552925_at	PCDH10	-1,624955693	-1,715576125	0,090620432
1561468_at	-	-1,624955693	-1,715576125	0,090620432
235960_at	FXR1	-1,624955693	-1,715576125	0,090620432
243635_at	-	-1,624955693	-1,715576125	0,090620432
244382_at	-	-1,624955693	-1,715576125	0,090620432
229518_at	FAM46B	-1,624955693	-1,715576125	0,090620432
244460_at	TMEM225	-1,624955693	-1,715576125	0,090620432
221120_at	-	-1,624955693	-1,715576125	0,090620432
203772_at	BLVRA	-1,624955693	-1,715576125	0,090620432
1563244_at	-	-1,624955693	-1,715576125	0,090620432
228481_at	-	-1,624955693	-1,715576125	0,090620432
1563691_at	LOC285300	-1,624955693	-1,715576125	0,090620432
1555162_at	ARHGAP8 /// PRR5	-1,624955693	-1,715576125	0,090620432
1570212_a_at	GNN	-1,624955693	-1,715576125	0,090620432
209168_at	GPM6B	-1,624955693	-1,715576125	0,090620432

230695_s_at	RSPH9	-1,624955693	-1,715576125	0,090620432
220481_at	GPR75	-1,624955693	-1,715576125	0,090620432
241573_at	LOC100505735	-1,624955693	-1,715576125	0,090620432
243173_at	CABP7	-1,624955693	-1,715576125	0,090620432
237696_at	-	-1,624955693	-1,715576125	0,090620432
211905_s_at	ITGB4	-1,624955693	-1,715576125	0,090620432
206882_at	SLC1A6	-1,624955693	-1,715576125	0,090620432
1565306_a_at	SLC8A1	-1,624955693	-1,715576125	0,090620432
241046_at	LOC100505903	-1,624955693	-1,715576125	0,090620432
235184_at	AEBP2	-1,624955693	-1,715576125	0,090620432
1559777_at	LOC731424	-1,624955693	-1,715576125	0,090620432
200730_s_at	PTP4A1	2,752615529	2,662083808	0,090531721
233651_s_at	MPND	1,983901861	1,893444329	0,090457532
1569114_at	LOC100506847	0,58788177	0,497424714	0,090457056
203063_at	PPM1F	0,58788177	0,497424714	0,090457056
220905_at	-	0,355751005	0,265475485	0,09027552
222791_at	RSBN1	1,995527416	1,905294823	0,090232593
1569775_at	RNF157	0,127732831	0,03784737	0,08988546
239197_s_at	EZH1	0,127732831	0,03784737	0,08988546
1560156_at	-	0,707657549	0,61779983	0,089857719
231548_at	FOXO3	0,707657549	0,61779983	0,089857719
222351_at	PPP2R1B	0,707657549	0,61779983	0,089857719
207484_s_at	EHMT2	1,246401134	1,156599976	0,089801157
229806_at	QRICH1	1,246401134	1,156599976	0,089801157
218574_s_at	LMCD1	3,117098466	3,027559376	0,089539089
200653_s_at	CALM1 /// CALM2	5,721536845	5,632098876	0,08943797
212484_at	FAM89B	2,339581693	2,250228409	0,089353284
213361_at	TDRD7	2,914752575	2,825679048	0,089073528
210395_x_at	MYL4	1,267146969	1,178100917	0,089046052
201270_x_at	NUDCD3	3,618399139	3,52937772	0,089021419
200641_s_at	YWHAZ	5,798914403	5,710078304	0,088836099
225958_at	PHC1	2,138605583	2,050286491	0,088319093
217747_s_at	RPS9	7,73044054	7,64228132	0,088159221
211165_x_at	EPHB2	-0,609040214	-0,697182596	0,088142383
242035_at	-	-0,609040214	-0,697182596	0,088142383
205247_at	NOTCH4	-0,609040214	-0,697182596	0,088142383
225322_s_at	C17orf70	-0,609040214	-0,697182596	0,088142383
1553400_a_at	C17orf69	-0,609040214	-0,697182596	0,088142383
218394_at	ROGDI	1,024284941	0,936232181	0,08805276
37892_at	COL11A1	-2,159709296	-2,247696104	0,087986809
211990_at	HLA-DPA1	7,011498445	6,923720396	0,087778049
218078_s_at	ZDHHC3	2,817838865	2,730083062	0,087755803
1558094_s_at	C3orf19	1,071336699	0,98381571	0,087520989
209556_at	NCDN	0,873789652	0,786391549	0,087398103
203899_s_at	CRCP	1,871521385	1,784182261	0,087339124
203049_s_at	TTC37	4,041098071	3,953789653	0,087308418
204947_at	E2F1	1,88491011	1,797682937	0,087227173
230977_at	NPM2	1,326463531	1,239268196	0,087195335
224648_at	GPBP1	3,509819135	3,422991983	0,086827153
208072_s_at	DGKD	1,307201325	1,220470658	0,086730667

219221_at	ZBTB38	2,365121417	2,278572464	0,086548953
222130_s_at	FTSJ2	2,523100136	2,436714502	0,086385634
201857_at	ZFR	5,086681681	5,000406733	0,086274948
209181_s_at	RABGGTB /// SNOI	5,508704656	5,422575923	0,086128732
222105_s_at	NKIRAS2	1,476175815	1,390070295	0,08610552
244777_at	DCP2	2,605420261	2,519368159	0,086052102
203985_at	ZNF212	1,686984721	1,601076277	0,085908444
1557777_at	PDE6B	-0,332405896	-0,418248858	0,085842962
231207_at	-	-0,332405896	-0,418248858	0,085842962
1559989_at	TPM4	-0,332405896	-0,418248858	0,085842962
206238_s_at	YAF2	2,096248267	2,010406245	0,085842023
201999_s_at	DYNLT1	5,938043502	5,852420828	0,085622674
49878_at	PEX16	3,515818668	3,430209153	0,085609514
201033_x_at	RPLP0	8,402524479	8,316934268	0,085590212
235057_at	ITCH	1,493647653	1,408220608	0,085427045
217912_at	DUS1L	2,966903803	2,881480309	0,085423495
200631_s_at	SET	6,443049058	6,357691345	0,085357713
200958_s_at	SDCBP	5,198678095	5,113450939	0,085227156
217952_x_at	PHF3	3,204847647	3,119733444	0,085114203
1554317_s_at	TRMT61A	0,618277321	0,533258442	0,085018879
210871_x_at	SSX2IP	0,900065578	0,815200271	0,084865307
212118_at	TRIM27	3,142467702	3,05768517	0,084782532
236644_at	RNF180	-2,416625063	-2,501359113	0,08473405
1552732_at	ABRA	-2,416625063	-2,501359113	0,08473405
242524_at	CBLN4	-2,416625063	-2,501359113	0,08473405
1569277_at	ZNF91	-2,416625063	-2,501359113	0,08473405
219651_at	DPPA4	-2,416625063	-2,501359113	0,08473405
1570101_at	-	-2,416625063	-2,501359113	0,08473405
1569916_at	-	-2,416625063	-2,501359113	0,08473405
1566605_at	TEX9	-2,416625063	-2,501359113	0,08473405
244441_at	-	-2,416625063	-2,501359113	0,08473405
206676_at	CEACAM8	-2,416625063	-2,501359113	0,08473405
241510_at	PLSCR2	-2,416625063	-2,501359113	0,08473405
243549_at	-	-2,416625063	-2,501359113	0,08473405
214702_at	FN1	-2,416625063	-2,501359113	0,08473405
220338_at	RALGPS2	-2,416625063	-2,501359113	0,08473405
234493_at	LOC116437	-2,416625063	-2,501359113	0,08473405
1552674_at	DIRC1	-2,416625063	-2,501359113	0,08473405
240186_at	-	-2,416625063	-2,501359113	0,08473405
241444_at	-	-2,416625063	-2,501359113	0,08473405
240034_at	-	-2,416625063	-2,501359113	0,08473405
1557863_at	-	-2,416625063	-2,501359113	0,08473405
219230_at	TMEM100	-2,416625063	-2,501359113	0,08473405
243700_x_at	FAM47A	-2,416625063	-2,501359113	0,08473405
1556695_a_at	FLJ42709	-2,416625063	-2,501359113	0,08473405
1564292_at	-	-2,416625063	-2,501359113	0,08473405
1567244_at	OR5J2	-2,416625063	-2,501359113	0,08473405
244444_at	LOC100653085 ///	-2,416625063	-2,501359113	0,08473405
1564601_at	LINC00221	-2,416625063	-2,501359113	0,08473405
206727_at	C9	-2,416625063	-2,501359113	0,08473405

242186_x_at	LPHN3	-2,416625063	-2,501359113	0,08473405
217083_at	-	-2,416625063	-2,501359113	0,08473405
1558496_at	LOC100505609	-2,416625063	-2,501359113	0,08473405
1569819_at	PWRN1	-2,416625063	-2,501359113	0,08473405
217265_at	-	-2,416625063	-2,501359113	0,08473405
1563704_at	-	-2,416625063	-2,501359113	0,08473405
1560838_at	FAM217A	-2,416625063	-2,501359113	0,08473405
244340_x_at	-	-2,416625063	-2,501359113	0,08473405
216575_at	-	-2,416625063	-2,501359113	0,08473405
208989_s_at	KDM2A	1,65554524	1,571112806	0,084432434
207431_s_at	DEGS1	4,801719103	4,71743974	0,084279363
221850_x_at	AGAP11 /// AGAP4	2,199941239	2,116147513	0,083793726
205261_at	PGC	-0,088974936	-0,172194225	0,083219289
203059_s_at	PAPSS2	-0,088974936	-0,172194225	0,083219289
1557656_at	LOC400238	-0,088974936	-0,172194225	0,083219289
230844_at	LOC100507800 ///	-1,289805289	-1,372734086	0,082928797
1555410_at	-	-1,289805289	-1,372734086	0,082928797
225894_at	SYNPO2	-1,289805289	-1,372734086	0,082928797
216279_at	ZNF460	-1,289805289	-1,372734086	0,082928797
210619_s_at	HYAL1	-1,289805289	-1,372734086	0,082928797
207288_at	ASMTL-AS1	-1,289805289	-1,372734086	0,082928797
243103_at	-	-1,289805289	-1,372734086	0,082928797
232747_at	-	-1,289805289	-1,372734086	0,082928797
1558324_a_at	TMEM72	-1,289805289	-1,372734086	0,082928797
1570529_at	KIAA0020	-1,289805289	-1,372734086	0,082928797
1559421_at	SNED1	-1,289805289	-1,372734086	0,082928797
237093_at	-	-1,289805289	-1,372734086	0,082928797
1569724_at	-	-1,289805289	-1,372734086	0,082928797
241134_at	-	-1,289805289	-1,372734086	0,082928797
1570133_at	-	-1,289805289	-1,372734086	0,082928797
230949_at	SLC23A3	-1,289805289	-1,372734086	0,082928797
205406_s_at	SPA17	2,138605583	2,055739171	0,082866413
207305_s_at	TRAPPC8	3,867546929	3,784889559	0,08265737
205839_s_at	BZRAP1	0,95222556	0,869824768	0,082400792
1554686_at	STAU2	-1,06282519	-1,145057014	0,082231824
239423_at	-	-1,06282519	-1,145057014	0,082231824
202036_s_at	SFRP1	-1,06282519	-1,145057014	0,082231824
215356_at	TDRD12	-1,06282519	-1,145057014	0,082231824
242861_at	NEDD9	-1,06282519	-1,145057014	0,082231824
240140_s_at	LRIG1	-1,06282519	-1,145057014	0,082231824
205602_x_at	PSG7	-1,06282519	-1,145057014	0,082231824
204740_at	CNKSR1	-1,06282519	-1,145057014	0,082231824
207977_s_at	DPT	-1,06282519	-1,145057014	0,082231824
237012_at	-	-1,06282519	-1,145057014	0,082231824
241983_at	PTPN2	-1,06282519	-1,145057014	0,082231824
210835_s_at	CTBP2	-1,06282519	-1,145057014	0,082231824
229364_at	LOC100631377	0,167727503	0,085534992	0,082192511
208090_s_at	AIRE	0,167727503	0,085534992	0,082192511
231888_at	-	0,167727503	0,085534992	0,082192511
200001_at	CAPNS1	4,531363785	4,449190565	0,08217322

201612_at	ALDH9A1	4,642573329	4,56049157	0,082081758
206505_at	UGT2B4	-2,774503732	-2,856570137	0,082066404
1555111_at	C1orf114	-2,774503732	-2,856570137	0,082066404
1567702_at	-	-2,774503732	-2,856570137	0,082066404
1562553_at	-	-2,774503732	-2,856570137	0,082066404
1555708_a_at	OFCC1	-2,774503732	-2,856570137	0,082066404
1569604_at	LOC100133612	0,045517965	-0,036543687	0,082061652
202649_x_at	RPS19	8,140451541	8,058441918	0,082009623
224318_s_at	KANSL3	3,8107521	3,728800162	0,081951938
237717_x_at	-	-2,641232718	-2,723114724	0,081882007
1566749_at	-	-2,641232718	-2,723114724	0,081882007
207744_at	-	-2,641232718	-2,723114724	0,081882007
244351_at	RBM46	-2,641232718	-2,723114724	0,081882007
241044_x_at	-	-2,641232718	-2,723114724	0,081882007
224278_at	CYP4F30P	-2,641232718	-2,723114724	0,081882007
1559839_at	TBX18	-2,641232718	-2,723114724	0,081882007
1556207_a_at	LOC100652856	-2,641232718	-2,723114724	0,081882007
235065_at	FAM59A	-2,641232718	-2,723114724	0,081882007
228115_at	FAM59A	-2,641232718	-2,723114724	0,081882007
214224_s_at	PIN4	3,745344686	3,663499786	0,081844899
240099_at	-	-0,730013898	-0,811769547	0,081755648
236721_at	ALKBH1	-0,730013898	-0,811769547	0,081755648
1570439_at	-	-0,730013898	-0,811769547	0,081755648
1557089_at	KIF5C	-0,730013898	-0,811769547	0,081755648
1553972_a_at	CBS	-0,730013898	-0,811769547	0,081755648
207382_at	TP63	0,282358733	0,20062106	0,081737673
201472_at	VBP1	5,656285576	5,574995021	0,081290555
207091_at	P2RX7	-0,437449947	-0,518232988	0,080783041
1566885_at	-	-0,437449947	-0,518232988	0,080783041
234958_at	-	-0,437449947	-0,518232988	0,080783041
241924_at	MECP2	-0,437449947	-0,518232988	0,080783041
238086_at	LOC100129617	-0,437449947	-0,518232988	0,080783041
206536_s_at	XIAP	-0,437449947	-0,518232988	0,080783041
216784_at	-	-0,437449947	-0,518232988	0,080783041
220992_s_at	TRMT1L	1,871521385	1,790914523	0,080606862
227871_at	CHM	2,896717728	2,81616114	0,080556588
226135_at	UHRF1BP1	3,161901883	3,081435405	0,080466478
225508_at	KIAA1468	1,910557155	1,830129603	0,080427552
227640_s_at	RP9 /// RP9P	1,92317941	1,842852096	0,080327314
203633_at	CPT1A	3,112433079	3,032393954	0,080039124
202901_x_at	CTSS	0,791908897	0,711934445	0,079974452
214548_x_at	GNAS	6,222737734	6,143154256	0,079583478
200643_at	HDLBP	2,582572508	2,503049671	0,079522837
228851_s_at	ENSA	2,914752575	2,835336991	0,079415584
242135_at	LOC100509302	-1,537851782	-1,616924751	0,079072969
1559403_at	LOC100288198	-1,537851782	-1,616924751	0,079072969
216967_at	GAP43	-1,537851782	-1,616924751	0,079072969
240766_at	-	-1,537851782	-1,616924751	0,079072969
238498_at	-	-1,537851782	-1,616924751	0,079072969
1557833_at	-	-1,537851782	-1,616924751	0,079072969

230315_at	-	-1,537851782	-1,616924751	0,079072969
234223_at	-	-1,537851782	-1,616924751	0,079072969
208345_s_at	POU3F1	-1,537851782	-1,616924751	0,079072969
1561702_at	-	-1,537851782	-1,616924751	0,079072969
205303_at	KCNJ8	-1,537851782	-1,616924751	0,079072969
1562621_at	-	-1,537851782	-1,616924751	0,079072969
241210_at	-	-1,537851782	-1,616924751	0,079072969
206421_s_at	SERPINB7	-1,537851782	-1,616924751	0,079072969
210546_x_at	CTAG1A /// CTAG1	-1,537851782	-1,616924751	0,079072969
1561075_at	-	-1,537851782	-1,616924751	0,079072969
1568765_at	SERPINE1	-1,537851782	-1,616924751	0,079072969
232925_at	-	-1,537851782	-1,616924751	0,079072969
1564138_at	FBXW12	-1,537851782	-1,616924751	0,079072969
1553241_at	NKAIN3	-1,537851782	-1,616924751	0,079072969
207797_s_at	LRP2BP	-1,537851782	-1,616924751	0,079072969
211882_x_at	FUT6	-1,537851782	-1,616924751	0,079072969
206811_at	ADCY8	-1,537851782	-1,616924751	0,079072969
1564211_at	C14orf64	-1,537851782	-1,616924751	0,079072969
214814_at	YTHDC1	-1,537851782	-1,616924751	0,079072969
1560058_at	LOC399900	-1,537851782	-1,616924751	0,079072969
1553234_at	ADAMTS18	-1,537851782	-1,616924751	0,079072969
217178_at	RARG	-1,537851782	-1,616924751	0,079072969
214371_at	TSSK2	-1,537851782	-1,616924751	0,079072969
205991_s_at	PRRX1	-1,537851782	-1,616924751	0,079072969
237029_at	HGD	-1,537851782	-1,616924751	0,079072969
224419_x_at	PMCHL1	-1,537851782	-1,616924751	0,079072969
223963_s_at	IGF2BP2	-1,537851782	-1,616924751	0,079072969
237959_at	-	-1,537851782	-1,616924751	0,079072969
1562724_at	LOC286114	-1,537851782	-1,616924751	0,079072969
1564237_at	-	-1,537851782	-1,616924751	0,079072969
222546_s_at	EPS8L2	-1,537851782	-1,616924751	0,079072969
234198_at	KCNIP3	-1,537851782	-1,616924751	0,079072969
211147_s_at	P2RX6	-1,537851782	-1,616924751	0,079072969
229310_at	KLHL29	-1,537851782	-1,616924751	0,079072969
233022_at	TRPM3	-1,537851782	-1,616924751	0,079072969
225538_at	ZCCHC9	3,732463367	3,653742074	0,078721293
203218_at	MAPK9	3,936891546	3,858181176	0,07871037
226374_at	CXADR	1,578719607	1,500059916	0,07865969
227696_at	EXOSC6	3,18076822	3,1021766	0,07859162
217919_s_at	MRPL42	4,473867663	4,39537907	0,078488593
206288_at	PGGT1B	1,246401134	1,167919222	0,078481912
228248_at	RICTOR	1,246401134	1,167919222	0,078481912
230085_at	PDK3	1,610353504	1,531877749	0,078475755
215773_x_at	PARP2	3,840618883	3,762464514	0,078154369
218843_at	FNDC4	0,425036312	0,346964736	0,078071577
225934_at	LOC148413	1,267146969	1,189078962	0,078068007
235670_at	STX11	2,042564225	1,964670566	0,077893659
219357_at	GTPBP1	2,811067138	2,733194118	0,07787302
208137_x_at	ZNF611	1,686984721	1,60918327	0,077801451
218459_at	TOR3A	2,949541692	2,872127595	0,077414097

209333_at	ULK1	0,677745787	0,600385088	0,077360699
210050_at	TPI1	0,556428218	0,479089184	0,077339034
1555762_s_at	RBM15	3,282538749	3,205255599	0,07728315
235407_at	-	2,138605583	2,06133574	0,077269844
1553983_at	DTYMK	1,000459215	0,923203038	0,077256176
1555105_a_at	MIER1	1,476175815	1,399283506	0,076892309
208093_s_at	NDEL1	3,286844008	3,210051096	0,076792912
218047_at	OSBPL9	5,183311485	5,106686237	0,076625248
239147_at	ARSK	1,493647653	1,417157926	0,076489727
217893_s_at	AKIRIN1	3,688415399	3,612221266	0,076194133
203669_s_at	DGAT1	1,047341799	0,971607464	0,075734334
211935_at	ARL6IP1	6,22352144	6,147838411	0,075683029
225430_at	GATC	3,161901883	3,086398747	0,075503137
222551_s_at	C8orf33	2,765070895	2,689594859	0,075476036
203057_s_at	PRDM2	4,540832283	4,465395989	0,075436294
235118_at	CADM2	-2,495152701	-2,570522741	0,075370041
244435_at	FAM196A	-2,495152701	-2,570522741	0,075370041
1558668_s_at	SPATA22	-2,495152701	-2,570522741	0,075370041
1564294_at	-	-2,495152701	-2,570522741	0,075370041
1553885_x_at	ZNF99	-2,495152701	-2,570522741	0,075370041
241764_at	-	-2,495152701	-2,570522741	0,075370041
1556395_at	-	-2,495152701	-2,570522741	0,075370041
1558867_at	DSE	-2,495152701	-2,570522741	0,075370041
234209_at	-	-2,495152701	-2,570522741	0,075370041
233318_at	-	-2,495152701	-2,570522741	0,075370041
240989_at	-	-2,495152701	-2,570522741	0,075370041
1564468_at	-	-2,495152701	-2,570522741	0,075370041
1555710_at	-	-2,495152701	-2,570522741	0,075370041
224116_at	-	-2,495152701	-2,570522741	0,075370041
1555798_at	LOC400692	-2,495152701	-2,570522741	0,075370041
215729_s_at	VGLL1	-2,495152701	-2,570522741	0,075370041
216752_at	PIK3R4	-2,495152701	-2,570522741	0,075370041
233139_at	-	-2,495152701	-2,570522741	0,075370041
214021_x_at	ITGB5	-2,495152701	-2,570522741	0,075370041
1562389_at	-	-2,495152701	-2,570522741	0,075370041
202015_x_at	-	-2,495152701	-2,570522741	0,075370041
1561272_at	-	-2,495152701	-2,570522741	0,075370041
1560791_at	-	-2,495152701	-2,570522741	0,075370041
239371_at	-	-2,495152701	-2,570522741	0,075370041
239806_at	-	-2,495152701	-2,570522741	0,075370041
1559434_at	-	-2,495152701	-2,570522741	0,075370041
1570355_a_at	DPY19L1	-2,495152701	-2,570522741	0,075370041
206211_at	SELE	-2,495152701	-2,570522741	0,075370041
240819_at	-	-2,495152701	-2,570522741	0,075370041
205533_s_at	CDH6	-2,495152701	-2,570522741	0,075370041
1554629_at	EPHA7	-2,495152701	-2,570522741	0,075370041
1569904_x_at	-	-2,495152701	-2,570522741	0,075370041
225645_at	EHF	-2,495152701	-2,570522741	0,075370041
229114_at	GAB1	-2,495152701	-2,570522741	0,075370041
244361_at	-	-0,856259172	-0,931539147	0,075279975

241339_at	-	-0,856259172	-0,931539147	0,075279975
230138_at	NCS1	-0,856259172	-0,931539147	0,075279975
236131_at	-	-0,856259172	-0,931539147	0,075279975
221854_at	PKP1	-0,856259172	-0,931539147	0,075279975
240020_at	-	-0,856259172	-0,931539147	0,075279975
1558976_x_at	FAM100A	-0,856259172	-0,931539147	0,075279975
209443_at	SERPINA5	-0,856259172	-0,931539147	0,075279975
226971_at	CCDC136	-0,856259172	-0,931539147	0,075279975
210280_at	MPZ	-0,856259172	-0,931539147	0,075279975
220457_at	SAMD4B	-0,856259172	-0,931539147	0,075279975
209078_s_at	TXN2	3,57383031	3,498658433	0,075171877
222962_s_at	MCM10	2,692288672	2,617393502	0,07489517
37872_at	JRK	0,649691725	0,574810511	0,074881213
202156_s_at	CELF2	3,960533133	3,885830539	0,074702595
231765_at	ZFYVE20	0,319287178	0,244646697	0,074640481
1552315_at	GIMAP1	-0,280273599	-0,354781582	0,074507982
239419_at	PTPRA	-0,280273599	-0,354781582	0,074507982
243261_at	LOC154822	-0,280273599	-0,354781582	0,074507982
1556662_at	LOC100506142	-0,280273599	-0,354781582	0,074507982
230125_at	GUSB	-0,280273599	-0,354781582	0,074507982
203657_s_at	CTSF	-0,280273599	-0,354781582	0,074507982
219279_at	DOCK10	3,233229368	3,158887039	0,074342329
226030_at	ACADSB	1,910557155	1,836262316	0,074294839
233588_x_at	PFDN6	3,581038515	3,506795122	0,074243392
201406_at	RPL36A /// RPL36/	7,766662452	7,692451145	0,074211308
227754_at	-	1,094118704	1,020514937	0,073603767
219219_at	TMEM160	2,914752575	2,841176366	0,07357621
214879_x_at	USF2	2,285848435	2,212304392	0,073544043
212997_s_at	TLK2	2,872644433	2,799505781	0,073138652
55616_at	PGAP3	0,70716665	0,634144475	0,073022175
227299_at	CCNI	0,08619576	0,013190398	0,073005362
236624_at	LOC643837	-0,043925712	-0,116767475	0,072841763
222923_s_at	KCNE3	-0,043925712	-0,116767475	0,072841763
203793_x_at	PCGF2	-0,043925712	-0,116767475	0,072841763
210879_s_at	RAB11FIP5	-0,043925712	-0,116767475	0,072841763
213087_s_at	EEF1D	0,873789652	0,80114751	0,072642142
208938_at	PRCC	2,199941239	2,127337555	0,072603684
213750_at	RSL1D1	3,410696467	3,338139532	0,072556935
45288_at	ABHD6	0,449120285	0,37665821	0,072462075
203870_at	USP46	0,58788177	0,515457602	0,072424168
229231_at	LRRC37B	1,995527416	1,923216608	0,072310808
210156_s_at	PCMT1	5,210892881	5,138766644	0,072126237
221227_x_at	COQ3	2,149438457	2,077419168	0,072019289
222965_at	PRO2214	-0,551284523	-0,623254098	0,071969576
1561762_s_at	-	-0,551284523	-0,623254098	0,071969576
227610_at	TSPAN11	-0,551284523	-0,623254098	0,071969576
1567253_at	OR10D3	-0,551284523	-0,623254098	0,071969576
1560399_a_at	OTUD7A	-0,551284523	-0,623254098	0,071969576
235823_at	ACSF3	-0,551284523	-0,623254098	0,071969576
242933_at	-	-0,551284523	-0,623254098	0,071969576

225566_at	NRP2	-0,551284523	-0,623254098	0,071969576
216014_s_at	ZXDA /// ZXDB	-0,551284523	-0,623254098	0,071969576
33646_g_at	GM2A	-0,018386475	-0,090343686	0,071957211
1557599_a_at	-	-2,708365344	-2,780225148	0,071859804
244434_at	GPR82	-2,708365344	-2,780225148	0,071859804
207107_at	RPE65	-2,708365344	-2,780225148	0,071859804
1553441_at	CNTNAP4	-2,708365344	-2,780225148	0,071859804
241567_at	-	-2,708365344	-2,780225148	0,071859804
242090_x_at	-	-2,708365344	-2,780225148	0,071859804
1567861_at	DNAH14	-2,708365344	-2,780225148	0,071859804
1562290_at	-	-2,708365344	-2,780225148	0,071859804
220656_at	-	-2,708365344	-2,780225148	0,071859804
237477_at	PDCL2	-2,708365344	-2,780225148	0,071859804
203401_at	PRPS2	4,418104951	4,346466111	0,07163884
226399_at	DNAJB14	2,804738618	2,733194118	0,0715445
224523_s_at	C3orf26	5,081560195	5,010208945	0,071351249
225041_at	MPHOSPH8	3,300874993	3,229534127	0,071340866
222429_at	DBNL	2,2388565	2,167793428	0,071063072
226059_at	TOMM40L	1,5944803	1,523429058	0,071051242
209473_at	ENTPD1	4,221194173	4,150580971	0,070613202
56829_at	TRAPPC9	1,984206498	1,913729716	0,070476782
231288_at	CCDC88C	1,182925501	1,112548615	0,070376886
218748_s_at	EXOC5	1,160996507	1,090640407	0,070356101
64432_at	MAPKAPK5-AS1	2,184216265	2,114132326	0,070083939
201613_s_at	AP1G2	2,560606634	2,490640503	0,069966131
1553158_at	CEP19	2,339581693	2,269637658	0,069944035
1569108_a_at	ZNF589	-1,212652659	-1,282520722	0,069868063
210654_at	TNFRSF10D	-1,212652659	-1,282520722	0,069868063
231261_at	CATSPERG	-1,212652659	-1,282520722	0,069868063
240416_at	-	-1,212652659	-1,282520722	0,069868063
244136_at	LOC100507261	-1,212652659	-1,282520722	0,069868063
222961_at	-	-1,212652659	-1,282520722	0,069868063
238024_at	-	-1,212652659	-1,282520722	0,069868063
1561434_at	-	-1,212652659	-1,282520722	0,069868063
237903_at	-	-1,212652659	-1,282520722	0,069868063
1553181_at	DDX31	-1,212652659	-1,282520722	0,069868063
226096_at	FNDC5	-1,212652659	-1,282520722	0,069868063
238567_at	SGPP2	-1,212652659	-1,282520722	0,069868063
235805_at	-	-1,212652659	-1,282520722	0,069868063
1556048_at	LOC100507564	-1,212652659	-1,282520722	0,069868063
237635_at	LOC100128164	-1,212652659	-1,282520722	0,069868063
210876_at	ANXA2P1	-1,212652659	-1,282520722	0,069868063
204733_at	KLK6	-1,212652659	-1,282520722	0,069868063
228886_at	LRRC27	-1,212652659	-1,282520722	0,069868063
203666_at	CXCL12	-1,212652659	-1,282520722	0,069868063
1560981_a_at	PPARA	-1,212652659	-1,282520722	0,069868063
210956_at	PPYR1	-1,212652659	-1,282520722	0,069868063
214824_at	-	-1,212652659	-1,282520722	0,069868063
230115_at	DKFZp779M0652	-1,212652659	-1,282520722	0,069868063
1560883_s_at	-	-1,212652659	-1,282520722	0,069868063

223148_at	PIGS	2,523100136	2,45323331	0,069866826
230974_at	DDX19B	0,926325262	0,85655369	0,069771572
224296_x_at	-	-2,834722577	-2,904470894	0,069748317
238441_at	PRKAA2	-2,834722577	-2,904470894	0,069748317
1561177_at	-	-2,834722577	-2,904470894	0,069748317
243159_x_at	-	-2,834722577	-2,904470894	0,069748317
1562080_at	LINC00424	-2,834722577	-2,904470894	0,069748317
241961_at	TECRL	-2,834722577	-2,904470894	0,069748317
224930_x_at	RPL7A /// SNORD2	8,39921957	8,3295446	0,069674969
200726_at	PPP1CC	6,828287193	6,758784731	0,069502461
233564_s_at	CDADC1	0,355751005	0,286279868	0,069471137
200663_at	CD63	6,002256791	5,93332577	0,068931021
212637_s_at	WWP1	1,421391637	1,352622437	0,0687692
212287_at	SUZ12	5,241506811	5,172780458	0,068726352
226208_at	ZSWIM6	1,20396005	1,135235995	0,068724055
203787_at	SSBP2	3,639446351	3,570749938	0,068696413
200820_at	PSMD8	5,048031556	4,9794364	0,068595156
228019_s_at	LOC100509751 ///	4,263008992	4,194620233	0,068388759
227915_at	ASB2	3,200057698	3,131713063	0,068344635
1558688_at	LOC441461	2,855122714	2,786807892	0,068314822
209523_at	TAF2	4,030423661	3,962122762	0,0683009
223133_at	TMEM14B	5,358104722	5,289808844	0,068295878
215735_s_at	TSC2	0,618277321	0,550017041	0,068260281
241825_at	TYW5	0,618277321	0,550017041	0,068260281
1557758_at	-	-2,074223907	-2,142286822	0,068062915
1552841_s_at	BCL2L14	-2,074223907	-2,142286822	0,068062915
215306_at	-	-2,074223907	-2,142286822	0,068062915
209869_at	ADRA2A	-2,074223907	-2,142286822	0,068062915
241750_x_at	-	-2,074223907	-2,142286822	0,068062915
244350_at	MYO10	-2,074223907	-2,142286822	0,068062915
1569176_at	TMPRSS12	-2,074223907	-2,142286822	0,068062915
1566176_at	-	-2,074223907	-2,142286822	0,068062915
1562969_at	DNMBP	-2,074223907	-2,142286822	0,068062915
217401_at	-	-2,074223907	-2,142286822	0,068062915
216864_at	-	-2,074223907	-2,142286822	0,068062915
224136_at	-	-2,074223907	-2,142286822	0,068062915
1561863_a_at	-	-2,074223907	-2,142286822	0,068062915
230825_at	-	-2,074223907	-2,142286822	0,068062915
205534_at	PCDH7	-2,074223907	-2,142286822	0,068062915
231556_at	TECR	-2,074223907	-2,142286822	0,068062915
207994_s_at	OPRM1	-2,074223907	-2,142286822	0,068062915
221731_x_at	VCAN	-2,074223907	-2,142286822	0,068062915
1554196_at	IZUMO1	-2,074223907	-2,142286822	0,068062915
1569842_at	LOC728114	-2,074223907	-2,142286822	0,068062915
241551_at	-	-2,074223907	-2,142286822	0,068062915
211303_x_at	FOLH1B	-2,074223907	-2,142286822	0,068062915
236768_at	LOC100128139	-2,074223907	-2,142286822	0,068062915
239588_s_at	-	-2,074223907	-2,142286822	0,068062915
235111_at	LSAMP	-2,074223907	-2,142286822	0,068062915
241338_at	-	-2,074223907	-2,142286822	0,068062915

216865_at	COL14A1	-2,074223907	-2,142286822	0,068062915
1561915_at	-	-2,074223907	-2,142286822	0,068062915
241167_at	ITIH4 /// MUSTN1	-2,074223907	-2,142286822	0,068062915
220381_at	ARHGAP28	-2,074223907	-2,142286822	0,068062915
210772_at	FPR2	-2,074223907	-2,142286822	0,068062915
1558623_at	LOC729121	-2,074223907	-2,142286822	0,068062915
1570361_a_at	-	-2,074223907	-2,142286822	0,068062915
1552658_a_at	NAV3	-2,074223907	-2,142286822	0,068062915
211555_s_at	GUCY1B3	-2,074223907	-2,142286822	0,068062915
239471_at	LRRC28	-2,074223907	-2,142286822	0,068062915
231267_at	LOC100506206	-2,074223907	-2,142286822	0,068062915
1559526_at	-	-2,074223907	-2,142286822	0,068062915
209367_at	STXBP2	1,307201325	1,239268196	0,067933129
219733_s_at	SLC27A5	2,118167848	2,050286491	0,067881357
221518_s_at	USP47	4,111472006	4,043683659	0,067788347
209271_at	BPTF	2,817838865	2,750185881	0,067652984
202160_at	CREBBP	2,725848647	2,65824137	0,067607277
213468_at	ERCC2	1,383446753	1,315897482	0,06754927
240714_at	-	-1,982324124	-2,049747196	0,067423072
1569290_s_at	GRIA3	-1,982324124	-2,049747196	0,067423072
215542_at	-	-1,982324124	-2,049747196	0,067423072
237375_at	LOC100506816	-1,982324124	-2,049747196	0,067423072
234502_at	-	-1,982324124	-2,049747196	0,067423072
221152_at	COL8A1	-1,982324124	-2,049747196	0,067423072
234184_at	-	-1,982324124	-2,049747196	0,067423072
206909_at	-	-1,982324124	-2,049747196	0,067423072
237605_at	-	-1,982324124	-2,049747196	0,067423072
236088_at	NTNG1	-1,982324124	-2,049747196	0,067423072
1563139_at	-	-1,982324124	-2,049747196	0,067423072
237966_at	-	-1,982324124	-2,049747196	0,067423072
237637_at	-	-1,982324124	-2,049747196	0,067423072
234805_at	-	-1,982324124	-2,049747196	0,067423072
236156_at	LIPA /// LOC10050	-1,982324124	-2,049747196	0,067423072
1561187_at	-	-1,982324124	-2,049747196	0,067423072
1557295_a_at	-	-1,982324124	-2,049747196	0,067423072
242173_at	-	-1,982324124	-2,049747196	0,067423072
1562572_at	-	-1,982324124	-2,049747196	0,067423072
1564472_at	-	-1,982324124	-2,049747196	0,067423072
209937_at	TM4SF4	-1,982324124	-2,049747196	0,067423072
244150_at	-	-1,982324124	-2,049747196	0,067423072
1562189_at	-	-1,982324124	-2,049747196	0,067423072
1557593_at	SPAG17	-1,982324124	-2,049747196	0,067423072
241397_at	-	-1,982324124	-2,049747196	0,067423072
1568932_at	-	-1,982324124	-2,049747196	0,067423072
208043_at	-	-1,982324124	-2,049747196	0,067423072
207751_at	-	-1,982324124	-2,049747196	0,067423072
1556907_at	ZNF474	-1,982324124	-2,049747196	0,067423072
239433_at	LRRC8E	-1,982324124	-2,049747196	0,067423072
221329_at	OR52A1	-1,982324124	-2,049747196	0,067423072
1560559_at	-	-1,982324124	-2,049747196	0,067423072

1555059_at	-	-1,982324124	-2,049747196	0,067423072
236534_at	BNIPL	-1,982324124	-2,049747196	0,067423072
1554963_at	-	-1,982324124	-2,049747196	0,067423072
243391_x_at	-	-1,982324124	-2,049747196	0,067423072
207264_at	KDELR3	-1,982324124	-2,049747196	0,067423072
241679_at	-	-1,982324124	-2,049747196	0,067423072
1570536_at	-	-1,982324124	-2,049747196	0,067423072
214023_x_at	TUBB2B	-1,982324124	-2,049747196	0,067423072
223986_x_at	DMRT2	-1,982324124	-2,049747196	0,067423072
231597_x_at	-	-1,982324124	-2,049747196	0,067423072
1552414_at	WFDC9	-1,982324124	-2,049747196	0,067423072
227140_at	INHBA	-1,982324124	-2,049747196	0,067423072
228790_at	FAM110B	-1,982324124	-2,049747196	0,067423072
210891_s_at	GTF2I /// GTF2IP1	4,885515979	4,818097083	0,067418895
203314_at	GTPBP6	1,960819106	1,893444329	0,067374777
226895_at	NFIC	1,287793882	1,220470658	0,067323224
241915_at	ACSM2B	-1,452713826	-1,520022123	0,067308297
229849_at	WIPF3	-1,452713826	-1,520022123	0,067308297
214379_at	HNRPDL	-1,452713826	-1,520022123	0,067308297
243721_at	C18orf61	-1,452713826	-1,520022123	0,067308297
231937_at	-	-1,452713826	-1,520022123	0,067308297
235636_at	-	-1,452713826	-1,520022123	0,067308297
243168_at	-	-1,452713826	-1,520022123	0,067308297
220887_at	C14orf162	-1,452713826	-1,520022123	0,067308297
244588_at	-	-1,452713826	-1,520022123	0,067308297
206406_at	SMCP	-1,452713826	-1,520022123	0,067308297
1562812_at	ZNF32-AS3	-1,452713826	-1,520022123	0,067308297
237558_at	-	-1,452713826	-1,520022123	0,067308297
1565582_at	-	-1,452713826	-1,520022123	0,067308297
238118_s_at	PPOX	-1,452713826	-1,520022123	0,067308297
1554916_a_at	JRK	-1,452713826	-1,520022123	0,067308297
239685_at	-	-1,452713826	-1,520022123	0,067308297
240831_at	-	-1,452713826	-1,520022123	0,067308297
217569_x_at	-	-1,452713826	-1,520022123	0,067308297
234829_at	C20orf57 /// DUSP	-1,452713826	-1,520022123	0,067308297
1561820_at	SCN8A	-1,452713826	-1,520022123	0,067308297
217260_x_at	IGHG1 /// SKAP2	-1,452713826	-1,520022123	0,067308297
207254_at	SLC15A1	-1,452713826	-1,520022123	0,067308297
243129_at	LOC642980	-1,452713826	-1,520022123	0,067308297
238691_at	SNHG10	-1,452713826	-1,520022123	0,067308297
224049_at	KCNK17	-1,452713826	-1,520022123	0,067308297
219867_at	CHODL	-1,452713826	-1,520022123	0,067308297
241768_at	-	-1,452713826	-1,520022123	0,067308297
230595_at	LOC100653336 ///	-1,452713826	-1,520022123	0,067308297
208302_at	HMHB1	-1,452713826	-1,520022123	0,067308297
233076_at	JAKMIP3	-1,452713826	-1,520022123	0,067308297
220157_x_at	PLEKHA8P1	1,97249974	1,905294823	0,067204916
238715_at	LOC646014	-0,384193355	-0,45121326	0,067019905
202738_s_at	PHKB	1,871521385	1,804519951	0,067001434
201978_s_at	KIAA0141	2,507817181	2,440833194	0,066983988

209479_at	CCDC28A	4,106824781	4,040084244	0,066740537
238416_x_at	ZC3H18	-0,135065865	-0,201789721	0,066723856
1554043_a_at	-	-0,135065865	-0,201789721	0,066723856
239284_at	-	-0,135065865	-0,201789721	0,066723856
221294_at	GPR21	-0,135065865	-0,201789721	0,066723856
217159_x_at	SIGLEC7	-0,135065865	-0,201789721	0,066723856
219400_at	CNTNAP1	-0,135065865	-0,201789721	0,066723856
204281_at	TEAD4	-0,135065865	-0,201789721	0,066723856
225449_at	RDH13	0,244681185	0,178191865	0,06648932
1569663_at	-	-2,569309811	-2,635549085	0,066239273
1568711_a_at	LOC100507498	-2,569309811	-2,635549085	0,066239273
228706_s_at	CLDN23	-2,569309811	-2,635549085	0,066239273
1553024_at	DAOA-AS1	-2,569309811	-2,635549085	0,066239273
242516_x_at	RBM46	-2,569309811	-2,635549085	0,066239273
1567387_at	-	-2,569309811	-2,635549085	0,066239273
1553915_at	C10orf126	-2,569309811	-2,635549085	0,066239273
1562579_at	-	-2,569309811	-2,635549085	0,066239273
242948_x_at	-	-2,569309811	-2,635549085	0,066239273
1568644_at	ZNF208	-2,569309811	-2,635549085	0,066239273
1560202_at	LOC338739	-2,569309811	-2,635549085	0,066239273
1553266_at	CNOT6L	-2,569309811	-2,635549085	0,066239273
1559621_at	-	-2,569309811	-2,635549085	0,066239273
1570080_at	-	-2,569309811	-2,635549085	0,066239273
244613_at	-	-2,569309811	-2,635549085	0,066239273
237874_at	-	-2,569309811	-2,635549085	0,066239273
1554190_s_at	PLEKHS1	-2,569309811	-2,635549085	0,066239273
241975_at	-	-2,569309811	-2,635549085	0,066239273
1562009_a_at	-	-2,569309811	-2,635549085	0,066239273
231711_at	ACPP	-2,569309811	-2,635549085	0,066239273
219795_at	SLC6A14	-2,569309811	-2,635549085	0,066239273
221057_at	SPATA1	-2,569309811	-2,635549085	0,066239273
240863_at	CYP19A1	-2,569309811	-2,635549085	0,066239273
215617_at	SPATS2L	-2,569309811	-2,635549085	0,066239273
1563331_at	-	-2,569309811	-2,635549085	0,066239273
1561581_at	-	-2,569309811	-2,635549085	0,066239273
231678_s_at	ADH4	-2,569309811	-2,635549085	0,066239273
207276_at	CDR1	-2,569309811	-2,635549085	0,066239273
1556448_a_at	LINC00606	-2,569309811	-2,635549085	0,066239273
1553605_a_at	ABCA13	-2,569309811	-2,635549085	0,066239273
1562656_at	-	-2,163157732	-2,229233437	0,066075706
1564372_s_at	CASC2	-2,163157732	-2,229233437	0,066075706
223873_s_at	-	-2,163157732	-2,229233437	0,066075706
210464_at	LINC00588	-2,163157732	-2,229233437	0,066075706
234221_at	-	-2,163157732	-2,229233437	0,066075706
240132_at	-	-2,163157732	-2,229233437	0,066075706
1560712_at	TMPRSS11B	-2,163157732	-2,229233437	0,066075706
234129_at	ARHGEF12	-2,163157732	-2,229233437	0,066075706
1561262_at	-	-2,163157732	-2,229233437	0,066075706
1559792_at	-	-2,163157732	-2,229233437	0,066075706
1555373_at	-	-2,163157732	-2,229233437	0,066075706

234596_at	-	-2,163157732	-2,229233437	0,066075706
230545_at	LOC100505601	-2,163157732	-2,229233437	0,066075706
241162_at	-	-2,163157732	-2,229233437	0,066075706
1561087_at	-	-2,163157732	-2,229233437	0,066075706
232738_at	FAM190A	-2,163157732	-2,229233437	0,066075706
201062_at	STOM	-2,163157732	-2,229233437	0,066075706
1554165_at	FAM53B	-2,163157732	-2,229233437	0,066075706
231127_at	-	-2,163157732	-2,229233437	0,066075706
208462_s_at	ABCC9	-2,163157732	-2,229233437	0,066075706
207285_x_at	CSHL1	-2,163157732	-2,229233437	0,066075706
1562546_at	-	-2,163157732	-2,229233437	0,066075706
207588_at	-	-2,163157732	-2,229233437	0,066075706
1552839_at	FAM71D	-2,163157732	-2,229233437	0,066075706
1553006_at	GPR112	-2,163157732	-2,229233437	0,066075706
232303_at	ZNF608	-2,163157732	-2,229233437	0,066075706
235641_at	TRIB1	-2,163157732	-2,229233437	0,066075706
230718_at	HSF5	-2,163157732	-2,229233437	0,066075706
235979_at	C7	-2,163157732	-2,229233437	0,066075706
1566217_at	-	-2,163157732	-2,229233437	0,066075706
1554745_at	RALGPS1	-2,163157732	-2,229233437	0,066075706
244720_at	-	-2,163157732	-2,229233437	0,066075706
243526_at	WDR86	-2,163157732	-2,229233437	0,066075706
1559523_at	-	-2,163157732	-2,229233437	0,066075706
1553492_a_at	PAX1	-2,163157732	-2,229233437	0,066075706
215524_x_at	MGC40069 /// TR/	-2,163157732	-2,229233437	0,066075706
238636_at	CACNA1C	-2,163157732	-2,229233437	0,066075706
244814_at	UGGT1	0,127732831	0,061773582	0,065959249
1555852_at	LOC100507463	2,983826368	2,918087241	0,065739127
218941_at	FBXW2	2,605420261	2,539809146	0,065611114
213890_x_at	RPS16	8,196648553	8,131362173	0,06528638
231194_at	SNX32	-0,66934516	-0,734355396	0,065010236
240464_at	-	-0,66934516	-0,734355396	0,065010236
217397_at	YME1L1	-0,66934516	-0,734355396	0,065010236
1554625_at	BCL6B	-0,66934516	-0,734355396	0,065010236
238438_at	CNOT6L	-0,66934516	-0,734355396	0,065010236
244124_at	RAB37	-0,66934516	-0,734355396	0,065010236
215905_s_at	SNRNP40	4,046717183	3,981792554	0,064924629
203489_at	SIVA1	3,67542249	3,610508368	0,064914122
212324_s_at	VPS13D	0,791908897	0,727123109	0,064785789
222481_at	FXC1	2,771643417	2,707013102	0,064630315
221971_x_at	AGAP10 /// AGAP1	2,063971513	1,999354538	0,064616975
228888_at	STAC2	0,648195588	0,583607503	0,064588085
232682_at	MREG	1,745502609	1,680963921	0,064538688
201934_at	WDR82	5,28476506	5,2204235	0,06434156
238078_at	SEC22A	0,39105295	0,326777209	0,064275741
205953_at	LRIG2	1,024284941	0,960042218	0,064242723
214299_at	TOP3A	1,024284941	0,960042218	0,064242723
212781_at	RBBP6	3,012642182	2,948635566	0,064006616
201100_s_at	USP9X	4,789213414	4,725383431	0,063829983
212546_s_at	FRYL	3,200057698	3,136334485	0,063723212

214394_x_at	EEF1D	6,998777218	6,935172837	0,063604381
1569947_at	-	-1,893506789	-1,956681069	0,063174281
1565900_at	METTL15	-1,893506789	-1,956681069	0,063174281
1563523_at	-	-1,893506789	-1,956681069	0,063174281
223878_at	INPP4B	-1,893506789	-1,956681069	0,063174281
216632_at	NAV3	-1,893506789	-1,956681069	0,063174281
240228_at	CSMD3	-1,893506789	-1,956681069	0,063174281
234028_at	-	-1,893506789	-1,956681069	0,063174281
243184_at	-	-1,893506789	-1,956681069	0,063174281
224209_s_at	GDA	-1,893506789	-1,956681069	0,063174281
233172_at	-	-1,893506789	-1,956681069	0,063174281
220116_at	KCNN2	-1,893506789	-1,956681069	0,063174281
1559804_at	-	-1,893506789	-1,956681069	0,063174281
1558101_at	NFIA	-1,893506789	-1,956681069	0,063174281
1563528_at	RAPGEF4-AS1	-1,893506789	-1,956681069	0,063174281
1561398_at	-	-1,893506789	-1,956681069	0,063174281
1562311_at	-	-1,893506789	-1,956681069	0,063174281
1562023_at	-	-1,893506789	-1,956681069	0,063174281
241790_at	-	-1,893506789	-1,956681069	0,063174281
232947_at	-	-1,893506789	-1,956681069	0,063174281
1560898_at	-	-1,893506789	-1,956681069	0,063174281
1566777_at	-	-1,893506789	-1,956681069	0,063174281
1559711_at	FAM120A	-1,893506789	-1,956681069	0,063174281
201983_s_at	EGFR	-1,893506789	-1,956681069	0,063174281
1557517_a_at	-	-1,893506789	-1,956681069	0,063174281
224235_at	-	-1,893506789	-1,956681069	0,063174281
239546_at	LOC100131053	-1,893506789	-1,956681069	0,063174281
1560619_at	LOC255411	-1,893506789	-1,956681069	0,063174281
209601_at	ACOX1	-1,893506789	-1,956681069	0,063174281
241097_at	-	-1,893506789	-1,956681069	0,063174281
1565849_a_at	SRRM5	-1,893506789	-1,956681069	0,063174281
230862_at	-	-1,893506789	-1,956681069	0,063174281
242591_at	DNAH1	-1,893506789	-1,956681069	0,063174281
229510_at	MS4A14	-1,893506789	-1,956681069	0,063174281
234658_at	-	-1,893506789	-1,956681069	0,063174281
214503_x_at	GPR135	-1,893506789	-1,956681069	0,063174281
1554923_at	ANKS6	-1,893506789	-1,956681069	0,063174281
221343_at	OR11A1	-1,893506789	-1,956681069	0,063174281
223859_at	EPB41L4B	-1,893506789	-1,956681069	0,063174281
1563117_at	LOC388456	-1,893506789	-1,956681069	0,063174281
207772_s_at	PRMT8	-1,893506789	-1,956681069	0,063174281
221416_at	PLA2G2F	-1,893506789	-1,956681069	0,063174281
205364_at	ACOX2	-1,893506789	-1,956681069	0,063174281
241095_at	-	-1,893506789	-1,956681069	0,063174281
1562988_at	ZSCAN30	-1,893506789	-1,956681069	0,063174281
242064_at	SDK2	-1,893506789	-1,956681069	0,063174281
227694_at	C1orf201	1,071336699	1,008210238	0,063126461
229967_at	CMTM2	-0,994186487	-1,057301851	0,063115364
221409_at	OR2S2	-0,994186487	-1,057301851	0,063115364
220719_at	-	-0,994186487	-1,057301851	0,063115364

1553016_at	GPR113	-0,994186487	-1,057301851	0,063115364
233583_at	-	-0,994186487	-1,057301851	0,063115364
1552813_at	KLF14	-0,994186487	-1,057301851	0,063115364
237948_at	-	-0,994186487	-1,057301851	0,063115364
1567624_at	TREML5P	-0,994186487	-1,057301851	0,063115364
211570_s_at	RAPSN	-0,994186487	-1,057301851	0,063115364
236762_at	ATP9B	-0,994186487	-1,057301851	0,063115364
239560_at	-	-0,994186487	-1,057301851	0,063115364
204819_at	FGD1	-0,994186487	-1,057301851	0,063115364
205338_s_at	DCT	-0,994186487	-1,057301851	0,063115364
214058_at	MYCL1	-0,994186487	-1,057301851	0,063115364
236233_at	TRIM32	-0,994186487	-1,057301851	0,063115364
223280_x_at	MS4A6A	-0,994186487	-1,057301851	0,063115364
231570_at	FAM154A	-0,994186487	-1,057301851	0,063115364
240514_at	PGA3	-0,994186487	-1,057301851	0,063115364
221835_at	DTX3	-0,994186487	-1,057301851	0,063115364
218058_at	CXXC1	1,610353504	1,547376668	0,062976836
200741_s_at	RPS27	8,436841886	8,374238909	0,062602977
215215_s_at	LOC81691	1,5944803	1,531877749	0,062602551
213344_s_at	H2AFX	1,671397663	1,60918327	0,062214392
223361_at	ABRACL	5,863737956	5,801563986	0,06217397
202950_at	CRYZ	3,406503548	3,344597303	0,061906245
222552_at	GOLT1B	3,402275492	3,340400371	0,061875121
227330_x_at	LOC100233156 ///	1,094118704	1,032267596	0,061851107
239833_at	-	-0,49284695	-0,554696666	0,061849716
243051_at	CNIH4	-0,49284695	-0,554696666	0,061849716
242499_at	U2AF1	-0,49284695	-0,554696666	0,061849716
1562657_a_at	C10orf90	-0,49284695	-0,554696666	0,061849716
1562937_at	-	-0,49284695	-0,554696666	0,061849716
1552676_at	UCN3	-0,49284695	-0,554696666	0,061849716
1559110_at	-	-0,49284695	-0,554696666	0,061849716
238836_at	-	-0,49284695	-0,554696666	0,061849716
215600_x_at	FBXW12	1,898032047	1,836262316	0,061769731
1558225_at	-	-2,250580859	-2,312054429	0,06147357
220327_at	VGLL3	-2,250580859	-2,312054429	0,06147357
1556842_at	LOC286087	-2,250580859	-2,312054429	0,06147357
238533_at	EPHA7	-2,250580859	-2,312054429	0,06147357
205399_at	DCLK1	-2,250580859	-2,312054429	0,06147357
1567663_at	-	-2,250580859	-2,312054429	0,06147357
220393_at	LGSN	-2,250580859	-2,312054429	0,06147357
233744_at	-	-2,250580859	-2,312054429	0,06147357
237836_at	-	-2,250580859	-2,312054429	0,06147357
242529_x_at	-	-2,250580859	-2,312054429	0,06147357
1561642_at	-	-2,250580859	-2,312054429	0,06147357
234569_at	-	-2,250580859	-2,312054429	0,06147357
241967_at	-	-2,250580859	-2,312054429	0,06147357
237169_at	TNC	-2,250580859	-2,312054429	0,06147357
244655_at	LOC100507311	-2,250580859	-2,312054429	0,06147357
233798_at	-	-2,250580859	-2,312054429	0,06147357
241311_at	-	-2,250580859	-2,312054429	0,06147357

1553708_at	LINC00525	-2,250580859	-2,312054429	0,06147357
1553931_at	OSTCP1	-2,250580859	-2,312054429	0,06147357
220129_at	CCDC169-SOHLH2	-2,250580859	-2,312054429	0,06147357
1552771_a_at	LIN9	-2,250580859	-2,312054429	0,06147357
1561027_at	LOC646241	-2,250580859	-2,312054429	0,06147357
235770_at	MASP1	-2,250580859	-2,312054429	0,06147357
216951_at	FCGR1A	-2,250580859	-2,312054429	0,06147357
239420_at	LOC100507267	-2,250580859	-2,312054429	0,06147357
222922_at	KCNE3	-2,250580859	-2,312054429	0,06147357
1552801_at	CAPN13	-2,250580859	-2,312054429	0,06147357
243143_at	FAM24A	-2,250580859	-2,312054429	0,06147357
234274_at	-	-2,250580859	-2,312054429	0,06147357
1558947_at	-	-2,250580859	-2,312054429	0,06147357
1557103_a_at	LMTK3	-2,250580859	-2,312054429	0,06147357
1557578_at	PHLDB2	-2,250580859	-2,312054429	0,06147357
232805_at	-	-2,250580859	-2,312054429	0,06147357
1564996_at	-	-2,250580859	-2,312054429	0,06147357
239003_at	-	-2,250580859	-2,312054429	0,06147357
234329_at	CLIC5	-2,250580859	-2,312054429	0,06147357
216276_s_at	ADAM3A	-2,250580859	-2,312054429	0,06147357
231669_at	SEPP1	-2,250580859	-2,312054429	0,06147357
216627_s_at	B4GALT1	-2,250580859	-2,312054429	0,06147357
240932_at	UBN2	-2,250580859	-2,312054429	0,06147357
240522_at	-	-2,250580859	-2,312054429	0,06147357
217564_s_at	CPS1	-2,250580859	-2,312054429	0,06147357
241848_x_at	-	-2,250580859	-2,312054429	0,06147357
243161_x_at	ZFP42	-2,250580859	-2,312054429	0,06147357
207952_at	IL5	-2,250580859	-2,312054429	0,06147357
225812_at	C6orf225	1,92317941	1,861868165	0,061311246
213009_s_at	TRIM37	3,912706782	3,851518797	0,061187985
202417_at	KEAP1	3,273854609	3,212688281	0,061166328
220925_at	NAA35	3,012642182	2,951484533	0,061157649
212100_s_at	POLDIP3	3,18076822	3,119733444	0,061034776
209208_at	MPDU1	1,88491011	1,823960141	0,06094997
223020_at	CLPTM1L	4,133352155	4,07248815	0,060864005
203810_at	DNAJB4	1,995527416	1,935144844	0,060382572
212066_s_at	USP34	4,515915598	4,455535827	0,060379771
1563253_s_at	ERBB3	-0,230432956	-0,290694975	0,060262019
243656_at	LOC642852	-0,230432956	-0,290694975	0,060262019
209969_s_at	STAT1	5,05583032	4,995602626	0,060227693
200074_s_at	RPL14	7,471800069	7,411695957	0,060104113
230466_s_at	RASSF3	2,492739983	2,432792627	0,059947356
204980_at	CLOCK	0,677745787	0,61779983	0,059945957
213074_at	PHIP	3,931608669	3,871747013	0,059861656
209224_s_at	NDUFA2	5,306335943	5,246483322	0,05985262
202612_s_at	MED14	-1,802716385	-1,862269442	0,059553057
1567527_at	-	-1,802716385	-1,862269442	0,059553057
235667_at	USP46-AS1	-1,802716385	-1,862269442	0,059553057
220699_s_at	KIF2A	-1,802716385	-1,862269442	0,059553057
241288_at	-	-1,802716385	-1,862269442	0,059553057

1563044_at	-	-1,802716385	-1,862269442	0,059553057
240081_at	-	-1,802716385	-1,862269442	0,059553057
207327_at	EYA4	-1,802716385	-1,862269442	0,059553057
1558534_at	LOC100271836 ///	-1,802716385	-1,862269442	0,059553057
242730_at	MYRIP	-1,802716385	-1,862269442	0,059553057
205712_at	PTPRD	-1,802716385	-1,862269442	0,059553057
241298_x_at	-	-1,802716385	-1,862269442	0,059553057
234837_at	-	-1,802716385	-1,862269442	0,059553057
1557576_at	PAQR3	-1,802716385	-1,862269442	0,059553057
1568846_at	-	-1,802716385	-1,862269442	0,059553057
238942_at	-	-1,802716385	-1,862269442	0,059553057
1562790_at	NCALD	-1,802716385	-1,862269442	0,059553057
1564008_at	COL27A1	-1,802716385	-1,862269442	0,059553057
232803_at	-	-1,802716385	-1,862269442	0,059553057
210764_s_at	CYR61	-1,802716385	-1,862269442	0,059553057
240183_at	TMEM213	-1,802716385	-1,862269442	0,059553057
231546_at	-	-1,802716385	-1,862269442	0,059553057
1560750_at	LOC151121	-1,802716385	-1,862269442	0,059553057
224181_at	LINC00470	-1,802716385	-1,862269442	0,059553057
236881_at	-	-1,802716385	-1,862269442	0,059553057
236063_at	-	-1,802716385	-1,862269442	0,059553057
225242_s_at	CCDC80	-1,802716385	-1,862269442	0,059553057
216106_at	LOC145678	-1,802716385	-1,862269442	0,059553057
238638_at	SLC37A2	-1,802716385	-1,862269442	0,059553057
234016_at	LOC90499	-1,802716385	-1,862269442	0,059553057
232808_at	-	-1,802716385	-1,862269442	0,059553057
223114_at	COQ5	3,414594082	3,355074548	0,059519533
222056_s_at	FAHD2A	2,989672611	2,930238085	0,059434526
222706_at	CWC25	1,402495885	1,343200523	0,059295361
221508_at	TAOK3	0,425036312	0,366031214	0,059005099
228433_at	NFYA	0,556428218	0,497424714	0,059003504
225742_at	MDM4	0,556428218	0,497424714	0,059003504
235026_at	C12orf66	1,493647653	1,434743608	0,058904045
214687_x_at	ALDOA	7,17966708	7,120767466	0,058899614
204328_at	TMC6	2,085817135	2,026957145	0,05885999
226254_s_at	KIAA1430	4,317231729	4,258615677	0,058616052
235461_at	TET2	0,873789652	0,815200271	0,058589381
235200_at	ZNF561	0,873789652	0,815200271	0,058589381
200922_at	KDELR1	1,760255626	1,701800759	0,058454866
214330_at	ATPAF2	0,167727503	0,109458907	0,058268597
203336_s_at	ITGB1BP1	5,095686705	5,037444404	0,058242301
211783_s_at	MTA1	3,023747815	2,965609445	0,058138371
201160_s_at	CSDA	5,964736786	5,906630572	0,058106214
226525_at	STK17B	4,496331177	4,438273285	0,058057892
208848_at	ADH5	3,813758784	3,755752479	0,058006305
1560966_at	-	-0,791294935	-0,84928999	0,057995055
234681_s_at	CHD6	-0,791294935	-0,84928999	0,057995055
233122_at	KRTCAP2	-0,791294935	-0,84928999	0,057995055
1554942_a_at	KLHL14	-0,791294935	-0,84928999	0,057995055
232558_at	LAMC3	-0,791294935	-0,84928999	0,057995055

210471_s_at	KCNAB1	-0,791294935	-0,84928999	0,057995055
227023_at	GLI4 /// ZFP41	-0,791294935	-0,84928999	0,057995055
1570071_at	-	-0,791294935	-0,84928999	0,057995055
211743_s_at	PRG2	-0,791294935	-0,84928999	0,057995055
243323_s_at	ZFHX3	-0,791294935	-0,84928999	0,057995055
201314_at	STK25	1,20396005	1,146010634	0,057949416
232323_s_at	TTC17	2,636121902	2,578283758	0,057838144
212694_s_at	PCCB	3,960533133	3,902746728	0,057786405
222432_s_at	CCDC47	3,570321555	3,512665066	0,05765649
242107_x_at	-	-2,774503732	-2,832036647	0,057532915
206201_s_at	MEOX2	-2,774503732	-2,832036647	0,057532915
203189_s_at	MIR4691 /// NDUF	4,005144537	3,947720163	0,057424374
201191_at	PITPNA	2,56794744	2,5107204	0,057227039
209954_x_at	SS18	1,345611839	1,28853674	0,057075099
226224_at	FOXK2	2,321828846	2,264929589	0,056899257
33814_at	PAK4	1,105794789	1,048946718	0,056848071
220682_s_at	-	0,045517965	-0,011001236	0,0565192
1556747_a_at	-	0,045517965	-0,011001236	0,0565192
226531_at	ORAI1	0,926325262	0,869824768	0,056500494
230490_x_at	RSU1	2,339581693	2,283169614	0,056412079
1558953_s_at	CEP164	2,2388565	2,182711118	0,056145382
1555447_at	GPR114	-0,088974936	-0,144765583	0,055790647
207833_s_at	HLCS	-0,088974936	-0,144765583	0,055790647
218556_at	ORMDL2	4,106824781	4,051142838	0,055681943
207157_s_at	GNG5	6,033185115	5,977512843	0,055672272
209330_s_at	HNRNPD	4,779371898	4,723859191	0,055512708
204176_at	KLHL20	2,031158928	1,975797592	0,055361336
1558107_at	LOC283788	-2,336678463	-2,391863529	0,055185065
1555929_s_at	-	-2,336678463	-2,391863529	0,055185065
1560959_a_at	-	-2,336678463	-2,391863529	0,055185065
237521_x_at	-	-2,336678463	-2,391863529	0,055185065
201789_at	DHRS7	-2,336678463	-2,391863529	0,055185065
1561216_at	-	-2,336678463	-2,391863529	0,055185065
230331_at	METTL21C	-2,336678463	-2,391863529	0,055185065
240420_at	AADACL2	-2,336678463	-2,391863529	0,055185065
207415_at	PLA2R1	-2,336678463	-2,391863529	0,055185065
230250_at	PTPRB	-2,336678463	-2,391863529	0,055185065
1559370_at	DAB2	-2,336678463	-2,391863529	0,055185065
215065_at	PHF8	-2,336678463	-2,391863529	0,055185065
1569122_at	-	-2,336678463	-2,391863529	0,055185065
234428_at	-	-2,336678463	-2,391863529	0,055185065
207932_at	IFNA8	-2,336678463	-2,391863529	0,055185065
207174_at	GPC5	-2,336678463	-2,391863529	0,055185065
1561795_at	-	-2,336678463	-2,391863529	0,055185065
240591_at	-	-2,336678463	-2,391863529	0,055185065
239291_at	-	-2,336678463	-2,391863529	0,055185065
214410_at	-	-2,336678463	-2,391863529	0,055185065
239043_at	ZNF404	-2,336678463	-2,391863529	0,055185065
1559884_at	CDKN2B-AS1	-2,336678463	-2,391863529	0,055185065
243681_at	SHANK2	-2,336678463	-2,391863529	0,055185065

207780_at	CYLC2	-2,336678463	-2,391863529	0,055185065
210852_s_at	AASS	-2,336678463	-2,391863529	0,055185065
240980_at	-	-2,336678463	-2,391863529	0,055185065
220845_at	ACOXL	-2,336678463	-2,391863529	0,055185065
208543_at	OR10H2	-2,336678463	-2,391863529	0,055185065
1561143_at	-	-2,336678463	-2,391863529	0,055185065
242771_at	TTN	-2,336678463	-2,391863529	0,055185065
241145_at	-	-2,336678463	-2,391863529	0,055185065
1555485_s_at	FAM153B /// LOC1	-2,336678463	-2,391863529	0,055185065
231279_at	PATE1	-2,336678463	-2,391863529	0,055185065
1560810_at	-	-2,336678463	-2,391863529	0,055185065
243607_at	-	-2,336678463	-2,391863529	0,055185065
236521_at	-	-2,336678463	-2,391863529	0,055185065
234331_s_at	FAM84A	-2,336678463	-2,391863529	0,055185065
231615_at	-	-2,336678463	-2,391863529	0,055185065
1566126_at	-	-2,336678463	-2,391863529	0,055185065
1558944_at	LOC100507353	-2,336678463	-2,391863529	0,055185065
221689_s_at	PIGP	4,758446588	4,7035806	0,054865988
201161_s_at	CSDA	4,931690933	4,876912218	0,054778716
235280_at	POLR1A	0,58788177	0,533258442	0,054623329
214813_at	ZNF75D	-0,332405896	-0,386896102	0,054490206
237171_at	-	-0,332405896	-0,386896102	0,054490206
237872_at	LOC100505697	-0,332405896	-0,386896102	0,054490206
224670_at	SYS1	2,436165655	2,381744287	0,054421368
1567878_at	DEFB114	-2,641232718	-2,695640852	0,054408134
206935_at	PCDH8	-2,641232718	-2,695640852	0,054408134
1558900_at	-	-2,641232718	-2,695640852	0,054408134
1570633_at	-	-2,641232718	-2,695640852	0,054408134
211239_s_at	ADAM7	-2,641232718	-2,695640852	0,054408134
244386_at	-	-2,641232718	-2,695640852	0,054408134
241587_at	NAALADL2-AS3	-2,641232718	-2,695640852	0,054408134
235503_at	ASB5	-2,641232718	-2,695640852	0,054408134
209632_at	PPP2R3A	-2,641232718	-2,695640852	0,054408134
244111_at	KRT222	-2,641232718	-2,695640852	0,054408134
235489_at	RHOJ	-2,641232718	-2,695640852	0,054408134
221894_at	ADCK2	0,735999505	0,681874787	0,054124718
213041_s_at	ATP5D	4,030423661	3,976310891	0,054112771
202538_s_at	CHMP2B	3,309267982	3,255452586	0,053815396
202836_s_at	TXNL4A	5,521132257	5,467358367	0,05377389
204050_s_at	CLTA	4,938142664	4,884438794	0,05370387
1567273_at	OR2K2	-1,137208348	-1,19085694	0,053648591
233033_at	ZEB2	-1,137208348	-1,19085694	0,053648591
1562970_at	-	-1,137208348	-1,19085694	0,053648591
244135_at	-	-1,137208348	-1,19085694	0,053648591
242174_at	-	-1,137208348	-1,19085694	0,053648591
234447_at	-	-1,137208348	-1,19085694	0,053648591
216718_at	LINC00302	-1,137208348	-1,19085694	0,053648591
216240_at	MIR1204	-1,137208348	-1,19085694	0,053648591
230660_at	SERTAD4	-1,137208348	-1,19085694	0,053648591
222766_at	POLR3K	-1,137208348	-1,19085694	0,053648591

236693_at	LOC100130155	-1,137208348	-1,19085694	0,053648591
215753_at	-	-1,137208348	-1,19085694	0,053648591
231147_at	-	-1,137208348	-1,19085694	0,053648591
1560812_at	-	-1,137208348	-1,19085694	0,053648591
241528_at	MAPK15	-1,137208348	-1,19085694	0,053648591
243156_at	-	-1,137208348	-1,19085694	0,053648591
237371_at	-	-1,137208348	-1,19085694	0,053648591
1558179_at	ATP5J2	-1,137208348	-1,19085694	0,053648591
206330_s_at	SHC3	-1,137208348	-1,19085694	0,053648591
219171_s_at	ZNF236	-1,137208348	-1,19085694	0,053648591
227371_at	BAIAP2L1	-1,137208348	-1,19085694	0,053648591
1552868_at	CIRBP-AS1	0,458905032	0,405752839	0,053152193
213584_s_at	CREBZF	0,458905032	0,405752839	0,053152193
1568592_at	TRIM69	3,282538749	3,229534127	0,053004623
233933_s_at	OTUD5	1,024284941	0,971607464	0,052677477
208496_x_at	HIST1H3A /// HIST	1,024284941	0,971607464	0,052677477
224627_at	GBA2	1,024284941	0,971607464	0,052677477
241253_at	-	-1,369772723	-1,422257026	0,052484304
216742_at	-	-1,369772723	-1,422257026	0,052484304
236651_at	KALRN	-1,369772723	-1,422257026	0,052484304
211718_at	MGC2889	-1,369772723	-1,422257026	0,052484304
216970_at	RGS7	-1,369772723	-1,422257026	0,052484304
1565002_at	-	-1,369772723	-1,422257026	0,052484304
235137_at	PCBP1-AS1	-1,369772723	-1,422257026	0,052484304
1569746_s_at	LOC100505783	-1,369772723	-1,422257026	0,052484304
232413_at	-	-1,369772723	-1,422257026	0,052484304
234637_at	KRTAP4-5	-1,369772723	-1,422257026	0,052484304
231172_at	C9orf117	-1,369772723	-1,422257026	0,052484304
219606_at	PHF20L1	-1,369772723	-1,422257026	0,052484304
1555338_s_at	AQP10	-1,369772723	-1,422257026	0,052484304
1560913_at	-	-1,369772723	-1,422257026	0,052484304
244259_s_at	-	-1,369772723	-1,422257026	0,052484304
232929_at	-	-1,369772723	-1,422257026	0,052484304
244577_at	-	-1,369772723	-1,422257026	0,052484304
226508_at	PHC3	3,505636113	3,453397238	0,052238875
235597_s_at	RGPD1 /// RGPD2	-0,609040214	-0,661178575	0,052138361
1565701_at	-	-0,609040214	-0,661178575	0,052138361
202087_s_at	CTSL1	-0,609040214	-0,661178575	0,052138361
208675_s_at	DDOST	6,002256791	5,950255498	0,052001293
232037_at	IGDCC3	0,20740896	0,155747281	0,05166168
231059_x_at	SCAND1	2,860630824	2,809015785	0,051615039
228151_at	ARL15	1,510970097	1,459545271	0,051424826
210115_at	RPL39L	2,765070895	2,713775735	0,05129516
201736_s_at	06.03.15	3,473999051	3,422991983	0,051007068
243130_at	-	-1,712732543	-1,763664074	0,050931531
204337_at	RGS4	-1,712732543	-1,763664074	0,050931531
240525_at	-	-1,712732543	-1,763664074	0,050931531
1562348_at	LOC400680	-1,712732543	-1,763664074	0,050931531
216773_at	-	-1,712732543	-1,763664074	0,050931531
1570292_at	-	-1,712732543	-1,763664074	0,050931531

220333_at	PAQR5	-1,712732543	-1,763664074	0,050931531
212336_at	EPB41L1	-1,712732543	-1,763664074	0,050931531
217288_at	-	-1,712732543	-1,763664074	0,050931531
241711_at	-	-1,712732543	-1,763664074	0,050931531
206071_s_at	EPHA3	-1,712732543	-1,763664074	0,050931531
206191_at	ENTPD3	-1,712732543	-1,763664074	0,050931531
207865_s_at	BMP8B	-1,712732543	-1,763664074	0,050931531
1557633_at	POM121L8P	-1,712732543	-1,763664074	0,050931531
1553675_at	KLC4	-1,712732543	-1,763664074	0,050931531
1561169_at	-	-1,712732543	-1,763664074	0,050931531
1554008_at	OSMR	-1,712732543	-1,763664074	0,050931531
219912_s_at	-	-1,712732543	-1,763664074	0,050931531
217689_at	PTPN1	-1,712732543	-1,763664074	0,050931531
229049_at	LOC100506655	-1,712732543	-1,763664074	0,050931531
1554203_at	GRIK1-AS1	-1,712732543	-1,763664074	0,050931531
236972_at	TRIM63	-1,712732543	-1,763664074	0,050931531
244172_at	-	-1,712732543	-1,763664074	0,050931531
1563391_at	-	-1,712732543	-1,763664074	0,050931531
231373_at	RNF133	-1,712732543	-1,763664074	0,050931531
1552506_at	CRB2	-1,712732543	-1,763664074	0,050931531
1566853_at	-	-1,712732543	-1,763664074	0,050931531
225790_at	MSRB3	-1,712732543	-1,763664074	0,050931531
208133_at	-	-1,712732543	-1,763664074	0,050931531
213638_at	PHACTR1	-1,712732543	-1,763664074	0,050931531
1564591_a_at	TMC1	-1,712732543	-1,763664074	0,050931531
235379_at	LOC100506114	-1,712732543	-1,763664074	0,050931531
236618_at	C20orf132	-1,712732543	-1,763664074	0,050931531
218114_at	GGA1	1,071336699	1,020514937	0,050821762
214525_x_at	MLH3	1,071336699	1,020514937	0,050821762
209972_s_at	AIMP2	1,071336699	1,020514937	0,050821762
1554767_s_at	CRYZL1	1,440265662	1,390070295	0,050195367
207292_s_at	MAPK7	0,618277321	0,568155442	0,050121879
241839_at	-	-0,180990326	-0,231045907	0,050055581
1557714_at	CTBP1	-0,180990326	-0,231045907	0,050055581
216730_at	-	-0,180990326	-0,231045907	0,050055581
214354_x_at	SFTPB	-0,180990326	-0,231045907	0,050055581
1562245_a_at	ZNF578	-2,416625063	-2,466349414	0,049724351
224405_at	FCRL5	-2,416625063	-2,466349414	0,049724351
242815_x_at	-	-2,416625063	-2,466349414	0,049724351
210893_at	-	-2,416625063	-2,466349414	0,049724351
232275_s_at	HS6ST3	-2,416625063	-2,466349414	0,049724351
1558428_at	ALS2CR11	-2,416625063	-2,466349414	0,049724351
241649_at	-	-2,416625063	-2,466349414	0,049724351
1553317_s_at	GPR82	-2,416625063	-2,466349414	0,049724351
215312_at	-	-2,416625063	-2,466349414	0,049724351
210738_s_at	SLC4A4	-2,416625063	-2,466349414	0,049724351
1561482_at	LOC100133308	-2,416625063	-2,466349414	0,049724351
1554012_at	RSPO2	-2,416625063	-2,466349414	0,049724351
1560842_a_at	TEX26-AS1	-2,416625063	-2,466349414	0,049724351
1555153_s_at	FCHO2	-2,416625063	-2,466349414	0,049724351

244134_at	-	-2,416625063	-2,466349414	0,049724351
1552950_at	C15orf26	-2,416625063	-2,466349414	0,049724351
236385_at	-	-2,416625063	-2,466349414	0,049724351
211606_at	-	-2,416625063	-2,466349414	0,049724351
1554233_at	C1QTNF9	-2,416625063	-2,466349414	0,049724351
240596_at	-	-2,416625063	-2,466349414	0,049724351
1554272_at	ERAP2	-2,416625063	-2,466349414	0,049724351
1570300_at	-	-2,416625063	-2,466349414	0,049724351
1560895_at	LOC645188	-2,416625063	-2,466349414	0,049724351
238371_s_at	-	-2,416625063	-2,466349414	0,049724351
210330_at	SGCD	-2,416625063	-2,466349414	0,049724351
1566787_at	-	-2,416625063	-2,466349414	0,049724351
238835_at	AVPR1A	-2,416625063	-2,466349414	0,049724351
239805_at	SLC13A2	-2,416625063	-2,466349414	0,049724351
210572_at	PCDHA2	-2,416625063	-2,466349414	0,049724351
1554131_at	FAM190B	-2,416625063	-2,466349414	0,049724351
1565896_at	-	-2,416625063	-2,466349414	0,049724351
209493_at	PDZD2	-2,416625063	-2,466349414	0,049724351
207256_at	MBL2	-2,416625063	-2,466349414	0,049724351
1570471_at	ARHGEF12	-2,416625063	-2,466349414	0,049724351
227064_at	ANKRD40	2,752615529	2,703479652	0,049135877
211209_x_at	SH2D1A	1,716003175	1,666953618	0,049049557
223509_at	CLDN2	-0,924000698	-0,972893339	0,048892641
238616_at	QDPR	-0,924000698	-0,972893339	0,048892641
215511_at	TCF20	-0,924000698	-0,972893339	0,048892641
209985_s_at	ASCL1	-0,924000698	-0,972893339	0,048892641
209212_s_at	KLF5	-0,924000698	-0,972893339	0,048892641
243921_at	-	-0,924000698	-0,972893339	0,048892641
244310_at	-	-0,924000698	-0,972893339	0,048892641
231236_at	ZFP57	-0,924000698	-0,972893339	0,048892641
243820_at	LOC401463	-0,924000698	-0,972893339	0,048892641
1563340_at	-	-0,924000698	-0,972893339	0,048892641
1569430_at	PTRH1	-0,924000698	-0,972893339	0,048892641
1552672_a_at	IGSF3	-0,924000698	-0,972893339	0,048892641
1556092_s_at	HEMK1	-0,924000698	-0,972893339	0,048892641
242484_at	TTC9B	-0,924000698	-0,972893339	0,048892641
1565558_at	-	-0,924000698	-0,972893339	0,048892641
212734_x_at	RPL13 /// SNORD6	8,140048079	8,091456231	0,048591849
227928_at	PARPBP	1,287793882	1,239268196	0,048525687
231338_at	C15orf55	0,08619576	0,03784737	0,04834839
219148_at	PBK	5,112028025	5,063728887	0,048299138
205443_at	SNAPC1	2,649978302	2,601822502	0,048155801
212380_at	FTSJD2	2,530390387	2,482273566	0,048116821
222201_s_at	CASP8AP2	3,142467702	3,094388414	0,048079288
225859_at	XIAP	1,671397663	1,623485524	0,047912139
41512_at	BRAP	2,4401817	2,392280877	0,047900823
224844_at	SLAIN2	3,060716358	3,012928641	0,047787717
216554_s_at	ENO1	2,657308324	2,609615156	0,047693168
209879_at	SELPLG	3,588695979	3,54107059	0,047625389
200747_s_at	NUMA1	2,575362572	2,527778509	0,047584063

218190_s_at	UQCR10	6,095517202	6,047976241	0,047540961
228990_at	SNHG12 /// SNOR/	2,983826368	2,936370826	0,047455542
200630_x_at	SET	6,766210199	6,71882272	0,047387479
207231_at	DZIP3	1,345611839	1,298349073	0,047262765
230873_at	ARPC4-TTLL3 /// T	-0,437449947	-0,484558493	0,047108546
217187_at	MUC5AC	-0,437449947	-0,484558493	0,047108546
242843_at	-	-0,437449947	-0,484558493	0,047108546
229164_s_at	ABTB1	1,225123479	1,178100917	0,047022562
219137_s_at	MFF	4,270249493	4,22363244	0,046617053
217856_at	RBM8A	1,625482993	1,578991006	0,046491988
201838_s_at	SUPT7L	0,847529938	0,80114751	0,046382428
208055_s_at	HERC4	0,847529938	0,80114751	0,046382428
243955_at	-	-0,043925712	-0,089635713	0,045710001
1554206_at	TMLHE	-0,043925712	-0,089635713	0,045710001
202627_s_at	SERPINE1	-0,043925712	-0,089635713	0,045710001
201492_s_at	RPL41	8,777333331	8,731816628	0,045516703
205060_at	PARG	2,642918178	2,597466271	0,045451908
213911_s_at	H2AFZ	7,823586472	7,778328814	0,045257658
212513_s_at	USP33	3,822956033	3,77783183	0,045124203
226353_at	SPPL2A	4,055088167	4,010484498	0,044603669
1565951_s_at	CHML	0,873789652	0,829309537	0,044480115
210224_at	MR1	1,760255626	1,715863686	0,04439194
215091_s_at	GTF3A	7,078426659	7,034340217	0,044086442
230290_at	SCUBE3	0,244681185	0,20062106	0,044060126
229176_at	ANKH	0,244681185	0,20062106	0,044060126
232926_x_at	ANKRD19P	0,244681185	0,20062106	0,044060126
215716_s_at	ATP2B1	2,811067138	2,767331883	0,043735254
224636_at	ZFP91	3,840618883	3,796883855	0,043735028
1555925_at	-	-2,708365344	-2,752097204	0,04373186
1560372_at	-	-2,708365344	-2,752097204	0,04373186
237937_x_at	-	-2,708365344	-2,752097204	0,04373186
232712_at	-	-2,708365344	-2,752097204	0,04373186
216709_at	-	-2,708365344	-2,752097204	0,04373186
220972_s_at	KRTAP9-9	-2,708365344	-2,752097204	0,04373186
233297_s_at	CCDC169 /// CCDC	-2,708365344	-2,752097204	0,04373186
1559965_at	ZFHX4-AS1	-2,708365344	-2,752097204	0,04373186
228440_at	-	-2,708365344	-2,752097204	0,04373186
1553604_at	ABCA13	-2,708365344	-2,752097204	0,04373186
216307_at	DGKB	-2,708365344	-2,752097204	0,04373186
202140_s_at	CLK3	2,492739983	2,449078728	0,043661255
200994_at	IPO7	5,31516215	5,271501086	0,043661064
206452_x_at	PPP2R4	0,900065578	0,85655369	0,043511888
211691_x_at	-	0,900065578	0,85655369	0,043511888
223093_at	ANKH	1,97249974	1,929092307	0,043407433
238554_at	CYB5B	1,730929079	1,687645398	0,043283681
221873_at	ZNF143	2,50022283	2,456960019	0,043262811
237561_x_at	-	0,926325262	0,883374248	0,042951014
223182_s_at	AGPAT3	3,050461597	3,007560601	0,042900996
201350_at	FLOT2	2,732422196	2,689594859	0,042827338
209091_s_at	SH3GLB1	4,454387658	4,411660604	0,042727054

219418_at	NHEJ1 /// SLC23A3	1,686984721	1,644429915	0,042554806
237289_at	CREB1	2,063971513	2,021531611	0,042439902
202786_at	STK39	3,493994216	3,451657924	0,042336293
1562026_at	WNK2	0,127732831	0,085534992	0,042197838
242015_x_at	APEH	-0,280273599	-0,322340048	0,042066449
218865_at	MARC1	-0,280273599	-0,322340048	0,042066449
201333_s_at	ARHGEF12	-0,280273599	-0,322340048	0,042066449
221774_x_at	FAM48A	2,752615529	2,710570973	0,042044555
1558943_x_at	ZNF765	1,92317941	1,881145546	0,042033864
210733_at	-	1,92317941	1,881145546	0,042033864
200880_at	DNAJA1	4,93324721	4,891450703	0,041796506
219307_at	PDSS2	1,871521385	1,830129603	0,041391782
1560255_at	C10orf31	-0,730013898	-0,771340337	0,041326439
220379_at	FSCN3	-0,730013898	-0,771340337	0,041326439
230601_s_at	LRRC46	-0,730013898	-0,771340337	0,041326439
233398_at	-	-0,730013898	-0,771340337	0,041326439
230611_at	SYPL2	-0,730013898	-0,771340337	0,041326439
221592_at	TBC1D8	-0,730013898	-0,771340337	0,041326439
222307_at	LOC282997	-0,730013898	-0,771340337	0,041326439
206217_at	EDA	-0,730013898	-0,771340337	0,041326439
211701_s_at	TRO	-0,730013898	-0,771340337	0,041326439
238741_at	FAM83A	-0,730013898	-0,771340337	0,041326439
211084_x_at	PRKD3	2,560606634	2,519368159	0,041238475
1560980_a_at	-	-2,495152701	-2,536339282	0,041186581
1562991_at	ZNF292	-2,495152701	-2,536339282	0,041186581
1560425_s_at	LOC100506247	-2,495152701	-2,536339282	0,041186581
224351_at	LOC100131508	-2,495152701	-2,536339282	0,041186581
217709_at	-	-2,495152701	-2,536339282	0,041186581
1553356_at	-	-2,495152701	-2,536339282	0,041186581
227827_at	-	-2,495152701	-2,536339282	0,041186581
243753_at	C1orf173	-2,495152701	-2,536339282	0,041186581
1561200_at	VWA3B	-2,495152701	-2,536339282	0,041186581
239250_at	ZNF542	-2,495152701	-2,536339282	0,041186581
240718_at	-	-2,495152701	-2,536339282	0,041186581
221950_at	EMX2	-2,495152701	-2,536339282	0,041186581
231494_at	-	-2,495152701	-2,536339282	0,041186581
1556439_at	-	-2,495152701	-2,536339282	0,041186581
202196_s_at	DKK3	-2,495152701	-2,536339282	0,041186581
208288_at	ABCB11	-2,495152701	-2,536339282	0,041186581
242604_at	-	-2,495152701	-2,536339282	0,041186581
210146_x_at	LILRB2	-2,495152701	-2,536339282	0,041186581
1555502_at	NPSA	-2,495152701	-2,536339282	0,041186581
1566449_at	LRRC40	-2,495152701	-2,536339282	0,041186581
201445_at	CNN3	-2,495152701	-2,536339282	0,041186581
1558645_at	MIB1	-2,495152701	-2,536339282	0,041186581
239664_at	-	-2,495152701	-2,536339282	0,041186581
237992_at	-	-2,495152701	-2,536339282	0,041186581
1560890_at	LOC100505875	-2,495152701	-2,536339282	0,041186581
1559221_at	LOC100507254	-2,495152701	-2,536339282	0,041186581
237873_s_at	-	-2,495152701	-2,536339282	0,041186581

243781_at	-	-2,495152701	-2,536339282	0,041186581
206835_at	STATH	-2,495152701	-2,536339282	0,041186581
216982_x_at	-	-2,495152701	-2,536339282	0,041186581
1558336_at	-	-1,624955693	-1,665791776	0,040836083
1559810_at	-	-1,624955693	-1,665791776	0,040836083
1570411_at	LOC100133131	-1,624955693	-1,665791776	0,040836083
239902_at	-	-1,624955693	-1,665791776	0,040836083
1563272_at	TBC1D8B	-1,624955693	-1,665791776	0,040836083
1569764_at	-	-1,624955693	-1,665791776	0,040836083
1562829_at	LOC339568	-1,624955693	-1,665791776	0,040836083
1556769_a_at	-	-1,624955693	-1,665791776	0,040836083
220996_s_at	SHCBP1L	-1,624955693	-1,665791776	0,040836083
230286_at	SLC6A11	-1,624955693	-1,665791776	0,040836083
244776_at	-	-1,624955693	-1,665791776	0,040836083
1562300_at	-	-1,624955693	-1,665791776	0,040836083
1562145_at	-	-1,624955693	-1,665791776	0,040836083
1559111_a_at	-	-1,624955693	-1,665791776	0,040836083
1563512_at	NOS1AP	-1,624955693	-1,665791776	0,040836083
1562630_at	-	-1,624955693	-1,665791776	0,040836083
203864_s_at	ACTN2	-1,624955693	-1,665791776	0,040836083
240628_at	-	-1,624955693	-1,665791776	0,040836083
232479_at	FERMT1	-1,624955693	-1,665791776	0,040836083
1559413_at	TCP11L2	-1,624955693	-1,665791776	0,040836083
239981_x_at	-	-1,624955693	-1,665791776	0,040836083
244073_at	-	-1,624955693	-1,665791776	0,040836083
238421_at	RC3H2	-1,624955693	-1,665791776	0,040836083
1552712_a_at	NMNAT2	-1,624955693	-1,665791776	0,040836083
229311_at	GKAP1	-1,624955693	-1,665791776	0,040836083
237789_at	-	-1,624955693	-1,665791776	0,040836083
233424_at	-	-1,624955693	-1,665791776	0,040836083
219602_s_at	PIEZO2	-1,624955693	-1,665791776	0,040836083
220811_at	PRG3	-1,624955693	-1,665791776	0,040836083
205001_s_at	DDX3Y	-1,624955693	-1,665791776	0,040836083
226351_at	NSUN4	1,671397663	1,630734622	0,040663041
229347_at	LOC100505738	0,425036312	0,385582632	0,039453681
227737_at	SRPRB	1,625482993	1,586121802	0,039361191
229970_at	KBTBD7	1,5944803	1,555165465	0,039314835
215082_at	ELOVL5	1,071336699	1,032267596	0,039069102
217118_s_at	KIAA0930	2,739067176	2,700070732	0,038996444
228686_at	FLJ33630	0,735999505	0,697221375	0,03877813
224618_at	PTBP3	3,012642182	2,973964204	0,038677978
205796_at	TCP11L1	1,307201325	1,268569037	0,038632288
1556340_at	MAPK12	1,287793882	1,249194639	0,038599243
208794_s_at	SMARCA4	3,739061547	3,70056079	0,038500756
1553218_a_at	ZNF512	2,983826368	2,945366812	0,038459556
202034_x_at	RB1CC1	2,771643417	2,733194118	0,038449299
40020_at	CELSR3	1,695043175	1,656613669	0,038429505
207974_s_at	SKP1	5,600653734	5,562249857	0,038403877
209658_at	CDC16	3,732463367	3,694322866	0,038140502
1566490_at	-	-1,06282519	-1,100943374	0,038118184

222184_at	-	-1,06282519	-1,100943374	0,038118184
1565882_at	-	-1,06282519	-1,100943374	0,038118184
225301_s_at	MYO5B	-1,06282519	-1,100943374	0,038118184
210757_x_at	DAB2	-1,06282519	-1,100943374	0,038118184
220693_at	CCDC82	-1,06282519	-1,100943374	0,038118184
214729_at	TWISTNB	-1,06282519	-1,100943374	0,038118184
223745_at	C16orf95	-1,06282519	-1,100943374	0,038118184
227279_at	TCEAL3	-1,06282519	-1,100943374	0,038118184
206012_at	LEFTY2	-1,06282519	-1,100943374	0,038118184
220531_at	-	-1,06282519	-1,100943374	0,038118184
243534_at	CC2D2B /// LOC10	-1,06282519	-1,100943374	0,038118184
239341_at	-	-1,06282519	-1,100943374	0,038118184
213040_s_at	NPTXR	-1,06282519	-1,100943374	0,038118184
225168_at	FRMD4A	-1,06282519	-1,100943374	0,038118184
204997_at	GPD1	-1,06282519	-1,100943374	0,038118184
208558_at	OR10H1	-1,06282519	-1,100943374	0,038118184
230277_at	LOC100289187	0,58788177	0,550017041	0,03786473
208846_s_at	VDAC3	5,633477277	5,59567262	0,037804657
241534_at	LOC100505549	-0,551284523	-0,589001171	0,037716648
1555234_a_at	RHOJ	-0,551284523	-0,589001171	0,037716648
218406_x_at	NENF	-0,551284523	-0,589001171	0,037716648
204534_at	VTN	-0,551284523	-0,589001171	0,037716648
216219_at	AQP6	-0,551284523	-0,589001171	0,037716648
200714_x_at	OS9	2,41945542	2,381744287	0,037711133
244014_x_at	-	-1,289805289	-1,32740143	0,037596141
1562259_at	TEX9	-1,289805289	-1,32740143	0,037596141
205554_s_at	DNASE1L3	-1,289805289	-1,32740143	0,037596141
244844_at	-	-1,289805289	-1,32740143	0,037596141
1569555_at	GDA	-1,289805289	-1,32740143	0,037596141
1566937_at	-	-1,289805289	-1,32740143	0,037596141
211118_x_at	ESR2	-1,289805289	-1,32740143	0,037596141
240202_x_at	PFKP	-1,289805289	-1,32740143	0,037596141
229585_at	ADAMTSL1	-1,289805289	-1,32740143	0,037596141
1566498_at	-	-1,289805289	-1,32740143	0,037596141
213426_s_at	CAV2	-1,289805289	-1,32740143	0,037596141
230663_at	-	-1,289805289	-1,32740143	0,037596141
207282_s_at	MYOG	-1,289805289	-1,32740143	0,037596141
231404_at	SPTY2D1-AS1	-1,289805289	-1,32740143	0,037596141
209752_at	REG1A	-1,289805289	-1,32740143	0,037596141
211102_s_at	LILRA2	-1,289805289	-1,32740143	0,037596141
219080_s_at	CTPS2	-1,289805289	-1,32740143	0,037596141
220475_at	SLC28A3	-1,289805289	-1,32740143	0,037596141
228240_at	AGAP1	-1,289805289	-1,32740143	0,037596141
229550_at	UNC79	-1,289805289	-1,32740143	0,037596141
208087_s_at	ZBP1	-1,289805289	-1,32740143	0,037596141
242784_at	-	-1,289805289	-1,32740143	0,037596141
235435_at	AASDH	2,51558601	2,478123977	0,037462032
223151_at	DCUN1D5	4,827919576	4,790491591	0,037427985
204286_s_at	PMAIP1	2,199941239	2,162541631	0,037399608
214205_x_at	GLRX3	2,427650693	2,390281999	0,037368695

220200_s_at	SETD8	1,139057614	1,101780212	0,037277403
219387_at	CCDC88A	2,436165655	2,398910172	0,037255483
201587_s_at	IRAK1	4,568872906	4,531717919	0,037154987
239728_at	-	-0,135065865	-0,172194225	0,03712836
64064_at	GIMAP1-GIMAP5 /	0,451865002	0,414759093	0,037105909
228647_at	LOC100049716	1,160996507	1,123905343	0,037091165
208770_s_at	EIF4EBP2	2,966903803	2,930238085	0,036665718
235339_at	SETDB2	1,246401134	1,209973561	0,036427572
207734_at	LAX1	1,730929079	1,694547353	0,036381726
218320_s_at	NDUFB11	5,590492943	5,554244702	0,03624824
202909_at	EPM2AIP1	3,603313992	3,567108567	0,036205426
226356_at	FAM73B	1,225123479	1,189078962	0,036044517
226923_at	SCFD2	2,530390387	2,49476365	0,035626737
217988_at	CCNB1IP1	4,610466181	4,575073435	0,035392745
210975_x_at	FASTK	1,686984721	1,651676651	0,03530807
200619_at	SF3B2	4,065340446	4,030053209	0,035287237
200966_x_at	ALDOA	7,17116732	7,136383124	0,034784196
217836_s_at	YY1AP1	4,158437147	4,123687269	0,034749878
231222_at	-	0,618277321	0,583607503	0,034669818
236070_at	C2orf81	0,618277321	0,583607503	0,034669818
222276_at	METTL2B	0,167727503	0,133104519	0,034622984
212430_at	RBM38	0,791908897	0,757638486	0,034270411
204535_s_at	REST	0,458905032	0,424693306	0,034211726
1560647_at	TSPYL1	-2,569309811	-2,603471267	0,034161455
233591_at	-	-2,569309811	-2,603471267	0,034161455
1557746_at	-	-2,569309811	-2,603471267	0,034161455
213817_at	IRAK3	-2,569309811	-2,603471267	0,034161455
1562431_x_at	-	-2,569309811	-2,603471267	0,034161455
1553247_a_at	ZNF709	-2,569309811	-2,603471267	0,034161455
1560526_at	MAGI2-IT1	-2,569309811	-2,603471267	0,034161455
1567281_at	-	-2,569309811	-2,603471267	0,034161455
241235_at	-	-2,569309811	-2,603471267	0,034161455
1553333_at	MAB21L3	-2,569309811	-2,603471267	0,034161455
1566637_at	-	-2,569309811	-2,603471267	0,034161455
210292_s_at	PCDH11X /// PCDH	-2,569309811	-2,603471267	0,034161455
214621_at	GYS2	-2,569309811	-2,603471267	0,034161455
1570515_a_at	FILIP1	-2,569309811	-2,603471267	0,034161455
242698_at	AKAP14	-2,569309811	-2,603471267	0,034161455
233170_at	LINC00348	-2,569309811	-2,603471267	0,034161455
235481_at	COMMD10	-2,569309811	-2,603471267	0,034161455
1556954_at	LOC283854	-2,569309811	-2,603471267	0,034161455
238370_x_at	-	-2,569309811	-2,603471267	0,034161455
1560426_at	C12orf55 /// C12o	-2,569309811	-2,603471267	0,034161455
1568935_at	-	-2,569309811	-2,603471267	0,034161455
224007_at	HSFY1 /// HSFY2	-2,569309811	-2,603471267	0,034161455
1558557_at	C16orf62	-2,569309811	-2,603471267	0,034161455
224029_x_at	SCN11A	-2,569309811	-2,603471267	0,034161455
235228_at	CCDC85A	-2,569309811	-2,603471267	0,034161455
232192_at	LOC153811	-0,384193355	-0,418248858	0,034055503
204924_at	TLR2	-0,384193355	-0,418248858	0,034055503

239117_at	-	-0,384193355	-0,418248858	0,034055503
207984_s_at	MPP2	-0,384193355	-0,418248858	0,034055503
228634_s_at	CSDA	-0,384193355	-0,418248858	0,034055503
233003_at	-	-0,384193355	-0,418248858	0,034055503
231823_s_at	SH3PXD2B	-0,384193355	-0,418248858	0,034055503
207748_at	-	-0,384193355	-0,418248858	0,034055503
233061_at	FITM2	-0,384193355	-0,418248858	0,034055503
220918_at	RUNX1-IT1	-0,384193355	-0,418248858	0,034055503
212924_s_at	LSM4	-0,384193355	-0,418248858	0,034055503
235557_at	GPAT2	-0,384193355	-0,418248858	0,034055503
208907_s_at	MRPS18B	4,082216372	4,04859535	0,033621023
222703_s_at	YRDC	3,200057698	3,166549502	0,033508196
38487_at	STAB1	-0,50967422	-0,543124488	0,033450268
234696_at	-	0,319287178	0,286279868	0,03300731
223456_s_at	TCHP	0,319287178	0,286279868	0,03300731
210595_at	ZNF235	-0,856259172	-0,889222211	0,032963039
1562983_at	LOC441025	-0,856259172	-0,889222211	0,032963039
205948_at	PTPRT	-0,856259172	-0,889222211	0,032963039
1569419_at	-	-0,856259172	-0,889222211	0,032963039
1569446_x_at	LOC100287576 ///	-0,856259172	-0,889222211	0,032963039
236468_at	TMEM132B	-0,856259172	-0,889222211	0,032963039
220901_at	GPR157	-0,856259172	-0,889222211	0,032963039
213075_at	OLFML2A	-0,856259172	-0,889222211	0,032963039
208216_at	DLX4	-0,856259172	-0,889222211	0,032963039
244862_at	-	-0,856259172	-0,889222211	0,032963039
244054_at	-	-0,856259172	-0,889222211	0,032963039
208627_s_at	YBX1	7,140315226	7,107457537	0,032857689
209849_s_at	RAD51C	4,323104796	4,290339887	0,03276491
225875_s_at	NIPAL3	2,16969527	2,137342905	0,032352365
229007_at	LOC283788	0,847529938	0,815200271	0,032329667
236671_at	-	0,847529938	0,815200271	0,032329667
205752_s_at	GSTM5	0,045517965	0,013190398	0,032327567
210396_s_at	BOLA2 /// LOC440	4,765988171	4,733678108	0,032310063
217956_s_at	ENOPH1	5,174373083	5,142140618	0,032232466
202530_at	MAPK14	3,81981316	3,787622346	0,032190814
219151_s_at	RABL2A /// RABL2I	1,440265662	1,408220608	0,032045054
221504_s_at	ATP6V1H	3,621852128	3,589866014	0,031986114
1564469_at	LMOD3	-2,774503732	-2,8064286	0,031924867
220744_s_at	IFT122	1,960819106	1,929092307	0,0317268
236814_at	MDM4	2,943764123	2,912181979	0,031582145
200015_s_at	37500	5,781865051	5,750403014	0,031462037
208834_x_at	RPL23A /// SNORD	8,732413437	8,701057131	0,031356307
230777_s_at	PRDM15	1,578719607	1,547376668	0,031342939
227813_at	THAP6	1,5944803	1,563256447	0,031223853
201963_at	ACSL1	3,4540469	3,422991983	0,031054917
218841_at	ASB8	2,685337919	2,654387125	0,030950794
232914_s_at	SYTL2	1,760255626	1,72935563	0,030899996
203664_s_at	POLR2D	2,50022283	2,469496389	0,030726441
1560760_s_at	-	-1,537851782	-1,568385657	0,030533874
214827_at	PARD6B	-1,537851782	-1,568385657	0,030533874

1570503_at	-	-1,537851782	-1,568385657	0,030533874
241231_at	-	-1,537851782	-1,568385657	0,030533874
224019_at	SCD5	-1,537851782	-1,568385657	0,030533874
222123_s_at	HIF3A	-1,537851782	-1,568385657	0,030533874
1562275_at	-	-1,537851782	-1,568385657	0,030533874
244099_at	CACNG2	-1,537851782	-1,568385657	0,030533874
1561224_at	-	-1,537851782	-1,568385657	0,030533874
1569518_at	-	-1,537851782	-1,568385657	0,030533874
237820_at	-	-1,537851782	-1,568385657	0,030533874
1569723_a_at	SPIRE2	-1,537851782	-1,568385657	0,030533874
217004_s_at	MCF2	-1,537851782	-1,568385657	0,030533874
207017_at	RAB27B	-1,537851782	-1,568385657	0,030533874
1557244_a_at	-	-1,537851782	-1,568385657	0,030533874
1556826_s_at	C1orf187	-1,537851782	-1,568385657	0,030533874
206228_at	PAX2	-1,537851782	-1,568385657	0,030533874
1557162_at	C16orf92	-1,537851782	-1,568385657	0,030533874
231333_at	LOC100505515	-1,537851782	-1,568385657	0,030533874
1559579_at	LOC283688	-1,537851782	-1,568385657	0,030533874
230572_at	WWC2-AS2	-1,537851782	-1,568385657	0,030533874
206384_at	CACNG3	-1,537851782	-1,568385657	0,030533874
1557731_at	LOC400620	-1,537851782	-1,568385657	0,030533874
214460_at	LSAMP	-1,537851782	-1,568385657	0,030533874
240360_at	BRD4	-1,537851782	-1,568385657	0,030533874
202823_at	TCEB1	2,843031657	2,812515887	0,030515771
1567985_at	-	0,900065578	0,869824768	0,03024081
229685_at	LOC100134937	-0,230432956	-0,260526297	0,030093341
1564155_x_at	-	-0,230432956	-0,260526297	0,030093341
242743_at	IL4R	-0,230432956	-0,260526297	0,030093341
239048_at	-	-0,230432956	-0,260526297	0,030093341
232240_at	DNHD1	-0,230432956	-0,260526297	0,030093341
225215_s_at	MTRF1L	2,719381633	2,689594859	0,029786774
202344_at	HSF1	1,745502609	1,715863686	0,029638923
202093_s_at	PAF1	1,88491011	1,855279162	0,029630949
1568796_at	CCDC157	0,926325262	0,896834102	0,02949116
238771_at	-	0,20740896	0,178191865	0,029217095
209941_at	RIPK1	0,97663406	0,947673886	0,028960174
201713_s_at	RANBP2	4,025386557	3,996494541	0,028892016
209077_at	TXN2	3,603313992	3,5744736	0,028840392
220956_s_at	EGLN2 /// RAB4B-I	1,871521385	1,842852096	0,028669289
219802_at	PYROXD1	2,452674045	2,424202743	0,028471302
1320_at	PTPN21	-1,70474736	-1,733092925	0,028345565
223513_at	CENPJ	3,102023286	3,074131603	0,027891683
208174_x_at	ZRSR2	1,857989965	1,830129603	0,027860362
206186_at	MPP3	-0,66934516	-0,697182596	0,027837437
218835_at	SFTPA1 /// SFTPA2	-0,66934516	-0,697182596	0,027837437
240497_at	-	-0,66934516	-0,697182596	0,027837437
236979_at	BCL2L15	-0,66934516	-0,697182596	0,027837437
217686_at	PTPN1	-0,66934516	-0,697182596	0,027837437
1557433_at	-	-0,088974936	-0,116767475	0,027792539
223536_at	PSD2	-0,088974936	-0,116767475	0,027792539

1557021_s_at	LOC100507250	-0,088974936	-0,116767475	0,027792539
215031_x_at	RNF126	2,159837409	2,132145748	0,027691661
221020_s_at	SLC25A32	4,221194173	4,193538582	0,027655591
209038_s_at	EHD1	0,677745787	0,65030602	0,027439767
241114_s_at	-	0,677745787	0,65030602	0,027439767
1552474_a_at	GAMT	2,16969527	2,142313983	0,027381287
208740_at	SAP18	2,791258167	2,76399677	0,027261396
231094_s_at	MTHFD1L	2,053550345	2,026957145	0,0265932
201620_at	MBTPS1	2,636121902	2,609615156	0,026506745
225106_s_at	OGFOD1	2,932161212	2,906008589	0,026152623
212638_s_at	WWP1	3,362258367	3,336180482	0,026077885
204632_at	RPS6KA4	1,246401134	1,220470658	0,025930476
1554903_at	FRMD8	1,20396005	1,178100917	0,025859133
209889_at	SEC31B	1,20396005	1,178100917	0,025859133
208882_s_at	UBR5	3,252865595	3,227213858	0,025651738
225740_x_at	MDM4	2,507817181	2,482273566	0,025543616
213947_s_at	NUP210	3,639446351	3,614020495	0,025425856
242806_at	-	-0,49284695	-0,518232988	0,025386038
206089_at	NELL1	-0,49284695	-0,518232988	0,025386038
229680_at	TACO1	-0,49284695	-0,518232988	0,025386038
220464_at	MCF2L	-0,49284695	-0,518232988	0,025386038
1553019_at	TMC2	-0,49284695	-0,518232988	0,025386038
218756_s_at	DHRS11	-0,49284695	-0,518232988	0,025386038
225204_at	PPTC7	3,57383031	3,548671762	0,025158548
220990_s_at	MIR21 /// VMP1	4,840637435	4,815598205	0,025039229
243030_at	-	0,39105295	0,366031214	0,025021737
201634_s_at	CYB5B	4,00240646	3,977561363	0,024845097
218750_at	TAF1D	1,65554524	1,630734622	0,024810618
202663_at	WIPF1	2,902957057	2,878165848	0,02479121
228002_at	GTPBP4 /// IDI2	1,476175815	1,451507543	0,024668272
235195_at	FBXW2	0,08619576	0,061773582	0,024422178
1557174_a_at	-	0,735999505	0,711934445	0,02406506
224083_s_at	PRO1596	-2,641232718	-2,665156778	0,02392406
237850_at	-	-2,641232718	-2,665156778	0,02392406
1552372_at	C4orf33	-2,641232718	-2,665156778	0,02392406
1564134_at	-	-2,641232718	-2,665156778	0,02392406
1556598_at	ARPP21 /// LOC10	-2,641232718	-2,665156778	0,02392406
1562997_a_at	-	-2,641232718	-2,665156778	0,02392406
206977_at	PTH	-2,641232718	-2,665156778	0,02392406
228772_at	HNMT	-2,641232718	-2,665156778	0,02392406
1570250_at	LPPR1	-2,641232718	-2,665156778	0,02392406
241607_at	LOC730102	-2,641232718	-2,665156778	0,02392406
1565628_at	-	-2,641232718	-2,665156778	0,02392406
209720_s_at	SERPINB3	-2,641232718	-2,665156778	0,02392406
202533_s_at	DHFR	2,582572508	2,558652146	0,023920362
232231_at	RUNX2	4,16769828	4,143962533	0,023735746
204573_at	CROT	1,578719607	1,555165465	0,023554141
224448_s_at	MNF1	4,335356502	4,311836632	0,02351987
211417_x_at	GGT1 /// GGT2 ///	2,705643918	2,682180306	0,023463612
1555736_a_at	AGTRAP	1,5944803	1,571112806	0,023367494

223312_at	PRADC1	2,492739983	2,469496389	0,023243594
227107_at	-	2,537731447	2,514786608	0,022944839
1556062_at	RPP30	-2,163157732	-2,186086603	0,022928871
1569609_at	-	-2,163157732	-2,186086603	0,022928871
1552818_a_at	BRS3	-2,163157732	-2,186086603	0,022928871
240907_at	-	-2,163157732	-2,186086603	0,022928871
1561938_at	-	-2,163157732	-2,186086603	0,022928871
228368_at	ARHGAP20	-2,163157732	-2,186086603	0,022928871
239153_at	HOTAIR	-2,163157732	-2,186086603	0,022928871
1557448_a_at	-	-2,163157732	-2,186086603	0,022928871
223794_at	ARMC4	-2,163157732	-2,186086603	0,022928871
231799_at	TANC2	-2,163157732	-2,186086603	0,022928871
243779_at	GALNT13	-2,163157732	-2,186086603	0,022928871
1559283_a_at	CNPY1	-2,163157732	-2,186086603	0,022928871
236654_s_at	-	-2,163157732	-2,186086603	0,022928871
1558881_at	LOC145820	-2,163157732	-2,186086603	0,022928871
236111_at	-	-2,163157732	-2,186086603	0,022928871
1566511_at	FBXO9	-2,163157732	-2,186086603	0,022928871
221451_s_at	OR2W1	-2,163157732	-2,186086603	0,022928871
1569770_x_at	-	-2,163157732	-2,186086603	0,022928871
1558852_at	-	-2,163157732	-2,186086603	0,022928871
1570298_at	-	-2,163157732	-2,186086603	0,022928871
1560646_at	METTL21CP1	-2,163157732	-2,186086603	0,022928871
240556_at	DCN	-2,163157732	-2,186086603	0,022928871
244436_at	-	-2,163157732	-2,186086603	0,022928871
1554473_at	SRGAP1	-2,163157732	-2,186086603	0,022928871
241765_at	CPM	-2,163157732	-2,186086603	0,022928871
1562683_a_at	LOC285547	-2,163157732	-2,186086603	0,022928871
204818_at	HSD17B2	-2,163157732	-2,186086603	0,022928871
203902_at	HEPH	-2,163157732	-2,186086603	0,022928871
238740_at	AARSD1 /// PTGES	-2,163157732	-2,186086603	0,022928871
1557214_at	LOC100506107	-2,163157732	-2,186086603	0,022928871
244402_at	-	-2,163157732	-2,186086603	0,022928871
240667_at	SERP2	-2,163157732	-2,186086603	0,022928871
1563950_at	-	-2,163157732	-2,186086603	0,022928871
237780_at	-	-2,163157732	-2,186086603	0,022928871
206995_x_at	SCARF1	-2,163157732	-2,186086603	0,022928871
1556267_at	C12orf28	-2,163157732	-2,186086603	0,022928871
243879_at	-	-2,163157732	-2,186086603	0,022928871
220115_s_at	CDH10	-2,163157732	-2,186086603	0,022928871
226979_at	MAP3K2	3,237994092	3,215288307	0,022705784
241124_at	-	-1,982324124	-2,005029581	0,022705457
221933_at	NLGN4X	-1,982324124	-2,005029581	0,022705457
1554355_a_at	SIAE	-1,982324124	-2,005029581	0,022705457
1562470_at	TCEANC2	-1,982324124	-2,005029581	0,022705457
1560692_at	LOC285878	-1,982324124	-2,005029581	0,022705457
232544_at	-	-1,982324124	-2,005029581	0,022705457
1563693_at	-	-1,982324124	-2,005029581	0,022705457
239065_at	DNAJC22	-1,982324124	-2,005029581	0,022705457
1559268_at	ADAM23	-1,982324124	-2,005029581	0,022705457

204591_at	CHL1	-1,982324124	-2,005029581	0,022705457
1561670_at	-	-1,982324124	-2,005029581	0,022705457
243592_at	REV1	-1,982324124	-2,005029581	0,022705457
1557798_at	CNPY2	-1,982324124	-2,005029581	0,022705457
1564121_at	-	-1,982324124	-2,005029581	0,022705457
241175_at	-	-1,982324124	-2,005029581	0,022705457
241856_at	IMPG2	-1,982324124	-2,005029581	0,022705457
1561519_at	-	-1,982324124	-2,005029581	0,022705457
240719_at	LOC100505536	-1,982324124	-2,005029581	0,022705457
1559889_at	-	-1,982324124	-2,005029581	0,022705457
224103_at	LOC100132661	-1,982324124	-2,005029581	0,022705457
215443_at	TSHR	-1,982324124	-2,005029581	0,022705457
234025_at	-	-1,982324124	-2,005029581	0,022705457
237982_at	-	-1,982324124	-2,005029581	0,022705457
241575_at	-	-1,982324124	-2,005029581	0,022705457
1554594_at	ARHGAP27	-1,982324124	-2,005029581	0,022705457
244143_at	-	-1,982324124	-2,005029581	0,022705457
1560554_a_at	C1orf145	-1,982324124	-2,005029581	0,022705457
1562320_at	NAV2-AS5	-1,982324124	-2,005029581	0,022705457
207164_s_at	ZNF238	-1,982324124	-2,005029581	0,022705457
240158_at	-	-1,982324124	-2,005029581	0,022705457
1555163_at	-	-1,982324124	-2,005029581	0,022705457
1561062_a_at	-	-1,982324124	-2,005029581	0,022705457
227760_at	IGFBPL1	-1,982324124	-2,005029581	0,022705457
244322_at	LOC646329	-1,982324124	-2,005029581	0,022705457
244091_at	-	-1,982324124	-2,005029581	0,022705457
217304_at	SHMT1	-1,982324124	-2,005029581	0,022705457
211790_s_at	MLL2	-1,982324124	-2,005029581	0,022705457
1563018_at	TBX5	-1,982324124	-2,005029581	0,022705457
237935_at	-	-1,982324124	-2,005029581	0,022705457
210632_s_at	SGCA	-1,982324124	-2,005029581	0,022705457
236475_at	MICAL2	-1,982324124	-2,005029581	0,022705457
239457_at	ATP8B3	-1,982324124	-2,005029581	0,022705457
1553887_at	C19orf75	-1,982324124	-2,005029581	0,022705457
209488_s_at	RBPMS	-1,982324124	-2,005029581	0,022705457
213027_at	TROVE2	3,156891301	3,134198147	0,022693154
223264_at	MESDC1	2,365121417	2,34250725	0,022614167
244845_at	-	-2,074223907	-2,09678811	0,022564203
216529_at	-	-2,074223907	-2,09678811	0,022564203
231489_x_at	-	-2,074223907	-2,09678811	0,022564203
234155_at	-	-2,074223907	-2,09678811	0,022564203
236843_at	NOX4	-2,074223907	-2,09678811	0,022564203
216719_s_at	-	-2,074223907	-2,09678811	0,022564203
1556832_at	-	-2,074223907	-2,09678811	0,022564203
232250_at	KIAA1257	-2,074223907	-2,09678811	0,022564203
237252_at	THBD	-2,074223907	-2,09678811	0,022564203
1560522_at	LOC201477	-2,074223907	-2,09678811	0,022564203
1568898_at	-	-2,074223907	-2,09678811	0,022564203
239214_at	LOC100130458	-2,074223907	-2,09678811	0,022564203
242355_at	-	-2,074223907	-2,09678811	0,022564203

237692_at	-	-2,074223907	-2,09678811	0,022564203
208531_at	SERPINA2	-2,074223907	-2,09678811	0,022564203
211556_at	MAPRE2	-2,074223907	-2,09678811	0,022564203
1556927_at	KCNK10	-2,074223907	-2,09678811	0,022564203
1565666_s_at	MUC6	-2,074223907	-2,09678811	0,022564203
234398_at	-	-2,074223907	-2,09678811	0,022564203
233915_at	KIAA0825	-2,074223907	-2,09678811	0,022564203
1560240_at	-	-2,074223907	-2,09678811	0,022564203
1561253_at	-	-2,074223907	-2,09678811	0,022564203
225987_at	STEAP4	-2,074223907	-2,09678811	0,022564203
217901_at	DSG2	-2,074223907	-2,09678811	0,022564203
241834_at	IPW /// LOC10050	-2,074223907	-2,09678811	0,022564203
1560932_at	FLJ31356	-2,074223907	-2,09678811	0,022564203
234614_at	FLI1	-2,074223907	-2,09678811	0,022564203
215352_at	GIMAP1-GIMAP5 /	-2,074223907	-2,09678811	0,022564203
237444_at	-	-2,074223907	-2,09678811	0,022564203
233257_at	-	-2,074223907	-2,09678811	0,022564203
1568601_at	GAD2	-2,074223907	-2,09678811	0,022564203
1566362_at	DNTT	-2,074223907	-2,09678811	0,022564203
1553537_at	KRT73	-2,074223907	-2,09678811	0,022564203
231721_at	JAM3	-2,074223907	-2,09678811	0,022564203
243357_at	NEGR1	-2,074223907	-2,09678811	0,022564203
234424_at	-	-2,074223907	-2,09678811	0,022564203
1559692_at	-	-2,074223907	-2,09678811	0,022564203
1553514_a_at	VNN3	-2,074223907	-2,09678811	0,022564203
1553352_x_at	ERVW-1	-2,074223907	-2,09678811	0,022564203
232514_at	KIF27	-2,074223907	-2,09678811	0,022564203
211184_s_at	USH1C	-2,074223907	-2,09678811	0,022564203
216465_at	-	-2,074223907	-2,09678811	0,022564203
244486_at	PINK1-AS	-0,332405896	-0,354781582	0,022375686
1568978_s_at	C11orf21	-0,332405896	-0,354781582	0,022375686
235393_at	-	-0,332405896	-0,354781582	0,022375686
222094_at	SLX1A /// SLX1A-SI	-0,332405896	-0,354781582	0,022375686
201424_s_at	CUL4A	3,389999385	3,367769667	0,022229717
213678_at	TMEM151B	-1,212652659	-1,234835326	0,022182667
1558348_at	-	-1,212652659	-1,234835326	0,022182667
228657_at	KIF1B	-1,212652659	-1,234835326	0,022182667
208588_at	FKSG2	-1,212652659	-1,234835326	0,022182667
1557841_at	-	-1,212652659	-1,234835326	0,022182667
229693_at	TMEM220	-1,212652659	-1,234835326	0,022182667
202403_s_at	COL1A2	-1,212652659	-1,234835326	0,022182667
210315_at	SYN2	-1,212652659	-1,234835326	0,022182667
1553504_at	MRGPRX4	-1,212652659	-1,234835326	0,022182667
1553881_at	MGC16142	-1,212652659	-1,234835326	0,022182667
241625_at	-	-1,212652659	-1,234835326	0,022182667
217687_at	ADCY2	-1,212652659	-1,234835326	0,022182667
1558007_s_at	LOC100506655	-1,212652659	-1,234835326	0,022182667
217277_at	-	-1,212652659	-1,234835326	0,022182667
220204_s_at	BMP8A	-1,212652659	-1,234835326	0,022182667
244352_at	CD84	-1,212652659	-1,234835326	0,022182667

221129_at	FAM215A	-1,212652659	-1,234835326	0,022182667
224010_at	ANAPC11	-1,212652659	-1,234835326	0,022182667
240674_at	JARID2	-1,212652659	-1,234835326	0,022182667
244305_at	GGN	-1,212652659	-1,234835326	0,022182667
233100_at	EEPD1	-1,212652659	-1,234835326	0,022182667
220802_at	KCNH4	-1,212652659	-1,234835326	0,022182667
211631_x_at	B4GALT1	-1,212652659	-1,234835326	0,022182667
219712_s_at	SHPK /// TRPV1	-1,212652659	-1,234835326	0,022182667
235411_at	PGBD1	-1,212652659	-1,234835326	0,022182667
219756_s_at	POF1B	-1,212652659	-1,234835326	0,022182667
203371_s_at	NDUFB3	4,64066268	4,618481456	0,022181225
208825_x_at	RPL23A /// SNORD	8,702839372	8,680687575	0,022151797
225932_s_at	HNRNPA2B1	5,345228075	5,323084942	0,022143133
214760_at	ZNF337	1,730929079	1,709047448	0,021881631
200077_s_at	OAZ1	7,721741392	7,700115274	0,021626118
215022_x_at	ZNF33B	1,544940605	1,523429058	0,021511547
230837_at	LOC100132057	-0,994186487	-1,015398016	0,021211529
221415_s_at	GJA9 /// GJA9-MY(	-0,994186487	-1,015398016	0,021211529
236568_at	CC2D1B	-0,994186487	-1,015398016	0,021211529
229965_at	-	-0,994186487	-1,015398016	0,021211529
220508_at	CCT8L2	-0,994186487	-1,015398016	0,021211529
237443_at	-	-0,994186487	-1,015398016	0,021211529
220826_at	TCP10L	-0,994186487	-1,015398016	0,021211529
207055_at	GPR37L1	-0,994186487	-1,015398016	0,021211529
210832_x_at	PTGER3	-0,994186487	-1,015398016	0,021211529
1561369_at	-	-0,994186487	-1,015398016	0,021211529
217792_at	SNX5	3,855094415	3,834044437	0,021049978
225533_at	PHF19	2,978070706	2,957089023	0,020981683
221693_s_at	MRPS18A	3,305120269	3,284313623	0,020806646
1558673_s_at	ZNF77	-0,180990326	-0,201789721	0,020799395
242852_at	LOC285147	-0,180990326	-0,201789721	0,020799395
203146_s_at	GABBR1	-0,180990326	-0,201789721	0,020799395
1567622_at	ABLIM2	-0,180990326	-0,201789721	0,020799395
231493_at	-	-0,180990326	-0,201789721	0,020799395
225248_at	SPPL2B	-0,180990326	-0,201789721	0,020799395
222817_at	HSD3B7	-0,180990326	-0,201789721	0,020799395
202217_at	C21orf33	4,424844786	4,404237854	0,020606932
209302_at	POLR2H	4,603614396	4,583098448	0,020515949
1563511_at	DKFZp451B082	-0,791294935	-0,811769547	0,020474612
217239_x_at	LOC100508797	-0,791294935	-0,811769547	0,020474612
243911_at	-	-0,791294935	-0,811769547	0,020474612
232446_at	MGC45800	-0,791294935	-0,811769547	0,020474612
1558174_at	FAM43A	-0,791294935	-0,811769547	0,020474612
1553166_at	CDH24	-0,791294935	-0,811769547	0,020474612
217023_x_at	TPSAB1 /// TPSB2	-0,791294935	-0,811769547	0,020474612
205316_at	SLC15A2	-0,791294935	-0,811769547	0,020474612
219215_s_at	SLC39A4	2,484824532	2,465016194	0,019808337
205252_at	ZNF174	0,58788177	0,568155442	0,019726328
229138_at	PARP11	1,960819106	1,941115499	0,019703607
200993_at	IPO7	5,087996835	5,068314985	0,019681849

1558142_at	TNRC6B	2,705643918	2,686001625	0,019642293
230296_at	C16orf52	2,705643918	2,686001625	0,019642293
224625_x_at	C15orf63 /// MIR1	6,401154699	6,38159864	0,019556059
223208_at	KCTD10	2,085817135	2,066329863	0,019487271
212119_at	RHOQ	2,860630824	2,841176366	0,019454458
239483_at	FLJ37035	-0,043925712	-0,063377083	0,019451371
1556560_a_at	LOC100128071	-0,043925712	-0,063377083	0,019451371
232737_s_at	ENPP3	-2,250580859	-2,27001641	0,019435551
210390_s_at	CCL14 /// CCL14-C	-2,250580859	-2,27001641	0,019435551
1570228_at	CFHR3	-2,250580859	-2,27001641	0,019435551
1561857_at	-	-2,250580859	-2,27001641	0,019435551
1560182_at	-	-2,250580859	-2,27001641	0,019435551
234247_at	-	-2,250580859	-2,27001641	0,019435551
1565602_at	-	-2,250580859	-2,27001641	0,019435551
1566869_at	-	-2,250580859	-2,27001641	0,019435551
244380_at	-	-2,250580859	-2,27001641	0,019435551
233910_at	TMEFF2	-2,250580859	-2,27001641	0,019435551
234314_at	RALGAPA2	-2,250580859	-2,27001641	0,019435551
213993_at	SPON1	-2,250580859	-2,27001641	0,019435551
1569942_at	-	-2,250580859	-2,27001641	0,019435551
241902_at	MKX	-2,250580859	-2,27001641	0,019435551
214547_at	ADCY10	-2,250580859	-2,27001641	0,019435551
1561002_at	-	-2,250580859	-2,27001641	0,019435551
242447_at	C3orf70	-2,250580859	-2,27001641	0,019435551
1555250_a_at	CPEB3	-2,250580859	-2,27001641	0,019435551
1556630_at	CASC2	-2,250580859	-2,27001641	0,019435551
214932_at	KIDINS220	-2,250580859	-2,27001641	0,019435551
234064_at	-	-2,250580859	-2,27001641	0,019435551
243968_x_at	FCRL1	-2,250580859	-2,27001641	0,019435551
1555719_a_at	C3orf15 /// LOC10	-2,250580859	-2,27001641	0,019435551
231381_at	ESRG	-2,250580859	-2,27001641	0,019435551
233948_at	TBX5-AS1	-2,250580859	-2,27001641	0,019435551
1563219_at	-	-2,250580859	-2,27001641	0,019435551
237358_at	-	-2,250580859	-2,27001641	0,019435551
1566139_at	HOPX	-2,250580859	-2,27001641	0,019435551
211844_s_at	NRP2	-2,250580859	-2,27001641	0,019435551
237183_at	GALNT5	-2,250580859	-2,27001641	0,019435551
1560499_at	C14orf64	-2,250580859	-2,27001641	0,019435551
228218_at	LSAMP	-2,250580859	-2,27001641	0,019435551
232204_at	EBF1	-2,250580859	-2,27001641	0,019435551
216929_x_at	ABO	-2,250580859	-2,27001641	0,019435551
242722_at	LMO7	1,510970097	1,491644074	0,019326024
236133_x_at	ZNF254	-1,452713826	-1,471833228	0,019119402
242302_at	PDS5B	-1,452713826	-1,471833228	0,019119402
221468_at	XCR1	-1,452713826	-1,471833228	0,019119402
220156_at	EFCAB1	-1,452713826	-1,471833228	0,019119402
231923_at	TMEM150C	-1,452713826	-1,471833228	0,019119402
224256_at	LOC100129449	-1,452713826	-1,471833228	0,019119402
225960_at	ZNRF1	-1,452713826	-1,471833228	0,019119402
1553031_at	GPR115	-1,452713826	-1,471833228	0,019119402

1562691_at	-	-1,452713826	-1,471833228	0,019119402
231243_s_at	BHLHE41	-1,452713826	-1,471833228	0,019119402
207317_s_at	CASQ2	-1,452713826	-1,471833228	0,019119402
207553_at	OPRK1	-1,452713826	-1,471833228	0,019119402
211400_at	FGFR2	-1,452713826	-1,471833228	0,019119402
1552559_a_at	CDK15	-1,452713826	-1,471833228	0,019119402
242105_at	CCNE1	-1,452713826	-1,471833228	0,019119402
233452_at	-	-1,452713826	-1,471833228	0,019119402
242986_at	NAV1	-1,452713826	-1,471833228	0,019119402
215547_at	TSC22D2	-1,452713826	-1,471833228	0,019119402
1566457_at	-	-1,452713826	-1,471833228	0,019119402
240021_at	-	-1,452713826	-1,471833228	0,019119402
235871_at	LIPH	-1,452713826	-1,471833228	0,019119402
206866_at	CDH4	-1,452713826	-1,471833228	0,019119402
217005_at	LDLR	-1,452713826	-1,471833228	0,019119402
205465_x_at	HS3ST1	-1,452713826	-1,471833228	0,019119402
1566363_at	DNTT	-1,452713826	-1,471833228	0,019119402
226082_s_at	SCAF4	3,336134047	3,317257552	0,018876495
228108_at	PPM1L	1,307201325	1,28853674	0,018664585
209534_x_at	AKAP13	1,307201325	1,28853674	0,018664585
201748_s_at	SAFB	2,732422196	2,713775735	0,018646461
228089_x_at	TMEM179B	3,200057698	3,181622175	0,018435523
209460_at	ABAT	0,127732831	0,109458907	0,018273924
214694_at	MPRIP	0,127732831	0,109458907	0,018273924
236092_at	-	0,127732831	0,109458907	0,018273924
220606_s_at	C17orf48	2,16969527	2,151985997	0,017709273
1562619_at	NME9	-1,893506789	-1,911206569	0,01769978
1567575_at	-	-1,893506789	-1,911206569	0,01769978
221169_s_at	HRH4	-1,893506789	-1,911206569	0,01769978
236158_at	-	-1,893506789	-1,911206569	0,01769978
220415_at	FPGT-TNNI3K /// T	-1,893506789	-1,911206569	0,01769978
207302_at	SGCG	-1,893506789	-1,911206569	0,01769978
237468_at	-	-1,893506789	-1,911206569	0,01769978
1559053_at	-	-1,893506789	-1,911206569	0,01769978
240488_at	-	-1,893506789	-1,911206569	0,01769978
1565732_at	-	-1,893506789	-1,911206569	0,01769978
209109_s_at	TSPAN6	-1,893506789	-1,911206569	0,01769978
239080_at	-	-1,893506789	-1,911206569	0,01769978
1562703_at	LOC157381	-1,893506789	-1,911206569	0,01769978
236051_at	-	-1,893506789	-1,911206569	0,01769978
1561473_at	-	-1,893506789	-1,911206569	0,01769978
220873_at	-	-1,893506789	-1,911206569	0,01769978
1556244_s_at	LOC375196	-1,893506789	-1,911206569	0,01769978
242620_at	-	-1,893506789	-1,911206569	0,01769978
1564744_at	-	-1,893506789	-1,911206569	0,01769978
207452_s_at	CNTN5	-1,893506789	-1,911206569	0,01769978
210873_x_at	APOBEC3A	-1,893506789	-1,911206569	0,01769978
1564203_at	LOC147004	-1,893506789	-1,911206569	0,01769978
217242_at	ZNF154	-1,893506789	-1,911206569	0,01769978
216884_at	PTPN12	-1,893506789	-1,911206569	0,01769978

237693_at	-	-1,893506789	-1,911206569	0,01769978
210181_s_at	CABP1	-1,893506789	-1,911206569	0,01769978
237871_x_at	-	-1,893506789	-1,911206569	0,01769978
233048_at	-	-1,893506789	-1,911206569	0,01769978
1565621_at	-	-1,893506789	-1,911206569	0,01769978
209976_s_at	CYP2E1	-1,893506789	-1,911206569	0,01769978
1564561_at	-	-1,893506789	-1,911206569	0,01769978
208134_x_at	PSG2	-1,893506789	-1,911206569	0,01769978
237590_at	-	-1,893506789	-1,911206569	0,01769978
203757_s_at	CEACAM6	-1,893506789	-1,911206569	0,01769978
1566880_at	-	-1,893506789	-1,911206569	0,01769978
1558574_at	LOC100508631	-1,893506789	-1,911206569	0,01769978
210810_s_at	SLC6A5	-1,893506789	-1,911206569	0,01769978
232634_at	DKFZp566F0947	-1,893506789	-1,911206569	0,01769978
1553074_at	ASB11	-1,893506789	-1,911206569	0,01769978
1558475_at	-	-1,893506789	-1,911206569	0,01769978
1569780_at	-	-1,893506789	-1,911206569	0,01769978
1570650_at	CCBL1	-1,893506789	-1,911206569	0,01769978
237074_at	LOC100506030	-1,893506789	-1,911206569	0,01769978
219670_at	BEND5	-1,893506789	-1,911206569	0,01769978
213924_at	GNAL	-1,893506789	-1,911206569	0,01769978
217764_s_at	RAB31	-1,893506789	-1,911206569	0,01769978
227333_at	-	0,873789652	0,85655369	0,017235962
209838_at	COPS2	0,873789652	0,85655369	0,017235962
217959_s_at	MIR3656 /// TRAP	4,703587657	4,686498043	0,017089614
217025_s_at	DBN1	0,282358733	0,265475485	0,016883248
242748_at	SREBF2	1,000459215	0,98381571	0,016643505
1557276_at	LOC100507584	0,97663406	0,960042218	0,016591842
208459_s_at	XPO7	2,295108393	2,278572464	0,01653593
217740_x_at	RPL7A /// SNORD2	8,52083268	8,504383088	0,016449592
209281_s_at	ATP2B1	2,523100136	2,506994817	0,01610532
225070_at	NUS1	3,273854609	3,257790126	0,016064483
210201_x_at	BIN1	1,071336699	1,055317091	0,016019607
217948_at	FAM127B	0,95222556	0,936232181	0,015993379
232635_at	CEP128	0,95222556	0,936232181	0,015993379
241411_at	-	0,458905032	0,443143223	0,015761809
227205_at	TAF1	2,739067176	2,723341187	0,015725988
224644_at	FGD5-AS1	4,37922506	4,363697406	0,015527654
229436_x_at	BRCC3	2,872644433	2,857256661	0,015387772
217329_x_at	-	3,948420174	3,933228921	0,015191253
211801_x_at	MFN1	1,139057614	1,123905343	0,015152272
218137_s_at	SMAP1	4,221194173	4,206178	0,015016172
1565563_at	-	-2,708365344	-2,723114724	0,01474938
237007_at	-	-2,708365344	-2,723114724	0,01474938
234833_at	-	-2,708365344	-2,723114724	0,01474938
239672_at	-	-2,708365344	-2,723114724	0,01474938
241557_x_at	-	-2,708365344	-2,723114724	0,01474938
241088_at	-	-2,708365344	-2,723114724	0,01474938
1568287_at	HMGA2	-2,708365344	-2,723114724	0,01474938
1553075_a_at	OFCC1	-2,708365344	-2,723114724	0,01474938

1559506_x_at	MIR137HG /// MIF	-2,708365344	-2,723114724	0,01474938
231432_at	-	-2,708365344	-2,723114724	0,01474938
222833_at	LPCAT2	-2,708365344	-2,723114724	0,01474938
236739_at	LOC150622	-2,708365344	-2,723114724	0,01474938
211917_s_at	PRLR	-2,708365344	-2,723114724	0,01474938
1563787_a_at	CAGE1	-2,708365344	-2,723114724	0,01474938
1560999_a_at	-	-2,708365344	-2,723114724	0,01474938
214051_at	TMSB15A /// TMSI	1,116462765	1,101780212	0,014682553
217150_s_at	NF2	-0,609040214	-0,623254098	0,014213885
206681_x_at	GP2	-0,609040214	-0,623254098	0,014213885
236217_at	SLC31A1	-0,609040214	-0,623254098	0,014213885
240381_at	ZBED5	-0,609040214	-0,623254098	0,014213885
1569700_s_at	AK7	-0,609040214	-0,623254098	0,014213885
226346_at	MEX3A	-0,609040214	-0,623254098	0,014213885
202440_s_at	ST5	-0,609040214	-0,623254098	0,014213885
230613_at	GMEB1	-0,609040214	-0,623254098	0,014213885
204909_at	DDX6	-0,609040214	-0,623254098	0,014213885
222392_x_at	PERP	4,27728562	4,263096467	0,014189153
230444_at	PDE7A	1,701542258	1,687645398	0,01389686
226130_at	RPS16	0,648195588	0,634301128	0,01389446
212192_at	KCTD12	1,843966654	1,830129603	0,013837051
229647_at	NDUFS1	2,657308324	2,64347734	0,013830984
217470_at	-	-2,336678463	-2,350444759	0,013766296
234480_at	DKFZP761C1711	-2,336678463	-2,350444759	0,013766296
234123_at	-	-2,336678463	-2,350444759	0,013766296
1569587_at	-	-2,336678463	-2,350444759	0,013766296
1563827_at	LOC158434	-2,336678463	-2,350444759	0,013766296
1557256_a_at	-	-2,336678463	-2,350444759	0,013766296
215646_s_at	VCAN	-2,336678463	-2,350444759	0,013766296
234544_at	ARHGEF12	-2,336678463	-2,350444759	0,013766296
227911_at	ARHGAP28	-2,336678463	-2,350444759	0,013766296
1555538_s_at	FAM9B	-2,336678463	-2,350444759	0,013766296
236573_at	MIB1	-2,336678463	-2,350444759	0,013766296
1555123_at	ST6GAL2	-2,336678463	-2,350444759	0,013766296
1553433_at	CCDC171	-2,336678463	-2,350444759	0,013766296
1552825_at	ZNF396	-2,336678463	-2,350444759	0,013766296
215059_at	-	-2,336678463	-2,350444759	0,013766296
240597_at	-	-2,336678463	-2,350444759	0,013766296
242626_at	SAMD5	-2,336678463	-2,350444759	0,013766296
224189_x_at	EHF	-2,336678463	-2,350444759	0,013766296
1561014_at	-	-2,336678463	-2,350444759	0,013766296
234389_x_at	-	-2,336678463	-2,350444759	0,013766296
242605_at	DCN	-2,336678463	-2,350444759	0,013766296
1566469_at	-	-2,336678463	-2,350444759	0,013766296
1569835_at	-	-2,336678463	-2,350444759	0,013766296
231703_s_at	ADH4	-2,336678463	-2,350444759	0,013766296
215079_at	-	-2,336678463	-2,350444759	0,013766296
1552509_a_at	CD300LG	-2,336678463	-2,350444759	0,013766296
1563010_at	ITGB8	-2,336678463	-2,350444759	0,013766296
232768_at	CCNB2	-2,336678463	-2,350444759	0,013766296

233120_at	-	-2,336678463	-2,350444759	0,013766296
1560086_at	-	-2,336678463	-2,350444759	0,013766296
210516_at	FAM120A	-2,336678463	-2,350444759	0,013766296
243190_at	-	-2,336678463	-2,350444759	0,013766296
243672_at	SALL3	-2,336678463	-2,350444759	0,013766296
208298_at	EVI5	-2,336678463	-2,350444759	0,013766296
216844_at	ZC3H7B	-2,336678463	-2,350444759	0,013766296
1555985_at	C17orf64	-2,336678463	-2,350444759	0,013766296
228948_at	EPHA4	-2,336678463	-2,350444759	0,013766296
244463_at	ADAM23	-2,336678463	-2,350444759	0,013766296
1561207_at	-	-0,437449947	-0,45121326	0,013763312
228208_x_at	ZNF354C	-0,437449947	-0,45121326	0,013763312
202713_s_at	KIAA0391	2,791258167	2,777528602	0,013729564
202658_at	PEX11B	3,313347235	3,299705477	0,013641757
219809_at	WDR55	1,830468611	1,817403386	0,013065225
208993_s_at	PPIG	4,102051194	4,089053866	0,012997328
243931_at	-	2,685337919	2,672497494	0,012840425
239138_at	NAA50	0,319287178	0,306454867	0,012832311
235107_at	-	0,319287178	0,306454867	0,012832311
220291_at	GDPD2	0,319287178	0,306454867	0,012832311
201067_at	PSMC2	3,807915835	3,795295041	0,012620794
209313_at	GPN1	4,046717183	4,034565906	0,012151277
202744_at	SLC20A2	0,001114523	-0,011001236	0,012115759
207999_s_at	ADARB1	0,001114523	-0,011001236	0,012115759
227559_at	NDUFAF4	1,345611839	1,333668715	0,011943124
1558636_s_at	ADAMTS5	-2,416625063	-2,4284334	0,011808337
216314_at	CRISP1	-2,416625063	-2,4284334	0,011808337
206165_s_at	CLCA2	-2,416625063	-2,4284334	0,011808337
233280_at	-	-2,416625063	-2,4284334	0,011808337
216722_at	VENTXP1	-2,416625063	-2,4284334	0,011808337
216466_at	NAV3	-2,416625063	-2,4284334	0,011808337
214914_at	FAM13C	-2,416625063	-2,4284334	0,011808337
241441_at	-	-2,416625063	-2,4284334	0,011808337
1560915_at	DPY19L1	-2,416625063	-2,4284334	0,011808337
235944_at	HMCN1	-2,416625063	-2,4284334	0,011808337
1569809_at	-	-2,416625063	-2,4284334	0,011808337
1558757_at	-	-2,416625063	-2,4284334	0,011808337
237923_at	-	-2,416625063	-2,4284334	0,011808337
244083_at	-	-2,416625063	-2,4284334	0,011808337
1557791_at	-	-2,416625063	-2,4284334	0,011808337
1554722_at	TRPM3	-2,416625063	-2,4284334	0,011808337
1566081_at	DLEU7	-2,416625063	-2,4284334	0,011808337
1553440_at	CHST9-AS1	-2,416625063	-2,4284334	0,011808337
1561600_at	-	-2,416625063	-2,4284334	0,011808337
224363_at	-	-2,416625063	-2,4284334	0,011808337
206214_at	PLA2G7	-2,416625063	-2,4284334	0,011808337
224226_at	PKD2L2	-2,416625063	-2,4284334	0,011808337
1568690_a_at	LOC100631378	-2,416625063	-2,4284334	0,011808337
205358_at	GRIA2	-2,416625063	-2,4284334	0,011808337
1557133_at	RP1-177G6.2	-2,416625063	-2,4284334	0,011808337

213362_at	PTPRD	-2,416625063	-2,4284334	0,011808337
207810_at	F13B	-2,416625063	-2,4284334	0,011808337
234798_x_at	-	-2,416625063	-2,4284334	0,011808337
1559840_s_at	TBX18	-2,416625063	-2,4284334	0,011808337
216513_at	DCT	-2,416625063	-2,4284334	0,011808337
206819_at	POM121L9P	-2,416625063	-2,4284334	0,011808337
1555636_at	CD300LG	-2,416625063	-2,4284334	0,011808337
219872_at	FAM198B	-2,416625063	-2,4284334	0,011808337
1555640_at	DAOA	-2,416625063	-2,4284334	0,011808337
1554494_at	MTHFSD	-2,416625063	-2,4284334	0,011808337
1564276_at	C5orf56	-2,416625063	-2,4284334	0,011808337
223962_at	TTC29	-2,416625063	-2,4284334	0,011808337
206084_at	PTPRR	-2,416625063	-2,4284334	0,011808337
1563445_x_at	CTSL1P8	-2,416625063	-2,4284334	0,011808337
1564359_a_at	LOC339260	-2,416625063	-2,4284334	0,011808337
205000_at	DDX3Y	-2,416625063	-2,4284334	0,011808337
202833_s_at	SERPINA1	-2,416625063	-2,4284334	0,011808337
210338_s_at	HSPA8 /// SNORD1	7,650882442	7,639282452	0,011599989
223101_s_at	ARPC5L	4,317231729	4,306269579	0,01096215
218431_at	C14orf133	3,19484646	3,18412401	0,01072245
219904_at	ZSCAN5A	-0,280273599	-0,290694975	0,010421376
222260_at	-	-0,280273599	-0,290694975	0,010421376
1553656_at	TECTB	-0,280273599	-0,290694975	0,010421376
213035_at	ANKRD28	2,348178434	2,338020736	0,010157698
232524_x_at	ANAPC4	3,986286762	3,976310891	0,009975871
1554650_a_at	-	-0,135065865	-0,144765583	0,009699718
212505_s_at	MAU2	2,374261973	2,364676067	0,009585906
1554539_a_at	RHOF	2,866549889	2,857256661	0,009293228
208595_s_at	MBD1	1,287793882	1,278654284	0,009139599
220382_s_at	ARHGAP28	-1,802716385	-1,811780055	0,00906367
227870_at	IGDCC4	-1,802716385	-1,811780055	0,00906367
1556813_at	-	-1,802716385	-1,811780055	0,00906367
240634_x_at	-	-1,802716385	-1,811780055	0,00906367
206932_at	CH25H	-1,802716385	-1,811780055	0,00906367
234401_at	-	-1,802716385	-1,811780055	0,00906367
1561104_at	-	-1,802716385	-1,811780055	0,00906367
237613_at	FOXR1	-1,802716385	-1,811780055	0,00906367
1554440_at	KIAA0513	-1,802716385	-1,811780055	0,00906367
1562509_at	-	-1,802716385	-1,811780055	0,00906367
213003_s_at	KIAA0146	-1,802716385	-1,811780055	0,00906367
232855_at	-	-1,802716385	-1,811780055	0,00906367
230903_s_at	C8orf42	-1,802716385	-1,811780055	0,00906367
204885_s_at	MSLN	-1,802716385	-1,811780055	0,00906367
1557793_at	ESYT3	-1,802716385	-1,811780055	0,00906367
243564_at	PDE1C	-1,802716385	-1,811780055	0,00906367
1561132_at	DHX35	-1,802716385	-1,811780055	0,00906367
240697_at	-	-1,802716385	-1,811780055	0,00906367
230583_s_at	LOC100287728 ///	-1,802716385	-1,811780055	0,00906367
244856_at	-	-1,802716385	-1,811780055	0,00906367
215730_at	VGLL1	-1,802716385	-1,811780055	0,00906367

221118_at	PKD2L2	-1,802716385	-1,811780055	0,00906367
1565339_at	DNAH10	-1,802716385	-1,811780055	0,00906367
1552860_at	LOC100506888 ///	-1,802716385	-1,811780055	0,00906367
222137_at	CC2D1A	-1,802716385	-1,811780055	0,00906367
211095_at	NF1	-1,802716385	-1,811780055	0,00906367
214200_s_at	COL6A1	-1,802716385	-1,811780055	0,00906367
234419_x_at	IGHA1 /// IGHG1 /,	-1,802716385	-1,811780055	0,00906367
241214_at	-	-1,802716385	-1,811780055	0,00906367
1556801_at	LOC400794	-1,802716385	-1,811780055	0,00906367
243322_at	-	-1,802716385	-1,811780055	0,00906367
1561245_at	-	-1,802716385	-1,811780055	0,00906367
219975_x_at	OLAH	-1,802716385	-1,811780055	0,00906367
205268_s_at	ADD2	-1,802716385	-1,811780055	0,00906367
227772_at	LATS1	2,71254166	2,703479652	0,009062008
226652_at	USP3	1,745502609	1,736450458	0,009052151
225446_at	BRWD1	2,053550345	2,044580955	0,00896939
225995_x_at	WASH1 /// WASH2	2,019346222	2,010406245	0,008939977
203904_x_at	CD82	2,914752575	2,906008589	0,008743987
201444_s_at	ATP6AP2	3,768156719	3,759583763	0,008572956
208840_s_at	G3BP2	2,811067138	2,802594885	0,008472252
213263_s_at	PCBP2	2,107136847	2,099079406	0,008057441
232126_at	COQ2	-1,137208348	-1,145057014	0,007848666
1562336_at	CASC2	-1,137208348	-1,145057014	0,007848666
1558553_at	-	-1,137208348	-1,145057014	0,007848666
1554398_at	LYG2	-1,137208348	-1,145057014	0,007848666
1564701_at	-	-1,137208348	-1,145057014	0,007848666
1567623_at	ABLIM2	-1,137208348	-1,145057014	0,007848666
220861_at	-	-1,137208348	-1,145057014	0,007848666
229260_at	C5orf15	-1,137208348	-1,145057014	0,007848666
1563744_a_at	DISC1	-1,137208348	-1,145057014	0,007848666
242138_at	DLX1	-1,137208348	-1,145057014	0,007848666
214673_s_at	HUWE1	-1,137208348	-1,145057014	0,007848666
218141_at	UBE2O	1,843966654	1,836262316	0,007704338
1566954_at	-	0,045517965	0,03784737	0,007670594
212497_at	MAPK1IP1L	0,045517965	0,03784737	0,007670594
1554168_a_at	SH3KBP1	6,130360359	6,122821738	0,00753862
222086_s_at	WNT6	-0,924000698	-0,931539147	0,007538449
211091_s_at	NF2	-0,924000698	-0,931539147	0,007538449
208556_at	GPR31	-0,924000698	-0,931539147	0,007538449
230094_at	-	-0,924000698	-0,931539147	0,007538449
231890_at	-	-0,924000698	-0,931539147	0,007538449
233265_at	-	-0,924000698	-0,931539147	0,007538449
1554181_at	SNX32	-0,924000698	-0,931539147	0,007538449
239194_at	DNAJC30	-0,924000698	-0,931539147	0,007538449
243797_at	STK17B	-0,924000698	-0,931539147	0,007538449
204548_at	STAR	-0,924000698	-0,931539147	0,007538449
241362_at	SOGA1	-0,924000698	-0,931539147	0,007538449
1562749_at	LOC644090	-0,924000698	-0,931539147	0,007538449
223795_at	TSPAN10	-0,924000698	-0,931539147	0,007538449
236998_at	CCDC56	-0,924000698	-0,931539147	0,007538449

241966_at	MYO5A	-0,924000698	-0,931539147	0,007538449
209995_s_at	TCL1A	-0,924000698	-0,931539147	0,007538449
233402_at	-	-0,924000698	-0,931539147	0,007538449
1556753_s_at	-	-0,924000698	-0,931539147	0,007538449
241907_at	-	-0,924000698	-0,931539147	0,007538449
241262_at	-	0,20740896	0,20062106	0,006787901
229146_at	C7orf31	0,20740896	0,20062106	0,006787901
233186_s_at	BANP	1,561816652	1,555165465	0,006651187
214045_at	LIAS	2,492739983	2,486505579	0,006234404
1560168_at	LOC646168	-2,495152701	-2,501359113	0,006206413
215514_at	-	-2,495152701	-2,501359113	0,006206413
243632_at	-	-2,495152701	-2,501359113	0,006206413
1566096_x_at	-	-2,495152701	-2,501359113	0,006206413
238481_at	MGP	-2,495152701	-2,501359113	0,006206413
1560538_at	-	-2,495152701	-2,501359113	0,006206413
241506_at	-	-2,495152701	-2,501359113	0,006206413
1561247_at	LOC283682	-2,495152701	-2,501359113	0,006206413
234853_s_at	EFCAB6	-2,495152701	-2,501359113	0,006206413
215944_at	-	-2,495152701	-2,501359113	0,006206413
1563825_at	-	-2,495152701	-2,501359113	0,006206413
1558793_at	FRY-AS1	-2,495152701	-2,501359113	0,006206413
243478_at	CHST2	-2,495152701	-2,501359113	0,006206413
216625_at	-	-2,495152701	-2,501359113	0,006206413
1562715_at	-	-2,495152701	-2,501359113	0,006206413
229245_at	PLEKHA6	-2,495152701	-2,501359113	0,006206413
243146_at	-	-2,495152701	-2,501359113	0,006206413
239667_at	SLC3A1	-2,495152701	-2,501359113	0,006206413
210055_at	TSHR	-2,495152701	-2,501359113	0,006206413
240772_at	-	-2,495152701	-2,501359113	0,006206413
221805_at	NEFL	-2,495152701	-2,501359113	0,006206413
244432_at	-	-2,495152701	-2,501359113	0,006206413
1560863_a_at	-	-2,495152701	-2,501359113	0,006206413
234038_at	-	-2,495152701	-2,501359113	0,006206413
1563519_at	-	-2,495152701	-2,501359113	0,006206413
238796_at	-	-2,495152701	-2,501359113	0,006206413
235852_at	STON2	-2,495152701	-2,501359113	0,006206413
1557864_x_at	-	-2,495152701	-2,501359113	0,006206413
1553472_at	FLJ32955	-2,495152701	-2,501359113	0,006206413
233884_at	HIVEP3	-2,495152701	-2,501359113	0,006206413
1559640_at	ANKFN1	-2,495152701	-2,501359113	0,006206413
220794_at	GREM2	-2,495152701	-2,501359113	0,006206413
234852_at	-	-2,495152701	-2,501359113	0,006206413
238300_s_at	DAZAP2	-2,495152701	-2,501359113	0,006206413
220655_at	TNIP3	-2,495152701	-2,501359113	0,006206413
212232_at	FNBP4	3,618399139	3,612221266	0,006177873
205642_at	CNTRL	2,38349585	2,37755543	0,00594042
221708_s_at	UNC45A	1,816938997	1,811004388	0,005934609
209858_x_at	MPPE1	3,161901883	3,155995136	0,005906747
229632_s_at	INTS10	3,969866565	3,963985389	0,005881176
231612_at	CABS1	-2,774503732	-2,780225148	0,005721415

1561331_at	ERVMER61-1	-2,774503732	-2,780225148	0,005721415
1562201_x_at	-	-2,774503732	-2,780225148	0,005721415
207802_at	CRISP3	-2,774503732	-2,780225148	0,005721415
1564466_at	FLJ37644	-2,774503732	-2,780225148	0,005721415
227198_at	AFF3	-2,774503732	-2,780225148	0,005721415
208785_s_at	MAP1LC3B	3,881435528	3,875872341	0,005563187
201705_at	PSMD7	4,834334905	4,829088974	0,005245931
225844_at	POLE4	3,739061547	3,733850354	0,005211192
218181_s_at	MAP4K4	2,824160383	2,819209749	0,004950634
1554170_a_at	MBLAC1	0,847529938	0,842599219	0,004930718
227894_at	WDR90	0,847529938	0,842599219	0,004930718
227153_at	IMMP2L	2,507817181	2,503049671	0,00476751
1567014_s_at	CSNK2A1 /// CSNK	1,788922638	1,784182261	0,004740377
225582_at	ITPRIP	1,20396005	1,199296812	0,004663238
241686_x_at	-	0,95222556	0,947673886	0,004551674
236004_at	-	1,160996507	1,156599976	0,004396531
224390_s_at	RGS8	-0,730013898	-0,734355396	0,004341498
1563109_at	-	-0,730013898	-0,734355396	0,004341498
216079_at	EPM2A	-0,730013898	-0,734355396	0,004341498
239737_at	-	-0,730013898	-0,734355396	0,004341498
235861_at	-	-0,730013898	-0,734355396	0,004341498
221976_s_at	HDGFRP3	-0,730013898	-0,734355396	0,004341498
234998_at	RAB11A	0,58788177	0,583607503	0,004274267
208947_s_at	UPF1	1,000459215	0,996453274	0,004005941
223089_at	VEZT	1,775035811	1,771157334	0,003878477
224886_at	JMJD8	1,047341799	1,043469112	0,003872686
207513_s_at	ZNF189	2,613382867	2,609615156	0,003767711
219176_at	C2orf47	3,657457797	3,653742074	0,003715723
207941_s_at	RBM39	4,572368635	4,568759722	0,003608912
236265_at	SP4	3,050461597	3,046977905	0,003483692
239749_at	FAF1	-0,551284523	-0,554696666	0,003412144
217603_at	ATP6V0A2	-0,551284523	-0,554696666	0,003412144
1553386_at	MFSD2A	-0,551284523	-0,554696666	0,003412144
1565715_at	-	-0,551284523	-0,554696666	0,003412144
214158_s_at	PRDM10	-0,551284523	-0,554696666	0,003412144
203727_at	SKIV2L	1,871521385	1,868130962	0,003390423
1569871_at	LMF1	0,900065578	0,896834102	0,003231476
227098_at	DUSP18	0,900065578	0,896834102	0,003231476
208872_s_at	REEP5	4,42686741	4,42372331	0,0031441
1569318_at	LOC284440	-1,369772723	-1,372734086	0,002961363
205715_at	BST1	-1,369772723	-1,372734086	0,002961363
221371_at	TNFSF18	-1,369772723	-1,372734086	0,002961363
242654_at	FANCC	-1,369772723	-1,372734086	0,002961363
209842_at	SOX10	-1,369772723	-1,372734086	0,002961363
1559714_at	RGR	-1,369772723	-1,372734086	0,002961363
240024_at	SEC14L2	-1,369772723	-1,372734086	0,002961363
224105_x_at	-	-1,369772723	-1,372734086	0,002961363
1562319_at	FAM169B	-1,369772723	-1,372734086	0,002961363
1564193_at	FLJ39061 /// LOC1	-1,369772723	-1,372734086	0,002961363
234152_at	-	-1,369772723	-1,372734086	0,002961363

1561065_at	-	-1,369772723	-1,372734086	0,002961363
241123_at	-	-1,369772723	-1,372734086	0,002961363
240384_at	-	-1,369772723	-1,372734086	0,002961363
232078_at	PVRL2	-1,369772723	-1,372734086	0,002961363
227306_at	-	-1,369772723	-1,372734086	0,002961363
238603_at	LOC254559	-1,369772723	-1,372734086	0,002961363
227175_at	MCL1	-1,369772723	-1,372734086	0,002961363
1552384_a_at	FAM71A	-1,369772723	-1,372734086	0,002961363
1561419_at	-	-1,369772723	-1,372734086	0,002961363
208425_s_at	TANC2	-1,369772723	-1,372734086	0,002961363
235655_at	-	-1,712732543	-1,715576125	0,002843582
1560562_a_at	ZNF677	-1,712732543	-1,715576125	0,002843582
1567008_at	-	-1,712732543	-1,715576125	0,002843582
243525_at	-	-1,712732543	-1,715576125	0,002843582
1556638_at	PAX7	-1,712732543	-1,715576125	0,002843582
236948_x_at	SRSF11	-1,712732543	-1,715576125	0,002843582
1568695_s_at	INTS6	-1,712732543	-1,715576125	0,002843582
1553420_at	SATB2-AS1	-1,712732543	-1,715576125	0,002843582
217003_s_at	ADAM5P	-1,712732543	-1,715576125	0,002843582
208521_at	OR5I1	-1,712732543	-1,715576125	0,002843582
237924_at	-	-1,712732543	-1,715576125	0,002843582
1559756_at	DKFZp667F0711	-1,712732543	-1,715576125	0,002843582
1562036_at	-	-1,712732543	-1,715576125	0,002843582
202917_s_at	S100A8	-1,712732543	-1,715576125	0,002843582
221089_at	NOX3	-1,712732543	-1,715576125	0,002843582
231408_at	TMEM92	-1,712732543	-1,715576125	0,002843582
213308_at	SHANK2	-1,712732543	-1,715576125	0,002843582
208513_at	FOXB1	-1,712732543	-1,715576125	0,002843582
238564_at	FAM171B	-1,712732543	-1,715576125	0,002843582
233723_at	-	-1,712732543	-1,715576125	0,002843582
214396_s_at	MBD2	-1,712732543	-1,715576125	0,002843582
234179_at	-	-1,712732543	-1,715576125	0,002843582
1555248_a_at	WNK3	-1,712732543	-1,715576125	0,002843582
210370_s_at	LY9	-1,712732543	-1,715576125	0,002843582
241884_at	-	-1,712732543	-1,715576125	0,002843582
212551_at	CAP2	-1,712732543	-1,715576125	0,002843582
236628_at	-	-1,712732543	-1,715576125	0,002843582
214841_at	CNIH3 /// LOC100!	-1,712732543	-1,715576125	0,002843582
215216_at	VPS16	-1,712732543	-1,715576125	0,002843582
204466_s_at	SNCA	-1,712732543	-1,715576125	0,002843582
216144_at	-	-1,712732543	-1,715576125	0,002843582
215263_at	ZXDA /// ZXDB	-1,712732543	-1,715576125	0,002843582
1555488_at	-	-1,712732543	-1,715576125	0,002843582
225079_at	EMP2	-1,712732543	-1,715576125	0,002843582
231318_at	DNM1P46	-1,712732543	-1,715576125	0,002843582
234742_at	SIRPG	-1,712732543	-1,715576125	0,002843582
244637_at	-	-1,712732543	-1,715576125	0,002843582
242203_at	-	-1,712732543	-1,715576125	0,002843582
240754_at	LOC284933	-1,712732543	-1,715576125	0,002843582
227376_at	GLI3	-1,712732543	-1,715576125	0,002843582

232426_at	SV2B	-0,384193355	-0,386896102	0,002702747
221489_s_at	SPRY4	-0,384193355	-0,386896102	0,002702747
220353_at	FAM86C1	-0,384193355	-0,386896102	0,002702747
230845_at	HOXB-AS5	-0,384193355	-0,386896102	0,002702747
229556_at	LOC100288893	-0,384193355	-0,386896102	0,002702747
1570569_at	-	-0,384193355	-0,386896102	0,002702747
220605_s_at	SIRT2	2,096248267	2,093850742	0,002397526
218601_at	URGCP	2,007220798	2,004917973	0,002302825
209435_s_at	ARHGEF2	3,394274216	3,392128215	0,002146001
212388_at	USP24	3,007119335	3,005076416	0,002042919
212348_s_at	KDM1A	3,485638812	3,483950333	0,001688479
206918_s_at	CPNE1	3,331722254	3,330174376	0,001547878
203014_x_at	SGSM3	1,326463531	1,324949741	0,00151379
242820_at	-	-2,569309811	-2,570522741	0,00121293
213910_at	IGFBP7	-2,569309811	-2,570522741	0,00121293
1569794_at	-	-2,569309811	-2,570522741	0,00121293
215047_at	TRIM58	-2,569309811	-2,570522741	0,00121293
1558754_at	ZNF763	-2,569309811	-2,570522741	0,00121293
1569874_s_at	LIPJ	-2,569309811	-2,570522741	0,00121293
1570246_at	-	-2,569309811	-2,570522741	0,00121293
215309_at	ZNF674	-2,569309811	-2,570522741	0,00121293
204111_at	HNMT	-2,569309811	-2,570522741	0,00121293
1569168_at	-	-2,569309811	-2,570522741	0,00121293
1552783_at	ZNF417	-2,569309811	-2,570522741	0,00121293
1563135_at	-	-2,569309811	-2,570522741	0,00121293
222359_x_at	KDSR	-2,569309811	-2,570522741	0,00121293
1556666_a_at	LOC100652860 ///	-2,569309811	-2,570522741	0,00121293
1561740_at	-	-2,569309811	-2,570522741	0,00121293
1562093_at	-	-2,569309811	-2,570522741	0,00121293
216874_at	-	-2,569309811	-2,570522741	0,00121293
216030_s_at	SEMG2	-2,569309811	-2,570522741	0,00121293
1564015_at	PIEZO2	-2,569309811	-2,570522741	0,00121293
1562133_x_at	ZNF90	-2,569309811	-2,570522741	0,00121293
209335_at	DCN	-2,569309811	-2,570522741	0,00121293
215249_at	RPL35A	-2,569309811	-2,570522741	0,00121293
241323_at	-	-2,569309811	-2,570522741	0,00121293
212328_at	LIMCH1	-2,569309811	-2,570522741	0,00121293
223475_at	CRISPLD1	-2,569309811	-2,570522741	0,00121293
221686_s_at	RECQL5	1,5944803	1,593605508	0,000874792
218052_s_at	ATP13A1	1,5944803	1,593605508	0,000874792
1555764_s_at	TIMM10	4,500752401	4,499991499	0,000760901
240097_at	-	-0,088974936	-0,089635713	0,000660777
236424_at	CEP250	-0,088974936	-0,089635713	0,000660777
242217_s_at	FBRS	0,08619576	0,085534992	0,000660767
225992_at	MLLT10	3,86465425	3,864033023	0,000621227
235194_at	TPCN2	-0,230432956	-0,231045907	0,000612951
226646_at	KLF2	-0,230432956	-0,231045907	0,000612951
231272_at	MED7	-0,230432956	-0,231045907	0,000612951
218621_at	HEMK1	-0,230432956	-0,231045907	0,000612951
228160_at	LOC339290	1,476175815	1,475630127	0,000545688

223440_at	C16orf70	1,476175815	1,475630127	0,000545688
213511_s_at	MTMR1	3,430413158	3,429874957	0,000538201
1554192_s_at	TRMT44	0,618277321	0,61779983	0,000477491
202161_at	PKN1	0,618277321	0,61779983	0,000477491
214119_s_at	FKBP1A	3,081633533	3,081435405	0,000198127
1562890_at	-	0,244681185	0,244646697	3,44882E-05
217939_s_at	AFTPH	3,840618883	3,84088223	-0,000263346
211023_at	PDHB	5,915781666	5,916130517	-0,000348851
235171_at	LOC100505501	2,436165655	2,436714502	-0,000548848
202231_at	EIF3M	6,726150408	6,726843089	-0,000692681
1568613_at	RSPH3	1,287793882	1,28853674	-0,000742858
221547_at	PRPF18	2,99543179	2,99622929	-0,0007975
209891_at	SPC25	2,725848647	2,726850891	-0,001002244
228258_at	TBC1D10C	2,739067176	2,740247037	-0,001179862
203048_s_at	TTC37	2,972517974	2,973964204	-0,00144623
224855_at	PYCR2	3,086998005	3,08892491	-0,001926905
231815_at	PHF12	0,648195588	0,65030602	-0,002110432
215541_s_at	DIAPH1	2,552836808	2,55501164	-0,002174832
229563_s_at	RPL10A	8,285246714	8,287663443	-0,002416729
211087_x_at	MAPK14	0,458905032	0,461385738	-0,002480706
228725_x_at	PRMT2	3,889146356	3,891666284	-0,002519928
1552972_at	LOC100507431	-2,834722577	-2,832036647	-0,00268593
213253_at	SMC2	3,007119335	3,010094234	-0,002974899
212706_at	RASA4 /// RASA4B	2,16969527	2,173028091	-0,003332822
200963_x_at	RPL31	8,038826489	8,042281172	-0,003454682
219486_at	DUS2L	2,138605583	2,142313983	-0,0037084
200689_x_at	EEF1G /// MIR365	8,078988308	8,082972152	-0,003983844
209899_s_at	PUF60	4,239531398	4,243633606	-0,004102209
213589_s_at	B3GNTL1	0,677745787	0,681874787	-0,004129
218659_at	ASXL2	2,725848647	2,730083062	-0,004234415
222526_at	GATAD2A	1,640193838	1,644429915	-0,004236077
221913_at	SIRT3	0,707657549	0,711934445	-0,004276896
211746_x_at	PSMA1	6,919763312	6,924057197	-0,004293886
211727_s_at	COX11	4,679156164	4,683596099	-0,004439935
202303_x_at	SMARCA5	3,050461597	3,05501254	-0,004550943
202227_s_at	BRD8	4,153515595	4,158360228	-0,004844633
217958_at	MIR3656 /// TRAP	4,721089228	4,726291071	-0,005201843
226278_at	SVIP	3,533714224	3,538959266	-0,005245042
222250_s_at	INTS7	3,422378845	3,427694878	-0,005316033
206654_s_at	POLR3G	0,127732831	0,133104519	-0,005371688
218632_at	HECTD3	0,491906512	0,497424714	-0,005518203
238620_at	-	-1,06282519	-1,057301851	-0,005523339
239990_at	-	-1,06282519	-1,057301851	-0,005523339
1553498_at	-	-1,06282519	-1,057301851	-0,005523339
235101_at	FNBP4	-1,06282519	-1,057301851	-0,005523339
241020_at	-	-1,06282519	-1,057301851	-0,005523339
1555655_at	OR10A4	-1,06282519	-1,057301851	-0,005523339
207191_s_at	ISLR	-1,06282519	-1,057301851	-0,005523339
237383_at	-	-1,06282519	-1,057301851	-0,005523339
1552604_at	LINC00308	-1,06282519	-1,057301851	-0,005523339

232781_at	LHX4	-1,06282519	-1,057301851	-0,005523339
232208_at	ISLR2	-1,06282519	-1,057301851	-0,005523339
236001_at	LOC100289255	-1,06282519	-1,057301851	-0,005523339
206946_at	HCN4	-1,06282519	-1,057301851	-0,005523339
215433_at	DPY19L1	-1,06282519	-1,057301851	-0,005523339
232060_at	ROR1	-2,641232718	-2,635549085	-0,005683633
233662_at	CDH26	-2,641232718	-2,635549085	-0,005683633
1556834_at	LOC100652770	-2,641232718	-2,635549085	-0,005683633
1559652_at	-	-2,641232718	-2,635549085	-0,005683633
216435_at	MAP2K5	-2,641232718	-2,635549085	-0,005683633
240724_at	-	-2,641232718	-2,635549085	-0,005683633
233133_at	-	-2,641232718	-2,635549085	-0,005683633
1555601_at	-	-2,641232718	-2,635549085	-0,005683633
1557822_at	LOC401134	-2,641232718	-2,635549085	-0,005683633
1553792_at	KIAA1109	-2,641232718	-2,635549085	-0,005683633
237138_at	-	-2,641232718	-2,635549085	-0,005683633
1566515_at	CWF19L2	-2,641232718	-2,635549085	-0,005683633
205440_s_at	NPY1R	-2,641232718	-2,635549085	-0,005683633
235591_at	SSTR1	-2,641232718	-2,635549085	-0,005683633
228202_at	-	-2,641232718	-2,635549085	-0,005683633
206135_at	ST18	-2,641232718	-2,635549085	-0,005683633
237160_at	CCDC83	-2,641232718	-2,635549085	-0,005683633
1561953_at	-	-2,641232718	-2,635549085	-0,005683633
1564392_at	LINC00320	-2,641232718	-2,635549085	-0,005683633
244560_at	-	-2,641232718	-2,635549085	-0,005683633
229222_at	ACSS3	-2,641232718	-2,635549085	-0,005683633
227850_x_at	CDC42EP5	-2,641232718	-2,635549085	-0,005683633
244723_at	ACSM2A	-2,641232718	-2,635549085	-0,005683633
240880_at	-	-2,641232718	-2,635549085	-0,005683633
91703_at	EHBP1L1	1,617114952	1,622908225	-0,005793273
201781_s_at	AIP	3,649735397	3,655528924	-0,005793527
235780_at	PRKACB	2,295108393	2,300902761	-0,005794368
200060_s_at	RNPS1	4,631436665	4,637292432	-0,005855767
229526_at	AQP11	1,182925501	1,189078962	-0,006153461
222893_s_at	RPAP2	2,560606634	2,56688207	-0,006275436
217845_x_at	HIGD1A	6,021772433	6,028172542	-0,006400109
239316_at	METTL12	0,735999505	0,742671819	-0,006672314
241602_at	ZNF582	0,735999505	0,742671819	-0,006672314
232190_x_at	LOC100133445 ///	1,160996507	1,167919222	-0,006922715
210188_at	GABPA	1,160996507	1,167919222	-0,006922715
238803_at	HECTD2	-0,856259172	-0,84928999	-0,006969182
1564336_at	-	-0,856259172	-0,84928999	-0,006969182
213925_at	C1orf95	-0,856259172	-0,84928999	-0,006969182
1559127_x_at	RRP12	-0,856259172	-0,84928999	-0,006969182
214675_at	NUP188	-0,856259172	-0,84928999	-0,006969182
214777_at	-	-0,856259172	-0,84928999	-0,006969182
242119_at	PROX1	-0,856259172	-0,84928999	-0,006969182
236516_at	-	-0,856259172	-0,84928999	-0,006969182
224342_x_at	LOC96610	-0,856259172	-0,84928999	-0,006969182
208454_s_at	CPQ	-0,856259172	-0,84928999	-0,006969182

225829_at	PDZD8	-0,856259172	-0,84928999	-0,006969182
225270_at	NEO1	0,97663406	0,98381571	-0,00718165
227584_at	NAV1	1,071336699	1,078619386	-0,007282688
239173_at	INADL	-1,289805289	-1,282520722	-0,007284567
232757_at	-	-1,289805289	-1,282520722	-0,007284567
1561273_at	-	-1,289805289	-1,282520722	-0,007284567
221266_s_at	DCSTAMP	-1,289805289	-1,282520722	-0,007284567
236333_at	-	-1,289805289	-1,282520722	-0,007284567
214767_s_at	HSPB6	-1,289805289	-1,282520722	-0,007284567
1552546_a_at	LETM2	-1,289805289	-1,282520722	-0,007284567
1569953_at	-	-1,289805289	-1,282520722	-0,007284567
1569583_at	EREG	-1,289805289	-1,282520722	-0,007284567
1560219_at	ASB3	-1,289805289	-1,282520722	-0,007284567
233331_at	-	-1,289805289	-1,282520722	-0,007284567
1554978_at	ERICH1-AS1	-1,289805289	-1,282520722	-0,007284567
219832_s_at	HOXC13	-1,289805289	-1,282520722	-0,007284567
206877_at	MXD1	-1,289805289	-1,282520722	-0,007284567
243207_at	-	-1,289805289	-1,282520722	-0,007284567
203343_at	UGDH	2,25755351	2,264929589	-0,007376079
217547_x_at	ZNF675	-0,043925712	-0,036543687	-0,007382025
210610_at	CEACAM1	-0,043925712	-0,036543687	-0,007382025
210154_at	ME2	3,166767593	3,174156454	-0,007388861
217200_x_at	CYB561	0,319287178	0,326777209	-0,007490032
200084_at	C11orf58	5,807751717	5,815423292	-0,007671574
217829_s_at	USP39	3,64600241	3,653742074	-0,007739664
219178_at	QTRTD1	1,000459215	1,008210238	-0,007751023
219129_s_at	SAP30L	2,053550345	2,06133574	-0,007785395
226289_at	CAPRIN1	0,95222556	0,960042218	-0,007816658
232934_at	-	-1,624955693	-1,616924751	-0,008030942
230010_at	SRRM4	-1,624955693	-1,616924751	-0,008030942
1553772_at	GK5	-1,624955693	-1,616924751	-0,008030942
229808_at	CHAF1A	-1,624955693	-1,616924751	-0,008030942
230785_at	-	-1,624955693	-1,616924751	-0,008030942
244114_x_at	-	-1,624955693	-1,616924751	-0,008030942
240504_at	-	-1,624955693	-1,616924751	-0,008030942
244638_at	SUCLG1	-1,624955693	-1,616924751	-0,008030942
1562337_at	OR7D2	-1,624955693	-1,616924751	-0,008030942
237946_at	-	-1,624955693	-1,616924751	-0,008030942
207519_at	SLC6A4	-1,624955693	-1,616924751	-0,008030942
215774_s_at	SUCLG2	-1,624955693	-1,616924751	-0,008030942
1563860_at	LOC400965	-1,624955693	-1,616924751	-0,008030942
222357_at	ZBTB20	-1,624955693	-1,616924751	-0,008030942
217587_at	-	-1,624955693	-1,616924751	-0,008030942
1552848_a_at	PTCHD1	-1,624955693	-1,616924751	-0,008030942
240212_at	-	-1,624955693	-1,616924751	-0,008030942
239271_at	SMAD2	-1,624955693	-1,616924751	-0,008030942
1555855_at	AKR1C1	-1,624955693	-1,616924751	-0,008030942
1559568_at	-	-1,624955693	-1,616924751	-0,008030942
206004_at	TGM3	-1,624955693	-1,616924751	-0,008030942
210961_s_at	ADRA1D	-1,624955693	-1,616924751	-0,008030942

1566598_at	-	-1,624955693	-1,616924751	-0,008030942
1561526_at	-	-1,624955693	-1,616924751	-0,008030942
220645_at	NXPE4	-1,624955693	-1,616924751	-0,008030942
241550_at	DPPA5	-1,624955693	-1,616924751	-0,008030942
1562510_at	LOC339442	-1,624955693	-1,616924751	-0,008030942
204620_s_at	VCAN	-1,624955693	-1,616924751	-0,008030942
239909_at	ADAMTSL1	-1,624955693	-1,616924751	-0,008030942
233749_at	-	-1,624955693	-1,616924751	-0,008030942
217444_at	-	-1,624955693	-1,616924751	-0,008030942
214083_at	PPP2R5C	2,365121417	2,373249966	-0,008128549
1558712_at	TMEM134	-0,66934516	-0,661178575	-0,008166585
234125_at	-	-0,66934516	-0,661178575	-0,008166585
237599_at	-	-0,66934516	-0,661178575	-0,008166585
216912_at	ARHGEF4	-0,66934516	-0,661178575	-0,008166585
239718_at	-	-0,66934516	-0,661178575	-0,008166585
233475_at	MGC32805	-0,66934516	-0,661178575	-0,008166585
1568847_at	-	-0,66934516	-0,661178575	-0,008166585
212618_at	ZNF609	-0,66934516	-0,661178575	-0,008166585
1558211_s_at	SRC	-0,66934516	-0,661178575	-0,008166585
221242_at	-	-0,66934516	-0,661178575	-0,008166585
221533_at	FAM162A	-0,66934516	-0,661178575	-0,008166585
201286_at	SDC1	-0,49284695	-0,484558493	-0,008288457
215246_at	LARP7	-0,49284695	-0,484558493	-0,008288457
227632_at	TBC1D24	-0,49284695	-0,484558493	-0,008288457
1570479_at	ART1	-0,49284695	-0,484558493	-0,008288457
217464_at	-	-0,49284695	-0,484558493	-0,008288457
210087_s_at	MPZL1	-0,49284695	-0,484558493	-0,008288457
1552833_at	B3GNT6	-0,49284695	-0,484558493	-0,008288457
222825_at	OTUD6B	3,137734155	3,14605266	-0,008318506
217726_at	COPZ1	4,113919949	4,122411329	-0,008491381
211297_s_at	CDK7	3,664722055	3,673370032	-0,008647977
201759_at	TBCD	0,764143511	0,772793615	-0,008650104
210474_s_at	CDK11A /// CDK11	1,307201325	1,315897482	-0,008696157
203179_at	GALT	1,307201325	1,315897482	-0,008696157
235114_x_at	HOOK3	0,524545436	0,533258442	-0,008713005
1557817_a_at	-	0,524545436	0,533258442	-0,008713005
233753_at	SCAF4	-0,180990326	-0,172194225	-0,008796101
232873_at	ZNF33A	-0,180990326	-0,172194225	-0,008796101
212545_s_at	ZHX3	-0,180990326	-0,172194225	-0,008796101
223770_x_at	MRI1	0,847529938	0,85655369	-0,009023752
223229_at	UBE2T	4,885515979	4,894556606	-0,009040627
223430_at	SIK2	0,791908897	0,80114751	-0,009238612
226456_at	RMI2	3,382641505	3,392128215	-0,00948671
204710_s_at	WIPI2	3,122148189	3,131713063	-0,009564873
225062_at	LOC389831	2,914752575	2,924357093	-0,009604517
223057_s_at	XPO5	2,476788561	2,486505579	-0,009717018
225655_at	UHRF1	4,142807036	4,152535046	-0,00972801
208804_s_at	SRSF6	4,721089228	4,730861472	-0,009772244
1555548_at	NDUFA10	-0,332405896	-0,322340048	-0,010065848
229827_at	BUB3	-0,332405896	-0,322340048	-0,010065848

242256_x_at	-	-0,332405896	-0,322340048	-0,010065848
228095_at	PHF14	3,739061547	3,749161949	-0,010100402
210288_at	KLRG1	0,355751005	0,366031214	-0,010280209
239083_at	ZNF786	0,355751005	0,366031214	-0,010280209
222603_at	ERMP1	2,492739983	2,503049671	-0,010309688
224875_at	C5orf24	0,900065578	0,910483921	-0,010418343
226113_at	ZNF436	0,900065578	0,910483921	-0,010418343
226379_s_at	C19orf25	0,167727503	0,178191865	-0,010464362
232970_at	-	0,167727503	0,178191865	-0,010464362
236267_at	ZNF346	1,287793882	1,298349073	-0,010555191
214144_at	POLR2D	1,287793882	1,298349073	-0,010555191
232843_s_at	DOCK8	3,442765386	3,453397238	-0,010631852
208717_at	OXA1L	4,733987652	4,744668046	-0,010680394
1557965_at	MTERFD2	2,725848647	2,736663738	-0,010815091
224882_at	ACSS1	1,440265662	1,451507543	-0,01124188
225674_at	BCAP29	3,628556015	3,639810352	-0,011254337
227395_at	TBCEL	1,65554524	1,666953618	-0,011408378
224469_s_at	INF2	0,556428218	0,568155442	-0,011727224
203960_s_at	HSPB11	3,720319513	3,732068422	-0,011748909
223033_s_at	SCYL1	1,510970097	1,523429058	-0,012458961
212110_at	SLC39A14	3,313347235	3,325881269	-0,012534034
1555102_at	FGF7	-2,708365344	-2,695640852	-0,012724492
1555816_at	THSD4	-2,708365344	-2,695640852	-0,012724492
1561222_at	LINC00485	-2,708365344	-2,695640852	-0,012724492
215294_s_at	SMARCA1	-2,708365344	-2,695640852	-0,012724492
233305_at	NECAB1	-2,708365344	-2,695640852	-0,012724492
1565937_a_at	LMO3	-2,708365344	-2,695640852	-0,012724492
1557057_a_at	C5orf47	-2,708365344	-2,695640852	-0,012724492
1562628_at	KRT40	-2,708365344	-2,695640852	-0,012724492
243346_at	LMOD3	-2,708365344	-2,695640852	-0,012724492
1556885_at	LAYN	-2,708365344	-2,695640852	-0,012724492
223907_s_at	PINX1	2,053550345	2,066329863	-0,012779518
227960_s_at	FAHD1	2,159837409	2,173028091	-0,013190683
211939_x_at	BTF3	7,660123244	7,673331408	-0,013208164
237367_x_at	CFLAR	2,085817135	2,099079406	-0,013262271
204331_s_at	MRPS12	4,37038231	4,383687696	-0,013305386
223345_at	HDAC8	1,421391637	1,434743608	-0,013351971
209207_s_at	SEC22B	3,223556135	3,236954658	-0,013398523
202941_at	NDUFV2	5,254434357	5,26790303	-0,013468673
200675_at	CD81	3,939815341	3,953789653	-0,013974311
62212_at	C1orf50 /// LOC10	2,709081266	2,723668445	-0,01458718
224835_at	GPCPD1	1,402495885	1,417157926	-0,014662042
221783_at	WIZ	0,39105295	0,405752839	-0,014699888
235686_at	TYW5	0,39105295	0,405752839	-0,014699888
227026_at	MPHOSPH8	2,621163856	2,635864822	-0,014700966
236192_at	HOOK3	1,5944803	1,60918327	-0,01470297
218698_at	APIP	4,567201338	4,582127682	-0,014926344
209385_s_at	PROSC	4,719538164	4,734546004	-0,01500784
201405_s_at	COPS6	4,840637435	4,855654877	-0,015017443
240344_x_at	LYRM7	3,286844008	3,301884701	-0,015040693

226994_at	DNAJA2	3,422378845	3,437599225	-0,01522038
203291_at	CNOT4	1,97249974	1,987810614	-0,015310875
202550_s_at	VAPB	3,681878794	3,697224187	-0,015345393
209187_at	DR1	4,699939684	4,715707406	-0,015767722
201513_at	TSN	1,775035811	1,790914523	-0,015878711
230481_at	ACY3	0,045517965	0,061773582	-0,016255617
1559927_a_at	LOC728353	0,045517965	0,061773582	-0,016255617
205743_at	STAC	0,045517965	0,061773582	-0,016255617
223375_at	TBC1D22B	1,182925501	1,199296812	-0,016371311
225017_at	CCDC14	3,152091004	3,169167796	-0,017076792
219445_at	GLTSCR1	1,160996507	1,178100917	-0,01710441
225332_at	OIP5-AS1	2,849132494	2,86626857	-0,017136076
226495_at	MAVS	2,545254728	2,562621495	-0,017366767
218842_at	RPAP3	3,470020347	3,487551166	-0,01753082
1556761_at	-	-1,537851782	-1,520022123	-0,017829659
233916_at	NYAP2	-1,537851782	-1,520022123	-0,017829659
1564802_at	-	-1,537851782	-1,520022123	-0,017829659
1556844_at	LOC100132078	-1,537851782	-1,520022123	-0,017829659
1562835_at	-	-1,537851782	-1,520022123	-0,017829659
1564280_x_at	LOC100133920	-1,537851782	-1,520022123	-0,017829659
234516_at	-	-1,537851782	-1,520022123	-0,017829659
1569616_at	GMNC	-1,537851782	-1,520022123	-0,017829659
1552507_at	KCNE4	-1,537851782	-1,520022123	-0,017829659
230614_at	-	-1,537851782	-1,520022123	-0,017829659
1560523_at	-	-1,537851782	-1,520022123	-0,017829659
243359_at	-	-1,537851782	-1,520022123	-0,017829659
1556789_a_at	GCFC1-AS1	-1,537851782	-1,520022123	-0,017829659
207716_at	KRT38	-1,537851782	-1,520022123	-0,017829659
233490_at	DCTN4	-1,537851782	-1,520022123	-0,017829659
230485_at	PHGR1	-1,537851782	-1,520022123	-0,017829659
234463_at	-	-1,537851782	-1,520022123	-0,017829659
1553533_at	JPH1	-1,537851782	-1,520022123	-0,017829659
1569307_s_at	-	-1,537851782	-1,520022123	-0,017829659
216621_at	-	-1,537851782	-1,520022123	-0,017829659
1569670_at	PP12613	-1,537851782	-1,520022123	-0,017829659
1561671_at	LOC286121	-1,537851782	-1,520022123	-0,017829659
234598_at	-	-1,537851782	-1,520022123	-0,017829659
239369_at	LCN8	-1,537851782	-1,520022123	-0,017829659
236547_at	-	-1,537851782	-1,520022123	-0,017829659
1561890_at	C10orf128	-1,537851782	-1,520022123	-0,017829659
1552887_at	TBATA	-1,537851782	-1,520022123	-0,017829659
236775_s_at	-	-1,537851782	-1,520022123	-0,017829659
217195_at	-	-1,537851782	-1,520022123	-0,017829659
221457_s_at	BTNL2	-1,537851782	-1,520022123	-0,017829659
204542_at	ST6GALNAC2	-1,537851782	-1,520022123	-0,017829659
206549_at	INSL4	-1,537851782	-1,520022123	-0,017829659
206276_at	LY6D	-1,537851782	-1,520022123	-0,017829659
236464_at	-	-1,537851782	-1,520022123	-0,017829659
211840_s_at	PDE4D	-1,537851782	-1,520022123	-0,017829659
200881_s_at	DNAJA1	6,20885429	6,226713971	-0,017859682

219100_at	OBFC1	2,829929672	2,848029002	-0,01809933
205026_at	STAT5B	0,648195588	0,666486137	-0,018290549
226377_at	NFIC	-0,135065865	-0,116767475	-0,01829839
235833_at	PPAT	-0,135065865	-0,116767475	-0,01829839
238273_at	C7orf73 /// LOC10	-0,135065865	-0,116767475	-0,01829839
226252_at	-	-0,437449947	-0,418248858	-0,019201089
230810_at	JMJD4	-0,437449947	-0,418248858	-0,019201089
216824_at	-	-0,437449947	-0,418248858	-0,019201089
210761_s_at	GRB7	-0,437449947	-0,418248858	-0,019201089
1565845_at	-	-0,437449947	-0,418248858	-0,019201089
203004_s_at	MEF2D	-0,437449947	-0,418248858	-0,019201089
217971_at	LAMTOR3	4,0992582	4,11856427	-0,019306069
238650_x_at	WDR89	1,640193838	1,659532492	-0,019338653
230846_at	AKAP5	0,707657549	0,727123109	-0,01946556
1554126_at	MSRB3	0,677745787	0,697221375	-0,019475588
1563546_at	-	-2,947665486	-2,928183529	-0,019481957
207277_at	CD209	-0,280273599	-0,260526297	-0,019747302
239074_at	GRAPL	-0,280273599	-0,260526297	-0,019747302
229365_at	PPP1R3F	-0,280273599	-0,260526297	-0,019747302
1559489_a_at	LOC257358	-0,280273599	-0,260526297	-0,019747302
235782_at	-	-0,791294935	-0,771340337	-0,019954598
1558305_at	GIGYF2	-0,791294935	-0,771340337	-0,019954598
1563065_at	-	-0,791294935	-0,771340337	-0,019954598
228703_at	P4HA3	-0,791294935	-0,771340337	-0,019954598
244166_at	-	-0,791294935	-0,771340337	-0,019954598
230385_at	03.03.15	-0,609040214	-0,589001171	-0,020039043
1560656_at	-	-0,609040214	-0,589001171	-0,020039043
1556987_s_at	OR2H1	-0,609040214	-0,589001171	-0,020039043
207990_x_at	ACRV1	-0,609040214	-0,589001171	-0,020039043
239531_at	-	-0,609040214	-0,589001171	-0,020039043
233108_at	-	-0,609040214	-0,589001171	-0,020039043
235756_at	-	-0,609040214	-0,589001171	-0,020039043
226173_at	TBC1D25	1,000459215	1,020514937	-0,020055722
216806_at	-	2,107136847	2,127337555	-0,020200708
213364_s_at	SNX1	1,995527416	2,016139502	-0,020612086
1563637_at	LOC729652	-2,163157732	-2,142286822	-0,02087091
1553894_at	CCDC122	-2,163157732	-2,142286822	-0,02087091
233485_at	-	-2,163157732	-2,142286822	-0,02087091
207477_at	-	-2,163157732	-2,142286822	-0,02087091
216999_at	EPOR	-2,163157732	-2,142286822	-0,02087091
240433_x_at	CADM2	-2,163157732	-2,142286822	-0,02087091
240768_x_at	-	-2,163157732	-2,142286822	-0,02087091
217451_at	-	-2,163157732	-2,142286822	-0,02087091
222967_at	SLC5A7	-2,163157732	-2,142286822	-0,02087091
224021_at	RP1	-2,163157732	-2,142286822	-0,02087091
234529_at	PCGEM1	-2,163157732	-2,142286822	-0,02087091
1564533_at	LOC643711	-2,163157732	-2,142286822	-0,02087091
242122_at	-	-2,163157732	-2,142286822	-0,02087091
1570268_at	-	-2,163157732	-2,142286822	-0,02087091
206957_at	AGXT	-2,163157732	-2,142286822	-0,02087091

1569721_at	-	-2,163157732	-2,142286822	-0,02087091
215002_at	61E3.4 /// LOC100	-2,163157732	-2,142286822	-0,02087091
1568722_at	LOC100506832	-2,163157732	-2,142286822	-0,02087091
1562873_at	PWRN1	-2,163157732	-2,142286822	-0,02087091
1558262_at	SPTY2D1-AS1	-2,163157732	-2,142286822	-0,02087091
239123_at	TSC22D1	-2,163157732	-2,142286822	-0,02087091
220228_at	AP4E1	-2,163157732	-2,142286822	-0,02087091
1553411_s_at	SALL3	-2,163157732	-2,142286822	-0,02087091
220293_at	CATSPERB	-2,163157732	-2,142286822	-0,02087091
1552827_s_at	SLC26A7	-2,163157732	-2,142286822	-0,02087091
214026_s_at	SPRED2	-2,163157732	-2,142286822	-0,02087091
233553_at	-	-2,163157732	-2,142286822	-0,02087091
233183_at	SLC4A9	-2,163157732	-2,142286822	-0,02087091
240050_s_at	-	-2,163157732	-2,142286822	-0,02087091
1562165_at	-	-2,163157732	-2,142286822	-0,02087091
232377_at	NXPH1	-2,163157732	-2,142286822	-0,02087091
1552975_x_at	-	-2,163157732	-2,142286822	-0,02087091
1568856_at	NBR1	-2,163157732	-2,142286822	-0,02087091
243232_at	-	-2,163157732	-2,142286822	-0,02087091
225575_at	LIFR	-2,163157732	-2,142286822	-0,02087091
213640_s_at	LOX	-2,163157732	-2,142286822	-0,02087091
1564323_at	-	-2,163157732	-2,142286822	-0,02087091
1553151_at	ATP6V0D2	-2,163157732	-2,142286822	-0,02087091
208525_s_at	OR2F1 /// OR2F2	-2,163157732	-2,142286822	-0,02087091
1564446_at	LOC284930	-2,163157732	-2,142286822	-0,02087091
228850_s_at	SLIT2	-2,163157732	-2,142286822	-0,02087091
242412_at	-	-2,163157732	-2,142286822	-0,02087091
234387_at	COL4A5	-2,163157732	-2,142286822	-0,02087091
241642_x_at	TLK1	-2,163157732	-2,142286822	-0,02087091
1566867_at	-	-2,163157732	-2,142286822	-0,02087091
233371_at	ABCC13	-2,163157732	-2,142286822	-0,02087091
207951_at	CSN2	-2,163157732	-2,142286822	-0,02087091
223991_s_at	GALNT2	1,510970097	1,531877749	-0,020907652
239585_at	-	-0,994186487	-0,972893339	-0,021293148
240937_at	-	-0,994186487	-0,972893339	-0,021293148
243360_at	-	-0,994186487	-0,972893339	-0,021293148
204406_at	FLT1	-0,994186487	-0,972893339	-0,021293148
239062_at	LOC100131096	-0,994186487	-0,972893339	-0,021293148
1564050_at	LOC100131347	-0,994186487	-0,972893339	-0,021293148
1563019_at	-	-0,994186487	-0,972893339	-0,021293148
205326_at	RAMP3	-0,994186487	-0,972893339	-0,021293148
211295_x_at	CYP2A6	-0,994186487	-0,972893339	-0,021293148
221930_at	PHF7	-0,994186487	-0,972893339	-0,021293148
238855_at	AHNAK	-0,994186487	-0,972893339	-0,021293148
1561705_at	-	-0,994186487	-0,972893339	-0,021293148
228317_at	ZFAND5	-0,994186487	-0,972893339	-0,021293148
215989_at	CBX2	-0,994186487	-0,972893339	-0,021293148
229623_at	-	-2,250580859	-2,229233437	-0,021347422
241068_at	-	-2,250580859	-2,229233437	-0,021347422
206528_at	TRPC6	-2,250580859	-2,229233437	-0,021347422

231490_at	-	-2,250580859	-2,229233437	-0,021347422
234358_at	-	-2,250580859	-2,229233437	-0,021347422
1555854_at	AKR1C1 /// AKR1C	-2,250580859	-2,229233437	-0,021347422
229877_at	NEO1	-2,250580859	-2,229233437	-0,021347422
213802_at	PRSS12	-2,250580859	-2,229233437	-0,021347422
1569677_a_at	MCMDC2	-2,250580859	-2,229233437	-0,021347422
1569272_at	PIK3C3	-2,250580859	-2,229233437	-0,021347422
1555199_at	GOSR1	-2,250580859	-2,229233437	-0,021347422
223843_at	SCARA3	-2,250580859	-2,229233437	-0,021347422
1556200_a_at	C10orf90	-2,250580859	-2,229233437	-0,021347422
210274_at	MAGEA8	-2,250580859	-2,229233437	-0,021347422
217345_at	-	-2,250580859	-2,229233437	-0,021347422
233609_at	PTPRK	-2,250580859	-2,229233437	-0,021347422
215751_at	-	-2,250580859	-2,229233437	-0,021347422
1555022_at	RGS12	-2,250580859	-2,229233437	-0,021347422
229782_at	RMST	-2,250580859	-2,229233437	-0,021347422
243424_at	-	-2,250580859	-2,229233437	-0,021347422
1560734_at	LOC727924 /// OR	-2,250580859	-2,229233437	-0,021347422
211479_s_at	HTR2C	-2,250580859	-2,229233437	-0,021347422
214087_s_at	MYBPC1	-2,250580859	-2,229233437	-0,021347422
228665_at	CYYR1	-2,250580859	-2,229233437	-0,021347422
221138_s_at	-	-2,250580859	-2,229233437	-0,021347422
1561609_at	-	-2,250580859	-2,229233437	-0,021347422
1569740_at	-	-2,250580859	-2,229233437	-0,021347422
1560491_at	LOC100507193	-2,250580859	-2,229233437	-0,021347422
1563246_at	LOC100270679	-2,250580859	-2,229233437	-0,021347422
207889_at	SARDH	-2,250580859	-2,229233437	-0,021347422
234772_s_at	KRTAP2-2	-2,250580859	-2,229233437	-0,021347422
1567376_at	DNAH1	-2,250580859	-2,229233437	-0,021347422
205380_at	PDZK1	-2,250580859	-2,229233437	-0,021347422
1566043_at	-	-2,250580859	-2,229233437	-0,021347422
241641_at	-	-2,250580859	-2,229233437	-0,021347422
204515_at	HSD3B1	-2,250580859	-2,229233437	-0,021347422
243689_s_at	FRG1B	-2,250580859	-2,229233437	-0,021347422
215363_x_at	FOLH1 /// FOLH1B	-2,250580859	-2,229233437	-0,021347422
1565407_at	LHX9	-2,250580859	-2,229233437	-0,021347422
205381_at	LRRC17	-2,250580859	-2,229233437	-0,021347422
217402_at	-	-2,250580859	-2,229233437	-0,021347422
241529_at	-	-2,250580859	-2,229233437	-0,021347422
240067_at	-	-2,250580859	-2,229233437	-0,021347422
217060_at	-	-2,250580859	-2,229233437	-0,021347422
233249_at	-	-2,250580859	-2,229233437	-0,021347422
1559429_a_at	C3orf43	-2,250580859	-2,229233437	-0,021347422
1554032_at	ARSB	-2,250580859	-2,229233437	-0,021347422
233340_at	SPINK13	-2,250580859	-2,229233437	-0,021347422
220921_at	SPANXB1 /// SPAN	-2,250580859	-2,229233437	-0,021347422
226228_at	AQP4	-2,250580859	-2,229233437	-0,021347422
242313_at	LOC728730	-2,250580859	-2,229233437	-0,021347422
231767_at	HOXB4	1,948628481	1,970106792	-0,021478312
201066_at	CYC1	5,33347877	5,35506109	-0,021582319

219520_s_at	WWC3	2,199941239	2,221565736	-0,021624497
1557373_at	LOC339505	-1,212652659	-1,19085694	-0,021795719
203453_at	SCNN1A	-1,212652659	-1,19085694	-0,021795719
1562617_at	LOC340074	-1,212652659	-1,19085694	-0,021795719
1552543_a_at	STON2	-1,212652659	-1,19085694	-0,021795719
1561378_at	C12orf42	-1,212652659	-1,19085694	-0,021795719
214803_at	CDH6	-1,212652659	-1,19085694	-0,021795719
228803_at	PMS1	-1,212652659	-1,19085694	-0,021795719
234225_at	-	-1,212652659	-1,19085694	-0,021795719
1565802_at	-	-1,212652659	-1,19085694	-0,021795719
210314_x_at	TNFSF13	-1,212652659	-1,19085694	-0,021795719
211258_s_at	TGFA	-1,212652659	-1,19085694	-0,021795719
236372_at	-	-1,212652659	-1,19085694	-0,021795719
216325_x_at	RTEL1 /// RTEL1-TI	-1,212652659	-1,19085694	-0,021795719
231734_at	RBP2	-1,212652659	-1,19085694	-0,021795719
221166_at	FGF23	-1,212652659	-1,19085694	-0,021795719
211009_s_at	ZNF271	1,686984721	1,709047448	-0,022062727
217818_s_at	ARPC4	2,62869474	2,65087728	-0,02218254
226664_at	TBC1D20	0,764143511	0,786391549	-0,022248038
214989_x_at	-	0,847529938	0,869824768	-0,022294831
234601_x_at	-	-2,774503732	-2,752097204	-0,022406529
1570244_at	-	-2,774503732	-2,752097204	-0,022406529
1565657_at	CMTM6	-2,774503732	-2,752097204	-0,022406529
1555166_a_at	ZNF396	-2,774503732	-2,752097204	-0,022406529
1562916_at	LINC00102	-2,774503732	-2,752097204	-0,022406529
1569934_at	PREX2	-2,774503732	-2,752097204	-0,022406529
1567576_at	-	-2,774503732	-2,752097204	-0,022406529
217846_at	QARS	5,63842425	5,661533253	-0,023109003
218333_at	DERL2	3,813758784	3,837037831	-0,023279047
214681_at	GK	0,791908897	0,815200271	-0,023291374
222619_at	ZNF281	3,055618382	3,078940895	-0,023322513
200830_at	PSMD2	4,672908132	4,696675878	-0,023767747
89977_at	ACSM5	-1,198521215	-1,174742224	-0,023778991
205967_at	HIST1H4A /// HIST	7,467701547	7,49151065	-0,023809103
228164_at	AP4E1	1,476175815	1,500059916	-0,023884101
201081_s_at	PIP4K2B	0,282358733	0,306454867	-0,024096134
216634_at	PLCH1	-2,074223907	-2,049747196	-0,024476711
1556760_a_at	-	-2,074223907	-2,049747196	-0,024476711
241953_at	FLJ25694 /// KRTA	-2,074223907	-2,049747196	-0,024476711
221398_at	TAS2R8	-2,074223907	-2,049747196	-0,024476711
236603_at	-	-2,074223907	-2,049747196	-0,024476711
1555671_at	ICA1L	-2,074223907	-2,049747196	-0,024476711
1559709_at	-	-2,074223907	-2,049747196	-0,024476711
1557177_at	LOC100506258	-2,074223907	-2,049747196	-0,024476711
224325_at	FZD8	-2,074223907	-2,049747196	-0,024476711
1568735_at	-	-2,074223907	-2,049747196	-0,024476711
1560867_a_at	-	-2,074223907	-2,049747196	-0,024476711
240462_at	CLYBL	-2,074223907	-2,049747196	-0,024476711
226213_at	ERBB3	-2,074223907	-2,049747196	-0,024476711
244799_s_at	-	-2,074223907	-2,049747196	-0,024476711

244390_at	-	-2,074223907	-2,049747196	-0,024476711
221304_at	UGT1A8	-2,074223907	-2,049747196	-0,024476711
231507_at	-	-2,074223907	-2,049747196	-0,024476711
243535_at	-	-2,074223907	-2,049747196	-0,024476711
1560031_at	FRMD4A	-2,074223907	-2,049747196	-0,024476711
235504_at	GREM2	-2,074223907	-2,049747196	-0,024476711
230829_at	CST9L	-2,074223907	-2,049747196	-0,024476711
243348_at	-	-2,074223907	-2,049747196	-0,024476711
1553036_at	GPR111	-2,074223907	-2,049747196	-0,024476711
241749_at	MURC	-2,074223907	-2,049747196	-0,024476711
236925_at	-	-2,074223907	-2,049747196	-0,024476711
244651_at	-	-2,074223907	-2,049747196	-0,024476711
1555098_a_at	CACNB2	-2,074223907	-2,049747196	-0,024476711
238932_at	-	-2,074223907	-2,049747196	-0,024476711
231766_s_at	COL12A1	-2,074223907	-2,049747196	-0,024476711
1554394_at	NELL1	-2,074223907	-2,049747196	-0,024476711
1569816_at	-	-2,074223907	-2,049747196	-0,024476711
213120_at	UHRF1BP1L	-2,074223907	-2,049747196	-0,024476711
1560836_at	-	-2,074223907	-2,049747196	-0,024476711
217531_at	-	-2,074223907	-2,049747196	-0,024476711
1555666_at	PTPRS	-2,074223907	-2,049747196	-0,024476711
231589_at	-	-2,074223907	-2,049747196	-0,024476711
207469_s_at	PIR	-2,074223907	-2,049747196	-0,024476711
235337_at	SERTAD4	-2,336678463	-2,312054429	-0,024624034
241485_at	MYT1L	-2,336678463	-2,312054429	-0,024624034
238309_x_at	-	-2,336678463	-2,312054429	-0,024624034
229266_at	SHISA6	-2,336678463	-2,312054429	-0,024624034
220405_at	SNTG1	-2,336678463	-2,312054429	-0,024624034
215704_at	FLG	-2,336678463	-2,312054429	-0,024624034
206639_x_at	HTN1	-2,336678463	-2,312054429	-0,024624034
217505_at	KLHL23 /// PHOSPI	-2,336678463	-2,312054429	-0,024624034
1569476_at	DKFZP434L187	-2,336678463	-2,312054429	-0,024624034
238368_at	-	-2,336678463	-2,312054429	-0,024624034
205430_at	BMP5	-2,336678463	-2,312054429	-0,024624034
234320_at	CD244	-2,336678463	-2,312054429	-0,024624034
238162_at	-	-2,336678463	-2,312054429	-0,024624034
1562573_at	CYP17A1	-2,336678463	-2,312054429	-0,024624034
222940_at	SULT1E1	-2,336678463	-2,312054429	-0,024624034
1552852_a_at	ZSCAN4	-2,336678463	-2,312054429	-0,024624034
230624_at	SLC25A27	-2,336678463	-2,312054429	-0,024624034
1553365_at	DEPDC4	-2,336678463	-2,312054429	-0,024624034
233098_s_at	EPB41L4B	-2,336678463	-2,312054429	-0,024624034
214416_at	-	-2,336678463	-2,312054429	-0,024624034
233760_at	UTP11L	-2,336678463	-2,312054429	-0,024624034
244747_at	NPNT	-2,336678463	-2,312054429	-0,024624034
1560850_at	-	-2,336678463	-2,312054429	-0,024624034
242686_at	STARD13	-2,336678463	-2,312054429	-0,024624034
1557024_at	-	-2,336678463	-2,312054429	-0,024624034
1555019_at	CDHR1	-2,336678463	-2,312054429	-0,024624034
206740_x_at	SYCP1	-2,336678463	-2,312054429	-0,024624034

1552693_at	ARL11	-2,336678463	-2,312054429	-0,024624034
1555547_at	-	-2,336678463	-2,312054429	-0,024624034
203779_s_at	MPZL2	-2,336678463	-2,312054429	-0,024624034
233770_at	-	-2,336678463	-2,312054429	-0,024624034
241085_at	-	-2,336678463	-2,312054429	-0,024624034
243325_at	-	-2,336678463	-2,312054429	-0,024624034
201939_at	PLK2	-2,336678463	-2,312054429	-0,024624034
1552321_a_at	CCDC65	-2,336678463	-2,312054429	-0,024624034
226653_at	MARK1	-2,336678463	-2,312054429	-0,024624034
1558552_s_at	C3orf55	-2,336678463	-2,312054429	-0,024624034
238294_at	-	-2,336678463	-2,312054429	-0,024624034
227514_at	ITPRIPL2	-2,336678463	-2,312054429	-0,024624034
214053_at	ERBB4	-2,336678463	-2,312054429	-0,024624034
208974_x_at	KPNB1	5,784719278	5,80935506	-0,024635782
218447_at	CMC2	5,245449884	5,270102752	-0,024652868
213101_s_at	ACTR3	7,42884323	7,453604465	-0,024761235
243548_x_at	-	-2,416625063	-2,391863529	-0,024761534
1559256_at	MAGI1	-2,416625063	-2,391863529	-0,024761534
1566969_at	-	-2,416625063	-2,391863529	-0,024761534
236705_at	TMEM196	-2,416625063	-2,391863529	-0,024761534
231455_at	LINC00487	-2,416625063	-2,391863529	-0,024761534
1561912_at	-	-2,416625063	-2,391863529	-0,024761534
234105_at	-	-2,416625063	-2,391863529	-0,024761534
1556904_at	-	-2,416625063	-2,391863529	-0,024761534
1558667_at	SPATA22	-2,416625063	-2,391863529	-0,024761534
207102_at	AKR1D1	-2,416625063	-2,391863529	-0,024761534
217613_at	TMEM144	-2,416625063	-2,391863529	-0,024761534
220552_at	TRPC5	-2,416625063	-2,391863529	-0,024761534
1569938_at	SIRT5	-2,416625063	-2,391863529	-0,024761534
1565976_at	-	-2,416625063	-2,391863529	-0,024761534
242221_at	-	-2,416625063	-2,391863529	-0,024761534
222237_s_at	ZFP112 /// ZNF285	-2,416625063	-2,391863529	-0,024761534
1569023_a_at	LINC00458	-2,416625063	-2,391863529	-0,024761534
244832_at	-	-2,416625063	-2,391863529	-0,024761534
1554981_at	C9orf11	-2,416625063	-2,391863529	-0,024761534
215917_at	RAD21L1	-2,416625063	-2,391863529	-0,024761534
233604_at	FLJ22763	-2,416625063	-2,391863529	-0,024761534
207879_at	-	-2,416625063	-2,391863529	-0,024761534
224358_s_at	MS4A7	-2,416625063	-2,391863529	-0,024761534
1563920_at	FAM45A	-2,416625063	-2,391863529	-0,024761534
217431_x_at	CYBB	-2,416625063	-2,391863529	-0,024761534
241651_at	-	-2,416625063	-2,391863529	-0,024761534
1559763_at	ZC3H12C	-2,416625063	-2,391863529	-0,024761534
233882_s_at	SEMA6D	-2,416625063	-2,391863529	-0,024761534
237573_at	-	-2,416625063	-2,391863529	-0,024761534
237805_at	-	-2,416625063	-2,391863529	-0,024761534
1558806_at	-	-2,416625063	-2,391863529	-0,024761534
1563541_at	-	-2,416625063	-2,391863529	-0,024761534
219936_s_at	GPR87	-2,416625063	-2,391863529	-0,024761534
236740_at	-	-2,416625063	-2,391863529	-0,024761534

1554789_a_at	PDE8B	-2,416625063	-2,391863529	-0,024761534
243994_at	-	-2,416625063	-2,391863529	-0,024761534
244056_at	SFTA2	-2,416625063	-2,391863529	-0,024761534
207373_at	HOXD10	-2,416625063	-2,391863529	-0,024761534
227792_at	ITPRIPL2	-2,416625063	-2,391863529	-0,024761534
222113_s_at	EPS15L1	1,383446753	1,408220608	-0,024773855
221579_s_at	NUDT3 /// RPS10-I	2,436165655	2,460974054	-0,024808399
224721_at	WDR75	2,752615529	2,777528602	-0,024913074
202691_at	SNRPD1	3,513985141	3,538959266	-0,024974125
202263_at	CYB5R1	2,107136847	2,132145748	-0,025008902
214686_at	ZNF266	3,592169709	3,617435945	-0,025266236
233080_s_at	PRPF40A	4,20258728	4,227865102	-0,025277822
221983_at	FAM134A	3,218770562	3,244090313	-0,025319752
223716_s_at	ZRANB2	4,713199262	4,738630498	-0,025431236
206652_at	ZMYM5	1,364555912	1,390070295	-0,025514383
239313_at	LOC401320	-0,088974936	-0,063377083	-0,025597853
217416_x_at	-	-0,088974936	-0,063377083	-0,025597853
1560198_at	LINC00523	-0,088974936	-0,063377083	-0,025597853
1561300_at	-	-1,982324124	-1,956681069	-0,025643054
236276_at	-	-1,982324124	-1,956681069	-0,025643054
1565668_at	LOC100288366 ///	-1,982324124	-1,956681069	-0,025643054
215422_at	-	-1,982324124	-1,956681069	-0,025643054
1569512_at	-	-1,982324124	-1,956681069	-0,025643054
1570345_at	LINC00474	-1,982324124	-1,956681069	-0,025643054
240180_at	-	-1,982324124	-1,956681069	-0,025643054
204400_at	EFS	-1,982324124	-1,956681069	-0,025643054
1556097_at	HOMER2	-1,982324124	-1,956681069	-0,025643054
229288_at	EPHA7	-1,982324124	-1,956681069	-0,025643054
243776_at	-	-1,982324124	-1,956681069	-0,025643054
221019_s_at	COLEC12	-1,982324124	-1,956681069	-0,025643054
236130_at	SNORA37	-1,982324124	-1,956681069	-0,025643054
1565839_a_at	-	-1,982324124	-1,956681069	-0,025643054
228950_s_at	WLS	-1,982324124	-1,956681069	-0,025643054
1553093_a_at	DEFB119	-1,982324124	-1,956681069	-0,025643054
1570274_at	PRSS55	-1,982324124	-1,956681069	-0,025643054
243231_at	SLC38A11	-1,982324124	-1,956681069	-0,025643054
240467_at	-	-1,982324124	-1,956681069	-0,025643054
233808_at	-	-1,982324124	-1,956681069	-0,025643054
241487_at	-	-1,982324124	-1,956681069	-0,025643054
1562247_at	LOC286058	-1,982324124	-1,956681069	-0,025643054
239850_at	-	-1,982324124	-1,956681069	-0,025643054
240883_at	PFKFB1	-1,982324124	-1,956681069	-0,025643054
205615_at	CPA1	-1,982324124	-1,956681069	-0,025643054
1561430_s_at	C3orf15 /// LOC10	-1,982324124	-1,956681069	-0,025643054
1562847_at	-	-1,982324124	-1,956681069	-0,025643054
243805_at	CCBE1	-1,982324124	-1,956681069	-0,025643054
240184_at	-	-1,982324124	-1,956681069	-0,025643054
233389_at	C20orf26	-1,982324124	-1,956681069	-0,025643054
208400_at	GLP1R	-1,982324124	-1,956681069	-0,025643054
1552408_at	ODF4	-1,982324124	-1,956681069	-0,025643054

1564287_at	LINC00410	-1,982324124	-1,956681069	-0,025643054
1557575_at	STBD1	-1,982324124	-1,956681069	-0,025643054
225511_at	GPRC5B	-1,982324124	-1,956681069	-0,025643054
240009_at	-	-1,982324124	-1,956681069	-0,025643054
243793_at	AHDC1	-1,982324124	-1,956681069	-0,025643054
206645_s_at	NR0B1	-1,982324124	-1,956681069	-0,025643054
1563814_at	C2orf50	-1,982324124	-1,956681069	-0,025643054
205517_at	GATA4	-1,982324124	-1,956681069	-0,025643054
241312_at	-	-1,982324124	-1,956681069	-0,025643054
231956_at	RNF213	1,20396005	1,2298509	-0,02589085
200735_x_at	NACA	7,931174707	7,957331509	-0,026156802
1557070_at	LOC100130275	1,326463531	1,352622437	-0,026158906
219349_s_at	EXOC2	1,544940605	1,571112806	-0,026172201
200845_s_at	PRDX6	5,323188936	5,349417202	-0,026228266
37586_at	ZNF142	1,139613426	1,165869707	-0,02625628
211956_s_at	EIF1	7,858587226	7,885239194	-0,026651968
232652_x_at	SCAND1	3,410696467	3,437599225	-0,026902757
202220_at	KIAA0907	4,258822873	4,285931953	-0,02710908
219611_s_at	CEP85	1,948628481	1,975797592	-0,027169111
226669_at	USP42	1,948628481	1,975797592	-0,027169111
201048_x_at	RAB6A	0,556428218	0,583607503	-0,027179285
211323_s_at	ITPR1	1,802733148	1,830129603	-0,027396456
214876_s_at	TUBGCP5	0,319287178	0,346964736	-0,027677558
213112_s_at	SQSTM1	0,319287178	0,346964736	-0,027677558
204020_at	PURA	3,895112363	3,923044459	-0,027932096
218868_at	ACTR3B	1,287793882	1,315897482	-0,0281036
209463_s_at	TAF12	3,060716358	3,08892491	-0,028208552
212855_at	DCUN1D4	1,510970097	1,539450683	-0,028480586
228050_at	UTP15	1,788922638	1,817403386	-0,028480748
1555653_at	-	6,501163288	6,529772418	-0,02860913
239038_at	C1orf52	-0,230432956	-0,201789721	-0,028643235
214789_x_at	SRSF8	4,42686741	4,455535827	-0,028668417
1555756_a_at	CLEC7A	-2,495152701	-2,466349414	-0,028803287
237233_at	-	-2,495152701	-2,466349414	-0,028803287
217452_s_at	B3GALT2	-2,495152701	-2,466349414	-0,028803287
1558708_at	NRXN1	-2,495152701	-2,466349414	-0,028803287
1565806_at	FLJ37035	-2,495152701	-2,466349414	-0,028803287
241562_x_at	-	-2,495152701	-2,466349414	-0,028803287
1560184_at	TMCO5A	-2,495152701	-2,466349414	-0,028803287
1552878_at	ATOH7	-2,495152701	-2,466349414	-0,028803287
1570359_at	DDX3Y	-2,495152701	-2,466349414	-0,028803287
1562990_at	LINC00564	-2,495152701	-2,466349414	-0,028803287
240509_s_at	GREM2	-2,495152701	-2,466349414	-0,028803287
1561672_at	-	-2,495152701	-2,466349414	-0,028803287
202620_s_at	PLOD2	-2,495152701	-2,466349414	-0,028803287
233071_at	RSPH4A	-2,495152701	-2,466349414	-0,028803287
1557606_at	-	-2,495152701	-2,466349414	-0,028803287
231202_at	ALDH1L2	-2,495152701	-2,466349414	-0,028803287
230895_at	HAPLN1	-2,495152701	-2,466349414	-0,028803287
1570117_at	-	-2,495152701	-2,466349414	-0,028803287

237910_x_at	FAM92A1	-2,495152701	-2,466349414	-0,028803287
224158_s_at	-	-2,495152701	-2,466349414	-0,028803287
1558893_a_at	CCDC67	-2,495152701	-2,466349414	-0,028803287
237514_at	LOC100507274	-2,495152701	-2,466349414	-0,028803287
1557395_at	LOC255130	-2,495152701	-2,466349414	-0,028803287
1554946_at	LOC100506465	-2,495152701	-2,466349414	-0,028803287
231800_s_at	DMRT3	-2,495152701	-2,466349414	-0,028803287
1560733_at	ALG5	-2,495152701	-2,466349414	-0,028803287
1554751_at	-	-2,495152701	-2,466349414	-0,028803287
206456_at	GABRA5	-2,495152701	-2,466349414	-0,028803287
1563801_at	-	-2,495152701	-2,466349414	-0,028803287
238018_at	FAM150B	-2,495152701	-2,466349414	-0,028803287
221441_at	GSC2	-2,495152701	-2,466349414	-0,028803287
1562464_at	-	-2,495152701	-2,466349414	-0,028803287
50376_at	ZNF444	1,745656434	1,774507178	-0,028850744
218385_at	MRPS18A	2,849132494	2,878165848	-0,029033353
218738_s_at	RNF138	4,554653912	4,583949023	-0,029295112
214672_at	TTLL5	2,053550345	2,082919929	-0,029369584
1568618_a_at	GALNT1	3,789279973	3,818669263	-0,029389289
229098_s_at	C5orf24	-0,384193355	-0,354781582	-0,029411773
236486_at	-	-0,384193355	-0,354781582	-0,029411773
221647_s_at	RIC8A	3,19484646	3,224658402	-0,029811942
237257_at	RAB4B	0,355751005	0,385582632	-0,029831627
213728_at	LAMP1	0,355751005	0,385582632	-0,029831627
225626_at	PAG1	4,027733188	4,057569918	-0,02983673
214669_x_at	IGKC	1,92317941	1,953032682	-0,029853272
244589_at	-	-1,452713826	-1,422257026	-0,0304568
240178_at	-	-1,452713826	-1,422257026	-0,0304568
234101_at	-	-1,452713826	-1,422257026	-0,0304568
240783_at	-	-1,452713826	-1,422257026	-0,0304568
240095_at	-	-1,452713826	-1,422257026	-0,0304568
1556874_a_at	MEX3C	-1,452713826	-1,422257026	-0,0304568
240797_at	PERP	-1,452713826	-1,422257026	-0,0304568
1565748_at	LOC100132815	-1,452713826	-1,422257026	-0,0304568
231563_at	LOC100505824 ///	-1,452713826	-1,422257026	-0,0304568
1562295_at	ADAMTS9-AS2	-1,452713826	-1,422257026	-0,0304568
211887_x_at	MSR1	-1,452713826	-1,422257026	-0,0304568
1568755_a_at	-	-1,452713826	-1,422257026	-0,0304568
211885_x_at	FUT6	-1,452713826	-1,422257026	-0,0304568
234743_at	LIMD1	-1,452713826	-1,422257026	-0,0304568
244769_at	FLJ33630	-1,452713826	-1,422257026	-0,0304568
234925_at	LOC732275	-1,452713826	-1,422257026	-0,0304568
239856_at	-	-1,452713826	-1,422257026	-0,0304568
211794_at	FYB	-1,452713826	-1,422257026	-0,0304568
233374_at	-	-1,452713826	-1,422257026	-0,0304568
240385_at	-	-1,452713826	-1,422257026	-0,0304568
236272_at	UNC119B	-1,452713826	-1,422257026	-0,0304568
237227_at	NEK10	-1,452713826	-1,422257026	-0,0304568
1561313_at	-	-1,452713826	-1,422257026	-0,0304568
216931_at	-	-1,452713826	-1,422257026	-0,0304568

205478_at	PPP1R1A	-1,452713826	-1,422257026	-0,0304568
1553559_at	TMEM171	-1,452713826	-1,422257026	-0,0304568
1554984_a_at	HLA-DOB	-1,452713826	-1,422257026	-0,0304568
231372_at	SLC25A48	-1,452713826	-1,422257026	-0,0304568
222007_s_at	FKBP8	-1,452713826	-1,422257026	-0,0304568
219168_s_at	PRR5	-1,452713826	-1,422257026	-0,0304568
205919_at	HBE1	-1,452713826	-1,422257026	-0,0304568
213804_at	INPP5B	1,760255626	1,790914523	-0,030658897
229236_s_at	SFXN4	1,760255626	1,790914523	-0,030658897
236679_x_at	-	1,024284941	1,055317091	-0,03103215
200674_s_at	RPL32	8,418737238	8,449869302	-0,031132064
224717_s_at	C19orf42	3,505636113	3,536807561	-0,031171448
204867_at	GCHFR	1,267146969	1,298349073	-0,031202104
232100_at	RNF217	-1,893506789	-1,862269442	-0,031237347
241675_s_at	-	-1,893506789	-1,862269442	-0,031237347
231679_at	-	-1,893506789	-1,862269442	-0,031237347
241835_at	-	-1,893506789	-1,862269442	-0,031237347
1566294_at	-	-1,893506789	-1,862269442	-0,031237347
1561927_at	ANKUB1	-1,893506789	-1,862269442	-0,031237347
1562787_at	-	-1,893506789	-1,862269442	-0,031237347
233043_at	LOC221814	-1,893506789	-1,862269442	-0,031237347
1565684_at	LOC400940	-1,893506789	-1,862269442	-0,031237347
1555448_at	AP5M1	-1,893506789	-1,862269442	-0,031237347
221040_at	CAPN10	-1,893506789	-1,862269442	-0,031237347
214921_at	KCNMA1	-1,893506789	-1,862269442	-0,031237347
1564212_at	-	-1,893506789	-1,862269442	-0,031237347
1558903_at	LOC284260	-1,893506789	-1,862269442	-0,031237347
216099_at	HTR7P1	-1,893506789	-1,862269442	-0,031237347
220881_at	-	-1,893506789	-1,862269442	-0,031237347
237928_at	-	-1,893506789	-1,862269442	-0,031237347
234704_at	-	-1,893506789	-1,862269442	-0,031237347
239104_at	MGC42157	-1,893506789	-1,862269442	-0,031237347
232924_at	LRRC17	-1,893506789	-1,862269442	-0,031237347
229230_at	OSTalpha	-1,893506789	-1,862269442	-0,031237347
234440_at	TRDV3	-1,893506789	-1,862269442	-0,031237347
1561192_at	-	-1,893506789	-1,862269442	-0,031237347
213492_at	COL2A1	-1,893506789	-1,862269442	-0,031237347
1553002_at	DEFB105A /// DEFI	-1,893506789	-1,862269442	-0,031237347
229580_at	-	-1,893506789	-1,862269442	-0,031237347
208409_at	SLC14A2	-1,893506789	-1,862269442	-0,031237347
244518_at	LOC100130452	-1,893506789	-1,862269442	-0,031237347
221352_at	-	-1,893506789	-1,862269442	-0,031237347
1569936_a_at	ZNF664	-1,893506789	-1,862269442	-0,031237347
234671_at	KRTAP4-2	-1,893506789	-1,862269442	-0,031237347
210953_at	TSC22D2	-1,893506789	-1,862269442	-0,031237347
220496_at	CLEC1B	-1,893506789	-1,862269442	-0,031237347
227648_at	C22orf32	-1,893506789	-1,862269442	-0,031237347
215078_at	LOC100129518 ///	-1,893506789	-1,862269442	-0,031237347
216424_at	CD4	-1,893506789	-1,862269442	-0,031237347
240736_at	-	-1,893506789	-1,862269442	-0,031237347

235377_at	MLIP	-1,893506789	-1,862269442	-0,031237347
244378_at	C12orf51	-1,893506789	-1,862269442	-0,031237347
1564194_a_at	MS4A15	-1,893506789	-1,862269442	-0,031237347
244141_at	-	-1,893506789	-1,862269442	-0,031237347
220196_at	MUC16	-1,893506789	-1,862269442	-0,031237347
220133_at	ODAM	-1,893506789	-1,862269442	-0,031237347
237268_at	DSCAM	-1,893506789	-1,862269442	-0,031237347
223524_s_at	TMEM108	-1,893506789	-1,862269442	-0,031237347
1554295_x_at	TTBK2	-1,893506789	-1,862269442	-0,031237347
241163_at	-	-1,893506789	-1,862269442	-0,031237347
243818_at	SFTA1P	-1,893506789	-1,862269442	-0,031237347
214343_s_at	ATXN7L1	-1,893506789	-1,862269442	-0,031237347
234284_at	GNG8	-1,893506789	-1,862269442	-0,031237347
211214_s_at	DAPK1	-1,893506789	-1,862269442	-0,031237347
1557094_at	LOC100652762	-1,893506789	-1,862269442	-0,031237347
202423_at	KAT6A	3,03433073	3,065641037	-0,031310307
222440_s_at	THRAP3	3,117098466	3,14842776	-0,031329294
228736_at	HELQ	0,95222556	0,98381571	-0,031590149
203546_at	IPO13	1,000459215	1,032267596	-0,031808382
202540_s_at	HMGCR	3,742498599	3,774776136	-0,032277537
205788_s_at	ZC3H11A	4,859875458	4,892505567	-0,032630109
223983_s_at	C19orf12	3,086998005	3,119733444	-0,03273544
223070_at	SELK	5,22851938	5,2613288	-0,03280942
239845_at	-	-0,730013898	-0,697182596	-0,032831302
243336_at	LOC100132292	-0,730013898	-0,697182596	-0,032831302
219735_s_at	TFCP2L1	-0,730013898	-0,697182596	-0,032831302
213631_x_at	DHODH	-0,730013898	-0,697182596	-0,032831302
1556669_a_at	-	-0,730013898	-0,697182596	-0,032831302
1554523_a_at	CNNM2	-0,730013898	-0,697182596	-0,032831302
204498_s_at	ADCY9	-0,730013898	-0,697182596	-0,032831302
203167_at	TIMP2	-0,730013898	-0,697182596	-0,032831302
223703_at	C10orf11	-0,730013898	-0,697182596	-0,032831302
216897_s_at	FAM76A	-0,043925712	-0,011001236	-0,032924476
1562981_at	HBB	-0,043925712	-0,011001236	-0,032924476
239838_at	ZNF776	-0,043925712	-0,011001236	-0,032924476
237865_x_at	-	-0,043925712	-0,011001236	-0,032924476
201200_at	CREG1	3,807915835	3,84088223	-0,032966395
1559842_at	-	-2,569309811	-2,536339282	-0,03297053
243436_at	-	-2,569309811	-2,536339282	-0,03297053
240570_at	INADL	-2,569309811	-2,536339282	-0,03297053
215112_x_at	MCF2L2	-2,569309811	-2,536339282	-0,03297053
240179_at	LOC100505801	-2,569309811	-2,536339282	-0,03297053
238727_at	-	-2,569309811	-2,536339282	-0,03297053
240771_at	C1orf101	-2,569309811	-2,536339282	-0,03297053
1566970_at	-	-2,569309811	-2,536339282	-0,03297053
1553298_at	C17orf77	-2,569309811	-2,536339282	-0,03297053
1560458_s_at	CAPS2	-2,569309811	-2,536339282	-0,03297053
1557741_at	LOC100128176	-2,569309811	-2,536339282	-0,03297053
235127_at	PMP2	-2,569309811	-2,536339282	-0,03297053
205152_at	SLC6A1	-2,569309811	-2,536339282	-0,03297053

239092_at	ITGA8	-2,569309811	-2,536339282	-0,03297053
1563207_at	C3orf65	-2,569309811	-2,536339282	-0,03297053
205041_s_at	ORM1 /// ORM2	-2,569309811	-2,536339282	-0,03297053
1568739_at	LOC285692	-2,569309811	-2,536339282	-0,03297053
1552582_at	ABCC13	-2,569309811	-2,536339282	-0,03297053
234823_at	-	-2,569309811	-2,536339282	-0,03297053
207166_at	GNGT1	-2,569309811	-2,536339282	-0,03297053
1559546_s_at	LOC100506948 ///	-2,569309811	-2,536339282	-0,03297053
1569680_at	-	-2,569309811	-2,536339282	-0,03297053
1561741_at	-	-2,569309811	-2,536339282	-0,03297053
201119_s_at	COX8A	6,621198463	6,654171365	-0,032972902
206060_s_at	PTPN22	3,389999385	3,422991983	-0,032992598
221464_at	OR1D2	-0,551284523	-0,518232988	-0,033051534
1554584_at	LRRC48	-0,551284523	-0,518232988	-0,033051534
230983_at	FAM129C	-0,551284523	-0,518232988	-0,033051534
233348_at	-	-0,551284523	-0,518232988	-0,033051534
235741_at	PPIA	-0,551284523	-0,518232988	-0,033051534
1555763_x_at	MKL1	-0,551284523	-0,518232988	-0,033051534
228436_at	KCNC4	-0,551284523	-0,518232988	-0,033051534
207347_at	ERCC6	-0,551284523	-0,518232988	-0,033051534
1555780_a_at	RHEB	4,016622037	4,049836996	-0,033214959
200094_s_at	EEF2	7,149075431	7,182473637	-0,033398206
200622_x_at	CALM1 /// CALM2	3,960533133	3,99396405	-0,033430917
44669_at	SDHAF1	2,539323516	2,572938138	-0,033614622
215068_s_at	FBXL18	0,39105295	0,424693306	-0,033640355
213126_at	MED8	2,356619952	2,390281999	-0,033662047
208713_at	HNRNPUL1	4,165622052	4,199298039	-0,033675987
222382_x_at	NUP205	0,648195588	0,681874787	-0,033679199
226298_at	RUNDC1	1,383446753	1,417157926	-0,033711174
226134_s_at	-	2,321828846	2,355777584	-0,033948738
200777_s_at	BZW1	5,344203926	5,378305639	-0,034101713
200807_s_at	HSPD1	7,656752112	7,690893786	-0,034141675
210666_at	IDS	0,677745787	0,711934445	-0,034188658
234584_s_at	ATE1	0,677745787	0,711934445	-0,034188658
226958_s_at	MED11	2,597647262	2,63203137	-0,034384108
203634_s_at	CPT1A	-0,924000698	-0,889222211	-0,034778487
242739_at	-	-0,924000698	-0,889222211	-0,034778487
214337_at	COPA	-0,924000698	-0,889222211	-0,034778487
215296_at	CDC42BPA	-0,924000698	-0,889222211	-0,034778487
224556_s_at	LHX6	-0,924000698	-0,889222211	-0,034778487
207571_x_at	C1orf38	-0,924000698	-0,889222211	-0,034778487
230503_at	-	-0,924000698	-0,889222211	-0,034778487
201568_at	UQCRQ	5,606731501	5,641785817	-0,035054316
224605_at	C4orf3	3,237994092	3,27333514	-0,035341049
226924_at	LOC400657	2,678352494	2,713775735	-0,035423241
227374_at	EARS2	1,857989965	1,893444329	-0,035454364
225884_s_at	GZF1	2,671492308	2,707013102	-0,035520794
1566930_at	TFB2M	-2,892362142	-2,856570137	-0,035792005
223602_at	USP30	0,900065578	0,936232181	-0,036166603
1560921_at	ZNF169	-0,180990326	-0,144765583	-0,036224743

1561044_at	-	-0,180990326	-0,144765583	-0,036224743
234714_x_at	ATP2B2	-0,180990326	-0,144765583	-0,036224743
225094_at	SETD8	2,38349585	2,419753897	-0,036258046
1568864_at	LOC100131691	-1,137208348	-1,100943374	-0,036264974
241033_at	-	-1,137208348	-1,100943374	-0,036264974
224245_at	INGX	-1,137208348	-1,100943374	-0,036264974
215932_at	MAGEC2	-1,137208348	-1,100943374	-0,036264974
230981_at	CATSPER3	-1,137208348	-1,100943374	-0,036264974
220910_at	FRAS1	-1,137208348	-1,100943374	-0,036264974
1553775_at	FLJ31715	-1,137208348	-1,100943374	-0,036264974
244576_at	-	-1,137208348	-1,100943374	-0,036264974
232951_at	-	-1,137208348	-1,100943374	-0,036264974
211221_at	TADA3	-1,137208348	-1,100943374	-0,036264974
238260_at	-	-1,137208348	-1,100943374	-0,036264974
228422_at	LHFPL4	-1,137208348	-1,100943374	-0,036264974
207593_at	ABCG4	-1,137208348	-1,100943374	-0,036264974
232485_at	SNX29	-1,137208348	-1,100943374	-0,036264974
207600_at	KCNC3	-1,137208348	-1,100943374	-0,036264974
229078_s_at	KIAA1704	0,425036312	0,461385738	-0,036349425
208227_x_at	ADAM22	0,425036312	0,461385738	-0,036349425
228745_at	SGTB	2,392346173	2,428719338	-0,036373165
200925_at	COX6A1	6,76527833	6,801823348	-0,036545018
215152_at	MYB	0,001114523	0,03784737	-0,036732847
212541_at	FLAD1	2,552836808	2,589579418	-0,03674261
214076_at	GFOD2	0,735999505	0,772793615	-0,036794109
209248_at	GHITM	4,274951805	4,311836632	-0,036884827
208625_s_at	EIF4G1	3,012642182	3,049587049	-0,036944867
1570347_at	MLX	0,764143511	0,80114751	-0,037003999
218682_s_at	SLC4A1AP	3,096793374	3,134198147	-0,037404773
228585_at	ENTPD1	1,760255626	1,797682937	-0,037427312
202025_x_at	ACAA1	2,71254166	2,750185881	-0,037644221
243885_x_at	-	-2,641232718	-2,603471267	-0,037761451
241099_at	-	-2,641232718	-2,603471267	-0,037761451
234433_at	-	-2,641232718	-2,603471267	-0,037761451
243126_x_at	-	-2,641232718	-2,603471267	-0,037761451
1569873_at	LIPJ	-2,641232718	-2,603471267	-0,037761451
1561437_at	-	-2,641232718	-2,603471267	-0,037761451
229802_at	WISP1	-2,641232718	-2,603471267	-0,037761451
231213_at	PDE1A	-2,641232718	-2,603471267	-0,037761451
1562276_at	-	-2,641232718	-2,603471267	-0,037761451
212298_at	NRP1	-2,641232718	-2,603471267	-0,037761451
1561997_at	-	-2,641232718	-2,603471267	-0,037761451
206439_at	EPYC	-2,641232718	-2,603471267	-0,037761451
223315_at	NTN4	-2,641232718	-2,603471267	-0,037761451
243040_at	-	-2,641232718	-2,603471267	-0,037761451
237760_at	LOC100505768	-2,641232718	-2,603471267	-0,037761451
210170_at	PDLIM3	-2,641232718	-2,603471267	-0,037761451
240707_at	-	-2,641232718	-2,603471267	-0,037761451
231425_at	PLCZ1	-2,641232718	-2,603471267	-0,037761451
208339_at	XKRY /// XKRY2	-2,641232718	-2,603471267	-0,037761451

209300_s_at	NECAP1	2,285848435	2,323616375	-0,037767939
203941_at	INTS9	2,605420261	2,64347734	-0,03805708
242470_at	EID2B	1,421391637	1,459545271	-0,038153634
221715_at	-	0,458905032	0,497424714	-0,038519683
1557326_at	-	-1,802716385	-1,763664074	-0,039052311
211516_at	IL5RA	-1,802716385	-1,763664074	-0,039052311
1553329_at	C7orf45	-1,802716385	-1,763664074	-0,039052311
1561106_at	LINC00271	-1,802716385	-1,763664074	-0,039052311
237100_at	-	-1,802716385	-1,763664074	-0,039052311
1560334_at	MEGF11	-1,802716385	-1,763664074	-0,039052311
1562863_at	-	-1,802716385	-1,763664074	-0,039052311
203962_s_at	NEBL	-1,802716385	-1,763664074	-0,039052311
241157_at	-	-1,802716385	-1,763664074	-0,039052311
231468_at	SH3BP4	-1,802716385	-1,763664074	-0,039052311
1560527_at	NFE4	-1,802716385	-1,763664074	-0,039052311
1555430_at	TSTD2	-1,802716385	-1,763664074	-0,039052311
244301_at	-	-1,802716385	-1,763664074	-0,039052311
1556171_a_at	-	-1,802716385	-1,763664074	-0,039052311
1561370_at	LOC100505716	-1,802716385	-1,763664074	-0,039052311
236374_at	CTXN3	-1,802716385	-1,763664074	-0,039052311
220348_at	KLHL29	-1,802716385	-1,763664074	-0,039052311
1557301_a_at	-	-1,802716385	-1,763664074	-0,039052311
227253_at	CP	-1,802716385	-1,763664074	-0,039052311
203681_at	IVD	-1,802716385	-1,763664074	-0,039052311
240227_at	MIER1	-1,802716385	-1,763664074	-0,039052311
237090_at	LOC100506113	-1,802716385	-1,763664074	-0,039052311
241634_at	-	-1,802716385	-1,763664074	-0,039052311
221296_at	TECTA	-1,802716385	-1,763664074	-0,039052311
1557060_at	-	-1,802716385	-1,763664074	-0,039052311
217541_x_at	ZNF816 /// ZNF81(	-1,802716385	-1,763664074	-0,039052311
238881_at	-	-1,802716385	-1,763664074	-0,039052311
234412_at	-	-1,802716385	-1,763664074	-0,039052311
205306_x_at	KMO	-1,802716385	-1,763664074	-0,039052311
241882_at	CAMTA1	-1,802716385	-1,763664074	-0,039052311
1552288_at	CILP2	-1,802716385	-1,763664074	-0,039052311
235916_at	YPEL4	-1,802716385	-1,763664074	-0,039052311
208388_at	NR2E3	-1,802716385	-1,763664074	-0,039052311
229818_at	SVOP	-1,802716385	-1,763664074	-0,039052311
215972_at	PART1	-1,802716385	-1,763664074	-0,039052311
210791_s_at	ARHGAP32	-1,802716385	-1,763664074	-0,039052311
1558166_at	MGC16275	-1,802716385	-1,763664074	-0,039052311
225372_at	C10orf54	-1,802716385	-1,763664074	-0,039052311
229469_at	XAGE2 /// XAGE2B	-1,802716385	-1,763664074	-0,039052311
208677_s_at	BSG	3,35759121	3,39740877	-0,03981756
212290_at	SLC7A1	2,127965541	2,167793428	-0,039827887
239343_at	LOC728705	0,045517965	0,085534992	-0,040017028
[truncated: 1,656,429 more chars]
